# Fertility, mortality, migration, and population scenarios for 195 countries and territories from 2017 to 2100: a forecasting analysis for the Global Burden of Disease Study

**DOI:** 10.1016/S0140-6736(20)30677-2

**Published:** 2020-10-17

**Authors:** Stein Emil Vollset, Emily Goren, Chun-Wei Yuan, Jackie Cao, Amanda E Smith, Thomas Hsiao, Catherine Bisignano, Gulrez S Azhar, Emma Castro, Julian Chalek, Andrew J Dolgert, Tahvi Frank, Kai Fukutaki, Simon I Hay, Rafael Lozano, Ali H Mokdad, Vishnu Nandakumar, Maxwell Pierce, Martin Pletcher, Toshana Robalik, Krista M Steuben, Han Yong Wunrow, Bianca S Zlavog, Christopher J L Murray

**Affiliations:** a Institute for Health Metrics and Evaluation, University of Washington, Seattle, WA, USA; b Department of Health Metrics Sciences, School of Medicine, University of Washington, Seattle, WA, USA

## Abstract

**Background:**

Understanding potential patterns in future population levels is crucial for anticipating and planning for changing age structures, resource and health-care needs, and environmental and economic landscapes. Future fertility patterns are a key input to estimation of future population size, but they are surrounded by substantial uncertainty and diverging methodologies of estimation and forecasting, leading to important differences in global population projections. Changing population size and age structure might have profound economic, social, and geopolitical impacts in many countries. In this study, we developed novel methods for forecasting mortality, fertility, migration, and population. We also assessed potential economic and geopolitical effects of future demographic shifts.

**Methods:**

We modelled future population in reference and alternative scenarios as a function of fertility, migration, and mortality rates. We developed statistical models for completed cohort fertility at age 50 years (CCF50). Completed cohort fertility is much more stable over time than the period measure of the total fertility rate (TFR). We modelled CCF50 as a time-series random walk function of educational attainment and contraceptive met need. Age-specific fertility rates were modelled as a function of CCF50 and covariates. We modelled age-specific mortality to 2100 using underlying mortality, a risk factor scalar, and an autoregressive integrated moving average (ARIMA) model. Net migration was modelled as a function of the Socio-demographic Index, crude population growth rate, and deaths from war and natural disasters; and use of an ARIMA model. The model framework was used to develop a reference scenario and alternative scenarios based on the pace of change in educational attainment and contraceptive met need. We estimated the size of gross domestic product for each country and territory in the reference scenario. Forecast uncertainty intervals (UIs) incorporated uncertainty propagated from past data inputs, model estimation, and forecast data distributions.

**Findings:**

The global TFR in the reference scenario was forecasted to be 1·66 (95% UI 1·33–2·08) in 2100. In the reference scenario, the global population was projected to peak in 2064 at 9·73 billion (8·84–10·9) people and decline to 8·79 billion (6·83–11·8) in 2100. The reference projections for the five largest countries in 2100 were India (1·09 billion [0·72–1·71], Nigeria (791 million [594–1056]), China (732 million [456–1499]), the USA (336 million [248–456]), and Pakistan (248 million [151–427]). Findings also suggest a shifting age structure in many parts of the world, with 2·37 billion (1·91–2·87) individuals older than 65 years and 1·70 billion (1·11–2·81) individuals younger than 20 years, forecasted globally in 2100. By 2050, 151 countries were forecasted to have a TFR lower than the replacement level (TFR <2·1), and 183 were forecasted to have a TFR lower than replacement by 2100. 23 countries in the reference scenario, including Japan, Thailand, and Spain, were forecasted to have population declines greater than 50% from 2017 to 2100; China's population was forecasted to decline by 48·0% (−6·1 to 68·4). China was forecasted to become the largest economy by 2035 but in the reference scenario, the USA was forecasted to once again become the largest economy in 2098. Our alternative scenarios suggest that meeting the Sustainable Development Goals targets for education and contraceptive met need would result in a global population of 6·29 billion (4·82–8·73) in 2100 and a population of 6·88 billion (5·27–9·51) when assuming 99th percentile rates of change in these drivers.

**Interpretation:**

Our findings suggest that continued trends in female educational attainment and access to contraception will hasten declines in fertility and slow population growth. A sustained TFR lower than the replacement level in many countries, including China and India, would have economic, social, environmental, and geopolitical consequences. Policy options to adapt to continued low fertility, while sustaining and enhancing female reproductive health, will be crucial in the years to come.

**Funding:**

Bill & Melinda Gates Foundation.

**Research in context**

**Evidence before this study**

Global population projections have been produced by the Population Division of the Department of Economic and Social Affairs of the UN Secretariat (UNPD) since the 1950s. For many years, UNPD used a deterministic model for fertility, mortality, and migration. Structural scenarios were also computed by assuming a fixed difference of 0·5 children in the total fertility rate (TFR) in each time period and country. Beginning in 2010, UNPD adopted a statistical model for the TFR and life expectancy as functions of calendar year and a deterministic model for migration. This blend of statistical models for two of the components of population growth has been used to generate uncertainty intervals (UIs). In fitting their global model for low fertility recovery, UNPD has excluded countries with sustained low fertility such as Thailand, South Korea, Canada, and Greece. Estimated in this way, the UNPD predicts TFRs will rebound to approximately 1·75 in all countries with TFR lower than the replacement level (<2·1).

Since the 1990s, the International Institute for Applied Systems Analysis–Wittgenstein Centre has generated alternative population projections. The Wittgenstein Centre fertility forecasts are a blend of expert opinions about future fertility patterns and statistical modelling. For low-fertility countries, they assume that the TFR will converge to 1·75 in the year 2200. Expert judgment is also used by the Wittgenstein Centre to set assumptions of future mortality, migration, and education that are combined with statistical modelling to produce future population scenarios. Their hybrid approach does not generate UIs for population projections. By accounting for educational attainment in the qualitative assessment, Wittgenstein predicts much faster declines in the TFR in sub-Saharan Africa than those by UNPD.

**Added value of this study**

In our study, we improved on UNPD and Wittgenstein forecasts in seven important ways. First, we modelled completed cohort fertility at age 50 years (CCF50) rather than the TFR. CCF50 is much less affected by the delay of childbearing that occurs as females become more educated, which leads the period measure of the TFR to initially decline to low levels and then increase. By contrast, completed cohort fertility rarely increases, making the modelling of CCF50 much more stable. Second, we modelled CCF50 as a function of educational attainment and contraceptive met need. These two variables alone account for 80·5% of the variance in CCF50 over time and location. Third, we used the causal model to explore the effect of faster or slower than expected changes in educational attainment and contraceptive met need. These scenarios, unlike structural scenarios, can provide direct guidance to policy debates on the impact of faster or slower scale-up of educational attainment or access to reproductive health services. Fourth, we leveraged the previously published future health scenarios model for cause-specific and all-cause mortality; this model also allows the effect of faster scale-up of educational attainment on mortality to be captured. Fifth, rather than assume deterministic patterns of migration, we fitted a time-series model with covariates (Socio-demographic Index, crude population growth rate, and deaths from war and natural disasters) to national net migration rates. By making explicit the pathways through which fertility, mortality, and migration patterns can change, our model is able to identify where future time trends might be different from past trends. Sixth, uncertainty in all three components (fertility, mortality, and migration) were propagated into the uncertainty distributions for each country and territory in each year. Seventh, we traced the changes in age structure expected in the reference and alternative scenarios on total gross domestic product (GDP) using previously published forecasts of GDP per adult of working age.

**Implications of all the available evidence**

Our reference forecast of the global population in 2100 was lower than the Wittgenstein Centre forecast and much lower than the UNPD forecast. Our findings suggest that, because of progress in female educational attainment and access to contraception contributing to declining fertility rates, continued global population growth through the century is no longer the most likely trajectory for the world's population. By contrast, world population might peak just after mid-century and substantially decline by 2100. The difference in population forecasts between our reference scenario and the UNPD forecasts is a third due to faster declines in sub-Saharan African fertility and two thirds due to the lower level of TFR expected in populations with fertility lower than the replacement level, especially China and India. Our findings show that some countries with fertility lower than replacement level, such as the USA, Australia, and Canada, will probably maintain their working-age populations through net immigration. Our forecasts for a shrinking global population have positive implications for the environment, climate change, and food production, but possible negative implications for labour forces, economic growth, and social support systems in parts of the world with the greatest fertility declines.

## Introduction

Population forecasts and scenarios are an important planning and risk management tool for governments, businesses, non-governmental organisations, and individuals. Governments need short-term and mid-term scenarios to estimate need for schools, hospitals, and other public services; to help inform infrastructure investments with long-term benefits; to plan for the necessary skills and knowledge for the future workforce; and to invest wisely in health research and development resources. Governments need long-term scenarios to understand potential environmental, military, geopolitical, and other risks and to implement prevention or mitigation strategies. Population scenarios are equally important for businesses that are engaged in investments with long-term returns, such as those in the pharmaceutical industry and in industries connected to heavy infrastructure projects. Likewise, individuals might have profound concerns over population in the future: will there be enough workers to pay taxes to support pension and health benefits for the retired? Will demographic change enhance global and national security and stability or make societies more precarious?

The main provider of population forecasts for the world since the 1950s is the Population Division of the Department of Economic and Social Affairs of the UN Secretariat (UNPD), which now produces regular forecasts for each country in 5-year calendar intervals such as 2095–2100.^1^ Since the 1950s, the people in the UNPD making forecasts and the methods used to develop forecasts have changed considerably. In 2010, the UNPD adopted a new statistical method to project fertility,^2^, ^3^ and in 2012 they adopted a new statistical method to project mortality.^4^, ^5^ While the methods for these crucial components are now based on statistical models fit to past data, UNPD forecasts of long-term migration for each country remain arbitrary assumptions without uncertainty. The UNPD's latest forecasts used time alone as the determinant of future trajectories for fertility and mortality; they are sophisticated curve-fitting exercises, which do not allow for alternative scenarios linked to policies or other drivers of fertility and mortality.^6^ In their latest revision, UNPD forecasted global population in 2100 to be 10·88 billion (95% prediction interval 9·42–12·66) and that of sub-Saharan Africa to be 3·78 billion (2·97–4·78).^1^

Population scenarios have also been developed by groups other than UNPD,^7^, ^8^, ^9^, ^10^ of which the most widely published are produced by the Austrian Wittgenstein Centre and collaborators.^7^, ^11^ However, Wittgenstein does not produce forecasts with uncertainty intervals (UIs). Their scenarios are a blend of statistical models fit to past data and expert judgment on likely trends in fertility, particularly regarding rising education levels in many parts of the world.^7^, ^12^ The Wittgenstein Centre forecasts have been the most widely used forecasts in climate modelling,^13^ although neither the climate models nor the Wittgenstein forecasts explicitly model the interrelationship between climate change and population.

Global population forecasts appear to depend primarily on two key issues: the pace of fertility decline in sub-Saharan Africa, and what happens to countries when fertility levels drop below a total fertility rate (TFR) of 2·1, traditionally considered the minimum rate necessary for generational replacement of the population (the replacement level). Wittgenstein Centre's reference population scenario assumes that countries with low fertility will slowly converge to a TFR of 1·75 by the year 2200;^11^ in this scenario, a location like Taiwan (province of China), with a TFR of 1·04 in 2017,^14^ will have a steady increase in fertility over the century. The UNPD fits a model to a selected set of countries with low fertility that have had fertility increases towards replacement level.^6^, ^15^ This results in a model that predicts convergence of fertility towards 1·75.^16^ Their statistical effort to calibrate a global model of low fertility recovery excludes several countries with a low fertility rate that has not shown any evidence of increasing, such as Thailand, Greece, South Korea, and Canada. In the past few years, several groups have questioned the validity of these assumptions about post-transition fertility.^17^, ^18^ Post-transition fertility refers to the fertility rate in countries and territories that have undergone what demographers describe as the demographic transition—a shift from high mortality and high fertility rates to low mortality and low fertility rates.^19^, ^20^ The challenge of modelling TFR in societies in which fertility has fallen below replacement level is compounded by fluctuations in TFR at low levels.^21^ However, this variable pattern is, in large part, due to TFR being a measure of the fertility of a hypothetical cohort of 15-year-old females subjected to present-day observed age-specific fertility rates and no mortality. Observed fertility rates for real cohorts of females do not seem to show the same fluctuating pattern.^21^

In this study, we addressed some of the limitations of these previous forecasting efforts. We extended the model in Foreman and colleagues^22^ for all-cause mortality to 2100. We developed statistical models for completed cohort fertility at age 50 years (CCF50) and age-specific fertility as a function of educational attainment and contraceptive met need, a measure of the proportion of women in a population of reproductive age whose need for contraception has been met with modern contraceptive methods. Finally, we developed a statistical model with uncertainty for net migration up to 2100. Because each model—mortality, fertility, and migration—has independent drivers, we explored alternative scenarios related to faster or slower changes in educational attainment and contraceptive met need. We traced potential economic and geopolitical consequences of the demographic shifts coming in this century.

## Methods

### Overview

We forecasted population from 2018 to 2100 for 195 countries and territories with the standard cohort-component method of projection, using estimates from the Global Burden of Diseases, Injuries, and Risk Factors Study (GBD) 2017.^14^ This approach uses inputs of population by age and sex in 2017, sex ratios at birth in 2017 (kept constant through 2100), and forecasts for age-specific fertility rates, age-specific mortality rates, and net migration for all locations through 2100. This analysis complies with the Guidelines on Accurate and Transparent Health Estimate Reporting (appendix 1, section 2).^23^ All code used in the analysis can be found online. We describe here the modelling used to generate forecasts of fertility, all-cause mortality, migration, and population. Additional details are presented in appendix 1, and a detailed description of the cause-specific mortality forecasting framework was reported previously.^22^ Uncertainty in past data inputs, covariate and health driver forecasts, and estimated model parameters were propagated by combining draw-level data from GBD 2017 with draws from the forecast-generating model incorporating, when feasible, parameter draws from estimated sampling or posterior distributions. This approach captures uncertainty in each modelling stage and propagates it through the entire forecasting framework. Point estimates were computed as the mean of 1000 draws from the corresponding draw distribution and 95% UIs were computed with use of the 2·5 and 97·5 percentiles.

### Mortality

We used the mortality model previously published by Foreman and colleagues,^22^ and extended it to 2100 with slight modifications. Briefly, the cause-specific model included three components: the underlying mortality, modelled as a function of the Socio-demographic Index (SDI), time, and additional cause-specific covariates where appropriate; a risk factor scalar that captured the combined risk factor effects for specific causes, based on the GBD 2017 cause-risk hierarchy and accounting for risk factor mediation;^24^ and an autoregressive integrated moving average (ARIMA) model^25^ that accounted for unexplained residual mortality.

To accommodate long-range forecasts, we removed the spline on SDI and used a random walk with attenuated drift for the ARIMA model. Foreman and colleagues found that our mortality model had better out-of-sample predictive validity than the most widely used demographic forecasting model.^22^ The method used to develop reference scenario values for each of the independent drivers in the mortality model was not modified from Foreman and colleagues.^22^

### Fertility

#### Modelling CCF50 versus TFR

Previous forecasting studies have forecasted TFR and then used assumed age patterns of fertility to estimate age-specific fertility rates.^15^, ^22^, ^26^ Forecasting declines in fertility when a nation has a TFR higher than the replacement level is fairly straightforward given the strong relationship between fertility rates of decline and variables such as maternal education and access to reproductive health services.^3^ However, when TFR is lower than the replacement level, trends are more complex, with several countries showing declines followed by upturns and others such as Singapore showing declines followed by stagnation at very low levels. Because females tend to delay marriage and childbirth as they become more educated and enter the labour force, the TFR as a period measure often declines and then increases, even though completed fertility over the course of a reproductive lifespan for any cohort of females is still declining or stagnant.

For this reason, we modelled fertility with use of CCF50. CCF50 is defined as the average number of children born to an individual female from an observed birth cohort if she lived to the end of her reproductive lifespan (age 15–49 years). An illustration of TFR and CCF50 for five countries is shown in appendix 2 (section 5). These countries were selected to show the relative stability of CCF50 and TFR in countries with high fertility rates versus those with low fertility rates. In countries with low fertility, CCF50 declined or remained constant and did not manifest the fluctuating pattern seen with TFR. In the period of 1965–2017, in countries with fertility lower than replacement level, the TFR increased in 29·4% of country-years, whereas the CCF50 increased only in 13·9% of country-years.

#### The CCF50 model

We modelled CCF50 for females in birth cohort *c* in location *l*, denoted by *CCF*_l,c_ using the regression model given by:

$$CCF_{l,c}=\beta_{0}+\beta_{mn}mn_{l,c}+ns(edu_{l,c})+\eta_{l,c},$$

where β_0_ is an intercept, β_mn_ is a slope on the proportion of contraceptive met need, *ns(edu*_l,c_*)* represents a natural cubic spline applied to average female educational attainment, and η_l,c_ is a residual term modelled by use of a random walk (ARIMA_(0,1,0)_) in logit space (bounded between 1 and 10). Our model used the proportion of contraceptive met need (*mn*_l,c_) and average educational attainment (*edu*_l,c_) at age 25 years for each cohort and location, because we found that this was the most relevant age for explaining CCF50.

**Figure 1:**
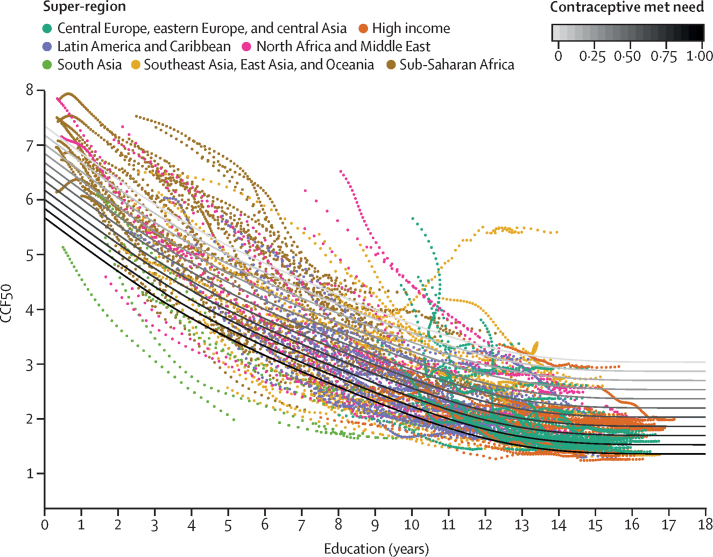
Model fit for CCF50 CCF50-fitted trends are presented as a function of education across varying levels of contraceptive met need. Each point represents a single location-year of past data, and they are coloured by GBD 2017 super-regions. Education is measured in years of attainment (0–18 years), and contraceptive met need is measured on a scale of 0% to 100%, reflecting the portion of the female population whose need for contraception has been met. CCF50=completed cohort fertility by age 50 years. GBD=Global Burden of Diseases, Injuries, and Risk Factors Study.

Figure 1 shows CCF50 plotted against educational attainment and contraceptive met need, with the model fit shown in both. This model with just two variables accounted for 80·5% of the variance in CCF50 across all countries over a 48-year period. We also investigated the inclusion of urbanicity (defined here as the proportion of the population living in an urban area for each location) as an additional covariate in the model, but it did not provide a substantial improvement to the model fit (0·01 increase in the adjusted R^2^). We developed statistical models for age-specific fertility rates within a cohort as a function of CCF50. More details on the modelling steps are available in appendix 1 (section 5). We also assessed the out-of-time predictive validity of our model and compared it with alternative statistical models^3^, ^27^ used by UNPD since 2012. Our model has smaller forecast errors than those of the alternatives (appendix 2, section 5).

### Migration

We modelled net migration rates as a function of SDI, death due to conflict and natural disasters, and the difference between birth and death rates, as well as a random walk with drift attenuation. We used 2017 UN data for past migration.^28^ Details of the model specification are provided in appendix 1 (section 7). We should note that migration forecasts for each country and territory have large UIs.

### Independent drivers

Contraceptive met need and educational attainment were modelled by applying location-specific annualised rates of change from past years, weighted by recency. Details of this model are described in appendix 1 (section 4), as are details of the models used for additional independent drivers in the mortality model. Contraceptive met need refers to the proportion of women who are using, or whose sexual partner is using, a method of modern contraception, from among those who are fertile and sexually active and who report not wanting children or more children or wanting to delay having a child. Met need lower than 100% shows a gap between reproductive intentions and behaviour.^29^

### Population

Each location's population was projected separately, starting from a mid-year estimate of population in 2017. The cohort-component method of projection used weekly time steps to align with GBD's youngest age groups. This required disaggregating the initial population into 1-week age groups for projection.^30^ Mortality rate, fertility rate, and migration were considered constant for each age, sex, and location during a calendar year. Additional details of the life table calculation are described in appendix 1 (section 9).

### Alternate scenarios

In addition to the reference scenario, we developed four alternative scenarios that reflected faster or slower trajectories for two key drivers of fertility rates: education of females, and access to modern reproductive health services, measured by contraceptive met need.^31^ The slower, faster, and fastest alternate scenarios were derived by setting the annualised rate of change for education and contraceptive met need to their respective 15th, 85th, and 99th percentile rates of change across locations in the period 1990–2017. For the UN Sustainable Development Goal (SDG) pace alternate scenario, we set a rate of change to one that would allow all locations to meet the SDG targets for educational attainment (universal secondary education by 2030)^32^ and contraceptive met need (universal coverage by 2030).^33^ We held those rates constant past 2030 in the education SDG scenario, and held contraceptive met need at 100% coverage past 2030 (appendix 1, section 10). This scenario shows what we can expect population trends to look like if every country and territory meets the SDGs for education and contraceptive met need by 2030. Many countries are not on track to achieve these goals.^34^

### GDP forecasts

We traced the economic consequences of population scenarios using the work of Chang and colleagues.^35^ They forecasted gross domestic product (GDP) per working-age adult and showed that forecasts of GDP per capita have smaller prediction errors when modelling GDP per working-age adult and multiplying by the number of working-age adults than those through direct modelling of GDP per capita. Using their long-range GDP per working-age adult forecasts, we computed GDP for each country and territory at various points in time for each of the scenarios.

### Comparison with other models

For comparison, we evaluated our reference scenario against the UNPD median variant and Wittgenstein SSP2 (medium) scenarios (see appendix 2, sections 7–9 for more details).

### Role of the funding source

The funders had no role in study design, data collection, data analysis, data interpretation, or writing of the report. All authors had full access to all the data in the study and had final responsibility for the decision to submit for publication.

## Results

### Global mortality scenarios

**Figure 2:**
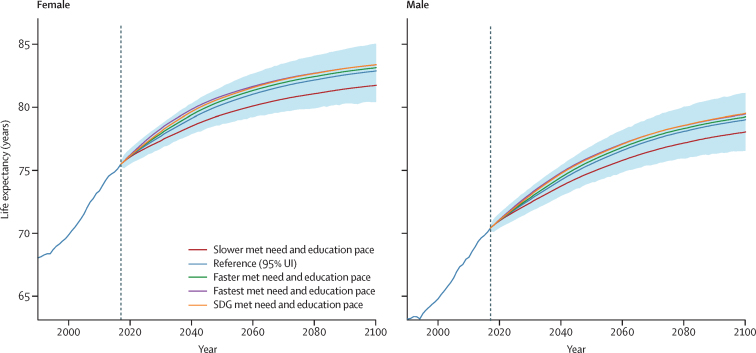
Global life expectancy in the reference, slower, faster, fastest, and SDG pace scenarios, 1990–2100 The reference scenario is presented with 95% UIs, which are represented by the shaded area. Life expectancy was computed at birth, and values are reported in years. Past estimates are from GBD 2017. GBD=Global Burden of Diseases, Injuries, and Risk Factors Study. SDG=Sustainable Development Goal. UI=uncertainty interval.

We plotted the evolution of global life expectancy in the reference scenario and in the slower, faster, fastest, and SDG pace alternate scenarios reflecting education attainment and contraceptive met need (figure 2). Although life expectancy was forecasted to increase, the rate of progress is likely to slow. Large inequalities remained at the global level in 2100, with forecasts of country and territory life expectancies for both sexes combined ranging from 69·4 years (95% UI 61·4–76·0) to 88·9 years (85·0–92·6) in the reference scenario (appendix 2, section 3). The standard deviation of life expectancy across countries and territories narrowed from 6·9 years in 2017 to 3·6 years in 2100 (data not shown). Ten countries were forecasted to still have life expectancies lower than 75 years in 2100, seven of them in sub-Saharan Africa. The range of global life expectancy in 2100 across scenarios was moderate (from 79·9 years [95% UI 77·4–82·0] in the slower scenario to 81·4 years [79·0–83·5] in the SDG pace scenario; appendix 2, section 3). Appendix 2 has additional life expectancy results (section 3) and life tables for all locations in 2017 and 2100 (section 13).

### Global fertility scenarios

**Figure 3:**
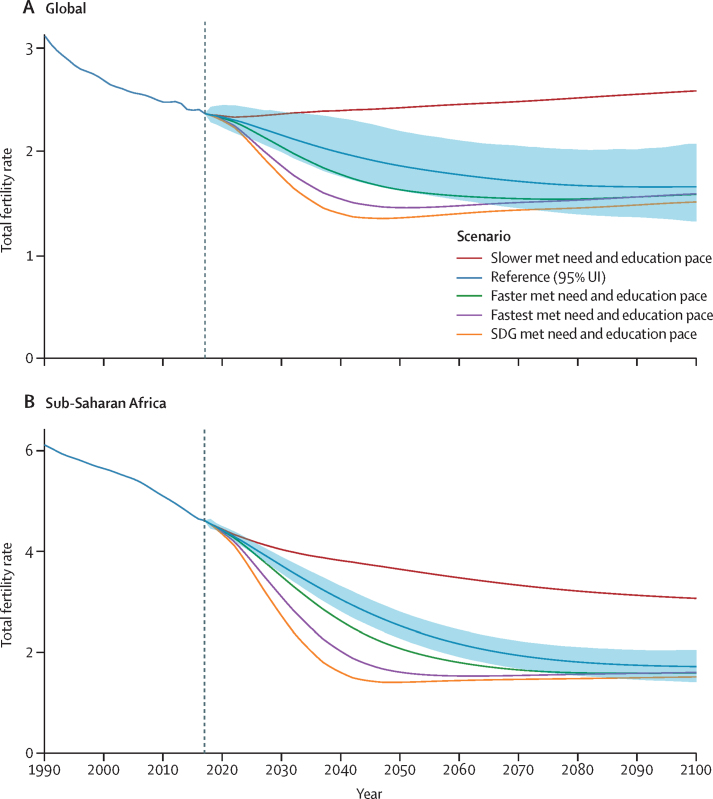
Global and sub-Saharan African total fertility rates, 1990–2100 Past data reflect GBD 2017 estimates, with future results for the reference, slower, faster, fastest, and SDG pace scenarios. The reference scenario is presented with 95% UIs, which are represented by the shaded area. GBD=Global Burden of Diseases, Injuries, and Risk Factors Study. SDG=Sustainable Development Goal. UI=uncertainty interval.

**Figure 4:**
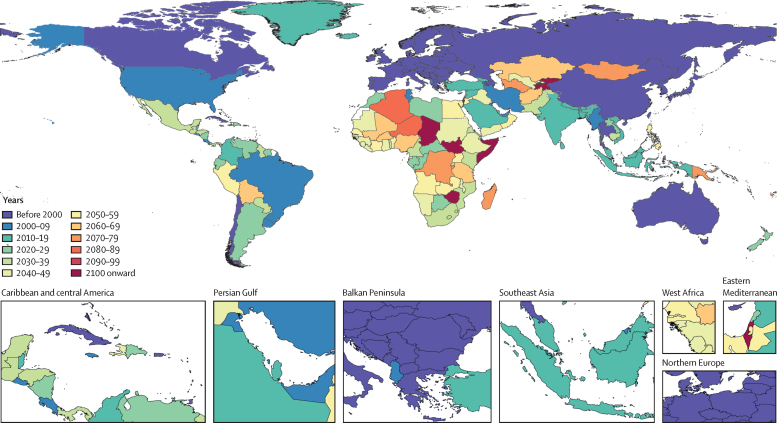
Map of the year that the net reproduction rate falls below the replacement level Replacement is defined as a net reproduction rate of 1. Past estimates are calculated on the basis of GBD 2017 estimates and future estimates are calculated from the reference forecast. GBD=Global Burden of Diseases, Injuries, and Risk Factors Study.

The global TFR in the reference scenario declined steadily, reaching 1·66 (95% UI 1·33–2·08) in 2100 (table, figure 3). The range in global TFR was wide across the five scenarios, from 1·52 (1·15–1·99) in the SDG pace scenario to 2·59 (2·27–2·93) in the slower pace scenario (appendix 2, section 54). The slight increase in TFR in the slower scenario was due not to increasing TFR in any particular location but to the progressive shift of the global birth cohort to locations with higher fertility rates. In the slower scenario, the world forecast for this century did not drop below replacement fertility levels. The global TFR forecast dropped below the replacement level (a rate of 2·1) in 2034 in the reference scenario, and earlier in the faster (2029), fastest (2026), and SDG (2025) scenarios. The difference in TFR between the faster and slower scenarios revealed the great effect of educational attainment and the provision of reproductive health services on the global trajectory of fertility. The SDG pace scenario reached a minimum global TFR forecast around mid-century of 1·36 (1·14–1·70), which increased to 1·52 (1·15–1·99) by 2100 (table). Similar, but less pronounced, rebounds were seen for the fastest and faster scenarios. Because we estimated no upturn in future CCF50 at the national level, these upturns are partly explained by changes in maternal age at birth over time and by shifts of the global birth cohort to higher-fertility locations. Sub-Saharan Africa was forecasted to have the highest fertility rates among the super regions, staying above replacement level until 2063 (figure 3B). We mapped the years in which the TFRs of countries dropped or were forecast to drop below replacement levels (figure 4); for this map, we used the net reproductive rate, because this also accounts for sex imbalance in births. Countries such as China or India have a lower effective fertility rate than the TFR would suggest because the sex ratio at birth is skewed toward boys (additional results in appendix 2, section 5). Additional fertility results are available online.

**Table:** Population and total fertility rate in 2017, in 2100 with the reference scenario, and in 2100 with the SDG pace scenario and the year of peak population

		**Population (millions)**				**Total fertility rate**		
		2017	2100 reference scenario	2100 SDG pace scenario	Peak population (year)	2017	2100 reference scenario	2100 SDG pace scenario
**Global**		**7640·47 (7394·65–7867·14)**	**8785·55 (6825·31–11829·48)**	**6289·42 (4821·12–8733·4)**	**9732·92 (2064)**	**2·37 (2·22–2·55)**	**1·66 (1·33–2·08)**	**1·52 (1·15–1·99)**
**Central Europe, eastern Europe, and central Asia**		**415·93 (395·18–435·49)**	**324·99 (217·65–498·18)**	**248·22 (171·88–387·22)**	**417·71 (2023)**	**1·78 (1·59–1·99)**	**1·80 (1·25–2·40)**	**1·58 (1·07–2·23)**
Central Asia		90·93 (83·02–99·04)	138·93 (85·13–221·17)	91·14 (54·62–153·13)	138·93 (2100)	2·47 (2·26–2·69)	2·14 (1·5–2·83)	1·89 (1·23–2·65)
	Armenia	3·03 (2·7–3·35)	1·78 (0·9–3·83)	1·33 (0·83–2·83)	3·04 (2022)	1·58 (1·44–1·72)	1·43 (0·96–2·51)	1·27 (0·95–2·24)
	Azerbaijan	10·23 (8·96–11·43)	8·69 (4·42–17·67)	5·75 (3·6–11·91)	11·46 (2045)	1·96 (1·73–2·23)	1·50 (0·98–2·45)	1·29 (0·96–2·17)
	Georgia	3·69 (3·37–4·04)	2·78 (1·39–5·26)	1·85 (1·06–3·56)	3·69 (2017)	2·05 (1·86–2·25)	1·59 (1·01–2·55)	1·38 (0·95–2·25)
	Kazakhstan	17·90 (16·48–19·23)	30·28 (17·99–48·62)	23·24 (13·87–38·52)	30·28 (2100)	2·39 (2·16–2·63)	1·97 (1·29–2·8)	1·81 (1·12–2·63)
	Kyrgyzstan	6·37 (5·59–7·10)	12·45 (6·11–23·68)	6·03 (2·56–12·97)	12·45 (2100)	2·78 (2·59–2·97)	2·25 (1·4–3·29)	1·67 (1·0–2·67)
	Mongolia	3·25 (2·87–3·62)	6·07 (1·78–19·73)	4·41 (1·47–14·43)	6·07 (2100)	2·70 (2·49–2·91)	1·98 (0·98–4·27)	1·81 (0·96–3·96)
	Tajikistan	9·24 (8·19–10·25)	23·76 (14·82–34·95)	8·20 (4·47–13·75)	23·76 (2100)	3·55 (3·22–3·87)	2·25 (1·68–2·86)	1·54 (1·03–2·14)
	Turkmenistan	4·98 (4·56–5·4)	8·96 (3·49–21·58)	5·94 (2·58–15·39)	8·96 (2100)	2·76 (2·47–3·11)	1·91 (1·01–3·51)	1·66 (0·97–3·19)
	Uzbekistan	32·24 (24·56–39·91)	44·18 (23·4–76·27)	34·38 (17·71–60·95)	44·99 (2076)	2·35 (2·14–2·57)	1·89 (1·23–2·65)	1·75 (1·12–2·54)
Central Europe		114·80 (112·04–117·49)	52·30 (39·66–70·26)	44·51 (34·98–59·28)	114·80 (2017)	1·43 (1·29–1·59)	1·35 (1·04–1·74)	1·25 (0·96–1·65)
	Albania	2·77 (2·47–3·07)	1·97 (1·41–2·7)	1·32 (1·02–1·81)	2·86 (2031)	1·88 (1·63–2·18)	1·49 (1·08–1·97)	1·17 (0·96–1·55)
	Bosnia and Herzegovina	3·40 (3·09–3·72)	1·42 (1·13–1·72)	1·19 (0·97–1·45)	3·40 (2017)	1·26 (1·17–1·36)	1·19 (1·0–1·45)	1·09 (0·97–1·31)
	Bulgaria	7·05 (6·53–7·58)	2·62 (1·82–3·89)	2·28 (1·71–3·3)	7·05 (2017)	1·47 (1·29–1·67)	1·25 (0·95–1·76)	1·21 (0·94–1·72)
	Croatia	4·28 (3·83–4·73)	1·62 (1·27–2·00)	1·22 (1·00–1·48)	4·28 (2017)	1·37 (1·3–1·45)	1·39 (1·13–1·69)	1·15 (0·98–1·37)
	Czech Republic	10·59 (10·52–10·67)	6·73 (4·95–9·24)	6·04 (4·67–8·26)	10·60 (2020)	1·58 (1·45–1·73)	1·37 (0·99–1·87)	1·31 (0·96–1·82)
	Hungary	9·73 (8·74–10·79)	5·20 (3·62–7·99)	4·53 (3·40–6·82)	9·73 (2017)	1·43 (1·26–1·61)	1·38 (0·98–2·01)	1·25 (0·95–1·86)
	Montenegro	0·63 (0·56–0·69)	0·44 (0·38–0·52)	0·30 (0·25–0·34)	0·63 (2022)	1·67 (1·59–1·76)	1·62 (1·42–1·82)	1·18 (1·03–1·34)
	North Macedonia	2·17 (1·82–2·52)	1·27 (1·07–1·51)	0·92 (0·8–1·09)	2·19 (2024)	1·51 (1·41–1·61)	1·39 (1·2–1·62)	1·11 (0·97–1·3)
	Poland	38·39 (38·12–38·67)	15·42 (11·67–20·66)	13·66 (10·87–18·07)	38·39 (2017)	1·31 (1·16–1·48)	1·17 (0·96–1·55)	1·14 (0·96–1·5)
	Romania	19·43 (17·34–21·55)	7·77 (5·02–13·3)	6·60 (4·62–11·17)	19·43 (2017)	1·56 (1·4–1·74)	1·28 (0·92–2·0)	1·24 (0·93–1·93)
	Serbia	8·87 (7·85–9·84)	4·14 (3·31–5·14)	3·18 (2·64–3·85)	8·87 (2017)	1·37 (1·21–1·56)	1·34 (1·09–1·62)	1·13 (0·96–1·36)
	Slovakia	5·42 (5·01–5·82)	2·56 (1·93–3·47)	2·29 (1·80–3·08)	5·42 (2018)	1·39 (1·23–1·58)	1·30 (0·99–1·70)	1·24 (0·97–1·65)
	Slovenia	2·07 (2·05–2·09)	1·15 (0·89–1·50)	0·99 (0·79–1·28)	2·07 (2018)	1·52 (1·39–1·66)	1·36 (1·04–1·77)	1·26 (0·98–1·62)
Eastern Europe		210·20 (192·44–228·28)	133·75 (81·62–226·07)	112·57 (73·79–190·72)	210·20 (2017)	1·55 (1·35–1·79)	1·45 (0·94–2·25)	1·34 (0·88–2·14)
	Belarus	9·49 (8·37–10·56)	5·53 (3·72–8·89)	4·91 (3·46–7·84)	9·49 (2017)	1·57 (1·4–1·78)	1·40 (0·99–2·08)	1·34 (0·96–1·96)
	Estonia	1·31 (1·3–1·33)	0·82 (0·56–1·24)	0·70 (0·49–1·04)	1·31 (2017)	1·57 (1·38–1·79)	1·48 (1·0–2·13)	1·36 (0·96–2·02)
	Latvia	1·95 (1·93–1·96)	0·43 (0·22–0·77)	0·34 (0·17–0·66)	1·95 (2017)	1·58 (1·39–1·79)	1·37 (0·96–2·03)	1·31 (0·93–1·92)
	Lithuania	2·85 (2·83–2·87)	1·47 (0·92–2·33)	1·24 (0·85–2·01)	2·85 (2017)	1·62 (1·44–1·82)	1·44 (0·99–2·16)	1·37 (0·96–2·07)
	Moldova	3·72 (3·15–4·28)	1·51 (0·94–2·51)	1·27 (0·88–2·0)	3·72 (2017)	1·31 (1·16–1·49)	1·32 (0·97–1·96)	1·19 (0·94–1·8)
	Russia	146·19 (129·8–162·59)	106·45 (66·58–178·81)	89·37 (59·76–155·49)	146·19 (2017)	1·61 (1·39–1·85)	1·43 (0·96–2·23)	1·32 (0·92–2·12)
	Ukraine	44·69 (37·16–51·85)	17·55 (11·25–28·24)	14·74 (10·32–22·82)	44·69 (2017)	1·40 (1·22–1·61)	1·32 (0·96–1·92)	1·20 (0·94–1·77)
**High income**		**1074·89 (1033·31–1116·72)**	**956·89 (763·16–1215·89)**	**827·90 (667·17–1052·54)**	**1135·08 (2041)**	**1·67 (1·52–1·84)**	**1·59 (1·25–1·98)**	**1·45 (1·12–1·83)**
Australasia		28·39 (26·43–30·17)	42·35 (31·04–58·1)	36·02 (26·53–48·89)	42·39 (2096)	1·89 (1·71–2·09)	1·71 (1·22–2·29)	1·55 (1·07–2·11)
	Australia	23·94 (22·09–25·64)	36·34 (26·99–49·76)	31·06 (23·32–41·75)	36·38 (2096)	1·86 (1·65–2·09)	1·69 (1·19–2·26)	1·52 (1·06–2·07)
	New Zealand	4·45 (4·04–4·85)	6·01 (3·82–9·5)	4·97 (3·31–8·06)	6·01 (2095)	2·10 (1·92–2·32)	1·71 (1·07–2·54)	1·56 (1·01–2·4)
High-income Asia Pacific		187·03 (175·66–198·89)	93·70 (69·78–137·27)	84·10 (66·98–121·59)	187·03 (2017)	1·30 (1·15–1·47)	1·33 (0·95–1·97)	1·23 (0·91–1·82)
	Brunei	0·43 (0·39–0·48)	0·42 (0·25–0·68)	0·30 (0·2–0·48)	0·53 (2050)	1·88 (1·74–2·03)	1·67 (1·06–2·44)	1·37 (0·99–2·06)
	Japan	128·36 (118·33–139·14)	59·72 (42·89–91·91)	52·69 (42·12–79·96)	128·36 (2017)	1·33 (1·11–1·59)	1·32 (0·96–2·03)	1·20 (0·95–1·85)
	Singapore	5·57 (4·91–6·19)	6·78 (5·4–9·68)	6·39 (5·23–9·04)	8·04 (2062)	1·26 (1·05–1·52)	1·27 (0·91–1·99)	1·22 (0·89–1·95)
	South Korea	52·67 (48·44–56·79)	26·78 (20·94–36·35)	24·72 (20·26–32·79)	54·29 (2031)	1·24 (1·18–1·30)	1·24 (0·94–1·78)	1·20 (0·92–1·68)
High-income North America		360·88 (324·22–398·6)	379·95 (282·96–509·77)	322·69 (244·94–432·12)	408·40 (2064)	1·79 (1·65–1·95)	1·54 (1·13–2·03)	1·40 (1·03–1·86)
	Canada	35·98 (33·29–38·59)	44·09 (36·8–53·16)	37·06 (32·09–43·28)	45·17 (2078)	1·66 (1·42–1·94)	1·58 (1·29–1·90)	1·37 (1·12–1·64)
	Greenland	0·06 (0·06–0·06)	0·05 (0·03–0·07)	0·03 (0·02–0·05)	0·06 (2039)	2·02 (1·79–2·26)	1·52 (1·08–2·06)	1·29 (0·99–1·79)
	USA	324·84 (288·6–362·83)	335·81 (247·53–456·32)	285·59 (215·82–385·94)	363·75 (2062)	1·81 (1·68–1·95)	1·53 (1·1–2·02)	1·40 (1·02–1·86)
Southern Latin America		65·61 (60·27–70·62)	66·50 (46·02–93·09)	55·32 (38·37–78·46)	78·02 (2057)	2·06 (1·9–2·25)	1·58 (1·14–2·06)	1·48 (1·05–1·98)
	Argentina	44·27 (39·11–49·25)	48·27 (31·99–70·54)	39·62 (26·4–57·99)	54·59 (2062)	2·17 (2·02–2·33)	1·62 (1·14–2·13)	1·51 (1·04–2·06)
	Chile	17·92 (16·67–19·08)	15·52 (11·73–20·64)	13·43 (10·59–17·41)	20·29 (2046)	1·81 (1·59–2·06)	1·37 (1·04–1·78)	1·29 (1·01–1·68)
	Uruguay	3·42 (3·06–3·77)	2·71 (2·07–3·51)	2·27 (1·77–2·92)	3·60 (2042)	1·97 (1·72–2·27)	1·44 (1·16–1·76)	1·37 (1·08–1·70)
Western Europe		432·97 (420·94–445·9)	374·39 (303·66–465·27)	329·75 (270·49–410·49)	447·92 (2038)	1·59 (1·43–1·78)	1·64 (1·28–2·05)	1·50 (1·16–1·88)
	Andorra	0·08 (0·08–0·08)	0·03 (0·03–0·04)	0·03 (0·03–0·04)	0·08 (2021)	1·20 (1·06–1·35)	1·23 (1·02–1·50)	1·17 (1·0–1·44)
	Austria	8·79 (8·73–8·86)	6·58 (5·22–8·37)	6·01 (4·83–7·60)	9·07 (2033)	1·51 (1·38–1·66)	1·37 (1·02–1·76)	1·31 (1·00–1·71)
	Belgium	11·32 (11·23–11·41)	13·48 (10·41–17·46)	11·57 (9·08–14·8)	13·63 (2084)	1·69 (1·52–1·87)	1·60 (1·21–2·06)	1·46 (1·05–1·9)
	Cyprus	1·26 (1·14–1·39)	0·79 (0·66–1·00)	0·76 (0·64–0·96)	1·37 (2038)	1·01 (0·87–1·17)	1·18 (0·96–1·52)	1·16 (0·95–1·49)
	Denmark	5·73 (5·68–5·78)	6·06 (4·17–8·66)	4·88 (3·66–6·96)	6·24 (2071)	1·75 (1·57–1·95)	1·66 (1·08–2·31)	1·39 (0·99–1·98)
	Finland	5·52 (5·47–5·56)	5·24 (4·04–6·70)	4·59 (3·61–5·83)	5·73 (2038)	1·64 (1·47–1·83)	1·60 (1·22–2·01)	1·47 (1·1–1·85)
	France	65·71 (59·68–71·56)	67·15 (53·33–85·37)	60·13 (47·63–76·53)	70·64 (2046)	1·84 (1·66–2·05)	1·78 (1·42–2·20)	1·65 (1·28–2·07)
	Germany	83·29 (74·7–92·02)	66·42 (53·96–80·86)	60·06 (49·98–72·53)	85·08 (2035)	1·39 (1·24–1·57)	1·35 (1·05–1·69)	1·26 (1·0–1·58)
	Greece	10·40 (9·3–11·47)	5·48 (4·07–7·64)	4·73 (3·78–6·5)	10·40 (2017)	1·42 (1·27–1·6)	1·29 (0·97–1·82)	1·19 (0·97–1·68)
	Iceland	0·34 (0·33–0·34)	0·38 (0·25–0·56)	0·32 (0·21–0·49)	0·40 (2063)	1·83 (1·68–2·00)	1·72 (1·16–2·40)	1·59 (1·04–2·25)
	Ireland	4·86 (4·52–5·22)	5·44 (3·76–8·27)	4·82 (3·3–7·29)	5·77 (2057)	1·84 (1·64–2·07)	1·68 (1·1–2·46)	1·57 (1·01–2·32)
	Israel	8·95 (7·82–10·12)	24·07 (13·89–41·48)	17·65 (10·4–30·45)	24·07 (2100)	2·90 (2·64–3·19)	2·36 (1·45–3·54)	2·05 (1·19–3·19)
	Italy	60·60 (60·15–61·03)	30·54 (24·61–39·44)	27·79 (23·41–35·51)	60·60 (2017)	1·33 (1·18–1·5)	1·23 (0·99–1·64)	1·17 (0·98–1·58)
	Luxembourg	0·59 (0·59–0·6)	0·71 (0·57–0·88)	0·64 (0·52–0·79)	0·77 (2063)	1·48 (1·35–1·61)	1·50 (1·13–1·9)	1·39 (1·06–1·79)
	Malta	0·43 (0·39–0·48)	0·29 (0·23–0·36)	0·26 (0·22–0·33)	0·44 (2027)	1·49 (1·32–1·68)	1·27 (1·0–1·64)	1·21 (0·96–1·57)
	Netherlands	17·03 (16·89–17·18)	13·58 (10·59–17·52)	11·15 (8·92–13·76)	17·50 (2033)	1·66 (1·49–1·85)	1·59 (1·24–2·01)	1·39 (1·06–1·73)
	Norway	5·26 (5·22–5·31)	7·47 (5·15–10·95)	6·47 (4·65–9·82)	7·47 (2099)	1·74 (1·59–1·9)	1·67 (1·09–2·35)	1·52 (1·02–2·28)
	Portugal	10·68 (9·53–11·86)	4·50 (3·43–6·1)	4·16 (3·29–5·68)	10·68 (2017)	1·29 (1·14–1·48)	1·26 (0·98–1·73)	1·21 (0·97–1·7)
	Spain	46·39 (42·86–49·88)	22·91 (17·89–32·95)	21·54 (17·39–30·84)	46·43 (2019)	1·35 (1·23–1·49)	1·24 (0·96–1·83)	1·21 (0·96–1·78)
	Sweden	10·04 (9·34–10·73)	13·11 (9·77–17·63)	10·72 (8·2–14·34)	13·11 (2100)	1·84 (1·69–1·99)	1·72 (1·24–2·31)	1·46 (1·02–2·01)
	Switzerland	8·59 (7·91–9·21)	8·33 (7·09–9·84)	7·39 (6·42–8·52)	9·82 (2048)	1·50 (1·34–1·67)	1·43 (1·17–1·73)	1·28 (1·04–1·54)
	UK	66·64 (60·8–72·58)	71·45 (55·76–90·29)	63·74 (50·26–82·78)	74·87 (2063)	1·73 (1·55–1·94)	1·61 (1·21–2·04)	1·50 (1·13–1·96)
**Latin America and Caribbean**		**581·95 (553·22–607·72)**	**575·16 (429·01–787·74)**	**467·80 (348·39–651·4)**	**715·51 (2055)**	**2·18 (1·99–2·4)**	**1·58 (1·22–2·01)**	**1·50 (1·13–1·94)**
Andean Latin America		61·45 (59·14–63·65)	93·29 (58·24–148·05)	69·71 (42·09–112·88)	97·44 (2078)	2·82 (2·57–3·1)	1·79 (1·17–2·51)	1·69 (1·09–2·39)
	Bolivia	11·54 (10·29–12·73)	23·45 (13·29–40·55)	15·82 (8·81–28·66)	23·46 (2099)	3·24 (2·92–3·61)	1·79 (1·07–2·71)	1·66 (1·03–2·59)
	Ecuador	16·69 (14·86–18·48)	18·02 (9·97–32·78)	14·54 (8·8–25·8)	21·89 (2060)	2·27 (1·93–2·67)	1·47 (0·99–2·25)	1·39 (0·98–2·14)
	Peru	33·22 (33·06–33·36)	51·81 (31·48–85·24)	39·34 (22·54–66·38)	53·92 (2079)	2·96 (2·64–3·33)	1·75 (1·1–2·61)	1·63 (1·04–2·44)
Caribbean		46·27 (43·67–48·92)	31·75 (18·27–54·94)	22·94 (13·48–41·52)	50·19 (2040)	2·24 (2·05–2·44)	1·55 (1·08–2·22)	1·51 (1·02–2·2)
	Antigua and Barbuda	0·09 (0·08–0·1)	0·06 (0·04–0·07)	0·05 (0·04–0·06)	0·10 (2037)	1·51 (1·28–1·78)	1·26 (1·01–1·57)	1·22 (1·0–1·54)
	The Bahamas	0·38 (0·33–0·42)	0·28 (0·19–0·41)	0·24 (0·17–0·35)	0·42 (2041)	1·54 (1·28–1·85)	1·38 (1·0–1·88)	1·32 (0·98–1·78)
	Barbados	0·30 (0·26–0·33)	0·18 (0·13–0·25)	0·15 (0·12–0·21)	0·30 (2031)	1·43 (1·2–1·7)	1·36 (1·02–1·78)	1·22 (0·99–1·64)
	Belize	0·39 (0·35–0·44)	0·51 (0·35–0·76)	0·44 (0·32–0·65)	0·60 (2066)	2·23 (1·96–2·53)	1·32 (1·0–1·9)	1·28 (0·99–1·83)
	Bermuda	0·07 (0·06–0·07)	0·03 (0·03–0·05)	0·03 (0·02–0·04)	0·07 (2022)	1·30 (1·16–1·46)	1·33 (1·01–1·76)	1·27 (1·0–1·7)
	Cuba	11·38 (10·25–12·44)	4·52 (2·49–9·0)	4·07 (2·37–8·24)	11·38 (2017)	1·51 (1·43–1·6)	1·41 (0·98–2·24)	1·35 (0·97–2·15)
	Dominica	0·07 (0·06–0·08)	0·04 (0·03–0·08)	0·04 (0·02–0·07)	0·07 (2033)	1·60 (1·35–1·92)	1·41 (0·99–2·16)	1·35 (0·99–2·14)
	Dominican Republic	10·45 (9·31–11·57)	7·73 (4·22–13·55)	5·52 (2·82–10·34)	12·09 (2047)	2·37 (2·04–2·75)	1·46 (1·01–2·1)	1·39 (1·0–2·01)
	Grenada	0·11 (0·1–0·12)	0·07 (0·04–0·13)	0·06 (0·04–0·11)	0·11 (2036)	1·88 (1·59–2·23)	1·43 (0·99–2·24)	1·37 (0·98–2·17)
	Guyana	0·74 (0·67–0·82)	0·59 (0·38–0·84)	0·29 (0·18–0·44)	0·83 (2048)	2·50 (2·16–2·88)	1·61 (1·27–1·95)	1·31 (1·02–1·65)
	Haiti	11·82 (9·85–13·74)	12·94 (5·93–26·7)	8·19 (4·28–18·56)	16·25 (2058)	3·14 (2·81–3·52)	1·42 (0·98–2·42)	1·39 (0·99–2·39)
	Jamaica	2·78 (2·47–3·08)	0·85 (0·41–1·63)	0·71 (0·33–1·39)	2·78 (2023)	1·58 (1·39–1·79)	1·36 (1·0–1·88)	1·31 (1·0–1·87)
	Puerto Rico	3·67 (3·24–4·09)	1·11 (0·83–1·47)	1·04 (0·81–1·37)	3·67 (2017)	1·21 (1·1–1·33)	1·19 (1·0–1·46)	1·16 (0·99–1·45)
	Saint Lucia	0·18 (0·16–0·2)	0·11 (0·09–0·15)	0·10 (0·08–0·13)	0·19 (2038)	1·54 (1·28–1·84)	1·28 (1·01–1·66)	1·22 (1·0–1·6)
	Saint Vincent and the Grenadines	0·11 (0·1–0·13)	0·05 (0·02–0·09)	0·04 (0·02–0·07)	0·11 (2017)	1·86 (1·56–2·2)	1·37 (0·99–1·99)	1·31 (0·98–1·91)
	Suriname	0·57 (0·52–0·63)	0·54 (0·31–0·95)	0·39 (0·24–0·67)	0·66 (2051)	2·20 (1·91–2·53)	1·50 (1·01–2·25)	1·34 (0·98–2·09)
	Trinidad and Tobago	1·39 (1·24–1·55)	0·91 (0·56–1·58)	0·69 (0·49–1·17)	1·43 (2033)	1·70 (1·49–1·94)	1·37 (0·98–2·11)	1·22 (0·97–1·93)
	Virgin Islands	0·10 (0·09–0·12)	0·07 (0·03–0·15)	0·06 (0·03–0·13)	0·10 (2017)	2·04 (1·74–2·38)	1·71 (1·03–2·65)	1·60 (1·01–2·56)
Central Latin America		255·49 (238·67–271·43)	277·23 (212·16–369·14)	216·55 (165·24–288·6)	334·73 (2059)	2·35 (2·12–2·63)	1·52 (1·23–1·9)	1·44 (1·12–1·83)
	Colombia	50·61 (43·06–58·08)	46·55 (37·52–57·43)	42·36 (33·74–53·16)	61·49 (2052)	2·12 (1·82–2·45)	1·45 (1·18–1·75)	1·38 (1·09–1·66)
	Costa Rica	4·65 (4·18–5·15)	3·87 (2·73–5·94)	3·50 (2·49–5·51)	5·48 (2049)	1·75 (1·61–1·92)	1·30 (0·99–1·89)	1·27 (0·99–1·86)
	El Salvador	6·09 (5·31–6·83)	1·43 (0·83–2·51)	1·06 (0·67–1·71)	6·27 (2029)	1·95 (1·7–2·22)	1·32 (1·02–1·68)	1·27 (1·0–1·62)
	Guatemala	16·92 (14·23–19·63)	21·89 (12·54–43·16)	17·51 (11·06–34·45)	25·56 (2067)	2·80 (2·41–3·24)	1·32 (0·96–2·33)	1·31 (0·97–2·28)
	Honduras	9·50 (8·57–10·41)	14·39 (9·06–22·6)	11·18 (7·34–17·84)	15·91 (2072)	2·89 (2·51–3·36)	1·48 (1·01–2·16)	1·41 (1·01–2·07)
	Mexico	126·57 (112·47–141·47)	145·97 (117·7–184·64)	107·62 (84·36–134·39)	170·71 (2062)	2·42 (2·07–2·86)	1·44 (1·18–1·71)	1·36 (1·08–1·63)
	Nicaragua	6·40 (5·48–7·34)	4·81 (2·2–10·53)	3·84 (1·94–8·73)	7·92 (2049)	2·46 (2·1–2·88)	1·44 (0·99–2·37)	1·39 (0·99–2·27)
	Panama	3·92 (3·48–4·38)	6·94 (5·19–9·14)	5·00 (3·89–6·69)	6·94 (2100)	2·31 (1·97–2·69)	1·81 (1·39–2·29)	1·48 (1·11–1·93)
	Venezuela	30·83 (27·57–34·14)	31·37 (17·79–55·21)	24·48 (14·22–43·11)	38·05 (2056)	2·24 (1·99–2·52)	1·63 (1·07–2·35)	1·52 (1·03–2·27)
Tropical Latin America		218·74 (195·05–242·09)	172·89 (116·09–257·01)	158·60 (110·0–239·95)	244·46 (2044)	1·78 (1·61–1·99)	1·47 (1·03–2·04)	1·40 (0·98–2·0)
	Brazil	211·81 (187·73–234·87)	164·75 (114·27–240·69)	151·64 (108·04–225·76)	235·49 (2043)	1·76 (1·58–1·97)	1·44 (1·03–1·97)	1·37 (1·01–1·93)
	Paraguay	6·93 (5·88–8·05)	8·14 (4·52–13·93)	6·96 (3·68–12·18)	9·42 (2063)	2·55 (2·18–2·97)	1·84 (1·18–2·6)	1·70 (1·06–2·49)
**North Africa and Middle East**		**600·18 (579·17–621·86)**	**978·20 (714·8–1403·72)**	**697·16 (491·18–1041·03)**	**996·53 (2084)**	**2·71 (2·51–2·94)**	**1·78 (1·29–2·49)**	**1·69 (1·13–2·44)**
Afghanistan		32·85 (22·83–42·06)	129·77 (99·93–163·5)	42·72 (30·95–56·47)	129·78 (2099)	6·01 (5·71–6·3)	1·65 (1·34–1·98)	1·36 (1·05–1·68)
Algeria		40·46 (35·82–45·76)	78·83 (42·12–138·32)	60·07 (32·98–108·92)	78·83 (2100)	2·81 (2·49–3·12)	2·01 (1·11–3·17)	1·82 (1·02–3·01)
Bahrain		1·47 (1·31–1·64)	1·86 (1·44–2·43)	1·54 (1·23–1·99)	2·25 (2057)	2·05 (1·87–2·22)	1·37 (1·03–1·8)	1·28 (1·0–1·71)
Egypt		96·48 (90·07–102·86)	199·06 (100·92–389·44)	163·92 (78·68–318·32)	199·06 (2100)	2·66 (2·43–2·91)	2·08 (1·24–3·18)	1·91 (1·1–2·97)
Iran		82·18 (75·84–88·06)	70·00 (37·48–155·1)	62·23 (35·86–134·17)	95·32 (2049)	1·73 (1·47–2·03)	1·55 (1·0–2·77)	1·48 (0·98–2·68)
Iraq		43·30 (31·84–54·1)	108·12 (81·77–140·03)	73·25 (57·14–96·24)	108·19 (2097)	3·76 (3·42–4·15)	1·53 (1·1–1·95)	1·39 (1·01–1·84)
Jordan		10·65 (9·76–11·56)	21·17 (13·45–33·82)	14·13 (9·05–23·47)	21·39 (2090)	3·05 (2·8–3·35)	1·70 (1·09–2·46)	1·51 (1·02–2·26)
Kuwait		4·26 (3·82–4·71)	4·54 (2·72–11·2)	4·15 (2·64–9·8)	5·67 (2057)	1·42 (1·28–1·57)	1·47 (0·88–3·15)	1·44 (0·88–3·1)
Lebanon		8·51 (5·68–11·78)	8·75 (6·44–11·72)	6·56 (4·94–8·57)	11·54 (2058)	2·40 (2·06–2·81)	1·45 (1·15–1·76)	1·37 (1·07–1·69)
Libya		6·91 (5·97–7·83)	8·58 (4·33–18·42)	6·99 (3·79–14·63)	9·11 (2062)	2·12 (1·78–2·55)	1·66 (1·0–3·01)	1·57 (0·98–2·85)
Morocco		35·49 (32·61–38·85)	32·80 (24·7–42·98)	28·44 (21·38–37·14)	42·45 (2051)	2·14 (1·88–2·45)	1·39 (1·06–1·79)	1·33 (1·02–1·73)
Oman		4·54 (4·51–4·56)	9·30 (6·61–13·15)	6·56 (4·96–9·2)	9·30 (2098)	2·55 (2·3–2·81)	1·64 (1·08–2·3)	1·40 (1·0–2·03)
Palestine		4·85 (4·53–5·16)	9·85 (7·43–12·79)	5·56 (4·2–7·07)	10·06 (2087)	3·49 (3·16–3·86)	1·80 (1·46–2·13)	1·49 (1·17–1·79)
Qatar		2·75 (2·53–2·98)	2·33 (1·65–3·28)	1·88 (1·39–2·71)	3·53 (2052)	2·04 (1·87–2·22)	1·51 (1·04–2·12)	1·39 (1·01–2·0)
Saudi Arabia		34·44 (30·56–38·38)	33·04 (20·11–64·71)	27·30 (19·0–50·78)	44·68 (2052)	1·67 (1·47–1·88)	1·39 (0·97–2·49)	1·32 (0·97–2·37)
Sudan		40·26 (34·71–45·49)	81·94 (64·79–100·52)	51·16 (41·32–62·99)	84·37 (2086)	4·22 (3·86–4·62)	1·45 (1·16–1·74)	1·27 (1·02–1·55)
Syria		18·13 (15·31–20·58)	13·46 (7·19–25·63)	10·15 (6·41–19·48)	20·12 (2044)	2·17 (1·88–2·51)	1·38 (0·95–2·18)	1·28 (0·93–2·13)
Tunisia		11·44 (10·35–12·47)	11·68 (7·67–18·46)	9·93 (6·71–15·74)	13·63 (2055)	1·77 (1·52–2·09)	1·52 (1·01–2·29)	1·44 (1·0–2·22)
Turkey		80·46 (80·02–80·94)	101·64 (79·79–127·94)	86·10 (70·31–109·64)	112·51 (2068)	1·79 (1·61–2·0)	1·34 (1·01–1·73)	1·26 (1·0–1·63)
United Arab Emirates		9·73 (8·43–11·17)	3·45 (2·54–4·79)	3·15 (2·37–4·36)	10·17 (2029)	1·31 (1·17–1·49)	1·27 (1·0–1·72)	1·23 (0·99–1·71)
Yemen		30·45 (25·79–35·17)	47·13 (31·0–65·81)	30·70 (20·55–45·36)	56·15 (2069)	4·53 (4·16–4·96)	1·39 (1·02–1·82)	1·33 (1·01–1·78)
**South Asia**		**1782·68 (1637·81–1941·51)**	**1441·70 (955·35–2242·79)**	**1200·21 (853·24–1858·76)**	**2117·38 (2049)**	**2·27 (2·04–2·54)**	**1·33 (0·96–1·90)**	**1·27 (0·95–1·82)**
Bangladesh		156·98 (140·23–173·23)	81·30 (55·58–120·99)	74·19 (52·72–110·08)	173·49 (2039)	2·00 (1·81–2·22)	1·19 (0·99–1·59)	1·17 (0·99–1·56)
Bhutan		0·96 (0·82–1·09)	0·77 (0·54–1·14)	0·72 (0·52–1·05)	1·19 (2051)	1·98 (1·76–2·27)	1·35 (1·02–1·83)	1·30 (1·01–1·79)
India		1380·56 (1235·54–1535·78)	1093·15 (724·48–1714·29)	929·87 (663·75–1443·47)	1605·60 (2048)	2·14 (1·93–2·39)	1·29 (0·99–1·89)	1·24 (0·98–1·8)
Nepal		29·89 (26·62–32·83)	18·09 (11·14–34·73)	15·55 (10·34–29·96)	34·57 (2043)	2·21 (1·96–2·52)	1·20 (0·98–1·91)	1·19 (0·98–1·89)
Pakistan		214·29 (198·94–228·96)	248·39 (151·17–427·14)	179·88 (120·72–320·32)	314·08 (2062)	3·40 (2·99–3·9)	1·31 (0·99–2·04)	1·27 (0·99–1·99)
**Southeast Asia, east Asia, and Oceania**		**2158·80 (1981·55–2321·29)**	**1437·41 (979·55–2435·23)**	**1263·48 (864·11–2156·81)**	**2249·56 (2032)**	**1·72 (1·63–1·83)**	**1·61 (1·14–2·44)**	**1·50 (1·03–2·36)**
East Asia		1485·71 (1316·09–1646·5)	768·13 (463·86–1587·39)	733·09 (457·41–1486·17)	1506·02 (2024)	1·52 (1·43–1·61)	1·47 (0·91–2·66)	1·41 (0·89–2·56)
	China	1412·48 (1244·31–1571·26)	731·89 (455·61–1499·32)	699·74 (452·81–1402·01)	1431·91 (2024)	1·53 (1·43–1·63)	1·47 (0·96–2·55)	1·41 (0·95–2·49)
	North Korea	25·72 (22·82–28·77)	12·98 (10·6–15·87)	11·01 (8·87–13·29)	26·08 (2028)	1·32 (1·17–1·51)	1·30 (1·09–1·53)	1·22 (1·03–1·45)
	Taiwan (province of China)	23·58 (23·4–23·77)	10·89 (8·32–14·78)	10·53 (8·29–14·58)	23·87 (2027)	1·04 (0·92–1·19)	1·30 (0·98–1·8)	1·26 (0·95–1·78)
Oceania		12·60 (11·58–13·65)	34·16 (25·28–44·82)	14·28 (11·29–18·26)	34·16 (2100)	4·02 (3·67–4·37)	1·99 (1·58–2·56)	1·41 (1·1–1·88)
	American Samoa	0·06 (0·05–0·06)	0·11 (0·06–0·2)	0·07 (0·04–0·12)	0·11 (2100)	2·92 (2·55–3·34)	2·13 (1·37–3·06)	1·67 (1·03–2·56)
	Federated States of Micronesia	0·10 (0·09–0·12)	0·17 (0·1–0·29)	0·11 (0·07–0·18)	0·17 (2094)	2·72 (2·42–3·07)	1·79 (1·14–2·58)	1·48 (1·0–2·22)
	Fiji	0·91 (0·85–0·97)	1·12 (0·67–1·71)	0·62 (0·38–0·92)	1·14 (2077)	2·61 (2·3–2·96)	1·98 (1·48–2·51)	1·61 (1·17–2·09)
	Guam	0·17 (0·15–0·19)	0·27 (0·07–0·7)	0·17 (0·05–0·5)	0·27 (2100)	2·95 (2·71–3·19)	2·26 (1·06–3·95)	1·92 (0·99–3·64)
	Kiribati	0·12 (0·11–0·13)	0·34 (0·24–0·48)	0·14 (0·1–0·19)	0·34 (2100)	3·71 (3·26–4·2)	2·32 (1·82–2·9)	1·40 (1·02–1·86)
	Marshall Islands	0·06 (0·05–0·06)	0·08 (0·05–0·11)	0·06 (0·04–0·09)	0·08 (2074)	2·86 (2·55–3·22)	1·75 (1·31–2·2)	1·63 (1·21–2·13)
	Northern Mariana Islands	0·04 (0·04–0·05)	0·04 (0·03–0·06)	0·03 (0·02–0·04)	0·05 (2039)	2·06 (1·82–2·31)	1·72 (1·32–2·18)	1·48 (1·11–1·91)
	Papua New Guinea	9·23 (8·26–10·22)	27·01 (20·42–34·21)	11·37 (9·41–13·71)	27·01 (2100)	4·21 (3·83–4·59)	1·83 (1·52–2·18)	1·32 (1·08–1·57)
	Samoa	0·20 (0·18–0·21)	1·06 (0·08–3·56)	0·17 (0·02–1·01)	1·06 (2100)	4·69 (4·23–5·22)	4·47 (2·26–7·32)	2·51 (1·02–5·21)
	Solomon Islands	0·64 (0·56–0·71)	1·08 (0·61–1·69)	0·29 (0·18–0·47)	1·18 (2078)	4·20 (3·8–4·64)	1·91 (1·44–2·43)	1·41 (1·04–1·84)
	Tonga	0·10 (0·1–0·11)	0·31 (0·18–0·51)	0·13 (0·08–0·24)	0·31 (2100)	3·17 (2·77–3·61)	2·62 (1·81–3·67)	1·48 (0·99–2·46)
	Vanuatu	0·29 (0·27–0·31)	0·68 (0·52–0·88)	0·34 (0·26–0·46)	0·68 (2097)	3·73 (3·41–4·12)	1·77 (1·41–2·13)	1·40 (1·05–1·75)
Southeast Asia		660·48 (625·64–694·46)	635·12 (457·86–894·48)	516·10 (371·34–744·67)	786·84 (2052)	2·08 (1·88–2·32)	1·61 (1·21–2·07)	1·49 (1·11–1·99)
	Cambodia	16·12 (14·15–18·18)	15·93 (10·77–24·35)	13·14 (9·58–20·41)	20·77 (2056)	2·73 (2·49–3·04)	1·30 (1·0–1·9)	1·27 (0·99–1·85)
	Indonesia	258·13 (228·44–286·87)	228·69 (141·13–387·53)	202·69 (128·87–346·57)	300·51 (2047)	1·97 (1·7–2·3)	1·51 (1·01–2·21)	1·43 (1·0–2·14)
	Laos	6·97 (6·44–7·47)	6·81 (5·23–8·77)	5·45 (4·25–7·1)	9·23 (2056)	2·90 (2·64–3·21)	1·28 (1·01–1·6)	1·24 (1·0–1·55)
	Malaysia	30·64 (27·09–34·12)	41·33 (31·7–54·14)	35·13 (27·51–45·19)	44·19 (2070)	2·02 (1·81–2·26)	1·64 (1·26–2·08)	1·53 (1·18–1·97)
	Maldives	0·46 (0·42–0·5)	0·57 (0·37–1·01)	0·48 (0·34–0·83)	0·68 (2062)	1·87 (1·72–2·02)	1·38 (0·96–2·36)	1·32 (0·95–2·26)
	Mauritius	1·27 (1·15–1·4)	0·72 (0·5–1·12)	0·66 (0·49–1·02)	1·30 (2031)	1·32 (1·22–1·44)	1·27 (0·97–1·88)	1·23 (0·97–1·82)
	Myanmar	52·80 (48·36–57·31)	49·77 (36·66–69·05)	45·82 (34·4–64·21)	63·51 (2051)	2·02 (1·85–2·21)	1·38 (1·03–1·84)	1·32 (1·02–1·79)
	Philippines	103·47 (94·56–111·89)	169·46 (117·16–237·9)	107·03 (70·59–159·4)	173·28 (2085)	3·12 (2·85–3·45)	1·78 (1·3–2·3)	1·59 (1·1–2·1)
	Seychelles	0·10 (0·09–0·11)	0·09 (0·05–0·16)	0·07 (0·04–0·14)	0·11 (2042)	2·15 (1·9–2·43)	1·68 (1·05–2·54)	1·57 (1·02–2·46)
	Sri Lanka	21·60 (19·45–23·8)	10·45 (6·85–15·11)	7·31 (4·49–10·97)	22·34 (2031)	1·80 (1·52–2·11)	1·46 (1·11–1·87)	1·38 (1·06–1·77)
	Thailand	70·63 (62·64–78·58)	34·66 (26·07–49·31)	33·20 (25·66–47·55)	71·97 (2028)	1·21 (1·07–1·38)	1·28 (1·0–1·76)	1·24 (0·99–1·73)
	Timor-Leste	1·29 (1·19–1·39)	2·94 (2·16–3·93)	1·69 (1·24–2·24)	2·94 (2098)	4·14 (3·63–4·69)	1·81 (1·39–2·26)	1·39 (1·04–1·82)
	Vietnam	96·14 (84·73–108·05)	72·85 (51·69–104·23)	62·76 (45·22–89·57)	107·25 (2044)	1·85 (1·68–2·05)	1·39 (1·02–1·86)	1·33 (1·01–1·79)
**Sub-Saharan Africa**		**1026·04 (988·37–1062·59)**	**3071·21 (2477·11–3838·43)**	**1584·66 (1226·15–2057·39)**	**3071·21 (2100)**	**4·62 (4·33–4·93)**	**1·73 (1·42–2·06)**	**1·52 (1·18–1·89)**
Central sub-Saharan Africa		121·67 (99·15–143·25)	343·47 (251·51–459·5)	161·60 (118·8–225·04)	343·76 (2097)	4·88 (4·62–5·13)	1·70 (1·29–2·18)	1·38 (1·06–1·81)
	Angola	28·20 (25·98–30·71)	84·34 (69·18–101·51)	46·71 (38·24–56·04)	84·68 (2095)	5·12 (4·72–5·54)	1·55 (1·3–1·81)	1·41 (1·14–1·68)
	Central African Republic	4·62 (3·94–5·33)	2·56 (1·52–3·74)	1·17 (0·62–1·8)	5·15 (2041)	3·56 (3·18–4·0)	1·34 (1·1–1·63)	1·15 (1·0–1·4)
	Congo (Brazzaville)	4·91 (4·24–5·61)	5·20 (3·76–6·95)	3·92 (2·79–5·3)	6·72 (2059)	3·30 (2·99–3·66)	1·29 (1·02–1·63)	1·24 (1·01–1·58)
	Democratic Republic of the Congo	80·88 (57·85–102·65)	246·35 (170·29–346·15)	106·12 (73·79–156·78)	246·35 (2100)	5·05 (4·73–5·37)	1·75 (1·27–2·3)	1·34 (1·01–1·85)
	Equatorial Guinea	1·35 (1·24–1·45)	3·03 (2·49–3·65)	2·09 (1·83–2·35)	3·17 (2083)	3·88 (3·36–4·44)	1·39 (1·21–1·55)	1·29 (1·13–1·44)
	Gabon	1·70 (1·55–1·86)	1·99 (1·55–2·53)	1·60 (1·27–2·02)	2·47 (2063)	2·79 (2·47–3·17)	1·30 (1·03–1·62)	1·25 (1·01–1·54)
Eastern sub-Saharan Africa		393·18 (375·73–410·77)	1056·10 (843·46–1316·08)	570·85 (443·91–734·14)	1062·77 (2093)	4·65 (4·36–4·98)	1·73 (1·43–2·04)	1·48 (1·15–1·83)
	Burundi	10·91 (9·54–12·33)	42·57 (23·09–71·74)	14·89 (8·04–30·36)	42·57 (2100)	5·30 (4·99–5·65)	1·61 (1·0–2·53)	1·46 (1·0–2·39)
	Comoros	0·72 (0·61–0·83)	0·78 (0·53–1·14)	0·51 (0·37–0·77)	0·99 (2060)	3·39 (2·92–3·9)	1·39 (1·03–1·88)	1·22 (0·99–1·69)
	Djibouti	1·11 (0·98–1·23)	1·19 (0·86–1·58)	0·91 (0·68–1·22)	1·59 (2057)	3·82 (3·33–4·34)	1·33 (1·04–1·71)	1·28 (1·01–1·65)
	Eritrea	5·86 (4·22–7·49)	7·01 (5·45–8·75)	4·90 (3·92–5·9)	9·05 (2062)	4·03 (3·55–4·57)	1·27 (1·12–1·44)	1·22 (1·06–1·38)
	Ethiopia	102·88 (89·58–116·2)	223·45 (180·08–276·35)	153·93 (124·17–192·18)	240·29 (2080)	4·79 (4·42–5·2)	1·33 (1·04–1·63)	1·28 (1·02–1·59)
	Kenya	48·33 (42·49–53·83)	74·14 (43·62–121·46)	59·25 (34·84–98·73)	83·85 (2071)	3·38 (2·95–3·84)	1·59 (1·03–2·29)	1·49 (1·01–2·15)
	Madagascar	26·11 (20·4–31·8)	105·77 (70·49–159·25)	42·22 (28·81–63·17)	105·77 (2100)	4·89 (4·36–5·45)	1·61 (1·16–2·15)	1·45 (1·02–2·0)
	Malawi	17·19 (14·94–19·28)	36·52 (28·13–46·18)	25·73 (19·35–33·03)	38·73 (2080)	4·46 (4·16–4·79)	1·60 (1·29–1·92)	1·51 (1·16–1·87)
	Mozambique	30·04 (27·82–32·01)	46·36 (35·88–58·68)	32·08 (25·71–40·46)	54·73 (2070)	4·16 (3·84–4·49)	1·26 (1·01–1·52)	1·20 (1·0–1·48)
	Rwanda	12·55 (11·27–13·78)	33·02 (20·94–47·92)	19·57 (13·0–29·36)	33·33 (2092)	4·43 (4·01–4·91)	1·49 (1·01–2·08)	1·41 (1·0–2·02)
	Somalia	16·88 (12·48–21·43)	62·52 (47·58–77·67)	6·59 (4·33–9·24)	62·52 (2100)	6·10 (5·72–6·5)	2·57 (2·24–2·88)	1·31 (1·18–1·45)
	South Sudan	9·94 (8·73–11·24)	68·95 (52·11–88·18)	17·54 (13·85–21·6)	68·95 (2100)	5·93 (5·56–6·35)	2·46 (2·07–2·83)	1·30 (1·03–1·58)
	Tanzania	53·97 (48·57–59·61)	185·96 (129·06–258·26)	88·79 (59·28–129·12)	185·96 (2100)	4·79 (4·44–5·18)	1·60 (1·12–2·1)	1·46 (1·01–1·99)
	Uganda	39·08 (35·69–42·45)	119·86 (102·59–138·26)	73·35 (61·22–86·28)	120·63 (2093)	5·24 (4·99–5·51)	1·72 (1·48–1·97)	1·60 (1·32–1·87)
	Zambia	17·36 (15·31–19·45)	47·33 (33·77–63·96)	30·22 (20·43–42·41)	47·65 (2093)	4·68 (4·21–5·22)	1·69 (1·23–2·12)	1·58 (1·1–2·04)
Southern sub-Saharan Africa		77·37 (71·35–83·41)	124·03 (81·94–180·27)	96·98 (61·99–144·44)	125·14 (2087)	2·62 (2·38–2·9)	1·92 (1·39–2·54)	1·77 (1·24–2·38)
	Botswana	2·28 (2·05–2·53)	3·09 (2·19–4·41)	2·78 (1·96–3·94)	3·41 (2067)	2·36 (2·2–2·53)	1·61 (1·15–2·15)	1·51 (1·07–2·02)
	eSwatini	1·12 (1·05–1·2)	1·54 (0·84–2·63)	1·20 (0·64–2·08)	1·72 (2068)	3·04 (2·65–3·51)	1·63 (1·03–2·35)	1·54 (1·01–2·26)
	Lesotho	1·95 (1·67–2·21)	2·13 (1·5–2·93)	1·65 (1·11–2·33)	2·55 (2061)	2·87 (2·63–3·16)	1·57 (1·24–1·91)	1·47 (1·12–1·86)
	Namibia	2·35 (2·11–2·6)	4·33 (3·39–5·49)	3·75 (2·94–4·82)	4·39 (2086)	3·01 (2·76–3·3)	1·79 (1·44–2·16)	1·66 (1·31–2·03)
	South Africa	54·95 (49·03–60·64)	74·69 (53·07–101·36)	60·36 (41·88–84·31)	77·97 (2073)	2·29 (2·05–2·61)	1·70 (1·28–2·19)	1·57 (1·14–2·03)
	Zimbabwe	14·71 (13·32–16·04)	38·25 (18·75–70·08)	27·24 (11·74–52·86)	38·25 (2100)	3·78 (3·53–4·04)	2·22 (1·46–3·12)	2·03 (1·21–2·96)
Western sub-Saharan Africa		433·82 (413·56–453·7)	1547·61 (1215·77–1972·7)	755·23 (564·94–1007·71)	1547·61 (2100)	4·94 (4·63–5·27)	1·70 (1·38–2·07)	1·53 (1·14–1·95)
	Benin	11·59 (10·51–12·74)	30·30 (23·72–38·02)	17·45 (14·1–21·57)	31·06 (2088)	4·78 (4·44–5·17)	1·33 (1·07–1·63)	1·25 (1·02–1·53)
	Burkina Faso	21·12 (18·12–24·13)	71·89 (52·65–94·98)	34·25 (25·43–45·13)	72·11 (2096)	5·40 (5·07–5·77)	1·41 (1·04–1·8)	1·35 (1·01–1·72)
	Cameroon	27·77 (23·78–31·89)	43·10 (31·7–57·42)	32·84 (23·58–44·69)	49·67 (2071)	3·94 (3·51–4·42)	1·41 (1·06–1·81)	1·35 (1·02–1·74)
	Cape Verde	0·55 (0·48–0·61)	0·50 (0·34–0·81)	0·46 (0·32–0·74)	0·68 (2053)	2·19 (1·82–2·61)	1·33 (0·99–2·01)	1·30 (0·98–1·97)
	Chad	15·22 (13·39–17·03)	123·32 (101·78–148·38)	32·91 (27·13–39·34)	123·32 (2100)	6·72 (6·42–7·04)	2·19 (1·9–2·54)	1·46 (1·23–1·71)
	Côte d'Ivoire	24·97 (22·78–27·06)	60·60 (36·7–103·99)	35·06 (24·56–63·47)	61·50 (2090)	4·50 (4·13–4·93)	1·35 (0·98–2·24)	1·32 (1·0–2·24)
	The Gambia	2·13 (1·93–2·33)	3·63 (2·92–4·4)	2·26 (1·86–2·72)	4·10 (2074)	4·14 (3·69–4·66)	1·32 (1·09–1·56)	1·22 (1·01–1·45)
	Ghana	30·21 (26·66–33·57)	51·91 (36·61–73·17)	36·29 (26·62–52·53)	55·12 (2079)	3·47 (3·02–3·98)	1·43 (1·01–1·95)	1·35 (1·0–1·86)
	Guinea	11·82 (10·84–12·84)	26·79 (19·56–35·59)	14·11 (11·12–18·93)	27·93 (2084)	4·63 (4·38–4·9)	1·36 (1·03–1·73)	1·21 (1·01–1·58)
	Guinea-Bissau	1·86 (1·63–2·07)	3·42 (2·09–5·64)	2·23 (1·54–3·81)	3·75 (2076)	4·63 (4·23–5·04)	1·33 (1·0–2·06)	1·29 (1·0–2·01)
	Liberia	4·72 (4·14–5·28)	7·46 (5·79–9·22)	5·00 (3·9–6·28)	8·70 (2071)	4·25 (3·84–4·74)	1·32 (1·08–1·57)	1·27 (1·02–1·52)
	Mali	20·25 (17·82–22·67)	85·21 (65·68–109·09)	34·82 (26·47–45·41)	85·21 (2100)	6·02 (5·72–6·37)	1·47 (1·13–1·82)	1·39 (1·05–1·77)
	Mauritania	3·91 (3·56–4·29)	8·87 (5·97–13·47)	6·30 (4·67–9·57)	9·19 (2085)	4·15 (3·85–4·5)	1·34 (0·99–2·02)	1·31 (0·99–1·97)
	Niger	21·38 (19·34–23·65)	185·04 (129·68–257·03)	57·81 (40·17–81·88)	185·04 (2100)	7·08 (6·76–7·43)	1·79 (1·34–2·25)	1·65 (1·16–2·15)
	Nigeria	206·09 (188·25–224·28)	790·73 (594·39–1055·64)	408·54 (292·03–567·19)	790·73 (2100)	5·11 (4·71–5·51)	1·69 (1·25–2·16)	1·57 (1·11–2·07)
	São Tomé and Príncipe	0·20 (0·18–0·22)	0·20 (0·14–0·28)	0·16 (0·12–0·22)	0·28 (2058)	3·25 (2·83–3·71)	1·25 (1·0–1·63)	1·21 (1·0–1·6)
	Senegal	14·69 (13·24–16·1)	32·21 (21·14–47·97)	18·79 (13·11–29·99)	33·77 (2083)	4·57 (4·23–4·97)	1·34 (1·0–1·97)	1·30 (1·0–1·95)
	Sierra Leone	7·83 (7·21–8·48)	12·03 (8·72–16·31)	8·03 (6·02–10·88)	14·20 (2070)	4·25 (3·83–4·7)	1·28 (1·02–1·66)	1·23 (1·01–1·59)
	Togo	7·52 (6·72–8·35)	10·39 (7·35–13·92)	7·90 (5·96–10·86)	12·39 (2068)	3·82 (3·54–4·12)	1·27 (1·0–1·68)	1·23 (1·0–1·65)

Data in parentheses are 95% uncertainty intervals, unless specified otherwise. The SDG pace scenario had a custom rate of change that would allow all locations to meet the SDG targets for educational attainment and contraceptive met need by 2030. Population and total fertility rate values are presented as means. Estimates in 2017 are from GBD 2017 estimates. Peak population was calculated from 1990 up to 2100. Super-regions, regions, and countries are listed in alphabetical order. GBD=Global Burden of Diseases, Injuries, and Risk Factors Study. SDG=Sustainable Development Goal.

### Global population scenarios

**Figure 5:**
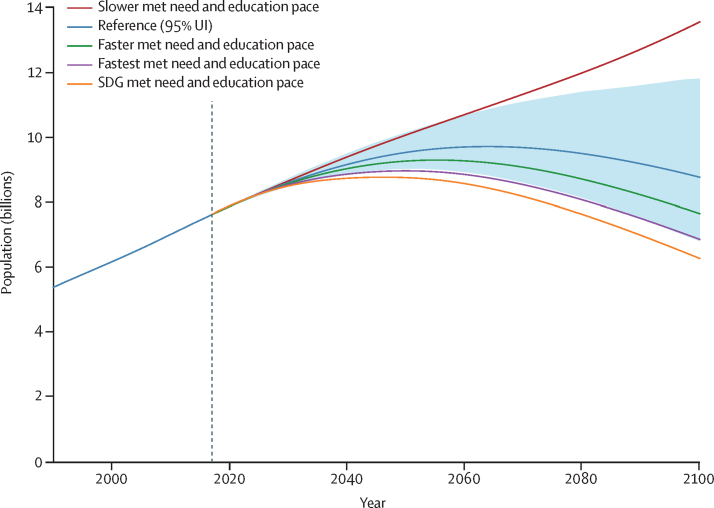
Global population in the reference, slower, faster, fastest, and SDG pace scenarios, 1990–2100 The reference scenario is presented with 95% UIs, which are represented by the shaded area. Past estimates are from GBD 2017, and values are in billions. GBD=Global Burden of Diseases, Injuries, and Risk Factors Study. SDG=Sustainable Development Goal. UI=uncertainty interval.

**Figure 6:**
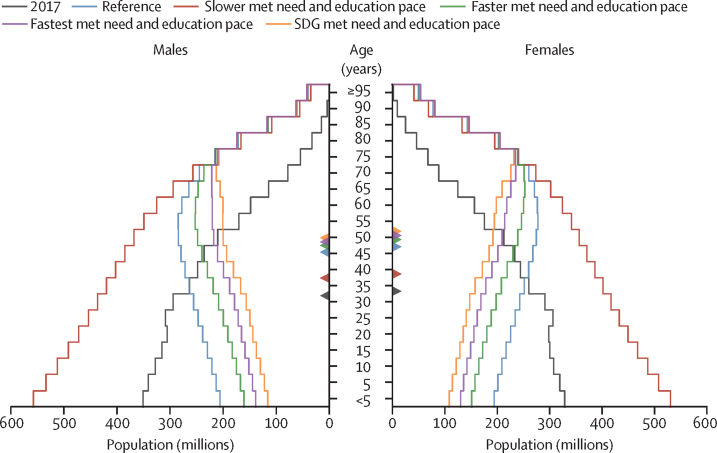
Global population age structure in 2017 and in 2100 in the reference, slower, faster, fastest, and SDG pace scenarios Estimates for 2017 are from GBD 2017. Triangles indicate the mean age for each scenario. GBD=Global Burden of Diseases, Injuries, and Risk Factors Study. SDG=Sustainable Development Goal.

Combining the scenarios for mortality, fertility, and migration, we forecasted global population in the reference scenario to peak at 9·73 billion (95% UI 8·84–10·9) people in 2064 and then decline to 8·79 billion (6·83–11·8) in 2100 (table, figure 5). Across alternative scenarios, global population in 2100 ranged from 13·6 billion (10·7–17·7) people in the slower met need and education scenario to 6·29 billion (4·82–8·73) in the SDG pace scenario (table, figure 5). The faster scenario forecasted a 2100 global population of 7·67 billion (5·88–10·4) people, whereas the fastest scenario forecasted 6·88 billion (5·27–9·51). Peak population in the SDG scenario was forecasted to occur in 2046, whereas the global population continued to grow after 2100 in the slower scenario. The large range in global TFR across the scenarios translated into a total difference of 7·29 billion (5·95–8·71) people between the slower scenario and SDG scenario population projections, globally in 2100. All five scenarios forecasted large shifts in the age structure of the global population in 2100 (as depicted by the age pyramids in figure 6). Mean age was forecasted to increase in the reference scenario from 32·6 years in 2017 to 46·2 years (43·4–47·8) in 2100. In 2100, we forecasted 2·37 billion (1·91–2·87) individuals older than 65 years globally compared with 1·70 billion (1·11–2·81) younger than 20 years. The number of children younger than 5 years was forecasted to decline from 681 million in 2017 to 401 million (251–704) in 2100, a drop of 41·0% (23·5–51·8). At the same time, the number of individuals older than 80 years was forecasted to increase from 141 million in 2017 to 866 million (617–1140) in 2100. The number of births and people turning 80 years old in the world from 1950 to 2100, as well as the ratio of births to new 80-year-olds, are shown in appendix 2 (section 2.3). In 1950, 25 births occurred for every person turning 80 years old; in 2017 that number was seven and in 2100 we forecasted one birth for every person turning 80 years old. The ratio of the population older than 80 years to that younger than 15 years was forecasted to increase from 0·16 in 2017 to 1·50 (0·54–3·25) in 2100 in countries with a population decline higher than 25% (appendix 2, section 2). Additional population results are available online.

### Comparison with other scenarios

We compared the global TFR, life expectancy at birth, and population forecasts of our reference scenario with the UNPD median variant and the Wittgenstein's Shared Socioeconomic Pathway 2 scenario (appendix 2, section 9). The differences between 2100 population forecasts from the UNPD and those of our reference scenario can be largely explained by differences in population in sub-Saharan Africa (702 million fewer people in our reference scenario), south Asia (584 million fewer people), and southeast Asia, east Asia, and Oceania (447 million fewer people), which were calculated by mapping UNPD locations to GBD regions and super-regions (appendix 2, section 9). These differences are likely to be driven by fertility rates. Comparing UNPD forecasts with those of our reference scenario for cumulative births from 2017 to 2100, the largest differences by super-region were found in sub-Saharan Africa (891 million births), south Asia (630 million), and southeast Asia, east Asia, and Oceania (402 million). While the global difference in cumulative births was 2·17 billion, differences in cumulative deaths were much smaller, accounting for only 81·8 million deaths globally from 2017 to 2100 (appendix 2, section 8).

### Country-level migration scenarios

We forecasted that 118 of 195 countries and territories will have net migration rates between −1 and 1 per 1000 population in 2100, with an additional 44 countries having net migration rates between −2 and 2 per 1000. The countries with the largest immigration forecasts in absolute numbers in 2100 were the USA, India, and China, whereas emigration was forecasted to be largest in Somalia, the Philippines, and Afghanistan. Net immigration rates were forecasted to be highest in Canada, Turkey, and Sweden, whereas emigration rates were highest in El Salvador, Samoa, and Jamaica (appendix 2, section 4).

### Regional-level and country-level mortality scenarios

Both global and super-region life expectancy forecasts showed a future slowdown, particularly in the latter half of the century, compared with past values from 1990 to 2017. The slowdown was more evident in the high-income; south Asia; southeast Asia, east Asia, and Oceania; and Latin America and the Caribbean super-regions. By contrast, the slowdown was less noticeable in the sub-Saharan Africa; north Africa and the Middle East; and central Europe, eastern Europe, and central Asia super-regions (appendix 2, section 3). Overall, the observed pattern was one of global convergence of life expectancy towards the end of the century. Among the ten countries with the largest populations in 2017 or 2100, China, Bangladesh, Brazil, Ethiopia, the USA, and Nigeria were forecasted to have the highest life expectancies in 2100 according to the reference scenario, ranging from 84·2 years (95% UI 80·9–87·5) in China to 80·4 years (76·6–84·0) in Nigeria (appendix 2, section 3). DR Congo, Pakistan, India, and Indonesia were forecasted to have the lowest life expectancies among these ten large countries, ranging from 76·9 years (71·4–81·2) in DR Congo to 79·5 years (77·5–81·1) in Indonesia (appendix 2, section 3).

### Regional-level and country-level fertility scenarios

By 2017, three GBD super-regions had reached a TFR lower than replacement levels (<2·1): high-income; central Europe, eastern Europe, and central Asia; and southeast Asia, east Asia, and Oceania. By 2100, our reference TFR forecasts for GBD super-regions ranged between 1·33 (95% UI 0·96–1·90) for south Asia and 1·80 (1·25–2·40) for central Europe, eastern Europe, and central Asia (table). Sub-Saharan Africa was forecasted to decline from 4·62 (4·33–4·93) in 2017 to 1·73 (1·42–2·06) in 2100 and reach a TFR lower than replacement level in 2063 (figure 3B).

We plotted trajectories of the TFR for the ten countries with the largest populations now or in 2100 (appendix 2, section 5). Our reference forecast suggested that India reached a TFR lower than replacement level in 2018. Thereafter, India was forecasted to have a continued steep decline until about 2040, reaching a TFR of 1·29 (95% UI 0·99–1·89) in 2100 (table). China's TFR was forecasted to decline moderately to 1·42 (1·04–2·04) around 2030, increasing slowly afterwards to 1·47 (0·96–2·55) by 2100. The USA was forecasted to decline to a TFR of 1·53 (1·10–2·02) in 2100, whereas Indonesia declined to 1·51 (1·01–2·21) and Pakistan to 1·31 (0·99–2·04) in 2100. Japan was forecasted to stay stable, with a TFR of 1·32 (0·96–2·03) in 2100, and Russia was forecasted to moderately decline to a TFR of 1·43 (0·96–2·23) in 2100, contributing to population declines that led them to no longer be forecasted to be among the ten most populous countries in 2100.

In 2017, estimates of TFR were 5·11 (95% UI 4·71–5·51) in Nigeria, 7·08 (6·76–7·43) in Niger, and 2·29 (2·05–2·61) in South Africa; by 2100, we forecasted 1·69 (1·25–2·16) in Nigeria, 1·79 (1·34–2·25) in Niger, and 1·70 (1·28–2·19) in South Africa (table). Only four countries in sub-Saharan Africa were forecasted to have TFRs higher than replacement level in 2100: Somalia, South Sudan, Zimbabwe, and Chad (table). The lowest TFR reference forecasts for sub-Saharan Africa in 2100 were for São Tomé and Príncipe, Mozambique, Eritrea, Togo, and Sierra Leone.

We observed large variations in the forecasted TFRs by country and territory across scenarios (appendix 2, section 5). For example, the forecasted TFR range in China for 2100 was narrow, between 1·41 (95% UI 0·95–2·49) in the SDG pace scenario (table) and 1·47 (0·96–2·59) in the other scenarios. By contrast, for Nigeria, the forecasted TFR range was much wider, from 1·57 (1·11–2·07) in the SDG pace scenario (table) to 3·11 (2·61–3·61) in the slower contraceptive met need and education scenario. This variation in fertility forecasts across scenarios is explained by the present attained levels of education and contraceptive met need and their modelled future effects on fertility, which are much larger in high-fertility than low-fertility settings (more results are present in appendix 2, section 5).

### Regional-level and country-level population scenarios

Other than the central Europe, eastern Europe, and central Asia super-region, where the population peaked in 1992, population size in the remaining super-regions was forecasted to peak in the future before 2100, except sub-Saharan Africa, which was not forecasted to peak until 2100 or later in the reference scenario (appendix 2, section 2). In the reference scenario, sub-Saharan Africa and north Africa and the Middle East were the only super-regions forecasted to have higher populations in 2100 than in 2017 (3·07 billion [95% UI 2·48–3·84] people in sub-Saharan Africa and 978 million [715–1404] in north Africa and the Middle East; table). All super-regions except sub-Saharan Africa were forecasted to have substantial population declines in the coming eight decades. The declines were forecasted to be most severe in south Asia; southeast Asia, east Asia, and Oceania; and central Europe, eastern Europe, and central Asia.

**Figure 7:**
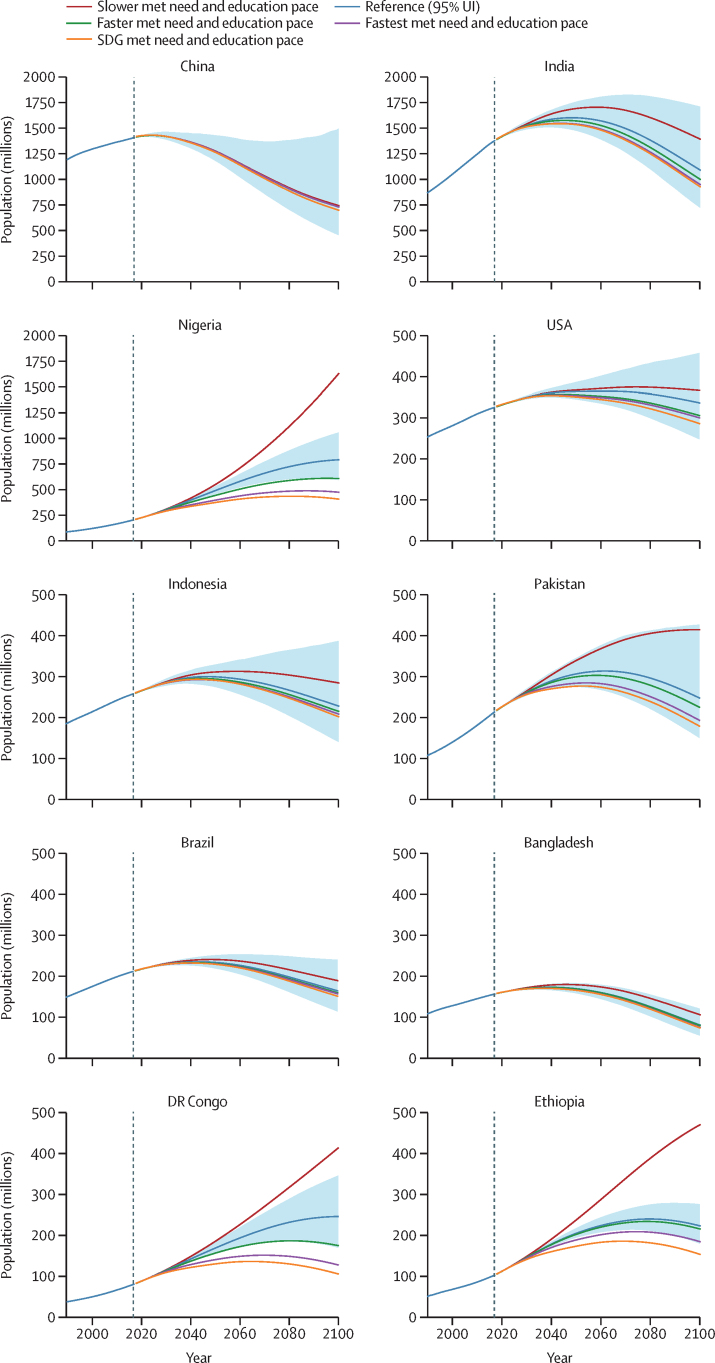
Population size in ten countries in the reference, slower, faster, fastest, and SDG pace scenarios, 1990–2100 The reference scenario is presented with 95% UIs, which are represented by the shaded area. Countries were selected on the basis of the most populous countries in 2017 and those projected to be the most populous in 2100. Past estimates up to 2017 are from GBD 2017, and estimates for 2018–2100 are means of each scenario. GBD=Global Burden of Diseases, Injuries, and Risk Factors Study. SDG=Sustainable Development Goal. UI=uncertainty interval.

We plotted the trajectory of total population for the ten largest countries today or in 2100 for all five scenarios (figure 7). With the reference scenario, we forecasted the five largest countries in 2100 to be India, Nigeria, China, the USA, and Pakistan (table). However, these forecasts showed different future trajectories between countries. Nigeria was forecasted to have continued population growth through 2100 and was expected to be the second most populous country by then. The reference forecasts for China and India peaked before 2050 and both countries thereafter had steep declining trajectories, with China down to 51·1% (95% UI 32·1–103·0) and India down to 68·1% (48·7–98·0) of their peak populations in 2100. The USA was projected to have population growth until mid-century, followed by a moderate decline of less than 10% of the peak population by 2100. We forecasted that the number of countries in sub-Saharan Africa among the ten most populous would increase from only Nigeria in 2017 to also include DR Congo, Ethiopia, and Tanzania in 2100 (additional results in appendix 2, section 2).

We forecasted large changes in age structures across the different super-regions (appendix 2, section 2). Under the reference scenario, sub-Saharan Africa had a moderate increase in the population younger than 15 years, but large increases in the working-age population aged 20–64 years and in the older population aged 65 years or older by 2100. By contrast, under the slower pace scenario, the age structure forecast was very different, with a 4-times to 5-times increase in the population younger than 15 years. High-income countries were forecasted to accentuate the inverted shape of the age pyramid, with strong absolute decreases in the population younger than 65 years and absolute increases in the population aged 65 years or older under all scenarios. The south Asia; southeast Asia, east Asia, and Oceania; and Latin America and the Caribbean super-regions were forecasted to have even more accentuated inverted age pyramids in 2100 than in 2017, under all scenarios. For all super-regions except sub-Saharan Africa and north Africa and the Middle East, we forecasted similar age structures across all scenarios. In these two super-regions and globally, the slower paced scenario on future trajectories for education and contraceptive met need resulted in substantially different age structures in 2100 (appendix 2, section 2.2). Among large countries, similar patterns were forecasted for China, India, and Indonesia on one side, with limited variation between scenarios, and Nigeria on the other, with a substantially different age pattern in the slower scenario than in the other scenarios (additional results in appendix 2, section 2).

### Economic consequences

**Figure 8:**
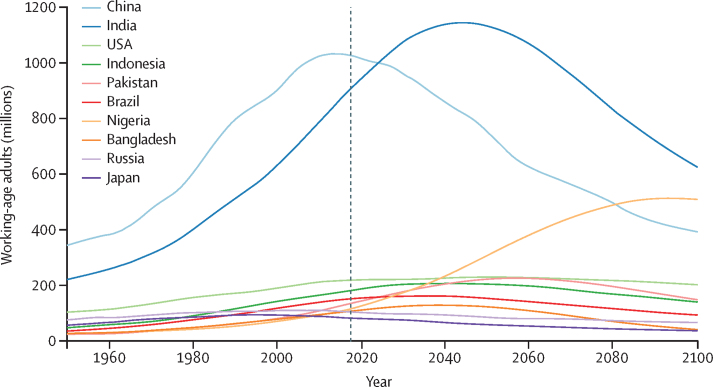
Number of working-age adults from 1950 to 2100 in the reference scenario in the ten most populous countries in 2017 Working-age adults are defined as individuals aged 20–64 years. Past data are from GBD 2017. GBD=Global Burden of Diseases, Injuries, and Risk Factors Study.

**Figure 9:**
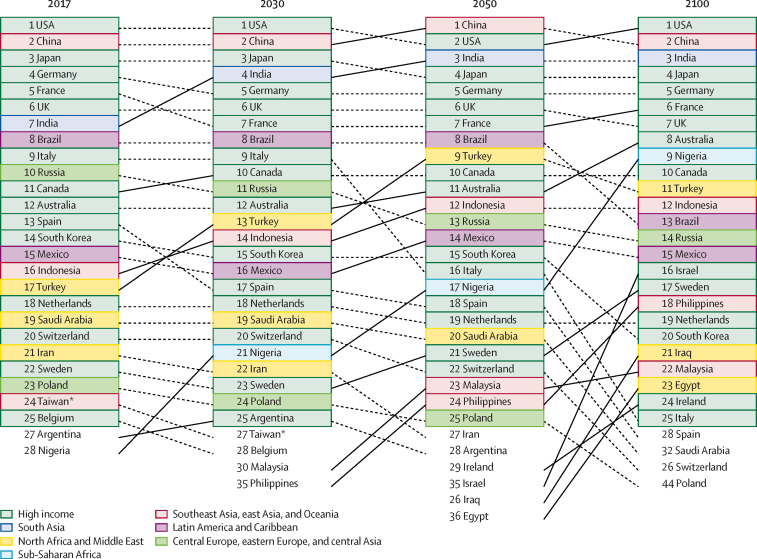
Ranking the top 25 economies by total GDP in 2017 and the reference scenario in 2030, 2050, and 2100 Countries are coloured by GBD super-region. GBD=Global Burden of Diseases, Injuries, and Risk Factors Study. GDP=gross domestic product. *(province of China).

We plotted the forecasted number of working-age individuals (aged 20–64 years) for the ten largest countries in 2017, in the reference scenario (figure 8). Huge declines in the number of workers were forecasted in China and India, alongside steady increases in Nigeria. By 2100, India was forecasted to still have the largest working-age population in the world, followed by Nigeria, China, and the USA. In our reference scenario, despite fertility rates lower than the replacement level, immigration sustained the US workforce. We translated these forecasts of working-age population into scenarios for total GDP, showing the rank order of the top 25 national economies in 2017, 2030, 2050, and 2100 under the reference scenario (figure 9). China was forecasted to rise to the top in 2035 in the reference scenario, but then was superseded by the USA again in 2098, as population decline curtailed economic growth. Other countries bolstered by immigration that rose up in the global rankings by GDP were Australia and Israel. Despite huge declines in population forecasted this century, Japan remained the fourth-largest economy in 2100. Additionally, if global labour force participation by age and sex remains the same from 2017 to 2100, the ratio of the non-working adult population to the working population might reach 1·16 globally, up from 0·80 in 2017. Detailed results for life expectancy, fertility, migration, and population are available online from the Global Health Data Exchange.

## Discussion

Our reference scenario, based on robust statistical models of fertility, mortality, and migration, suggested that global population will peak in 2064 at 9·73 billion (95% UI 8·84–10·9) and then decline to 8·79 billion (6·83–11·8) in 2100 (table). The wide variation across our fastest, faster, and slower education and contraceptive met need scenarios and our SDG pace scenario highlights the importance of policies that influence the expansion of these factors in each country. One important determinant of population growth is the rate of fertility decline in high-fertility countries, particularly those in sub-Saharan Africa. This rate of decline was driven largely by improvements in access to education and modern contraceptives. Our alternative scenarios allow us to show the demographic implications of policies that will hasten or reduce the rates of change in educational attainment and expand access to contraception, with a clear indication that deprioritising education and family planning will lead to greater population in some parts of the world.

Perhaps the most important determinant of world population in our 2100 forecasts was the rate of fertility that countries moved towards once they reached a rate lower than the replacement level of 2·1. Even differences in the post-transition TFR as small as 0·1 births per female for populations with a rate already lower than replacement level translated into 528 million (95% UI 421–573) more people on the planet in 2100. A TFR lower than replacement level was forecasted for 183 of 195 countries and territories by 2100. The UNPD forecasts have been constructed to assume that all countries will follow the pattern seen in selected low-fertility countries in Europe, east and southeast Asia, and North America, where the TFRs converge towards a level of approximately 1·75. In our model, in a population where all females have 16 years of education and 95% of females have access to contraception, the global TFR was projected to converge to 1·41 (1·35–1·47). The difference between a convergent TFR of 1·75 or 1·41 is profound in terms of long-term consequences on global population size and age structure in this century and beyond. Modelling fertility rates lower than replacement level, particularly when modelling the TFR directly, is challenging given the widely divergent realities of countries such as Singapore, Taiwan (province of China), or Japan compared with those in northern Europe. Responding to sustained low fertility is likely to become an overriding policy concern in many nations given the economic, social, environmental, and geopolitical consequences of low birth rates.

A decline in total world population in the latter half of the century is potentially good news for the global environment. Fewer people on the planet in every year between now and 2100 than the number forecasted by the UNPD would mean less carbon emission, less stress on global food systems, and less likelihood of transgressing planetary boundaries.^36^, ^37^, ^38^, ^39^ However, despite lower population, environmental and climate change might still have major and serious consequences in the intervening years unless preventive action and mitigation is vigorously pursued.^13^ Of note, the climate models of the Intergovernmental Panel on Climate Change, for the most part, have already used the Wittgenstein forecasts for population, which are much closer to those we have estimated in this study, and thus they do not fully reflect the UNPD view of continued population growth through the century.^36^, ^40^

Although good for the environment, population decline and associated shifts in age structure in many nations might have other profound and often negative consequences. In 23 countries, including Japan, Thailand, Spain, and Ukraine, populations are expected to decline by 50% or more. Another 34 countries will probably decline by 25–50%, including China, with a forecasted 48·0% decline (95% UI −6·1% to 68·4%). Population percentage declines do not immediately convey the associated profound shifts in age structure in these nations. Our findings suggest that the ratio of the population older than 80 years to the population younger than 15 years will increase in countries with more than 25% population decline, from 0·16 today to 1·50 (0·54–3·25) in 2100. These population shifts have economic and fiscal consequences that will be extremely challenging. With all other things being equal, the decline in the numbers of working-aged adults alone will reduce GDP growth rates. The forecasts in the paper by Chang and colleagues of productivity per working-aged adult that we have used assume that past trends in productivity will continue.^35^ Having fewer individuals between the ages of 15 and 64 years might, however, have larger effects on GDP growth than what we have captured here. For example, having fewer individuals in these age groups might reduce innovation in economies, and fewer workers in general might reduce domestic markets for consumer goods, because many retirees are less likely to purchase consumer durables than middle aged and young adults.^17^ Developments such as advancements in robotics could substantially change the trajectory of GDP per working-age adult, reducing the effect of the age structure on GDP growth. However, these effects are very difficult to model at this stage. Furthermore, the impact of robotics might have complex effects on countries for which the trajectory for economic growth might be through low-cost labour supply.

In countries with slower economic growth and with rising shares of the population who are retired compared with those who are still working, the fiscal sustainability of national health insurance and social security programmes will be challenged. In 2100, if labour force participation by age and sex does not change, the ratio of the non-working adult population to the working population might reach 1·16 globally, up from 0·80 in 2017. This ratio implies that, at the global level, each person working would have to support, through taxation and intra-family income transfers, 1·16 non-working individuals aged 15 years or older (the working age population is defined by the International Labour Organization as those aged 15 years or older).^41^ Moreover, the number of countries with a dependency ratio higher than 1 is expected to increase from 59 in 2017 to 145 in 2100. Taxation rates required to sustain national health insurance and social security programmes might be so large as to further reduce economic growth and investment. Insecurity from the risk that these programmes could fail might generate considerable political stress in societies with this demographic contraction. Fiscal sustainability will add profound political pressure on governments to address the challenge of population decline.

Some historians argue that the size of economies translates into geopolitical power.^42^ The rise of China and the identification of the 21st century as the Chinese century are linked to the idea that soon China will have the world's largest economy.^43^ Although GDP is not the only determinant of global political influence and power, it is a crucial factor. If the assumptions used in our reference scenario were to hold true, Russia and Brazil's relative ranking of GDP would decline moderately, whereas Spain and Italy would see substantial declines. Nigeria would increase considerably. Nations that sustain their working-age populations over the long-term through migration, such as Canada, Australia, and the USA, would fare well. Geopolitical power is also linked to military might and, at least for now, armies require individuals to serve in them. The 63·6% decline (95% UI 85·4–10·8) forecasted for China's population aged 20–24 years is a factor that should not be ignored when considering possible shifts in global power later in the century.

Some nations are aware of the economic, fiscal, and geopolitical risks of demographic decline.^17^, ^44^ Popular authors and political commentators have called attention to issues of demographic security for well over 100 years.^17^, ^42^ As nations come to recognise the challenges of fertility rates lower than the replacement level and the potential for demographic contraction, they have four options to pursue: attempt to increase the fertility rate by creating a supportive environment for females to have children and pursue their careers, restrict access to reproductive health services, increase labour force participation especially at older ages, and promote immigration. It is worth considering how each of these options might play out in different countries.

Several governments have pursued explicit policies to increase fertility rates. Some, such as Sweden, Singapore, and Taiwan (province of China), have tried to create positive environments that facilitate females choosing to have more children. These programmes include paid maternity and paternity leave, protection of re-employment rights, child care, and financial incentives for more children. Sweden has seen an increase in its TFR from 1·5 in the late 1990s to 1·9 in 2019,^17^ although the country's CCF50 has been much less affected. By contrast, positive incentives have had little effect in Singapore and Taiwan (province of China), where 2017 TFR levels were 1·26 (95% UI 1·05–1·52) for Singapore and 1·04 (0·92–1·19) for Taiwan (province of China). Unfortunately, some countries have in the past sought to increase the total fertility rate by restricting access to reproductive health services, such as the banning of abortion in Romania in 1966 and in the Soviet Union from 1936 to 1955.^45^, ^46^ A very real danger exists that, in the face of declining population, some states might consider adopting policies that restrict female reproductive health rights and access to services. Low fertility in these settings might become a major challenge to progress for females' freedom and rights.

A short-term solution to declining working-age populations is to increase labour force participation. For example, in Japan, where the number of adults aged 15–64 years declined by 7·4% between 1990 and 2015, labour force participation at ages 65–69 years increased from 15·3% to 20·8% in the same period.^47^ More generally, labour force participation among females is lower than that of males in almost all countries, so considerable opportunity exists for increased labour supply through greater access to education and employment opportunities for females. Many societies that do not choose immigration as a strategy will probably try to increase labour force participation as a temporary strategy. However, such increases are not a long-term solution because once the higher labour force participation rates are achieved, the inexorable decline in population numbers, even in those aged 15–74 years, will eventually manifest unless stabilising forces are implemented. However, developments in robotics and artificial intelligence, which have not been explicitly modelled in the economic forecasts, could substantially change economic growth. Already, economic output in some countries is becoming less tied to labour inputs, with digital technologies widening the gap between productivity and employment.^48^ In the future, technological advances might provide a solution to the decline in the workforce.

For high-income countries with fertility rates lower than the replacement level, the most immediate solution is liberal immigration policies. Among high-income countries, Canada, Australia, New Zealand, and the USA have consistently pursued this approach in the past 30 years.^28^ As long as these immigration policies continue, our reference scenario showed sustained population growth and workforce expansion in these countries, with concomitant economic growth. However, although not the case in Canada, Australia, and New Zealand, liberal immigration policies in the USA have faced a political backlash in recent years, which threatens the country's potential to sustain population and economic growth. The optimal strategy for economic growth, fiscal stability, and geopolitical security is liberal immigration with effective assimilation into these societies. Nevertheless, many other countries facing demographic decline have not adopted immigration as a strategy. Japan, Hungary, Slovakia, the Baltic states, and others are facing substantial declines in population but have not adopted immigration as a compensating strategy. In these societies, so far, the desire to maintain a linguistic and culturally homogeneous society has outweighed the economic, fiscal, and geopolitical risks of declining populations. If pronatalist policies do not yield sufficient increases in birth rates, these choices will probably have to be revisited. While a steady supply of individuals willing to migrate exists nowadays, this might change in the future as countries supplying migrants today increase education access and quality and the standard of living at home. Continued emigration supplying working-age adults to high-income economies with liberal migration policies can also have profound adverse effects on some economies through selective migration of more skilled workers.

### Technical limitations and challenges

Our model used multiple covariates, including many risk factors as drivers of mortality rates in the future and educational attainment and contraceptive met need as drivers of fertility. This approach is in sharp contrast to UNPD models, which are non-causal time-series models that do not include any covariates. Modellers disagree on whether the use of covariates beyond time is a strength or a limitation.^3^, ^49^, ^50^ The use of time alone as the driver has the advantage that time is easily forecasted, but has the strong limitation that, because time is not causal per se, these models assume that the correlation between time and true causal determinants remains the same in the past as in the future. By contrast, we explicitly built into each component of our population model the associations between drivers and the outcome, such as tobacco and lung cancer, and CCF50 and educational attainment. Explicit modelling of these associations also means that we can develop policy-relevant scenarios: what will happen in a particular place if the government invests in schools and increases educational attainment? The limitation of this approach is that each of these independent drivers must be forecasted into the future for the reference scenario. The issue is the trade-off between the benefits of modelling the explicit driver compared with the challenges of forecasting the independent drivers into the future. As Foreman and colleagues^22^ showed for mortality and we have shown here for fertility, one can include explicit drivers and maintain comparable or even better out-of-sample predictive validity than models including only time as a driver. Although our model for mortality included many drivers and our model for fertility included two dominant drivers, other factors have not been included that might limit the validity of our results for some low-income countries in the Sahel region of sub-Saharan Africa. We did not explicitly model whether countries might exceed their capacity to feed their own populations. For many, if not most countries, domestic food production is not a limit on fertility and population growth because food deficits can be solved by importing food. But for countries such as Niger, Chad, and South Sudan, which are forecasted to have large population growth while remaining low-income, a real possibility exists that such levels of population would not be sustainable. UNPD models also forecasted very large population increases for these countries, because models using time alone as the driver do not capture the Malthusian limits either.^51^ Higher rates of emigration or potentially faster declines in fertility in these countries might be the mechanism through which increases in mortality might be avoided. Although we included the natural rate of population increase in the migration model, the association might be non-linear in the future; this has not been captured in our model.

A large share of the difference between our reference scenario of 8·79 billion (95% UI 6·83–11·8) and the UNPD forecast of 10·88 billion (9·42–12·66) is related to the level of fertility that countries converge to after their fertility rate lowers below the replacement level. By focusing on CCF50, we directly captured how desired family size, access to means of managing fertility and its timing, and age-related declines in fertility interact for each cohort of females. The higher levels of forecasted fertility from UNPD models are a result of fitting their model of fertility rates lower than replacement level to a subset of countries that have had increases in TFR. Several countries with sustained low fertility were excluded from their modelling exercise. We re-estimated their exact model using all countries with fertility rates lower than the replacement level and found that their model predicted an average TFR lower than replacement of 1·48, slightly higher than the TFR forecasted by our CCF50 model (appendix 2, section 6). Additionally, the UNPD increases the predicted TFR by excluding draws from their model that are considered too low (ie, all TFR trajectories lower than 0·5 children).^15^ We conclude that both their model and ours suggest that on average, with considerable national variation, societies will tend towards a TFR of 1·5 or lower. Our analysis also showed that slight differences (0·1 difference in global TFR) in this equilibrium TFR translate into a difference of approximately 500 million individuals on the planet in 2100. We cannot know precisely what the TFR will converge towards in societies with fertility rates lower than replacement level. Given its profound importance, more research on the other determinants of fertility in low-fertility settings is needed. Another limitation of our fertility model is the random walk component. This component of the model, which improves out-of-sample predictive validity, implies that fertility rates above or below the expected observed today (ie, fertility exceptionality), conditional on educational attainment and contraceptive met need, will continue far into the future. In past data from 1950 to 2017, this appears to be the case. Assuming that average exceptionality will remain constant in logit space over the next 80 years or more is a strong assumption. Countries might tend towards the mean, which would imply less variation in fertility in 2100 and, on average, lower global TFR and lower population than in our current predictions.

Because this forecasting study is based on imperfect past data, our study had many limitations related to GBD and other data sources we used. This is particularly relevant for our approach because we rely on past time series for all countries and territories by age and sex, not only of migration, fertility, and total mortality, but also of the drivers of these three inputs to the population forecasting model. These drivers include income per capita and mean years of education, as well as all 68 individual risk factors included in GBD, vaccine coverage, and met need for contraceptives. Although data for some key drivers of population are available back to 1950, most drivers are available from 1990 to the present. Therefore, our long-term forecasts of independent drivers would improve with longer past time series. Furthermore, past trends are not always predictive of future trends, and some factors that we have not included in our model could change the pace of fertility, mortality, or migration. A fundamental challenge in making long-range forecasts, regardless of the modelling strategy, is the existence of potential changes in trends far in the future that cannot be predicted. This is a limitation of not just our methods, but also those of UNPD and Wittgenstein. Importantly, because of the challenges in incorporating climate change models, we did not include climate change in our modelling framework. Climate change is likely to have a role in future migration patterns, with populations being forced to migrate because of sea level rise, extreme weather events, environmental degradation, and more. However, better location-specific climate change forecasts are necessary to accurately incorporate these patterns into our model.

An additional limitation of our migration model is that migration data are scarce and often incomplete. Not all countries provide migration estimates and even those that do cannot fully account for illegal migration. Many unpredictable drivers of migration also exist, including war and conflict, natural disaster, economic collapse, and migration policies. These factors make future migration patterns difficult to predict, and thus the UIs for migration forecasts are very wide. Although our migration model has limitations, it is among the first to use a statistical model or to provide UIs. Better reporting of past migration rates and age-sex patterns of migration around the world would further strengthen our migration forecasts in the future.

Additionally, because our UIs for geographical aggregate values incorporate assumptions on the spatial correlation between locations, the accuracy of our uncertainty quantification could be improved through a strategy for empirically determining spatial correlation. Another limitation is that it is difficult to predict future sex ratios at birth in locations where sex ratios are skewed or have fluctuated substantially in recent history, such as China. Finally, our study is also subject to the same limitations described by Foreman and colleagues.^22^ These include the use of a rate of change model to extrapolate future trends in the independent drivers of mortality, which could be improved upon in subsequent iterations; the use of SDI as a composite measure of education, fertility, and income per capita to handle collinearity between the three drivers; and the incorporation of causal relationships between risk factors and causes of death for risk factors that are fully or partly mediated through other risks. We use risk mediation data from the GBD comparative risk assessment framework,^24^ but this work is limited by poor quantification of some mediation pathways, while others might not be known at the present. Ongoing work is needed to improve the estimation and modelling of these pathways. A full description of these limitations can be found elsewhere.^22^

### Conclusion

Global population is likely to peak well before the end of the century. Given that we forecasted that societies tend towards a TFR lower than 1·5, once global population decline begins, it will probably continue inexorably. Within the declining total world population some countries will sustain their populations through liberal immigration policies and social policies more supportive of females working and achieving their desired family size. These countries are likely to have larger overall GDP than other countries, with the various economic, social, and geopolitical benefits that come with stable working-age populations. Our UIs and scenario analysis showed that for no country or territory is the demographic future cast in stone. Policies that countries pursue today can alter the trajectory for fertility, mortality, and migration. Population size and composition are not exogenous factors for countries to account for in their planning, but rather outcomes that they can help direct.

## Data sharing

To download GBD data used in these analyses, please visit the Global Health Data Exchange GBD 2017 website. For more information on the data included in these analyses, see appendix 1, section 2.

Editorial note: the *Lancet* Group takes a neutral position with respect to territorial claims in published maps and institutional affiliations.

## References

[1] UN, Department of Economic and Social Affairs, Population Division (2019). World population prospects 2019: volume I: comprehensive tables.

[2] UN, Department of Economic and Social Affairs, Population Division (2011). World population prospects: the 2010 revision, volume 1: comprehensive tables.

[3] Alkema L, Raftery AE, Gerland P (2011). Probabilistic projections of the total fertility rate for all countries. Demography.

[4] UN, Department of Economic and Social Affairs, Population Division (2014). World population prospects: the 2012 revision, methodology of the United Nations population estimates and projections.

[5] Raftery AE, Li N, Sevcikova H, Gerland P, Heilig GK (2012). Bayesian probabilistic population projections for all countries. Proc Natl Acad Sci.

[6] UN, Department of Economic and Social Affairs, Population Division (2019). World population prospects 2019: summary of methodological updates introduced in the 2019 revision.

[7] European Commission, Joint Research Centre (2018). Demographic and human capital scenarios in the twenty-first century: 2018 assessment for 201 countries.

[8] O'Neill BC, Balk D, Brickman M, Ezra M (2001). A guide to global population projections. Demogr Res.

[9] Bos E, Vu MT, Massiah E, Bulatao RA (1994). World population projections 1994–95 edition: estimates and projections with related demographic statistics.

[10] Kaneda T, Greenbaum C, Patierno K (2018). 2018 world population data sheet.

[11] Lutz W, Butz WP, KC S (2014). World population and human capital in the twenty-first century 2014.

[12] Wittgenstein Centre (2018). Wittgenstein Centre human capital data explorer. http://dataexplorer.wittgensteincentre.org/wcde-v2/.

[13] IPCC (2018). Global warming of 1·5°C. An IPCC special report on the impacts of global warming of 1·5°C above pre-industrial levels and related global greenhouse gas emission pathways, in the context of strengthening the global response to the threat of climate change, sustainable development, and efforts to eradicate poverty.

[14] Murray CJL, Callender CSKH, Kulikoff XR (2018). Population and fertility by age and sex for 195 countries and territories, 1950–2017: a systematic analysis for the Global Burden of Disease Study 2017. Lancet.

[15] UN, Department of Economic and Social Affairs, Population Division (2017). World population prospects: the 2017 revision, methodology of the United Nations population estimates and projections.

[16] UN, Department of Economic and Social Affairs, Population Division (2019). World population prospects 2019: data booklet.

[17] Bricker D, Ibbitson J (2019). Empty planet: the shock of global population decline.

[18] Sanyal S (2011). The end of population growth.

[19] Kirk D (1996). Demographic transition theory. Popul Stud.

[20] Lee RD, Reher DS (2011). Introduction: the landscape of demographic transition and its aftermath. Popul Dev Rev.

[21] Lee R (2011). The outlook of population growth. Science.

[22] Foreman KJ, Marquez N, Dolgert A (2018). Forecasting life expectancy, years of life lost, and all-cause and cause-specific mortality for 250 causes of death: reference and alternative scenarios for 2016–40 for 195 countries and territories. Lancet.

[23] Stevens GA, Alkema L, Black RE (2016). Guidelines for Accurate and Transparent Health Estimates Reporting: the GATHER statement. Lancet.

[24] Stanaway JD, Afshin A, Gakidou E (2018). Global, regional, and national comparative risk assessment of 84 behavioural, environmental and occupational, and metabolic risks or clusters of risks for 195 countries and territories, 1990–2017: a systematic analysis for the Global Burden of Disease Study 2017. Lancet.

[25] Brockwell PJ, Davis RA (1987). Time series: theory and methods.

[26] UN, Department of Economic and Social Affairs, Population Division (2019). World population prospects 2019—fertility indicators. https://population.un.org/wpp/Download/Standard/Fertility/.

[27] Ševčíková H, Li N, Kantorová V, Gerland P, Raftery AE (2016). Age-specific mortality and fertility rates for probabilistic population projections. Dynam Dem Anal.

[28] UN, Department of Economic and Social Affairs, Population Division (2017). International migration report 2017.

[29] UN, Department of Economic and Social Affairs, Population Division (2019). Family planning and the 2030 agenda for sustainable development: data booklet.

[30] Rizzi S, Gampe J, Eilers PHC (2015). Efficient estimation of smooth distributions from coarsely grouped data. Am J Epidemiol.

[31] Westoff CF (2006). New estimates of unmet need and the demand for family planning.

[32] UN Quality education. https://www.un.org/sustainabledevelopment/education/.

[33] UN Good health and well-being. https://www.un.org/sustainabledevelopment/health/.

[34] Bill & Melinda Gates Foundation, IHME (2019). Examining inequality 2019.

[35] Chang AY, Cowling K, Micah AE (2019). Past, present, and future of global health financing: a review of development assistance, government, out-of-pocket, and other private spending on health for 195 countries, 1995–2050. Lancet.

[36] O'Neill BC, Dalton M, Fuchs R, Jiang L, Pachauri S, Zigova K (2010). Global demographic trends and future carbon emissions. Proc Natl Acad Sci USA.

[37] Shi A (2003). The impact of population pressure on global carbon dioxide emissions, 1975–1996: evidence from pooled cross-country data. Ecol Econ.

[38] Rockström J, Steffen W, Noone K (2009). Planetary boundaries: exploring the safe operating space for humanity. Ecol Soc.

[39] Steffen W, Richardson K, Rockström J (2015). Planetary boundaries: guiding human development on a changing planet. Science.

[40] KC S, Lutz W (2017). The human core of the shared socioeconomic pathways: population scenarios by age, sex and level of education for all countries to 2100. Glob Environ Change.

[41] International Labour Organization (2019). World employment social outlook: trends 2019.

[42] Morland P (2019). The human tide: how population shaped the modern world.

[43] Shenkar O (2006). The Chinese century: the rising Chinese economy and its impact on the global economy, the balance of power, and your job.

[44] Government of Japan (2019). Abenomics. https://www.japan.go.jp/abenomics/index.html.

[45] Pop-Eleches C (2006). The impact of an abortion ban on socioeconomic outcomes of children: evidence from Romania. J Polit Econ.

[46] Savage M (1988). The law of abortion in the Union of Soviet Socialist Republics and the People's Republic of China: women's rights in two socialist countries. Stanford Law Rev.

[47] Roth GA, Abate D, Abate KH (2018). Global, regional, and national age-sex-specific mortality for 282 causes of death in 195 countries and territories, 1980–2017: a systematic analysis for the Global Burden of Disease Study 2017. Lancet.

[48] Brynjolfsson E, McAfee A (2016). The second machine age: work, progress, and prosperity in a time of brilliant technologies.

[49] Girosi F, King G (2008). Demographic forecasting.

[50] Blakely T (2018). Major strides in forecasting future health. Lancet.

[51] Malthus TR (1798). An essay on the principle of population.

