# Supplementary material for: Fertility, mortality, migration, and population scenarios for 195 countries and territories from 2017 to 2100: a forecasting analysis for the Global Burden of Disease Study
Source: Lancet. 2020 Oct 17;396(10258):1285–306. doi: 10.1016/S0140-6736(20)30677-2 (PMC7561721; doi:10.1016/S0140-6736(20)30677-2)
Supplement: Supplementary appendix 1 [file mmc1.pdf]

THE LANCET

Supplementary appendix

This appendix formed part of the original submission and has been peer reviewed. We post it as supplied by the authors.

Supplement to: Vollset SE, Goren E, Yuan C-W, et al. Fertility, mortality, migration, and population scenarios for 195 countries and territories from 2017 to 2100: a forecasting analysis for the Global Burden of Disease Study. *Lancet* 2020; published online July 14. http://dx.doi.org/10.1016/S0140-6736(20)30677-2.

Appendix 1: Methods appendix to "Fertility, mortality, migration, and population scenarios for 195 countries and territories from 2017 to 2100: a forecasting analysis for the Global Burden of Disease Study"

Preamble

This appendix provides further methodological detail for "Fertility, mortality, migration, and population scenarios for 195 countries and territories from 2017 to 2100: a forecasting analysis for the Global Burden of Disease Study." This study complies with the Guidelines for Accurate and Transparent Health Estimates Reporting (GATHER) recommendations (section 2).[1] It provides a comprehensive description of our analytical processes.

Table of contents

Section 1 List of abbreviations 3
Section 2 GATHER compliance 4
Section 3 Overview of the forecasting framework 6
Section 4 Forecasting independent drivers 7
Section 4.1 Education 7
Section 4.2 Lag-distributed income 8
Section 4.3 Risk factor scalar bias correction 8
Section 5 Forecasting fertility 9
Section 5.1 Incomplete birth cohort completion 9
Section 5.2 Forecasting future birth cohorts 10
Section 5.3 Forecasting age patterns of fertility 10
Section 5.4 Age-specific fertility rates 11
Section 6 Forecasting mortality 12
Section 6.1 Forecasting cause-specific mortality 12
Section 6.1.1 All causes except HIV, disasters, war and terrorism, legal interventions 12
Section 6.1.2 HIV 13
Section 6.1.3 Disasters, war and terrorism, legal interventions 14
Section 6.2 Aggregating to all-cause mortality 14
Section 7 Forecasting migration 15
Section 7.1 Migration rates 15
Section 7.2 Converting migration rates to migrant counts 15
Section 7.3 Balancing migration globally 16
Section 8 Forecasting populations 16
Section 8.1 Cohort-component method of projection 16
Section 8.2 Robust age-specific mortality input to CCMP 16
Section 8.3 Extending age-specific mortality to 100–105 17
Section 9 Life table and life expectancy 18
Section 10 Alternative scenarios based on educational attainment and met need for contraception 19
Section 10.1 Slower, faster, fastest met need and education scenarios 19
Section 10.2 SDG pace contraceptive met need and education scenario 19
Section 10.2.1 SDG pace met need for contraception 19
Section 10.2.2 SDG pace education 19
Section 11 Uncertainty interval estimation 20
Section 12 Model evaluation 21
Section 13 References 21

Section 1 List of abbreviations

Abbreviation	Full phrase
ARIMA	Autoregressive Integrated Moving Average
AROC	Annualised rate of change
ASFR	Age-specific fertility rate
CCF	Completed cohort fertility
CCMP	Cohort-component method of projection
GBD	Global Burden of Diseases, Injuries, and Risk Factors Study
GDP	Gross domestic product
ICF	Incremental cohort fertility
IHME	Institute for Health Metrics and Evaluation
LDI	Lag-distributed income
NPI	Natural population increase
PAF	Population attributable fraction
PASFR	Proportional age-specific fertility rate
SDGs	Sustainable Development Goals
SDI	Socio-demographic Index
SEV	Summary exposure value
TFR	Total fertility rate
UI	Uncertainty interval
UNPD	United Nations Population Division

Section 2 GATHER compliance

This study complies with the Guidelines for Accurate and Transparent Health Estimates Reporting (GATHER) recommendations. We have documented the steps involved in our analytical procedures and detailed the data sources in the GATHER checklist below. The GATHER recommendations can be found here: http://gather-statement.org/

#	GATHER checklist item	Description of compliance	Reference
	Objectives and funding		
**1**	Define the indicator(s), populations (including age, sex, and geographic entities), and time period(s) for which estimates were made.	Narrative provided in the paper and appendix describing indicators, definitions, and populations	Main text (Methods - Overview) and appendix
**2**	List the funding sources for the work.	Funding sources listed in paper	Main text (Summary – Funding)
	Data Inputs		
	For all data inputs from multiple sources that are synthesised as part of the study:		
**3**	Describe how the data were identified and how the data were accessed.	Narrative provided in paper and appendix describing data seeking methods	Main text (Methods) and appendix
**4**	Specify the inclusion and exclusion criteria. Identify all ad-hoc exclusions.	Narrative provided in paper and appendix describing inclusion and exclusion criteria by data type	Main text (Methods) and appendix
**5**	Provide information on all included data sources and their main characteristics. For each data source used, report reference information or contact name/institution, population represented, data collection method, year(s) of data collection, sex and age range, diagnostic criteria or measurement method, and sample size, as relevant.	Metadata for data sources by component, geography, cause, risk, or impairment is available through an interactive, online data source tool; information on metadata for UNPD data available in the appendix	Main text (Methods), appendix, http://ghdx.healthdata.org/gbd-2017, https://population.un.org/wpp/Download/Standard/Migration/
**6**	Identify and describe any categories of input data that have potentially important biases (e.g., based on characteristics listed in item 5).	Limitations of and biases in data included in paper	Main text (Discussion – Limitations)
	For data inputs that contribute to the analysis but were not synthesised as part of the study:		
**7**	Describe and give sources for any other data inputs.	Included in online data source tools	UNPD data from: https://population.un.org/wpp/Download/Standard/Migration Wittgenstein Centre data from: https://tntcat.iiasa.ac.at/SspDb/dsd?Action=htmlpage&page=10
	For all data inputs:		
**8**	Provide all data inputs in a file format from which data can be efficiently extracted (e.g., a spreadsheet rather than a PDF), including all relevant meta-data listed in item 5. For any data inputs that cannot be shared because of ethical or legal reasons, such as third-party ownership, provide a contact name or the name of the institution that retains the right to the data.	Input data (GBD 2017 results and UNPD migration data) are available for download through the Global Health Data Exchange (GHDx) and UNPD website	GBD 2017 data: http://ghdx.healthdata.org/gbd-2017, UNPD migration data: https://population.un.org/wpp/Download/Standard/Migration/
	Data analysis		
**9**	Provide a conceptual overview of the data analysis method. A diagram may be helpful.	A brief overview and flow diagram of the overall methodological processes have been provided	Main text (Methods) and appendix
**10**	Provide a detailed description of all steps of the analysis, including mathematical formulae. This description should cover, as relevant, data cleaning, data pre-processing, data adjustments and weighting of data sources, and mathematical or statistical model(s).	Detailed descriptions of all steps of the analysis, as well as relevant mathematical formulae, have been provided	Main text (Methods) and appendix
**11**	Describe how candidate models were evaluated and how the final model(s) were selected.	Details on model evaluation and finalisation have been provided	Appendix
**12**	Provide the results of an evaluation of model performance, if done, as well as the results of any relevant sensitivity analysis.	Details on evaluation of model performance have been provided	Main text (Methods) and appendix
**13**	Describe methods for calculating uncertainty of the estimates. State which sources of uncertainty were, and were not, accounted for in the uncertainty analysis.	Details on uncertainty calculations have been provided	Main text (Methods) and appendix
**14**	State how analytic or statistical source code used to generate estimates can be accessed.	Access statement provided	Code is provided in an online repository [GitHub link to be added upon acceptance]
	Results and Discussion		

**15**	Provide published estimates in a file format from which data can be efficiently extracted.	Results are available through online tools: http://ghdx.healthdata.org/record/ihme-data/global-population-forecasts-2017-2100	Online data tools: http://ghdx.healthdata.org/record/ihme-data/global-population-forecasts-2017-2100
**16**	Report a quantitative measure of the uncertainty of the estimates (e.g. uncertainty intervals).	Uncertainty intervals are provided with results	Main text (Results and Discussion) and online data tools: http://ghdx.healthdata.org/record/ihme-data/global-population-forecasts-2017-2100
**17**	Interpret results in light of existing evidence. If updating a previous set of estimates, describe the reasons for changes in estimates.	Discussion of methodological differences between this and existing evidence (by IHME, UNPD, and Wittgenstein Centre)	Main text (Research in Context, Introduction, Methods, Discussion) and appendix
**18**	Discuss limitations of the estimates. Include a discussion of any modelling assumptions or data limitations that affect interpretation of the estimates.	Discussion of limitations was provided	Main text (Discussion – Limitations)

Section 3 Overview of the forecasting framework

The Institute for Health Metrics and Evaluation (IHME) forecasting framework uses the following inputs: estimates of past population, cause-specific mortality, fertility, and independent drivers of health from the Global Burden of Diseases, Injuries, and Risk Factors Study (GBD) 2017[2–5] as well as past migration from the United Nations Population Division (UNPD) 2017.[6] Population forecast probability distributions are obtained by generating Monte Carlo draws through a multi-stage modelling approach overviewed in appendix figure 1. First, independent health drivers (including risk exposure, education, met need for contraceptives, income, vehicles per capita, and others) are forecasted into the future, allowing for alternative scenarios based on varying rates of change of educational attainment and contraceptive met need. Next, fertility is forecasted based on education and contraception met need. Independent drivers and the Socio-demographic Index (SDI), a country-year specific composite index of fertility under 25 years, educational attainment and lag-distributed income (LDI), are then utilised to forecast cause-specific mortality. All-cause mortality rate forecasts are obtained by aggregating cause-specific mortality and then utilised to generate life tables. Migration is forecasted using SDI, mortality from war and natural disasters, and the natural rate of population increase as covariates. Finally, we obtain population forecasts applying forecasts of mortality, fertility, migration, and sex ratio at birth to the GBD 2017 starting population. Uncertainty in past data inputs, covariate and health driver forecasts, and estimated model parameters are propagated by combining draw-level data from GBD 2017 with draws from the forecast-generating model incorporating, when feasible, parameter draws from estimated sampling or posterior distributions. This allows uncertainty from each modelling stage to be propagated through the entire forecasting framework. Point estimates were computed as the mean of 1000 draws from the corresponding draw distribution and 95% uncertainty intervals (UIs) were computed using the 2.5 and 97.5 percentiles.

Many core methods used to forecast the independent drivers and mortality are described in detail in Foreman et al, 2018, a previous publication on mortality forecasting by IHME's Future Health Scenarios team.[4] Where methods did not change from this publication, the methods have only been described briefly, using language from Foreman et al.[4] Modifications made to extend independent driver and mortality forecasts to 2100, as well as detailed information on the modelling used to generate forecasts of fertility, migration, and populations are described in subsequent sections of this document.

Appendix Figure 1. Flowchart of the forecasting modelling framework

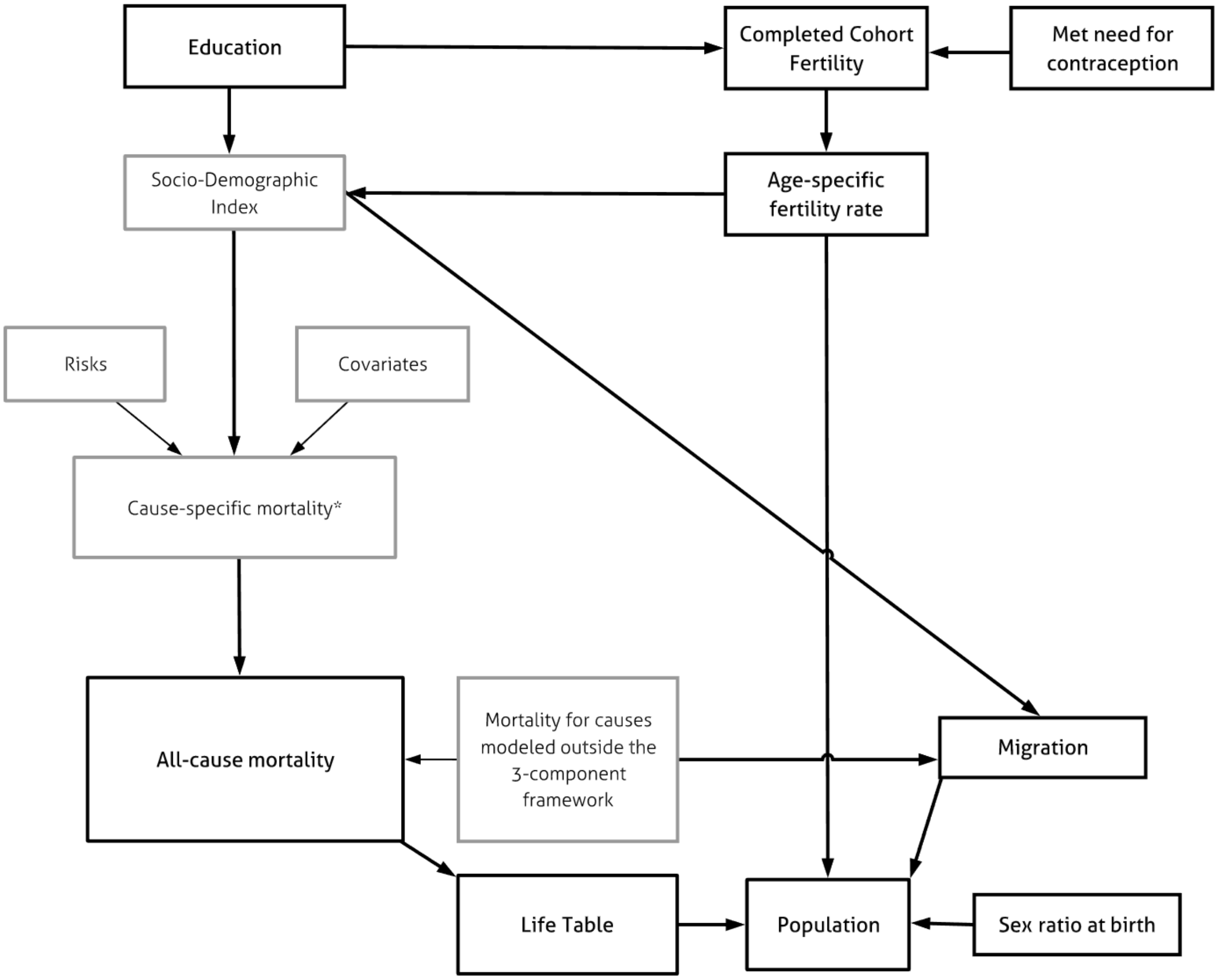

The following causes were modelled separately from the three component model: HIV, forces of nature, conflict and terrorism, and executions and police conflict.

Section 4 Forecasting independent drivers

Independent drivers of health entered our forecasting framework as determinants of fertility (section 5), cause-specific mortality (section 6.1), and migration (section 7). Forecasts of vehicles per capita, summary exposure values (SEVs), and met need for contraception were all forecasted to 2100 utilising the methods described in Foreman et al, 2018.[4] SEVs are the relative risk-weighted prevalence of exposure, where 0 is no risk in the population and 1 is the entire population at maximum risk.[7] Forecasting methods for education and lag-distributed income, two of the three components of SDI, as well as a bias-correction step in generating risk factor scalars for cause-specific mortality modelling, differed from that of Foreman et al, 2018[4] and are described in detail below. Additionally, in order to reduce the impact of extreme growth in long-range forecasts for locations with high rates of change, age-specific caps were placed at the 1st and 99th percentiles for met need for contraception, 5th and 95th percentiles for summary exposure values (SEVs), and 10th and 90th percentiles for vehicles per capita. Education forecasts utilised age-specific caps as follows: 3 years for 5-9 year olds, 8 years for 10-14 year olds, 13 years for 15-19 year olds, and 18 years for those 20 years and above. Summary exposure value forecasts utilised a narrower range of recency weights (restricted to range between 0 and 3) than that in Foreman et al, 2018.[4]

Section 4.1 Education

Education was forecasted using the methodology described in Foreman et al, 2018[4] with an added assumption that educational attainment (up to a maximum of 18 years of education) does not change after age 25. Educational

forecasts for ages 25 and below were obtained using the methods of Foreman et al, 2018[4] subject to the caps described above. After age 25, we held forecasted education constant within each location- and sex-specific birth cohort (all individuals born in a certain year). This prevented implausible within-cohort changes in education during older age and was more congruent with our cohort-specific modelling approach for fertility forecasting (section 5), for which education was a key input.

Briefly, for age groups with a starting interval of 25 years or below, we computed age-, sex-, and location-specific annualised rates of change (AROCs) by a recency-weighted average of annual differences in logit space after scaling mean years of education (based on GBD 2017 estimates) by 18 years. The recency-weighting parameters were chosen using cross-validation, where to reduce the potential for overfitting, we selected the parameter producing the smallest root-mean square error at least 5% greater than the minimum. These AROCs were applied to GBD 2017 draws to produce forecast draws, denoted $\widehat{EDU}_{lastd}$, of mean years of education for location l, age $a \leq 25$, sex s, future years $t = 2018, \ldots, 2100$, and draw d. For age groups with interval starts $a > 25$, the forecasted value was set to the previous value on the cohort trajectory, which is lagged in time by the age-group interval (5 years) due to the relationship

$$cohort\ birth = time - age.$$

Specifically, for age groups indexed by the interval start $a = 30, 35, \ldots, 95$ this is given by

$$\widehat{EDU}_{lastd} = \begin{cases} EDU_{l(a-5)s(t-5)d}, & t \leq 2017 + 5 \\ \widehat{EDU}_{l(a-5)s(t-5)d}, & t > 2017 + 5 \end{cases}$$

where $EDU_{l(a-5)s(t-5)d}$ and $\widehat{EDU}_{l(a-5)s(t-5)d}$ denote draws of past GBD and future forecasts, respectively.

Section 4.2 Lag-distributed income

Lag-distributed income (LDI) per capita, which is a moving average transformation of gross domestic product (GDP) per capita and is one of three components of the SDI, was used in forecasting cause-specific mortality. Retrospective (past) GDP was estimated using methods from James et al, 2012[8] and computed as GDP per working age adult as follows:

$$GDP_{working\ population} = GDP_{total\ population} \times \frac{Workingpopulation(20\text{–}64yearsold)}{Total\ population}.$$

Using GDP per worker, as compared to GDP per capita, more accurately estimated economic growth in countries and territories with demographic trend bubbles in out-of-sample predictive validity testing. LDI was computed by conducting a natural log transformation of average GDP with a 10-year lag.[9] Future LDI values were forecasted to capture country-specific trends that shrink towards the global trend over time. This was accomplished by projecting the previous 35 years of retrospective data forward for approximately 35 years without any changes, and then applying a decay function to the random intercept effect of country-specific time trends for the next 20 years. We did not adjust other covariates in this decay function.[2]

Section 4.3 Risk factor scalar bias correction

Cause-specific mortality risk factor scalars were input to the mortality modelling described in section 6. These involved estimation of a risk-specific population attributable fraction (PAF), which is detailed in Foreman et al, 2018.[4] To allow for protective effects in certain risk-cause pairs (eg, alcohol and ischemic heart disease in certain populations and age groups), we made the following adjustment to the PAF correction factor estimation and application. We let $PAF_{rila(2017)d}$ and $\widehat{PAF}_{rila(2017)d}$ denote the GBD and estimated PAFs, respectively, for risk factor r, cause of death i, location l, age a, and draw d in the year 2017. We computed the correction factor by

$$\widehat{CF}_{rilad} = \text{logit}\left(\frac{1 + PAF_{rila(2017)d}}{2}\right) - \text{logit}\ \left(\frac{1 + \widehat{PAF}_{rila(2017)d}}{2}\right)$$

This was used to adjust forecast draws of the PAFs according to

$$\widehat{PAF}^{*}_{rilatd} = 2\ \times\ \text{expit}\left(\text{logit}\left(\frac{1 + \widehat{PAF}_{rilatd}}{2}\right) + \widehat{CF}_{rilad}\right) - 1$$

where $\widehat{PAF}^{*}_{rilatd}$ and $\widehat{PAF}_{rilatd}$ represent the corrected and uncorrected PAF, respectively, for risk factor r, cause i, location l, age a, time t, and draw d. This procedure constrains the forecasted PAFs to range from -1 to 1, as opposed to 0 to 1 in the correction procedure described by Foreman et al, 2018.[4]

Section 5 Forecasting fertility

Forecasts for age-specific fertility rates (ASFR) were used as a direct input to the populations forecasting model (section 8) and in forecasting future SDI, which is an input to cause-specific mortality (section 6.1) and migration (section 7) models. ASFR measures the number of livebirths each woman in that age group has in a given year. We modelled fertility by first forecasting completed cohort fertility by age 50 (CCF50) using GBD 2017 fertility estimates where the time series begins in 1950, and then deriving the implied ASFR using a series of cohort age-specific models.

CCF50 is defined as the average number of children born to an individual female from an observed birth cohort (indexed by year of birth), if she lived to the end of her reproductive lifespan (from age 15 to 49). Calculation of CCF50 requires follow-up through age 49 in order to reach end of reproductive lifespan. Consequently, cohorts born between 1969 and 2002 (corresponding to women aged 15 to 48 in 2017, the last observed year) require fertility forecasts for the unobservable reproductive years (up to age 50) they have not yet experienced. To overcome this challenge of incomplete cohorts, we first forecasted the remaining fertility for incomplete birth cohorts from 1969 to 2002 using past history of fertility at the last observed age for that cohort (section 5.3). These partially forecasted and past CCF50 for birth cohorts from 1955 to 1968 were then used to forecast CCF50 for birth cohorts from 2003 to 2085 (section 5.2). We derived ASFR forecasts from the year 2018 to 2100 using incremental cohort models to estimate the age pattern of fertility for each cohort (sections 5.3-5.4).

Section 5.1 Incomplete birth cohort completion

For birth cohorts that are only partially completed at the last observed year (2017) we forecasted fertility that would occur from last observed age up to age 50 using the past observed fertility. We utilise cumulative (as opposed to completed) cohort fertility up to age a, defined as the average number of children born to an individual female from an observed birth cohort by age a, denoted here by $CUCF_{lca}$ for location l, cohort c, and age interval start within reproductive lifespan $a = 20, 25, \ldots, 45, 50$. Note that cumulative cohort fertility at age 50, the assumed end of reproductive lifespan, equals CCF50, denoted here by CCF_{lc} for location l and birth cohort c.

To forecasting the remaining fertility between age $a = 20{,}25, \ldots 45$ and age 50, represented by the difference $CCF_{lc} - CUCF_{lca}$, we used the model given by

$$E[Y_{lca}] = \beta_{0a} + ns_{df=3}(CUCF_{lca})$$
$$Y_{lca} = \ln(CCF_{lc} - CUCF_{lca})$$

where β_0 is an intercept and $ns_{df=3}(CUCF_{lca})$ represents a natural cubic spline on $CUCF_{lca}$with two quantile based knots. This model was fit with least squares independently for ages $a = 20{,}25, \ldots 45$ using past data from complete cohorts $c = 1955, \ldots, 1968$.

Past estimates of log-space completed fertility based on the fitted values from the above model, $\widehat{Y_{lca}}$, were computed by

$$\ln \widehat{CCF}_{lca} = \ln\left[CUCF_{lca} + \exp(\widehat{Y_{lca}})\right]$$

To account for trends in cumulative fertility by age 50 unexplained by fertility up to age a, we utilised a random walk ($ARIMA_{(0,1,0)}$) model for the log-space residuals in CCF50 fit using the past residuals

$$\hat{e}_{lca} = \ln(CCF_{lc}) - \ln(\widehat{CCF}_{lca}).$$

Future residual draws $\hat{e}_{lcad}$were generated from the fitted random walk model and added to the past fertility $CUCF_{lca}$ at the oldest observed age available for the past, which corresponds to cohorts $c = 1969, \ldots, 1973$ for $a = 45$; $c = 1974, \ldots, 1978$ for $a = 40$; …; $c = 1994, \ldots, 2002$ for $a = 20$. Final forecast draws of completed CCF50 were obtained by exponentiating

$$\ln\left[CUCF_{lca} + \exp(\widehat{Y_{lcad}})\right] + \hat{e}_{lcad}$$

with correction for mean bias arising from modelling in log space using a similar procedure as done for mortality (section 6.2), where $\hat{Y}_{lcad}$ represents draw d of $E[Y_{lca}]$ from its sampling distribution. All forecasted CCF50 draws below one were set to one.

Section 5.2 Forecasting future birth cohorts

Using the combined data of observed CCF50 for birth cohorts from 1955 to 1968 and partially forecasted CCF50 for incomplete birth cohorts from 1969 to 2002, we forecasted future CCF50 for each location and birth cohort using female education and the proportion of met need for contraception (both at age 25). We utilised the following regression model:

$$CCF_{lc} = \beta_0 + ns_{df=4}\left(edu_{lc(25)}\right) + \beta_{mn} mn_{lc(25)} + \eta_{lc}$$

where β_0 is an intercept, $ns_{df=4}\left(edu_{lc(25)}\right)$ represents a natural cubic spline with 4 degrees of freedom applied to female education at age 25, β_{mn} is a slope on proportion of met need for contraception at age 25, and η_{lc} is a residual term. Values of CCF50 for location l and cohort c, CCF_{lc}, are either GBD 2017 estimates ($c = 1955, \ldots, 1968$) or those forecasted to completion using incremental cohort models ($c = 1969, \ldots, 2002$). Internal knot placements in the natural cubic spline were set to 3.1, 8.14, and 12.8 approximating the 10th, 36.5th, and 75th percentiles, respectively, of education.

We assumed a random walk model for the residual terms, which capture trends in CCF50 unexplained by education and contraceptive met need, after transformation to scaled logit space. This is defined for transformation of x by the logit transformation of x scaled from (a, b) to (0, 1):

$$\text{logit}_{(a,b)}(x) = \ln\left(\frac{x-a}{b-x}\right).$$

For each location, we estimated the random walk model variance using the past-time scaled logit-space residuals

$$\hat{\eta}_{lc} = \text{logit}_{(1,10)}(CCF_{lc}) - \text{logit}_{(1,10)}\left(\widehat{CCF}_{lc}\right).$$

Draws of future scaled logit-space residual draws $\hat{\eta}_{lcd}$ for future cohorts $c = 2003, \ldots, 2085$ were generated from the fitted random walk model centred at GBD draws of the last observed cohort residual for that location, $\hat{\eta}_{l(2002)d}$ to incorporate draw-level uncertainty from GBD 2017:

$$\hat{\eta}_{lcd} \sim N\left(\hat{\eta}_{l(2002)d}, (c-2002)\hat{\sigma}_l^2\right)$$

These forecasted residuals were added to forecast draws of CCF50 that were obtained from the regression model evaluated at draws of the regression coefficients from their estimated sampling distribution and forecast draws of education and proportion of met need for contraception, denoted $\widehat{CCF}^*_{lcd}$. Final forecast draws of CCF50, denoted $\widehat{CCF}_{lcd}$, were obtained by

$$\widehat{CCF}_{lcd} = \text{expit}_{(1,10)}\left[\text{logit}_{(1,10)}\left(\widehat{CCF}^*_{lcd}\right) + \hat{\eta}_{lcd}\right] - BC_{lc},$$

where $\text{expit}_{(a,b)}(x) = (b-a)\frac{\exp(x)}{1+\exp(x)} + a$ is the inverse scaled-logit (expit) transformation and BC_{lc} is a bias correction factor that mitigates bias in the mean of the residuals introduced by modelling in scaled-logit space and is computed by

$$BC_{lc} = \frac{1}{1000}\sum_{d=1}^{1000} \text{expit}_{(1,10)}\left[\text{logit}_{(1,10)}\left(\widehat{CCF}^*_{lcd}\right) + \hat{\eta}_{lcd}\right] - \text{expit}_{(1,10)}\left(\frac{1}{1000}\sum_{d=1}^{1000} \text{logit}_{(1,10)}\left(\widehat{CCF}^*_{lcd}\right) + \hat{\eta}_{lcd}\right).$$

As with cohort completion forecasting, all forecasted CCF50 draws below one were set to one.

Section 5.3 Forecasting age patterns of fertility

Age-specific fertility patterns are required to obtain ASFR forecasts from CCF50 forecasts. To estimate these patterns, we utilised incremental cohort fertility models on the log difference between cumulative cohort fertility at subsequent five-year age intervals. We modelled incremental cohort fertility (ICF) in five-year age intervals, defined

as the average additional number of children birthed during that age interval. Let $ICF_{lc(a,a-5)}$ be the ICF from age a to $a-5$ for location l and cohort c, which can be determined by the difference in cumulative cohort fertility as

$$ICF_{lc(a,a-5)} = CUCF_{lca} - CUCF_{lc(a-5)},$$

where $CUCF_{lca}$ is the cumulative fertility up to age a for location l and cohort c (as defined in section 5.1).

Using past time data (based on GBD 2017 estimates), we fit the following age-specific models (in log space) using least-squares estimation (fit separately to each age):

$$\ln\left(ICF_{lc(a,a-5)}\right) = \begin{cases} \beta_{0,a} + ns_{kt@9}(edu_{lca}) + \beta_{mn,a}mn_{lca} + \eta_{lca}, & a = 20 \\ \beta_{0,a} + cs_{kt@12}(edu_{lca}) + \beta_{mn,a}mn_{lca} + ns_{df=5}(\ln\left(ICF_{lc(a-5,a-10)}\right) + \eta_{lca}, & a = 25 \\ \beta_{0,a} + \beta_{edu,a}edu_{lca} + \beta_{mn,a}mn_{lca} + ns_{df=5}(\ln\left(ICF_{lc(a-5,a-10)}\right) + \eta_{lca}\,, & a = 30, \ldots, 50 \end{cases}$$

where edu_{lca} and mn_{lca} are years of education and proportion of met need for contraception, respectively, for location l, birth cohort c, and age a; $\beta_{0,a}$ is an age-specific intercept; $\beta_{mn,a}$ is an age-specific slope on met need; $\beta_{edu,a}$ is an age-specific slope on education; $ns_{kt@K}(x)$ denotes a natural cubic spline with one internal knot at K on the covariate x; $cs_{kt@K}(x)$ denotes a cubic spline with one internal knot at K on the covariate x; $ns_{df=K}(x)$ denotes a cubic spline with K degrees of freedom ($K-1$ knots based on quantiles of x); and η_{lca} is a residual term.

Residuals η_{lca} in the above model represent trends not captured by education and contraceptive met need. We fit a random walk model to the past-time estimated residuals,

$$\hat{\eta}_{lca} = \ln\left(ICF_{lc(a,a-5)}\right) - \ln\left(\widehat{ICF_{lc(a,a-5)}}\right),$$

for each location l and age bin $a = 20, 25, \ldots, 50$. Using the fitted random walk model variance estimate $\hat{\sigma}^2_{la}$, we generated draws of forecasted future residual trends, $\hat{\eta}_{lcad}$, for incomplete cohorts $c = 1969, \ldots, 2002$ centered at the last observed cohort residual for that location and age, $\hat{\eta}_{l(2017-a)a}$:

$$\hat{\eta}_{lcad} \sim N\left(\hat{\eta}_{l(2017-a)a}, (c + a - 2017)\hat{\sigma}^2_{la}\right)$$

These residual draws were added to forecasted log ICF draws, $\ln\left(\widehat{ICF_{lc(a,a-5)d}}\right)$, obtained by evaluating the regression model at forecasted covariate draws $\widehat{edu}_{lcad}$ and $\widehat{mn}_{lcad}$ and covariate draws from a multivariate normal distribution with mean and variance set to the estimated regression coefficients and their variance-covariance matrix, respectively.

Final forecast draws of ICF, denoted $\widehat{ICF}_{lc(a,a-5)d}$, were obtained by exponentiating

$$\ln\left(\widehat{ICF_{lc(a,a-5)d}}\right) + \hat{\eta}_{lcad}$$

with correction for mean bias arising from modelling in log space using a similar procedure as done for mortality (section 6.2).

For completion of incomplete cohorts, CCF50 was obtained by combining the observed cumulative cohort fertility in 2017 with the cumulative sum of the forecasted ICFs up to age 50:

$$\widehat{CCF}_{lcad} = ICF_{lc(15,2017-c)d} + \sum_{a=2018-c}^{50} \widehat{ICF}_{lc(a,a-5)d},$$

Where $ICF_{lc(15,2017-c)d}$ denotes the GBD ICF from age 15 to the last observed age for that incomplete cohort, age $2017-c$.

Section 5.4 Age-specific fertility rates

ASFRs were forecasted using the fitted ICF models described in section 5.3 to estimate the proportional age-specific fertility rate (PASFR), defined as the proportion of CCF50 composed by a given age group. We then obtained ASFR

utilising the relationship between PASFR, CCF50, and ASFR. At a given single-year age a, the PASFR can be expressed as

$$PASFR(a) = \frac{ASFR(a)}{\sum_{a=15}^{49} ASFR(a)} = \frac{ASFR(a)}{CCF50}.$$

Thus, the single-year ASFRs can be computed as

$$ASFR(a) = PASFR(a) \times CCF50.$$

Forecast draws of PASFR were estimated using location-, cohort-, and age-specific forecast draws of ICF for cohort births $c = 2018 - a, \dots, 2085$ by extending the forecast horizon in the ICF model of section 5.3 and computing the implied PASFR between age a and $a - 5$:

$$\widehat{PASFR}_{lc(a,a-5)d} = \frac{\widehat{ICF}_{lc(a,a-5)d}}{I(c < 2003)ICF_{lc(15,2017-c)d} + \sum_{a=2018-c}^{50} \widehat{ICF}_{lc(a,a-5)d}}$$

where $I(\cdot)$ is the indicator function. Note the denominator is CCF50 as forecasted using the ICF model, as opposed to CCF50 forecasted using the model of Section 5.2 which considers only total, rather than incremental, fertility.

We then applied these forecasted PASFR draws to the forecasted CCF50 draws (obtained using the methods of section 5.2) to obtain forecasted ASFR draws by single-year age a as follows:

$$\widehat{ASFR}_{lcad} = \frac{\widehat{PASFR}_{lcad} \times \widehat{CCF}_{lcd}}{5}$$

where $\widehat{PASFR}_{lcad}$ is the single-year age PASFR value obtained by assuming it is constant for all single-year ages in the corresponding 5-year age interval. To obtain final ASFR forecast draws by age group for years $t = 2018, \dots, 2100$, we averaged over the ages within the age group interval for that year. Discontinuities between location-, age-, and draw-specific ASFR values for the last past year, 2017, and first forecasted year, 2018, were reduced using a three-point moving average iterated five times over future years in period space. The resulting adjusted ASFRs were raked to cohort space CCF50 to preserve those forecasted values.

Section 6 Forecasting mortality

All-cause mortality was forecasted by modelling 215 collectively exhaustive and mutually-exclusive causes independently, and separately for males and females, then aggregating to all-cause and modelling all-cause latent trends. Details of this approach are described in Foreman et al, 2018,[4] which we extended with some modifications to ensure stability of forecasts out to 2100. Briefly, a three-component cause-specific model was utilised for 270 of the 274 causes and cause groups, while the remaining four causes (HIV, exposure to forces of nature, conflict and terrorism, executions) were modelled with alternative approaches. From the all-cause mortality rates produced from aggregating cause-specific mortality and modelling all-cause latent trends, we generated life tables for use in the population cohort-component model described in section 8.1.

Section 6.1 Forecasting cause-specific mortality

Section 6.1.1 All causes except HIV, disasters, war and terrorism, legal interventions

The cause-specific model, used for 270 of 274 causes and cause groups, is composed of three components:

1. The underlying (or risk deleted) mortality, modelled as a function of the SDI[3], time, and additional cause-specific covariates where appropriate.
2. A risk factor scalar that captures cause-specific combined risk factor effects based upon the GBD comparative risk assessment, which quantifies risk-outcome associations accounting for risk factor mediation.[5]
3. Unexplained residual mortality.

Specifically, the total mortality rate m_{ilast}^{T} for cause i, location l, age group a, and sex s at time t was decomposed in log space into an underlying mortality rate m_{ilast}^{U}, a risk factor scalar $\mathbb{S}_{ilast}$, and residual ϵ_{ilast} as

$$\ln(m_{ilast}^T) = \underbrace{\alpha_{ilas} + \beta_{is}\mathrm{SDI}_{lt} + \theta_{ias}\mathrm{t}}_{\ln(m_{ilast}^U)} + \ln(\mathbb{S}_{ilast}) + \epsilon_{ilast}$$

where for the ith cause and sth sex , $\alpha_{ilas} \sim \mathrm{N}(\beta_{\alpha,is}, \tau_{\alpha,is}^2)$ is a location-age-specific random intercept, β_{is} is a global fixed slope on SDI, and $\theta_{ias} \sim \mathrm{N}(\beta_{\theta,is}, \tau_{\theta,is}^2)$ is an age-specific random slope on the secular time trend. This model has the option to place Girosi-King type priors[10] on the log total cause-specific mortality rate to smooth forecasts over age, time, and location by penalising large differences in adjacent ages, times, and locations. The residuals ϵ_{ilast} represent latent trends in total cause-specific mortality not captured by risk factors, SDI, and global secular trends. Note our model lacks the spline on SDI used in Foreman et al, 2018[4] to increase long-term forecast stability. Forecasting methodology for the independent drivers used to compute SDI and the risk factor scalar is described in section 4. Additional covariates for specific causes were included as in Foreman et al, 2018[4] with the modification of applying the natural log transformation to the SEV covariates high plasma fasting glucose and impaired kidney function. To prevent time trend reversals in demographic subgroups, when the mean slope on time, $\beta_{\theta,is}$, was estimated to be non-positive, the overall slope on time, $\theta_{ias} + \beta_{\theta,is}$ was constrained to be non-positive.

Draws of non-latent cause-specific total mortality forecasts at time t are generated in several steps. First, independent drivers (detailed in section 4) used to compute the forecasted risk factor scalar $\widehat{\mathbb{S}}_{ilastd}$ and covariate $\widehat{\mathrm{SDI}}_{ltd}$, where d has been added to index draw. To account for uncertainty in model estimation, draws of the fixed and random effects were generated from a multivariate normal distribution with mean and variance matrix set to the estimates and their estimated joint covariance matrix, respectively, computed using the TMB package for R[11] using the GBD estimates of past mortality and assuming $\epsilon_{ilast} \sim N(0, \sigma_{is}^2)$. Forecast draws of the underlying mortality, $\ln(\widehat{m_{ilastd}^U})$, are obtained by evaluating the model at these parameter and SDI forecast draws. Cause-specific non-latent mortality forecast draws are obtained by adding draw-specific forecasts of the underlying mortality rate and risk factor scalar, and used in obtaining all-cause mortality forecasts as described in section 6.2.

Section 6.1.2 HIV

HIV mortality was forecasted using the Spectrum modelling process, as described in Foreman et al[4] with the following modifications. HIV-related health financing was input based on HIV care and treatment estimates from IHME, 2018.[12] These direct estimates of overall HIV-specific spending showed better performance and predictive validity for modelling HIV rates than the previously utilised measures, which were HIV-specific development assistance for health and government health expenditure per capita.

Secular trends in the counterfactual incidence hazard were projected based on a time-dynamic AROC model. The Spectrum model uses incidence hazard, the rate of new HIV infections among the susceptible population, as a key input. We defined rates of change relative to the counterfactual incidence hazard, which is the expected rate when ART coverage is zero. We let HC_{lkd} denote the counterfactual hazard for location l, number of years k since that location's epidemic start at year t_l^*, and draw d (calculated for both sexes aged 15-49). The AROC was computed in log space based on the draw-averages $\overline{HC}_{lk} = \frac{1}{D}\sum_d HC_{lkd}$ as

$$d_{lk} = \ln\left(\overline{HC}_{l(t_l^*+k)}\right) - \ln(\overline{HC}_{l(t_l^*+k-1)})$$

for $k = 1, \dots, x$. The maximum years since epidemic start, x, was determined such that after x years, fewer than half of locations (countries or territories) had an HIV epidemic extending that many years. All scenarios utilised the median AROC across all locations:

$$\delta_k = \underset{l}{\mathrm{median}}\{d_{lk}\},$$

for each year $k = 1, \dots, x$ during the epidemic. We allowed the AROC for each location to follow the characteristic course of the HIV epidemic over time: growth in incidence at the outset, followed by levelling off, then decline to zero. To capture such trends, we defined time-dynamic AROCs based on the year of epidemic start for each location. For each location, we projected counterfactual incidence starting in year 2018. We assumed each epidemic reaches equilibrium within 50 years, so we interpolated to 1 at $k = 50$ years after the epidemic start:

$$\Delta_{lt} = \begin{cases} \delta_{(t-t_l^*)} & \text{for } t = 2018, \ldots, t_l^* + x \\ \dfrac{1-\delta_x}{t_l^* + 50 - t} & \text{for } t = t_l^* + x + 1, \ldots, t_l^* + 50 \\ 1 & \text{for } t = t_l^* + 51, \ldots, 2100 \end{cases}$$

These time-dynamic AROCs were used to forecast counterfactual hazards at future times $t = 2018, \ldots, 2100$ according to

$$\widehat{HC}_{ltd} = \exp\left[\ln\left(HC_{l(2017)d}\right) + \Delta_{lt}\right].$$

Section 6.1.3 Disasters, war and terrorism, legal interventions

We forecasted cause-specific mortality due to exposure to forces of nature, conflict and terrorism, and executions and police conflict following the methods in Foreman et al, 2018[4] with alterations for long-range forecast stability. We removed the LOESS regression model with SDI utilised for discounting mortality forecasts generated by resampling from past mortality. Additionally, for the causes conflict and terrorism and executions, we adjusted the probability of sampling past years 1950-2017 to up-weight more recent years according to an exponential decay function, as opposed to being uniformly sampled from the past years. The probability of sampling past year t for a given forecast year was set to be proportional to $\exp[-0.15(2017 - t)]$.

Section 6.2 Aggregating to all-cause mortality

All-cause total mortality forecasts were obtained by aggregating cause-specific non-latent mortality forecasts (based on risk factors, SDI and global secular trends) and adding in forecasted all-cause latent trends, described previously[4] for forecasting at each cause-level in the GBD hierarchy but only performed here for all-cause aggregation with slight modifications detailed below.

At future (past) times t, the age-, sex-, and location-specific all-cause non-latent mortality, denoted by m_{lastd}^{NL}, was forecasted (estimated) by aggregation of cause-specific estimates and forecasts (after exponentiation out of log space):

$$\widehat{m_{lastd}^{NL}} = \sum_{i=1}^{274} \exp\left[\ln\left(\widehat{m_{ilastd}^{U}}\right) + \ln\left(\widehat{\mathbb{S}}_{ilastd}\right)\right].$$

Latent all-cause trends were modelled using the unexplained residual mortality at the all-cause level by a random walk with attenuated drift to diminish latent trends over future time. Past-time all-cause residuals were computed in log space (using the mean of the reference scenario draws) as

$$\hat{\epsilon}_{last} = \frac{1}{D}\sum_{d}\left[\ln\left(m_{lastd}^{T}\right) - \ln\left(\widehat{m_{lastd}^{NL}}\right)\right]$$

for $t = 1990, \ldots, 2017$ where m_{lastd}^{T} is the GBD draw of all-cause total mortality for location l, age a, sex s, time t, and draw d. We estimated past time drift using the linear regression model with mean

$$\mathrm{E}(\hat{\epsilon}_{last}) = \gamma_{0,las} + \gamma_{1,las} t,$$

where $\gamma_{0,las}$ is an intercept and $\gamma_{1,las}$ is a slope on time for past times $t = 1990, \ldots, 2017$. Beginning at the first forecasted year $t_0 = 2018$, future latent trend forecasts were generated with slope attenuation according to

$$\hat{\epsilon}_{las(t+1)} = \hat{\epsilon}_{last} + \gamma_{1,las} \exp[-0.1(t - t_0)].$$

This approach produced more plausible long-term forecasts than the ARIMA blend used by Foreman and colleagues, 2018.[4]

Final all-cause log total mortality forecasts were generated by drawing latent trends from the fitted random walk with drift model, denoted by $\hat{\epsilon}_{lastd}$, and adding these to the non-latent forecasts with an adjustment to account for uncertainty in the input GBD estimates. Notably, estimation of both the non-latent and latent trends made use of past-time GBD estimates, which are means across the corresponding GBD draw distributions. To incorporate

uncertainty in the supplied GBD estimates and ensure continuity between past and forecast means, we intercept-shifted the non-latent all-cause mortality forecast draws (in log space) by the draw-level residual in 2017 non-latent all-cause mortality. Specifically, the shifts were defined by

$$\hat{\zeta}_{lasd} = \ln(m^T_{las(2017)d}) - \ln(\widehat{m^T_{las(2017)d}}),$$

where $m^T_{las(2017)d}$ is the dth GBD draw of location-, age-, and sex-specific all-cause total mortality in 2017. In log space, the all-cause total mortality forecasts were computed as

$$\ln(\widehat{m^T_{lastd}}) = \ln(\widehat{m^{NL}_{lastd}}) + \hat{\epsilon}_{lastd} + \hat{\zeta}_{lasd}.$$

Since the log of the mean of a log normally distributed random variable is biased away from the mean of the corresponding normal distribution by an additive factor of the half the variance of the normal distribution, we applied the following bias correction in exponentiation to obtain final all-cause total mortality forecasts in the original mortality rate space:

$$\widehat{m^T_{lastd}} = \exp\left[\ln(\widehat{m^T_{lastd}}) + \frac{\hat{s}^2_{last}}{2}\right].$$

where $\hat{s}^2_{last}$is the sample variance (across the draws) of $\ln(\widehat{m^T_{lastd}})$.

Section 7 Forecasting migration

We forecasted net migration rates for each country or territory as a function of natural population increase (NPI); SDI; and mortality resulting from natural disasters, wars, execution and police conflict, and terrorism (eg, events that can be considered cultural and/or economic shocks). We utilised UNPD estimates of past migration rates and NPI[6,13] in conjunction with GBD 2017 estimates of past mortality and SDI to model migration rates (section 7.1). Migration rate forecasts were then used to adjust migrant counts using Eurostat migrant population data[14,15] (section 7.2) and balanced across locations to achieve zero-net migration globally (section 7.3). The resulting balanced net migration counts by sex, age, location, and time were utilised to generate population forecasts using the cohort-component method of projection (CCMP) (section 8.1).

Section 7.1 Migration rates

For country or territory l at year t, we fit the following multiple linear regression model for the migrate rate using least squares estimation with past times $t = 1990, 1991, \ldots, 2016, 2017$:

$$MIGR_{lt} = \beta_0 + \beta_{NPI} NPI_{lt} + \beta_{SDI} SDI_{lt} + \beta_{shock} SHOCK_{lt} + \varepsilon_{lt},$$

where $MIGR_{lt}$is the UNPD migration rate estimate (annual net number of migrants per 1000 people); $NPI_{lt}$is the UNPD natural population increase estimate (difference between crude birth and death rates per 1000 people); SDI_{lt} is the GBD SDI; $SHOCK_{lt}$ is the GBD mortality rate estimate aggregated over all ages and both sexes and summed across the causes natural disasters, war, terrorism, and legal interventions; and ε_{lt} is a residual accounting for variation in location- and time-specific migration unexplained by the model.

We utilised a random walk with attenuated drift model to forecast the residuals. For each location, past-time residuals were computed as $\hat{\varepsilon}_{lt} = MIGR_{lt} - \widehat{MIGR}_{lt}$ and were used to fit the random walk model using the same approach described in section 6. Future forecasts of the migration rate, denoted $\widehat{MIGR}_{ltd}$, were generated by evaluating the estimated regression model at the UNPD forecasted estimates for NPI and our mean forecasts of SDI (section 4) and cause-specific mortality for natural disasters, war, terrorism and legal interventions (section 6.1.3) then adding this value to forecast draws of the residuals, $\hat{\varepsilon}_{ltd}$. Finally, to prevent implausibly extreme long-range trends in migration, migration rate forecast draws $\widehat{MIGR}_{ltd}$ were capped between -10 and 10, which approximates the 5th and 95th percentiles of past migration rates.

Section 7.2 Converting migration rates to migrant counts

Forecast draws of location-specific migration rates, $\widehat{MIGR}_{ltd}$, were converted to net migrant counts, denoted by $\hat{Y}_{ltd}$, using a population forecast generated with CCMP under zero migration. Since CCMP requires input on migration by age and sex, we disaggregated the net migrant counts by age group and sex. We computed age- and sex-specific patterns of migration that were applied to each location by scaling the net migrant counts. For most locations, these

were computed by the geometric mean of migrants for each age and sex using Eurostat migration data from 2006-2015.[14,15] Specifically, we applied past age-sex patterns from European Union member countries to decompose migration rate forecasts by age and sex in all locations except the gulf countries Saudi Arabia, Bahrain, United Arab Emirates, and Oman. In these gulf countries, most recent migration was by young adult male temporary workers, so we instead utilised Qatar census data from 2015. Unlike in countries that Eurostat provides data for, Qatar's recent census captures this uptick in young adult male migration. We produced age- and sex-specific migration patterns for that subset of gulf countries using a Bayesian demographic balancing model fit with Qatar. This methodology is described in full elsewhere.[3]

Section 7.3 Balancing migration globally

In order to balance the global net migration to zero (ie, no loss or gain of people globally), we applied an adjustment to the migration forecasts for each age, sex, year, and draw. We let $\hat{I}_{lastd}$ and $\hat{E}_{lastd}$ be the forecasted net immigrants and emigrants, respectively, for location l, age a, sex s, year t, and draw d. These were computed from the forecasted migration counts, denoted by $\hat{Y}_{lastd}$, as

$$\hat{I}_{lastd} = I(\hat{Y}_{lastd} > 0)\hat{Y}_{lastd}$$

$$\hat{E}_{lastd} = -I(\hat{Y}_{lastd} < 0)\hat{Y}_{lastd}$$

where $I(\cdot)$ is the indicator function. For each age, sex, draw, and year, we imposed the following constraint to balance migration across locations:

$$\frac{\sum_{l=1}^{195} \hat{I}_{lastd}}{k_{astd}} + k_{astd} \sum_{l=1}^{195} \hat{E}_{lastd} = 0$$

where k_{astd} is the age-, sex-, year-, and draw-specific square root of the global ratio of immigrants to emigrants defined by

$$k_{astd} = \sqrt{\frac{\sum_{l=1}^{195} \hat{I}_{lastd}}{\sum_{l=1}^{195} \hat{E}_{lastd}}}$$

To uphold the above constraint, we adjusted the forecasted migration counts according to:

$$\hat{Y}^*_{lastd} = \begin{cases} \hat{Y}_{lastd}(1/k_{astd}), & \hat{Y}_{lastd} > 0 \\ \hat{Y}_{lastd}(k_{astd}), & \hat{Y}_{lastd} < 0 \end{cases}$$

Section 8 Forecasting populations

We used the cohort-component method of projection (CCMP) to forecast population.[16] The inputs were estimates from GBD 2017 of past population and sex ratio at birth, and forecasts of age-specific fertility, all-cause mortality, and net migration rates.

Section 8.1 Cohort-component method of projection

This method calculates future populations, N_{t+1}, from current populations, N_t, using the population balancing equation,

$$N_{t+1} = L_t N_t + M_t$$

where L_t is the Leslie matrix and M_t is migration. The time step of the CCMP is usually chosen as five-year or one-year intervals, to match the granularity of ages in population data. In order to capture behaviour for the youngest ages, this forecast used a one-week time step, which required interpolation and disaggregation to one-week age intervals.

Section 8.2 Robust age-specific mortality input to CCMP

Age-specific mortality inputs were taken from forecasted age-specific all-cause mortality, m_x. The graduation method[16] was applied to estimate ${}_n a_x$ of those 5-year age groups with 5-year neighbours. Armed with ${}_n m_x$ and ${}_n a_x$, the rest of the life table columns could be readily computed using standard formulas. However, additional work was needed for the terminal age groups.

Section 8.3 Extending age-specific mortality to 100–105

The CCMP model requires knowledge of the survivorship of the terminal age group (95+), and calculating this survivorship of the terminal age group (95+) necessitates completing the l_x curve beyond 95 years of age. Therefore, we extrapolated age specific probabilities of dying ${}_5q_{95}$ and ${}_5q_{100}$, iteratively, from ${}_5q_{90}$:

$$\text{logit}({}_5q_{x+5}) - \text{logit}({}_5q_x) = \alpha_s + \beta_{s,x} + \gamma_s \,\text{logit}({}_5q_{90})$$

Where x is the start of the age group, s is sex. α_s, $\beta_{s,x}$, and γ_s are fitting parameters that depend on sex-only, age-and-sex, and sex-only. Note that the logit term on the right hand side is pinned at the starting age point of the extrapolation, which is 90 years in our case. This method has been previously described elsewhere.[17]

The fitting parameters are provided as follows:

Parameter	Value
α_{male}	0.958635
α_{female}	1.038949
$\beta_{\text{male,90}}$	0.020551
$\beta_{\text{female,90}}$	0.030907
$\beta_{\text{male,95}}$	–0.059321
$\beta_{\text{female,95}}$	–0.052221
γ_{male}	–0.146208
γ_{female}	–0.197924

The above method allowed us to extrapolate for ${}_5q_{95}$ and ${}_5q_{100}$, which in turn yielded l_{100} and l_{105}. We then declared l_{110} = 0 to complete the l_x curve.

Our extrapolated l_x values needed to conform to the universal relationship

$${}_{¥}m_{95} = \frac{l_{95}}{T_{95}}$$

where T_{95} is the integral under the l_{95} curve beyond 95. For self-consistency, we scaled the extrapolated ${}_nq_x$ values such that the back-calculated ${}_{¥}m_{95}$ equals the forecasted value. This was done with the help of Simpson's 3/8 rule:

$$T_{95} = \int_{95}^{\infty} l_x \; dx \approx \frac{15}{8}\left(l_{95} + 3\; l'_{100} + 3\; l'_{105} + 3\; l'_{110}\right) = T'_{95}$$

Where the apostrophe denotes the current tentative value, and l_x at 110 is set to zero.

The above formulas allowed us to compute directly

$$\alpha = \frac{T_{95}}{T'_{95}} = \frac{l_{95}/{}_{¥}m_{95}}{T'_{95}}$$

as a "mismatch factor".

Furthermore, we maintained the relationship between ${}_5q_{95}$ and ${}_5q_{100}$, that is,

$$\beta = \frac{{}_5q'_{95}}{{}_5q'_{100}} = \frac{{}_5q_{95}}{{}_5q_{100}} = const$$

Having both α and β computed, we proceeded with

$$\alpha \approx \frac{\frac{15}{8}(l_{95} + 3\, l_{100} + 3\, l_{105})}{\frac{15}{8}(l_{95} + 3\, l'_{100} + 3\, l'_{105})}$$

and substituted $l_{100}$and $l_{105}$with

$$l_{100} = l_{95}\,(1 - \beta\, {}_5q_{100})$$

$$l_{105} = l_{95}\,(1 - \beta\, {}_5q_{100})(1 - \, {}_5q_{100})$$

to finally arrive at the quadratic equation for ${}_5q_{100}$:

$$\beta\, {}_5q_{100}^2 \; - \; (2\,\beta + 1)\;\, {}_5q_{100} \; + \; \gamma \; = \; 0$$

where

$$\gamma \; = \; -\alpha \left(\frac{7}{3} - (2\,\beta + 1)\;\, {}_5q'_{100} + \beta\;\, {}_5{q'_{100}}^2\right)$$

with the solution

$${}_5q_{100} = \; \frac{(2\beta + 1) \pm \sqrt{(2\beta + 1)^2 \; - \; 4\,\beta\,\gamma}}{2\,\beta}$$

Since ${}_5q_{100}$< 1, the only viable solution was subtraction in the numerator of the above equation. Computing ${}_5q_{95}$ and tail of the l_x curve was then straightforward.

It should be noted that, due to approximation errors, the above equation will not always yield sensible solutions. In those instances, we reverted back to the original values.

For calculation of life expectancy at birth we employed the method used in Foreman *et al*[4] and described in section 2.5.3 of the supplemental appendix of Wang *et al.*[18]

Section 9 Life table and life expectancy

Life tables and life expectancy were calculated as in Foreman et al. (2018)[4] with 23 age groups (early neonatal, late neonatal, post neonatal, 1-4 years, 5-9, … to 95 years and older). Older ages were handled as described in supplementary appendix (section 3.2) in Wang et al (2016)[17]:

> "To extrapolate age-specific mortality beyond age 85, the Gompertz law of mortality and other functional model age pattern of mortality methods are generally used.[19,20] Here, we have developed a new model with better predictive validity than existing methods. Age-group dummies and probability of dying from age 80 to 84 in logarithmic scales are used to estimate the difference in age-specific probability of dying in logit scale between two consecutive age-groups, as described in the following equation:

$$\text{logit}\left({}_5q_x^{j,t,g}\right) - \text{logit}\left({}_5q_{x+5}^{j,t,g}\right) = \alpha^g + \beta_x^g \cdot \text{age} + \gamma^g \cdot \text{logit}\left({}_5q_{80}^{j,t,g}\right) + \eta_j^g + \xi_x^{j,t,g}$$

> Here, j refers to country, g refers to sex, and t refers to time. Parameters are estimated using data from selected countries in Human Mortality Database with high quality VR data in the oldest old age groups above age 80.[21] The parameters estimated from the above model are then used to generate age-specific probability of death from age 85 to 109."

Section 10 Alternative scenarios based on educational attainment and met need for contraception

Our forecasting reference scenario captures the future population trajectory if recent trends continue by assuming future trends will continue at the current rate of change. We also considered four alternative scenarios based on hypothetical future rates of change for contraceptive met need and educational attainment:

1. Slower (15th percentile rate of change)
2. Faster (85th percentile rate of change)
3. Fastest (99th percentile rate of change)
4. SDG pace (rate of change required to meet 2030 UN Sustainable Development Goal [SDG] targets)

Changes in contraceptive met need and educational attainment impact our population forecasts through their effects as covariates (directly or through SDI) in our fertility, mortality, and migration models.

The slower, faster, and fastest scenarios were derived from the 15th, 85th, and 99th percentile annualised rates of change of contraceptive met need and education in past location-years, respectively. The slower and faster scenarios are intended capture the future trajectories, given plausibly slower or faster (compared to the past) rates of change of contraceptive met need and education. The fastest scenario captures population trajectories under the highest achievable (as seen in the past) rate of change of contraceptive met need and education. Finally, the SDG pace scenario assumes the population trajectory if the rate of change needed to meet relevant 2030 SDG targets, and this rate of change is carried out from 2018 until 2100.

Section 10.1 Slower, faster, fastest met need and education scenarios

Percentile-based scenarios were implemented using the strategy of Foreman and colleagues, 2018[4] applied only to contraceptive met need and education (while all other independent drivers of health were forecasted using the reference scenario methodology).

Section 10.2 SDG pace contraceptive met need and education scenario

Our population scenario for change at the SDG pace adopted the rate of change needed to meet United Nations SDGs on educational attainment and met need for contraceptives in 2030 specified by targets 3.7 and 4.1, respectively.[22,23]

Section 10.2.1 SDG pace met need for contraception

Our SDG pace scenario for met need for contraceptives Target 3.7 states "by 2030, ensure universal access to sexual and reproductive health-care services, including for family planning, information and education, and the integration of reproductive health into national strategies and programmes." We incorporated this into the SDG pace scenario by requiring met need for contraceptives to be 100% in 2030, and computing the required location- and age-specific AROCs (in normal, not logit space) to reach it from the 2017 value as:

$$\delta_{la}^{[SDG-MN]} \frac{= 1 - MN_{la(2017)}}{2030 - 2017}$$

were $MN_{la(2017)}$ is the proportion of contraceptive met need for females in age group a and location l in 2017. The above AROC was applied to reach 100% contraceptive met need in 2030, then held at 100% after 2030, by applying the following AROCs at each time:

$$\Delta_{lat}^{[SDG-MN]} = \begin{cases} \delta_{la}^{[SDG-MN]}, & t = 2018, \ldots 2030 \\ 0, & t = 2031, \ldots 2100 \end{cases}$$

Section 10.2.2 SDG pace education

We based the SDG pace scenario for education on Target 4.1, which specifies "by 2030, ensure that all girls and boys complete free, equitable and quality primary and secondary education leading to relevant and effective learning outcomes." Due to the lack of comprehensive global data in GBD on the cost, equitability, quality, and outcomes of education, we only considered completion of primary and secondary education prior to reaching adulthood as defined by 100% of the population completing at least 12 years of education by age group 20-24.

Since both GBD 2017 and our framework measure educational attainment by the mean years of education for a given location, age, sex, and time, we utilised forecasts of single-year education distributions in 2030 for age 20-24 years, each sex, and each location[24] to imply the smallest mean years of education required to meet the SDG Target 4.1 without changing the trajectory of those that would already attain 12 or more years. We let $\hat{p}_{ls}(y)$ denote the forecasted proportion of individuals in country or territory l of sex s that have y years of education in 2030 at age 20-24 years. The most conservative adjustment to this distribution to meet SDG Target 4.1 sets all individuals with <12 years of education to instead have 12 years, accomplished by the following single-year education distribution adjustment:

$$\hat{p}_{ls}^{[SDG]}(y) = \begin{cases} 0, & y = 0, 1, \ldots, 11 \\ \sum_{i=0}^{12} \hat{p}_{ls}(i)\ , & y = 12 \\ \hat{p}_{ls}(y)\ , & y = 13, 14, \ldots, 18 \end{cases}$$

The mean years of education of this adjusted distribution, $EDU_{ls}^{[SDG]}$, represents the minimum mean required to meet SDG 4.1 in 2030 given the forecasted distribution, and is computed by

$$EDU_{ls}^{[SDG]} = \sum_{i=12}^{18} i \times \hat{p}_{ls}^{[SDG]}(i)$$

For each location and sex, we set the SDG pace by deriving the AROC required to reach $EDU_{ls}^{[SDG]}$ in 2030 for 20-24 year olds starting from the corresponding value in 2017. We assumed a constant rate of change over the 23-year timespan in logit space (after scaling by 18 years to represent proportion out of maximum educational attainment) and computed the SDG AROC by

$$\delta_{ls}^{[SDG-EDU]} = \frac{\text{logit}\left(EDU_{ls}^{[SDG]}/18\right) - \text{logit}\left(EDU_{l(20-24)s(2017)}/18\right)}{2030 - 2017}$$

where $EDU_{l(20-24)s(2017)}$ is the mean years of education for 20-24 year olds of sex s at location l in 2017.

Some locations may have high AROCs under the reference scenario for a particular sex and age group. To avoid having the SDG pace scenario slow down progress relative to the reference scenario, the location-, age-, and sex-specific AROCs for the SDG pace were taken to be

$$\Delta_{las}^{[SDG-EDU]} = \max\left(\delta_{ls}^{[SDG-EDU]}, \Delta_{las}^{[REF-EDU]}\right)$$

where $\Delta_{las}^{[REF-EDU]}$ is the education AROC under the reference scenario for location l, age a, and sex s (obtained using methods in Foreman et al, 2018[4]).

Section 11 Uncertainty interval estimation

Uncertainty intervals (UIs) were estimated using the 0.025 and 0.975 quantiles of the draw distributions for the measure of interest. When total fertility rate and population forecasts were aggregated over geographical locations (eg, global total fertility rate and population), we performed an ad-hoc adjustment to account for unmodelled spatial correlation. Due to the large number of interdependent models in the forecasting framework, directly accounting for spatial correlation in our forecast draws was infeasible. Nevertheless, producing valid uncertainty estimates for location-aggregated quantities requires accounting for spatial correlation. To this end, we applied an ad-hoc adjustment to our UIs for quantities aggregated over locations that has been utilised previously in similar situations.[25] We generated two sets of draw distributions: one assuming spatial independence, and another generated under maximal rank dependence of the draws induced by an ordering strategy. For each aggregated quantity (TFR and population), we ordered the age-, sex- and location-specific draws at each year according to their rank in 2100. This induced increasing rank correlation between the draws across locations over time. Our final estimates of all UIs for aggregate quantities were computed by averaging the 0.025 and 0.975 quantiles of these two draw distributions.

Section 12 Model evaluation

The overall performance of our fertility forecasting framework was evaluated by out-of-time predictive performance and is described in the supplementary results appendix 2.

Section 13 References

1 Stevens GA, Alkema L, Black RE, *et al.* Guidelines for Accurate and Transparent Health Estimates Reporting: the GATHER statement. *The Lancet* 2016; **388**: e19–23.

2 Dicker D, Nguyen G, Abate D, *et al.* Global, regional, and national age-sex-specific mortality and life expectancy, 1950–2017: a systematic analysis for the Global Burden of Disease Study 2017. *The Lancet* 2018; **392**: 1684–735.

3 Murray CJL, Callender CSKH, Kulikoff XR, *et al.* Population and fertility by age and sex for 195 countries and territories, 1950–2017: a systematic analysis for the Global Burden of Disease Study 2017. *The Lancet* 2018; **392**: 1995–2051.

4 Foreman KJ, Marquez N, Dolgert A, *et al.* Forecasting life expectancy, years of life lost, and all-cause and cause-specific mortality for 250 causes of death: reference and alternative scenarios for 2016–40 for 195 countries and territories. *The Lancet* 2018; **392**: 2052–90.

5 Stanaway JD, Afshin A, Gakidou E, *et al.* Global, regional, and national comparative risk assessment of 84 behavioural, environmental and occupational, and metabolic risks or clusters of risks for 195 countries and territories, 1990–2017: a systematic analysis for the Global Burden of Disease Study 2017. *The Lancet* 2018; **392**: 1923–94.

6 United Nations Population Division. World Population Prospects 2019 - migration indicators. 2019. https://population.un.org/wpp/Download/Standard/Migration/ (accessed June 17, 2019).

7 Forouzanfar MH, Afshin A, Alexander LT, *et al.* Global, regional, and national comparative risk assessment of 79 behavioural, environmental and occupational, and metabolic risks or clusters of risks, 1990–2015: a systematic analysis for the Global Burden of Disease Study 2015. *The Lancet* 2016; **388**: 1659–724.

8 James SL, Gubbins P, Murray CJ, Gakidou E. Developing a comprehensive time series of GDP per capita for 210 countries from 1950 to 2015. *Popul Health Metr* 2012; **10**: 12.

9 Micah AE, Dieleman JL, Kahn Case M, *et al.* Financing global health 2018: countries and programs in transition. Institute for Health Metrics and Evaluation, 2019.

10 Girosi F, King G. Demographic forecasting. New Jersey: Princeton University Press, 2008.

11 Kristensen K, Nielsen A, Berg CW, Skaug H, Bell BM. TMB: automatic differentiation and laplace approximation. *J Stat Softw* 2016; **70**: 1–21.

12 Chang AY, Cowling K, Micah AE, *et al.* Past, present, and future of global health financing: a review of development assistance, government, out-of-pocket, and other private spending on health for 195 countries, 1995–2050. *The Lancet* 2019; **393**: 2233–60.

13 United Nations Population Division. World Population Prospects 2019 - population indicators. 2019. https://population.un.org/wpp/Download/Standard/Migration/ (accessed May 29, 2019).

14 Eurostat. Emigration by age and sex. 2019; published online Feb 25. http://appsso.eurostat.ec.europa.eu/nui/show.do?dataset=migr_emi2&lang=en (accessed May 10, 2019).

15 Eurostat. Immigration by age and sex. 2019; published online April 3. http://appsso.eurostat.ec.europa.eu/nui/show.do?dataset=migr_imm8&lang=en (accessed May 10, 2019).

16 Preston S, Heuveline P, Guillot M. Demography: measuring and modeling population processes, 1 edition. Wiley-Blackwell, 2000.

17 Wang H, Naghavi M, Allen C, *et al.* Global, regional, and national life expectancy, all-cause mortality, and cause-specific mortality for 249 causes of death, 1980–2015: a systematic analysis for the Global Burden of Disease Study 2015. *The Lancet* 2016; **388**: 1459–544.

18 Wang H, Abajobir AA, Abate KH, *et al.* Global, regional, and national under-5 mortality, adult mortality, age-specific mortality, and life expectancy, 1970–2016: a systematic analysis for the Global Burden of Disease Study 2016. *The Lancet* 2017; **390**: 1084–150.

19 Gompertz B. On one uniform law of mortality from birth to extreme old age, and on the law of sickness. *J Inst Actuar* 1871; **16**: 329–44.

20 Thatcher AR. The long-term pattern of adult mortality and the highest attained age. *J R Stat Soc Ser A Stat Soc* 1999; **162**: 5–43.

21 Human Mortality Database. The human mortality database. Berkeley, USA: University of California; Rostock, Germany: Max Planck Institute for Demographic Research. https://www.mortality.org/ (accessed April 11, 2011).

22 United Nations. Quality education. https://www.un.org/sustainabledevelopment/education/ (accessed June 12, 2019).

23 United Nations. Good health and well-being. https://www.un.org/sustainabledevelopment/health/ (accessed June 12, 2019).

24 Friedman J, York H, Graetz N, *et al.* Exploring and forecasting levels and inequality of education globally. *The Lancet* in review.

25 Wilmoth JR, Mizoguchi N, Oestergaard MZ, *et al.* A new method for deriving global estimates of maternal mortality. *Stat Polit Policy* 2012; **3**. https://www.ncbi.nlm.nih.gov/pmc/articles/PMC3886639/ (accessed June 7, 2019).
