# Supplementary material for: Fertility, mortality, migration, and population scenarios for 195 countries and territories from 2017 to 2100: a forecasting analysis for the Global Burden of Disease Study
Source: Lancet. 2020 Oct 17;396(10258):1285–306. doi: 10.1016/S0140-6736(20)30677-2 (PMC7561721; doi:10.1016/S0140-6736(20)30677-2)
Supplement: Supplementary appendix 2 [file mmc2.pdf]

THE LANCET

Supplementary appendix

This appendix formed part of the original submission and has been peer reviewed. We post it as supplied by the authors.

Supplement to: Vollset SE, Goren E, Yuan C-W, et al. Fertility, mortality, migration, and population scenarios for 195 countries and territories from 2017 to 2100: a forecasting analysis for the Global Burden of Disease Study. *Lancet* 2020; published online July 14. http://dx.doi.org/10.1016/S0140-6736(20)30677-2.

Appendix 2: Supplementary results to “Fertility, mortality, migration, and population scenarios for 195 countries and territories from 2017 to 2100: a forecasting analysis for the Global Burden of Disease Study”

Preamble

This appendix provides supplemental figures and more detailed results for "Fertility, mortality, migration, and population scenarios for 195 countries and territories from 2017 to 2100: a forecasting analysis for the Global Burden of Disease Study.” This study complies with the Guidelines for Accurate and Transparent Health Estimates Reporting (GATHER) recommendations (appendix 1, section 2).[1]

Table of Contents

Section 1 List of tables and figures 2
Section 1.1 Figures 2
Section 1.2 Tables 2
Section 2 Population through 2100 3
Section 2.1 Time Series: Global and GBD super-regions 3
Section 2.2 Population age structure in 2017 and 2100 – Global and GBD super-regions 8
Section 2.3 Ratio of births to 80-year-olds 12
Section 3 Life expectancy through 2100 13
Section 3.1 Time Series: Global and GBD super-regions 13
Section 4 Migration through 2100 32
Section 5 Total fertility rate through 2100 40
Section 6 Completed cohort fertility versus total fertility rate 62
Section 7 Predictive validity of fertility model 62
Section 8 UNPD below replacement fertility forecast convergence 66
Section 9 Comparison of population, fertility, and life expectancy forecasts 67
Section 10 Evaluation of long range UN population projections: World Population Prospects 1982 projections for 2015 comparison with World Population Prospects 2017 estimates 71
Section 10.1 Background 71
Section 10.2 Data 71
Section 10.3 Methods 72
Section 10.4 Results 73
Section 10.4.1 Population 73
Section 10.4.2 Fertility 75
Section 10.5 Conclusion 78
Section 11 Detailed results files in GHDx 79
Section 12 References 79
Section 13 Location-year life tables 80

Section 1 List of tables and figures

Section 1.1 Figures

Figure 1. Global and super-region population 1990–2100 in the reference, slower, faster, fastest, and SDG pace scenarios

Figure 2. Global and super-region population age structure in 2017 and in 2100 in the reference, slower, faster, fastest, and SDG scenario

Figure 3. Forecasting the global number of births (green), the number of new 80-year-old adults (red), and the ratio of births to 80-year-olds through 2100 (blue)

Figure 4. Global and super-region life expectancy 1990–2100 in the reference, slower, faster, fastest, and SDG pace scenarios

Figure 5. Global and super-region total fertility rate from 1990 to 2100

Figure 6. Total fertility rate from 1950 to 2100 for ten sample countries in the reference (with uncertainty), slower, faster, fastest, and SDG pace scenarios

Figure 7. Total fertility rate (TFR) and completed cohort fertility (CCF) through 2017 for five sample countries

Figure 8. Forecasts of total fertility, global and by country, in the UNPD medium variant scenario, Wittgenstein SSP2 scenario, and IHME reference scenario, through 2100

Figure 9. Comparison of the total fertility rate (TFR) in 2100 from the IHME reference forecast, and the United Nations Population Division (UNPD) medium variant scenario, by location

Figure 10. Population, total fertility rate, and life expectancy through 2100 in the IHME reference scenario, UNPHD medium variant scenario, and Wittgenstein SSP2 scenario

Figure 11. Accuracy of country-specific population forecasts for year 2015 as projected by UNPD 1982

Figure 12. Accuracy of country-specific population forecasts for year 2015 as projected in 1982, assessed against UNPD 2017 by difference and ratio

Figure 13. Accuracy of country-specific TFR forecasts for year 2015 as projected by UNPD 1982

Figure 14. Accuracy of country-specific TFR forecasts for year 2015 as projected in 1982, assessed against UNPD 2017 by difference and ratio

Section 1.2 Tables

Table 1. Life expectancy by sex in the reference forecast.

Table 2. Net migration counts for all ages and both sexes combined: 2018, 2050, and 2100.

Table 3. Total fertility rate in the reference forecast and faster met need and education pace scenario: 1990, 2017, 2050, and 2100.

Table 4. Out-of-time forecast errors in TFR for holdout years 2008-2017.

Table 5. Omega weights selected and the computed root mean squared error for forecasting risk factors, education, met need for contraception, and vehicles per capita.

Table 6a. UNPD mean TFR levels in UNPD low fertility countries and countries that were below replacement in 2015-2020.

Table 6b. UNPD 2019 re-estimation with the modified Phase III entrance criteria

Table 7. Population and total fertility rate (TFR) in 2100 in the IHME reference scenario, UNPD medium variant scenario, and Wittgenstein SSP2 scenario.

Table 8. Countries having broken apart since 1982, for which adjustment was made in analysis.

Table 9. Countries having been formed since 1982 for which adjustments were made in analysis.

Table 10a. Mean percentage error (MPE) of UNPD 1982 population forecast compared to UNPD 2017 population estimates by projection length.

Table 10b. Mean absolute percentage error (MAPE) of UNPD 1982 population forecast compared to UNPD 2017 population estimates by projection length.

Table 11. Correlation between forecasted and estimated country-level populations in the year 2015 (UNPD 1982 vs UNPD 2017).

Table 12a. Mean percentage error (MPE) of UNPD 1982 TFR forecast compared to UNPD 2017 TFR estimates by projection length.

Table 12b. Mean absolute percentage error (MAPE) of UNPD 1982 TFR forecast as compared with UNPD 2017 TFR estimates by projection length.

Table 13. Number of countries with absolute percentage error greater than 20% for projection of 2015 population or TFR (UNPD 1982 vs UNPD 2017).

Table 14. Correlation between forecasted and estimated country-level TFR in the year 2015 (UNPD 1982 vs UNPD 2017).

Table 15. Location-year life tables, by age and sex for 222 locations in 2017 and 2100. mx=mortality rate, ax=mean person-years lived in an age interval among those who die in that age interval, lx=number of persons left alive at age x, nLx=person-years lived between age x and x+n, ex=life expectancy at age x.

Section 2 Population through 2100

Section 2.1 Time Series: Global and GBD super-regions

Figure 1. Global and super-region population 1990–2100 in the reference, slower, faster, fastest, and SDG pace scenarios. Past estimates are from GBD 2017, and values are in billions. SDG=Sustainable Development Goals. GBD=Global Burden of Disease.

A. Global population from 1990 to 2100, for both sexes combined, all ages

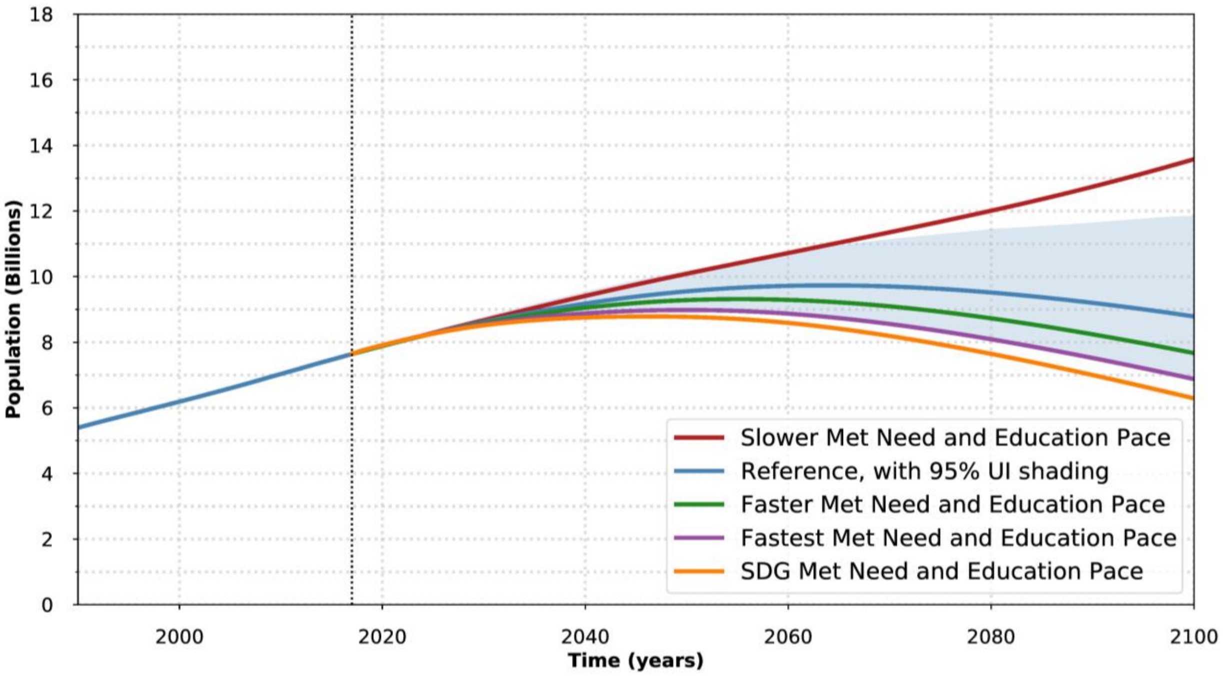

B. Southeast Asia, east Asia, and Oceania population from 1990 to 2100, for both sexes combined, all ages

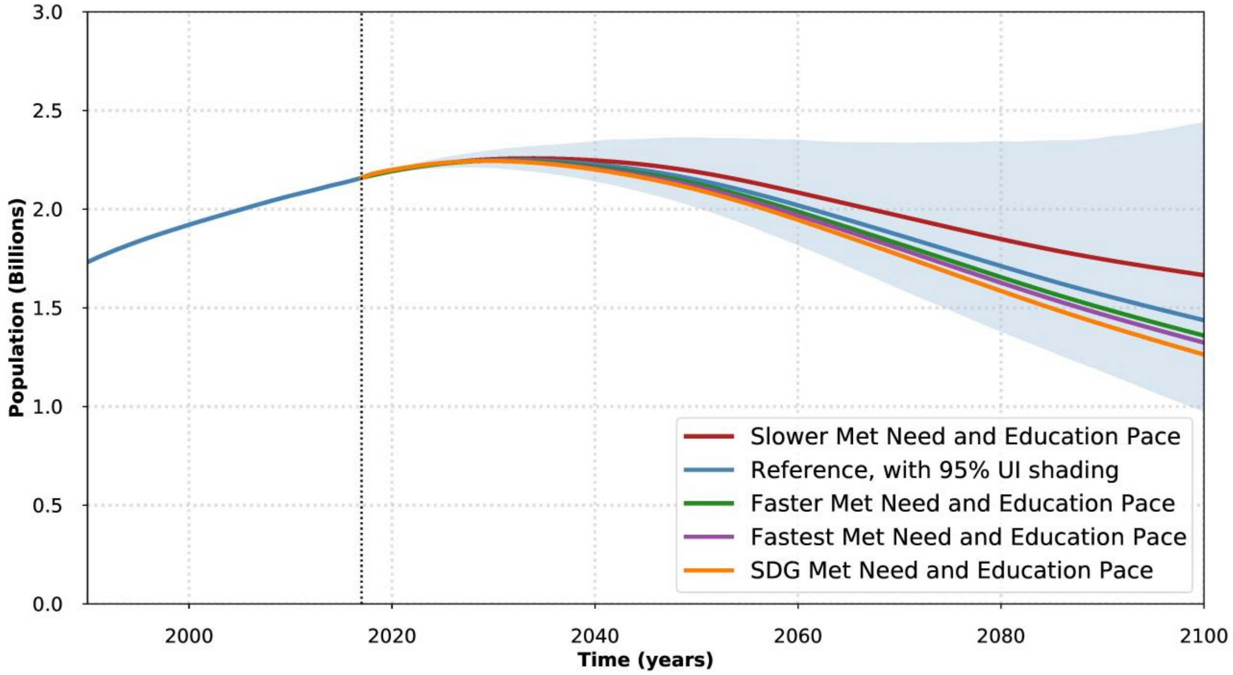

C. Central Europe, eastern Europe, and central Asia population from 1990 to 2100, for both sexes combined, all ages

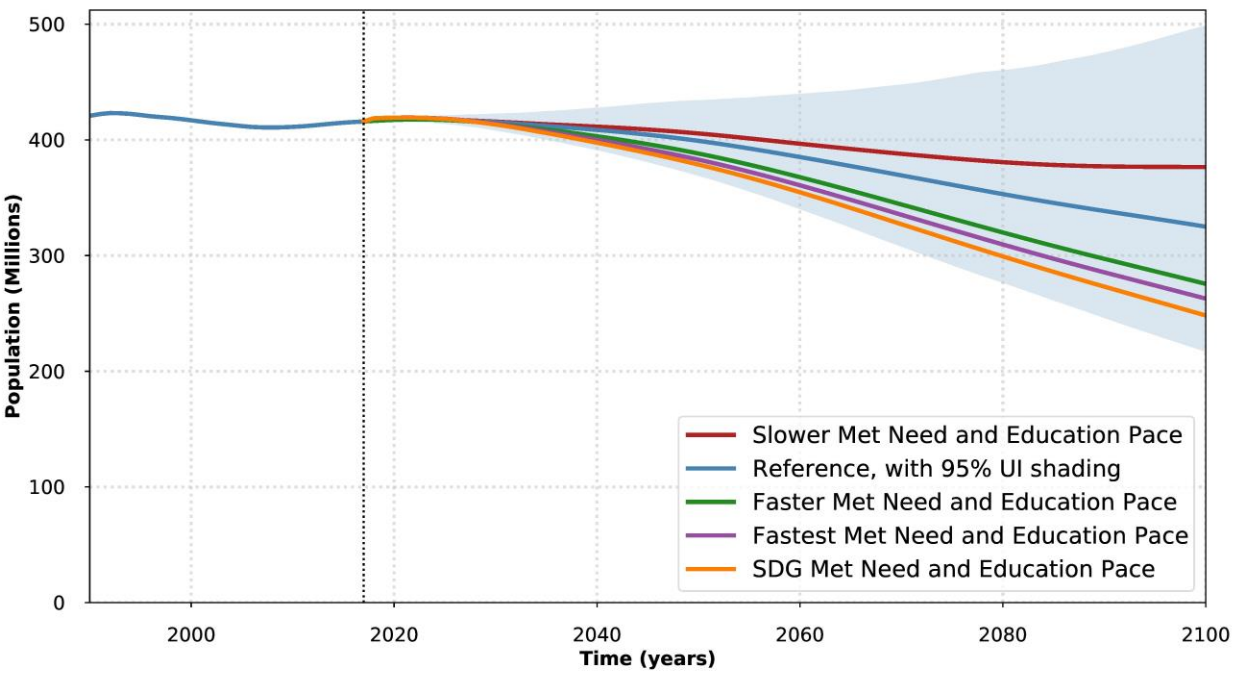

D. High-income population from 1990 to 2100, for both sexes combined, all ages

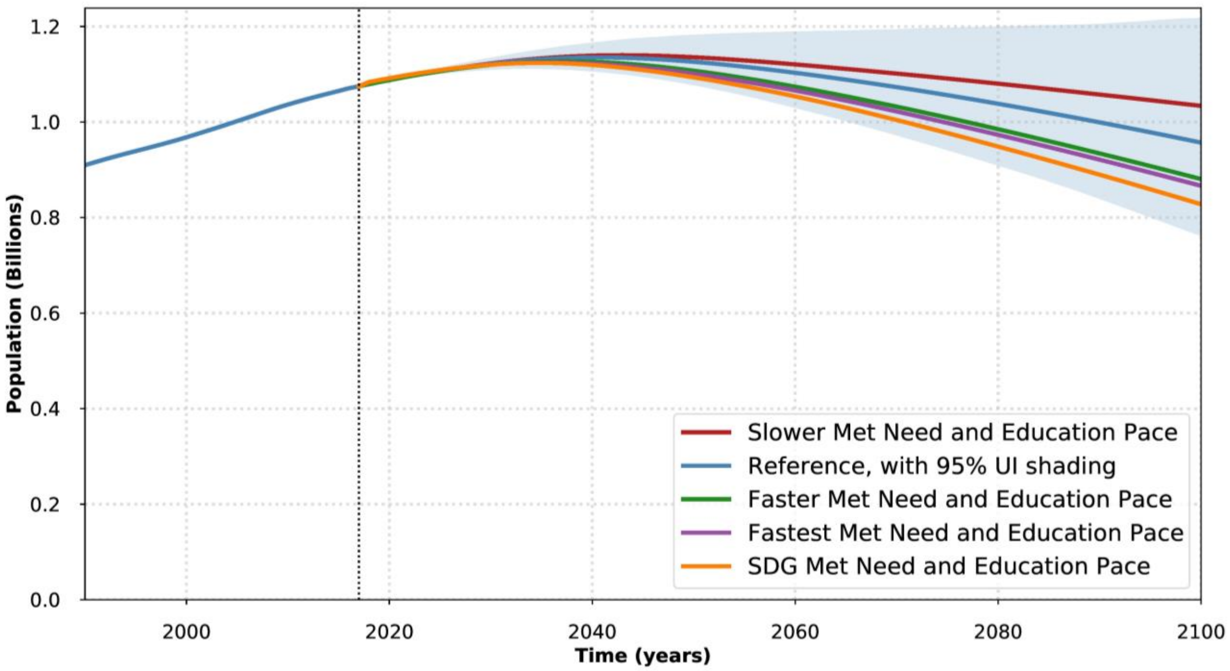

E. Latin American and Caribbean population from 1990 to 2100, for both sexes combined, all ages

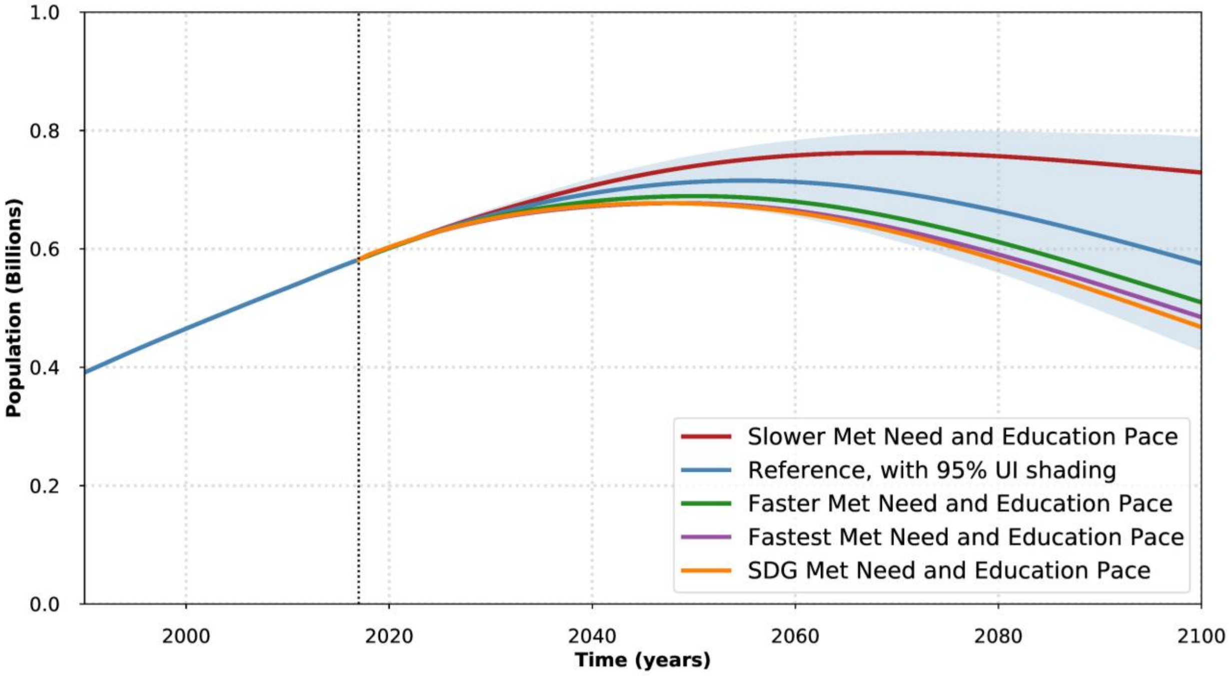

F. South Asia population from 1990 to 2100, for both sexes combined, all ages

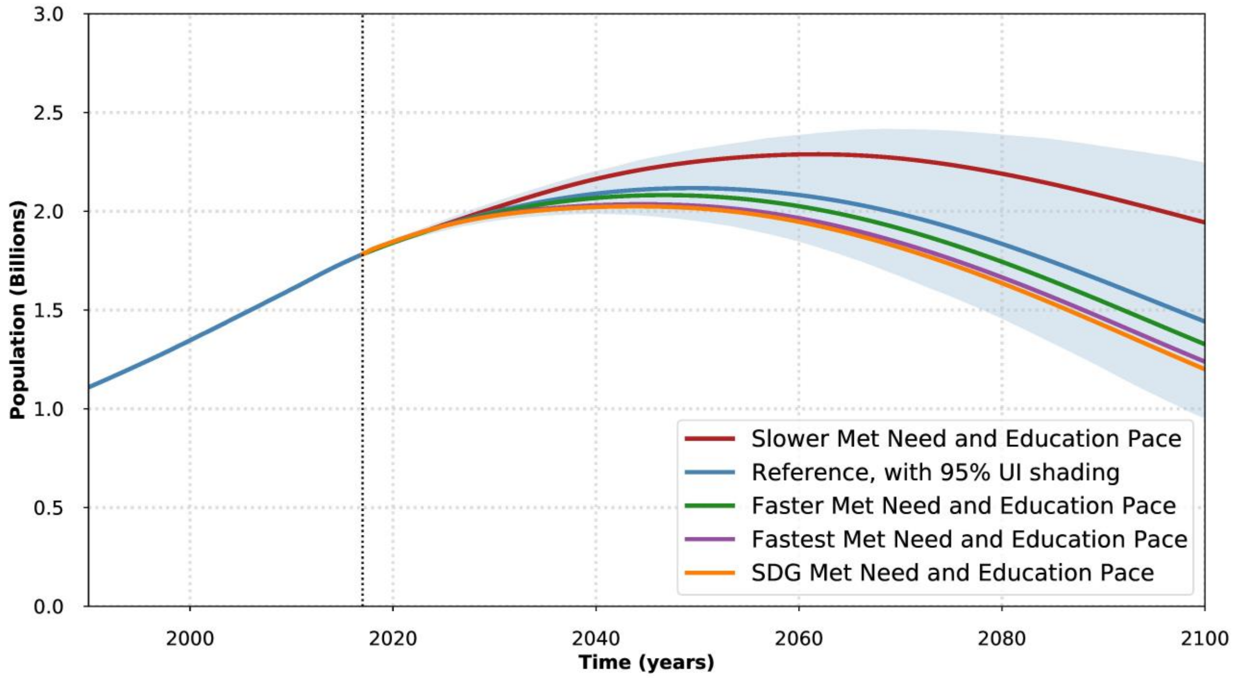

G. North Africa and Middle East population from 1990 to 2100, for both sexes combined, all ages

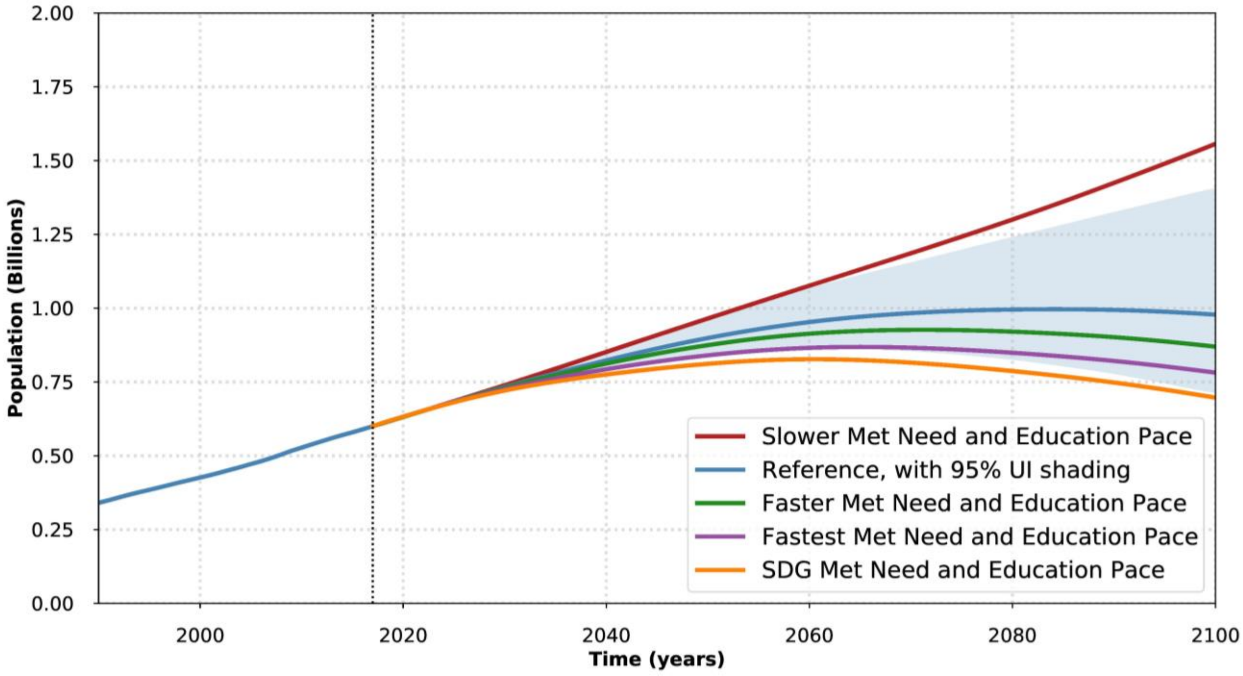

H. Sub-Saharan Africa population from 1990 to 2100, for both sexes combined, all ages

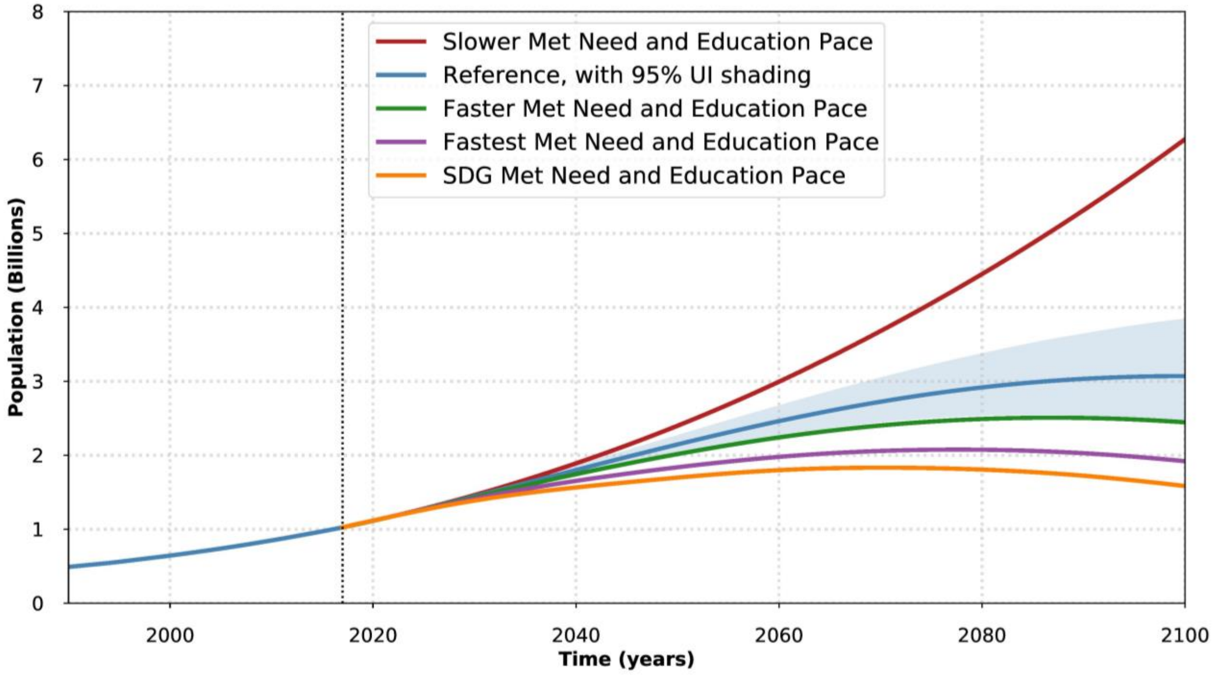

Section 2.2 Population age structure in 2017 and 2100 – Global and GBD super-regions

Figure 2. Global and super-region population age structure in 2017 and in 2100 in the reference, slower, faster, fastest, and SDG scenario. Estimates for 2017 are from GBD 2017. Triangles indicate the mean age, and populations are in millions. SDG=Sustainable Development Goals. GBD=Global Burden of Disease.

A. Global

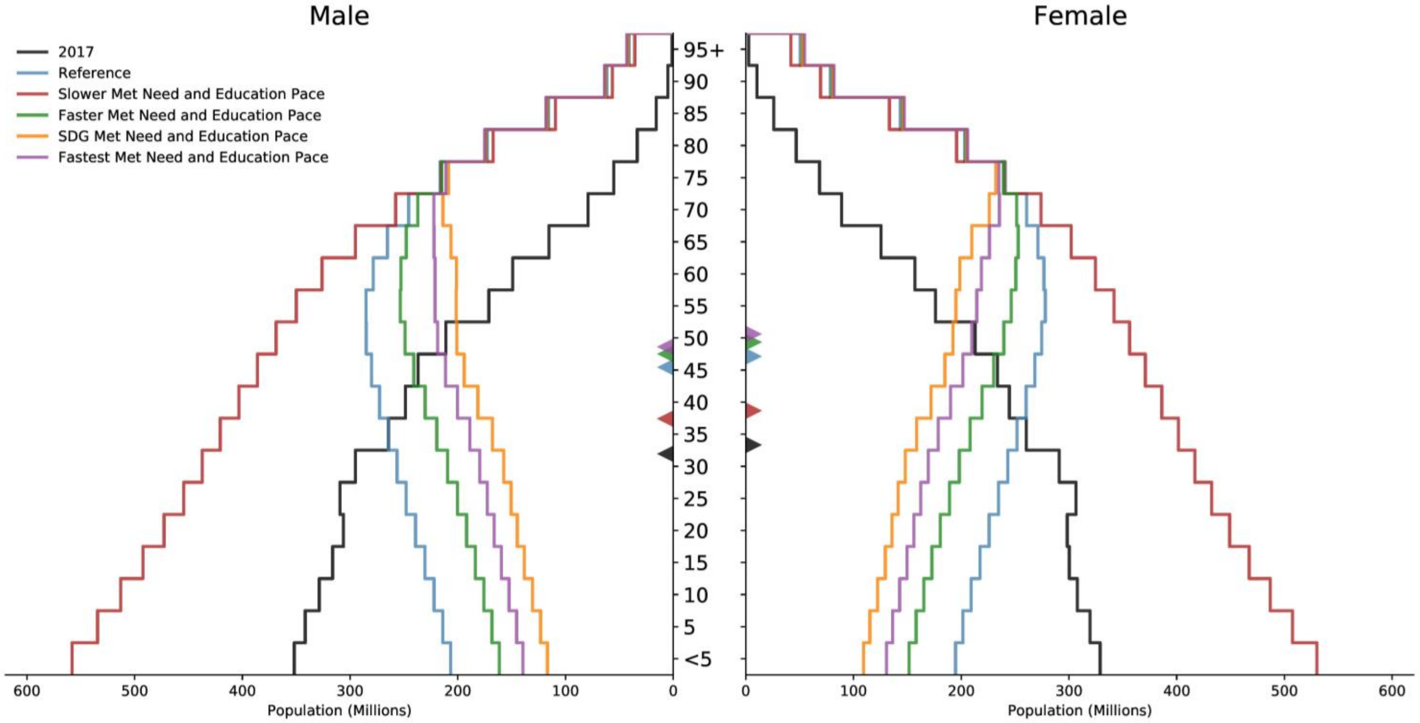

B. Southeast Asia, east Asia, and Oceania

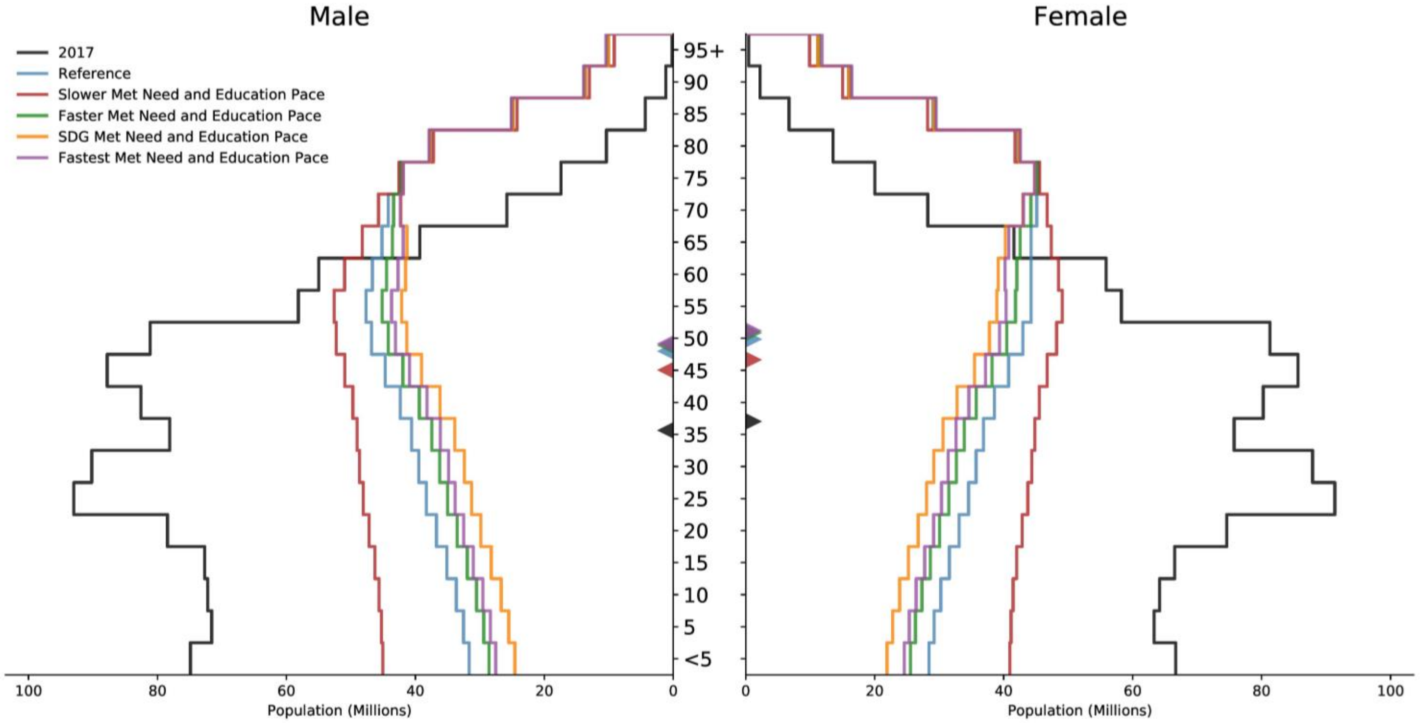

C. Central Europe, eastern Europe, and central Asia

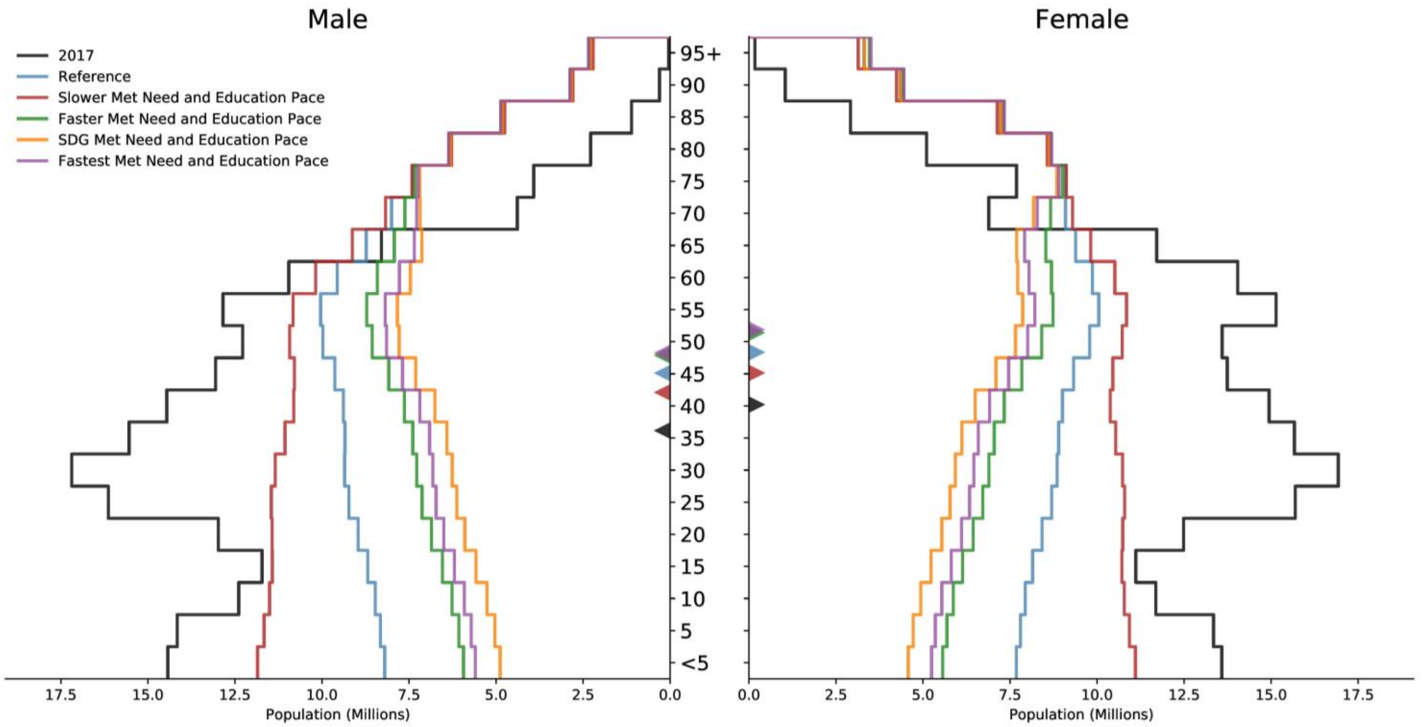

D. High-income

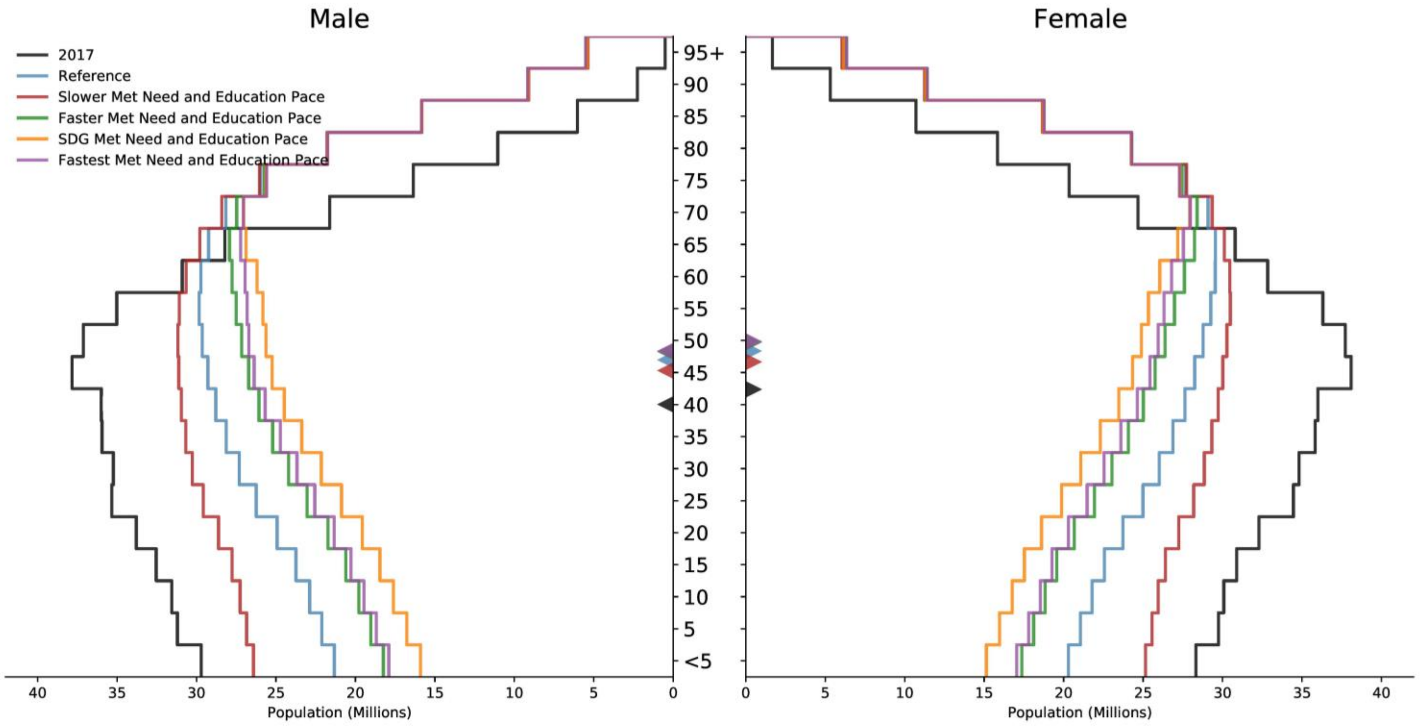

E. Latin America and Caribbean

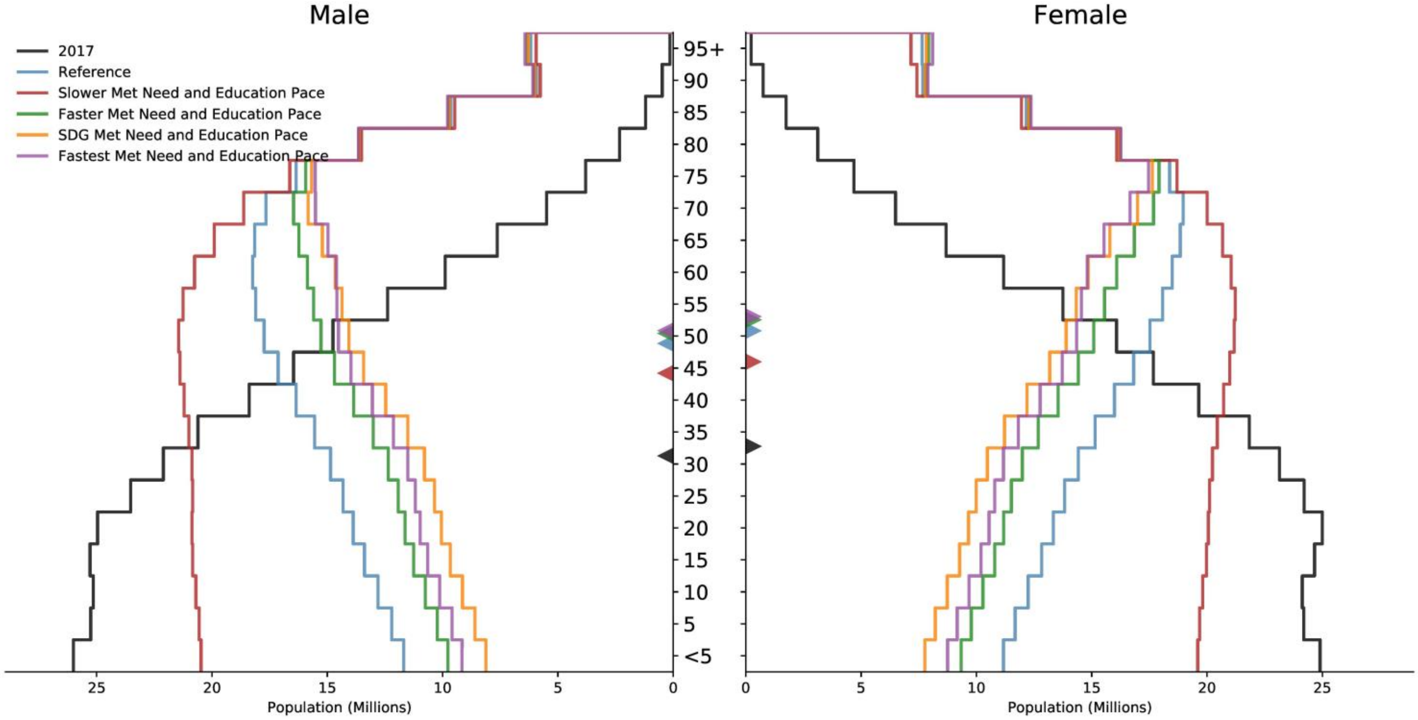

F. South Asia

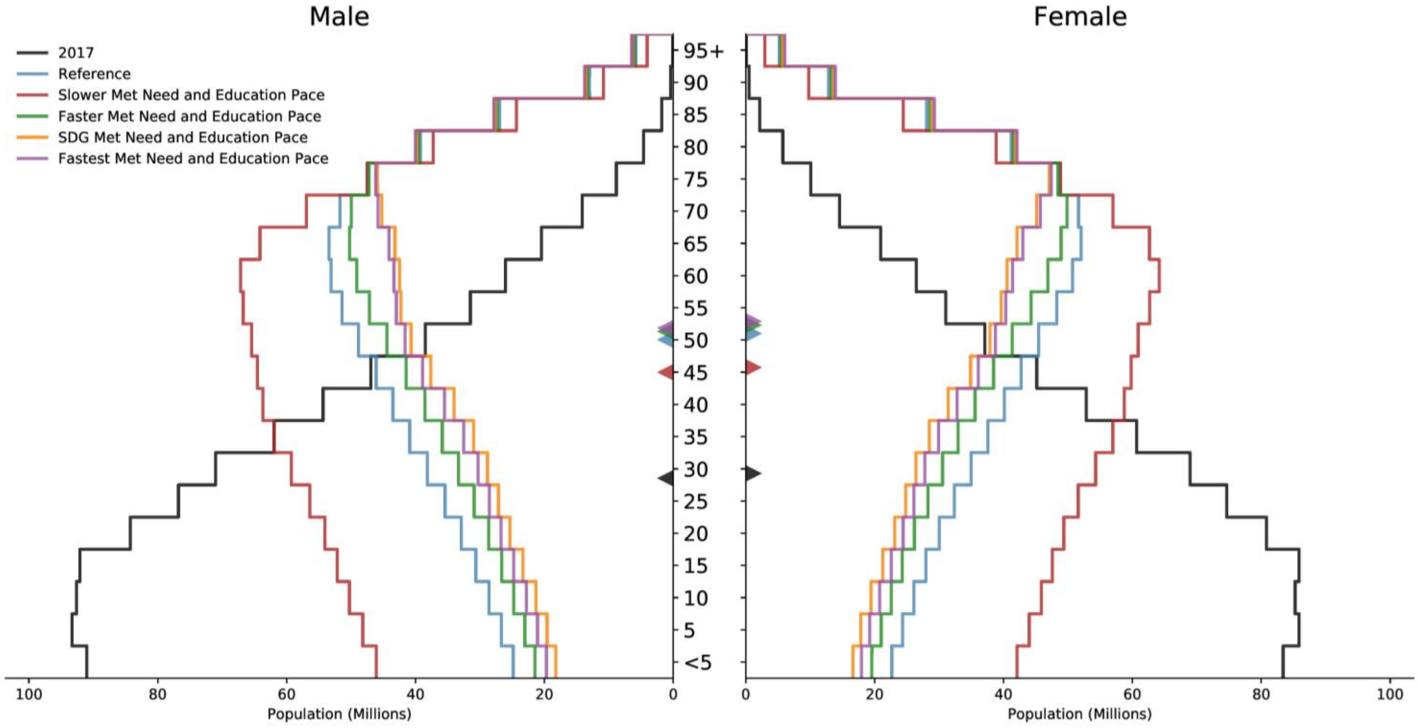

G. North Africa and Middle East

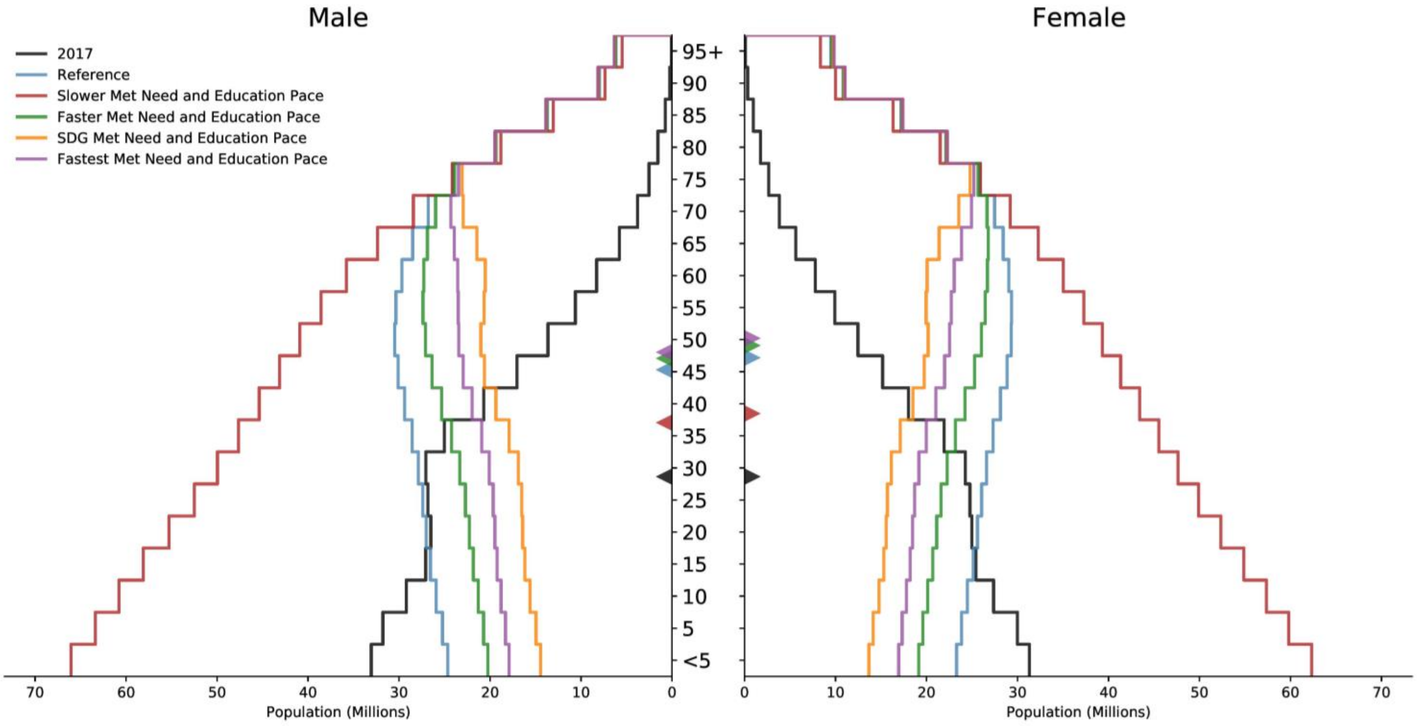

H. Sub-Saharan Africa

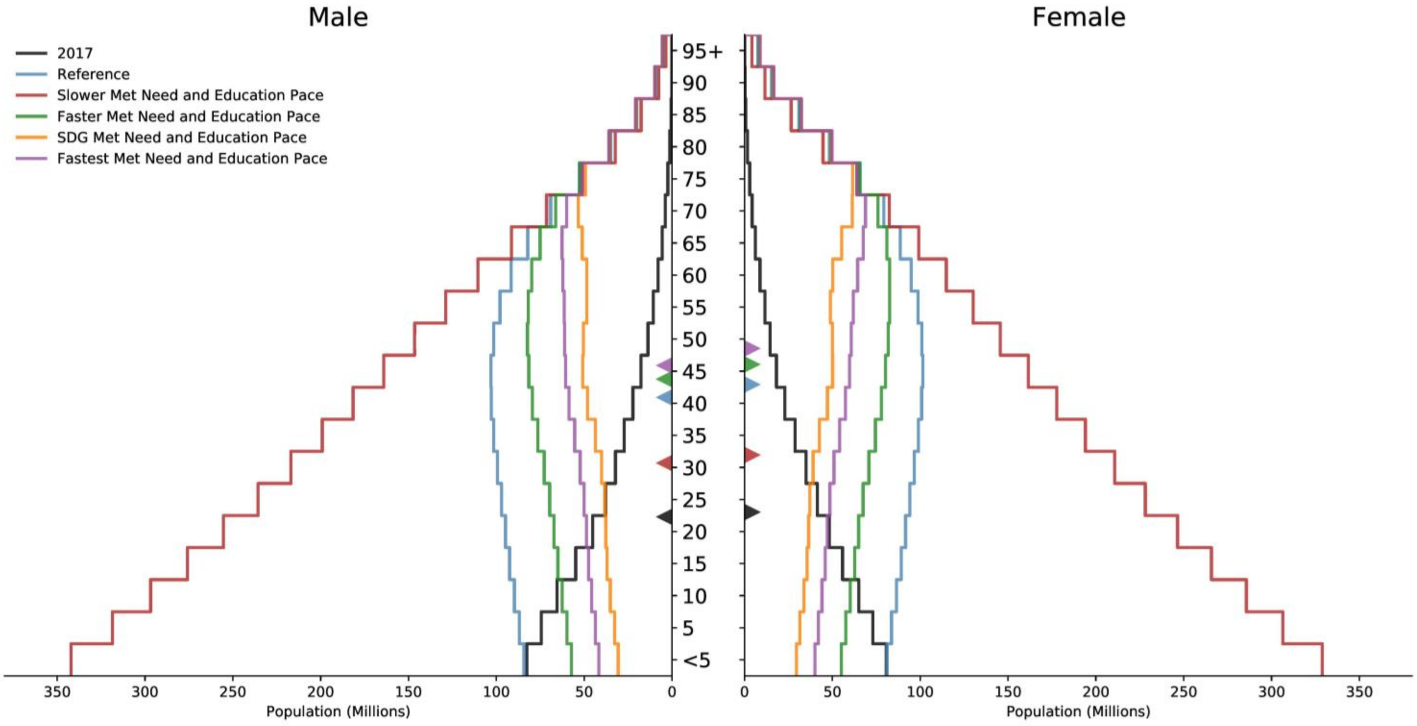

Section 2.3 Ratio of births to 80-year-olds

Figure 3. Forecasting the global number of births (green), the number of new 80-year-old adults (red), and the ratio of births to 80-year-olds through 2100 (blue). Estimates through 2017 are from the Global Burden of Disease Study 2017 and estimates from 2018 to 2100 are from the reference forecast.

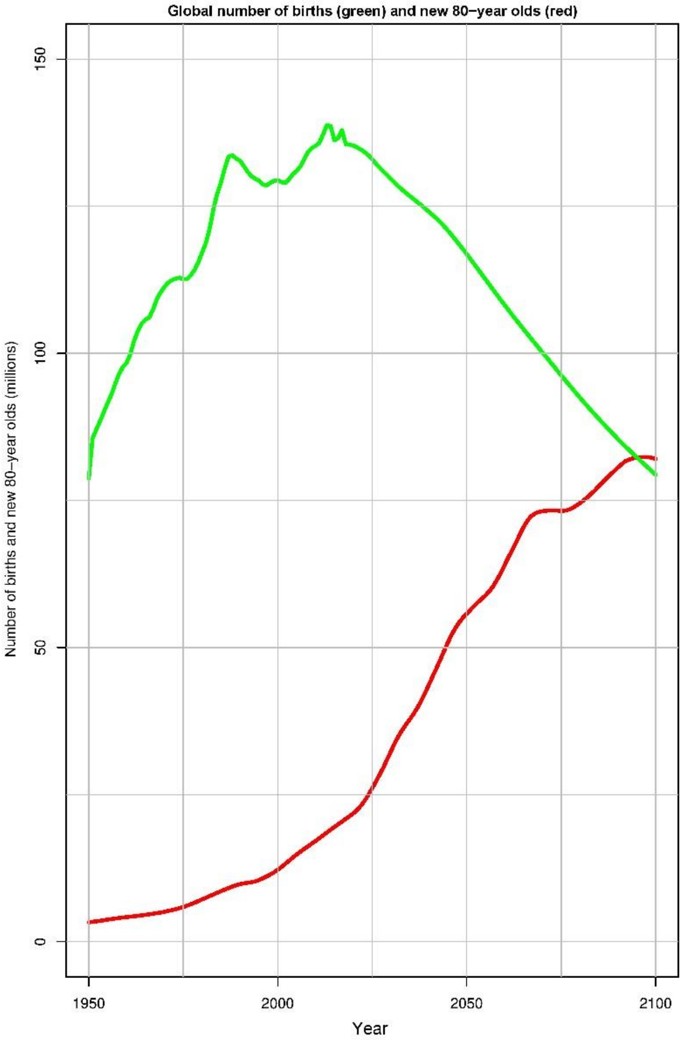

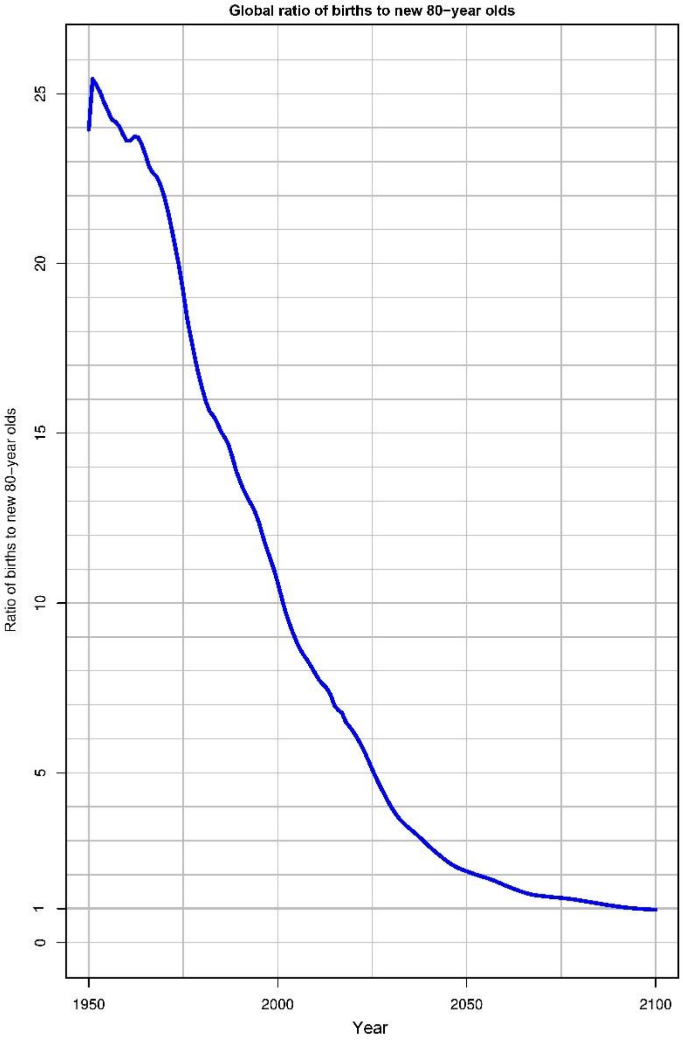

Section 3 Life expectancy through 2100

Section 3.1 Time Series: Global and GBD super-regions

Figure 4. Global and super-region life expectancy 1990 - 2100 in the reference, slower, faster, fastest, and SDG pace scenarios, by sex. Life expectancy is computed at birth, and values are reported in years. Past estimates are from GBD 2017. SDG=Sustainable Development Goals. GBD=Global Burden of Disease.

A. Global

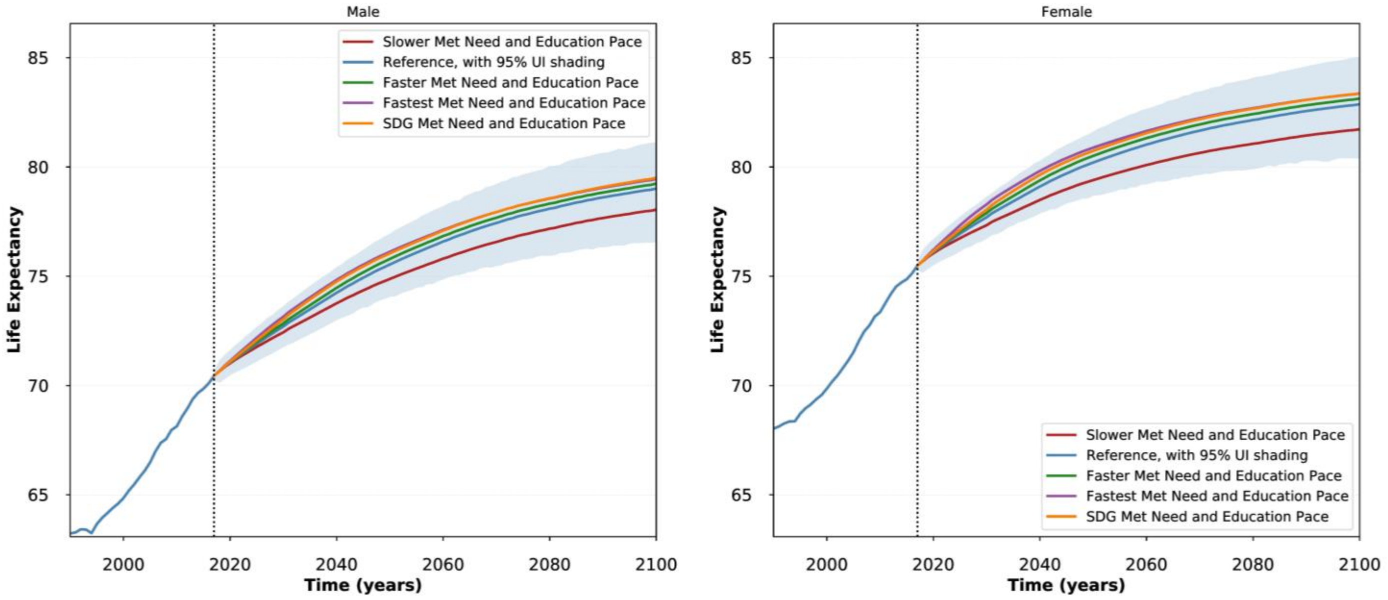

B. Southeast Asia, east Asia, and Oceania

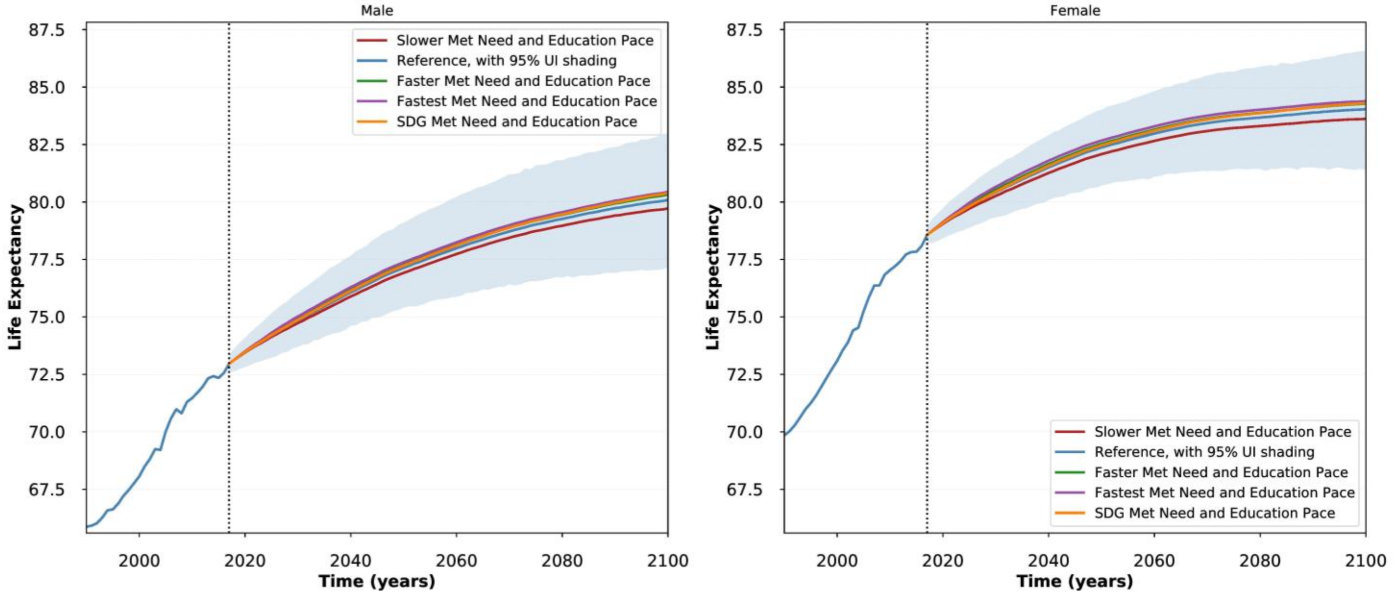

C. Central Europe, eastern Europe, and central Asia

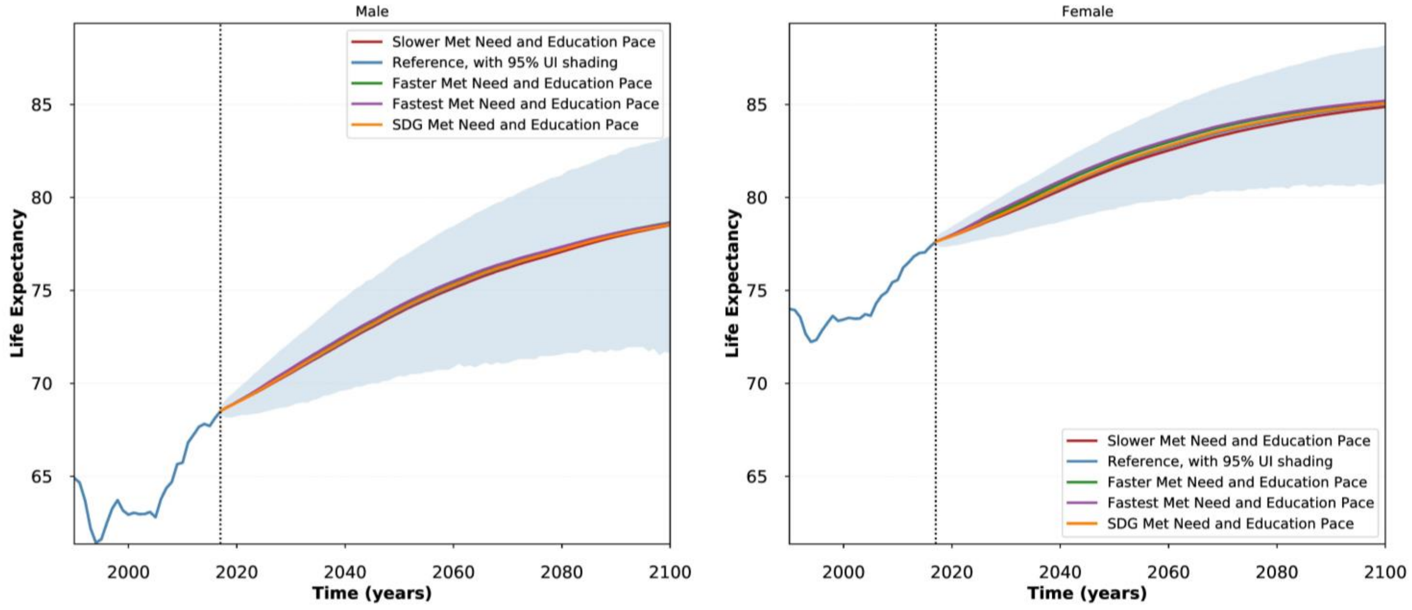

D. High-income

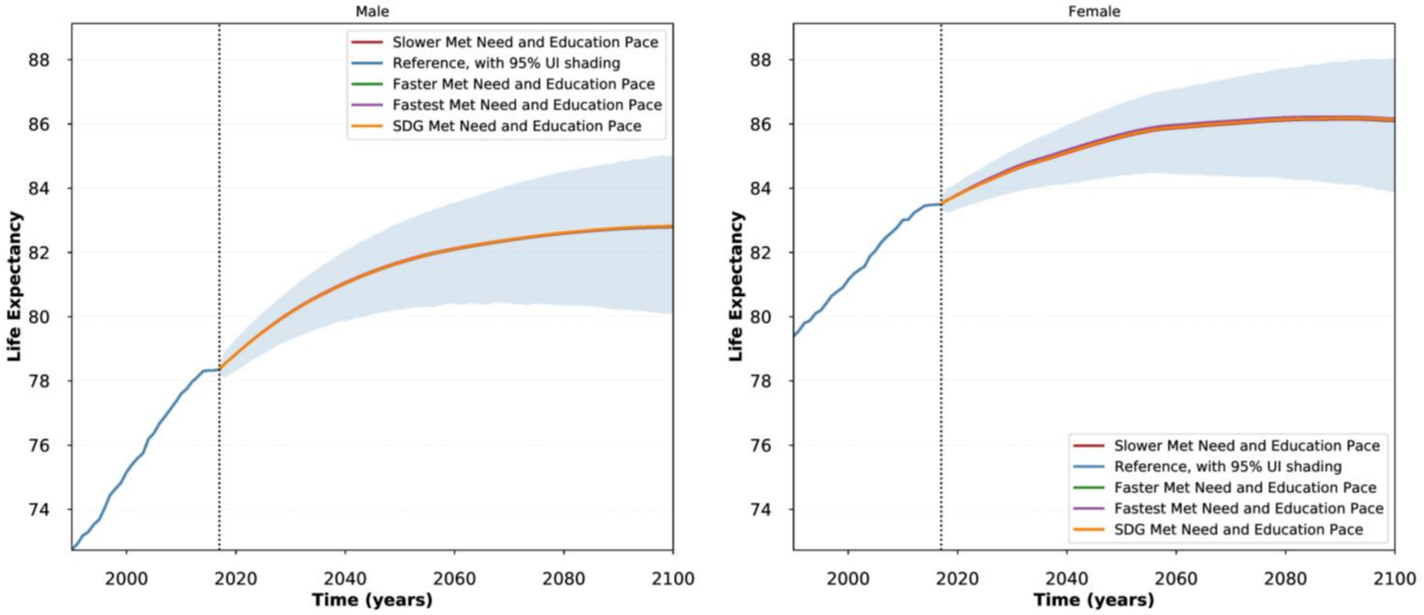

E. Latin America and Caribbean

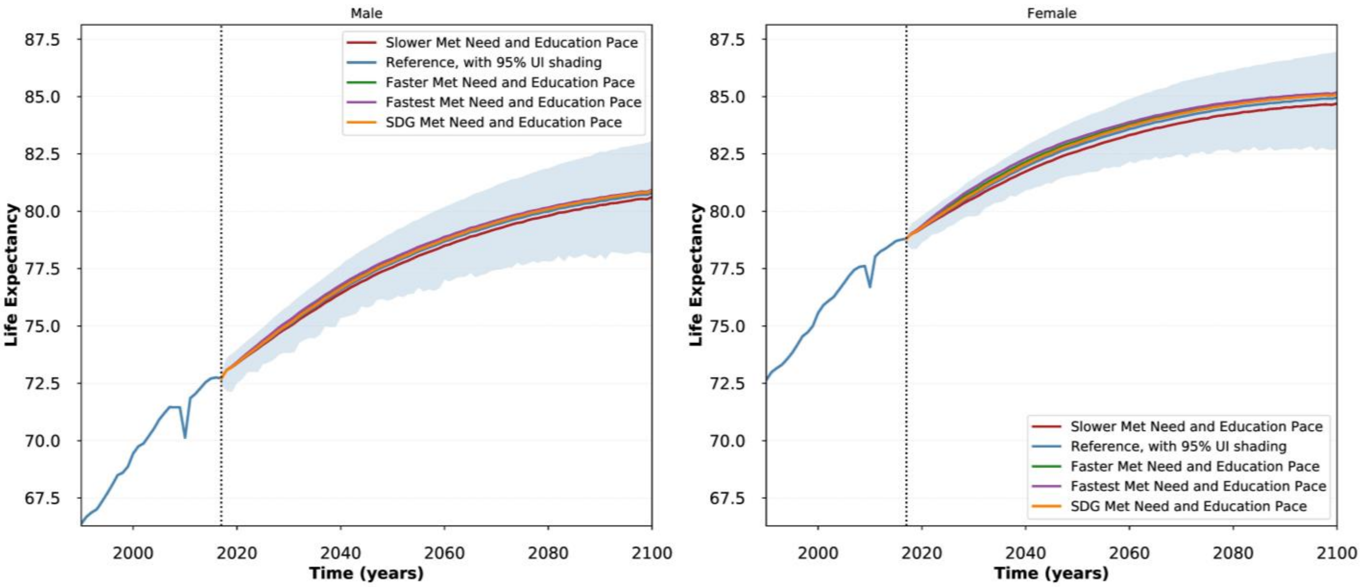

F. South Asia

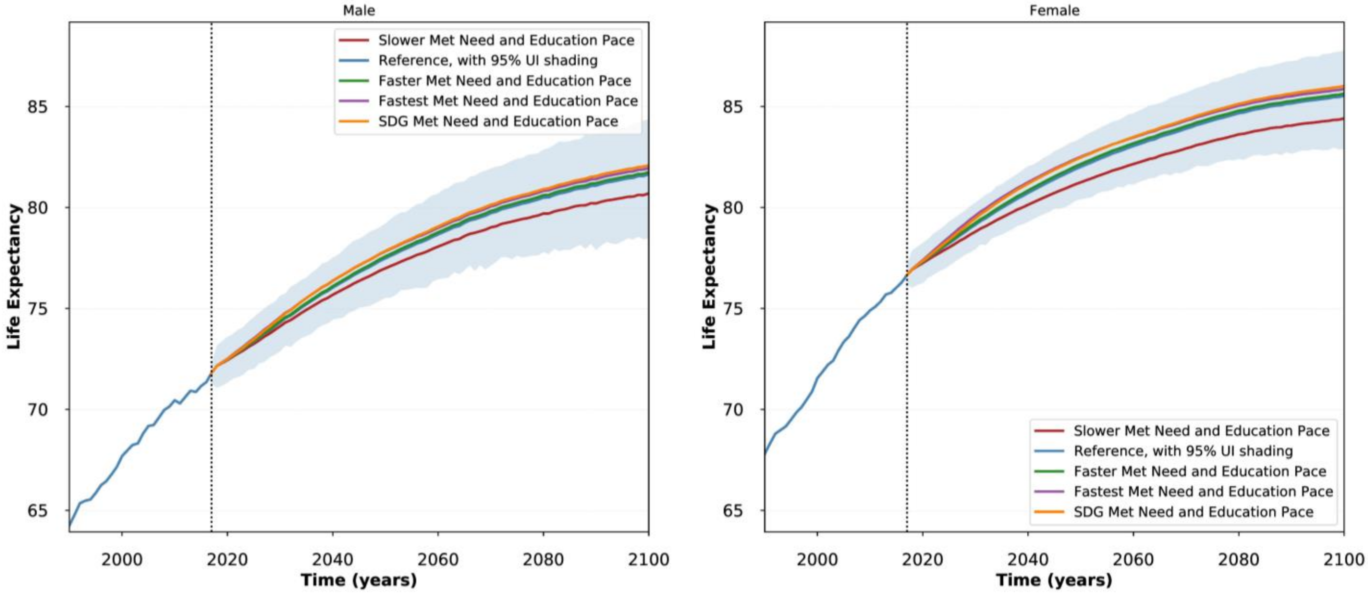

G. North Africa and Middle East

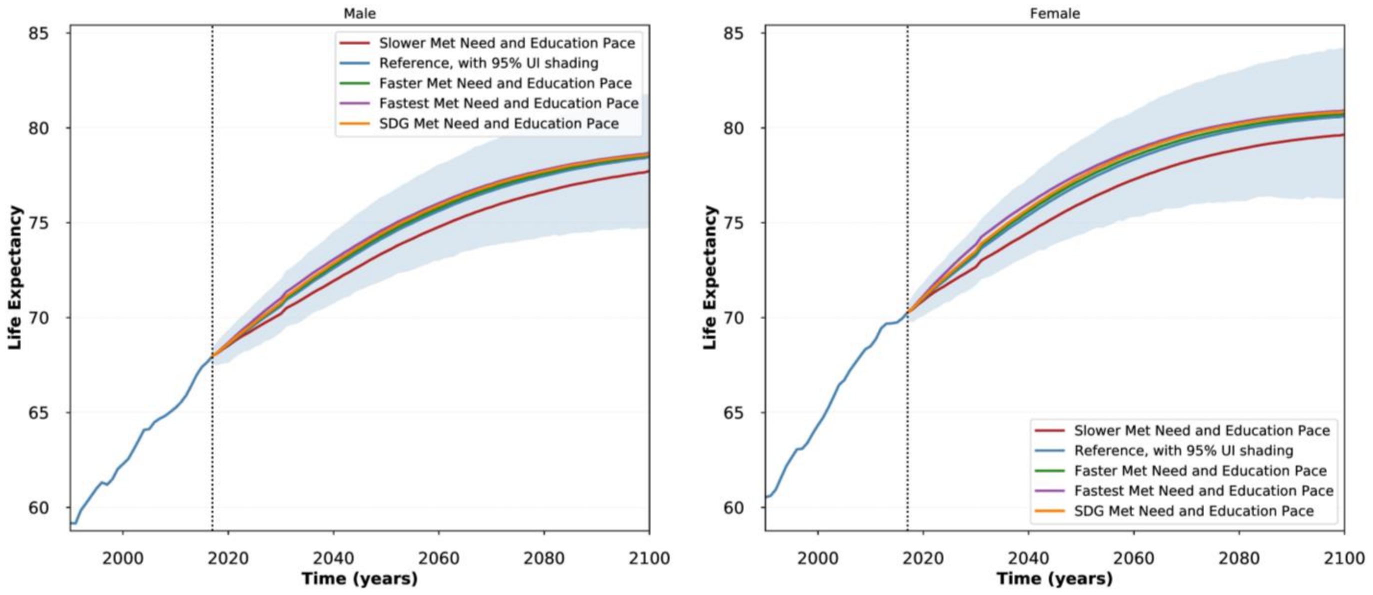

H. Sub-Saharan Africa

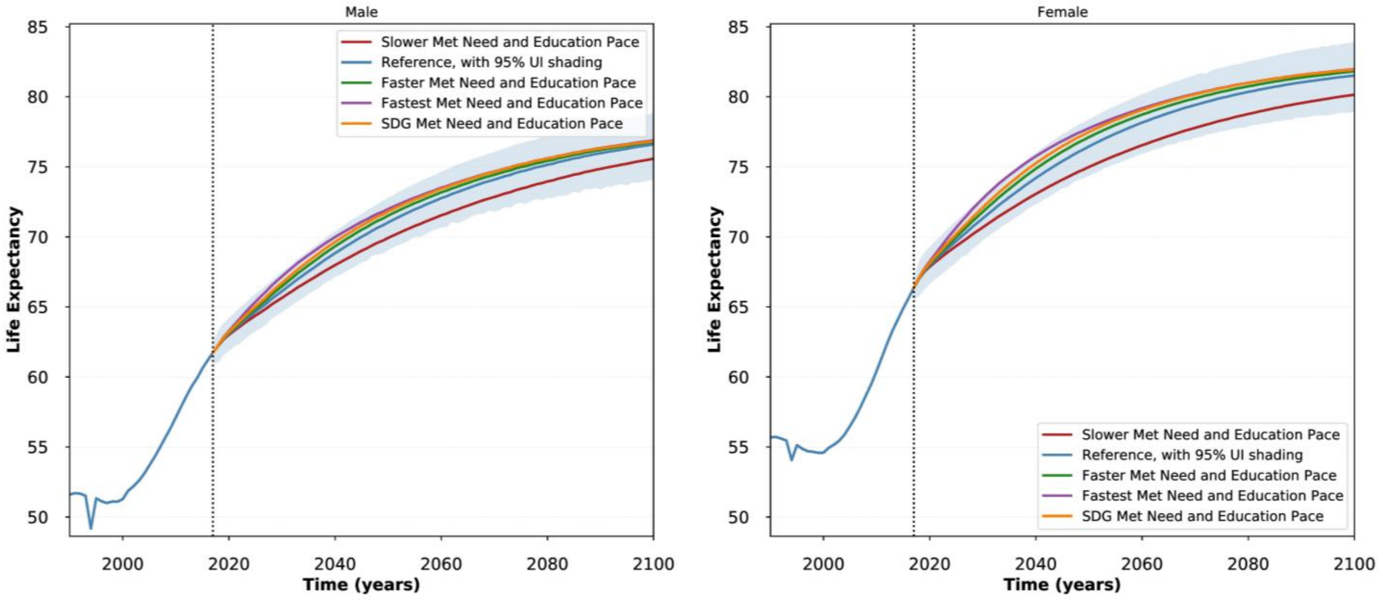

Table 1. Life expectancy by sex in the reference forecast. Values are presented as life expectancy at birth, measured in years, with 95% uncertainty intervals in parentheses. Past estimates are from the Global Burden of Disease Study (GBD) 2017. Highlighted rows indicate region and super-region results from the GBD location hierarchy.

Location	Males				Females				Both			
	1990	2017	2050	2100	1990	2017	2050	2100	1990	2017	2050	2100
**Global**	63·2 (63·1, 63·4)	70·4 (70·2, 70·7)	75·5 (74·1, 76·9)	79·0 (76·6, 81·1)	68·0 (67·9, 68·2)	75·5 (75·3, 75·7)	80·2 (78·7, 81·6)	82·9 (80·4, 85·0)	65·6 (65·4, 65·7)	72·9 (72·7, 73·1)	77·8 (76·4, 79·2)	80·9 (78·7, 83·0)
**Central Europe, Eastern Europe, and Central Asia**	64·9 (64·9, 65·0)	68·5 (68·3, 68·7)	73·9 (70·4, 76·7)	78·6 (71·6, 83·3)	74·0 (73·9, 74·1)	77·6 (77·5, 77·8)	81·7 (79·4, 83·5)	85·0 (80·7, 88·2)	69·5 (69·5, 69·6)	73·1 (72·9, 73·2)	77·8 (75·0, 80·0)	81·7 (76·5, 85·4)
**Central Asia**	64·1 (63·9, 64·3)	67·3 (66·7, 67·8)	72·4 (69·8, 74·8)	78·1 (73·5, 82·2)	71·8 (71·6, 72·0)	74·7 (74·2, 75·3)	79·7 (77·4, 81·7)	84·7 (80·7, 88·1)	68·0 (67·9, 68·2)	71·0 (70·5, 71·4)	76·0 (73·8, 77·9)	81·2 (77·3, 84·6)
Armenia	66·5 (65·9, 67·1)	72·3 (71·9, 72·8)	76·2 (73·4, 79·2)	79·8 (73·7, 85·3)	73·2 (72·7, 73·6)	78·8 (78·4, 79·2)	81·9 (79·9, 83·8)	84·4 (80·4, 88·0)	69·9 (69·5, 70·4)	75·6 (75·4, 75·9)	79·1 (77·1, 81·1)	82·0 (77·7, 85·9)
Azerbaijan	63·4 (62·5, 64·3)	67·0 (65·9, 68·2)	72·7 (69·5, 76·1)	79·0 (73·3, 85·3)	71·1 (70·3, 71·9)	74·7 (73·6, 75·8)	80·1 (77·2, 82·9)	85·2 (80·4, 89·1)	67·4 (66·6, 68·1)	70·7 (69·7, 71·7)	76·3 (73·7, 78·9)	81·7 (77·1, 86·2)
Georgia	65·7 (65·0, 66·4)	68·4 (67·9, 68·8)	72·9 (69·1, 76·5)	78·2 (71·1, 84·5)	73·6 (73·0, 74·2)	77·2 (76·8, 77·7)	81·8 (78·6, 85·0)	85·7 (79·9, 90·9)	69·8 (69·2, 70·3)	72·8 (72·4, 73·1)	77·3 (74·3, 80·4)	81·8 (76·0, 87·2)
Kazakhstan	63·4 (63·1, 63·7)	67·2 (66·5, 67·9)	73·0 (69·2, 77·0)	78·8 (71·4, 85·3)	73·1 (72·9, 73·3)	76·3 (75·6, 77·0)	81·1 (78·4, 83·6)	85·0 (80·2, 88·9)	68·4 (68·1, 68·6)	71·8 (71·2, 72·3)	77·1 (74·2, 79·9)	81·8 (76·5, 86·6)
Kyrgyzstan	61·9 (61·1, 62·7)	69·0 (68·6, 69·4)	74·5 (71·8, 77·1)	79·7 (74·8, 84·1)	70·3 (69·6, 71·1)	76·1 (75·7, 76·5)	81·0 (78·8, 83·2)	85·4 (81·5, 88·9)	66·2 (65·5, 66·8)	72·5 (72·2, 72·9)	77·7 (75·4, 79·9)	82·4 (78·2, 86·1)
Mongolia	58·8 (58·0, 59·6)	64·7 (63·3, 66·1)	70·2 (65·7, 74·4)	75·7 (68·7, 82·5)	64·1 (63·4, 64·8)	73·7 (72·5, 74·9)	80·7 (76·8, 84·3)	84·9 (78·2, 90·7)	61·4 (60·7, 62·0)	69·1 (68·0, 70·1)	75·3 (71·6, 78·8)	80·2 (74·2, 85·9)
Tajikistan	65·1 (64·4, 65·7)	68·2 (67·0, 69·4)	74·3 (70·5, 77·7)	80·0 (74·0, 84·6)	70·2 (69·6, 70·9)	73·9 (72·6, 75·3)	80·0 (76·5, 83·2)	85·4 (80·3, 89·6)	67·7 (67·0, 68·3)	70·9 (69·9, 71·8)	77·1 (73·6, 80·1)	82·5 (77·6, 86·9)
Turkmenistan	62·6 (62·0, 63·2)	66·8 (65·6, 68·0)	74·0 (69·6, 78·1)	80·7 (73·0, 87·3)	69·3 (68·8, 69·8)	74·0 (72·9, 75·0)	80·6 (77·6, 83·1)	85·6 (81·1, 89·6)	66·0 (65·4, 66·6)	70·3 (69·3, 71·2)	77·1 (74·0, 80·2)	83·2 (77·6, 88·1)
Uzbekistan	66·1 (65·7, 66·4)	66·8 (65·3, 68·2)	70·8 (67·6, 73·8)	76·7 (71·0, 81·5)	72·5 (72·2, 72·8)	73·2 (71·6, 74·7)	77·9 (73·9, 81·6)	84·1 (77·9, 89·2)	69·4 (69·0, 69·7)	69·9 (68·8, 71·0)	74·3 (70·9, 77·2)	80·1 (74·7, 84·6)
**Central Europe**	67·2 (67·2, 67·2)	73·6 (73·3, 73·9)	79·1 (77·1, 80·9)	82·0 (78·4, 85·1)	75·0 (74·9, 75·0)	80·5 (80·2, 80·7)	84·3 (82·5, 85·7)	86·3 (82·9, 89·0)	71·0 (71·0, 71·0)	77·0 (76·8, 77·2)	81·6 (79·8, 83·1)	84·1 (80·8, 86·8)
Albania	70·2 (69·9, 70·6)	75·1 (72·9, 77·2)	79·1 (76·2, 82·0)	82·2 (77·3, 86·2)	77·9 (77·6, 78·2)	82·2 (80·0, 84·4)	85·4 (81·9, 88·5)	87·8 (82·4, 92·5)	73·7 (73·4, 74·0)	78·3 (76·7, 79·8)	82·2 (79·3, 84·7)	84·7 (80·5, 88·6)

Location	Males				Females				Both			
	1990	2017	2050	2100	1990	2017	2050	2100	1990	2017	2050	2100
Bosnia and Herzegovina	70·5 (70·3, 70·6)	74·4 (73·6, 75·1)	78·3 (74·9, 81·3)	80·9 (74·6, 85·9)	76·5 (76·3, 76·7)	79·1 (78·4, 79·7)	82·1 (79·6, 84·3)	84·3 (79·9, 88·0)	73·5 (73·4, 73·6)	76·7 (76·2, 77·2)	80·2 (77·3, 82·6)	82·6 (77·6, 86·9)
Bulgaria	68·4 (68·3, 68·5)	71·4 (70·6, 72·2)	76·1 (73·5, 78·7)	80·6 (76·1, 84·7)	75·6 (75·5, 75·7)	78·6 (77·9, 79·3)	82·5 (80·0, 84·8)	85·5 (80·9, 89·3)	71·8 (71·7, 71·9)	74·9 (74·4, 75·4)	79·2 (77·0, 81·4)	83·0 (78·9, 86·7)
Croatia	68·8 (68·7, 68·9)	75·5 (74·8, 76·2)	80·5 (78·1, 82·8)	82·8 (78·2, 86·8)	76·3 (76·2, 76·5)	81·8 (81·1, 82·4)	85·1 (83·0, 87·1)	86·9 (83·3, 90·2)	72·6 (72·5, 72·7)	78·6 (78·1, 79·1)	82·8 (80·8, 84·7)	84·8 (80·9, 88·2)
Czech Republic	67·6 (67·5, 67·6)	76·4 (75·6, 77·0)	81·6 (79·6, 83·5)	84·2 (81·0, 87·1)	75·5 (75·4, 75·7)	82·1 (81·4, 82·7)	85·8 (83·8, 87·5)	87·5 (84·1, 90·5)	71·5 (71·4, 71·6)	79·2 (78·7, 79·7)	83·7 (81·9, 85·3)	85·9 (82·8, 88·5)
Hungary	65·3 (65·2, 65·4)	73·1 (72·4, 73·8)	79·0 (76·8, 81·1)	81·7 (77·8, 85·1)	73·9 (73·8, 74·1)	80·2 (79·5, 80·8)	83·8 (82·0, 85·7)	85·6 (82·2, 88·5)	69·5 (69·4, 69·6)	76·7 (76·2, 77·2)	81·4 (79·7, 83·0)	83·6 (80·3, 86·4)
Montenegro	71·3 (71·0, 71·6)	74·1 (73·0, 75·2)	77·3 (75·1, 79·3)	80·0 (76·3, 83·4)	77·7 (77·3, 78·0)	78·9 (78·1, 79·7)	81·5 (79·4, 83·4)	83·8 (80·2, 87·3)	74·5 (74·2, 74·7)	76·5 (75·8, 77·2)	79·4 (77·4, 81·2)	81·8 (78·3, 85·1)
North Macedonia	69·7 (69·5, 69·9)	73·9 (73·2, 74·6)	77·9 (75·4, 80·4)	81·5 (76·8, 85·7)	74·4 (74·2, 74·7)	80·2 (79·6, 80·8)	84·5 (82·2, 86·5)	87·8 (84·0, 90·9)	71·9 (71·7, 72·1)	76·8 (76·3, 77·3)	81·0 (78·8, 82·9)	84·4 (80·5, 87·8)
Poland	66·8 (66·7, 66·8)	74·0 (73·3, 74·7)	79·6 (77·1, 81·9)	82·2 (77·8, 86·2)	75·7 (75·7, 75·8)	81·9 (81·3, 82·5)	85·4 (83·5, 87·2)	86·9 (83·5, 89·7)	71·2 (71·1, 71·2)	78·0 (77·5, 78·5)	82·5 (80·6, 84·2)	84·5 (80·9, 87·6)
Romania	66·8 (66·8, 66·9)	71·5 (70·8, 72·3)	77·6 (75·1, 80·0)	81·1 (76·5, 84·9)	73·4 (73·4, 73·5)	79·0 (78·4, 79·7)	83·7 (81·8, 85·4)	86·2 (83·1, 89·1)	70·1 (70·0, 70·1)	75·2 (74·7, 75·7)	80·6 (78·7, 82·3)	83·6 (80·2, 86·6)
Serbia	67·3 (67·1, 67·4)	73·6 (73·0, 74·3)	77·9 (75·6, 80·2)	80·7 (76·4, 84·5)	74·2 (74·1, 74·4)	77·9 (77·3, 78·5)	81·2 (78·5, 83·5)	83·6 (78·5, 87·8)	70·6 (70·5, 70·7)	75·7 (75·3, 76·2)	79·5 (77·1, 81·5)	82·1 (77·6, 85·9)
Slovakia	66·7 (66·6, 66·8)	74·1 (73·4, 74·8)	79·6 (77·7, 81·4)	82·2 (78·9, 85·4)	75·5 (75·3, 75·6)	80·7 (80·0, 81·4)	84·2 (82·1, 85·9)	86·1 (82·5, 89·1)	70·9 (70·8, 71·0)	77·5 (77·0, 78·0)	81·8 (80·1, 83·4)	84·2 (81·0, 87·0)
Slovenia	69·7 (69·5, 69·9)	77·9 (77·2, 78·7)	82·4 (79·8, 84·8)	84·0 (79·1, 88·2)	77·8 (77·6, 78·0)	84·3 (83·5, 85·0)	87·4 (85·6, 89·1)	88·5 (85·2, 91·3)	73·9 (73·7, 74·0)	81·1 (80·5, 81·6)	84·8 (82·7, 86·7)	86·2 (82·2, 89·5)
**Eastern Europe**	64·6 (64·5, 64·7)	66·5 (66·3, 66·7)	72·1 (66·3, 76·4)	77·7 (65·3, 85·1)	74·7 (74·7, 74·8)	77·3 (77·1, 77·5)	81·6 (78·4, 84·3)	85·0 (78·4, 89·7)	69·9 (69·8, 69·9)	71·9 (71·8, 72·1)	76·8 (72·7, 80·0)	81·3 (72·3, 87·1)
Belarus	66·1 (65·8, 66·3)	68·9 (68·2, 69·7)	73·8 (70·2, 77·0)	78·5 (71·5, 83·6)	75·6 (75·4, 75·8)	78·8 (78·2, 79·4)	83·0 (80·5, 85·4)	86·3 (81·9, 90·1)	71·0 (70·8, 71·3)	74·0 (73·5, 74·5)	78·4 (75·7, 80·8)	82·3 (77·1, 86·3)
Estonia	64·8 (64·6, 65·0)	73·6 (72·0, 75·3)	79·9 (75·5, 83·8)	83·1 (75·4, 88·9)	75·1 (74·8, 75·3)	82·2 (80·8, 83·6)	86·5 (83·3, 89·5)	88·4 (82·9, 93·0)	70·0 (69·9, 70·2)	78·1 (76·9, 79·3)	83·1 (79·7, 86·4)	85·7 (79·5, 90·8)

Location	Males				Females				Both			
	1990	2017	2050	2100	1990	2017	2050	2100	1990	2017	2050	2100
Latvia	64·7 (64·5, 64·9)	70·1 (68·5, 71·7)	76·0 (70·1, 80·8)	79·7 (69·0, 87·1)	74·9 (74·7, 75·1)	79·9 (78·5, 81·4)	84·0 (80·0, 87·1)	86·1 (78·8, 91·1)	69·9 (69·8, 70·1)	75·2 (74·1, 76·3)	79·9 (75·4, 83·5)	82·8 (74·4, 88·6)
Lithuania	66·4 (66·2, 66·5)	69·7 (68·8, 70·5)	75·3 (70·7, 79·3)	79·3 (70·5, 85·7)	76·3 (76·1, 76·5)	80·4 (79·6, 81·2)	84·0 (81·0, 86·8)	86·1 (80·1, 90·5)	71·4 (71·3, 71·6)	75·1 (74·5, 75·7)	79·6 (76·1, 82·7)	82·6 (76·0, 87·7)
Moldova	64·2 (63·9, 64·6)	68·2 (67·6, 68·7)	73·3 (68·6, 78·1)	79·3 (70·3, 88·0)	71·3 (71·0, 71·6)	76·8 (76·3, 77·3)	82·3 (79·2, 85·2)	87·0 (80·9, 91·9)	67·8 (67·5, 68·2)	72·5 (72·1, 73·0)	77·8 (74·5, 81·3)	83·1 (76·3, 89·3)
Russia	64·1 (64·1, 64·2)	66·7 (66·6, 66·9)	72·3 (65·5, 77·4)	77·6 (63·8, 85·9)	74·7 (74·7, 74·7)	77·3 (77·2, 77·4)	81·5 (77·7, 84·8)	84·7 (77·2, 90·4)	69·6 (69·6, 69·6)	72·1 (72·0, 72·2)	76·9 (72·1, 80·7)	81·1 (70·9, 87·7)
Ukraine	65·6 (65·3, 65·8)	64·7 (63·9, 65·4)	70·2 (63·3, 75·8)	77·7 (63·1, 87·2)	74·9 (74·7, 75·1)	76·6 (75·9, 77·3)	81·2 (77·8, 84·5)	85·6 (78·7, 90·9)	70·4 (70·2, 70·6)	70·6 (70·0, 71·2)	75·6 (70·9, 79·8)	81·6 (72·0, 88·2)
**High-income**	72·7 (72·7, 72·8)	78·4 (78·2, 78·5)	81·7 (80·2, 82·9)	82·8 (80·1, 85·0)	79·4 (79·4, 79·4)	83·5 (83·3, 83·7)	85·6 (84·4, 86·6)	86·1 (83·9, 88·0)	76·1 (76·1, 76·1)	80·9 (80·8, 81·1)	83·7 (82·4, 84·7)	84·5 (82·1, 86·4)
**Australasia**	73·5 (73·4, 73·6)	80·0 (79·0, 81·1)	83·3 (81·3, 85·0)	84·2 (80·7, 87·1)	79·6 (79·5, 79·7)	84·4 (83·5, 85·4)	87·1 (85·6, 88·5)	87·7 (85·1, 89·9)	76·5 (76·5, 76·6)	82·2 (81·5, 82·9)	85·2 (83·6, 86·7)	85·9 (83·2, 88·3)
Australia	73·7 (73·7, 73·8)	80·1 (78·9, 81·3)	83·4 (81·2, 85·2)	84·3 (80·9, 87·1)	80·0 (79·9, 80·1)	84·6 (83·5, 85·7)	87·2 (85·7, 88·7)	87·8 (85·2, 90·1)	76·8 (76·7, 76·9)	82·3 (81·5, 83·1)	85·3 (83·6, 86·8)	86·0 (83·2, 88·5)
New Zealand	72·5 (72·3, 72·6)	79·6 (79·0, 80·2)	83·1 (81·3, 84·9)	84·1 (80·5, 87·2)	78·0 (77·8, 78·1)	83·5 (83·0, 84·1)	86·3 (84·5, 87·9)	86·8 (83·5, 89·8)	75·2 (75·1, 75·3)	81·6 (81·1, 82·0)	84·8 (83·2, 86·3)	85·5 (82·3, 88·2)
**High-income Asia Pacific**	74·4 (74·4, 74·5)	80·6 (80·4, 80·9)	84·2 (82·9, 85·4)	85·1 (82·7, 87·4)	81·0 (81·0, 81·1)	86·7 (86·5, 87·0)	89·1 (87·9, 90·1)	89·9 (87·9, 91·6)	77·8 (77·7, 77·8)	83·6 (83·4, 83·8)	86·6 (85·4, 87·6)	87·4 (85·4, 89·4)
Brunei	69·1 (68·5, 69·7)	73·3 (72·2, 74·4)	77·0 (75·1, 78·7)	79·0 (76·1, 81·7)	72·2 (71·6, 72·8)	77·5 (76·7, 78·4)	80·4 (79·0, 81·9)	82·5 (80·2, 84·8)	70·5 (70·0, 70·9)	75·3 (74·5, 76·1)	78·6 (77·2, 80·0)	80·7 (78·5, 82·9)
Japan	76·2 (76·2, 76·2)	80·9 (80·7, 81·2)	84·0 (82·6, 85·2)	84·8 (82·0, 87·1)	82·2 (82·2, 82·2)	87·0 (86·8, 87·3)	89·2 (88·2, 90·3)	89·9 (88·1, 91·8)	79·3 (79·3, 79·3)	83·9 (83·7, 84·1)	86·5 (85·4, 87·6)	87·2 (85·1, 89·2)
Singapore	73·5 (73·3, 73·7)	81·2 (80·6, 81·9)	84·6 (82·7, 86·4)	85·6 (82·4, 88·8)	78·6 (78·4, 78·8)	87·4 (86·7, 87·9)	90·1 (88·5, 91·8)	90·8 (88·0, 93·3)	76·0 (75·9, 76·1)	84·3 (83·8, 84·7)	87·3 (85·7, 88·9)	88·1 (85·2, 90·8)
South Korea	68·0 (67·9, 68·2)	79·5 (78·7, 80·2)	84·6 (82·9, 86·3)	86·0 (83·3, 88·7)	76·4 (76·3, 76·5)	85·5 (85·0, 86·2)	88·8 (87·3, 90·2)	89·7 (87·3, 92·0)	72·2 (72·1, 72·3)	82·5 (82·0, 83·0)	86·6 (85·2, 88·1)	87·8 (85·5, 90·3)
**High-income North America**	72·2 (72·2, 72·2)	76·3 (76·0, 76·6)	79·5 (78·0, 80·9)	80·9 (78·0, 83·5)	78·9 (78·9, 79·0)	81·2 (80·9, 81·4)	83·3 (82·1, 84·5)	84·3 (82·2, 86·3)	75·6 (75·5, 75·6)	78·8 (78·5, 79·0)	81·4 (80·2, 82·6)	82·6 (80·3, 84·8)

Location	Males				Females				Both			
	1990	2017	2050	2100	1990	2017	2050	2100	1990	2017	2050	2100
Canada	74·0 (74·0, 74·1)	79·6 (79·0, 80·3)	82·9 (81·4, 84·3)	83·8 (81·3, 86·3)	80·4 (80·3, 80·5)	83·7 (83·1, 84·3)	85·9 (84·6, 87·1)	86·8 (84·6, 88·6)	77·2 (77·1, 77·2)	81·7 (81·2, 82·1)	84·5 (83·3, 85·5)	85·3 (83·2, 87·3)
Greenland	61·9 (61·4, 62·5)	70·8 (70·2, 71·5)	75·9 (74·1, 77·8)	78·5 (75·3, 81·3)	69·0 (68·3, 69·6)	77·1 (76·1, 77·9)	81·0 (79·4, 82·3)	82·3 (79·7, 84·5)	65·1 (64·6, 65·5)	73·4 (72·9, 74·1)	78·3 (76·9, 79·7)	80·3 (78·0, 82·5)
USA	72·0 (72·0, 72·0)	76·0 (75·7, 76·3)	79·1 (77·6, 80·6)	80·6 (77·7, 83·2)	78·8 (78·8, 78·8)	80·9 (80·6, 81·2)	83·0 (81·7, 84·3)	84·0 (81·8, 86·1)	75·4 (75·4, 75·4)	78·4 (78·2, 78·6)	81·1 (79·9, 82·3)	82·3 (80·0, 84·5)
**Southern Latin America**	69·2 (69·1, 69·2)	74·4 (73·3, 75·4)	78·8 (76·6, 80·7)	81·0 (77·4, 84·1)	76·1 (76·1, 76·2)	80·4 (79·4, 81·3)	83·5 (81·7, 85·2)	85·1 (82·1, 87·6)	72·6 (72·6, 72·6)	77·4 (76·6, 78·1)	81·1 (79·4, 82·7)	83·0 (80·1, 85·6)
Argentina	68·9 (68·9, 69·0)	73·5 (72·0, 74·9)	78·0 (75·8, 80·1)	80·4 (76·8, 83·7)	75·9 (75·8, 76·0)	79·7 (78·4, 81·0)	82·8 (81·0, 84·5)	84·6 (81·6, 87·3)	72·3 (72·3, 72·4)	76·6 (75·5, 77·6)	80·4 (78·7, 82·1)	82·4 (79·5, 85·1)
Chile	69·8 (69·7, 69·9)	76·9 (75·4, 78·3)	80·9 (78·2, 83·3)	82·9 (78·9, 86·6)	76·4 (76·3, 76·5)	82·0 (80·7, 83·3)	85·2 (82·8, 87·4)	86·7 (82·8, 89·8)	73·1 (73·0, 73·1)	79·5 (78·5, 80·5)	83·1 (80·6, 85·2)	84·8 (81·0, 88·1)
Uruguay	69·4 (69·2, 69·6)	73·5 (72·1, 74·9)	77·8 (74·9, 80·6)	80·0 (74·3, 84·5)	76·8 (76·6, 77·0)	80·4 (79·0, 81·8)	83·6 (81·1, 86·1)	85·4 (81·2, 89·0)	73·0 (72·9, 73·2)	77·0 (75·9, 78·0)	80·7 (78·3, 83·0)	82·6 (78·2, 86·4)
**Western Europe**	73·0 (73·0, 73·0)	79·5 (79·2, 79·8)	83·2 (81·7, 84·5)	84·4 (81·7, 86·8)	79·6 (79·6, 79·6)	84·3 (84·0, 84·6)	86·6 (85·1, 87·8)	87·2 (84·4, 89·3)	76·3 (76·3, 76·4)	81·9 (81·7, 82·1)	84·9 (83·5, 86·1)	85·8 (83·3, 88·0)
Andorra	76·0 (74·4, 77·2)	80·4 (79·3, 81·5)	82·7 (81·3, 84·0)	83·9 (81·8, 85·8)	82·5 (80·7, 84·4)	84·9 (83·4, 86·6)	86·4 (84·0, 88·7)	86·9 (83·6, 90·1)	78·8 (77·6, 79·7)	82·5 (81·6, 83·3)	84·5 (82·8, 86·1)	85·4 (82·8, 87·7)
Austria	72·4 (72·3, 72·5)	79·4 (78·8, 80·1)	83·3 (81·4, 85·3)	85·0 (81·6, 88·2)	79·0 (78·8, 79·1)	84·2 (83·6, 84·8)	86·9 (84·9, 88·7)	87·8 (84·3, 90·8)	75·9 (75·8, 76·0)	81·8 (81·3, 82·3)	85·1 (83·3, 86·9)	86·4 (83·0, 89·4)
Belgium	72·7 (72·6, 72·8)	78·9 (78·3, 79·6)	83·4 (81·7, 84·9)	84·6 (81·6, 87·2)	79·3 (79·2, 79·4)	84·0 (83·3, 84·6)	86·4 (84·5, 87·9)	87·2 (84·0, 89·6)	76·0 (76·0, 76·1)	81·4 (81·0, 81·9)	84·9 (83·3, 86·3)	85·9 (83·2, 88·3)
Cyprus	73·6 (73·4, 73·8)	78·4 (77·4, 79·4)	81·8 (79·4, 84·1)	83·8 (79·8, 87·7)	78·4 (78·2, 78·6)	85·1 (84·2, 85·9)	88·3 (86·4, 89·9)	89·1 (86·1, 91·4)	76·0 (75·8, 76·1)	81·5 (80·8, 82·2)	85·0 (83·2, 86·8)	86·3 (83·1, 89·6)
Denmark	72·2 (72·1, 72·4)	78·9 (78·2, 79·5)	83·0 (81·0, 84·8)	84·0 (80·3, 87·0)	77·8 (77·6, 77·9)	82·6 (81·9, 83·2)	84·9 (82·6, 86·8)	85·6 (81·9, 88·7)	75·0 (74·9, 75·1)	80·7 (80·2, 81·2)	84·0 (82·1, 85·7)	84·8 (81·4, 87·6)
Finland	71·0 (70·9, 71·2)	78·6 (77·8, 79·2)	82·3 (80·4, 84·1)	83·8 (80·5, 86·8)	79·1 (78·9, 79·3)	84·5 (83·7, 85·1)	86·8 (84·7, 88·5)	87·4 (83·8, 90·2)	75·1 (75·0, 75·2)	81·5 (80·9, 81·9)	84·5 (82·7, 86·1)	85·6 (82·4, 88·4)
France	73·1 (73·1, 73·2)	79·9 (79·3, 80·5)	83·6 (81·8, 85·3)	84·8 (81·7, 87·6)	81·3 (81·3, 81·4)	85·9 (85·3, 86·4)	88·1 (86·6, 89·6)	88·6 (86·0, 90·9)	77·2 (77·1, 77·2)	82·9 (82·4, 83·3)	85·9 (84·3, 87·2)	86·7 (84·0, 89·0)

Location	Males				Females				Both			
	1990	2017	2050	2100	1990	2017	2050	2100	1990	2017	2050	2100
Germany	72·1 (72·1, 72·2)	78·2 (76·9, 79·4)	81·9 (79·4, 84·4)	83·7 (79·8, 87·5)	78·6 (78·6, 78·7)	83·2 (82·0, 84·4)	85·4 (82·9, 87·7)	86·3 (82·0, 90·2)	75·5 (75·5, 75·6)	80·7 (79·8, 81·6)	83·7 (81·3, 85·9)	85·0 (81·1, 88·6)
Greece	74·8 (74·7, 74·9)	78·5 (77·8, 79·1)	82·1 (79·8, 84·4)	84·5 (81·0, 87·7)	80·5 (80·4, 80·7)	83·7 (83·1, 84·3)	85·5 (83·5, 87·4)	86·2 (82·4, 89·5)	77·6 (77·5, 77·7)	81·1 (80·6, 81·6)	83·8 (81·9, 85·7)	85·4 (82·2, 88·4)
Iceland	75·5 (75·1, 75·8)	79·9 (79·4, 80·3)	82·7 (80·4, 84·8)	83·6 (78·8, 87·4)	80·2 (79·9, 80·5)	85·8 (85·3, 86·2)	88·4 (86·9, 89·6)	88·8 (86·5, 90·7)	77·8 (77·6, 78·1)	82·6 (82·3, 82·9)	85·4 (83·7, 87·0)	86·1 (82·7, 88·9)
Ireland	72·3 (72·1, 72·5)	80·0 (79·3, 80·7)	84·5 (82·4, 86·4)	85·6 (81·7, 88·9)	77·7 (77·5, 77·9)	83·7 (83·0, 84·4)	86·9 (84·7, 88·8)	87·8 (84·1, 91·0)	74·9 (74·8, 75·0)	81·8 (81·4, 82·3)	85·7 (83·9, 87·4)	86·7 (83·3, 89·6)
Israel	75·8 (75·7, 76·0)	81·1 (80·4, 81·6)	84·2 (82·2, 86·1)	84·9 (81·3, 88·2)	79·1 (78·9, 79·3)	84·5 (83·9, 85·2)	87·2 (85·2, 88·9)	87·6 (84·3, 90·4)	77·5 (77·4, 77·6)	82·9 (82·4, 83·3)	85·7 (83·8, 87·4)	86·3 (82·9, 89·2)
Italy	73·7 (73·7, 73·8)	80·9 (80·3, 81·5)	84·3 (82·8, 85·8)	85·4 (82·8, 87·8)	80·4 (80·3, 80·4)	85·4 (84·8, 86·0)	87·6 (86·0, 89·2)	88·4 (85·5, 90·9)	77·1 (77·0, 77·1)	83·2 (82·8, 83·6)	86·0 (84·5, 87·4)	86·9 (84·4, 89·2)
Luxembourg	71·8 (71·6, 72·1)	79·9 (78·9, 81·0)	84·3 (82·7, 86·1)	85·8 (82·7, 88·4)	79·0 (78·7, 79·2)	83·3 (82·4, 84·3)	86·0 (84·2, 87·7)	86·9 (84·0, 89·5)	75·4 (75·2, 75·6)	81·6 (80·9, 82·3)	85·2 (83·7, 86·7)	86·4 (83·8, 88·8)
Malta	74·2 (73·9, 74·4)	79·0 (78·5, 79·5)	82·5 (80·3, 84·5)	84·0 (79·7, 87·5)	78·8 (78·5, 79·0)	83·0 (82·4, 83·5)	85·3 (83·5, 86·9)	86·4 (83·3, 89·0)	76·5 (76·3, 76·7)	81·0 (80·6, 81·4)	83·9 (82·2, 85·6)	85·2 (81·9, 88·2)
Netherlands	73·8 (73·7, 73·9)	80·0 (79·4, 80·6)	83·8 (82·2, 85·3)	84·8 (81·8, 87·3)	80·2 (80·1, 80·3)	83·2 (82·6, 83·9)	85·1 (83·4, 86·7)	85·7 (82·8, 88·5)	77·0 (76·9, 77·1)	81·6 (81·1, 82·0)	84·5 (83·1, 85·7)	85·3 (82·7, 87·4)
Norway	73·4 (73·3, 73·5)	80·5 (80·3, 80·8)	83·9 (82·6, 85·2)	84·7 (81·8, 87·2)	80·0 (80·0, 80·1)	84·2 (84·0, 84·4)	86·4 (84·8, 87·9)	86·9 (83·9, 89·5)	76·6 (76·6, 76·7)	82·4 (82·2, 82·5)	85·2 (83·9, 86·4)	85·8 (83·2, 88·1)
Portugal	70·7 (70·6, 70·8)	78·6 (77·9, 79·3)	82·7 (81·0, 84·3)	84·2 (81·0, 86·8)	77·8 (77·7, 77·9)	84·4 (83·8, 85·0)	87·3 (85·5, 88·9)	88·1 (84·8, 90·8)	74·2 (74·2, 74·3)	81·5 (81·1, 82·0)	85·0 (83·5, 86·6)	86·1 (83·2, 88·6)
Spain	73·5 (73·4, 73·6)	80·2 (79·6, 80·8)	84·2 (82·9, 85·6)	85·4 (83·1, 87·6)	80·6 (80·6, 80·7)	86·0 (85·4, 86·5)	88·1 (86·6, 89·4)	88·6 (86·1, 90·8)	77·1 (77·0, 77·1)	83·1 (82·7, 83·5)	86·2 (84·9, 87·3)	87·0 (84·7, 89·0)
Sweden	75·0 (74·8, 75·1)	80·9 (80·3, 81·4)	83·9 (82·5, 85·3)	84·7 (82·0, 87·0)	80·5 (80·4, 80·6)	84·3 (83·8, 84·8)	86·2 (84·5, 87·6)	86·8 (83·9, 89·3)	77·7 (77·6, 77·8)	82·6 (82·2, 83·0)	85·1 (83·7, 86·3)	85·8 (83·3, 87·9)
Switzerland	74·4 (74·3, 74·6)	82·2 (81·6, 82·8)	85·6 (83·9, 87·2)	86·7 (83·8, 89·3)	81·2 (81·0, 81·3)	85·9 (85·3, 86·5)	88·0 (86·7, 89·4)	88·6 (86·3, 90·8)	77·8 (77·7, 77·9)	84·1 (83·6, 84·5)	86·9 (85·5, 88·1)	87·7 (85·3, 89·9)
UK	73·0 (72·9, 73·0)	79·2 (79·1, 79·3)	82·5 (80·8, 84·1)	83·7 (80·5, 86·5)	78·5 (78·4, 78·5)	82·8 (82·6, 82·9)	85·2 (83·6, 86·6)	86·1 (83·3, 88·5)	75·7 (75·7, 75·8)	81·0 (80·9, 81·1)	83·9 (82·5, 85·2)	84·9 (82·2, 87·3)

Location	Males				Females				Both			
	1990	2017	2050	2100	1990	2017	2050	2100	1990	2017	2050	2100
**Latin America and Caribbean**	66·4 (66·3, 66·5)	72·7 (72·5, 73·0)	77·7 (76·2, 79·1)	80·8 (78·2, 83·1)	72·6 (72·5, 72·7)	78·8 (78·6, 79·0)	82·9 (81·6, 84·0)	85·0 (82·7, 87·0)	69·4 (69·4, 69·5)	75·7 (75·6, 75·9)	80·3 (78·9, 81·5)	82·9 (80·5, 84·9)
**Andean Latin America**	66·8 (66·1, 67·4)	76·1 (74·9, 77·2)	80·3 (77·7, 82·5)	82·6 (78·7, 85·8)	70·6 (70·1, 71·2)	79·3 (78·2, 80·3)	83·2 (80·7, 85·7)	85·2 (81·0, 88·9)	68·7 (68·2, 69·2)	77·7 (76·9, 78·5)	81·8 (79·3, 84·0)	83·9 (80·2, 87·1)
Bolivia	59·8 (58·5, 61·1)	71·6 (69·0, 74·3)	76·6 (73·8, 79·1)	79·4 (76·2, 82·4)	62·2 (61·0, 63·4)	74·5 (72·4, 76·8)	80·0 (77·9, 82·2)	82·8 (80·1, 85·5)	61·0 (60·0, 61·9)	73·0 (71·3, 74·8)	78·3 (76·0, 80·4)	81·1 (78·3, 83·7)
Ecuador	69·8 (69·4, 70·1)	75·0 (73·5, 76·3)	79·0 (75·5, 81·8)	82·2 (76·7, 86·6)	74·4 (74·1, 74·7)	78·7 (77·5, 79·9)	82·1 (78·8, 85·0)	84·6 (78·8, 89·3)	72·0 (71·8, 72·3)	76·8 (75·8, 77·8)	80·5 (77·3, 83·2)	83·4 (78·0, 87·9)
Peru	68·0 (66·9, 69·1)	78·3 (76·4, 80·2)	82·2 (79·0, 85·4)	84·3 (79·7, 88·7)	72·3 (71·3, 73·2)	81·5 (79·7, 83·2)	85·1 (81·7, 88·5)	86·8 (81·4, 92·0)	70·1 (69·3, 70·9)	79·9 (78·6, 81·2)	83·6 (80·9, 86·6)	85·5 (80·8, 90·1)
**Caribbean**	66·4 (65·9, 66·9)	70·2 (69·2, 71·1)	74·3 (71·6, 77·1)	77·5 (73·8, 81·2)	70·5 (70·1, 71·0)	75·2 (74·2, 76·1)	79·2 (76·9, 81·5)	82·4 (79·1, 85·5)	68·4 (68·1, 68·8)	72·7 (71·9, 73·4)	76·7 (74·5, 79·2)	79·9 (76·6, 83·2)
Antigua and Barbuda	70·8 (70·2, 71·4)	75·0 (74·0, 75·9)	78·4 (76·9, 79·9)	80·8 (78·2, 82·9)	77·8 (77·1, 78·5)	78·6 (77·8, 79·4)	81·0 (79·6, 82·5)	83·3 (80·9, 85·5)	74·3 (73·7, 74·8)	76·8 (76·1, 77·6)	79·7 (78·4, 81·1)	82·0 (79·8, 84·1)
The Bahamas	67·4 (67·0, 67·8)	70·6 (69·3, 71·9)	74·6 (71·1, 77·6)	78·1 (71·9, 82·8)	74·7 (74·3, 75·1)	76·5 (75·3, 77·7)	79·0 (76·4, 81·5)	81·6 (77·3, 85·6)	71·0 (70·7, 71·3)	73·5 (72·7, 74·5)	76·8 (74·3, 79·0)	79·8 (75·3, 83·5)
Barbados	71·3 (71·0, 71·7)	75·5 (74·4, 76·6)	78·9 (76·1, 81·4)	81·0 (76·2, 84·9)	76·3 (75·9, 76·6)	78·8 (77·8, 79·8)	81·5 (79·6, 83·4)	83·3 (80·2, 86·0)	73·9 (73·6, 74·2)	77·2 (76·4, 78·0)	80·3 (78·2, 82·1)	82·1 (78·5, 85·2)
Belize	70·4 (69·6, 71·1)	70·8 (70·1, 71·5)	75·6 (69·8, 79·8)	80·1 (71·0, 86·6)	73·9 (73·2, 74·5)	77·1 (76·4, 77·7)	80·7 (77·3, 83·4)	83·6 (78·1, 87·8)	72·1 (71·5, 72·6)	73·8 (73·2, 74·3)	78·1 (73·6, 81·3)	81·9 (74·7, 87·0)
Bermuda	69·7 (69·3, 70·2)	77·0 (76·3, 77·6)	80·7 (78·6, 82·7)	82·4 (78·5, 85·9)	78·2 (77·8, 78·6)	85·5 (84·7, 86·4)	88·6 (87·5, 89·7)	89·0 (87·1, 90·9)	73·8 (73·5, 74·2)	81·1 (80·6, 81·7)	84·6 (83·0, 85·9)	85·6 (82·8, 88·1)
Cuba	73·0 (72·9, 73·1)	76·0 (74·5, 77·4)	79·5 (76·7, 82·2)	82·1 (77·7, 86·2)	76·8 (76·7, 76·9)	80·5 (79·1, 81·8)	83·6 (81·4, 86·0)	85·7 (81·9, 89·5)	74·8 (74·8, 74·9)	78·2 (77·2, 79·1)	81·5 (79·3, 83·6)	83·8 (80·3, 87·3)
Dominica	70·5 (70·0, 71·0)	70·2 (68·8, 71·4)	72·3 (70·0, 74·4)	75·8 (72·4, 78·9)	75·2 (74·7, 75·7)	74·9 (73·7, 76·1)	76·8 (74·8, 78·5)	80·1 (77·6, 82·3)	73·0 (72·6, 73·3)	72·4 (71·2, 73·4)	74·5 (72·6, 76·1)	77·9 (75·3, 80·3)
Dominican Republic	69·5 (68·3, 70·7)	70·0 (67·9, 72·1)	74·5 (71·5, 77·5)	78·6 (73·8, 83·3)	74·3 (73·4, 75·4)	77·0 (75·2, 78·8)	81·0 (77·9, 83·8)	84·3 (79·4, 88·8)	71·9 (71·0, 72·8)	73·3 (71·8, 74·8)	77·6 (74·8, 80·2)	81·4 (76·9, 85·7)
Grenada	67·3 (66·7, 67·8)	73·5 (72·7, 74·3)	77·9 (75·6, 80·2)	80·8 (76·7, 85·0)	71·8 (71·2, 72·4)	75·5 (74·7, 76·4)	78·8 (77·1, 80·7)	81·5 (78·4, 84·7)	69·6 (69·1, 70·1)	74·4 (73·7, 75·1)	78·4 (76·5, 80·3)	81·2 (77·6, 84·6)

Location	Males				Females				Both			
	1990	2017	2050	2100	1990	2017	2050	2100	1990	2017	2050	2100
Guyana	63·0 (62·5, 63·5)	66·6 (64·7, 68·4)	71·6 (67·1, 75·7)	77·0 (69·6, 83·2)	69·5 (69·1, 69·9)	72·2 (70·5, 74·0)	77·3 (73·4, 81·2)	82·1 (76·0, 87·8)	66·1 (65·7, 66·5)	69·3 (68·0, 70·7)	74·4 (70·8, 77·8)	79·5 (73·4, 84·9)
Haiti	54·2 (52·6, 55·8)	63·8 (61·3, 66·3)	70·0 (65·7, 73·4)	74·9 (70·3, 78·8)	55·5 (53·9, 57·0)	66·0 (63·3, 68·8)	73·8 (70·1, 77·2)	79·8 (75·7, 83·3)	54·8 (53·6, 56·1)	64·9 (62·8, 66·9)	71·9 (68·3, 75·2)	77·3 (73·1, 80·7)
Jamaica	73·6 (73·0, 74·3)	71·6 (69·5, 73·7)	74·0 (67·2, 79·9)	78·5 (66·9, 87·2)	76·4 (75·7, 77·2)	77·2 (75·1, 79·1)	79·8 (76·9, 82·4)	82·9 (78·6, 86·6)	75·1 (74·5, 75·6)	74·3 (72·8, 75·8)	76·9 (72·4, 80·8)	80·7 (73·5, 86·7)
Puerto Rico	70·0 (69·8, 70·2)	74·2 (73·4, 75·0)	79·8 (76·9, 82·5)	81·6 (76·3, 86·0)	78·5 (78·3, 78·7)	81·2 (80·5, 81·8)	85·2 (83·0, 87·3)	86·2 (81·7, 89·9)	74·2 (74·0, 74·3)	77·7 (77·2, 78·2)	82·6 (80·3, 84·7)	83·8 (79·4, 87·7)
Saint Lucia	67·9 (67·4, 68·3)	72·8 (71·9, 73·7)	77·4 (74·5, 79·9)	80·5 (75·5, 85·0)	73·3 (72·9, 73·7)	78·0 (77·0, 78·9)	81·7 (79·8, 83·4)	84·2 (81·2, 86·8)	70·6 (70·2, 70·9)	75·3 (74·6, 76·0)	79·5 (77·4, 81·4)	82·4 (78·5, 85·8)
Saint Vincent and the Grenadines	69·2 (68·7, 69·8)	70·0 (69·0, 70·9)	73·0 (70·0, 75·9)	76·5 (71·2, 81·3)	73·1 (72·5, 73·6)	75·7 (74·8, 76·6)	79·3 (75·9, 82·1)	82·4 (77·0, 87·4)	71·2 (70·7, 71·6)	72·6 (71·8, 73·3)	76·1 (73·3, 78·5)	79·4 (74·7, 84·0)
Suriname	66·0 (65·0, 67·1)	68·8 (67·0, 70·4)	73·1 (69·5, 76·6)	77·0 (70·6, 82·7)	71·1 (70·2, 72·1)	75·1 (73·8, 76·4)	79·2 (76·4, 81·8)	82·4 (77·7, 86·5)	68·5 (67·7, 69·4)	71·8 (70·7, 73·0)	76·1 (73·2, 78·9)	79·7 (74·5, 84·3)
Trinidad and Tobago	67·4 (67·0, 67·8)	71·0 (68·3, 73·8)	76·0 (71·5, 79·8)	79·7 (72·7, 85·6)	72·4 (72·1, 72·7)	77·3 (74·6, 79·9)	82·2 (78·9, 85·4)	85·5 (80·5, 90·0)	69·8 (69·5, 70·1)	74·0 (72·0, 75·9)	79·0 (75·5, 82·3)	82·5 (77·0, 87·5)
Virgin Islands	68·9 (68·2, 69·7)	69·5 (67·9, 71·7)	71·9 (69·4, 75·4)	74·2 (70·9, 78·9)	76·1 (75·1, 76·9)	78·7 (77·2, 80·0)	81·3 (79·8, 82·8)	83·0 (80·8, 85·0)	72·4 (71·7, 73·0)	73·8 (72·6, 75·3)	76·7 (74·9, 79·0)	78·5 (75·8, 81·5)
**Central Latin America**	68·2 (68·1, 68·3)	73·2 (72·7, 73·7)	78·2 (76·4, 79·7)	81·2 (78·1, 84·0)	74·1 (74·0, 74·2)	79·3 (79·0, 79·6)	83·0 (81·6, 84·4)	85·1 (82·4, 87·6)	71·1 (71·0, 71·2)	76·3 (76·0, 76·6)	80·6 (79·1, 82·0)	83·1 (80·4, 85·9)
Colombia	68·0 (67·7, 68·3)	77·0 (75·5, 78·5)	82·3 (79·4, 85·3)	84·8 (80·4, 89·2)	74·7 (74·4, 74·9)	82·2 (80·9, 83·4)	86·6 (84·7, 88·3)	88·4 (85·6, 90·9)	71·3 (71·0, 71·5)	79·7 (78·7, 80·7)	84·5 (82·4, 86·6)	86·6 (83·2, 89·8)
Costa Rica	74·4 (74·2, 74·6)	76·1 (75·3, 76·9)	79·4 (76·6, 82·2)	81·7 (76·7, 86·3)	78·8 (78·6, 79·0)	82·2 (81·4, 82·9)	85·4 (82·9, 87·8)	87·3 (82·8, 91·2)	76·6 (76·4, 76·7)	79·1 (78·6, 79·7)	82·5 (80·1, 84·7)	84·4 (80·1, 88·3)
El Salvador	64·9 (64·5, 65·3)	69·3 (66·6, 71·9)	73·6 (69·1, 77·5)	77·4 (70·9, 82·8)	73·7 (73·3, 74·0)	78·1 (75·9, 80·2)	81·3 (77·8, 84·9)	83·8 (77·5, 89·1)	69·3 (68·9, 69·6)	73·9 (72·0, 75·7)	77·6 (73·9, 80·9)	80·6 (74·8, 85·5)
Guatemala	60·3 (59·8, 60·8)	69·3 (67·4, 71·0)	75·9 (70·3, 80·3)	80·4 (68·8, 87·5)	65·6 (65·2, 66·1)	76·2 (74·7, 77·8)	82·4 (77·3, 86·7)	86·0 (76·4, 92·6)	62·9 (62·4, 63·4)	72·8 (71·5, 74·0)	79·2 (74·1, 83·3)	83·2 (72·9, 89·7)
Honduras	66·7 (64·9, 68·7)	73·0 (70·3, 75·7)	77·7 (68·5, 80·5)	81·5 (72·8, 84·7)	71·3 (69·8, 72·8)	75·0 (72·5, 78·0)	79·0 (73·5, 82·5)	82·7 (76·8, 87·2)	69·0 (67·8, 70·3)	74·0 (72·2, 75·9)	78·4 (70·8, 81·5)	82·1 (75·3, 85·9)

Location	Males				Females				Both			
	1990	2017	2050	2100	1990	2017	2050	2100	1990	2017	2050	2100
Mexico	68·8 (68·7, 68·9)	72·6 (72·4, 72·8)	77·6 (76·0, 79·2)	80·5 (77·3, 83·4)	74·6 (74·5, 74·7)	78·6 (78·4, 78·8)	82·1 (80·5, 83·6)	84·2 (81·3, 87·2)	71·6 (71·5, 71·7)	75·6 (75·5, 75·7)	79·9 (78·4, 81·4)	82·3 (79·5, 85·2)
Nicaragua	70·0 (69·0, 71·0)	76·7 (75·1, 78·1)	80·3 (76·5, 83·0)	83·5 (78·9, 87·1)	74·4 (73·5, 75·3)	80·7 (79·3, 82·0)	84·0 (81·2, 86·3)	86·6 (82·9, 89·9)	72·2 (71·4, 73·0)	78·7 (77·7, 79·7)	82·2 (79·0, 84·6)	85·2 (81·3, 88·5)
Panama	73·8 (73·3, 74·2)	76·6 (75·7, 77·5)	80·3 (77·9, 82·7)	83·0 (79·0, 86·7)	78·1 (77·7, 78·5)	81·7 (80·9, 82·5)	85·0 (82·7, 87·1)	86·7 (82·7, 90·1)	75·8 (75·5, 76·2)	79·1 (78·4, 79·7)	82·6 (80·5, 84·6)	84·9 (81·1, 88·3)
Venezuela	69·2 (69·1, 69·3)	71·1 (68·7, 73·5)	75·4 (71·3, 78·6)	79·6 (74·2, 84·0)	75·0 (74·9, 75·1)	79·4 (77·6, 81·3)	83·2 (80·5, 85·5)	85·5 (81·4, 89·2)	72·0 (72·0, 72·1)	75·1 (73·3, 76·6)	79·2 (76·1, 81·6)	82·5 (78·0, 86·1)
**Tropical Latin America**	64·5 (64·3, 64·6)	72·0 (71·8, 72·1)	77·2 (76·2, 78·4)	80·1 (78·1, 82·2)	72·0 (71·9, 72·2)	78·9 (78·8, 79·1)	83·3 (82·3, 84·4)	85·2 (83·5, 87·1)	68·1 (68·0, 68·3)	75·4 (75·3, 75·6)	80·3 (79·4, 81·3)	82·6 (80·9, 84·5)
Brazil	64·3 (64·1, 64·4)	71·9 (71·8, 72·1)	77·3 (76·2, 78·4)	80·1 (78·1, 82·2)	71·9 (71·8, 72·1)	78·9 (78·8, 79·1)	83·3 (82·3, 84·4)	85·3 (83·5, 87·1)	68·0 (67·8, 68·1)	75·4 (75·3, 75·5)	80·3 (79·4, 81·2)	82·6 (81·0, 84·5)
Paraguay	72·1 (71·3, 72·9)	73·5 (71·1, 76·0)	76·7 (73·5, 79·9)	80·1 (74·9, 85·1)	76·3 (75·5, 77·1)	78·9 (76·8, 81·1)	82·4 (78·9, 85·7)	85·0 (78·8, 89·9)	74·2 (73·6, 74·8)	76·1 (74·4, 77·6)	79·5 (76·4, 82·4)	82·5 (77·5, 87·0)
**North Africa and Middle East**	64·3 (63·9, 64·6)	71·8 (71·3, 72·3)	77·5 (75·5, 79·2)	81·7 (78·5, 84·4)	67·8 (67·4, 68·1)	76·7 (76·2, 77·1)	82·0 (80·4, 83·4)	85·5 (82·9, 87·8)	65·9 (65·6, 66·2)	74·0 (73·7, 74·4)	79·6 (77·9, 81·2)	83·6 (80·8, 86·0)
**North Africa and Middle East**	64·3 (63·9, 64·6)	71·8 (71·3, 72·3)	77·5 (75·5, 79·2)	81·7 (78·5, 84·4)	67·8 (67·4, 68·1)	76·7 (76·2, 77·1)	82·0 (80·4, 83·4)	85·5 (82·9, 87·8)	65·9 (65·6, 66·2)	74·0 (73·7, 74·4)	79·6 (77·9, 81·2)	83·6 (80·8, 86·0)
Afghanistan	49·2 (46·9, 51·5)	62·8 (60·2, 65·2)	70·8 (66·4, 74·8)	78·0 (71·9, 82·8)	47·9 (45·5, 50·1)	62·5 (59·8, 65·1)	72·3 (69·0, 75·2)	80·6 (77·3, 83·6)	48·5 (46·6, 50·4)	62·6 (60·7, 64·4)	71·6 (67·9, 74·7)	79·3 (74·9, 83·0)
Algeria	70·4 (69·5, 71·3)	77·6 (76·9, 78·2)	82·7 (81·5, 83·8)	85·9 (83·9, 87·7)	73·3 (72·4, 74·1)	79·0 (78·4, 79·6)	83·1 (81·7, 84·5)	86·2 (83·9, 88·4)	71·8 (71·1, 72·5)	78·3 (77·8, 78·8)	83·0 (81·8, 84·2)	86·1 (84·2, 88·1)
Bahrain	69·4 (68·9, 69·9)	79·0 (78·0, 80·0)	84·1 (81·8, 86·3)	84·6 (80·5, 88·2)	71·9 (71·4, 72·4)	80·5 (79·6, 81·4)	85·5 (83·1, 87·7)	86·4 (82·4, 89·8)	70·4 (70·1, 70·8)	79·6 (78·9, 80·3)	84·7 (82·4, 86·7)	85·4 (81·5, 88·8)
Egypt	61·8 (61·2, 62·4)	68·0 (66·6, 69·3)	73·9 (71·4, 76·4)	80·0 (75·6, 83·9)	65·8 (65·2, 66·3)	74·3 (72·9, 75·8)	81·0 (78·0, 83·8)	85·8 (81·3, 89·8)	63·5 (63·0, 64·1)	70·6 (69·5, 71·7)	77·3 (74·6, 79·8)	82·8 (78·5, 86·6)
Iran	66·1 (65·6, 66·5)	75·4 (75·3, 75·5)	79·3 (76·8, 80·7)	82·7 (79·8, 85·1)	71·0 (70·5, 71·5)	79·3 (79·2, 79·3)	82·9 (81·1, 84·4)	85·7 (82·8, 88·2)	68·4 (68·0, 68·7)	77·3 (77·2, 77·3)	81·0 (79·0, 82·5)	84·2 (81·4, 86·6)
Iraq	64·5 (62·5, 66·4)	74·3 (73·4, 75·1)	83·1 (79·3, 86·7)	86·5 (81·8, 91·0)	67·7 (65·9, 69·5)	79·2 (78·5, 80·1)	87·1 (84·2, 89·8)	90·2 (86·0, 93·9)	66·0 (64·7, 67·3)	76·7 (76·0, 77·3)	85·1 (82·0, 88·0)	88·5 (84·2, 92·3)

Location	Males				Females				Both			
	1990	2017	2050	2100	1990	2017	2050	2100	1990	2017	2050	2100
Jordan	70·3 (68·8, 71·8)	77·5 (76·1, 78·8)	82·2 (80·1, 84·0)	84·7 (81·4, 87·6)	71·6 (70·1, 73·0)	80·6 (79·3, 81·8)	85·8 (82·9, 88·5)	88·0 (83·1, 92·6)	70·9 (69·8, 72·0)	78·9 (77·9, 79·8)	83·8 (81·5, 85·8)	86·3 (82·5, 89·7)
Kuwait	74·0 (73·7, 74·3)	81·2 (80·5, 81·8)	84·5 (81·7, 87·2)	86·2 (80·7, 90·7)	77·0 (76·7, 77·2)	86·6 (86·2, 87·1)	90·6 (88·8, 92·5)	91·7 (88·5, 94·6)	75·2 (75·0, 75·4)	83·2 (82·7, 83·7)	87·2 (84·9, 89·5)	88·9 (84·5, 92·6)
Lebanon	67·3 (66·1, 68·9)	75·6 (74·7, 76·3)	80·0 (78·0, 82·0)	82·8 (79·5, 86·3)	73·5 (72·2, 75·2)	79·6 (78·9, 80·4)	83·0 (81·4, 84·5)	84·7 (82·0, 87·1)	70·4 (69·4, 71·6)	77·6 (77·0, 78·3)	81·5 (79·8, 83·2)	83·8 (81·0, 86·6)
Libya	71·0 (69·1, 72·8)	71·4 (69·6, 73·4)	74·7 (53·1, 79·1)	77·5 (54·3, 83·7)	73·7 (72·0, 75·4)	75·3 (73·5, 77·1)	78·7 (67·2, 82·9)	81·6 (69·0, 87·8)	72·2 (70·9, 73·5)	73·2 (71·9, 74·5)	76·6 (59·2, 80·6)	79·5 (61·1, 85·2)
Morocco	66·9 (65·9, 67·9)	73·1 (70·7, 75·3)	79·1 (76·0, 82·0)	83·5 (79·1, 87·4)	66·1 (65·0, 67·1)	74·5 (72·3, 76·6)	81·4 (79·3, 83·3)	85·9 (83·1, 88·2)	66·4 (65·6, 67·3)	73·8 (72·1, 75·4)	80·3 (77·8, 82·6)	84·7 (81·2, 87·6)
Oman	67·4 (64·8, 70·1)	75·4 (73·2, 77·6)	81·1 (78·3, 83·8)	83·8 (79·6, 87·9)	72·1 (69·7, 74·4)	79·4 (78·1, 81·2)	84·2 (82·5, 86·0)	86·3 (83·8, 88·9)	69·3 (67·3, 71·3)	77·0 (75·6, 78·5)	82·2 (79·7, 84·6)	85·0 (81·6, 88·2)
Palestine	69·0 (67·1, 71·1)	76·1 (75·3, 76·9)	79·0 (73·1, 82·0)	82·5 (76·0, 86·2)	72·3 (70·4, 74·1)	77·5 (76·8, 78·2)	80·0 (76·7, 82·6)	83·0 (78·8, 86·6)	70·7 (69·3, 72·1)	76·7 (76·2, 77·2)	79·5 (75·1, 82·2)	82·8 (77·6, 86·4)
Qatar	70·3 (68·8, 71·9)	79·2 (77·4, 81·0)	84·2 (81·3, 87·2)	85·6 (80·8, 90·3)	72·3 (70·8, 73·7)	81·0 (79·3, 82·7)	85·5 (83·3, 87·9)	86·8 (83·1, 90·3)	71·0 (69·9, 72·2)	79·7 (78·3, 81·1)	84·7 (81·9, 87·5)	86·2 (82·1, 90·3)
Saudi Arabia	69·9 (67·7, 72·1)	75·0 (73·5, 76·3)	78·4 (75·8, 80·8)	80·6 (75·9, 84·8)	73·1 (71·0, 75·1)	79·3 (77·9, 80·2)	82·7 (80·8, 84·3)	84·7 (81·9, 87·1)	71·2 (69·4, 72·9)	76·6 (75·4, 77·5)	80·1 (77·8, 82·2)	82·5 (78·9, 85·8)
Sudan	57·2 (55·4, 59·0)	68·6 (65·9, 71·3)	77·0 (74·5, 79·2)	82·9 (80·3, 85·3)	59·1 (57·2, 60·9)	71·8 (69·3, 74·4)	81·0 (78·6, 83·2)	86·4 (83·9, 88·7)	58·1 (56·7, 59·4)	70·0 (68·1, 72·0)	79·0 (76·6, 81·0)	84·6 (82·2, 86·8)
Syria	67·8 (66·3, 69·2)	65·0 (63·3, 66·6)	72·9 (62·0, 82·2)	76·1 (64·9, 87·2)	72·3 (71·0, 73·4)	74·7 (73·6, 76·0)	80·9 (75·5, 86·1)	83·4 (77·0, 90·0)	69·8 (68·8, 70·9)	69·4 (68·2, 70·5)	76·7 (68·9, 84·0)	79·6 (71·1, 88·6)
Tunisia	70·6 (69·9, 71·2)	75·9 (73·4, 78·2)	80·2 (77·7, 82·7)	83·3 (79·6, 86·8)	74·1 (73·6, 74·6)	80·4 (78·3, 82·6)	84·8 (81·8, 87·6)	87·7 (83·2, 91·8)	72·2 (71·7, 72·7)	78·0 (76·3, 79·7)	82·5 (80·1, 84·7)	85·3 (81·5, 88·5)
Turkey	65·5 (64·8, 66·3)	74·9 (73·9, 75·9)	80·4 (78·0, 82·8)	82·1 (77·8, 86·2)	71·9 (71·1, 72·6)	82·9 (81·9, 83·8)	87·2 (85·6, 88·8)	88·4 (85·6, 90·9)	68·5 (67·9, 69·1)	78·6 (77·8, 79·3)	83·5 (81·6, 85·3)	84·9 (81·3, 88·2)
United Arab Emirates	69·9 (67·9, 72·0)	71·3 (69·0, 73·7)	74·7 (70·7, 79·1)	77·1 (70·1, 83·8)	72·7 (70·9, 74·6)	76·3 (74·1, 78·5)	80·2 (77·4, 82·9)	82·4 (78·2, 86·2)	70·9 (69·5, 72·5)	72·8 (70·9, 74·7)	76·3 (72·7, 80·1)	79·6 (74·1, 84·4)
Yemen	57·5 (54·6, 60·2)	66·2 (62·9, 69·1)	73·4 (68·7, 76·9)	79·5 (73·3, 83·6)	59·7 (57·0, 62·4)	70·5 (66·9, 73·5)	77·1 (72·0, 80·6)	83·4 (77·0, 87·3)	58·5 (55·8, 61·2)	68·2 (64·8, 71·2)	75·2 (70·4, 78·7)	81·4 (75·0, 85·4)

Location	Males				Females				Both			
	1990	2017	2050	2100	1990	2017	2050	2100	1990	2017	2050	2100
**South Asia**	59·2 (58·8, 59·6)	68·0 (67·6, 68·4)	74·3 (72·1, 76·5)	78·5 (74·7, 81·8)	60·5 (60·1, 61·0)	70·3 (69·9, 70·7)	77·1 (74·4, 79·4)	80·6 (76·3, 84·2)	59·8 (59·4, 60·1)	69·1 (68·8, 69·4)	75·6 (73·4, 77·8)	79·5 (75·7, 82·9)
**South Asia**	59·2 (58·8, 59·6)	68·0 (67·6, 68·4)	74·3 (72·1, 76·5)	78·5 (74·7, 81·8)	60·5 (60·1, 61·0)	70·3 (69·9, 70·7)	77·1 (74·4, 79·4)	80·6 (76·3, 84·2)	59·8 (59·4, 60·1)	69·1 (68·8, 69·4)	75·6 (73·4, 77·8)	79·5 (75·7, 82·9)
Bangladesh	57·6 (56·6, 58·6)	71·5 (69·9, 73·1)	78·5 (73·6, 82·1)	82·6 (76·8, 87·2)	59·7 (58·7, 60·8)	74·2 (72·7, 75·7)	81·3 (76·7, 85·0)	84·5 (78·7, 89·2)	58·5 (57·6, 59·3)	72·8 (71·6, 73·9)	80·0 (75·4, 83·4)	83·6 (78·3, 88·1)
Bhutan	61·7 (59·3, 64·0)	72·9 (70·3, 75·2)	77·7 (75·1, 80·3)	80·3 (77·4, 83·2)	61·6 (59·3, 63·9)	76·6 (74·6, 78·6)	83·2 (81·2, 85·3)	85·7 (83·2, 88·1)	61·6 (59·7, 63·5)	74·5 (72·8, 76·3)	80·2 (77·9, 82·3)	82·9 (80·4, 85·3)
India	59·3 (58·9, 59·8)	68·0 (67·6, 68·3)	74·2 (71·8, 76·5)	78·0 (73·8, 81·7)	60·8 (60·3, 61·3)	70·3 (70·0, 70·7)	77·2 (74·2, 79·7)	80·7 (75·9, 84·8)	60·0 (59·6, 60·4)	69·1 (68·9, 69·4)	75·6 (73·2, 77·9)	79·3 (74·9, 83·1)
Nepal	57·5 (55·8, 59·3)	68·9 (67·3, 70·8)	74·9 (72·1, 78·3)	78·6 (74·7, 83·0)	58·7 (56·8, 60·6)	73·5 (71·6, 75·2)	80·2 (77·7, 82·7)	83·5 (80·0, 86·9)	58·1 (56·7, 59·4)	71·1 (69·9, 72·4)	77·8 (75·3, 80·6)	81·0 (77·7, 84·7)
Pakistan	60·8 (59·8, 61·8)	65·9 (63·3, 68·6)	72·9 (69·5, 75·9)	79·2 (75·4, 82·6)	60·6 (59·8, 61·6)	66·9 (64·5, 69·6)	73·7 (70·0, 77·2)	79·0 (74·1, 83·3)	60·7 (59·9, 61·5)	66·4 (64·4, 68·4)	73·3 (70·0, 76·4)	79·1 (75·0, 82·8)
**Southeast Asia, East Asia, and Oceania**	65·9 (65·6, 66·2)	73·0 (72·6, 73·3)	77·1 (75·4, 79·1)	80·1 (77·2, 83·0)	69·9 (69·6, 70·1)	78·6 (78·2, 78·9)	82·4 (80·7, 84·0)	84·0 (81·4, 86·5)	67·8 (67·6, 68·0)	75·6 (75·4, 75·9)	79·7 (78·2, 81·2)	82·0 (79·6, 84·4)
**East Asia**	67·1 (66·7, 67·4)	74·6 (74·2, 75·1)	78·9 (76·7, 81·4)	82·5 (77·8, 86·7)	70·6 (70·3, 71·0)	79·9 (79·5, 80·3)	83·9 (82·1, 85·7)	85·9 (82·9, 88·8)	68·8 (68·5, 69·0)	77·1 (76·8, 77·4)	81·3 (79·8, 83·1)	84·1 (80·9, 87·3)
China	67·0 (66·6, 67·3)	74·7 (74·2, 75·1)	79·1 (76·8, 81·6)	82·6 (77·9, 86·9)	70·5 (70·2, 70·8)	80·0 (79·6, 80·4)	84·0 (82·2, 85·9)	86·0 (82·9, 89·0)	68·6 (68·4, 68·9)	77·2 (76·8, 77·5)	81·5 (79·8, 83·3)	84·2 (80·9, 87·5)
North Korea	68·8 (66·9, 71·1)	68·5 (66·7, 70·2)	72·4 (69·7, 75·0)	78·3 (74·0, 82·2)	74·5 (72·3, 76·7)	74·8 (72·7, 77·0)	78·8 (75·8, 81·5)	83·7 (79·8, 87·1)	72·0 (70·4, 73·5)	71·8 (70·4, 73·3)	75·5 (72·8, 77·9)	81·0 (77·0, 84·4)
Taiwan (province of China)	72·0 (71·9, 72·1)	76·6 (75·9, 77·2)	78·7 (76·7, 80·6)	80·6 (77·3, 84·0)	77·1 (77·0, 77·2)	83·0 (82·4, 83·6)	85·0 (83·4, 86·6)	85·6 (82·8, 88·2)	74·3 (74·2, 74·4)	79·7 (79·2, 80·1)	81·8 (80·2, 83·3)	83·0 (80·2, 85·7)
**Oceania**	55·7 (53·8, 57·7)	58·7 (56·3, 61·1)	65·9 (63·1, 68·2)	72·7 (69·8, 75·3)	61·0 (59·2, 62·9)	63·8 (61·4, 66·0)	71·2 (68·5, 73·6)	77·6 (74·3, 80·3)	58·1 (56·7, 59·5)	61·0 (59·1, 62·7)	68·4 (65·9, 70·7)	75·1 (72·0, 77·7)
American Samoa	67·7 (67·0, 68·4)	70·1 (68·7, 71·8)	73·3 (71·2, 75·4)	74·3 (71·7, 77·0)	75·0 (74·2, 75·8)	73·9 (73·0, 75·0)	75·5 (74·1, 76·9)	76·8 (74·7, 78·8)	70·9 (70·3, 71·4)	71·9 (71·0, 73·0)	74·4 (72·8, 76·0)	75·5 (73·2, 77·7)
Federated States of Micronesia	61·8 (59·3, 64·2)	65·0 (62·8, 67·3)	69·9 (66·3, 72·8)	73·6 (69·4, 76·6)	65·7 (63·5, 67·8)	69·7 (67·2, 71·9)	74·7 (71·4, 77·7)	77·9 (74·1, 81·3)	63·6 (61·9, 65·2)	67·3 (65·6, 68·9)	72·2 (68·9, 75·1)	75·7 (71·8, 78·8)

Location	Males				Females				Both			
	1990	2017	2050	2100	1990	2017	2050	2100	1990	2017	2050	2100
Fiji	65·3 (63·8, 66·8)	66·0 (64·1, 67·9)	70·2 (67·5, 72·6)	74·3 (70·0, 78·2)	69·9 (68·5, 71·4)	70·5 (68·4, 72·6)	74·6 (71·9, 77·2)	78·6 (74·5, 82·4)	67·5 (66·2, 68·6)	68·2 (66·7, 69·6)	72·3 (69·9, 74·7)	76·4 (72·3, 80·0)
Guam	70·8 (70·3, 71·3)	70·4 (69·3, 71·5)	73·4 (70·8, 75·6)	76·4 (72·3, 80·1)	76·7 (76·2, 77·3)	76·3 (75·2, 77·3)	78·6 (76·5, 80·5)	80·9 (77·6, 83·8)	73·5 (73·1, 73·9)	73·1 (72·3, 73·9)	75·9 (73·8, 77·8)	78·6 (75·2, 81·7)
Kiribati	55·7 (54·3, 57·1)	58·9 (56·4, 61·4)	65·2 (62·6, 67·7)	71·4 (68·1, 74·6)	61·4 (60·0, 62·6)	66·6 (64·1, 69·1)	73·1 (70·4, 76·0)	77·7 (74·3, 81·2)	58·5 (57·3, 59·6)	62·7 (60·8, 64·4)	69·1 (66·5, 71·7)	74·5 (71·3, 77·6)
Marshall Islands	59·9 (59·2, 60·7)	62·7 (60·6, 64·8)	67·1 (63·6, 70·5)	71·7 (66·2, 77·0)	66·3 (65·6, 67·0)	67·0 (64·7, 69·2)	71·2 (68·4, 74·0)	75·7 (71·3, 79·7)	62·8 (62·2, 63·4)	64·7 (63·0, 66·3)	69·1 (66·1, 72·0)	73·6 (68·8, 78·1)
Northern Mariana Islands	73·1 (70·8, 74·7)	73·8 (72·5, 75·2)	76·5 (74·6, 78·2)	78·0 (75·5, 80·4)	75·9 (74·3, 77·8)	79·3 (78·1, 80·3)	82·7 (81·2, 84·2)	83·6 (81·4, 85·7)	74·2 (72·7, 75·5)	76·3 (75·4, 77·4)	79·4 (77·8, 80·7)	80·7 (78·5, 82·6)
Papua New Guinea	52·5 (50·1, 55·0)	56·8 (53·9, 59·8)	64·9 (61·7, 67·6)	72·4 (69·1, 75·2)	57·8 (55·4, 60·3)	61·7 (58·9, 64·6)	70·3 (67·2, 73·1)	77·3 (73·7, 80·3)	54·9 (53·1, 56·7)	59·0 (56·8, 61·1)	67·5 (64·6, 70·0)	74·8 (71·5, 77·5)
Samoa	68·3 (66·1, 70·3)	71·4 (70·1, 72·9)	75·1 (72·8, 77·1)	77·9 (74·5, 80·5)	74·0 (72·0, 76·0)	74·7 (73·0, 76·8)	77·7 (75·0, 80·5)	80·6 (76·7, 84·3)	70·9 (69·3, 72·4)	73·0 (71·9, 74·2)	76·3 (74·0, 78·6)	79·1 (75·5, 82·1)
Solomon Islands	60·1 (57·3, 62·8)	64·3 (62·0, 66·5)	69·9 (66·6, 72·4)	74·7 (71·4, 77·5)	63·6 (61·2, 66·0)	67·6 (65·4, 69·6)	73·9 (71·3, 76·3)	79·1 (76·2, 81·6)	61·7 (59·7, 63·5)	65·9 (64·2, 67·5)	71·9 (68·9, 74·1)	76·8 (73·6, 79·3)
Tonga	68·3 (67·5, 69·2)	68·6 (66·8, 70·1)	71·6 (69·4, 73·5)	74·5 (71·3, 77·0)	72·0 (71·0, 73·0)	75·0 (73·2, 77·0)	78·6 (76·8, 80·5)	80·8 (78·3, 83·1)	70·1 (69·3, 70·8)	71·7 (70·3, 73·0)	75·0 (73·1, 76·8)	77·5 (74·9, 79·8)
Vanuatu	59·9 (56·8, 63·0)	62·3 (58·5, 65·6)	67·6 (64·2, 70·1)	72·7 (69·4, 75·3)	65·9 (63·0, 68·5)	67·9 (64·6, 70·7)	73·3 (70·4, 75·6)	78·1 (75·4, 80·6)	62·5 (59·4, 65·4)	64·8 (61·2, 67·9)	70·4 (67·3, 72·9)	75·4 (72·2, 77·8)
**Southeast Asia**	62·7 (62·2, 63·2)	69·4 (68·8, 69·9)	74·5 (72·9, 76·1)	77·8 (75·6, 79·9)	68·0 (67·6, 68·4)	75·7 (75·1, 76·2)	80·1 (78·3, 81·9)	82·4 (79·8, 84·9)	65·3 (64·9, 65·6)	72·5 (72·0, 72·8)	77·3 (75·7, 78·9)	80·1 (77·8, 82·2)
Cambodia	55·7 (54·3, 57·1)	66·8 (65·1, 68·3)	74·9 (72·9, 77·0)	80·1 (77·6, 82·7)	60·1 (58·8, 61·4)	72·8 (70·8, 74·4)	80·3 (77·7, 82·7)	84·1 (81·2, 86·7)	58·0 (57·0, 59·0)	69·9 (68·6, 71·1)	77·6 (75·4, 79·8)	82·1 (79·6, 84·4)
Indonesia	62·6 (62·1, 63·2)	69·1 (68·4, 69·9)	73·7 (72·2, 75·1)	77·3 (75·2, 78·9)	65·7 (65·1, 66·2)	73·9 (73·1, 74·6)	78·9 (77·2, 80·5)	81·8 (79·5, 83·8)	64·2 (63·8, 64·5)	71·4 (70·9, 72·0)	76·3 (74·7, 77·6)	79·5 (77·5, 81·1)
Laos	50·4 (47·8, 52·8)	65·0 (62·8, 67·1)	74·8 (71·8, 77·4)	80·4 (77·1, 83·2)	55·0 (52·6, 57·4)	70·3 (68·1, 72·3)	79·6 (77·1, 81·8)	84·5 (81·8, 86·9)	52·6 (50·6, 54·6)	67·5 (65·9, 69·0)	77·2 (74·6, 79·4)	82·4 (79·6, 84·8)
Malaysia	69·2 (69·1, 69·3)	72·5 (71·4, 73·6)	77·0 (74·3, 79·8)	79·3 (74·2, 84·2)	73·7 (73·6, 73·7)	77·2 (76·2, 78·2)	80·6 (78·6, 82·6)	82·2 (78·9, 85·5)	71·3 (71·3, 71·4)	74·7 (73·9, 75·5)	78·7 (76·7, 80·8)	80·7 (77·3, 84·2)

Location	Males				Females				Both			
	1990	2017	2050	2100	1990	2017	2050	2100	1990	2017	2050	2100
Maldives	66·7 (66·1, 67·2)	79·4 (78·8, 80·1)	85·0 (82·6, 87·2)	87·4 (83·2, 91·1)	65·6 (65·1, 66·1)	82·9 (82·1, 83·7)	88·7 (86·1, 91·3)	90·4 (86·3, 94·3)	66·2 (65·7, 66·7)	81·0 (80·5, 81·5)	86·6 (84·3, 88·7)	88·9 (85·0, 92·6)
Mauritius	66·3 (66·1, 66·6)	71·6 (70·7, 72·5)	76·2 (73·6, 78·8)	79·7 (75·1, 84·2)	74·2 (73·9, 74·5)	78·1 (77·2, 79·0)	82·1 (79·6, 84·5)	84·7 (80·4, 89·1)	70·1 (69·9, 70·3)	74·8 (74·1, 75·4)	79·1 (77·0, 81·3)	82·2 (78·4, 86·1)
Myanmar	53·1 (50·4, 55·6)	65·0 (63·2, 66·9)	73·2 (71·1, 75·3)	77·4 (74·7, 79·5)	58·8 (56·5, 61·3)	72·2 (70·3, 74·2)	79·5 (77·0, 81·7)	82·9 (79·2, 85·8)	55·8 (53·6, 57·7)	68·6 (67·1, 70·1)	76·4 (74·6, 78·3)	80·1 (77·8, 82·2)
Philippines	64·6 (63·7, 65·5)	66·6 (64·5, 68·7)	71·9 (69·1, 74·9)	76·8 (73·1, 80·8)	71·4 (70·6, 72·2)	73·2 (71·2, 75·0)	77·1 (74·0, 80·2)	81·0 (76·7, 85·4)	67·9 (67·2, 68·5)	69·8 (68·3, 71·2)	74·4 (71·6, 77·4)	78·8 (75·0, 83·0)
Sri Lanka	65·5 (65·2, 65·9)	73·9 (71·6, 76·0)	78·0 (73·4, 81·9)	81·3 (74·5, 87·0)	74·5 (74·2, 74·9)	81·1 (79·4, 83·4)	85·1 (81·5, 88·5)	87·2 (82·0, 92·0)	69·5 (69·3, 69·8)	77·4 (75·9, 79·0)	81·5 (77·9, 84·8)	84·1 (78·3, 89·1)
Seychelles	66·1 (65·7, 66·5)	70·1 (69·5, 70·8)	74·6 (72·2, 76·7)	77·6 (73·4, 81·2)	75·5 (75·0, 76·0)	77·6 (76·9, 78·3)	80·0 (78·3, 81·7)	82·1 (79·4, 84·8)	70·5 (70·1, 70·8)	73·6 (73·0, 74·1)	77·1 (75·2, 78·8)	79·8 (76·7, 82·9)
Thailand	67·4 (66·7, 68·1)	73·9 (72·5, 75·3)	79·3 (76·5, 82·0)	81·3 (76·6, 85·7)	74·3 (73·7, 74·9)	81·3 (80·2, 82·4)	85·2 (83·0, 87·4)	86·3 (82·8, 89·7)	70·8 (70·2, 71·3)	77·5 (76·6, 78·5)	82·2 (80·1, 84·4)	83·8 (80·0, 87·4)
Timor-Leste	59·5 (57·8, 61·2)	69·1 (67·5, 71·1)	75·7 (71·1, 79·0)	80·2 (74·6, 84·2)	60·6 (58·5, 62·6)	73·3 (71·5, 75·0)	80·4 (76·5, 83·4)	83·5 (79·1, 87·5)	60·0 (58·5, 61·4)	71·1 (69·8, 72·4)	78·0 (74·0, 81·1)	81·8 (77·0, 85·6)
Vietnam	65·1 (63·7, 66·7)	70·0 (68·3, 71·3)	75·3 (72·7, 77·7)	78·6 (74·8, 81·4)	72·8 (71·4, 74·4)	79·2 (77·9, 81·0)	82·5 (80·2, 84·8)	84·4 (81·7, 87·0)	69·0 (67·9, 70·1)	74·5 (73·4, 75·6)	78·9 (76·5, 81·0)	81·4 (78·4, 83·9)
**Sub-Saharan Africa**	51·6 (51·0, 52·2)	61·7 (60·9, 62·4)	71·0 (69·1, 72·8)	76·6 (74·1, 78·8)	55·7 (55·1, 56·2)	66·3 (65·5, 67·0)	76·5 (74·4, 78·4)	81·5 (78·9, 84·0)	53·6 (53·0, 54·1)	64·0 (63·2, 64·6)	73·8 (71·8, 75·5)	79·0 (76·5, 81·2)
**Central sub-Saharan Africa**	50·2 (48·9, 51·6)	60·0 (58·2, 61·6)	68·8 (65·5, 71·7)	74·4 (70·0, 77·8)	54·7 (53·4, 56·1)	64·1 (62·3, 65·8)	74·4 (70·8, 77·7)	80·1 (75·8, 84·0)	52·4 (51·4, 53·4)	62·0 (60·7, 63·2)	71·6 (68·1, 74·7)	77·2 (72·9, 80·8)
Angola	45·8 (43·5, 47·9)	61·8 (59·5, 64·1)	70·5 (67·5, 73·5)	75·4 (71·6, 78·8)	50·7 (48·4, 53·0)	66·8 (64·4, 69·1)	76·9 (73·6, 80·0)	81·5 (77·8, 84·9)	48·0 (46·2, 49·9)	64·2 (62·4, 66·1)	73·8 (70·6, 76·5)	78·4 (74·8, 81·5)
Central African Republic	45·4 (43·5, 47·2)	46·9 (43·1, 50·2)	57·5 (51·3, 62·7)	66·6 (59·0, 72·5)	50·9 (49·0, 52·7)	52·6 (49·0, 56·3)	63·6 (56·3, 69·8)	72·2 (63·3, 79·2)	48·0 (46·3, 49·6)	49·5 (46·4, 52·4)	60·5 (53·9, 66·0)	69·4 (61·4, 76·0)
Congo (Brazzaville)	51·3 (49·2, 53·3)	62·9 (60·5, 65·2)	71·5 (67·1, 75·5)	75·7 (68·5, 81·9)	56·0 (54·1, 58·1)	63·0 (60·3, 65·9)	72·2 (67·2, 77·2)	77·2 (69·5, 85·2)	53·6 (51·9, 55·3)	62·9 (60·8, 64·8)	71·8 (67·4, 75·9)	76·4 (69·1, 82·7)
Democratic Republic of the Congo	51·9 (50·0, 53·9)	60·0 (57·5, 62·3)	68·4 (64·0, 72·3)	74·1 (68·6, 78·3)	56·1 (54·2, 57·9)	64·0 (61·5, 66·3)	73·9 (69·2, 78·1)	79·8 (74·1, 84·3)	53·9 (52·5, 55·4)	62·0 (60·1, 63·8)	71·1 (66·5, 75·0)	76·9 (71·4, 81·2)

Location	Males				Females				Both			
	1990	2017	2050	2100	1990	2017	2050	2100	1990	2017	2050	2100
Equatorial Guinea	46·6 (43·5, 49·7)	65·0 (60·6, 68·8)	75·7 (72·1, 79·1)	78·7 (73·5, 83·5)	51·5 (48·7, 54·3)	67·1 (62·2, 72·0)	78·8 (74·5, 82·5)	81·7 (76·9, 85·9)	49·0 (46·1, 52·0)	66·2 (61·4, 70·4)	77·3 (73·6, 80·7)	80·2 (75·4, 84·5)
Gabon	56·7 (55·1, 58·3)	64·5 (62·6, 66·4)	70·8 (68·5, 72·8)	74·6 (71·8, 77·3)	64·4 (62·7, 65·9)	71·7 (69·5, 74·0)	78·2 (75·4, 81·0)	81·4 (77·2, 85·4)	60·4 (59·1, 61·6)	67·9 (66·3, 69·4)	74·5 (72·2, 76·9)	78·0 (74·9, 80·9)
**Eastern sub-Saharan Africa**	49·1 (48·5, 49·8)	62·7 (62·1, 63·2)	71·1 (69·3, 72·9)	76·1 (73·5, 78·4)	53·3 (52·8, 53·8)	67·6 (67·1, 68·3)	77·2 (75·5, 78·8)	81·9 (79·5, 83·9)	51·2 (50·7, 51·7)	65·1 (64·6, 65·6)	74·2 (72·4, 75·8)	79·0 (76·7, 81·1)
Burundi	47·5 (45·5, 49·5)	60·1 (57·6, 62·7)	68·3 (59·7, 73·5)	75·4 (64·9, 82·3)	51·2 (49·3, 53·2)	64·0 (61·5, 66·4)	72·2 (66·0, 77·0)	79·3 (69·2, 86·1)	49·3 (47·8, 50·9)	61·9 (59·9, 63·7)	70·2 (63·0, 75·2)	77·4 (66·9, 84·2)
Comoros	57·2 (55·0, 59·3)	67·7 (65·6, 69·9)	74·1 (70·0, 77·4)	77·7 (72·4, 81·6)	59·8 (57·6, 62·1)	70·7 (68·4, 72·9)	77·3 (73·0, 80·8)	81·1 (75·5, 85·0)	58·4 (56·7, 60·2)	69·2 (67·5, 70·9)	75·7 (71·4, 78·9)	79·3 (74·0, 83·1)
Djibouti	60·7 (57·5, 63·7)	67·1 (62·9, 70·7)	73·5 (70·2, 76·7)	77·6 (73·3, 81·5)	62·7 (59·6, 65·7)	69·8 (65·2, 73·5)	77·6 (74·1, 81·0)	81·2 (76·7, 85·2)	61·6 (58·5, 64·7)	68·3 (64·0, 71·9)	75·5 (72·3, 78·3)	79·3 (75·3, 82·9)
Eritrea	29·2 (28·3, 30·2)	58·1 (55·2, 61·0)	67·0 (59·4, 72·7)	71·9 (61·7, 78·6)	41·8 (40·5, 43·1)	64·7 (62·1, 67·7)	72·6 (66·3, 77·8)	77·2 (68·7, 83·3)	34·4 (33·5, 35·4)	61·4 (59·1, 63·7)	69·8 (62·7, 74·7)	74·5 (65·6, 80·5)
Ethiopia	46·9 (46·0, 47·8)	66·8 (65·7, 67·7)	76·3 (74·1, 78·1)	80·1 (77·0, 82·4)	50·4 (49·5, 51·4)	70·8 (69·8, 71·8)	80·9 (78·9, 82·8)	85·0 (82·5, 87·1)	48·6 (47·9, 49·3)	68·6 (67·8, 69·4)	78·6 (76·6, 80·4)	82·5 (79·9, 84·6)
Kenya	61·0 (60·4, 61·6)	63·3 (62·8, 63·9)	69·5 (67·3, 71·5)	73·7 (70·4, 76·6)	63·6 (63·0, 64·3)	68·9 (68·2, 69·5)	77·6 (75·7, 79·5)	81·2 (78·3, 83·8)	62·3 (61·8, 62·8)	66·0 (65·5, 66·4)	73·5 (71·5, 75·4)	77·4 (74·4, 80·0)
Madagascar	54·0 (52·8, 55·3)	62·4 (59·4, 65·4)	69·3 (64·5, 73·9)	76·0 (69·2, 82·4)	57·0 (55·8, 58·2)	64·7 (62·0, 67·6)	71·8 (66·3, 76·2)	78·5 (71·3, 83·4)	55·5 (54·4, 56·5)	63·5 (61·2, 65·7)	70·5 (65·6, 74·7)	77·3 (70·8, 82·7)
Malawi	46·7 (44·6, 48·4)	59·8 (57·8, 61·6)	69·5 (66·3, 72·5)	74·5 (70·4, 78·2)	50·5 (48·9, 52·0)	67·2 (65·0, 69·2)	79·1 (75·2, 82·4)	83·6 (78·7, 87·5)	48·6 (46·9, 50·0)	63·4 (61·7, 64·8)	74·4 (71·0, 77·3)	79·0 (74·4, 82·5)
Mozambique	47·6 (46·2, 49·2)	54·9 (52·5, 57·1)	64·5 (60·7, 68·3)	70·7 (65·8, 75·0)	52·2 (50·7, 53·7)	62·2 (59·6, 64·8)	73·4 (69·7, 77·1)	79·0 (75·0, 83·2)	49·9 (48·6, 51·1)	58·5 (56·4, 60·3)	69·2 (65·6, 72·6)	74·8 (70·5, 78·5)
Rwanda	47·9 (46·6, 49·3)	65·8 (63·9, 67·7)	75·4 (71·4, 79·3)	79·6 (74·6, 84·1)	51·8 (50·5, 53·2)	70·9 (68·9, 72·8)	80·7 (77·3, 84·2)	85·2 (81·1, 88·8)	49·8 (48·8, 50·9)	68·5 (67·0, 69·9)	78·1 (74·6, 81·8)	82·4 (78·0, 86·0)
Somalia	46·1 (43·3, 49·1)	57·7 (53·1, 61·8)	64·0 (59·6, 68·1)	70·4 (61·2, 77·7)	50·2 (47·5, 52·9)	61·7 (57·4, 65·5)	67·9 (63·6, 72·0)	75·6 (66·4, 83·4)	48·0 (45·3, 50·9)	59·6 (55·1, 63·5)	65·8 (61·6, 70·0)	72·9 (63·7, 80·5)
South Sudan	50·6 (47·2, 53·9)	56·3 (52·4, 59·7)	67·5 (62·3, 72·0)	75·0 (68·6, 80·3)	54·3 (51·2, 57·5)	61·1 (57·3, 64·6)	73·2 (68·1, 77·3)	80·2 (74·2, 84·7)	52·2 (49·0, 55·4)	58·5 (54·7, 61·9)	70·3 (65·7, 74·4)	77·6 (71·9, 82·2)

Location	Males				Females				Both			
	1990	2017	2050	2100	1990	2017	2050	2100	1990	2017	2050	2100
Tanzania	53·6 (51·4, 55·5)	64·8 (63·0, 66·6)	73·5 (70·8, 75·6)	78·4 (75·4, 80·8)	56·9 (55·3, 58·5)	69·1 (67·3, 70·9)	78·5 (75·8, 81·0)	82·9 (79·4, 85·8)	55·2 (53·6, 56·6)	66·9 (65·4, 68·3)	76·1 (73·5, 78·3)	80·6 (77·4, 83·2)
Uganda	44·4 (40·2, 48·4)	62·6 (60·6, 64·4)	69·8 (65·9, 73·3)	74·5 (70·1, 79·0)	50·9 (48·5, 53·2)	69·4 (67·3, 71·4)	78·9 (75·8, 81·5)	83·3 (79·7, 86·5)	47·5 (44·3, 50·6)	65·9 (64·3, 67·4)	74·4 (71·1, 77·3)	78·8 (75·0, 82·3)
Zambia	49·4 (46·5, 51·4)	60·7 (58·7, 62·6)	70·2 (67·3, 73·0)	74·5 (70·6, 78·2)	52·2 (50·2, 53·8)	66·6 (64·5, 68·8)	77·7 (74·3, 81·2)	81·6 (76·9, 86·3)	50·8 (48·5, 52·4)	63·5 (61·9, 65·0)	73·9 (71·0, 76·7)	78·0 (74·0, 81·9)
**Southern sub-Saharan Africa**	60·4 (59·4, 61·2)	61·8 (61·1, 62·4)	69·5 (65·8, 73·0)	73·2 (66·4, 78·7)	68·0 (67·3, 68·7)	68·7 (67·9, 69·5)	76·8 (72·6, 81·3)	80·1 (72·4, 86·7)	64·1 (63·3, 64·8)	65·3 (64·7, 65·9)	73·2 (69·4, 76·7)	76·6 (69·8, 82·3)
Botswana	58·9 (55·4, 61·6)	67·1 (64·2, 69·2)	76·4 (72·6, 79·0)	78·7 (74·4, 82·1)	69·1 (66·3, 71·4)	71·1 (68·8, 72·6)	77·3 (72·8, 81·6)	80·1 (73·2, 86·4)	63·8 (60·9, 65·9)	69·1 (67·4, 70·4)	76·8 (73·6, 79·8)	79·4 (74·2, 83·8)
eSwatini	58·0 (56·3, 59·8)	55·3 (52·8, 57·9)	62·9 (58·1, 67·5)	68·0 (61·5, 73·9)	66·6 (65·0, 68·4)	65·3 (62·3, 68·4)	74·7 (69·1, 79·5)	78·7 (71·2, 85·1)	62·2 (60·9, 63·5)	60·1 (58·0, 62·0)	68·8 (63·9, 73·4)	73·3 (66·5, 79·2)
Lesotho	56·5 (54·6, 58·2)	51·2 (48·7, 53·6)	58·1 (53·2, 63·0)	64·8 (58·1, 71·0)	65·6 (63·8, 67·4)	60·0 (56·9, 63·2)	69·4 (63·7, 75·5)	74·9 (67·2, 83·0)	60·8 (59·3, 62·2)	55·4 (53·4, 57·6)	63·7 (58·8, 68·8)	69·9 (63·0, 76·3)
Namibia	58·4 (56·3, 59·9)	62·4 (60·4, 64·3)	71·3 (67·3, 74·8)	74·9 (69·8, 79·5)	65·6 (64·1, 67·0)	71·0 (67·9, 73·7)	80·9 (76·0, 85·1)	83·7 (77·4, 89·3)	61·9 (60·2, 63·0)	66·6 (64·7, 68·4)	76·1 (72·1, 79·7)	79·2 (73·9, 83·9)
South Africa	61·5 (60·8, 62·1)	63·1 (62·3, 63·8)	72·0 (67·8, 75·8)	75·1 (67·7, 81·7)	68·8 (68·2, 69·4)	70·0 (68·9, 70·8)	79·3 (74·2, 84·4)	82·3 (72·8, 90·4)	65·2 (64·6, 65·7)	66·6 (65·8, 67·2)	75·6 (71·4, 79·6)	78·6 (71·1, 85·4)
Zimbabwe	57·8 (52·9, 60·9)	58·5 (56·5, 60·6)	63·4 (56·7, 69·4)	69·9 (57·5, 78·8)	65·1 (61·5, 67·5)	64·7 (62·4, 67·1)	70·0 (61·8, 76·9)	76·2 (63·1, 85·9)	61·2 (57·1, 63·7)	61·7 (60·1, 63·3)	66·8 (59·3, 73·1)	73·0 (60·2, 82·0)
**Western sub-Saharan Africa**	52·7 (51·4, 54·0)	61·8 (60·1, 63·2)	72·0 (69·5, 74·3)	77·8 (74·5, 80·5)	55·8 (54·5, 57·0)	65·5 (63·7, 67·0)	76·4 (73·8, 79·0)	81·7 (78·4, 84·7)	54·2 (52·9, 55·4)	63·6 (61·9, 65·0)	74·3 (71·7, 76·6)	79·8 (76·7, 82·5)
Benin	54·3 (52·8, 55·8)	62·9 (59·9, 65·5)	72·7 (69·5, 75·6)	77·5 (73·8, 80·6)	58·6 (57·1, 60·1)	66·8 (64·1, 69·4)	77·2 (74·3, 80·0)	82·0 (78·9, 85·1)	56·4 (55·2, 57·5)	64·8 (62·6, 66·9)	75·0 (72·3, 77·8)	79·7 (76·5, 82·6)
Burkina Faso	49·8 (48·1, 51·4)	59·1 (56·5, 61·5)	69·1 (66·0, 72·0)	75·8 (72·4, 79·0)	53·0 (51·6, 54·4)	64·6 (62·3, 66·6)	75·4 (72·8, 77·9)	81·6 (78·8, 84·3)	51·4 (50·1, 52·7)	61·8 (59·6, 63·6)	72·3 (69·6, 74·9)	78·7 (75·8, 81·4)
Cameroon	56·0 (54·8, 57·4)	61·7 (59·2, 64·2)	70·4 (67·3, 73·6)	74·4 (70·6, 78·2)	58·9 (57·6, 60·2)	65·7 (63·0, 68·5)	76·0 (72·6, 79·2)	79·9 (76·2, 83·5)	57·4 (56·4, 58·4)	63·6 (61·7, 65·6)	73·2 (70·1, 76·2)	77·1 (73·4, 80·7)
Cape Verde	66·3 (65·5, 67·0)	72·3 (70·9, 73·6)	77·3 (75·0, 79·5)	80·3 (76·6, 83·5)	73·3 (72·7, 74·0)	78·7 (77·7, 79·8)	82·7 (81·0, 84·4)	84·8 (82·4, 87·3)	70·1 (69·4, 70·7)	75·5 (74·4, 76·5)	79·9 (78·1, 81·8)	82·6 (79·8, 85·4)

Location	Males				Females				Both			
	1990	2017	2050	2100	1990	2017	2050	2100	1990	2017	2050	2100
Chad	51·5 (49·8, 53·1)	58·8 (56·4, 61·2)	68·9 (64·8, 72·4)	75·7 (70·5, 80·2)	54·1 (52·6, 55·6)	61·7 (59·3, 64·1)	72·9 (68·7, 76·5)	80·1 (74·7, 84·8)	52·8 (51·4, 54·1)	60·2 (58·1, 62·1)	70·9 (67·0, 74·4)	77·9 (72·9, 82·3)
Côte d'Ivoire	53·1 (49·4, 55·4)	60·6 (58·3, 62·8)	71·0 (67·8, 73·9)	77·1 (72·9, 80·6)	58·7 (56·3, 60·7)	65·6 (63·3, 68·0)	76·2 (73·1, 79·1)	81·8 (78·2, 85·2)	55·6 (52·6, 57·4)	62·9 (61·0, 64·7)	73·5 (70·3, 76·2)	79·4 (75·6, 82·8)
The Gambia	58·1 (55·7, 60·4)	63·8 (61·8, 65·8)	71·3 (69·2, 73·5)	76·2 (73·8, 78·6)	62·8 (60·5, 65·1)	67·9 (65·3, 70·2)	75·7 (73·1, 78·2)	80·4 (77·5, 83·1)	60·2 (58·3, 62·1)	65·7 (63·8, 67·3)	73·5 (71·3, 75·7)	78·3 (75·8, 80·6)
Ghana	58·3 (56·2, 60·3)	63·0 (61·3, 64·8)	69·2 (66·6, 71·8)	73·6 (70·4, 76·8)	61·1 (59·2, 63·1)	68·8 (66·9, 70·7)	77·1 (74·6, 79·6)	81·3 (78·4, 84·1)	59·6 (58·2, 61·1)	65·9 (64·5, 67·2)	73·2 (70·7, 75·6)	77·3 (74·5, 80·1)
Guinea	51·4 (50·0, 53·0)	59·6 (57·5, 61·6)	67·8 (65·0, 70·4)	74·4 (71·0, 77·5)	51·7 (50·3, 53·2)	62·4 (60·3, 64·2)	73·0 (70·5, 75·3)	79·8 (77·0, 82·4)	51·5 (50·3, 52·8)	60·9 (59·1, 62·5)	70·5 (68·1, 72·8)	77·1 (74·2, 79·6)
Guinea-Bissau	45·7 (43·1, 48·2)	57·3 (54·8, 59·9)	67·8 (64·7, 70·5)	74·6 (71·2, 77·7)	52·0 (49·7, 54·4)	62·5 (60·2, 64·8)	73·4 (70·3, 76·3)	79·5 (75·9, 82·8)	48·7 (46·7, 50·7)	59·9 (57·9, 61·8)	70·7 (67·7, 73·3)	77·0 (73·6, 80·0)
Liberia	43·3 (41·7, 44·9)	64·3 (61·8, 66·6)	72·7 (66·8, 76·9)	77·8 (70·2, 82·9)	48·0 (46·2, 49·8)	65·6 (63·3, 67·9)	74·9 (69·3, 79·1)	80·4 (73·9, 85·1)	45·6 (44·1, 47·0)	64·9 (63·0, 66·8)	73·8 (68·3, 78·0)	79·1 (72·7, 83·9)
Mali	48·3 (47·0, 49·7)	60·8 (58·2, 63·4)	72·8 (69·7, 75·5)	79·3 (76·1, 82·2)	48·5 (47·4, 49·7)	62·8 (60·6, 65·2)	76·3 (73·2, 79·3)	83·5 (80·3, 86·5)	48·4 (47·3, 49·4)	61·8 (59·7, 63·8)	74·6 (71·5, 77·3)	81·4 (78·5, 84·1)
Mauritania	59·1 (57·8, 60·6)	70·4 (68·2, 72·7)	77·3 (74·9, 79·5)	80·4 (77·1, 83·5)	60·3 (58·8, 61·7)	71·3 (69·1, 73·3)	79·2 (76·8, 81·6)	82·6 (79·4, 85·8)	59·7 (58·6, 60·8)	70·8 (69·3, 72·5)	78·3 (76·1, 80·5)	81·5 (78·4, 84·4)
Niger	45·8 (44·1, 47·5)	61·8 (59·1, 64·1)	72·0 (68·8, 75·0)	78·2 (73·9, 81·9)	47·4 (45·6, 48·9)	64·2 (61·9, 66·7)	76·0 (72·4, 79·4)	83·1 (78·7, 86·9)	46·5 (45·0, 47·9)	63·0 (60·8, 64·9)	74·0 (70·7, 77·0)	80·6 (76·5, 84·3)
Nigeria	52·6 (49·9, 55·3)	62·7 (58·9, 65·7)	73·8 (70·2, 76·9)	79·0 (75·0, 82·2)	56·2 (53·7, 58·8)	66·0 (62·2, 69·3)	77·2 (73·3, 81·0)	81·9 (77·6, 85·8)	54·3 (51·7, 56·9)	64·4 (60·5, 67·4)	75·6 (72·0, 78·8)	80·4 (76·6, 84·0)
São Tomé and Príncipe	62·5 (61·3, 63·7)	68·0 (66·4, 69·7)	72·2 (69·8, 74·5)	76·3 (73·1, 79·5)	65·7 (64·3, 67·0)	71·7 (69·9, 73·6)	76·7 (74·2, 79·2)	79·9 (76·4, 83·4)	64·1 (63·1, 65·1)	69·8 (68·6, 71·1)	74·4 (72·1, 76·6)	78·1 (74·9, 81·4)
Senegal	56·4 (55·0, 57·7)	66·3 (64·4, 68·1)	73·1 (70·8, 75·4)	77·4 (74·1, 80·4)	60·3 (58·9, 61·6)	70·2 (68·3, 72·1)	78·3 (75·5, 80·9)	82·8 (79·2, 86·0)	58·2 (57·2, 59·3)	68·2 (66·7, 69·6)	75·7 (73·4, 78·1)	80·0 (76·8, 83·2)
Sierra Leone	47·7 (45·8, 49·6)	59·5 (56·7, 62·1)	70·5 (66·6, 73·6)	77·0 (72·8, 80·4)	51·6 (49·6, 53·7)	61·3 (58·8, 63·7)	71·8 (68·1, 74·9)	78·5 (74·1, 82·0)	49·5 (48·0, 51·1)	60·3 (58·1, 62·5)	71·1 (67·4, 74·2)	77·8 (73·6, 81·2)
Togo	56·4 (54·7, 58·1)	61·8 (59·4, 64·2)	69·6 (66·7, 72·7)	74·8 (70·7, 78·8)	59·5 (57·8, 61·2)	67·6 (65·1, 70·0)	77·0 (73·9, 79·7)	81·4 (77·4, 84·8)	57·9 (56·6, 59·3)	64·7 (62·7, 66·6)	73·4 (70·6, 76·1)	78·1 (74·3, 81·5)

Section 4 Migration through 2100

Table 2. Net migration counts for all ages and both sexes combined: 2018, 2050, and 2100. Estimates are listed as means rounded to three significant figures, with 95% uncertainty intervals in parentheses. Positive counts indicate net immigration while negative counts indicate net emigration.

	Net migration count		
**Location**	**2018**	**2050**	**2100**
Armenia	-5000 (-28 100 to 19 200)	-1770 (-38 000 to 35 000)	-1100 (-22 400 to 19 900)
Azerbaijan	545 (-13 000 to 13 300)	-5760 (-93 500 to 81 600)	-3430 (-84 300 to 75 300)
Georgia	-9600 (-32 200 to 11 900)	-3780 (-47 200 to 44 700)	-1910 (-44 900 to 45 500)
Kazakhstan	-1600 (-109 000 to 103 000)	3480 (-287 000 to 339 000)	-326 (-397 000 to 440 000)
Kyrgyzstan	-21 900 (-52 000 to 9030)	-25 000 (-136 000 to 132 000)	-33 200 (-235 000 to 197 000)
Mongolia	-3260 (-10 300 to 3910)	-7200 (-60 000 to 52 600)	-6040 (-105 000 to 96 800)
Tajikistan	-20 800 (-51 800 to 9840)	-42 400 (-220 000 to 187 000)	-75 200 (-409 000 to 335 000)
Turkmenistan	-4220 (-14 200 to 5590)	-8640 (-73 600 to 78 200)	-8950 (-107 000 to 104 000)
Uzbekistan	-8940 (-86 200 to 67 800)	-43 100 (-489 000 to 478 000)	-36 500 (-576 000 to 522 000)
Albania	-6850 (-28 100 to 15 600)	-2070 (-38 100 to 34 200)	-462 (-27 200 to 24 300)
Bosnia and Herzegovina	-107 (-33 400 to 31 700)	-92·8 (-34 800 to 33 400)	668 (-14 100 to 14 700)
Bulgaria	-4990 (-20 100 to 8380)	-1930 (-56 100 to 53 200)	-149 (-31 400 to 32 400)
Croatia	-7790 (-19 800 to 4270)	-5670 (-40 300 to 36 700)	-1240 (-20 900 to 20 900)
Czech Republic	12 000 (-3850 to 30 200)	9890 (-84 900 to 100 000)	7390 (-64 700 to 75 100)
Hungary	5550 (-11 300 to 21 800)	5260 (-70 200 to 78 800)	4390 (-50 000 to 53 900)
Montenegro	-382 (-5300 to 4300)	45·7 (-7120 to 7630)	-17·3 (-4990 to 5170)
North Macedonia	-1130 (-8750 to 6650)	-101 (-24 900 to 23 400)	209 (-13 800 to 13 200)
Poland	-10 800 (-41 400 to 17 100)	-13600 (-175 000 to 146 000)	-1330 (-105 000 to 104 000)
Romania	-28 700 (-58 800 to 1600)	-15 400 (-156 000 to 117 000)	-4120 (-103 000 to 106 000)
Serbia	-8480 (-43 300 to 22 200)	-3880 (-90 900 to 85 100)	607 (-48 000 to 49 900)
Slovakia	1270 (-4510 to 7000)	-435 (-29 900 to 26 400)	653 (-21 900 to 20 900)
Slovenia	1200 (-3410 to 5850)	442 (-17 700 to 20 500)	930 (-11 500 to 13 700)

	Net migration count		
Location	2018	2050	2100
Belarus	2240 (-15 400 to 18 900)	4130 (-80 400 to 81 600)	5980 (-54 100 to 62 500)
Estonia	-1140 (-8270 to 5970)	291 (-13 200 to 14 700)	327 (-8410 to 9310)
Latvia	-10 200 (-17 600 to -3630)	-5960 (-20 500 to 18 800)	-2040 (-15 900 to 16 100)
Lithuania	-4460 (-13 300 to 3590)	-1800 (-25 900 to 27 400)	-63 (-17 700 to 18 600)
Moldova	-2850 (-12 900 to 7200)	-3940 (-37 800 to 29 200)	-542 (-19 600 to 16 100)
Russia	156 000 (-23 500 to 327 000)	159 000 (-747 000 to 1 080 000)	166 000 (-842 000 to 1 070 000)
Ukraine	-23 000 (-85 300 to 37 500)	-18 900 (-335 000 to 277 000)	-2000 (-212 000 to 196 000)
Australia	165 000 (85 500 to 248 000)	117 000 (-232 000 to 376 000)	64 300 (-204 000 to 275 000)
New Zealand	14 800 (-3190 to 33 200)	10 100 (-54 200 to 70 300)	6870 (-56 300 to 63 300)
Brunei	344 (-862 to 1540)	-425 (-5680 to 6440)	-228 (-5060 to 5410)
Japan	55 000 (-16 400 to 125 000)	41 500 (-28 9000 to 378 000)	45 100 (-192 000 to 291 000)
South Korea	43 300 (-28 800 to 114 000)	10 300 (-385 000 to 370 000)	13 400 (-197 000 to 216 000)
Singapore	55 700 (35 000 to 72 400)	31 700 (-61 000 to 79 500)	10 300 (-36 100 to 43 100)
Canada	214 000 (137 000 to 297 000)	163 000 (-237 000 to 490 000)	90 400 (-247 000 to 342 000)
USA	901 000 (538 000 to 1 250 000)	831 000 (-1 160 000 to 2 920 000)	577 000 (-1 730 000 to 2 520 000)
Argentina	4250 (-56 300 to 72 500)	-10 400 (-455 000 to 435 000)	-19 700 (-600 000 to 566 000)
Chile	17 100 (9250 to 25 800)	16 200 (-29 400 to 67 900)	14 200 (-42 300 to 66 100)
Uruguay	-3130 (-12 500 to 6360)	-763 (-42 400 to 43 200)	-51·1 (-36 800 to 36 000)
Austria	20 700 (3770 to 40 400)	17 700 (-70 800 to 94 500)	9230 (-48 000 to 61 200)
Belgium	47 400 (34 400 to 62 100)	41 800 (-20 000 to 10 000)	25 700 (-32 500 to 78 900)
Cyprus	5490 (-2230 to 13 700)	2550 (-12 500 to 15 500)	519 (-4680 to 5380)
Denmark	14 800 (10 400 to 20 000)	13 500 (-9860 to 39 800)	9410 (-18 300 to 37 200)
Finland	13 800 (3590 to 24 900)	12 800 (-37 600 to 58 300)	6140 (-30 000 to 38 900)
France	79 700 (-16 700 to 185 000)	65 800 (-499 000 to 614 000)	44 600 (-501 000 to 617 000)
Germany	367 000 (200 000 to 540 000)	263 000 (-455 000 to 834 000)	110 000 (-360 000 to 485 000)

	Net migration count		
Location	2018	2050	2100
Greece	9260 (-25 500 to 43 200)	3320 (-100 000 to 103 000)	3990 (-52 300 to 54 300)
Iceland	365 (-658 to 1350)	393 (-3460 to 4610)	97·3 (-2870 to 3280)
Ireland	5100 (-21 100 to 30 600)	7060 (-57 700 to 76 200)	3780 (-46 200 to 56 100)
Israel	10 700 (-56 400 to 76 300)	16 800 (-172 000 to 237 000)	20 200 (-374 000 to 445 000)
Italy	75 500 (-12 200 to 173 000)	45 300 (-408 000 to 490 000)	29 500 (-236 000 to 267 000)
Luxembourg	5010 (2490 to 7470)	2990 (-5230 to 8530)	1030 (-3400 to 4650)
Malta	955 (-334 to 2310)	864 (-4070 to 4970)	482 (-2340 to 2780)
Netherlands	15 800 (2410 to 31 200)	10 700 (-64 700 to 88 500)	7460 (-73 800 to 85 300)
Norway	27 800 (19 700 to 36800)	25900 (-15800 to 66000)	13800 (-25900 to 42000)
Portugal	-5300 (-57900 to 50 200)	-1500 (-110 000 to 105 000)	1330 (-46 800 to 47 700)
Spain	47 300 (-141 000 to 235 000)	17 600 (-458 000 to 493 000)	11 900 (-222 000 to 247 000)
Sweden	40 000 (25 200 to 56 200)	39 200 (-38 500 to 121 000)	25 700 (-67 600 to 102 000)
Switzerland	50 500 (20 300 to 83 900)	29 200 (-80 100 to 111 000)	13 800 (-53 000 to 65 800)
UK	185 000 (98 800 to 279 000)	186 000 (-319 000 to 683 000)	116 000 (-357 000 to 535 000)
Bolivia	-10 200 (-15 400 to -5330)	-11 600 (-65 700 to 36 900)	-18 100 (-151 000 to 94 700)
Ecuador	-6230 (-13 100 to 380)	-13 700 (-69 900 to 36 100)	-10 100 (-84 900 to 53 200)
Peru	-36 900 (-74 100 to -1490)	-72 500 (-466 000 to 275 000)	-110 000 (-850 000 to 592 000)
Antigua and Barbuda	-13 (-810 to 863)	20·4 (-1070 to 1170)	10·4 (-611 to 634)
The Bahamas	872 (-1910 to 3460)	107 (-4670 to 5020)	31 (-2890 to 2870)
Barbados	458 (-318 to 1250)	344 (-2820 to 2960)	144 (-1480 to 1600)
Belize	1540 (-1160 to 4080)	1040 (-6380 to 6750)	391 (-5610 to 5410)
Cuba	-21 100 (-40 900 to -2060)	-20 000 (-115 000 to 81 900)	-5990 (-71 200 to 60 200)
Dominican Republic	-29 500 (-36 600 to -23 400)	-36 800 (-73 200 to -5130)	-33 400 (-89 400 to 12 000)
Grenada	-598 (-1210 to 567)	-131 (-1530 to 1470)	-112 (-1150 to 976)
Guyana	-4590 (-8230 to -532)	-3150 (-13 500 to 11 200)	-2810 (-14 600 to 11 300)

	Net migration count		
Location	2018	2050	2100
Haiti	-41 300 (-91 800 to 1140)	-52 300 (-261 000 to 176 000)	-39 100 (-255 000 to 175 000)
Jamaica	-17 800 (-28 300 to -6090)	-12 100 (-39 400 to 31 300)	-6320 (-25 600 to 17 300)
Puerto Rico	-12 500 (-28 700 to 2710)	-4430 (-37 600 to 35 300)	-1360 (-17 000 to 15 100)
Saint Lucia	26·4 (-694 to 736)	71 (-2240 to 2020)	32 (-1240 to 1080)
Saint Vincent and the Grenadines	-604 (-973 to -258)	-409 (-1580 to 1110)	-164 (-1050 to 826)
Suriname	-1100 (-5140 to 2790)	-555 (-8700 to 8470)	-347 (-7880 to 7100)
Trinidad and Tobago	-939 (-5680 to 4080)	-804 (-18 100 to 15 900)	103 (-11 300 to 10 800)
Virgin Islands	-340 (-1160 to 1090)	-107 (-1320 to 1220)	-105 (-1280 to 1050)
Colombia	-27 400 (-51 000 to -6210)	-29 200 (-209 000 to 137 000)	-5110 (-227 000 to 195 000)
Costa Rica	3410 (-1690 to 8460)	1920 (-31 200 to 33 200)	1230 (-32 600 to 30 300)
El Salvador	-39 200 (-50 600 to -28 800)	-43 900 (-91 700 to 15 400)	-28 300 (-69 000 to 28 000)
Guatemala	-8260 (-38 900 to 21 500)	-5440 (-252 000 to 243 000)	-8320 (-304 000 to 267 000)
Honduras	-2700 (-11 600 to 6050)	-4450 (-88 500 to 70 600)	-5920 (-154 000 to 136 000)
Mexico	-55 100 (-232 000 to 110 000)	-93 600 (-1 490 000 to 1 230 000)	-53 500 (-1 650 000 to 1 560 000)
Nicaragua	-22 300 (-33 300 to -10 700)	-32 100 (-110 000 to 51 500)	-26 600 (-121 000 to 77 100)
Panama	6090 (4240 to 8150)	8920 (-4400 to 23 700)	9720 (-12 300 to 33 300)
Venezuela	-12 000 (-59 100 to 33 600)	-41 100 (-386 000 to 299 000)	-44 100 (-466 000 to 364 000)
Brazil	3940 (-64 600 to 75 200)	-5770 (-440 000 to 416 000)	52 700 (-357 000 to 462 000)
Paraguay	-16 600 (-25 300 to -8620)	-21 000 (-92 500 to 44 200)	-21 400 (-127 000 to 87 600)
Afghanistan	-48 900 (-397 000 to 315 000)	-143 000 (-1 340 000 to 992 000)	-241 000 (-2 590 000 to 1 960 000)
Algeria	-6630 (-64 000 to 47 500)	-37 300 (-523 000 to 445 000)	-18 300 (-772 000 to 794 000)
Bahrain	15 600 (12 900 to 18 800)	9200 (-19 700 to 24 400)	3370 (-14 100 to 15 000)
Egypt	-59 500 (-124 000 to -461)	-63 500 (-602 000 to 463 000)	-78 400 (-1 150 000 to 778 000)
Iran	-60 500 (-403 000 to 271 000)	-57 000 (-1 110 000 to 1 120 000)	-34 200 (-751 000 to 719 000)
Iraq	24 500 (-102 000 to 154 000)	97 100 (-742 000 to 1 030 000)	76 700 (-1 080 000 to 1 350 000)

Location	Net migration count		
	2018	2050	2100
Jordan	6460 (-94 300 to 132 000)	8030 (-216 000 to 253 000)	-18 400 (-315 000 to 312 000)
Kuwait	10 500 (-40 000 to 56 600)	6750 (-53 200 to 68 000)	2250 (-24 700 to 29 600)
Lebanon	-26 000 (-89 000 to 95 300)	-15 200 (-168 000 to 159 000)	-13 900 (-194 000 to 168 000)
Libya	-1410 (-38 200 to 34 300)	-2360 (-102 000 to 114 000)	915 (-88 300 to 91 300)
Morocco	-48 600 (-95 100 to -5830)	-45 200 (-364 000 to 235 000)	-13 300 (-296 000 to 257 000)
Oman	51 300 (42 800 to 61 500)	36 500 (-62 600 to 87 000)	16 500 (-58 600 to 61 700)
Palestine	-4170 (-52 900 to 53 200)	-3500 (-117 000 to 99 800)	-17 500 (-183 000 to 128 000)
Qatar	17 400 (-27 500 to 34 000)	6590 (-36 000 to 43 000)	2130 (-19 200 to 21 500)
Saudi Arabia	116 000 (-65 500 to 286 000)	50 900 (-399 000 to 512 000)	14 900 (-202 000 to 232 000)
Sudan	-46 600 (-175 000 to 90 800)	-9740 (-736 000 to 845 000)	-692 (-820 000 to 899 000)
Syria	-145 000 (-199 000 to 10 500)	-46 500 (-289 000 to 308 000)	-29 900 (-252 000 to 222 000)
Tunisia	-3220 (-38 100 to 29 300)	2400 (-143 000 to 158 000)	3740 (-99 200 to 105 000)
Turkey	311 000 (216 000 to 419 000)	346 000 (-192 000 to 918 000)	196 000 (-312 000 to 655 000)
United Arab Emirates	23 800 (-96 400 to 118 000)	6120 (-92 500 to 115 000)	1450 (-28 000 to 33 400)
Yemen	-28 200 (-155 000 to 109 000)	-80 300 (-748 000 to 551 000)	-132 000 (-883 000 to 512 000)
Bangladesh	-443 000 (-774 000 to -99 900)	-401 000 (-2 140 000 to 1 510 000)	-198 000 (-1 370 000 to 1 080 000)
Bhutan	-28 (-9700 to 9490)	-399 (-14 800 to 14 500)	-255 (-8740 to 8370)
India	-508 000 (-833 000 to -218 000)	238 000 (-1 680 000 to 2 300 000)	520 000 (-1 140 000 to 2 140 000)
Nepal	-72 200 (-138 000 to -8630)	-75 200 (-465 000 to 311 000)	-39 600 (-292 000 to 212 000)
Pakistan	-237 000 (-481 000 to 7100)	-300 000 (-2 460 000 to 1 620 000)	-186 000 (-2 440 000 to 1 990 000)
China	-263 000 (-846 000 to 361 000)	-280 000 (-3 390 000 to 2 690 000)	359 000 (-2 590 000 to 3 090 000)
North Korea	-2170 (-77 300 to 70 000)	-27 800 (-318 000 to 168 000)	-6720 (-122 000 to 74300)
Taiwan (province of China)	31 000 (-35 100 to 96 300)	9610 (-218 000 to 235 000)	7060 (-91 600 to 103 000)
Fiji	-4010 (-8660 to 832)	-2890 (-16 600 to 15 300)	-2240 (-21 100 to 19 000)
Guam	-515 (-1170 to 107)	-508 (-3040 to 3180)	-642 (-5890 to 5580)

	Net migration count		
Location	2018	2050	2100
Kiribati	-426 (-884 to 34·4)	-465 (-2520 to 2080)	-497 (-3120 to 2480)
Federated States of Micronesia	-882 (-1180 to -116)	-455 (-2050 to 1920)	-530 (-2770 to 2110)
Papua New Guinea	310 (-3610 to 4430)	3560 (-40 500 to 48 300)	14 500 (-87 500 to 112 000)
Samoa	-1940 (-2300 to -1610)	-3550 (-7060 to 5320)	-9200 (-26 200 to 20 600)
Solomon Islands	-2590 (-3560 to -1790)	-5440 (-13 700 to 2150)	-7160 (-21 100 to 7640)
Tonga	-801 (-1080 to -225)	-454 (-2080 to 2350)	-466 (-3640 to 3580)
Vanuatu	117 (-855 to 1100)	129 (-6190 to 5990)	-187 (-8870 to 7720)
Cambodia	-32 000 (-148 000 to 84 500)	-27 400 (-292 000 to 256 000)	-12 500 (-223 000 to 208 000)
Indonesia	-150 000 (-224 000 to -83 200)	-174 000 (-662 000 to 249 000)	-17 100 (-531 000 to 408 000)
Laos	-15 100 (-46 300 to 15 000)	-11 700 (-124 000 to 119 000)	-6020 (-112 000 to 104 000)
Malaysia	50 800 (-13 300 to 117 000)	54 600 (-341 000 to 454 000)	27 300 (-337 000 to 382 000)
Maldives	2090 (220 to 4020)	1340 (-6230 to 7130)	431 (-3730 to 3970)
Mauritius	71·5 (-3510 to 3570)	623 (-12 500 to 13 000)	330 (-5700 to 6140)
Myanmar	-14 700 (-92 000 to 65 900)	11 000 (-519 000 to 511 000)	43 200 (-422 000 to 445 000)
Philippines	-124 000 (-207 000 to -47 300)	-189 000 (-906 000 to 511 000)	-253 000 (-1 680 000 to 1 090 000)
Seychelles	-227 (-868 to 346)	-75·5 (-1450 to 1450)	-10 (-1310 to 1280)
Sri Lanka	-93 300 (-129 000 to -59 300)	-104 000 (-270 000 to 77 000)	-57 600 (-187 000 to 120 000)
Thailand	20 900 (-91 000 to 138 000)	20 400 (-544 000 to 542 000)	20 300 (-279 000 to 277 000)
Timor-Leste	-5840 (-12 600 to 10 300)	542 (-25 800 to 35 700)	-697 (-38 100 to 42 700)
Vietnam	-41 300 (-186 000 to 103 000)	-38 500 (-893 000 to 786 000)	14 600 (-630 000 to 617 000)
Angola	-530 (-92 100 to 86 000)	37 800 (-544 000 to 728 000)	50 100 (-694 000 to 861 000)
Central African Republic	-28 700 (-52 300 to -6170)	-24 900 (-87 000 to 61 000)	-15 000 (-70 700 to 45 300)
Congo (Brazzaville)	-3950 (-14 400 to 6210)	-3210 (-69 000 to 81 500)	-2900 (-70 300 to 82 100)
DR Congo	25 700 (-289 000 to 287 000)	-6050 (-1 880 000 to 1 790 000)	-185 000 (-2 970 000 to 2 440 000)
Equatorial Guinea	10 700 (-11 300 to 18 400)	8300 (-21 800 to 35 000)	5120 (-24 400 to 32 000)

Location	Net migration count		
	2018	2050	2100
Gabon	774 (-4380 to 5910)	1270 (-25 500 to 34 100)	371 (-28 400 to 32 700)
Burundi	3290 (-92 900 to 92 200)	-51 000 (-444 000 to 274 000)	-101 000 (-932 000 to 615 000)
Comoros	-1800 (-4850 to 1160)	-1570 (-13 600 to 12 600)	-1140 (-12 400 to 10 900)
Djibouti	303 (-13 200 to 11 600)	-1560 (-24 000 to 20 500)	-1910 (-21 000 to 17 300)
Eritrea	-11 200 (-60 500 to 36 500)	-11 000 (-125 000 to 107 000)	-10 300 (-110 000 to 87 500)
Ethiopia	-2890 (-423 000 to 441 000)	-45 500 (-2 530 000 to 2 170 000)	-25 000 (-2 870 000 to 2 490 000)
Kenya	-7500 (-46 200 to 32 600)	-42 100 (-416 000 to 343 000)	-73 700 (-719 000 to 575 000)
Madagascar	-2580 (-9350 to 3610)	-5120 (-89 800 to 75 800)	27 800 (-235 000 to 257 000)
Malawi	-7010 (-176 000 to 175 000)	-739 (-412 000 to 429 000)	-29 000 (-567 000 to 514 000)
Mozambique	485 (-242 000 to 240 000)	25 000 (-568 000 to 637 000)	5270 (-496 000 to 546 000)
Rwanda	-8900 (-145 000 to 132 000)	-8850 (-370 000 to 334 000)	-22 200 (-543 000 to 469 000)
Somalia	-50 300 (-237 000 to 145 000)	-143 000 (-715 000 to 381 000)	-291 000 (-1 390 000 to 668 000)
South Sudan	17 800 (-106 000 to 102 000)	11 100 (-333 000 to 325 000)	50 100 (-636 000 to 727 000)
Uganda	-26 000 (-115 000 to 62 700)	-29 100 (-883 000 to 92 5000)	-96 500 (-1 540 000 to 1 540 000)
Tanzania	-41 600 (-146 000 to 60 300)	-67 100 (-1 210 000 to 1 120 000)	-109 000 (-2 230 000 to 2 260 000)
Zambia	-8610 (-30 200 to 12 800)	-1610 (-266 000 to 238 000)	-8430 (-506 000 to 495 000)
Botswana	3210 (-2090 to 8030)	2980 (-34 100 to 36 900)	1690 (-36 500 to 38 600)
eSwatini	-1760 (-7120 to 3650)	-1630 (-21 600 to 23 100)	-1750 (-26 700 to 26 200)
Lesotho	-3370 (-7420 to 583)	-1600 (-29 200 to 27 100)	-2040 (-32 500 to 28 700)
Namibia	-442 (-13 400 to 12 900)	1820 (-45 900 to 53 700)	85 (-65 600 to 69 700)
South Africa	60 600 (-19 400 to 141 000)	61 900 (-604 000 to 717 000)	32 400 (-831 000 to 854 000)
Zimbabwe	-17 300 (-52 600 to 16 500)	-42 400 (-355 000 to 304 000)	-96 600 (-764 000 to 552 000)
Benin	-2050 (-14 000 to 10 800)	12 500 (-141 000 to 161 000)	21 300 (-231 000 to 237 000)
Burkina Faso	-26 700 (-44 100 to -10 400)	-13 100 (-237 000 to 185 000)	21 000 (-490 000 to 464 000)
Cape Verde	-1330 (-5730 to 4220)	-43·6 (-8470 to 8570)	-87·7 (-5430 to 5220)

	Net migration count		
Location	2018	2050	2100
Cameroon	-7980<br>(-91 900 to 78 700)	143<br>(-461 000 to 552 000)	-2010<br>(-498 000 to 528 000)
Chad	1680<br>(-37 400 to 38 800)	20 500<br>(-512 000 to 521 000)	37 900<br>(-1 270 000 to 1 150 000)
Côte d'Ivoire	3400<br>(-66 100 to 70 600)	18 600<br>(-527 000 to 584 000)	39 700<br>(-668 000 to 754 000)
The Gambia	-2660<br>(-19 000 to 12 600)	-1610<br>(-49 500 to 50 000)	-4010<br>(-57 300 to 50 100)
Ghana	-25 900<br>(-173 000 to 130 000)	15 200<br>(-578 000 to 699 000)	13 900<br>(-665 000 to 753 000)
Guinea	-11 500<br>(-124 000 to 102 000)	1310<br>(-284 000 to 296 000)	7270<br>(-321 000 to 337 000)
Guinea-Bissau	-2330<br>(-13 500 to 7860)	-1030<br>(-44 800 to 41 100)	-1100<br>(-46 200 to 42 300)
Liberia	-4530<br>(-57 200 to 49 400)	-1320<br>(-108 000 to 98 500)	-5540<br>(-109 000 to 91 300)
Mali	-43 700<br>(-98 600 to 12 400)	-49 500<br>(-648 000 to 602 000)	-15 600<br>(-1 250 000 to 1 280 000)
Mauritania	4540<br>(298 to 9470)	10 500<br>(-36 600 to 60 600)	9630<br>(-63 300 to 78 500)
Niger	-4370<br>(-28 700 to 16 700)	2330<br>(-496 000 to 432 000)	9230<br>(-1 800 000 to 1 650 000)
Nigeria	-67 700<br>(-346 000 to 186 000)	98 800<br>(-2 930 000 to 3 050 000)	-32 400<br>(-6 010 000 to 6 070 000)
São Tomé and Príncipe	-864<br>(-2180 to 966)	-131<br>(-3680 to 4000)	-135<br>(-3220 to 3220)
Senegal	-17 400<br>(-52 100 to 15 600)	-21 500<br>(-328 000 to 325 000)	-20 100<br>(-453 000 to 473 000)
Sierra Leone	-2970<br>(-80 800 to 71 500)	-9260<br>(-191 000 to 165 000)	-15 800<br>(-192 000 to 157 000)
Togo	-1690<br>(-67 400 to 61 600)	2550<br>(-142 000 to 150 000)	-1650<br>(-122 000 to 115 000)

Section 5 Total fertility rate through 2100

Figure 5. Global and super-region total fertility rate from 1990 to 2100. Past estimates are from GBD 2017, with future estimates for the reference forecast and each of the four alternative scenarios. SDG=Sustainable Development Goal. GBD=Global Burden of Disease.

A. Global

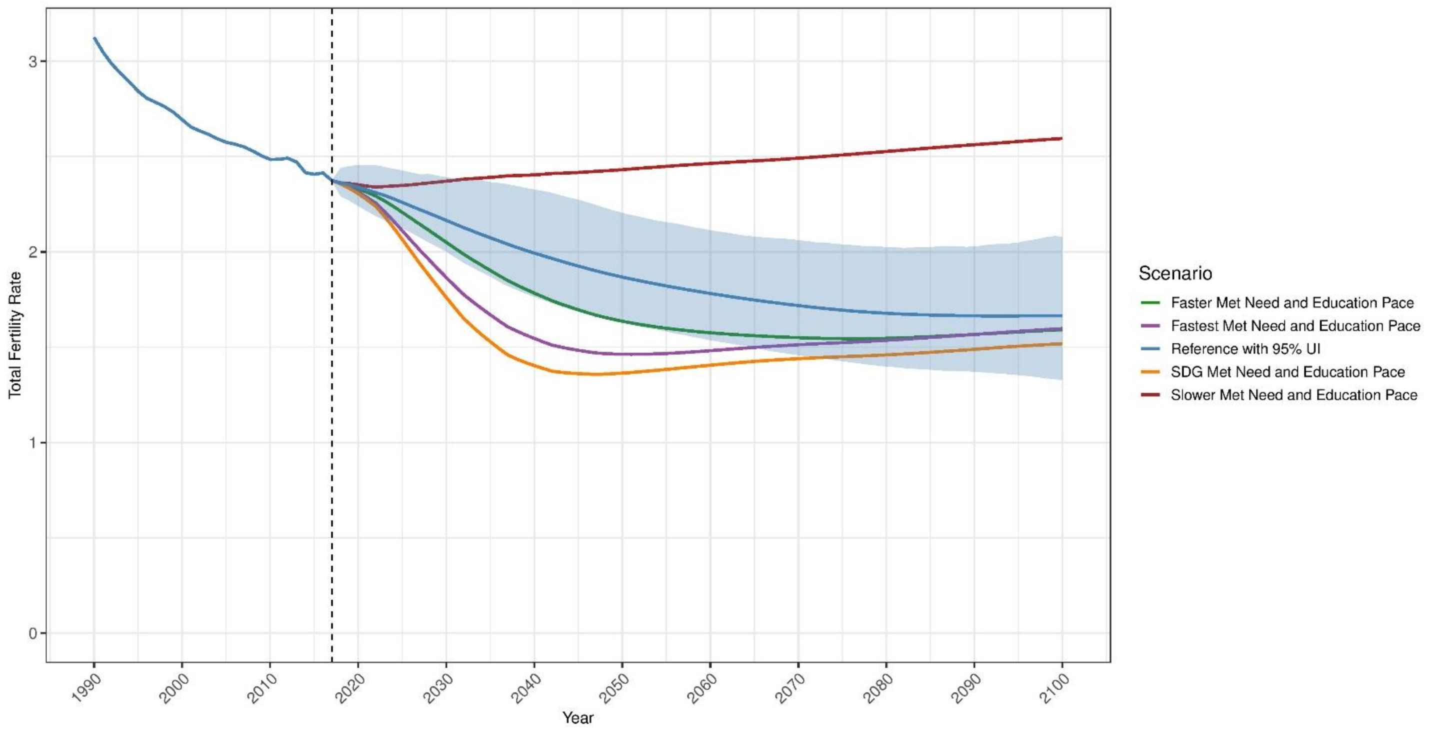

B. Southeast Asia, east Asia, and Oceania

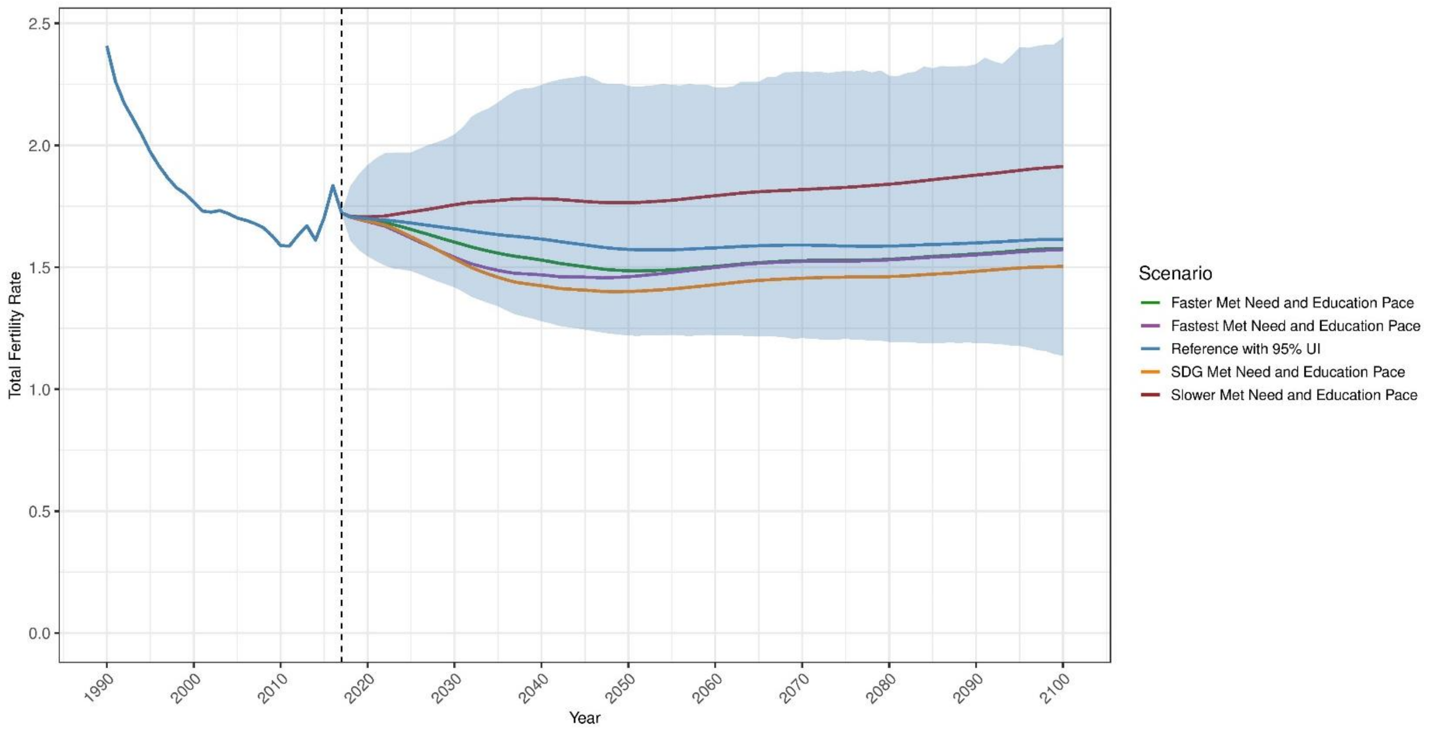

C. Central Europe, eastern Europe, and central Asia

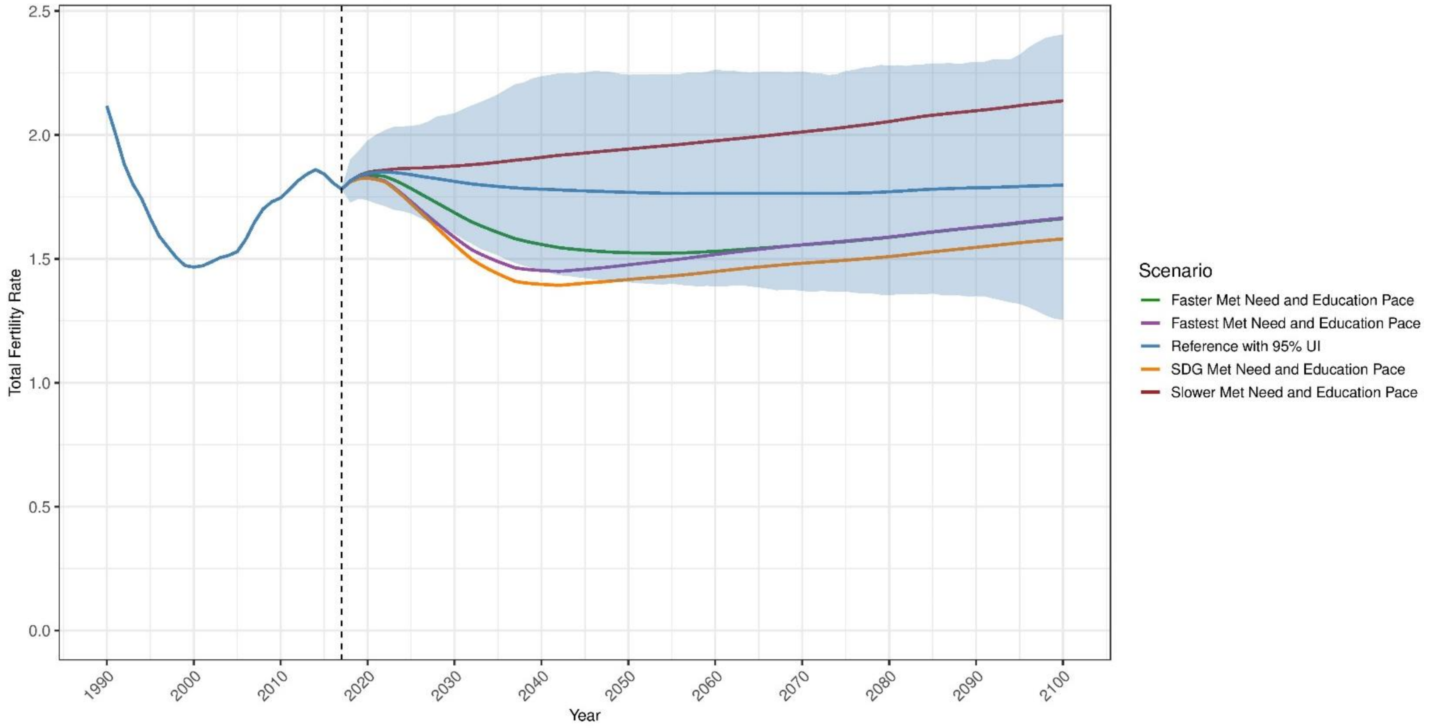

D. High-income

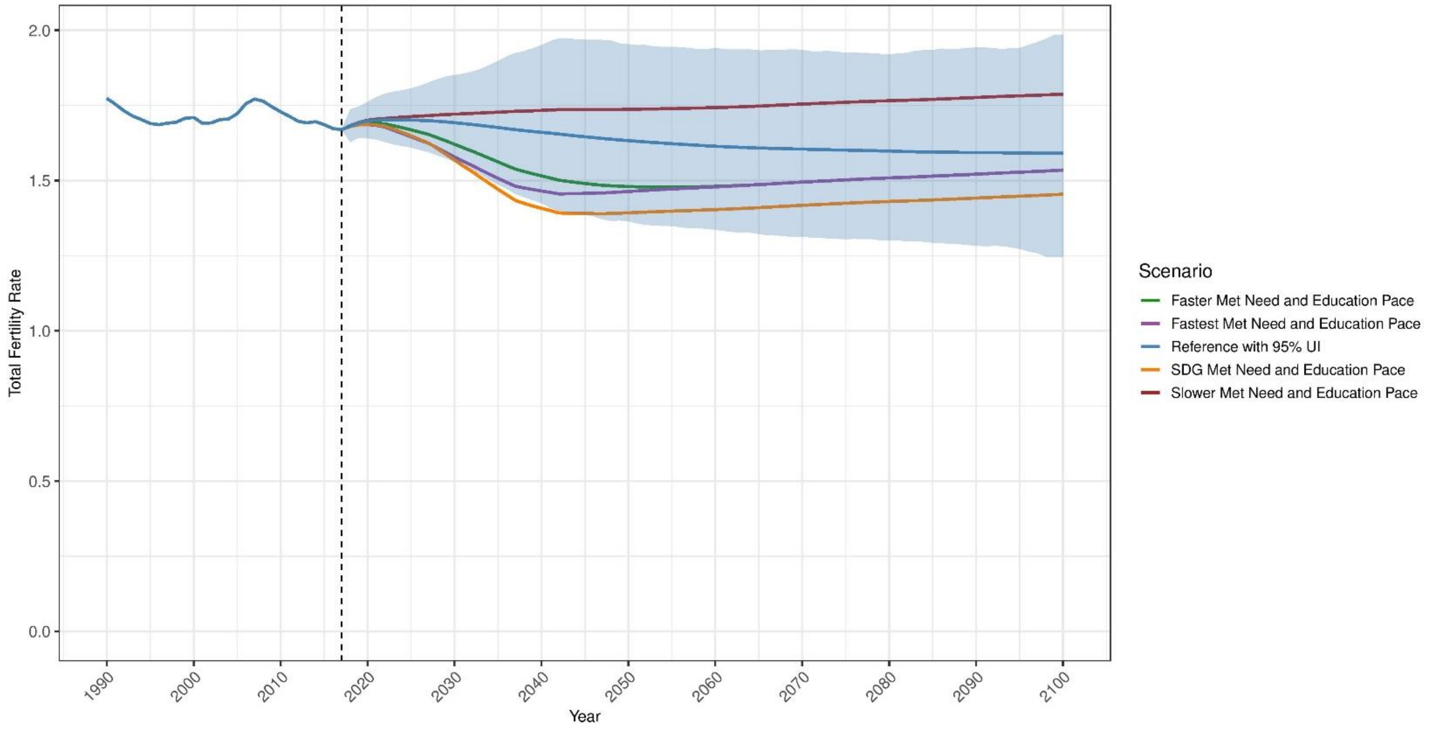

E. Latin America and Caribbean

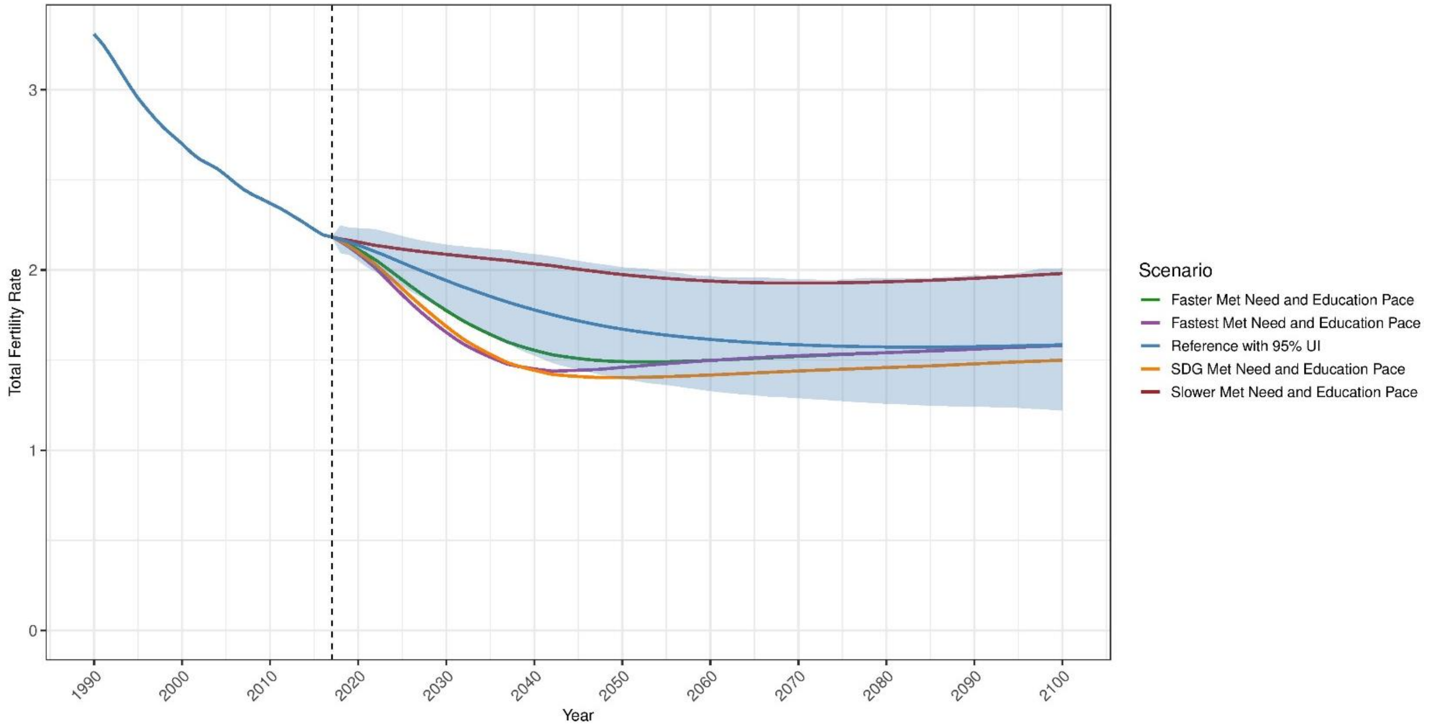

F. South Asia

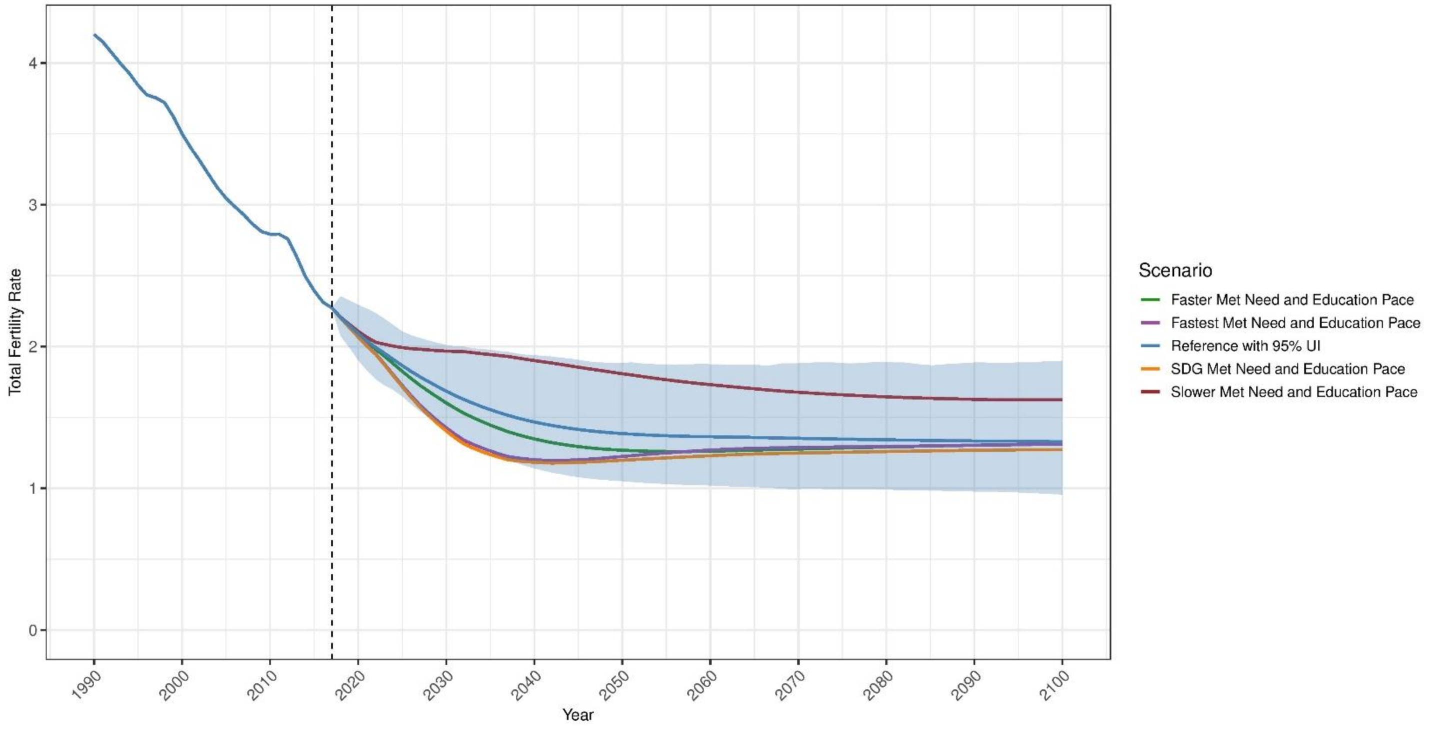

G. North Africa and Middle East

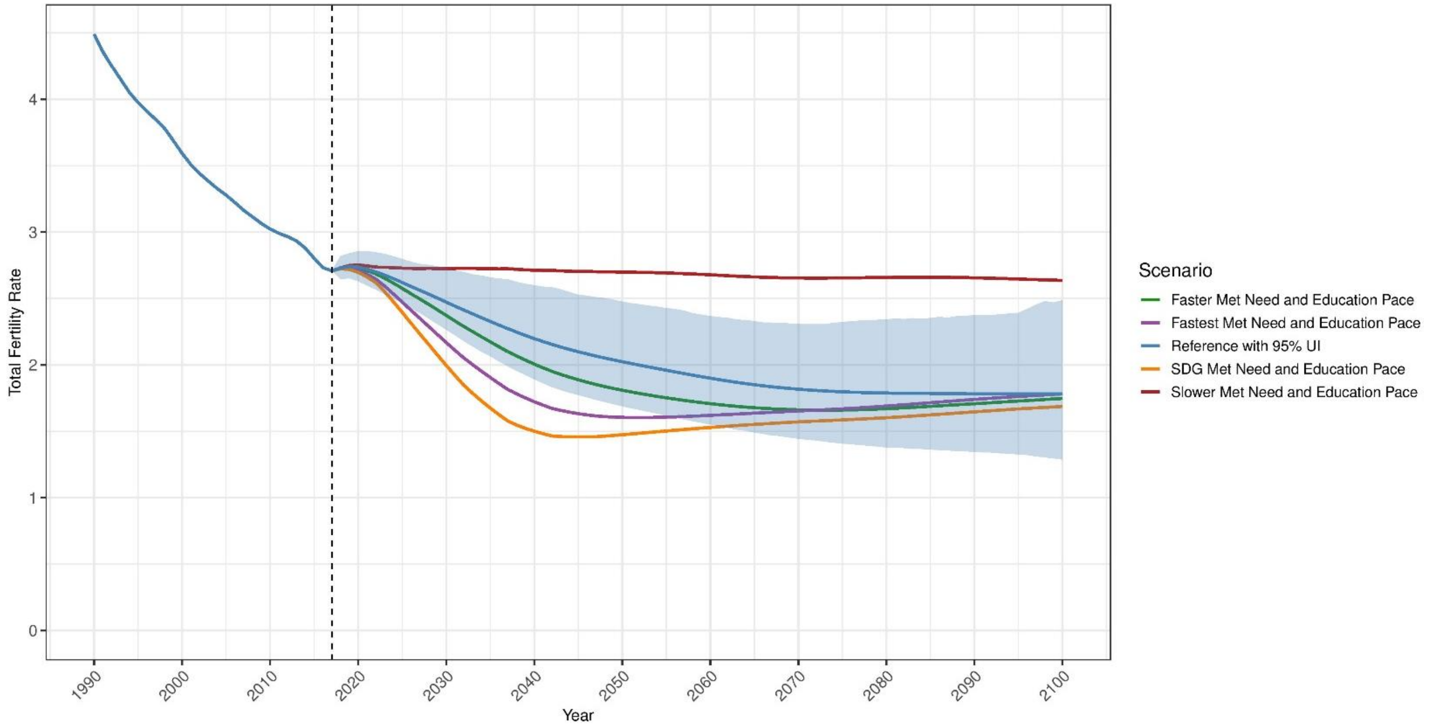

H. Sub-Saharan Africa

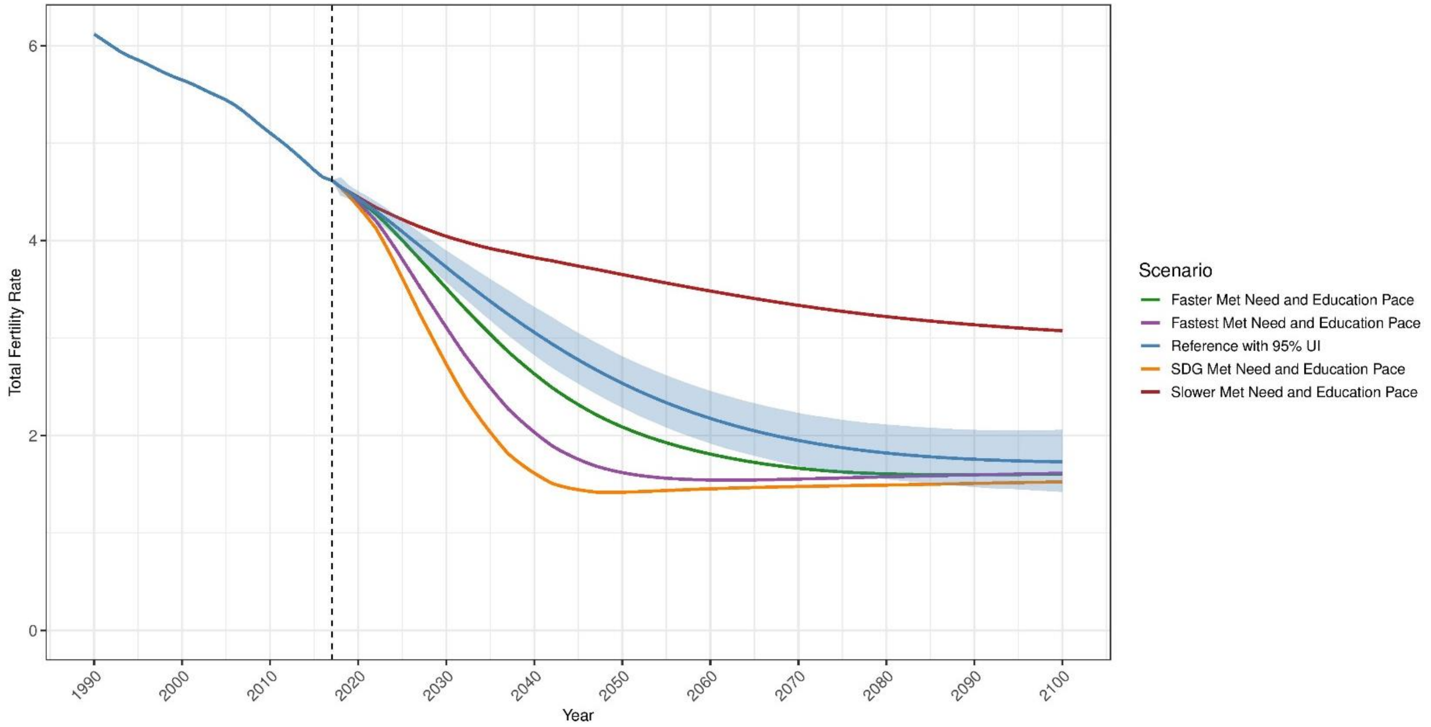

Figure 6. Total fertility rate from 1950 to 2100 for ten sample countries in the reference (with uncertainty), slower, faster, fastest, and SDG pace scenarios. Past estimates are from GBD 2017, with future estimates for the reference forecast and each of the four alternative scenarios. SDG=Sustainable Development Goal. GBD=Global Burden of Disease.

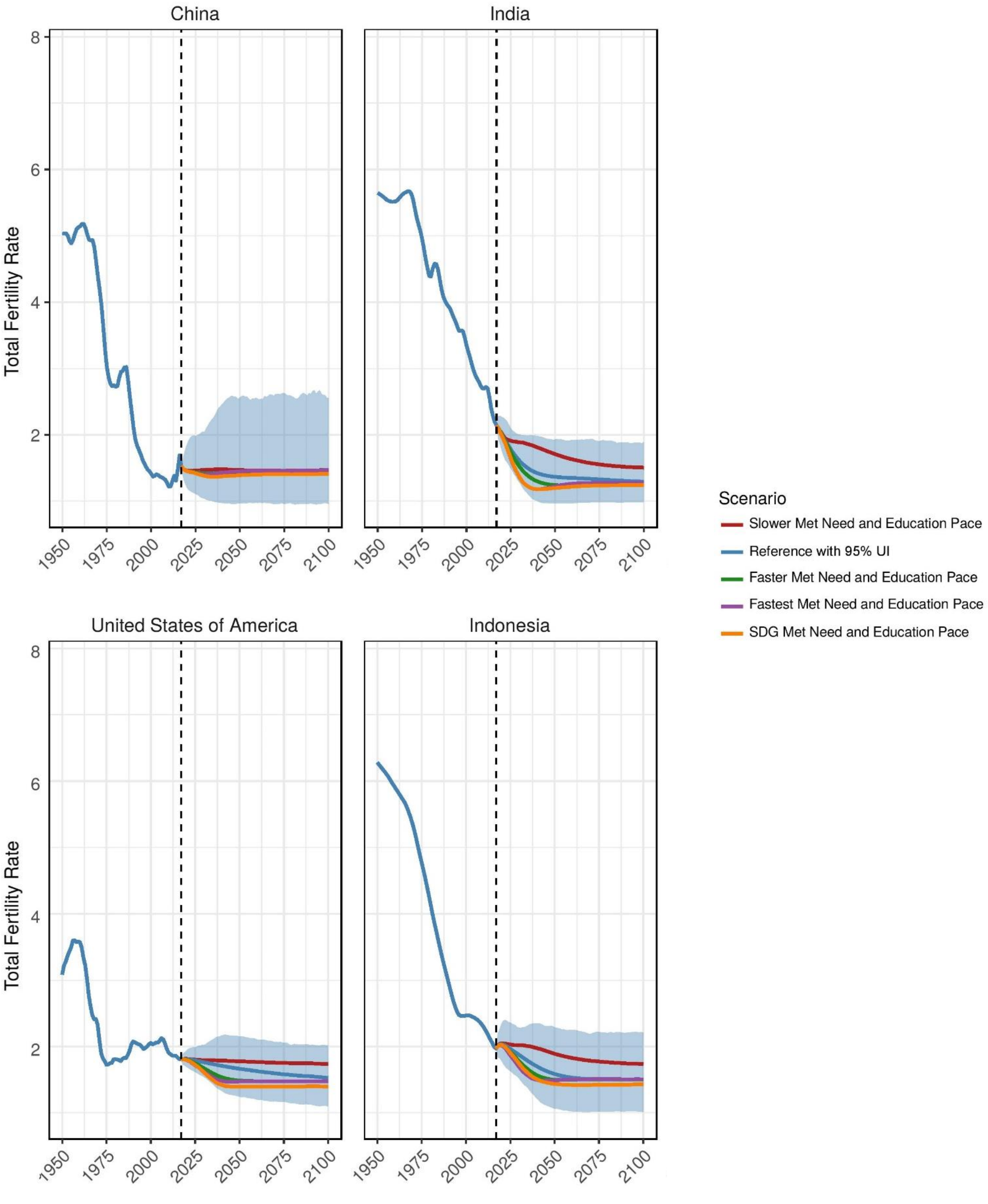

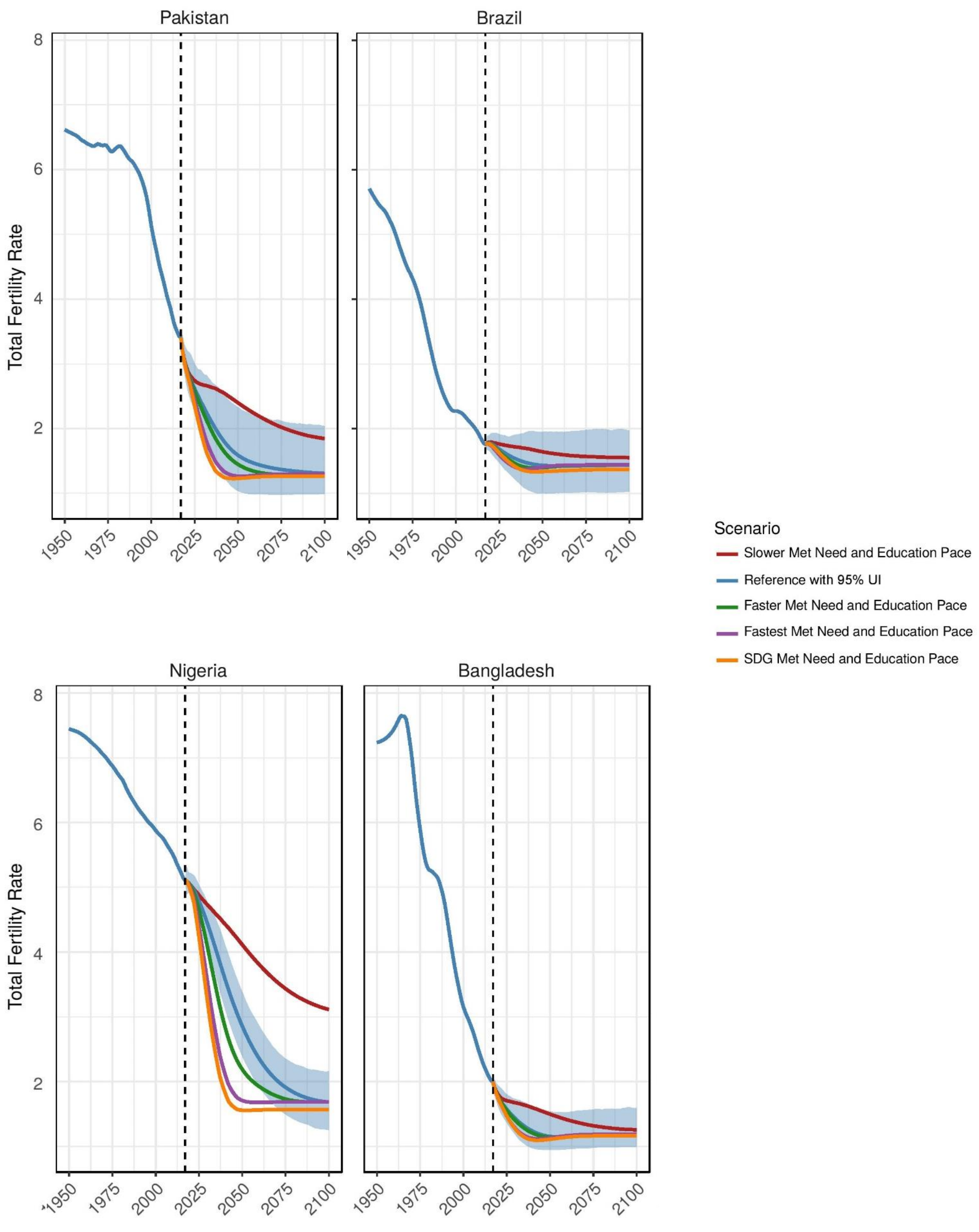
Pakistan
Brazil
Nigeria
Bangladesh
Total Fertility Rate
8
6
4
2
1950
1975
2000
2025
2050
2075
2100
Scenario
Slower Met Need and Education Pace
Reference with 95% UI
Faster Met Need and Education Pace
Fastest Met Need and Education Pace
SDG Met Need and Education Pace

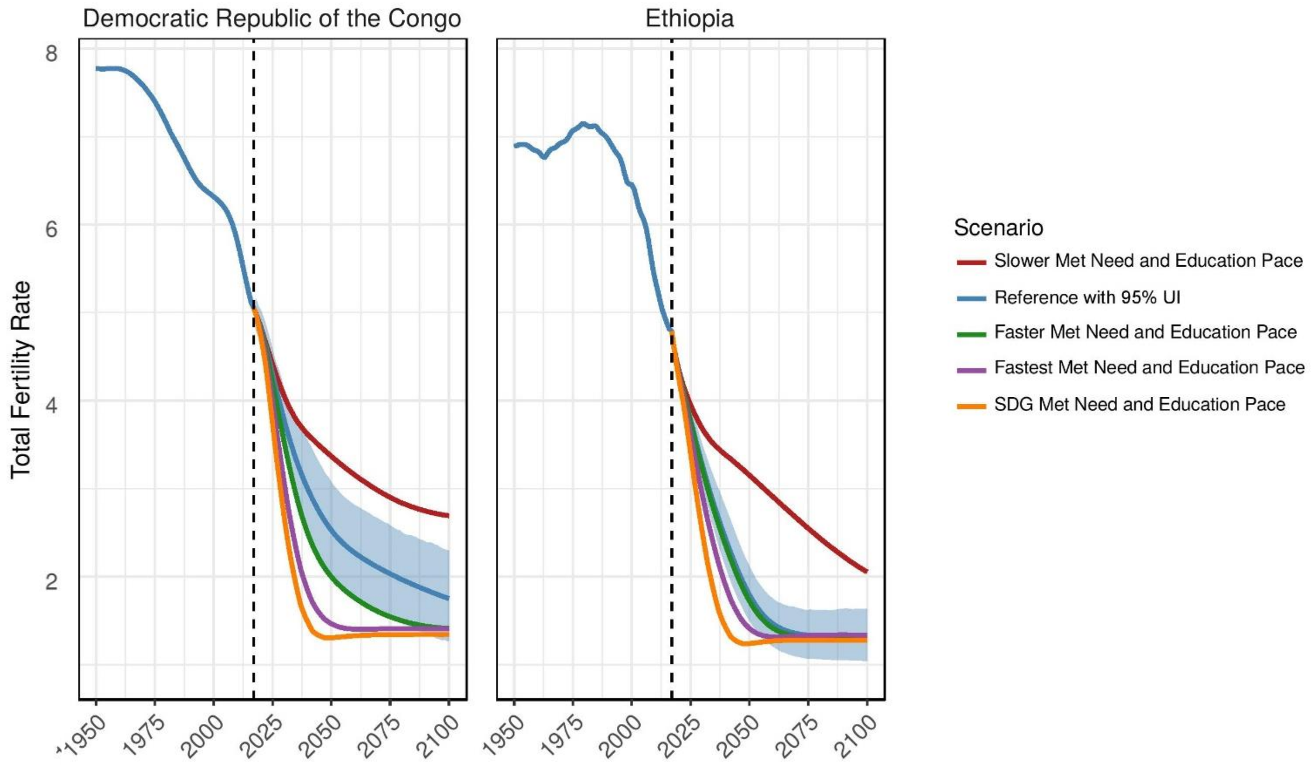

Table 3. Total fertility rate in the reference forecast and faster met need and education pace scenario: 1990, 2017, 2050, and 2100. Past estimates are from the Global Burden of Disease Study (GBD) 2017. Estimates are listed as means with 95% uncertainty intervals in parentheses. Highlighted rows indicate region and super-region results from the GBD location hierarchy.

Location	Total fertility rate, reference				Total fertility rate – faster met need and education pace			
	1990	2017	2050	2100	1990	2017	2050	2100
**Global**	3·12 (3·06, 3·19)	2·37 (2·22, 2·55)	1·87 (1·63, 2·2)	1·66 (1·33, 2·08)	3·12 (3·06, 3·19)	2·37 (2·22, 2·55)	1·64 (1·4, 1·97)	1·59 (1·22, 2·05)
**Central Europe, Eastern Europe, and Central Asia**	2·12 (2·09, 2·14)	1·78 (1·59, 1·99)	1·77 (1·41, 2·24)	1·8 (1·25, 2·4)	2·12 (2·09, 2·14)	1·78 (1·59, 1·99)	1·52 (1·2, 2·01)	1·66 (1·11, 2·32)
**Central Asia**	3·4 (3·32, 3·47)	2·47 (2·26, 2·69)	2·24 (1·76, 2·8)	2·14 (1·5, 2·83)	3·4 (3·32, 3·47)	2·47 (2·26, 2·69)	1·85 (1·37, 2·46)	1·99 (1·31, 2·76)
Armenia	2·61 (2·53, 2·7)	1·58 (1·44, 1·72)	1·54 (0·98, 2·67)	1·43 (0·96, 2·51)	2·61 (2·53, 2·7)	1·58 (1·44, 1·72)	1·32 (0·94, 2·35)	1·28 (0·94, 2·36)
Azerbaijan	2·78 (2·68, 2·9)	1·96 (1·73, 2·23)	1·73 (1·02, 2·73)	1·5 (0·98, 2·45)	2·78 (2·68, 2·9)	1·96 (1·73, 2·23)	1·41 (0·95, 2·43)	1·32 (0·96, 2·23)
Georgia	1·85 (1·76, 1·94)	2·05 (1·86, 2·25)	1·85 (1·11, 2·85)	1·59 (1·01, 2·55)	1·85 (1·76, 1·94)	2·05 (1·86, 2·25)	1·53 (0·98, 2·37)	1·44 (0·97, 2·33)
Kazakhstan	2·71 (2·65, 2·78)	2·39 (2·16, 2·63)	2·23 (1·54, 2·97)	1·97 (1·29, 2·8)	2·71 (2·65, 2·78)	2·39 (2·16, 2·63)	1·97 (1·31, 2·79)	1·96 (1·23, 2·81)

Location	Total fertility rate, reference				Total fertility rate – faster met need and education pace			
	1990	2017	2050	2100	1990	2017	2050	2100
Kyrgyzstan	3·48 (3·38, 3·58)	2·78 (2·59, 2·97)	2·6 (1·78, 3·6)	2·25 (1·4, 3·29)	3·48 (3·38, 3·58)	2·78 (2·59, 2·97)	1·94 (1·12, 2·98)	1·79 (1·03, 2·79)
Mongolia	3·91 (3·8, 4·01)	2·7 (2·49, 2·91)	2·38 (1·07, 4·72)	1·98 (0·98, 4·27)	3·91 (3·8, 4·01)	2·7 (2·49, 2·91)	1·94 (0·96, 4·21)	1·91 (0·97, 4·33)
Tajikistan	5·1 (4·89, 5·3)	3·55 (3·22, 3·87)	2·85 (2·29, 3·44)	2·25 (1·68, 2·86)	5·1 (4·89, 5·3)	3·55 (3·22, 3·87)	1·88 (1·37, 2·46)	1·64 (1·1, 2·25)
Turkmenistan	4·1 (3·99, 4·21)	2·76 (2·47, 3·11)	2·34 (1·2, 3·88)	1·91 (1·01, 3·51)	4·1 (3·99, 4·21)	2·76 (2·47, 3·11)	1·81 (0·99, 3·36)	1·74 (0·98, 3·28)
Uzbekistan	4·17 (4·03, 4·32)	2·35 (2·14, 2·57)	2·02 (1·37, 2·75)	1·89 (1·23, 2·65)	4·17 (4·03, 4·32)	2·35 (2·14, 2·57)	1·86 (1·23, 2·59)	1·89 (1·21, 2·71)
**Central Europe**	1·92 (1·91, 1·93)	1·43 (1·29, 1·59)	1·38 (1·17, 1·68)	1·35 (1·04, 1·74)	1·92 (1·91, 1·93)	1·43 (1·29, 1·59)	1·26 (1·06, 1·55)	1·28 (0·98, 1·69)
Albania	3·16 (3·07, 3·25)	1·88 (1·63, 2·18)	1·7 (1·29, 2·15)	1·49 (1·08, 1·97)	3·16 (3·07, 3·25)	1·88 (1·63, 2·18)	1·4 (1·02, 1·84)	1·2 (0·97, 1·61)
Bosnia and Herzegovina	1·8 (1·75, 1·85)	1·26 (1·17, 1·36)	1·29 (1·07, 1·55)	1·19 (1·0, 1·45)	1·8 (1·75, 1·85)	1·26 (1·17, 1·36)	1·16 (0·97, 1·39)	1·1 (0·97, 1·32)
Bulgaria	1·7 (1·67, 1·72)	1·47 (1·29, 1·67)	1·34 (0·97, 1·81)	1·25 (0·95, 1·76)	1·7 (1·67, 1·72)	1·47 (1·29, 1·67)	1·25 (0·95, 1·75)	1·25 (0·96, 1·77)
Croatia	1·52 (1·49, 1·54)	1·37 (1·3, 1·45)	1·46 (1·21, 1·72)	1·39 (1·13, 1·69)	1·52 (1·49, 1·54)	1·37 (1·3, 1·45)	1·23 (1·02, 1·46)	1·18 (0·99, 1·41)
Czech Republic	1·86 (1·84, 1·89)	1·58 (1·45, 1·73)	1·48 (1·06, 2·0)	1·37 (0·99, 1·87)	1·86 (1·84, 1·89)	1·58 (1·45, 1·73)	1·38 (1·0, 1·89)	1·37 (0·99, 1·87)
Hungary	1·84 (1·81, 1·86)	1·43 (1·26, 1·61)	1·43 (1·0, 2·07)	1·38 (0·98, 2·01)	1·84 (1·81, 1·86)	1·43 (1·26, 1·61)	1·28 (0·96, 1·9)	1·29 (0·96, 1·92)
Montenegro	1·94 (1·85, 2·03)	1·67 (1·59, 1·76)	1·7 (1·51, 1·91)	1·62 (1·42, 1·82)	1·94 (1·85, 2·03)	1·67 (1·59, 1·76)	1·36 (1·19, 1·54)	1·22 (1·05, 1·36)
North Macedonia	2·19 (2·16, 2·22)	1·51 (1·41, 1·61)	1·52 (1·33, 1·74)	1·39 (1·2, 1·62)	2·19 (2·16, 2·22)	1·51 (1·41, 1·61)	1·3 (1·1, 1·52)	1·13 (0·98, 1·34)
Poland	2·01 (2·0, 2·03)	1·31 (1·16, 1·48)	1·27 (0·99, 1·67)	1·17 (0·96, 1·55)	2·01 (2·0, 2·03)	1·31 (1·16, 1·48)	1·18 (0·96, 1·54)	1·15 (0·96, 1·51)

Location	Total fertility rate, reference				Total fertility rate – faster met need and education pace			
	**1990**	**2017**	**2050**	**2100**	**1990**	**2017**	**2050**	**2100**
Romania	1·71 (1·69, 1·73)	1·56 (1·4, 1·74)	1·39 (0·96, 2·11)	1·28 (0·92, 2·0)	1·71 (1·69, 1·73)	1·56 (1·4, 1·74)	1·28 (0·93, 1·97)	1·27 (0·92, 2·01)
Serbia	2·07 (2·05, 2·1)	1·37 (1·21, 1·56)	1·42 (1·2, 1·68)	1·34 (1·09, 1·62)	2·07 (2·05, 2·1)	1·37 (1·21, 1·56)	1·21 (1·0, 1·44)	1·15 (0·97, 1·4)
Slovakia	2·09 (2·06, 2·12)	1·39 (1·23, 1·58)	1·37 (1·02, 1·79)	1·3 (0·99, 1·7)	2·09 (2·06, 2·12)	1·39 (1·23, 1·58)	1·27 (0·98, 1·67)	1·28 (0·99, 1·68)
Slovenia	1·53 (1·49, 1·58)	1·52 (1·39, 1·66)	1·48 (1·14, 1·88)	1·36 (1·04, 1·77)	1·53 (1·49, 1·58)	1·52 (1·39, 1·66)	1·33 (1·03, 1·7)	1·31 (1·01, 1·7)
**Eastern Europe**	1·8 (1·78, 1·82)	1·55 (1·35, 1·79)	1·53 (1·09, 2·16)	1·45 (0·94, 2·25)	1·8 (1·78, 1·82)	1·55 (1·35, 1·79)	1·37 (1·0, 2·0)	1·38 (0·9, 2·2)
Belarus	1·77 (1·72, 1·83)	1·57 (1·4, 1·78)	1·48 (1·0, 2·17)	1·4 (0·99, 2·08)	1·77 (1·72, 1·83)	1·57 (1·4, 1·78)	1·4 (0·98, 2·07)	1·4 (0·98, 2·05)
Estonia	1·92 (1·87, 1·96)	1·57 (1·38, 1·79)	1·6 (1·11, 2·27)	1·48 (1·0, 2·13)	1·92 (1·87, 1·96)	1·57 (1·38, 1·79)	1·43 (0·98, 2·03)	1·43 (0·98, 2·06)
Latvia	1·87 (1·83, 1·91)	1·58 (1·39, 1·79)	1·5 (1·01, 2·17)	1·37 (0·96, 2·03)	1·87 (1·83, 1·91)	1·58 (1·39, 1·79)	1·39 (0·99, 2·04)	1·36 (0·96, 1·97)
Lithuania	1·99 (1·95, 2·02)	1·62 (1·44, 1·82)	1·59 (1·06, 2·28)	1·44 (0·99, 2·16)	1·99 (1·95, 2·02)	1·62 (1·44, 1·82)	1·46 (0·99, 2·1)	1·44 (0·99, 2·1)
Moldova	2·26 (2·18, 2·33)	1·31 (1·16, 1·49)	1·36 (0·98, 1·97)	1·32 (0·97, 1·96)	2·26 (2·18, 2·33)	1·31 (1·16, 1·49)	1·22 (0·95, 1·82)	1·21 (0·95, 1·81)
Russia	1·81 (1·8, 1·82)	1·61 (1·39, 1·85)	1·56 (1·01, 2·37)	1·43 (0·96, 2·23)	1·81 (1·8, 1·82)	1·61 (1·39, 1·85)	1·39 (0·96, 2·16)	1·36 (0·94, 2·2)
Ukraine	1·7 (1·64, 1·76)	1·4 (1·22, 1·61)	1·38 (0·99, 1·98)	1·32 (0·96, 1·92)	1·7 (1·64, 1·76)	1·4 (1·22, 1·61)	1·23 (0·96, 1·8)	1·22 (0·95, 1·78)
**High-income**	1·77 (1·76, 1·78)	1·67 (1·52, 1·84)	1·63 (1·37, 1·95)	1·59 (1·25, 1·98)	1·77 (1·76, 1·78)	1·67 (1·52, 1·84)	1·48 (1·21, 1·81)	1·53 (1·18, 1·95)
**Australasia**	1·93 (1·91, 1·95)	1·89 (1·71, 2·09)	1·84 (1·38, 2·39)	1·71 (1·22, 2·29)	1·93 (1·91, 1·95)	1·89 (1·71, 2·09)	1·65 (1·22, 2·19)	1·64 (1·15, 2·24)
Australia	1·89 (1·87, 1·91)	1·86 (1·65, 2·09)	1·82 (1·34, 2·44)	1·69 (1·19, 2·26)	1·89 (1·87, 1·91)	1·86 (1·65, 2·09)	1·64 (1·19, 2·2)	1·62 (1·14, 2·17)

Location	Total fertility rate, reference				Total fertility rate – faster met need and education pace			
	**1990**	**2017**	**2050**	**2100**	**1990**	**2017**	**2050**	**2100**
New Zealand	2·12 (2·07, 2·16)	2·1 (1·92, 2·32)	1·92 (1·28, 2·73)	1·71 (1·07, 2·54)	2·12 (2·07, 2·16)	2·1 (1·92, 2·32)	1·71 (1·05, 2·51)	1·66 (1·04, 2·48)
**High-income Asia-Pacific**	1·55 (1·51, 1·59)	1·3 (1·15, 1·47)	1·36 (1·04, 1·9)	1·33 (0·95, 1·97)	1·55 (1·51, 1·59)	1·3 (1·15, 1·47)	1·25 (0·99, 1·76)	1·25 (0·92, 1·86)
Brunei	3·06 (2·93, 3·18)	1·88 (1·74, 2·03)	1·84 (1·18, 2·61)	1·67 (1·06, 2·44)	3·06 (2·93, 3·18)	1·88 (1·74, 2·03)	1·52 (1·01, 2·22)	1·44 (1·0, 2·17)
Japan	1·47 (1·41, 1·54)	1·33 (1·11, 1·59)	1·38 (0·99, 2·08)	1·32 (0·96, 2·03)	1·47 (1·41, 1·54)	1·33 (1·11, 1·59)	1·25 (0·97, 1·92)	1·22 (0·96, 1·89)
Singapore	1·62 (1·54, 1·69)	1·26 (1·05, 1·52)	1·32 (0·94, 2·04)	1·27 (0·91, 1·99)	1·62 (1·54, 1·69)	1·26 (1·05, 1·52)	1·24 (0·93, 1·93)	1·24 (0·91, 1·97)
South Korea	1·66 (1·64, 1·68)	1·24 (1·18, 1·3)	1·32 (0·99, 1·86)	1·24 (0·94, 1·78)	1·66 (1·64, 1·68)	1·24 (1·18, 1·3)	1·24 (0·96, 1·77)	1·22 (0·93, 1·75)
**High-income North America**	2·04 (2·03, 2·05)	1·79 (1·65, 1·95)	1·67 (1·28, 2·11)	1·54 (1·13, 2·03)	2·04 (2·03, 2·05)	1·79 (1·65, 1·95)	1·49 (1·11, 1·93)	1·48 (1·07, 1·98)
Canada	1·74 (1·72, 1·75)	1·66 (1·42, 1·94)	1·68 (1·38, 2·03)	1·58 (1·29, 1·9)	1·74 (1·72, 1·75)	1·66 (1·42, 1·94)	1·47 (1·22, 1·76)	1·44 (1·18, 1·74)
Greenland	2·43 (2·31, 2·56)	2·02 (1·79, 2·26)	1·71 (1·24, 2·29)	1·52 (1·08, 2·06)	2·43 (2·31, 2·56)	2·02 (1·79, 2·26)	1·35 (0·98, 1·82)	1·34 (1·0, 1·83)
USA	2·08 (2·07, 2·09)	1·81 (1·68, 1·95)	1·66 (1·24, 2·15)	1·53 (1·1, 2·02)	2·08 (2·07, 2·09)	1·81 (1·68, 1·95)	1·49 (1·09, 1·97)	1·47 (1·06, 1·96)
**Southern Latin America**	2·68 (2·66, 2·69)	2·06 (1·9, 2·25)	1·73 (1·34, 2·14)	1·58 (1·14, 2·06)	2·68 (2·66, 2·69)	2·06 (1·9, 2·25)	1·53 (1·15, 1·93)	1·56 (1·11, 2·08)
Argentina	2·81 (2·79, 2·83)	2·17 (2·02, 2·33)	1·8 (1·31, 2·33)	1·62 (1·14, 2·13)	2·81 (2·79, 2·83)	2·17 (2·02, 2·33)	1·59 (1·11, 2·1)	1·61 (1·12, 2·15)
Chile	2·45 (2·43, 2·48)	1·81 (1·59, 2·06)	1·52 (1·14, 1·94)	1·37 (1·04, 1·78)	2·45 (2·43, 2·48)	1·81 (1·59, 2·06)	1·36 (1·03, 1·74)	1·35 (1·03, 1·76)
Uruguay	2·41 (2·37, 2·45)	1·97 (1·72, 2·27)	1·61 (1·28, 1·97)	1·44 (1·16, 1·76)	2·41 (2·37, 2·45)	1·97 (1·72, 2·27)	1·43 (1·15, 1·75)	1·44 (1·13, 1·79)
**Western Europe**	1·57 (1·56, 1·58)	1·59 (1·43, 1·78)	1·63 (1·39, 1·91)	1·64 (1·28, 2·05)	1·57 (1·56, 1·58)	1·59 (1·43, 1·78)	1·51 (1·27, 1·8)	1·59 (1·23, 1·99)

Location	Total fertility rate, reference				Total fertility rate – faster met need and education pace			
	**1990**	**2017**	**2050**	**2100**	**1990**	**2017**	**2050**	**2100**
Andorra	1·25 (1·2, 1·3)	1·2 (1·06, 1·35)	1·26 (1·02, 1·53)	1·23 (1·02, 1·5)	1·25 (1·2, 1·3)	1·2 (1·06, 1·35)	1·2 (1·0, 1·45)	1·21 (1·01, 1·47)
Austria	1·48 (1·45, 1·51)	1·51 (1·38, 1·66)	1·47 (1·1, 1·88)	1·37 (1·02, 1·76)	1·48 (1·45, 1·51)	1·51 (1·38, 1·66)	1·38 (1·03, 1·78)	1·36 (1·02, 1·78)
Belgium	1·65 (1·63, 1·67)	1·69 (1·52, 1·87)	1·73 (1·32, 2·2)	1·6 (1·21, 2·06)	1·65 (1·63, 1·67)	1·69 (1·52, 1·87)	1·56 (1·16, 1·98)	1·54 (1·14, 1·98)
Cyprus	1·94 (1·85, 2·04)	1·01 (0·87, 1·17)	1·19 (0·98, 1·52)	1·18 (0·96, 1·52)	1·94 (1·85, 2·04)	1·01 (0·87, 1·17)	1·17 (0·97, 1·49)	1·18 (0·96, 1·54)
Denmark	1·69 (1·67, 1·72)	1·75 (1·57, 1·95)	1·75 (1·17, 2·43)	1·66 (1·08, 2·31)	1·69 (1·67, 1·72)	1·75 (1·57, 1·95)	1·49 (1·02, 2·1)	1·46 (1·01, 2·07)
Finland	1·77 (1·74, 1·79)	1·64 (1·47, 1·83)	1·69 (1·3, 2·13)	1·6 (1·22, 2·01)	1·77 (1·74, 1·79)	1·64 (1·47, 1·83)	1·57 (1·2, 2·0)	1·56 (1·19, 2·0)
France	1·74 (1·73, 1·75)	1·84 (1·66, 2·05)	1·84 (1·49, 2·25)	1·78 (1·42, 2·2)	1·74 (1·73, 1·75)	1·84 (1·66, 2·05)	1·78 (1·44, 2·17)	1·78 (1·39, 2·19)
Germany	1·4 (1·39, 1·42)	1·39 (1·24, 1·57)	1·43 (1·12, 1·79)	1·35 (1·05, 1·69)	1·4 (1·39, 1·42)	1·39 (1·24, 1·57)	1·32 (1·03, 1·64)	1·31 (1·02, 1·63)
Greece	1·34 (1·32, 1·37)	1·42 (1·27, 1·6)	1·41 (1·01, 1·92)	1·29 (0·97, 1·82)	1·34 (1·32, 1·37)	1·42 (1·27, 1·6)	1·27 (0·97, 1·78)	1·22 (0·96, 1·74)
Iceland	2·22 (2·12, 2·31)	1·83 (1·68, 2·0)	1·85 (1·27, 2·51)	1·72 (1·16, 2·4)	2·22 (2·12, 2·31)	1·83 (1·68, 2·0)	1·71 (1·12, 2·4)	1·7 (1·11, 2·4)
Ireland	2·03 (1·98, 2·07)	1·84 (1·64, 2·07)	1·8 (1·2, 2·56)	1·68 (1·1, 2·46)	2·03 (1·98, 2·07)	1·84 (1·64, 2·07)	1·69 (1·11, 2·44)	1·67 (1·08, 2·44)
Israel	2·82 (2·79, 2·86)	2·9 (2·64, 3·19)	2·71 (1·77, 3·91)	2·36 (1·45, 3·54)	2·82 (2·79, 2·86)	2·9 (2·64, 3·19)	2·31 (1·46, 3·38)	2·22 (1·35, 3·32)
Italy	1·32 (1·31, 1·33)	1·33 (1·18, 1·5)	1·32 (1·0, 1·74)	1·23 (0·99, 1·64)	1·32 (1·31, 1·33)	1·33 (1·18, 1·5)	1·22 (0·98, 1·62)	1·2 (0·98, 1·6)
Luxembourg	1·53 (1·48, 1·59)	1·48 (1·35, 1·61)	1·58 (1·22, 1·99)	1·5 (1·13, 1·9)	1·53 (1·48, 1·59)	1·48 (1·35, 1·61)	1·48 (1·12, 1·86)	1·47 (1·11, 1·86)
Malta	2·05 (1·96, 2·14)	1·49 (1·32, 1·68)	1·38 (1·04, 1·76)	1·27 (1·0, 1·64)	2·05 (1·96, 2·14)	1·49 (1·32, 1·68)	1·27 (1·0, 1·66)	1·24 (0·99, 1·63)

Location	Total fertility rate, reference				Total fertility rate – faster met need and education pace			
	1990	2017	2050	2100	1990	2017	2050	2100
Netherlands	1·62 (1·6, 1·64)	1·66 (1·49, 1·85)	1·71 (1·35, 2·13)	1·59 (1·24, 2·01)	1·62 (1·6, 1·64)	1·66 (1·49, 1·85)	1·49 (1·17, 1·84)	1·46 (1·13, 1·81)
Norway	1·92 (1·89, 1·95)	1·74 (1·59, 1·9)	1·79 (1·19, 2·52)	1·67 (1·09, 2·35)	1·92 (1·89, 1·95)	1·74 (1·59, 1·9)	1·62 (1·04, 2·34)	1·61 (1·04, 2·36)
Portugal	1·52 (1·49, 1·55)	1·29 (1·14, 1·48)	1·32 (0·99, 1·8)	1·26 (0·98, 1·73)	1·52 (1·49, 1·55)	1·29 (1·14, 1·48)	1·25 (0·98, 1·7)	1·25 (0·98, 1·71)
Spain	1·33 (1·31, 1·35)	1·35 (1·23, 1·49)	1·3 (0·97, 1·92)	1·24 (0·96, 1·83)	1·33 (1·31, 1·35)	1·35 (1·23, 1·49)	1·24 (0·97, 1·81)	1·23 (0·96, 1·82)
Sweden	2·11 (2·09, 2·14)	1·84 (1·69, 1·99)	1·82 (1·35, 2·4)	1·72 (1·24, 2·31)	2·11 (2·09, 2·14)	1·84 (1·69, 1·99)	1·57 (1·12, 2·13)	1·54 (1·09, 2·13)
Switzerland	1·52 (1·5, 1·55)	1·5 (1·34, 1·67)	1·5 (1·23, 1·79)	1·43 (1·17, 1·73)	1·52 (1·5, 1·55)	1·5 (1·34, 1·67)	1·36 (1·12, 1·6)	1·34 (1·09, 1·59)
UK	1·84 (1·82, 1·85)	1·73 (1·55, 1·94)	1·7 (1·34, 2·12)	1·61 (1·21, 2·04)	1·84 (1·82, 1·85)	1·73 (1·55, 1·94)	1·6 (1·25, 2·01)	1·6 (1·22, 2·05)
**Latin America and Caribbean**	3·31 (3·19, 3·42)	2·18 (1·99, 2·4)	1·67 (1·4, 2·01)	1·58 (1·22, 2·01)	3·31 (3·19, 3·42)	2·18 (1·99, 2·4)	1·49 (1·21, 1·84)	1·58 (1·18, 2·03)
**Andean Latin America**	4·27 (4·13, 4·41)	2·82 (2·57, 3·1)	2·06 (1·56, 2·67)	1·79 (1·17, 2·51)	4·27 (4·13, 4·41)	2·82 (2·57, 3·1)	1·81 (1·31, 2·44)	1·8 (1·14, 2·52)
Bolivia	5·38 (5·12, 5·64)	3·24 (2·92, 3·61)	2·29 (1·58, 3·16)	1·79 (1·07, 2·71)	5·38 (5·12, 5·64)	3·24 (2·92, 3·61)	2·03 (1·29, 2·92)	1·78 (1·08, 2·73)
Ecuador	3·65 (3·49, 3·82)	2·27 (1·93, 2·67)	1·65 (1·02, 2·46)	1·47 (0·99, 2·25)	3·65 (3·49, 3·82)	2·27 (1·93, 2·67)	1·45 (0·97, 2·21)	1·46 (0·99, 2·24)
Peru	4·25 (4·06, 4·45)	2·96 (2·64, 3·33)	2·15 (1·53, 2·89)	1·75 (1·1, 2·61)	4·25 (4·06, 4·45)	2·96 (2·64, 3·33)	1·87 (1·25, 2·66)	1·75 (1·08, 2·59)
**Caribbean**	2·99 (2·92, 3·06)	2·24 (2·05, 2·44)	1·69 (1·26, 2·29)	1·55 (1·08, 2·22)	2·99 (2·92, 3·06)	2·24 (2·05, 2·44)	1·47 (1·08, 2·06)	1·56 (1·04, 2·25)
Antigua and Barbuda	2·17 (2·07, 2·27)	1·51 (1·28, 1·78)	1·33 (1·06, 1·64)	1·26 (1·01, 1·57)	2·17 (2·07, 2·27)	1·51 (1·28, 1·78)	1·23 (0·98, 1·52)	1·28 (1·01, 1·58)
The Bahamas	2·25 (2·16, 2·34)	1·54 (1·28, 1·85)	1·46 (1·06, 1·97)	1·38 (1·0, 1·88)	2·25 (2·16, 2·34)	1·54 (1·28, 1·85)	1·35 (0·99, 1·8)	1·38 (1·0, 1·85)

Location	Total fertility rate, reference				Total fertility rate – faster met need and education pace			
	**1990**	**2017**	**2050**	**2100**	**1990**	**2017**	**2050**	**2100**
Barbados	1·9 (1·83, 1·97)	1·43 (1·2, 1·7)	1·41 (1·05, 1·84)	1·36 (1·02, 1·78)	1·9 (1·83, 1·97)	1·43 (1·2, 1·7)	1·24 (0·97, 1·64)	1·26 (0·99, 1·7)
Belize	4·7 (4·58, 4·82)	2·23 (1·96, 2·53)	1·48 (1·04, 2·02)	1·32 (1·0, 1·9)	4·7 (4·58, 4·82)	2·23 (1·96, 2·53)	1·32 (0·97, 1·85)	1·33 (1·0, 1·88)
Bermuda	1·58 (1·53, 1·64)	1·3 (1·16, 1·46)	1·44 (1·07, 1·86)	1·33 (1·01, 1·76)	1·58 (1·53, 1·64)	1·3 (1·16, 1·46)	1·29 (0·98, 1·7)	1·32 (1·01, 1·76)
Cuba	1·71 (1·69, 1·74)	1·51 (1·43, 1·6)	1·44 (0·99, 2·24)	1·41 (0·98, 2·24)	1·71 (1·69, 1·74)	1·51 (1·43, 1·6)	1·39 (0·98, 2·19)	1·41 (0·98, 2·2)
Dominica	3·0 (2·87, 3·13)	1·6 (1·35, 1·92)	1·48 (1·0, 2·24)	1·41 (0·99, 2·16)	3·0 (2·87, 3·13)	1·6 (1·35, 1·92)	1·39 (0·98, 2·15)	1·4 (0·99, 2·2)
Dominican Republic	3·56 (3·38, 3·74)	2·37 (2·04, 2·75)	1·67 (1·19, 2·31)	1·46 (1·01, 2·1)	3·56 (3·38, 3·74)	2·37 (2·04, 2·75)	1·43 (0·98, 2·09)	1·46 (1·01, 2·14)
Grenada	3·56 (3·22, 3·89)	1·88 (1·59, 2·23)	1·53 (0·99, 2·37)	1·43 (0·99, 2·24)	3·56 (3·22, 3·89)	1·88 (1·59, 2·23)	1·4 (0·96, 2·17)	1·43 (1·0, 2·25)
Guyana	3·69 (3·54, 3·84)	2·5 (2·16, 2·88)	1·88 (1·53, 2·25)	1·61 (1·27, 1·95)	3·69 (3·54, 3·84)	2·5 (2·16, 2·88)	1·42 (1·11, 1·74)	1·37 (1·05, 1·72)
Haiti	5·36 (5·18, 5·52)	3·14 (2·81, 3·52)	1·9 (1·1, 2·92)	1·42 (0·98, 2·42)	5·36 (5·18, 5·52)	3·14 (2·81, 3·52)	1·62 (0·98, 2·6)	1·43 (1·0, 2·42)
Jamaica	2·9 (2·79, 2·99)	1·58 (1·39, 1·79)	1·41 (1·01, 1·96)	1·36 (1·0, 1·88)	2·9 (2·79, 2·99)	1·58 (1·39, 1·79)	1·34 (0·99, 1·89)	1·36 (1·01, 1·92)
Puerto Rico	2·2 (2·16, 2·24)	1·21 (1·1, 1·33)	1·2 (0·99, 1·48)	1·19 (1·0, 1·46)	2·2 (2·16, 2·24)	1·21 (1·1, 1·33)	1·17 (0·98, 1·46)	1·19 (1·0, 1·48)
Saint Lucia	3·02 (2·89, 3·16)	1·54 (1·28, 1·84)	1·36 (1·03, 1·73)	1·28 (1·01, 1·66)	3·02 (2·89, 3·16)	1·54 (1·28, 1·84)	1·24 (0·97, 1·58)	1·26 (1·01, 1·64)
Saint Vincent and the Grenadines	2·94 (2·73, 3·13)	1·86 (1·56, 2·2)	1·47 (1·0, 2·08)	1·37 (0·99, 1·99)	2·94 (2·73, 3·13)	1·86 (1·56, 2·2)	1·32 (0·96, 1·91)	1·36 (1·0, 1·97)
Suriname	2·94 (2·86, 3·02)	2·2 (1·91, 2·53)	1·74 (1·15, 2·53)	1·5 (1·01, 2·25)	2·94 (2·86, 3·02)	2·2 (1·91, 2·53)	1·4 (0·97, 2·13)	1·39 (0·99, 2·13)
Trinidad and Tobago	2·34 (2·27, 2·42)	1·7 (1·49, 1·94)	1·5 (1·0, 2·23)	1·37 (0·98, 2·11)	2·34 (2·27, 2·42)	1·7 (1·49, 1·94)	1·22 (0·93, 1·88)	1·24 (0·98, 1·97)

Location	Total fertility rate, reference				Total fertility rate – faster met need and education pace			
	1990	2017	2050	2100	1990	2017	2050	2100
Virgin Islands	2·95 (2·81, 3·1)	2·04 (1·74, 2·38)	1·79 (1·05, 2·73)	1·71 (1·03, 2·65)	2·95 (2·81, 3·1)	2·04 (1·74, 2·38)	1·71 (1·01, 2·66)	1·71 (1·03, 2·64)
**Central Latin America**	3·66 (3·51, 3·82)	2·35 (2·12, 2·63)	1·69 (1·45, 1·98)	1·52 (1·23, 1·9)	3·66 (3·51, 3·82)	2·35 (2·12, 2·63)	1·45 (1·22, 1·74)	1·51 (1·18, 1·91)
Colombia	3·34 (3·23, 3·46)	2·12 (1·82, 2·45)	1·49 (1·24, 1·75)	1·45 (1·18, 1·75)	3·34 (3·23, 3·46)	2·12 (1·82, 2·45)	1·42 (1·17, 1·67)	1·45 (1·17, 1·75)
Costa Rica	3·08 (3·02, 3·14)	1·75 (1·61, 1·92)	1·38 (0·98, 1·96)	1·3 (0·99, 1·89)	3·08 (3·02, 3·14)	1·75 (1·61, 1·92)	1·26 (0·95, 1·86)	1·31 (0·99, 1·95)
El Salvador	3·57 (3·45, 3·69)	1·95 (1·7, 2·22)	1·42 (1·1, 1·75)	1·32 (1·02, 1·68)	3·57 (3·45, 3·69)	1·95 (1·7, 2·22)	1·28 (0·99, 1·61)	1·32 (1·03, 1·68)
Guatemala	6·15 (6·11, 6·18)	2·8 (2·41, 3·24)	1·57 (0·98, 2·64)	1·32 (0·96, 2·33)	6·15 (6·11, 6·18)	2·8 (2·41, 3·24)	1·35 (0·94, 2·33)	1·33 (0·97, 2·32)
Honduras	5·26 (5·16, 5·36)	2·89 (2·51, 3·36)	1·74 (1·18, 2·38)	1·48 (1·01, 2·16)	5·26 (5·16, 5·36)	2·89 (2·51, 3·36)	1·49 (1·0, 2·13)	1·48 (1·02, 2·16)
Mexico	3·54 (3·25, 3·81)	2·42 (2·07, 2·86)	1·76 (1·5, 2·05)	1·44 (1·18, 1·71)	3·54 (3·25, 3·81)	2·42 (2·07, 2·86)	1·44 (1·19, 1·7)	1·43 (1·16, 1·74)
Nicaragua	4·76 (4·6, 4·91)	2·46 (2·1, 2·88)	1·54 (0·98, 2·45)	1·44 (0·99, 2·37)	4·76 (4·6, 4·91)	2·46 (2·1, 2·88)	1·41 (0·96, 2·32)	1·45 (0·99, 2·36)
Panama	2·79 (2·67, 2·9)	2·31 (1·97, 2·69)	1·98 (1·61, 2·43)	1·81 (1·39, 2·29)	2·79 (2·67, 2·9)	2·31 (1·97, 2·69)	1·58 (1·24, 2·02)	1·58 (1·2, 2·03)
Venezuela	3·59 (3·56, 3·62)	2·24 (1·99, 2·52)	1·77 (1·2, 2·54)	1·63 (1·07, 2·35)	3·59 (3·56, 3·62)	2·24 (1·99, 2·52)	1·6 (1·05, 2·31)	1·61 (1·07, 2·31)
**Tropical Latin America**	2·78 (2·66, 2·9)	1·78 (1·61, 1·99)	1·45 (1·04, 1·95)	1·47 (1·03, 2·04)	2·78 (2·66, 2·9)	1·78 (1·61, 1·99)	1·42 (1·02, 1·91)	1·47 (1·02, 2·04)
Brazil	2·75 (2·63, 2·87)	1·76 (1·58, 1·97)	1·43 (1·0, 1·95)	1·44 (1·03, 1·97)	2·75 (2·63, 2·87)	1·76 (1·58, 1·97)	1·4 (0·99, 1·9)	1·44 (1·02, 1·97)
Paraguay	3·98 (3·82, 4·14)	2·55 (2·18, 2·97)	1·88 (1·26, 2·67)	1·84 (1·18, 2·6)	3·98 (3·82, 4·14)	2·55 (2·18, 2·97)	1·83 (1·22, 2·6)	1·83 (1·18, 2·61)
**North Africa and Middle East**	4·49 (4·37, 4·62)	2·71 (2·51, 2·94)	2·02 (1·69, 2·48)	1·78 (1·29, 2·49)	4·49 (4·37, 4·62)	2·71 (2·51, 2·94)	1·81 (1·47, 2·24)	1·75 (1·21, 2·51)

Location	Total fertility rate, reference				Total fertility rate – faster met need and education pace			
	1990	2017	2050	2100	1990	2017	2050	2100
**North Africa and Middle East**	4·49 (4·37, 4·62)	2·71 (2·51, 2·94)	2·02 (1·69, 2·48)	1·78 (1·29, 2·49)	4·49 (4·37, 4·62)	2·71 (2·51, 2·94)	1·81 (1·47, 2·24)	1·75 (1·21, 2·51)
Afghanistan	7·23 (7·01, 7·43)	6·01 (5·71, 6·3)	3·33 (2·8, 3·85)	1·65 (1·34, 1·98)	7·23 (7·01, 7·43)	6·01 (5·71, 6·3)	2·82 (2·34, 3·3)	1·43 (1·14, 1·75)
Algeria	4·74 (4·55, 4·93)	2·81 (2·49, 3·12)	2·38 (1·44, 3·55)	2·01 (1·11, 3·17)	4·74 (4·55, 4·93)	2·81 (2·49, 3·12)	2·06 (1·12, 3·23)	1·97 (1·06, 3·19)
Bahrain	3·43 (3·34, 3·51)	2·05 (1·87, 2·22)	1·62 (1·21, 2·1)	1·37 (1·03, 1·8)	3·43 (3·34, 3·51)	2·05 (1·87, 2·22)	1·4 (1·03, 1·82)	1·33 (1·01, 1·8)
Egypt	3·97 (3·8, 4·13)	2·66 (2·43, 2·91)	2·1 (1·31, 3·14)	2·08 (1·24, 3·18)	3·97 (3·8, 4·13)	2·66 (2·43, 2·91)	2·06 (1·22, 3·12)	2·08 (1·2, 3·18)
Iran	3·84 (3·62, 4·08)	1·73 (1·47, 2·03)	1·59 (0·98, 2·77)	1·55 (1·0, 2·77)	3·84 (3·62, 4·08)	1·73 (1·47, 2·03)	1·53 (0·97, 2·68)	1·55 (1·0, 2·77)
Iraq	5·77 (5·56, 5·98)	3·76 (3·42, 4·15)	2·05 (1·61, 2·5)	1·53 (1·1, 1·95)	5·77 (5·56, 5·98)	3·76 (3·42, 4·15)	1·6 (1·21, 2·04)	1·47 (1·04, 1·96)
Jordan	5·24 (5·05, 5·42)	3·05 (2·8, 3·35)	2·15 (1·54, 2·92)	1·7 (1·09, 2·46)	5·24 (5·05, 5·42)	3·05 (2·8, 3·35)	1·82 (1·24, 2·57)	1·61 (1·03, 2·38)
Kuwait	2·02 (1·94, 2·11)	1·42 (1·28, 1·57)	1·54 (0·92, 3·19)	1·47 (0·88, 3·15)	2·02 (1·94, 2·11)	1·42 (1·28, 1·57)	1·47 (0·91, 3·1)	1·47 (0·89, 3·15)
Lebanon	4·34 (3·94, 4·72)	2·4 (2·06, 2·81)	1·69 (1·35, 2·06)	1·45 (1·15, 1·76)	4·34 (3·94, 4·72)	2·4 (2·06, 2·81)	1·52 (1·23, 1·85)	1·44 (1·15, 1·75)
Libya	4·87 (4·54, 5·17)	2·12 (1·78, 2·55)	1·84 (1·0, 3·28)	1·66 (1·0, 3·01)	4·87 (4·54, 5·17)	2·12 (1·78, 2·55)	1·69 (0·96, 3·05)	1·65 (0·99, 2·99)
Morocco	4·02 (3·8, 4·22)	2·14 (1·88, 2·45)	1·52 (1·15, 1·93)	1·39 (1·06, 1·79)	4·02 (3·8, 4·22)	2·14 (1·88, 2·45)	1·43 (1·08, 1·82)	1·39 (1·05, 1·78)
Oman	5·81 (5·42, 6·2)	2·55 (2·3, 2·81)	2·14 (1·57, 2·8)	1·64 (1·08, 2·3)	5·81 (5·42, 6·2)	2·55 (2·3, 2·81)	1·78 (1·22, 2·4)	1·46 (1·01, 2·11)
Palestine	6·28 (6·02, 6·51)	3·49 (3·16, 3·86)	2·13 (1·78, 2·49)	1·8 (1·46, 2·13)	6·28 (6·02, 6·51)	3·49 (3·16, 3·86)	1·67 (1·39, 1·97)	1·59 (1·28, 1·9)
Qatar	4·16 (4·0, 4·32)	2·04 (1·87, 2·22)	1·75 (1·23, 2·38)	1·51 (1·04, 2·12)	4·16 (4·0, 4·32)	2·04 (1·87, 2·22)	1·52 (1·02, 2·11)	1·46 (1·02, 2·09)

Location	Total fertility rate, reference				Total fertility rate – faster met need and education pace			
	**1990**	**2017**	**2050**	**2100**	**1990**	**2017**	**2050**	**2100**
Saudi Arabia	6·57 (6·22, 6·91)	1·67 (1·47, 1·88)	1·57 (0·96, 2·7)	1·39 (0·97, 2·49)	6·57 (6·22, 6·91)	1·67 (1·47, 1·88)	1·41 (0·94, 2·52)	1·34 (0·97, 2·39)
Sudan	6·84 (6·56, 7·1)	4·22 (3·86, 4·62)	1·85 (1·56, 2·17)	1·45 (1·16, 1·74)	6·84 (6·56, 7·1)	4·22 (3·86, 4·62)	1·58 (1·32, 1·87)	1·33 (1·07, 1·62)
Syria	4·66 (4·31, 5·01)	2·17 (1·88, 2·51)	1·53 (0·98, 2·37)	1·38 (0·95, 2·18)	4·66 (4·31, 5·01)	2·17 (1·88, 2·51)	1·34 (0·94, 2·12)	1·32 (0·94, 2·13)
Tunisia	3·55 (3·36, 3·75)	1·77 (1·52, 2·09)	1·7 (1·11, 2·48)	1·52 (1·01, 2·29)	3·55 (3·36, 3·75)	1·77 (1·52, 2·09)	1·56 (1·02, 2·35)	1·52 (1·01, 2·29)
Turkey	3·25 (3·11, 3·4)	1·79 (1·61, 2·0)	1·51 (1·17, 1·89)	1·34 (1·01, 1·73)	3·25 (3·11, 3·4)	1·79 (1·61, 2·0)	1·34 (0·99, 1·7)	1·3 (1·01, 1·68)
United Arab Emirates	4·1 (3·87, 4·34)	1·31 (1·17, 1·49)	1·32 (0·98, 1·76)	1·27 (1·0, 1·72)	4·1 (3·87, 4·34)	1·31 (1·17, 1·49)	1·28 (0·97, 1·73)	1·27 (1·0, 1·72)
Yemen	7·81 (7·58, 8·03)	4·53 (4·16, 4·96)	1·68 (1·27, 2·11)	1·39 (1·02, 1·82)	7·81 (7·58, 8·03)	4·53 (4·16, 4·96)	1·64 (1·23, 2·06)	1·39 (1·02, 1·82)
**South Asia**	4·2 (4·1, 4·3)	2·27 (2·04, 2·54)	1·39 (1·05, 1·88)	1·33 (0·96, 1·9)	4·2 (4·1, 4·3)	2·27 (2·04, 2·54)	1·27 (0·99, 1·74)	1·31 (0·96, 1·87)
**South Asia**	4·2 (4·1, 4·3)	2·27 (2·04, 2·54)	1·39 (1·05, 1·88)	1·33 (0·96, 1·9)	4·2 (4·1, 4·3)	2·27 (2·04, 2·54)	1·27 (0·99, 1·74)	1·31 (0·96, 1·87)
Bangladesh	4·62 (4·51, 4·73)	2·0 (1·81, 2·22)	1·15 (0·95, 1·54)	1·19 (0·99, 1·59)	4·62 (4·51, 4·73)	2·0 (1·81, 2·22)	1·14 (0·95, 1·54)	1·19 (0·99, 1·58)
Bhutan	4·63 (4·17, 5·12)	1·98 (1·76, 2·27)	1·34 (0·99, 1·81)	1·35 (1·02, 1·83)	4·63 (4·17, 5·12)	1·98 (1·76, 2·27)	1·32 (0·99, 1·8)	1·35 (1·03, 1·85)
India	3·93 (3·83, 4·04)	2·14 (1·93, 2·39)	1·37 (0·97, 1·93)	1·29 (0·99, 1·89)	3·93 (3·83, 4·04)	2·14 (1·93, 2·39)	1·24 (0·94, 1·78)	1·28 (0·98, 1·84)
Nepal	5·03 (4·82, 5·22)	2·21 (1·96, 2·52)	1·19 (0·92, 1·88)	1·2 (0·98, 1·91)	5·03 (4·82, 5·22)	2·21 (1·96, 2·52)	1·16 (0·91, 1·82)	1·21 (0·98, 1·91)
Pakistan	6·09 (5·85, 6·29)	3·4 (2·99, 3·9)	1·59 (1·04, 2·33)	1·31 (0·99, 2·04)	6·09 (5·85, 6·29)	3·4 (2·99, 3·9)	1·45 (0·99, 2·19)	1·3 (0·99, 2·04)
**Southeast Asia, East Asia, and Oceania**	2·41 (2·31, 2·51)	1·72 (1·63, 1·83)	1·57 (1·22, 2·24)	1·61 (1·14, 2·44)	2·41 (2·31, 2·51)	1·72 (1·63, 1·83)	1·49 (1·13, 2·17)	1·58 (1·08, 2·44)

Location	Total fertility rate, reference				Total fertility rate – faster met need and education pace			
	1990	2017	2050	2100	1990	2017	2050	2100
**East Asia**	2·12 (2·0, 2·25)	1·52 (1·43, 1·61)	1·44 (0·99, 2·48)	1·47 (0·91, 2·66)	2·12 (2·0, 2·25)	1·52 (1·43, 1·61)	1·43 (0·98, 2·47)	1·47 (0·9, 2·68)
China	2·12 (2·0, 2·26)	1·53 (1·43, 1·63)	1·44 (0·97, 2·56)	1·47 (0·96, 2·55)	2·12 (2·0, 2·26)	1·53 (1·43, 1·63)	1·44 (0·96, 2·56)	1·47 (0·97, 2·57)
North Korea	2·43 (2·16, 2·72)	1·32 (1·17, 1·51)	1·38 (1·19, 1·6)	1·3 (1·09, 1·53)	2·43 (2·16, 2·72)	1·32 (1·17, 1·51)	1·24 (1·05, 1·46)	1·26 (1·06, 1·48)
Taiwan (province of China)	1·76 (1·74, 1·77)	1·04 (0·92, 1·19)	1·31 (0·99, 1·81)	1·3 (0·98, 1·8)	1·76 (1·74, 1·77)	1·04 (0·92, 1·19)	1·3 (0·99, 1·81)	1·3 (0·98, 1·8)
**Oceania**	4·56 (4·41, 4·7)	4·02 (3·67, 4·37)	2·82 (2·39, 3·25)	1·99 (1·58, 2·56)	4·56 (4·41, 4·7)	4·02 (3·67, 4·37)	1·84 (1·58, 2·09)	1·49 (1·18, 1·98)
American Samoa	4·36 (4·18, 4·54)	2·92 (2·55, 3·34)	2·33 (1·57, 3·3)	2·13 (1·37, 3·06)	4·36 (4·18, 4·54)	2·92 (2·55, 3·34)	1·86 (1·2, 2·68)	1·8 (1·12, 2·63)
Federated States of Micronesia	4·47 (4·24, 4·69)	2·72 (2·42, 3·07)	2·15 (1·47, 2·93)	1·79 (1·14, 2·58)	4·47 (4·24, 4·69)	2·72 (2·42, 3·07)	1·71 (1·1, 2·44)	1·57 (1·02, 2·33)
Fiji	3·05 (2·91, 3·17)	2·61 (2·3, 2·96)	2·26 (1·74, 2·86)	1·98 (1·48, 2·51)	3·05 (2·91, 3·17)	2·61 (2·3, 2·96)	1·8 (1·38, 2·27)	1·72 (1·3, 2·22)
Guam	3·31 (3·19, 3·42)	2·95 (2·71, 3·19)	2·51 (1·21, 4·21)	2·26 (1·06, 3·95)	3·31 (3·19, 3·42)	2·95 (2·71, 3·19)	2·1 (1·01, 3·75)	2·07 (1·02, 3·85)
Kiribati	4·42 (4·16, 4·69)	3·71 (3·26, 4·2)	2·68 (2·17, 3·22)	2·32 (1·82, 2·9)	4·42 (4·16, 4·69)	3·71 (3·26, 4·2)	1·82 (1·42, 2·3)	1·49 (1·08, 1·93)
Marshall Islands	4·35 (4·21, 4·51)	2·86 (2·55, 3·22)	1·92 (1·5, 2·38)	1·75 (1·31, 2·2)	4·35 (4·21, 4·51)	2·86 (2·55, 3·22)	1·71 (1·33, 2·15)	1·76 (1·33, 2·23)
Northern Mariana Islands	2·48 (2·24, 2·74)	2·06 (1·82, 2·31)	1·86 (1·41, 2·34)	1·72 (1·32, 2·18)	2·48 (2·24, 2·74)	2·06 (1·82, 2·31)	1·57 (1·19, 1·97)	1·58 (1·19, 2·0)
Papua New Guinea	4·92 (4·77, 5·07)	4·21 (3·83, 4·59)	2·85 (2·35, 3·35)	1·83 (1·52, 2·18)	4·92 (4·77, 5·07)	4·21 (3·83, 4·59)	1·82 (1·51, 2·14)	1·39 (1·14, 1·62)
Samoa	3·43 (3·01, 3·85)	4·69 (4·23, 5·22)	5·07 (2·96, 7·81)	4·47 (2·26, 7·32)	3·43 (3·01, 3·85)	4·69 (4·23, 5·22)	3·51 (1·48, 6·16)	2·73 (1·05, 5·41)
Solomon Islands	5·89 (5·56, 6·24)	4·2 (3·8, 4·64)	2·64 (2·05, 3·28)	1·91 (1·44, 2·43)	5·89 (5·56, 6·24)	4·2 (3·8, 4·64)	1·78 (1·37, 2·27)	1·5 (1·11, 1·89)

Location	Total fertility rate, reference				Total fertility rate – faster met need and education pace			
	1990	2017	2050	2100	1990	2017	2050	2100
Tonga	3·95 (3·8, 4·11)	3·17 (2·77, 3·61)	2·82 (2·02, 3·86)	2·62 (1·81, 3·67)	3·95 (3·8, 4·11)	3·17 (2·77, 3·61)	2·03 (1·24, 3·01)	1·56 (1·0, 2·49)
Vanuatu	4·85 (4·46, 5·22)	3·73 (3·41, 4·12)	2·42 (2·03, 2·82)	1·77 (1·41, 2·13)	4·85 (4·46, 5·22)	3·73 (3·41, 4·12)	1·67 (1·35, 2·02)	1·48 (1·14, 1·84)
**Southeast Asia**	3·18 (3·09, 3·27)	2·08 (1·88, 2·32)	1·7 (1·38, 2·1)	1·61 (1·21, 2·07)	3·18 (3·09, 3·27)	2·08 (1·88, 2·32)	1·54 (1·23, 1·95)	1·58 (1·17, 2·08)
Cambodia	5·66 (5·52, 5·79)	2·73 (2·49, 3·04)	1·47 (1·04, 2·03)	1·3 (1·0, 1·9)	5·66 (5·52, 5·79)	2·73 (2·49, 3·04)	1·4 (1·0, 1·96)	1·31 (1·0, 1·9)
Indonesia	2·98 (2·88, 3·08)	1·97 (1·7, 2·3)	1·59 (1·06, 2·29)	1·51 (1·01, 2·21)	2·98 (2·88, 3·08)	1·97 (1·7, 2·3)	1·5 (1·01, 2·22)	1·51 (1·02, 2·24)
Laos	5·16 (5·01, 5·29)	2·9 (2·64, 3·21)	1·44 (1·15, 1·74)	1·28 (1·01, 1·6)	5·16 (5·01, 5·29)	2·9 (2·64, 3·21)	1·29 (1·02, 1·59)	1·28 (1·02, 1·61)
Malaysia	3·46 (3·44, 3·48)	2·02 (1·81, 2·26)	1·82 (1·44, 2·24)	1·64 (1·26, 2·08)	3·46 (3·44, 3·48)	2·02 (1·81, 2·26)	1·68 (1·35, 2·07)	1·64 (1·27, 2·07)
Maldives	5·08 (4·89, 5·26)	1·87 (1·72, 2·02)	1·56 (0·98, 2·54)	1·38 (0·96, 2·36)	5·08 (4·89, 5·26)	1·87 (1·72, 2·02)	1·42 (0·95, 2·41)	1·35 (0·96, 2·32)
Mauritius	2·28 (2·22, 2·33)	1·32 (1·22, 1·44)	1·33 (0·97, 1·91)	1·27 (0·97, 1·88)	2·28 (2·22, 2·33)	1·32 (1·22, 1·44)	1·25 (0·97, 1·84)	1·26 (0·97, 1·89)
Myanmar	3·51 (3·18, 3·82)	2·02 (1·85, 2·21)	1·43 (1·05, 1·9)	1·38 (1·03, 1·84)	3·51 (3·18, 3·82)	2·02 (1·85, 2·21)	1·37 (1·0, 1·83)	1·38 (1·03, 1·86)
Philippines	4·38 (4·22, 4·55)	3·12 (2·85, 3·45)	2·25 (1·79, 2·77)	1·78 (1·3, 2·3)	4·38 (4·22, 4·55)	3·12 (2·85, 3·45)	1·89 (1·43, 2·38)	1·7 (1·2, 2·22)
Sri Lanka	2·6 (2·44, 2·77)	1·8 (1·52, 2·11)	1·69 (1·34, 2·08)	1·46 (1·11, 1·87)	2·6 (2·44, 2·77)	1·8 (1·52, 2·11)	1·51 (1·16, 1·89)	1·45 (1·1, 1·86)
Seychelles	2·64 (2·52, 2·77)	2·15 (1·9, 2·43)	1·81 (1·11, 2·65)	1·68 (1·05, 2·54)	2·64 (2·52, 2·77)	2·15 (1·9, 2·43)	1·68 (1·02, 2·51)	1·67 (1·04, 2·56)
Thailand	2·15 (2·04, 2·26)	1·21 (1·07, 1·38)	1·26 (0·98, 1·71)	1·28 (1·0, 1·76)	2·15 (2·04, 2·26)	1·21 (1·07, 1·38)	1·26 (0·98, 1·71)	1·28 (1·0, 1·77)
Timor-Leste	6·32 (6·03, 6·6)	4·14 (3·63, 4·69)	2·16 (1·72, 2·6)	1·81 (1·39, 2·26)	6·32 (6·03, 6·6)	4·14 (3·63, 4·69)	1·68 (1·3, 2·08)	1·46 (1·11, 1·89)

Location	Total fertility rate, reference				Total fertility rate – faster met need and education pace			
	**1990**	**2017**	**2050**	**2100**	**1990**	**2017**	**2050**	**2100**
Vietnam	3·02 (2·94, 3·11)	1·85 (1·68, 2·05)	1·5 (1·09, 1·97)	1·39 (1·02, 1·86)	3·02 (2·94, 3·11)	1·85 (1·68, 2·05)	1·39 (1·0, 1·86)	1·39 (1·02, 1·87)
**Sub-Saharan Africa**	6·12 (5·95, 6·29)	4·62 (4·33, 4·93)	2·54 (2·29, 2·81)	1·73 (1·42, 2·06)	6·12 (5·95, 6·29)	4·62 (4·33, 4·93)	2·09 (1·86, 2·33)	1·6 (1·27, 1·95)
**Central sub-Saharan Africa**	6·56 (6·33, 6·78)	4·88 (4·62, 5·13)	2·38 (2·02, 2·76)	1·7 (1·29, 2·18)	6·56 (6·33, 6·78)	4·88 (4·62, 5·13)	1·94 (1·62, 2·3)	1·45 (1·11, 1·87)
Angola	7·02 (6·64, 7·38)	5·12 (4·72, 5·54)	2·17 (1·89, 2·44)	1·55 (1·3, 1·81)	7·02 (6·64, 7·38)	5·12 (4·72, 5·54)	1·89 (1·64, 2·15)	1·49 (1·24, 1·74)
Central African Republic	5·66 (5·24, 6·07)	3·56 (3·18, 4·0)	1·55 (1·31, 1·82)	1·34 (1·1, 1·63)	5·66 (5·24, 6·07)	3·56 (3·18, 4·0)	1·33 (1·1, 1·59)	1·17 (1·0, 1·44)
Congo (Brazzaville)	5·33 (5·07, 5·58)	3·3 (2·99, 3·66)	1·53 (1·24, 1·86)	1·29 (1·02, 1·63)	5·33 (5·07, 5·58)	3·3 (2·99, 3·66)	1·41 (1·12, 1·73)	1·29 (1·02, 1·62)
Democratic Republic of the Congo	6·62 (6·41, 6·83)	5·05 (4·73, 5·37)	2·53 (2·03, 3·07)	1·75 (1·27, 2·3)	6·62 (6·41, 6·83)	5·05 (4·73, 5·37)	2·0 (1·54, 2·53)	1·41 (1·02, 1·9)
Equatorial Guinea	6·88 (6·53, 7·22)	3·88 (3·36, 4·44)	1·82 (1·53, 2·14)	1·39 (1·21, 1·55)	6·88 (6·53, 7·22)	3·88 (3·36, 4·44)	1·7 (1·44, 2·0)	1·35 (1·2, 1·5)
Gabon	4·95 (4·45, 5·43)	2·79 (2·47, 3·17)	1·52 (1·23, 1·84)	1·3 (1·03, 1·62)	4·95 (4·45, 5·43)	2·79 (2·47, 3·17)	1·42 (1·14, 1·72)	1·3 (1·02, 1·61)
**Eastern sub-Saharan Africa**	6·6 (6·45, 6·74)	4·65 (4·36, 4·98)	2·31 (2·07, 2·56)	1·73 (1·43, 2·04)	6·6 (6·45, 6·74)	4·65 (4·36, 4·98)	1·91 (1·69, 2·14)	1·55 (1·24, 1·88)
Burundi	6·39 (6·21, 6·57)	5·3 (4·99, 5·65)	3·42 (2·49, 4·43)	1·61 (1·0, 2·53)	6·39 (6·21, 6·57)	5·3 (4·99, 5·65)	2·38 (1·6, 3·28)	1·53 (1·0, 2·44)
Comoros	6·15 (5·84, 6·44)	3·39 (2·92, 3·9)	1·7 (1·29, 2·2)	1·39 (1·03, 1·88)	6·15 (5·84, 6·44)	3·39 (2·92, 3·9)	1·46 (1·06, 1·92)	1·26 (1·0, 1·74)
Djibouti	6·41 (6·14, 6·67)	3·82 (3·33, 4·34)	1·6 (1·24, 1·98)	1·33 (1·04, 1·71)	6·41 (6·14, 6·67)	3·82 (3·33, 4·34)	1·48 (1·13, 1·85)	1·33 (1·03, 1·72)
Eritrea	6·57 (6·22, 6·91)	4·03 (3·55, 4·57)	1·62 (1·39, 1·89)	1·27 (1·12, 1·44)	6·57 (6·22, 6·91)	4·03 (3·55, 4·57)	1·51 (1·31, 1·75)	1·26 (1·11, 1·41)
Ethiopia	6·96 (6·74, 7·18)	4·79 (4·42, 5·2)	1·79 (1·5, 2·12)	1·33 (1·04, 1·63)	6·96 (6·74, 7·18)	4·79 (4·42, 5·2)	1·72 (1·42, 2·03)	1·34 (1·04, 1·64)

Location	Total fertility rate, reference				Total fertility rate – faster met need and education pace			
	**1990**	**2017**	**2050**	**2100**	**1990**	**2017**	**2050**	**2100**
Kenya	5·76 (5·52, 5·98)	3·38 (2·95, 3·84)	1·75 (1·18, 2·4)	1·59 (1·03, 2·29)	5·76 (5·52, 5·98)	3·38 (2·95, 3·84)	1·57 (1·02, 2·21)	1·59 (1·03, 2·28)
Madagascar	6·19 (5·93, 6·44)	4·89 (4·36, 5·45)	3·12 (2·45, 3·89)	1·61 (1·16, 2·15)	6·19 (5·93, 6·44)	4·89 (4·36, 5·45)	1·97 (1·46, 2·55)	1·54 (1·07, 2·08)
Malawi	6·86 (6·74, 6·99)	4·46 (4·16, 4·79)	1·85 (1·56, 2·13)	1·6 (1·29, 1·92)	6·86 (6·74, 6·99)	4·46 (4·16, 4·79)	1·66 (1·42, 1·93)	1·61 (1·28, 1·94)
Mozambique	6·06 (5·92, 6·19)	4·16 (3·84, 4·49)	1·52 (1·26, 1·8)	1·26 (1·01, 1·52)	6·06 (5·92, 6·19)	4·16 (3·84, 4·49)	1·39 (1·13, 1·66)	1·24 (1·01, 1·49)
Rwanda	6·54 (6·38, 6·69)	4·43 (4·01, 4·91)	2·48 (1·89, 3·12)	1·49 (1·01, 2·08)	6·54 (6·38, 6·69)	4·43 (4·01, 4·91)	1·91 (1·34, 2·53)	1·49 (1·01, 2·07)
Somalia	7·4 (7·04, 7·73)	6·1 (5·72, 6·5)	3·53 (3·06, 4·02)	2·57 (2·24, 2·88)	7·4 (7·04, 7·73)	6·1 (5·72, 6·5)	3·12 (2·62, 3·64)	1·5 (1·32, 1·72)
South Sudan	6·26 (5·8, 6·69)	5·93 (5·56, 6·35)	4·06 (3·35, 4·74)	2·46 (2·07, 2·83)	6·26 (5·8, 6·69)	5·93 (5·56, 6·35)	3·58 (2·76, 4·32)	1·43 (1·15, 1·74)
Tanzania	6·37 (6·24, 6·52)	4·79 (4·44, 5·18)	2·73 (2·19, 3·3)	1·6 (1·12, 2·1)	6·37 (6·24, 6·52)	4·79 (4·44, 5·18)	1·88 (1·41, 2·4)	1·56 (1·04, 2·08)
Uganda	7·45 (7·33, 7·58)	5·24 (4·99, 5·51)	2·26 (2·05, 2·46)	1·72 (1·48, 1·97)	7·45 (7·33, 7·58)	5·24 (4·99, 5·51)	1·98 (1·81, 2·17)	1·72 (1·47, 1·98)
Zambia	6·55 (6·19, 6·89)	4·68 (4·21, 5·22)	2·21 (1·76, 2·7)	1·69 (1·23, 2·12)	6·55 (6·19, 6·89)	4·68 (4·21, 5·22)	1·8 (1·39, 2·23)	1·69 (1·25, 2·16)
**Southern sub-Saharan Africa**	3·54 (3·28, 3·8)	2·62 (2·38, 2·9)	2·08 (1·68, 2·52)	1·92 (1·39, 2·54)	3·54 (3·28, 3·8)	2·62 (2·38, 2·9)	1·82 (1·41, 2·28)	1·9 (1·35, 2·58)
Botswana	4·12 (4·03, 4·22)	2·36 (2·2, 2·53)	1·67 (1·24, 2·18)	1·61 (1·15, 2·15)	4·12 (4·03, 4·22)	2·36 (2·2, 2·53)	1·61 (1·18, 2·11)	1·61 (1·14, 2·14)
eSwatini	5·09 (4·9, 5·28)	3·04 (2·65, 3·51)	1·85 (1·23, 2·55)	1·63 (1·03, 2·35)	5·09 (4·9, 5·28)	3·04 (2·65, 3·51)	1·62 (1·02, 2·3)	1·64 (1·03, 2·36)
Lesotho	4·55 (4·4, 4·69)	2·87 (2·63, 3·16)	1·81 (1·5, 2·17)	1·57 (1·24, 1·91)	4·55 (4·4, 4·69)	2·87 (2·63, 3·16)	1·56 (1·26, 1·89)	1·57 (1·22, 1·95)
Namibia	4·88 (4·77, 4·98)	3·01 (2·76, 3·3)	1·94 (1·65, 2·27)	1·79 (1·44, 2·16)	4·88 (4·77, 4·98)	3·01 (2·76, 3·3)	1·79 (1·5, 2·13)	1·78 (1·41, 2·19)

Location	Total fertility rate, reference				Total fertility rate – faster met need and education pace			
	**1990**	**2017**	**2050**	**2100**	**1990**	**2017**	**2050**	**2100**
South Africa	3·04 (2·71, 3·37)	2·29 (2·05, 2·61)	1·88 (1·49, 2·29)	1·7 (1·28, 2·19)	3·04 (2·71, 3·37)	2·29 (2·05, 2·61)	1·67 (1·29, 2·11)	1·68 (1·25, 2·15)
Zimbabwe	5·06 (4·96, 5·17)	3·78 (3·53, 4·04)	2·58 (1·82, 3·48)	2·22 (1·46, 3·12)	5·06 (4·96, 5·17)	3·78 (3·53, 4·04)	2·21 (1·44, 3·07)	2·22 (1·39, 3·21)
**Western sub-Saharan Africa**	6·31 (6·11, 6·51)	4·94 (4·63, 5·27)	2·82 (2·5, 3·19)	1·7 (1·38, 2·07)	6·31 (6·11, 6·51)	4·94 (4·63, 5·27)	2·31 (2·03, 2·62)	1·63 (1·27, 2·02)
Benin	6·49 (6·29, 6·67)	4·78 (4·44, 5·17)	2·09 (1·72, 2·47)	1·33 (1·07, 1·63)	6·49 (6·29, 6·67)	4·78 (4·44, 5·17)	1·96 (1·61, 2·33)	1·3 (1·04, 1·58)
Burkina Faso	6·87 (6·73, 7·02)	5·4 (5·07, 5·77)	2·81 (2·17, 3·4)	1·41 (1·04, 1·8)	6·87 (6·73, 7·02)	5·4 (5·07, 5·77)	2·56 (2·0, 3·11)	1·41 (1·04, 1·79)
Cameroon	5·76 (5·51, 6·0)	3·94 (3·51, 4·42)	1·61 (1·28, 1·96)	1·41 (1·06, 1·81)	5·76 (5·51, 6·0)	3·94 (3·51, 4·42)	1·56 (1·22, 1·92)	1·41 (1·06, 1·8)
Cape Verde	4·49 (4·34, 4·64)	2·19 (1·82, 2·61)	1·39 (0·97, 2·12)	1·33 (0·99, 2·01)	4·49 (4·34, 4·64)	2·19 (1·82, 2·61)	1·34 (0·96, 2·02)	1·33 (1·0, 2·03)
Chad	7·46 (7·25, 7·65)	6·72 (6·42, 7·04)	4·28 (3·75, 4·82)	2·19 (1·9, 2·54)	7·46 (7·25, 7·65)	6·72 (6·42, 7·04)	3·61 (3·05, 4·17)	1·58 (1·36, 1·8)
Côte d'Ivoire	6·35 (6·02, 6·68)	4·5 (4·13, 4·93)	2·24 (1·47, 3·2)	1·35 (0·98, 2·24)	6·35 (6·02, 6·68)	4·5 (4·13, 4·93)	1·76 (1·1, 2·6)	1·36 (1·0, 2·21)
The Gambia	6·39 (5·97, 6·76)	4·14 (3·69, 4·66)	1·81 (1·55, 2·09)	1·32 (1·09, 1·56)	6·39 (5·97, 6·76)	4·14 (3·69, 4·66)	1·55 (1·31, 1·79)	1·26 (1·05, 1·48)
Ghana	5·21 (4·96, 5·45)	3·47 (3·02, 3·98)	1·93 (1·46, 2·48)	1·43 (1·01, 1·95)	5·21 (4·96, 5·45)	3·47 (3·02, 3·98)	1·68 (1·24, 2·19)	1·41 (1·02, 1·91)
Guinea	6·78 (6·55, 6·99)	4·63 (4·38, 4·9)	2·04 (1·62, 2·5)	1·36 (1·03, 1·73)	6·78 (6·55, 6·99)	4·63 (4·38, 4·9)	1·79 (1·41, 2·23)	1·25 (1·0, 1·6)
Guinea-Bissau	6·84 (6·43, 7·21)	4·63 (4·23, 5·04)	1·82 (1·16, 2·55)	1·33 (1·0, 2·06)	6·84 (6·43, 7·21)	4·63 (4·23, 5·04)	1·72 (1·08, 2·45)	1·33 (1·0, 2·03)
Liberia	6·49 (6·24, 6·74)	4·25 (3·84, 4·74)	1·67 (1·42, 1·93)	1·32 (1·08, 1·57)	6·49 (6·24, 6·74)	4·25 (3·84, 4·74)	1·54 (1·32, 1·79)	1·33 (1·1, 1·59)
Mali	7·36 (7·19, 7·52)	6·02 (5·72, 6·37)	3·22 (2·63, 3·73)	1·47 (1·13, 1·82)	7·36 (7·19, 7·52)	6·02 (5·72, 6·37)	2·91 (2·33, 3·44)	1·46 (1·13, 1·81)

Location	Total fertility rate, reference				Total fertility rate – faster met need and education pace			
	1990	2017	2050	2100	1990	2017	2050	2100
Mauritania	6·07 (5·86, 6·28)	4·15 (3·85, 4·5)	1·84 (1·21, 2·55)	1·34 (0·99, 2·02)	6·07 (5·86, 6·28)	4·15 (3·85, 4·5)	1·68 (1·12, 2·33)	1·35 (0·99, 2·02)
Niger	7·83 (7·69, 7·95)	7·08 (6·76, 7·43)	4·69 (3·72, 5·59)	1·79 (1·34, 2·25)	7·83 (7·69, 7·95)	7·08 (6·76, 7·43)	3·95 (3·0, 4·86)	1·77 (1·31, 2·25)
Nigeria	6·21 (5·95, 6·46)	5·11 (4·71, 5·51)	2·86 (2·39, 3·39)	1·69 (1·25, 2·16)	6·21 (5·95, 6·46)	5·11 (4·71, 5·51)	2·19 (1·78, 2·65)	1·68 (1·24, 2·17)
São Tomé and Príncipe	5·89 (5·72, 6·06)	3·25 (2·83, 3·71)	1·37 (1·03, 1·75)	1·25 (1·0, 1·63)	5·89 (5·72, 6·06)	3·25 (2·83, 3·71)	1·28 (0·98, 1·65)	1·24 (1·0, 1·63)
Senegal	6·32 (5·92, 6·69)	4·57 (4·23, 4·97)	2·2 (1·6, 2·85)	1·34 (1·0, 1·97)	6·32 (5·92, 6·69)	4·57 (4·23, 4·97)	1·83 (1·31, 2·47)	1·35 (1·0, 2·0)
Sierra Leone	6·06 (5·73, 6·38)	4·25 (3·83, 4·7)	1·68 (1·33, 2·08)	1·28 (1·02, 1·66)	6·06 (5·73, 6·38)	4·25 (3·83, 4·7)	1·58 (1·24, 1·98)	1·27 (1·02, 1·65)
Togo	6·05 (5·88, 6·22)	3·82 (3·54, 4·12)	1·55 (1·14, 1·95)	1·27 (1·0, 1·68)	6·05 (5·88, 6·22)	3·82 (3·54, 4·12)	1·47 (1·07, 1·87)	1·27 (1·0, 1·67)

Section 6 Completed cohort fertility versus total fertility rate

Figure 7. Total fertility rate (TFR) and completed cohort fertility (CCF) through 2017 for five sample countries. Estimates are from the Global Burden of Disease Study 2017. TFR is defined as the rate per female of childbearing age (10–54 years). These countries were selected to show the relative stability of CCF50 and TFR in countries with high versus low fertility rates.

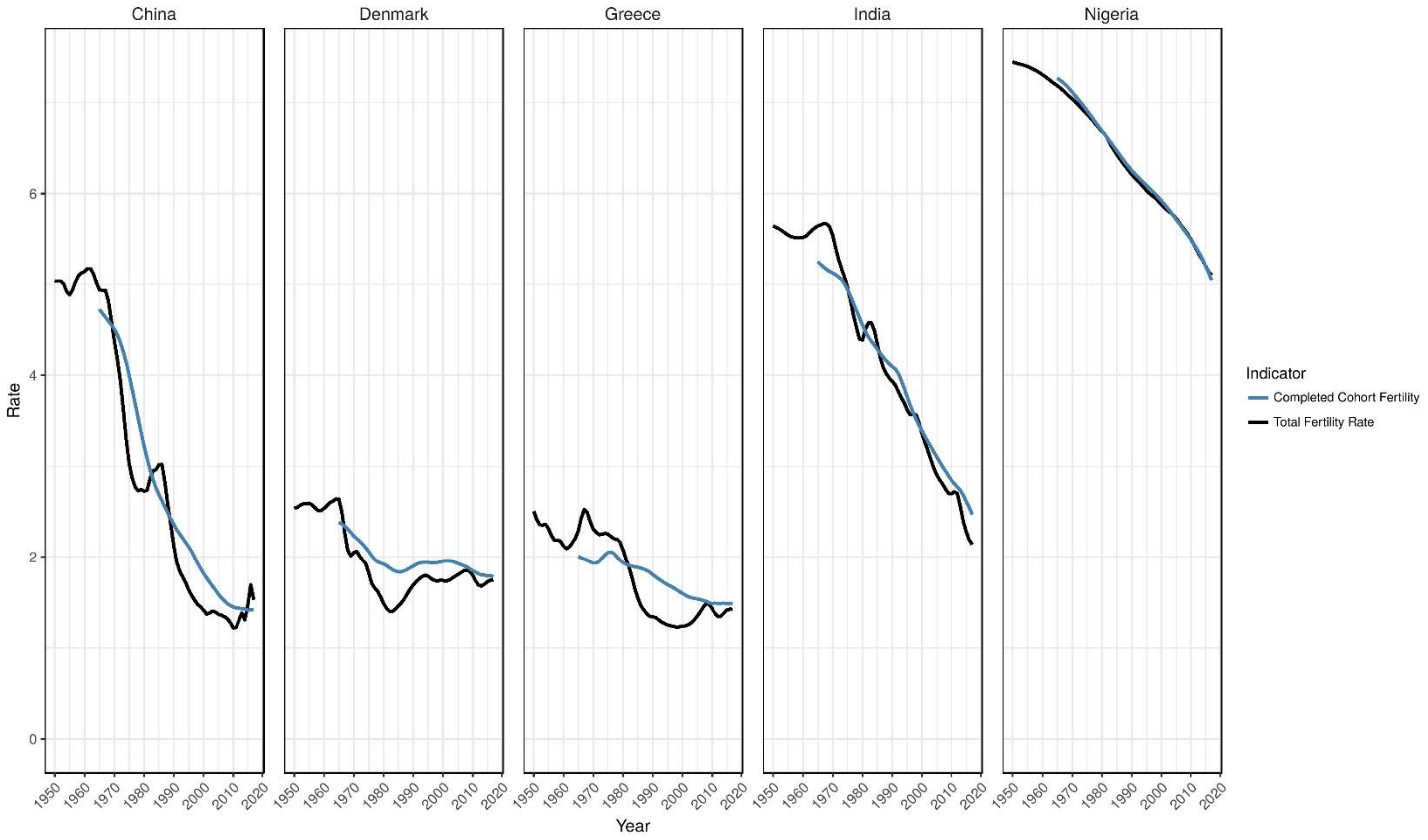

Section 7 Predictive validity of fertility model

We evaluated our fertility forecasting methodology using out-of-time predictive validation for the years 2008–2017 (table 3). We held out both fertility and input covariates in our hold out samples, thereby testing the accuracy in the combined forecasting of education, met need for contraception, and fertility. Forecast error in location- and year-specific TFR was evaluated according to the following metrics, where $TFR_{l,t}$ and $\widehat{TFR}_{l,t}$ denote the GBD 2017 TFR and forecasted TFR, respectively, for location (country or territory) l and year t. The number of locations is $L = 195$ and the total number of holdout years is $T = 10$ for these applied to all locations. Additionally, we subset on locations with below replacement fertility (TFR < 2.1) as of 2017.

- Root mean square error (RMSE): $\sqrt{\frac{1}{LT}\sum_{l,t}\left(TFR_{l,t} - \widehat{TFR}_{l,t}\right)^2}$
- Mean absolute percentage error: $\frac{1}{LT}\sum_{l,t}\frac{\left|TFR_{l,t}-\widehat{TFR}_{l,t}\right|}{TFR_{l,t}} \times 100\%$
- Median absolute deviation: $median_{l,t}\left|TFR_{l,t} - \widehat{TFR}_{l,t}\right|$
- Mean absolute deviation: $\frac{1}{LT}\sum_{l,t}\left|TFR_{l,t} - \widehat{TFR}_{l,t}\right|$

To compare our out-of-time predictive performance to the UNPD 2019 model, we re-estimated their model with the bayesTFR software[2] using GBD 2017 past data from 1980-2007 in order to allow for direct comparisons in forecast accuracy metrics. These years correspond to those used in holdout estimation with our data, since the earliest data on contraceptive met need began in 1980. We recomputed the same diagnostics using the midpoints of the five-year interval forecasts (table 3).

Table 4. Out-of-time forecast errors in TFR for holdout years 2008-2017

	Model	RMSE	Mean absolute % error	Median absolute deviation	Mean absolute error
**All locations**	**IHME**	**0.29**	**7.35**	**0.13**	**0.20**
	UNPD 2019	0.31	10.03	0.20	0.24
**Below replacement locations (2017)**	**IHME**	**0.16**	**6.90**	**0.08**	**0.11**
	UNPD 2019	0.22	11.10	0.16	0.18

Table 5. Omega weights and root mean squared error (RMSE) for selected forecasting risk factors, education, met need for contraception, and vehicles per capita. For education, met need for contraception, and vehicles per capita, values are the omega weights selected. For risk factors, omega values are the mean, 2.5th percentile, and 97.5th percentile of the distribution of omega values and RMSEs are the mean value from the distribution. Additional covariates in the model that did not use omega weight selection for forecasting include: diphtheria, tetanus, pertussis dose 3 vaccination coverage, *Haemophilus influenzae* type B vaccination coverage, measles conjugate vaccination coverage, pneumococcal conjugate vaccination coverage, rotavirus vaccination coverage, anti-retroviral therapy price, prevention of mother-to-child transmission coverage, total HIV spending on care and treatment, and co-trimoxazole coverage.

Independent driver	Weight distribution summary	Mean RMSE
Childhood sexual abuse	0.000 (0.000 to 0.000)	0.001
Childhood sexual abuse against females	0.000 (0.000 to 0.000)	0.002
Childhood sexual abuse against males	0.000 (0.000 to 0.000)	0.001
Intimate partner violence	0.130 (0.000 to 0.250)	0.009
Intimate partner violence (exposure approach)	0.130 (0.000 to 0.250)	0.009
Low physical activity	0.000 (0.000 to 0.000)	0.000
Household air pollution from solid fuels	1.434 (0.000 to 2.750)	0.021
Ambient ozone pollution	1.417 (0.000 to 2.750)	0.042
Ambient particulate matter pollution	1.406 (0.000 to 2.750)	0.030
Bullying victimisation	0.000 (0.000 to 0.000)	0.005
Diet low in calcium	1.442 (0.000 to 2.750)	0.020
Diet low in fibre	1.451 (0.000 to 2.750)	0.020
Diet low in seafood omega-3 fatty acids	1.434 (0.000 to 2.750)	0.022
Diet low in fruits	1.308 (0.000 to 2.500)	0.025
Diet low in whole grains	1.434 (0.000 to 2.750)	0.010
Diet low in legumes	1.462 (0.000 to 2.750)	0.032

Independent driver	Weight distribution summary	Mean RMSE
Diet low in milk	1.459<br>(0.000 to 2.750)	0.011
Diet low in nuts and seeds	1.426<br>(0.000 to 2.750)	0.020
Diet high in processed meat	1.407<br>(0.000 to 2.750)	0.010
Diet low in polyunsaturated fatty acids	1.448<br>(0.000 to 2.750)	0.034
Diet high in red meat	1.516<br>(0.000 to 2.750)	0.022
Diet high in sodium	1.437<br>(0.000 to 2.750)	0.023
Diet high in sugar-sweetened beverages	1.442<br>(0.000 to 2.750)	0.013
Diet high in trans fatty acids	1.410<br>(0.000 to 2.750)	0.018
Diet low in vegetables	1.444<br>(0.000 to 2.750)	0.033
Alcohol use	1.417<br>(0.000 to 2.750)	0.017
Drug use	1.411<br>(0.000 to 2.750)	0.001
Suicide due to drug use disorders	1.411<br>(0.000 to 2.750)	0.001
Lead exposure	0.000<br>(0.000 to 0.000)	0.019
Lead exposure in bone	0.000<br>(0.000 to 0.000)	0.019
Residential radon	0.000<br>(0.000 to 0.000)	0.000
Low bone mineral density	0.000<br>(0.000 to 0.000)	0.004
High body-mass index	1.431<br>(0.000 to 2.750)	0.013
High body-mass index in adults	1.429<br>(0.000 to 2.750)	0.010
High body-mass index in children	1.445<br>(0.000 to 2.750)	0.021
High fasting plasma glucose	0.510<br>(0.000 to 1.000)	0.015
High fasting plasma glucose (categorical)	1.417<br>(0.000 to 2.750)	0.012
High fasting plasma glucose (continuous)	0.510<br>(0.000 to 1.000)	0.015
Impaired kidney function	0.000<br>(0.000 to 0.000)	0.002
High LDL cholesterol	1.419<br>(0.000 to 2.750)	0.005
High systolic blood pressure	0.385<br>(0.000 to 0.750)	0.011

Independent driver	Weight distribution summary	Mean RMSE
Discontinued breastfeeding	0.000<br>(0.000 to 0.000)	0.011
Non-exclusive breastfeeding	1.417<br>(0.000 to 2.750)	0.018
Iron deficiency	1.039<br>(0.000 to 2.000)	0.010
Low birth weight for gestation	1.435<br>(0.000 to 2.750)	0.001
Short gestation for birth weight	1.427<br>(0.000 to 2.750)	0.001
Child stunting	1.423<br>(0.000 to 2.750)	0.020
Child underweight	1.42100<br>(0.000 to 2.750)	0.011
Vitamin A deficiency	1.426<br>(0.000 to 2.750)	0.022
Child wasting	1.414<br>(0.000 to 2.750)	0.007
Zinc deficiency	1.419<br>(0.000 to 2.750)	0.012
Occupational asthmagens	1.411<br>(0.000 to 2.750)	0.013
Occupational ergonomic factors	1.423<br>(0.000 to 2.750)	0.016
Occupational exposure to sulfuric acid	1.406<br>(0.000 to 2.750)	0.001
Occupational exposure to arsenic	1.411<br>(0.000 to 2.750)	0.000
Occupational exposure to asbestos	0.130<br>(0.000 to 0.250)	0.020
Occupational exposure to benzene	1.411<br>(0.000 to 2.750)	0.001
Occupational exposure to beryllium	1.407<br>(0.000 to 2.750)	0.000
Occupational exposure to cadmium	1.411<br>(0.000 to 2.750)	0.000
Occupational exposure to chromium	1.411<br>(0.000 to 2.750)	0.000
Occupational exposure to diesel engine exhaust	1.411<br>(0.000 to 2.750)	0.002
Occupational exposure to formaldehyde	1.416<br>(0.000 to 2.750)	0.001
Occupational exposure to nickel	1.411<br>(0.000 to 2.750)	0.000
Occupational exposure to polycyclic aromatic hydrocarbons	1.411<br>(0.000 to 2.750)	0.001
Occupational exposure to silica	1.411<br>(0.000 to 2.750)	0.004
Occupational exposure to trichloroethylene	1.411<br>(0.000 to 2.750)	0.000

Independent driver	Weight distribution summary	Mean RMSE
Occupational noise	0.000<br>(0.000 to 0.000)	0.003
Occupational particulate matter, gases, and fumes	0.000<br>(0.000 to 0.000)	0.003
Smoking	1.420<br>(0.000 to 2.750)	0.009
Secondhand smoke	1.418<br>(0.000 to 2.750)	0.024
Chewing tobacco	0.000<br>(0.000 to 0.000)	0.004
No access to handwashing facility	1.440<br>(0.000 to 2.750)	0.018
Unsafe sanitation	1.436<br>(0.000 to 2.750)	0.030
Unsafe water source	1.441<br>(0.000 to 2.750)	0.022
Met need for modern contraception	0.000	0.046
Educational attainment (years)	5.500	0.278
Vehicles per capita	0.000	0.081

Section 8 UNPD below replacement fertility forecast convergence

To characterise the level of fertility that countries converge to after dropping below replacement fertility (TFR < 2.1) in UNPD forecasts, we computed the mean TFR in 2015-2020 and mean forecasted TFR in 2095-2100 among locations categorised, as of 2015-2020, to be in fertility transition Phase III by UNPD as well as locations with below replacement fertility (table 4a). UNPD estimates their model for Phase III fertility using only locations that meet Phase III criteria in the past data, defined as having TFR < 2.1 and a fertility upturn in two consecutive 5-year periods.[3] For UNPD 2017 and 2019, this corresponds to 36 and 40 countries, respectively. As this omits many low fertility countries in model estimation, we re-estimated the models of UNPD 2019 using bayesTFR[2] with UNPD 2019 past data under Phase III entrance criteria modified to only TFR < 2.0, thus removing the fertility upturn requirement. This model re-estimation results in substantially lower mean TFR forecasts in 2095-2100 (table 5b).

Table 6a. UNPD mean TFR levels in 2015-2020 and 2095-2100 in UNPD low fertility countries (Phase III) and countries that were below replacement in 2015-2020. Means are not weighted by population size**.**

	UNPD 2017			UNPD 2019		
	**n**	**2015–2020**	**2095–2100**	**n**	**2015–2020**	**2095–2100**
**UNPD phase III 2015-2020**	36	1.64	1.82	40	1.58	1.75
**Below replacement 2015-2020**	86	1.70	1.80	84	1.67	1.73

Table 6b. UNPD 2019 re-estimation with the modified Phase III entrance criteria: UNPD mean TFR levels in 2095-2100. Means are not weighted by population size**.**

**Total fertility rate**	**UNPD 2019**	**UNPD 2019 modified Phase III**
**Location average in 2095-2100**	1.84	1.78
**Below replacement in 2015-2020: location-average in 2095-2100**	1.73	1.48

Section 9 Comparison of population, fertility, and life expectancy forecasts

Figure 8. Forecasts of total fertility, global and by country and territory, in the UNPD medium variant scenario, Wittgenstein SSP2 scenario, and IHME reference scenario, through 2100. The grey lines show the TFR trajectory for each country for UNPD, Wittgenstein, and IHME, respectively. Global TFRs are shown in red. UNPD=United Nations Population Division. TFR=total fertility rate. SSP2=Shared Socioeconomic Pathway 2.

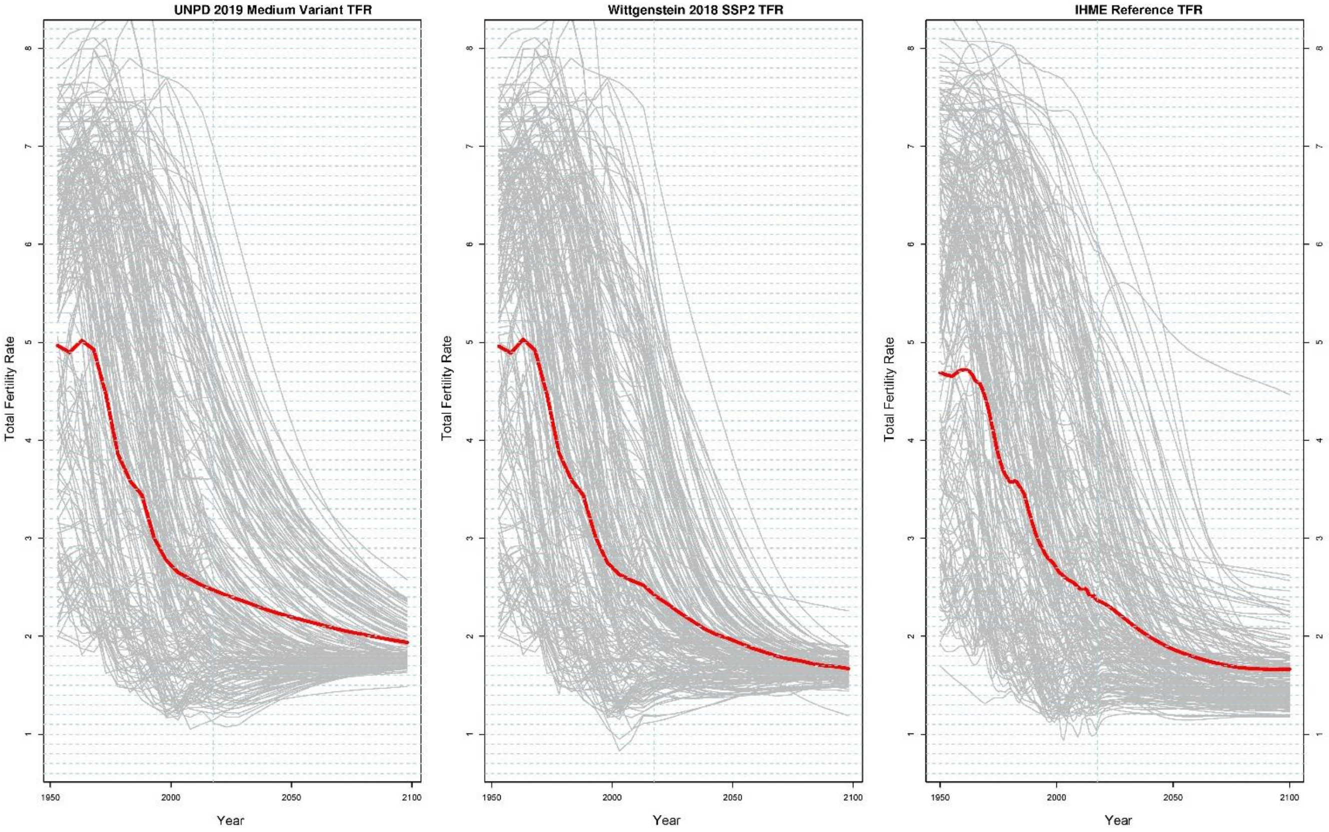

Figure 9. Comparison of the total fertility rate (TFR) in 2100 from the IHME reference forecast, and the United Nations Population Division (UNPD) medium variant scenario, by location.

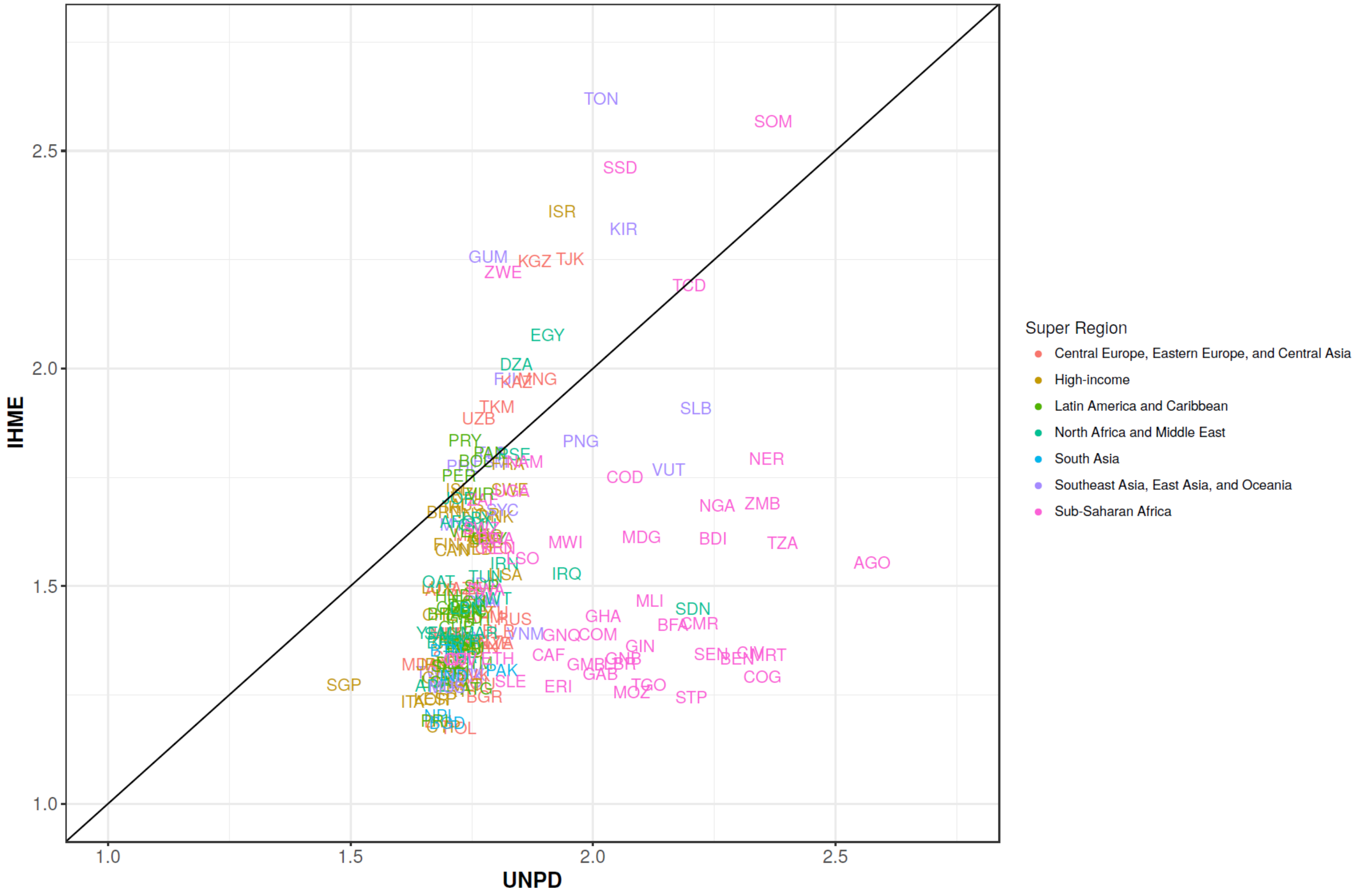

Figure 10. Population, total fertility rate, and life expectancy through 2100 in the IHME reference scenario, UNPD medium variant scenario, and Wittgenstein SSP2 scenario. Estimates through 2017 in the IHME reference scenario are from the Global Burden of Disease 2017 study. Estimates from UNPD are from the World Population Prospects 2019 report, and estimates from the Wittgenstein SSP2 scenario are from Lutz et al, 2018. UNPD=United Nations Population Division. SSP2=Shared Socioeconomic Pathway 2.

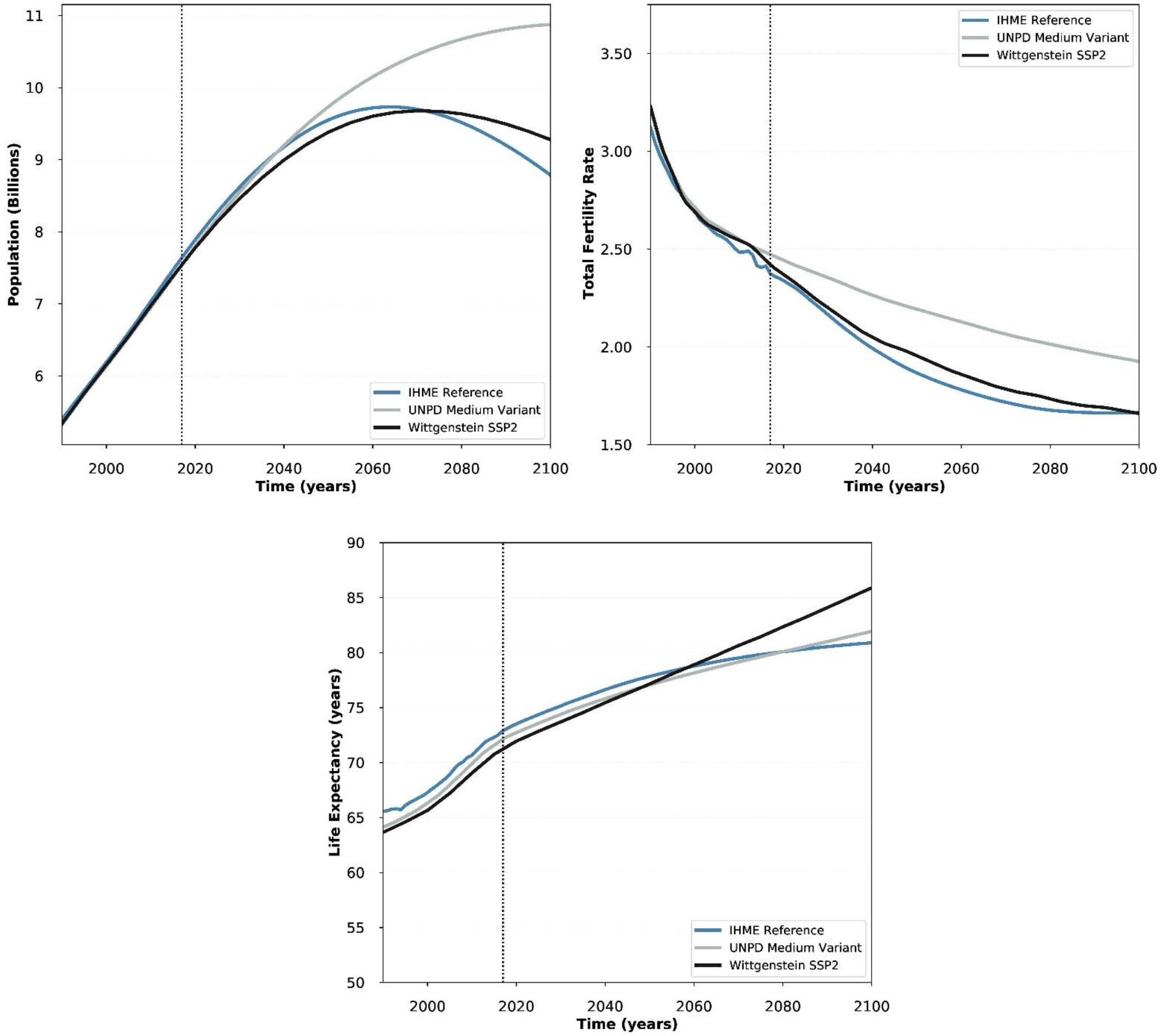

Table 7. Population and total fertility rate (TFR) in 2100 in the IHME reference scenario, UNPD medium variant scenario, and Wittgenstein SSP2 scenario. Results for super-regions were computed and mapped to the GBD super-region hierarchy for comparison. IHME=Institute for Health Metrics and Evaluation. UNPD=United Nations Population Division. SSP2=Shared Socioeconomic Pathways 2.

Location	Population (millions)			Total fertility rates		
	IHME reference 2100	UNPD medium variant 2100	Wittgenstein SSP2 2100	IHME reference 2100	UNPD medium variant 2100	Wittgenstein SSP2 2100
Global	8786	10875	9278	1·66	1·94	1·67
Central Europe, eastern Europe, and central Asia	323	370	345	1·80	1·81	1·66
High-income	957	1141	1291	1·59	1·77	1·77
Latin America and Caribbean	575	601	579	1·58	1·72	1·59
North Africa and Middle East	978	1067	920	1·78	1·88	1·66
South Asia	1442	2026	2011	1·33	1·73	1·60
Southeast Asia, east Asia, and Oceania	1437	1884	1533	1·61	1·77	1·54
Sub-Saharan Africa	3071	3773	2583	1·73	2·16	1·73

Section 10 Evaluation of long range UN population projections: World Population Prospects 1982 projections for 2015 comparison with World Population Prospects 2017 estimates

Section 10.1 Background

The United Nations Population Division (UNPD) has been the most consistent producer of global population projections for the longest time. Publishing their first projections in 1951,[4] they currently produce the World Population Prospects report every two years, which forecasts demographic indicators for all countries out to the year 2100.[5]

Retrospective assessment of the long-range accuracy of UN population projections has been conducted in only a few instances during the last two decades.[6,7] The most recent studies, by Keilman 2001[6] and the US National Research Council, 2001,[7] use estimates from the 1998 revision of the UNPD as a benchmark against which to assess the accuracy of past UN projections. Because the 1998 revision contains demographic estimates up to the year 1995, both studies are limited in their ability to assess the accuracy of projections extending beyond this year.

We sought to update information on the long-range accuracy of UN projections of population and TFR by evaluating historical demographic forecasts against the UN's most current estimates. Specifically, we evaluated the accuracy of population projections for 198 countries and TFR projections for 149 countries, comparing projections from the 1982 revision[8] with estimates from the 2017 revision.[9] UNPD 1982 makes projections beginning from base year 1980; UNPD 2017 reports estimates up to the year 2015. Thus, we were able to assess the 35-year projection accuracy by evaluating forecasts made from base year 1980 to target year 2015. Assessments of accuracy were made predominately for 35-year country-specific projections, with brief consideration of global and regional projection accuracy of varying projection length.

Section 10.2 Data

The data for this analysis were extracted from two versions of the UNPD World Population Prospects report. Forecasted country-, region-, and global-level population size and TFR were extracted from the *World Population Prospects as assessed in 1982*[8] and compared with estimates as published in the *World Population Prospects: 2017 revision.*[9] Forecasted values from UNPD 1982 are the medium variant projections of total population and TFR. Estimated values as reported by UNPD 2017 are treated as the true value of total population and TFR for all years prior to 2015.

World Populations Prospects 2017 was selected as the reference dataset because it was the most recently available revision of UNPD forecasts at time of analysis. Because we were particularly interested in long-range forecasts, we sought to identify the earliest revision with country-level projections out to at least the year 2015. The 1980 revision met these criteria but, due to poor print quality, which hampered data digitisation, World Population Prospects 1982 was used instead.

Data from UNPD 1982 were not available in soft format. As such, tables 2 (pp. 78–85) and 12 (pp. 150–157) from the report were scanned and digitised using PDF2XL software (Cogniview, LLC).

Because country boundaries have changed over time, the units of projection in UNPD 1982 do not completely align with those reported in UNPD 2017. Countries that have broken apart since 1982 have been maintained as a single entity for the purpose of comparison because UNPD 1982 projections (made prior to dissolution) did not distinguish between subunits. These countries are detailed in table 6.

Table 8. Countries having broken apart since 1982, for which adjustment was made in analysis

1982	2017
Czechoslovakia	Czech Republic and Slovakia
Sudan	Sudan and South Sudan
USSR	Estonia, Latvia, Lithuania, Kazakhstan, Kyrgyzstan, Tajikistan, Turkmenistan, Uzbekistan, Belarus, Moldova, Ukraine, Russia, Armenia, Azerbaijan, and Georgia
Yugoslavia	Serbia, Croatia, Bosnia & Herzegovina, Macedonia, Slovenia, and Montenegro

UNPD 2017 population estimates for these countries were aggregated to reflect 1982 country borders by taking the sum of the populations of the current-day countries that now comprise the dissolved states. TFR estimates were aggregated using a weighted mean based on the number of women of childbearing age (ages 15-49) per country per year.

Countries that have arisen from the unification of two formerly separate states were handled in a similar fashion (table 7). UNPD 1982 population projections were aggregated to reflect 2017 borders by taking the sum of the populations of the former countries that now

constitute a single entity. However, adjustment was not made for TFR because of the absence of age-sex-specific population estimates for countries that no longer exist in present day. As such, no aggregation could be performed for TFR.

Table 9. Countries having been formed since 1982 for which adjustments were made in analysis

1982	2017
Germany	East Germany and West Germany
Yemen	Yemen and Democratic Yemen

Additionally, we acknowledge the following changes in name that occurred between the publication of UNPD 1982 and UNPD 2017:

- Burma (1982) to Myanmar (2017)
- Cape Verde (1982) to Cabo Verde (2017)
- East Timor (1982) to Timor-Leste (2017)
- Democratic Kampuchea (1982) to Cambodia (2017)
- Zaire (1982) to Democratic Republic of the Congo (2017)

UNPD 1982 reported population projections for 206 countries. Of these, 194 also had population estimates reported in UNPD 2017. After adjustment for changing country boundaries, as detailed above, a total of 198 country pairs were included in analysis of the accuracy of population projections.

UNPD 1982 reported TFR projections for 152 unique countries (those with population greater than 225 000 in 1980).
Of these, a total of 146 countries also had TFR estimates reported in UNPD 2017.[10] After adjustment for changing country boundaries, a total of 149 country pairs were included in analysis of the accuracy of TFR projections.

Section 10.3 Methods

To quantify the accuracy of UNPD forecasts, we compare projections of population and TFR as forecast in UNPD 1982 with estimates of population and TFR as assessed in UNPD 2017. Specifically, we report two measures of error:

1. Percentage error (PE):

$$pe_{it} = \frac{\hat{Y}_{it} - Y_{it}}{Y_{it}}$$

2. Absolute percentage error (APE):

$$ape_{it} = |pe_{it}|$$

where $\hat{Y}_{it}$ is the projected value for country i in target year t as forecast in 1982 and Y_{it} is the actual value as estimated in 2017. Both measures can be averaged across target years, across locations, or both to produce mean percentage error (MPE) or mean absolute percentage error (MPE).

To assess the total correlation of country-level forecasts with observed values, we report on three measures of correlation:

1. Pearson's correlation coefficient:

$$\rho_p = \frac{cov(\hat{Y}, Y)}{\sigma_{\hat{Y}}\ \sigma_Y}$$

where $\sigma_{\hat{Y}}$ is the standard deviation of the projected values and σ_Y is the standard deviation of the estimated values.

2. Spearman correlation coefficient:

$$\rho_s = \frac{cov(rg_{\hat{Y}}, rg_Y)}{\sigma_{rg_{\hat{Y}}}\ \sigma_{rg_Y}}$$

where $rg_{\hat{Y}}$ and rg_Y are the rank variables for the projected and estimated values, respectively, and $\sigma_{rg_{\hat{Y}}}$ and σ_{rg_Y} are the corresponding standard deviations.

3. Concordance (Lin's) correlation coefficient[11]

$$\rho_c = \frac{2\sigma_{\hat{Y}\,Y}}{\sigma_{\hat{Y}}^2 + \sigma_Y^2 + (\mu_{\hat{Y}} - \mu_Y)^2}$$

where $\mu_{\hat{Y}}$ and μ_Y are the means of the projected and estimated values, respectively, and $\sigma_{\hat{Y}}^2$ and σ_Y^2 are the corresponding variances.

Section 10.4 Results

Section 10.4.1 Population

Section 10.4.1.1 Global

Global population projections were found to be highly accurate, even for long-range projections, though the APE did increase modestly with increasing projection length (table 8b). The five-year projection of global population made in 1982 was off by just 0.4%, the 20-year by 1.7%, and the 35-year by 3.5%. This finding confirms that long-range forecasts are more difficult to project accurately, given the longer period during which unanticipated developments can emerge to alter the expected course of fertility, mortality, and migration.

Table 10a. Mean percentage error (MPE) of UNPD 1982 population forecast compared to UNPD 2017 population estimates by projection length

		**Projection length**							
	n	**0**	**5**	**10**	**15**	**20**	**25**	**30**	**35**
Global	1	-0.002	-0.004	-0.007	0.003	0.017	0.028	0.033	0.035
Region average	6	-0.058	-0.058	-0.060	-0.057	-0.051	-0.048	-0.052	-0.057
Country average	193	0.014	0.013	0.016	0.029	0.049	0.058	0.063	0.069
		1980	*1985*	*1990*	*1995*	*2000*	*2005*	*2010*	*2015*

Table 10b. Mean absolute percentage error (MAPE) of UNPD 1982 population forecast compared to UNPD 2017 population estimates by projection length

		**Projection length**							
	n	**0**	**5**	**10**	**15**	**20**	**25**	**30**	**35**
Global	1	0.002	0.004	0.007	0.003	0.017	0.028	0.033	0.035
Region average	6	0.059	0.061	0.068	0.075	0.083	0.092	0.105	0.112
Country average	193	0.050	0.057	0.080	0.108	0.141	0.171	0.199	0.220
		1980	*1985*	*1990*	*1995*	*2000*	*2005*	*2010*	*2015*

The base year 1980 data used to construct UNPD 1982 projections slightly underestimated the 1980 world population (as estimated in UNPD 2017[9]). As a result, short-term projections of global population to the years 1985 and 1990 were also slightly underestimated. Thereafter, population was overestimated at each subsequent interval, due in part to increasingly large overestimates of TFR.

Section 10.4.1.2 Region

Region-level population projections were substantially less accurate than global projections. The average region was off by 6.1% for five-year projection, increasing steadily with projection length to a mean APE of 11.2% for 35-year projection (table 8b).

Section 10.4.1.3 Country and territory

With regard to the mean APE, country-level projections performed worse still than regions, supporting findings put forth by others[6,7,12] that projections are less accurate for smaller areas (table 8b). 39% of 35-year country-level population projections reported in UNPD 1982 were off by 20% or more in either direction when evaluated against UNPD 2017 (table 6). Over-estimation was more common than under-estimation, with 55% of countries exhibiting a projected 2015 population that was larger than reported in UNPD 2017.

However, with regard to the *correlation* between projected and estimated population values, countries perform exceedingly well. The Pearson, Spearman, and Lin's concordance correlation coefficients calculated for 35-year projections all surpass 0.99, indicating substantial agreement between projected and observed country-specific populations for 2015 (table 9). This agreement can be clearly observed in figure 8.

Table 11. Correlation between forecasted and estimated country-level populations in the year 2015 (UNPD 1982 vs. UNPD 2017)

Correlation	Value
Pearson	0.998
Spearman	0.995
Concordance	0.998

1. Forecasted data are as published in UNPD 1982 (base year 1980)
2. Estimated data are as published in UNPD 2017 (base year 2015)

Figure 11. Accuracy of country-specific population forecasts for year 2015 as projected by UNPD 1982

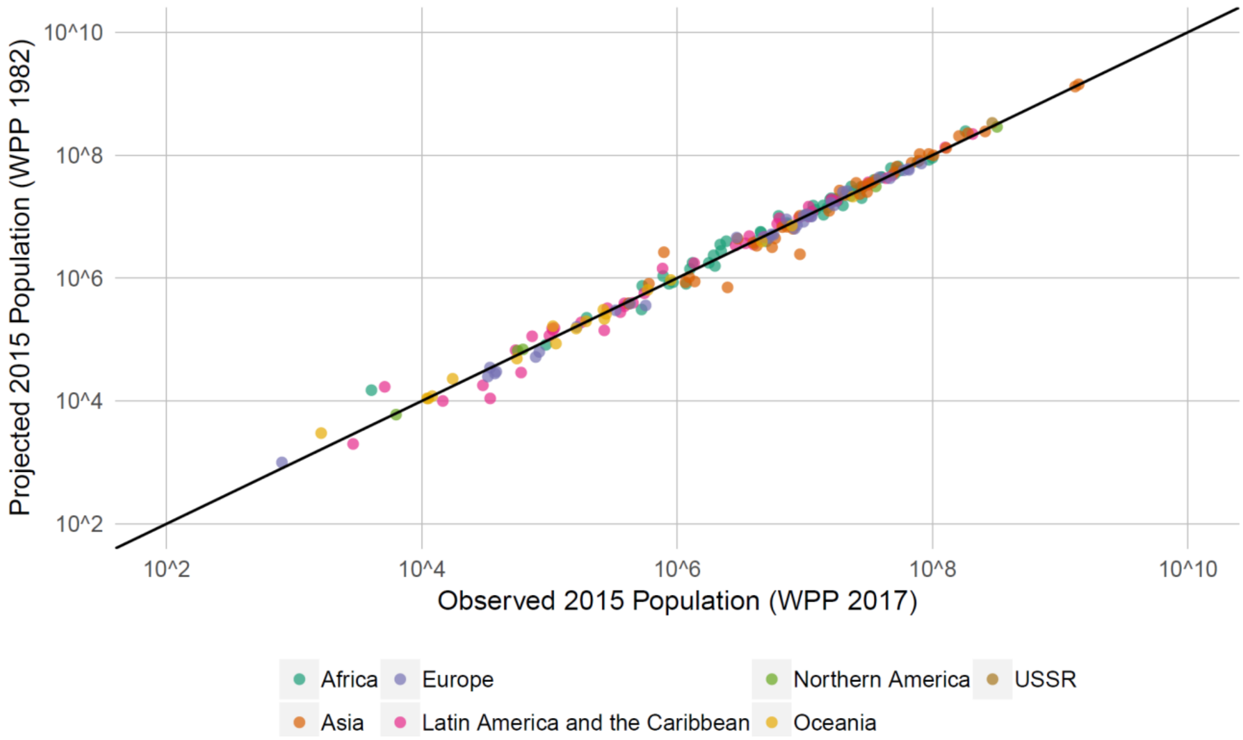

Figure 12. Accuracy of country-specific population forecasts for year 2015 as projected in 1982, assessed against UNPD 2017 by difference and ratio

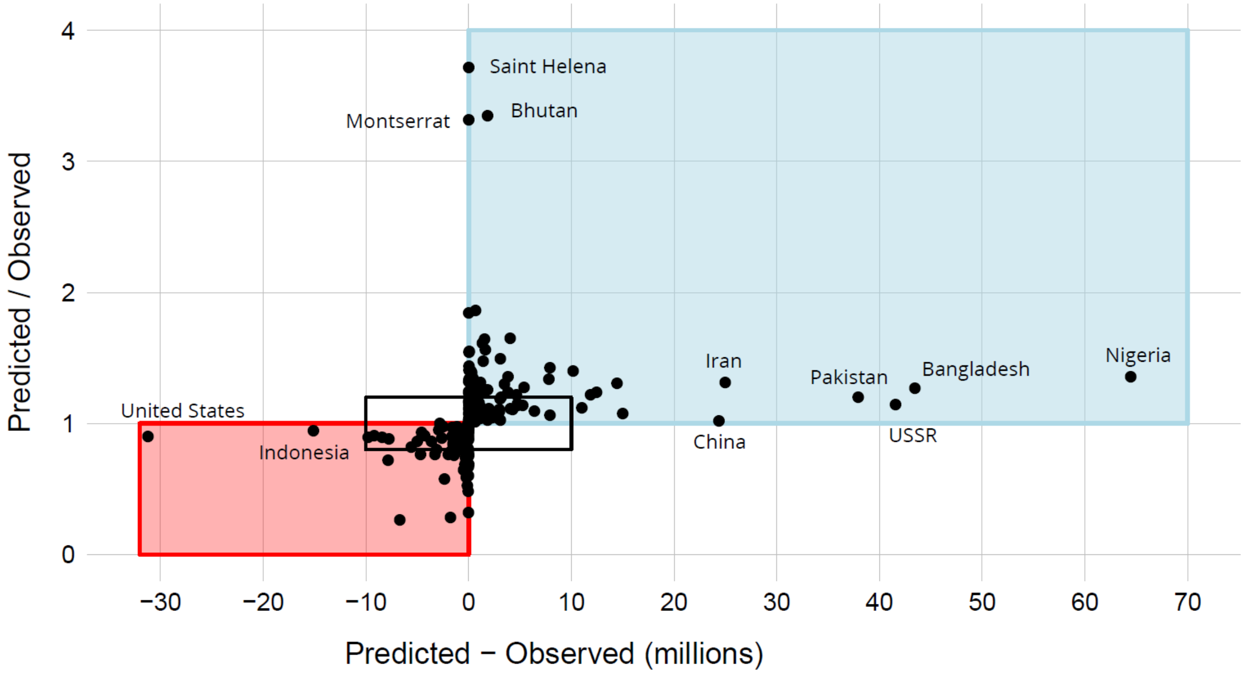

Section 10.4.2 Fertility

Section 10.4.2.1 Global

Global projections of TFR were, on the whole, less accurate than population projections. UNPD 1982 correctly predicted that global TFR would decline, but assumed this decline would occur more rapidly than it did. As such, the 15- and 20-year projections were both off by more than 12%, and the size of the errors declined thereafter (table 11). Global TFR was consistently over-estimated.

Table 12a. Mean percentage error (MPE) of UNPD 1982 TFR forecast compared to UNPD 2017 TFR estimates by projection length

		**Projection length**						
	n	**0**	**5**	**10**	**15**	**20**	**25**	**30**
Global	1	0.003	0.000	0.089	0.138	0.125	0.078	0.024
Region average	6	-0.004	-0.002	0.039	0.082	0.070	0.051	0.047
Country average	149	-0.009	0.014	0.062	0.125	0.160	0.139	0.112
		1980-85	*1985-90*	*1990-95*	*1995-00*	*2000-05*	*2005-10*	*2010-15*

Table 12b. Mean absolute percentage error (MAPE) of UNPD 1982 TFR forecast as compared with UNPD 2017 TFR estimates by projection length

		**Projection length**						
	n	**0**	**5**	**10**	**15**	**20**	**25**	**30**
Global	1	0.003	0.000	0.089	0.138	0.125	0.078	0.024
Region average	6	0.014	0.028	0.070	0.102	0.093	0.097	0.101
Country average	149	0.053	0.085	0.128	0.186	0.225	0.219	0.208
		1980-85	*1985-90*	*1990-95*	*1995-00*	*2000-05*	*2005-10*	*2010-15*

Section 10.4.2.2 Regional

Region-level TFR projection errors steadily increased with increasing projection length until 15-years, at which point, projections were consistently off by about 10%. With the exception of the five-year projection, the average region's TFR was over-estimated (table 10).

Section 10.4.2.3 Country or territory

With regard to the mean APE, country-level TFR projections performed worse than either regional or global forecasts (table 10b). 40% of 35-year country-level projections reported in UNPD 1982 were off by 20% or more in either direction when evaluated against UNPD 2017 (table 11). Over-estimation was more common than under-estimation, with 64% of countries exhibiting a projected 2015 TFR that was larger than reported in UNPD 2017.

Table 13. Number of countries with absolute percentage error greater than 20% for projection of 2015 population or TFR (UNPD 1982 vs UNPD 2017)

	Population	TFR
# countries off by >20%	78 (39%)	59 (40%)

1. Forecasted data are as published in UNPD 1982
2. Estimated data are as published in UNPD 2017

The correlation between projected and estimated TFR was not as robust as had been observed with population forecasts (table 12). Agreement, as measured by Lin's correlation coefficient, was moderate for TFR ($\rho_c = 0.832$) and particularly weak for countries with below-replacement fertility in 2015 ($\rho_c = 0.235$), though countries with above-replacement fertility performed better ($\rho_c = 0.718$).

Table 14. Correlation between forecasted and estimated country-level TFR in the year 2015 (UNPD 1982 vs UNPD 2017)

Correlation	Below-replacement fertility[1]	Above-replacement fertility[2]	All
Pearson	0.408	0.740	0.853
Spearman	0.472	0.726	0.870
Concordance	0.235	0.718	0.832

1. 57 countries included in analysis had TFR < 2.1 in 2015 as reported in UNPD 2017
2. 92 countries included in analysis had TFR ≥ 2.1 in 2015 as reported in UNPD 2017
3. Forecasted data are as published in UNPD 1982 (base year 1980)
4. Estimated data are as published in UNPD 2017 (base year 2015)

The TFRs of countries with below-replacement fertility in 2015 (as reported by UNPD 2017[9]) were almost exclusively over-estimated by UNPD 1982 (figure 9). In general, countries with 2015 TFR below 3.0 tended to be over-estimated by UNPD 1982 while countries with 2015 TFR above 5.0 tended to be underestimated. Countries such as Oman and Saudi Arabia were substantially over-estimated, whereas Nigeria and Timor-Leste were seriously underestimated. The interpretation of the 1982 projections need to account for data availability and quality at the time the projections were made.

Figure 13. Accuracy of country-specific TFR forecasts for year 2015 as projected by UNPD 1982

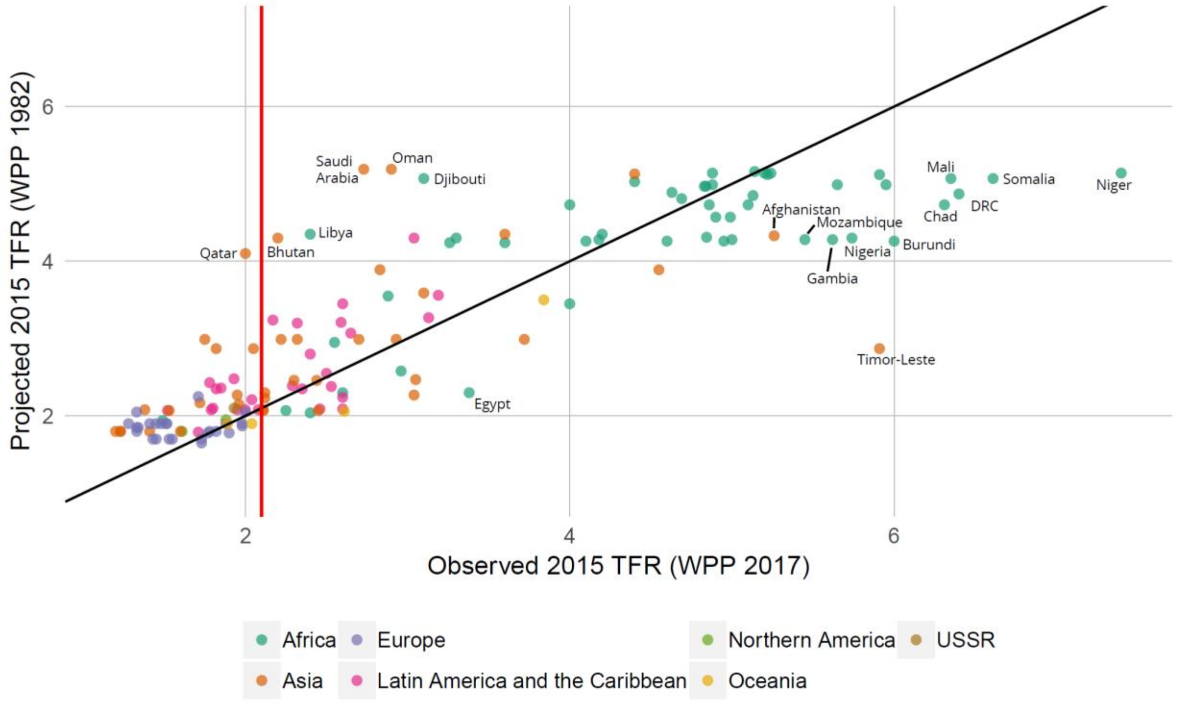

Figure 14. Accuracy of country-specific TFR forecasts for year 2015 as projected in 1982, assessed against UNPD 2017 by difference and ratio

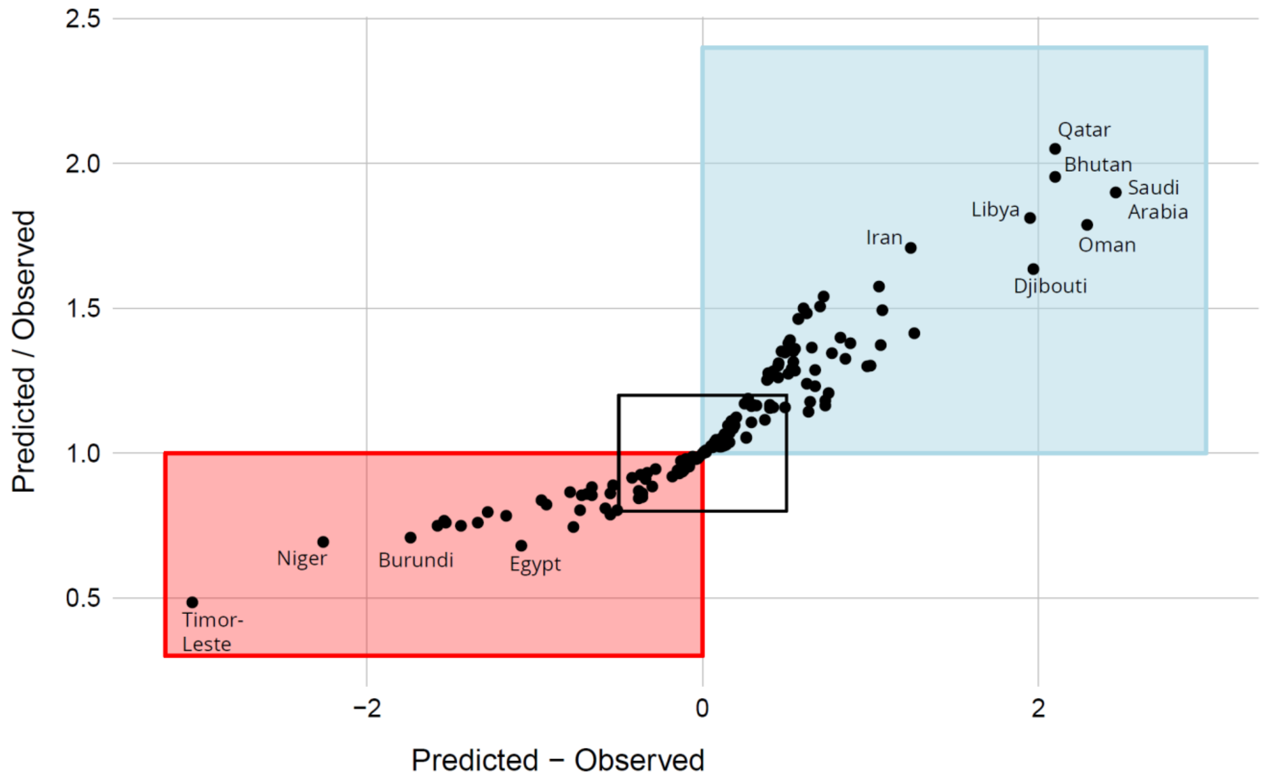

Section 10.5 Conclusion

In general, UN population projections are highly accurate, while projections of TFR are less so. Projection accuracy tends to decrease with increasing projection length and decreasing size of the area being estimated. Small populations and low TFR are especially hard to forecast accurately. Further investigation into the accuracy of forecasted mortality and migration would help to understand why population projections are mostly accurate, despite TFR being projected incorrectly.

Section 11 Detailed results files in GHDx

Results files in csv-format will be available for life expectancy, ASFR, TFR, and migration for all locations and location aggregates and scenarios from

Section 12 References

1 Stevens GA, Alkema L, Black RE, *et al.* Guidelines for Accurate and Transparent Health Estimates Reporting: the GATHER statement. *The Lancet* 2016; **388**: e19–23.

2 Ševčíková H, Alkema L, Raftery A. bayesTFR: An R package for probabilistic projections of the total fertility rate. *J Stat Softw* 2011; **43**: 1–29.

3 United Nations, Department of Economic and Social Affairs, Population Division. World Population Prospects 2019: summary of methodological updates introduced in the 2019 revision. New York: United Nations, 2019.

4 Population bulletin of the United Nations. 1951. http://digitallibrary.un.org/record/93987 (accessed Aug 22, 2019).

5 United Nations, Department of Economic and Social Affairs, Population Division. World Population Prospects: The 2017 revision, key findings and advance tables. New York: United Nations, 2017.

6 Keilman N. Data quality and accuracy of United Nations population projections, 1950-95. *Popul Stud* 2001; **55**: 149–64.

7 National Research Council. Beyond six billion: forecasting the world's population. Washington DC: National Academy Press, 2000.

8 United Nations, Department of International Economic and Social Affairs, Population Division. World population prospects: 1982 assessment. *Popul Newsl* 1985; **36**: 4–14.

9 United Nations, Department of Economic and Social Affairs, Population Division. World Population Prospects: The 2017 revision, volume I: comprehensive tables. New York: United Nations, 2017.

10 United Nations, Department of Economic and Social Affairs, Population Division. World Population Prospects 2017: data booklet. New York: United Nations, 2017.

11 Lin L. A concordance correlation coefficient to evaluate reproducibility. *Biometrics* 1989; **45**: 255–68.

12 Bos E, Vu MT, Massiah E, Bulatao RA. World population projections 1994-95 edition: estimates and projections with related demographic statistics. Baltimore: The World Bank; The Johns Hopkins University Press, 1994.

Section 13 Location-year life tables

Table 15: Global 2017 life table, by age and sex. mx=mortality rate, ax=mean person-years lived in an age interval among those who die in that age interval, lx=number of persons left alive at age x, nLx=person-years lived between age x and x+n, ex=life expectancy at age x.

Age Group	Male					Female				
	mx	ax	lx	nLx	ex	mx	ax	lx	nLx	ex
Early Neonatal	0.768501054	0.009565487	100000	0.019037447	70.44499015	0.6093526	0.009570365	100000	0.019066458	75.485232
Late Neonatal	0.065918637	0.02874894	98536.97474	0.056585137	71.47158823	0.060910979	0.028750321	98838.18304	0.056766279	76.35324276
Post Neonatal	0.01283591	0.460731996	98163.97579	0.900986481	71.68551806	0.012637835	0.460746067	98492.41586	0.904083521	76.56365228
1 to 4	0.002598592	1.996535217	97007.48801	3.860202902	71.61134514	0.002571498	1.996571342	97349.85756	3.874036236	76.53355488
5 to 9	0.000646945	2.498652199	96004.39597	4.792464528	68.33871593	0.000597971	2.498754228	96353.66307	4.810488281	73.30419424
10 to 14	0.000554151	2.624655447	95694.35231	4.778427794	63.55203507	0.00044705	2.53843724	96066.01153	4.798020687	68.51620848
15 to 19	0.000984071	2.691400742	95429.55624	4.760662447	58.72109492	0.000683918	2.627816995	95851.51682	4.784813136	63.6638515
20 to 24	0.001469362	2.597834767	94961.07418	4.731353703	53.99750997	0.000871329	2.580340925	95524.27579	4.766165248	58.87294261
25 to 29	0.001693523	2.579487058	94265.86857	4.69405164	49.37657632	0.001027487	2.581165451	95108.98729	4.743659868	54.11873833
30 to 34	0.002147528	2.598948683	93470.92205	4.649571459	44.77456987	0.001283074	2.60103056	94621.58423	4.716561343	49.38420928
35 to 39	0.002763172	2.605378028	92472.41665	4.593228688	40.22997264	0.001667659	2.614675474	94016.41753	4.682195538	44.68534138
40 to 44	0.003628329	2.630998991	91203.234	4.521298993	35.75355043	0.002231827	2.617839208	93235.59111	4.637125984	40.03766978
45 to 49	0.00520046	2.649076549	89562.7663	4.424050717	31.36022903	0.00298413	2.66504806	92200.66979	4.578134074	35.45768982
50 to 54	0.007668841	2.651306017	87262.07089	4.285907285	27.11719759	0.004713437	2.646600065	90834.50205	4.491898482	30.95089069
55 to 59	0.011465092	2.651901785	83975.30521	4.088693834	23.07477061	0.006536851	2.661085705	88717.28699	4.36906556	26.62635621
60 to 64	0.017618046	2.631740809	79287.62631	3.805598255	19.2822086	0.010318305	2.664671075	85861.31642	4.192052434	22.42349495
65 to 69	0.026141411	2.602155784	72582.99856	3.415085921	15.82022799	0.015967796	2.656702454	81535.87314	3.929754764	18.47167973
70 to 74	0.038018665	2.583361104	63655.65563	2.914970082	12.6739774	0.025485453	2.635893034	75261.00915	3.549213241	14.79023518
75 to 79	0.058030441	2.559406377	52573.63626	2.302582163	9.800990166	0.040295072	2.623605501	66215.86674	3.02147376	11.45052155
80 to 84	0.093830573	2.492450837	39212.15668	1.587185426	7.26859009	0.07057078	2.561300733	54041.15763	2.305323875	8.439110502
85 to 89	0.156165149	2.362447622	24320.21433	0.86127518	5.193260033	0.126705013	2.452552356	37772.90296	1.427805919	5.970647949
90 to 94	0.251238251	2.144116088	10870.73368	0.316474015	3.695786058	0.213739348	2.2436061	19682.62574	0.619291201	4.204247808
95 plus	0.342345815	2.921029873	2919.993555	0.085296053	2.921029873	0.309583053	3.230164329	6446.428938	0.208236113	3.230164329

Table 15: Global 2100 life table, by age and sex. mx=mortality rate, ax=mean person-years lived in an age interval among those who die in that age interval, lx=number of persons left alive at age x, nLx=person-years lived between age x and x+n, ex=life expectancy at age x.

Age Group	Male					Female				
	mx	ax	lx	nLx	ex	mx	ax	lx	nLx	ex
Early Neonatal	0.163255896	0.009584037	100000	0.019148093	79.01767089	0.136372822	0.009584861	100000	0.019153026	82.87315711
Late Neonatal	0.013049669	0.028763524	99687.42431	0.057332884	79.24617244	0.014451283	0.028763137	99738.8179	0.057360129	83.07092355
Post Neonatal	0.002198422	0.461487663	99612.61074	0.918778229	79.24811009	0.002209418	0.461486882	99655.92943	0.919173109	83.08244429
1 to 4	0.000427885	1.999429487	99410.63701	3.97302484	78.48486247	0.000414955	1.999446727	99452.85647	3.974814854	82.32782028
5 to 9	0.000160179	2.499666294	99240.65483	4.960046365	74.61583608	0.000153399	2.499680418	99287.93305	4.962493423	78.46122732
10 to 14	0.000183567	2.82856117	99161.21059	4.956083774	69.67358755	0.000155499	2.638534754	99211.81383	4.958768674	73.51949124
15 to 19	0.000449174	2.760287982	99070.23947	4.948535422	64.73493822	0.000255965	2.64816849	99134.71072	4.953753258	68.57460658
20 to 24	0.000748685	2.622205273	98847.98456	4.93361697	59.87415935	0.000337788	2.632256304	99007.92243	4.946440223	63.65901072
25 to 29	0.000892132	2.58644473	98478.69916	4.913355309	55.08862652	0.000471614	2.628291327	98840.8533	4.936520404	58.76213718
30 to 34	0.001124267	2.580733877	98040.5031	4.888725973	50.3229903	0.000629439	2.606575746	98608.06667	4.922982765	53.89456752
35 to 39	0.00133319	2.596180641	97491.09621	4.858987786	45.59171261	0.000792685	2.62152831	98298.24321	4.905666395	49.0560836
40 to 44	0.001757227	2.647983065	96843.62504	4.82225604	40.8786527	0.001100859	2.665620421	97909.4479	4.882927831	44.24016197
45 to 49	0.002613634	2.687785092	95996.73602	4.771018115	36.21512904	0.001683675	2.703552865	97372.04818	4.849871556	39.46901826
50 to 54	0.004195186	2.698634774	94750.69841	4.692265274	31.65474253	0.002798752	2.666606809	96555.84712	4.796468209	34.77865896
55 to 59	0.006829023	2.674162858	92784.32743	4.566711752	27.26642609	0.00397607	2.665049484	95214.84418	4.716926497	30.22879816
60 to 64	0.010350845	2.650849379	89671.26838	4.377319408	23.11767571	0.006054663	2.659603033	93342.19863	4.602009067	25.7797666
65 to 69	0.015554899	2.634682781	85150.40626	4.106767274	19.20046047	0.009043687	2.676171291	90560.99113	4.434977045	21.48632532
70 to 74	0.022991857	2.628434866	78784.12716	3.736245865	15.53626421	0.014605901	2.6912129	86562.27054	4.187354652	17.35199683
75 to 79	0.036093586	2.623738024	70229.37458	3.235617903	12.10618613	0.025186339	2.712897295	80471.22158	3.805356196	13.45785763
80 to 84	0.06070146	2.569851712	58627.10117	2.557706458	8.982702478	0.049786665	2.643095477	70944.1736	3.177508605	9.898440706
85 to 89	0.114640686	2.480702116	43253.46425	1.68272624	6.265344845	0.097616056	2.550474231	55266.89266	2.235342778	6.954659907
90 to 94	0.208761609	2.219833431	24198.04818	0.768825098	4.274264643	0.180259494	2.307561921	33715.50347	1.13986103	4.790613181
95 plus	0.307871987	3.251894353	8320.033244	0.272575819	3.251894353	0.278614046	3.596808332	13423.66222	0.48742739	3.596808332

Table 15: Central Europe, Eastern Europe, and Central Asia 2017 life table, by age and sex. mx=mortality rate, ax=mean person-years lived in an age interval among those who die in that age interval, lx=number of persons left alive at age x, nLx=person-years lived between age x and x+n, ex=life expectancy at age x.

Age Group	Male					Female				
	mx	ax	lx	nLx	ex	mx	ax	lx	nLx	ex
Early Neonatal	0.317367288	0.009579314	100000	0.019119837	68.50618311	0.219181219	0.009582323	100000	0.019137831	77.61488245
Late Neonatal	0.032216457	0.028758236	99393.20151	0.057132165	68.90517354	0.028870977	0.028759159	99580.53543	0.057245354	77.92260005
Post Neonatal	0.004517376	0.461322929	99209.14363	0.914078255	68.97542188	0.003886349	0.461367756	99415.26337	0.916244057	77.9945594
1 to 4	0.00062126	1.999171654	98796.22578	3.946942924	68.33848979	0.000498244	1.999335675	99059.18184	3.958421481	77.34997623
5 to 9	0.000297841	2.499379498	98551.02213	4.923883897	64.50355003	0.000209074	2.49956443	98861.95782	4.940515121	73.50029763
10 to 14	0.000320296	2.822794597	98404.37017	4.916789737	59.59595567	0.000214124	2.665071605	98758.66502	4.935465713	68.57455835
15 to 19	0.000796592	2.794014948	98246.88809	4.903727216	54.68695845	0.000379509	2.657183002	98652.98522	4.928267473	63.645162
20 to 24	0.001453417	2.691570872	97856.26148	4.876452011	49.89410384	0.000501901	2.642727016	98465.953	4.917479683	58.76100475
25 to 29	0.002156634	2.673548829	97147.51057	4.833126257	45.23847063	0.000726356	2.669173007	98219.14474	4.902656931	53.90201845
30 to 34	0.003307473	2.642375311	96105.18345	4.768078823	40.70011105	0.001098115	2.657974596	97863.03777	4.880599897	49.08844356
35 to 39	0.00453443	2.599332125	94528.15745	4.675512016	36.33502607	0.001572708	2.631686924	97327.09256	4.848296377	44.34411627
40 to 44	0.005676887	2.613195194	92408.08587	4.55863679	32.10899688	0.002118814	2.635335456	96564.59889	4.804159779	39.67348142
45 to 49	0.007999824	2.640883269	89820.21195	4.407824379	27.95880977	0.003001154	2.653639608	95546.69004	4.743928855	35.06806276
50 to 54	0.011846506	2.640384177	86294.05507	4.197373717	23.99333999	0.004439581	2.660356207	94122.96966	4.657768241	30.55836258
55 to 59	0.017619963	2.6259317	81321.68336	3.902826878	20.29894134	0.006653128	2.660435456	92055.12684	4.532210934	26.18503065
60 to 64	0.026069284	2.586145358	74445.00362	3.501888505	16.93143218	0.010093444	2.659774672	89039.80888	4.349257679	21.98167857
65 to 69	0.035434524	2.560964533	65315.9743	3.006006514	13.9364265	0.015629107	2.663958093	84649.94654	4.083412542	17.98368343
70 to 74	0.050325913	2.551537326	54664.54698	2.433390865	11.15294714	0.025477403	2.674267975	78268.00775	3.694492062	14.23283507
75 to 79	0.07687808	2.505035261	42418.64621	1.779603181	8.636126451	0.045915826	2.628369165	68855.57657	3.104699501	10.81286066
80 to 84	0.11301397	2.423717777	28737.92214	1.112887905	6.55491326	0.079666424	2.549145896	54600.46001	2.284071526	7.949680552
85 to 89	0.177451177	2.30836863	16161.31478	0.546873393	4.769915295	0.138592618	2.417431301	36404.70716	1.340468023	5.649022387
90 to 94	0.271465466	2.101975037	6457.39983	0.18070915	3.469231574	0.226573308	2.215234029	17827.52015	0.546545026	4.016632871
95 plus	0.358268065	2.791210739	1551.933334	0.043318698	2.791210739	0.321158018	3.113743012	5444.698156	0.16953836	3.113743012

Table 15: Central Europe, Eastern Europe, and Central Asia 2100 life table, by age and sex. mx=mortality rate, ax=mean person-years lived in an age interval among those who die in that age interval, lx=number of persons left alive at age x, nLx=person-years lived between age x and x+n, ex=life expectancy at age x.

Age Group	Male					Female				
	mx	ax	lx	nLx	ex	mx	ax	lx	nLx	ex
Early Neonatal	0.0917	0.00958623	100000	0.019161229	78.61094428	0.062512649	0.009587125	100000	0.019166591	85.03414231
Late Neonatal	0.009606759	0.028764473	99824.30643	0.057417295	78.72997649	0.010873272	0.028764124	99880.19166	0.057447346	85.11684108
Post Neonatal	0.001132894	0.461563357	99769.15276	0.920674764	78.71588205	0.001121675	0.461564154	99817.73277	0.921127833	85.11249286
1 to 4	0.00012782	1.999829573	99664.86873	3.985576026	77.87444318	0.00011039	1.999852813	99714.43117	3.98769698	84.27685443
5 to 9	8.79451E-05	2.499816781	99613.93762	4.979602104	73.91322523	7.71735E-05	2.499839222	99670.4217	4.982559866	80.31317119
10 to 14	0.000131925	2.993678316	99570.15016	4.977173918	68.94457045	0.00011044	2.753928267	99631.97574	4.980356609	75.34315219
15 to 19	0.000386908	2.789816031	99504.49941	4.971001127	63.98800644	0.000205264	2.65842635	99576.98144	4.976457796	70.38317518
20 to 24	0.00069964	2.703970514	99312.23035	4.957660035	59.10486727	0.000267165	2.670569548	99474.84927	4.970649956	65.45250889
25 to 29	0.00108408	2.704544966	98965.77351	4.935994196	54.29858826	0.000425273	2.716731958	99342.08162	4.962281624	60.53606225
30 to 34	0.001744534	2.641518999	98432.09424	4.90144543	49.57282313	0.000706383	2.652622618	99131.14128	4.9483395	55.65841738
35 to 39	0.002220131	2.573480879	97581.24308	4.853087035	44.97497935	0.000930116	2.599270357	98781.86878	4.928115553	50.84460491
40 to 44	0.002569611	2.590426691	96511.67686	4.796171491	40.43829172	0.001172132	2.630434661	98324.08893	4.902607993	46.06741299
45 to 49	0.003401678	2.664353332	95292.585	4.727361549	35.91637662	0.001670596	2.657749721	97750.84347	4.868491256	41.31967769
50 to 54	0.005122127	2.693473545	93709.12801	4.63115793	31.47193912	0.002405973	2.669486608	96940.41571	4.819993334	36.63961676
55 to 59	0.008024412	2.663660069	91374.17695	4.485490487	27.20113815	0.003567349	2.660625902	95786.47737	4.74970966	32.0443564
60 to 64	0.011656578	2.620027355	87832.15568	4.274664286	23.18290171	0.005093047	2.642737037	94101.96651	4.649488154	27.56549458
65 to 69	0.015724081	2.615914696	82935.123	3.999105146	19.38949955	0.007213807	2.657769961	91748.71821	4.511609728	23.19917756
70 to 74	0.022771582	2.634395496	76759.36226	3.645073405	15.73427981	0.01104969	2.706851379	88518.11011	4.317322664	18.94338313
75 to 79	0.036336272	2.617002239	68623.90101	3.163560732	12.28716008	0.01968679	2.722663027	83794.13204	4.011699232	14.85277383
80 to 84	0.05857608	2.532029223	57409.42888	2.518683891	9.185186004	0.037465807	2.658482926	75995.55084	3.498296275	11.091702
85 to 89	0.111080515	2.49267522	43155.25903	1.702078233	6.408857502	0.078450422	2.624525305	63132.13593	2.670411713	7.806508069
90 to 94	0.204448496	2.22301574	24959.12468	0.805750815	4.354976317	0.156059671	2.338378902	42684.99811	1.518858523	5.318569227
95 plus	0.30419647	3.2981554	8999.287655	0.303053152	3.2981554	0.255643295	3.929899307	19545.84665	0.781454127	3.929899307

Table 15: Central Asia 2017 life table, by age and sex. mx=mortality rate, ax=mean person-years lived in an age interval among those who die in that age interval, lx=number of persons left alive at age x, nLx=person-years lived between age x and x+n, ex=life expectancy at age x.

Age Group	Male					Female				
	mx	ax	lx	nLx	ex	mx	ax	lx	nLx	ex
Early Neonatal	0.588629614	0.009571	100000	0.019070241	67.28508165	0.383973466	0.009577272	100000	0.019107643	74.74282659
Late Neonatal	0.056574633	0.028751517	98877.48826	0.056795936	68.02961529	0.053063356	0.028752486	99266.3228	0.057025041	75.27598585
Post Neonatal	0.008672136	0.461027782	98556.17959	0.906323985	68.19377141	0.007724851	0.461095075	98963.73519	0.910469349	75.4485221
1 to 4	0.001145143	1.998473144	97770.2461	3.90186723	67.81497053	0.00092928	1.99876096	98260.43368	3.923121752	75.06196715
5 to 9	0.0005085	2.498940626	97323.45758	4.859992311	64.11713576	0.000333827	2.499304526	97895.88032	4.890711344	71.33405544
10 to 14	0.000468057	2.64719397	97076.33973	4.848477599	59.27399883	0.000297171	2.59122421	97732.6189	4.883135669	66.44904514
15 to 19	0.000842877	2.700571514	96849.41012	4.833103463	54.40667673	0.000465443	2.663129367	97587.50808	4.874074249	61.54399377
20 to 24	0.001287914	2.637399901	96442.04292	4.807473819	49.62504587	0.000664289	2.61201594	97360.64926	4.860322655	56.68117044
25 to 29	0.001709302	2.629503657	95822.88756	4.771809694	44.92860573	0.000827095	2.609484279	97037.78693	4.842315266	51.86103592
30 to 34	0.002383985	2.64096921	95007.25297	4.7237972	40.29168458	0.001106617	2.626319098	96637.28808	4.819205879	47.06511259
35 to 39	0.00338797	2.643153793	93881.13108	4.656873069	35.74323721	0.001511875	2.644809626	96103.99802	4.788150822	42.31166011
40 to 44	0.004843918	2.645387139	92303.4491	4.563129781	31.3088896	0.002184368	2.658017877	95380.11042	4.744732989	37.6126448
45 to 49	0.007040719	2.66079507	90093.21636	4.431677404	27.01194843	0.00322445	2.682438157	94343.72699	4.682198118	32.99654655
50 to 54	0.01089044	2.667940212	86973.22498	4.240963079	22.88533204	0.005141116	2.684654698	92834.05339	4.587103166	28.48941863
55 to 59	0.017291888	2.65557027	82355.13456	3.957349513	19.0188242	0.008106377	2.685898218	90475.93793	4.440504268	24.16185106
60 to 64	0.027450154	2.617970285	75513.17974	3.543976185	15.50122747	0.013217865	2.668447455	86876.64045	4.213979886	20.05144652
65 to 69	0.041301068	2.5787235	65787.09476	2.990391884	12.40576797	0.020904242	2.661680412	81307.41713	3.875943734	16.24193677
70 to 74	0.062116782	2.53885652	53439.86525	2.317814843	9.676325542	0.035094674	2.629445327	73206.46538	3.379271196	12.74456211
75 to 79	0.095503571	2.485861078	39049.19612	1.574600382	7.307174678	0.0574197	2.584759681	61350.8397	2.694058134	9.699229296
80 to 84	0.157278336	2.382098606	24019.57813	0.850888044	5.32521435	0.098621539	2.500109518	45887.37482	1.840768243	7.097063414
85 to 89	0.224350545	2.202916108	10646.0165	0.327153303	4.02513057	0.161877649	2.354961336	27742.41912	0.971421484	5.104815054
90 to 94	0.313997562	2.011248223	3311.074123	0.085430343	3.065775084	0.250844654	2.159530813	12025.47962	0.351184256	3.700925183
95 plus	0.390891352	2.558311939	629.9707823	0.01612234	2.558311939	0.342638379	2.918640834	3220.29069	0.094021444	2.918640834

Table 15: Central Asia 2100 life table, by age and sex. mx=mortality rate, ax=mean person-years lived in an age interval among those who die in that age interval, lx=number of persons left alive at age x, nLx=person-years lived between age x and x+n, ex=life expectancy at age x.

Age Group	Male					Female				
	mx	ax	lx	nLx	ex	mx	ax	lx	nLx	ex
Early Neonatal	0.130327375	0.009585047	100000	0.019154137	78.14722908	0.08593157	0.009586407	100000	0.019162289	84.72105312
Late Neonatal	0.013233068	0.028763473	99750.39531	0.057368799	78.32325722	0.015645454	0.028762808	99835.34737	0.057413673	84.84144081
Post Neonatal	0.001577343	0.461531784	99674.48955	0.919612599	78.32515978	0.00161533	0.461529085	99745.53134	0.920251928	84.86016001
1 to 4	0.000161365	1.999784846	99529.47054	3.979894705	77.51527698	0.000138867	1.999814844	99596.92121	3.982771035	84.06264386
5 to 9	0.000118818	2.499752462	99465.27313	4.971786912	73.5640218	9.60137E-05	2.499799971	99541.63706	4.975887729	80.1081881
10 to 14	0.000166546	2.877334267	99406.21003	4.968533164	68.60617856	0.00012992	2.732102234	99493.8768	4.973218068	75.14535649
15 to 19	0.000400612	2.729685996	99323.47943	4.961672389	63.66088327	0.0002305	2.648257535	99429.28448	4.968766631	70.19230594
20 to 24	0.000619739	2.657029109	99124.77094	4.94904797	58.78248222	0.00028971	2.619422047	99314.78681	4.962316079	65.26999687
25 to 29	0.000862756	2.65462997	98818.29815	4.930925421	53.95543674	0.000395744	2.648081152	99171.06728	4.953937682	60.36055804
30 to 34	0.001246576	2.633988583	98393.39328	4.905185449	49.17573199	0.000566412	2.633979457	98975.1006	4.942116855	55.47440459
35 to 39	0.001640564	2.600509631	97783.01566	4.8699996	44.46481423	0.00074575	2.623939386	98695.30826	4.926051037	50.62363105
40 to 44	0.002040868	2.613771798	96986.00275	4.825888458	39.80672976	0.001025869	2.65956822	98328.15761	4.904666747	45.80186694
45 to 49	0.002830932	2.690570605	96004.03791	4.768966367	35.18452302	0.001570586	2.680428222	97825.45774	4.873475064	41.02222429
50 to 54	0.00451348	2.710287534	94661.053	4.684685449	30.64035254	0.002338375	2.682935908	97061.65287	4.82685285	36.32147986
55 to 59	0.007487648	2.676402598	92556.32296	4.548862888	26.26902287	0.003540053	2.668038901	95936.24933	4.757489762	31.71202541
60 to 64	0.011151085	2.640330022	89172.83407	4.345037461	22.15481758	0.005154903	2.652402089	94257.90275	4.656661092	27.22462661
65 to 69	0.016358868	2.628213214	84363.80199	4.062198193	18.25655522	0.007516024	2.662931938	91867.23818	4.514386962	22.85808946
70 to 74	0.023943697	2.662175988	77801.96466	3.686741036	14.56263472	0.011642178	2.712284629	88493.25837	4.310614047	18.62107726
75 to 79	0.04245688	2.657476693	69126.9998	3.149932611	11.04837639	0.021036262	2.722401774	83522.81065	3.987043437	14.55832755
80 to 84	0.080173885	2.537560967	56077.03873	2.355682164	8.003072265	0.040063321	2.659965405	75246.91659	3.445714712	10.85106904
85 to 89	0.138970311	2.41228282	37882.88276	1.410821685	5.649581516	0.082445212	2.609378307	61732.34611	2.590174895	7.635044622
90 to 94	0.233510528	2.175585844	19164.0745	0.587369031	3.941650924	0.161039934	2.331622837	40993.23803	1.4467902	5.212757291
95 plus	0.327904328	3.061565183	5977.005479	0.187862587	3.061565183	0.260354384	3.863206182	18375.15192	0.725290341	3.863206182

Table 15: Armenia 2017 life table, by age and sex. mx=mortality rate, ax=mean person-years lived in an age interval among those who die in that age interval, lx=number of persons left alive at age x, nLx=person-years lived between age x and x+n, ex=life expectancy at age x.

Age Group	Male					Female				
	mx	ax	lx	nLx	ex	mx	ax	lx	nLx	ex
Early Neonatal	0.234563258	0.009581852	100000	0.019135011	72.33299649	0.1963957	0.009583022	100000	0.019142011	78.78454157
Late Neonatal	0.024376516	0.028760399	99551.1663	0.057235869	72.63988697	0.018472767	0.028762028	99624.06203	0.057287505	79.06262319
Post Neonatal	0.003004725	0.461430385	99411.64786	0.916583528	72.6842582	0.00255841	0.46146209	99518.23642	0.917755262	79.08913175
1 to 4	0.000423462	1.999435385	99136.24705	3.962093388	71.96160911	0.000263625	1.999648501	99283.44137	3.96924453	78.35179251
5 to 9	0.000252397	2.499474173	98968.47089	4.945302503	68.08021216	0.000177533	2.499630139	99178.804	4.956739937	74.43234939
10 to 14	0.000204876	2.856546072	98843.65553	4.940013195	63.16302623	0.000171775	2.471289056	99090.80653	4.952389135	69.49623015
15 to 19	0.000604244	2.69788387	98742.44798	4.930264243	58.2248374	0.000154095	2.663251322	99005.73751	4.948505053	64.55381843
20 to 24	0.000781991	2.553941918	98444.5409	4.912829816	53.39285773	0.000293023	2.595785213	98929.48366	4.942991925	59.6015197
25 to 29	0.000812056	2.570540804	98060.36345	4.893364292	48.59201687	0.000289429	2.597859019	98784.64318	4.935800571	54.68509598
30 to 34	0.001064929	2.641784725	97662.99677	4.870917474	43.77925191	0.000430162	2.693898182	98641.78732	4.927201672	49.76052301
35 to 39	0.001550311	2.767014822	97144.28152	4.840457496	38.99889161	0.000692228	2.730870385	98429.83895	4.913773622	44.86186057
40 to 44	0.003093504	2.72569868	96393.86658	4.786021479	34.28092235	0.001203979	2.727527739	98089.69555	4.89110264	40.00793802
45 to 49	0.005017632	2.732799866	94913.33063	4.692288232	29.77308872	0.002026245	2.693946369	97500.82243	4.852367933	35.2330698
50 to 54	0.009071271	2.674905092	92558.97219	4.532357306	25.46080955	0.003139186	2.708504537	96517.62748	4.791415194	30.56449191
55 to 59	0.013547287	2.645471334	88447.71846	4.285690539	21.51982964	0.005302054	2.700450349	95013.54552	4.693454637	26.0053979
60 to 64	0.020734369	2.622203848	82642.14066	3.93797268	17.84562183	0.008592803	2.704502556	92525.13343	4.536774055	21.63208461
65 to 69	0.030545994	2.590666446	74477.73766	3.468642266	14.5143372	0.014731249	2.713310429	88626.97312	4.2869505	17.4644784
70 to 74	0.044273373	2.586022071	63883.81325	2.885825036	11.49160111	0.026735094	2.695796422	82312.29334	3.876821513	13.59597705
75 to 79	0.073320048	2.537206876	51109.73338	2.164705088	8.717489541	0.049885419	2.640203164	71949.09048	3.2186428	10.16582249
80 to 84	0.114657776	2.434305125	35242.35138	1.361681429	6.500474386	0.093078612	2.540858515	55896.69044	2.274419625	7.327024671
85 to 89	0.17944428	2.303524781	19634.84637	0.661688248	4.733590484	0.155519208	2.371255205	34734.31465	1.232904635	5.243648424
90 to 94	0.273321638	2.098033208	7765.074596	0.216546577	3.449745024	0.24431295	2.17466633	15568.25498	0.460610662	3.781372825
95 plus	0.359714228	2.780022332	1847.895518	0.051380797	2.780022332	0.33690926	2.968270082	4319.290187	0.128246149	2.968270082

Table 15: Armenia 2100 life table, by age and sex. mx=mortality rate, ax=mean person-years lived in an age interval among those who die in that age interval, lx=number of persons left alive at age x, nLx=person-years lived between age x and x+n, ex=life expectancy at age x.

Age Group	Male					Female				
	mx	ax	lx	nLx	ex	mx	ax	lx	nLx	ex
Early Neonatal	0.042245857	0.009587746	100000	0.019170316	79.79408222	0.036297567	0.009587929	100000	0.019171409	84.4022565
Late Neonatal	0.004072629	0.028766	99919.02786	0.057480927	79.83956938	0.005797989	0.028765524	99930.42258	0.057484631	84.44157689
Post Neonatal	0.000537427	0.461605658	99895.62327	0.922095297	79.8005976	0.000540894	0.461605411	99897.10788	0.922107524	84.41192384
1 to 4	0.000169472	1.999774038	99846.09912	3.992500186	78.91500257	0.0001549	1.999793468	99847.26292	3.992664201	83.52925536
5 to 9	0.000148299	2.499691047	99779.05411	4.987141246	74.95937149	0.000140741	2.499706791	99786.11364	4.987586584	79.57339052
10 to 14	0.000193808	3.056127908	99706.78068	4.983266988	70.00456599	0.000182311	2.525463955	99717.50609	4.98367298	74.62189625
15 to 19	0.000495126	2.705997911	99614.32304	4.975052	65.05862067	0.000149105	2.66308575	99629.18512	4.979702559	69.68225898
20 to 24	0.000592768	2.615544716	99375.75296	4.961821862	60.1991421	0.000210134	2.648184106	99557.8602	4.975419993	64.7280644
25 to 29	0.00054595	2.621924817	99094.20091	4.948425197	55.35563879	0.000203565	2.653553743	99456.80821	4.970484229	59.78959955
30 to 34	0.000677159	2.633465657	98839.64424	4.934352325	50.48820384	0.000279644	2.701599092	99359.83841	4.964854985	54.84433845
35 to 39	0.00087306	2.724005344	98523.67586	4.916674963	45.64037809	0.000408293	2.764117438	99226.07898	4.956802327	49.91401391
40 to 44	0.001520355	2.718953092	98115.31432	4.889093574	40.81863394	0.000685231	2.796958773	99029.57674	4.944022769	45.00698925
45 to 49	0.002416573	2.757190471	97396.72494	4.843874377	36.09901855	0.001226472	2.716891896	98698.20698	4.92123326	40.14824467
50 to 54	0.004588703	2.700000193	96255.86285	4.762976646	31.49260689	0.00187738	2.687843183	98103.36245	4.884189095	35.37460644
55 to 59	0.007216113	2.656148486	94110.4012	4.62802429	27.14315704	0.002925847	2.691586358	97197.57301	4.827473726	30.67819506
60 to 64	0.01107955	2.619793843	90820.19499	4.425793268	23.0239232	0.004491736	2.688972433	95801.56865	4.741264023	26.08372148
65 to 69	0.016027012	2.614571578	85985.85945	4.143600475	19.16142181	0.007119673	2.745724268	93694.87086	4.611248551	21.60770231
70 to 74	0.024666449	2.636888135	79471.16928	3.759107792	15.50237863	0.013416095	2.77536391	90444.98436	4.392160923	17.28145478
75 to 79	0.041939228	2.557720838	70450.61911	3.203271674	12.14064302	0.027566251	2.713007516	84610.20523	3.982340529	13.27497345
80 to 84	0.060882564	2.486281098	57388.03223	2.512471985	9.31334905	0.052664597	2.623018057	73770.35537	3.288163866	9.809773991
85 to 89	0.112009171	2.494682837	43202.51716	1.723623835	6.521968464	0.101017348	2.542506595	56944.48682	2.300155892	6.908237864
90 to 94	0.203625184	2.211347699	25781.7186	0.850622878	4.426579533	0.18345333	2.296799148	34710.26689	1.178701001	4.768873123
95 plus	0.303082589	3.339310477	9976.051636	0.353640121	3.339310477	0.281355041	3.584144244	14053.30763	0.521351442	3.584144244

Table 15: Azerbaijan 2017 life table, by age and sex. mx=mortality rate, ax=mean person-years lived in an age interval among those who die in that age interval, lx=number of persons left alive at age x, nLx=person-years lived between age x and x+n, ex=life expectancy at age x.

Age Group	Male					Female				
	mx	ax	lx	nLx	ex	mx	ax	lx	nLx	ex
Early Neonatal	1.079461942	0.009555956	100000	0.018980961	67.03962378	0.569886362	0.009571574	100000	0.019073667	74.71221069
Late Neonatal	0.070050589	0.0287478	97951.52574	0.056242311	68.42167753	0.088948757	0.028742587	98913.10928	0.05676357	75.51361467
Post Neonatal	0.010685449	0.46088476	97557.73141	0.896312368	68.64024431	0.013652247	0.460674005	98408.3098	0.902891113	75.84325719
1 to 4	0.001210454	1.998386062	96600.55766	3.854689134	68.39277762	0.000953995	1.998728007	97176.07024	3.879638741	75.87602512
5 to 9	0.000572588	2.498807109	96134.28131	4.799842955	64.71500252	0.000579258	2.498793212	96806.10485	4.833303911	72.15846701
10 to 14	0.000434385	2.635317372	95859.56871	4.788060537	59.89337658	0.000293946	2.345712222	96526.18703	4.822547275	67.36050795
15 to 19	0.00085762	2.670539684	95651.63594	4.773047435	55.01785734	0.000363521	2.618992095	96384.44459	4.815055056	62.45610828
20 to 24	0.001143947	2.540581638	95242.32163	4.748756107	50.24274171	0.000503415	2.617145506	96209.41427	4.804707585	57.56493175
25 to 29	0.001090751	2.529666755	94699.11091	4.722231822	45.51624756	0.00064936	2.600925777	95967.5513	4.790914487	52.70336334
30 to 34	0.001313199	2.656398917	94184.05597	4.694755354	40.75121784	0.000822519	2.600365236	95656.4669	4.773402773	47.86623363
35 to 39	0.002100774	2.722930087	93567.57307	4.656107663	36.00208392	0.001053198	2.652797802	95263.86834	4.751448162	43.05269403
40 to 44	0.00362333	2.693443927	92589.4966	4.591109276	31.35341426	0.00160917	2.695775662	94763.47584	4.720670443	38.26589638
45 to 49	0.005620867	2.711352518	90926.17151	4.488575391	26.87734111	0.002597971	2.727140754	94003.88978	4.672605199	33.55314014
50 to 54	0.009768232	2.696296766	88403.66002	4.322926127	22.56640779	0.004536292	2.692507923	92790.07224	4.591447823	28.95613651
55 to 59	0.016016552	2.683164982	84182.12588	4.058556542	18.5621805	0.007030242	2.716472201	90707.53493	4.463728886	24.5587363
60 to 64	0.027206829	2.658678398	77684.5042	3.651739361	14.88958295	0.01255267	2.715386538	87570.04826	4.25646826	20.34081333
65 to 69	0.046151717	2.582147022	67755.39078	3.047956412	11.68128948	0.022156574	2.668398683	82228.64413	3.909556323	16.4850199
70 to 74	0.067272878	2.522678385	53701.61586	2.301976108	9.062429377	0.03649999	2.601861992	73570.70156	3.382678364	13.11012755
75 to 79	0.103990387	2.483443503	38237.50456	1.515966326	6.708636043	0.054385851	2.558600926	61234.41063	2.70324893	10.22661096
80 to 84	0.189595611	2.374868744	22504.64302	0.751955106	4.666687829	0.085990754	2.505816763	46552.19974	1.917159117	7.645918962
85 to 89	0.257475335	2.138627959	8282.466133	0.238730199	3.613721445	0.146268473	2.396088252	30095.74681	1.090352126	5.459903306
90 to 94	0.34302079	1.951000967	2150.820428	0.052631373	2.836214154	0.234670654	2.196818629	14177.44705	0.427919134	3.906794795
95 plus	0.412617335	2.423754904	349.1572179	0.008474909	2.423754904	0.328374695	3.045773891	4152.306924	0.126629748	3.045773891

Table 15: Azerbaijan 2100 life table, by age and sex. mx=mortality rate, ax=mean person-years lived in an age interval among those who die in that age interval, lx=number of persons left alive at age x, nLx=person-years lived between age x and x+n, ex=life expectancy at age x.										
Age Group	Male					Female				
	mx	ax	lx	nLx	ex	mx	ax	lx	nLx	ex
Early Neonatal	0.222058092	0.009582235	100000	0.019137316	78.96732453	0.128954204	0.009585089	100000	0.019154391	85.16768516
Late Neonatal	0.010598354	0.0287642	99575.23318	0.057272403	79.28431368	0.025021368	0.028760221	99753.05375	0.057350891	85.35853047
Post Neonatal	0.001948997	0.461505382	99514.55669	0.91797972	79.27510536	0.003177289	0.461418126	99609.61048	0.918336246	85.42344401
1 to 4	0.000194347	1.999740871	99335.71854	3.971885508	78.493874	0.000168464	1.999775381	99318.00956	3.971383103	84.74917325
5 to 9	0.000126681	2.499736081	99258.56874	4.961358298	74.55336207	0.000191708	2.499600608	99251.1543	4.960181861	80.80485853
10 to 14	0.000158648	2.839534768	99195.77465	4.958019147	69.59895228	0.000141483	2.503755764	99156.1389	4.956044077	75.87947128
15 to 19	0.000344827	2.668186634	99117.17567	4.951854721	64.65203742	0.000181132	2.586992474	99086.07094	4.952135287	70.93111988
20 to 24	0.00045329	2.546181514	98946.58469	4.941816406	59.75863264	0.000212634	2.615140966	98996.41692	4.947313657	65.99280977
25 to 29	0.000429037	2.524004537	98722.99758	4.930929517	54.88784858	0.000299748	2.626320364	98891.26557	4.941042487	61.06000579
30 to 34	0.000517009	2.636483135	98511.82293	4.919566333	49.99973838	0.000388298	2.595160246	98743.24619	4.932550819	56.14716001
35 to 39	0.000784402	2.688477686	98257.96153	4.903963335	45.12084331	0.000470321	2.654118561	98551.84219	4.922159699	51.2505354
40 to 44	0.001253355	2.667084313	97873.92096	4.879428193	40.28486562	0.000734179	2.72496638	98320.49054	4.907813552	46.36399772
45 to 49	0.001903043	2.732678941	97263.37846	4.842236853	35.51702244	0.001251972	2.738308776	97960.42367	4.884166052	41.52317947
50 to 54	0.003586819	2.71868525	96344.49201	4.778126044	30.8224309	0.002150594	2.67330139	97349.53771	4.843231107	36.76445352
55 to 59	0.006116864	2.70483522	94638.55733	4.666406301	26.31592935	0.00305036	2.679916447	96309.30225	4.781653342	32.12935334
60 to 64	0.010153932	2.702646111	91811.60029	4.486656131	22.01939135	0.004874933	2.702850725	94853.56437	4.690355539	27.5762226
65 to 69	0.018261115	2.61625894	87316.20505	4.187021841	17.98506721	0.00819152	2.672237964	92574.12398	4.542492061	23.17983578
70 to 74	0.024890183	2.623235196	79846.60711	3.776075536	14.38101732	0.012237356	2.671416982	88879.98782	4.321944771	19.01638673
75 to 79	0.043328583	2.673837324	70810.88212	3.232231803	10.84637132	0.019916222	2.700856804	83653.69979	4.002343857	15.01959041
80 to 84	0.095082502	2.535322952	57726.38056	2.386899189	7.678154575	0.037171696	2.665371173	75830.32481	3.498996232	11.27054802
85 to 89	0.154034714	2.38078114	37577.24576	1.399187105	5.459591709	0.077368467	2.632253346	63287.28082	2.694358607	7.94938406
90 to 94	0.246629695	2.144124929	19124.3496	0.594376453	3.840742722	0.153730859	2.333203586	43499.33138	1.566740495	5.41783795
95 plus	0.337782212	3.002965484	6268.995793	0.204896379	3.002965484	0.25322206	3.993720948	20655.92115	0.855937032	3.993720948

Table 15: Georgia 2017 life table, by age and sex. mx=mortality rate, ax=mean person-years lived in an age interval among those who die in that age interval, lx=number of persons left alive at age x, nLx=person-years lived between age x and x+n, ex=life expectancy at age x.

Age Group	Male					Female				
	mx	ax	lx	nLx	ex	mx	ax	lx	nLx	ex
Early Neonatal	0.260146994	0.009581068	100000	0.019130322	68.35254379	0.208065609	0.009582664	100000	0.019139871	77.24309745
Late Neonatal	0.040690804	0.028755899	99502.34968	0.057180973	68.67513037	0.028764093	0.028759189	99601.77478	0.057257742	77.53269089
Post Neonatal	0.003664919	0.461383486	99269.69781	0.914996138	68.77847489	0.002845988	0.461441661	99437.08764	0.916885303	77.60351823
1 to 4	0.000433423	1.999422102	98934.40536	3.953948239	68.08673203	0.000349609	1.999533855	99176.16479	3.964274272	76.88319407
5 to 9	0.000275037	2.499427006	98763.05778	4.934759363	64.20139986	0.000183213	2.499618307	99037.58205	4.949611792	72.98798476
10 to 14	0.000362336	2.792851181	98627.34807	4.927425455	59.28630853	0.000151436	2.707361433	98946.90352	4.94562786	68.05258568
15 to 19	0.000785335	2.763085231	98448.82169	4.913808801	54.38875856	0.000334185	2.680715478	98872.01093	4.939771931	63.10208227
20 to 24	0.00136214	2.627564702	98062.93089	4.887352713	49.59190396	0.000442385	2.550154566	98706.93289	4.930003717	58.20312518
25 to 29	0.001635733	2.626217367	97397.21095	4.851024768	44.91289082	0.000442332	2.642904828	98488.83941	4.919313062	53.32635402
30 to 34	0.002386809	2.651355044	96603.72016	4.803260392	40.26020691	0.000748948	2.656584091	98271.24496	4.90495371	48.43856896
35 to 39	0.003434917	2.672427013	95457.28511	4.735008333	35.71185586	0.001010966	2.646191749	97903.89395	4.883573734	43.61033409
40 to 44	0.005379136	2.686776339	93830.86953	4.633884482	31.28449724	0.001470525	2.691668255	97410.18559	4.854032484	38.81792926
45 to 49	0.008625219	2.661216451	91338.29014	4.476612514	27.06484834	0.002392321	2.70617579	96696.39688	4.80843351	34.08457215
50 to 54	0.012913653	2.629681378	87477.23917	4.243962126	23.14188079	0.003911531	2.718748399	95546.08564	4.735053346	29.46230174
55 to 59	0.018525462	2.604522206	81997.00469	3.92565123	19.51271019	0.006709973	2.667412174	93694.00249	4.612509384	24.99088717
60 to 64	0.025965559	2.584534676	74725.05961	3.515766915	16.15807147	0.009797357	2.669729125	90599.14247	4.428849718	20.75336333
65 to 69	0.036395128	2.595601693	65597.00642	3.015959896	13.04683983	0.015934853	2.703174446	86260.29454	4.160746045	16.66285495
70 to 74	0.059396601	2.556811789	54621.73447	2.38503491	10.14685667	0.028938479	2.707650108	79630.81397	3.733879255	12.82490635
75 to 79	0.089992775	2.480774472	40457.82209	1.649106126	7.80423528	0.057427996	2.634677254	68827.16652	3.029885692	9.412859877
80 to 84	0.134920917	2.402074165	25620.47214	0.9486166	5.887659684	0.108934586	2.518496712	51431.40966	2.024502144	6.705502232
85 to 89	0.201128349	2.252880985	12825.15011	0.413083764	4.366118943	0.174537953	2.32410329	29385.54425	1.00166522	4.847439791
90 to 94	0.293217228	2.05552427	4518.962896	0.121270835	3.251782999	0.26367075	2.129730247	11909.95815	0.339032429	3.551701084
95 plus	0.375085487	2.666082158	963.7917371	0.0256989	2.666082158	0.3537798	2.826712545	2974.134158	0.084095234	2.826712545

Table 15: Georgia 2100 life table, by age and sex. mx=mortality rate, ax=mean person-years lived in an age interval among those who die in that age interval, lx=number of persons left alive at age x, nLx=person-years lived between age x and x+n, ex=life expectancy at age x.

Age Group	Male					Female				
	mx	ax	lx	nLx	ex	mx	ax	lx	nLx	ex
Early Neonatal	0.04729434	0.009587592	100000	0.019169389	78.204941	0.039756326	0.009587823	100000	0.019170773	85.71042268
Late Neonatal	0.016412501	0.028762596	99909.36876	0.057454978	78.25620572	0.010049133	0.028764351	99923.79232	0.057473789	85.75627293
Post Neonatal	0.000609239	0.461600556	99815.09767	0.92132147	78.27221936	0.000504525	0.461607995	99866.05455	0.921836326	85.74789697
1 to 4	8.24977E-05	1.999890003	99758.99736	3.989701766	77.39246056	7.22904E-05	1.999903613	99819.56512	3.992205569	84.86404345
5 to 9	6.20857E-05	2.499870655	99726.09331	4.985531472	73.41728439	5.16973E-05	2.499892296	99790.71535	4.98889145	80.88792988
10 to 14	0.000125536	3.138405834	99695.17215	4.983456559	68.43897891	6.53138E-05	2.973267911	99764.94466	4.987549698	75.9079585
15 to 19	0.000366124	2.756821168	99632.69114	4.977503509	63.47957847	0.000166558	2.693065176	99732.39841	4.984682388	70.93150506
20 to 24	0.000601355	2.630734176	99450.76203	4.965368665	58.58794532	0.000199102	2.541479096	99649.45214	4.980025815	65.98759939
25 to 29	0.000648034	2.672653101	99153.33808	4.950016031	53.75165919	0.000195651	2.692841303	99550.38567	4.975253561	61.05008452
30 to 34	0.000950061	2.656863772	98834.10712	4.930688721	48.91290714	0.000362482	2.677074303	99453.14869	4.96845929	56.10642796
35 to 39	0.00135429	2.683770804	98367.57307	4.902992361	44.12863042	0.000491643	2.642230157	99273.25272	4.957937859	51.20178806
40 to 44	0.002197927	2.769195993	97707.42881	4.861196581	39.40300112	0.000721641	2.77266739	99029.8199	4.943452093	46.31901527
45 to 49	0.003951084	2.692563088	96646.36511	4.788701359	34.79750856	0.00135379	2.736665761	98673.90819	4.91854683	41.47270817
50 to 54	0.006100014	2.673257347	94764.90434	4.672266465	30.42453241	0.002191715	2.727421692	98009.91796	4.876180188	36.72967689
55 to 59	0.009877886	2.626579816	91935.07786	4.492426642	26.26317675	0.003718618	2.64005341	96946.18863	4.805238674	32.09396785
60 to 64	0.013631132	2.576920466	87547.88546	4.239601882	22.42225111	0.004684109	2.599391963	95172.5551	4.706076244	27.63032806
65 to 69	0.017230424	2.625256641	81870.61187	3.936050346	18.76954349	0.006033355	2.678622532	92992.56398	4.5860503	23.2040226
70 to 74	0.028242856	2.607434676	75270.41804	3.532445406	15.15933189	0.010288291	2.749904062	90263.38395	4.412512719	18.81097834
75 to 79	0.042407747	2.565799931	65633.56186	2.991044775	11.97983376	0.019899405	2.774612932	85812.84546	4.112687845	14.62351901
80 to 84	0.064459355	2.524918843	53728.03981	2.347163181	9.06848103	0.042680046	2.674116097	77882.42488	3.559093801	10.80076366
85 to 89	0.117110877	2.479317334	40017.73625	1.584970218	6.352198399	0.085622248	2.602167303	63462.16829	2.663342724	7.615349254
90 to 94	0.209356108	2.205940453	23466.30974	0.767552591	4.330527428	0.163807746	2.318633235	42270.74877	1.502603871	5.212354914
95 plus	0.307842483	3.284222947	8845.814392	0.308316634	3.284222947	0.262685775	3.864363348	19420.46276	0.791150873	3.864363348

Table 15: Kazakhstan 2017 life table, by age and sex. mx=mortality rate, ax=mean person-years lived in an age interval among those who die in that age interval, lx=number of persons left alive at age x, nLx=person-years lived between age x and x+n, ex=life expectancy at age x.

Age Group	Male					Female				
	mx	ax	lx	nLx	ex	mx	ax	lx	nLx	ex
Early Neonatal	0.286103032	0.009580272	100000	0.019125567	67.23539608	0.212310241	0.009582534	100000	0.019139093	76.27108079
Late Neonatal	0.042045003	0.028755525	99452.85357	0.057150303	67.58602582	0.032822686	0.028758069	99593.67974	0.057246406	76.56301326
Post Neonatal	0.005344349	0.461264182	99212.58379	0.913761575	67.69208381	0.004372341	0.461333232	99405.79384	0.915951535	76.65013112
1 to 4	0.000795277	1.998939631	98724.30885	3.942698957	67.10132307	0.000623392	1.999168811	99005.34815	3.955281036	76.03502041
5 to 9	0.000375688	2.499217317	98410.81086	4.91592266	63.30873869	0.000252735	2.499473469	98758.80945	4.934822128	72.21986152
10 to 14	0.000377391	2.740472088	98226.15369	4.907122232	58.42307244	0.000242269	2.648115812	98634.10208	4.928896369	67.30802147
15 to 19	0.000812809	2.796058529	98040.9804	4.893283454	53.5282443	0.000425515	2.632952297	98514.69641	4.920778712	62.38639059
20 to 24	0.001542375	2.697375006	97643.26077	4.864885436	48.7348498	0.000515499	2.621250777	98305.31372	4.909245968	57.51364228
25 to 29	0.002300398	2.676045886	96892.92385	4.818884727	44.0912888	0.000728837	2.677427625	98052.24782	4.894327591	52.65529083
30 to 34	0.003552112	2.640597495	95784.41023	4.749417747	39.57048851	0.001143078	2.660280978	97695.53921	4.871748061	47.83773448
35 to 39	0.004831736	2.604320332	94097.4132	4.651036415	35.23243051	0.001622944	2.634430951	97138.67392	4.838358736	43.09666527
40 to 44	0.006215496	2.603871996	91850.26228	4.525124955	31.0304934	0.002219219	2.63536734	96353.45372	4.792523618	38.42631494
45 to 49	0.008360382	2.632078267	89037.89169	4.36548144	26.92816068	0.003120717	2.656108777	95289.92672	4.729900073	33.82566867
50 to 54	0.012356072	2.646521963	85388.61	4.148801984	22.96626548	0.004674923	2.684028237	93813.93487	4.640457468	29.31593735
55 to 59	0.01888847	2.622436089	80263.16828	3.840712637	19.26358328	0.007532904	2.667822329	91644.71186	4.503131361	24.9461506
60 to 64	0.027405619	2.607192683	73010.27345	3.42591613	15.91650088	0.011402908	2.674020345	88252.89609	4.29864764	20.80215651
65 to 69	0.041871437	2.558827971	63624.06664	2.886293144	12.87986441	0.018754408	2.653319262	83351.9153	3.991945751	16.867817
70 to 74	0.056616102	2.532040665	51543.51058	2.261373866	10.29901172	0.029330481	2.661434682	75866.96912	3.549935866	13.26991846
75 to 79	0.088692019	2.491571515	38747.52108	1.585005655	7.864597487	0.053592673	2.617689706	65458.64111	2.902546806	9.956542194
80 to 84	0.133704978	2.387076988	24699.88929	0.915456091	5.921996979	0.095085485	2.517220446	49911.8018	2.019257814	7.242736311
85 to 89	0.199524048	2.256513478	12470.11004	0.403063838	4.391820285	0.157737537	2.365538432	30726.322	1.085603578	5.194817333
90 to 94	0.291760863	2.058642845	4434.247987	0.119356626	3.26568384	0.246595509	2.169380246	13616.79477	0.401108612	3.753077246
95 plus	0.373967368	2.67410054	953.9767592	0.025520641	2.67410054	0.338913552	2.950811078	3733.271817	0.110226567	2.950811078

Table 15: Kazakhstan 2100 life table, by age and sex. mx=mortality rate, ax=mean person-years lived in an age interval among those who die in that age interval, lx=number of persons left alive at age x, nLx=person-years lived between age x and x+n, ex=life expectancy at age x.

Age Group	Male					Female				
	mx	ax	lx	nLx	ex	mx	ax	lx	nLx	ex
Early Neonatal	0.070313355	0.009586886	100000	0.019165162	78.82562426	0.047784521	0.009587577	100000	0.019169299	84.9754881
Late Neonatal	0.012521048	0.028763669	99865.32222	0.057436077	78.91150447	0.009604978	0.028764474	99908.43108	0.057465688	85.03396
Post Neonatal	0.000970581	0.461574887	99793.43195	0.920967799	78.91028891	0.000929966	0.461577772	99853.25293	0.921537135	85.02334274
1 to 4	0.000119234	1.999841022	99704.06112	3.987211767	78.05739959	9.81821E-05	1.99986909	99767.56629	3.989919325	84.1725178
5 to 9	7.14176E-05	2.499851213	99656.53171	4.981937147	74.09370708	7.14672E-05	2.49985111	99728.40311	4.985529591	80.20472383
10 to 14	0.000111906	3.088767847	99620.95707	4.979928151	69.11928615	9.82845E-05	2.865757167	99692.78316	4.983559993	75.23237232
15 to 19	0.000344315	2.83679139	99565.2376	4.974577828	64.15637391	0.000211236	2.63068561	99643.82154	4.979707465	70.26780108
20 to 24	0.000711064	2.747372007	99394.0073	4.961682384	59.26097403	0.000238896	2.633347532	99538.66125	4.974116797	65.33892425
25 to 29	0.001140235	2.751075756	99041.7965	4.939262429	54.45868446	0.000359546	2.73516175	99419.90993	4.966905981	60.41328483
30 to 34	0.001921818	2.659673365	98480.81319	4.901946768	49.74962498	0.000598745	2.677040106	99241.5414	4.955108888	55.51594425
35 to 39	0.002496515	2.563834501	97544.65877	4.848002279	45.19693824	0.000787431	2.629384633	98945.3351	4.938034104	50.6726988
40 to 44	0.00273087	2.562120872	96346.38172	4.785773659	40.72033355	0.001064431	2.649202851	98557.21845	4.915532624	45.85992085
45 to 49	0.00331806	2.658761203	95055.80665	4.716455471	36.23229087	0.001507439	2.665787523	98035.28466	4.884525366	41.08769209
50 to 54	0.005131812	2.688197629	93515.63258	4.621328189	31.77487837	0.002182333	2.691212237	97301.54561	4.840628884	36.37431727
55 to 59	0.007812604	2.642841941	91181.78419	4.477693491	27.501902	0.003421332	2.678602539	96249.39915	4.774486037	31.73820788
60 to 64	0.010519697	2.648592551	87755.44356	4.283604066	23.45021064	0.004973749	2.678348305	94625.13295	4.677324064	27.23137989
65 to 69	0.016016492	2.615998479	83355.8789	4.018158995	19.52714548	0.007717258	2.657736513	92314.07378	4.534189785	22.83918536
70 to 74	0.02163596	2.656379441	77103.94348	3.675023969	15.87531499	0.011254541	2.725679763	88844.73739	4.332145387	18.6192225
75 to 79	0.037578428	2.625838142	69469.38789	3.200787723	12.31709976	0.021371566	2.730304093	84028.31559	4.009365212	14.52116951
80 to 84	0.0596699	2.5279468	58027.04078	2.550461951	9.237847168	0.040662286	2.669726799	75605.82899	3.460389661	10.82339107
85 to 89	0.111642451	2.493084981	43842.68314	1.742693254	6.454632739	0.083248398	2.607041198	61903.84061	2.595833274	7.616848102
90 to 94	0.204237437	2.217625668	25875.33405	0.845777516	4.384241941	0.161873003	2.329261328	41067.42073	1.449910404	5.20303118
95 plus	0.303828258	3.31500896	9704.728922	0.33574818	3.31500896	0.261101168	3.857265118	18439.68139	0.730103133	3.857265118

Table 15: Kyrgyzstan 2017 life table, by age and sex. mx=mortality rate, ax=mean person-years lived in an age interval among those who die in that age interval, lx=number of persons left alive at age x, nLx=person-years lived between age x and x+n, ex=life expectancy at age x.

Age Group	Male					Female				
	mx	ax	lx	nLx	ex	mx	ax	lx	nLx	ex
Early Neonatal	0.700072765	0.009567584	100000	0.019049916	68.97115392	0.554130236	0.009572057	100000	0.019076539	76.06126014
Late Neonatal	0.031798677	0.028758352	98666.39965	0.056715076	69.88404982	0.028734816	0.028759197	98942.92081	0.056879036	76.85458771
Post Neonatal	0.005057735	0.461284543	98486.06221	0.907189971	69.95442998	0.004274898	0.461340154	98779.48922	0.9102214	76.92417641
1 to 4	0.000863975	1.998848034	98027.2572	3.914322779	69.35641076	0.000722406	1.999036793	98390.38789	3.929934734	76.30327877
5 to 9	0.000366931	2.499235561	97689.08161	4.879976306	65.589588	0.000178528	2.499628066	98106.48895	4.903135778	72.51829032
10 to 14	0.000441393	2.60867458	97510.02542	4.870360508	60.70544312	0.000270593	2.698461476	98018.95518	4.897897447	67.58081994
15 to 19	0.000599304	2.67264746	97295.05467	4.857976951	55.83380578	0.000437241	2.607738864	97886.42254	4.889207096	62.66866489
20 to 24	0.000942767	2.677191333	97003.91595	4.839597737	50.99335089	0.000498404	2.566879938	97672.6468	4.877717294	57.80011302
25 to 29	0.00141156	2.676849885	96547.65644	4.811604346	46.22166764	0.000599915	2.640259331	97429.5402	4.864590522	52.93792254
30 to 34	0.002165331	2.705627839	95868.47259	4.769727488	41.53014474	0.000906991	2.680765282	97137.70723	4.846690357	48.08902204
35 to 39	0.003612544	2.658794327	94835.67505	4.70201595	36.95293234	0.001397093	2.672395025	96698.11927	4.819234488	43.29543088
40 to 44	0.005058267	2.622672437	93137.06847	4.601520358	32.57833332	0.002086252	2.628941866	96024.83281	4.777608728	38.58023524
45 to 49	0.006869858	2.626450818	90809.53688	4.467629706	28.34606654	0.002732714	2.644594192	95028.11536	4.721018543	33.95728051
50 to 54	0.009770002	2.662888314	87740.42274	4.289089158	24.24565862	0.004074376	2.723243277	93738.01737	4.643823844	29.38819101
55 to 59	0.01575093	2.653106804	83550.14783	4.028594549	20.32802267	0.00734602	2.703240001	91845.99196	4.516105791	24.93743681
60 to 64	0.024259293	2.619796472	77205.08473	3.649536891	16.7805505	0.011905881	2.680324878	88528.56079	4.307470606	20.7705403
65 to 69	0.036412016	2.579415541	68352.21706	3.140812318	13.61457147	0.019440124	2.678447317	83400.38848	3.989960389	16.88278322
70 to 74	0.052389665	2.549603922	56917.0676	2.522121628	10.83163143	0.033608423	2.626585289	75644.45758	3.502845732	13.33905999
75 to 79	0.080065513	2.504332511	43705.70297	1.821410208	8.335246055	0.052641876	2.576101818	63873.45151	2.832339302	10.31314469
80 to 84	0.122338289	2.400592476	29125.41887	1.104970024	6.254600453	0.085434482	2.485745622	48966.47823	2.015502367	7.668662546
85 to 89	0.187436607	2.284390668	15610.61634	0.517292065	4.59195404	0.145406486	2.39833494	31751.7641	1.151944211	5.479152088
90 to 94	0.280720708	2.082268097	5916.820903	0.162648161	3.373658252	0.233781277	2.198882562	15006.56872	0.453464963	3.917939695
95 plus	0.365460143	2.736299108	1351.735485	0.036991823	2.736299108	0.327592007	3.052651238	4408.132852	0.134590974	3.052651238

Table 15: Kyrgyzstan 2100 life table, by age and sex. mx=mortality rate, ax=mean person-years lived in an age interval among those who die in that age interval, lx=number of persons left alive at age x, nLx=person-years lived between age x and x+n, ex=life expectancy at age x.										
Age Group	Male					Female				
	mx	ax	lx	nLx	ex	mx	ax	lx	nLx	ex
Early Neonatal	0.165681022	0.009583963	100000	0.019147656	79.70729152	0.129481063	0.009585073	100000	0.019154296	85.40930197
Late Neonatal	0.005922368	0.02876549	99682.91977	0.057342052	79.9402878	0.005439683	0.028765623	99752.08207	0.057382633	85.60109273
Post Neonatal	0.000858433	0.461582854	99648.97705	0.919682344	79.90982804	0.000824749	0.461585247	99720.88256	0.920360279	85.57024361
1 to 4	0.000118574	1.999841901	99570.06024	3.981858557	79.04915881	0.000113136	1.999849152	99645.00687	3.984899015	84.71144016
5 to 9	7.42498E-05	2.499845313	99522.87245	4.975220525	75.08550393	4.92677E-05	2.499897359	99599.94849	4.979384406	80.7487168
10 to 14	0.000138407	2.823756215	99485.95124	4.972761042	70.11226801	0.000107688	2.81915824	99575.42895	4.977594787	75.76786637
15 to 19	0.000251311	2.712899626	99417.16936	4.967992121	65.1585529	0.000206397	2.603777439	99521.85602	4.973629821	70.80687491
20 to 24	0.00038374	2.706444867	99292.39521	4.960225505	60.2363759	0.000206439	2.56203357	99419.25855	4.968462014	65.87684219
25 to 29	0.000603209	2.722454023	99102.16992	4.948302872	55.34600434	0.000266798	2.683487965	99316.74647	4.96276716	60.94183348
30 to 34	0.001020719	2.717682857	98803.93514	4.928690257	50.50353041	0.000437368	2.683249539	99184.44244	4.954187321	56.01900963
35 to 39	0.00164392	2.637726632	98301.59211	4.896105233	45.74537364	0.000634818	2.663480878	98967.96059	4.94106703	51.13486703
40 to 44	0.002138099	2.597588472	97498.36084	4.850118991	41.09730454	0.000932027	2.609927046	98654.57869	4.921796643	46.28782687
45 to 49	0.002730388	2.652600351	96464.94146	4.792497217	36.50535659	0.001154617	2.653688656	98196.39348	4.896554561	41.49021114
50 to 54	0.004015513	2.699746075	95164.22217	4.714662813	31.96240953	0.001773849	2.744255026	97632.2887	4.862040654	36.7122793
55 to 59	0.006543773	2.671660244	93282.10206	4.594402198	27.54693595	0.003111432	2.698040647	96772.10031	4.804146001	32.01147004
60 to 64	0.009648064	2.668713499	90299.12353	4.416245917	23.36024675	0.004708181	2.671149487	95281.14975	4.712488957	27.46677818
65 to 69	0.015210039	2.623625733	86078.65547	4.155293543	19.36459942	0.007186507	2.68506943	93069.88192	4.577604962	23.05073224
70 to 74	0.020972734	2.658669133	79830.98485	3.807263447	15.66429779	0.011675432	2.723959406	89796.61185	4.374098739	18.78603409
75 to 79	0.03671881	2.642255583	71985.30001	3.317404079	12.07397979	0.021229653	2.699519537	84732.8873	4.0410514	14.73706391
80 to 84	0.063016769	2.508559402	60046.59304	2.608373483	8.945681774	0.037969843	2.592092504	76246.2121	3.49875533	11.06827495
85 to 89	0.116691028	2.476568837	44229.66696	1.727102124	6.259396059	0.078975609	2.623094138	63227.00835	2.672894409	7.801012801
90 to 94	0.210249842	2.213139018	25012.98213	0.801298335	4.273971983	0.156543828	2.336101524	42712.63443	1.520763704	5.317452203
95 plus	0.308919797	3.251803921	8838.646589	0.295301209	3.251803921	0.256066343	3.929432674	19611.77134	0.788217851	3.929432674

Table 15: Mongolia 2017 life table, by age and sex. mx=mortality rate, ax=mean person-years lived in an age interval among those who die in that age interval, lx=number of persons left alive at age x, nLx=person-years lived between age x and x+n, ex=life expectancy at age x.

Age Group	Male					Female				
	mx	ax	lx	nLx	ex	mx	ax	lx	nLx	ex
Early Neonatal	0.696111498	0.009567705	100000	0.019050646	64.68965897	0.535251429	0.009572636	100000	0.019079987	73.74183485
Late Neonatal	0.069517069	0.028747947	98674.02916	0.056657974	65.53959313	0.025357161	0.028760129	98978.7651	0.056905169	74.48335246
Post Neonatal	0.009632542	0.460959557	98280.14771	0.903385609	65.74451498	0.007328015	0.461123266	98834.47764	0.909446683	74.53451253
1 to 4	0.001014857	1.998646858	97410.03743	3.888507255	65.40430205	0.001121538	1.998504617	98168.08101	3.91793023	74.11407065
5 to 9	0.000559671	2.498834018	97015.61285	4.844001324	61.66214067	0.00033964	2.499292416	97728.77977	4.882292696	70.43823646
10 to 14	0.000583258	2.619629861	96744.56723	4.830522186	56.82791995	0.000332737	2.611586569	97562.97516	4.874275504	65.5537049
15 to 19	0.000898703	2.72445832	96462.86174	4.813301098	51.98618335	0.000519659	2.623956952	97400.799	4.864034849	60.65847761
20 to 24	0.001563831	2.679114628	96030.31434	4.784152096	47.20788389	0.000645016	2.577950964	97148.04215	4.84982604	55.80938865
25 to 29	0.002269561	2.658828646	95282.1949	4.73893131	42.55718243	0.000765548	2.5964061	96835.23249	4.832869417	50.98123405
30 to 34	0.003355038	2.649770627	94206.75244	4.673492307	38.01223341	0.001006023	2.69126556	96465.27204	4.812088254	46.16666277
35 to 39	0.004810897	2.653306626	92638.97245	4.580250717	33.61010496	0.001704409	2.708716218	95981.19637	4.780392116	41.3857544
40 to 44	0.007174245	2.659117418	90435.92154	4.447134564	29.36337984	0.002749795	2.685861388	95166.49489	4.728238885	36.71654532
45 to 49	0.010911053	2.648927864	87246.52531	4.253270719	25.3384631	0.004246633	2.700922227	93866.49895	4.647951155	32.18741425
50 to 54	0.016365728	2.627560003	82608.24589	3.976141197	21.61106169	0.00708609	2.660822678	91893.05882	4.519752833	27.82008514
55 to 59	0.023965161	2.593210128	76106.15917	3.597989149	18.23152315	0.010265577	2.657725193	88691.20146	4.330471784	23.72759494
60 to 64	0.033164883	2.581778393	67492.96574	3.124409811	15.22624622	0.016180936	2.659367575	84247.5248	4.058739748	19.83805135
65 to 69	0.049871859	2.53292408	57145.58646	2.544758704	12.51567061	0.025882044	2.618126515	77683.92599	3.658829412	16.28850088
70 to 74	0.064773288	2.463360089	44478.31038	1.910773755	10.36026309	0.038267487	2.560763584	68222.58002	3.120267839	13.18344859
75 to 79	0.078636109	2.453042562	32133.51515	1.339351267	8.398525659	0.051879035	2.548349157	56298.93571	2.497886918	10.43323639
80 to 84	0.121270736	2.444733036	21636.7431	0.826623801	6.290651343	0.083064188	2.532778894	43367.21564	1.800372356	7.786381301
85 to 89	0.186950274	2.285632521	11646.43877	0.386734225	4.601612661	0.142786005	2.405770655	28451.66672	1.038883274	5.545716785
90 to 94	0.280259305	2.083242237	4438.090188	0.122242196	3.378814066	0.230996749	2.205170214	13657.80873	0.415419547	3.956639576
95 plus	0.365096936	2.739251045	1019.817141	0.027977062	2.739251045	0.325100421	3.076619288	4084.325255	0.125880153	3.076619288

Table 15: Mongolia 2100 life table, by age and sex. mx=mortality rate, ax=mean person-years lived in an age interval among those who die in that age interval, lx=number of persons left alive at age x, nLx=person-years lived between age x and x+n, ex=life expectancy at age x.

Age Group	Male					Female				
	mx	ax	lx	nLx	ex	mx	ax	lx	nLx	ex
Early Neonatal	0.131975406	0.009584996	100000	0.019153836	75.72793353	0.116810145	0.009585461	100000	0.019156619	84.8954849
Late Neonatal	0.014331795	0.02876317	99747.26707	0.057365192	75.90026637	0.007228616	0.028765129	99776.26284	0.057393588	85.06595411
Post Neonatal	0.001792051	0.461516531	99665.08056	0.919434936	75.90520584	0.001454918	0.461540481	99734.78536	0.920220962	85.04360874
1 to 4	0.000133658	1.999821789	99500.42139	3.978954537	75.10645853	0.000162341	1.999783545	99600.95486	3.982746115	84.23336471
5 to 9	0.000109184	2.499772534	99447.31639	4.971010754	71.14528694	8.1789E-05	2.499829605	99536.36519	4.975802621	80.28639978
10 to 14	0.000213542	2.944361606	99393.12658	4.967327959	66.18232088	0.000135433	2.844372432	99495.74885	4.973247009	75.31762793
15 to 19	0.000428123	2.732880805	99287.2441	4.959567099	61.24946683	0.000221529	2.589018541	99428.57588	4.968749142	70.36578368
20 to 24	0.000719209	2.709031775	99075.28002	4.945526663	56.37323341	0.000201658	2.552097022	99318.74978	4.963456961	65.439797
25 to 29	0.001058634	2.687514009	98720.53465	4.924021263	51.56327705	0.000236	2.643440825	99218.90569	4.958174416	60.50238011
30 to 34	0.001721858	2.690023832	98201.0432	4.890488135	46.81799622	0.000345537	2.791093999	99102.21347	4.951231603	55.5696587
35 to 39	0.002452749	2.672782747	97364.27215	4.840541877	42.19138872	0.000614536	2.723055234	98931.62881	4.939622599	50.65933421
40 to 44	0.003589548	2.673190938	96185.98927	4.769582894	37.6688009	0.000956403	2.717928991	98628.67648	4.920714799	45.80451043
45 to 49	0.005389007	2.699836454	94488.58189	4.666756243	33.28642422	0.001639521	2.743189001	98159.03772	4.889699455	41.0079322
50 to 54	0.008785652	2.682772426	91997.73191	4.508647595	29.0945533	0.002702971	2.709912304	97360.37151	4.837969783	36.31600347
55 to 59	0.013706243	2.590625815	88077.66268	4.264630694	25.24674282	0.004247545	2.693501497	96058.38965	4.75620286	31.76440997
60 to 64	0.016310684	2.589782494	82307.78681	3.962455834	21.8094944	0.006507702	2.677175194	94049.97175	4.63298525	27.37405922
65 to 69	0.024524048	2.559171149	75963.09232	3.590005093	18.39000414	0.010609329	2.624814181	91052.63002	4.442607988	23.17034677
70 to 74	0.029568226	2.515563237	67435.11511	3.152721943	15.36726432	0.014616364	2.614753608	86413.33292	4.178911749	19.24010675
75 to 79	0.036233981	2.595835483	58628.23726	2.711416541	12.30868805	0.020079481	2.663672147	80516.55342	3.851427589	15.42574185
80 to 84	0.060150166	2.609773	49542.23675	2.18300935	9.149769989	0.034798851	2.7223981	73114.79358	3.400170019	11.70422605
85 to 89	0.113056117	2.487764844	37234.07033	1.471848467	6.379715935	0.072806507	2.654042932	62026.05598	2.678646286	8.283010783
90 to 94	0.206183857	2.217791633	21672.23409	0.70297585	4.340958415	0.146698733	2.329818905	44103.13848	1.622384451	5.642556476
95 plus	0.305534086	3.290181498	7938.558159	0.270490774	3.290181498	0.246274785	4.137366125	22233.54475	0.973963034	4.137366125

Table 15: Tajikistan 2017 life table, by age and sex. mx=mortality rate, ax=mean person-years lived in an age interval among those who die in that age interval, lx=number of persons left alive at age x, nLx=person-years lived between age x and x+n, ex=life expectancy at age x.

Age Group	Male					Female				
	mx	ax	lx	nLx	ex	mx	ax	lx	nLx	ex
Early Neonatal	0.881807306	0.009562014	100000	0.019016841	68.2033597	0.622692917	0.009569956	100000	0.019064028	73.93190895
Late Neonatal	0.066638448	0.028748741	98323.27094	0.056461275	69.34669819	0.071282207	0.02874746	98812.96683	0.056734893	74.80052501
Post Neonatal	0.018724672	0.460313673	97947.11861	0.896564115	69.55535128	0.014327557	0.460626033	98408.60749	0.902612524	75.05019585
1 to 4	0.002310896	1.99691881	96268.99792	3.833026713	69.83675994	0.001920081	1.997439895	97115.67803	3.86975224	75.12001846
5 to 9	0.000716527	2.498507235	95383.71821	4.760656101	66.46674469	0.000448523	2.499065576	96372.89351	4.813246465	71.68375906
10 to 14	0.000565613	2.591369588	95042.73086	4.745669482	61.69640665	0.000383575	2.552890718	96157.04601	4.803344306	66.83908005
15 to 19	0.000966673	2.681859596	94774.37742	4.728124295	56.86384324	0.000548267	2.656567466	95972.81834	4.792484398	61.96245376
20 to 24	0.001420221	2.586176887	94317.33792	4.699756951	52.12637427	0.000799831	2.605138942	95710.07635	4.776355641	57.12517741
25 to 29	0.001572506	2.554960338	93649.90705	4.664563361	47.47937216	0.000958211	2.618360037	95328.07417	4.755552206	52.34351656
30 to 34	0.001862544	2.598052504	92916.47067	4.62513601	42.83384113	0.00135577	2.625081882	94872.43083	4.728398991	47.58217536
35 to 39	0.002489969	2.634269236	92055.1593	4.575810834	38.21019373	0.001792405	2.633453513	94231.42952	4.691672465	42.88775616
40 to 44	0.003541921	2.669173702	90915.99753	4.508587819	33.65572301	0.002541335	2.66187176	93390.59475	4.641950426	38.24986238
45 to 49	0.005527886	2.685646583	89319.36789	4.409572018	29.20911593	0.003845391	2.6487098	92211.10152	4.569248506	33.70467071
50 to 54	0.008859468	2.675374639	86882.4383	4.256487105	24.95230588	0.00544831	2.670727155	90454.41762	4.466057969	29.30726234
55 to 59	0.013938649	2.665483827	83112.91427	4.024741623	20.96144836	0.008693505	2.6834052	88021.87115	4.314239966	25.04274558
60 to 64	0.022333518	2.631527496	77506.40519	3.680768731	17.28371349	0.014059421	2.65097846	84272.82742	4.079003237	21.03661233
65 to 69	0.033689469	2.597727829	69292.73557	3.20551344	14.01950888	0.021131132	2.648921784	78541.48014	3.741354833	17.37727494
70 to 74	0.051088172	2.555426994	58506.92481	2.601070877	11.12449327	0.034962198	2.585711888	70642.57358	3.257529979	14.02332465
75 to 79	0.075232424	2.507101132	45242.72862	1.905557643	8.637989245	0.047442777	2.538146551	59269.55109	2.654056051	11.2180693
80 to 84	0.115482048	2.435017335	30938.73802	1.194188793	6.476261384	0.068617936	2.516106322	46701.75064	1.995686542	8.555834068
85 to 89	0.180346571	2.301434877	17184.4731	0.5784727	4.718581164	0.123764068	2.46174401	33037.44448	1.257693195	6.057728177
90 to 94	0.274146171	2.096263002	6777.851138	0.188876662	3.441656253	0.21048599	2.250510174	17505.34572	0.554835049	4.255359885
95 plus	0.36034994	2.775364926	1609.833577	0.044736315	2.775364926	0.306616448	3.261972502	5848.445213	0.191035042	3.261972502

Table 15: Tajikistan 2100 life table, by age and sex. mx=mortality rate, ax=mean person-years lived in an age interval among those who die in that age interval, lx=number of persons left alive at age x, nLx=person-years lived between age x and x+n, ex=life expectancy at age x.

Age Group	Male					Female				
	mx	ax	lx	nLx	ex	mx	ax	lx	nLx	ex
Early Neonatal	0.181098552	0.00958349	100000	0.019144821	79.97943185	0.116351672	0.009585475	100000	0.019156703	85.39621684
Late Neonatal	0.011601525	0.028763923	99653.35854	0.057315685	80.23682809	0.017001504	0.028762433	99777.14162	0.057377969	85.56649523
Post Neonatal	0.002628216	0.461457132	99586.88903	0.918359171	80.23244915	0.002163758	0.461490126	99679.62554	0.919411367	85.59212245
1 to 4	0.000238758	1.999681656	99345.6369	3.971930031	79.50235731	0.00020076	1.99973232	99480.78617	3.977635874	84.83842404
5 to 9	0.000194198	2.499595422	99250.88732	4.960137887	75.57583149	0.00013361	2.499721644	99401.02946	4.968395396	80.90450054
10 to 14	0.00022426	2.781832691	99154.64862	4.955265764	70.64607585	0.000166147	2.729986907	99334.80374	4.96481994	75.95588579
15 to 19	0.000495073	2.714302556	99043.63784	4.946578703	65.72150358	0.000267349	2.656341615	99252.48471	4.959502048	71.01599103
20 to 24	0.00072092	2.591093874	98799.26981	4.931417985	60.8754498	0.000347391	2.605747723	99120.1493	4.951887155	66.10655109
25 to 29	0.000796618	2.56167959	98445.37322	4.912740751	56.08251775	0.000435689	2.64148133	98948.45547	4.942335598	61.21593487
30 to 34	0.000928892	2.58075764	98056.08891	4.891841776	51.29315554	0.000627654	2.645176939	98733.5427	4.929374658	56.34239911
35 to 39	0.001135962	2.628235852	97604.17769	4.867115364	46.51701646	0.000847219	2.639029744	98424.73964	4.911424224	51.50962482
40 to 44	0.001597612	2.652580353	97054.04017	4.834577689	41.76339789	0.001195595	2.663697425	98009.37895	4.886866757	46.71520298
45 to 49	0.002255952	2.696631644	96285.36835	4.789444705	37.07266733	0.001826172	2.653583162	97426.37156	4.85040262	41.97664975
50 to 54	0.0037297	2.717223067	95210.84254	4.720283679	32.45481529	0.002365547	2.6425096	96543.97342	4.800450181	37.33254337
55 to 59	0.005959092	2.686452013	93463.24291	4.609653936	28.00232495	0.003459018	2.645576304	95411.84811	4.732180932	32.74067594
60 to 64	0.008927124	2.673987231	90741.57667	4.445373669	23.75173456	0.004866936	2.640915623	93783.22153	4.636199004	28.25733707
65 to 69	0.013985127	2.646353713	86817.92268	4.204008661	19.69280979	0.006872789	2.674808733	91545.08449	4.505739506	23.87531934
70 to 74	0.020196518	2.655446767	81030.88744	3.871282051	15.89884269	0.011027542	2.689343575	88480.60808	4.315194115	19.60219115
75 to 79	0.033936686	2.668511677	73368.79752	3.40615591	12.27473903	0.017802837	2.704132553	83795.81916	4.02738299	15.54133753
80 to 84	0.062458829	2.580180423	62162.59712	2.714750231	9.008993257	0.03198931	2.681086342	76755.2943	3.577426637	11.71720507
85 to 89	0.116063846	2.478982275	45922.40601	1.798010579	6.290992538	0.069549854	2.661003144	65540.03174	2.827724104	8.26332451
90 to 94	0.209413434	2.213431532	26142.49293	0.840306377	4.291842271	0.144202876	2.347827881	46355.50088	1.688244543	5.609369104
95 plus	0.308187247	3.262027506	9332.207584	0.313736426	3.262027506	0.244263152	4.114174377	22609.54314	0.94708452	4.114174377

Table 15: Turkmenistan 2017 life table, by age and sex. mx=mortality rate, ax=mean person-years lived in an age interval among those who die in that age interval, lx=number of persons left alive at age x, nLx=person-years lived between age x and x+n, ex=life expectancy at age x.

Age Group	Male					Female				
	mx	ax	lx	nLx	ex	mx	ax	lx	nLx	ex
Early Neonatal	0.65667247	0.009568914	100000	0.019057837	66.79514509	0.450568785	0.009575231	100000	0.019095465	73.98655051
Late Neonatal	0.057389263	0.028751293	98748.69433	0.056720654	67.62188747	0.048699936	0.028753689	99139.68071	0.056959448	74.60919329
Post Neonatal	0.009019735	0.461003089	98423.28576	0.904958292	67.78781258	0.007015464	0.461145469	98862.34277	0.909834738	74.76087474
1 to 4	0.001216404	1.998378128	97607.4137	3.894819207	67.42739841	0.001149584	1.998467222	98224.22155	3.919952609	74.32035546
5 to 9	0.00040988	2.499146083	97133.93956	4.851725319	63.74648671	0.000297062	2.49938112	97773.76066	4.885059804	70.65367771
10 to 14	0.000468827	2.762276538	96935.14145	4.841677812	58.87213075	0.000277739	2.675954404	97628.66751	4.878284786	65.75498329
15 to 19	0.00100351	2.75439551	96708.19441	4.824538773	54.00379938	0.000533016	2.679862363	97493.19	4.868639095	60.84262919
20 to 24	0.001707754	2.640153406	96224.077	4.791892814	49.26151821	0.000741173	2.62247251	97233.68964	4.853133064	55.99779645
25 to 29	0.002180013	2.593044417	95405.7758	4.745390092	44.66117724	0.000974871	2.597009697	96874.00221	4.832380637	51.19588991
30 to 34	0.002730805	2.627280949	94371.34105	4.688193445	40.12205779	0.001205054	2.639673889	96402.93314	4.806477226	46.43325
35 to 39	0.00393995	2.650042871	93091.19778	4.611865945	35.63737921	0.00180222	2.64864371	95823.76426	4.770972634	41.6977931
40 to 44	0.005749966	2.668854778	91274.37553	4.503366639	31.29357237	0.002527493	2.659329807	94963.99541	4.720276331	37.05107594
45 to 49	0.009020854	2.617789964	88685.45816	4.341011738	27.12863194	0.003812366	2.682826583	93771.07846	4.647501676	32.48825898
50 to 54	0.011642487	2.648464337	84770.65338	4.125623924	23.25986	0.006054507	2.69364739	91999.55697	4.536639151	28.06176632
55 to 59	0.019183787	2.631205192	79969.44035	3.824754963	19.49666292	0.009912145	2.665716715	89253.46055	4.361776669	23.84176203
60 to 64	0.027661403	2.603513103	72636.27663	3.406174988	16.19871394	0.015078195	2.646375637	84931.35652	4.101083682	19.91877053
65 to 69	0.041891282	2.567003952	63221.28529	2.868940582	13.22301738	0.023225429	2.630792263	78750.26389	3.732266666	16.27383808
70 to 74	0.059612518	2.504600691	51214.87044	2.229535652	10.7214816	0.036049461	2.600107776	70087.43724	3.225573714	12.95948436
75 to 79	0.077713191	2.465145736	37942.26842	1.585389461	8.597630946	0.055332909	2.569782203	58470.75843	2.577460604	10.01737544
80 to 84	0.113442595	2.404158291	25644.40701	0.991011899	6.542425598	0.091087459	2.49792187	44230.89706	1.801763575	7.416150629
85 to 89	0.177629296	2.307998141	14424.96897	0.488252271	4.767565356	0.152565031	2.379149119	27852.99399	0.99553247	5.312211967
90 to 94	0.271621345	2.101628724	5767.973725	0.161478176	3.467951295	0.241238795	2.181741688	12697.96814	0.378288518	3.821125591
95 plus	0.35838535	2.7904681	1387.907234	0.038764608	2.7904681	0.33419071	2.992829465	3589.959176	0.107597944	2.992829465

Table 15: Turkmenistan 2100 life table, by age and sex. mx=mortality rate, ax=mean person-years lived in an age interval among those who die in that age interval, lx=number of persons left alive at age x, nLx=person-years lived between age x and x+n, ex=life expectancy at age x.

Age Group	Male					Female				
	mx	ax	lx	nLx	ex	mx	ax	lx	nLx	ex
Early Neonatal	0.117581415	0.009585437	100000	0.01915648	80.73859353	0.082447688	0.009586514	100000	0.01916293	85.59043664
Late Neonatal	0.009799825	0.02876442	99774.82588	0.057388518	80.90128515	0.010343208	0.02876427	99842.04152	0.057426281	85.70636985
Post Neonatal	0.001474916	0.46153906	99718.6016	0.920063103	80.88923777	0.001216503	0.461557417	99782.6558	0.92076384	85.6997158
1 to 4	0.000122702	1.999836397	99582.93903	3.982340632	80.07539388	0.000125553	1.999832595	99670.6629	3.985825826	84.87204466
5 to 9	7.52314E-05	2.499843268	99534.09758	4.975769279	76.11367656	7.0541E-05	2.499853039	99620.63333	4.980153454	80.91360733
10 to 14	0.000144593	3.125301938	99496.67651	4.973435609	71.14122587	9.93802E-05	2.829233641	99585.50698	4.978193976	75.94122719
15 to 19	0.000470813	2.794376312	99424.78699	4.966091069	66.19029502	0.000220434	2.685792624	99536.04369	4.974261573	70.97748084
20 to 24	0.000833789	2.691854666	99191.10925	4.949917672	61.33816562	0.000292961	2.637757203	99426.41686	4.967864178	66.05259872
25 to 29	0.001123362	2.599239727	98779.20361	4.925681276	56.5796242	0.000396065	2.616893587	99280.92544	4.959369963	61.14527862
30 to 34	0.00134625	2.602022945	98227.59475	4.8955288	51.8782956	0.000519315	2.654757944	99084.60101	4.948166996	56.2605706
35 to 39	0.001698423	2.598380883	97571.34397	4.858867217	47.20601967	0.000745147	2.644711935	98827.84486	4.932726361	51.39880675
40 to 44	0.002203304	2.66389672	96750.6841	4.81299291	42.57823505	0.001025362	2.701505967	98460.7129	4.911418471	46.57874235
45 to 49	0.003566742	2.625451296	95698.51421	4.744932084	38.00747232	0.001692952	2.684448443	97957.99674	4.878668519	41.80169446
50 to 54	0.00414451	2.694329121	94027.9716	4.657096878	33.61678699	0.002396569	2.677733957	97134.51439	4.829753899	37.1297269
55 to 59	0.007327098	2.664411776	92125.28094	4.529338015	29.23925176	0.003628922	2.644154808	95981.04278	4.758378122	32.53925848
60 to 64	0.009427949	2.626406771	88873.08732	4.347926919	25.18880984	0.004866849	2.635734688	94262.37477	4.659715218	28.07769592
65 to 69	0.013945529	2.624304194	84852.9598	4.110077864	21.23847636	0.006981953	2.65278266	92008.41514	4.526546528	23.69226513
70 to 74	0.019260851	2.594918207	79302.78278	3.795848215	17.51999132	0.010223516	2.68605922	88875.92132	4.342063178	19.42393641
75 to 79	0.025974435	2.627176085	72294.76763	3.414299484	13.95823982	0.017320672	2.754489984	84491.66556	4.068535731	15.28112404
80 to 84	0.043894848	2.54153341	63879.22972	2.902833084	10.45771105	0.035640902	2.682811418	77579.88883	3.590628987	11.3830689
85 to 89	0.089072943	2.564017536	52045.31513	2.169329078	7.274780512	0.075153173	2.639873219	65157.13475	2.782992616	8.025473084
90 to 94	0.17801732	2.238022007	34280.1367	1.17628535	4.849850607	0.151160562	2.338186111	45087.84239	1.627268499	5.462826311
95 plus	0.281820486	3.581956874	14773.75887	0.553393996	3.581956874	0.250838249	4.02183815	21502.15517	0.890551496	4.02183815

Table 15: Uzbekistan 2017 life table, by age and sex. mx=mortality rate, ax=mean person-years lived in an age interval among those who die in that age interval, lx=number of persons left alive at age x, nLx=person-years lived between age x and x+n, ex=life expectancy at age x.

Age Group	Male					Female				
	mx	ax	lx	nLx	ex	mx	ax	lx	nLx	ex
Early Neonatal	0.499296032	0.009573738	100000	0.019086558	66.82656276	0.287943076	0.009580216	100000	0.019125228	73.23455299
Late Neonatal	0.063675696	0.028749558	99047.08069	0.05688175	67.45006949	0.060893317	0.028750326	99449.31873	0.057117311	73.62079394
Post Neonatal	0.007267149	0.46112759	98684.93997	0.908096611	67.6399371	0.007036835	0.461143951	99101.53949	0.912026704	73.82151129
1 to 4	0.001076923	1.998564104	98025.16256	3.912575691	67.1688501	0.00081297	1.99891604	98459.84304	3.931998084	73.37637559
5 to 9	0.00056708	2.498818583	97603.92804	4.873285746	63.45016776	0.000336607	2.499298735	98140.23591	4.90288518	69.60886451
10 to 14	0.000509388	2.614765072	97327.6332	4.860476665	58.62322648	0.000319352	2.605587873	97975.21772	4.895018757	64.72190497
15 to 19	0.00085165	2.664006568	97080.07897	4.844368205	53.76600025	0.000500651	2.701282564	97818.90249	4.885323941	59.82111953
20 to 24	0.001188847	2.637842906	96667.53107	4.819841371	48.98386057	0.000807318	2.631223423	97574.32329	4.869405104	54.96415459
25 to 29	0.001653572	2.631431959	96094.55902	4.785984389	44.25989149	0.001016013	2.588353903	97181.22568	4.847185711	50.17561014
30 to 34	0.002261956	2.630006318	95303.23395	4.739757585	39.60515786	0.001249361	2.598679545	96688.78428	4.819982019	45.41767827
35 to 39	0.003124333	2.632557574	94231.2707	4.676978869	35.02528242	0.001626764	2.63661185	96086.66508	4.785936257	40.68564593
40 to 44	0.004362487	2.643846362	92770.36067	4.591341889	30.53468847	0.002348118	2.660521809	95308.24703	4.739380526	35.99590568
45 to 49	0.006360883	2.679041284	90768.11689	4.472409621	26.14902964	0.003498631	2.701411221	94195.6699	4.672217714	31.38901487
50 to 54	0.01035717	2.692640333	87924.83068	4.293700566	21.90686627	0.005893949	2.686701809	92561.60245	4.565851311	26.89467542
55 to 59	0.017324525	2.690732281	83481.33981	4.013649543	17.92818753	0.009190977	2.703971558	89871.79077	4.400773324	22.61812731
60 to 64	0.030267509	2.623118231	76535.82791	3.570315347	14.30947545	0.016070606	2.672099457	85829.81278	4.136864805	18.55458683
65 to 69	0.044982062	2.581816281	65746.56898	2.965483652	11.22615683	0.025531872	2.648229685	79188.04826	3.735428118	14.88500466
70 to 74	0.072018147	2.547070877	52438.45341	2.229410845	8.420364675	0.042483932	2.615548422	69665.29652	3.163537626	11.55598499
75 to 79	0.121924978	2.471377258	36437.31011	1.394182142	6.003238192	0.07088031	2.556987471	56258.17261	2.399042918	8.6859495
80 to 84	0.226616547	2.317433556	19521.15123	0.60857069	4.075225405	0.120264395	2.451220139	39318.9901	1.50667099	6.329865586
85 to 89	0.292395428	2.07874863	5814.115652	0.157311073	3.254777505	0.186680329	2.296749567	21297.21788	0.709232566	4.626149128
90 to 94	0.373238906	1.891378914	1243.877704	0.028895178	2.628573656	0.275746302	2.101832575	8134.195654	0.226681832	3.422934687
95 plus	0.434930443	2.299710013	171.4487122	0.00395877	2.299710013	0.36411283	2.747502993	1916.509126	0.052866962	2.747502993

Table 15: Uzbekistan 2100 life table, by age and sex. mx=mortality rate, ax=mean person-years lived in an age interval among those who die in that age interval, lx=number of persons left alive at age x, nLx=person-years lived between age x and x+n, ex=life expectancy at age x.

Age Group	Male					Female				
	mx	ax	lx	nLx	ex	mx	ax	lx	nLx	ex
Early Neonatal	0.121330451	0.009585322	100000	0.019155789	76.73970713	0.069875789	0.009586899	100000	0.019165239	84.09433513
Late Neonatal	0.018948545	0.028761896	99767.61681	0.05736928	76.89856286	0.025536506	0.028760079	99866.09619	0.057415031	84.1872312
Post Neonatal	0.001574311	0.461531999	99658.95332	0.919470712	76.92449533	0.002037405	0.461499102	99719.53766	0.919833245	84.25260855
1 to 4	0.000164582	1.999780557	99514.276	3.979261745	76.11198826	0.000129237	1.999827685	99532.25894	3.980262353	83.48582306
5 to 9	0.000129077	2.49973109	99448.82	4.9708372	72.16066678	9.7891E-05	2.49979606	99480.86449	4.972826945	79.52761761
10 to 14	0.000173387	2.861260529	99384.6757	4.967375878	67.20545829	0.000140885	2.71567633	99432.21818	4.970008159	74.56488567
15 to 19	0.000418986	2.676444848	99298.57224	4.960104076	62.26108934	0.000239806	2.686929215	99362.24806	4.965347727	69.61491463
20 to 24	0.000537983	2.650607968	99090.83493	4.948283169	57.38506951	0.000344273	2.656252677	99243.28022	4.958157428	64.69406626
25 to 29	0.000812476	2.647771212	98824.7994	4.931800502	52.53109049	0.000490364	2.627640251	99072.74036	4.947875111	59.79960246
30 to 34	0.001109965	2.621652054	98424.52354	4.908253446	47.73194781	0.000633846	2.605199601	98830.45383	4.934007841	54.93770285
35 to 39	0.001449833	2.590674629	97880.5411	4.877016117	42.9802797	0.000778729	2.599117609	98518.19101	4.916748537	50.10138503
40 to 44	0.001764338	2.598617724	97174.85907	4.838490788	38.2706262	0.001029185	2.649580984	98135.87899	4.895102718	45.28387575
45 to 49	0.002572644	2.771554071	96323.55886	4.788141023	33.58203307	0.001661765	2.712511917	97633.15841	4.863118636	40.49994942
50 to 54	0.004361525	2.754146229	95104.56364	4.709023504	28.96760745	0.002607821	2.716285733	96829.99037	4.812492584	35.80599558
55 to 59	0.007875648	2.721670718	93063.47192	4.571185393	24.5351087	0.003799983	2.685830651	95588.10984	4.73756583	31.22336093
60 to 64	0.012941232	2.624082438	89495.81354	4.34235498	20.3924749	0.005563308	2.638679453	93805.70408	4.629842707	26.75469116
65 to 69	0.017417763	2.637083563	83927.21184	4.033061441	16.55634572	0.007559239	2.661047482	91257.99219	4.484616833	22.41453445
70 to 74	0.02831664	2.703591652	77038.94875	3.622213095	12.78034238	0.01220403	2.74500162	87915.44248	4.280316807	18.15051737
75 to 79	0.05658793	2.68101996	67097.59335	2.979583069	9.252891613	0.023543766	2.747928642	82832.86652	3.938941192	14.0707638
80 to 84	0.128349402	2.518438085	50998.19332	1.970614789	6.322719958	0.046723807	2.659379211	73917.9573	3.348578649	10.41595196
85 to 89	0.194003912	2.278571216	27670.61124	0.941054059	4.600387101	0.091669456	2.579241871	59153.49506	2.452157875	7.341482824
90 to 94	0.285609056	2.071662949	11269.01872	0.321383425	3.373532652	0.171479603	2.310465921	38302.04459	1.341877507	5.039650227
95 plus	0.368697286	2.734561505	2883.986339	0.083673652	2.734561505	0.269949314	3.755196449	16907.70164	0.668076168	3.755196449

Table 15: Central Europe 2017 life table, by age and sex. mx=mortality rate, ax=mean person-years lived in an age interval among those who die in that age interval, lx=number of persons left alive at age x, nLx=person-years lived between age x and x+n, ex=life expectancy at age x.

Age Group	Male					Female				
	mx	ax	lx	nLx	ex	mx	ax	lx	nLx	ex
Early Neonatal	0.124156137	0.009585236	100000	0.019155268	73.60855761	0.098869767	0.009586011	100000	0.019159912	80.46194562
Late Neonatal	0.018327558	0.028762068	99762.17634	0.057367166	73.76483082	0.015850746	0.028762751	99810.56687	0.057399081	80.59545994
Post Neonatal	0.001942723	0.461505828	99657.03696	0.919296428	73.78508865	0.001544411	0.461534123	99719.58543	0.920042525	80.61143276
1 to 4	0.000233471	1.999688705	99478.44459	3.97728035	72.9934378	0.000188149	1.999749134	99577.4939	3.981601297	79.80251534
5 to 9	0.00012409	2.49974148	99385.58737	4.967738106	69.05976853	0.000100755	2.499790093	99502.5804	4.973876055	75.86109124
10 to 14	0.000165709	2.936206911	99323.94325	4.964499339	64.10107828	0.000111084	2.694885059	99452.46601	4.971350338	70.89805834
15 to 19	0.00047183	2.767749236	99241.67736	4.956863115	59.15177997	0.000204886	2.623375939	99397.2424	4.967443305	65.93595044
20 to 24	0.00077482	2.61104788	99007.79798	4.941243622	54.28496716	0.000232774	2.585951398	99295.46663	4.96198507	61.00084258
25 to 29	0.000890026	2.582454325	98624.941	4.920659398	49.48555431	0.00030155	2.635047296	99179.96482	4.955464255	56.06886837
30 to 34	0.001135959	2.639167907	98186.99065	4.896218889	44.69475061	0.000429324	2.684490839	99030.53309	4.94660924	51.14949396
35 to 39	0.001664692	2.684090325	97630.80236	4.862792801	39.93432367	0.000684159	2.715115494	98818.16347	4.933196479	46.25364531
40 to 44	0.002642194	2.735788497	96821.30153	4.812275882	35.24574742	0.001141958	2.741575446	98480.65523	4.911366195	41.40285061
45 to 49	0.004757611	2.719653493	95549.81549	4.726216501	30.67833419	0.00202693	2.722838091	97919.8001	4.87349569	36.6242792
50 to 54	0.007950142	2.698384423	93301.29541	4.581237851	26.35208723	0.003363016	2.700562874	96931.98228	4.809408041	31.96974548
55 to 59	0.01314526	2.654101688	89659.21964	4.348856837	22.31288108	0.005406955	2.681898516	95314.58465	4.706736327	27.46639022
60 to 64	0.019630319	2.614218925	83942.71184	4.009369581	18.6515884	0.008440097	2.668715127	92769.71003	4.548980275	23.14624681
65 to 69	0.028164679	2.588850661	76072.53229	3.56176389	15.31071021	0.013104714	2.666559596	88930.41139	4.314588529	19.03025799
70 to 74	0.04038799	2.57935183	66041.555	3.008021415	12.24300467	0.021056787	2.68252538	83276.46198	3.970097114	15.14120162
75 to 79	0.062757876	2.551692181	53893.75585	2.335825985	9.421256802	0.037690851	2.668917301	74917.14999	3.443345545	11.53130576
80 to 84	0.100939246	2.479537281	39236.22977	1.563971941	6.987675703	0.07147905	2.588369038	61939.92939	2.641673525	8.388082118
85 to 89	0.164381305	2.34109283	23451.78512	0.815992116	5.022272574	0.128102875	2.448309914	43059.70843	1.622651568	5.931190141
90 to 94	0.259133677	2.12787749	10040.18552	0.28782061	3.604346364	0.215265464	2.240288976	22275.92617	0.698749337	4.181171506
95 plus	0.348594772	2.868677447	2582.55988	0.074090521	2.868677447	0.310966718	3.215825866	7236.187803	0.232725912	3.215825866

Table 15: Central Europe 2100 life table, by age and sex. mx=mortality rate, ax=mean person-years lived in an age interval among those who die in that age interval, lx=number of persons left alive at age x, nLx=person-years lived between age x and x+n, ex=life expectancy at age x.

Age Group	Male					Female				
	mx	ax	lx	nLx	ex	mx	ax	lx	nLx	ex
Early Neonatal	0.028538443	0.009588166	100000	0.019172835	81.99899103	0.02494399	0.009588277	100000	0.019173496	86.2929979
Late Neonatal	0.004601599	0.028765854	99945.28804	0.057495158	82.02468763	0.004922553	0.028765765	99952.17653	0.05749859	86.31509089
Post Neonatal	0.000358414	0.461618374	99918.83349	0.922385657	81.98886547	0.000282779	0.461623747	99923.87501	0.9224644	86.28198861
1 to 4	4.65862E-05	1.999937886	99885.77715	3.995058881	81.09257209	3.89004E-05	1.999948133	99897.79189	3.995600837	85.38111476
5 to 9	3.4447E-05	2.499928236	99867.1676	4.992928396	77.10731417	3.65994E-05	2.499923752	99882.25045	4.993655613	81.39409311
10 to 14	6.40428E-05	3.302801421	99849.96886	4.991922091	72.12014882	5.54183E-05	2.856568198	99863.9748	4.992588645	76.40852261
15 to 19	0.000250139	2.830941619	99818.00158	4.988181705	67.14219393	0.000115201	2.632655831	99836.3091	4.990454367	71.42891659
20 to 24	0.000452962	2.61544375	99693.24622	4.979270247	62.22235651	0.000128776	2.5781355	99778.82465	4.987381571	66.46848075
25 to 29	0.000489033	2.546110089	99467.79345	4.967421129	57.35694477	0.000159366	2.614092646	99714.60992	4.983829862	61.509556
30 to 34	0.00055413	2.572073349	99225.0119	4.954589356	52.49056426	0.000210542	2.648126581	99635.20229	4.979286903	56.55636422
35 to 39	0.000685051	2.646401292	98950.67949	4.93957394	47.62845062	0.000301776	2.698015284	99530.39242	4.973061553	51.61291406
40 to 44	0.001039984	2.738635228	98612.6773	4.919046063	42.78195739	0.000494665	2.795037087	99380.36009	4.963583498	46.6863423
45 to 49	0.001864198	2.769176546	98101.85175	4.88473998	37.98934295	0.000985084	2.781782656	99134.93411	4.945915957	41.79420982
50 to 54	0.003436157	2.747062203	97192.96925	4.822300109	33.31640906	0.001814628	2.739779346	98648.02053	4.912230368	36.98516696
55 to 59	0.006020029	2.667330758	95539.85941	4.710914115	28.84091253	0.003078765	2.690198362	97757.54798	4.853354456	32.29421609
60 to 64	0.008535727	2.638432693	92713.11666	4.544334887	24.63173275	0.004677727	2.652641655	96265.82035	4.761086561	27.74876343
65 to 69	0.012478825	2.634235272	88850.40081	4.315766483	20.58007507	0.006708883	2.650060801	94044.28018	4.629443503	23.33627942
70 to 74	0.018322073	2.647699518	83497.42874	4.00365236	16.72180689	0.010008691	2.713262686	90950.23037	4.446246247	19.03365785
75 to 79	0.0297003	2.648635019	76225.99406	3.565421599	13.0562402	0.018399679	2.759547096	86526.80431	4.156361634	14.8594242
80 to 84	0.051105749	2.600735574	65779.37385	2.937453453	9.703115049	0.038582911	2.695007785	78952.38706	3.63035743	11.00805573
85 to 89	0.100978955	2.523827159	51134.58089	2.057956678	6.739221115	0.080166822	2.618466434	65213.3115	2.749523864	7.744666189
90 to 94	0.193207257	2.236716451	30991.45467	1.020240824	4.53800615	0.158077394	2.334615915	43788.23029	1.554236042	5.281733189
95 plus	0.294840925	3.402984506	11808.90423	0.409165081	3.402984506	0.257525733	3.906833207	19935.04763	0.795662506	3.906833207

Table 15: Albania 2017 life table, by age and sex. mx=mortality rate, ax=mean person-years lived in an age interval among those who die in that age interval, lx=number of persons left alive at age x, nLx=person-years lived between age x and x+n, ex=life expectancy at age x.

Age Group	Male					Female				
	mx	ax	lx	nLx	ex	mx	ax	lx	nLx	ex
Early Neonatal	0.253250297	0.009581279	100000	0.019131589	75.08709679	0.302883059	0.009579758	100000	0.019122494	82.21173139
Late Neonatal	0.015753335	0.028762778	99515.54984	0.057229586	75.43340475	0.042662319	0.028755355	99420.8731	0.057130915	82.67142808
Post Neonatal	0.005897346	0.461224898	99425.40478	0.915488413	75.44423009	0.002449856	0.461469802	99177.17272	0.914656017	82.8170089
1 to 4	0.000779591	1.998960545	98885.65446	3.949267649	74.93030972	0.000250579	1.999665895	98953.15074	3.956143558	82.080249
5 to 9	0.000381677	2.49920484	98577.89238	4.924195581	71.15812617	0.000237966	2.499504238	98854.04408	4.939763221	78.16056996
10 to 14	0.000330522	2.640069381	98389.99109	4.915667176	66.28929331	0.000173312	2.497059972	98736.50817	4.934686175	73.25066236
15 to 19	0.000607162	2.74551747	98227.53931	4.904666355	61.39448473	0.000237164	2.618649273	98650.99085	4.929766561	68.31194809
20 to 24	0.001053935	2.6238469	97929.75854	4.884255889	56.5724126	0.000310039	2.597169192	98534.07894	4.923037358	63.38975914
25 to 29	0.001242257	2.541741281	97415.03052	4.855923873	51.85678996	0.000383582	2.575726755	98381.44964	4.914503108	58.48388074
30 to 34	0.001316678	2.515206914	96811.89146	4.824814894	47.16331218	0.000451345	2.62544646	98192.94902	4.904393102	53.5909488
35 to 39	0.001357379	2.579713216	96176.76802	4.793100435	42.45743218	0.000659377	2.658989585	97971.61197	4.891031931	48.70579761
40 to 44	0.001862808	2.640226751	95526.39348	4.755427813	37.72814882	0.000959779	2.684036741	97649.15414	4.871630468	43.85737416
45 to 49	0.002653365	2.682346577	94640.99935	4.703146293	33.05535435	0.00151855	2.662924756	97181.68357	4.841904496	39.05478987
50 to 54	0.004277105	2.693267994	93393.98865	4.624114472	28.4594874	0.002171905	2.67282692	96446.6534	4.798091965	34.33116557
55 to 59	0.00684256	2.7233118	91418.23003	4.500871404	24.01437203	0.003379673	2.694441223	95405.06823	4.733394082	29.67553469
60 to 64	0.012268698	2.704491732	88342.89722	4.296357261	19.75297779	0.005470514	2.712752114	93806.55459	4.632426368	25.13349938
65 to 69	0.020810198	2.684280236	83082.39178	3.963635877	15.82909018	0.009373659	2.726454566	91275.28392	4.468694321	20.75277491
70 to 74	0.036446338	2.647352315	74859.04173	3.448567425	12.26945455	0.016917276	2.712437472	87094.17559	4.192899754	16.61455082
75 to 79	0.063366373	2.58451737	62350.51757	2.706264387	9.197764458	0.030579165	2.673361661	80023.14513	3.736560677	12.83832656
80 to 84	0.1096984	2.497945784	45329.51518	1.782292806	6.686512454	0.054480429	2.63332533	68658.0035	3.043443664	9.51776433
85 to 89	0.174565026	2.316013801	25974.47825	0.887611179	4.831763724	0.104533198	2.525598909	52211.63175	2.078356079	6.690924357
90 to 94	0.268673548	2.107722755	10641.49343	0.300702328	3.502233386	0.188536477	2.2936224	30698.3581	1.019800939	4.631207398
95 plus	0.356050092	2.810085239	2630.902209	0.074364108	2.810085239	0.286366393	3.496744083	11650.70769	0.410294134	3.496744083

Table 15: Albania 2100 life table, by age and sex. mx=mortality rate, ax=mean person-years lived in an age interval among those who die in that age interval, lx=number of persons left alive at age x, nLx=person-years lived between age x and x+n, ex=life expectancy at age x.

Age Group	Male					Female				
	mx	ax	lx	nLx	ex	mx	ax	lx	nLx	ex
Early Neonatal	0.078305817	0.009586641	100000	0.019163696	82.17443525	0.119767292	0.00958537	100000	0.019156091	87.81931835
Late Neonatal	0.005739637	0.02876554	99850.04235	0.057438493	82.27835186	0.023223552	0.028760717	99770.8441	0.057364146	88.00042708
Post Neonatal	0.001363637	0.461546965	99817.10372	0.921019658	82.2479148	0.000323307	0.461620868	99637.93139	0.919807659	88.05946472
1 to 4	0.000130583	1.999825889	99691.65437	3.986625571	81.42733728	3.24815E-05	1.99995669	99608.242	3.984071206	87.16214803
5 to 9	0.000106454	2.499778222	99639.63053	4.980655886	77.46877602	6.96862E-05	2.49985482	99595.31959	4.97889908	83.17314144
10 to 14	0.000123867	2.768922553	99586.61105	4.977947939	72.50861992	7.69901E-05	2.632237317	99560.64618	4.977121611	78.20118732
15 to 19	0.000259786	2.765095453	99524.96415	4.973386806	67.55176973	0.000114776	2.601087664	99522.35265	4.974749839	73.23020943
20 to 24	0.000479544	2.660015035	99395.83643	4.964199497	62.63546711	0.000133818	2.604757461	99465.29211	4.971668447	68.27063082
25 to 29	0.000601711	2.598322292	99158.12956	4.950704657	57.77843949	0.000178592	2.618808413	99398.81291	4.967822742	63.31442349
30 to 34	0.000707539	2.52305906	98860.83516	4.9343838	52.94346885	0.000229383	2.653510062	99310.15817	4.962822397	58.36837352
35 to 39	0.000656133	2.577537027	98512.46947	4.917811539	48.12103488	0.000332155	2.672351974	99196.41589	4.955988101	53.43181031
40 to 44	0.000943459	2.66881478	98190.60659	4.898762668	43.26944023	0.000502272	2.756863976	99031.89961	4.946005714	48.51542613
45 to 49	0.001411709	2.754568984	97729.615	4.870860161	38.45949968	0.00093556	2.710375905	98783.6549	4.92862213	43.62918826
50 to 54	0.002466526	2.723277798	97044.33075	4.825030411	33.70898299	0.001444348	2.689665674	98323.04996	4.899803127	38.81848813
55 to 59	0.003961101	2.687299395	95857.81112	4.749481363	29.08835639	0.002251336	2.674404942	97616.64125	4.855438911	34.07616341
60 to 64	0.006175089	2.720665895	93983.63225	4.634028895	24.60837381	0.003348003	2.672214817	96526.82974	4.789180131	29.4253545
65 to 69	0.010642693	2.721462306	91143.37883	4.449600362	20.27833141	0.005154674	2.701160162	94931.39557	4.69116477	24.86662629
70 to 74	0.018164856	2.719601308	86459.4632	4.152792199	16.21499409	0.008422793	2.741717616	92533.82517	4.540750411	20.43000697
75 to 79	0.033524369	2.682069465	79045.14031	3.672969306	12.463436	0.01527358	2.783577367	88761.95009	4.293464955	16.16654583
80 to 84	0.06023937	2.627994542	67076.21126	2.949088503	9.197316338	0.030176195	2.75542576	82353.48564	3.862792723	12.19124937
85 to 89	0.112915573	2.489162345	50092.8406	1.977387373	6.411821121	0.065637697	2.682080848	71103.66079	3.10558789	8.637390243
90 to 94	0.205671127	2.216101015	29091.67566	0.944499527	4.360289724	0.137322908	2.336971971	51838.11664	1.929901811	5.870176738
95 plus	0.305023313	3.301281799	10703.72295	0.366596941	3.301281799	0.237335978	4.28171002	26957.69139	1.207134462	4.28171002

Table 15: Bosnia and Herzegovina 2017 life table, by age and sex. mx=mortality rate, ax=mean person-years lived in an age interval among those who die in that age interval, lx=number of persons left alive at age x, nLx=person-years lived between age x and x+n, ex=life expectancy at age x.

Age Group	Male					Female				
	mx	ax	lx	nLx	ex	mx	ax	lx	nLx	ex
Early Neonatal	0.092693436	0.0095862	100000	0.019161046	74.35944525	0.075416834	0.00958673	100000	0.01916422	79.07071339
Late Neonatal	0.063608288	0.028749577	99822.39062	0.0573271	74.47255166	0.05246256	0.028752652	99855.47016	0.057364476	79.16596352
Post Neonatal	0.000711518	0.461593291	99457.75055	0.917979599	74.68795241	0.000658925	0.461597027	99554.52683	0.918895134	79.34765113
1 to 4	0.000217842	1.999709544	99392.43687	3.973965891	73.8134428	0.000151868	1.999797509	99493.98023	3.978550689	78.47236988
5 to 9	0.000167897	2.499650215	99305.87034	4.963210014	69.87604636	9.82832E-05	2.499795243	99433.56035	4.970456661	74.51883867
10 to 14	0.000168496	2.919124875	99222.54192	4.959387993	64.93263156	0.000116761	2.670471258	99384.71012	4.96788427	69.55423874
15 to 19	0.000507559	2.774332837	99138.97944	4.951355755	59.98489765	0.000194031	2.555013655	99326.70518	4.963980435	64.59329337
20 to 24	0.000840112	2.536179805	98887.67077	4.93417039	55.13025451	0.000168333	2.590699946	99230.38897	4.959508118	59.65349648
25 to 29	0.000657638	2.502090572	98473.14863	4.915582545	50.35159108	0.000267824	2.69159901	99146.9049	4.954282336	54.70153215
30 to 34	0.000852976	2.59296473	98149.88566	4.897439467	45.50914521	0.000415541	2.699673952	99014.21859	4.945983368	49.77120899
35 to 39	0.001045678	2.692864307	97732.15339	4.874847521	40.69252698	0.000668479	2.671955709	98808.69509	4.932758273	44.86908644
40 to 44	0.001837864	2.769661789	97222.41467	4.841276903	35.8916904	0.000972241	2.772904503	98478.95619	4.913308946	40.01032049
45 to 49	0.003476457	2.754459633	96332.68926	4.779326388	31.19748411	0.001958113	2.746289277	98001.27711	4.878535006	35.19174996
50 to 54	0.006253545	2.746292735	94671.28422	4.667782909	26.69642459	0.003336883	2.682911304	97046.04377	4.815074485	30.51098448
55 to 59	0.011439032	2.67331181	91752.57408	4.468709982	22.45797286	0.005014575	2.70768878	95439.41257	4.717744609	25.97925314
60 to 64	0.017228899	2.625088473	86641.6725	4.161832546	18.62461396	0.0086904	2.69637249	93073.90239	4.562371852	21.57046239
65 to 69	0.025097557	2.644870553	79473.12974	3.751964123	15.06737039	0.014138709	2.723389478	89109.63823	4.316570562	17.40977495
70 to 74	0.043205252	2.609919403	70060.23292	3.175299032	11.73606896	0.026881612	2.707449007	83008.12621	3.909561832	13.48886174
75 to 79	0.069159709	2.542624358	56349.26327	2.408487848	8.956644489	0.051226347	2.638686744	72503.16546	3.234203216	10.05049716
80 to 84	0.110402976	2.466303043	39706.78552	1.551773381	6.646005704	0.095082966	2.526116152	55947.4742	2.265117122	7.243776922
85 to 89	0.175085434	2.314230924	22594.23426	0.768693275	4.8146629	0.157826152	2.365346811	34432.64563	1.216464087	5.193321195
90 to 94	0.269243992	2.106663517	9150.749875	0.257304184	3.493202167	0.246682314	2.169174975	15256.87536	0.449416596	3.752211345
95 plus	0.356529724	2.804960214	2229.226584	0.062567359	2.804960214	0.338987264	2.950280456	4183.017337	0.123517094	2.950280456

Table 15: Bosnia and Herzegovina 2100 life table, by age and sex. mx=mortality rate, ax=mean person-years lived in an age interval among those who die in that age interval, lx=number of persons left alive at age x, nLx=person-years lived between age x and x+n, ex=life expectancy at age x.

Age Group	Male					Female				
	mx	ax	lx	nLx	ex	mx	ax	lx	nLx	ex
Early Neonatal	0.011426871	0.009588691	100000	0.019175982	80.94354958	0.011123271	0.0095887	100000	0.019176037	84.26528863
Late Neonatal	0.019364788	0.028761781	99978.09948	0.05748969	80.94209706	0.016021298	0.028762704	99978.67521	0.057495518	84.26404268
Post Neonatal	0.000125661	0.461634903	99867.14846	0.922007608	80.97452132	0.000112776	0.461635822	99886.77504	0.922194287	84.28399643
1 to 4	5.74294E-05	1.999923425	99855.56842	3.993764503	80.06053453	4.64669E-05	1.999938053	99876.37966	3.99468474	83.36939621
5 to 9	6.04409E-05	2.499874081	99832.6632	4.990879951	76.07806937	4.81172E-05	2.499899756	99857.86813	4.992294351	79.38432587
10 to 14	7.87854E-05	3.11696067	99802.54	4.989330399	71.09997649	6.85583E-05	2.738666839	99833.91099	4.990909311	74.40272649
15 to 19	0.000293286	2.857975553	99763.30497	4.984884611	66.12641997	0.00011296	2.509752504	99799.77402	4.988598001	69.42724375
20 to 24	0.000495695	2.539663357	99617.55181	4.974826902	61.21796085	8.27437E-05	2.649484587	99743.56785	4.986184452	64.46495708
25 to 29	0.000348425	2.496810251	99372.63755	4.964318419	56.36155981	0.00014484	2.759010453	99702.49279	4.983478134	59.49046907
30 to 34	0.000446574	2.557836443	99201.72643	4.954700721	51.45436414	0.000231255	2.68038372	99630.47738	4.978830513	54.53145886
35 to 39	0.000453942	2.651927469	98982.71256	4.943861003	46.56273929	0.000319135	2.655998931	99515.53977	4.97205657	49.59097499
40 to 44	0.000766268	2.793484782	98760.41378	4.929636601	41.66151345	0.000465251	2.90320514	99357.08373	4.962946522	44.66521101
45 to 49	0.001579327	2.782655426	98384.76284	4.901999835	36.8085808	0.001154457	2.81642969	99126.47446	4.943821926	39.76124187
50 to 54	0.003051856	2.811597171	97613.45786	4.848124269	32.07234998	0.002187693	2.683255579	98556.38605	4.902977429	34.972259
55 to 59	0.006444606	2.685184557	96140.79209	4.736485157	27.50825437	0.003099307	2.680703203	97485.57838	4.839521121	30.32154252
60 to 64	0.009443879	2.625971514	93107.94709	4.554198663	23.2944364	0.00494274	2.662054778	95989.89712	4.74485055	25.74496721
65 to 69	0.013888154	2.661820939	88848.7042	4.304884976	19.26056765	0.007190849	2.718875168	93654.65729	4.607513899	21.31023232
70 to 74	0.024028833	2.648289827	82982.35881	3.933017292	15.40176451	0.013129499	2.782760319	90365.36512	4.391466368	16.9735242
75 to 79	0.040426594	2.600518352	73821.79512	3.378846278	11.95193787	0.027776655	2.756282185	84663.72297	3.988707786	12.90962775
80 to 84	0.064301391	2.553028523	60872.07916	2.653982525	8.941812337	0.05940536	2.620320546	73819.63118	3.249871264	9.372634861
85 to 89	0.118009205	2.473961965	44969.55641	1.763421919	6.25637759	0.110259145	2.512810948	55338.50171	2.1998455	6.609848159
90 to 94	0.211199464	2.208752447	25712.47669	0.829191734	4.274351218	0.193947904	2.277185306	32539.02154	1.088560456	4.589884728
95 plus	0.309585678	3.252053628	9279.315704	0.314520542	3.252053628	0.290976459	3.472220163	12667.32605	0.459303891	3.472220163

Table 15: Bulgaria 2017 life table, by age and sex. mx=mortality rate, ax=mean person-years lived in an age interval among those who die in that age interval, lx=number of persons left alive at age x, nLx=person-years lived between age x and x+n, ex=life expectancy at age x.

Age Group	Male					Female				
	mx	ax	lx	nLx	ex	mx	ax	lx	nLx	ex
Early Neonatal	0.167421656	0.00958391	100000	0.019147327	71.36322794	0.11491316	0.009585519	100000	0.019156965	78.62784449
Late Neonatal	0.022569358	0.028760898	99679.44266	0.0573126	71.57348691	0.018446122	0.028762035	99779.86486	0.057377143	78.78210755
Post Neonatal	0.002951798	0.461434145	99550.10182	0.917882592	71.6089046	0.002343709	0.461477342	99674.03055	0.919283102	78.80819453
1 to 4	0.000292824	1.999609567	99279.19021	3.968843043	70.87977099	0.000296194	1.999605074	99458.58958	3.97598793	78.05462477
5 to 9	0.000160613	2.499665389	99162.98487	4.956159045	66.9604952	0.000160022	2.49966662	99340.83036	4.965055012	74.14478726
10 to 14	0.000223175	2.909799423	99083.3876	4.951859181	62.01228162	0.000129452	2.719469179	99261.38075	4.961604332	69.20213464
15 to 19	0.000600601	2.742173343	98972.87863	4.941942558	57.0782685	0.000296852	2.623802387	99197.1525	4.956361625	64.24517682
20 to 24	0.000925427	2.583018005	98676.06922	4.922792494	52.24166703	0.000306554	2.596731187	99050.02255	4.948855266	59.33669221
25 to 29	0.000976239	2.585354187	98220.50648	4.89947605	47.47193065	0.00044053	2.645492412	98898.31472	4.939792124	54.42371013
30 to 34	0.001337227	2.686091055	97742.20847	4.87203585	42.69152265	0.000616413	2.651850544	98680.70451	4.926904172	49.53786128
35 to 39	0.002195445	2.680221095	97090.72124	4.829938569	37.95988317	0.00089445	2.717777892	98377.00807	4.908829944	44.68255997
40 to 44	0.003292185	2.720692824	96030.37156	4.765758815	33.34929746	0.001562855	2.755380159	97937.94643	4.879778919	39.87062439
45 to 49	0.005840864	2.728189598	94461.48468	4.661227549	28.85780287	0.002845746	2.697576201	97175.33078	4.827139389	35.16179694
50 to 54	0.010154041	2.688902739	91739.1786	4.481797508	24.63287044	0.004354907	2.675091593	95801.71457	4.742076098	30.62711419
55 to 59	0.016312703	2.643204501	87189.0277	4.198085009	20.77763599	0.006738473	2.654791183	93736.73619	4.613929314	26.24267974
60 to 64	0.024391776	2.600881774	80342.39353	3.795102284	17.3225645	0.009880039	2.637086219	90628.00199	4.428040556	22.05153298
65 to 69	0.034565497	2.558906302	71088.49325	3.277963574	14.23861259	0.014350634	2.660245856	86253.81098	4.172620504	18.03580228
70 to 74	0.046444724	2.551287745	59763.40393	2.683210868	11.451847	0.023593242	2.69944388	80267.36557	3.806818423	14.1822143
75 to 79	0.071648831	2.529595373	47309.75425	2.010035471	8.795266977	0.045257908	2.657594805	71289.45139	3.222991712	10.62801518
80 to 84	0.113648077	2.454530815	32921.34643	1.277056084	6.535209566	0.085309958	2.552172553	56711.96625	2.346101873	7.676815413
85 to 89	0.178595991	2.305617905	18423.87883	0.622157383	4.749500897	0.145848845	2.397183815	36715.0418	1.331020103	5.469300225
90 to 94	0.27252662	2.099714191	7324.055927	0.204622605	3.458269994	0.234237418	2.197823143	17321.90087	0.523107043	3.912236487
95 plus	0.359092729	2.784913175	1752.100916	0.048821478	2.784913175	0.327993305	3.049132229	5080.214327	0.155012938	3.049132229

Table 15: Bulgaria 2100 life table, by age and sex. mx=mortality rate, ax=mean person-years lived in an age interval among those who die in that age interval, lx=number of persons left alive at age x, nLx=person-years lived between age x and x+n, ex=life expectancy at age x.

Age Group	Male					Female				
	mx	ax	lx	nLx	ex	mx	ax	lx	nLx	ex
Early Neonatal	0.044810426	0.009587668	100000	0.019169846	80.58459631	0.031260788	0.009588083	100000	0.019172335	85.51972431
Late Neonatal	0.005703627	0.02876555	99914.12925	0.057475414	80.63466231	0.005289813	0.028765664	99940.07847	0.057491024	85.55165279
Post Neonatal	0.000548423	0.461604877	99881.36271	0.921958921	80.60355266	0.00046855	0.46161055	99909.67531	0.922254246	85.52004736
1 to 4	5.06203E-05	1.999932505	99830.81513	3.992828525	79.72087626	5.52611E-05	1.999926318	99866.47262	3.994217631	84.63353239
5 to 9	3.8958E-05	2.499918839	99810.61229	4.990044643	75.73662815	5.98109E-05	2.499875396	99844.41053	4.991474483	80.65178305
10 to 14	8.56534E-05	3.270637607	99791.17468	4.988772026	70.75085807	6.32181E-05	3.037690226	99814.57254	4.990042269	75.6750362
15 to 19	0.000323312	2.83782183	99748.45231	4.983875859	65.77975022	0.000168232	2.611191786	99783.05154	4.987146415	70.69791061
20 to 24	0.00056788	2.596359635	99587.38751	4.972560238	60.88087554	0.00015908	2.616160115	99699.19693	4.98304229	65.75481513
25 to 29	0.000569146	2.551799243	99305.24168	4.958339514	56.04543178	0.000229654	2.667213929	99619.98348	4.978300506	60.80475414
30 to 34	0.000696801	2.603577667	99023.40679	4.94294714	51.19671482	0.000319672	2.6443167	99505.74322	4.971514342	55.87110623
35 to 39	0.000953122	2.655894587	98679.56083	4.922920573	46.3649512	0.00043323	2.723891516	99346.93638	4.962448844	50.95561865
40 to 44	0.001351709	2.725685474	98211.74019	4.895456258	41.57160719	0.00080894	2.825071688	99132.08704	4.947737816	46.05935558
45 to 49	0.002349419	2.811418231	97551.9133	4.852298392	36.83261339	0.001600784	2.736452371	98732.39557	4.918724639	41.23239956
50 to 54	0.004595415	2.775063919	96416.15586	4.771639704	32.22996252	0.002667126	2.684418449	97946.14502	4.867219218	36.53797679
55 to 59	0.008303168	2.662988875	94232.8193	4.621845011	27.90725289	0.004096341	2.628786253	96650.90991	4.786128546	31.98618773
60 to 64	0.011238616	2.610585894	90420.49811	4.403297355	23.9640066	0.005453151	2.597526266	94697.46475	4.673877046	27.58456007
65 to 69	0.015376652	2.573824949	85511.79984	4.123074585	20.18307718	0.007013587	2.627667901	92163.48892	4.533119036	23.26235779
70 to 74	0.019170245	2.619766899	79231.61876	3.790775545	16.5675316	0.010404352	2.729975838	89009.032	4.348286586	18.98466735
75 to 79	0.031072301	2.63962048	72080.34977	3.361498676	12.94221541	0.020227436	2.717601986	84537.39437	4.042451553	14.83414955
80 to 84	0.053883855	2.574631501	61819.2439	2.746242414	9.639365316	0.038578277	2.642121918	76483.22623	3.513735946	11.09681874
85 to 89	0.103860892	2.517059961	47637.32712	1.916529276	6.712289425	0.079615297	2.622726763	63344.02005	2.681047805	7.824241107
90 to 94	0.195534009	2.227532637	28912.15484	0.956263409	4.527788693	0.156819172	2.331609327	42953.30999	1.535668212	5.337008631
95 plus	0.296581518	3.397238658	11211.63032	0.394782988	3.397238658	0.256206159	3.942381912	19983.68038	0.814623519	3.942381912

Table 15: Croatia 2017 life table, by age and sex. mx=mortality rate, ax=mean person-years lived in an age interval among those who die in that age interval, lx=number of persons left alive at age x, nLx=person-years lived between age x and x+n, ex=life expectancy at age x.

Age Group	Male					Female				
	mx	ax	lx	nLx	ex	mx	ax	lx	nLx	ex
Early Neonatal	0.115873698	0.00958549	100000	0.019156789	75.45741648	0.089938784	0.009586284	100000	0.019161552	81.7520666
Late Neonatal	0.013283793	0.028763459	99778.02486	0.057384604	75.60608304	0.012917405	0.02876356	99827.66385	0.057413757	81.87400357
Post Neonatal	0.000949656	0.461576374	99701.79767	0.920130968	75.60633143	0.000897769	0.46158006	99753.50072	0.92063017	81.87731878
1 to 4	0.000166863	1.999777517	99614.41888	3.983247338	74.74895906	0.000139077	1.999814564	99670.85024	3.985725282	81.02154472
5 to 9	0.000120743	2.499748452	99547.9555	4.975895652	70.79753123	7.62165E-05	2.499841216	99615.41904	4.97982204	77.0655177
10 to 14	0.000133717	3.012348452	99487.87666	4.973071681	65.83877584	5.63796E-05	2.607689074	99577.46499	4.978201826	72.09393901
15 to 19	0.000449892	2.719459304	99421.37935	4.965973953	60.8807941	0.000105445	2.700546977	99549.39821	4.976263402	67.11352763
20 to 24	0.000609502	2.55761091	99197.96561	4.952525732	56.01175631	0.00015807	2.642361604	99496.92611	4.972993062	62.1474899
25 to 29	0.000621504	2.569178019	98896.11024	4.937346367	51.17487302	0.000213773	2.635409995	99418.31817	4.968404491	57.19452716
30 to 34	0.000820615	2.622640625	98589.25526	4.919864899	46.32612017	0.000297559	2.697676745	99312.10765	4.962206059	52.25285876
35 to 39	0.001112165	2.697234971	98185.5298	4.896736155	41.50577577	0.000497492	2.699079576	99164.45374	4.952553577	47.32662132
40 to 44	0.001891358	2.731600315	97640.9441	4.861191751	36.72215972	0.000775885	2.740374374	98918.07169	4.937247372	42.43773379
45 to 49	0.003263623	2.74349764	96721.55092	4.800724778	32.04514572	0.001401309	2.737977335	98535.00617	4.911182945	37.59199929
50 to 54	0.005857948	2.733929843	95154.86482	4.695417876	27.52740302	0.002402583	2.720654701	97846.82007	4.86569579	32.83703793
55 to 59	0.010314888	2.688897772	92404.56711	4.512664298	23.26506978	0.004021597	2.704551243	96677.85553	4.78968025	28.20103245
60 to 64	0.016503896	2.643275398	87750.48369	4.223290652	19.35601442	0.006556599	2.696997831	94751.79733	4.667125269	23.71908485
65 to 69	0.024775133	2.61038105	80781.93263	3.81340184	15.7973376	0.010774216	2.695232621	91692.14737	4.473542647	19.42025868
70 to 74	0.03651294	2.603741365	71337.33215	3.280020648	12.54291313	0.018168073	2.71840515	86873.2964	4.170830714	15.34758526
75 to 79	0.059388984	2.577651929	59367.07537	2.595264661	9.54695003	0.034742962	2.718032001	79298.50132	3.673810594	11.55348452
80 to 84	0.100946722	2.500862376	43965.4543	1.755759892	6.989082927	0.074074104	2.602699522	66542.48015	2.825776483	8.246914276
85 to 89	0.16463068	2.340494548	26258.51657	0.913419829	5.017881329	0.131647446	2.437778376	45630.37773	1.706572782	5.834449897
90 to 94	0.259363999	2.127380417	11235.30585	0.322040755	3.601993066	0.219100854	2.231835644	23189.7717	0.722082575	4.124714598
95 plus	0.348773543	2.867325936	2889.162618	0.082885357	2.867325936	0.314429694	3.180783447	7386.347689	0.235143965	3.180783447

Table 15: Croatia 2100 life table, by age and sex. mx=mortality rate, ax=mean person-years lived in an age interval among those who die in that age interval, lx=number of persons left alive at age x, nLx=person-years lived between age x and x+n, ex=life expectancy at age x.

Age Group	Male					Female				
	mx	ax	lx	nLx	ex	mx	ax	lx	nLx	ex
Early Neonatal	0.032086044	0.009588058	100000	0.019172183	82.79484576	0.026769865	0.009588221	100000	0.01917316	86.85185067
Late Neonatal	0.004137209	0.028765982	99938.49298	0.057492018	82.82666901	0.005711037	0.028765548	99948.67812	0.057495274	86.87723122
Post Neonatal	0.000207524	0.461629093	99914.71264	0.922411858	82.78887288	0.000193745	0.461630072	99915.84699	0.922428196	86.84822584
1 to 4	4.34523E-05	1.999942064	99895.57199	3.995475663	81.8813724	3.44148E-05	1.999954113	99897.97669	3.995644061	85.94038448
5 to 9	3.97898E-05	2.499917104	99878.21182	4.993413872	77.89525064	3.08446E-05	2.499935741	99884.22677	4.993826275	81.95192673
10 to 14	5.57918E-05	3.15311373	99858.34406	4.992366032	72.91021375	2.79906E-05	2.774199159	99868.82486	4.99311351	76.96416732
15 to 19	0.000233151	2.813524185	99830.49566	4.988884648	67.92966919	5.44216E-05	2.741717663	99854.85232	4.992117661	71.97455017
20 to 24	0.000379024	2.630401067	99714.26048	4.981162626	63.00486867	8.52768E-05	2.719714827	99827.68977	4.990392304	66.99334335
25 to 29	0.00039139	2.577193059	99525.79489	4.971550113	58.11817806	0.000125672	2.656041518	99785.14601	4.987767939	62.02070858
30 to 34	0.00050195	2.562443165	99331.58689	4.960497313	53.22616133	0.000162197	2.672371025	99722.48808	4.984222153	57.0579086
35 to 39	0.000547641	2.603826997	99083.20861	4.947640363	48.35242851	0.000245536	2.685500777	99641.67927	4.979216435	52.10184231
40 to 44	0.000772573	2.696949321	98813.00689	4.931802773	43.47671347	0.000350382	2.773586047	99519.48261	4.972059	47.16196339
45 to 49	0.001276114	2.763896142	98432.98904	4.907555484	38.63279472	0.000685554	2.77645375	99345.3595	4.959641389	42.23912435
50 to 54	0.00248555	2.758538702	97808.25926	4.863232913	33.85970941	0.001236675	2.760396419	99005.63047	4.936519913	37.37326696
55 to 59	0.004700447	2.685345922	96602.91919	4.778153868	29.24299684	0.00221681	2.724678237	98395.95644	4.894942281	32.58531449
60 to 64	0.007258372	2.647533176	94366.13052	4.639398136	24.86354104	0.003575737	2.686504532	97313.41662	4.825637344	27.91379985
65 to 69	0.011034856	2.639697488	91018.29319	4.436083885	20.66850797	0.005474332	2.691386454	95593.83359	4.719850739	23.36265209
70 to 74	0.016553099	2.667930514	86171.92531	4.149961189	16.66700722	0.008635425	2.755597725	93025.87414	4.563207294	18.92667999
75 to 79	0.02983186	2.660653133	79384.90474	3.714697947	12.85010302	0.017519261	2.823396089	89114.61783	4.293180378	14.62363568
80 to 84	0.056146345	2.599631324	68523.50751	3.033404759	9.449394517	0.042775452	2.699546684	81688.56148	3.726241328	10.67691069
85 to 89	0.107324964	2.505829661	52203.46267	2.083517251	6.580360375	0.086344241	2.596572717	66171.92985	2.758905412	7.513271314
90 to 94	0.199633106	2.224436544	31089.76106	1.019293047	4.4531844	0.165428431	2.322205679	43364.26594	1.52455321	5.141825197
95 plus	0.300045109	3.354479295	11750.85067	0.407677823	3.354479295	0.264390909	3.819031306	19283.45226	0.761715416	3.819031306

Table 15: Czech Republic 2017 life table, by age and sex. mx=mortality rate, ax=mean person-years lived in an age interval among those who die in that age interval, lx=number of persons left alive at age x, nLx=person-years lived between age x and x+n, ex=life expectancy at age x.

Age Group	Male					Female				
	mx	ax	lx	nLx	ex	mx	ax	lx	nLx	ex
Early Neonatal	0.052875371	0.00958742	100000	0.019168362	76.35206553	0.03563387	0.009587949	100000	0.019171531	82.09830395
Late Neonatal	0.008460485	0.028764789	99898.6475	0.057461948	76.41033794	0.006991531	0.028765195	99931.68473	0.05748338	82.13524278
Post Neonatal	0.001082966	0.461566903	99850.033	0.921442311	76.38999153	0.00075995	0.46158985	99891.49552	0.921962381	82.1107424
1 to 4	0.000140549	1.999812601	99750.2471	3.988888543	75.54266031	0.00012949	1.999827346	99821.43214	3.99182341	81.24476409
5 to 9	7.223E-05	2.499849521	99694.18535	4.983809282	71.58401722	5.84804E-05	2.499878166	99769.74288	4.987757901	77.28582074
10 to 14	9.60433E-05	3.149543576	99658.18809	4.982023787	66.60897139	9.18553E-05	2.774272785	99740.57459	4.986009386	72.30769156
15 to 19	0.000371911	2.843009595	99610.33973	4.976524823	61.6394522	0.000179565	2.645061887	99694.7757	4.982631891	67.33963159
20 to 24	0.000710463	2.594466878	99425.25848	4.962781306	56.74887726	0.000217236	2.594584067	99605.3055	4.977664299	62.39773279
25 to 29	0.000697509	2.53212342	99072.67365	4.94512136	51.94155735	0.000278766	2.621346935	99497.1732	4.971562132	57.46271227
30 to 34	0.000823567	2.615157713	98727.75104	4.926711412	47.11413542	0.000380515	2.680361423	99358.58405	4.96354827	52.53918852
35 to 39	0.001161153	2.658822725	98322.00958	4.902772985	42.29771816	0.000610264	2.674862843	99169.71539	4.951459967	47.63411931
40 to 44	0.001725046	2.730926141	97752.73652	4.868580729	37.52848536	0.000896914	2.728963721	98867.55004	4.933328558	42.77148216
45 to 49	0.003118235	2.71384656	96912.91521	4.811348456	32.8299237	0.001607756	2.704541536	98425.08244	4.903159024	37.95143386
50 to 54	0.005045267	2.73869557	95412.71279	4.716825362	28.30326544	0.002505686	2.717816578	97636.7989	4.854083078	33.23591153
55 to 59	0.009279222	2.705403915	93033.16875	4.554690227	23.95688682	0.004315049	2.695307997	96420.57592	4.773559194	28.62073055
60 to 64	0.015267893	2.66074095	88807.37092	4.287274709	19.9677546	0.006774555	2.694919135	94360.91282	4.645509321	24.18642518
65 to 69	0.023832746	2.617988493	82262.99416	3.89225432	16.34420629	0.011259253	2.681523875	91214.14169	4.444701409	19.92760432
70 to 74	0.035064063	2.589855091	72989.71715	3.365228074	13.08777504	0.018150497	2.68294868	86210.65879	4.136613409	15.9281986
75 to 79	0.052970952	2.579407085	61195.80256	2.712268101	10.11093496	0.031613477	2.70067534	78704.81686	3.668681015	12.19100847
80 to 84	0.090265194	2.521380195	46839.28665	1.914120848	7.420018481	0.064126066	2.626165642	67112.60544	2.912568727	8.830075894
85 to 89	0.152176427	2.373082248	29577.75416	1.056846409	5.280843479	0.118313726	2.478866135	48448.94392	1.866271239	6.220547685
90 to 94	0.247350734	2.151942739	13510.37538	0.396464003	3.742634007	0.204441438	2.263265626	26387.66803	0.846305768	4.351010978
95 plus	0.339249142	2.94783373	3711.25623	0.109458176	2.94783373	0.301099187	3.321517194	9099.976812	0.30244866	3.321517194

Table 15: Czech Republic 2100 life table, by age and sex. mx=mortality rate, ax=mean person-years lived in an age interval among those who die in that age interval, lx=number of persons left alive at age x, nLx=person-years lived between age x and x+n, ex=life expectancy at age x.

Age Group	Male					Female				
	mx	ax	lx	nLx	ex	mx	ax	lx	nLx	ex
Early Neonatal	0.011692924	0.009588683	100000	0.019175932	84.1855977	0.007418823	0.009588814	100000	0.019176718	87.53026267
Late Neonatal	0.00257186	0.028766414	99977.57961	0.057517092	84.1853272	0.002081491	0.028766549	99985.77373	0.057522617	87.52353538
Post Neonatal	0.000248599	0.461626176	99962.78813	0.922838195	84.14026197	0.000167667	0.461631925	99973.80123	0.922974342	87.47648573
1 to 4	3.30632E-05	1.999955918	99939.84881	3.997329638	83.23618632	3.22207E-05	1.999957039	99958.32683	3.998075448	86.56666638
5 to 9	2.09592E-05	2.499956337	99926.63349	4.99606989	79.24693064	2.41113E-05	2.49994977	99945.44598	4.996971098	82.57756076
10 to 14	4.01012E-05	3.502057407	99916.16243	4.995483591	74.25495866	5.54246E-05	3.123374843	99933.39833	4.996055303	77.58720368
15 to 19	0.00021579	2.93656306	99896.1317	4.992514234	69.26915098	0.000111899	2.662983766	99905.73338	4.993957724	72.60792457
20 to 24	0.000472583	2.598077232	99788.42088	4.983750866	64.34056261	0.000137539	2.586728319	99849.86866	4.990833028	67.64701832
25 to 29	0.000446305	2.506073383	99552.98631	4.972110792	59.48616929	0.000171237	2.580699109	99781.24521	4.986993245	62.69168695
30 to 34	0.000482928	2.546772575	99331.21945	4.960682388	54.61304514	0.00021021	2.64916604	99695.89263	4.982295379	57.74296985
35 to 39	0.000564621	2.588211452	99091.84641	4.947845159	49.73851476	0.000303255	2.635479799	99591.22432	4.975975918	52.80062781
40 to 44	0.00073486	2.681383737	98812.78556	4.932206017	44.87132711	0.000419797	2.728148361	99440.40206	4.967247986	47.87624098
45 to 49	0.001235074	2.696056375	98450.74676	4.908566901	40.0260418	0.000798817	2.698707587	99232.00717	4.952485053	42.97027911
50 to 54	0.002019173	2.745596633	97845.16855	4.870028937	35.25609192	0.00128445	2.699423199	98836.65797	4.927263733	38.12997061
55 to 59	0.003820641	2.700852178	96863.29212	4.800986651	30.58336091	0.002219488	2.670507741	98204.4768	4.884936781	33.35564473
60 to 64	0.006189813	2.660039476	95032.00126	4.68387262	26.11675966	0.003368142	2.661917102	97122.11675	4.8181857	28.69403804
65 to 69	0.009515271	2.634113363	92139.54987	4.505868113	21.84744389	0.005339036	2.654134085	95502.96585	4.716216165	24.13094026
70 to 74	0.013945448	2.648639298	87870.01921	4.254656489	17.77342536	0.008257699	2.692645395	92994.51768	4.563110071	19.70442867
75 to 79	0.022811598	2.684486215	81977.04334	3.894756471	13.85328948	0.015085702	2.77145025	89250.32703	4.31827718	15.40978901
80 to 84	0.044835706	2.623883612	73169.49521	3.312011552	10.19052626	0.035255184	2.681111095	82802.90236	3.832197313	11.38219901
85 to 89	0.091943729	2.552434171	58598.16138	2.402787713	7.063198587	0.074716058	2.640678751	69544.00412	2.967727084	8.020611393
90 to 94	0.182773564	2.246343427	37078.26199	1.243516297	4.720016295	0.150854726	2.340710275	48002.59898	1.72834217	5.456969269
95 plus	0.286080262	3.507303972	14894.4362	0.53102216	3.507303972	0.250600682	4.017832583	22722.53374	0.933442619	4.017832583

Table 15: Hungary 2017 life table, by age and sex. mx=mortality rate, ax=mean person-years lived in an age interval among those who die in that age interval, lx=number of persons left alive at age x, nLx=person-years lived between age x and x+n, ex=life expectancy at age x.

Age Group	Male					Female				
	mx	ax	lx	nLx	ex	mx	ax	lx	nLx	ex
Early Neonatal	0.095136498	0.009586125	100000	0.019160598	73.13017847	0.076312924	0.009586702	100000	0.019164055	80.17844697
Late Neonatal	0.017366139	0.028762333	99817.71873	0.057400693	73.24452504	0.012695082	0.028763621	99853.75675	0.057429131	80.27668028
Post Neonatal	0.001685977	0.461524067	99718.0395	0.919968189	73.26017611	0.001475064	0.461539049	99780.85127	0.920637262	80.27777673
1 to 4	0.00019673	1.999737693	99562.9446	3.980951334	72.4502949	0.000158391	1.999788812	99645.05593	3.984539922	79.46326076
5 to 9	8.77735E-05	2.499817139	99484.63249	4.973140315	68.50575571	7.15689E-05	2.499850898	99581.9469	4.978206597	75.51235389
10 to 14	0.000150759	2.92483726	99440.98332	4.970493937	63.53472997	8.98011E-05	2.699353065	99546.3191	4.97628788	70.53848554
15 to 19	0.000395536	2.697991608	99366.05043	4.963782957	58.58043364	0.000157639	2.665646875	99501.63195	4.973251593	65.5689497
20 to 24	0.000528144	2.604404625	99169.71668	4.952220174	53.69104352	0.000215463	2.584097139	99423.23453	4.968575454	60.61854087
25 to 29	0.000663045	2.594068598	98908.16994	4.937531974	48.82610058	0.000245086	2.658241472	99316.18059	4.962960679	55.68108266
30 to 34	0.000832114	2.660842399	98580.79202	4.919464401	43.97959441	0.000402521	2.721001744	99194.54655	4.955181904	50.74608323
35 to 39	0.001314909	2.717546821	98171.44148	4.893884942	39.1518352	0.000674376	2.719762291	98995.09228	4.942154909	45.84281586
40 to 44	0.002228468	2.815065025	97527.95306	4.852770038	34.39214678	0.001119727	2.765106082	98661.81026	4.920776265	40.98843565
45 to 49	0.004775723	2.796253852	96446.56502	4.772106404	29.74606279	0.002118238	2.777019847	98110.83012	4.882550759	36.20301839
50 to 54	0.009375566	2.721360464	94167.6754	4.609907796	25.3979893	0.004004042	2.729546426	97076.62552	4.810104417	31.5589967
55 to 59	0.015866288	2.642020888	89846.09869	4.330322411	21.48830378	0.006738272	2.666456311	95150.74862	4.683893448	27.14230855
60 to 64	0.022898913	2.5948573	82976.75591	3.932319846	18.04802759	0.009895285	2.633636879	91994.91367	4.494517414	22.98165442
65 to 69	0.031971305	2.560866072	73974.67695	3.431261515	14.92816835	0.014188621	2.632371473	87548.12905	4.235165263	19.01485973
70 to 74	0.042817308	2.553002168	63009.00253	2.851826482	12.08035814	0.021281595	2.667230793	81540.44811	3.884255315	15.22154443
75 to 79	0.064174398	2.537929107	50805.55034	2.193916961	9.369140511	0.03754199	2.66083905	73277.26319	3.368232335	11.6369655
80 to 84	0.101006042	2.468000031	36737.79872	1.46309191	6.986146509	0.06942765	2.582735211	60639.56643	2.596552452	8.50765761
85 to 89	0.164310971	2.341307512	21974.39388	0.764914902	5.024243762	0.125294231	2.456954032	42626.81606	1.61670964	6.012288649
90 to 94	0.259060178	2.128014147	9417.933525	0.270105032	3.605396968	0.212180112	2.246922569	22389.00951	0.706904196	4.228679818
95 plus	0.34853436	2.869276251	2425.739106	0.069635663	2.869276251	0.308161723	3.245367012	7402.608655	0.240396079	3.245367012

Table 15: Hungary 2100 life table, by age and sex. mx=mortality rate, ax=mean person-years lived in an age interval among those who die in that age interval, lx=number of persons left alive at age x, nLx=person-years lived between age x and x+n, ex=life expectancy at age x.

Age Group	Male					Female				
	mx	ax	lx	nLx	ex	mx	ax	lx	nLx	ex
Early Neonatal	0.020016473	0.009588428	100000	0.019174402	81.68607067	0.016613702	0.009588532	100000	0.019175027	85.64095265
Late Neonatal	0.004837236	0.028765789	99961.62298	0.057504165	81.69825698	0.003624998	0.028766123	99968.14554	0.057509922	85.6490607
Post Neonatal	0.000440247	0.461612561	99933.81082	0.922489089	81.66346613	0.000378892	0.46161692	99947.29962	0.922639718	85.60937471
1 to 4	4.44244E-05	1.999940768	99893.20666	3.995373314	80.77317839	3.70535E-05	1.999950595	99912.34526	3.996197682	84.71586837
5 to 9	2.41791E-05	2.499949622	99875.45977	4.993471135	76.78717323	2.69627E-05	2.499943826	99897.53926	4.994540319	80.72812772
10 to 14	6.23629E-05	3.246158682	99863.38598	4.992558202	71.79615122	4.70165E-05	2.866497493	99884.07415	4.993673463	75.73866078
15 to 19	0.000207759	2.685360411	99832.25392	4.989206925	66.81758628	8.41257E-05	2.619773399	99860.59896	4.992033611	70.7558182
20 to 24	0.000268213	2.57971294	99728.61156	4.983177482	61.88403739	0.000100949	2.560911554	99818.60815	4.989697371	65.78443087
25 to 29	0.000297653	2.511169947	99595.00356	4.976069091	56.96342044	0.000109126	2.602891037	99768.24841	4.987096078	60.81630367
30 to 34	0.000290108	2.535385619	99446.95509	4.968812027	52.04411832	0.000144462	2.65825475	99713.84373	4.983993573	55.84800513
35 to 39	0.000374313	2.66253886	99302.91282	4.960808976	47.11548446	0.000210618	2.7391946	99641.8654	4.979686082	50.88633095
40 to 44	0.000618093	2.91782106	99117.49336	4.949299445	42.19802474	0.000359235	2.908361736	99537.03031	4.973029846	45.93677313
45 to 49	0.001505734	2.902500297	98812.27255	4.924901304	37.31834829	0.00084759	2.923637115	99358.47934	4.958983637	41.01364207
50 to 54	0.003630011	2.796717319	98072.4615	4.864423914	32.57459313	0.001920111	2.865308299	98938.61494	4.926267939	36.17415871
55 to 59	0.006854227	2.676316641	96314.19873	4.740030623	28.11092703	0.003837459	2.731812907	97994.9084	4.856838619	31.49243832
60 to 64	0.009586291	2.61617356	93088.7732	4.550832104	23.98213439	0.005634436	2.617310915	96138.32684	4.743091152	27.04373756
65 to 69	0.013441221	2.607755184	88751.4468	4.300379698	20.0202595	0.006993806	2.620011586	93475.67864	4.597341939	22.73586349
70 to 74	0.019109973	2.636384244	83023.0228	3.97345597	16.2152449	0.010063942	2.749494002	90276.20423	4.414191561	18.44482694
75 to 79	0.031906113	2.647556947	75515.02033	3.515798155	12.56125718	0.020945248	2.764770423	85862.13653	4.102293418	14.24368058
80 to 84	0.05762726	2.564467328	64488.94038	2.836082571	9.254930079	0.044211769	2.67500772	77357.33848	3.513441975	10.48942787
85 to 89	0.109885109	2.496466916	48551.75454	1.917381462	6.449387925	0.088727823	2.586204848	62158.41671	2.574000943	7.377178177
90 to 94	0.203102158	2.224669515	28167.0433	0.910623813	4.37734175	0.168747355	2.320527123	40078.03905	1.395655308	5.054203097
95 plus	0.303069742	3.310954341	10198.46	0.3442168	3.310954341	0.267608785	3.763378038	17341.20512	0.66957347	3.763378038

Table 15: Macedonia 2017 life table, by age and sex. mx=mortality rate, ax=mean person-years lived in an age interval among those who die in that age interval, lx=number of persons left alive at age x, nLx=person-years lived between age x and x+n, ex=life expectancy at age x.

Age Group	Male					Female				
	mx	ax	lx	nLx	ex	mx	ax	lx	nLx	ex
Early Neonatal	0.250777111	0.009581355	100000	0.01913204	73.89152051	0.21130383	0.009582565	100000	0.019139277	80.2113603
Late Neonatal	0.025442094	0.028760105	99520.2368	0.057216331	74.22849963	0.027213911	0.028759616	99595.59688	0.057256739	80.51784898
Post Neonatal	0.002391035	0.46147398	99374.66317	0.916502003	74.27965298	0.00222656	0.461485665	99439.77403	0.917172098	80.58643183
1 to 4	0.000287572	1.99961657	99155.52641	3.963940922	73.51950533	0.000219605	1.999707193	99235.55854	3.967679498	79.82802705
5 to 9	0.000171881	2.499641914	99041.54171	4.949949873	69.60181911	0.000161825	2.499662864	99148.42917	4.955416439	75.89642261
10 to 14	0.000133541	2.934669417	98956.46544	4.94645866	64.65951121	0.000161774	2.698298504	99068.23924	4.951568971	70.9558304
15 to 19	0.000450894	2.664315977	98890.41163	4.939318885	59.70073753	0.000317023	2.640221236	98988.1364	4.945705453	66.011054
20 to 24	0.000490595	2.58422023	98667.70317	4.927545163	54.8294467	0.000374536	2.560901888	98831.34794	4.937056691	61.11155084
25 to 29	0.000652093	2.57028469	98425.96216	4.913513175	49.95773024	0.000427321	2.58411189	98646.43967	4.927235211	56.22128943
30 to 34	0.000714339	2.641009527	98105.55922	4.897026158	45.11245141	0.000548974	2.640574272	98435.89097	4.915427884	51.3360129
35 to 39	0.001143061	2.71998544	97755.75076	4.875082593	40.26438608	0.000801474	2.690267151	98166.04877	4.899233513	46.46985972
40 to 44	0.001939019	2.794892219	97198.51188	4.839235067	35.4795522	0.001289724	2.708256743	97773.39243	4.874263073	41.64564942
45 to 49	0.003952403	2.749617756	96260.21048	4.770581082	30.7980216	0.002112556	2.696508289	97144.75844	4.833718193	36.8975594
50 to 54	0.006883485	2.692557097	94374.80787	4.644969381	26.35813131	0.003339485	2.689215761	96123.63753	4.769383089	32.26078527
55 to 59	0.01088608	2.671602555	91177.80722	4.446207601	22.18758714	0.005288303	2.695156566	94531.02268	4.669643216	27.75880791
60 to 64	0.01731683	2.647719545	86338.48278	4.147998215	18.28107659	0.008657677	2.682651246	92061.94144	4.512577302	23.43075825
65 to 69	0.026675419	2.640238211	79157.31377	3.723560537	14.69893803	0.013794272	2.673823035	88156.15224	4.270801451	19.34978051
70 to 74	0.044312467	2.609141644	69228.40479	3.129999176	11.42810972	0.022562101	2.660363842	82267.09012	3.907175987	15.54350923
75 to 79	0.073322617	2.542358567	55366.80062	2.345962249	8.636042157	0.037572639	2.618447491	73455.51254	3.371212092	12.08925183
80 to 84	0.118396761	2.454498742	38180.75192	1.46733794	6.380047722	0.060498814	2.545071366	60793.20856	2.646663742	9.062307246
85 to 89	0.183922814	2.292778956	20827.89862	0.695520875	4.653892637	0.112850526	2.496559159	44784.47987	1.746050081	6.392244347
90 to 94	0.277471364	2.089195571	8050.332565	0.222779055	3.406940649	0.198293047	2.275843412	25084.40065	0.814392247	4.452324324
95 plus	0.362938346	2.755428001	1874.520072	0.05168353	2.755428001	0.295453978	3.384692759	8938.624982	0.302588049	3.384692759

Table 15: Macedonia 2100 life table, by age and sex. mx=mortality rate, ax=mean person-years lived in an age interval among those who die in that age interval, lx=number of persons left alive at age x, nLx=person-years lived between age x and x+n, ex=life expectancy at age x.

Age Group	Male					Female				
	mx	ax	lx	nLx	ex	mx	ax	lx	nLx	ex
Early Neonatal	0.057089287	0.009587291	100000	0.019167599	81.49032058	0.046028856	0.00958763	100000	0.019169626	87.82202727
Late Neonatal	0.007685573	0.028765003	99890.76288	0.057458721	81.56085755	0.010598334	0.0287642	99911.85226	0.057466032	87.88020204
Post Neonatal	0.000377147	0.461617043	99846.70917	0.921712051	81.53976742	0.000332149	0.461620239	99851.04373	0.921771138	87.87612524
1 to 4	6.60829E-05	1.999911887	99811.9929	3.991952606	80.64472943	5.00822E-05	1.999933226	99820.4544	3.992418597	86.97956716
5 to 9	5.15389E-05	2.499892639	99785.64164	4.988639706	76.665364	6.67204E-05	2.499860999	99800.47865	4.989192602	82.99642628
10 to 14	5.66477E-05	3.03774298	99759.95155	4.987387517	71.68433453	9.02306E-05	2.896025499	99767.23056	4.987347486	78.02310552
15 to 19	0.000211155	2.600117043	99731.75232	4.984060957	66.70361329	0.000174321	2.623412056	99722.28393	4.984036338	73.05687686
20 to 24	0.000235279	2.661407546	99626.60078	4.978513121	61.77042916	0.000181001	2.566635186	99635.47535	4.979568349	68.11803792
25 to 29	0.000354921	2.568917038	99509.68463	4.97117238	56.83896377	0.000218445	2.614430487	99545.42672	4.974655688	63.17717464
30 to 34	0.000361699	2.570927115	99333.58126	4.96227906	51.93417562	0.000276576	2.617645634	99436.87032	4.968553255	58.24303551
35 to 39	0.000458175	2.656658151	99154.59267	4.952369409	47.02225081	0.000357056	2.684280194	99299.5807	4.960868163	53.3195936
40 to 44	0.000771331	2.761010876	98928.24324	4.937786385	42.12285119	0.000584548	2.768535457	99122.58466	4.949648727	48.40956689
45 to 49	0.00167548	2.751219131	98548.33161	4.908711658	37.27283363	0.001093434	2.738321029	98833.47156	4.929453575	43.54237041
50 to 54	0.00326163	2.685709818	97728.43175	4.849553561	32.55887929	0.001817879	2.710546483	98294.96876	4.894323154	38.76443858
55 to 59	0.005260366	2.648369532	96153.68037	4.748909077	28.04172163	0.002893355	2.681872608	97406.76844	4.837835287	34.090497
60 to 64	0.008484964	2.635081359	93667.16859	4.591548239	23.70906121	0.004318213	2.655522058	96011.20374	4.752390329	29.54330227
65 to 69	0.013662539	2.626139534	89798.2643	4.350013397	19.60716503	0.0060548	2.666640361	93969.69613	4.63308555	25.12191433
70 to 74	0.022156157	2.609454764	83934.96268	3.988250101	15.78007185	0.00920514	2.714791932	91185.73949	4.465461237	20.80272058
75 to 79	0.035553801	2.575166748	75274.72224	3.470122098	12.28950674	0.015858426	2.6685312	87117.98813	4.201655918	16.64359771
80 to 84	0.060677052	2.525387539	63197.18099	2.760664519	9.138731376	0.024206342	2.608812773	80532.61688	3.808859618	12.78359609
85 to 89	0.113219475	2.487802691	47091.96956	1.854643608	6.3863481	0.056038858	2.71998754	71429.94558	3.17228413	9.073080984
90 to 94	0.206215727	2.21705831	27186.6333	0.879486661	4.344895241	0.125055171	2.352353013	53954.33405	2.035992896	6.138987664
95 plus	0.305519662	3.292424387	9888.378597	0.33591815	3.292424387	0.225668541	4.451171317	28960.75335	1.305989577	4.451171317

Table 15: Montenegro 2017 life table, by age and sex. mx=mortality rate, ax=mean person-years lived in an age interval among those who die in that age interval, lx=number of persons left alive at age x, nLx=person-years lived between age x and x+n, ex=life expectancy at age x.

Age Group	Male					Female				
	mx	ax	lx	nLx	ex	mx	ax	lx	nLx	ex
Early Neonatal	0.104130591	0.00958585	100000	0.019158945	74.08449753	0.088228572	0.009586337	100000	0.019161866	78.90473856
Late Neonatal	0.010198492	0.02876431	99800.49837	0.057402623	74.21339283	0.01076899	0.028764153	99830.93822	0.057419188	79.0191659
Post Neonatal	0.00078003	0.461588424	99741.95743	0.920573669	74.19939944	0.000938696	0.461577152	99769.10411	0.920756781	79.01058749
1 to 4	0.00020913	1.99972116	99670.15163	3.985139066	73.32923573	0.000137033	1.99981729	99682.67411	3.986214405	78.15540597
5 to 9	0.000135076	2.499718592	99586.81343	4.977659625	69.38892892	0.000118936	2.499752216	99628.05117	4.979921695	74.19716027
10 to 14	0.000219723	2.648555282	99519.57919	4.973409218	64.43411987	0.000118709	2.744074331	99568.8225	4.977108311	69.23981013
15 to 19	0.000292024	2.925463356	99410.30391	4.967506166	59.50202184	0.000258355	2.600050415	99509.74033	4.972404073	64.27928477
20 to 24	0.000818975	2.620466324	99265.2422	4.953608584	54.58464918	0.000243324	2.59388892	99381.27631	4.966156439	59.35899116
25 to 29	0.000769346	2.515836532	98859.56047	4.933549219	49.79775105	0.000369003	2.63292515	99260.43871	4.958690828	54.42807266
30 to 34	0.000884206	2.630335918	98480.01054	4.913705848	44.9798604	0.000480179	2.662994027	99077.46389	4.948320613	49.52369058
35 to 39	0.001333409	2.619651504	98045.55565	4.886768846	40.16738272	0.000747911	2.738418693	98839.85995	4.933647996	44.63629296
40 to 44	0.001667423	2.787657124	97393.9928	4.851803707	35.41836003	0.001344325	2.679649788	98470.87692	4.908233592	39.79320936
45 to 49	0.003692887	2.771380128	96585.07315	4.789837885	30.69138171	0.001925814	2.717783401	97811.07767	4.869153829	35.04343658
50 to 54	0.006664657	2.742913058	94816.52238	4.67058097	26.21160307	0.003411561	2.72890476	96873.42556	4.806433533	30.35616095
55 to 59	0.012099316	2.684666217	91704.56406	4.460319493	22.00716518	0.00583031	2.675506486	95233.82722	4.698028617	25.8315583
60 to 64	0.01911491	2.641643351	86310.18484	4.129461303	18.21369337	0.008705207	2.698846603	92495.11249	4.53394887	21.51682941
65 to 69	0.029368463	2.613121845	78421.85663	3.664456085	14.77894876	0.015204823	2.720181617	88549.05441	4.279165071	17.3549863
70 to 74	0.045023543	2.580107855	67670.54983	3.051536761	11.71101993	0.028112105	2.672474114	82044.88473	3.850438037	13.51457917
75 to 79	0.069738267	2.528152171	53952.17843	2.30170043	9.032997574	0.0483818	2.643712396	71226.86237	3.197185352	10.16067736
80 to 84	0.106599268	2.460259185	37935.96522	1.493603072	6.782227167	0.095112041	2.53627331	55773.40005	2.259852814	7.243510237
85 to 89	0.170639625	2.325353407	22059.11105	0.758032443	4.900375278	0.15796826	2.365018142	34309.55776	1.211910737	5.190656903
90 to 94	0.265050691	2.115473666	9159.404534	0.25982054	3.539096067	0.246824076	2.168842456	15196.34803	0.447589247	3.750668424
95 plus	0.353241008	2.831274021	2287.619664	0.064863027	2.831274021	0.339109178	2.949332143	4165.485712	0.122996663	2.949332143

Table 15: Montenegro 2100 life table, by age and sex. mx=mortality rate, ax=mean person-years lived in an age interval among those who die in that age interval, lx=number of persons left alive at age x, nLx=person-years lived between age x and x+n, ex=life expectancy at age x.

Age Group	Male					Female				
	mx	ax	lx	nLx	ex	mx	ax	lx	nLx	ex
Early Neonatal	0.020946385	0.009588399	100000	0.019174231	79.9830105	0.014675406	0.009588591	100000	0.019175384	83.80892671
Late Neonatal	0.002357214	0.028766473	99959.84255	0.057507243	79.99596435	0.002225653	0.028766509	99971.86127	0.057514375	83.8132695
Post Neonatal	0.000140561	0.461633851	99946.28877	0.922731895	79.94928101	0.000161025	0.461632397	99959.06176	0.922841098	83.76643868
1 to 4	5.80913E-05	1.999922545	99933.32094	3.996868508	79.03631298	4.13898E-05	1.999944813	99944.20321	3.997437264	82.85550835
5 to 9	3.89868E-05	2.49991878	99910.10587	4.995018488	75.05419305	5.36166E-05	2.499888298	99927.6608	4.995713542	78.86886148
10 to 14	0.00010639	2.781656814	99890.63508	4.993323332	70.06830398	6.60174E-05	2.804270647	99900.88287	4.994317103	73.8892754
15 to 19	0.000153817	3.066375616	99837.52475	4.99036397	65.10407752	0.000156888	2.706878277	99867.91993	4.991542232	68.91269359
20 to 24	0.000500058	2.656361901	99760.79622	4.982101783	60.15158455	0.000167077	2.637929333	99789.68487	4.987479614	63.96441718
25 to 29	0.00042971	2.493889793	99511.93071	4.970204946	55.29482682	0.000224837	2.632244574	99706.41002	4.982638614	59.0155397
30 to 34	0.000445625	2.587931637	99298.56987	4.95958458	50.40799513	0.000280188	2.654152986	99594.48037	4.976429984	54.07868576
35 to 39	0.000603228	2.591747132	99077.77067	4.946718552	45.51430736	0.000409293	2.733742165	99455.1434	4.968112527	49.15047015
40 to 44	0.000724122	2.837604894	98779.68341	4.931182813	40.6433858	0.000707488	2.712700016	99251.93252	4.954564729	44.24496918
45 to 49	0.001701212	2.836338328	98423.13084	4.90303453	35.77955236	0.001123954	2.821229919	98901.61854	4.932910323	39.39118885
50 to 54	0.003425791	2.792362639	97590.11162	4.842769388	31.05888327	0.002379407	2.760747076	98347.70054	4.891220233	34.59542115
55 to 59	0.006523989	2.71531748	95933.96131	4.726296759	26.54249729	0.004066415	2.652706702	97185.46446	4.813270306	29.97250969
60 to 64	0.010628853	2.671622996	92856.53896	4.530995876	22.32544621	0.005393351	2.660669055	95232.34992	4.702359092	25.52742515
65 to 69	0.016630262	2.654417759	88056.84773	4.238511578	18.38603219	0.008579848	2.706223027	92702.5339	4.545933201	21.14596093
70 to 74	0.026541663	2.655265424	81052.54284	3.817698182	14.73119318	0.014959387	2.724462762	88817.57253	4.295261119	16.94417774
75 to 79	0.044598806	2.596088817	71067.47596	3.214479883	11.41180018	0.027509532	2.71992445	82442.11585	3.880957001	13.02971641
80 to 84	0.069136371	2.531067761	56986.37889	2.446349145	8.585553372	0.057006873	2.624214016	71876.62956	3.176334205	9.528093304
85 to 89	0.124864805	2.452449429	40666.55889	1.559962546	6.023195012	0.107022803	2.523174357	54340.08945	2.170381848	6.715702465
90 to 94	0.218941148	2.19996686	22062.26831	0.695141926	4.144774267	0.190258288	2.283781364	32300.34505	1.085913031	4.653865183
95 plus	0.316057426	3.17784784	7440.981186	0.242990347	3.17784784	0.287595514	3.512275025	12746.14849	0.466619887	3.512275025

Table 15: Poland 2017 life table, by age and sex. mx=mortality rate, ax=mean person-years lived in an age interval among those who die in that age interval, lx=number of persons left alive at age x, nLx=person-years lived between age x and x+n, ex=life expectancy at age x.										
**Age Group**	**Male**					**Female**				
	**mx**	**ax**	**lx**	**nLx**	**ex**	**mx**	**ax**	**lx**	**nLx**	**ex**
Early Neonatal	0.111050238	0.009585637	100000	0.019157675	74.02532657	0.087363267	0.009586363	100000	0.019162025	81.89135191
Late Neonatal	0.01452483	0.028763117	99787.25728	0.057387865	74.16393938	0.011317322	0.028764001	99832.59676	0.057419237	82.00947394
Post Neonatal	0.001171381	0.461560623	99703.90586	0.920056275	74.16838118	0.001074943	0.461567473	99767.61585	0.920685159	82.00533671
1 to 4	0.000168528	1.999775296	99596.13773	3.982503094	73.32485017	0.000143029	1.999809294	99668.65171	3.985605816	81.16301986
5 to 9	9.64143E-05	2.499799137	99529.02456	4.975251953	69.37294686	7.88891E-05	2.499835648	99611.64463	4.97960009	77.20832312
10 to 14	0.000128888	3.126508307	99481.05741	4.972851907	64.40519209	9.00506E-05	2.770471377	99572.36155	4.97761876	72.23779739
15 to 19	0.000484575	2.817870574	99416.96453	4.965597636	59.44469493	0.000196001	2.626520121	99527.53812	4.974063026	67.26908016
20 to 24	0.000871447	2.622774998	99176.34547	4.948565688	54.58205199	0.000209423	2.561230544	99430.04646	4.968964567	62.33245207
25 to 29	0.001004439	2.592301674	98745.10741	4.925344064	49.80891541	0.000258097	2.633803348	99325.9856	4.963268219	57.39506041
30 to 34	0.001328123	2.638125194	98250.39238	4.897158379	45.04660799	0.000376032	2.690383961	99197.88617	4.955590585	52.46576075
35 to 39	0.001906061	2.669851625	97600.00083	4.858422672	40.32914087	0.000603688	2.722877232	99011.54162	4.943781022	47.55941752
40 to 44	0.002927278	2.7112474	96673.98225	4.801531242	35.68976418	0.001026771	2.740851245	98713.09493	4.924232243	42.69493479
45 to 49	0.004992913	2.700198032	95268.50533	4.709352513	31.17613235	0.001805241	2.733214772	98207.49843	4.890363268	37.90056689
50 to 54	0.008035561	2.681736213	92917.34157	4.560911871	26.89644105	0.003091243	2.711451035	97324.69554	4.832051701	33.21946427
55 to 59	0.012806019	2.649204321	89252.82089	4.332240996	22.89033004	0.005064772	2.689751698	95831.05825	4.736139113	28.69482436
60 to 64	0.01907737	2.616905211	83705.90849	4.003331768	19.2312935	0.008021406	2.661566101	93432.48456	4.58561828	24.36221981
65 to 69	0.027576551	2.581294528	76070.46205	3.565767407	15.89863829	0.012032749	2.649013569	89754.58689	4.364289987	20.25119968
70 to 74	0.037830462	2.569665153	66240.74754	3.033290314	12.8747031	0.018352624	2.659504116	84504.09598	4.051233964	16.34458608
75 to 79	0.056873392	2.550849584	54771.46113	2.403952334	10.03279507	0.030408318	2.673899093	77071.19891	3.599089477	12.66413157
80 to 84	0.087928254	2.496344817	41108.9001	1.684858739	7.520285113	0.056858939	2.629006888	66131.94389	2.913991883	9.316586019
85 to 89	0.149117737	2.381294136	26306.8281	0.946188361	5.349034173	0.108108804	2.512478795	49573.81146	1.95369856	6.55081346
90 to 94	0.244357204	2.157925613	12208.98185	0.360363535	3.779118497	0.192855336	2.286459097	28468.29485	0.934658788	4.546481621
95 plus	0.33686026	2.968705263	3408.803838	0.101240281	2.968705263	0.290426653	3.443529035	10455.44329	0.360226137	3.443529035

Table 15: Poland 2100 life table, by age and sex. mx=mortality rate, ax=mean person-years lived in an age interval among those who die in that age interval, lx=number of persons left alive at age x, nLx=person-years lived between age x and x+n, ex=life expectancy at age x.

Age Group	Male					Female				
	mx	ax	lx	nLx	ex	mx	ax	lx	nLx	ex
Early Neonatal	0.02007542	0.009588426	100000	0.019174391	82.21709989	0.017330669	0.00958851	100000	0.019174896	86.87873717
Late Neonatal	0.003116681	0.028766264	99961.51066	0.057506946	82.22952187	0.002756789	0.028766363	99966.77154	0.057510568	86.88842522
Post Neonatal	0.000214485	0.461628599	99943.58907	0.922675479	82.18671494	0.000217745	0.461628367	99950.91846	0.922741754	86.8446658
1 to 4	3.74058E-05	1.999950126	99923.8005	3.996653034	81.27961316	3.53085E-05	1.999952922	99930.82731	3.996950852	85.93874677
5 to 9	3.27809E-05	2.499931707	99908.85168	4.995033221	77.29148027	3.58944E-05	2.499925221	99916.7158	4.995387524	81.95060839
10 to 14	5.75339E-05	3.538691115	99892.47768	4.994189107	72.3037177	5.41596E-05	2.953404492	99898.78588	4.994364088	76.96486072
15 to 19	0.000321629	2.876870443	99863.74533	4.98974268	67.32348327	0.000133024	2.638942165	99871.73857	4.992017123	71.98491887
20 to 24	0.000628504	2.621695205	99703.29786	4.977698617	62.42648951	0.000141004	2.541928191	99805.33963	4.988533708	67.03096755
25 to 29	0.000670997	2.546968843	99390.66793	4.961349837	57.61328727	0.000157242	2.587264284	99735.0116	4.984853608	62.07637137
30 to 34	0.000765379	2.573573099	99058.10728	4.94373601	52.79704207	0.000201133	2.637015723	99656.64749	4.980458365	57.12302386
35 to 39	0.000962521	2.631108467	98680.2433	4.922805836	47.98824339	0.000282802	2.693320384	99556.49693	4.974577535	52.17767595
40 to 44	0.001408165	2.69402047	98207.40991	4.894465452	43.20527095	0.000461726	2.778433544	99415.85088	4.965679087	47.24736864
45 to 49	0.002302358	2.705060674	97520.16828	4.850340829	38.48891955	0.000882343	2.783974216	99186.65742	4.949615665	42.34949899
50 to 54	0.003680084	2.700828519	96407.87769	4.779934532	33.89854272	0.001633446	2.769339729	98750.14934	4.919538394	37.52318847
55 to 59	0.005952655	2.65003357	94656.63361	4.667644998	29.47083712	0.002980214	2.737336315	97947.1177	4.864455071	32.80616288
60 to 64	0.008252209	2.637901143	91892.3557	4.507082912	25.26964089	0.005009337	2.657407655	96499.38515	4.768959755	28.25409031
65 to 69	0.012267166	2.626492489	88193.15385	4.285597182	21.21055396	0.006858238	2.629935557	94115.95352	4.630574686	23.89811352
70 to 74	0.01748924	2.625762201	82973.50882	3.984650406	17.36938277	0.009596004	2.678599794	90951.6025	4.448861826	19.63314284
75 to 79	0.027116407	2.627805263	76067.24411	3.576902333	13.69703318	0.016289057	2.753383192	86702.99368	4.183262694	15.455546
80 to 84	0.044473448	2.609408612	66528.89871	3.016105605	10.27226315	0.034352878	2.720090674	79940.12016	3.711451491	11.51359298
85 to 89	0.091022701	2.556064198	53557.55228	2.206950356	7.125560115	0.07321409	2.647383726	67442.78408	2.888912262	8.116040763
90 to 94	0.181319395	2.244427462	34301.16778	1.158190612	4.757711561	0.148672817	2.340716043	46968.92057	1.700305122	5.52002105
95 plus	0.28478533	3.52897785	14068.27452	0.508669516	3.52897785	0.248473453	4.058009326	22579.60791	0.940551097	4.058009326

Table 15: Romania 2017 life table, by age and sex. mx=mortality rate, ax=mean person-years lived in an age interval among those who die in that age interval, lx=number of persons left alive at age x, nLx=person-years lived between age x and x+n, ex=life expectancy at age x.

Age Group	Male					Female				
	mx	ax	lx	nLx	ex	mx	ax	lx	nLx	ex
Early Neonatal	0.166083269	0.009583951	100000	0.019147572	71.53581933	0.125802645	0.009585185	100000	0.019154966	79.00785046
Late Neonatal	0.02556746	0.028760071	99681.99549	0.057309125	71.7448068	0.020781316	0.028761391	99759.02744	0.057361307	79.17949112
Post Neonatal	0.003626037	0.461386248	99535.47577	0.917462214	71.79283793	0.002908893	0.461437193	99639.82547	0.918727972	79.21664668
1 to 4	0.000372014	1.999503981	99202.81698	3.965161882	71.1087495	0.000325143	1.999566476	99372.58544	3.972319741	78.5051576
5 to 9	0.000187069	2.499610272	99055.31388	4.950450203	67.2116629	0.000150179	2.499687126	99243.43009	4.960308958	74.60472156
10 to 14	0.000261168	2.794993908	98962.70871	4.945287452	62.27221901	0.00017794	2.635924835	99168.93757	4.95636193	69.65888535
15 to 19	0.000558032	2.71528979	98833.55578	4.935385609	57.34993662	0.000266687	2.594533596	99080.74464	4.950861334	64.7185382
20 to 24	0.000841407	2.620818256	98558.14754	4.918062105	52.50257446	0.000299668	2.59795718	98948.71217	4.943877019	59.80141987
25 to 29	0.001052869	2.577859523	98144.34194	4.894734665	47.71284347	0.000408748	2.613844868	98800.56094	4.935214622	54.8871796
30 to 34	0.00124586	2.65440604	97628.99699	4.867226885	42.95103952	0.000524786	2.67678908	98598.83667	4.923938847	49.99410226
35 to 39	0.001998039	2.721273772	97022.61923	4.829144787	38.20282526	0.00085804	2.724245367	98340.43832	4.907439292	45.11840233
40 to 44	0.00342292	2.735715785	96057.7644	4.765951563	33.55911446	0.001458299	2.713858523	97919.36756	4.879700049	40.30065036
45 to 49	0.006034756	2.732269615	94426.49241	4.657588572	29.0914316	0.002381736	2.723164825	97207.77961	4.834174587	35.57571449
50 to 54	0.010658249	2.686657403	91615.96885	4.470582332	24.89978286	0.004087446	2.685762819	96056.45124	4.757818875	30.9693682
55 to 59	0.016961146	2.615025477	86851.71727	4.173777821	21.11790698	0.006220813	2.659313449	94111.83182	4.63806204	26.55361841
60 to 64	0.022951012	2.578066826	79773.90787	3.778707211	17.75916148	0.009298457	2.656127676	91226.84028	4.464062527	22.30904456
65 to 69	0.031249347	2.579490286	71103.80368	3.305274947	14.61003599	0.014226422	2.680130589	87076.50707	4.214751187	18.24550801
70 to 74	0.046178201	2.562999311	60779.07176	2.73170264	11.65365937	0.024328763	2.678250811	81081.6706	3.837393584	14.39605245
75 to 79	0.068705613	2.526258373	48171.49829	2.058906766	9.033255214	0.042910395	2.649913623	71748.89957	3.258960984	10.9199674
80 to 84	0.10779122	2.458077588	34036.33971	1.336078356	6.736783655	0.080280713	2.563218351	57771.9847	2.416260619	7.920790907
85 to 89	0.172002785	2.321857594	19647.57479	0.672764463	4.872808442	0.139490577	2.414942516	38388.55257	1.41107186	5.627026666
90 to 94	0.266351079	2.112770375	8085.715013	0.228613515	3.52435481	0.227519195	2.213076596	18722.2862	0.573069396	4.003865442
95 plus	0.354266725	2.822830894	2000.596301	0.056498643	2.822830894	0.322000504	3.105844356	5694.165352	0.176958763	3.105844356

Table 15: Romania 2100 life table, by age and sex. mx=mortality rate, ax=mean person-years lived in an age interval among those who die in that age interval, lx=number of persons left alive at age x, nLx=person-years lived between age x and x+n, ex=life expectancy at age x.

Age Group	Male					Female				
	mx	ax	lx	nLx	ex	mx	ax	lx	nLx	ex
Early Neonatal	0.054514538	0.00958737	100000	0.019168063	81.05088983	0.040178409	0.00958781	100000	0.019170696	86.21818043
Late Neonatal	0.006345298	0.028765373	99895.54429	0.057463661	81.1163519	0.005003001	0.028765743	99922.99291	0.057481669	86.26531998
Post Neonatal	0.000400451	0.461615388	99859.09004	0.921816263	81.08837119	0.00040525	0.461615047	99894.24079	0.922138709	86.23257913
1 to 4	4.90441E-05	1.999934608	99822.18133	3.992495685	80.19491771	5.05567E-05	1.999932591	99856.87794	3.993871367	85.34138393
5 to 9	3.34412E-05	2.499930331	99802.60372	4.989713026	76.21026461	3.85924E-05	2.4999196	99836.69132	4.991353062	81.35823264
10 to 14	8.22326E-05	3.092565864	99785.9179	4.988489463	71.22258101	7.05755E-05	2.834744218	99817.43199	4.990092817	76.37342686
15 to 19	0.000245667	2.766673492	99744.89825	4.984497582	66.25060904	0.000136341	2.600253079	99782.2204	4.987479512	71.39936567
20 to 24	0.00038652	2.62326751	99622.46215	4.976539773	61.32836037	0.000136232	2.599505106	99714.23775	4.984069897	66.44612662
25 to 29	0.00046401	2.541693727	99430.16723	4.965858207	56.44145617	0.000189592	2.619645762	99646.35823	4.980057251	61.48948801
30 to 34	0.000493462	2.570646835	99199.85641	4.954080381	51.56600187	0.000231925	2.638264662	99551.97678	4.974870804	56.54512616
35 to 39	0.000656102	2.703404537	98955.60922	4.940373119	46.6861155	0.000340371	2.733924042	99436.63663	4.967992118	51.60743456
40 to 44	0.001169864	2.800816018	98631.98431	4.918812849	41.82899379	0.000606406	2.788907353	99267.63	4.956703111	46.6901807
45 to 49	0.002208852	2.834854516	98058.30979	4.879322258	37.05474982	0.001151367	2.793540918	98967.27259	4.935772356	41.82255944
50 to 54	0.00446133	2.770310083	96985.28288	4.801296553	32.42881657	0.00218681	2.723119233	98399.6035	4.895526529	37.04596461
55 to 59	0.007804725	2.642560537	94855.95403	4.657354331	28.08716667	0.0034328	2.648648793	97331.02836	4.827551798	32.41962654
60 to 64	0.00996455	2.603896612	91249.16899	4.456744056	24.08371438	0.00462851	2.630482685	95678.20748	4.732102183	27.93030839
65 to 69	0.01365584	2.629866733	86847.82475	4.207424199	20.16319525	0.006492928	2.672547654	93495.59927	4.605314615	23.51681575
70 to 74	0.020496796	2.636952398	81165.42267	3.873040371	16.38375595	0.010362458	2.716400104	90519.41171	4.421621174	19.19825145
75 to 79	0.031487664	2.636358348	73351.27313	3.417525696	12.84525078	0.018405198	2.739512982	85965.92001	4.127486599	15.06706538
80 to 84	0.053309861	2.587217849	62797.05986	2.790668121	9.563641386	0.036236614	2.674576866	78435.54959	3.620461532	11.2449314
85 to 89	0.103909694	2.515023931	48327.41079	1.934503931	6.650278422	0.076419471	2.633101945	65506.1255	2.782525483	7.918744201
90 to 94	0.19637884	2.232251711	28936.26038	0.948289371	4.489140262	0.153273524	2.339858896	44730.14858	1.600733159	5.390956943
95 plus	0.297459631	3.375005556	10892.76743	0.375412867	3.375005556	0.252954736	3.975861132	20816.78658	0.843803991	3.975861132

Table 15: Serbia 2017 life table, by age and sex. mx=mortality rate, ax=mean person-years lived in an age interval among those who die in that age interval, lx=number of persons left alive at age x, nLx=person-years lived between age x and x+n, ex=life expectancy at age x.										
Age Group	Male					Female				
	mx	ax	lx	nLx	ex	mx	ax	lx	nLx	ex
Early Neonatal	0.128711707	0.009585096	100000	0.019154432	73.62374853	0.094276983	0.009586152	100000	0.019160755	77.88317709
Late Neonatal	0.018624317	0.028761986	99753.4627	0.057361666	73.78650331	0.015054588	0.02876297	99819.35969	0.057405452	78.00492193
Post Neonatal	0.001532029	0.461535003	99646.6333	0.919374718	73.80804498	0.00122694	0.461556676	99732.93992	0.920300587	78.01495529
1 to 4	0.000140309	1.999812921	99505.78722	3.9791148	72.98857998	0.000126619	1.999831175	99620.02769	3.983792188	77.17957086
5 to 9	9.31049E-05	2.499806031	99449.95802	4.971340689	69.02843302	7.20799E-05	2.499849834	99569.5861	4.977582303	73.21765823
10 to 14	0.000124451	2.894882114	99403.67314	4.968881814	64.05941053	7.31117E-05	2.68494881	99533.70819	4.97584321	68.24314959
15 to 19	0.000329313	2.778335981	99341.83545	4.963460477	59.0974807	0.000137074	2.64475724	99497.32916	4.973260931	63.26711723
20 to 24	0.000565895	2.64884945	99178.38272	4.952329951	54.19027757	0.000168572	2.689999552	99429.1587	4.969522853	58.30867331
25 to 29	0.000736653	2.619298586	98898.1341	4.936249717	49.3362958	0.000291286	2.712706872	99345.38694	4.963962115	53.35556265
30 to 34	0.000993721	2.628514466	98534.50684	4.915142653	44.50865807	0.000467049	2.709468549	99200.7946	4.954739396	48.42935946
35 to 39	0.001360792	2.684653498	98046.08557	4.886907655	39.71723443	0.000763873	2.717162422	98969.38584	4.939855218	43.5362309
40 to 44	0.002224999	2.755198441	97381.09396	4.844857029	34.97004517	0.001270863	2.764445216	98592.049	4.915636549	38.69240199
45 to 49	0.004162077	2.751554613	96303.15279	4.770516108	30.33051213	0.002401723	2.737712238	97967.35348	4.871897145	33.92141962
50 to 54	0.007495194	2.717933845	94317.74823	4.63658648	25.91085046	0.004086952	2.694925008	96797.30269	4.794697618	29.29822658
55 to 59	0.012741224	2.666577364	90842.88726	4.411018705	21.79771542	0.006428386	2.672950758	94837.85182	4.672008848	24.8476886
60 to 64	0.019572025	2.613291854	85223.59808	4.071050503	18.05879442	0.009916513	2.715829668	91834.79843	4.4900493	20.57256432
65 to 69	0.027463991	2.59868484	77257.58272	3.623958891	14.6510704	0.018169391	2.670383639	87382.90376	4.191758423	16.48200341
70 to 74	0.041411464	2.612227213	67308.22145	3.062714291	11.43246495	0.028554198	2.683138572	79768.61292	3.741031669	12.79997697
75 to 79	0.072239094	2.568777888	54631.58833	2.323749576	8.479243941	0.055661977	2.651794676	69090.87534	3.055431602	9.363160776
80 to 84	0.127532548	2.454632838	37857.08494	1.429299524	6.099126788	0.113325018	2.533253177	52095.2422	2.036141293	6.552785488
85 to 89	0.19406218	2.268979577	19644.73442	0.64220562	4.480579667	0.179959757	2.311566019	29043.74591	0.979095825	4.745052752
90 to 94	0.286789645	2.069285878	7192.808779	0.195477467	3.313636942	0.269092906	2.11715874	11444.67456	0.322431431	3.492222946
95 plus	0.370144685	2.701744679	1590.641599	0.042995766	2.701744679	0.358442533	2.790113324	2777.886859	0.077572374	2.790113324

Table 15: Serbia 2100 life table, by age and sex. mx=mortality rate, ax=mean person-years lived in an age interval among those who die in that age interval, lx=number of persons left alive at age x, nLx=person-years lived between age x and x+n, ex=life expectancy at age x.

Age Group	Male					Female				
	mx	ax	lx	nLx	ex	mx	ax	lx	nLx	ex
Early Neonatal	0.018025612	0.009588489	100000	0.019174768	80.69499213	0.014775303	0.009588588	100000	0.019175366	83.57399771
Late Neonatal	0.003797531	0.028766076	99965.44555	0.057508085	80.70363133	0.003680538	0.028766108	99971.67377	0.057511861	83.5783931
Post Neonatal	0.000217585	0.461628379	99943.61248	0.922674411	80.66370678	0.000185239	0.461630677	99950.51102	0.92275186	83.53849397
1 to 4	2.96971E-05	1.999960403	99923.54664	3.996704541	79.75652365	3.09086E-05	1.999958789	99933.42407	3.997090002	82.62935455
5 to 9	2.56178E-05	2.499946629	99911.68133	4.995264226	75.76570832	2.45757E-05	2.4999488	99921.07751	4.995747161	78.6392891
10 to 14	4.77147E-05	3.106015629	99898.88853	4.994464183	70.77504442	2.79885E-05	2.760620193	99908.80981	4.995117081	73.64861052
15 to 19	0.000137021	2.831589391	99875.06618	4.992272369	65.79116551	4.95468E-05	2.646249853	99894.84118	4.994159682	68.6585119
20 to 24	0.000268858	2.71156793	99806.71181	4.987243419	60.83406121	6.24191E-05	2.787345212	99870.12026	4.992796761	63.67477482
25 to 29	0.000384898	2.643163268	99672.83343	4.979101347	55.91176477	0.000117042	2.774770354	99838.98568	4.990624398	58.69367619
30 to 34	0.000506955	2.595630053	99481.48932	4.968010005	51.01380339	0.000192222	2.755291159	99780.6169	4.986853163	53.72614744
35 to 39	0.000603012	2.656851458	99230.0337	4.954503269	46.1360174	0.000326476	2.763737798	99684.82738	4.980558217	48.77465712
40 to 44	0.000958731	2.780663516	98931.74114	4.936064402	41.26645232	0.000559544	2.910276914	99522.37756	4.970195267	43.8486299
45 to 49	0.001878809	2.816831883	98459.3082	4.902743016	36.44966357	0.001327876	2.831519026	99244.64061	4.94780515	38.96116794
50 to 54	0.003729988	2.776898526	97540.20981	4.836820252	31.7629563	0.002506424	2.726172112	98588.91271	4.901458101	34.19715724
55 to 59	0.006870754	2.673241941	95741.1044	4.711933953	27.30064993	0.004006339	2.666909473	97364.11485	4.823228164	29.5852021
60 to 64	0.009883495	2.64052787	92517.25194	4.520932329	23.14659444	0.005803639	2.705665604	95441.80192	4.709672373	25.11608228
65 to 69	0.014422519	2.630101796	88082.73375	4.25982661	19.1651081	0.010060139	2.648394006	92731.1899	4.53054632	20.75681787
70 to 74	0.021249016	2.674244211	82000.88356	3.9094631	15.37822866	0.013937888	2.731371724	88231.07557	4.278199799	16.66203498
75 to 79	0.038022412	2.655052608	73834.92453	3.395152488	11.77177495	0.027969398	2.779255544	82394.19148	3.883425621	12.63099672
80 to 84	0.068270121	2.565376279	61211.62383	2.638392281	8.648398035	0.064059618	2.684585575	71833.24998	3.144896239	9.063876667
85 to 89	0.123801036	2.455793942	43892.08554	1.689162242	6.059040213	0.117063848	2.490711204	52619.09468	2.063700682	6.386647311
90 to 94	0.217744785	2.201407001	23999.83374	0.758899839	4.164599529	0.201712065	2.262799317	29993.35622	0.989681588	4.45591994
95 plus	0.315057522	3.189198591	8180.072235	0.268680993	3.189198591	0.298108936	3.388417592	11251.72242	0.398802852	3.388417592

Table 15: Slovakia 2017 life table, by age and sex. mx=mortality rate, ax=mean person-years lived in an age interval among those who die in that age interval, lx=number of persons left alive at age x, nLx=person-years lived between age x and x+n, ex=life expectancy at age x.

Age Group	Male					Female				
	mx	ax	lx	nLx	ex	mx	ax	lx	nLx	ex
Early Neonatal	0.113513481	0.009585562	100000	0.019157223	74.12792692	0.09454676	0.009586143	100000	0.019160706	80.7126355
Late Neonatal	0.017577126	0.028762275	99782.54826	0.05738012	74.27026302	0.013474454	0.028763406	99818.84755	0.057407767	80.83991376
Post Neonatal	0.002257217	0.461483487	99681.69658	0.919390503	74.28784456	0.00188242	0.461510112	99741.4948	0.920101144	80.845051
1 to 4	0.000269056	1.999641258	99474.18398	3.976827172	73.5185752	0.000221319	1.999704908	99568.29983	3.980969694	80.06159431
5 to 9	0.000110411	2.499769978	99367.19404	4.966988627	69.59558806	0.000118615	2.499752885	99480.19797	4.972535243	76.13073186
10 to 14	0.000131099	2.97723382	99312.35609	4.964301103	64.63263919	0.000109455	2.661242047	99421.2176	4.969788722	71.17441388
15 to 19	0.00041102	2.784623183	99247.27655	4.957849468	59.67306665	0.000203588	2.675038638	99366.8212	4.965990601	66.21191617
20 to 24	0.000694792	2.596382591	99043.50071	4.943918611	54.79008516	0.000281037	2.563268127	99265.72001	4.959889505	61.27661436
25 to 29	0.000736194	2.569191063	98700.00344	4.92618457	49.97168616	0.000289658	2.652805492	99126.32991	4.952949141	56.35915597
30 to 34	0.000945624	2.663455477	98337.34422	4.906027717	45.14645923	0.000494876	2.663814646	98982.86528	4.9434283	51.43697541
35 to 39	0.001490134	2.713938773	97873.42521	4.877057974	40.34777346	0.000681484	2.691414589	98738.22975	4.9291567	46.55777932
40 to 44	0.002506019	2.723720919	97146.69481	4.829784826	35.62921115	0.001127365	2.715920961	98402.32148	4.907479265	41.70746599
45 to 49	0.004265973	2.717833752	95936.38848	4.750571914	31.04418804	0.001866109	2.720641399	97849.0847	4.871732465	36.92784036
50 to 54	0.007207676	2.702261265	93909.94113	4.619006335	26.65519967	0.003150254	2.681571566	96940.00338	4.81185774	32.24850718
55 to 59	0.011931512	2.678531612	90581.07141	4.407002197	22.53515087	0.004725024	2.691335052	95424.24043	4.719730804	27.71798453
60 to 64	0.01919941	2.628377709	85323.73384	4.08043072	18.75825432	0.007785705	2.686618565	93194.36984	4.577286526	23.31656993
65 to 69	0.027769695	2.591685295	77491.47239	3.631771845	15.38816294	0.012449372	2.67733687	89631.14412	4.355638497	19.13639072
70 to 74	0.039925661	2.583516916	67409.86403	3.074063498	12.30173432	0.020343689	2.682839601	84209.90861	4.021012479	15.19560146
75 to 79	0.06279436	2.551269783	55143.15386	2.389944091	9.463699764	0.035894664	2.691158402	76032.83682	3.510839155	11.54091492
80 to 84	0.099437334	2.484617542	40147.28113	1.606053549	7.046587366	0.073141417	2.602070029	63438.55674	2.698964944	8.297700576
85 to 89	0.162675867	2.345511973	24192.62699	0.84508562	5.057534719	0.130403712	2.441469904	43715.41334	1.639403948	5.868346392
90 to 94	0.257497552	2.131250887	10458.14685	0.300856576	3.623203275	0.217755556	2.234801295	22359.52229	0.698094249	4.144496285
95 plus	0.347300835	2.879474617	2716.927144	0.078272657	2.879474617	0.313215271	3.193061456	7173.180567	0.229217905	3.193061456

Table 15: Slovakia 2100 life table, by age and sex. mx=mortality rate, ax=mean person-years lived in an age interval among those who die in that age interval, lx=number of persons left alive at age x, nLx=person-years lived between age x and x+n, ex=life expectancy at age x.

Age Group	Male					Female				
	mx	ax	lx	nLx	ex	mx	ax	lx	nLx	ex
Early Neonatal	0.023451868	0.009588322	100000	0.01917377	82.23264515	0.021958721	0.009588368	100000	0.019174045	86.1216343
Late Neonatal	0.004431727	0.028765901	99955.0408	0.05750105	82.25050736	0.003696625	0.028766104	99957.90213	0.057503912	86.13872065
Post Neonatal	0.000608299	0.461600623	99929.56498	0.922378363	82.21399253	0.00057148	0.461603239	99936.64915	0.922459425	86.09948519
1 to 4	6.1952E-05	1.999917397	99873.47313	3.994444093	81.33659383	5.33194E-05	1.999928908	99883.94659	3.99493196	85.2213797
5 to 9	3.26843E-05	2.499931903	99848.73288	4.992028753	77.35624496	4.48805E-05	2.499906496	99862.65249	4.992572581	81.23913816
10 to 14	4.95318E-05	3.26508034	99832.41821	4.991152314	72.36844477	6.10861E-05	2.972657986	99840.25307	4.991319009	76.25676398
15 to 19	0.00021763	2.875849658	99807.70157	4.987976709	67.3855748	0.000120778	2.691825698	99809.78816	4.98909435	71.27919557
20 to 24	0.00040739	2.606070997	99699.18661	4.980061801	62.4554127	0.000183766	2.552806185	99749.54608	4.985234872	66.32051059
25 to 29	0.000404637	2.518507939	99496.42003	4.969835017	57.57676953	0.00016711	2.611323576	99657.98465	4.980895292	61.37894858
30 to 34	0.000453184	2.588716682	99295.46045	4.959336495	52.68766453	0.000262733	2.657260331	99574.82166	4.975617898	56.42784661
35 to 39	0.000611548	2.659405713	99070.9808	4.946459209	47.80051765	0.000319279	2.676359645	99444.22618	4.968493517	51.49808083
40 to 44	0.001000473	2.709822661	98768.86055	4.927081848	42.93795346	0.000523745	2.745626608	99285.71947	4.9583869	46.57551031
45 to 49	0.00171426	2.727198447	98276.91588	4.894666777	38.13810994	0.000944558	2.735057175	99026.25742	4.940728846	41.68939771
50 to 54	0.003054729	2.713733301	97439.92666	4.838106925	33.44022534	0.001668715	2.680246893	98560.1069	4.908976905	36.87181691
55 to 59	0.005247663	2.666335946	95966.30007	4.740260539	28.90891783	0.002485602	2.679219495	97742.57404	4.859041743	32.15453581
60 to 64	0.007996889	2.63206816	93487.53063	4.587668928	24.59988977	0.003943128	2.66555294	96538.28131	4.782941204	27.51828247
65 to 69	0.011671928	2.629283286	89833.5717	4.371172258	20.4883732	0.006011516	2.684669133	94658.56206	4.668096868	23.00733545
70 to 74	0.018076919	2.644407899	84756.3055	4.065698007	16.55235604	0.010152421	2.724112599	91865.03888	4.489822401	18.62013157
75 to 79	0.031057611	2.628850554	77455.48755	3.609981203	12.85638384	0.01922268	2.764039046	87334.60446	4.187770054	14.43823092
80 to 84	0.053108468	2.574984401	66396.82819	2.947760151	9.554846543	0.043045697	2.67470768	79364.58692	3.61292177	10.60135641
85 to 89	0.103720407	2.515214499	51064.29497	2.041557922	6.644900176	0.086924583	2.593120796	64126.09871	2.665624955	7.457405861
90 to 94	0.196318974	2.233317429	30478.03872	0.996881904	4.485494651	0.166470781	2.323317503	41709.72844	1.458830998	5.104140158
95 plus	0.297444918	3.372900968	11403.85749	0.391461109	3.372900968	0.265450324	3.794896337	18263.85099	0.711194792	3.794896337

Table 15: Slovenia 2017 life table, by age and sex. mx=mortality rate, ax=mean person-years lived in an age interval among those who die in that age interval, lx=number of persons left alive at age x, nLx=person-years lived between age x and x+n, ex=life expectancy at age x.

Age Group	Male					Female				
	mx	ax	lx	nLx	ex	mx	ax	lx	nLx	ex
Early Neonatal	0.043828135	0.009587698	100000	0.019170025	77.94089701	0.037707679	0.009587885	100000	0.019171149	84.28448165
Late Neonatal	0.010090849	0.02876434	99915.98172	0.057469223	77.98725141	0.009958494	0.028764376	99927.71022	0.057476188	84.32626927
Post Neonatal	0.000328745	0.461620482	99857.99105	0.921836614	77.97499137	0.000389347	0.461616177	99870.47297	0.921926049	84.31704707
1 to 4	0.000132638	1.999823149	99827.68654	3.992048387	77.07523505	8.95704E-05	1.999880573	99834.57833	3.992667849	83.423909
5 to 9	4.16506E-05	2.499913228	99774.73757	4.988217462	73.11507764	7.31286E-05	2.499847649	99798.81625	4.989028661	79.45308683
10 to 14	0.000174924	2.986154594	99753.96163	4.985941358	68.12978513	6.37903E-05	2.746802036	99762.3324	4.987399837	74.48122936
15 to 19	0.000450231	2.725955969	99666.74611	4.978240486	63.18678531	0.000148862	2.658921137	99730.51774	4.984788794	69.50410947
20 to 24	0.000665512	2.551388587	99442.61129	4.964041252	58.32302096	0.000177609	2.508788312	99656.3136	4.980612024	64.55386849
25 to 29	0.000617594	2.534027308	99112.25118	4.948076822	53.50886372	0.000156669	2.611813731	99567.85385	4.976530767	59.60897394
30 to 34	0.000771094	2.616139963	98806.66503	4.931269041	48.66647066	0.000262151	2.714844767	99489.88782	4.971516359	54.6536235
35 to 39	0.001054503	2.632573645	98426.42484	4.909066557	43.84431466	0.000428044	2.738839746	99359.56019	4.963174253	49.72172236
40 to 44	0.001454732	2.711017651	97908.77742	4.879192679	39.06211951	0.000755394	2.746168279	99147.11812	4.948929978	44.82234251
45 to 49	0.002559134	2.774159838	97199.01706	4.832425655	34.32744706	0.001328196	2.726935686	98773.28865	4.923799223	39.98151012
50 to 54	0.004925743	2.713499134	95962.41728	4.744686887	29.7338645	0.002225051	2.681545241	98119.33847	4.880789667	35.22968252
55 to 59	0.007898968	2.681765833	93625.55458	4.597107575	25.40797443	0.003323796	2.669083733	97033.40756	4.814374098	30.59375431
60 to 64	0.012594042	2.651434093	89994.91138	4.37050248	21.32443051	0.005056081	2.672615903	95433.36951	4.716179126	26.06169565
65 to 69	0.018873519	2.626900608	84492.00404	4.043555475	17.54017323	0.007825261	2.691895037	93049.19632	4.569939148	21.6606712
70 to 74	0.028192453	2.617541404	76863.1553	3.601386751	14.01998084	0.013071543	2.728659744	89473.96773	4.344748193	17.41821899
75 to 79	0.044258548	2.626207104	66715.53213	3.018854833	10.75417199	0.024724465	2.732156717	83796.97199	3.967505539	13.41288136
80 to 84	0.082083237	2.557289732	53365.29207	2.22300398	7.787943587	0.05013504	2.664291649	73993.88735	3.31218128	9.827468331
85 to 89	0.14248587	2.399441372	35137.23961	1.282281295	5.503277808	0.098451663	2.54621492	57404.54478	2.312311623	6.897871031
90 to 94	0.237788654	2.170758118	16886.54089	0.50495325	3.861730588	0.181524047	2.307099007	34667.50528	1.164747469	4.754339947
95 plus	0.331591929	3.015950218	4889.978498	0.147568924	3.015950218	0.279871318	3.573733311	13549.96767	0.484684375	3.573733311

Table 15: Slovenia 2100 life table, by age and sex. mx=mortality rate, ax=mean person-years lived in an age interval among those who die in that age interval, lx=number of persons left alive at age x, nLx=person-years lived between age x and x+n, ex=life expectancy at age x.

Age Group	Male					Female				
	mx	ax	lx	nLx	ex	mx	ax	lx	nLx	ex
Early Neonatal	0.007948649	0.009588797	100000	0.019176621	84.04054662	0.008314508	0.009588786	100000	0.019176553	88.49984601
Late Neonatal	0.00263337	0.028766397	99984.75756	0.05752112	84.0342131	0.003623169	0.028766124	99984.05617	0.057519079	88.49477603
Post Neonatal	5.64307E-05	0.461639827	99969.61098	0.922983049	83.98947272	6.90857E-05	0.461638927	99963.21791	0.922918632	88.45568804
1 to 4	3.54119E-05	1.999952783	99964.40265	3.998292934	83.07054161	2.30083E-05	1.999969319	99956.84204	3.998089707	87.53801407
5 to 9	1.46469E-05	2.499969549	99950.24451	4.997329282	79.08203041	3.20492E-05	2.499933224	99947.64352	4.996981902	83.54588349
10 to 14	8.79766E-05	3.171058636	99942.92795	4.996269093	74.08750964	3.76062E-05	2.873397401	99931.63512	4.996147542	78.55881685
15 to 19	0.000295017	2.687729982	99899.00606	4.991539202	69.11823653	8.42195E-05	2.6676179	99912.86033	4.99464369	73.57303212
20 to 24	0.000445411	2.552920989	99751.94668	4.982161364	64.21416969	0.000112842	2.498248023	99870.82705	4.992127553	68.60272499
25 to 29	0.000422568	2.546053326	99530.74612	4.971310333	59.34843581	7.11131E-05	2.582052997	99814.57888	4.989849382	63.63977603
30 to 34	0.000453382	2.541486762	99321.69143	4.960541736	54.46595913	0.000119138	2.799792071	99779.15073	4.987562049	58.66134151
35 to 39	0.000502684	2.560993382	99097.84695	4.948807643	49.58169694	0.000178032	2.702988483	99719.81555	4.983924022	53.69435385
40 to 44	0.000595781	2.662396704	98850.44496	4.935598039	44.69774657	0.000303097	2.745227861	99631.15898	4.97811143	48.73946429
45 to 49	0.001004764	2.764625668	98557.99034	4.916823883	39.82096719	0.000580759	2.794680224	99480.44478	4.967486811	43.80854399
50 to 54	0.002187517	2.697563898	98066.63043	4.878696332	35.00425376	0.001112227	2.72250595	99192.46347	4.946900715	38.92653501
55 to 59	0.003474283	2.667516978	97007.03599	4.811334535	30.35213617	0.001816193	2.661872671	98643.36139	4.911220175	34.12638273
60 to 64	0.00550917	2.643405655	95348.86296	4.706616815	25.82745145	0.002789175	2.661401345	97753.25976	4.855878675	29.41081805
65 to 69	0.008510374	2.649730843	92777.00071	4.548447776	21.46265396	0.004422123	2.650579247	96403.0694	4.770479715	24.78256889
70 to 74	0.013793272	2.658486339	88949.31144	4.309535879	17.26269108	0.006688542	2.722279204	94303.27397	4.644306698	20.2710162
75 to 79	0.024477472	2.684265287	83074.03496	3.933990375	13.28602393	0.013715311	2.760379401	91216.99952	4.425293369	15.85943444
80 to 84	0.052836502	2.603650724	73607.73368	3.277834357	9.638587362	0.031720449	2.68806675	85197.84384	3.971877938	11.77522487
85 to 89	0.102976555	2.518497301	56863.14794	2.284742122	6.702370756	0.068995376	2.663831877	72780.31261	3.142588491	8.30842317
90 to 94	0.19505523	2.231598063	34368.46561	1.132347087	4.51982537	0.143296805	2.347074671	51583.22492	1.881815841	5.639931653
95 plus	0.296298489	3.392628009	13155.64533	0.458647041	3.392628009	0.243361552	4.133728262	25288.53651	1.06494604	4.133728262

Table 15: Eastern Europe 2017 life table, by age and sex. mx=mortality rate, ax=mean person-years lived in an age interval among those who die in that age interval, lx=number of persons left alive at age x, nLx=person-years lived between age x and x+n, ex=life expectancy at age x.

Age Group	Male					Female				
	mx	ax	lx	nLx	ex	mx	ax	lx	nLx	ex
Early Neonatal	0.180730006	0.009583502	100000	0.019144884	66.49318728	0.139947827	0.009584752	100000	0.019152369	77.2840364
Late Neonatal	0.018471723	0.028762028	99653.99484	0.057304719	66.70484436	0.015168933	0.028762939	99731.96685	0.057355004	77.47253576
Post Neonatal	0.002353	0.461476682	99548.14357	0.918118073	66.71820776	0.001916844	0.461507666	99644.96561	0.919196048	77.48261862
1 to 4	0.000401181	1.999465092	99332.11086	3.970098131	65.93901868	0.00031793	1.999576093	99468.77033	3.97622196	76.69576327
5 to 9	0.000235502	2.49950937	99172.83834	4.955723637	62.04170644	0.000175718	2.499633922	99342.35448	4.964936338	72.79081647
10 to 14	0.000303717	2.993321391	99056.13006	4.949789806	57.11185933	0.000213732	2.740005243	99255.1118	4.960359587	67.85260061
15 to 19	0.00095781	2.859367427	98905.79658	4.935171167	52.19411704	0.000422779	2.661948913	99149.09327	4.952559181	62.92222393
20 to 24	0.001971025	2.713786753	98433.1014	4.899576657	47.43102729	0.000544032	2.672176141	98939.70956	4.940728481	58.04974939
25 to 29	0.003024469	2.681481518	97467.38367	4.839433694	42.87408138	0.000876432	2.700604345	98670.91816	4.923623496	53.2006019
30 to 34	0.004719834	2.64703159	96003.71411	4.747462357	38.48684504	0.001397651	2.673423997	98239.39629	4.89604917	48.42242217
35 to 39	0.006598739	2.586315387	93762.99653	4.614651427	34.34331749	0.002062584	2.621432681	97555.10036	4.853941669	43.74332126
40 to 44	0.007868445	2.581396635	90717.92344	4.451187927	30.40926322	0.00264285	2.604171571	96553.93645	4.797321153	39.16970404
45 to 49	0.010372228	2.606424893	87215.56067	4.25513822	26.52673801	0.00346031	2.616998998	95286.08118	4.725339411	34.65622737
50 to 54	0.014384019	2.606991179	82802.09216	4.002341815	22.80169953	0.004720707	2.636962288	93650.97534	4.630890465	30.21560799
55 to 59	0.020047191	2.612636347	77045.21764	3.676316115	19.31064095	0.006825515	2.653717333	91464.8813	4.50116034	25.87475115
60 to 64	0.02999797	2.573397059	69675.39565	3.247388119	16.07683546	0.010318373	2.662599701	88392.63197	4.315549539	21.68182751
65 to 69	0.039534187	2.552344394	59934.14558	2.73232058	13.27157948	0.016188591	2.673640386	83939.73413	4.044665242	17.69075694
70 to 74	0.057959598	2.53049431	49132.52136	2.14904591	10.62818249	0.027236757	2.669575119	77392.09913	3.638654251	13.96123407
75 to 79	0.083363514	2.470394747	36677.39619	1.514516338	8.378104859	0.048301546	2.615923031	67481.88431	3.025687124	10.61947741
80 to 84	0.115287091	2.394255036	24052.74608	0.92483333	6.479062392	0.082033004	2.538997904	52868.00993	2.199403519	7.831852455
85 to 89	0.179559314	2.303233688	13391.48603	0.451137941	4.7313309	0.141558365	2.409025396	34826.70647	1.274071213	5.573812801
90 to 94	0.273430575	2.097804338	5291.478923	0.147518457	3.448536764	0.229723471	2.208118286	16792.32144	0.511549776	3.972908965
95 plus	0.359799861	2.779330031	1258.097517	0.03496798	2.779330031	0.323976145	3.086664551	5041.502327	0.155621063	3.086664551

Table 15: Eastern Europe 2100 life table, by age and sex. mx=mortality rate, ax=mean person-years lived in an age interval among those who die in that age interval, lx=number of persons left alive at age x, nLx=person-years lived between age x and x+n, ex=life expectancy at age x.

Age Group	Male					Female				
	mx	ax	lx	nLx	ex	mx	ax	lx	nLx	ex
Early Neonatal	0.044178732	0.009587687	100000	0.019169961	77.72256896	0.03439909	0.009587987	100000	0.019171758	84.95700065
Late Neonatal	0.004732343	0.028765818	99915.32489	0.057477706	77.76880462	0.004437974	0.028765899	99934.05776	0.057488968	84.99341521
Post Neonatal	0.000614212	0.461600203	99888.1297	0.921993372	77.73226497	0.000541352	0.461605379	99908.54753	0.922212846	84.95743921
1 to 4	9.84744E-05	1.999868701	99831.50932	3.992474139	76.85257944	8.69183E-05	1.999884109	99858.63211	3.993651086	84.07622043
5 to 9	5.5393E-05	2.499884598	99792.20054	4.988919191	72.88194726	6.10829E-05	2.499872744	99823.92444	4.990434208	80.10468497
10 to 14	0.000101357	3.188995788	99764.56851	4.987297535	67.90130988	9.95444E-05	2.807401521	99793.44557	4.988575131	75.12826612
15 to 19	0.000418953	2.876737157	99714.028	4.981231268	62.9338245	0.000199925	2.684371576	99743.79697	4.984891011	70.16401742
20 to 24	0.000899497	2.775005764	99505.51519	4.96524528	58.05410835	0.000284932	2.765925474	99644.16557	4.979029023	65.23077344
25 to 29	0.001576109	2.766768329	99060.21399	4.935436244	53.28930554	0.000554105	2.797800128	99502.38236	4.969019725	60.31843506
30 to 34	0.002767636	2.650101151	98287.75987	4.882716381	48.66505105	0.001040851	2.67038963	99227.39498	4.94934764	55.47528378
35 to 39	0.003478553	2.544628931	96954.0203	4.807482824	44.26847658	0.001370534	2.573262303	98713.47175	4.919421993	50.74540053
40 to 44	0.003811366	2.551226135	95315.23454	4.72311442	39.95883181	0.001590774	2.585050761	98042.01786	4.883483061	46.06898625
45 to 49	0.004734169	2.624155604	93575.29444	4.628733177	35.63042365	0.002051194	2.615443328	97271.41349	4.840080204	41.40555801
50 to 54	0.006613451	2.671774766	91489.29906	4.507795938	31.36541552	0.002718144	2.641630192	96289.26668	4.784070484	36.7927873
55 to 59	0.009653358	2.662613315	88673.15626	4.339877617	27.26689929	0.003815978	2.654225389	95008.3263	4.708572477	32.24363163
60 to 64	0.013923392	2.603983874	84713.40587	4.105603338	23.41177307	0.005248739	2.637984003	93243.46294	4.605739231	27.79483228
65 to 69	0.017075795	2.603250224	79329.99624	3.817599804	19.82866944	0.007230937	2.663868263	90869.20449	4.46902273	23.44382424
70 to 74	0.024595928	2.607820092	73155.96425	3.463850789	16.29783959	0.011134007	2.706095716	87701.51943	4.277752495	19.18820948
75 to 79	0.035541269	2.564465534	65080.67115	3.007563739	13.02288784	0.019436961	2.714408604	83045.03766	3.979382536	15.10804705
80 to 84	0.048198198	2.491450534	54974.47908	2.468312646	9.985924971	0.035593781	2.64509914	75514.84024	3.491742206	11.34218074
85 to 89	0.095952841	2.540497232	43784.68115	1.792625963	6.951043722	0.075192533	2.638666715	63473.27611	2.709811263	7.995924995
90 to 94	0.186981791	2.238921705	27635.85241	0.927692404	4.659911427	0.151494681	2.340134527	43852.44813	1.579555817	5.441439683
95 plus	0.289534459	3.472931562	11155.59702	0.400355221	3.472931562	0.251217091	4.008001265	20780.35207	0.854544759	4.008001265

Table 15: Belarus 2017 life table, by age and sex. mx=mortality rate, ax=mean person-years lived in an age interval among those who die in that age interval, lx=number of persons left alive at age x, nLx=person-years lived between age x and x+n, ex=life expectancy at age x.

Age Group	Male					Female				
	mx	ax	lx	nLx	ex	mx	ax	lx	nLx	ex
Early Neonatal	0.154167175	0.009584316	100000	0.019149759	68.93393259	0.113552685	0.009585561	100000	0.019157215	78.78481896
Late Neonatal	0.016031092	0.028762701	99704.78352	0.057337951	69.11881831	0.012506065	0.028763674	99782.46912	0.057388444	78.9373658
Post Neonatal	0.001863304	0.46151147	99612.87165	0.918922773	69.12503385	0.001436329	0.461541801	99710.70217	0.920006491	78.93662624
1 to 4	0.000388437	1.999482083	99441.66632	3.974578467	68.31997045	0.000284505	1.999620659	99578.56752	3.980877272	78.1174741
5 to 9	0.000225329	2.499530565	99287.2977	4.961569624	64.42309608	0.000151931	2.499683478	99465.31786	4.971377472	74.20414663
10 to 14	0.000160314	2.847326936	99175.50836	4.957064031	59.49290214	0.000142816	2.679511389	99389.79062	4.967843122	69.25863793
15 to 19	0.00049277	2.887777777	99096.04361	4.94965043	54.53832466	0.000275292	2.596183863	99318.84346	4.962658238	64.30619388
20 to 24	0.00108196	2.686109508	98852.14312	4.930264041	49.6657334	0.000270507	2.634025312	99182.22666	4.955939581	59.39117973
25 to 29	0.001470416	2.668472933	98318.71293	4.899140003	44.92055447	0.000450545	2.732660027	99048.1665	4.947354502	54.46798282
30 to 34	0.002298981	2.688854967	97598.34561	4.854126866	40.2323309	0.00077648	2.718388352	98825.26906	4.932525224	49.584641
35 to 39	0.003621025	2.666581175	96482.41631	4.783703517	35.66643764	0.001272522	2.664622637	98442.27471	4.907529686	44.76692638
40 to 44	0.005345483	2.667514052	94750.29647	4.679177665	31.2695004	0.001799554	2.651260945	97817.79513	4.870304703	40.03562283
45 to 49	0.008273451	2.656020909	92249.23223	4.52472387	27.04468311	0.0026167	2.651463727	96941.38964	4.817465101	35.37348975
50 to 54	0.012336885	2.633241869	88506.17769	4.29978237	23.07575662	0.00378016	2.672553195	95680.86982	4.742322426	30.80444118
55 to 59	0.017876112	2.637731402	83202.57491	3.991609666	19.37843757	0.005929029	2.656318457	93888.32922	4.630083909	26.34137491
60 to 64	0.028116201	2.609981441	76069.01387	3.564033107	15.94799098	0.008622029	2.682854377	91143.4333	4.46792185	22.05445686
65 to 69	0.041418097	2.563454644	66051.83688	2.999986628	12.97055316	0.014503238	2.707459488	87291.76653	4.224168262	17.90895007
70 to 74	0.058992868	2.527541108	53632.59359	2.340487	10.38052409	0.026006179	2.679245061	81166.7805	3.827419095	14.05574349
75 to 79	0.087360921	2.472314822	39835.00062	1.631765386	8.101307143	0.045705979	2.63901922	71216.9122	3.214193788	10.64484044
80 to 84	0.122980516	2.393615798	25593.12779	0.969323361	6.235758158	0.084311455	2.550434493	56535.07284	2.34322632	7.723966379
85 to 89	0.188014986	2.283058363	13685.69049	0.453094828	4.582408322	0.144560372	2.400732432	36796.00657	1.337690146	5.500590871
90 to 94	0.281248582	2.081137091	5175.461842	0.142169277	3.368508331	0.232882775	2.200914032	17477.30899	0.529235173	3.930400887
95 plus	0.365866762	2.733332411	1180.069069	0.032271974	2.733332411	0.32678825	3.060368034	5163.488384	0.158130938	3.060368034

Table 15: Belarus 2100 life table, by age and sex. mx=mortality rate, ax=mean person-years lived in an age interval among those who die in that age interval, lx=number of persons left alive at age x, nLx=person-years lived between age x and x+n, ex=life expectancy at age x.

Age Group	Male					Female				
	mx	ax	lx	nLx	ex	mx	ax	lx	nLx	ex
Early Neonatal	0.028914656	0.009588155	100000	0.019172766	78.45760022	0.022354348	0.009588356	100000	0.019173972	86.34777193
Late Neonatal	0.003403322	0.028766184	99944.56702	0.057496725	78.48188278	0.002836033	0.028766341	99957.13973	0.057504896	86.3655345
Post Neonatal	0.000338069	0.46161982	99925.00076	0.922451252	78.43970008	0.000280711	0.461623894	99940.832	0.922621821	86.32207343
1 to 4	7.88646E-05	1.999894847	99893.8183	3.995122592	77.54072461	6.37181E-05	1.999915043	99914.93459	3.996088148	85.42099993
5 to 9	5.48776E-05	2.499885672	99862.31312	4.992430724	73.56454595	5.00253E-05	2.499895781	99889.47403	4.993849241	81.44222817
10 to 14	5.18529E-05	3.123807401	99834.91749	4.991231604	68.5840225	6.69906E-05	2.788517257	99864.4973	4.992465088	76.46188577
15 to 19	0.000203882	2.987001767	99809.03934	4.988334641	63.60100542	0.000125342	2.625519059	99831.05923	4.990067674	71.48650055
20 to 24	0.000500209	2.732581062	99707.36394	4.979670594	58.66207504	0.000143327	2.753676373	99768.54262	4.986789107	66.52930688
25 to 29	0.000746601	2.718075758	99458.46362	4.964455495	53.79989829	0.000267907	2.802669217	99697.10253	4.981905968	61.57475526
30 to 34	0.001324403	2.687780711	99088.44296	4.9391748	48.9870382	0.000518572	2.737792408	99563.71702	4.972267588	56.65301345
35 to 39	0.00186561	2.641863445	98436.79289	4.900351285	44.28816912	0.000777512	2.635732042	99306.1714	4.956188352	51.7918778
40 to 44	0.002693087	2.655352138	97527.49731	4.845763724	39.67022788	0.001014472	2.651494233	98921.32426	4.934267466	46.98191852
45 to 49	0.003771611	2.655473217	96234.50919	4.769802919	35.16070705	0.001466142	2.642793249	98421.84438	4.904140209	42.20504748
50 to 54	0.005554963	2.694458464	94458.4284	4.663360214	30.76382063	0.001995024	2.670310416	97704.95641	4.862695565	37.49240318
55 to 59	0.008753648	2.687472649	91913.39043	4.505265153	26.5320011	0.003129298	2.651557799	96738.55218	4.801594182	32.83564163
60 to 64	0.013550375	2.641598809	88053.6829	4.268396039	22.5610301	0.004110003	2.659927914	95246.03006	4.716917466	28.30243794
65 to 69	0.01879847	2.612390157	82415.54666	3.947281904	18.90672288	0.006114471	2.692184957	93323.3057	4.601464293	23.82464878
70 to 74	0.026428671	2.607932148	75206.44696	3.542704339	15.46329377	0.009877908	2.723015336	90536.10348	4.4277804	19.46975703
75 to 79	0.039776577	2.572315287	66159.41727	3.025503754	12.22546335	0.017445737	2.750238059	86214.6593	4.149463219	15.3026646
80 to 84	0.057161999	2.535480127	54547.8907	2.402717728	9.30468914	0.034929034	2.721259782	79082.82162	3.667362818	11.4275667
85 to 89	0.108947837	2.499566143	41371.17801	1.641905041	6.489527294	0.074221576	2.642814868	66545.4018	2.84392349	8.050984796
90 to 94	0.201931027	2.224976453	24287.98542	0.789732662	4.400533549	0.150149522	2.340736991	46090.50112	1.662867515	5.477101122
95 plus	0.302070607	3.324271161	8947.533282	0.305274267	3.324271161	0.249915923	4.030660169	21943.91715	0.90637345	4.030660169

Table 15: Estonia 2017 life table, by age and sex. mx=mortality rate, ax=mean person-years lived in an age interval among those who die in that age interval, lx=number of persons left alive at age x, nLx=person-years lived between age x and x+n, ex=life expectancy at age x.

Age Group	Male					Female				
	mx	ax	lx	nLx	ex	mx	ax	lx	nLx	ex
Early Neonatal	0.046456126	0.009587617	100000	0.019169542	73.64733162	0.049330239	0.009587529	100000	0.019169013	82.16526563
Late Neonatal	0.006859248	0.028765231	99910.94624	0.057471669	73.69378707	0.005824304	0.028765517	99905.43926	0.057470212	82.22384884
Post Neonatal	0.001058837	0.461568618	99871.52555	0.921650909	73.665329	0.000838237	0.461584289	99871.96725	0.921748833	82.19386283
1 to 4	0.000219501	1.999707332	99773.93968	3.989206102	72.81363984	0.000180479	1.999759361	99794.70402	3.990347648	81.333856
5 to 9	0.000152626	2.49968203	99686.37878	4.982417629	68.8758447	8.53338E-05	2.499822221	99722.68735	4.985070815	77.39114866
10 to 14	0.000146607	3.006174208	99610.33612	4.979061469	63.92651909	0.000127729	2.788358394	99680.14829	4.982600304	72.42310938
15 to 19	0.00050976	2.880047186	99537.34085	4.971494942	58.97117451	0.000262826	2.618240092	99616.50662	4.977709852	67.46757557
20 to 24	0.001081416	2.679827306	99283.91685	4.951771377	54.11418346	0.000277872	2.607184999	99485.68033	4.970979285	62.55278206
25 to 29	0.001453978	2.630641238	98748.44204	4.920471951	49.39275154	0.000407212	2.691067902	99347.5532	4.962711943	57.63604364
30 to 34	0.002017379	2.58527805	98033.06365	4.877895087	44.73357499	0.00065374	2.613356812	99145.47228	4.949552457	52.74790678
35 to 39	0.002318883	2.612017685	97049.12053	4.825740716	40.16034554	0.000767658	2.612675148	98821.91804	4.932058136	47.91185178
40 to 44	0.003333861	2.675550459	95930.31072	4.759642233	35.59759935	0.001076211	2.695458456	98443.34144	4.909989146	43.08579777
45 to 49	0.005271606	2.666320912	94343.99917	4.659896218	31.15026058	0.001791491	2.711356334	97914.99545	4.875760202	38.30342236
50 to 54	0.007862309	2.669303931	91888.63612	4.511801632	26.91013983	0.002933169	2.689340738	97041.67139	4.819426134	33.62326872
55 to 59	0.012431154	2.668088061	88343.76323	4.292847269	22.88135079	0.004560199	2.66929399	95628.43044	4.731153035	29.07976516
60 to 64	0.019798747	2.616310392	83012.40913	3.96378031	19.17789826	0.006882794	2.668196495	93471.78399	4.599805011	24.68824531
65 to 69	0.027573922	2.576163199	75174.91551	3.523662577	15.90313236	0.010700004	2.667725025	90307.65952	4.405535182	20.45867929
70 to 74	0.038052464	2.564951109	65476.90841	2.996867927	12.87650627	0.016906616	2.686767928	85597.92978	4.11901457	16.43623027
75 to 79	0.055762824	2.554782249	54103.26485	2.381580206	10.04544496	0.029776116	2.695202682	78643.98969	3.680131808	12.65061514
80 to 84	0.089085171	2.506631385	40870.7317	1.673453726	7.475189858	0.057820576	2.627554858	67710.29979	2.978194647	9.257515846
85 to 89	0.150566484	2.377522534	26026.548	0.934128247	5.318875351	0.109418651	2.508333574	50544.56555	1.987473438	6.511468435
90 to 94	0.245749183	2.15505169	12019.11715	0.354205776	3.763003271	0.194310058	2.283364628	28879.81172	0.94637156	4.52341446
95 plus	0.337962123	2.95948041	3341.97543	0.099114109	2.95948041	0.291756361	3.429171507	10556.52383	0.362992392	3.429171507

Table 15: Estonia 2100 life table, by age and sex. mx=mortality rate, ax=mean person-years lived in an age interval among those who die in that age interval, lx=number of persons left alive at age x, nLx=person-years lived between age x and x+n, ex=life expectancy at age x.

Age Group	Male					Female				
	mx	ax	lx	nLx	ex	mx	ax	lx	nLx	ex
Early Neonatal	0.007802149	0.009588802	100000	0.019176648	83.10623492	0.010587274	0.009588717	100000	0.019176135	88.36621846
Late Neonatal	0.001253966	0.028766778	99985.03902	0.057523564	83.09951594	0.00147953	0.028766715	99979.69928	0.057520119	88.3649789
Post Neonatal	0.000168516	0.461631865	99977.8264	0.92301114	83.04800956	0.000141468	0.461633786	99971.1902	0.922961398	88.31497937
1 to 4	3.79031E-05	1.999949462	99962.27282	3.998187835	82.13757227	3.18459E-05	1.999957538	99958.13388	3.998070722	87.40315705
5 to 9	3.91964E-05	2.499918321	99947.11942	4.996866397	78.14972003	1.9492E-05	2.499959463	99945.40258	4.997026711	83.41403307
10 to 14	5.23706E-05	3.028199896	99927.53873	4.995809009	73.16447699	5.65735E-05	2.984836197	99935.66711	4.996151927	78.42187925
15 to 19	0.000217248	2.79567965	99901.4043	4.992587456	68.18285373	0.000144554	2.582233541	99907.44525	4.993625655	73.44322418
20 to 24	0.00049696	2.708251137	99793.01726	4.983805927	63.25284295	0.000160652	2.663167544	99835.31135	4.989853139	68.49410083
25 to 29	0.000784905	2.609714175	99545.93194	4.967890494	58.40028035	0.000275849	2.637762376	99755.31517	4.984470412	63.54640151
30 to 34	0.001036481	2.525390276	99157.63386	4.945240294	53.61538245	0.000404024	2.557668247	99618.11434	4.975962463	58.62965745
35 to 39	0.00099982	2.51059213	98648.7212	4.920371841	48.87536034	0.000377891	2.561566591	99417.7479	4.966297007	53.74089769
40 to 44	0.001327395	2.646116396	98161.709	4.892665847	44.10159732	0.000501481	2.648595555	99230.6312	4.95565703	48.83585961
45 to 49	0.002076272	2.650155545	97521.59624	4.852352636	39.36930026	0.000851682	2.674113615	98983.03977	4.939313253	43.94924501
50 to 54	0.003111235	2.650012672	96529.61648	4.791577904	34.73781764	0.001474459	2.644274326	98564.35432	4.911157234	39.12031691
55 to 59	0.004983673	2.670497105	95066.28876	4.698848787	30.21877178	0.002378843	2.637311694	97844.45416	4.864737806	34.38156955
60 to 64	0.008190347	2.603704877	92775.75376	4.55089286	25.88184393	0.003418468	2.615686309	96698.94232	4.79589215	29.74749155
65 to 69	0.011440259	2.573430365	89130.40656	4.339171281	21.81408675	0.005013315	2.599891596	95074.25196	4.697655268	25.20196286
70 to 74	0.015187301	2.593162166	84327.34722	4.071637593	17.89321086	0.007412878	2.651643416	92744.24024	4.558594528	20.75924385
75 to 79	0.024869725	2.593072974	78350.87006	3.703492934	14.0549024	0.013815859	2.661447632	89417.15265	4.333020935	16.42009683
80 to 84	0.042460534	2.545882298	69492.34676	3.161986722	10.51992964	0.028327037	2.62019886	83535.80768	3.920077897	12.37360966
85 to 89	0.087308682	2.568959549	56768.47506	2.366778396	7.310912273	0.06253035	2.694260093	72810.6666	3.199230728	8.781713151
90 to 94	0.176335998	2.242735073	37384.97628	1.280736215	4.867363506	0.133329551	2.341335809	53774.14288	2.012963259	5.960472262
95 plus	0.280475915	3.591946049	16011.88109	0.595623144	3.591946049	0.233538518	4.338722929	28344.36043	1.276802684	4.338722929

Table 15: Latvia 2017 life table, by age and sex. mx=mortality rate, ax=mean person-years lived in an age interval among those who die in that age interval, lx=number of persons left alive at age x, nLx=person-years lived between age x and x+n, ex=life expectancy at age x.										
**Age Group**	**Male**					**Female**				
	**mx**	**ax**	**lx**	**nLx**	**ex**	**mx**	**ax**	**lx**	**nLx**	**ex**
Early Neonatal	0.113459699	0.009585564	100000	0.019157232	70.12452401	0.078906838	0.009586623	100000	0.019163579	79.93442778
Late Neonatal	0.010710948	0.028764169	99782.64704	0.05739151	70.25805573	0.007926582	0.028764937	99848.78857	0.057434151	80.03628386
Post Neonatal	0.001689529	0.461523814	99721.17814	0.919995639	70.24380822	0.001310955	0.461550708	99803.26451	0.920913818	80.01524421
1 to 4	0.000237657	1.999683124	99565.75212	3.980737676	69.42945031	0.000201753	1.999730996	99682.54286	3.985693232	79.18830414
5 to 9	0.000186869	2.499610689	99471.14688	4.971234626	65.49358098	0.000142767	2.49970257	99602.12971	4.978329464	75.25062112
10 to 14	0.000178847	3.072886123	99378.25265	4.967201201	60.55246416	0.000154415	2.585019279	99531.05732	4.974698234	70.30257167
15 to 19	0.000680743	2.753458405	99289.41732	4.956890848	55.60386716	0.000206514	2.665540272	99454.24118	4.970316452	65.35484975
20 to 24	0.001012178	2.690603714	98951.98627	4.936061063	50.78389526	0.000319596	2.656417797	99351.59825	4.963862408	60.41955651
25 to 29	0.001619641	2.688678667	98452.3869	4.904260979	46.02765912	0.000447932	2.703236085	99192.95809	4.954551106	55.51184095
30 to 34	0.00251081	2.671402765	97658.12363	4.854527853	41.37972568	0.00075969	2.662853191	98971.03549	4.939782756	50.63011053
35 to 39	0.003761768	2.63998602	96439.38041	4.779547038	36.86828715	0.001048869	2.702732031	98595.78739	4.917939649	45.81243209
40 to 44	0.005194022	2.634897176	94641.7961	4.674683291	32.51748829	0.00179533	2.679610374	98080.01648	4.883656426	41.03878215
45 to 49	0.007437756	2.660173455	92214.59255	4.531897762	28.30288737	0.002629898	2.660660245	97203.38312	4.830454866	36.38422297
50 to 54	0.011605803	2.64717537	88845.69236	4.324284832	24.27383354	0.003902735	2.665143474	95933.32317	4.753363389	31.83006962
55 to 59	0.017163343	2.629911161	83830.98196	4.027861865	20.56592463	0.00590508	2.665856239	94078.82991	4.640012934	27.40419717
60 to 64	0.025741843	2.596047272	76925.45406	3.622417177	17.17458411	0.009023044	2.65057276	91340.18027	4.472262511	23.14494393
65 to 69	0.036081829	2.555884106	67614.28101	3.107225954	14.18132885	0.013365142	2.653669394	87307.60105	4.232776996	19.09036525
70 to 74	0.049005197	2.533815136	56425.32903	2.517805453	11.48701064	0.021099869	2.672115955	81656.32214	3.891940364	15.22661267
75 to 79	0.070434853	2.51114086	44121.01853	1.878040717	8.986530168	0.036774321	2.671080903	73457.86293	3.383750594	11.6266155
80 to 84	0.107487005	2.452899971	30941.61984	1.215711521	6.751340748	0.070596599	2.588617851	61045.42986	2.60951621	8.44759018
85 to 89	0.171548784	2.323106932	17929.42578	0.615186017	4.883268054	0.126854212	2.452440291	42686.199	1.614663165	5.97156806
90 to 94	0.265903426	2.113672774	7416.942008	0.210131129	3.529929747	0.213848666	2.243167755	22283.99374	0.70201068	4.205002761
95 plus	0.353907874	2.826015447	1846.028313	0.052272627	2.826015447	0.309660472	3.230694262	7326.014937	0.237334797	3.230694262

Table 15: Latvia 2100 life table, by age and sex. mx=mortality rate, ax=mean person-years lived in an age interval among those who die in that age interval, lx=number of persons left alive at age x, nLx=person-years lived between age x and x+n, ex=life expectancy at age x.

Age Group	Male					Female				
	mx	ax	lx	nLx	ex	mx	ax	lx	nLx	ex
Early Neonatal	0.030811822	0.009588097	100000	0.019172418	79.68171324	0.018841315	0.009588464	100000	0.019174618	86.13025761
Late Neonatal	0.002815254	0.028766347	99940.9341	0.057495609	79.70963652	0.00210865	0.028766542	99963.87787	0.057509976	86.14214762
Post Neonatal	0.000361207	0.461618176	99924.75445	0.92243915	79.66506085	0.000296989	0.461622738	99951.75379	0.922715724	86.09502409
1 to 4	4.86654E-05	1.999935113	99891.44418	3.995268938	78.76824417	4.69553E-05	1.999937393	99924.35438	3.996598874	85.19519293
5 to 9	4.43631E-05	2.499907577	99872.00347	4.993046444	74.78320877	4.89721E-05	2.49989798	99905.58997	4.994668301	81.21080552
10 to 14	5.59589E-05	3.249479088	99849.85572	4.991961998	69.79918547	7.21314E-05	2.774269426	99881.14585	4.993195474	76.22999442
15 to 19	0.000279813	2.703599866	99821.93133	4.987854425	64.81780705	9.6338E-05	2.763322223	99845.17145	4.991144567	71.25638514
20 to 24	0.000400863	2.702243395	99682.43059	4.979460351	59.90263625	0.000171207	2.767441271	99797.1389	4.987873542	66.28906013
25 to 29	0.000717082	2.700725866	99483.13005	4.965879203	55.01321512	0.00025145	2.717793934	99711.83685	4.982711322	61.34291764
30 to 34	0.001224234	2.636723533	99128.08223	4.942098858	50.19388781	0.000430553	2.655319589	99586.68273	4.974280557	56.41575977
35 to 39	0.001792354	2.572325202	98526.26689	4.905241433	45.47475061	0.00055764	2.690317886	99372.87891	4.962247021	51.52961016
40 to 44	0.002261422	2.615729738	97657.59135	4.856962497	40.84177681	0.000964519	2.668723638	99096.83872	4.943776446	46.66294643
45 to 49	0.003229643	2.679288657	96583.18978	4.793676655	36.25154507	0.001419952	2.706343336	98622.19854	4.915030045	41.86833491
50 to 54	0.005372265	2.695727283	95082.72123	4.696783094	31.75378756	0.00230174	2.679137999	97930.31048	4.870647019	37.1337401
55 to 59	0.008489539	2.674245141	92661.15402	4.545970874	27.48269465	0.003521172	2.697288031	96822.19018	4.802184437	32.51261288
60 to 64	0.012897686	2.614523099	89002.46546	4.322510993	23.4663229	0.005309505	2.639036305	95163.82645	4.699872278	28.01267165
65 to 69	0.017467154	2.574507241	83712.68255	4.024722962	19.74889425	0.007034702	2.627114184	92718.72531	4.561496053	23.6639411
70 to 74	0.02318382	2.593094595	77126.44091	3.664763211	16.20020974	0.010517821	2.701402035	89589.27022	4.376208967	19.38327295
75 to 79	0.034489499	2.580438846	69247.31055	3.211805527	12.76257931	0.01806178	2.729991844	85155.70626	4.094865198	15.23817041
80 to 84	0.054840412	2.518863548	58924.73663	2.620895004	9.588449223	0.036430025	2.653674735	78028.82288	3.607442817	11.3743863
85 to 89	0.104799556	2.514304525	45810.48037	1.852833456	6.690015851	0.075997896	2.63814878	65539.19731	2.803415171	8.03101477
90 to 94	0.196463925	2.225256664	28148.99431	0.936236285	4.516520971	0.151797971	2.333586902	45538.92177	1.649971065	5.471473851
95 plus	0.297335523	3.390819182	11095.66189	0.394633859	3.390819182	0.251348049	4.027866043	21981.65066	0.923228142	4.027866043

Table 15: Lithuania 2017 life table, by age and sex. mx=mortality rate, ax=mean person-years lived in an age interval among those who die in that age interval, lx=number of persons left alive at age x, nLx=person-years lived between age x and x+n, ex=life expectancy at age x.

Age Group	Male					Female				
	mx	ax	lx	nLx	ex	mx	ax	lx	nLx	ex
Early Neonatal	0.09262025	0.009586202	100000	0.01916106	69.65509722	0.065143721	0.009587044	100000	0.019166107	80.39426149
Late Neonatal	0.013337293	0.028763444	99822.53132	0.057410112	69.75973554	0.011425443	0.028763972	99875.14559	0.05744353	80.47556979
Post Neonatal	0.001756258	0.461519074	99745.96338	0.920195938	69.75572918	0.001848724	0.461512505	99809.51479	0.920742929	80.47093361
1 to 4	0.000255732	1.999659024	99584.35801	3.981337736	68.94489496	0.000214263	1.999714316	99639.29906	3.983864537	79.68432881
5 to 9	7.99036E-05	2.499833534	99482.54631	4.973133842	65.01340972	0.000112811	2.499764977	99553.94016	4.976293443	75.75093591
10 to 14	0.000268661	2.904953866	99442.81002	4.969343363	60.03838986	0.000109273	2.69605427	99497.80285	4.973638014	70.79226513
15 to 19	0.000603311	2.790029887	99309.30491	4.958853786	55.11518695	0.000215903	2.617076415	99443.45459	4.969616072	65.8294764
20 to 24	0.001113953	2.716966808	99010.13372	4.937948473	50.27324311	0.00023104	2.691164561	99336.15971	4.964160037	60.89773673
25 to 29	0.001778418	2.700830018	98460.07488	4.902956356	45.53882996	0.000428904	2.76777953	99221.46827	4.95632826	55.964999
30 to 34	0.002868989	2.663904617	97588.13997	4.846923283	40.92144882	0.000785081	2.683490916	99008.89157	4.941458235	51.07917843
35 to 39	0.004127507	2.654907514	96197.60846	4.763773267	36.47425673	0.001127307	2.642343083	98620.95332	4.917976953	46.26948175
40 to 44	0.006143105	2.636738272	94231.47876	4.644158019	32.1795998	0.001569294	2.710609097	98066.56232	4.885774854	41.51601147
45 to 49	0.008573927	2.61775039	91378.82583	4.477501319	28.10147218	0.002749608	2.670392261	97299.87405	4.834030464	36.82165857
50 to 54	0.011795392	2.631749674	87540.5019	4.258103243	24.21838579	0.003898133	2.644829266	95970.78965	4.754889219	32.29442682
55 to 59	0.017712962	2.634250226	82519.14859	3.960063476	20.5314967	0.005635464	2.660257821	94117.44305	4.644637162	27.87805741
60 to 64	0.026912069	2.58876881	75507.07404	3.545385582	17.19314166	0.008622176	2.661061167	91500.32611	4.484592836	23.59907089
65 to 69	0.036605009	2.54707613	65969.92394	3.026874928	14.30424248	0.013193384	2.646725027	87634.37175	4.2498088	19.52241291
70 to 74	0.049234612	2.524602984	54896.7456	2.446868362	11.67594956	0.019947978	2.657467422	82029.06769	3.918427331	15.67520732
75 to 79	0.068312956	2.501092639	42859.66443	1.830795109	9.246974421	0.033439821	2.678780723	74216.16281	3.44367216	12.04529464
80 to 84	0.100008179	2.467076374	30366.59275	1.211771316	7.02431794	0.065183458	2.621045754	62708.50589	2.71482213	8.764130927
85 to 89	0.163138211	2.344320535	18262.9456	0.637360318	5.048079632	0.119741863	2.474326392	45028.98849	1.729111333	6.177048352
90 to 94	0.257939891	2.130335925	7876.861604	0.226415107	3.618146251	0.20603407	2.259941668	24346.79538	0.778392711	4.325416036
95 plus	0.347650198	2.876578476	2041.665928	0.058763226	2.876578476	0.30255622	3.305573744	8325.426192	0.275414375	3.305573744

Table 15: Lithuania 2100 life table, by age and sex. mx=mortality rate, ax=mean person-years lived in an age interval among those who die in that age interval, lx=number of persons left alive at age x, nLx=person-years lived between age x and x+n, ex=life expectancy at age x.

Age Group	Male					Female				
	mx	ax	lx	nLx	ex	mx	ax	lx	nLx	ex
Early Neonatal	0.01719837	0.009588514	100000	0.01917492	79.3135293	0.012921564	0.009588645	100000	0.019175706	86.07653762
Late Neonatal	0.002815124	0.028766347	99967.02724	0.057510619	79.32051624	0.002872294	0.028766331	99975.22411	0.05751524	86.07869649
Post Neonatal	0.000373718	0.461617287	99950.84074	0.922674614	79.27581425	0.000442233	0.46161242	99958.70609	0.922718042	86.03539669
1 to 4	5.59228E-05	1.999925436	99916.36358	3.996207607	78.37973037	5.28264E-05	1.999929565	99917.90654	3.996294085	85.14704108
5 to 9	2.21563E-05	2.499953845	99894.01782	4.994424327	74.39681738	4.49156E-05	2.499906425	99896.79865	4.994279179	81.16461244
10 to 14	0.000126823	3.059458201	99882.95735	4.992879506	69.40473835	5.35859E-05	2.918413256	99874.37009	4.993105643	76.18225238
15 to 19	0.000354432	2.848961133	99819.64848	4.987074499	64.44679589	0.000118393	2.620840097	99847.63712	4.990965165	71.20185552
20 to 24	0.000653601	2.732172355	99643.06381	4.974694096	59.55344645	0.00012991	2.764501109	99788.57662	4.987946775	66.24208934
25 to 29	0.001015696	2.726363727	99318.65583	4.95438492	54.73335503	0.00026739	2.836603619	99723.82641	4.983200584	61.28300647
30 to 34	0.001649423	2.67231539	98817.82004	4.921949705	49.9888644	0.000515341	2.633765465	99590.7559	4.973449182	56.36041959
35 to 39	0.002289911	2.618617846	98011.43956	4.874340116	45.36724852	0.00064314	2.618384762	99334.87329	4.959079241	51.49697413
40 to 44	0.003136826	2.635086857	96907.7674	4.809978926	40.83927658	0.000844314	2.72989206	99017.0711	4.941264383	46.6510909
45 to 49	0.004021204	2.623058875	95429.856	4.727206816	36.41619878	0.001580784	2.669317783	98601.35812	4.911993491	41.83314959
50 to 54	0.005694131	2.714015592	93573.96262	4.619154363	32.07583089	0.002364203	2.667313701	97828.99408	4.864605143	37.13543391
55 to 59	0.008805083	2.683607302	91037.674	4.462912629	27.89114957	0.003613483	2.657405416	96689.09337	4.793988927	32.5312902
60 to 64	0.012918799	2.622461875	87253.60708	4.235497417	23.97852065	0.005345855	2.635754559	94976.69553	4.68992669	28.05908999
65 to 69	0.016693072	2.580130003	81952.32729	3.942112351	20.35130632	0.007520661	2.609505819	92506.70219	4.54454067	23.7265626
70 to 74	0.021897629	2.58107846	75543.86669	3.592660437	16.85633331	0.010316829	2.646956291	89142.73213	4.353004616	19.51470751
75 to 79	0.029635525	2.592031734	67940.56192	3.177032356	13.45963827	0.016917188	2.715889606	84743.86167	4.082090336	15.38211072
80 to 84	0.045747035	2.590463927	58814.17221	2.66045358	10.16060629	0.035138488	2.668949988	77992.56959	3.612661262	11.47090031
85 to 89	0.092877523	2.550031267	47187.9549	1.939698678	7.0521066	0.074139728	2.644622766	65734.62	2.816301952	8.095661988
90 to 94	0.183553549	2.243175662	30047.46023	1.011737666	4.715752615	0.149629453	2.337784433	45821.21548	1.661193389	5.509589851
95 plus	0.28667822	3.504913779	12223.35408	0.439769152	3.504913779	0.249333627	4.051681202	22133.91332	0.927237919	4.051681202

Table 15: Moldova 2017 life table, by age and sex. mx=mortality rate, ax=mean person-years lived in an age interval among those who die in that age interval, lx=number of persons left alive at age x, nLx=person-years lived between age x and x+n, ex=life expectancy at age x.

Age Group	Male					Female				
	mx	ax	lx	nLx	ex	mx	ax	lx	nLx	ex
Early Neonatal	0.516579119	0.009573208	100000	0.019083408	68.17557756	0.396851683	0.009576878	100000	0.01910529	76.83821752
Late Neonatal	0.056988166	0.028751403	99014.36341	0.056873882	68.83484156	0.047111613	0.028754128	99241.8572	0.057020755	77.40588893
Post Neonatal	0.002650284	0.461455564	98690.26557	0.910081548	69.00326893	0.002682248	0.461453293	98973.27269	0.912677675	77.55835104
1 to 4	0.000671556	1.999104592	98449.16258	3.932684026	68.24788643	0.000260065	1.999653247	98728.52238	3.947088024	76.82622133
5 to 9	0.000301333	2.499372223	98185.15937	4.905562289	64.42607897	0.000243225	2.499493281	98625.89692	4.928297853	72.90410009
10 to 14	0.000278143	2.776862981	98037.36951	4.898838512	59.51945222	0.000213765	2.552567754	98506.04157	4.922726503	67.98977308
15 to 19	0.000671722	2.741077848	97901.12821	4.887640209	54.59842536	0.000297525	2.590956688	98400.81685	4.916517021	63.05974993
20 to 24	0.001060514	2.654002904	97572.82546	4.86653361	49.772904	0.000344501	2.584987858	98254.54105	4.908643292	58.14976803
25 to 29	0.001467728	2.635850602	97056.72787	4.836055754	45.02344227	0.000439404	2.650819239	98085.44113	4.899214979	53.24555609
30 to 34	0.002041679	2.696292085	96346.93247	4.794794907	40.33569426	0.000665051	2.697627204	97870.17122	4.886027235	48.35683296
35 to 39	0.003450218	2.692787053	95367.99706	4.730741693	35.7220266	0.001076052	2.721182884	97545.22965	4.865331088	43.50891891
40 to 44	0.005377241	2.700089288	93735.80073	4.629536581	31.29710145	0.00182312	2.718948513	97021.69737	4.83099452	38.72898457
45 to 49	0.009003202	2.669751399	91246.42617	4.46857425	27.07720347	0.003035437	2.70698962	96140.95774	4.773820994	34.05883132
50 to 54	0.013650786	2.63879115	87223.39361	4.224993654	23.20284531	0.004957055	2.688055558	94691.9171	4.680951098	29.5385448
55 to 59	0.020241673	2.620286599	81456.21648	3.885652877	19.65867756	0.007810842	2.687991147	92371.59851	4.536656115	25.21293761
60 to 64	0.030051749	2.580508836	73591.55098	3.430190836	16.47944923	0.012797096	2.649664	88828.21816	4.311731578	21.11136637
65 to 69	0.041270959	2.524878036	63284.2024	2.870975844	13.74317026	0.018824318	2.636957755	83310.7646	3.9881496	17.33391874
70 to 74	0.051449415	2.499053449	51436.97644	2.278697104	11.32707884	0.029500455	2.639171636	75804.01982	3.543448198	13.78922132
75 to 79	0.06973943	2.504973815	39715.36895	1.691513524	8.93280526	0.049631932	2.59810657	65352.17103	2.919628331	10.57239702
80 to 84	0.110288025	2.438885623	27921.67781	1.088663345	6.648280731	0.081791515	2.505440042	50864.58155	2.112358143	7.843801216
85 to 89	0.174590398	2.315407667	15918.14011	0.541956624	4.823296236	0.140956783	2.410738171	33592.16489	1.230612904	5.589172886
90 to 94	0.268787361	2.10764114	6458.330524	0.181692748	3.497840713	0.229083424	2.209561516	16251.09509	0.49574862	3.981841028
95 plus	0.356175756	2.807626515	1575.532032	0.044240335	2.807626515	0.323403055	3.092197289	4897.387419	0.151467705	3.092197289

Table 15: Moldova 2100 life table, by age and sex. mx=mortality rate, ax=mean person-years lived in an age interval among those who die in that age interval, lx=number of persons left alive at age x, nLx=person-years lived between age x and x+n, ex=life expectancy at age x.

Age Group	Male					Female				
	mx	ax	lx	nLx	ex	mx	ax	lx	nLx	ex
Early Neonatal	0.136608094	0.009584854	100000	0.019153005	79.26310771	0.108905753	0.009585703	100000	0.019158076	87.01321941
Late Neonatal	0.013832832	0.028763308	99738.71286	0.057361102	79.44962992	0.014240194	0.028763195	99791.47565	0.057390771	87.17486043
Post Neonatal	0.000333098	0.461620173	99659.41931	0.920001719	79.45504686	0.000502502	0.461608139	99709.7889	0.920394717	87.18853574
1 to 4	0.000103322	1.999862238	99628.78961	3.984328423	78.55600306	3.5218E-05	1.999953043	99663.55247	3.986261441	86.30532722
5 to 9	4.93568E-05	2.499897168	99587.63508	4.978767278	74.58763841	6.61504E-05	2.499862187	99649.52001	4.981652544	82.31713045
10 to 14	8.28751E-05	3.210425697	99563.06001	4.977359572	69.60588951	8.34606E-05	2.689627316	99616.58431	4.979853598	77.34331853
15 to 19	0.000292845	2.748202818	99521.81648	4.972818761	64.63364426	0.000126901	2.636900644	99575.04344	4.977254372	72.37426339
20 to 24	0.000459032	2.697256131	99376.22078	4.963507746	59.72208416	0.000161223	2.620953447	99511.91363	4.973686003	67.41801969
25 to 29	0.000684584	2.678876931	99148.59939	4.949531138	54.84891944	0.000218184	2.688257647	99431.75897	4.96908141	62.46982175
30 to 34	0.001026571	2.705269152	98810.19986	4.928909094	50.02333885	0.000357246	2.676255302	99323.41881	4.962046537	57.53423966
35 to 39	0.00174577	2.685183986	98305.18134	4.89559082	45.26069082	0.000513273	2.688796294	99146.32082	4.951445322	52.63084842
40 to 44	0.002739508	2.704273696	97453.7348	4.842489048	40.62213803	0.000821796	2.72708834	98892.51861	4.935443475	47.75689965
45 to 49	0.004729268	2.738455748	96136.70303	4.75621193	36.12488802	0.001439216	2.755484748	98487.73933	4.908347376	42.93850028
50 to 54	0.008174415	2.685252019	93921.49848	4.609836499	31.88382466	0.002373003	2.699863038	97784.16434	4.862663599	38.22077909
55 to 59	0.011986535	2.613882203	90238.00929	4.389241391	28.03486492	0.003672352	2.687725081	96635.48874	4.791211226	33.63469032
60 to 64	0.015767721	2.55723587	85125.16373	4.103768666	24.51688244	0.005689651	2.632172696	94889.22324	4.681755015	29.1926153
65 to 69	0.018739848	2.505167214	78897.35626	3.777179599	21.21237493	0.007273554	2.593207376	92255.70294	4.534297523	24.93812902
70 to 74	0.019254825	2.52832626	72188.97098	3.454354549	17.93758842	0.009263945	2.671232431	89006.14438	4.357784156	20.74236433
75 to 79	0.025573728	2.598123318	65929.66445	3.118495001	14.40738846	0.015738485	2.691756599	85051.98058	4.106480663	16.57097146
80 to 84	0.038705305	2.571717669	58550.71516	2.696785822	10.925194	0.025872096	2.633906512	78763.52651	3.718125552	12.66839022
85 to 89	0.081311738	2.588944075	49070.78505	2.084327743	7.59741955	0.058524648	2.710937054	69484.72974	3.078529801	8.997980051
90 to 94	0.168456253	2.239478098	33755.36817	1.182538061	5.038856254	0.127890707	2.344552858	52264.4212	1.973587364	6.099722186
95 plus	0.273713126	3.690429799	15443.67971	0.600550748	3.690429799	0.228301485	4.427098149	28170.12074	1.286768872	4.427098149

Table 15: Russian Federation 2017 life table, by age and sex. mx=mortality rate, ax=mean person-years lived in an age interval among those who die in that age interval, lx=number of persons left alive at age x, nLx=person-years lived between age x and x+n, ex=life expectancy at age x.

Age Group	Male					Female				
	mx	ax	lx	nLx	ex	mx	ax	lx	nLx	ex
Early Neonatal	0.173907135	0.009583711	100000	0.019146136	66.7372013	0.135989285	0.009584873	100000	0.019153096	77.26875536
Late Neonatal	0.017255774	0.028762363	99667.03512	0.057314222	66.94094513	0.014208141	0.028763204	99739.53845	0.057360944	77.45133308
Post Neonatal	0.002151783	0.461490977	99568.13505	0.918387727	66.94987419	0.00176423	0.461518508	99658.03924	0.9193814	77.45711412
1 to 4	0.000371962	1.99950405	99370.51807	3.971865224	66.15881136	0.000302399	1.999596801	99495.83925	3.977427546	76.6593461
5 to 9	0.000229078	2.499522753	99222.77977	4.95829885	62.25434182	0.000165166	2.499655904	99375.56229	4.966726998	72.74970888
10 to 14	0.00029896	2.969440251	99109.19589	4.952453381	57.32282372	0.000208513	2.729796201	99293.52892	4.962327442	67.80774738
15 to 19	0.000905537	2.881792027	98961.13743	4.938584098	52.40414311	0.0003959	2.667874743	99190.05771	4.954928058	62.87563397
20 to 24	0.001973231	2.726366274	98513.93033	4.903696544	47.62895005	0.000529045	2.69969932	98993.89196	4.943678325	57.99494075
25 to 29	0.003094498	2.69005661	97546.31771	4.842699727	43.07435927	0.000907092	2.717929371	98732.34893	4.926419532	53.14141757
30 to 34	0.004917886	2.644828875	96047.74613	4.747400773	38.70444579	0.001488572	2.672420164	98285.47773	4.897305868	48.37067479
35 to 39	0.00677151	2.574617159	93713.03071	4.609940293	34.60280966	0.002164152	2.61235062	97556.47897	4.852748667	43.71215631
40 to 44	0.007766598	2.57008264	90591.41025	4.445671181	30.7064372	0.002701932	2.596985265	96506.27141	4.79418599	39.15941199
45 to 49	0.01006754	2.606063469	87138.64585	4.254397222	26.82129916	0.003501737	2.617495952	95210.91696	4.721157744	34.65684393
50 to 54	0.014054913	2.60905632	82855.53159	4.008087445	23.0730682	0.004816484	2.636476982	93557.69485	4.625231816	30.22299493
55 to 59	0.019606464	2.614308419	77222.22998	3.688579403	19.56589478	0.006927012	2.651619588	91329.96506	4.493403129	25.89588349
60 to 64	0.029395136	2.571142425	69990.28078	3.266312415	16.31746146	0.01043535	2.655843779	88217.39022	4.305547291	21.71600558
65 to 69	0.038048929	2.554585953	60388.99521	2.762422109	13.50299598	0.016042008	2.670006069	83724.42193	4.035388235	17.73883929
70 to 74	0.056433288	2.535100956	49878.39892	2.189377476	10.81009788	0.02689438	2.670565533	77250.893	3.634829622	14.0015813
75 to 79	0.080820095	2.474078626	37523.2306	1.558091787	8.53480156	0.04779282	2.619376035	67475.35108	3.02912859	10.6431642
80 to 84	0.111792053	2.390933928	24931.00017	0.965072391	6.596018958	0.081968292	2.535868919	52998.53248	2.204641754	7.834908237
85 to 89	0.175619434	2.312861998	14142.54313	0.480417602	4.803895091	0.141448949	2.40933007	34927.87019	1.278063035	5.576513407
90 to 94	0.269752934	2.105602023	5705.678991	0.16020387	3.487446692	0.229608003	2.208380955	16850.25573	0.513426302	3.97447729
95 plus	0.356931045	2.801664052	1384.211506	0.03878143	2.801664052	0.323873206	3.087635061	5061.844614	0.156293977	3.087635061

Table 15: Russian Federation 2100 life table, by age and sex. mx=mortality rate, ax=mean person-years lived in an age interval among those who die in that age interval, lx=number of persons left alive at age x, nLx=person-years lived between age x and x+n, ex=life expectancy at age x.

Age Group	Male					Female				
	mx	ax	lx	nLx	ex	mx	ax	lx	nLx	ex
Early Neonatal	0.043192835	0.009587717	100000	0.019170143	77.62040234	0.034121212	0.009587995	100000	0.019171809	84.71901216
Late Neonatal	0.004724389	0.02876582	99917.21875	0.057478809	77.66481842	0.00440385	0.028765908	99934.59234	0.057489333	84.75454649
Post Neonatal	0.000613919	0.461600224	99890.06989	0.922011414	77.62812717	0.000529678	0.461606208	99909.27944	0.922224583	84.71825592
1 to 4	9.43676E-05	1.999874176	99833.47758	3.992585681	76.74821278	8.37539E-05	1.999888328	99860.44275	3.993748795	83.83591775
5 to 9	5.57753E-05	2.499883802	99795.8092	4.989094864	72.77625562	6.06363E-05	2.499873674	99826.99913	4.990593526	79.86322069
10 to 14	0.000102071	3.178373615	99767.98678	4.987460909	67.79567055	0.000101995	2.782191497	99796.74357	4.98870588	74.88649757
15 to 19	0.000403174	2.877450589	99717.09097	4.981609204	62.8282302	0.000196005	2.680228605	99745.87313	4.985043974	69.92290877
20 to 24	0.00091228	2.779002944	99516.39599	4.965683074	57.94320397	0.000287716	2.779541313	99648.19581	4.979226003	64.98782977
25 to 29	0.001613464	2.79286931	99064.84643	4.935360354	53.17800076	0.00057912	2.812565046	99505.04488	4.968910605	60.07537433
30 to 34	0.002903021	2.654432423	98275.20455	4.88067579	48.55527129	0.00110785	2.669715664	99217.77454	4.948098832	55.23741317
35 to 39	0.003614417	2.532027236	96881.09593	4.802355189	44.17647351	0.001442245	2.563295413	98671.36626	4.916458871	50.52155932
40 to 44	0.003805028	2.528364262	95188.39093	4.717140607	39.8807464	0.001646044	2.573623565	97966.3463	4.879073848	45.85757528
45 to 49	0.004680142	2.630632047	93470.07456	4.625038633	35.5340926	0.002095027	2.620429391	97172.23075	4.834751192	41.20023158
50 to 54	0.006672374	2.691823103	91445.88544	4.506556743	31.23616822	0.002803217	2.653761019	96174.68838	4.7776534	36.58907119
55 to 59	0.009677452	2.675732834	88665.01232	4.340811455	27.11767738	0.003963053	2.663678988	94863.03024	4.700056866	32.04587884
60 to 64	0.014081969	2.61132867	84757.07699	4.108814801	23.23911869	0.005455515	2.63817442	93044.47931	4.593944891	27.60888947
65 to 69	0.017262011	2.621217285	79411.946	3.82338754	19.63528682	0.007441853	2.664073775	90595.64029	4.453811015	23.27304224
70 to 74	0.025628444	2.615418531	73281.67957	3.465430115	16.082137	0.01148567	2.708415446	87364.97688	4.258808668	19.02681974
75 to 79	0.037172636	2.566535742	64990.98803	2.997365154	12.83584673	0.020206607	2.717448437	82614.25695	3.953762855	14.95873072
80 to 84	0.050734842	2.466710496	54647.19196	2.442869224	9.835779946	0.037232054	2.626629336	74906.23881	3.45259134	11.21577513
85 to 89	0.099189289	2.53090481	43197.40693	1.762417874	6.858392168	0.077548239	2.630312282	62595.29774	2.664098329	7.911473113
90 to 94	0.190410289	2.233414314	27063.92413	0.906686978	4.609628335	0.154266372	2.334866224	42970.92774	1.545001318	5.391453506
95 plus	0.292350527	3.44415574	10877.93767	0.390397787	3.44415574	0.253802299	3.976727875	20290.55504	0.83578623	3.976727875

Table 15: Ukraine 2017 life table, by age and sex. mx=mortality rate, ax=mean person-years lived in an age interval among those who die in that age interval, lx=number of persons left alive at age x, nLx=person-years lived between age x and x+n, ex=life expectancy at age x.										
Age Group	Male					Female				
	mx	ax	lx	nLx	ex	mx	ax	lx	nLx	ex
Early Neonatal	0.199006491	0.009582942	100000	0.019141532	64.66783425	0.151221696	0.009584406	100000	0.0191503	76.6212935
Late Neonatal	0.021665673	0.028761147	99619.0775	0.057279381	64.89589076	0.017808502	0.028762211	99710.40762	0.057338253	76.82461494
Post Neonatal	0.003350115	0.461405849	99494.98811	0.917205747	64.91925377	0.002641907	0.461456159	99608.30104	0.918550447	76.84580137
1 to 4	0.000520267	1.999306311	99187.72253	3.963383518	64.19564458	0.000408447	1.999455403	99365.63373	3.971380268	76.10905767
5 to 9	0.000269523	2.499438494	98981.52617	4.945743128	60.32521099	0.000222614	2.499536222	99203.42461	4.957411759	72.2302403
10 to 14	0.0003673	3.090434077	98848.22889	4.938947525	55.40319022	0.000260001	2.792006513	99093.06623	4.951810723	67.30789853
15 to 19	0.001316255	2.812522967	98666.82312	4.919177705	50.49936009	0.000588671	2.656460748	98964.3188	4.941399111	62.39182147
20 to 24	0.002365051	2.683207235	98019.33802	4.87425929	45.81428164	0.000705107	2.608810114	98673.43387	4.925367509	57.56788639
25 to 29	0.003457546	2.652818035	96866.56745	4.804339566	41.32742589	0.000962959	2.637947578	98326.14527	4.905150394	52.76196061
30 to 34	0.005040322	2.645519228	95205.48876	4.704447979	37.0020145	0.00135337	2.669283969	97853.80571	4.877306363	48.00385082
35 to 39	0.007271974	2.60621028	92834.40085	4.562307962	32.8792771	0.002086241	2.642662789	97193.73982	4.83590491	43.31165107
40 to 44	0.009280538	2.591393655	89516.96752	4.377998522	29.00084665	0.002827977	2.610587594	96184.89207	4.776966737	38.73808807
45 to 49	0.012289982	2.593816654	85454.47813	4.150021844	25.25599717	0.003671418	2.60116078	94834.05219	4.700308701	34.25255683
50 to 54	0.016456162	2.591884145	80355.10187	3.864643538	21.69380815	0.004754228	2.624668588	93108.50185	4.603443421	29.83899214
55 to 59	0.022519622	2.599370622	73997.08723	3.510152106	18.33482101	0.006782727	2.654671714	90920.13396	4.474830522	25.49385579
60 to 64	0.033117699	2.568486139	66095.08194	3.058560116	15.21594035	0.010350385	2.682661241	87885.37105	4.291355393	21.28232275
65 to 69	0.044537977	2.542524366	55970.08626	2.522556147	12.50395757	0.017273178	2.684705283	83444.41286	4.011816112	17.2719785
70 to 74	0.064423843	2.515665813	44741.24361	1.928608248	10.00446835	0.029580479	2.665765948	76516.46828	3.578804779	13.59245993
75 to 79	0.093203908	2.450235531	32325.2557	1.306147787	7.882046083	0.052436593	2.597346502	65934.34999	2.928033475	10.34592957
80 to 84	0.125430652	2.384665105	20162.44597	0.759306083	6.161164076	0.084880665	2.537625174	50589.93079	2.092503249	7.696444743
85 to 89	0.190539588	2.277131688	10647.94029	0.350650922	4.539255157	0.145171575	2.399028938	32843.11381	1.192361681	5.485479346
90 to 94	0.28356866	2.076178488	3972.407411	0.10862638	3.345278147	0.233528064	2.199447467	15548.61981	0.470196316	3.921623532
95 plus	0.367661009	2.719966986	894.0853672	0.02432922	2.719966986	0.327363624	3.054936016	4576.860209	0.13990308	3.054936016

Table 15: Ukraine 2100 life table, by age and sex. mx=mortality rate, ax=mean person-years lived in an age interval among those who die in that age interval, lx=number of persons left alive at age x, nLx=person-years lived between age x and x+n, ex=life expectancy at age x.

Age Group	Male					Female				
	mx	ax	lx	nLx	ex	mx	ax	lx	nLx	ex
Early Neonatal	0.054327258	0.009587376	100000	0.019168097	77.65014054	0.040286849	0.009587806	100000	0.019170676	85.62300146
Late Neonatal	0.005067849	0.028765725	99895.89174	0.057465975	77.71162908	0.004956964	0.028765756	99922.78299	0.057481625	85.66949531
Post Neonatal	0.000832524	0.461584694	99866.78686	0.921703618	77.67656304	0.000780604	0.461588383	99894.29645	0.921979602	85.63629271
1 to 4	0.00014285	1.999809533	99790.09889	3.990464339	76.8124237	0.000128591	1.999828546	99822.37005	3.991868534	84.77375068
5 to 9	5.97649E-05	2.49987549	99733.12663	4.985911402	72.85508491	7.37776E-05	2.499846296	99771.06292	4.987633757	80.81611009
10 to 14	0.000122246	3.070764648	99703.33146	4.983955103	67.87607049	0.000110784	2.91392853	99734.29134	4.985491733	75.84470743
15 to 19	0.000618719	2.909844901	99642.43228	4.975084855	62.91566957	0.000270634	2.732362472	99679.14053	4.980793047	70.88464647
20 to 24	0.00105575	2.7964481	99335.92112	4.954818024	58.09130154	0.000351838	2.760128172	99544.62824	4.97316807	65.97531274
25 to 29	0.001847338	2.695910653	98816.59202	4.919780972	53.36403287	0.0005773	2.723752004	99370.12293	4.961924495	61.08463833
30 to 34	0.002893905	2.631974435	97918.98952	4.862926535	48.80130715	0.000943852	2.677184442	99084.63209	4.943338193	56.25017065
35 to 39	0.00383955	2.570049631	96545.80927	4.784395857	44.42259912	0.001348802	2.610290741	98620.61199	4.915253193	51.49854969
40 to 44	0.004997027	2.611030795	94784.39783	4.685991679	40.16687001	0.001668899	2.618612033	97962.94079	4.878732551	46.82269871
45 to 49	0.006234782	2.60532168	92602.40751	4.565950403	36.02956897	0.002184315	2.589287954	97156.7735	4.832532958	42.18558768
50 to 54	0.00760529	2.635348713	89971.9919	4.423234325	32.009838	0.002598183	2.593982145	96111.85342	4.77598283	37.61202903
55 to 59	0.010764389	2.639759004	86856.68317	4.240898534	28.07120031	0.003367956	2.620254556	94885.90467	4.70685677	33.05904897
60 to 64	0.014613882	2.587238067	82619.94502	3.997763763	24.37523326	0.004595238	2.643384639	93320.29261	4.616393726	28.56279257
65 to 69	0.01740617	2.533728493	77155.04504	3.709413919	20.92010513	0.006682083	2.672650823	91227.3242	4.492127298	24.15003401
70 to 74	0.022252984	2.595342397	71127.21539	3.388727538	17.45523145	0.010415003	2.699617182	88277.5365	4.311854385	19.85994236
75 to 79	0.031968006	2.550799709	64243.51316	2.994608158	14.06192752	0.017808236	2.69246989	83880.90605	4.031402023	15.75253862
80 to 84	0.04136479	2.584551563	55405.07085	2.541767437	10.90889474	0.031363286	2.707210958	76874.07154	3.592442684	11.93210817
85 to 89	0.083805882	2.583657829	46027.48515	1.953860922	7.604830913	0.06797042	2.670251498	65971.24528	2.863589046	8.435951901
90 to 94	0.170063533	2.227849219	31681.10019	1.114190488	5.051138135	0.141204576	2.341706581	47374.80762	1.745204008	5.730735219
95 plus	0.274808253	3.697639517	14692.36667	0.578058593	3.697639517	0.241213913	4.192213756	23902.63039	1.03778026	4.192213756

Table 15: High-income 2017 life table, by age and sex. mx=mortality rate, ax=mean person-years lived in an age interval among those who die in that age interval, lx=number of persons left alive at age x, nLx=person-years lived between age x and x+n, ex=life expectancy at age x.

Age Group	Male					Female				
	mx	ax	lx	nLx	ex	mx	ax	lx	nLx	ex
Early Neonatal	0.129726549	0.009585065	100000	0.019154245	78.36010015	0.107131006	0.009585758	100000	0.019158394	83.49982889
Late Neonatal	0.012914546	0.028763561	99751.5187	0.057369968	78.53609347	0.011150345	0.028764047	99794.75425	0.057397747	83.65236349
Post Neonatal	0.001720637	0.461521604	99677.42806	0.91957877	78.53691411	0.001423663	0.461542701	99730.75384	0.920196846	83.64849325
1 to 4	0.000225419	1.999699441	99519.2021	3.978973941	77.73775894	0.000187268	1.999750309	99599.74893	3.98249818	82.83462266
5 to 9	0.00010879	2.499773353	99429.50844	4.970123545	73.80608108	8.81515E-05	2.499816351	99525.16938	4.975161969	78.89519665
10 to 14	0.000131284	3.105541794	99375.43828	4.967536438	68.84487888	9.90271E-05	2.778350527	99481.31261	4.972971567	73.92887592
15 to 19	0.000491169	2.81350986	99310.22269	4.960184212	63.88804875	0.000220678	2.711176493	99432.06672	4.969093493	68.96411452
20 to 24	0.00087363	2.618581836	99066.59385	4.943045781	59.03824378	0.00032324	2.618560832	99322.41002	4.962300641	64.03726002
25 to 29	0.000994809	2.549115925	98634.75477	4.919742605	54.28525481	0.000405671	2.611875822	99162.00857	4.953301707	59.13660769
30 to 34	0.001118157	2.5617933	98145.33464	4.893924461	49.54324334	0.000542948	2.627342234	98961.06752	4.941687359	54.25137977
35 to 39	0.001339827	2.605615808	97598.11791	4.864300957	44.80665692	0.000740853	2.644336696	98692.75972	4.926041063	49.39172345
40 to 44	0.00181822	2.661444157	96946.38715	4.826795843	40.09035255	0.001062631	2.679768321	98327.81305	4.904298861	44.56522439
45 to 49	0.002790482	2.696873013	96068.77195	4.772764965	35.43226999	0.001672209	2.695866563	97806.66819	4.871563351	39.78839891
50 to 54	0.004557167	2.69264316	94736.94587	4.687557825	30.89245948	0.002670308	2.680607646	96992.04313	4.819751156	35.0999282
55 to 59	0.007263761	2.667970574	92600.76114	4.552915377	26.54297997	0.004072413	2.660776473	95705.02587	4.740095994	30.53588517
60 to 64	0.011037072	2.638514899	89293.66342	4.3512733	22.42720258	0.006005656	2.651008479	93774.67387	4.623509235	26.10968143
65 to 69	0.015966918	2.637441776	84491.19237	4.070992842	18.5519728	0.008844683	2.671297471	90997.9764	4.45807844	21.82547988
70 to 74	0.024670715	2.638089317	77991.18009	3.684847802	14.87832141	0.014285074	2.68395452	87054.99469	4.213354045	17.69301155
75 to 79	0.039795153	2.62538911	68900.61423	3.147599815	11.49325531	0.024118718	2.69461285	81036.29884	3.838395079	13.80773519
80 to 84	0.068852885	2.573428172	56375.18869	2.415248201	8.463526949	0.04518753	2.659875512	71778.89793	3.245741119	10.24098271
85 to 89	0.125773595	2.446913915	39746.34652	1.504297984	5.927863281	0.091015603	2.573342355	57112.92549	2.339063639	7.187742049
90 to 94	0.220788917	2.2019423	20827.2486	0.643709997	4.090057281	0.172558	2.321905005	35825.10938	1.225127419	4.92979609
95 plus	0.317814752	3.146498089	6615.455778	0.208161273	3.146498089	0.271451385	3.683936782	14685.83248	0.541041582	3.683936782

Table 15: High-income 2100 life table, by age and sex. mx=mortality rate, ax=mean person-years lived in an age interval among those who die in that age interval, lx=number of persons left alive at age x, nLx=person-years lived between age x and x+n, ex=life expectancy at age x.

Age Group	Male					Female				
	mx	ax	lx	nLx	ex	mx	ax	lx	nLx	ex
Early Neonatal	0.041756537	0.009587761	100000	0.019170405	82.80878271	0.036900241	0.00958791	100000	0.019171298	86.11328599
Late Neonatal	0.003979609	0.028766026	99919.95304	0.057481611	82.85593976	0.004246181	0.028765952	99929.25885	0.057486524	86.15505694
Post Neonatal	0.000424457	0.461613683	99897.07821	0.922156706	82.81738088	0.000375	0.461617196	99904.84948	0.922249496	86.11856555
1 to 4	6.46773E-05	1.999913764	99857.93805	3.993800896	81.92637245	6.00357E-05	1.999919952	99870.26609	3.99433103	85.22494245
5 to 9	4.17477E-05	2.499913026	99832.10793	4.991084469	77.9470535	4.42353E-05	2.499907843	99846.2864	4.991762274	81.24493188
10 to 14	6.0256E-05	3.397683838	99811.27163	4.990072265	72.96278922	5.94562E-05	2.905638645	99824.20546	4.990582859	76.26234611
15 to 19	0.000292977	2.897279391	99781.20445	4.985975302	67.98374303	0.000154334	2.765802753	99794.53416	4.988002196	71.28416074
20 to 24	0.000608442	2.668400056	99635.14184	4.974696847	63.07891682	0.000252165	2.64796027	99717.55622	4.982918086	66.33702388
25 to 29	0.000784683	2.577093733	99332.53936	4.957199236	58.26253794	0.000329647	2.619382649	99591.91529	4.975684441	61.41734995
30 to 34	0.000897713	2.545039363	98943.76922	4.936301973	53.48084684	0.000435451	2.597215382	99427.91405	4.96619421	56.51427318
35 to 39	0.000971096	2.573075717	98500.98202	4.913463219	48.70942274	0.000528779	2.612933732	99211.6935	4.954329265	51.63165321
40 to 44	0.001234011	2.636687085	98024.25482	4.8869549	43.93343206	0.000722523	2.682677576	98949.76232	4.939213847	46.76120404
45 to 49	0.00178158	2.693613067	97421.76376	4.851159485	39.18829103	0.001163448	2.690884439	98592.96452	4.916438699	41.9203818
50 to 54	0.00292144	2.69727491	96558.26695	4.795664611	34.51381572	0.001800627	2.671864493	98021.10884	4.880606147	37.14865026
55 to 59	0.004639664	2.65032307	95158.64969	4.706675281	29.98019403	0.002691228	2.655078202	97142.6157	4.826685498	32.4594434
60 to 64	0.006503795	2.637038224	92977.43917	4.578640818	25.61895622	0.003882918	2.658063876	95844.504	4.749080759	27.86148417
65 to 69	0.0094318	2.653885724	90005.08541	4.403066151	21.37425564	0.005818065	2.693211202	94002.40816	4.6379446	23.35309647
70 to 74	0.014552891	2.688742346	85864.09142	4.154033216	17.27254698	0.00970615	2.734385646	91308.47787	4.46738107	18.95977166
75 to 79	0.025538523	2.704034997	79845.94688	3.772497882	13.3660605	0.018090789	2.773404797	86983.60644	4.181280431	14.76244
80 to 84	0.049045496	2.647356789	70283.16593	3.154501284	9.811874606	0.039008671	2.709315537	79451.77439	3.648996422	10.89376329
85 to 89	0.098496544	2.530942125	55003.93308	2.219216287	6.800813937	0.081215633	2.612637228	65336.86582	2.74170647	7.653062929
90 to 94	0.19070524	2.241779605	33502.45756	1.103599864	4.570567432	0.159867183	2.336066994	43360.89649	1.527119381	5.220286649
95 plus	0.292813285	3.421582063	12768.10096	0.441370006	3.421582063	0.259326427	3.867522809	19290.92577	0.754120988	3.867522809

Table 15: Australasia 2017 life table, by age and sex. mx=mortality rate, ax=mean person-years lived in an age interval among those who die in that age interval, lx=number of persons left alive at age x, nLx=person-years lived between age x and x+n, ex=life expectancy at age x.

Age Group	Male					Female				
	mx	ax	lx	nLx	ex	mx	ax	lx	nLx	ex
Early Neonatal	0.105739211	0.0095858	100000	0.01915865	80.03558933	0.093460282	0.009586177	100000	0.019160905	84.41249689
Late Neonatal	0.007783611	0.028764976	99797.42093	0.05740484	80.17885055	0.006986144	0.028765196	99820.92339	0.057419676	84.5447382
Post Neonatal	0.001283277	0.461552674	99752.74084	0.920459374	80.15721625	0.00104598	0.461569531	99780.81009	0.92081922	84.52118199
1 to 4	0.000185461	1.999752719	99634.62495	3.983907164	79.3284092	0.000144329	1.999807561	99684.49652	3.986229121	83.67911489
5 to 9	0.000102057	2.499787381	99560.74247	4.976767262	75.38579589	8.46234E-05	2.499823701	99626.96508	4.980294571	79.72628455
10 to 14	0.000105944	3.175348133	99509.95232	4.974535993	70.42299804	9.07437E-05	2.815412445	99584.82074	4.978254338	74.75896769
15 to 19	0.000446124	2.771960517	99457.25081	4.9679248	65.45862537	0.000222379	2.659183573	99539.64654	4.974393088	69.79161126
20 to 24	0.000690697	2.604713108	99235.62154	4.953585847	60.59856062	0.000261203	2.575442079	99429.02712	4.968305088	64.86627067
25 to 29	0.000797506	2.561565674	98893.48442	4.935077441	55.79909362	0.000317831	2.601264959	99299.25471	4.96118058	59.9476424
30 to 34	0.000933019	2.580368761	98499.91997	4.913903622	51.01169616	0.000416983	2.648011272	99141.57496	4.952222481	55.03880335
35 to 39	0.001167512	2.622129558	98041.46375	4.88850318	46.23803542	0.000616686	2.665968739	98935.08011	4.939644117	50.14809046
40 to 44	0.001634065	2.644252135	97470.76543	4.854851699	41.49323495	0.000912574	2.681537575	98630.47042	4.921111307	45.29463287
45 to 49	0.002329802	2.663721459	96677.53291	4.807710991	36.81175671	0.001421583	2.682386442	98181.40576	4.892949846	40.48940898
50 to 54	0.003530875	2.674444596	95557.5991	4.738973007	32.21166911	0.002180855	2.667721125	97485.88298	4.849629026	35.75897024
55 to 59	0.005437239	2.667342244	93884.67485	4.635454354	27.73760169	0.003232425	2.663194201	96428.35706	4.785276725	31.12164392
60 to 64	0.00824732	2.668116677	91364.98525	4.482079954	23.42851672	0.00483695	2.674403924	94881.78252	4.691328373	26.58520334
65 to 69	0.012930365	2.673265389	87669.89235	4.255530136	19.30297695	0.007572989	2.697485526	92613.10357	4.551320453	22.17052212
70 to 74	0.021127242	2.671636025	82170.35594	3.916028151	15.41537663	0.01278385	2.713987349	89167.5433	4.331844768	17.92244546
75 to 79	0.036162482	2.653873804	73903.77714	3.406504628	11.84033753	0.022990696	2.71703542	83632.80952	3.973250551	13.92823846
80 to 84	0.065234121	2.592647336	61600.32531	2.662570098	8.675330214	0.044581671	2.67367619	74505.99429	3.375623709	10.30101071
85 to 89	0.121083572	2.460804628	44260.75443	1.693394043	6.060357275	0.09010145	2.576921948	59476.6418	2.441632668	7.228710077
90 to 94	0.215848307	2.210089301	23793.96439	0.743000332	4.161842595	0.17139927	2.323416754	37513.20299	1.286471969	4.955091092
95 plus	0.313761561	3.187551654	7780.552359	0.248263762	3.187551654	0.27034912	3.699893595	15498.64518	0.574134311	3.699893595

Table 15: Australasia 2100 life table, by age and sex. mx=mortality rate, ax=mean person-years lived in an age interval among those who die in that age interval, lx=number of persons left alive at age x, nLx=person-years lived between age x and x+n, ex=life expectancy at age x.

Age Group	Male					Female				
	mx	ax	lx	nLx	ex	mx	ax	lx	nLx	ex
Early Neonatal	0.037145033	0.009587903	100000	0.019171253	84.2328556	0.03510777	0.009587965	100000	0.019171628	87.67039957
Late Neonatal	0.002205082	0.028766515	99928.79735	0.057489634	84.27371252	0.002673193	0.028766386	99932.69858	0.057491104	87.71027019
Post Neonatal	0.000299268	0.461622576	99916.12192	0.922385808	84.22686786	0.000258053	0.461625504	99917.33132	0.922414519	87.66622195
1 to 4	5.17979E-05	1.999930936	99888.52068	3.99512696	83.32673221	4.3446E-05	1.999942072	99893.53011	3.995394045	86.76371669
5 to 9	4.05158E-05	2.499915592	99867.82818	4.992885682	79.34358992	4.43782E-05	2.499907545	99876.17277	4.99325468	82.77844485
10 to 14	5.23242E-05	3.527008051	99847.60026	4.991966271	74.35913398	5.41849E-05	3.086104224	99854.01576	4.99214863	77.79625519
15 to 19	0.000285397	2.794478355	99821.48303	4.987907302	69.37767462	0.000164434	2.676978146	99826.96878	4.989438052	72.81653296
20 to 24	0.00045061	2.618556565	99679.16128	4.978618351	64.47233024	0.000189941	2.579284051	99744.93946	4.984949165	67.87410027
25 to 29	0.000557754	2.600733766	99454.93552	4.966082088	59.61109698	0.000230712	2.625626318	99650.2894	4.979758764	62.93600125
30 to 34	0.000711639	2.595695438	99178.26434	4.950404729	54.76930257	0.000301871	2.640305831	99535.46501	4.973207008	58.00543609
35 to 39	0.000850304	2.607368131	98826.49151	4.931281894	49.95441381	0.000411117	2.65063648	99385.41483	4.964450053	53.08886581
40 to 44	0.00115219	2.626417483	98407.84331	4.906946929	45.15533821	0.000574746	2.676772476	99181.42254	4.952449796	48.19238233
45 to 49	0.001541571	2.631505576	97843.61382	4.874380273	40.40000619	0.00089336	2.6567301	98896.90932	4.934524181	43.32309781
50 to 54	0.002155275	2.647254184	97093.66411	4.830200503	35.69099602	0.001259755	2.646071538	98456.38329	4.908247746	38.50445998
55 to 59	0.003136508	2.640438551	96054.9233	4.767494195	31.04693335	0.001765314	2.662036023	97838.7852	4.871800798	33.72978609
60 to 64	0.004418001	2.66848449	94562.95114	4.680013535	26.49266803	0.002617838	2.676689035	96979.93154	4.819722757	29.00387291
65 to 69	0.007063761	2.707918438	92501.16041	4.551559659	22.0197013	0.004074159	2.731195046	95720.24757	4.742199427	24.34860899
70 to 74	0.012396806	2.726875466	89299.16829	4.343112493	17.70622974	0.007312132	2.790177907	93793.06081	4.615172162	19.79022536
75 to 79	0.023219262	2.733047655	83951.66767	3.989206318	13.65140914	0.015032583	2.830415825	90430.47	4.378709452	15.41843067
80 to 84	0.047315359	2.668514827	74794.96861	3.373419928	9.980304265	0.034599729	2.763793022	83888.11246	3.894679573	11.39598746
85 to 89	0.095793127	2.539981848	59116.67643	2.402781251	6.91442052	0.074070324	2.641951871	70533.15054	3.008422798	8.015493705
90 to 94	0.187362365	2.243278987	36645.81793	1.217546371	4.63550344	0.150383231	2.344308529	48594.22137	1.745180186	5.449821147
95 plus	0.289960647	3.458837628	14326.42839	0.502874169	3.458837628	0.250225522	4.012891551	22809.98828	0.927613046	4.012891551

Table 15: Australia 2017 life table, by age and sex. mx=mortality rate, ax=mean person-years lived in an age interval among those who die in that age interval, lx=number of persons left alive at age x, nLx=person-years lived between age x and x+n, ex=life expectancy at age x.

Age Group	Male					Female				
	mx	ax	lx	nLx	ex	mx	ax	lx	nLx	ex
Early Neonatal	0.104606335	0.009585835	100000	0.019158858	80.11664383	0.09190318	0.009586224	100000	0.019161191	84.58316304
Late Neonatal	0.007758934	0.028764983	99799.59044	0.057406129	80.25832087	0.006887094	0.028765223	99823.90505	0.057421555	84.7131824
Post Neonatal	0.001217458	0.461557349	99755.05169	0.92050867	80.23660737	0.00099134	0.461573412	99784.35959	0.920875205	84.68921174
1 to 4	0.000179197	1.999761071	99642.9901	3.984291579	79.4030395	0.000144123	1.999807835	99693.0727	3.986573724	83.84305535
5 to 9	9.20789E-05	2.499808169	99571.59751	4.977434045	75.45854021	7.77892E-05	2.499837939	99635.61906	4.980812278	79.89025315
10 to 14	0.000105118	3.145793204	99525.76782	4.975318711	70.49213734	8.75177E-05	2.834867797	99596.87456	4.978900541	74.92036047
15 to 19	0.000418522	2.777443972	99473.46953	4.969051638	65.52753536	0.000218891	2.647680642	99553.30079	4.975103629	69.95190236
20 to 24	0.000664681	2.608591722	99265.50681	4.955398537	60.65890406	0.000243293	2.567778993	99444.40072	4.969279751	65.02556519
25 to 29	0.000768664	2.568836632	98936.13832	4.937580002	55.85201411	0.000298907	2.610856919	99323.50319	4.962631376	60.10154008
30 to 34	0.000924783	2.589219465	98556.61938	4.916870265	51.05704314	0.000403483	2.662319905	99175.16968	4.954086362	55.1874641
35 to 39	0.001174622	2.622561002	98101.9423	4.891439344	46.28150067	0.000615846	2.663463854	98975.28635	4.941653798	50.29345511
40 to 44	0.001632732	2.64187647	97527.43647	4.857671807	41.53844559	0.000890948	2.679197917	98670.97133	4.923368213	45.44021107
45 to 49	0.00231763	2.661201153	96734.41953	4.810649597	36.85699582	0.00139137	2.678657496	98232.35699	4.895805754	40.63095926
50 to 54	0.003491132	2.672316639	95619.71809	4.742455963	32.25521854	0.002106367	2.666136768	97551.23974	4.853703961	35.89569549
55 to 59	0.005351698	2.669948803	93964.54598	4.640380637	27.77577158	0.003122538	2.666391562	96529.02421	4.791542507	31.24724731
60 to 64	0.008197762	2.669858897	91482.15091	4.488410343	23.4563564	0.004716753	2.676137167	95033.17317	4.700155181	26.69667145
65 to 69	0.012860238	2.674844311	87804.63559	4.262853871	19.32619753	0.007386653	2.700773141	92816.92287	4.56337979	22.26964786
70 to 74	0.021104051	2.671912297	82326.71378	3.923757037	15.43332477	0.012574456	2.715885971	89447.73748	4.34760379	18.00597281
75 to 79	0.036071168	2.653723438	74055.53277	3.414275467	11.85794118	0.022627782	2.71911012	83985.11228	3.993367788	13.99951638
80 to 84	0.065005043	2.593060074	61761.19192	2.67108267	8.690411579	0.04399188	2.677002304	74960.30469	3.401030771	10.35680674
85 to 89	0.120768356	2.46177061	44438.48285	1.701674582	6.070115259	0.089193007	2.580410263	60026.14507	2.469561515	7.267952601
90 to 94	0.215505209	2.210588769	23939.31757	0.748209245	4.167172408	0.170263384	2.325001184	38049.98774	1.308122384	4.97920714
95 plus	0.313477191	3.19060118	7848.399503	0.250764827	3.19060118	0.269272358	3.715090699	15827.72781	0.589021978	3.715090699

Table 15: Australia 2100 life table, by age and sex. mx=mortality rate, ax=mean person-years lived in an age interval among those who die in that age interval, lx=number of persons left alive at age x, nLx=person-years lived between age x and x+n, ex=life expectancy at age x.

Age Group	Male					Female				
	mx	ax	lx	nLx	ex	mx	ax	lx	nLx	ex
Early Neonatal	0.036881274	0.009587911	100000	0.019171302	84.26458241	0.034065994	0.009587997	100000	0.019171819	87.82412406
Late Neonatal	0.002228957	0.028766508	99929.30283	0.057489886	84.3050263	0.002672921	0.028766386	99934.69529	0.057492254	87.86233715
Post Neonatal	0.000290267	0.461623216	99916.49033	0.922393041	84.25830081	0.000248342	0.461626194	99919.32947	0.922437099	87.81831043
1 to 4	4.90922E-05	1.999934544	99889.719	3.995196505	83.35747689	4.30209E-05	1.999942638	99896.42317	3.995513153	86.91505567
5 to 9	3.70881E-05	2.499922733	99870.10701	4.993042381	79.37345994	4.17014E-05	2.499913121	99879.23506	4.993441164	82.92966645
10 to 14	5.22997E-05	3.463404653	99851.58895	4.992158706	74.38770214	5.25956E-05	3.102322319	99858.41241	4.992390419	77.94643507
15 to 19	0.000267926	2.804743427	99825.48144	4.98831688	69.40625263	0.000165054	2.663936809	99832.15705	4.989676132	72.96615314
20 to 24	0.000433004	2.624420047	99691.85675	4.979474315	64.49513478	0.000176453	2.569878523	99749.8136	4.985344661	68.02407148
25 to 29	0.000538315	2.616810469	99476.34903	4.967418048	59.62848784	0.000216139	2.64066602	99661.8734	4.980525834	63.08171793
30 to 34	0.000713145	2.608522504	99209.24128	4.951972689	54.7811465	0.000296293	2.658302778	99554.27617	4.974234337	58.14689559
35 to 39	0.000865497	2.610659362	98856.59102	4.932622397	49.96664553	0.000414914	2.650410825	99406.96657	4.965478696	53.22896756
40 to 44	0.001171652	2.627955468	98430.32637	4.907850083	45.17117635	0.000569686	2.676649839	99201.04723	4.953483741	48.33376736
45 to 49	0.001560907	2.629668143	97856.48252	4.874788187	40.42000463	0.000891813	2.64924062	98918.97763	4.93560979	43.4637904
50 to 54	0.002154854	2.644360583	97097.06499	4.830344679	35.71483082	0.001226635	2.640116904	98479.09738	4.909721036	38.64563071
55 to 59	0.003090518	2.642073388	96058.60133	4.768218536	31.0710237	0.001706213	2.662660908	97877.55546	4.874405419	33.8660078
60 to 64	0.004372656	2.672157023	94588.24069	4.681834208	26.51067832	0.002548407	2.673716215	97046.95618	4.82378995	29.13220255
65 to 69	0.006978868	2.71616542	92546.85349	4.554951908	22.0326172	0.003926956	2.734797156	95819.61762	4.748731783	24.46949818
70 to 74	0.012395479	2.735123597	89381.28724	4.347540989	17.71069849	0.007134369	2.793099733	93959.4601	4.625188213	19.8974853
75 to 79	0.023272913	2.73786709	84030.54619	3.992922896	13.6550798	0.014707961	2.833231361	90671.00663	4.393386318	15.5142571
80 to 84	0.047290973	2.674268749	74845.33311	3.376732371	9.987094143	0.033968857	2.766621288	84247.64162	3.916614671	11.47756276
85 to 89	0.095746531	2.54019426	59164.96333	2.405354651	6.918655182	0.072990617	2.646593568	71061.71895	3.038553569	8.076172917
90 to 94	0.187274369	2.243086485	36700.27682	1.219899696	4.63809703	0.148889703	2.345101333	49242.53953	1.774105407	5.488868334
95 plus	0.289880149	3.460330482	14368.44971	0.504888056	3.460330482	0.248780516	4.037685899	23319.29863	0.954828592	4.037685899

Table 15: New Zealand 2017 life table, by age and sex. mx=mortality rate, ax=mean person-years lived in an age interval among those who die in that age interval, lx=number of persons left alive at age x, nLx=person-years lived between age x and x+n, ex=life expectancy at age x.

Age Group	Male					Female				
	mx	ax	lx	nLx	ex	mx	ax	lx	nLx	ex
Early Neonatal	0.111630557	0.00958562	100000	0.019157568	79.6207123	0.101523253	0.009585929	100000	0.019159424	83.54774347
Late Neonatal	0.007911835	0.028764941	99786.14374	0.057398141	79.7721513	0.007498656	0.028765055	99805.48763	0.05740995	83.6913738
Post Neonatal	0.001621374	0.461528656	99740.73164	0.920204942	79.75092439	0.001325456	0.461549677	99762.43807	0.920530916	83.66994179
1 to 4	0.000217991	1.999709345	99591.53354	3.98192507	78.94642111	0.000145392	1.999806144	99640.4263	3.984458335	82.84854503
5 to 9	0.000152702	2.499681871	99504.73263	4.973337823	75.01354537	0.00011904	2.499752001	99582.4961	4.977643325	78.89557776
10 to 14	0.000110051	3.308361825	99428.78996	4.970513943	70.06893165	0.000106561	2.73442283	99523.24276	4.974961082	73.94106203
15 to 19	0.000580551	2.757998481	99374.08942	4.962245697	65.10567841	0.000239219	2.717759827	99470.22907	4.970797696	68.97900967
20 to 24	0.000832219	2.596933383	99086.00678	4.944412114	60.2869209	0.000357295	2.611267642	99351.31855	4.963329889	64.05830685
25 to 29	0.000974232	2.531624345	98674.52622	4.921890375	55.5274518	0.000431313	2.569112673	99173.98204	4.95350554	59.16816654
30 to 34	0.000988719	2.528805487	98195.02503	4.897784715	50.78619731	0.000502286	2.57777239	98960.33194	4.942004051	54.29034173
35 to 39	0.001121645	2.618253709	97710.77937	4.872522613	46.02531355	0.000621626	2.674155723	98712.10402	4.928479695	49.42035842
40 to 44	0.001641788	2.657769652	97164.26771	4.839603663	41.26941266	0.001027477	2.690982012	98405.74089	4.908641488	44.56586099
45 to 49	0.00239642	2.675622352	96369.73293	4.791796492	36.58768	0.001575901	2.696009573	97901.39854	4.877361069	39.78153118
50 to 54	0.003733943	2.684591429	95221.47092	4.720266139	31.99652042	0.002542171	2.674691858	97132.79441	4.828100023	35.07491977
55 to 59	0.005878865	2.655474057	93459.066	4.609425734	27.54913774	0.003782254	2.651659303	95905.4544	4.753057972	30.48946384
60 to 64	0.008506457	2.659475419	90749.50006	4.448909339	23.29222657	0.005458209	2.667198347	94107.82521	4.646235795	26.02109252
65 to 69	0.013294098	2.665498496	86965.53562	4.217412343	19.18978582	0.008537556	2.683786013	91572.01413	4.489826506	21.66765162
70 to 74	0.021255611	2.670124498	81359.91527	3.876091995	15.32806103	0.013921864	2.703421479	87739.2754	4.251071829	17.49671362
75 to 79	0.036634762	2.655155867	73123.43704	3.367052257	11.75361132	0.024832884	2.70866025	81822.21603	3.870918412	13.56623239
80 to 84	0.066502916	2.590786359	60793.88695	2.620153055	8.598873223	0.047878101	2.65778439	72212.89668	3.246729174	10.01080684
85 to 89	0.122776263	2.455746917	43380.21305	1.653027207	6.011383167	0.095070144	2.558401194	56676.35206	2.300232135	7.026623059
90 to 94	0.217646369	2.207212739	23099.16053	0.718501965	4.135252018	0.177479369	2.313991177	34822.43623	1.179314735	4.832020027
95 plus	0.315240604	3.172342817	7470.329014	0.237079115	3.172342817	0.276081603	3.622486028	13905.54621	0.503974429	3.622486028

Table 15: New Zealand 2100 life table, by age and sex. mx=mortality rate, ax=mean person-years lived in an age interval among those who die in that age interval, lx=number of persons left alive at age x, nLx=person-years lived between age x and x+n, ex=life expectancy at age x.

Age Group	Male					Female				
	mx	ax	lx	nLx	ex	mx	ax	lx	nLx	ex
Early Neonatal	0.037704931	0.009587885	100000	0.019171151	84.05585654	0.039894964	0.009587818	100000	0.019170749	86.82102436
Late Neonatal	0.002062692	0.028766554	99927.73597	0.057489259	84.09754212	0.002572559	0.028766414	99923.54145	0.057486004	86.86829056
Post Neonatal	0.000341062	0.461619607	99915.88075	0.922365814	84.04999518	0.000302006	0.461622382	99908.75759	0.922316691	86.82360037
1 to 4	6.42533E-05	1.999914329	99884.43162	3.994863958	83.15306426	4.54585E-05	1.999939382	99880.91386	3.994873465	85.92439395
5 to 9	5.88309E-05	2.499877436	99858.76829	4.992204458	79.17393408	5.71611E-05	2.499880929	99862.76044	4.992425468	81.9396403
10 to 14	5.23E-05	3.159068273	99829.4181	4.990984999	74.19636509	6.51157E-05	3.029632251	99834.27029	4.991001104	76.96227123
15 to 19	0.000388506	2.677964594	99803.33684	4.985605532	69.21488725	0.000158172	2.754129656	99801.79979	4.988304289	71.98636837
20 to 24	0.000553589	2.560629449	99609.74139	4.973775009	64.3436324	0.000261733	2.629030494	99722.94115	4.983042051	67.04100653
25 to 29	0.000671199	2.522050432	99334.61111	4.9584992	59.51404927	0.000308334	2.593795376	99592.6687	4.975873051	62.12500253
30 to 34	0.000707638	2.518258639	99002.33072	4.941436105	54.70445604	0.000329805	2.57013309	99439.56436	4.967957131	57.21654218
35 to 39	0.00076434	2.561756009	98653.50033	4.923475006	49.88830688	0.000383774	2.666127849	99275.93465	4.959307628	52.30645079
40 to 44	0.001036392	2.584201886	98278.15741	4.901613216	45.06867884	0.000604406	2.67008305	99085.85375	4.947321355	47.40123045
45 to 49	0.001428031	2.597061266	97771.40232	4.871846348	40.28846432	0.000904506	2.705809492	98787.1408	4.92904969	42.53565572
50 to 54	0.002160242	2.61026594	97077.58965	4.828957011	35.55685897	0.001460306	2.669560637	98342.17568	4.900399339	37.71400992
55 to 59	0.003417914	2.589859535	96037.08102	4.762643384	30.9116777	0.002121715	2.654593017	97627.97274	4.857153225	32.96814486
60 to 64	0.004690308	2.59985615	94415.09948	4.668292861	26.39439937	0.003039355	2.678606548	96600.38769	4.796204186	28.28787486
65 to 69	0.007579384	2.606777219	92234.05354	4.529764733	21.95198447	0.00496505	2.702387536	95146.52685	4.703789589	23.67618644
70 to 74	0.012427692	2.615780699	88816.75005	4.313698087	17.69031814	0.008426823	2.757667334	92820.16808	4.555303164	19.19713977
75 to 79	0.02293527	2.62540722	83490.48968	3.960964539	13.64383225	0.01717486	2.795666917	89004.20692	4.288621351	14.89453411
80 to 84	0.047636558	2.560125263	74523.46112	3.344446402	9.961023226	0.038951601	2.728018515	81714.8079	3.757286484	10.96495575
85 to 89	0.095840726	2.539929	58901.72897	2.394764728	6.917678421	0.080838697	2.615645681	67314.47863	2.832456238	7.709390958
90 to 94	0.187350311	2.242674682	36544.58766	1.215092344	4.637868928	0.158982891	2.333896072	44991.8982	1.593148253	5.259358297
95 plus	0.289938974	3.460208789	14323.95373	0.503902397	3.460208789	0.258397666	3.892653351	20349.6157	0.808533852	3.892653351

Table 15: High-income Asia Pacific 2017 life table, by age and sex. mx=mortality rate, ax=mean person-years lived in an age interval among those who die in that age interval, lx=number of persons left alive at age x, nLx=person-years lived between age x and x+n, ex=life expectancy at age x.

Age Group	Male					Female				
	mx	ax	lx	nLx	ex	mx	ax	lx	nLx	ex
Early Neonatal	0.047178309	0.009587595	100000	0.019169409	80.61703709	0.039976448	0.009587816	100000	0.019170732	86.74628477
Late Neonatal	0.006291737	0.028765388	99909.56203	0.057471811	80.67082477	0.005496384	0.028765607	99923.36226	0.057481065	86.79363079
Post Neonatal	0.00105995	0.461568539	99873.40235	0.921667748	80.64248749	0.000925582	0.461578084	99891.76851	0.921894412	86.7635386
1 to 4	0.00017706	1.99976392	99775.71027	3.989615445	79.79770624	0.00015091	1.999798787	99806.4397	3.991052888	85.91403423
5 to 9	9.41002E-05	2.499803958	99705.07031	4.984080917	75.85282539	7.1487E-05	2.499851069	99746.21076	4.986419325	81.96470354
10 to 14	9.46591E-05	2.900288656	99658.17004	4.981918321	70.88734621	6.70211E-05	2.704585661	99710.56435	4.984761366	76.99311216
15 to 19	0.000276271	2.775895234	99611.01168	4.977492147	65.91953256	0.000137403	2.721158561	99677.15594	4.982297756	72.01801084
20 to 24	0.00046148	2.613818326	99473.49806	4.968204046	61.00681995	0.000213119	2.624067076	99608.69759	4.977914291	67.06563551
25 to 29	0.000530224	2.568580565	99244.22581	4.955822276	56.14171313	0.000264767	2.619472904	99502.60873	4.971996674	62.13434053
30 to 34	0.000639027	2.601597486	98981.45672	4.941499337	51.2839296	0.000365789	2.631302298	99370.96675	4.964247128	57.21318041
35 to 39	0.000846507	2.671181088	98665.68231	4.923578044	46.43972793	0.0004968	2.654733883	99189.3803	4.953697307	52.31310005
40 to 44	0.001344124	2.695945638	98248.89977	4.897278588	41.62538895	0.000737762	2.667829983	98943.28131	4.938666642	47.43660875
45 to 49	0.002133412	2.703580255	97590.64908	4.855743403	36.88795357	0.001097542	2.670108092	98578.92675	4.916374412	42.60207056
50 to 54	0.003488247	2.68744911	96554.73061	4.789104459	32.25468896	0.001648386	2.652151257	98039.33656	4.883068644	37.8218398
55 to 59	0.005418914	2.679987271	94884.2025	4.685307311	27.77521856	0.002331643	2.658509997	97234.42394	4.835322778	33.11296519
60 to 64	0.008534689	2.662952201	92345.34109	4.526974063	23.46513229	0.003488136	2.669189052	96107.01043	4.76659754	28.47020686
65 to 69	0.012948738	2.666107025	88481.82692	4.294317325	19.37341641	0.005310716	2.704192928	94444.37659	4.66533824	23.92440045
70 to 74	0.020991547	2.668514409	82921.4316	3.952633718	15.49370924	0.009079539	2.739606474	91966.78833	4.505866299	19.49605057
75 to 79	0.035641228	2.652685394	74624.70357	3.443195087	11.91957964	0.016972509	2.748787844	87875.77868	4.232091497	15.27610547
80 to 84	0.064017768	2.589461442	62353.74569	2.700928737	8.743288075	0.033834247	2.707302474	80693.17096	3.744227631	11.39114578
85 to 89	0.11947531	2.465511866	45064.76718	1.729569261	6.104315191	0.073116162	2.643946696	68025.63846	2.901491977	8.008221616
90 to 94	0.214173215	2.212966115	24402.92323	0.764095568	4.18558487	0.149683689	2.349817181	46812.70653	1.675884448	5.439107036
95 plus	0.312392359	3.201127213	8039.496626	0.257370072	3.201127213	0.249661634	4.005482864	21729.45705	0.870421716	4.005482864

Table 15: High-income Asia Pacific 2100 life table, by age and sex. mx=mortality rate, ax=mean person-years lived in an age interval among those who die in that age interval, lx=number of persons left alive at age x, nLx=person-years lived between age x and x+n, ex=life expectancy at age x.

Age Group	Male					Female				
	mx	ax	lx	nLx	ex	mx	ax	lx	nLx	ex
Early Neonatal	0.013736163	0.00958862	100000	0.019175556	85.14162727	0.01228855	0.009588664	100000	0.019175823	89.85481886
Late Neonatal	0.001703656	0.028766653	99973.66061	0.057516274	85.14488263	0.001824097	0.02876662	99976.43601	0.057517671	89.85682055
Post Neonatal	0.000270714	0.461624605	99963.86206	0.922838683	85.09569359	0.000242429	0.461626614	99965.94441	0.922869955	89.80871407
1 to 4	3.76606E-05	1.999949786	99938.8806	3.997254144	84.19356565	3.53972E-05	1.999952804	99943.57196	3.997459879	88.90542784
5 to 9	2.7493E-05	2.499942723	99923.82704	4.995847972	80.20594777	2.92606E-05	2.499939042	99929.42236	4.996105642	84.91773356
10 to 14	4.09521E-05	3.215847371	99910.09222	4.995123749	75.21662344	3.48987E-05	2.797284278	99914.80371	4.995349362	79.92978826
15 to 19	0.000152994	2.87494511	99889.63698	4.992857539	70.2313772	7.25167E-05	2.714063917	99897.37115	4.994041604	74.94325199
20 to 24	0.000315666	2.624688876	99813.2531	4.986923561	65.28279637	0.000110317	2.618289069	99861.15709	4.991745047	69.96942362
25 to 29	0.000342248	2.543436208	99655.86005	4.978603081	60.3814675	0.000133975	2.623459965	99806.09355	4.988710848	65.00653845
30 to 34	0.000383004	2.556668086	99485.52459	4.969624593	55.48021113	0.000184526	2.615040654	99739.26474	4.984765828	60.04828534
35 to 39	0.000445125	2.643261556	99295.26811	4.959555508	50.58135783	0.000232435	2.65006485	99647.29347	4.979641921	55.1012273
40 to 44	0.000683858	2.693563268	99074.64023	4.945927949	45.68781216	0.000349543	2.70068483	99531.56554	4.972579089	50.16210174
45 to 49	0.001078571	2.731500746	98736.63987	4.924773785	40.83459737	0.000567587	2.705664368	99357.78052	4.961419593	45.2449345
50 to 54	0.001880158	2.722613163	98205.95757	4.889353812	36.0398407	0.000904283	2.683382049	99076.24282	4.943453248	40.36553986
55 to 59	0.00310276	2.694626437	97287.70959	4.829852937	31.35322772	0.001367913	2.672762963	98629.33345	4.915818969	35.53584943
60 to 64	0.004866971	2.674561847	95791.20333	4.736004734	26.79940448	0.002057274	2.665487066	97957.15192	4.874452009	30.76069399
65 to 69	0.007412052	2.687241079	93490.50049	4.595834286	22.39055212	0.003043197	2.717421097	96954.96194	4.814297798	26.05001019
70 to 74	0.012149253	2.710729139	90092.1679	4.383021423	18.13064639	0.00532796	2.790203131	95491.18241	4.719053077	21.40615657
75 to 79	0.021766554	2.717844803	84783.02847	4.039407278	14.09193744	0.010943565	2.82513222	92979.78139	4.541073978	16.90668336
80 to 84	0.041930347	2.670108889	76036.76943	3.466242322	10.39455935	0.024569525	2.788796664	88022.09049	4.175189086	12.69521974
85 to 89	0.087973958	2.564734069	61649.71137	2.544771364	7.191643719	0.05701228	2.715032632	77817.64242	3.445396583	8.985637955
90 to 94	0.178377176	2.252135414	39582.5176	1.334241401	4.790661329	0.126708333	2.354806526	58350.58793	2.191051704	6.077926277
95 plus	0.282421002	3.547750299	16106.60739	0.576875322	3.547750299	0.227319684	4.411977056	30886.06677	1.373650147	4.411977056

Table 15: Brunei 2017 life table, by age and sex. mx=mortality rate, ax=mean person-years lived in an age interval among those who die in that age interval, lx=number of persons left alive at age x, nLx=person-years lived between age x and x+n, ex=life expectancy at age x.

Age Group	Male					Female				
	mx	ax	lx	nLx	ex	mx	ax	lx	nLx	ex
Early Neonatal	0.182331662	0.009583453	100000	0.019144591	73.30454958	0.135462087	0.009584889	100000	0.019153192	77.48749576
Late Neonatal	0.02268673	0.028760865	99650.93871	0.057296017	73.54198976	0.021004675	0.028761329	99740.54884	0.057350313	77.66984623
Post Neonatal	0.0039792	0.46136116	99520.95736	0.91717895	73.5804274	0.00341848	0.461400993	99620.08863	0.918330039	77.70619211
1 to 4	0.000357822	1.999522904	99156.01382	3.963403727	72.92614738	0.000292423	1.999610102	99306.16897	3.969924613	77.02708236
5 to 9	0.000272914	2.49943143	99014.20671	4.947334437	69.02768018	0.000186048	2.4996124	99190.08445	4.957198214	73.11488915
10 to 14	0.000328469	2.857571026	98879.20199	4.940477199	64.1184634	0.000248006	2.706693947	99097.85844	4.95207626	68.18060096
15 to 19	0.00083297	2.785127583	98716.93872	4.926756426	59.21911398	0.000432833	2.600890718	98975.0464	4.943618904	63.26183707
20 to 24	0.00147675	2.581419629	98306.56722	4.897836413	54.45459927	0.000459087	2.561359169	98761.07378	4.932533651	58.39324881
25 to 29	0.001426559	2.490818774	97583.29075	4.861767243	49.83896446	0.000572519	2.646130905	98534.63001	4.920103444	53.52150791
30 to 34	0.001440226	2.550854468	96889.73647	4.827461766	45.17783782	0.00086717	2.675266528	98252.95062	4.902763211	48.66726347
35 to 39	0.001804722	2.651152931	96194.5098	4.789420921	40.48590495	0.001310019	2.657169644	97827.82142	4.8764233	43.86698062
40 to 44	0.002787927	2.654753635	95330.26324	4.735554885	35.82890184	0.001872172	2.66021536	97189.06541	4.838259274	39.13764446
45 to 49	0.003960146	2.704788348	94010.2712	4.658174322	31.29463498	0.002788423	2.731562234	96283.39443	4.7839057	34.48057026
50 to 54	0.006896563	2.688974117	92166.09331	4.536021416	26.86675063	0.005079564	2.701484382	94949.70503	4.692698687	29.92632596
55 to 59	0.010770301	2.666477466	89038.8007	4.342827378	22.71581814	0.008001149	2.670541478	92566.49157	4.54364923	25.62703104
60 to 64	0.01692056	2.653161131	84363.07108	4.057122551	18.8264716	0.01239558	2.66006598	88931.7915	4.321273203	21.56504739
65 to 69	0.026694781	2.640977289	77501.09826	3.645653971	15.25691065	0.019399208	2.629137541	83576.34423	3.995116129	17.7762297
70 to 74	0.043945903	2.585408225	67776.57617	3.06417992	12.06449329	0.028628468	2.618093776	75828.13529	3.549463584	14.32372248
75 to 79	0.065217809	2.526789299	54332.79055	2.340206528	9.407643621	0.045683177	2.589966166	65670.70054	2.958061673	11.13404701
80 to 84	0.098547907	2.470163146	39114.30749	1.566702134	7.086661121	0.072259201	2.538597832	52166.00104	2.214798104	8.346041443
85 to 89	0.16141434	2.348934799	23736.56476	0.83226875	5.0861416	0.12878207	2.446351045	36179.38238	1.361728686	5.913530666
90 to 94	0.256257628	2.13372638	10357.34248	0.299045899	3.638493352	0.215991252	2.238656553	18664.34296	0.584842557	4.170900868
95 plus	0.346309158	2.888217366	2719.242099	0.078714135	2.888217366	0.31161823	3.209461399	6046.469465	0.194223797	3.209461399

Table 15: Brunei 2100 life table, by age and sex. mx=mortality rate, ax=mean person-years lived in an age interval among those who die in that age interval, lx=number of persons left alive at age x, nLx=person-years lived between age x and x+n, ex=life expectancy at age x.										
**Age Group**	**Male**					**Female**				
	**mx**	**ax**	**lx**	**nLx**	**ex**	**mx**	**ax**	**lx**	**nLx**	**ex**
Early Neonatal	0.075462371	0.009586728	100000	0.019164212	79.0299339	0.055248104	0.009587348	100000	0.019167926	82.52863807
Late Neonatal	0.009611235	0.028764472	99855.39197	0.057435167	79.12525347	0.010301333	0.028764282	99894.1058	0.057456294	82.59692428
Post Neonatal	0.001785361	0.461517007	99800.19365	0.920683947	79.11149341	0.001510435	0.461536537	99834.92084	0.921121156	82.5883282
1 to 4	0.000106215	1.999858379	99635.84859	3.984587575	78.31785744	8.72899E-05	1.999883613	99695.81312	3.987136517	81.7795876
5 to 9	9.78045E-05	2.499796241	99593.53341	4.978459458	74.35027906	8.52887E-05	2.499822315	99661.01498	4.981988442	77.80743949
10 to 14	0.000158416	3.204478591	99544.85029	4.975743536	69.38531949	0.000131801	2.835174733	99618.52587	4.979492464	72.8395458
15 to 19	0.000556648	2.781830233	99466.04171	4.967175508	64.4377513	0.000284941	2.654117944	99552.90297	4.974315736	67.88569108
20 to 24	0.000925806	2.571656285	99189.58675	4.948370592	59.60900051	0.000338732	2.584198675	99411.17736	4.966492971	62.97862007
25 to 29	0.000894489	2.48314066	98731.67675	4.92550113	54.87244954	0.000420993	2.612212211	99242.9653	4.957160186	58.08093691
30 to 34	0.000858226	2.519211653	98291.5064	4.904122973	50.10605021	0.000561643	2.629332349	99034.32566	4.945097724	53.19770683
35 to 39	0.000961144	2.619535052	97871.07736	4.882372837	45.30974165	0.000736838	2.653899549	98756.71561	4.929287401	48.33970896
40 to 44	0.001402754	2.667266651	97402.34877	4.854240148	40.51444051	0.001082541	2.696483011	98393.6635	4.90740572	43.50808376
45 to 49	0.002108917	2.740394695	96722.14467	4.813120453	35.77969513	0.00172845	2.71434925	97862.65012	4.873833933	38.72928059
50 to 54	0.003829063	2.729653196	95708.66807	4.744185543	31.12820219	0.002848647	2.69192202	97020.61597	4.819328161	34.04138625
55 to 59	0.006483085	2.683238502	93894.64439	4.625305229	26.67519735	0.004418979	2.679727722	95648.50803	4.733859716	29.49017697
60 to 64	0.009979475	2.679334415	90901.41849	4.44237512	22.4630682	0.006830609	2.666067801	93558.44479	4.604551914	25.08754599
65 to 69	0.01635821	2.677536	86479.71605	4.166139219	18.47101357	0.01037376	2.670149854	90417.10711	4.414178521	20.86389926
70 to 74	0.027219413	2.63377803	79695.56754	3.745064329	14.81228781	0.016377576	2.700919268	85848.74796	4.136879887	16.82955173
75 to 79	0.041758245	2.597422824	69587.87711	3.165022445	11.57767834	0.029870367	2.678193842	79091.98124	3.699038268	13.03264932
80 to 84	0.066718786	2.549548199	56525.24707	2.433867821	8.655420086	0.053098819	2.620814091	68110.02693	3.027318909	9.696677135
85 to 89	0.122286928	2.45858472	40516.58197	1.553173961	6.060506673	0.102045596	2.536555402	52238.98961	2.096226288	6.822938797
90 to 94	0.216676708	2.206162315	21915.75584	0.687973618	4.163538677	0.185167662	2.297100898	31324.61299	1.052759801	4.713427248
95 plus	0.314324035	3.188564695	7295.882646	0.235849083	3.188564695	0.283093537	3.548770632	12297.79243	0.444540141	3.548770632

Table 15: Japan 2017 life table, by age and sex. mx=mortality rate, ax=mean person-years lived in an age interval among those who die in that age interval, lx=number of persons left alive at age x, nLx=person-years lived between age x and x+n, ex=life expectancy at age x.

Age Group	Male					Female				
	mx	ax	lx	nLx	ex	mx	ax	lx	nLx	ex
Early Neonatal	0.042961494	0.009587724	100000	0.019170184	80.94709334	0.037262598	0.009587899	100000	0.019171231	87.03011594
Late Neonatal	0.004868394	0.02876578	99917.64209	0.057478812	80.99462848	0.004479416	0.028765888	99928.56305	0.057485738	87.07314713
Post Neonatal	0.000919579	0.46157851	99889.65919	0.9218775	80.95977585	0.000810239	0.461586278	99902.81283	0.922045428	87.03804884
1 to 4	0.000181333	1.999758222	99804.88548	3.990747937	80.104863	0.00015764	1.999789813	99828.10525	3.991865521	86.1795519
5 to 9	8.82519E-05	2.499816142	99732.52013	4.985525972	76.16153571	6.8607E-05	2.499857069	99765.17742	4.987403396	82.23264917
10 to 14	9.04929E-05	2.90558742	99688.52198	4.983481582	71.19404681	6.47081E-05	2.687311764	99730.96037	4.985801902	77.26000502
15 to 19	0.000264682	2.819319662	99643.42504	4.979297279	66.22495265	0.000126873	2.729823351	99698.6982	4.983499557	72.28413619
20 to 24	0.000497231	2.613564905	99511.63186	4.969684519	61.30892425	0.000204883	2.626378977	99635.47105	4.979352033	67.3282732
25 to 29	0.000537641	2.546424758	99264.52403	4.956687625	56.45503433	0.000251565	2.601483189	99533.45254	4.973671612	62.39458841
30 to 34	0.000620113	2.589569671	98998.03238	4.942513892	51.60014496	0.000328135	2.633876386	99408.33263	4.966560577	57.46984409
35 to 39	0.000808644	2.6554895	98691.54134	4.925239495	46.75234331	0.000463704	2.669531991	99245.36263	4.956911462	52.55988631
40 to 44	0.001231988	2.690004735	98293.26611	4.900716522	41.93101108	0.000708142	2.687722607	99015.50912	4.942682195	47.675696
45 to 49	0.001951471	2.704524722	97689.50669	4.86269279	37.17352287	0.00110803	2.683096255	98665.49822	4.920642649	42.83528142
50 to 54	0.003197496	2.699605496	96740.5744	4.801709943	32.51161105	0.001696802	2.659270631	98120.27913	4.886605602	38.0583827
55 to 59	0.005143617	2.699749668	95205.24906	4.70460004	27.99234613	0.002437633	2.658968198	97291.12478	4.836953941	33.36005508
60 to 64	0.008474509	2.667167459	92785.42579	4.549334584	23.65193745	0.003628071	2.655865654	96112.06385	4.765078193	28.73666668
65 to 69	0.012769745	2.660298097	88930.17943	4.317517151	19.56161601	0.005303559	2.686448656	94383.28125	4.661962676	24.21435874
70 to 74	0.020336538	2.664927422	83417.01859	3.981776804	15.67860678	0.008747629	2.72779561	91910.82884	4.505981191	19.79344532
75 to 79	0.034196755	2.658559716	75319.9159	3.486827962	12.0775782	0.015974355	2.74917034	87969.28289	4.24580926	15.55805303
80 to 84	0.0622767	2.597885311	63397.10287	2.757403318	8.848982386	0.031851236	2.716599531	81187.19969	3.784157111	11.62802545
85 to 89	0.117182031	2.472376384	46226.80369	1.783227847	6.170994161	0.069823069	2.657757058	69135.00006	2.970917576	8.181534409
90 to 94	0.211732921	2.21684444	25333.05751	0.7970265	4.221804916	0.145244425	2.35326653	48392.91481	1.747807633	5.549218605
95 plus	0.31038393	3.221844351	8459.034287	0.272555081	3.221844351	0.245386548	4.075273842	23009.21065	0.937749455	4.075273842

Table 15: Japan 2100 life table, by age and sex. mx=mortality rate, ax=mean person-years lived in an age interval among those who die in that age interval, lx=number of persons left alive at age x, nLx=person-years lived between age x and x+n, ex=life expectancy at age x.

Age Group	Male					Female				
	mx	ax	lx	nLx	ex	mx	ax	lx	nLx	ex
Early Neonatal	0.013170897	0.009588637	100000	0.01917566	84.76378285	0.012320623	0.009588663	100000	0.019175817	89.9287602
Late Neonatal	0.001189611	0.028766795	99974.74428	0.057517748	84.76602195	0.001416823	0.028766732	99976.37451	0.057518309	89.93083362
Post Neonatal	0.000213554	0.461628665	99967.90197	0.922900327	84.71429095	0.000201599	0.461629514	99968.22524	0.922908404	89.88062826
1 to 4	4.30139E-05	1.999942648	99948.19341	3.99758383	83.80761434	4.24453E-05	1.999943406	99949.61965	3.99764542	88.97398553
5 to 9	3.08586E-05	2.49993571	99930.99863	4.996164494	79.82168851	3.35263E-05	2.499930154	99932.65189	4.996213831	84.98875181
10 to 14	4.46652E-05	3.241593004	99915.58161	4.995365294	74.83361242	3.96528E-05	2.781567242	99915.90188	4.995346383	80.00257511
15 to 19	0.000169783	2.910239216	99893.27103	4.992891928	69.84961796	7.82038E-05	2.722389784	99896.09488	4.99391527	75.01789238
20 to 24	0.000380661	2.625779612	99808.50444	4.985920403	64.90634206	0.000123206	2.610602727	99857.04218	4.991380243	70.04614235
25 to 29	0.000402154	2.532295243	99618.74004	4.975997052	60.02460143	0.000141273	2.59170815	99795.55026	4.988075531	65.08764888
30 to 34	0.000441753	2.548162405	99418.69062	4.965558385	55.13992186	0.000180724	2.608569551	99725.08986	4.984097666	60.13175311
35 to 39	0.00050659	2.621025695	99199.43113	4.954002096	50.25581499	0.000232938	2.660023367	99635.02476	4.979034219	55.18369197
40 to 44	0.000737836	2.677845745	98948.63031	4.938968588	45.3761501	0.000357006	2.719520131	99519.06005	4.97190259	50.24479555
45 to 49	0.001138869	2.725927602	98584.51437	4.91648062	40.53333984	0.00060786	2.720575675	99341.58755	4.960194537	45.32953561
50 to 54	0.001970388	2.727178166	98025.22424	4.879398655	35.74828438	0.000992021	2.6924175	99040.151	4.940692484	40.45897618
55 to 59	0.003306198	2.705426453	97065.13943	4.816731966	31.07368128	0.001528249	2.675921187	98550.16352	4.910068766	35.64632847
60 to 64	0.005334979	2.671229392	95475.41266	4.715254032	26.54422637	0.002303818	2.654825603	97800.1091	4.863724617	30.89851251
65 to 69	0.007968679	2.669962862	92965.59996	4.563685468	22.18577782	0.003279452	2.693976216	96680.38553	4.797721779	26.2244753
70 to 74	0.012525931	2.700726529	89340.06807	4.342313085	17.97417015	0.005455656	2.761410997	95108.45044	4.698085103	21.61205367
75 to 79	0.022179361	2.719795743	83921.83349	3.99506649	13.955887	0.010574492	2.813118699	92548.30456	4.522917338	17.13155057
80 to 84	0.043459301	2.66409001	75112.77904	3.412634285	10.26846435	0.023254821	2.791963467	87776.58	4.175100552	12.90627259
85 to 89	0.090234709	2.557465387	60448.10531	2.483402026	7.107129323	0.054620666	2.725985954	78114.72653	3.476903109	9.148361373
90 to 94	0.181033264	2.249978258	38393.76342	1.288205948	4.742999671	0.123132983	2.35381986	59287.14015	2.24169476	6.186745941
95 plus	0.284661163	3.520426516	15421.36786	0.548564472	3.520426516	0.223816385	4.481432243	31977.01102	1.444443473	4.481432243

Table 15: South Korea 2017 life table, by age and sex. mx=mortality rate, ax=mean person-years lived in an age interval among those who die in that age interval, lx=number of persons left alive at age x, nLx=person-years lived between age x and x+n, ex=life expectancy at age x.

Age Group	Male					Female				
	mx	ax	lx	nLx	ex	mx	ax	lx	nLx	ex
Early Neonatal	0.056774801	0.009587301	100000	0.019167645	79.45715833	0.047336435	0.00958759	100000	0.01916938	85.54574609
Late Neonatal	0.009449598	0.028764517	99891.17645	0.057456016	79.52453159	0.007817982	0.028764967	99909.25928	0.057469114	85.60425599
Post Neonatal	0.001381515	0.461545695	99836.88334	0.921194	79.51022884	0.001202485	0.461558413	99864.33042	0.921523398	85.58522323
1 to 4	0.000176323	1.999764903	99709.61889	3.986978606	78.68783256	0.000145412	1.999806118	99753.51853	3.988980545	84.75649266
5 to 9	0.000107495	2.499776052	99639.3197	4.980627386	74.74193858	7.77818E-05	2.499837955	99695.51434	4.983806534	80.80464174
10 to 14	9.9789E-05	2.895627785	99585.78055	4.978243735	69.78077715	7.09936E-05	2.733688271	99656.74953	4.982035968	75.83510105
15 to 19	0.000297434	2.707319151	99536.10332	4.973413809	64.81415493	0.000157596	2.70614899	99621.38031	4.97926908	70.86105222
20 to 24	0.000396666	2.618728075	99388.17776	4.96471932	59.90656708	0.000227272	2.621196423	99542.90907	4.974456154	65.91477067
25 to 29	0.00052515	2.616595073	99191.24521	4.953362426	55.02027245	0.000290393	2.658566293	99429.85386	4.968114744	60.9867234
30 to 34	0.000693542	2.622295451	98931.1217	4.938412761	50.15801944	0.000449446	2.625534841	99285.58406	4.958987207	56.0714599
35 to 39	0.000937762	2.703594471	98588.62751	4.918839258	45.32310504	0.000563312	2.630853415	99062.70675	4.946533949	51.19167695
40 to 44	0.001623133	2.70180797	98127.36926	4.888135238	40.52336778	0.000807122	2.629407779	98784.06741	4.929770914	46.328613
45 to 49	0.002542009	2.695361146	97333.99242	4.838356065	35.83152786	0.001071289	2.640137036	98386.1846	4.90690423	41.50528705
50 to 54	0.004089877	2.662041041	96104.16031	4.75969948	31.25538356	0.00154117	2.638103456	97860.5306	4.875280654	36.71399146
55 to 59	0.005908712	2.647241957	94157.70515	4.643342133	26.84622188	0.002118766	2.650584159	97109.19987	4.831411023	31.97756027
60 to 64	0.008671793	2.66336004	91414.50819	4.479965073	22.57210703	0.00312613	2.703130166	96085.59738	4.770032175	27.28988747
65 to 69	0.013644977	2.691149655	87530.38937	4.242887519	18.45523871	0.005300356	2.771386921	94594.54249	4.674516127	22.67731484
70 to 74	0.023737898	2.685642334	81742.72803	3.874375261	14.5710436	0.010484187	2.781146554	92117.17466	4.501165891	18.21246854
75 to 79	0.042403748	2.642569544	72550.00877	3.298032886	11.07671901	0.021203487	2.761407481	87398.98478	4.171973651	14.04521654
80 to 84	0.075924297	2.562002563	58575.06347	2.471677918	8.089092659	0.044394187	2.68898372	78555.9027	3.562471011	10.31509754
85 to 89	0.134786423	2.421006157	39827.42565	1.478129866	5.69225796	0.089877061	2.577683211	62749.01009	2.576831302	7.236159093
90 to 94	0.230040102	2.185371076	19925.23023	0.604948679	3.963160227	0.171142426	2.323942999	39605.42777	1.3585396	4.95947055
95 plus	0.325337411	3.073944462	6021.035311	0.185193256	3.073944462	0.270111238	3.70262707	16371.47796	0.606503445	3.70262707

Table 15: South Korea 2100 life table, by age and sex. mx=mortality rate, ax=mean person-years lived in an age interval among those who die in that age interval, lx=number of persons left alive at age x, nLx=person-years lived between age x and x+n, ex=life expectancy at age x.

Age Group	Male					Female				
	mx	ax	lx	nLx	ex	mx	ax	lx	nLx	ex
Early Neonatal	0.015708716	0.00958856	100000	0.019175194	85.98062191	0.013513329	0.009588627	100000	0.019175597	89.70619445
Late Neonatal	0.002926452	0.028766316	99969.87954	0.057512075	85.98732958	0.002804759	0.02876635	99974.08817	0.057514698	89.71026008
Post Neonatal	0.000402446	0.461615247	99953.04997	0.922682774	85.94425555	0.000346278	0.461619237	99957.95741	0.922751993	89.66719069
1 to 4	2.91196E-05	1.999961174	99915.92242	3.996404153	85.05273258	2.43699E-05	1.999967506	99926.00785	3.996845514	88.77241213
5 to 9	2.01195E-05	2.499958082	99904.28547	4.99496303	81.06240691	1.99851E-05	2.499958363	99916.26803	4.995563814	84.78087046
10 to 14	3.01989E-05	3.225884432	99894.23596	4.994435197	76.07030155	2.50337E-05	2.896183975	99906.28472	4.995042565	79.78908953
15 to 19	0.000117348	2.773422973	99879.15366	4.992644767	71.08130418	5.933E-05	2.694032577	99893.78085	4.99400647	74.79871973
20 to 24	0.000176681	2.634598055	99820.57191	4.988942388	66.12125618	8.09422E-05	2.645845926	99864.15293	4.992256758	69.82008314
25 to 29	0.000230357	2.614844381	99732.44922	4.983865653	61.17714073	0.000116357	2.73654267	99823.74905	4.989856016	64.84721726
30 to 34	0.000285555	2.59533189	99617.69487	4.977457389	56.24435569	0.000196372	2.63500868	99765.70123	4.985958371	59.88329462
35 to 39	0.000351453	2.706148415	99475.62876	4.969757899	51.32076542	0.00023275	2.628216009	99667.81302	4.980636677	54.93941587
40 to 44	0.000617254	2.724177007	99301.07383	4.958082331	46.40594013	0.000335303	2.651834013	99551.91798	4.973678213	50.00013423
45 to 49	0.001011777	2.735185497	98995.21786	4.938439698	41.54044138	0.000475772	2.666656839	99385.19658	4.963738317	45.07926051
50 to 54	0.001763083	2.697137656	98495.97453	4.904865083	36.73622462	0.000705738	2.657406872	99149.1342	4.949265562	40.17975109
55 to 59	0.002679953	2.655450301	97632.18631	4.851147759	32.03586842	0.001002929	2.663369538	98799.99018	4.928449976	35.31172624
60 to 64	0.003825374	2.680801183	96333.99671	4.774349987	27.42987601	0.001499157	2.707750565	98305.95073	4.89847299	30.47498907
65 to 69	0.006111194	2.727220863	94511.73687	4.660839117	22.90410518	0.002526601	2.800108747	97572.10927	4.851595663	25.68259008
70 to 74	0.010842611	2.741201684	91671.15941	4.474310054	18.52612037	0.00517367	2.856538932	96347.75429	4.764608628	20.97126159
75 to 79	0.020519454	2.730201947	86834.58221	4.14944263	14.40057942	0.011896726	2.847719747	93886.35711	4.577364352	16.44273942
80 to 84	0.039342754	2.698328062	78383.15062	3.596895941	10.65164516	0.027686001	2.788948642	88459.19767	4.16949774	12.26909403
85 to 89	0.083909012	2.578307594	64410.0828	2.684581201	7.367033527	0.062426463	2.691395905	77009.56906	3.370597967	8.664109779
90 to 94	0.173286785	2.25352069	42301.6927	1.441421732	4.891887758	0.134359254	2.352598834	56267.42366	2.084417955	5.868337399
95 plus	0.278072554	3.605845902	17765.78836	0.648405862	3.605845902	0.234746463	4.278671678	28730.35938	1.244898614	4.278671678

Table 15: Singapore 2017 life table, by age and sex. mx=mortality rate, ax=mean person-years lived in an age interval among those who die in that age interval, lx=number of persons left alive at age x, nLx=person-years lived between age x and x+n, ex=life expectancy at age x.

Age Group	Male					Female				
	mx	ax	lx	nLx	ex	mx	ax	lx	nLx	ex
Early Neonatal	0.030149625	0.009588117	100000	0.019172539	81.24278612	0.021307236	0.009588388	100000	0.019174164	87.36024971
Late Neonatal	0.004206416	0.028765963	99942.19599	0.057494032	81.27058963	0.003349849	0.028766199	99959.14534	0.0575052	87.37677186
Post Neonatal	0.000656369	0.461597208	99918.01207	0.922251214	81.23271874	0.000522688	0.461606705	99939.88217	0.922509998	87.33607341
1 to 4	8.35733E-05	1.999888569	99857.47974	3.993631644	80.35839382	6.32088E-05	1.999915722	99891.66429	3.995161497	86.45472006
5 to 9	7.3657E-05	2.499846548	99824.10435	4.99028625	76.38459275	6.20086E-05	2.499870815	99866.41163	4.992546593	82.4760757
10 to 14	0.000109563	2.801768074	99787.34791	4.988165926	71.41180813	5.87308E-05	2.723353717	99835.45368	4.991105359	77.50087407
15 to 19	0.00023248	2.761199144	99732.69646	4.984040815	66.44940212	0.000125076	2.735180586	99806.14059	4.988893845	72.52283417
20 to 24	0.000401834	2.585863105	99616.82776	4.976014217	61.52346563	0.000200152	2.621162928	99743.7417	4.98481372	67.56648458
25 to 29	0.000399229	2.52303267	99416.87555	4.965933073	56.64197809	0.000241885	2.58398925	99643.97026	4.979288664	62.63150024
30 to 34	0.000447683	2.586064268	99218.6225	4.955575894	51.75009237	0.000298301	2.625736355	99523.52946	4.972654628	57.70415239
35 to 39	0.000586577	2.695178446	98996.77184	4.943155878	46.86024244	0.000422977	2.674755934	99375.19588	4.96387772	52.7863468
40 to 44	0.00100236	2.69507911	98706.8219	4.92396532	41.98993993	0.000655333	2.689505923	99165.23787	4.950765783	47.89241839
45 to 49	0.00153705	2.731434282	98213.27479	4.893600665	37.18734497	0.001024366	2.685853928	98840.80359	4.930353086	43.040752
50 to 54	0.002744159	2.757155487	97461.13147	4.843248581	32.45316181	0.001582232	2.671434105	98335.76737	4.898740826	38.24794773
55 to 59	0.005034588	2.714737753	96132.14045	4.751937265	27.863553	0.002356214	2.655409255	97560.70355	4.851237062	33.53048761
60 to 64	0.008250121	2.685468912	93739.94149	4.599184182	23.50511942	0.003403591	2.668053307	96417.72185	4.782927785	28.89636062
65 to 69	0.01322185	2.687697829	89946.04663	4.363908757	19.38301579	0.005245512	2.691526651	94789.97295	4.682801405	24.34658372
70 to 74	0.022487804	2.665470262	84177.29784	3.99898534	15.52685378	0.008580982	2.725508204	92333.99206	4.528334345	19.92235993
75 to 79	0.037410368	2.615630676	75187.3332	3.45162462	12.06438061	0.0155902	2.749464365	88449.14888	4.272589358	15.67741407
80 to 84	0.059496114	2.534379807	62281.19369	2.715917001	9.022445832	0.031054176	2.718075599	81790.37347	3.819008875	11.72964734
85 to 89	0.11308877	2.484775923	46133.73381	1.796168084	6.294156503	0.068468076	2.663568353	69936.90668	3.014856525	8.25691845
90 to 94	0.207323144	2.223506904	25835.93686	0.82005367	4.288943867	0.143376758	2.354341414	49308.49867	1.787753338	5.597596414
95 plus	0.306741002	3.260255408	8844.521624	0.288471155	3.260255408	0.243580756	4.105982413	23693.78146	0.973349931	4.105982413

Table 15: Singapore 2100 life table, by age and sex. mx=mortality rate, ax=mean person-years lived in an age interval among those who die in that age interval, lx=number of persons left alive at age x, nLx=person-years lived between age x and x+n, ex=life expectancy at age x.

Age Group	Male					Female				
	mx	ax	lx	nLx	ex	mx	ax	lx	nLx	ex
Early Neonatal	0.005401644	0.009588876	100000	0.019177089	85.63478921	0.003832393	0.009588922	100000	0.019177377	90.75593588
Late Neonatal	0.000777436	0.028766897	99989.64323	0.057527001	85.62448899	0.000724033	0.028766929	99992.65143	0.05752882	90.7434165
Post Neonatal	0.000118277	0.461635448	99985.17131	0.923100364	85.57078147	9.29308E-05	0.461637239	99988.48654	0.923141769	90.68965327
1 to 4	1.8448E-05	1.999975405	99974.25571	3.9988227	84.65677893	1.26016E-05	1.999983198	99979.90892	3.999095572	89.77409988
5 to 9	2.05274E-05	2.49995724	99966.87942	4.998087486	80.66287724	2.32832E-05	2.499951494	99974.86974	4.998452536	85.7785229
10 to 14	3.79041E-05	3.132502876	99956.62036	4.997449392	75.67089134	2.30954E-05	2.814990744	99963.23197	4.99790619	80.78821516
15 to 19	0.000112867	2.873911711	99937.67964	4.995662729	70.68465145	5.51654E-05	2.74993752	99951.68934	4.996964231	75.79721888
20 to 24	0.000224784	2.58571018	99881.30088	4.991349296	65.72279899	8.94315E-05	2.632008221	99924.12367	4.995149482	70.81736219
25 to 29	0.000199979	2.489013991	99769.13179	4.985952153	60.79355207	0.000113042	2.604441508	99879.453	4.992618748	65.84782547
30 to 34	0.00021332	2.560946298	99669.46958	4.98087944	55.85162941	0.000144294	2.624856627	99823.02058	4.989435037	60.88352705
35 to 39	0.000260881	2.70848699	99563.27831	4.975164921	50.90824998	0.000193756	2.669401897	99751.03585	4.98529261	55.92547628
40 to 44	0.00045502	2.732141911	99433.58828	4.966549738	45.97083081	0.00029435	2.732013988	99654.45806	4.979385424	50.97695782
45 to 49	0.000775036	2.768847103	99207.75422	4.951780961	41.06881479	0.000509565	2.707412189	99507.91957	4.969575993	46.04781158
50 to 54	0.00143281	2.788616174	98824.39505	4.925552751	36.21638092	0.000789801	2.678848349	99254.75864	4.953638966	41.15797657
55 to 59	0.002754045	2.737193464	98119.55129	4.875490901	31.4549617	0.001174251	2.678658957	98863.70397	4.929697939	36.30945724
60 to 64	0.004569547	2.709697538	96779.29705	4.78883585	26.84999217	0.001756225	2.676940486	98285.26176	4.89430742	31.506331
65 to 69	0.007588571	2.736426403	94596.15122	4.650030342	22.4029355	0.002699558	2.704979307	97426.60606	4.841218612	26.7588925
70 to 74	0.014044949	2.697998482	91079.99484	4.411799306	18.15621296	0.004323829	2.808682747	96122.69934	4.760796229	22.08267142
75 to 79	0.023160137	2.664741663	84924.06559	4.029952698	14.26587052	0.009090958	2.895108137	94070.6427	4.614585177	17.50006627
80 to 84	0.039881379	2.598151761	75675.74255	3.459148669	10.67335736	0.022256443	2.806493925	89896.94405	4.286213751	13.17408937
85 to 89	0.084055805	2.578590117	62179.44073	2.596849469	7.402308477	0.052450157	2.737452876	80450.65188	3.601681302	9.366534894
90 to 94	0.172912207	2.248579766	41072.28169	1.406413636	4.916545234	0.119247533	2.345977014	61928.85023	2.366034643	6.341845404
95 plus	0.277666675	3.620090185	17537.25122	0.649212323	3.620090185	0.219903875	4.581246599	34404.31951	1.604038049	4.581246599

Table 15: High-income North America 2017 life table, by age and sex. mx=mortality rate, ax=mean person-years lived in an age interval among those who die in that age interval, lx=number of persons left alive at age x, nLx=person-years lived between age x and x+n, ex=life expectancy at age x.

Age Group	Male					Female				
	mx	ax	lx	nLx	ex	mx	ax	lx	nLx	ex
Early Neonatal	0.168627517	0.009583873	100000	0.019147105	76.33859384	0.142562078	0.009584672	100000	0.019151889	81.17537286
Late Neonatal	0.013777396	0.028763323	99677.12735	0.057325761	76.56665932	0.012362118	0.028763713	99726.9668	0.057356759	81.37841074
Post Neonatal	0.002201536	0.461487442	99598.14753	0.918643462	76.56981854	0.001787942	0.461516823	99656.06178	0.919353097	81.37875652
1 to 4	0.000270214	1.999639714	99395.90528	3.973688335	75.80138971	0.000216561	1.999711253	99491.68696	3.9779443	80.58915604
5 to 9	0.000124394	2.499740846	99288.53081	4.962882999	71.88120194	0.000104073	2.49978318	99405.54048	4.968984065	76.65726299
10 to 14	0.00016117	3.233021409	99226.79554	4.959927292	66.92436858	0.000118738	2.835234499	99353.82662	4.966414781	71.69586212
15 to 19	0.000692953	2.862574992	99146.85638	4.950011208	61.97572028	0.000295503	2.755377369	99294.8564	4.961451943	66.73675712
20 to 24	0.001374672	2.637592903	98803.84396	4.924200693	57.1809329	0.000482044	2.647762868	99148.24402	4.951797471	61.83136502
25 to 29	0.001616678	2.550693297	98126.92916	4.886995265	52.55717782	0.000639777	2.619486064	98909.54599	4.937956824	56.97418798
30 to 34	0.001793718	2.5500259	97336.86179	4.845549162	47.96305717	0.000853495	2.618066415	98593.62755	4.919679884	52.14834772
35 to 39	0.002081182	2.57630996	96467.71126	4.799178068	43.37220173	0.001131432	2.628580341	98173.73635	4.895551613	47.36018174
40 to 44	0.002603278	2.626369467	95468.92267	4.744131422	38.79898669	0.001566364	2.661339109	97619.84024	4.863177274	42.61397799
45 to 49	0.003742113	2.67258292	94233.90663	4.671013862	34.27303804	0.002374785	2.680807854	96858.09395	4.816378214	37.92817416
50 to 54	0.005881406	2.671978293	92485.98684	4.561839498	29.87023623	0.003697516	2.665161196	95714.31668	4.744754228	33.34935905
55 to 59	0.009012672	2.643909934	89803.03869	4.396789754	25.68277342	0.005464487	2.644005136	93959.95509	4.63828386	28.92225158
60 to 64	0.013020307	2.616826419	85840.46625	4.162855582	21.74625556	0.007811539	2.64305944	91425.40966	4.488630427	24.65072255
65 to 69	0.018166193	2.62078526	80420.49559	3.854438622	18.03545644	0.011546034	2.664465469	87919.17239	4.280532474	20.52836089
70 to 74	0.027514365	2.620554193	73418.7861	3.445387841	14.50547985	0.018633476	2.661915047	82977.00681	3.975652032	16.59231017
75 to 79	0.043101234	2.599954996	63939.65737	2.897298903	11.26743635	0.030337536	2.648955007	75569.33245	3.526925133	12.95780144
80 to 84	0.0705221	2.551045744	51453.10597	2.193815631	8.370915352	0.052221358	2.608949846	64870.26248	2.883500747	9.658039887
85 to 89	0.127802619	2.44100562	35983.66405	1.355822083	5.873083574	0.101332317	2.535859936	49813.66345	1.993073872	6.788766238
90 to 94	0.222893674	2.198286266	18657.92305	0.574294743	4.060490085	0.184963512	2.30119393	29619.56681	0.98789401	4.688569069
95 plus	0.319532654	3.129592096	5858.437119	0.183356554	3.129592096	0.283092447	3.532462664	11348.9619	0.400927959	3.532462664

Table 15: High-income North America 2100 life table, by age and sex. mx=mortality rate, ax=mean person-years lived in an age interval among those who die in that age interval, lx=number of persons left alive at age x, nLx=person-years lived between age x and x+n, ex=life expectancy at age x.

Age Group	Male					Female				
	mx	ax	lx	nLx	ex	mx	ax	lx	nLx	ex
Early Neonatal	0.061113606	0.009587168	100000	0.019166848	80.9475616	0.054693906	0.009587365	100000	0.019168028	84.33203611
Late Neonatal	0.00485887	0.028765783	99882.86844	0.057458824	81.02328285	0.005409367	0.028765631	99895.1652	0.057464988	84.40133158
Post Neonatal	0.000585335	0.461602254	99854.95081	0.921699378	80.98839738	0.000518098	0.461607031	99864.08089	0.921812259	84.37005703
1 to 4	8.15296E-05	1.999891294	99801.00293	3.991389264	80.10863668	7.44577E-05	1.999900723	99816.32352	3.992058446	83.48691707
5 to 9	5.11292E-05	2.499893481	99768.46211	4.987785529	76.13411292	5.40753E-05	2.499887343	99786.60035	4.988655588	79.51118956
10 to 14	7.4771E-05	3.488419108	99742.96021	4.98658185	71.15292076	7.08465E-05	2.99584373	99759.62447	4.987266863	74.53200605
15 to 19	0.000404548	2.964135125	99705.67598	4.981144603	66.17819745	0.000216854	2.838199307	99724.29224	4.983863271	69.55735277
20 to 24	0.000945028	2.691877269	99504.19605	4.964372841	61.30573834	0.000409046	2.67480334	99616.22268	4.976069023	64.62968407
25 to 29	0.001272915	2.576041816	99035.23854	4.93652996	56.58251788	0.000552329	2.61889706	99412.69948	4.964093427	59.75648104
30 to 34	0.001412547	2.529977514	98407.44563	4.903258766	51.92606531	0.000713913	2.584228356	99138.56511	4.948386494	54.91438257
35 to 39	0.001467175	2.550815938	97715.79583	4.868292129	47.27497627	0.000836845	2.59129865	98785.37226	4.929331457	50.10127932
40 to 44	0.001765356	2.618334494	97002.59667	4.829816968	42.60322487	0.001085092	2.657766224	98372.96908	4.906171292	45.30012316
45 to 49	0.002470965	2.68170054	96151.2657	4.780194938	37.9564888	0.001662225	2.666441802	97840.77275	4.873130612	40.5316205
50 to 54	0.003982601	2.68196398	94971.61461	4.705172689	33.39360038	0.002432763	2.639434933	97031.05156	4.823867698	35.84693008
55 to 59	0.006113833	2.624341283	93100.31314	4.588462851	29.00906696	0.003361424	2.625280678	95858.05333	4.754973371	31.25233299
60 to 64	0.008019004	2.606110116	90298.96268	4.430134958	24.82521057	0.004574812	2.645830465	94261.09562	4.662882499	26.73575586
65 to 69	0.010966103	2.632161091	86755.10795	4.228306312	20.72906626	0.006801149	2.701683853	92130.66683	4.535700331	22.29083577
70 to 74	0.016381365	2.672527855	82135.55386	3.956615633	16.7429217	0.011751259	2.734907071	89051.25888	4.337313222	17.96612942
75 to 79	0.028104333	2.692200486	75688.64378	3.555409737	12.93703535	0.021990504	2.756978797	83967.77988	4.00148828	13.88513924
80 to 84	0.053517182	2.630048919	65778.13131	2.922530202	9.479015437	0.046653978	2.672439154	75206.49059	3.394438818	10.17795997
85 to 89	0.10486584	2.510906718	50324.65503	2.0019104	6.581856605	0.092940949	2.567980862	59499.88267	2.432326685	7.147653612
90 to 94	0.197931386	2.233777709	29662.24289	0.963516918	4.448804328	0.174509586	2.316167727	37202.89508	1.273085013	4.908591193
95 plus	0.298861221	3.351829227	10863.92934	0.367810007	3.351829227	0.273187999	3.671056866	15312.48816	0.568488451	3.671056866

Table 15: Canada 2017 life table, by age and sex. mx=mortality rate, ax=mean person-years lived in an age interval among those who die in that age interval, lx=number of persons left alive at age x, nLx=person-years lived between age x and x+n, ex=life expectancy at age x.

Age Group	Male					Female				
	mx	ax	lx	nLx	ex	mx	ax	lx	nLx	ex
Early Neonatal	0.127996787	0.009585118	100000	0.019154563	79.63218898	0.109471124	0.009585686	100000	0.019157965	83.73914208
Late Neonatal	0.013578794	0.028763378	99754.83431	0.05737078	79.80868623	0.011983056	0.028763818	99790.27902	0.057393799	83.8959218
Post Neonatal	0.001922699	0.46150725	99676.93673	0.919488552	79.81350077	0.00162055	0.461528714	99721.50642	0.920027948	83.89622558
1 to 4	0.00018204	1.99975728	99500.16193	3.978557902	79.03120106	0.000160331	1.999786226	99572.41903	3.981619932	83.09786501
5 to 9	9.15908E-05	2.499809186	99427.74206	4.970248991	75.0873128	8.27913E-05	2.499827518	99508.58444	4.974399577	79.14989077
10 to 14	0.000140519	3.130832911	99382.22108	4.967806081	70.12056225	0.000107934	2.847548489	99467.40147	4.972214988	74.181627
15 to 19	0.000517756	2.752169825	99312.4158	4.959848486	65.16764638	0.000263193	2.661431048	99413.73468	4.967629291	69.22013231
20 to 24	0.000770032	2.57130407	99055.61868	4.943535672	60.32942705	0.000312515	2.555880834	99282.99106	4.96036081	64.30776858
25 to 29	0.000786037	2.519263439	98674.95369	4.924145905	55.55220217	0.000348039	2.587683519	99127.97368	4.952240958	59.40431957
30 to 34	0.000849133	2.558825538	98287.90229	4.904229508	50.76100333	0.000460464	2.612036616	98955.61821	4.942346639	54.50325963
35 to 39	0.001033882	2.639624648	97871.47442	4.881661207	45.96605466	0.000598033	2.667903386	98728.04386	4.929527207	49.62284591
40 to 44	0.001555987	2.679066351	97366.7798	4.85082166	41.19058108	0.00094717	2.713023917	98433.24671	4.911024241	44.76343365
45 to 49	0.002401127	2.697207405	96612.02334	4.804039298	36.49136384	0.001578261	2.700021005	97968.09969	4.880688469	39.96303397
50 to 54	0.003903962	2.684292824	95458.56948	4.73016802	31.89959561	0.00249303	2.668552047	97197.82383	4.831807891	35.25825964
55 to 59	0.006037928	2.662940005	93612.06371	4.615479351	27.47571202	0.003666332	2.662698153	95993.2939	4.758886463	30.66709008
60 to 64	0.009052472	2.659507318	90825.5586	4.447068498	23.2367691	0.005517115	2.678833186	94248.64294	4.652852318	26.18534575
65 to 69	0.013964725	2.656480911	86800.43211	4.202513674	19.19077279	0.008788412	2.68538111	91681.85305	4.492715403	21.84327647
70 to 74	0.021910431	2.658632383	80932.9366	3.849235319	15.38926214	0.014320474	2.690239873	87734.04106	4.246278195	17.70510574
75 to 79	0.03685317	2.63929517	72501.67113	3.335057538	11.86955833	0.024595759	2.686463773	81654.58682	3.862983594	13.82275363
80 to 84	0.063870283	2.573087802	60216.51538	2.606965714	8.752792611	0.044672679	2.643674867	72156.77582	3.264385157	10.28842945
85 to 89	0.119179268	2.466417829	43575.86987	1.673746424	6.113453391	0.090169123	2.576568844	57581.54803	2.363035167	7.223714603
90 to 94	0.213851314	2.213436087	23641.10021	0.740835979	4.190575697	0.171505806	2.323422171	36287.68997	1.24378976	4.951840266
95 plus	0.312125653	3.20398281	7806.550488	0.250209856	3.20398281	0.270455304	3.697820842	14968.91442	0.553776312	3.697820842

Table 15: Canada 2100 life table, by age and sex. mx=mortality rate, ax=mean person-years lived in an age interval among those who die in that age interval, lx=number of persons left alive at age x, nLx=person-years lived between age x and x+n, ex=life expectancy at age x.

Age Group	Male					Female				
	mx	ax	lx	nLx	ex	mx	ax	lx	nLx	ex
Early Neonatal	0.045418095	0.009587649	100000	0.019169733	83.84650608	0.041178316	0.009587779	100000	0.019170512	86.77323394
Late Neonatal	0.005523038	0.0287656	99912.94543	0.057475029	83.90034782	0.005802372	0.028765523	99921.0664	0.057479239	86.82258534
Post Neonatal	0.000634945	0.46159873	99881.20639	0.921920645	83.86947296	0.000566213	0.461603613	99887.71837	0.922009991	86.79401996
1 to 4	5.05279E-05	1.999932629	99822.68069	3.992503783	82.99509929	5.04019E-05	1.999932797	99835.52057	3.993018332	85.91586806
5 to 9	3.51192E-05	2.499926835	99802.50929	4.989687376	79.0114745	4.06471E-05	2.499915318	99815.39684	4.990262754	81.93278619
10 to 14	6.92736E-05	3.465918292	99784.98643	4.988665847	74.02489271	6.4056E-05	3.119418775	99795.11444	4.989116974	76.94892609
15 to 19	0.00031595	2.829568656	99750.43205	4.984063788	69.049353	0.000194869	2.717560626	99763.15877	4.985930508	71.97260828
20 to 24	0.000548202	2.613331186	99592.99163	4.973135387	64.15368404	0.000258277	2.573597228	99666.00712	4.980173153	67.04006772
25 to 29	0.000617263	2.541206293	99320.43754	4.958497087	59.32222537	0.000280334	2.583492769	99537.39807	4.973495614	62.12335246
30 to 34	0.000677484	2.540569779	99014.51154	4.942492807	54.49726017	0.000366171	2.572991765	99397.99527	4.965484542	57.2068263
35 to 39	0.000758498	2.6168674	98679.87165	4.925092432	49.67307393	0.000406683	2.628646589	99216.21172	4.95602489	52.30684506
40 to 44	0.001122198	2.666280368	98306.62818	4.902492642	44.85133927	0.000612379	2.710255319	99014.71245	4.943791632	47.40787035
45 to 49	0.001689942	2.679571373	97757.06916	4.86875941	40.08793308	0.001016149	2.687619854	98712.07179	4.924003552	42.54479931
50 to 54	0.0026397	2.663849163	96935.56086	4.81704298	35.4040736	0.001503988	2.648425594	98212.00008	4.893270155	37.74743227
55 to 59	0.00382313	2.62581846	95666.51598	4.740302908	30.83687799	0.002078932	2.653358327	97476.51684	4.850139033	33.0119409
60 to 64	0.005092516	2.650919035	93857.54891	4.637477228	26.37876559	0.003043808	2.702253501	96468.9736	4.7898675	28.32870334
65 to 69	0.007954886	2.660274141	91500.26053	4.491604834	21.98792158	0.00504023	2.759378463	95012.63972	4.697455701	23.72112763
70 to 74	0.012152874	2.716612841	87936.93729	4.278489521	17.7678553	0.009516126	2.766323129	92648.05634	4.535758732	19.25544848
75 to 79	0.023219272	2.724955838	82759.12136	3.931332021	13.70526493	0.018122479	2.760808823	88341.33886	4.244557985	15.05744905
80 to 84	0.04611315	2.637931132	73692.51453	3.326132641	10.0508678	0.036137539	2.707008791	80685.30668	3.726686817	11.22218024
85 to 89	0.094102708	2.545041549	58529.62499	2.3844172	6.963688474	0.076461509	2.632217815	67310.50275	2.854930164	7.89442357
90 to 94	0.185561732	2.246124764	36462.12871	1.213227684	4.662276728	0.153551952	2.341658907	45785.78872	1.633545574	5.372897274
95 plus	0.288478376	3.474153497	14304.37611	0.502523094	3.474153497	0.253263033	3.96416425	21109.49034	0.84706059	3.96416425

Table 15: Greenland 2017 life table, by age and sex. mx=mortality rate, ax=mean person-years lived in an age interval among those who die in that age interval, lx=number of persons left alive at age x, nLx=person-years lived between age x and x+n, ex=life expectancy at age x.

Age Group	Male					Female				
	mx	ax	lx	nLx	ex	mx	ax	lx	nLx	ex
Early Neonatal	0.335684929	0.009578752	100000	0.019116484	70.83690962	0.22062752	0.009582279	100000	0.019137567	77.06179811
Late Neonatal	0.026806763	0.028759729	99358.31792	0.057121004	71.27500443	0.022669656	0.02876087	99577.7886	0.057253988	77.36925671
Post Neonatal	0.004778716	0.461304364	99205.2105	0.913932065	71.3274042	0.003465717	0.461397637	99448.00698	0.916723882	77.41264391
1 to 4	0.000573092	1.999235878	98768.52951	3.946217854	70.71739391	0.001074379	1.998567495	99130.33565	3.956706607	76.73594739
5 to 9	0.000302046	2.499370737	98542.45418	4.92340458	66.87503986	0.000226848	2.4995274	98705.30334	4.932467646	73.05780032
10 to 14	0.000343605	3.020165113	98393.76665	4.916342874	61.97231985	0.00043683	2.827029994	98593.42377	4.92499531	68.13786086
15 to 19	0.001163335	2.729903695	98224.85552	4.89830733	57.07369213	0.000914597	2.558532247	98378.30797	4.907955799	63.28068369
20 to 24	0.001639939	2.556373141	97655.05438	4.863265919	52.39075358	0.000697014	2.451156371	97929.46059	4.887790708	58.55898529
25 to 29	0.001630158	2.512440971	96857.54371	4.823322238	47.80104985	0.000758605	2.504683516	97588.78382	4.870221926	53.7547997
30 to 34	0.001769478	2.566281761	96071.27865	4.782965993	43.17165939	0.000720911	2.627398161	97219.32943	4.852667717	48.94947838
35 to 39	0.002227792	2.6347659	95224.96215	4.736290129	38.53253511	0.001209372	2.758879608	96869.48709	4.830376954	44.11664954
40 to 44	0.00326832	2.684703143	94169.90998	4.673132661	33.93475932	0.002240351	2.721926361	96285.32697	4.789818168	39.36740376
45 to 49	0.005258473	2.696183249	92642.79706	4.57669809	29.44996886	0.003655827	2.61179791	95212.31517	4.719415205	34.78018738
50 to 54	0.008568221	2.683667784	90236.55185	4.424033297	25.16350787	0.004319174	2.70509496	93487.13989	4.628478203	30.37357648
55 to 59	0.013719909	2.661185713	86446.54248	4.187958421	21.149187	0.00822047	2.681610578	91488.29054	4.488874483	25.97779114
60 to 64	0.021444766	2.632722241	80701.44667	3.840150228	17.4653396	0.01222009	2.656806461	87798.74264	4.267756897	21.95642867
65 to 69	0.032722989	2.603522522	72467.26493	3.359928329	14.15035669	0.019287312	2.627001043	82584.53941	3.948559568	18.17452238
70 to 74	0.050007257	2.563996688	61474.59889	2.740054024	11.21447399	0.028076545	2.600944222	74971.06559	3.512112754	14.75286145
75 to 79	0.075656637	2.506846306	47777.1851	2.009960456	8.693837646	0.041938474	2.586493478	65115.52957	2.956771572	11.59149578
80 to 84	0.112329146	2.43275056	32579.99562	1.264653357	6.580009385	0.066129902	2.564898833	52727.07602	2.271200339	8.70707114
85 to 89	0.176792232	2.31004144	18387.73159	0.623315413	4.782978197	0.12069227	2.471396881	37732.45264	1.446167602	6.149538226
90 to 94	0.270839891	2.103282056	7380.141716	0.206880904	3.476219868	0.20707882	2.257698105	20312.00535	0.648223786	4.309297481
95 plus	0.357775804	2.795214919	1782.414257	0.049857974	2.795214919	0.303506318	3.295550269	6912.725097	0.228121428	3.295550269

Table 15: Greenland 2100 life table, by age and sex. mx=mortality rate, ax=mean person-years lived in an age interval among those who die in that age interval, lx=number of persons left alive at age x, nLx=person-years lived between age x and x+n, ex=life expectancy at age x.

Age Group	Male					Female				
	mx	ax	lx	nLx	ex	mx	ax	lx	nLx	ex
Early Neonatal	0.079562441	0.009586603	100000	0.019163461	78.51737266	0.046927758	0.009587603	100000	0.019169456	82.25242734
Late Neonatal	0.005789149	0.028765526	99847.57721	0.057436988	78.61827572	0.005801598	0.028765523	99910.05743	0.057472908	82.30727695
Post Neonatal	0.000947426	0.461576532	99814.33548	0.921170551	78.58699182	0.000675667	0.461595837	99876.71964	0.921861888	82.2772067
1 to 4	0.000128074	1.999829235	99727.07602	3.988061709	77.73202934	0.00023763	1.99968316	99814.43916	3.990681287	81.40497366
5 to 9	5.40642E-05	2.499887364	99676.01538	4.983127251	73.7708184	7.18337E-05	2.499850346	99719.6464	4.985087089	77.48047258
10 to 14	9.10136E-05	3.285840108	99649.07837	4.981617481	68.79004839	0.000143023	2.884929739	99683.83954	4.982652918	72.50739802
15 to 19	0.000442714	2.696713484	99603.75211	4.975090738	63.81995665	0.000305671	2.554441704	99612.58515	4.976911623	67.5572264
20 to 24	0.000633009	2.632625734	99383.62058	4.961621383	58.95397584	0.000225624	2.471252593	99460.47408	4.970180213	62.65654505
25 to 29	0.000747844	2.621004112	99070.45512	4.944571148	54.13027922	0.000267371	2.568680635	99348.35159	4.964192456	57.72442996
30 to 34	0.000885516	2.574837238	98701.72928	4.924480985	49.32196296	0.000315868	2.660721023	99215.64623	4.957109937	52.7981673
35 to 39	0.001049934	2.619865146	98266.40522	4.901056538	44.52861529	0.000502038	2.76534301	99059.1188	4.947384112	47.87741565
40 to 44	0.001560526	2.691581058	97752.8097	4.870050434	39.748284	0.000936533	2.766245756	98810.82395	4.930216189	42.99060928
45 to 49	0.002654711	2.69738307	96994.18637	4.820215873	35.0375666	0.001698186	2.672159403	98349.23603	4.898082935	38.17899052
50 to 54	0.004493774	2.687743811	95716.5873	4.736655753	30.46837172	0.002343716	2.778889473	97517.89016	4.850517036	33.48086022
55 to 59	0.007635661	2.648575817	93592.22424	4.597156123	26.09720381	0.004788947	2.719070855	96382.00756	4.767017765	28.84147466
60 to 64	0.011231942	2.636466399	90096.29515	4.388489462	22.0028581	0.00759422	2.682470022	94101.25699	4.623751875	24.47198394
65 to 69	0.01712832	2.630908151	85191.41559	4.094081164	18.11448455	0.012104266	2.65732131	90596.02172	4.405135671	20.31115956
70 to 74	0.02731772	2.61872414	78213.54817	3.673340345	14.49252595	0.018292032	2.665215669	85281.71566	4.089961433	16.40676481
75 to 79	0.044105376	2.579979708	68269.98611	3.086807064	11.22142495	0.030404332	2.679745422	77844.20683	3.636904172	12.71565599
80 to 84	0.071580446	2.519818504	54776.01187	2.33004228	8.355038142	0.057392307	2.636627369	66859.48269	2.947328981	9.362844453
85 to 89	0.128629072	2.439502854	38288.08639	1.445438751	5.871967958	0.108388141	2.514186039	50152.24536	1.983572171	6.58885957
90 to 94	0.223481309	2.19582675	19964.36138	0.617011378	4.060712989	0.192673907	2.284038552	29082.11648	0.962161928	4.572368012
95 plus	0.319935982	3.129732694	6353.4631	0.2006858	3.129732694	0.290098709	3.460308816	10940.44484	0.385073299	3.460308816

Table 15: United States 2017 life table, by age and sex. mx=mortality rate, ax=mean person-years lived in an age interval among those who die in that age interval, lx=number of persons left alive at age x, nLx=person-years lived between age x and x+n, ex=life expectancy at age x.

Age Group	Male					Female				
	mx	ax	lx	nLx	ex	mx	ax	lx	nLx	ex
Early Neonatal	0.172669076	0.009583749	100000	0.019146363	75.9788858	0.145864089	0.00958457	100000	0.019151283	80.89343841
Late Neonatal	0.013794555	0.028763318	99669.40171	0.05732129	76.21169383	0.01239799	0.028763703	99720.65165	0.057353068	81.10084055
Post Neonatal	0.002229064	0.461485487	99590.32966	0.918559684	76.21464687	0.001804432	0.461515652	99649.54544	0.919285986	81.10115621
1 to 4	0.000279162	1.999627783	99385.57718	3.97320434	75.44742466	0.000222106	1.999703859	99483.66671	3.977579519	80.31232676
5 to 9	0.000127712	2.499733934	99274.66045	4.962148545	71.52948591	0.000106206	2.499778738	99395.32233	4.968446807	76.38193245
10 to 14	0.000163064	3.241349519	99211.2881	4.959142291	66.5735795	0.00011968	2.834003075	99342.55462	4.965840476	71.42117638
15 to 19	0.00070952	2.871735702	99130.42253	4.949047861	61.62524184	0.000298452	2.764330949	99283.12361	4.960846123	66.46223202
20 to 24	0.001437213	2.641380361	98779.27782	4.922278165	56.8340924	0.000499489	2.653974406	99135.06605	4.95095173	61.55736148
25 to 29	0.00170218	2.552677676	98071.843	4.883249639	52.22499059	0.000670177	2.622102044	98887.77178	4.936521706	56.70465918
30 to 34	0.001896049	2.5501569	97240.62897	4.839551783	47.64957209	0.000897227	2.61907731	98556.93796	4.917342398	51.88619509
35 to 39	0.002196285	2.573682532	96323.03116	4.790623154	43.07918175	0.001191818	2.627061181	98115.7417	4.89195217	47.10772467
40 to 44	0.002725108	2.62280179	95270.88267	4.732884332	38.52649242	0.001640164	2.657545068	97532.713	4.857971319	42.37360676
45 to 49	0.003891953	2.671455712	93981.13682	4.656854351	34.01918907	0.002465101	2.679840281	96735.93149	4.80929033	37.70071857
50 to 54	0.006121367	2.671215163	92168.74281	4.543666878	29.63557344	0.003844004	2.664879658	95550.40347	4.73501807	33.13521438
55 to 59	0.009372018	2.642098169	89387.46412	4.372745626	25.47452327	0.005678348	2.642149185	93730.28322	4.62459776	28.72688117
60 to 64	0.013497742	2.613119924	85289.45176	4.131375175	21.57153589	0.008080293	2.63985969	91104.32211	4.469972859	24.47870537
65 to 69	0.01867428	2.617556509	79713.26328	3.815901255	17.89769514	0.011865765	2.662647174	87492.54104	4.256577092	20.38019792
70 to 74	0.028222051	2.616838382	72587.74736	3.400682538	14.39763069	0.019149812	2.659362587	82441.96529	3.945269255	16.46557292
75 to 79	0.043907486	2.595666049	62991.08398	2.848839112	11.1924339	0.031037249	2.645298267	74887.25632	3.489366708	12.85832071
80 to 84	0.071417971	2.548289967	50483.926	2.148120659	8.322328816	0.053176582	2.605153008	64058.09764	2.841126081	9.584853736
85 to 89	0.128941924	2.437704737	35144.60842	1.320889737	5.842719407	0.102708311	2.531027943	48951.61609	1.952517096	6.738886996
90 to 94	0.224070464	2.196215173	18115.15074	0.556304213	4.044116552	0.186582616	2.298266991	28900.12917	0.960753855	4.658672404
95 plus	0.320491729	3.120230334	5651.374122	0.176348804	3.120230334	0.284602154	3.513730294	10976.18586	0.385706722	3.513730294

Table 15: United States 2100 life table, by age and sex. mx=mortality rate, ax=mean person-years lived in an age interval among those who die in that age interval, lx=number of persons left alive at age x, nLx=person-years lived between age x and x+n, ex=life expectancy at age x.

Age Group	Male					Female				
	mx	ax	lx	nLx	ex	mx	ax	lx	nLx	ex
Early Neonatal	0.063430664	0.009587097	100000	0.019166422	80.58601779	0.056680109	0.009587304	100000	0.019167663	84.04562535
Late Neonatal	0.004759729	0.02876581	99878.43002	0.057456435	80.66490348	0.005353436	0.028765647	99891.36015	0.057462892	84.11782139
Post Neonatal	0.000577444	0.461602815	99851.08307	0.921667033	80.62945767	0.000511024	0.461607533	99860.59833	0.921783122	84.08618788
1 to 4	8.59895E-05	1.999885347	99797.86405	3.991228129	79.748916	7.79515E-05	1.999896065	99813.49432	3.991917402	83.20236812
5 to 9	5.34085E-05	2.499888732	99763.54444	4.987511258	75.77566222	5.59634E-05	2.49988341	99782.37747	4.988420927	79.22769103
10 to 14	7.55145E-05	3.497243033	99736.90711	4.986280234	70.79521444	7.17416E-05	2.991666193	99754.46101	4.986998937	74.24915532
15 to 19	0.000416721	2.978876076	99699.25419	4.980727573	65.82060459	0.000220065	2.854956689	99718.68425	4.983563388	69.27472188
20 to 24	0.000999504	2.697762495	99491.72895	4.963155194	60.95120939	0.00042963	2.683341701	99609.02109	4.975488161	64.3477977
25 to 29	0.001362297	2.577924524	98995.87289	4.933514316	56.24214956	0.00058917	2.621111601	99395.28226	4.96279332	59.48035764
30 to 34	0.001511617	2.528948898	98324.46182	4.897921601	51.60752196	0.000760055	2.584656614	99102.9422	4.946058582	54.64798388
35 to 39	0.001561771	2.545946989	97585.20239	4.860627235	46.97855797	0.000893336	2.588917691	98727.10311	4.925744753	49.84596027
40 to 44	0.001850333	2.613902918	96827.30163	4.820077506	42.32575674	0.001146712	2.654264187	98287.18724	4.901166781	45.05709548
45 to 49	0.002573605	2.681323194	95936.87179	4.768404199	37.69354871	0.001746328	2.665142125	97725.35115	4.866419053	40.3003554
50 to 54	0.004158541	2.683136385	94711.24912	4.69040326	33.14554851	0.002553079	2.638730009	96875.84089	4.814786314	35.62962993
55 to 59	0.006412937	2.623868419	92763.37801	4.568650365	28.78332834	0.003526442	2.623021493	95647.16359	4.742636695	31.05235941
60 to 64	0.008399451	2.601953405	89837.68328	4.403441903	24.63277555	0.00476941	2.641360089	93976.2765	4.646601233	26.55602671
65 to 69	0.01135882	2.629031817	86148.47797	4.194818666	20.57218598	0.007023381	2.696926005	91763.26662	4.515210204	22.13048673
70 to 74	0.016942029	2.667730395	81402.22645	3.91611628	16.61427105	0.012036488	2.733054037	88598.30496	4.312469503	17.82228662
75 to 79	0.028770134	2.687889954	74804.45451	3.508511213	12.83993509	0.022499012	2.758092317	83423.25619	3.971383446	13.75489604
80 to 84	0.054569712	2.628484372	64796.6571	2.87255633	9.406666518	0.048093155	2.66963252	74531.66675	3.353851547	10.06293101
85 to 89	0.106333531	2.506378203	49315.46532	1.955673636	6.534479286	0.095056921	2.560444324	58551.63924	2.383087442	7.06779022
90 to 94	0.199555679	2.231678979	28859.12676	0.934669969	4.422679645	0.177032819	2.311927466	36248.92845	1.234961658	4.860270009
95 plus	0.30021211	3.336871249	10482.71282	0.353439029	3.336871249	0.275547089	3.640726538	14746.06347	0.543652326	3.640726538

Table 15: Southern Latin America 2017 life table, by age and sex. mx=mortality rate, ax=mean person-years lived in an age interval among those who die in that age interval, lx=number of persons left alive at age x, nLx=person-years lived between age x and x+n, ex=life expectancy at age x.

Age Group	Male					Female				
	mx	ax	lx	nLx	ex	mx	ax	lx	nLx	ex
Early Neonatal	0.257349369	0.009581153	100000	0.019130834	74.4120692	0.196095337	0.009583031	100000	0.019142066	80.35721639
Late Neonatal	0.029500399	0.028758986	99507.67472	0.057202434	74.76098858	0.025168366	0.028760181	99624.63616	0.057276805	80.64076099
Post Neonatal	0.00315609	0.461419632	99338.9297	0.915849102	74.83039792	0.002700786	0.461451976	99480.48271	0.917346836	80.70003757
1 to 4	0.000399668	1.99946711	99049.88929	3.958830422	74.12413098	0.000342346	1.999543538	99232.73361	3.96659291	79.97707784
5 to 9	0.000188386	2.499607529	98891.67412	4.94225579	70.23952409	0.00015096	2.4996855	99096.94299	4.952977708	76.08392982
10 to 14	0.000243912	3.111761563	98798.57212	4.93765485	65.30335953	0.000175861	2.749851975	99022.17474	4.949150556	71.13949116
15 to 19	0.000907633	2.737803077	98678.13928	4.923797615	60.37924586	0.000362698	2.63805451	98935.13965	4.94252319	66.19964135
20 to 24	0.001288114	2.558214045	98231.2462	4.896162321	55.64133845	0.000417558	2.555493094	98755.87692	4.932759116	61.31497222
25 to 29	0.001280422	2.521112038	97600.58402	4.864589242	50.98415824	0.000475858	2.597049624	98549.90919	4.921867714	56.43772153
30 to 34	0.001435556	2.564815911	96977.73953	4.831996618	46.29525773	0.00064201	2.648858646	98315.70406	4.908376875	51.56591302
35 to 39	0.001749928	2.608888791	96284.12373	4.794148541	41.6101136	0.000939954	2.666151128	98000.59238	4.889304211	46.72311292
40 to 44	0.002386169	2.667470042	95445.26164	4.745852088	36.95267617	0.001401987	2.678934177	97541.04469	4.861233302	41.93054205
45 to 49	0.003741819	2.691892515	94312.97072	4.675276738	32.36401242	0.0021674	2.682347157	96859.55664	4.818772796	37.20653242
50 to 54	0.006009911	2.692069454	92563.89448	4.564891005	27.92429197	0.003355366	2.685001005	95815.23983	4.753839526	32.58258706
55 to 59	0.009740232	2.680263561	89821.15193	4.3918553	23.69424184	0.005286855	2.680491583	94220.36722	4.653956941	28.08835844
60 to 64	0.015615736	2.654570397	85544.95017	4.126193183	19.74403211	0.008278851	2.6706001	91760.3624	4.501235947	23.76910469
65 to 69	0.02421859	2.621334543	79104.88859	3.739944774	16.1346284	0.012901297	2.658441604	88034.9134	4.272724133	19.66142135
70 to 74	0.036382924	2.594990593	70054.02394	3.221141722	12.88001134	0.020095661	2.661570971	82524.96336	3.941162288	15.79604448
75 to 79	0.056099486	2.567841853	58347.42115	2.56757445	9.943689355	0.033700466	2.663573946	74610.45178	3.458468662	12.18877292
80 to 84	0.091754206	2.501322484	43965.87107	1.788953357	7.358004237	0.062279556	2.607069726	62967.58513	2.740528321	8.949960392
85 to 89	0.153752118	2.36892012	27583.03897	0.982511654	5.246918952	0.115631191	2.487581869	45924.55894	1.780010931	6.305327532
90 to 94	0.248878384	2.148841664	12505.2029	0.365993537	3.724490504	0.201417441	2.26942701	25376.43982	0.819096403	4.401065792
95 plus	0.340463147	2.937450599	3409.887033	0.100263618	2.937450599	0.298320943	3.352735721	8903.829447	0.29886915	3.352735721

Table 15: Southern Latin America 2100 life table, by age and sex. mx=mortality rate, ax=mean person-years lived in an age interval among those who die in that age interval, lx=number of persons left alive at age x, nLx=person-years lived between age x and x+n, ex=life expectancy at age x.

Age Group	Male					Female				
	mx	ax	lx	nLx	ex	mx	ax	lx	nLx	ex
Early Neonatal	0.061004515	0.009587171	100000	0.019166868	81.00188181	0.046634669	0.009587612	100000	0.019169509	85.08304576
Late Neonatal	0.008988423	0.028764644	99883.08236	0.057452124	81.07752896	0.009618388	0.02876447	99910.60925	0.057466915	85.13995006
Post Neonatal	0.000617243	0.461599988	99831.44804	0.92146889	81.06195707	0.000559467	0.461604092	99855.34069	0.921714004	85.1294936
1 to 4	8.77818E-05	1.999882958	99774.57923	3.99028268	80.18461163	7.95391E-05	1.999893948	99803.78142	3.99151636	84.24992962
5 to 9	5.61731E-05	2.499882972	99739.55723	4.986277615	76.21206847	6.34141E-05	2.499867887	99772.03862	4.987811247	80.27609148
10 to 14	9.55996E-05	3.394770387	99711.54897	4.984771799	71.23276519	9.08972E-05	2.827621365	99740.41325	4.986019561	75.30074162
15 to 19	0.000451048	2.747910014	99663.90003	4.978116198	66.26522999	0.000190397	2.60635159	99695.10007	4.982487073	70.33367769
20 to 24	0.000638908	2.557580178	99439.4087	4.964216734	61.40803356	0.000190816	2.54932123	99600.24526	4.977676844	65.39815226
25 to 29	0.000622492	2.516253888	99122.44541	4.948469018	56.59541199	0.000228162	2.603773602	99505.28097	4.972542288	60.45812058
30 to 34	0.000681729	2.548592099	98814.71385	4.932481654	51.76321401	0.00030138	2.622889798	99391.85594	4.966022543	55.52402929
35 to 39	0.000767481	2.57870192	98478.89743	4.914816191	46.9305309	0.000393736	2.658593539	99242.23621	4.957539527	50.60362888
40 to 44	0.000979204	2.681230538	98102.23812	4.893988553	42.10017523	0.000599966	2.744707526	99047.0964	4.945643357	45.69778465
45 to 49	0.00163021	2.744275885	97623.83204	4.863291243	37.29232006	0.001082482	2.741154405	98750.49493	4.925460583	40.82627861
50 to 54	0.002928801	2.739688216	96832.57246	4.809742448	32.57273911	0.001842185	2.730946192	98217.64002	4.89042765	36.03195267
55 to 59	0.005074986	2.691465081	95427.36287	4.716132919	28.0086299	0.003142304	2.713532547	97317.57799	4.831045207	31.33842252
60 to 64	0.007875602	2.672540863	93040.98915	4.56855535	23.65321661	0.005005989	2.68446586	95802.06704	4.735222785	26.78860797
65 to 69	0.012507334	2.670386862	89456.88946	4.346717859	19.48700639	0.007796928	2.676189762	93436.04608	4.58870546	22.39557673
70 to 74	0.020103975	2.674920038	84053.13041	4.016331064	15.55986702	0.012095597	2.696907452	89870.95557	4.372066005	18.17343058
75 to 79	0.035216444	2.670772219	76048.24901	3.517667469	11.905886	0.020938165	2.764621144	84610.33369	4.041996186	14.12981731
80 to 84	0.067104051	2.583873299	63847.88975	2.756379736	8.66392973	0.045047905	2.696075795	76229.03169	3.45687115	10.37491359
85 to 89	0.122802562	2.457706002	45807.54673	1.759312754	6.061310225	0.090272091	2.579372484	60882.62507	2.508452001	7.289160894
90 to 94	0.21702433	2.20453248	24902.04961	0.78408338	4.164585656	0.170898827	2.319200033	38786.20666	1.34144694	4.998006631
95 plus	0.314555226	3.189171472	8369.061268	0.27215926	3.189171472	0.269700205	3.727711325	16457.6543	0.625742563	3.727711325

Table 15: Argentina 2017 life table, by age and sex. mx=mortality rate, ax=mean person-years lived in an age interval among those who die in that age interval, lx=number of persons left alive at age x, nLx=person-years lived between age x and x+n, ex=life expectancy at age x.

Age Group	Male					Female				
	mx	ax	lx	nLx	ex	mx	ax	lx	nLx	ex
Early Neonatal	0.273693268	0.009580652	100000	0.019127839	73.53308944	0.209807112	0.009582611	100000	0.019139551	79.71611509
Late Neonatal	0.033422538	0.028757904	99476.49263	0.05717806	73.90080722	0.028200549	0.028759344	99598.44381	0.057256754	80.01828049
Post Neonatal	0.003546757	0.46139188	99285.39654	0.915190607	73.9854508	0.003040857	0.461427818	99436.98164	0.916801843	80.09062953
1 to 4	0.000452038	1.999397283	98960.81818	3.954856384	73.30331198	0.000373968	1.999501376	99158.20757	3.963363366	79.3912159
5 to 9	0.000204884	2.499573158	98782.05522	4.936573906	69.43235196	0.000162187	2.49966211	99009.99789	4.948493255	75.50706725
10 to 14	0.000265599	3.128961277	98680.91835	4.9315958	64.50095266	0.000185559	2.765363286	98929.74318	4.944437346	70.56629413
15 to 19	0.001010582	2.736353627	98549.94056	4.916251593	59.58248811	0.000399627	2.645105023	98837.99663	4.937254082	65.62920564
20 to 24	0.001422492	2.54562862	98053.12684	4.885599589	54.87024058	0.000465707	2.555093877	98640.69381	4.926425952	60.75509835
25 to 29	0.001337322	2.507251975	97358.19314	4.85173683	50.24336122	0.000525332	2.593409632	98411.27321	4.914351102	55.8906651
30 to 34	0.001488975	2.560392316	96709.4165	4.817972423	45.56329328	0.000704718	2.64150782	98153.1179	4.899513801	51.03071369
35 to 39	0.001793758	2.607363775	95992.1201	4.779099799	40.88430144	0.00101063	2.665397563	97807.86145	4.878882567	46.20135359
40 to 44	0.002452149	2.675927347	95135.01958	4.729803018	36.22873756	0.001519269	2.681541175	97314.83402	4.848663443	41.42165606
45 to 49	0.00394496	2.706088963	93975.49584	4.65664812	31.64218799	0.002361581	2.685863893	96578.29116	4.802670707	36.71675523
50 to 54	0.00655867	2.698172655	92139.13195	4.538468329	27.21809528	0.003693568	2.682004952	95444.31317	4.731712775	32.12061115
55 to 59	0.010727648	2.6787878	89164.04412	4.349949373	23.03513834	0.005754608	2.675573475	93697.0829	4.623035727	27.6689435
60 to 64	0.01718191	2.644975661	84500.99525	4.060881704	19.1571516	0.008946574	2.659248211	91037.72594	4.458564517	23.39821403
65 to 69	0.026084924	2.613613203	77530.68084	3.649661594	15.6403503	0.01353481	2.65274252	87051.05265	4.21863646	19.34698579
70 to 74	0.039160295	2.587212619	68024.52725	3.108157929	12.45989817	0.021098254	2.657607863	81346.07077	3.875987877	15.51666395
75 to 79	0.059978547	2.558467582	55879.14468	2.438010239	9.606179589	0.035188995	2.660778258	73179.37795	3.381164543	11.95074597
80 to 84	0.098249771	2.489721164	41301.24822	1.657790464	7.097292744	0.065487758	2.600412561	61305.59965	2.649970477	8.750096686
85 to 89	0.161301098	2.349191593	25075.66586	0.879340008	5.087887376	0.120003684	2.473674947	43999.6573	1.68948374	6.171951153
90 to 94	0.256155974	2.133954527	10944.85183	0.316019862	3.639429414	0.20629573	2.25926411	23789.33183	0.760734008	4.322562122
95 plus	0.346231385	2.88875629	2873.588125	0.083175975	2.88875629	0.302784357	3.303830811	8141.387404	0.269571199	3.303830811

Table 15: Argentina 2100 life table, by age and sex. mx=mortality rate, ax=mean person-years lived in an age interval among those who die in that age interval, lx=number of persons left alive at age x, nLx=person-years lived between age x and x+n, ex=life expectancy at age x.

Age Group	Male					Female				
	mx	ax	lx	nLx	ex	mx	ax	lx	nLx	ex
Early Neonatal	0.060090298	0.009587199	100000	0.019167037	80.44655252	0.046509224	0.009587616	100000	0.019169532	84.5705834
Late Neonatal	0.010325231	0.028764275	99884.83633	0.057450924	80.52012211	0.010863115	0.028764127	99910.85072	0.057464997	84.62681335
Post Neonatal	0.000673142	0.461596017	99825.52534	0.921390461	80.51048749	0.00061266	0.461600313	99848.43348	0.921627631	84.62212149
1 to 4	9.4674E-05	1.999873768	99763.51429	3.989785181	79.63695737	8.20241E-05	1.999890634	99791.98009	3.991024574	83.74641514
5 to 9	5.78293E-05	2.499879522	99725.7475	4.98556654	75.66636137	6.47448E-05	2.499865115	99759.25069	4.987155373	79.77322776
10 to 14	9.95395E-05	3.411402754	99696.91564	4.984025192	70.68750885	9.18485E-05	2.846547177	99726.9662	4.985346665	74.79823254
15 to 19	0.000492573	2.742467448	99647.30633	4.97680886	65.72102513	0.00020249	2.608100199	99681.18566	4.981650599	69.83127243
20 to 24	0.000687421	2.544336301	99402.21664	4.961731835	60.87562921	0.000200876	2.548646297	99580.32446	4.976554898	64.89933181
25 to 29	0.000636478	2.503005105	99061.38409	4.945212169	56.07538425	0.00023902	2.59924169	99480.37772	4.971164347	59.96196952
30 to 34	0.000692657	2.540744545	98746.99033	4.928939979	51.24525468	0.000312745	2.62294966	99361.59144	4.964372062	55.03043527
35 to 39	0.00075206	2.573514235	98406.10348	4.911344025	46.413382	0.000406977	2.661547276	99206.38822	4.955599496	50.11230434
40 to 44	0.00095872	2.694479565	98037.3385	4.891032237	41.57755229	0.000625534	2.759247122	99004.76806	4.943279014	45.20870268
45 to 49	0.001647748	2.762991011	97569.26308	4.86051437	36.76296744	0.00115906	2.749234396	98695.6819	4.921917373	40.34123295
50 to 54	0.0030737	2.748441603	96769.95183	4.80517213	32.04180395	0.001997798	2.734058633	98125.5344	4.88414204	35.55892188
55 to 59	0.005391612	2.694151167	95296.7087	4.706347061	27.49114119	0.0034158	2.720171344	97150.70497	4.819830655	30.88709978
60 to 64	0.008468255	2.666432997	92766.49956	4.548723492	23.16264624	0.005521308	2.681640171	95507.25069	4.714975302	26.36943806
65 to 69	0.013227008	2.671194608	88930.22638	4.314190028	19.03930332	0.008449691	2.675257479	92909.17464	4.55592113	22.02883273
70 to 74	0.021649602	2.673032888	83260.92943	3.964907309	15.14483398	0.013105713	2.690878332	89074.67959	4.323075372	17.85830432
75 to 79	0.038048074	2.662492691	74755.40882	3.43661956	11.55204714	0.022183432	2.762878176	83440.71179	3.975082952	13.87801839
80 to 84	0.072425203	2.570663163	61901.13534	2.643206759	8.390262429	0.047640788	2.688502293	74714.73447	3.368878149	10.17450083
85 to 89	0.129491207	2.438810182	43310.06135	1.642493598	5.88912969	0.094019355	2.566263701	58906.30489	2.408459007	7.150675933
90 to 94	0.223866541	2.192595693	22866.38296	0.712336648	4.071343395	0.175335688	2.311724004	36888.95944	1.266469375	4.914122991
95 plus	0.320102741	3.135807662	7463.504171	0.239587122	3.135807662	0.273834706	3.6750739	15355.45995	0.577422855	3.6750739

Table 15: Chile 2017 life table, by age and sex. mx=mortality rate, ax=mean person-years lived in an age interval among those who die in that age interval, lx=number of persons left alive at age x, nLx=person-years lived between age x and x+n, ex=life expectancy at age x.

Age Group	Male					Female				
	mx	ax	lx	nLx	ex	mx	ax	lx	nLx	ex
Early Neonatal	0.215480572	0.009582437	100000	0.019138511	76.91157187	0.16283296	0.00958405	100000	0.019148169	82.02134884
Late Neonatal	0.017798703	0.028762214	99587.62282	0.057267665	77.21079025	0.016068008	0.028762691	99688.2139	0.057328362	82.25865647
Post Neonatal	0.001866473	0.461511244	99485.70517	0.917748335	77.23232395	0.001716473	0.4615219	99596.10486	0.918830327	82.27717192
1 to 4	0.000254912	1.999660118	99314.4302	3.970552819	76.44144312	0.000249032	1.999667957	99438.40059	3.97555575	81.48363893
5 to 9	0.000145316	2.499697257	99213.22814	4.958859823	72.51738283	0.000123427	2.499742861	99339.40352	4.965437911	77.56285335
10 to 14	0.000181133	3.028777555	99141.17354	4.955289558	67.5682738	0.000150054	2.687183697	99278.11925	4.962184252	72.60919142
15 to 19	0.000606394	2.748913721	99051.42133	4.945820414	62.62673663	0.000258946	2.615382089	99203.66112	4.957122586	67.66165235
20 to 24	0.00090992	2.612048032	98751.51714	4.926870528	57.80843315	0.000294461	2.574777931	99075.30036	4.950230258	62.74586711
25 to 29	0.001103325	2.565266761	98303.22781	4.901993618	53.05992435	0.000365877	2.592213835	98929.53875	4.94212343	57.83445073
30 to 34	0.001267407	2.575616967	97762.40988	4.873148835	48.33902066	0.000457951	2.667739279	98748.72366	4.932169185	52.93550995
35 to 39	0.001581743	2.614992543	97144.83637	4.83899021	43.62968614	0.000738129	2.671971956	98522.86472	4.917693144	48.05063134
40 to 44	0.002170468	2.653649038	96379.53286	4.794564479	38.95502226	0.001073214	2.689919038	98159.90119	4.895857048	43.2182256
45 to 49	0.003240651	2.668132985	95339.08931	4.731210634	34.35073139	0.001730382	2.689139597	97634.52427	4.862284784	38.43605036
50 to 54	0.004911848	2.671503476	93806.30175	4.637294283	29.86780666	0.002679117	2.694754571	96793.28331	4.809962229	33.74636137
55 to 59	0.007575673	2.672631802	91529.44668	4.497215444	25.54354676	0.004326417	2.688479268	95504.90085	4.727974167	29.16475038
60 to 64	0.011889967	2.673754642	88124.38439	4.287703426	21.42633133	0.006821899	2.696806776	93459.99963	4.600742152	24.74333954
65 to 69	0.019282544	2.640779144	83030.08919	3.97103455	17.57588295	0.011389681	2.669626698	90322.82283	4.399449379	20.50815312
70 to 74	0.029145053	2.619686688	75381.06545	3.524881393	14.09040673	0.017620678	2.672469247	85315.55812	4.09789125	16.55389816
75 to 79	0.046271122	2.596640408	65124.042	2.930974475	10.89674192	0.030111892	2.670426504	78103.25105	3.649571865	12.83455694
80 to 84	0.07625533	2.534784743	51593.23909	2.172470096	8.075051569	0.054497029	2.627450753	67133.89646	2.973209323	9.494836838
85 to 89	0.134976681	2.420544839	35075.84077	1.301947167	5.689134375	0.104663897	2.524486738	50972.8644	2.025485636	6.674058344
90 to 94	0.230212145	2.184952864	17554.90832	0.533148794	3.961530925	0.188819176	2.293852446	29837.90279	0.988410052	4.620242763
95 plus	0.325470342	3.07301261	5310.265351	0.163446813	3.07301261	0.286668442	3.489725863	11228.28982	0.392687582	3.489725863

Table 15: Chile 2100 life table, by age and sex. mx=mortality rate, ax=mean person-years lived in an age interval among those who die in that age interval, lx=number of persons left alive at age x, nLx=person-years lived between age x and x+n, ex=life expectancy at age x.

Age Group	Male					Female				
	mx	ax	lx	nLx	ex	mx	ax	lx	nLx	ex
Early Neonatal	0.065561414	0.009587032	100000	0.019166033	82.89933075	0.049132006	0.009587535	100000	0.019169051	86.67113957
Late Neonatal	0.004819651	0.028765794	99874.37953	0.057454008	82.98429702	0.005522867	0.0287656	99905.84069	0.057470943	86.73356629
Post Neonatal	0.000386957	0.461616347	99846.69741	0.921707622	82.94973569	0.000380789	0.461616785	99874.10609	0.921963252	86.70356208
1 to 4	6.57322E-05	1.999912357	99811.04002	3.991917022	82.05587489	7.0766E-05	1.999905645	99839.00486	3.992995267	85.81054766
5 to 9	4.94184E-05	2.499897045	99784.81443	4.988624906	78.07678015	5.85788E-05	2.499877961	99810.76226	4.989807882	81.83414517
10 to 14	8.03463E-05	3.283108381	99760.18543	4.987244055	73.09530269	8.61882E-05	2.806997506	99781.55675	4.988095689	76.85728567
15 to 19	0.00028485	2.74057779	99720.17446	4.98278406	68.1232362	0.000146857	2.587895125	99738.60796	4.985166064	71.88912628
20 to 24	0.000400797	2.626052679	99578.37063	4.974162017	63.21566457	0.000148644	2.563857931	99665.44847	4.981466387	66.93984173
25 to 29	0.000503247	2.581509255	99379.25585	4.962909614	58.33619534	0.000189152	2.611975333	99591.4659	4.97731613	61.98751556
30 to 34	0.000582586	2.586229247	99129.85372	4.949530842	53.47573386	0.000239941	2.636218488	99497.413	4.97204065	57.04340157
35 to 39	0.000741273	2.598729963	98841.98767	4.933358101	48.62319535	0.000334287	2.661789896	99378.23484	4.965019163	52.10835796
40 to 44	0.000977197	2.657691665	98476.99322	4.912646836	43.7926963	0.000485382	2.724754824	99212.44386	4.955129274	47.19035253
45 to 49	0.001528142	2.70422386	97998.32173	4.882776984	38.99167352	0.000835273	2.725178896	98972.2119	4.93916998	42.29739434
50 to 54	0.002467953	2.717259514	97255.16683	4.835449962	34.26581477	0.001330006	2.731319964	98560.23859	4.913167399	37.46105555
55 to 59	0.004021299	2.688274039	96068.13062	4.759089196	29.65073791	0.002296205	2.703407518	97907.82229	4.869655674	32.69023913
60 to 64	0.005972714	2.700054508	94166.51156	4.644745365	25.18935487	0.003523139	2.715420143	96792.22775	4.801022329	28.03221424
65 to 69	0.010199655	2.676492071	91409.60469	4.465282394	20.86122817	0.00606541	2.693837574	95105.6884	4.689839119	23.47574312
70 to 74	0.015713185	2.689917526	86895.26417	4.193921041	16.79793307	0.009477097	2.732405685	92274.37002	4.517076263	19.10564369
75 to 79	0.027859595	2.706229363	80384.0846	3.780937444	12.93337947	0.017751805	2.795162053	88025.75445	4.236374776	14.88570273
80 to 84	0.054669565	2.629278541	70028.92997	3.10759051	9.443182	0.039194285	2.736160022	80599.3235	3.706674307	10.98944297
85 to 89	0.10617693	2.507555344	53427.00429	2.125461849	6.562336913	0.081012873	2.616129264	66391.75094	2.796657679	7.731141614
90 to 94	0.199081523	2.230111977	31523.13997	1.026055204	4.43966761	0.158900035	2.33155435	44512.04549	1.580710172	5.275954873
95 plus	0.29975125	3.346650757	11633.40938	0.396515684	3.346650757	0.258251753	3.903482637	20316.58733	0.815152125	3.903482637

Table 15: Uruguay 2017 life table, by age and sex. mx=mortality rate, ax=mean person-years lived in an age interval among those who die in that age interval, lx=number of persons left alive at age x, nLx=person-years lived between age x and x+n, ex=life expectancy at age x.

Age Group	Male					Female				
	mx	ax	lx	nLx	ex	mx	ax	lx	nLx	ex
Early Neonatal	0.215299162	0.009582442	100000	0.019138544	73.47277703	0.15068703	0.009584423	100000	0.019150398	80.39965641
Late Neonatal	0.027891307	0.028759429	99587.96475	0.057251241	73.75752711	0.02414743	0.028760462	99711.43457	0.057328392	80.61311144
Post Neonatal	0.003644373	0.461384945	99428.29837	0.916466584	73.8183954	0.002412851	0.461472431	99573.00867	0.918322062	80.66760453
1 to 4	0.000319431	1.999574092	99094.32863	3.961242232	73.14236065	0.000321758	1.99957099	99351.43907	3.971501471	79.92318937
5 to 9	0.000172373	2.49964089	98967.81022	4.946258887	69.23332209	0.0001289	2.499731459	99223.66216	4.959584782	76.02354123
10 to 14	0.00024164	3.192043246	98882.55758	4.94196889	64.29086382	0.000162065	2.787430617	99159.736	4.956210101	71.07094223
15 to 19	0.000977973	2.777217325	98763.14578	4.927446717	59.36471432	0.000353478	2.654544937	99079.41488	4.949867634	66.12627242
20 to 24	0.001554285	2.580844772	98281.26641	4.895655727	54.64191858	0.00042591	2.539506344	98904.45096	4.940046221	61.23846144
25 to 29	0.001599799	2.509212679	97520.37699	4.856667311	50.04773857	0.000436298	2.667629776	98694.05561	4.929686709	56.36347272
30 to 34	0.001650439	2.551950044	96743.46938	4.817711331	45.42915578	0.0007804	2.655862681	98478.98427	4.914959505	51.48060934
35 to 39	0.002043884	2.602592413	95948.42724	4.774033675	40.78409978	0.001026992	2.675042227	98095.44682	4.893089985	46.67127757
40 to 44	0.002704822	2.650580882	94972.84034	4.718663737	36.17587148	0.001657497	2.654101659	97592.98594	4.860750098	41.89747021
45 to 49	0.00409112	2.682827336	93696.84514	4.640861384	31.63187533	0.002279938	2.679338053	96787.44867	4.813905329	37.22362886
50 to 54	0.006505489	2.706888499	91798.89005	4.522505591	27.2296395	0.00368765	2.679328133	95690.15595	4.7439203	32.61921553
55 to 59	0.01112525	2.674930625	88858.25305	4.33094968	23.04018333	0.005613657	2.657390344	93941.31601	4.636121791	28.17584078
60 to 64	0.017296774	2.644946259	84043.33154	4.037828716	19.20567949	0.008291325	2.659646926	91339.9428	4.480114706	23.90165829
65 to 69	0.026708286	2.613781603	77065.86323	3.622710521	15.70388316	0.012810087	2.661152793	87627.74875	4.254051521	19.80042591
70 to 74	0.039927026	2.576228909	67403.49516	3.073331801	12.57957493	0.020218387	2.656411252	82183.61516	3.923534047	15.93450451
75 to 79	0.058778972	2.547224069	55157.27752	2.411209729	9.801095783	0.033097958	2.65674245	74263.05779	3.446424714	12.34954279
80 to 84	0.093115941	2.492205823	41024.91714	1.664058479	7.303345841	0.060033126	2.604091027	62882.65695	2.749817926	9.103722789
85 to 89	0.155263589	2.364973684	25584.36547	0.908796193	5.215170725	0.112440259	2.498210119	46426.81394	1.813195937	6.410629102
90 to 94	0.250334297	2.145856431	11522.15147	0.3364501	3.707513584	0.197773063	2.276611492	26112.55332	0.849657588	4.463517796
95 plus	0.341616739	2.927732122	3122.064208	0.091569965	2.927732122	0.294956727	3.391743791	9364.411985	0.318415193	3.391743791

Table 15: Uruguay 2100 life table, by age and sex. mx=mortality rate, ax=mean person-years lived in an age interval among those who die in that age interval, lx=number of persons left alive at age x, nLx=person-years lived between age x and x+n, ex=life expectancy at age x.

Age Group	Male					Female				
	mx	ax	lx	nLx	ex	mx	ax	lx	nLx	ex
Early Neonatal	0.047848649	0.009587575	100000	0.019169289	80.01880219	0.033848864	0.009588004	100000	0.01917186	85.40933593
Late Neonatal	0.00641375	0.028765354	99908.32563	0.057470903	80.07321723	0.00726335	0.02876512	99935.1247	0.057484912	85.44549865
Post Neonatal	0.000771729	0.461589013	99871.4834	0.921772733	80.04532798	0.000460376	0.461611131	99893.38234	0.922107319	85.42361397
1 to 4	6.94994E-05	1.999907334	99800.36777	3.991460038	79.1787632	7.44181E-05	1.999900775	99850.93698	3.993443261	84.53642342
5 to 9	5.62249E-05	2.499882865	99772.63581	4.987930634	75.20021842	5.79366E-05	2.499879299	99821.22825	4.990338902	80.56099167
10 to 14	9.86189E-05	3.216659573	99744.59204	4.986325866	70.22057755	9.0716E-05	2.990303504	99792.33146	4.988603931	75.58357456
15 to 19	0.000579144	2.890937618	99695.4312	4.97826216	65.25354534	0.000186977	2.636313497	99747.1165	4.985144429	70.61655685
20 to 24	0.001057288	2.586641031	99407.53955	4.957672816	60.43108566	0.000223874	2.554273432	99653.9605	4.979961042	65.68000099
25 to 29	0.001027383	2.491920748	98884.84493	4.931584712	55.73333406	0.000236624	2.726990001	99542.59221	4.974392016	60.75047391
30 to 34	0.001039114	2.538404159	98380.55297	4.906487146	51.00356527	0.000435923	2.644720708	99425.03176	4.96608687	55.81887282
35 to 39	0.001199254	2.567378939	97874.18923	4.879470582	46.25259438	0.000491549	2.660990325	99208.84832	4.954701314	50.93407901
40 to 44	0.001371234	2.603858105	97293.5188	4.848718633	41.5117444	0.000799901	2.678261132	98965.5917	4.939023683	46.05187203
45 to 49	0.001933668	2.657454329	96632.66869	4.809823837	36.77623267	0.001144721	2.71460495	98571.10174	4.915655605	41.22420893
50 to 54	0.003098582	2.708390452	95708.83258	4.751654478	32.10292943	0.002042085	2.701885145	98009.23596	4.877512174	36.44302384
55 to 59	0.005699033	2.669010376	94246.5064	4.650616556	27.55404194	0.003227505	2.669107921	97015.58566	4.814416239	31.78422983
60 to 64	0.008785788	2.648155126	91617.33082	4.48856068	23.25916464	0.004725669	2.648245032	95467.20091	4.720992916	27.25104007
65 to 69	0.014189266	2.622004167	87706.27459	4.24345581	19.16845326	0.007083005	2.661489718	93244.01984	4.58656951	22.83147474
70 to 74	0.021707543	2.623749864	81752.32215	3.889909797	15.36057006	0.011307605	2.700043591	90013.81278	4.387418842	18.547097
75 to 79	0.036781905	2.618951171	73458.02752	3.383310301	11.78460257	0.020669034	2.732421295	85099.151	4.066468558	14.44960178
80 to 84	0.069595465	2.536966224	61323.82835	2.633266438	8.592543173	0.042572941	2.668669289	76832.55447	3.502403757	10.69476487
85 to 89	0.125166919	2.452198028	43771.10996	1.681703867	6.02970096	0.085917438	2.598123638	62353.14986	2.602553354	7.530921552
90 to 94	0.21906945	2.198711552	23848.99418	0.753546386	4.148925915	0.164906504	2.322905756	40961.62581	1.441660387	5.15282234
95 plus	0.316110269	3.180222814	8116.082247	0.266628526	3.180222814	0.263899826	3.825962172	18267.90476	0.723225051	3.825962172

Table 15: Western Europe 2017 life table, by age and sex. mx=mortality rate, ax=mean person-years lived in an age interval among those who die in that age interval, lx=number of persons left alive at age x, nLx=person-years lived between age x and x+n, ex=life expectancy at age x.

Age Group	Male					Female				
	mx	ax	lx	nLx	ex	mx	ax	lx	nLx	ex
Early Neonatal	0.090161924	0.009586278	100000	0.019161511	79.53552017	0.073831131	0.009586778	100000	0.019164511	84.2960091
Late Neonatal	0.010748676	0.028764158	99827.23633	0.057417093	79.65397207	0.008798835	0.028764696	99858.50636	0.057438299	84.39625995
Post Neonatal	0.001162135	0.461561279	99765.5207	0.920628755	79.6456946	0.000952889	0.461576144	99807.96744	0.921109408	84.38144625
1 to 4	0.000162198	1.999783736	99658.53145	3.985048391	78.80741585	0.000138855	1.99981486	99720.19609	3.98770032	83.53202299
5 to 9	8.28287E-05	2.49982744	99593.8951	4.978663745	74.85726412	6.54983E-05	2.499863545	99664.82502	4.982425357	79.57732016
10 to 14	9.25745E-05	2.966383471	99552.65756	4.976695959	69.8872367	7.46459E-05	2.72760513	99632.19102	4.980764699	74.6025665
15 to 19	0.000290367	2.779324125	99506.58609	4.972123235	64.91822059	0.000147161	2.671799168	99595.01166	4.978045018	69.62939731
20 to 24	0.000482925	2.614973574	99362.21211	4.962394974	60.00850498	0.00019622	2.590819554	99521.75443	4.973736497	64.67868242
25 to 29	0.000558902	2.576387613	99122.56601	4.949424007	55.1472577	0.000233081	2.622237966	99424.15978	4.96845442	59.73962527
30 to 34	0.000691253	2.603437086	98845.94212	4.934123121	50.29437247	0.000333657	2.665415088	99308.35444	4.961552935	54.80622767
35 to 39	0.000907411	2.662004799	98504.87017	4.914816664	45.45949349	0.000499363	2.692957486	99142.80876	4.951436135	49.89328615
40 to 44	0.001407534	2.704478473	98058.89572	4.887154457	40.65412627	0.000799367	2.722930653	98895.55299	4.935793487	45.01128746
45 to 49	0.002315086	2.717670968	97371.0157	4.842962016	35.92220534	0.001363523	2.716445678	98501.00334	4.909762878	40.18066407
50 to 54	0.003898258	2.70886861	96249.83873	4.769890613	31.30896099	0.002240045	2.694201662	97831.54971	4.866442074	35.43700967
55 to 59	0.006419517	2.685996924	94390.44416	4.650442115	26.87230341	0.003513257	2.674877202	96741.45501	4.797880489	30.80592896
60 to 64	0.010134846	2.656901896	91405.16746	4.46424933	22.66216021	0.005336107	2.668824956	95055.86231	4.694398724	26.30472486
65 to 69	0.015243632	2.645968941	86880.89697	4.193570048	18.70385465	0.008181472	2.679482302	92550.9405	4.541331467	21.94438491
70 to 74	0.023614569	2.646737889	80488.71486	3.812581793	14.97906345	0.013236833	2.701555148	88835.59426	4.310640072	17.75003004
75 to 79	0.038903792	2.636698208	71486.16346	3.273387652	11.53207373	0.023409272	2.717998839	83129.98522	3.945737775	13.78279524
80 to 84	0.068928948	2.583884621	58753.2609	2.518331554	8.459894789	0.046054296	2.674864195	73894.35819	3.337393015	10.16563162
85 to 89	0.125935517	2.446446667	41397.72888	1.566292059	5.923595312	0.092380263	2.56826834	58526.73814	2.389629474	7.132560512
90 to 94	0.220955556	2.201645481	21676.19475	0.669758638	4.087758063	0.174224531	2.319293627	36455.7672	1.242573321	4.896226887
95 plus	0.317950393	3.145183551	6879.734078	0.216402443	3.145183551	0.273021904	3.662827244	14811.41129	0.542598723	3.662827244

Table 15: Western Europe 2100 life table, by age and sex. mx=mortality rate, ax=mean person-years lived in an age interval among those who die in that age interval, lx=number of persons left alive at age x, nLx=person-years lived between age x and x+n, ex=life expectancy at age x.

Age Group	Male					Female				
	mx	ax	lx	nLx	ex	mx	ax	lx	nLx	ex
Early Neonatal	0.024976854	0.009588276	100000	0.01917349	84.43597681	0.022191557	0.009588361	100000	0.019174002	87.17342897
Late Neonatal	0.002881119	0.028766329	99952.11184	0.057501929	84.45725987	0.002775087	0.028766358	99957.45087	0.057505175	87.19135404
Post Neonatal	0.000275506	0.461624264	99935.54521	0.922575228	84.41372954	0.000239815	0.4616268	99941.4929	0.922645335	87.14773871
1 to 4	5.0194E-05	1.999933075	99910.12848	3.996003982	83.51179938	4.8498E-05	1.999935336	99919.36697	3.996387039	86.24364788
5 to 9	3.24857E-05	2.499932322	99890.07134	4.99409797	79.52816709	3.34982E-05	2.499930212	99899.98567	4.994581005	82.25999085
10 to 14	4.38718E-05	3.269377118	99873.848	4.993296216	74.54066608	4.72725E-05	2.822409647	99883.2551	4.993640162	77.27334738
15 to 19	0.00017819	2.844548794	99851.94244	4.990672874	69.55630565	9.84627E-05	2.691157769	99859.64992	4.99184505	72.29095454
20 to 24	0.000331756	2.654892739	99763.0206	4.984268814	64.61566532	0.000135132	2.606689287	99810.50162	4.988907805	67.3252058
25 to 29	0.000421041	2.596154837	99597.69183	4.974846663	59.71831321	0.000164074	2.626415444	99743.0908	4.985209018	62.36892868
30 to 34	0.000524005	2.578011259	99388.28835	4.963109739	54.83842423	0.000230911	2.627295012	99661.30504	4.980332043	57.41790516
35 to 39	0.000611974	2.613033896	99128.32363	4.949181927	49.97524129	0.000301044	2.657247665	99546.31568	4.973806324	52.4810804
40 to 44	0.000852816	2.663206869	98825.58944	4.931446737	45.1200812	0.000457425	2.735659088	99396.5978	4.964683815	47.55593437
45 to 49	0.001278888	2.711262932	98405.2568	4.905903288	40.30105203	0.000817171	2.740018104	99169.53492	4.949334647	42.65818959
50 to 54	0.002163877	2.720025429	97778.24374	4.864915808	35.54132046	0.001404554	2.72222276	98765.17831	4.922515417	37.82089876
55 to 59	0.003614272	2.677589252	96726.33955	4.796089556	30.89662848	0.002341227	2.686608072	98074.04382	4.877302538	33.06690816
60 to 64	0.005385211	2.662461794	94994.73841	4.690777131	26.40813256	0.003566977	2.668754262	96932.95286	4.806728448	28.42232617
65 to 69	0.008164309	2.665276463	92472.78301	4.537396399	22.05160457	0.005388844	2.684604959	95220.56052	4.702470346	23.88213966
70 to 74	0.012717085	2.703369308	88778.41588	4.313489992	17.85255014	0.008718342	2.733653472	92691.97514	4.545062937	19.45585128
75 to 79	0.022869499	2.721054313	83320.57561	3.961064159	13.83726863	0.016264408	2.790280344	88744.2445	4.284030072	15.19319198
80 to 84	0.044734293	2.673415572	74338.91039	3.37103109	10.17257759	0.036155385	2.7406595	81820.08491	3.785277043	11.23342941
85 to 89	0.092115346	2.55153167	59484.10594	2.435562623	7.041704653	0.076530885	2.632208302	68309.87274	2.899627097	7.899761008
90 to 94	0.183177201	2.247695563	37495.18875	1.254277347	4.706536359	0.15355506	2.340821812	46558.21357	1.663470285	5.377368786
95 plus	0.28645747	3.499536363	14937.57728	0.529293851	3.499536363	0.253250195	3.967100679	21558.31556	0.868893362	3.967100679

Table 15: Andorra 2017 life table, by age and sex. mx=mortality rate, ax=mean person-years lived in an age interval among those who die in that age interval, lx=number of persons left alive at age x, nLx=person-years lived between age x and x+n, ex=life expectancy at age x.

Age Group	Male					Female				
	mx	ax	lx	nLx	ex	mx	ax	lx	nLx	ex
Early Neonatal	0.05330963	0.009587407	100000	0.019168282	80.36208804	0.038528966	0.00958786	100000	0.019170999	84.93068829
Late Neonatal	0.005917321	0.028765491	99897.81532	0.057465673	80.42509264	0.004608507	0.028765852	99926.1367	0.057484129	84.97428128
Post Neonatal	0.000694447	0.461594503	99863.8116	0.921734739	80.39493086	0.000546491	0.461605014	99899.64559	0.922128459	84.93927284
1 to 4	0.00024538	1.999672827	99799.80344	3.990033759	79.52290546	0.000186326	1.999751565	99849.25346	3.992482197	84.05862061
5 to 9	8.35121E-05	2.499826016	99701.90157	4.984054457	75.59901482	5.66802E-05	2.499881916	99774.86583	4.988036471	80.11980331
10 to 14	9.41985E-05	2.95215672	99660.27963	4.982052515	70.62953411	6.32346E-05	2.798991566	99746.59436	4.986635635	75.14181509
15 to 19	0.000287834	2.841879393	99613.35032	4.977575149	65.6614135	0.00014747	2.696872206	99715.06206	4.98405999	70.16468595
20 to 24	0.000567439	2.617223832	99470.08062	4.966788857	60.75183261	0.000202484	2.5982797	99641.56289	4.979656546	65.21442666
25 to 29	0.00060976	2.543605573	99188.25096	4.951996746	55.91692191	0.000243406	2.606544956	99540.73457	4.974139278	60.27785363
30 to 34	0.000700103	2.596833022	98886.30881	4.936012323	51.07977476	0.000327985	2.656716249	99419.66285	4.96716718	55.34806209
35 to 39	0.000942002	2.647890519	98540.75812	4.916146804	46.2496514	0.000492963	2.689143587	99256.75012	4.957191232	50.43444799
40 to 44	0.001379977	2.689415685	98077.70415	4.88830077	41.45531594	0.000779228	2.701466337	99012.38761	4.941768582	45.55210086
45 to 49	0.002221523	2.69784499	97403.23461	4.845384748	36.72349586	0.001254088	2.708895168	98627.3362	4.91724011	40.71911222
50 to 54	0.003550157	2.695574679	96327.07601	4.777277148	32.10326773	0.002057636	2.690331153	98010.74002	4.87736254	35.95780577
55 to 59	0.005708436	2.677636224	94631.65496	4.669687368	27.62969557	0.003186163	2.672052687	97007.35055	4.8146693	31.30121533
60 to 64	0.008790306	2.658612703	91967.23688	4.505662615	23.35214449	0.004812304	2.67701639	95473.82189	4.72094622	26.76007099
65 to 69	0.013300329	2.659803047	88008.58113	4.267670289	19.28260396	0.007567954	2.689703865	93203.23767	4.58015674	22.34539857
70 to 74	0.021190424	2.668653984	82335.92363	3.923131424	15.42759659	0.012453251	2.708617	89740.23807	4.362694685	18.10207898
75 to 79	0.036368362	2.649850691	74029.5863	3.41028125	11.85918098	0.022183473	2.720837723	84316.12766	4.013323377	14.09012813
80 to 84	0.064595454	2.587148731	61639.9643	2.66682092	8.710906695	0.043332203	2.669601492	75436.06807	3.426934504	10.42666471
85 to 89	0.120217058	2.463341445	44433.23437	1.702972748	6.084072833	0.088105373	2.584778717	60643.08538	2.502225539	7.319534494
90 to 94	0.214945353	2.211636066	23984.06006	0.750088469	4.174655226	0.168855172	2.326595461	38703.70845	1.335410121	5.01136423
95 plus	0.313023409	3.194877949	7875.943955	0.251780989	3.194877949	0.267926647	3.735411257	16263.84697	0.60980446	3.735411257

Table 15: Andorra 2100 life table, by age and sex. mx=mortality rate, ax=mean person-years lived in an age interval among those who die in that age interval, lx=number of persons left alive at age x, nLx=person-years lived between age x and x+n, ex=life expectancy at age x.

Age Group	Male					Female				
	mx	ax	lx	nLx	ex	mx	ax	lx	nLx	ex
Early Neonatal	0.01326795	0.009588634	100000	0.019175643	83.88515015	0.008454696	0.009588782	100000	0.019176528	86.94064792
Late Neonatal	0.001456211	0.028766722	99974.56117	0.057517201	83.88730226	0.001255705	0.028766777	99983.78829	0.057522842	86.93556552
Post Neonatal	0.000191776	0.461630213	99966.18635	0.922893773	83.83678474	0.00013752	0.461634064	99976.56572	0.92301271	86.88430592
1 to 4	5.95833E-05	1.999920556	99948.48938	3.997463223	82.92826094	4.65793E-05	1.999937895	99963.87351	3.998182487	85.9720011
5 to 9	3.68032E-05	2.499923326	99924.67312	4.995773994	78.94755444	3.0209E-05	2.499937065	99945.25165	4.9968852	81.98765182
10 to 14	5.39636E-05	3.213547787	99906.28725	4.994825565	73.96161811	4.2131E-05	2.964594373	99930.1568	4.99607589	76.99965672
15 to 19	0.000214775	2.924433471	99879.3338	4.991723441	68.98071037	0.000120835	2.722255534	99909.10796	4.994079647	72.01525781
20 to 24	0.000474935	2.64239033	99772.12933	4.983024534	64.05164686	0.000169992	2.605877218	99848.76289	4.990403831	67.05712297
25 to 29	0.000538567	2.547795481	99535.48183	4.970210973	59.1975651	0.000204247	2.588419598	99763.93358	4.985737149	62.11191379
30 to 34	0.000601627	2.554129562	99267.83468	4.956098283	54.35019315	0.000253365	2.610537236	99662.10712	4.98008885	57.17268623
35 to 39	0.000696884	2.588471751	98969.71849	4.940183647	49.50612443	0.000337794	2.654685937	99535.93479	4.97285618	52.24174476
40 to 44	0.000901027	2.649482493	98625.53342	4.920849705	44.66974395	0.000504539	2.706509694	99367.96356	4.962653566	47.32534638
45 to 49	0.001345346	2.689501431	98182.28971	4.8939006	39.85927682	0.00083888	2.724406534	99117.605	4.946440249	42.43759986
50 to 54	0.002141247	2.711587363	97524.12373	4.852421796	35.10968894	0.001418583	2.717563726	98702.74498	4.919207286	37.60345291
55 to 59	0.003564215	2.679123684	96485.55306	4.78471603	30.45748981	0.002338099	2.690027491	98005.26667	4.873940842	32.84947261
60 to 64	0.00535242	2.671378864	94781.04643	4.680769497	25.95547614	0.003593051	2.685621143	96867.04902	4.80341153	28.2000588
65 to 69	0.008332921	2.681511927	92277.97534	4.526586408	21.58445687	0.005609875	2.702606004	95145.75495	4.696764164	23.65579211
70 to 74	0.013505723	2.719195766	88512.37137	4.293675532	17.38475049	0.009196116	2.748844059	92523.65083	4.532533578	19.24125269
75 to 79	0.025180897	2.714828386	82731.98502	3.912437474	13.40460037	0.017344388	2.79326277	88388.42726	4.257487143	15.00208223
80 to 84	0.048530124	2.648036992	72934.40228	3.275891188	9.836328108	0.038086353	2.720661235	81095.14299	3.736161625	11.08608505
85 to 89	0.097841356	2.532806268	57180.67585	2.308358412	6.815754833	0.079308823	2.622408492	67168.93361	2.839006538	7.80154404
90 to 94	0.190063264	2.243290279	34864.42554	1.148365495	4.578246987	0.156806338	2.334489206	45371.98122	1.616584103	5.319394717
95 plus	0.29229619	3.42595934	13275.86839	0.458287731	3.42595934	0.256280848	3.930843374	20887.04368	0.842435984	3.930843374

Table 15: Austria 2017 life table, by age and sex. mx=mortality rate, ax=mean person-years lived in an age interval among those who die in that age interval, lx=number of persons left alive at age x, nLx=person-years lived between age x and x+n, ex=life expectancy at age x.

Age Group	Male					Female				
	mx	ax	lx	nLx	ex	mx	ax	lx	nLx	ex
Early Neonatal	0.094554865	0.009586143	100000	0.019160704	79.42293199	0.076056471	0.00958671	100000	0.019164102	84.20651049
Late Neonatal	0.00723966	0.028765126	99818.82868	0.057418052	79.54788389	0.006114607	0.028765437	99854.24576	0.057440284	84.31023025
Post Neonatal	0.000939376	0.461577104	99777.26122	0.920831782	79.52347738	0.000754788	0.461590217	99819.1239	0.921296614	84.28235098
1 to 4	0.000158928	1.999788096	99690.76353	3.986363362	78.66878919	0.000115652	1.999845797	99749.58689	3.989060733	83.41749675
5 to 9	7.2949E-05	2.499848023	99627.41134	4.980462237	74.71754337	8.24698E-05	2.499828188	99703.45332	4.984145004	79.45516993
10 to 14	7.00121E-05	3.408469969	99591.08034	4.97899899	69.74388901	8.82791E-05	2.780743796	99662.34969	4.982141441	74.4869089
15 to 19	0.00037844	2.752868183	99556.2219	4.973581569	64.76711426	0.000201632	2.585440093	99618.36806	4.978494683	69.51856432
20 to 24	0.000530673	2.556799018	99368.00268	4.961966728	59.88455633	0.000171265	2.528895726	99517.98582	4.973794384	64.58606683
25 to 29	0.000525338	2.559874185	99104.68622	4.948890387	55.03684154	0.000225851	2.627725979	99432.80271	4.968977891	59.6392196
30 to 34	0.000685067	2.591841777	98844.70446	4.934095414	50.1748377	0.000310328	2.658238703	99320.57847	4.9624228	54.70362279
35 to 39	0.000832132	2.645835804	98506.69026	4.915705107	45.33807584	0.000462833	2.716849881	99166.582	4.953095093	49.78442359
40 to 44	0.001276803	2.724684277	98097.64645	4.890674746	40.51604396	0.000795073	2.698523429	98937.33921	4.93783141	44.89344745
45 to 49	0.002232604	2.708613313	97473.22122	4.84885636	35.75806659	0.001227715	2.7141339	98544.75287	4.913448642	40.06149261
50 to 54	0.003573454	2.733379257	96390.71137	4.780814257	31.12910504	0.002077333	2.716543565	97941.5382	4.873957923	35.29143997
55 to 59	0.00642165	2.70989364	94682.4242	4.665513906	26.64125854	0.003443009	2.695850488	96929.09653	4.80831136	30.63158119
60 to 64	0.010561963	2.680871159	91686.69762	4.474742091	22.42293437	0.005459991	2.6947676	95273.69536	4.704476932	26.11681033
65 to 69	0.016978815	2.646054847	86961.19726	4.180992821	18.49543306	0.00893319	2.687675833	92705.3034	4.54146878	21.76550186
70 to 74	0.025800401	2.604540145	79864.00562	3.760844587	14.90360311	0.014519204	2.657057972	88648.96007	4.286661283	17.63816655
75 to 79	0.037112433	2.631518846	70164.38598	3.224893041	11.60367636	0.022306638	2.724291469	82426.71551	3.922296395	13.76874245
80 to 84	0.069171821	2.59249735	58202.49168	2.494926927	8.447842163	0.04707771	2.685759641	73681.06323	3.322290094	10.07946982
85 to 89	0.126297382	2.445414935	40956.74419	1.548595062	5.914357162	0.093957503	2.562489116	58049.89401	2.361957001	7.070586095
90 to 94	0.221324126	2.200967907	21412.89656	0.661266999	4.082793897	0.176131524	2.316176029	35874.099	1.218266686	4.858678806
95 plus	0.318249322	3.142345674	6786.30351	0.213336863	3.142345674	0.274814193	3.639237611	14432.17419	0.525510959	3.639237611

Table 15: Austria 2100 life table, by age and sex. mx=mortality rate, ax=mean person-years lived in an age interval among those who die in that age interval, lx=number of persons left alive at age x, nLx=person-years lived between age x and x+n, ex=life expectancy at age x.

Age Group	Male					Female				
	mx	ax	lx	nLx	ex	mx	ax	lx	nLx	ex
Early Neonatal	0.026987364	0.009588214	100000	0.01917312	84.9697013	0.021495983	0.009588382	100000	0.01917413	87.76498193
Late Neonatal	0.001568534	0.028766691	99948.26135	0.057501885	84.99454226	0.001417663	0.028766732	99958.78601	0.057508189	87.78198289
Post Neonatal	0.000192828	0.461630137	99939.24269	0.922644576	84.94468987	0.000158532	0.461632574	99950.63369	0.922764346	87.73161398
1 to 4	4.15869E-05	1.99994455	99921.45259	3.996525697	84.03644036	3.13025E-05	1.999958262	99936.00543	3.997189974	86.82109948
5 to 9	2.5924E-05	2.499945996	99904.83281	4.994917924	80.05008186	4.42201E-05	2.499907874	99923.49356	4.99562243	82.83171657
10 to 14	3.34166E-05	3.235047698	99891.8846	4.994293782	75.06010601	5.81187E-05	2.979860415	99901.40526	4.994437175	77.84946523
15 to 19	0.00022043	2.694144397	99875.19854	4.991197649	70.07207412	0.000147312	2.595374512	99872.3855	4.991842585	72.87124668
20 to 24	0.000348169	2.556990968	99765.19583	4.984010662	65.14604633	0.000126129	2.55433031	99798.87368	4.988386766	67.92282896
25 to 29	0.000356102	2.547724626	99591.73949	4.97522873	60.25441865	0.000158325	2.59448547	99735.98021	4.984885734	62.9639421
30 to 34	0.000451057	2.539453548	99414.68489	4.965205184	55.35661601	0.000185674	2.589171162	99657.08836	4.980618009	58.01159309
35 to 39	0.000454913	2.546605875	99190.91008	4.954012031	50.47521997	0.00023609	2.669943509	99564.64954	4.975485154	53.06281257
40 to 44	0.000604647	2.628222299	98965.70606	4.941189977	45.58387022	0.000382727	2.707124824	99447.24153	4.967988782	48.1219117
45 to 49	0.001046712	2.656844481	98667.22223	4.921253677	40.71319601	0.000628575	2.756275559	99257.20906	4.955857691	43.20819872
50 to 54	0.001814315	2.692335278	98152.82344	4.887073979	35.91128065	0.001201781	2.768861811	98945.93498	4.93400054	38.33438284
55 to 59	0.003544867	2.636348055	97267.73186	4.822981554	31.21111773	0.002225732	2.723920898	98353.74228	4.892843788	33.54646157
60 to 64	0.005611269	2.610185225	95561.48106	4.714964049	26.71720677	0.003716374	2.688274405	97266.72845	4.821913701	28.88756039
65 to 69	0.008612553	2.603956554	92925.91605	4.55267097	22.39417904	0.005883704	2.659523453	95479.64366	4.709293577	24.3730941
70 to 74	0.013178976	2.590825221	89029.64671	4.315294445	18.2528803	0.008861909	2.655473647	92720.93016	4.542108035	20.01310728
75 to 79	0.019860019	2.630899108	83390.84847	3.983674388	14.30582301	0.01375642	2.783561293	88724.09121	4.305677751	15.78782198
80 to 84	0.040951827	2.592300395	75554.17868	3.443852901	10.51246885	0.032298329	2.743996449	82860.45513	3.865021908	11.69979367
85 to 89	0.086063229	2.571335496	61698.27069	2.561481746	7.28580127	0.070088532	2.659135411	70570.9888	3.04015572	8.245603595
90 to 94	0.175826514	2.251437103	40163.80139	1.363662319	4.846159304	0.144819863	2.346467692	49752.27741	1.809731215	5.599158133
95 plus	0.280217696	3.579630391	16704.05573	0.606890058	3.579630391	0.244836131	4.107812637	24196.05198	1.012474729	4.107812637

Table 15: Belgium 2017 life table, by age and sex. mx=mortality rate, ax=mean person-years lived in an age interval among those who die in that age interval, lx=number of persons left alive at age x, nLx=person-years lived between age x and x+n, ex=life expectancy at age x.

Age Group	Male					Female				
	mx	ax	lx	nLx	ex	mx	ax	lx	nLx	ex
Early Neonatal	0.087663486	0.009586354	100000	0.01916197	78.92605874	0.063365227	0.009587099	100000	0.019166434	83.95646821
Late Neonatal	0.009149028	0.0287646	99832.02201	0.057422488	79.03965845	0.007566937	0.028765036	99878.55248	0.057451866	84.03936184
Post Neonatal	0.001309695	0.461550797	99779.48783	0.920694957	79.02372293	0.000902009	0.461579758	99835.07988	0.92138127	84.01840906
1 to 4	0.000162256	1.999783659	99658.91032	3.985063131	78.19548878	0.000150266	1.999799645	99751.97257	3.988880021	83.16473613
5 to 9	0.00011095	2.499768854	99594.25327	4.978331713	74.24495671	7.28746E-05	2.499848178	99692.03453	4.983693722	79.21353571
10 to 14	9.08474E-05	2.943912631	99539.02037	4.976021381	69.28476798	5.30101E-05	2.85440797	99655.71656	4.982219187	74.24149304
15 to 19	0.00030468	2.83022321	99493.8153	4.971404281	64.31490812	0.000163182	2.680289771	99629.30594	4.979580407	69.26041512
20 to 24	0.000575169	2.630285423	99342.34781	4.960356464	59.40863516	0.000194479	2.635256053	99548.04829	4.97511446	64.31475251
25 to 29	0.000667072	2.579726016	99057.04513	4.944868819	54.57213225	0.00028999	2.63323597	99451.29328	4.969154194	59.37474723
30 to 34	0.000835322	2.594580944	98727.19018	4.926461052	49.74580559	0.000380828	2.637002516	99307.19383	4.960895566	54.45706234
35 to 39	0.001053815	2.650633089	98315.67797	4.903643921	44.943117	0.000542254	2.686208239	99118.27077	4.949703377	49.55580775
40 to 44	0.001611899	2.663902555	97798.93597	4.871603206	40.16651744	0.000869287	2.743962696	98849.87505	4.932819663	44.68302768
45 to 49	0.002351725	2.69512449	97013.70912	4.824535487	35.46997145	0.001571119	2.713774729	98421.08071	4.903441384	39.86568802
50 to 54	0.003888789	2.716391007	95879.16921	4.751762801	30.85767194	0.002511521	2.680477816	97650.7159	4.854258136	35.15868798
55 to 59	0.006593138	2.704876939	94031.44873	4.631494096	26.4104691	0.003821428	2.69006862	96431.61663	4.779394382	30.56915351
60 to 64	0.010934401	2.664583329	90978.17948	4.435654226	22.20579699	0.006188214	2.652131777	94605.33681	4.66253131	26.10716784
65 to 69	0.016612437	2.645590695	86128.83129	4.144380609	18.30574701	0.008752381	2.663440063	91720.37533	4.494126906	21.84469431
70 to 74	0.025878367	2.631173392	79245.66278	3.733493508	14.66568761	0.014048349	2.678446603	87787.6124	4.250780145	17.70373107
75 to 79	0.040896796	2.621385584	69587.55103	3.171062956	11.33580266	0.023298581	2.710357762	81817.58859	3.883772746	13.79980215
80 to 84	0.071303506	2.578438809	56626.0646	2.414670181	8.330717986	0.045935118	2.680757988	72772.76782	3.288491404	10.17790703
85 to 89	0.128981406	2.437618755	39421.12177	1.48173768	5.842329394	0.092208516	2.568953832	57676.10267	2.356050863	7.140516718
90 to 94	0.224102817	2.196113966	20323.96299	0.624226789	4.043931057	0.174004068	2.31956476	35967.16346	1.226661861	4.901156342
95 plus	0.320515687	3.120124722	6343.470403	0.198007028	3.120124722	0.272811475	3.665939558	14637.80332	0.5368989	3.665939558

Table 15: Belgium 2100 life table, by age and sex. mx=mortality rate, ax=mean person-years lived in an age interval among those who die in that age interval, lx=number of persons left alive at age x, nLx=person-years lived between age x and x+n, ex=life expectancy at age x.

Age Group	Male					Female				
	mx	ax	lx	nLx	ex	mx	ax	lx	nLx	ex
Early Neonatal	0.025838543	0.009588249	100000	0.019173332	84.55539049	0.019882795	0.009588432	100000	0.019174426	87.15985347
Late Neonatal	0.002584619	0.02876641	99950.46256	0.05750147	84.57811365	0.00260267	0.028766405	99961.87778	0.057508008	87.17391488
Post Neonatal	0.000303552	0.461622272	99935.60175	0.922563813	84.53315079	0.000204519	0.461629307	99946.91111	0.922710392	87.1294362
1 to 4	4.6247E-05	1.999938337	99907.59991	3.995934402	83.63342999	4.90257E-05	1.999934632	99928.04112	3.996729763	86.22250722
5 to 9	4.09336E-05	2.499914724	99889.12088	4.993945051	79.64854013	3.37679E-05	2.499929646	99908.44779	4.995000778	82.23901831
10 to 14	4.06945E-05	3.330768884	99868.68336	4.993057601	74.66427048	2.79641E-05	3.16141402	99891.58492	4.994292818	77.2524611
15 to 19	0.000176584	2.92778154	99848.36882	4.990559988	69.67879438	0.000100935	2.669174571	99877.62223	4.992706811	72.26284073
20 to 24	0.000390998	2.674487853	99760.26544	4.983469645	64.73744069	0.000117811	2.679651796	99827.23796	4.989980371	67.29789845
25 to 29	0.000507669	2.600818892	99565.50516	4.972208	59.85816062	0.00019141	2.637681623	99768.47765	4.986148402	62.33590274
30 to 34	0.000638896	2.560740063	99313.30484	4.957923947	55.00278193	0.000233736	2.60323511	99673.08668	4.980845646	57.39291708
35 to 39	0.000687222	2.597660391	98996.9274	4.941670553	50.16963703	0.000296938	2.649167404	99556.73262	4.974336458	52.45673062
40 to 44	0.000955921	2.620365995	98657.82647	4.921688103	45.33250732	0.00043265	2.762616134	99409.10971	4.965622281	47.53038743
45 to 49	0.001254899	2.692712241	98188.06115	4.895251006	40.53617605	0.000858416	2.750843731	99194.38817	4.95013527	42.62685367
50 to 54	0.002209439	2.721485964	97574.87433	4.854262524	35.77308161	0.001523559	2.730558366	98769.69911	4.921407064	37.79733433
55 to 59	0.003679732	2.675794071	96505.32814	4.784301918	31.13756707	0.00262472	2.715737097	98020.72176	4.871708648	33.06354728
60 to 64	0.005437899	2.648480157	94749.9553	4.677788004	26.66242534	0.004362673	2.636563651	96744.78716	4.787843715	28.46069465
65 to 69	0.008046736	2.648634022	92212.80987	4.525323048	22.3204469	0.005699353	2.636442071	94663.21526	4.670305362	24.0250395
70 to 74	0.012265175	2.686267885	88584.83912	4.307546778	18.12215405	0.008544482	2.696629967	92014.13669	4.51213256	19.63779752
75 to 79	0.021507746	2.712630597	83332.66175	3.972409643	14.09087946	0.015137377	2.790418248	88180.01122	4.266694592	15.36997151
80 to 84	0.041980433	2.684309141	74857.08375	3.414280445	10.37719525	0.034949794	2.75508424	81770.68546	3.793709476	11.35188636
85 to 89	0.088180611	2.563913597	60670.9349	2.5027105	7.176289017	0.074663609	2.639438808	68654.8081	2.925003135	7.983242545
90 to 94	0.178722843	2.252770738	38885.75871	1.309142394	4.781222787	0.151193039	2.343787277	47176.20337	1.691786046	5.429179002
95 plus	0.282730161	3.542316812	15759.33219	0.562678325	3.542316812	0.251006905	3.999794823	22054.63514	0.894071446	3.999794823

Table 15: Cyprus 2017 life table, by age and sex. mx=mortality rate, ax=mean person-years lived in an age interval among those who die in that age interval, lx=number of persons left alive at age x, nLx=person-years lived between age x and x+n, ex=life expectancy at age x.

Age Group	Male					Female				
	mx	ax	lx	nLx	ex	mx	ax	lx	nLx	ex
Early Neonatal	0.075447094	0.009586729	100000	0.019164214	78.44086678	0.056985193	0.009587295	100000	0.019167606	85.09515427
Late Neonatal	0.008389998	0.028764809	99855.41247	0.057437195	78.53525111	0.006642486	0.028765291	99890.77349	0.057460423	85.16901364
Post Neonatal	0.000956599	0.46157588	99807.22338	0.921100971	78.51562091	0.000758175	0.461589976	99852.60586	0.921604198	85.14402369
1 to 4	7.85063E-05	1.999895325	99719.1127	3.988138304	77.66130052	9.71917E-05	1.999870411	99782.73311	3.990533594	84.28003583
5 to 9	8.83251E-05	2.499815989	99687.80414	4.983289766	73.68506314	6.12894E-05	2.499872314	99743.94913	4.986433384	80.31203005
10 to 14	0.000102005	3.312704503	99643.78977	4.981332344	68.71650532	5.62744E-05	2.842389882	99713.3878	4.98506409	75.33587728
15 to 19	0.000487157	2.781608998	99592.97826	4.97427336	63.74986619	0.000153845	2.727962297	99685.33482	4.982525321	70.35627592
20 to 24	0.000763236	2.554216874	99350.65488	4.958277089	58.89849738	0.000225023	2.603706728	99608.68156	4.977750003	65.40830119
25 to 29	0.00068974	2.498110901	98972.22832	4.940086669	54.11382044	0.000266359	2.558824266	99496.67119	4.971600872	60.4789787
30 to 34	0.000762425	2.549458757	98631.50074	4.922379185	49.29203699	0.000300895	2.638831046	99364.24933	4.96468538	55.55613793
35 to 39	0.000877279	2.619821656	98256.2219	4.902575272	44.4704636	0.000468216	2.674542658	99214.86673	4.955347758	50.63577864
40 to 44	0.001277284	2.686392282	97826.15704	4.876897437	39.65431707	0.00069548	2.707308588	98982.85511	4.941263859	45.74814415
45 to 49	0.002041374	2.716885017	97203.29513	4.837620238	34.89101881	0.001166776	2.683752853	98639.21094	4.918668322	40.89802123
50 to 54	0.003457995	2.746473223	96215.88636	4.773599608	30.22092533	0.00174027	2.697493438	98065.33947	4.88369976	36.12153544
55 to 59	0.006306676	2.710702116	94565.4882	4.660991751	25.70001586	0.002857386	2.683080956	97215.50546	4.828809395	31.41355348
60 to 64	0.010328182	2.68405765	91626.76219	4.474348162	21.43675305	0.004346029	2.704641761	95835.8885	4.744472103	26.82689455
65 to 69	0.016766016	2.67887287	87007.32836	4.187491819	17.43169667	0.007384513	2.725967028	93774.27254	4.611295102	22.35690831
70 to 74	0.028395776	2.662395737	79990.57093	3.750772057	13.72504747	0.01311874	2.707294618	90369.9786	4.386607531	18.09598329
75 to 79	0.049394988	2.614652509	69349.85528	3.10244785	10.42184497	0.0227046	2.699220223	84617.72718	4.020953526	14.14159817
80 to 84	0.085478859	2.533040953	54047.62528	2.232521865	7.632833151	0.042139166	2.658822426	75494.28783	3.436015291	10.52406992
85 to 89	0.14644816	2.388611003	35001.70837	1.266725263	5.411338577	0.086298006	2.591337408	61028.52398	2.526779926	7.388693292
90 to 94	0.241710507	2.163087228	16489.0498	0.489461421	3.812490277	0.166689114	2.330317055	39247.10099	1.358494398	5.053011519
95 plus	0.334736573	2.987789641	4678.155234	0.139934945	2.987789641	0.265894706	3.761548433	16626.75217	0.62592243	3.761548433

Table 15: Cyprus 2100 life table, by age and sex. mx=mortality rate, ax=mean person-years lived in an age interval among those who die in that age interval, lx=number of persons left alive at age x, nLx=person-years lived between age x and x+n, ex=life expectancy at age x.

Age Group	Male					Female				
	mx	ax	lx	nLx	ex	mx	ax	lx	nLx	ex
Early Neonatal	0.019680875	0.009588438	100000	0.019174464	83.84184858	0.012481273	0.009588659	100000	0.019175787	89.06940045
Late Neonatal	0.00257683	0.028766412	99962.26565	0.057508274	83.85428369	0.00158776	0.028766685	99976.06714	0.05751785	89.07152091
Post Neonatal	0.000210237	0.461628901	99947.44772	0.922712916	83.80917101	0.000116877	0.461635533	99966.93508	0.922932592	89.02211308
1 to 4	1.63904E-05	1.999978147	99928.05136	3.996991037	82.90206031	2.74423E-05	1.999963411	99956.14886	3.998026528	88.10837194
5 to 9	2.97535E-05	2.499938001	99921.50058	4.995703429	78.90736154	2.92476E-05	2.499939067	99945.17779	4.996893519	84.11781499
10 to 14	5.72305E-05	3.457431199	99906.63735	4.994866042	73.91869145	3.85415E-05	3.066155825	99930.56337	4.996127202	79.12974676
15 to 19	0.000353586	2.891626295	99878.05602	4.990018158	68.93882745	0.000106709	2.729616138	99911.30911	4.994352276	74.14442831
20 to 24	0.000681723	2.579558283	99701.7224	4.976871558	64.05536181	0.000153432	2.582058279	99858.01723	4.99105145	69.18254598
25 to 29	0.000640535	2.489485198	99362.86346	4.960172981	59.26461467	0.000169387	2.561882299	99781.44412	4.98700598	64.2336516
30 to 34	0.00063873	2.511104221	99045.95486	4.944419042	54.44619176	0.000197312	2.600012425	99696.98555	4.982485544	59.28587464
35 to 39	0.00063228	2.544663776	98730.99253	4.928866274	49.61204979	0.000260444	2.631277904	99598.68655	4.976868284	54.34177727
40 to 44	0.000730505	2.612528784	98420.22656	4.912413932	44.76092354	0.000366348	2.723475195	99469.08457	4.9692877	49.40899314
45 to 49	0.001040643	2.668163976	98062.0376	4.891228765	39.91478731	0.000633764	2.712449182	99287.0848	4.957156101	44.49419962
50 to 54	0.001702414	2.742372678	97553.73613	4.858909404	35.10788565	0.001006096	2.700179203	98973.00812	4.93721731	39.62620756
55 to 59	0.003255822	2.681880207	96727.74933	4.800180004	30.3814921	0.001601314	2.6765333	98476.52539	4.905569907	34.81134453
60 to 64	0.005129964	2.688843804	95167.43007	4.702479346	25.83108869	0.002378162	2.692168316	97691.63016	4.857922263	30.06807377
65 to 69	0.00865441	2.677671942	92764.54051	4.547133234	21.42310763	0.003840659	2.709787195	96537.97181	4.784817226	25.39297233
70 to 74	0.01478723	2.683520986	88848.6275	4.296218167	17.24121171	0.00636733	2.778528346	94704.2071	4.669154157	20.82915341
75 to 79	0.02696499	2.675230608	82568.42198	3.888824471	13.33724868	0.012819362	2.80770418	91741.14834	4.461780488	16.40796869
80 to 84	0.05139034	2.606907112	72340.37713	3.232674056	9.819313503	0.027732793	2.746223967	86051.86294	4.051764218	12.3003804
85 to 89	0.100428516	2.527579323	56347.21495	2.281459157	6.829244845	0.062302002	2.692624722	74936.60297	3.282686832	8.695980835
90 to 94	0.191646391	2.231198891	34705.91807	1.155235596	4.593799186	0.133946093	2.350456682	54881.74875	2.037559102	5.892024944
95 plus	0.293340976	3.435081829	13706.95159	0.487788274	3.435081829	0.234304743	4.294002647	28211.82998	1.232048137	4.294002647

Table 15: Denmark 2017 life table, by age and sex. mx=mortality rate, ax=mean person-years lived in an age interval among those who die in that age interval, lx=number of persons left alive at age x, nLx=person-years lived between age x and x+n, ex=life expectancy at age x.

Age Group	Male					Female				
	mx	ax	lx	nLx	ex	mx	ax	lx	nLx	ex
Early Neonatal	0.091711283	0.00958623	100000	0.019161227	78.85030578	0.078750354	0.009586627	100000	0.019163607	82.59926695
Late Neonatal	0.009536321	0.028764493	99824.27291	0.057417391	78.96991104	0.008112367	0.028764885	99849.08747	0.057434016	82.70490985
Post Neonatal	0.001248865	0.461555118	99769.51999	0.920628831	78.95569957	0.001039918	0.461569962	99802.496	0.921021927	82.68597119
1 to 4	0.000129612	1.999827184	99654.55204	3.985148998	78.12297121	0.000105363	1.999859516	99706.72028	3.987428518	81.84166626
5 to 9	6.89362E-05	2.499856383	99602.90235	4.979286965	74.16244723	9.51866E-05	2.499801695	99664.70857	4.982049793	77.87532238
10 to 14	6.43642E-05	2.83082459	99568.57823	4.977733795	69.18715228	5.23688E-05	2.791289152	99617.28691	4.980288321	72.91120433
15 to 19	0.000171104	2.962715451	99536.54012	4.975092773	64.20850932	0.000168557	2.737388937	99591.20598	4.977662005	67.92956523
20 to 24	0.000445128	2.663767074	99451.41468	4.967404991	59.26092113	0.000244784	2.568429421	99507.30463	4.972405684	62.98452155
25 to 29	0.000522507	2.563945346	99230.30284	4.955207896	54.38700464	0.000249502	2.55875023	99385.58883	4.966254571	58.05849379
30 to 34	0.00060826	2.62263465	98971.39202	4.941424234	49.52254201	0.000315917	2.651254522	99261.6808	4.959404281	53.12775442
35 to 39	0.000885377	2.677117963	98670.82826	4.92341614	44.66536736	0.000480263	2.69585804	99105.0065	4.949772977	48.20752762
40 to 44	0.001370901	2.718612243	98234.92822	4.896433058	39.8516242	0.00077032	2.752287101	98867.29122	4.93481998	43.3169141
45 to 49	0.002350165	2.719147966	97563.69511	4.852176204	35.10700797	0.001421746	2.770762159	98487.16058	4.9088001	38.47342169
50 to 54	0.003914465	2.745549178	96423.40738	4.778997838	30.48987721	0.002648029	2.726961143	97789.2766	4.860210868	33.72811957
55 to 59	0.007192364	2.694991665	94552.82604	4.650549046	26.03854643	0.00439572	2.707405754	96502.34394	4.77697974	29.14138171
60 to 64	0.011255773	2.645046694	91208.37694	4.442675891	21.89420949	0.007276914	2.657754491	94402.68969	4.641040687	24.72907821
65 to 69	0.016448031	2.648942794	86208.68947	4.149992927	18.01023445	0.010482669	2.670673073	91025.88528	4.442833029	20.54754614
70 to 74	0.026446151	2.640616313	79384.60952	3.736194257	14.33041248	0.017324823	2.685509712	86369.60099	4.152043568	16.51094858
75 to 79	0.042765776	2.629721401	69507.90486	3.155698954	10.99126793	0.029880297	2.679841375	79178.77785	3.702390064	12.76614615
80 to 84	0.077599408	2.564682851	56020.75414	2.356142304	8.004566997	0.055662946	2.617217292	68122.12694	3.007515508	9.403019722
85 to 89	0.136931396	2.414932747	37752.45945	1.394475441	5.638237225	0.106336715	2.518542332	51394.93069	2.033654478	6.612110916
90 to 94	0.232215905	2.181346038	18674.51642	0.564521796	3.934130241	0.190801468	2.290349754	29790.48214	0.982214236	4.583019098
95 plus	0.327099027	3.057346827	5574.813512	0.170524818	3.057346827	0.288520919	3.466386497	11066.70279	0.383878989	3.466386497

Table 15: Denmark 2100 life table, by age and sex. mx=mortality rate, ax=mean person-years lived in an age interval among those who die in that age interval, lx=number of persons left alive at age x, nLx=person-years lived between age x and x+n, ex=life expectancy at age x.

Age Group	Male					Female				
	mx	ax	lx	nLx	ex	mx	ax	lx	nLx	ex
Early Neonatal	0.023741992	0.009588313	100000	0.019173717	83.96116258	0.022994744	0.009588336	100000	0.019173854	85.58902463
Late Neonatal	0.002422386	0.028766455	99954.48334	0.057504052	83.98021184	0.002557216	0.028766418	99955.91534	0.057504653	85.6075962
Post Neonatal	0.000291882	0.4616231	99940.55478	0.922614513	83.93438328	0.00029504	0.461622877	99941.21148	0.922619227	85.56264377
1 to 4	3.10843E-05	1.999958554	99913.62962	3.996296753	83.03360555	2.85971E-05	1.999961872	99913.99395	3.9963312	84.66255466
5 to 9	2.02823E-05	2.499957742	99901.20835	4.994807168	79.04368316	4.93023E-05	2.499897226	99902.56632	4.994513076	80.67200615
10 to 14	2.44058E-05	3.20935863	99891.07889	4.99431006	74.05141692	2.71848E-05	2.963766997	99877.96526	4.993610131	75.69125308
15 to 19	9.4743E-05	3.051903616	99878.89271	4.99299213	69.06005491	0.000118211	2.800723866	99864.39282	4.991865245	70.70114197
20 to 24	0.000305322	2.675024214	99831.5981	4.987992159	64.09118861	0.000179701	2.597873305	99805.41976	4.988078632	65.74126141
25 to 29	0.000351763	2.558620627	99679.42864	4.979663381	59.18405035	0.00016687	2.584899641	99715.87062	4.983742209	60.79790314
30 to 34	0.000380203	2.592843281	99504.49525	4.970638774	54.28289249	0.000196409	2.610849195	99632.81378	4.979269026	55.84624786
35 to 39	0.000506761	2.634574749	99315.82921	4.959827187	49.38055937	0.000238431	2.683435401	99535.1039	4.973959796	50.89825794
40 to 44	0.000749344	2.726296618	99064.91549	4.944729731	44.4983438	0.000383214	2.756025939	99416.59838	4.966544377	45.9553238
45 to 49	0.001378121	2.71137671	98695.23915	4.919233694	39.65390832	0.000768383	2.773887062	99226.38757	4.952758155	41.03755556
50 to 54	0.002444848	2.721959481	98018.72729	4.873698382	34.90731783	0.001451676	2.704517681	98846.24648	4.925840231	36.18291537
55 to 59	0.004413402	2.63766587	96831.25635	4.791678562	30.29841679	0.002355034	2.665529313	98132.05956	4.879793886	31.42383459
60 to 64	0.006020335	2.605787674	94725.13006	4.669135538	25.90810507	0.003699726	2.676232918	96984.80203	4.807849169	26.7600498
65 to 69	0.00827789	2.638138549	91930.7345	4.508727549	21.61029076	0.005953311	2.744119654	95212.2676	4.697083423	22.2026768
70 to 74	0.013434019	2.669262642	88221.12737	4.277963165	17.40326757	0.011380175	2.748153897	92431.85047	4.506316098	17.78099653
75 to 79	0.024063395	2.68945882	82520.0756	3.910641539	13.41562433	0.023003501	2.736905655	87346.76395	4.153511318	13.64363256
80 to 84	0.049726451	2.619832114	73217.39101	3.278977101	9.774263219	0.050450562	2.628819549	77945.58766	3.489396519	9.939297767
85 to 89	0.099266287	2.528707476	57190.21586	2.305803604	6.781480357	0.097996005	2.551842469	60787.86297	2.46759533	6.994230795
90 to 94	0.191483542	2.240163374	34786.55305	1.145798054	4.560416371	0.180144689	2.304189196	37445.3458	1.275550681	4.818746915
95 plus	0.293445703	3.415786049	13261.86158	0.458955766	3.415786049	0.278346127	3.615116202	15260.81147	0.566118396	3.615116202

Table 15: Finland 2017 life table, by age and sex. mx=mortality rate, ax=mean person-years lived in an age interval among those who die in that age interval, lx=number of persons left alive at age x, nLx=person-years lived between age x and x+n, ex=life expectancy at age x.

Age Group	Male					Female				
	mx	ax	lx	nLx	ex	mx	ax	lx	nLx	ex
Early Neonatal	0.05233756	0.009587437	100000	0.019168461	78.57689396	0.049489862	0.009587524	100000	0.019168984	84.45375316
Late Neonatal	0.004727708	0.028765819	99899.67756	0.057468711	78.63661267	0.004427579	0.028765902	99905.13335	0.057472345	84.5147598
Post Neonatal	0.000757514	0.461590023	99872.5084	0.921788182	78.60046228	0.000493126	0.461608805	99879.68737	0.921966946	84.47874848
1 to 4	0.000118497	1.999842004	99802.6851	3.991161477	77.73184302	9.21532E-05	1.999877129	99834.22439	3.992633077	83.59372035
5 to 9	9.077E-05	2.499810896	99755.39256	4.986637978	73.76774737	8.87701E-05	2.499815062	99797.43175	4.988764387	79.62380209
10 to 14	0.000102196	3.038252904	99710.12999	4.984506855	68.80009964	9.47624E-05	2.727449625	99753.147	4.986583499	74.65804103
15 to 19	0.000354882	2.841898647	99659.19111	4.979146204	63.83371024	0.000192413	2.735819133	99705.89323	4.983123838	69.69212748
20 to 24	0.000686512	2.654694368	99482.49125	4.966128654	58.94201797	0.00031313	2.575279839	99610.01185	4.976722099	64.75656431
25 to 29	0.000868896	2.563626573	99141.56293	4.946606469	54.1355306	0.000306357	2.540774329	99454.17674	4.968965301	59.85397336
30 to 34	0.000958877	2.595464015	98711.75947	4.924234786	49.36002559	0.000374252	2.589201742	99301.95073	4.960622017	54.94180881
35 to 39	0.001319842	2.617051548	98239.59457	4.896580102	44.58472824	0.000468212	2.635547756	99116.30074	4.950334783	50.03983951
40 to 44	0.001718858	2.676674789	97593.34221	4.860258785	39.86254693	0.000681538	2.75381182	98884.52442	4.936668722	45.15091179
45 to 49	0.002818496	2.709093083	96757.97004	4.806862723	35.18348623	0.001305846	2.714045797	98548.08033	4.91273913	40.29560479
50 to 54	0.004651571	2.669747754	95403.2479	4.719015384	30.64445461	0.002043013	2.688041208	97906.57437	4.872315808	35.5417604
55 to 59	0.006851025	2.682715857	93208.38414	4.587595601	26.30296756	0.003201298	2.699481126	96911.20404	4.810137297	30.87907779
60 to 64	0.011283664	2.662013881	90065.86215	4.387564937	22.12684435	0.005242139	2.677419109	95371.45129	4.711218153	26.33386277
65 to 69	0.017097283	2.626724058	85116.06516	4.089894732	18.25848564	0.007988415	2.679947114	92902.0423	4.560592764	21.96243559
70 to 74	0.024957031	2.629063704	78125.50126	3.688131655	14.65691283	0.012960021	2.705975271	89259.49898	4.334151474	17.7490278
75 to 79	0.040348133	2.637273973	68925.02984	3.146471348	11.26232149	0.023146601	2.727612154	83644.0587	3.973296225	13.75859057
80 to 84	0.073608652	2.578675898	56237.64464	2.386823318	8.208431642	0.046606474	2.686098183	74451.50008	3.360403122	10.12030281
85 to 89	0.131953187	2.429070756	38683.01987	1.444548246	5.764530941	0.093242745	2.565135699	58800.57736	2.39642406	7.099263128
90 to 94	0.227154597	2.190653535	19638.12291	0.59957755	4.002026331	0.175261051	2.317555458	36474.76695	1.240881462	4.876106424
95 plus	0.322997693	3.096165701	6027.901483	0.186721801	3.096165701	0.273994493	3.650194061	14745.12779	0.53856767	3.650194061

Table 15: Finland 2100 life table, by age and sex. mx=mortality rate, ax=mean person-years lived in an age interval among those who die in that age interval, lx=number of persons left alive at age x, nLx=person-years lived between age x and x+n, ex=life expectancy at age x.

Age Group	Male					Female				
	mx	ax	lx	nLx	ex	mx	ax	lx	nLx	ex
Early Neonatal	0.015358765	0.00958857	100000	0.019175258	83.82454236	0.016022622	0.00958855	100000	0.019175136	87.4054144
Late Neonatal	0.001403299	0.028766736	99970.55159	0.057514982	83.83005336	0.001386329	0.028766741	99969.27917	0.057514278	87.41310679
Post Neonatal	0.000171066	0.461631684	99962.48127	0.922868388	83.77929137	0.000114515	0.461635701	99961.30676	0.922881634	87.36255337
1 to 4	3.3916E-05	1.999954779	99946.69551	3.997596665	82.86915066	2.57934E-05	1.999965608	99950.73894	3.997823328	86.44843881
5 to 9	3.21538E-05	2.499932987	99933.13822	4.996255313	78.88011759	4.30908E-05	2.499910241	99940.42771	4.996483286	82.45714187
10 to 14	4.98157E-05	3.25153332	99917.07559	4.995361114	73.89236216	6.26906E-05	2.981964604	99918.90785	4.995240101	77.47433897
15 to 19	0.000213201	2.842817932	99892.2053	4.992225784	68.90997692	0.000142652	2.735369542	99887.61362	4.992727365	72.49772714
20 to 24	0.00042976	2.649751938	99785.80893	4.984226787	63.98011497	0.000223094	2.5782346	99816.42215	4.988111296	67.54742645
25 to 29	0.000590187	2.548505529	99571.73014	4.971387784	59.11128484	0.000220419	2.585892679	99705.22255	4.982551197	62.61983512
30 to 34	0.000598946	2.536885063	99278.64212	4.956603152	54.27748752	0.000256342	2.541261937	99595.53852	4.976630508	57.68585254
35 to 39	0.000703158	2.564881792	98982.26747	4.940626471	49.43185149	0.000260513	2.582222737	99468.03938	4.970269256	52.75628982
40 to 44	0.000836573	2.659896221	98635.51969	4.922101528	44.59615614	0.000372805	2.790223147	99338.6757	4.9627978	47.8212261
45 to 49	0.001488358	2.685052561	98224.45855	4.894370458	39.77125022	0.000816807	2.741781138	99153.84501	4.948520375	42.90439593
50 to 54	0.002623747	2.649608512	97497.36577	4.844976414	35.04681834	0.001437222	2.682841407	98750.14937	4.921122021	38.06665663
55 to 59	0.003920796	2.641577404	96230.44209	4.767374601	30.47052572	0.002297219	2.671897379	98044.03147	4.8760911	33.31834392
60 to 64	0.005955121	2.62542129	94369.18302	4.652822235	26.01514909	0.003620349	2.643535261	96927.17611	4.805426783	28.66688614
65 to 69	0.008987602	2.615755391	91608.91158	4.484665396	21.71531996	0.005306262	2.648471065	95194.05311	4.701343859	24.13585713
70 to 74	0.013229396	2.640707561	87602.89483	4.248127751	17.58249076	0.008521096	2.683979155	92712.59857	4.546443549	19.70560215
75 to 79	0.023297727	2.665940327	82014.22153	3.890793392	13.59564999	0.014999234	2.759376658	88873.31075	4.30020741	15.43449497
80 to 84	0.04776922	2.6133757	73024.65316	3.282834122	9.934718792	0.034412002	2.717631706	82497.13795	3.827970739	11.40918403
85 to 89	0.096342075	2.538106401	57605.83188	2.337881107	6.890683022	0.073719265	2.643238975	69504.17206	2.968325062	8.029386292
90 to 94	0.188049438	2.24296667	35582.74468	1.180219947	4.621987123	0.149957816	2.344989398	48019.7595	1.726653236	5.458261388
95 plus	0.290549618	3.451083864	13842.18879	0.484533715	3.451083864	0.249825625	4.01819598	22610.45396	0.921105083	4.01819598

Table 15: France 2017 life table, by age and sex. mx=mortality rate, ax=mean person-years lived in an age interval among those who die in that age interval, lx=number of persons left alive at age x, nLx=person-years lived between age x and x+n, ex=life expectancy at age x.

Age Group	Male					Female				
	mx	ax	lx	nLx	ex	mx	ax	lx	nLx	ex
Early Neonatal	0.082773209	0.009586504	100000	0.019162868	79.87818805	0.067263276	0.009586979	100000	0.019165718	85.85517487
Late Neonatal	0.012446132	0.02876369	99841.38458	0.057422427	79.98588926	0.010126848	0.02876433	99871.08596	0.057443341	85.94680493
Post Neonatal	0.001243457	0.461555503	99769.91782	0.920634792	79.98562876	0.001019463	0.461571415	99812.91491	0.921126769	85.93934399
1 to 4	0.000201296	1.999731605	99655.44502	3.984613463	79.15369007	0.000168527	1.999775298	99719.0114	3.987416356	85.09655047
5 to 9	8.69898E-05	2.499818771	99575.23897	4.977679377	75.21583747	6.68417E-05	2.499860747	99651.81396	4.981758187	81.15258548
10 to 14	8.86146E-05	2.955860323	99531.93924	4.975695574	70.2474719	6.71164E-05	2.703290574	99618.51539	4.98015813	76.17887631
15 to 19	0.000281069	2.832594491	99487.84792	4.971363929	65.27729261	0.000132449	2.729881566	99585.09054	4.977757897	71.20353585
20 to 24	0.000538544	2.644478333	99348.11882	4.961112486	60.36510248	0.000213538	2.606276186	99519.16067	4.973415914	66.24889199
25 to 29	0.000657053	2.57666744	99080.94245	4.946171566	55.52071835	0.000241809	2.61054874	99412.95964	4.967777683	61.31686872
30 to 34	0.000784734	2.611002061	98755.95522	4.928558296	50.69491319	0.000342609	2.65883823	99292.83481	4.960662916	56.38787822
35 to 39	0.001082593	2.662259517	98369.1993	4.906044039	45.88392356	0.000504519	2.692167384	99122.87949	4.950380057	51.47998235
40 to 44	0.001642598	2.696587477	97838.08435	4.873465675	41.11849434	0.000811244	2.743627815	98873.12641	4.934623524	46.6031891
45 to 49	0.002667936	2.702411082	97037.59354	4.822320848	36.43536116	0.001462715	2.698343535	98472.81444	4.907120195	41.78144248
50 to 54	0.004325354	2.706566175	95751.08955	4.740531214	31.888464	0.002228333	2.679347516	97755.06018	4.862608213	37.06833443
55 to 59	0.007203791	2.670277052	93700.78875	4.607715291	27.52682354	0.003442201	2.651008284	96671.54991	4.794809911	32.45367698
60 to 64	0.010809612	2.62687169	90381.84161	4.406080011	23.43936049	0.004853438	2.638278237	95021.1744	4.697221267	27.97117763
65 to 69	0.015049901	2.612835213	85619.76532	4.132550673	19.59669484	0.006933549	2.654948458	92741.59881	4.562898732	23.59370784
70 to 74	0.021534776	2.612218413	79401.69622	3.775981788	15.9265341	0.010599786	2.670549145	89578.29383	4.371007048	19.33294829
75 to 79	0.031987131	2.641758331	71272.83506	3.313778965	12.44498189	0.017011251	2.731409612	84946.03865	4.089520171	15.24137951
80 to 84	0.057469531	2.625487364	60677.8831	2.669776353	9.156881661	0.034151515	2.726973639	77991.34032	3.618755715	11.35676557
85 to 89	0.110739278	2.491943491	45343.7365	1.774616524	6.366340225	0.073670413	2.641686768	65637.95075	2.796315905	7.980960345
90 to 94	0.204772441	2.227231144	25703.67508	0.819901124	4.328384108	0.150411377	2.34908242	45048.19088	1.610574276	5.421997064
95 plus	0.304629148	3.282823229	8922.619005	0.293010767	3.282823229	0.250358894	3.994658952	20836.09183	0.832656942	3.994658952

Table 15: France 2100 life table, by age and sex. mx=mortality rate, ax=mean person-years lived in an age interval among those who die in that age interval, lx=number of persons left alive at age x, nLx=person-years lived between age x and x+n, ex=life expectancy at age x.

Age Group	Male					Female				
	mx	ax	lx	nLx	ex	mx	ax	lx	nLx	ex
Early Neonatal	0.024908295	0.009588278	100000	0.019173502	84.81644409	0.022496682	0.009588352	100000	0.019173946	88.59006707
Late Neonatal	0.003448654	0.028766172	99952.24475	0.057501067	84.83778957	0.003499908	0.028766158	99956.86711	0.057503641	88.60910256
Post Neonatal	0.000265877	0.461624948	99932.4159	0.922550444	84.797089	0.000247428	0.461626259	99936.74228	0.922598239	88.5694036
1 to 4	5.63436E-05	1.999924875	99907.88933	3.995865287	83.89451395	5.53407E-05	1.999926213	99913.91587	3.996114338	87.66624384
5 to 9	3.11381E-05	2.499935128	99885.37601	4.993880042	79.91297367	3.22508E-05	2.499932811	99891.80199	4.994187428	83.68520312
10 to 14	3.80064E-05	3.206522495	99869.82619	4.993140933	74.92501069	3.77541E-05	2.76469509	99875.69566	4.993359832	78.69829424
15 to 19	0.000150724	2.895434444	99850.84935	4.990942452	69.9386356	7.68234E-05	2.742794274	99856.84406	4.991978245	73.71263234
20 to 24	0.00030853	2.680165516	99775.62892	4.985209255	64.98900677	0.000128963	2.622834426	99818.49514	4.989394246	68.73987842
25 to 29	0.000414521	2.586424143	99621.83958	4.976120788	60.08486745	0.000151994	2.61965666	99754.15641	4.985899956	63.78250319
30 to 34	0.000488503	2.581046177	99415.62432	4.964907543	55.20367679	0.000212421	2.635236012	99678.38782	4.981396793	58.82895045
35 to 39	0.000598368	2.612704598	99173.20806	4.951578905	50.33182195	0.000270101	2.65714597	99572.60117	4.975474711	53.88857738
40 to 44	0.000804585	2.678560478	98877.11132	4.934625132	45.47418357	0.000409904	2.793138075	99438.2396	4.967394182	48.95768913
45 to 49	0.001277565	2.738439217	98480.38172	4.909843955	40.64596962	0.000825124	2.73464151	99234.68091	4.952461078	44.05221173
50 to 54	0.002291034	2.753576798	97853.73492	4.867606093	35.88757578	0.001329766	2.718021989	98826.15806	4.926348127	39.22263673
55 to 59	0.004082309	2.689981998	96740.20165	4.791826727	31.26668987	0.002224348	2.681431104	98171.34935	4.883372344	34.46527252
60 to 64	0.006128941	2.641663995	94787.79659	4.672000869	26.8515086	0.003298837	2.647956317	97085.81435	4.816952591	29.81915612
65 to 69	0.008673128	2.617585106	91930.97161	4.503891951	22.59925479	0.004695268	2.666664437	95498.24476	4.723232504	25.26833363
70 to 74	0.01192627	2.658691017	88040.3273	4.283196921	18.4753946	0.007324475	2.690594166	93284.17528	4.586720309	20.80127451
75 to 79	0.019704405	2.730628965	82967.50744	3.972283345	14.43453321	0.011962713	2.803716612	89934.04438	4.381944338	16.47091686
80 to 84	0.039501439	2.695731904	75229.87214	3.451923408	10.63190928	0.027646808	2.784117457	84712.86225	3.992807544	12.30599992
85 to 89	0.084182553	2.577344977	61816.4182	2.575917076	7.353321633	0.062222575	2.692827538	73792.32886	3.232858883	8.696359819
90 to 94	0.173654619	2.253608561	40574.59557	1.381991875	4.883839309	0.133877449	2.350840906	54051.19587	2.006569962	5.891850743
95 plus	0.27839135	3.601223759	17017.51388	0.620466524	3.601223759	0.234245921	4.293850805	27775.78512	1.212323837	4.293850805

Table 15: Germany 2017 life table, by age and sex. mx=mortality rate, ax=mean person-years lived in an age interval among those who die in that age interval, lx=number of persons left alive at age x, nLx=person-years lived between age x and x+n, ex=life expectancy at age x.

Age Group	Male					Female				
	mx	ax	lx	nLx	ex	mx	ax	lx	nLx	ex
Early Neonatal	0.093521796	0.009586175	100000	0.019160894	78.21581906	0.082929477	0.009586499	100000	0.01916284	83.20894895
Late Neonatal	0.009236469	0.028764575	99820.80728	0.057415892	78.33703268	0.007137026	0.028765155	99841.08565	0.057431025	83.32219523
Post Neonatal	0.001239124	0.46155581	99767.77616	0.920616859	78.32112009	0.001034832	0.461570323	99800.09819	0.921001952	83.29886989
1 to 4	0.000157787	1.999789617	99653.70241	3.984890471	77.48695632	0.000136688	1.999817749	99704.79132	3.987101599	82.45476268
5 to 9	7.97813E-05	2.499833789	99590.8278	4.978548357	73.53461329	6.06401E-05	2.499873667	99650.29362	4.98175942	78.49876281
10 to 14	8.75952E-05	3.076354458	99551.10913	4.976717006	68.5629543	7.71003E-05	2.787597572	99620.0847	4.980154929	73.52180893
15 to 19	0.000322582	2.758764857	99507.51608	4.971781521	63.59163457	0.000167374	2.644173995	99581.68788	4.977122131	68.54907275
20 to 24	0.00048955	2.593739165	99347.13694	4.961512194	58.68976689	0.000193373	2.563553522	99498.38414	4.972576606	63.6042149
25 to 29	0.000544779	2.584231771	99104.25017	4.948699808	53.82714144	0.000226916	2.632647709	99402.22897	4.967443147	58.6632172
30 to 34	0.000713207	2.624946108	98834.66295	4.933377285	48.96680467	0.000338634	2.675909789	99289.51174	4.960572093	53.72677038
35 to 39	0.00097874	2.672615837	98482.82595	4.912951419	44.13222178	0.000514675	2.707357942	99121.53471	4.950235732	48.81320196
40 to 44	0.001537138	2.720417238	98002.00867	4.882992288	39.33542352	0.000854306	2.733532873	98866.77043	4.933785017	43.93187338
45 to 49	0.002638045	2.737180745	97251.50594	4.833724501	34.61766684	0.00148143	2.733150945	98445.30377	4.905791056	39.10804968
50 to 54	0.004637206	2.716469376	95976.56577	4.74855435	30.04065104	0.002540927	2.70057504	97718.62166	4.857553252	34.37823856
55 to 59	0.007732302	2.68593416	93775.16308	4.606362211	25.68132506	0.004005602	2.683697123	96484.55322	4.779888002	29.78293272
60 to 64	0.012266972	2.645047104	90214.88093	4.384162354	21.58783462	0.006269643	2.668090087	94570.40868	4.660409	25.33078154
65 to 69	0.017949855	2.635970005	84840.1451	4.069470958	17.78680889	0.009530493	2.670472034	91649.64182	4.483012162	21.05217029
70 to 74	0.027900653	2.626174632	77542.24546	3.63657225	14.21177893	0.015209892	2.69243743	87379.78327	4.220978731	16.9493642
75 to 79	0.044100513	2.614487474	67410.13669	3.050238735	10.95270575	0.026743713	2.712866688	80966.2795	3.815271553	13.07754055
80 to 84	0.077213538	2.553537433	53986.1429	2.271380453	8.027143206	0.053521287	2.658200615	70779.38323	3.145572749	9.568522275
85 to 89	0.136328864	2.416715377	36494.79657	1.35052206	5.655036108	0.103377746	2.528952361	53983.48671	2.15124054	6.719791856
90 to 94	0.231584371	2.182419811	18133.98405	0.549218112	3.943202288	0.187315256	2.29661952	31808.2769	1.056836479	4.647602659
95 plus	0.32658148	3.062533076	5443.13637	0.166949079	3.062533076	0.285269123	3.506858572	12066.62576	0.424048935	3.506858572

Table 15: Germany 2100 life table, by age and sex. mx=mortality rate, ax=mean person-years lived in an age interval among those who die in that age interval, lx=number of persons left alive at age x, nLx=person-years lived between age x and x+n, ex=life expectancy at age x.

Age Group	Male					Female				
	mx	ax	lx	nLx	ex	mx	ax	lx	nLx	ex
Early Neonatal	0.02747419	0.009588199	100000	0.019173031	83.65667439	0.026464001	0.00958823	100000	0.019173217	86.32069383
Late Neonatal	0.002298052	0.028766489	99947.32674	0.05750014	83.6816088	0.002031011	0.028766563	99949.26312	0.057501696	86.34532623
Post Neonatal	0.000267105	0.461624861	99934.11365	0.922565592	83.63515708	0.000237253	0.461626981	99937.585	0.922610352	86.29787582
1 to 4	4.04613E-05	1.999946051	99909.4728	3.99605554	82.73237994	3.81686E-05	1.999949108	99915.69696	3.996322809	85.3933952
5 to 9	2.73166E-05	2.499943091	99893.30471	4.994324159	78.74544656	2.68686E-05	2.499944023	99900.44392	4.994686694	81.40612915
10 to 14	4.07755E-05	3.406486049	99879.662	4.993640859	73.7558441	4.67901E-05	2.924502555	99887.0242	4.993853967	76.41672537
15 to 19	0.000192826	2.792416916	99859.30063	4.990835528	68.77018586	0.000110448	2.656049199	99863.65843	4.991889673	71.43392015
20 to 24	0.000314262	2.633723885	99763.0714	4.984435729	63.83352645	0.000128688	2.571714259	99808.52601	4.988867102	66.47182573
25 to 29	0.000386339	2.59964634	99606.46825	4.975699457	58.92912589	0.000154605	2.629901645	99744.33039	4.985385459	61.51284332
30 to 34	0.000491906	2.569895455	99414.31081	4.964777262	54.03739582	0.000221015	2.624065676	99667.26587	4.980740408	56.55817013
35 to 39	0.000551044	2.607513826	99170.22326	4.951988782	49.16338864	0.000279099	2.663091421	99557.20507	4.974613794	51.61740645
40 to 44	0.00078876	2.699725222	98897.5491	4.935900928	44.29100527	0.000438073	2.768670217	99418.39601	4.96605711	46.68525848
45 to 49	0.001299714	2.765049652	98508.65115	4.911146472	39.45405276	0.000837385	2.784368059	99200.92685	4.950860578	41.78064121
50 to 54	0.002462692	2.746689301	97871.13869	4.866555377	34.69125466	0.001587363	2.756390185	98786.57914	4.921797754	36.94254451
55 to 59	0.004321524	2.673104555	96674.58113	4.785677406	30.08307073	0.002808771	2.695505207	98006.1044	4.868856997	32.21162167
60 to 64	0.006293686	2.653255326	94611.57258	4.661903443	25.67416924	0.004334956	2.665814301	96641.03736	4.783802358	27.62306675
65 to 69	0.00944698	2.669607947	91688.86939	4.4862494	21.39921165	0.006476118	2.681093408	94574.90512	4.659095267	23.15922037
70 to 74	0.015374128	2.686106912	87475.51183	4.224875606	17.28977852	0.010437373	2.720792497	91576.01613	4.473063946	18.81875554
75 to 79	0.026366752	2.685954297	81054.71458	3.822998799	13.43034981	0.018729637	2.782765566	86955.21062	4.176274399	14.6594857
80 to 84	0.048989196	2.630072547	71150.76514	3.197751106	9.912900244	0.041611539	2.724713563	79260.3486	3.628772527	10.79375117
85 to 89	0.097719183	2.534758818	55981.77308	2.273871438	6.880213083	0.084603903	2.603296941	64614.54231	2.705561948	7.594899593
90 to 94	0.18912262	2.237884754	34688.49619	1.154794329	4.619294899	0.16319314	2.324397574	42761.99685	1.510954999	5.19352325
95 plus	0.291349329	3.449629397	13670.72162	0.483954008	3.449629397	0.262266614	3.851736869	19280.882	0.769950773	3.851736869

Table 15: Greece 2017 life table, by age and sex. mx=mortality rate, ax=mean person-years lived in an age interval among those who die in that age interval, lx=number of persons left alive at age x, nLx=person-years lived between age x and x+n, ex=life expectancy at age x.

Age Group	Male					Female				
	mx	ax	lx	nLx	ex	mx	ax	lx	nLx	ex
Early Neonatal	0.093885924	0.009586164	100000	0.019160827	78.47004522	0.070058381	0.009586894	100000	0.019165204	83.6915246
Late Neonatal	0.016742686	0.028762505	99820.10992	0.057403097	78.59225956	0.011865788	0.02876385	99865.73438	0.057437391	83.78485277
Post Neonatal	0.001315867	0.461550359	99724.0059	0.920180394	78.61043853	0.001178043	0.461560149	99797.5838	0.920917878	83.78451757
1 to 4	0.000185547	1.999752604	99602.92938	3.982639125	77.78215325	0.000164311	1.999780919	99689.09845	3.98625387	82.95190335
5 to 9	0.000109626	2.499771612	99529.03607	4.97508822	73.83841906	8.57136E-05	2.49982143	99623.60228	4.980112903	79.00512626
10 to 14	9.90768E-05	3.019929364	99474.49773	4.972749204	68.87753293	7.97885E-05	2.68409916	99580.91691	4.978125985	74.03792119
15 to 19	0.000357176	2.804941371	99425.23019	4.967367053	63.91016528	0.000156341	2.710176073	99541.19764	4.975278853	69.06639056
20 to 24	0.000623583	2.608255621	99247.80894	4.955000296	59.01937986	0.00023787	2.573374444	99463.41413	4.970301816	64.11827379
25 to 29	0.000684298	2.559457747	98938.82546	4.938693402	54.1955141	0.000240611	2.623052583	99345.18641	4.964420122	59.19150093
30 to 34	0.000823969	2.599712642	98600.87481	4.920312787	49.37245828	0.000380885	2.636794203	99225.73777	4.956825372	54.25958182
35 to 39	0.001086461	2.639991499	98195.46146	4.897216751	44.56552275	0.000492244	2.715637685	99036.94163	4.946285202	49.35796515
40 to 44	0.001568184	2.715470941	97663.40851	4.865739214	39.79386517	0.000894182	2.713727089	98793.46725	4.929595464	44.47287654
45 to 49	0.002744077	2.736698227	96900.39431	4.815115727	35.08574268	0.001418982	2.706894586	98352.68097	4.901684695	39.65996702
50 to 54	0.004789981	2.700233955	95579.15255	4.726889836	30.53278073	0.002329564	2.6766092	97657.16271	4.856572896	34.92306274
55 to 59	0.007628435	2.670861838	93315.15162	4.584312168	26.20784209	0.003455754	2.667489033	96525.84695	4.787702916	30.30087796
60 to 64	0.0117427	2.643925315	89818.43855	4.370035111	22.12390287	0.005252151	2.676187595	94871.45476	4.686381133	25.78259417
65 to 69	0.01731289	2.628324482	84687.67896	4.067411522	18.30381797	0.008240934	2.675676619	92410.36006	4.533691156	21.3977619
70 to 74	0.025891316	2.622582033	77647.52264	3.657327795	14.7248465	0.013033274	2.727528841	88674.80181	4.306229797	17.18618607
75 to 79	0.040575089	2.618472865	68181.6516	3.108824514	11.40493315	0.025096169	2.748984574	83063.89457	3.931193592	13.16254053
80 to 84	0.0700548	2.576191087	55574.2446	2.375607689	8.398459042	0.053483889	2.660397221	73202.39013	3.253277131	9.565125051
85 to 89	0.127347235	2.442354397	38943.36115	1.469046097	5.885920487	0.103370257	2.528788035	55813.97496	2.223245035	6.716482483
90 to 94	0.222414366	2.19908149	20248.68443	0.623968489	4.067440367	0.187344568	2.296790073	32851.21088	1.090682992	4.645359207
95 plus	0.319139495	3.133566624	6378.631967	0.199955904	3.133566624	0.285307656	3.505408433	12434.31526	0.4361432	3.505408433

Table 15: Greece 2100 life table, by age and sex. mx=mortality rate, ax=mean person-years lived in an age interval among those who die in that age interval, lx=number of persons left alive at age x, nLx=person-years lived between age x and x+n, ex=life expectancy at age x.

Age Group	Male					Female				
	mx	ax	lx	nLx	ex	mx	ax	lx	nLx	ex
Early Neonatal	0.017073266	0.009588518	100000	0.019174943	84.5483096	0.013269623	0.009588634	100000	0.019175642	86.19062021
Late Neonatal	0.003583352	0.028766135	99967.26745	0.057509486	84.55678131	0.002476996	0.02876644	99974.55742	0.05751551	86.19334894
Post Neonatal	0.000299533	0.461622557	99946.66291	0.922667638	84.51667782	0.00026183	0.461625236	99960.3124	0.922809704	86.14809437
1 to 4	6.39289E-05	1.999914762	99919.02925	3.996250227	83.61664698	6.20656E-05	1.999917246	99936.15325	3.99695001	85.24551611
5 to 9	5.17418E-05	2.499892205	99893.48364	4.994028227	79.63750016	4.78317E-05	2.499900352	99911.34858	4.994970177	81.26617937
10 to 14	5.50834E-05	3.366896503	99867.64813	4.992895149	74.65741336	5.69233E-05	2.93643974	99887.46042	4.993744782	76.28501137
15 to 19	0.000283265	2.84661355	99840.15209	4.988884067	69.67705076	0.000129876	2.803378824	99859.04099	4.991494648	71.30591853
20 to 24	0.00049841	2.624089864	99698.90366	4.979048263	64.77154781	0.00021792	2.565418929	99794.22978	4.987055442	66.35039249
25 to 29	0.000596991	2.576747151	99450.98002	4.965300007	59.9262466	0.000188374	2.590046093	99685.58527	4.982003618	61.41986736
30 to 34	0.000665278	2.557500169	99155.21492	4.949669097	55.09718512	0.000286799	2.608514236	99591.77875	4.976158829	56.47522767
35 to 39	0.000740858	2.565966172	98826.60717	4.932421644	50.27211184	0.000323197	2.678853826	99449.12073	4.968720184	51.55224038
40 to 44	0.000894343	2.667369197	98461.95364	4.912799633	45.44894297	0.000561567	2.757227181	99288.58393	4.958167745	46.63094457
45 to 49	0.00146758	2.714763064	98023.52171	4.884741056	40.64025602	0.001015407	2.741508733	99010.25064	4.93919148	41.75350771
50 to 54	0.002518318	2.696452067	97307.8569	4.837267115	35.91799448	0.00177555	2.708818984	98508.96952	4.905482094	36.95062382
55 to 59	0.004060894	2.648300411	96091.57591	4.759118084	31.3362387	0.002834492	2.668452544	97638.91738	4.849938075	32.2526699
60 to 64	0.005791395	2.629394875	94162.39164	4.644567186	26.91962367	0.004198388	2.650607343	96266.60272	4.766464855	27.67016868
65 to 69	0.008563253	2.638627403	91479.04446	4.483560006	22.62435082	0.006140491	2.65738219	94272.32331	4.646951099	23.19278138
70 to 74	0.01276866	2.653569918	87663.72935	4.256372007	18.48610766	0.009191846	2.748979305	91434.96834	4.479392127	18.82279411
75 to 79	0.020608559	2.673742649	82281.78732	3.927176992	14.51425313	0.018349048	2.824593962	87351.197	4.200328042	14.56584089
80 to 84	0.0387077	2.636910186	74252.93753	3.407619052	10.78713935	0.042999302	2.70932894	79750.15367	3.636274645	10.67508299
85 to 89	0.082304762	2.584438422	61368.08088	2.573279684	7.475837699	0.086598342	2.596068225	64507.84453	2.688245524	7.513051568
90 to 94	0.170747672	2.249526611	40909.81536	1.406460062	4.958612988	0.165635811	2.321109442	42238.88586	1.485244453	5.142707733
95 plus	0.275820221	3.644223971	17662.5095	0.657499977	3.644223971	0.264559937	3.819712635	18802.14734	0.744341527	3.819712635

Table 15: Iceland 2017 life table, by age and sex. mx=mortality rate, ax=mean person-years lived in an age interval among those who die in that age interval, lx=number of persons left alive at age x, nLx=person-years lived between age x and x+n, ex=life expectancy at age x.

Age Group	Male					Female				
	mx	ax	lx	nLx	ex	mx	ax	lx	nLx	ex
Early Neonatal	0.031444567	0.009588077	100000	0.019172301	79.85383959	0.031898468	0.009588063	100000	0.019172217	85.758871
Late Neonatal	0.007000073	0.028765192	99939.71391	0.057487984	79.88282441	0.005106192	0.028765715	99938.84405	0.057490616	85.79216507
Post Neonatal	0.000665984	0.461596525	99899.47262	0.922076003	79.85745676	0.000497518	0.461608493	99909.48832	0.92224016	85.75983015
1 to 4	0.000217505	1.999709993	99838.06559	3.991785968	78.98300379	0.000144693	1.999807076	99863.60625	3.993388531	84.87573311
5 to 9	9.96063E-05	2.499792487	99751.24559	4.986320537	75.05001023	6.68344E-05	2.499860762	99805.82594	4.989457592	80.92371342
10 to 14	0.000118016	3.040536844	99701.58001	4.983925947	70.08615179	9.37643E-05	2.612054637	99772.47962	4.987507203	75.94992481
15 to 19	0.000405841	2.74884579	99642.76249	4.977590533	65.1257278	0.000117293	2.630798655	99725.71493	4.984900521	70.98431417
20 to 24	0.000604435	2.602031714	99440.75266	4.964841454	60.25243413	0.0001676	2.607733436	99667.2457	4.981365055	66.02440925
25 to 29	0.000705131	2.590838608	99140.66121	4.948626494	55.42692245	0.000204239	2.611628527	99583.75858	4.97676029	61.07757
30 to 34	0.000917618	2.528090742	98791.71977	4.928407209	50.61353002	0.00027753	2.610177013	99482.11412	4.97080888	56.13730003
35 to 39	0.000835457	2.602785753	98339.48308	4.907146445	45.83464138	0.000351826	2.691847174	99344.16007	4.963177557	51.21162077
40 to 44	0.001340961	2.669662179	97929.51577	4.881222738	41.01560489	0.000603292	2.767568251	99169.54372	4.951808003	46.29703997
45 to 49	0.001947678	2.686253472	97274.97159	4.84192909	36.27359582	0.001132166	2.717281783	98870.80768	4.930797351	41.42854243
50 to 54	0.003131865	2.683865244	96331.93849	4.781910528	31.60235269	0.00179966	2.694610434	98312.56657	4.895318471	36.64831747
55 to 59	0.004829368	2.690459501	94834.35229	4.689415135	27.05896114	0.002854241	2.659203572	97431.59284	4.839248634	31.95526788
60 to 64	0.007845949	2.723402855	92569.75503	4.547267923	22.65502683	0.004070301	2.68423264	96050.39869	4.757676748	27.37645847
65 to 69	0.014128735	2.709347756	89002.192	4.310611501	18.45387005	0.006675348	2.70133013	94113.97888	4.634589009	22.8844016
70 to 74	0.024717697	2.664524285	82912.35591	3.91938907	14.61016439	0.0110976	2.727456535	91020.47643	4.439083394	18.57021676
75 to 79	0.041181586	2.637481203	73225.84716	3.336720097	11.19024909	0.020602588	2.725894999	86094.796	4.112111004	14.47643798
80 to 84	0.074413826	2.578758825	59487.69426	2.520407085	8.165463422	0.039713363	2.676098068	77624.53346	3.553381601	10.75845861
85 to 89	0.132994519	2.426070245	40738.03623	1.517584595	5.737165298	0.082550971	2.605850383	63517.5248	2.65196039	7.553522482
90 to 94	0.228225256	2.188743116	20561.58408	0.62633996	3.98728449	0.161972738	2.336658609	41634.63768	1.454538635	5.154596934
95 plus	0.323868865	3.087737168	6270.777776	0.193660992	3.087737168	0.261416832	3.825600258	18085.14794	0.692089137	3.825600258

Table 15: Iceland 2100 life table, by age and sex. mx=mortality rate, ax=mean person-years lived in an age interval among those who die in that age interval, lx=number of persons left alive at age x, nLx=person-years lived between age x and x+n, ex=life expectancy at age x.										
**Age Group**	**Male**					**Female**				
	**mx**	**ax**	**lx**	**nLx**	**ex**	**mx**	**ax**	**lx**	**nLx**	**ex**
Early Neonatal	0.00587744	0.009588861	100000	0.019177001	83.56555187	0.006678619	0.009588836	100000	0.019176854	88.8372732
Late Neonatal	0.002013118	0.028766568	99988.72894	0.05752443	83.55579236	0.001369129	0.028766746	99987.19268	0.057524612	88.82947039
Post Neonatal	0.000168805	0.461631844	99977.14874	0.923004759	83.50793696	0.000103921	0.461636453	99979.31689	0.923052424	88.77893004
1 to 4	7.95083E-05	1.999893989	99961.56813	3.997826994	82.59759192	5.37787E-05	1.999928295	99969.72459	3.998358935	87.86411703
5 to 9	4.40333E-05	2.499908269	99929.78367	4.995939256	78.62321845	3.07921E-05	2.499935851	99948.22329	4.997026518	83.88259077
10 to 14	6.90072E-05	3.260636073	99907.78858	4.994719768	73.63991807	6.62276E-05	2.74949957	99932.83814	4.995878162	78.89511866
15 to 19	0.000275516	2.765755289	99873.33632	4.990583096	68.66418792	9.18864E-05	2.75337818	99899.75967	4.993943322	73.92034271
20 to 24	0.000485429	2.596305051	99735.90353	4.980963087	63.75402357	0.000164889	2.644347399	99853.8812	4.990725619	68.95298187
25 to 29	0.000509493	2.721763781	99494.51451	4.968811559	58.90053234	0.000176984	2.571090385	99771.61267	4.986441741	64.00759148
30 to 34	0.000958509	2.533500235	99241.97143	4.950378785	54.04214847	0.000230044	2.521290036	99683.37569	4.981337875	59.06189567
35 to 39	0.000654625	2.525056965	98768.53812	4.930438332	49.28748625	0.00020811	2.648411736	99568.86405	4.976009908	54.12660088
40 to 44	0.001037335	2.636494318	98446.71391	4.910272334	44.43942575	0.000379648	2.810593481	99465.36675	4.969017757	49.17992916
45 to 49	0.001406335	2.638932952	97938.85287	4.880786305	39.65484422	0.000655541	2.722806686	99276.92314	4.956462437	44.26739001
50 to 54	0.002210278	2.631518417	97254.47041	4.837464332	34.91347088	0.001098159	2.734329195	98952.21317	4.935194058	39.40309828
55 to 59	0.003047402	2.647075677	96190.83576	4.775468982	30.26580571	0.001752876	2.648409483	98411.13177	4.900361038	34.60330475
60 to 64	0.004824762	2.715862087	94744.06762	4.685659903	25.68129905	0.002367819	2.689929854	97552.9123	4.851141115	29.88354584
65 to 69	0.008857253	2.700523152	92503.57167	4.53319144	21.2276339	0.003976315	2.707112127	96406.41179	4.776699315	25.20520274
70 to 74	0.015075116	2.663220069	88532.73555	4.277520644	17.04762011	0.0063447	2.781097034	94511.37697	4.659906252	20.65447909
75 to 79	0.026180869	2.68798644	82157.24141	3.877408306	13.15454509	0.013034803	2.806954158	91561.10333	4.45109114	16.22884319
80 to 84	0.05263387	2.629127108	72246.73258	3.220628094	9.586195493	0.028519922	2.749760931	85777.39989	4.031357645	12.13016801
85 to 89	0.103278296	2.516517218	55743.26562	2.232469564	6.657382227	0.063894217	2.684584553	74357.2978	3.242786369	8.560609497
90 to 94	0.195853102	2.234068854	33399.80205	1.093995104	4.492280093	0.136588466	2.353555408	53870.79713	1.985224534	5.798704254
95 plus	0.297062993	3.376782667	12544.39297	0.431319794	3.376782667	0.236935815	4.234193943	27103.72876	1.158555572	4.234193943

Table 15: Ireland 2017 life table, by age and sex. mx=mortality rate, ax=mean person-years lived in an age interval among those who die in that age interval, lx=number of persons left alive at age x, nLx=person-years lived between age x and x+n, ex=life expectancy at age x.

Age Group	Male					Female				
	mx	ax	lx	nLx	ex	mx	ax	lx	nLx	ex
Early Neonatal	0.097825501	0.009586043	100000	0.019160104	79.9976664	0.077920336	0.009586653	100000	0.01916376	83.73610291
Late Neonatal	0.008858673	0.02876468	99812.56723	0.057411777	80.12869241	0.00733208	0.028765101	99850.67625	0.057436219	83.84213427
Post Neonatal	0.001145212	0.461562482	99761.70919	0.920600786	80.11199342	0.000912808	0.461578991	99808.56414	0.921131961	83.81996355
1 to 4	0.000100382	1.999866157	99656.28399	3.985451191	79.27296955	9.49939E-05	1.999873342	99724.48386	3.988221603	82.96695859
5 to 9	7.59563E-05	2.499841758	99616.27827	4.97986824	75.30400333	5.35597E-05	2.499888417	99686.5987	4.983662602	78.99773017
10 to 14	9.01071E-05	3.061527271	99578.45375	4.97805295	70.33165842	8.17059E-05	2.698946889	99659.90658	4.982058686	74.01821907
15 to 19	0.000318933	2.860076894	99533.59849	4.973285752	65.3619721	0.000131698	2.611066325	99619.20039	4.979393494	69.04735802
20 to 24	0.000643029	2.620452954	99374.98495	4.961158066	60.46171028	0.000152147	2.636603372	99553.62271	4.97589195	64.09111096
25 to 29	0.000693789	2.517821936	99055.97003	4.944283942	55.64794728	0.000231825	2.699439287	99477.91633	4.971244557	59.13786779
30 to 34	0.000706983	2.542232368	98712.94464	4.9270862	50.83253042	0.000374813	2.613234498	99362.67116	4.963693261	54.2033079
35 to 39	0.000842858	2.628949585	98364.61379	4.908421837	46.00349425	0.000436904	2.671320489	99176.62724	4.953791385	49.30005335
40 to 44	0.001237854	2.675243987	97950.91364	4.883492994	41.18663458	0.000736923	2.730640192	98960.1974	4.939748768	44.40199162
45 to 49	0.001903064	2.708362333	97346.43009	4.84618752	36.42569729	0.001259761	2.744105485	98596.18437	4.915839007	39.5557789
50 to 54	0.003190186	2.696671118	96424.21757	4.786044651	31.74806029	0.002235233	2.720947649	97976.92693	4.874017895	34.78835099
55 to 59	0.00503869	2.693797541	94897.49655	4.690375768	27.21527469	0.003694876	2.653841472	96887.52614	4.80274519	30.14876742
60 to 64	0.008202605	2.693384385	92534.42087	4.540818787	22.84127015	0.005105141	2.702315857	95113.11583	4.700524855	25.66149116
65 to 69	0.013515819	2.693332405	88810.33996	4.306289851	18.6858636	0.009039097	2.703157593	92713.72729	4.541418009	21.25541098
70 to 74	0.023175861	2.677398156	82991.37658	3.937679746	14.80686889	0.014948036	2.708181323	88609.50421	4.283764497	17.11434208
75 to 79	0.040286539	2.645839157	73868.76561	3.373644765	11.30454232	0.027152662	2.695435795	82208.24936	3.868457608	13.23565882
80 to 84	0.072715023	2.578532432	60285.23018	2.563274799	8.255591673	0.050733081	2.649748789	71710.11057	3.203786377	9.778400274
85 to 89	0.130802251	2.432375612	41661.0817	1.559701698	5.794534149	0.099287842	2.54319668	55469.52296	2.230044209	6.86593837
90 to 94	0.225974286	2.192773438	21277.29796	0.651134353	4.018182822	0.182525224	2.305399274	33349.81502	1.118122112	4.735066662
95 plus	0.322038222	3.105403141	6573.747906	0.204240624	3.105403141	0.280809663	3.561636059	12960.67644	0.461944901	3.561636059

Table 15: Ireland 2100 life table, by age and sex. mx=mortality rate, ax=mean person-years lived in an age interval among those who die in that age interval, lx=number of persons left alive at age x, nLx=person-years lived between age x and x+n, ex=life expectancy at age x.

Age Group	Male					Female				
	mx	ax	lx	nLx	ex	mx	ax	lx	nLx	ex
Early Neonatal	0.028608141	0.009588164	100000	0.019172822	85.61461958	0.023462485	0.009588322	100000	0.019173768	87.77345742
Late Neonatal	0.002514196	0.02876643	99945.15484	0.057498533	85.64242242	0.002385804	0.028766465	99955.01655	0.057504419	87.79377679
Post Neonatal	0.000257477	0.461625545	99930.69976	0.922538176	85.59727434	0.000223778	0.461627939	99941.29803	0.922650369	87.74829388
1 to 4	2.50755E-05	1.999966566	99906.94794	3.996077517	84.69421697	2.66437E-05	1.999964476	99920.65241	3.99661313	86.84304061
5 to 9	2.45516E-05	2.499948831	99896.92812	4.994539859	80.70251233	2.76028E-05	2.4999425	99910.00434	4.995155609	82.85208587
10 to 14	3.53675E-05	3.057025751	99884.66688	4.993867671	75.71208545	4.55242E-05	2.815079761	99896.22246	4.994287977	77.86314565
15 to 19	0.000182287	2.883192084	99867.01125	4.99128244	70.72491637	7.88565E-05	2.669725777	99873.49195	4.992739722	72.88023881
20 to 24	0.000419951	2.639561331	99776.07987	4.983778364	65.78637054	0.000102674	2.816048446	99834.13825	4.990521661	67.90792586
25 to 29	0.000492823	2.545900035	99566.99655	4.972318348	60.91820163	0.000173315	2.769388281	99782.92596	4.987153819	62.94140262
30 to 34	0.000527085	2.529353103	99322.2898	4.959648338	56.0613032	0.000269928	2.562714111	99696.53505	4.981543682	57.99354441
35 to 39	0.000576161	2.557986419	99061.35353	4.946102234	51.20171287	0.000252337	2.587858219	99562.1228	4.975084904	53.06819227
40 to 44	0.000776944	2.597448761	98776.99217	4.929596641	46.34126075	0.00039198	2.726387988	99436.67488	4.967392648	48.1313892
45 to 49	0.001051271	2.63284065	98395.05173	4.907493911	41.51033812	0.000691921	2.768165727	99242.13507	4.954346935	43.21955821
50 to 54	0.001694762	2.62578939	97880.49548	4.874392063	36.71348037	0.001228788	2.718666868	98899.87706	4.931144176	38.35787056
55 to 59	0.002539077	2.617742911	97056.69948	4.823654547	32.00019161	0.002056303	2.644739915	98295.07407	4.890955617	33.57483163
60 to 64	0.003812817	2.623461222	95835.97065	4.748885333	27.37001352	0.002614326	2.679293601	97292.52448	4.835320463	28.89004581
65 to 69	0.00618817	2.632738126	94032.27225	4.63402907	22.83925582	0.004514972	2.724441377	96032.16657	4.752720744	24.23132224
70 to 74	0.010360235	2.662551954	91184.08422	4.452005948	18.4619484	0.007787832	2.76609215	93897.31883	4.614392128	19.71585977
75 to 79	0.019992828	2.660151959	86616.7175	4.139369545	14.28548362	0.015372413	2.815289639	90327.01766	4.369732143	15.37922244
80 to 84	0.041550074	2.604497897	78452.53527	3.57419607	10.48560297	0.035921177	2.731225983	83679.49014	3.871851351	11.37008239
85 to 89	0.086829169	2.569133799	63932.1935	2.652489226	7.268703842	0.075599847	2.638320358	70035.58813	2.984956661	8.014240197
90 to 94	0.17657181	2.249518919	41570.22434	1.411647148	4.837599369	0.151642554	2.336564	48243.11714	1.738402261	5.457424581
95 plus	0.280818524	3.574753424	17309.48984	0.630035777	3.574753424	0.251283029	4.018570339	22927.80719	0.949813233	4.018570339

Table 15: Israel 2017 life table, by age and sex. mx=mortality rate, ax=mean person-years lived in an age interval among those who die in that age interval, lx=number of persons left alive at age x, nLx=person-years lived between age x and x+n, ex=life expectancy at age x.

Age Group	Male					Female				
	mx	ax	lx	nLx	ex	mx	ax	lx	nLx	ex
Early Neonatal	0.073790202	0.009586779	100000	0.019164519	81.05795254	0.061458927	0.009587157	100000	0.019166784	84.52165455
Late Neonatal	0.010915476	0.028764112	99858.58539	0.057434848	81.15355058	0.009415569	0.028764526	99882.20336	0.057450911	84.60214773
Post Neonatal	0.001089005	0.461566475	99795.89334	0.920940123	81.14697974	0.000995177	0.46157314	99828.11046	0.921277324	84.59044155
1 to 4	0.000184931	1.999753425	99695.60427	3.986349616	80.30485937	0.000157785	1.99978962	99736.42792	3.988198437	83.74449159
5 to 9	0.000106831	2.499777436	99621.88566	4.979764203	76.36280522	8.20527E-05	2.499829057	99673.50057	4.982652864	79.79610144
10 to 14	0.000104173	2.955367816	99568.68722	4.977374032	71.40227016	0.000103148	2.623042719	99632.61678	4.980409798	74.82782026
15 to 19	0.000334765	2.710099638	99516.83681	4.97203046	66.43793019	0.000143143	2.560805887	99581.24489	4.977324484	69.86506568
20 to 24	0.000442816	2.585341062	99350.39134	4.962213697	61.54467415	0.000145181	2.578502799	99509.99801	4.973751407	64.91324504
25 to 29	0.000518023	2.546554441	99130.65768	4.950241438	56.6753356	0.000198169	2.648471524	99437.78874	4.969573649	59.95850108
30 to 34	0.000561125	2.566379307	98874.2258	4.936969731	51.815689	0.000286896	2.706826274	99339.30753	4.963699909	55.0153018
35 to 39	0.000700477	2.644331093	98597.20357	4.92173916	46.9540289	0.000484286	2.683656611	99196.90177	4.954287537	50.09037444
40 to 44	0.001052791	2.707758407	98252.45323	4.900796213	42.10945998	0.000716463	2.661034279	98956.97557	4.939571021	45.20527584
45 to 49	0.001765463	2.750385278	97736.5159	4.867494597	37.31739056	0.001043752	2.711114893	98603.08032	4.918403814	40.35791969
50 to 54	0.003220797	2.711515964	96877.21312	4.808420503	32.62389586	0.001789276	2.716653932	98089.73496	4.884531319	35.55487478
55 to 59	0.005162151	2.682104558	95328.61754	4.710077086	28.10964853	0.002945592	2.695295163	97215.79255	4.82801511	30.84997818
60 to 64	0.008057081	2.668346685	92897.4411	4.559231156	23.77488275	0.004657378	2.686827288	95793.74401	4.738640803	26.26778019
65 to 69	0.012455181	2.663031433	89224.54765	4.335069373	19.64346957	0.00739285	2.693122733	93586.98741	4.600895473	21.82360728
70 to 74	0.019700531	2.696797816	83826.28107	4.009441492	15.73669794	0.012213095	2.75816082	90186.13052	4.389158219	17.54472607
75 to 79	0.036820197	2.638166669	75930.0332	3.492889503	12.0925161	0.025026081	2.729607277	84826.96215	4.013390878	13.47855371
80 to 84	0.059573731	2.585407005	63075.62182	2.757406849	9.01926917	0.048964539	2.657731025	74787.27238	3.354833385	9.921170358
85 to 89	0.11343996	2.483709708	46660.27253	1.815258305	6.283518004	0.096692985	2.55251424	58371.29704	2.360424513	6.96406119
90 to 94	0.20770243	2.222940208	26083.4767	0.827303051	4.28314001	0.179428684	2.310721892	35566.6798	1.199864203	4.794212596
95 plus	0.307054556	3.256934768	8910.846675	0.29034258	3.256934768	0.277910271	3.598748125	14055.32013	0.506132705	3.598748125

Table 15: Israel 2100 life table, by age and sex. mx=mortality rate, ax=mean person-years lived in an age interval among those who die in that age interval, lx=number of persons left alive at age x, nLx=person-years lived between age x and x+n, ex=life expectancy at age x.

Age Group	Male					Female				
	mx	ax	lx	nLx	ex	mx	ax	lx	nLx	ex
Early Neonatal	0.018681613	0.009588469	100000	0.019174647	84.886963	0.01628372	0.009588542	100000	0.019175088	87.5557318
Late Neonatal	0.003220288	0.028766235	99964.17983	0.05750831	84.89819811	0.002912417	0.02876632	99968.77656	0.057511464	87.56388476
Post Neonatal	0.000273608	0.461624399	99945.66118	0.922669425	84.85638935	0.000252319	0.461625911	99952.02732	0.922737263	87.52100999
1 to 4	7.74365E-05	1.999896751	99920.41725	3.996197799	83.95442668	8.22228E-05	1.99989037	99928.74574	3.996492677	86.6179968
5 to 9	5.78369E-05	2.499879507	99889.47491	4.993751778	79.97977031	5.46013E-05	2.499886246	99895.89106	4.994113003	82.64580809
10 to 14	6.92488E-05	3.066642682	99860.59857	4.992332306	75.00210877	8.16891E-05	2.686225883	99868.6313	4.992459585	77.66769089
15 to 19	0.000239017	2.795999527	99826.03801	4.988640772	70.02700158	0.000101638	2.529923532	99827.86604	4.990140286	72.69833621
20 to 24	0.000399013	2.652426831	99706.88519	4.980609642	65.10706494	9.50537E-05	2.610021721	99777.17432	4.987701527	67.73395641
25 to 29	0.000467126	2.55703829	99508.45574	4.969699367	60.23129829	0.000127155	2.653197579	99729.79304	4.984983818	62.76491141
30 to 34	0.000474945	2.527711504	99276.74089	4.958003669	55.36567149	0.000171417	2.693408431	99666.43571	4.981330477	57.80304899
35 to 39	0.000515218	2.591327147	99041.71985	4.945926992	50.49093074	0.000268055	2.650044904	99581.08605	4.975914165	52.85011482
40 to 44	0.000683615	2.683861581	98787.50084	4.931522113	45.61395614	0.000362829	2.697949032	99447.75607	4.968198163	47.91704369
45 to 49	0.001096505	2.764437048	98451.03524	4.910452822	40.76015706	0.000579113	2.745322446	99267.6028	4.956905488	42.99854022
50 to 54	0.002085398	2.704161062	97913.62662	4.872260995	35.96705271	0.0010562	2.7365189	98980.69971	4.937163519	38.11440215
55 to 59	0.003147504	2.64822162	96899.79642	4.809407041	31.3117583	0.001731468	2.688468138	98459.85849	4.903347492	33.2994656
60 to 64	0.004478105	2.65787277	95389.82095	4.72011178	26.76117567	0.002642784	2.669155442	97611.99874	4.850752761	28.5630544
65 to 69	0.006918015	2.695462548	93284.30863	4.591201762	22.29886687	0.003971538	2.717579854	96332.92813	4.773367662	23.90297419
70 to 74	0.01181809	2.751728907	90125.19263	4.390220216	17.97958432	0.006931586	2.855048868	94443.48189	4.653085179	19.32211197
75 to 79	0.02407117	2.699040075	84980.61953	4.028314369	13.89165961	0.016860097	2.830086282	91231.39638	4.401361877	14.89521621
80 to 84	0.043730839	2.647974372	75412.87356	3.425288357	10.30169272	0.039557183	2.731697345	83863.28954	3.852299077	10.94143398
85 to 89	0.090224615	2.558166422	60763.93557	2.502760203	7.138114277	0.081631066	2.61345076	68904.83485	2.896772003	7.695626447
90 to 94	0.180610804	2.24675772	38857.64381	1.310042823	4.763462711	0.159762909	2.331172082	45983.24246	1.628734019	5.253001994
95 plus	0.284232352	3.532239437	15854.71732	0.57086581	3.532239437	0.259087756	3.888898428	20836.04312	0.831185208	3.888898428

Table 15: Italy 2017 life table, by age and sex. mx=mortality rate, ax=mean person-years lived in an age interval among those who die in that age interval, lx=number of persons left alive at age x, nLx=person-years lived between age x and x+n, ex=life expectancy at age x.

Age Group	Male					Female				
	mx	ax	lx	nLx	ex	mx	ax	lx	nLx	ex
Early Neonatal	0.077725629	0.009586659	100000	0.019163796	80.90143242	0.059919282	0.009587205	100000	0.019167067	85.40805086
Late Neonatal	0.010280628	0.028764287	99851.05023	0.057431563	81.0029192	0.009282246	0.028764563	99885.15317	0.057452828	85.48706308
Post Neonatal	0.000923772	0.461578212	99792.0089	0.920974521	80.99329342	0.00076986	0.461589146	99831.82503	0.921407432	85.47517997
1 to 4	0.000131548	1.999824603	99706.9353	3.987228329	80.13872059	0.000112421	1.999850105	99760.89121	3.98953858	84.61234222
5 to 9	8.64785E-05	2.499819837	99654.48577	4.981647222	76.17984746	6.96084E-05	2.499854982	99716.04116	4.984934532	80.64950037
10 to 14	0.000108062	2.871229383	99611.40622	4.979424747	71.21171309	7.4888E-05	2.629258163	99681.34213	4.983182441	75.67670461
15 to 19	0.000279221	2.718641841	99557.59831	4.974711081	66.24864731	0.000116194	2.664667839	99644.02423	4.980849719	70.70405946
20 to 24	0.000401949	2.579272759	99418.69466	4.966102655	61.33739414	0.000166948	2.596278849	99586.14978	4.977310164	65.74359411
25 to 29	0.000433522	2.560776139	99219.0835	4.955713722	56.45558401	0.000193643	2.576188194	99503.05506	4.972818769	60.79631822
30 to 34	0.000530503	2.592986609	99004.24356	4.94389932	51.57251522	0.000238186	2.68534152	99406.76019	4.967599394	55.85270361
35 to 39	0.00067341	2.664082626	98741.97071	4.929344758	46.70258647	0.000406486	2.70619627	99288.43955	4.959797468	50.91604739
40 to 44	0.00106709	2.704467485	98410.02834	4.908478215	41.85109624	0.000642487	2.725276555	99086.83249	4.94711139	46.01410933
45 to 49	0.001735449	2.727944331	97886.26048	4.875090839	37.06050701	0.00110677	2.717747654	98768.9917	4.926006843	41.15337354
50 to 54	0.003007778	2.717227102	97040.24097	4.818925982	32.35974235	0.001814793	2.690401218	98223.80797	4.890691774	36.36664565
55 to 59	0.004988396	2.695008976	95590.88792	4.725215702	27.80905458	0.002804937	2.675036542	97336.27872	4.8352825	31.67362078
60 to 64	0.00798607	2.68590021	93233.94365	4.577117403	23.44376028	0.00426532	2.687834661	95980.08372	4.752141453	27.08324463
65 to 69	0.012919877	2.682384985	89579.03277	4.34875566	19.2904892	0.006884557	2.705428825	93953.30365	4.624618592	22.60934371
70 to 74	0.021447289	2.663245115	83961.45223	3.997765705	15.40143618	0.011689007	2.711059017	90769.85965	4.420249416	18.30717928
75 to 79	0.035445666	2.657406299	75389.64463	3.480583048	11.84955014	0.020582434	2.740842775	85604.1152	4.090074448	14.24802877
80 to 84	0.065674558	2.605547503	63057.68812	2.724659245	8.647238041	0.042363369	2.695123427	77188.64973	3.516254617	10.50233063
85 to 89	0.121764412	2.458734481	45174.01079	1.725213721	6.039727459	0.086755202	2.589545595	62300.34919	2.576559752	7.368114013
90 to 94	0.216583563	2.208983594	24180.3993	0.753687526	4.15059737	0.167269532	2.329571219	39962.23108	1.381465661	5.040288252
95 plus	0.31436952	3.181118353	7865.383937	0.250297658	3.181118353	0.266446934	3.753520784	16869.89451	0.633536025	3.753520784

Table 15: Italy 2100 life table, by age and sex. mx=mortality rate, ax=mean person-years lived in an age interval among those who die in that age interval, lx=number of persons left alive at age x, nLx=person-years lived between age x and x+n, ex=life expectancy at age x.

Age Group	Male					Female				
	mx	ax	lx	nLx	ex	mx	ax	lx	nLx	ex
Early Neonatal	0.017948753	0.009588491	100000	0.019174782	85.42369296	0.013798776	0.009588618	100000	0.019175545	88.4050814
Late Neonatal	0.002482123	0.028766439	99965.58518	0.05751034	85.4339415	0.002632682	0.028766397	99973.54076	0.057514668	88.40929975
Post Neonatal	0.000237867	0.461626938	99951.31102	0.922736806	85.38861711	0.000200865	0.461629567	99958.39942	0.922818006	88.36515691
1 to 4	3.73772E-05	1.999950163	99929.36306	3.996875738	84.48398841	3.29372E-05	1.999956084	99939.86368	3.997331227	87.45817348
5 to 9	3.15563E-05	2.499934258	99914.42427	4.995327131	80.49631955	3.34371E-05	2.499930339	99926.69806	4.995917287	83.46943441
10 to 14	5.06318E-05	3.167989437	99898.66158	4.994445244	75.50860053	4.25685E-05	2.769757586	99909.99404	4.995016422	78.48296944
15 to 19	0.000170356	2.781930788	99873.37596	4.991783168	70.52689795	7.98446E-05	2.710821736	99888.73254	4.993523133	73.4990965
20 to 24	0.000281932	2.617233758	99788.34695	4.986067031	65.584421	0.000122628	2.594007557	99848.86507	4.990963842	68.52732334
25 to 29	0.000328111	2.581142478	99647.8154	4.978425017	60.67288287	0.000128497	2.541671915	99787.6709	4.987803631	63.56772224
30 to 34	0.000394761	2.576140652	99484.55964	4.969456152	55.76788452	0.00014407	2.653473352	99723.59079	4.984486178	58.60688458
35 to 39	0.000454556	2.617390392	99288.52034	4.959043492	50.87260752	0.000226608	2.692444762	99651.79392	4.979978663	53.64711574
40 to 44	0.000638241	2.668980057	99063.27124	4.945795279	45.98200331	0.000346845	2.745899117	99538.96465	4.973056753	48.70469832
45 to 49	0.000961833	2.72857776	98747.85494	4.926614856	41.11991144	0.000633809	2.744642781	99366.51234	4.961222783	43.78415567
50 to 54	0.001684071	2.714197436	98274.38604	4.894878035	36.30413163	0.0010839	2.71199089	99052.16574	4.940358213	38.91372339
55 to 59	0.002715739	2.677192388	97450.73051	4.842006156	31.58682672	0.001748936	2.688480789	98516.91467	4.906013469	34.10926804
60 to 64	0.00406157	2.688097683	96137.20336	4.762188649	26.97986711	0.002689867	2.69661823	97659.59056	4.852924942	29.38304781
65 to 69	0.00658254	2.715340488	94205.79958	4.640614495	22.47530663	0.0043588	2.716028664	96355.93724	4.770345805	24.74116607
70 to 74	0.011439169	2.726179093	91157.75642	4.442666471	18.13206489	0.007415751	2.742130689	94280.9744	4.636579158	20.22175698
75 to 79	0.02082041	2.752378007	86094.06689	4.113179231	14.03245714	0.01370912	2.813156775	90854.27437	4.411028819	15.87463801
80 to 84	0.043601819	2.704260303	77587.99751	3.529946982	10.26218316	0.03150532	2.765556814	84844.30088	3.965979894	11.79002915
85 to 89	0.090543789	2.556519979	62383.04981	2.561759057	7.09790815	0.068837297	2.664253239	72506.6708	3.130962285	8.309354311
90 to 94	0.18136499	2.249429564	39585.54367	1.327862966	4.738037456	0.143157281	2.347842137	51389.29808	1.874156494	5.639664422
95 plus	0.284935533	3.517588367	15891.91509	0.565335878	3.517588367	0.243241179	4.133474009	25165.12932	1.057924183	4.133474009

Table 15: Luxembourg 2017 life table, by age and sex. mx=mortality rate, ax=mean person-years lived in an age interval among those who die in that age interval, lx=number of persons left alive at age x, nLx=person-years lived between age x and x+n, ex=life expectancy at age x.

Age Group	Male					Female				
	mx	ax	lx	nLx	ex	mx	ax	lx	nLx	ex
Early Neonatal	0.049155817	0.009587534	100000	0.019169045	79.93480778	0.032467428	0.009588046	100000	0.019172113	83.33162154
Late Neonatal	0.006518503	0.028765325	99905.7744	0.057469258	79.99101344	0.004502261	0.028765881	99937.75376	0.057490987	83.36434334
Post Neonatal	0.000673379	0.461596	99868.3144	0.921785265	79.96347495	0.000510406	0.461607577	99911.87003	0.922256657	83.32839776
1 to 4	9.4942E-05	1.999873411	99806.24517	3.99149187	79.08963015	0.000116049	1.999845267	99864.79832	3.993664957	82.44416923
5 to 9	7.41779E-05	2.499845463	99768.35076	4.987492601	75.11891296	6.06333E-05	2.499873681	99818.45316	4.990166205	78.48152048
10 to 14	0.000100126	2.943496872	99731.35557	4.985541109	70.14585171	7.91151E-05	2.964757842	99788.19657	4.988606571	73.50456114
15 to 19	0.000287497	2.838235111	99681.43837	4.980976454	65.17949776	0.000237326	2.642968583	99748.72947	4.984648193	68.53246723
20 to 24	0.000568403	2.593601577	99538.23794	4.970113712	60.26913373	0.00024238	2.534943061	99630.43143	4.978547082	63.61067858
25 to 29	0.000544928	2.502942894	99255.73873	4.956043225	55.4331888	0.000278697	2.580990362	99509.76246	4.972136406	58.68471812
30 to 34	0.0005792	2.647781009	98985.67665	4.942550708	50.57751029	0.000351881	2.705093279	99371.19197	4.964551055	53.76292189
35 to 39	0.000961338	2.627617022	98699.41415	4.923742349	45.71643054	0.000627481	2.659589419	99196.50149	4.95255227	48.85278709
40 to 44	0.001176932	2.66880652	98226.10196	4.897868403	40.92389549	0.000836544	2.720900444	98885.74637	4.934878248	43.99785865
45 to 49	0.001934741	2.772465237	97649.70497	4.86153527	36.14950782	0.001524797	2.688252544	98472.94051	4.906352818	39.17077492
50 to 54	0.003767987	2.694898089	96709.24243	4.793830617	31.47378172	0.002239341	2.684841792	97724.86875	4.861043263	34.4498586
55 to 59	0.005613509	2.689896348	94903.28451	4.684429713	27.02091293	0.003575559	2.707755082	96636.42132	4.792545371	29.80739161
60 to 64	0.009302742	2.67417605	92274.44928	4.51604555	22.71347211	0.005974513	2.690318585	94923.06429	4.681564424	25.29619766
65 to 69	0.014411527	2.673594823	88075.03455	4.260974951	18.66820402	0.009470861	2.684836601	92126.66591	4.50753231	20.98189146
70 to 74	0.024025077	2.654844873	81938.14156	3.878573329	14.86536053	0.015526783	2.702732778	87859.14789	4.241737259	16.86999654
75 to 79	0.039320413	2.638876163	72628.86344	3.32332695	11.42983148	0.02796451	2.698291568	81277.00635	3.818281456	13.01660832
80 to 84	0.070901582	2.572755045	59580.83247	2.542364796	8.355364859	0.053380008	2.643808144	70609.45488	3.136486687	9.575038364
85 to 89	0.128398442	2.439370931	41590.96897	1.565876823	5.859355687	0.103135275	2.529680515	53889.74902	2.148118325	6.726277598
90 to 94	0.223481687	2.197105738	21528.06106	0.662323881	4.053170396	0.18705447	2.2972334	31771.7653	1.055751149	4.651325287
95 plus	0.320004076	3.125408588	6752.10048	0.211281898	3.125408588	0.285033204	3.509162626	12054.83281	0.423537739	3.509162626

Table 15: Luxembourg 2100 life table, by age and sex. mx=mortality rate, ax=mean person-years lived in an age interval among those who die in that age interval, lx=number of persons left alive at age x, nLx=person-years lived between age x and x+n, ex=life expectancy at age x.

Age Group	Male					Female				
	mx	ax	lx	nLx	ex	mx	ax	lx	nLx	ex
Early Neonatal	0.014383535	0.0095886	100000	0.019175437	85.75117151	0.008866651	0.009588769	100000	0.019176452	86.9070157
Late Neonatal	0.002095336	0.028766545	99972.42019	0.057514912	85.75564584	0.001245776	0.02876678	99982.99736	0.057522403	86.90261316
Post Neonatal	0.000129681	0.461634624	99960.36937	0.922866518	85.70844511	0.00010172	0.461636609	99975.8315	0.923021183	86.85130282
1 to 4	2.54924E-05	1.99996601	99948.40188	3.997732251	84.79536836	3.7537E-05	1.999949951	99966.44269	3.998357531	85.93612924
5 to 9	2.53094E-05	2.49994726	99938.21086	4.996594448	80.80381136	2.48388E-05	2.499948259	99951.43425	4.997261409	81.94873296
10 to 14	4.98895E-05	3.111046148	99925.5685	4.99574495	75.81371577	5.06821E-05	3.11510555	99939.02272	4.996470191	76.95857981
15 to 19	0.000137848	3.003519426	99900.65783	4.993534456	70.83189316	0.000174058	2.68020873	99913.70012	4.993638073	71.97729447
20 to 24	0.000319914	2.613885488	99831.85107	4.987762725	65.87857321	0.0001726	2.541497139	99826.80354	4.989214663	67.0375238
25 to 29	0.000317526	2.495253863	99672.35908	4.979670178	60.9794461	0.000198963	2.527923385	99740.70946	4.98458779	62.09314697
30 to 34	0.000319566	2.657437152	99514.37412	4.971938969	56.07194418	0.000202613	2.725323277	99641.57934	4.979739988	57.15225412
35 to 39	0.000525339	2.581316778	99355.67904	4.961483796	51.15704117	0.000376291	2.649474017	99540.73649	4.972624315	52.20720872
40 to 44	0.000556908	2.611708126	99095.29893	4.948172229	46.28433195	0.00045969	2.740421592	99353.69458	4.962505036	47.30018265
45 to 49	0.000853988	2.812156334	98820.2107	4.931670194	41.40537085	0.000885825	2.67284107	99125.68386	4.946115806	42.40220066
50 to 54	0.001885374	2.683142968	98399.82135	4.898558061	36.5694038	0.001231693	2.715763108	98687.76758	4.920433229	37.57756058
55 to 59	0.002719148	2.661271181	97477.67924	4.84312054	31.88814976	0.002060902	2.745276348	98082.56972	4.881459127	32.79070852
60 to 64	0.004387081	2.653141999	96163.12256	4.75919192	27.28479373	0.003731361	2.689911228	97077.49273	4.812388825	28.10016775
65 to 69	0.006564838	2.663053665	94081.23143	4.633162912	22.82527988	0.005541985	2.693079975	95286.03317	4.704134355	23.5740445
70 to 74	0.010863956	2.679978151	91050.85575	4.441241387	18.4914723	0.009082147	2.776359425	92687.42063	4.542539273	19.15592252
75 to 79	0.019176986	2.727505195	86255.88961	4.133934654	14.36359779	0.018516924	2.777404654	88581.85547	4.254071489	14.91062134
80 to 84	0.040538694	2.649230783	78425.3505	3.583908494	10.52091388	0.037711229	2.742602241	80772.7202	3.724584552	11.07875739
85 to 89	0.085759377	2.572012306	64090.31522	2.65968887	7.281118397	0.078971962	2.622675501	66904.59147	2.824981175	7.788513141
90 to 94	0.175685802	2.253584935	41660.36236	1.412115618	4.841710006	0.156666638	2.337108595	45062.55278	1.601074056	5.308115808
95 plus	0.280134645	3.577033346	17223.88344	0.622387995	3.577033346	0.256209145	3.923374534	20560.92559	0.82111478	3.923374534

Table 15: Malta 2017 life table, by age and sex. mx=mortality rate, ax=mean person-years lived in an age interval among those who die in that age interval, lx=number of persons left alive at age x, nLx=person-years lived between age x and x+n, ex=life expectancy at age x.

Age Group	Male					Female				
	mx	ax	lx	nLx	ex	mx	ax	lx	nLx	ex
Early Neonatal	0.184834657	0.009583376	100000	0.019144132	79.00958899	0.142444637	0.009584675	100000	0.019151911	82.95209688
Late Neonatal	0.014656828	0.02876308	99646.1565	0.0573065	79.27093799	0.014069028	0.028763242	99727.19408	0.057354074	83.15980311
Post Neonatal	0.001365977	0.461546799	99562.16648	0.918665821	79.28025169	0.001084422	0.4615668	99646.50407	0.919563483	83.1695803
1 to 4	0.000156005	1.999791993	99436.68874	3.97622687	78.45643212	0.000159663	1.999787117	99546.78983	3.980600392	82.32913904
5 to 9	0.000105176	2.499780884	99374.66131	4.967426865	74.50415787	0.000127006	2.499735404	99483.23657	4.972582813	78.38045576
10 to 14	0.000173985	2.697059284	99322.41788	4.964131579	69.54203353	8.48785E-05	2.523654515	99420.08264	4.969959486	73.42865635
15 to 19	0.000269766	2.751443999	99236.05135	4.958794688	64.60020858	0.000136758	2.659459086	99377.89869	4.967305019	68.45875159
20 to 24	0.000500758	2.596394615	99102.2807	4.949157096	59.68368372	0.000189802	2.596618104	99309.96716	4.963234333	63.50375373
25 to 29	0.000503205	2.666531162	98854.44841	4.936925418	54.82678529	0.000225158	2.592785632	99215.76423	4.958100938	58.56157357
30 to 34	0.000907401	2.595314126	98606.02109	4.919566636	49.95818018	0.000290657	2.674815199	99104.12951	4.951859964	53.62460656
35 to 39	0.000924412	2.603449116	98159.62234	4.897132248	45.17354118	0.000470301	2.666009965	98960.20132	4.942584775	48.69869248
40 to 44	0.00137849	2.635997702	97706.93071	4.869478417	40.37074268	0.000667835	2.708383335	98727.75364	4.928844366	43.80704512
45 to 49	0.001844019	2.705860071	97035.6898	4.83134619	35.63172408	0.001144422	2.741906446	98398.59295	4.90724832	38.9444904
50 to 54	0.00325135	2.728900579	96144.80414	4.772003965	30.93675822	0.002014985	2.705653115	97837.00885	4.869339633	34.15223693
55 to 59	0.005557727	2.700844381	94593.31948	4.669994856	26.39932023	0.003182468	2.712749022	96855.87595	4.80779877	29.47069518
60 to 64	0.008994641	2.693294401	91998.01413	4.506408442	22.06773511	0.005402412	2.718928885	95325.88555	4.708277177	24.90003507
65 to 69	0.014951892	2.6980455	87945.00168	4.250955125	17.9604622	0.009251631	2.717375922	92782.48107	4.54319282	20.50788469
70 to 74	0.026255499	2.677887205	81589.83394	3.845105986	14.14908834	0.016222581	2.728241551	88579.84488	4.271598949	16.35167288
75 to 79	0.046531154	2.620378161	71496.38898	3.21854938	10.76835503	0.030949603	2.697083938	81651.79821	3.811051671	12.50725788
80 to 84	0.079165078	2.556469594	56525.02296	2.368325273	7.926519502	0.059063119	2.61570444	69861.38347	3.062098834	9.162642869
85 to 89	0.138845012	2.409541779	37784.81863	1.389676869	5.5906371	0.111177911	2.502166181	51786.71496	2.026894033	6.448014936
90 to 94	0.234149555	2.177732984	18499.38728	0.557031976	3.90856859	0.196376676	2.279595474	29269.11248	0.954135938	4.485427158
95 plus	0.328662326	3.042732126	5461.808124	0.166234659	3.042732126	0.293682749	3.405375515	10545.67416	0.359319177	3.405375515

Table 15: Malta 2100 life table, by age and sex. mx=mortality rate, ax=mean person-years lived in an age interval among those who die in that age interval, lx=number of persons left alive at age x, nLx=person-years lived between age x and x+n, ex=life expectancy at age x.

Age Group	Male					Female				
	mx	ax	lx	nLx	ex	mx	ax	lx	nLx	ex
Early Neonatal	0.050607408	0.00958749	100000	0.019168779	83.98390344	0.040018413	0.009587815	100000	0.019170725	86.40727284
Late Neonatal	0.003298647	0.028766213	99902.99687	0.057472983	84.04628479	0.004166845	0.028765974	99923.28489	0.057483219	86.45442399
Post Neonatal	0.000375913	0.461617131	99884.04033	0.922057017	84.00469976	0.000304044	0.461622237	99899.33376	0.922228789	86.41760484
1 to 4	3.76887E-05	1.999949748	99849.38136	3.993674224	83.11042382	4.57909E-05	1.999938945	99871.29592	3.994486017	85.51845443
5 to 9	4.74471E-05	2.499901157	99834.33022	4.991124503	79.12265121	7.25722E-05	2.499848804	99853.00555	4.991744864	81.53375768
10 to 14	0.000133545	2.754899532	99810.65198	4.988991206	74.14074526	5.38165E-05	2.605639407	99816.79703	4.990190488	76.56236707
15 to 19	0.000166521	2.805897727	99744.05798	4.985403949	69.18829178	0.000102456	2.795394025	99789.95261	4.988293478	71.5822635
20 to 24	0.000400724	2.597274283	99661.10092	4.978293224	64.24284066	0.000136708	2.688657519	99738.8996	4.985317665	66.61736663
25 to 29	0.000376716	3.065320802	99462.03739	4.969059102	59.36467653	0.000172775	2.584179744	99670.80095	4.98144589	61.66097626
30 to 34	0.00100757	2.598742339	99275.60565	4.951727751	54.46952662	0.000194176	2.640388128	99584.79893	4.976939039	56.71175859
35 to 39	0.000751957	2.519142726	98778.37178	4.929687102	49.7289783	0.00029175	2.614652374	99488.22913	4.97092646	51.76392005
40 to 44	0.0010184	2.544078243	98409.06944	4.908244757	44.90519697	0.000341582	2.75287533	99343.30227	4.963343576	46.83520097
45 to 49	0.001003097	2.704391771	97910.97795	4.884185693	40.11924364	0.000738988	2.759642896	99173.86887	4.950435455	41.91006966
50 to 54	0.001845428	2.744159264	97423.89545	4.850907098	35.30422255	0.001295266	2.671160505	98808.4186	4.925543781	37.05366336
55 to 59	0.003055113	2.659862192	96531.99858	4.792405908	30.60249449	0.001896613	2.713303924	98171.17642	4.887315508	32.27507748
60 to 64	0.004277135	2.689166905	95072.22329	4.707303162	26.02803045	0.003399114	2.690560879	97245.85017	4.824432861	27.5540367
65 to 69	0.007273389	2.742086487	93066.99086	4.57851966	21.52535223	0.005412249	2.71556336	95609.36513	4.722125931	22.97646389
70 to 74	0.013479407	2.769360028	89759.03214	4.357816674	17.20850761	0.009925501	2.747967723	93060.73435	4.551509117	18.52745131
75 to 79	0.027405596	2.712129857	83946.50844	3.952763973	13.19643278	0.020032804	2.741979158	88557.81703	4.237193688	14.32430103
80 to 84	0.051085492	2.664228877	73294.37797	3.282027341	9.710141616	0.043416814	2.661129342	80108.83539	3.640674132	10.53554217
85 to 89	0.101134388	2.523353142	56946.4088	2.291320054	6.735812954	0.087618647	2.58965434	64549.53387	2.676056222	7.409723414
90 to 94	0.193356775	2.236235983	34498.2938	1.135711112	4.536428756	0.167522429	2.323139417	41714.9884	1.453347386	5.073276124
95 plus	0.294962497	3.402090059	13150.33697	0.456038172	3.402090059	0.266493191	3.775246212	18063.19895	0.697068551	3.775246212

Table 15: Netherlands 2017 life table, by age and sex. mx=mortality rate, ax=mean person-years lived in an age interval among those who die in that age interval, lx=number of persons left alive at age x, nLx=person-years lived between age x and x+n, ex=life expectancy at age x.

Age Group	Male					Female				
	mx	ax	lx	nLx	ex	mx	ax	lx	nLx	ex
Early Neonatal	0.104393049	0.009585841	100000	0.019158897	79.96898992	0.086478407	0.009586391	100000	0.019162188	83.22577179
Late Neonatal	0.012101495	0.028763785	99799.99779	0.057399193	80.11004095	0.010108375	0.028764335	99834.29012	0.057422208	83.3447175
Post Neonatal	0.00097506	0.461574569	99730.53894	0.920385435	80.10827888	0.000827299	0.461585066	99776.24697	0.920870056	83.33565133
1 to 4	0.000161024	1.999785302	99640.80038	3.984348785	79.25672246	0.00011731	1.999843587	99700.06574	3.987067134	82.47568959
5 to 9	6.10069E-05	2.499872902	99576.6458	4.978073032	75.30649768	6.33563E-05	2.499868008	99653.29465	4.981875618	78.51346085
10 to 14	8.43333E-05	2.910802673	99546.27702	4.976436959	70.32870917	7.64991E-05	2.701667328	99621.73174	4.980211001	73.53754438
15 to 19	0.000227377	2.762977422	99504.30984	4.972686192	65.3571418	0.00013753	2.676900048	99583.63383	4.977591441	68.56464159
20 to 24	0.000371998	2.614344097	99391.24329	4.965155772	60.42833571	0.000193544	2.623809177	99515.17714	4.973471631	63.60995754
25 to 29	0.000432722	2.582546848	99206.54162	4.955143593	55.53595276	0.00025299	2.637607263	99418.91917	4.967976837	58.66899194
30 to 34	0.000545501	2.603179488	98992.1234	4.943143328	50.6506286	0.000361433	2.664504292	99293.2353	4.96047467	53.73989818
35 to 39	0.000706196	2.659157341	98722.47738	4.927977722	45.7818358	0.000540092	2.669760809	99113.94908	4.949468382	48.8322631
40 to 44	0.001091588	2.705810124	98374.47132	4.906436682	40.93434724	0.000804935	2.725936611	98846.63584	4.933301394	43.95706069
45 to 49	0.001800403	2.721616681	97838.90334	4.871960905	36.14355139	0.001422152	2.761425691	98449.54522	4.906855888	39.12330956
50 to 54	0.003050275	2.725871608	96961.78585	4.814692288	31.44579546	0.002619314	2.717036733	97751.73572	4.858534625	34.38279637
55 to 59	0.005233208	2.714369117	95493.25367	4.718230242	26.88732458	0.004235947	2.673847629	96479.19005	4.776893312	29.80032321
60 to 64	0.00879374	2.690446011	93024.31227	4.558640977	22.52870184	0.006362673	2.663674493	94455.86971	4.65362325	25.38119813
65 to 69	0.014234143	2.68624531	89016.0551	4.308915971	18.42174393	0.009722822	2.678568586	91495.25151	4.473802153	21.1160529
70 to 74	0.024197478	2.665772286	82883.85324	3.922691184	14.58566215	0.015920752	2.677570701	87146.22569	4.201984884	17.03587562
75 to 79	0.040878976	2.648308629	73394.83018	3.348022308	11.12650681	0.026513084	2.695704975	80458.30046	3.791377657	13.22904837
80 to 84	0.076320618	2.577647516	59715.06092	2.520144816	8.068785633	0.051315485	2.660049066	70410.8442	3.143335686	9.731941407
85 to 89	0.13541064	2.419222145	40494.02316	1.500674286	5.676179347	0.100190913	2.539963521	54291.37931	2.178150382	6.831992861
90 to 94	0.230677787	2.184212913	20188.08709	0.6120925	3.954509181	0.183600729	2.303535555	32485.82436	1.086726698	4.714623781
95 plus	0.32585507	3.068998358	6076.961383	0.186578853	3.068998358	0.281816138	3.548811534	12548.80711	0.445594696	3.548811534

**Table 15: Netherlands 2100 life table, by age and sex. mx=mortality rate, ax=mean person-years lived in an age interval among those who die in that age interval, lx=number of persons left alive at age x, nLx=person-years lived between age x and x+n, ex=life expectancy at age x.**										
**Age Group**	**Male**					**Female**				
	**mx**	**ax**	**lx**	**nLx**	**ex**	**mx**	**ax**	**lx**	**nLx**	**ex**
Early Neonatal	0.033135496	0.009588025	100000	0.01917199	84.81862819	0.031593439	0.009588073	100000	0.019172274	85.6988444
Late Neonatal	0.003567213	0.028766139	99936.48044	0.057491801	84.85336405	0.003846894	0.028766062	99939.43419	0.057493038	85.73158981
Post Neonatal	0.000254204	0.461625777	99915.97321	0.92240362	84.81325036	0.000232553	0.461627315	99917.31845	0.922425255	85.69301968
1 to 4	4.33515E-05	1.999942198	99892.52724	3.995354688	83.9097671	3.62866E-05	1.999951618	99895.86846	3.995544781	84.78803772
5 to 9	2.20982E-05	2.499953971	99875.20784	4.993484537	79.92396397	3.25739E-05	2.499932138	99881.37109	4.993661906	80.8000431
10 to 14	4.6375E-05	3.204244361	99864.17407	4.992757978	74.93249466	5.04038E-05	2.817318472	99865.10589	4.992691327	75.81277671
15 to 19	0.000146396	2.832739173	99841.0231	4.990463058	69.94912939	9.52098E-05	2.660033159	99839.94332	4.990886689	70.83117323
20 to 24	0.000277929	2.660856003	99767.97606	4.985149617	64.99807116	0.000124875	2.619917571	99792.43174	4.988127573	65.86359036
25 to 29	0.000354379	2.606303468	99629.47941	4.977231233	60.0842841	0.000155835	2.62156087	99730.15941	4.984649631	60.90306062
30 to 34	0.000438842	2.575728264	99453.21111	4.967360168	55.18571399	0.000205109	2.627706801	99652.50665	4.980189981	55.94843081
35 to 39	0.000497596	2.595611081	99235.39559	4.95584724	50.30082671	0.000269332	2.660150258	99550.40425	4.97436241	51.00298814
40 to 44	0.000674612	2.677457683	98989.00545	4.941687988	45.41922034	0.000389392	2.766202211	99416.4914	4.966497273	46.06785691
45 to 49	0.001055065	2.729996987	98656.08389	4.920990621	40.56290856	0.00076075	2.82434859	99223.17974	4.952913632	41.15183197
50 to 54	0.00182159	2.723023164	98137.60861	4.886629029	35.76195667	0.001532308	2.777893402	98846.62965	4.925495865	36.29690764
55 to 59	0.003051259	2.680060822	97248.77741	4.828234005	31.06231346	0.002763582	2.730110038	98092.48735	4.873960766	31.55308059
60 to 64	0.004475148	2.676950434	95778.84646	4.739717639	26.49484045	0.004557706	2.695937019	96747.32587	4.787006714	26.95134566
65 to 69	0.007077788	2.694279256	93662.25007	4.608148742	22.02991586	0.00712453	2.684459239	94569.77427	4.651962265	22.50619734
70 to 74	0.01180649	2.735753743	90410.66438	4.403310284	17.72048274	0.011505189	2.720969357	91266.59159	4.446954766	18.21707236
75 to 79	0.022542906	2.756302354	85239.75894	4.058072538	13.62271671	0.020676926	2.770330998	86183.54294	4.120287037	14.12298143
80 to 84	0.047953335	2.687868262	76168.02273	3.432698601	9.909931765	0.045150057	2.720131974	77743.68678	3.528338719	10.34790904
85 to 89	0.096951901	2.536011216	59939.24248	2.427806316	6.862069741	0.090606862	2.57757735	62049.61709	2.55386307	7.265541353
90 to 94	0.188843998	2.242966742	36847.88771	1.219128665	4.605333776	0.171415074	2.318969437	39427.22001	1.361367376	4.983046046
95 plus	0.291233962	3.441520395	14225.3385	0.495487833	3.441520395	0.270217894	3.71819842	16650.85512	0.630992045	3.71819842

Table 15: Norway 2017 life table, by age and sex. mx=mortality rate, ax=mean person-years lived in an age interval among those who die in that age interval, lx=number of persons left alive at age x, nLx=person-years lived between age x and x+n, ex=life expectancy at age x.

Age Group	Male					Female				
	mx	ax	lx	nLx	ex	mx	ax	lx	nLx	ex
Early Neonatal	0.064653288	0.009587059	100000	0.019166197	80.53816145	0.047212703	0.009587594	100000	0.019169402	84.22434071
Late Neonatal	0.007479168	0.02876506	99876.08438	0.057450591	80.61889427	0.005639137	0.028765568	99909.49612	0.057472852	84.28144935
Post Neonatal	0.000756323	0.461590108	99833.11623	0.9214251	80.59604597	0.000680636	0.461595484	99877.08643	0.921863136	84.2512548
1 to 4	0.000111272	1.999851637	99763.42686	3.989649139	79.72873586	0.00010047	1.99986604	99814.34121	3.991771488	83.38063908
5 to 9	4.44947E-05	2.499907303	99719.03338	4.98539709	75.76333971	3.82198E-05	2.499920376	99774.23587	4.988235156	79.413351
10 to 14	0.000122688	2.945736538	99696.85105	4.983586492	70.77964068	6.54711E-05	3.026726209	99755.17097	4.987114248	74.42805048
15 to 19	0.000307301	2.74198	99635.70843	4.978331003	65.82126745	0.000203891	2.663137112	99722.51981	4.983751411	69.45142848
20 to 24	0.000480704	2.637486418	99482.72381	4.968493624	60.91826878	0.00022543	2.55163672	99620.90544	4.9782976	64.51955187
25 to 29	0.000626875	2.603021235	99243.88652	4.954749304	56.05852225	0.000260316	2.592931091	99508.67998	4.972318353	59.58943736
30 to 34	0.000794862	2.569222544	98933.28607	4.937125159	51.22634143	0.000342332	2.61744428	99379.24256	4.964912639	54.66367094
35 to 39	0.000897011	2.571638958	98540.85369	4.916333634	46.42011018	0.000454588	2.655594046	99209.27808	4.955182969	49.75283338
40 to 44	0.001112124	2.664558223	98099.85427	4.892286012	41.61722138	0.000684408	2.686983992	98984.02178	4.941378635	44.8600078
45 to 49	0.001792465	2.713968146	97555.77333	4.857882933	36.83445842	0.001074649	2.756717564	98645.83065	4.920429647	40.00458574
50 to 54	0.002995925	2.698257073	96685.01946	4.801143173	32.14174007	0.002026818	2.720900724	98117.05864	4.883295505	35.20531418
55 to 59	0.004753863	2.707434169	95246.64395	4.710989618	27.58636796	0.003274268	2.700673175	97127.30834	4.820077121	30.5363248
60 to 64	0.008033756	2.699239994	93007.12805	4.565961373	23.18540709	0.005317903	2.701678499	95549.09747	4.719769265	25.99607488
65 to 69	0.013261488	2.687302816	89338.99978	4.334028826	19.02651342	0.00879098	2.685459506	93039.19857	4.559195274	21.62445658
70 to 74	0.022238837	2.662323004	83591.55855	3.973037356	15.14990567	0.014095703	2.701963271	89031.29324	4.311895711	17.47699394
75 to 79	0.036699171	2.658003914	74756.29384	3.441992321	11.62575539	0.025254453	2.70138605	82953.5645	3.920124023	13.5594693
80 to 84	0.069127716	2.598292717	62125.15029	2.664000112	8.449061413	0.047617048	2.657126786	73054.01086	3.286123871	10.03081718
85 to 89	0.126287356	2.445415196	43710.90094	1.652481195	5.913928039	0.094686406	2.559750951	57407.65543	2.331679535	7.040530711
90 to 94	0.221322681	2.201019611	22843.86289	0.705305034	4.082533172	0.177027842	2.314812249	35331.90551	1.197442803	4.840347284
95 plus	0.318250642	3.142195878	7234.925676	0.227346166	3.142195878	0.275660461	3.627703101	14135.78246	0.512840068	3.627703101

Table 15: Norway 2100 life table, by age and sex. mx=mortality rate, ax=mean person-years lived in an age interval among those who die in that age interval, lx=number of persons left alive at age x, nLx=person-years lived between age x and x+n, ex=life expectancy at age x.

Age Group	Male					Female				
	mx	ax	lx	nLx	ex	mx	ax	lx	nLx	ex
Early Neonatal	0.018420633	0.009588477	100000	0.019174695	84.69171935	0.013130989	0.009588639	100000	0.019175668	86.92074943
Late Neonatal	0.002144271	0.028766532	99964.68039	0.057510378	84.70244505	0.001776389	0.028766633	99974.8212	0.057516821	86.92343636
Post Neonatal	0.000196349	0.461629887	99952.34919	0.922764074	84.65535448	0.000183341	0.461630811	99964.60429	0.922882755	86.87477217
1 to 4	3.01814E-05	1.999959757	99934.23143	3.997127977	83.74733304	2.85625E-05	1.999961918	99947.68458	3.997679016	85.96611661
5 to 9	1.68494E-05	2.499964909	99922.1677	4.995897955	79.75720448	1.75494E-05	2.499963463	99936.26645	4.996594119	81.97570986
10 to 14	5.72987E-05	3.259420086	99913.75092	4.995114401	74.76369482	4.33572E-05	3.134568477	99927.49881	4.995925653	76.98267118
15 to 19	0.000182528	2.844803146	99885.14056	4.992248869	69.78418917	0.000139982	2.757456812	99905.84296	4.993662647	71.99871251
20 to 24	0.000362223	2.733759054	99794.06141	4.985494406	64.84479142	0.000189373	2.606482256	99835.97541	4.989509664	67.04701141
25 to 29	0.000507978	2.625117712	99613.66998	4.974637399	59.95655342	0.000213183	2.584756916	99741.53318	4.984475754	62.10788569
30 to 34	0.000659442	2.531175226	99361.26037	4.960007111	55.10161674	0.000249472	2.581435182	99635.34541	4.978746675	57.17117579
35 to 39	0.00064826	2.521817756	99034.71795	4.943800467	50.27432532	0.000305809	2.603789837	99511.20379	4.971903381	52.23909193
40 to 44	0.000733722	2.640782777	98715.1204	4.927142327	45.42830822	0.000412881	2.666422026	99359.2566	4.963159139	47.3146942
45 to 49	0.00108735	2.699842606	98354.56651	4.905416349	40.58475537	0.000668463	2.750918482	99154.48718	4.950247555	42.40646733
50 to 54	0.001853063	2.674307079	97822.17605	4.870150719	35.79051134	0.001323772	2.692654513	98823.88517	4.926094661	37.53838219
55 to 59	0.002885838	2.666718464	96921.39084	4.813626415	31.09725262	0.00204009	2.688230204	98172.66401	4.885495532	32.76774394
60 to 64	0.004411551	2.677237773	95536.46717	4.728426408	26.50719322	0.003369574	2.694184152	97177.80976	4.821361178	28.07346794
65 to 69	0.007317855	2.676361996	93455.72364	4.594759115	22.03536891	0.005719826	2.669557731	95556.12573	4.714881836	23.50190966
70 to 74	0.011865905	2.695103786	90106.43269	4.385764832	17.75139064	0.008964848	2.732453317	92866.92423	4.550592897	19.10182415
75 to 79	0.022001578	2.72929029	84922.60705	4.045085507	13.66694985	0.017862751	2.75256646	88810.46804	4.269328588	14.84407394
80 to 84	0.047415583	2.663618825	76090.87289	3.428320698	9.932750005	0.038878584	2.68942461	81239.76201	3.73055441	10.96567138
85 to 89	0.096198106	2.538150916	60003.76195	2.432053279	6.879113681	0.08066312	2.616204102	66930.71483	2.816114914	7.713784412
90 to 94	0.188107119	2.244721147	36932.74193	1.221828491	4.614067547	0.158796181	2.334444594	44724.13641	1.583133485	5.261671757
95 plus	0.290640839	3.446499204	14244.64291	0.49536748	3.446499204	0.258227418	3.894068686	20205.59784	0.801713088	3.894068686

Table 15: Portugal 2017 life table, by age and sex. mx=mortality rate, ax=mean person-years lived in an age interval among those who die in that age interval, lx=number of persons left alive at age x, nLx=person-years lived between age x and x+n, ex=life expectancy at age x.

Age Group	Male					Female				
	mx	ax	lx	nLx	ex	mx	ax	lx	nLx	ex
Early Neonatal	0.083227217	0.00958649	100000	0.019162785	78.5696411	0.059070906	0.009587231	100000	0.019167223	84.41416042
Late Neonatal	0.008825684	0.028764689	99840.51628	0.057427908	78.67594775	0.006565361	0.028765312	99886.77867	0.057458253	84.49065228
Post Neonatal	0.001149592	0.46156217	99789.83428	0.920858472	78.65835739	0.000849715	0.461583473	99849.05617	0.921532503	84.46502744
1 to 4	0.000200037	1.999733285	99683.97851	3.985764401	77.8181103	0.000145878	1.999805496	99770.7546	3.989666091	83.60766821
5 to 9	6.39474E-05	2.499866776	99604.25229	4.979416543	73.87880003	6.43534E-05	2.49986593	99712.55567	4.984825775	79.65530114
10 to 14	0.000104974	2.933726357	99572.41114	4.977540767	68.90162613	7.3712E-05	2.765064433	99680.47707	4.983202938	74.68013122
15 to 19	0.000282629	2.78686775	99520.16068	4.972897568	63.93625885	0.000158268	2.61647975	99643.74519	4.980308586	69.70663902
20 to 24	0.000495248	2.628042732	99379.61309	4.963150387	59.0227223	0.000162436	2.508024241	99564.92328	4.976231905	64.75974243
25 to 29	0.000589138	2.527715053	99133.81549	4.949481798	54.16252038	0.000164828	2.641998768	99484.09171	4.972272075	59.8103119
30 to 34	0.000576602	2.632736054	98842.22527	4.935374825	49.31481332	0.000275222	2.804437252	99402.13519	4.967105464	54.85743561
35 to 39	0.000961831	2.775159618	98557.65367	4.917360269	44.4495696	0.000568495	2.730451295	99265.43038	4.956876026	49.92910218
40 to 44	0.001862024	2.734724135	98084.69481	4.883636225	39.65046456	0.000907878	2.691178507	98983.63803	4.938829365	45.06342703
45 to 49	0.00310397	2.724792338	97175.37597	4.824697247	34.99579621	0.001411439	2.677360482	98535.26185	4.910664776	40.2561771
50 to 54	0.005343955	2.677302492	95677.87855	4.725245933	30.50071913	0.002132988	2.66005351	97842.173	4.867813819	35.52229208
55 to 59	0.007976061	2.636535598	93152.93543	4.571477915	26.25464571	0.003102199	2.654883892	96803.92231	4.805239857	30.87463729
60 to 64	0.011275004	2.619543945	89507.15805	4.35839864	22.21638731	0.004551632	2.670707099	95313.34378	4.715675892	26.31581398
65 to 69	0.015861217	2.62815563	84593.93732	4.07637879	18.35432285	0.007088649	2.714943602	93167.15467	4.584115364	21.86031895
70 to 74	0.024044741	2.653697809	78129.95705	3.697945957	14.65519152	0.012579935	2.740151312	89918.12959	4.371652765	17.55185867
75 to 79	0.041287389	2.640627988	69241.64924	3.154910836	11.19566503	0.023946325	2.740008136	84419.97687	4.004360919	13.51619582
80 to 84	0.074208771	2.576942579	56222.88504	2.382959276	8.176861139	0.049272706	2.675724304	74834.90194	3.357452557	9.896138518
85 to 89	0.132712344	2.426894033	38551.78741	1.43719981	5.744818991	0.097218774	2.550607898	58302.05367	2.354801015	6.94381126
90 to 94	0.227932124	2.18925134	19492.56826	0.594202541	3.99141514	0.180060038	2.309661698	35426.92749	1.193609736	4.781976679
95 plus	0.323629463	3.090098877	5956.969933	0.18415221	3.090098877	0.278502493	3.591065882	13951.34882	0.501297516	3.591065882

Table 15: Portugal 2100 life table, by age and sex. mx=mortality rate, ax=mean person-years lived in an age interval among those who die in that age interval, lx=number of persons left alive at age x, nLx=person-years lived between age x and x+n, ex=life expectancy at age x.

Age Group	Male					Female				
	mx	ax	lx	nLx	ex	mx	ax	lx	nLx	ex
Early Neonatal	0.016589436	0.009588533	100000	0.019175032	84.16288723	0.012487101	0.009588658	100000	0.019175786	88.05228227
Late Neonatal	0.002001051	0.028766571	99968.19344	0.057512636	84.17049762	0.001741766	0.028766643	99976.057	0.057517589	88.05418505
Post Neonatal	0.000380674	0.461616793	99956.68562	0.922725597	84.12264994	0.000317217	0.461621301	99966.03931	0.922838971	88.00547365
1 to 4	0.000197051	1.999737265	99921.56206	3.995287795	83.22877628	0.000179664	1.999760447	99936.76617	3.996034643	87.10782914
5 to 9	6.16417E-05	2.49987158	99842.83889	4.991372728	79.29282122	6.6652E-05	2.499861142	99864.97533	4.992416869	83.16900918
10 to 14	5.47671E-05	2.7693542	99812.07198	4.989982543	74.31648896	5.13269E-05	2.63358627	99831.70164	4.990964802	78.19589365
15 to 19	0.000121661	2.802349369	99784.74469	4.987893693	69.33609078	8.59911E-05	2.550330305	99806.08831	4.989263992	73.21530537
20 to 24	0.00022223	2.657924	99724.06572	4.983601516	64.37649219	8.2225E-05	2.49763745	99763.18933	4.987132306	68.24561822
25 to 29	0.000282725	2.547177223	99613.33063	4.97721624	59.44492477	8.39433E-05	2.691780962	99722.19174	4.985128094	63.27259424
30 to 34	0.00028729	2.644199917	99472.64822	4.970260077	54.52516923	0.000144295	2.78976049	99680.35887	4.982422464	58.29795295
35 to 39	0.000473416	2.787339266	99329.92075	4.961282689	49.59947851	0.000278384	2.742250937	99608.48369	4.977273987	53.33785466
40 to 44	0.000925747	2.74690818	99095.19422	4.944405449	44.70998279	0.000446679	2.747614482	99469.97877	4.968494671	48.40788817
45 to 49	0.001533913	2.760212727	98637.86608	4.914960877	39.903689	0.000805197	2.735377672	99248.1391	4.953340368	43.50929523
50 to 54	0.002815336	2.698235153	97884.81877	4.86270794	35.18782736	0.001325147	2.687051467	98849.60659	4.92738273	38.67217097
55 to 59	0.004240101	2.637573734	96517.907	4.778059209	30.64516523	0.002007197	2.668460807	98197.33866	4.886964488	33.90905653
60 to 64	0.005725941	2.629672996	94496.70012	4.661687191	26.24041788	0.00291897	2.656839058	97218.43884	4.827956021	29.22038064
65 to 69	0.008136211	2.664108129	91835.13177	4.506352221	21.92070804	0.00428763	2.710313624	95812.54668	4.744136001	24.60681937
70 to 74	0.012843842	2.712205265	88184.99009	4.283954807	17.71292227	0.007413762	2.769074879	93786.58301	4.613276184	20.07487979
75 to 79	0.023578891	2.723304112	82713.88681	3.926520882	13.69994883	0.014611529	2.809955388	90384.96655	4.379754457	15.71966115
80 to 84	0.046111605	2.648165915	73546.63923	3.322162817	10.06266932	0.0324411	2.735789367	84045.02927	3.91844564	11.68366931
85 to 89	0.0940601	2.545342665	58459.0249	2.383626725	6.971111932	0.070326249	2.658188033	71521.22315	3.079388757	8.234129493
90 to 94	0.185434212	2.245672295	36497.98075	1.215939112	4.666853026	0.145129145	2.346161776	50360.16909	1.830765903	5.591924925
95 plus	0.288355248	3.476790322	14373.5269	0.506199455	3.476790322	0.245131462	4.103236997	24453.83695	1.022207544	4.103236997

Table 15: Spain 2017 life table, by age and sex. mx=mortality rate, ax=mean person-years lived in an age interval among those who die in that age interval, lx=number of persons left alive at age x, nLx=person-years lived between age x and x+n, ex=life expectancy at age x.

Age Group	Male					Female				
	mx	ax	lx	nLx	ex	mx	ax	lx	nLx	ex
Early Neonatal	0.068669693	0.009586936	100000	0.019165459	80.19583995	0.054679514	0.009587365	100000	0.01916803	85.95406871
Late Neonatal	0.011530513	0.028763943	99868.39249	0.057439473	80.28232956	0.009506366	0.028764501	99895.19063	0.057458231	86.02506196
Post Neonatal	0.001034892	0.461570319	99802.16321	0.92102099	80.27805211	0.000894171	0.461580315	99840.56937	0.921435263	86.01457511
1 to 4	0.00014662	1.999804507	99706.84996	3.987104738	79.43106509	0.000126936	1.999830752	99758.17848	3.98931429	85.16194697
5 to 9	8.22204E-05	2.499828707	99648.39268	4.981395652	75.47649011	6.20237E-05	2.499870784	99707.54032	4.984604073	81.20418282
10 to 14	9.00059E-05	2.833250892	99607.43622	4.979400621	70.50649716	6.82306E-05	2.696477716	99676.6242	4.98304805	76.2285943
15 to 19	0.000226286	2.731523525	99562.61918	4.975576922	65.53695808	0.000126476	2.637931216	99642.62471	4.980643347	71.25368272
20 to 24	0.000342075	2.605988715	99450.02936	4.968432646	60.60805028	0.000152156	2.558219053	99579.63163	4.977132458	66.29708246
25 to 29	0.000401354	2.592760077	99280.07237	4.959212249	55.70732649	0.000169253	2.59803728	99503.90178	4.973173329	61.34558471
30 to 34	0.000522592	2.603244427	99081.03328	4.947854477	50.8140071	0.000232166	2.6940273	99419.72993	4.968326701	56.39531321
35 to 39	0.00066325	2.690883028	98822.46444	4.933567551	45.94012633	0.000386301	2.724853295	99304.3827	4.960859078	51.45767768
40 to 44	0.001136825	2.764066102	98495.2492	4.912276391	41.08377357	0.000651057	2.738083955	99112.74592	4.948350069	46.55188107
45 to 49	0.002123831	2.763965509	97936.81943	4.873696474	36.30221416	0.001136059	2.729490319	98790.58446	4.926820812	41.6947259
50 to 54	0.003893724	2.711124814	96901.75995	4.802290197	31.6603549	0.001919549	2.680818513	98230.87913	4.889776165	36.91669084
55 to 59	0.006255281	2.681323493	95031.97529	4.68367082	27.2297824	0.002843949	2.649968447	97292.29099	4.832319542	32.24688758
60 to 64	0.009817475	2.653542382	92102.44426	4.501436585	23.01040219	0.004057998	2.650098503	95918.0679	4.750605085	27.67081954
65 to 69	0.014595857	2.645013768	87683.70057	4.238518707	19.03604192	0.005960909	2.68818877	93990.40626	4.635645542	23.18384521
70 to 74	0.022547895	2.648665786	81498.34684	3.869802425	15.27982237	0.009917102	2.735221673	91227.4322	4.461188127	18.80442795
75 to 79	0.037101085	2.638995771	72775.19258	3.345795754	11.79368893	0.018619604	2.76533383	86803.97194	4.166858272	14.62308846
80 to 84	0.065182507	2.592083233	60367.14793	2.609076707	8.675466111	0.039449312	2.715137291	79047.54666	3.625687509	10.78638749
85 to 89	0.121034503	2.46089477	43370.11792	1.658988266	6.060306741	0.082226943	2.607136634	64750.33899	2.70546996	7.5685442
90 to 94	0.215815184	2.210253881	23302.66614	0.727414257	4.161745088	0.16155684	2.337154799	42515.99965	1.48661606	5.16393322
95 plus	0.313739292	3.187493589	7611.669141	0.242702588	3.187493589	0.261020218	3.831496339	18511.73193	0.709568721	3.831496339

Table 15: Spain 2100 life table, by age and sex. mx=mortality rate, ax=mean person-years lived in an age interval among those who die in that age interval, lx=number of persons left alive at age x, nLx=person-years lived between age x and x+n, ex=life expectancy at age x.

Age Group	Male					Female				
	mx	ax	lx	nLx	ex	mx	ax	lx	nLx	ex
Early Neonatal	0.018625434	0.00958847	100000	0.019174658	85.40331214	0.015993844	0.009588551	100000	0.019175141	88.59255071
Late Neonatal	0.003267391	0.028766222	99964.28807	0.057508295	85.41463738	0.003284083	0.028766217	99969.33271	0.057511169	88.60053912
Post Neonatal	0.000242658	0.461626598	99945.49852	0.922681105	85.37315488	0.000234562	0.461627173	99950.44633	0.922730231	88.55973041
1 to 4	4.11308E-05	1.999945159	99923.1099	3.996595626	84.46889387	3.86289E-05	1.999948495	99928.80337	3.996843347	87.65552504
5 to 9	2.87151E-05	2.499940177	99906.67191	4.994975009	80.48246539	2.80379E-05	2.49994159	99913.36447	4.995318077	83.66876055
10 to 14	4.06546E-05	3.011277758	99892.32888	4.994209018	75.49365596	3.8091E-05	2.821706264	99899.359	4.994546795	78.68013651
15 to 19	0.000125364	2.804920188	99872.02544	4.992195177	70.50838844	8.03931E-05	2.665774871	99880.33512	4.993075582	73.69459232
20 to 24	0.000193321	2.624729589	99809.44947	4.988171954	65.55078772	9.80225E-05	2.561371129	99840.19734	4.990807279	68.72315188
25 to 29	0.000231432	2.601500804	99713.03269	4.982880328	60.61153041	9.99709E-05	2.595320214	99791.2834	4.988358482	63.75558552
30 to 34	0.000300992	2.58424863	99597.73711	4.976263543	55.67858262	0.000137492	2.682570616	99741.42041	4.985476074	58.78615639
35 to 39	0.000348449	2.667582729	99448.00201	4.968352798	50.75842072	0.000214357	2.714652068	99672.88081	4.981201742	53.82469189
40 to 44	0.00057274	2.748873164	99274.95498	4.957330316	45.8421115	0.000356696	2.797063097	99566.11682	4.974385333	48.87936318
45 to 49	0.001009879	2.782640409	98991.16031	4.938475936	40.96539142	0.000713246	2.80775714	99388.71269	4.961624176	43.96139463
50 to 54	0.001933118	2.734352911	98492.6554	4.903158749	36.1581491	0.001364164	2.712822358	99034.94313	4.936352069	39.10790229
55 to 59	0.003227627	2.685158706	97545.25778	4.841091693	31.48176485	0.002130859	2.634301947	98361.80457	4.893419395	34.35572338
60 to 64	0.00489871	2.663859471	95984.0395	4.744936157	26.94815824	0.002760598	2.615297084	97320.01452	4.834184841	29.69330156
65 to 69	0.007341434	2.663024397	93662.61277	4.6042375	22.54746712	0.003709879	2.686577993	95987.39543	4.758582072	25.06730105
70 to 74	0.011239904	2.714045615	90289.53935	4.401701823	18.28674637	0.00624587	2.779903375	94225.80115	4.646912155	20.48274962
75 to 79	0.020852132	2.738543434	85356.48346	4.076469048	14.18276788	0.012622038	2.853921727	91332.14633	4.446266861	16.0396269
80 to 84	0.0415128	2.692476278	76911.99062	3.512025039	10.43364643	0.030495493	2.78005753	85748.08724	4.017433432	11.89228871
85 to 89	0.087379081	2.566694972	62480.782	2.582508385	7.215756809	0.067253868	2.670484392	73609.14194	3.18771643	8.381435419
90 to 94	0.177653265	2.252529863	40239.74597	1.358282131	4.804406476	0.141137771	2.350349089	52497.32149	1.919597853	5.684327769
95 plus	0.281805655	3.55563481	16439.25452	0.590087848	3.55563481	0.241318779	4.161662328	25875.27379	1.091032176	4.161662328

Table 15: Sweden 2017 life table, by age and sex. mx=mortality rate, ax=mean person-years lived in an age interval among those who die in that age interval, lx=number of persons left alive at age x, nLx=person-years lived between age x and x+n, ex=life expectancy at age x.

Age Group	Male					Female				
	mx	ax	lx	nLx	ex	mx	ax	lx	nLx	ex
Early Neonatal	0.063748686	0.009587087	100000	0.019166364	80.89447227	0.050995988	0.009587478	100000	0.019168707	84.28740242
Late Neonatal	0.006731315	0.028765266	99877.81759	0.057452824	80.97423977	0.00610919	0.028765438	99902.24756	0.057467905	84.35068808
Post Neonatal	0.000940622	0.461577015	99839.14474	0.92140236	80.94805955	0.000744398	0.461590955	99867.13955	0.921744199	84.322797
1 to 4	0.000122824	1.999836235	99752.47695	3.989119098	80.09470082	0.000105775	1.999858966	99798.52568	3.991096656	83.45716632
5 to 9	8.47086E-05	2.499823524	99703.48198	4.984118543	76.13307746	6.80312E-05	2.499858268	99756.31012	4.986967289	79.49163828
10 to 14	6.17434E-05	3.112723266	99661.26271	4.982482459	71.16427074	9.23022E-05	2.634380248	99722.38335	4.985030703	74.51783196
15 to 19	0.000266444	2.864253092	99630.4994	4.978691822	66.18528236	0.000127685	2.744434635	99676.37056	4.982383655	69.55101255
20 to 24	0.000528706	2.667450783	99497.84539	4.968764601	61.269692	0.000242443	2.615137504	99612.75323	4.977759604	64.59367275
25 to 29	0.000694026	2.571569696	99235.14504	4.953408824	56.42480326	0.00026216	2.576993142	99492.07144	4.971445674	59.66884003
30 to 34	0.00077157	2.541032257	98891.36782	4.935205181	51.6119856	0.000340175	2.594950338	99361.74046	4.964025871	54.74371336
35 to 39	0.00085207	2.57887225	98510.58597	4.915389258	46.80163355	0.000418447	2.66425069	99192.87778	4.954801121	49.83247268
40 to 44	0.001102626	2.658211407	98091.76719	4.89195709	41.99041381	0.00067244	2.722438375	98985.54764	4.941708983	44.93124595
45 to 49	0.001705266	2.684124029	97552.37924	4.858432638	37.20784542	0.001142462	2.696718182	98653.25183	4.91971689	40.07338678
50 to 54	0.002645242	2.709809352	96723.91448	4.807074848	32.50348296	0.001766563	2.731741288	98091.2035	4.884986314	35.28749923
55 to 59	0.004463511	2.734015061	95452.38498	4.724833241	27.90027516	0.003153104	2.723633375	97228.26438	4.826769635	30.57637298
60 to 64	0.007941587	2.705782242	93343.58674	4.58367273	23.46869471	0.005280485	2.700219201	95706.39966	4.72790806	26.01915499
65 to 69	0.01310774	2.663014016	89703.76199	4.351895942	19.31098928	0.008580877	2.687865071	93210.00434	4.569843689	21.64352933
70 to 74	0.020201025	2.67383482	84000.23822	4.011544935	15.44115082	0.013947612	2.696385778	89289.11895	4.325501954	17.47569259
75 to 79	0.035573604	2.668676407	75898.36376	3.504381571	11.80382828	0.024365365	2.717182352	83257.23241	3.943572111	13.54617245
80 to 84	0.066512372	2.602143937	63436.49512	2.735725042	8.598379669	0.048507416	2.666930842	73651.62553	3.308335807	9.958272513
85 to 89	0.122857678	2.455500672	45249.77326	1.723896384	6.008953123	0.096046999	2.554833928	57611.42108	2.332993763	6.988481076
90 to 94	0.21773381	2.207078198	24082.27378	0.74891935	4.133929461	0.178657882	2.31204828	35217.13722	1.189828648	4.80892994
95 plus	0.315312787	3.171586272	7783.412842	0.246937164	3.171586272	0.27718854	3.607982561	13972.35412	0.504345184	3.607982561

Table 15: Sweden 2100 life table, by age and sex. mx=mortality rate, ax=mean person-years lived in an age interval among those who die in that age interval, lx=number of persons left alive at age x, nLx=person-years lived between age x and x+n, ex=life expectancy at age x.

Age Group	Male					Female				
	mx	ax	lx	nLx	ex	mx	ax	lx	nLx	ex
Early Neonatal	0.019079283	0.009588456	100000	0.019174574	84.72666846	0.016737147	0.009588528	100000	0.019175005	86.83629381
Late Neonatal	0.001879361	0.028766605	99963.42004	0.057510092	84.73848531	0.002077439	0.02876655	99967.90936	0.057512347	86.84497622
Post Neonatal	0.000253626	0.461625819	99952.61261	0.922742115	84.69011012	0.000200346	0.461629604	99955.96217	0.92279573	86.7978167
1 to 4	3.66659E-05	1.999951112	99929.21184	3.996875392	83.78654969	3.52999E-05	1.999952933	99937.4757	3.99721684	85.89050563
5 to 9	3.36225E-05	2.499929964	99914.55824	4.995308072	79.79854764	4.17071E-05	2.499913075	99923.36676	4.995647627	81.90234446
10 to 14	3.06448E-05	3.364609909	99897.76617	4.99462218	74.81151926	6.22741E-05	2.899813065	99902.54211	4.994386571	76.91884052
15 to 19	0.000193494	2.913162966	99882.4632	4.992026639	69.82246581	8.70631E-05	2.820881183	99871.48509	4.992585096	71.94198596
20 to 24	0.000433652	2.669900566	99785.90039	4.984231287	64.88695459	0.000180098	2.610960642	99828.03301	4.989252941	66.97205518
25 to 29	0.00061852	2.564306635	99569.86265	4.970993573	60.02139818	0.000194993	2.563072014	99738.20436	4.98453678	62.02993714
30 to 34	0.000672984	2.509488513	99262.69753	4.954819599	55.19851389	0.000240878	2.543784422	99641.07783	4.979098945	57.08775109
35 to 39	0.000631204	2.527802035	98929.74747	4.938760072	50.37534424	0.00024182	2.631829901	99521.23456	4.973188342	52.15327408
40 to 44	0.000732556	2.612865092	98618.48993	4.922302446	45.52608635	0.000387044	2.731077353	99401.09201	4.965635749	47.21285253
45 to 49	0.001106987	2.6585112	98258.38895	4.900180075	40.68311135	0.000670473	2.68973788	99209.08472	4.952762468	42.29855126
50 to 54	0.001741505	2.673104346	97716.85484	4.866106855	35.89337652	0.001071754	2.720589122	98877.29723	4.931774827	37.43078927
55 to 59	0.002917241	2.67246766	96870.66474	4.81083144	31.18272034	0.001938556	2.717827911	98349.32152	4.895713492	32.61601017
60 to 64	0.004735039	2.666192999	95470.14147	4.721335573	26.59868298	0.003349952	2.712042833	97401.48562	4.832951013	27.90544954
65 to 69	0.007797409	2.637887757	93239.32904	4.577786325	22.16845003	0.00591183	2.691422134	95784.84965	4.72466481	23.32813375
70 to 74	0.01188869	2.66068711	89679.57801	4.362984632	17.93993269	0.009826724	2.711065333	92999.99349	4.547724035	18.94138037
75 to 79	0.021394895	2.695067126	84513.76166	4.028015855	13.86951209	0.018574967	2.730394606	88550.83814	4.249032357	14.75148538
80 to 84	0.045132499	2.640271329	75951.37605	3.435314835	10.1245993	0.039324637	2.695136667	80708.25547	3.702973419	10.91362856
85 to 89	0.092681504	2.54952256	60620.87316	2.476872293	7.012663032	0.081412999	2.613067108	66332.88899	2.785802569	7.675232156
90 to 94	0.183947986	2.247921875	38016.89448	1.268317096	4.689490092	0.159797134	2.33353561	44136.10421	1.558849659	5.237354697
95 plus	0.287127104	3.489742334	15022.65963	0.529557444	3.489742334	0.25919129	3.878670328	19819.31134	0.783013189	3.878670328

Table 15: Switzerland 2017 life table, by age and sex. mx=mortality rate, ax=mean person-years lived in an age interval among those who die in that age interval, lx=number of persons left alive at age x, nLx=person-years lived between age x and x+n, ex=life expectancy at age x.

Age Group	Male					Female				
	mx	ax	lx	nLx	ex	mx	ax	lx	nLx	ex
Early Neonatal	0.119678216	0.009585373	100000	0.019156091	82.17499434	0.109044053	0.009585699	100000	0.019158043	85.87356696
Late Neonatal	0.006333335	0.028765376	99770.74889	0.057391892	82.3446121	0.005211673	0.028765686	99791.09622	0.057405449	86.03413616
Post Neonatal	0.000814135	0.461586001	99734.40258	0.920489462	82.31707765	0.000681685	0.46159541	99761.17938	0.92079288	86.00239434
1 to 4	0.000136293	1.999818276	99659.46669	3.985292289	81.45534262	0.000117613	1.999843183	99698.41262	3.986998616	85.13296229
5 to 9	6.61692E-05	2.499862148	99605.15276	4.979433908	77.49867186	4.82337E-05	2.499899513	99651.52186	4.981975331	81.17208165
10 to 14	7.74104E-05	3.054512571	99572.20537	4.977860426	72.52348924	8.0461E-05	2.734828724	99627.49235	4.980466917	76.19105725
15 to 19	0.000272275	2.754279263	99533.67233	4.973642517	67.55038203	0.00013904	2.595352836	99587.41932	4.977706781	71.22061292
20 to 24	0.00041055	2.578033661	99398.25309	4.964975771	62.63864324	0.000144309	2.586252465	99518.20953	4.974177894	66.26833046
25 to 29	0.00042739	2.52341089	99194.41701	4.954476693	57.76203778	0.000199096	2.63830428	99446.42789	4.969984522	61.31428812
30 to 34	0.000460426	2.573278	98982.66916	4.943609979	52.8801827	0.000276948	2.649890149	99347.47813	4.964143075	56.37271644
35 to 39	0.000591889	2.661338849	98755.05466	4.930927439	47.99610649	0.000399353	2.647781555	99209.99808	4.955844584	51.44714497
40 to 44	0.000923537	2.692389441	98463.2029	4.912690702	43.13044667	0.000561994	2.725365295	99012.08681	4.944283772	46.54465976
45 to 49	0.001455465	2.713561597	98009.5081	4.884221969	38.31758342	0.001011899	2.709689683	98734.2251	4.925296586	41.66794182
50 to 54	0.002443952	2.723963332	97298.65129	4.838021758	33.57762139	0.001592571	2.708601576	98235.84616	4.893933638	36.86553037
55 to 59	0.004162855	2.716241938	96116.32403	4.760559968	28.95702847	0.002640108	2.695182824	97456.47821	4.843353376	32.13860416
60 to 64	0.006992387	2.69202845	94134.72744	4.631991382	24.50923825	0.004150985	2.696337844	96177.84539	4.763346235	27.52991643
65 to 69	0.011214356	2.667621034	90896.23303	4.428985416	20.28632494	0.006775605	2.691313015	94200.74043	4.637502662	23.05096305
70 to 74	0.017525016	2.676825194	85930.29868	4.128472416	16.30424773	0.010950418	2.699530479	91058.94456	4.441092281	18.75319865
75 to 79	0.03020344	2.683504983	78697.18535	3.677648318	12.55650788	0.018868302	2.744154929	86196.78173	4.133930438	14.65846528
80 to 84	0.057158343	2.632993029	67594.29989	2.977156622	9.178157639	0.038893583	2.713647724	78399.40628	3.599984938	10.84316553
85 to 89	0.110338965	2.493177724	50587.81698	1.981653659	6.379037809	0.081336364	2.610663709	64404.88934	2.696531879	7.609629195
90 to 94	0.204332598	2.227837644	28737.47771	0.917490544	4.335347505	0.160415874	2.338532384	42486.61168	1.489033628	5.189448599
95 plus	0.304263748	3.286808715	10001.02527	0.328845119	3.286808715	0.259932601	3.847607573	18615.88621	0.716623439	3.847607573

Table 15: Switzerland 2100 life table, by age and sex. mx=mortality rate, ax=mean person-years lived in an age interval among those who die in that age interval, lx=number of persons left alive at age x, nLx=person-years lived between age x and x+n, ex=life expectancy at age x.

Age Group	Male					Female				
	mx	ax	lx	nLx	ex	mx	ax	lx	nLx	ex
Early Neonatal	0.045560217	0.009587645	100000	0.019169707	86.69013025	0.042886505	0.009587727	100000	0.019170198	88.60587803
Late Neonatal	0.001835846	0.028766617	99912.67395	0.057480969	86.7467592	0.001684829	0.028766659	99917.79523	0.057484165	88.65958781
Post Neonatal	0.000186253	0.461630605	99902.12235	0.92230468	86.6983938	0.000159984	0.461632471	99908.11076	0.922371147	88.61064381
1 to 4	3.3824E-05	1.999954902	99884.94545	3.99512756	85.78993425	3.13212E-05	1.999958238	99893.35488	3.995483909	87.70037854
5 to 9	2.14811E-05	2.499955256	99871.43294	4.993303511	81.80127283	2.22342E-05	2.499953679	99880.84087	4.993764465	83.711116
10 to 14	3.25101E-05	3.352736709	99860.70805	4.992750065	76.80975285	4.72275E-05	2.921319605	99869.73808	4.992962388	78.72014203
15 to 19	0.0001537	2.728562905	99844.47797	4.990471872	71.82168051	8.72333E-05	2.585790452	99846.15951	4.991258189	73.73808365
20 to 24	0.000213413	2.58213528	99767.7869	4.985814171	66.87451489	8.63643E-05	2.62133723	99802.62352	4.98909238	68.7691044
25 to 29	0.000244554	2.559097922	99661.42962	4.980092469	61.94269212	0.000124518	2.692581576	99759.54444	4.986520287	63.79765603
30 to 34	0.000282698	2.592292938	99539.73478	4.973589801	57.01483593	0.000179545	2.652343693	99697.46876	4.982753555	58.83569107
35 to 39	0.000369636	2.629214511	99399.27935	4.965591409	52.09130305	0.000237881	2.609514998	99608.02999	4.977569448	53.88606142
40 to 44	0.000513017	2.664313949	99215.98492	4.954828898	47.18216596	0.000305535	2.741432253	99489.65294	4.971027724	48.94694635
45 to 49	0.000776615	2.715857244	98962.19118	4.93931119	42.29574079	0.000574513	2.720758872	99337.83751	4.960369183	44.01726815
50 to 54	0.001358325	2.726593082	98579.26622	4.913759449	37.4486495	0.000898377	2.733776746	99053.0054	4.942585834	39.1353854
55 to 59	0.002373223	2.699223602	97913.15456	4.869015585	32.6834256	0.00160441	2.726740716	98609.27398	4.912444386	34.29820567
60 to 64	0.003787145	2.677384276	96760.57351	4.79585677	28.03828461	0.002574809	2.718457476	97822.16475	4.862444278	29.55059982
65 to 69	0.005967114	2.660127005	94949.49673	4.682225143	23.51885614	0.004284003	2.719450736	96572.15569	4.781755724	24.89598622
70 to 74	0.009137822	2.706420494	92165.65174	4.513942469	19.14489766	0.007152205	2.737784882	94528.75876	4.651125982	20.3733406
75 to 79	0.017002056	2.743714091	88060.45801	4.24120183	14.90687199	0.013013645	2.822446646	91213.77307	4.435079265	16.01095388
80 to 84	0.03588528	2.709264991	80898.09959	3.740597799	10.97800967	0.030393134	2.779747559	85471.42819	4.005084807	11.89235015
85 to 89	0.078526369	2.596039307	67636.55159	2.852042474	7.59207991	0.067130673	2.670765995	73395.42804	3.178520586	8.380019315
90 to 94	0.166641613	2.255823973	45620.75842	1.573430999	5.021602575	0.141049691	2.351166413	52339.34605	1.913057072	5.682420617
95 plus	0.272416037	3.680278418	19831.97716	0.738221428	3.680278418	0.24124782	4.160359666	25761.1656	1.083953056	4.160359666

Table 15: United Kingdom 2017 life table, by age and sex. mx=mortality rate, ax=mean person-years lived in an age interval among those who die in that age interval, lx=number of persons left alive at age x, nLx=person-years lived between age x and x+n, ex=life expectancy at age x.

Age Group	Male					Female				
	mx	ax	lx	nLx	ex	mx	ax	lx	nLx	ex
Early Neonatal	0.118261035	0.009585416	100000	0.01915635	79.19856827	0.096056859	0.009586097	100000	0.019160428	82.75570997
Late Neonatal	0.012408651	0.0287637	99773.45512	0.05738342	79.35919594	0.010022019	0.028764359	99815.95099	0.057411802	82.88910603
Post Neonatal	0.001405694	0.461543977	99702.2501	0.919941477	79.35831765	0.001109094	0.461565047	99758.41281	0.920585701	82.87936358
1 to 4	0.000170715	1.999772381	99572.9346	3.981557811	78.53749339	0.00015349	1.999795347	99656.31126	3.985029004	82.04051597
5 to 9	8.00218E-05	2.499833288	99504.96369	4.974252998	74.58977562	5.9866E-05	2.499875279	99595.14519	4.979012039	78.08967277
10 to 14	9.68111E-05	2.976331189	99465.15888	4.9722838	69.61862522	7.67948E-05	2.7751735	99565.33785	4.977416476	73.11230244
15 to 19	0.000301683	2.795768212	99417.02167	4.967547767	64.650893	0.000161413	2.69130869	99527.1139	4.974501939	68.13931574
20 to 24	0.000526297	2.62856133	99267.15922	4.957171007	59.74427417	0.000225284	2.612639929	99446.81908	4.969668097	63.1921591
25 to 29	0.000628908	2.599147041	99006.26465	4.942849951	54.89478036	0.00028373	2.668227584	99334.86034	4.963459219	58.26043679
30 to 34	0.000830113	2.622434309	98695.40503	4.92504994	50.0594936	0.000455503	2.675910487	99194.03192	4.954456672	53.33936189
35 to 39	0.001124404	2.674503526	98286.57027	4.901512078	45.25681178	0.000670718	2.691184771	98968.35514	4.940766664	48.45488831
40 to 44	0.001788737	2.663344828	97735.44283	4.866432132	40.49692943	0.001076887	2.688428825	98636.96906	4.919602082	43.6086368
45 to 49	0.002562423	2.653795802	96864.96759	4.814304979	35.83691585	0.001658718	2.684147093	98107.18422	4.886588132	38.82960598
50 to 54	0.003757574	2.676428116	95631.34188	4.740180715	31.26496615	0.002576937	2.692152216	97296.63791	4.836070961	34.13071867
55 to 59	0.005923377	2.687832546	93850.19029	4.629110196	26.80753174	0.004120114	2.682201857	96050.41514	4.757092567	29.5386194
60 to 64	0.009543621	2.679050671	91108.20761	4.456694024	22.53342718	0.006385794	2.680126707	94090.44417	4.63584596	25.09805143
65 to 69	0.015259295	2.651322021	86854.93647	4.192492718	18.50568296	0.010153329	2.671532258	91130.10073	4.451270076	20.82628529
70 to 74	0.023328317	2.665722814	80457.55115	3.815128379	14.76629389	0.015997204	2.697573117	86610.60801	4.176694164	16.77362205
75 to 79	0.041345351	2.638707005	71557.62695	3.259652506	11.27128737	0.028985546	2.686488046	79929.13301	3.745305917	12.95026347
80 to 84	0.072326193	2.578002866	58080.75698	2.471165752	8.274371065	0.053467756	2.642693551	69073.33357	3.067098087	9.563347769
85 to 89	0.130312807	2.433743932	40208.23701	1.506596829	5.806441507	0.103288624	2.52899305	52674.6172	2.09822257	6.717908182
90 to 94	0.225483164	2.193711518	20575.90411	0.630097818	4.02456392	0.187264848	2.297029916	31002.94178	1.029206488	4.646102596
95 plus	0.321642131	3.109051072	6368.584326	0.198005627	3.109051072	0.285238117	3.505855158	11730.00763	0.411244929	3.505855158

Table 15: United Kingdom 2100 life table, by age and sex. mx=mortality rate, ax=mean person-years lived in an age interval among those who die in that age interval, lx=number of persons left alive at age x, nLx=person-years lived between age x and x+n, ex=life expectancy at age x.

Age Group	Male					Female				
	mx	ax	lx	nLx	ex	mx	ax	lx	nLx	ex
Early Neonatal	0.02985644	0.009588126	100000	0.019172593	83.66108436	0.026209895	0.009588238	100000	0.019173263	86.09799199
Late Neonatal	0.003167025	0.02876625	99942.75864	0.057496075	83.68982248	0.003000418	0.028766296	99949.748	0.057500371	86.12208793
Post Neonatal	0.000342979	0.461619471	99924.54984	0.922444992	83.64753884	0.000281524	0.461623837	99932.49578	0.922544514	86.07941498
1 to 4	4.91064E-05	1.999934525	99892.91335	3.995324138	82.75059617	4.63142E-05	1.999938248	99906.52487	3.99589086	85.17838323
5 to 9	2.8419E-05	2.499940794	99873.29425	4.993309943	78.76645836	2.71303E-05	2.499943478	99888.01873	4.99406221	81.19379095
10 to 14	4.2046E-05	3.298430804	99859.10384	4.992588622	73.77728684	4.35572E-05	2.915820375	99874.47003	4.993264542	76.20446065
15 to 19	0.000181015	2.869643858	99838.11232	4.989971078	68.79210071	0.000108659	2.750183902	99852.72131	4.991403973	71.22041922
20 to 24	0.00035445	2.673901217	99747.79249	4.983275521	63.85163884	0.00016267	2.641345596	99798.48871	4.98800431	66.25759783
25 to 29	0.000472487	2.655713831	99571.19718	4.973039975	58.95983515	0.000213115	2.67930677	99717.35336	4.98339918	61.30932706
30 to 34	0.000697975	2.631059323	99336.32356	4.958590126	54.09257668	0.000342887	2.649783445	99611.15666	4.976539834	56.37176372
35 to 39	0.000885801	2.64244261	98990.44503	4.939191215	49.27194782	0.000453083	2.659361935	99440.53537	4.966757992	51.46376759
40 to 44	0.001291588	2.624909346	98553.20762	4.912597777	44.47843461	0.00069015	2.681170131	99215.52249	4.952851826	46.57422963
45 to 49	0.001675811	2.620874724	97919.16044	4.876530796	39.74880389	0.001059831	2.686120191	98873.7592	4.931588882	41.72541413
50 to 54	0.002292635	2.671279014	97102.80414	4.829345222	35.05964004	0.001642473	2.69082198	98351.25029	4.898989801	36.93183741
55 to 59	0.003587319	2.67258045	95996.96773	4.760149748	30.43066682	0.002599664	2.673951762	97546.93769	4.848058131	32.21277184
60 to 64	0.00542123	2.675583346	94291.37547	4.655993648	25.92999708	0.003899745	2.672896784	96287.58028	4.771136174	27.59676891
65 to 69	0.008513498	2.666048459	91772.3922	4.499509611	21.56345772	0.006024166	2.681671141	94429.58038	4.656597277	23.08359353
70 to 74	0.013140104	2.732563875	87953.24258	4.271016634	17.37722136	0.009629562	2.759778512	91630.78741	4.485097686	18.70180629
75 to 79	0.025916835	2.71625713	82374.45162	3.890499149	13.3591674	0.019525931	2.776987109	87330.50795	4.185851047	14.47920481
80 to 84	0.049673901	2.662327752	72393.51455	3.249737068	9.81419592	0.041749138	2.725977242	79220.36992	3.621762658	10.66641171
85 to 89	0.099145073	2.52959005	56579.05741	2.284867474	6.804855594	0.085367852	2.597544962	64322.1224	2.678440577	7.491606558
90 to 94	0.191090143	2.238554127	34566.61973	1.142105271	4.575053757	0.16487078	2.32785661	41971.75586	1.467906104	5.12247859
95 plus	0.293065466	3.424221706	13313.77416	0.464327683	3.424221706	0.264021489	3.806086282	18347.77817	0.711170777	3.806086282

Table 15: Latin America and Caribbean 2017 life table, by age and sex. mx=mortality rate, ax=mean person-years lived in an age interval among those who die in that age interval, lx=number of persons left alive at age x, nLx=person-years lived between age x and x+n, ex=life expectancy at age x.

Age Group	Male					Female				
	mx	ax	lx	nLx	ex	mx	ax	lx	nLx	ex
Early Neonatal	0.363059457	0.009577913	100000	0.019111471	72.71131393	0.286393625	0.009580263	100000	0.019125511	78.80427857
Late Neonatal	0.037433546	0.028756797	99306.14202	0.057073559	73.20010347	0.030147109	0.028758807	99452.25862	0.057169514	79.21906688
Post Neonatal	0.007655812	0.46109998	99092.49694	0.911682912	73.30032739	0.006236287	0.46120082	99279.90995	0.914005286	79.29900622
1 to 4	0.000777421	1.998963439	98394.53566	3.929668333	72.89372621	0.000688705	1.999081727	98709.91317	3.942963002	78.83096479
5 to 9	0.000343117	2.499285172	98089.03947	4.900247399	69.11452929	0.000268729	2.499440147	98438.36167	4.918612917	75.04291452
10 to 14	0.000401696	3.092766469	97920.90459	4.892297093	64.22891139	0.000284564	2.658646352	98306.1846	4.912036533	70.14045255
15 to 19	0.001493395	2.769441365	97724.38355	4.869996778	59.35185363	0.000486721	2.632836327	98166.40623	4.902671738	65.2365386
20 to 24	0.002358067	2.585244164	96997.10181	4.82239566	54.77609737	0.000597098	2.580743811	97927.78312	4.889326393	60.38908399
25 to 29	0.002503586	2.520560332	95859.95139	4.763428672	50.39520301	0.000721852	2.594762404	97635.84273	4.873330959	55.56193289
30 to 34	0.002668411	2.536708664	94667.39112	4.702460061	45.99828509	0.000931199	2.626778958	97284.06116	4.853477167	50.75345916
35 to 39	0.003049117	2.568185863	93412.58819	4.636252333	41.58209002	0.001298583	2.642796345	96832.10718	4.826830328	45.97808055
40 to 44	0.003767464	2.598633836	91998.95019	4.558704926	37.18155899	0.001840578	2.654848886	96205.3056	4.789591345	41.26041377
45 to 49	0.004991315	2.628932907	90281.48821	4.461276838	32.83943217	0.0027068	2.666422459	95323.74775	4.736270683	36.61743094
50 to 54	0.007149703	2.638632698	88054.74671	4.329640535	28.60338551	0.004091446	2.665781286	94041.74104	4.657605629	32.08025105
55 to 59	0.010337937	2.633565261	84959.22166	4.146521877	24.54940522	0.006161909	2.664231154	92136.12062	4.541442512	27.68860711
60 to 64	0.015004293	2.623014192	80672.64363	3.89472946	20.71389539	0.009407716	2.654823559	89337.75204	4.370464311	23.47244076
65 to 69	0.02175342	2.613113646	74829.00087	3.556776796	17.12665517	0.014196147	2.647895436	85226.19775	4.123621287	19.47672223
70 to 74	0.032262342	2.599080237	67092.01615	3.113444857	13.80033179	0.021905899	2.646199131	79372.35756	3.77402625	15.71785432
75 to 79	0.049108822	2.570033237	57047.71543	2.548305703	10.77252543	0.035547093	2.62912412	71105.24919	3.278932343	12.23762388
80 to 84	0.076089011	2.518264741	44533.97827	1.873034062	8.077419242	0.059628044	2.582477124	59450.09004	2.598015964	9.121369834
85 to 89	0.13469295	2.421223197	30283.26443	1.123836243	5.693580122	0.111822611	2.499952923	43959.52793	1.717782659	6.425620697
90 to 94	0.22995826	2.185584796	15147.05292	0.459793646	3.96383971	0.197124869	2.2781794	24752.086	0.805468008	4.472079547
95 plus	0.325275079	3.074333068	4574.307326	0.140634718	3.074333068	0.294377418	3.397024781	8875.256978	0.30150823	3.397024781

Table 15: Latin America and Caribbean 2100 life table, by age and sex. mx=mortality rate, ax=mean person-years lived in an age interval among those who die in that age interval, lx=number of persons left alive at age x, nLx=person-years lived between age x and x+n, ex=life expectancy at age x.

Age Group	Male					Female				
	mx	ax	lx	nLx	ex	mx	ax	lx	nLx	ex
Early Neonatal	0.070426573	0.009586883	100000	0.019165137	80.80351082	0.062168351	0.009587136	100000	0.019166654	84.95872582
Late Neonatal	0.007773843	0.028764979	99865.03465	0.057443749	80.89350166	0.008557837	0.028764763	99880.84922	0.05745155	85.04085673
Post Neonatal	0.001445767	0.461541131	99820.38122	0.921014469	80.87212292	0.001279193	0.461552964	99831.6862	0.921189594	85.02516866
1 to 4	0.000176109	1.999765189	99687.23711	3.986085624	80.05617757	0.000162834	1.999782888	99713.86005	3.987256005	84.20173922
5 to 9	0.000103009	2.499785398	99617.0549	4.979570753	76.11104065	9.97903E-05	2.499792104	99648.94947	4.981205164	80.2551917
10 to 14	0.000158095	3.123124279	99565.78166	4.976767898	71.14881933	0.000139009	2.714069734	99599.26312	4.978368327	75.29390201
15 to 19	0.000532856	2.777405604	99487.15549	4.968455323	66.20244544	0.000229639	2.607135837	99530.09548	4.973768098	70.34427984
20 to 24	0.000850622	2.605544601	99222.53709	4.951030723	61.37124874	0.000253171	2.57422676	99415.91554	4.967744827	65.42201666
25 to 29	0.000952251	2.54942389	98801.66682	4.928579462	56.62123007	0.000319432	2.603803857	99290.19311	4.960710432	60.50152736
30 to 34	0.001073754	2.549675327	98332.74842	4.903732564	51.87872443	0.000409747	2.611565022	99131.79987	4.951740167	55.59388756
35 to 39	0.001205013	2.584334251	97806.71942	4.876150924	47.14369912	0.000535114	2.648744373	98928.98574	4.940235353	50.7023206
40 to 44	0.001571799	2.632959291	97219.78449	4.842975734	42.41224285	0.000794574	2.701257119	98664.7395	4.924238755	45.83071609
45 to 49	0.002223356	2.688607299	96459.35869	4.79831925	37.72510669	0.001303253	2.706091604	98273.65862	4.899030521	41.00195672
50 to 54	0.003632476	2.686298888	95393.7897	4.729960095	33.11549601	0.002090376	2.690389702	97635.58739	4.858325484	36.25160189
55 to 59	0.005603434	2.658148455	93677.90448	4.623244039	28.67136049	0.00325218	2.668865668	96620.80838	4.794657665	31.60324477
60 to 64	0.008151719	2.655134537	91091.8202	4.469302109	24.40826826	0.004773692	2.661923455	95063.25385	4.700759635	27.07628652
65 to 69	0.012469023	2.652771211	87455.49258	4.248616042	20.3098506	0.007252444	2.685605395	92821.54231	4.564480347	22.66397096
70 to 74	0.018982496	2.657165739	82172.1492	3.934228409	16.44257738	0.011798112	2.720132335	89516.91691	4.358834739	18.39937974
75 to 79	0.031427439	2.642601195	74731.82295	3.48021769	12.81101543	0.021760323	2.717527878	84386.14939	4.020195615	14.34922289
80 to 84	0.052613082	2.577355284	63866.50915	2.835718694	9.538310109	0.041017798	2.648208502	75677.64473	3.453144545	10.68412756
85 to 89	0.103410552	2.515395594	49101.67663	1.958886153	6.628538046	0.084266996	2.600508556	61618.51135	2.567433418	7.509473677
90 to 94	0.196324966	2.235904644	29133.35071	0.948848793	4.47449254	0.163803665	2.331672421	40234.77238	1.405411081	5.130563917
95 plus	0.297525719	3.366535639	10748.87029	0.365197185	3.366535639	0.263081943	3.810824404	17501.38221	0.673377841	3.810824404

Table 15: Andean Latin America 2017 life table, by age and sex. mx=mortality rate, ax=mean person-years lived in an age interval among those who die in that age interval, lx=number of persons left alive at age x, nLx=person-years lived between age x and x+n, ex=life expectancy at age x.

Age Group	Male					Female				
	mx	ax	lx	nLx	ex	mx	ax	lx	nLx	ex
Early Neonatal	0.370543831	0.009577684	100000	0.019110102	76.06280461	0.278674752	0.0095805	100000	0.019126926	79.28726203
Late Neonatal	0.032093463	0.02875827	99291.91208	0.057074148	76.58593443	0.036055263	0.028757177	99466.99244	0.057168272	79.69289002
Post Neonatal	0.007411786	0.461117315	99108.75652	0.911935398	76.66987521	0.00579297	0.461232313	99260.88201	0.914017115	79.80077575
1 to 4	0.001040875	1.998612168	98432.92392	3.929132989	76.26985049	0.000848034	1.998869288	98731.43143	3.942567218	79.30297168
5 to 9	0.000445892	2.499071058	98024.01143	4.895741573	72.57971642	0.000349916	2.499271008	98397.11961	4.915554829	75.56564908
10 to 14	0.00041511	2.708721238	97805.73279	4.885640085	67.73613253	0.000337468	2.598832228	98225.12379	4.907280136	70.69358945
15 to 19	0.000865421	2.729016222	97602.93533	4.870575052	62.87122653	0.000511951	2.595045782	98059.52326	4.8969475	65.80856343
20 to 24	0.001375997	2.608927779	97181.43348	4.843137406	58.13195578	0.000573769	2.562259341	97808.82671	4.883611171	60.9705263
25 to 29	0.001601559	2.547994952	96515.03672	4.806875925	53.515135	0.000687372	2.589089149	97528.62591	4.868363895	56.13825222
30 to 34	0.001770225	2.54566297	95745.22257	4.766553815	48.92473612	0.000873044	2.619067001	97193.9988	4.84962024	51.32251387
35 to 39	0.002022824	2.573243054	94901.493	4.721898585	44.3368695	0.001195661	2.641532755	96770.62483	4.824926656	46.53546496
40 to 44	0.002527461	2.606600014	93946.43458	4.669082086	39.76118542	0.001702416	2.656399938	96193.76626	4.79057616	41.79850065
45 to 49	0.003388819	2.641389351	92766.5151	4.601553219	35.23344423	0.002507611	2.664529358	95378.29058	4.741150335	37.13290741
50 to 54	0.004965894	2.647827398	91207.45147	4.507732712	30.79015212	0.003756645	2.663664353	94189.55732	4.66850939	32.56759647
55 to 59	0.007194801	2.655595842	88969.58555	4.374713116	26.49749675	0.005624828	2.671443102	92436.09586	4.562064547	28.1344527
60 to 64	0.010967366	2.654639388	85823.26448	4.183600658	22.37092542	0.008776208	2.662039355	89870.69381	4.403217352	23.86080256
65 to 69	0.016768806	2.6443811	81237.30345	3.907615765	18.48326083	0.013362438	2.652685121	86007.72562	4.169667032	19.81232539
70 to 74	0.025947911	2.63241885	74689.48158	3.518533779	14.87117932	0.020723503	2.652518702	80439.0279	3.835502079	15.99959163
75 to 79	0.041434206	2.59978732	65569.37389	2.982313243	11.57320719	0.033947695	2.635893732	72496.92674	3.355829214	12.46119546
80 to 84	0.065582792	2.557106427	53231.60348	2.294679197	8.653860142	0.057262697	2.589012489	61118.68676	2.685757974	9.290335711
85 to 89	0.121343386	2.460035356	38212.86753	1.461253187	6.05301575	0.108478948	2.511324981	45765.92465	1.802632126	6.540016002
90 to 94	0.216122038	2.209638813	20517.90785	0.640394275	4.157863912	0.193262996	2.285571752	26249.52486	0.861451813	4.540177009
95 plus	0.31398612	3.185276203	6700.286531	0.213658289	3.185276203	0.290798187	3.439609679	9630.9783	0.331720047	3.439609679

Table 15: Andean Latin America 2100 life table, by age and sex. mx=mortality rate, ax=mean person-years lived in an age interval among those who die in that age interval, lx=number of persons left alive at age x, nLx=person-years lived between age x and x+n, ex=life expectancy at age x.

Age Group	Male					Female				
	mx	ax	lx	nLx	ex	mx	ax	lx	nLx	ex
Early Neonatal	0.07250239	0.009586819	100000	0.019164757	82.58104061	0.060670217	0.009587182	100000	0.01916693	85.20139151
Late Neonatal	0.006340903	0.028765374	99861.07526	0.05744384	82.67664824	0.009825819	0.028764413	99883.7295	0.057451113	85.28129223
Post Neonatal	0.001449376	0.461540874	99824.65657	0.921052444	82.64920711	0.001253746	0.461554772	99827.28857	0.921159878	85.27189076
1 to 4	0.000308089	1.999589215	99691.19146	3.98519291	81.83569386	0.000286011	1.999618652	99711.82173	3.986193616	84.44658905
5 to 9	0.000122975	2.499743803	99568.49676	4.976898523	77.93304221	0.000114322	2.49976183	99597.89929	4.978475615	80.54015144
10 to 14	0.000159951	2.838612967	99507.46855	4.973617601	72.97828857	0.000149075	2.6696809	99541.14579	4.975311582	75.58403177
15 to 19	0.000339409	2.750421896	99428.33879	4.967606278	68.03295201	0.000212876	2.584725807	99467.25555	4.970802779	70.63767945
20 to 24	0.000534021	2.63139619	99260.55019	4.956763301	63.1421217	0.000224411	2.579533796	99361.75875	4.965395719	65.70956606
25 to 29	0.000656293	2.578878364	98997.07453	4.942030539	58.3022129	0.000295114	2.618303895	99250.7061	4.959052398	60.77987743
30 to 34	0.000776687	2.551739452	98674.39801	4.924388238	53.48352759	0.000384218	2.615205703	99104.84225	4.950706051	55.86506885
35 to 39	0.000838917	2.575161306	98293.93401	4.904770472	48.67996403	0.000495503	2.655791636	98915.21874	4.940026045	50.9666844
40 to 44	0.001080454	2.640509343	97884.88723	4.881846282	43.87195247	0.000743816	2.706023756	98671.18205	4.925149305	46.0854147
45 to 49	0.00155721	2.70776622	97360.4518	4.850748246	39.09308035	0.001209237	2.71007407	98305.86614	4.90172261	41.24559788
50 to 54	0.002610148	2.707248796	96609.25799	4.801769908	34.37448037	0.001948299	2.692180302	97714.74152	4.863902223	36.47716245
55 to 59	0.004136686	2.693681458	95362.18206	4.723056519	29.7861547	0.003028109	2.687897975	96769.86461	4.804765043	31.80459132
60 to 64	0.006428506	2.688720918	93418.71314	4.602772764	25.34702235	0.004560265	2.676305783	95320.9463	4.716236918	27.243959
65 to 69	0.010352	2.686143173	90475.25057	4.418346139	21.0796472	0.007128915	2.697532902	93178.57982	4.583797534	22.80333576
70 to 74	0.01685718	2.686807017	85928.41972	4.136334075	17.04847959	0.011643021	2.738087972	89930.25413	4.381765284	18.5231698
75 to 79	0.029005379	2.659875516	79022.85384	3.702745538	13.29508009	0.022312675	2.72363091	84871.079	4.040219558	14.45269105
80 to 84	0.048381983	2.605145962	68450.94973	3.073737669	9.934492847	0.041508625	2.647457955	75991.4144	3.468419533	10.81069587
85 to 89	0.096876058	2.537100698	53913.16839	2.189508984	6.896060811	0.084146814	2.605246273	61976.77207	2.596648297	7.618212899
90 to 94	0.188340904	2.240276749	33379.95689	1.109838665	4.626783487	0.162584158	2.325044607	41074.8904	1.452572141	5.208043589
95 plus	0.290728239	3.453886243	13093.76593	0.461406922	3.453886243	0.261678509	3.86091454	18563.92322	0.742451339	3.86091454

Table 15: Bolivia 2017 life table, by age and sex. mx=mortality rate, ax=mean person-years lived in an age interval among those who die in that age interval, lx=number of persons left alive at age x, nLx=person-years lived between age x and x+n, ex=life expectancy at age x.

Age Group	Male					Female				
	mx	ax	lx	nLx	ex	mx	ax	lx	nLx	ex
Early Neonatal	0.545152248	0.009572332	100000	0.019078189	71.64798208	0.400094749	0.009576778	100000	0.019104698	74.46250073
Late Neonatal	0.060100426	0.028750545	98960.13067	0.05683768	72.38121292	0.056881558	0.028751433	99235.70744	0.057001215	75.01667899
Post Neonatal	0.010362664	0.46090769	98618.67356	0.906194518	72.57418803	0.008087735	0.461069297	98911.55428	0.909838123	75.20493788
1 to 4	0.001415451	1.998112733	97680.20053	3.896176856	72.3440229	0.001306407	1.998258125	98175.98072	3.916801522	74.84191316
5 to 9	0.000543295	2.498868136	97129.17778	4.849870818	68.74345152	0.000407677	2.499150673	97664.55113	4.878254466	71.22365055
10 to 14	0.000457081	2.646173099	96865.77467	4.838085138	63.92365845	0.000337861	2.602577995	97465.69556	4.869340858	66.36374475
15 to 19	0.00086836	2.678519662	96644.68472	4.822515422	59.06380439	0.000575712	2.636958528	97301.20243	4.858452888	61.47150862
20 to 24	0.001211541	2.562866475	96225.92801	4.797129543	54.30862219	0.000721401	2.580513333	97021.51106	4.842625912	56.64092292
25 to 29	0.001242677	2.533252291	95644.7881	4.767625957	49.6223517	0.000861859	2.609668912	96672.1936	4.823674837	51.83591706
30 to 34	0.001425126	2.584046183	95052.44389	4.736321738	44.91535613	0.001185105	2.648025021	96256.52051	4.79945352	47.04805786
35 to 39	0.001843584	2.633071564	94377.62854	4.698394239	40.21748132	0.001723264	2.663117567	95687.8504	4.765215079	42.31129874
40 to 44	0.00265219	2.671507866	93511.71919	4.646911415	35.56450638	0.002576944	2.665853512	94866.93868	4.715002344	37.65342785
45 to 49	0.004126576	2.696290517	92279.8417	4.570582639	31.00178971	0.003857326	2.666609763	93652.49964	4.640874781	33.10556709
50 to 54	0.006775366	2.690498585	90395.18362	4.450216589	26.58900511	0.005832121	2.653409035	91863.72244	4.531230489	28.69625299
55 to 59	0.0109107	2.68365629	87383.94344	4.261699279	22.40800122	0.008532854	2.661292033	89224.05347	4.374022576	24.46436862
60 to 64	0.017896604	2.656372385	82744.84733	3.971210481	18.50765802	0.013308856	2.657828108	85497.95263	4.145895841	20.4119528
65 to 69	0.028122584	2.623060688	75663.44257	3.547272367	14.98462811	0.020725753	2.65055641	79991.52025	3.814330422	16.63150415
70 to 74	0.043710767	2.586271577	65742.79437	2.975712473	11.84484707	0.033593073	2.626682459	72109.09031	3.340120392	13.15751132
75 to 79	0.067866789	2.538935205	52845.49667	2.268035144	9.104905306	0.054749087	2.582094274	60931.38199	2.692174228	10.08850503
80 to 84	0.107512239	2.466382705	37642.354	1.484512967	6.771113361	0.090194841	2.506533685	46276.83125	1.891886373	7.469052316
85 to 89	0.171557799	2.323722112	21942.39541	0.756090186	4.892603041	0.151431543	2.382703765	29354.05213	1.054056731	5.345405458
90 to 94	0.265804206	2.113676675	9178.632257	0.261348328	3.5347783	0.239990771	2.184482989	13538.41412	0.405543944	3.840479207
95 plus	0.353786812	2.828726946	2319.171827	0.06616011	2.828726946	0.333051639	3.004853683	3887.835921	0.11759949	3.004853683

Table 15: Bolivia 2100 life table, by age and sex. mx=mortality rate, ax=mean person-years lived in an age interval among those who die in that age interval, lx=number of persons left alive at age x, nLx=person-years lived between age x and x+n, ex=life expectancy at age x.

Age Group	Male					Female				
	mx	ax	lx	nLx	ex	mx	ax	lx	nLx	ex
Early Neonatal	0.103743446	0.009585861	100000	0.019159019	79.41644687	0.088363526	0.009586333	100000	0.019161843	82.81227176
Late Neonatal	0.011265939	0.028764016	99801.28402	0.057401315	79.55505955	0.016031794	0.028762701	99830.71001	0.057410371	82.93330645
Post Neonatal	0.001911451	0.461508049	99736.62976	0.920044205	79.54899846	0.001563586	0.461532761	99738.6929	0.920210832	82.95212659
1 to 4	0.000277818	1.999629575	99560.84758	3.980223701	78.76497315	0.000270757	1.999638992	99594.85454	3.98163941	82.14763689
5 to 9	0.000108185	2.499774615	99450.3786	4.971174742	74.84987703	9.52867E-05	2.499801486	99487.15543	4.973173446	78.23402716
10 to 14	0.000131165	2.79212618	99396.61615	4.968388404	69.8889542	0.000112333	2.690735205	99439.78641	4.970698748	73.27003696
15 to 19	0.000288689	2.707001804	99331.46215	4.963287772	64.93292857	0.000197027	2.622257738	99383.96264	4.966873975	68.30962958
20 to 24	0.000418493	2.577555217	99188.20518	4.954412122	60.02269233	0.000230314	2.576858512	99286.13198	4.961554068	63.37425638
25 to 29	0.000468767	2.535900217	98980.92835	4.943409583	55.14279132	0.00029745	2.636252527	99171.91538	4.955123013	58.44412818
30 to 34	0.0005676	2.593150984	98749.4553	4.93072975	50.26560713	0.000433115	2.660596543	99024.69442	4.946199918	53.52680556
35 to 39	0.000706069	2.666500596	98470.18244	4.915331026	45.40041164	0.000603952	2.690150144	98810.79397	4.933619015	48.6365909
40 to 44	0.001038432	2.724317288	98124.08203	4.894538602	40.55088102	0.000941162	2.718159315	98513.32849	4.915070147	43.77496577
45 to 49	0.001711438	2.762416405	97617.09048	4.862154105	35.74682666	0.00154681	2.704050863	98051.41033	4.885185175	38.96788345
50 to 54	0.003121069	2.753246015	96786.53108	4.805583147	31.0287236	0.002429382	2.68641581	97296.72811	4.837604328	34.24843901
55 to 59	0.005545138	2.729290877	95289.02787	4.705135737	26.47108007	0.003720998	2.677495399	96122.87992	4.764903267	29.63279498
60 to 64	0.009440377	2.70828548	92685.26523	4.536262208	22.13479873	0.005648592	2.685396445	94352.2736	4.656754992	25.13730175
65 to 69	0.016074526	2.681517042	88414.12544	4.262256733	18.06734464	0.009096529	2.711422509	91725.95515	4.4927711	20.77785505
70 to 74	0.026265968	2.660960872	81597.19107	3.845229939	14.34622582	0.015789087	2.734226206	87649.51463	4.231417133	16.61486756
75 to 79	0.045194898	2.624139902	71578.61764	3.235821254	10.9706852	0.030476034	2.697579462	80993.41118	3.785460842	12.74900992
80 to 84	0.077859714	2.548706241	57161.01704	2.408369639	8.071150287	0.057132916	2.603886321	69543.61951	3.063458396	9.394891987
85 to 89	0.136505094	2.418022241	38823.85792	1.446417428	5.688293474	0.107799501	2.516626293	52300.11841	2.072899819	6.617460252
90 to 94	0.231293385	2.180795678	19638.84101	0.600866887	3.962086987	0.19190459	2.284951104	30483.65572	1.011450611	4.59009148
95 plus	0.326201255	3.073303691	6085.191844	0.190249552	3.073303691	0.289359239	3.471505262	11564.30038	0.409631307	3.471505262

Table 15: Ecuador 2017 life table, by age and sex. mx=mortality rate, ax=mean person-years lived in an age interval among those who die in that age interval, lx=number of persons left alive at age x, nLx=person-years lived between age x and x+n, ex=life expectancy at age x.

Age Group	Male					Female				
	mx	ax	lx	nLx	ex	mx	ax	lx	nLx	ex
Early Neonatal	0.33128316	0.009578887	100000	0.019117293	74.95208579	0.24152106	0.009581639	100000	0.019133738	78.73512781
Late Neonatal	0.036051846	0.028757178	99366.74923	0.057110683	75.41041368	0.036965551	0.028756926	99537.92111	0.057207556	79.08135621
Post Neonatal	0.006635461	0.461172464	99160.94436	0.912742638	75.50931444	0.005186926	0.461275365	99326.5252	0.914877495	79.19205056
1 to 4	0.000970675	1.998705768	98555.46373	3.934578312	75.04712468	0.00093055	1.998759267	98852.0773	3.946735679	78.64663255
5 to 9	0.000469826	2.499021196	98173.70434	4.902925627	71.33129828	0.00036763	2.499234103	98484.93947	4.91972487	74.93245606
10 to 14	0.000499161	2.787890503	97943.41147	4.891769061	66.49318806	0.000384814	2.624307985	98304.11101	4.910716621	70.06572322
15 to 19	0.00116428	2.789036675	97699.27044	4.872422503	61.65235965	0.000599321	2.590235385	98115.15817	4.898684063	65.19557593
20 to 24	0.002134924	2.626679643	97132.01107	4.832117454	56.99587608	0.000648	2.562992932	97821.58158	4.883368172	60.38336512
25 to 29	0.002500749	2.534146765	96100.44693	4.775575951	52.57904444	0.000800432	2.59027849	97505.15545	4.865873177	55.57089023
30 to 34	0.002602776	2.513590637	94906.3056	4.714808105	48.20828436	0.001001853	2.606478427	97115.70081	4.844170592	50.7832071
35 to 39	0.002740052	2.540338453	93679.31513	4.652616584	43.80636191	0.001324444	2.614825434	96630.42471	4.816307762	46.02496974
40 to 44	0.003216886	2.579731489	92404.73211	4.584552937	39.3751204	0.001750871	2.648274737	95992.59226	4.779948827	41.31315044
45 to 49	0.004085851	2.615828538	90930.34813	4.502670805	34.97125724	0.002606968	2.66852247	95155.79381	4.729049343	36.65286912
50 to 54	0.005682505	2.623785334	89091.31802	4.395243416	30.63860438	0.003940065	2.67092836	93923.15744	4.65346266	32.09849368
55 to 59	0.007816343	2.631915415	86594.94116	4.251102803	26.44562097	0.006026455	2.680487744	92090.0896	4.54104553	27.68376085
60 to 64	0.011344278	2.628197202	83274.1815	4.054689091	22.39465057	0.00962191	2.647822229	89354.34088	4.368881458	23.44865062
65 to 69	0.016239262	2.635385995	78677.93081	3.788555767	18.5488209	0.013875313	2.631283501	85152.58447	4.122235553	19.47426348
70 to 74	0.025118128	2.632111012	72531.75534	3.423248399	14.89688222	0.020615009	2.650570046	79436.84018	3.788532401	15.68537009
75 to 79	0.039727457	2.620103481	63944.9703	2.921520627	11.54388119	0.034272693	2.660831051	71635.11446	3.316276068	12.10435972
80 to 84	0.067944535	2.589110729	52360.27078	2.250254917	8.519509571	0.063357777	2.598710795	60287.27337	2.617029521	8.882043498
85 to 89	0.124642395	2.450316688	37106.47765	1.40873819	5.961081202	0.117070942	2.482981714	43741.68891	1.690270924	6.261088479
90 to 94	0.21958115	2.203847341	19588.43676	0.607303791	4.108103777	0.20302711	2.266086718	24001.23993	0.772388465	4.375013579
95 plus	0.316819473	3.156819913	6277.721648	0.19842083	3.156819913	0.299795021	3.336502508	8354.323549	0.27920734	3.336502508

Table 15: Ecuador 2100 life table, by age and sex. mx=mortality rate, ax=mean person-years lived in an age interval among those who die in that age interval, lx=number of persons left alive at age x, nLx=person-years lived between age x and x+n, ex=life expectancy at age x.

Age Group	Male					Female				
	mx	ax	lx	nLx	ex	mx	ax	lx	nLx	ex
Early Neonatal	0.072880098	0.009586807	100000	0.019164693	82.20172565	0.054576424	0.009587368	100000	0.019168052	84.57669075
Late Neonatal	0.009916622	0.028764388	99860.43279	0.057437572	82.29697597	0.011880428	0.028763846	99895.42767	0.057454451	84.64569946
Post Neonatal	0.001400596	0.46154434	99803.51972	0.920878428	82.28616883	0.001248548	0.461555141	99827.20454	0.921161511	84.64590027
1 to 4	0.000195709	1.999739055	99674.64129	3.985427108	81.46809675	0.000190397	1.999746137	99712.2664	3.986973515	83.81911727
5 to 9	0.000118366	2.499753405	99596.73035	4.978364018	77.52988226	0.000119036	2.499752009	99636.42373	4.980340013	79.88109505
10 to 14	0.000173864	2.994361318	99537.83805	4.97509798	72.57414706	0.000166009	2.702260357	99577.18493	4.976938525	74.92688143
15 to 19	0.000475201	2.824845657	99451.39852	4.967404767	67.63453767	0.000257274	2.589050285	99494.6443	4.971641712	69.98656526
20 to 24	0.000886733	2.64243932	99215.52442	4.950397721	62.78704991	0.000267049	2.600761496	99366.86433	4.965152585	65.0724299
25 to 29	0.001050781	2.555562679	98777.01702	4.926183098	58.05202461	0.000375245	2.616425497	99234.40537	4.957280295	60.15519519
30 to 34	0.001145084	2.515198895	98260.18648	4.899075483	53.34185469	0.000467042	2.595329453	99048.62088	4.946861069	55.26211087
35 to 39	0.00112738	2.540573517	97700.18804	4.871551609	48.6313756	0.00056918	2.617076846	98817.93531	4.934222646	50.38391198
40 to 44	0.001402679	2.617015401	97152.1495	4.841463914	43.88925492	0.000799096	2.703268275	98537.51197	4.917838426	45.51831279
45 to 49	0.001932146	2.686188414	96475.08716	4.802319912	39.17557	0.001329849	2.720356409	98145.32364	4.892411939	40.68711166
50 to 54	0.003142122	2.678674745	95550.63007	4.742987007	34.52415447	0.002169526	2.706629284	97496.48338	4.850691424	35.93616355
55 to 59	0.004638768	2.658255579	94067.40287	4.6528384	30.02007599	0.003455612	2.711475101	96448.24836	4.784425991	31.29197919
60 to 64	0.006582177	2.648371782	91923.30647	4.526345802	25.65131846	0.005470458	2.65550667	94804.3056	4.680523367	26.78144365
65 to 69	0.009564032	2.672694888	88965.12898	4.352048986	21.40742976	0.007806844	2.667216657	92262.24249	4.530830192	22.43452234
70 to 74	0.015776568	2.692240751	84835.59798	4.094787856	17.31141218	0.011771003	2.75897552	88765.66049	4.32460241	18.2021949
75 to 79	0.027066646	2.701364274	78494.99583	3.699784912	13.47443876	0.023285099	2.780199389	83759.94109	3.984289536	14.10827001
80 to 84	0.048685061	2.676710473	68818.89137	3.102648786	9.981272996	0.047429146	2.672214088	74761.42132	3.380156251	10.44807049
85 to 89	0.097074537	2.537383972	54330.07273	2.214322668	6.925181703	0.092259188	2.579479424	59553.07343	2.467786281	7.373022887
90 to 94	0.188088557	2.236573945	33951.36948	1.135690928	4.646308789	0.171636533	2.306007326	38576.65088	1.355649267	5.064451808
95 plus	0.290423695	3.465158193	13579.58943	0.485504001	3.465158193	0.269949913	3.771492471	17214.92494	0.690291288	3.771492471

Table 15: Peru 2017 life table, by age and sex. mx=mortality rate, ax=mean person-years lived in an age interval among those who die in that age interval, lx=number of persons left alive at age x, nLx=person-years lived between age x and x+n, ex=life expectancy at age x.

Age Group	Male					Female				
	mx	ax	lx	nLx	ex	mx	ax	lx	nLx	ex
Early Neonatal	0.31777687	0.009579301	100000	0.019119765	78.31431096	0.245864804	0.009581505	100000	0.01913294	81.46344783
Late Neonatal	0.019413944	0.028761768	99392.46143	0.057152784	78.77363875	0.027410199	0.028759562	99529.60798	0.057218485	81.82919563
Post Neonatal	0.00658149	0.461176298	99281.52259	0.913875204	78.80408571	0.005142197	0.461278543	99372.78624	0.91532241	81.90075377
1 to 4	0.00092464	1.998767146	98680.19766	3.939919579	78.35821914	0.00063083	1.999158893	98902.1798	3.951101056	81.36502714
5 to 9	0.000397759	2.499171335	98316.00996	4.910916542	74.64114055	0.000318684	2.499336075	98652.9792	4.928721597	77.56555193
10 to 14	0.00035456	2.667723438	98120.71691	4.901983382	69.78475635	0.000310347	2.574142942	98495.92654	4.92109304	72.68526273
15 to 19	0.000686158	2.705866524	97946.93557	4.889652114	64.90378032	0.000431745	2.580209818	98343.21043	4.912030417	67.79406391
20 to 24	0.001039792	2.617120382	97611.44885	4.868509875	60.11723421	0.000479707	2.55812694	98131.14233	4.900817483	62.93481475
25 to 29	0.001280378	2.568307323	97105.2659	4.840194738	55.41650051	0.000568863	2.580645865	97896.05922	4.888076549	58.07958508
30 to 34	0.001475361	2.559644399	96485.61638	4.806978446	50.7553917	0.0007038	2.613734822	97618.01787	4.872719863	53.23739675
35 to 39	0.001726997	2.577566673	95776.54642	4.768884172	46.11164227	0.000960365	2.649992788	97275.11653	4.852805386	48.41552829
40 to 44	0.002153105	2.602412096	94953.19649	4.723287626	41.48845818	0.001406159	2.660276339	96809.15374	4.824585645	43.63533376
45 to 49	0.002838127	2.634991783	93936.63889	4.66553206	36.90847831	0.002062595	2.66249079	96130.91023	4.783487267	38.9238229
50 to 54	0.004087405	2.640304252	92613.2529	4.586454109	32.39727313	0.003062647	2.661406042	95144.60566	4.723412458	34.29895059
55 to 59	0.005774307	2.651490819	90740.008	4.476345644	28.01042123	0.004543806	2.666143665	93698.66461	4.635799749	29.78618266
60 to 64	0.00868056	2.665922217	88157.78028	4.320450168	23.75192876	0.006934367	2.66813411	91593.62324	4.506867537	25.40820144
65 to 69	0.013615969	2.658575248	84412.08571	4.090408853	19.68620195	0.010726946	2.663778873	88471.25656	4.315555905	21.20918219
70 to 74	0.02127558	2.65680492	78852.16909	3.755803354	15.88538147	0.016774903	2.667548923	83848.35492	4.034860529	17.22977129
75 to 79	0.035439536	2.619107435	70881.28237	3.269149287	12.37207934	0.027840078	2.651427837	77094.49131	3.618811944	13.50365286
80 to 84	0.055885308	2.574760105	59337.17344	2.614158289	9.270789324	0.046644213	2.618530964	67052.69048	3.018728744	10.12832517
85 to 89	0.108242885	2.499776428	44796.0989	1.764631248	6.449924904	0.093006814	2.566356891	53035.27529	2.164410027	7.11615879
90 to 94	0.201971359	2.230682555	25786.85381	0.828094042	4.374524883	0.174894955	2.317594395	33008.14494	1.125217374	4.887024909
95 plus	0.302289031	3.309243689	9126.624352	0.302809676	3.309243689	0.273629923	3.657150192	13423.25506	0.492671423	3.657150192

Table 15: Peru 2100 life table, by age and sex. mx=mortality rate, ax=mean person-years lived in an age interval among those who die in that age interval, lx=number of persons left alive at age x, nLx=person-years lived between age x and x+n, ex=life expectancy at age x.										
Age Group	Male					Female				
	mx	ax	lx	nLx	ex	mx	ax	lx	nLx	ex
Early Neonatal	0.055377416	0.009587344	100000	0.019167903	84.27433606	0.04714446	0.009587596	100000	0.019169416	86.79920812
Late Neonatal	0.002409163	0.028766459	99893.87484	0.057469206	84.34437702	0.005728169	0.028765543	99909.64816	0.057472795	86.85829529
Post Neonatal	0.001209376	0.461557924	99880.03184	0.921665433	84.29837897	0.001081857	0.461566982	99876.73646	0.921689286	86.82924883
1 to 4	0.000361748	1.999517669	99768.58888	3.987861298	83.46800439	0.00032556	1.999565921	99777.04662	3.988488042	85.99164785
5 to 9	0.000133569	2.499721732	99624.56143	4.979578664	79.58242127	0.000124753	2.499740099	99647.43848	4.980830717	82.0984472
10 to 14	0.000172907	2.850034695	99558.65448	4.976013311	74.63014014	0.000164719	2.657777288	99585.84781	4.977348955	77.14564629
15 to 19	0.000326565	2.730607876	99474.07571	4.970030697	69.68739509	0.00020574	2.553956887	99504.806	4.972749332	72.20458607
20 to 24	0.000483972	2.657519954	99314.54176	4.960131536	64.79086741	0.00020442	2.570621041	99403.56268	4.967728003	67.27426523
25 to 29	0.000626934	2.605110774	99078.63831	4.946595675	59.93546452	0.000262619	2.607883251	99303.26573	4.962067634	62.33860048
30 to 34	0.000761333	2.555850468	98774.12789	4.929665805	55.10947568	0.000325352	2.606427928	99174.52169	4.954892096	57.41512852
35 to 39	0.000807492	2.56002726	98405.49467	4.910779925	50.30436727	0.000409311	2.674370373	99015.20923	4.946058319	52.5022964
40 to 44	0.000989999	2.621375881	98016.86888	4.889514452	45.49222928	0.000622969	2.717738489	98815.03015	4.933738928	47.60187869
45 to 49	0.001354104	2.699206391	97542.23411	4.862118009	40.69946763	0.00099987	2.728026169	98510.59733	4.91437293	42.7389602
50 to 54	0.002178421	2.703578374	96895.90703	4.820837279	35.95115954	0.001645039	2.696429826	98023.26277	4.882761174	37.93544087
55 to 59	0.003306591	2.699320618	95862.32149	4.757040666	31.30697632	0.002549467	2.700009809	97226.14485	4.83280193	33.22044256
60 to 64	0.005029613	2.709927297	94312.84276	4.662299621	26.77437515	0.003715315	2.689240995	96006.33828	4.75973523	28.60292805
65 to 69	0.008253537	2.70563333	92001.70847	4.515303747	22.37468264	0.006026159	2.717454647	94254.09167	4.648967562	24.07649346
70 to 74	0.013705454	2.725599643	88323.02672	4.283930813	18.1896339	0.009936436	2.756095647	91486.53419	4.475467296	19.7117091
75 to 79	0.024525876	2.689299195	82562.9164	3.910034773	14.26086412	0.019052801	2.742265096	87117.36799	4.178557796	15.54472877
80 to 84	0.040503616	2.614429423	73228.76203	3.347779806	10.73793218	0.034758972	2.679273093	79372.33469	3.682202654	11.77827912
85 to 89	0.084265257	2.579239757	60152.10187	2.520430362	7.458234577	0.07235725	2.657387044	67171.85856	2.901702536	8.348414036
90 to 94	0.172309427	2.241272451	40098.93445	1.383382039	4.955018447	0.145685294	2.327142838	47833.66791	1.764288193	5.688709523
95 plus	0.277011035	3.642295241	17546.38456	0.662705045	3.642295241	0.245210045	4.167116505	24316.32282	1.076117301	4.167116505

Table 15: Caribbean 2017 life table, by age and sex. mx=mortality rate, ax=mean person-years lived in an age interval among those who die in that age interval, lx=number of persons left alive at age x, nLx=person-years lived between age x and x+n, ex=life expectancy at age x.										
Age Group	Male					Female				
	mx	ax	lx	nLx	ex	mx	ax	lx	nLx	ex
Early Neonatal	0.761489603	0.009565702	100000	0.01903873	70.19850231	0.592922193	0.009570868	100000	0.01906946	75.1889951
Late Neonatal	0.072971194	0.028746994	98550.31239	0.056581339	71.21173636	0.080174156	0.028745007	98869.38337	0.056752776	76.02948807
Post Neonatal	0.0136025	0.460677539	98137.4904	0.900426113	71.45366357	0.012327055	0.460768144	98414.42901	0.903497441	76.32332873
1 to 4	0.002108272	1.997188974	96912.93344	3.860222411	71.42740352	0.001410289	1.998119615	97300.82602	3.88107803	76.26819821
5 to 9	0.000540634	2.498873679	96099.34083	4.798479154	68.01539152	0.000474509	2.499011439	96753.59349	4.831945826	72.68833967
10 to 14	0.00051702	2.712084254	95839.94258	4.786335441	63.19271385	0.000399676	2.579912971	96524.33044	4.821553481	67.85503323
15 to 19	0.001072162	2.748399253	95592.4902	4.768114285	58.34925221	0.000630787	2.662952039	96331.63331	4.8094927	62.98558513
20 to 24	0.001810777	2.621662099	95081.27383	4.733677319	53.648095	0.000898399	2.628289869	96028.26454	4.791204939	58.17607576
25 to 29	0.002158248	2.576787708	94224.13278	4.686697237	49.11211437	0.001192654	2.623166086	95597.84543	4.766381526	53.42606498
30 to 34	0.002656362	2.584715092	93212.6827	4.630925533	44.61691919	0.001619137	2.627474158	95029.42534	4.733288911	48.72981597
35 to 39	0.00331356	2.585789472	91982.65392	4.562637642	40.17883382	0.002211156	2.605691404	94263.12274	4.688337385	44.10445511
40 to 44	0.004136268	2.603688637	90470.98754	4.4791583	35.8067854	0.00278974	2.605810743	93226.60232	4.630406101	39.56566091
45 to 49	0.00556362	2.635474409	88618.58832	4.373404671	31.50060502	0.003712958	2.631558672	91935.05541	4.556686412	35.08466171
50 to 54	0.008121706	2.644511548	86185.84804	4.22841608	27.31510438	0.005292993	2.645661707	90243.49909	4.456649056	30.69271953
55 to 59	0.011963481	2.643813327	82752.44294	4.024215268	23.33836944	0.007739727	2.661592376	87885.12836	4.316159976	26.44506741
60 to 64	0.018085009	2.613343416	77939.47584	3.735781472	19.61601225	0.01204031	2.634480924	84545.55451	4.110251966	22.38421109
65 to 69	0.025297173	2.601252336	71185.76746	3.355746263	16.22891132	0.017083405	2.624964103	79598.58972	3.824814717	18.61134891
70 to 74	0.037896943	2.576497314	62700.61346	2.871462051	13.073058	0.025756069	2.622229969	73067.80317	3.442681215	15.04003913
75 to 79	0.054646887	2.544758166	51825.26998	2.284935638	10.27600603	0.04006611	2.602487224	64206.48889	2.92917549	11.75390609
80 to 84	0.083317568	2.498162293	39348.93257	1.628353404	7.728319373	0.064590769	2.554809262	52480.22391	2.266402039	8.799286221
85 to 89	0.14351564	2.396584635	25794.91329	0.939188432	5.478569916	0.118548216	2.47810602	37855.22825	1.457482385	6.213168544
90 to 94	0.238817919	2.168785364	12328.52848	0.367884748	3.848483358	0.204705437	2.2627258	20594.14172	0.660156701	4.346656606
95 plus	0.332420521	3.008375468	3549.07427	0.106820778	3.008375468	0.301341624	3.318801872	7092.147907	0.235526571	3.318801872

Table 15: Caribbean 2100 life table, by age and sex. mx=mortality rate, ax=mean person-years lived in an age interval among those who die in that age interval, lx=number of persons left alive at age x, nLx=person-years lived between age x and x+n, ex=life expectancy at age x.

Age Group	Male					Female				
	mx	ax	lx	nLx	ex	mx	ax	lx	nLx	ex
Early Neonatal	0.174447141	0.009583694	100000	0.019146043	77.5361082	0.152980226	0.009584352	100000	0.019149981	82.39607014
Late Neonatal	0.013373398	0.028763434	99666.09439	0.057320085	77.77596864	0.02171701	0.028761133	99707.1025	0.057329916	82.61836436
Post Neonatal	0.002187554	0.461488435	99589.45146	0.918569604	77.77791598	0.002132667	0.461492335	99582.63759	0.918530152	82.66359656
1 to 4	0.000384473	1.999487371	99388.61742	3.972502696	77.00897018	0.000271153	1.999638466	99386.88647	3.973333974	81.90028961
5 to 9	0.000226247	2.499528656	99236.74477	4.959071581	73.11530236	0.000230008	2.499520821	99280.00165	4.961193889	77.9803167
10 to 14	0.000319438	2.858601292	99126.33063	4.952919186	68.18640432	0.000253435	2.656739261	99167.98848	4.955459413	73.06037397
15 to 19	0.000671229	2.754425117	98973.11024	4.941346221	63.2776417	0.000350643	2.642197931	99045.56528	4.948217155	68.1434082
20 to 24	0.000977084	2.636584037	98654.77753	4.921639246	58.46285515	0.000437064	2.624953139	98875.67901	4.938723407	63.25339489
25 to 29	0.001202189	2.59887603	98190.02447	4.895775762	53.72042331	0.000584304	2.625481841	98663.95303	4.926465077	58.38162854
30 to 34	0.001452612	2.580217771	97622.47775	4.864438571	49.01427638	0.000742908	2.620350368	98382.41572	4.910527018	53.53977621
35 to 39	0.001731619	2.599001957	96935.3854	4.827400516	44.34214814	0.000953062	2.635780967	98023.87771	4.890304536	48.72519319
40 to 44	0.002217633	2.645796653	96131.68244	4.782117802	39.69277449	0.001335342	2.679178273	97564.89277	4.863329868	43.94116837
45 to 49	0.003191037	2.691470099	95097.15657	4.720711393	35.09677768	0.002045662	2.686126161	96925.42337	4.823665222	39.21297738
50 to 54	0.005144826	2.687159031	93622.97682	4.627007378	30.60873297	0.003124219	2.661727321	95951.89545	4.763109803	34.58357195
55 to 59	0.007935719	2.669179186	91288.58283	4.482606285	26.32519262	0.004482781	2.655907173	94480.94552	4.675326851	30.08034131
60 to 64	0.011953772	2.64449389	87789.57763	4.270463469	22.27157938	0.006546738	2.653267475	92408.80365	4.551084183	25.6954817
65 to 69	0.017699028	2.637434605	82742.0925	3.972823508	18.4691259	0.009668153	2.671460024	89457.95538	4.375069109	21.45470584
70 to 74	0.02697604	2.614240939	75800.66359	3.563485264	14.92173809	0.015419189	2.696812802	85266.51374	4.118229611	17.37744862
75 to 79	0.040612306	2.598340488	66310.96786	3.025344375	11.68409905	0.02731762	2.676221681	78976.12269	3.71522821	13.54437181
80 to 84	0.066065776	2.544778991	54236.1897	2.341403983	8.712243773	0.047745375	2.612449283	68946.10523	3.100117718	10.12219087
85 to 89	0.12127602	2.461941917	39147.08458	1.507823027	6.099080403	0.094171595	2.564528975	54412.90825	2.223174404	7.1208417
90 to 94	0.215481632	2.207299833	21420.61281	0.676005019	4.185017244	0.17577058	2.312631642	33998.93805	1.164454414	4.894111746
95 plus	0.313310418	3.20086697	7243.529597	0.236187187	3.20086697	0.274311828	3.662218476	14043.6014	0.524046232	3.662218476

Table 15: Antigua and Barbuda 2017 life table, by age and sex. mx=mortality rate, ax=mean person-years lived in an age interval among those who die in that age interval, lx=number of persons left alive at age x, nLx=person-years lived between age x and x+n, ex=life expectancy at age x.

Age Group	Male					Female				
	mx	ax	lx	nLx	ex	mx	ax	lx	nLx	ex
Early Neonatal	0.28108918	0.009580426	100000	0.019126486	74.97640118	0.15327877	0.009584343	100000	0.019149923	78.59134367
Late Neonatal	0.042628876	0.028755364	99462.42807	0.057154856	75.36225198	0.024401057	0.028760392	99706.49147	0.057325133	78.80336226
Post Neonatal	0.002937053	0.461435192	99218.84264	0.914834778	75.48964795	0.002843622	0.461441829	99566.62361	0.918080813	78.85642213
1 to 4	0.000835649	1.998885802	98950.24107	3.951405083	74.77004629	0.000800447	1.998932737	99305.60027	3.965873477	78.13910807
5 to 9	0.000401836	2.499162842	98620.20485	4.926061002	71.01366505	0.000177864	2.49962945	98988.24949	4.947212661	74.38315516
10 to 14	0.000342229	2.846919627	98422.3019	4.917490746	66.15145793	0.000183372	2.765509507	98900.27008	4.942987562	69.44709635
15 to 19	0.00097404	2.72853366	98254.03312	4.901856876	61.25987398	0.000411567	2.638877481	98809.63607	4.935685775	64.50825793
20 to 24	0.001420597	2.532406601	97776.59255	4.871752211	56.54560496	0.000459231	2.550213316	98606.50084	4.924785781	59.63569766
25 to 29	0.001208803	2.532506713	97084.53126	4.839791163	51.93051967	0.000525651	2.590352516	98380.33754	4.912795515	54.7669108
30 to 34	0.001629768	2.600662097	96499.51509	4.806182912	47.22990186	0.000691885	2.643423028	98122.08806	4.898118473	49.90421527
35 to 39	0.002023615	2.540457288	95716.25169	4.762112499	42.59499733	0.001008345	2.680266001	97783.18497	4.877747139	45.06799961
40 to 44	0.002058879	2.612626678	94752.63927	4.714460936	38.00219573	0.001574631	2.698378375	97291.34452	4.846995453	40.28228551
45 to 49	0.003197804	2.701598013	93782.06765	4.654894027	33.36830634	0.00253336	2.701685427	96528.16855	4.798467372	35.57945281
50 to 54	0.005286594	2.755491747	92293.68907	4.560575852	28.86264666	0.004104519	2.691369458	95312.66769	4.720903291	30.99872768
55 to 59	0.010124887	2.653343558	89883.06531	4.389870763	24.56253627	0.006512406	2.68338337	93375.18826	4.599378898	26.58599846
60 to 64	0.013736532	2.607620553	85439.33022	4.136079463	20.70162586	0.010365347	2.666565071	90380.37929	4.412316465	22.37788038
65 to 69	0.019346943	2.643046932	79759.48663	3.814118981	16.98978055	0.016083669	2.655293917	85807.83954	4.134508301	18.42810361
70 to 74	0.031925336	2.650736774	72383.41158	3.366799447	13.45147108	0.025522314	2.642009511	79159.75339	3.733377287	14.75263525
75 to 79	0.05585945	2.565983036	61641.23548	2.71344346	10.33364099	0.041486729	2.617810268	69634.34425	3.168699589	11.40905186
80 to 84	0.080431391	2.480676945	46497.19054	1.933513166	7.864345092	0.07038909	2.560770776	56494.33177	2.411085458	8.453328617
85 to 89	0.139812565	2.406860384	30963.2657	1.136584436	5.567481655	0.126432899	2.453522031	39537.23227	1.495854396	5.980507404
90 to 94	0.235115884	2.175877195	15089.98531	0.45360332	3.896155586	0.213419489	2.244214349	20647.63267	0.65038736	4.210108651
95 plus	0.329440022	3.03563438	4434.326351	0.13468872	3.03563438	0.309283899	3.23383213	6784.263042	0.219607548	3.23383213

Table 15: Antigua and Barbuda 2100 life table, by age and sex. mx=mortality rate, ax=mean person-years lived in an age interval among those who die in that age interval, lx=number of persons left alive at age x, nLx=person-years lived between age x and x+n, ex=life expectancy at age x.

Age Group	Male					Female				
	mx	ax	lx	nLx	ex	mx	ax	lx	nLx	ex
Early Neonatal	0.060663853	0.009587182	100000	0.019166932	80.7591101	0.034001876	0.009587999	100000	0.019171831	83.26021133
Late Neonatal	0.01483589	0.028763031	99883.74885	0.057442847	80.83382168	0.007712459	0.028764996	99934.8219	0.057483993	83.29525627
Post Neonatal	0.000976466	0.461574469	99798.54284	0.921012454	80.84524524	0.001030644	0.46157062	99890.49271	0.921837994	83.27466635
1 to 4	0.000275677	1.999632431	99708.62153	3.98614736	79.99438392	0.000259225	1.999654366	99795.50118	3.989752211	82.43009786
5 to 9	0.000125436	2.499738676	99598.77177	4.978377437	76.08033421	7.67757E-05	2.499840051	99692.13441	4.983650286	78.51336846
10 to 14	0.00012677	3.201788034	99536.33279	4.975655028	71.12646268	8.58066E-05	2.854414484	99653.87986	4.981772158	73.54251938
15 to 19	0.000526835	2.765479146	99473.26572	4.96780931	66.16952695	0.000218323	2.624679513	99611.13912	4.977975182	68.57283119
20 to 24	0.000794529	2.531033739	99211.59548	4.950853384	61.33624743	0.000216124	2.542192571	99502.47642	4.972484792	63.64474613
25 to 29	0.000631463	2.595858433	98818.39388	4.933443702	56.56962381	0.000264297	2.611632773	99395.03462	4.966611436	58.7106784
30 to 34	0.001104058	2.591219336	98506.97153	4.912301763	51.73995214	0.000352753	2.63302161	99263.80436	4.959034515	53.78468503
35 to 39	0.001128456	2.461172695	97965.17776	4.884271603	47.01077523	0.000474695	2.684555486	99088.94103	4.94899126	48.87469322
40 to 44	0.000893345	2.593091699	97414.70089	4.860283223	42.26177369	0.000758907	2.730818927	98854.08819	4.934181691	43.9841427
45 to 49	0.001527456	2.742865413	96981.08154	4.832342136	37.43862611	0.001295488	2.737961085	98479.77063	4.909578083	39.14063306
50 to 54	0.00262828	2.823113807	96244.04659	4.784796595	32.70356418	0.002233207	2.715618244	97844.01206	4.867344117	34.37663275
55 to 59	0.005676419	2.676060732	94987.73958	4.687576989	28.09797619	0.003632539	2.681012747	96757.60716	4.79744292	29.73126633
60 to 64	0.00769393	2.622637189	92329.77318	4.533686576	23.82846316	0.005492273	2.693523376	95016.1033	4.691400798	25.22579889
65 to 69	0.010896572	2.691927911	88846.66458	4.333517994	19.65780662	0.009117017	2.709538286	92441.62225	4.527572148	20.85122094
70 to 74	0.019362277	2.745420532	84136.39463	4.031230738	15.60539995	0.015671944	2.73103287	88320.4418	4.264637426	16.6946032
75 to 79	0.039524223	2.635079593	76361.35929	3.4931866	11.91179227	0.030136834	2.696289918	81656.09823	3.818827756	12.82878549
80 to 84	0.061469821	2.529298805	62633.38225	2.722227635	8.942791525	0.055767433	2.61173931	70227.4337	3.102700006	9.470711161
85 to 89	0.115399571	2.47858253	46065.67709	1.789620818	6.247181182	0.106021572	2.521980044	53128.15019	2.111675745	6.666110717
90 to 94	0.209518912	2.218334593	25691.91598	0.815545835	4.264585258	0.189979351	2.289315066	31155.5467	1.035643553	4.618265068
95 plus	0.308482102	3.246362745	8814.299644	0.288613145	3.246362745	0.28761677	3.488977731	11866.29751	0.420441426	3.488977731

Table 15: The Bahamas 2017 life table, by age and sex. mx=mortality rate, ax=mean person-years lived in an age interval among those who die in that age interval, lx=number of persons left alive at age x, nLx=person-years lived between age x and x+n, ex=life expectancy at age x.

Age Group	Male					Female				
	mx	ax	lx	nLx	ex	mx	ax	lx	nLx	ex
Early Neonatal	0.258959924	0.009581104	100000	0.01913054	70.6268932	0.23540464	0.009581826	100000	0.019134857	76.45323346
Late Neonatal	0.032797711	0.028758076	99504.62087	0.057195267	70.95922042	0.032493273	0.02875816	99549.57159	0.057221601	76.77988607
Post Neonatal	0.004853875	0.461299025	99317.08629	0.914931148	71.03560317	0.003692895	0.461381499	99363.67632	0.915850541	76.865926
1 to 4	0.000678135	1.999095821	98873.0853	3.949566092	70.42928925	0.000404817	1.999460245	99025.50836	3.957815754	76.20357239
5 to 9	0.000316039	2.499341585	98605.34363	4.926374614	66.61516081	0.000259816	2.499458716	98865.32343	4.940057043	72.32381936
10 to 14	0.000374206	3.03806043	98449.68224	4.918871404	61.71655638	0.000264791	2.61264372	98736.98621	4.933730665	67.41457942
15 to 19	0.001286061	2.843778621	98265.63689	4.899695418	56.82644762	0.000403991	2.754485773	98606.35237	4.925849368	62.50040545
20 to 24	0.002521914	2.645310905	97635.5312	4.852958674	52.17465346	0.000761008	2.764659156	98407.35883	4.912012565	57.62116235
25 to 29	0.003100756	2.567781773	96411.7025	4.784503674	47.80301667	0.001379401	2.689755188	98033.56283	4.886108119	52.83021478
30 to 34	0.003627214	2.551822473	94928.23774	4.704639288	43.50954278	0.00203906	2.622938495	97359.59793	4.844500877	48.17709195
35 to 39	0.004135979	2.56697622	93221.92857	4.614667507	39.25881116	0.002625912	2.579525622	96371.83393	4.78816112	43.64369226
40 to 44	0.005145645	2.616318586	91313.60143	4.510370222	35.02514734	0.003109442	2.5856102	95114.63036	4.720297699	39.18607374
45 to 49	0.00732848	2.612452492	88993.24905	4.373167813	30.86962443	0.00400949	2.627371414	93647.10035	4.638238158	34.75924549
50 to 54	0.009661448	2.607354918	85789.42131	4.192592021	26.92427241	0.005751369	2.632587959	91787.76986	4.527754087	30.40972397
55 to 59	0.013363598	2.586980896	81740.56323	3.959417435	23.1281703	0.008037744	2.618593042	89184.36655	4.375493876	26.2200798
60 to 64	0.017037472	2.612189331	76452.37977	3.673281	19.54850212	0.011029703	2.66009788	85668.71135	4.175714562	22.18805112
65 to 69	0.026222724	2.606198177	70198.46913	3.302783993	16.05705345	0.018155598	2.638969995	81065.18258	3.886757727	18.2963999
70 to 74	0.037866108	2.578133512	61545.59234	2.819064008	12.9481591	0.026781078	2.61113767	74013.26468	3.478351105	14.78751406
75 to 79	0.05671082	2.545747182	50884.10259	2.233804149	10.1217004	0.040816662	2.605927869	64707.39394	2.947759581	11.53819241
80 to 84	0.08549624	2.490074589	38236.84097	1.574620593	7.629824936	0.068335778	2.561795766	52694.84922	2.259157468	8.574919931
85 to 89	0.146096543	2.389538051	24800.68239	0.898173932	5.418849656	0.123661915	2.462122217	37291.29731	1.420061921	6.061667135
90 to 94	0.24137041	2.163783339	11702.64226	0.347570485	3.816503111	0.210363389	2.250731354	19774.02132	0.626991927	4.2577144
95 plus	0.334466518	2.990085831	3325.034454	0.099513922	2.990085831	0.306500955	3.263448676	6614.032901	0.216212837	3.263448676

Table 15: The Bahamas 2100 life table, by age and sex. mx=mortality rate, ax=mean person-years lived in an age interval among those who die in that age interval, lx=number of persons left alive at age x, nLx=person-years lived between age x and x+n, ex=life expectancy at age x.

Age Group	Male					Female				
	mx	ax	lx	nLx	ex	mx	ax	lx	nLx	ex
Early Neonatal	0.076978757	0.009586682	100000	0.019163935	78.08424688	0.084474708	0.009586452	100000	0.01916256	81.60693562
Late Neonatal	0.010738461	0.028764161	99852.51047	0.057431649	78.18029167	0.015010991	0.028762983	99838.18643	0.057416356	81.71963775
Post Neonatal	0.00174505	0.46151987	99790.85018	0.920614922	78.17087854	0.001407355	0.461543859	99752.01989	0.920400081	81.73255181
1 to 4	0.00020604	1.99972528	99630.23682	3.983568355	77.37282072	0.000128044	1.999829274	99622.51043	3.983880353	80.91471209
5 to 9	7.80362E-05	2.499837425	99548.19431	4.976439325	73.43492539	0.000114805	2.499760822	99571.51201	4.977147309	76.95506872
10 to 14	0.00015522	3.15839525	99509.38393	4.973946383	68.4627308	0.000140527	2.735738409	99514.38682	4.974075236	71.99769487
15 to 19	0.000532217	2.848421073	99432.21909	4.965845503	63.51362484	0.000214168	2.756542206	99444.55363	4.969839952	67.04605661
20 to 24	0.001063801	2.663756042	99168.12744	4.946054563	58.67210161	0.000416876	2.784897076	99338.14367	4.962390783	62.11481414
25 to 29	0.001363935	2.646312147	98643.02012	4.916276939	53.96536518	0.000868387	2.806971164	99131.58283	4.946779532	57.23732086
30 to 34	0.001954706	2.589217291	97975.37922	4.875717881	49.30944585	0.001321605	2.679359637	98704.27582	4.919842467	52.47083828
35 to 39	0.00206022	2.564698403	97027.72013	4.827323664	44.76018868	0.001651922	2.571157016	98057.99404	4.883270814	47.79669642
40 to 44	0.00272284	2.625001904	96039.11486	4.771212358	40.18929532	0.001780524	2.588485367	97253.85211	4.841864259	43.16821464
45 to 49	0.003679162	2.644043319	94752.68262	4.696962023	35.69215456	0.002346812	2.640987619	96394.26506	4.79316793	38.52855152
50 to 54	0.005115074	2.641713572	93042.12637	4.596871406	31.2944141	0.003356526	2.645718856	95272.65611	4.726301314	33.94909491
55 to 59	0.00721414	2.613952945	90715.39845	4.459531319	27.02520646	0.004732279	2.627488145	93691.54282	4.632617333	29.47499183
60 to 64	0.009546054	2.653656929	87532.11986	4.281521768	22.91055371	0.006351998	2.681898987	91508.5833	4.509069486	25.1126538
65 to 69	0.015369237	2.657220956	83498.5394	4.031194065	18.88631104	0.010522445	2.685198754	88661.00928	4.327803107	20.82805902
70 to 74	0.023826049	2.648660067	77389.48929	3.666741137	15.16738953	0.016515866	2.691558249	84138.82373	4.05327401	16.79836569
75 to 79	0.039809023	2.62360768	68796.90172	3.1479653	11.73146374	0.028897791	2.704405923	77512.60803	3.636663244	12.99611281
80 to 84	0.067402643	2.555860183	56554.84379	2.439413974	8.707522739	0.055772283	2.613121784	67168.68565	2.972382719	9.575455552
85 to 89	0.122558392	2.459639753	40670.24432	1.568520074	6.099263777	0.10543758	2.527213046	51020.29465	2.040597248	6.747407642
90 to 94	0.216369362	2.203169267	22354.64916	0.708649044	4.18680489	0.188660023	2.287939876	30404.91889	1.022029586	4.671495976
95 plus	0.313913567	3.201906758	7676.851267	0.253281295	3.201906758	0.286197041	3.523059875	11979.00754	0.436959942	3.523059875

Table 15: Barbados 2017 life table, by age and sex. mx=mortality rate, ax=mean person-years lived in an age interval among those who die in that age interval, lx=number of persons left alive at age x, nLx=person-years lived between age x and x+n, ex=life expectancy at age x.

Age Group	Male					Female				
	mx	ax	lx	nLx	ex	mx	ax	lx	nLx	ex
Early Neonatal	0.393652058	0.009576976	100000	0.019105879	75.4986946	0.291302926	0.009580113	100000	0.019124615	78.79198314
Late Neonatal	0.029109072	0.028759094	99248.01362	0.057053824	76.05142562	0.035881387	0.028757225	99442.9455	0.057154746	79.21403875
Post Neonatal	0.002682038	0.461453308	99081.99433	0.913680581	76.12130971	0.00316466	0.461419023	99237.91088	0.914914481	79.32011797
1 to 4	0.000237866	1.999682845	98837.04979	3.951602773	75.38561432	0.00026939	1.999640814	98948.45751	3.955807373	78.62756607
5 to 9	0.000157746	2.499671362	98743.10445	4.935209141	71.4554794	0.000201942	2.499579288	98841.93094	4.939602681	74.71018128
10 to 14	0.000360258	2.870145039	98665.27148	4.929480601	66.50989141	0.000478128	2.58633371	98742.19314	4.931418984	69.78312922
15 to 19	0.000799175	2.642454318	98487.70591	4.915125517	61.62462628	0.000401252	2.675613133	98506.42445	4.92073254	64.94393596
20 to 24	0.000912061	2.659393202	98094.91328	4.894297294	56.86069627	0.000820442	2.586221363	98308.98521	4.905734712	60.06894655
25 to 29	0.001509152	2.619536423	97648.53	4.864949554	52.10834127	0.000745195	2.572960448	97906.51315	4.886488434	55.30514398
30 to 34	0.001798267	2.560931651	96914.35537	4.824558256	47.48305787	0.001089671	2.646641162	97542.39229	4.864646016	50.50189547
35 to 39	0.002068213	2.548705627	96046.81078	4.778118897	42.88860658	0.001525983	2.612019715	97012.33729	4.833006576	45.76323198
40 to 44	0.002324583	2.708333233	95058.66702	4.727750757	38.30769258	0.001933746	2.65512371	96274.88384	4.792016324	41.09356178
45 to 49	0.00449027	2.643180123	93959.78532	4.648799779	33.72376599	0.003014429	2.642565626	95348.32605	4.733780035	36.46686984
50 to 54	0.005587925	2.642123261	91872.70625	4.533910453	29.4293609	0.004090919	2.637349817	93921.55589	4.651129782	31.98040526
55 to 59	0.008715182	2.650918201	89339.82926	4.377400356	25.18833558	0.00590774	2.660903752	92019.16456	4.538258512	27.58670019
60 to 64	0.012754803	2.648973132	85526.15878	4.151858017	21.19274622	0.009084209	2.645503628	89338.74435	4.373424443	23.33413949
65 to 69	0.019835087	2.635586441	80232.96736	3.8320405	17.41560245	0.013177388	2.651237273	85367.17409	4.14027395	19.29616628
70 to 74	0.030250945	2.598026314	72636.96238	3.386031792	13.96077986	0.020811639	2.688106368	79914.0425	3.812392505	15.43148507
75 to 79	0.043993506	2.610219523	62403.48774	2.823683339	10.82412876	0.038214703	2.644150889	71985.57508	3.302273493	11.83464414
80 to 84	0.080515122	2.549776387	49997.624	2.088535184	7.863184487	0.064842489	2.600820229	59379.4521	2.569746946	8.785959217
85 to 89	0.1404631	2.405075591	33209.48779	1.217507432	5.552279185	0.119147633	2.476248278	42740.03418	1.643588395	6.19568162
90 to 94	0.235761049	2.174618298	16136.72647	0.484554499	3.888014406	0.205365758	2.261310887	23187.62358	0.742456654	4.336408719
95 plus	0.329957798	3.030978904	4728.152313	0.143440176	3.030978904	0.301942761	3.312427892	7961.758844	0.264010946	3.312427892

Table 15: Barbados 2100 life table, by age and sex. mx=mortality rate, ax=mean person-years lived in an age interval among those who die in that age interval, lx=number of persons left alive at age x, nLx=person-years lived between age x and x+n, ex=life expectancy at age x.

Age Group	Male					Female				
	mx	ax	lx	nLx	ex	mx	ax	lx	nLx	ex
Early Neonatal	0.116846564	0.00958546	100000	0.019156618	80.9883734	0.086776823	0.009586381	100000	0.019162135	83.31905477
Late Neonatal	0.009059775	0.028764624	99776.27607	0.057390573	81.15061273	0.017744259	0.028762229	99833.75785	0.057409301	83.4383337
Post Neonatal	0.000976482	0.461574468	99724.29448	0.92032727	81.13530192	0.001234354	0.461556149	99731.92931	0.920288211	83.46593233
1 to 4	7.01061E-05	1.999906525	99634.44801	3.984819322	80.28467308	7.59416E-05	1.999898745	99618.36214	3.984129472	82.63717025
5 to 9	6.31401E-05	2.499868458	99606.51986	4.979540162	76.30659288	0.000115121	2.499760166	99588.11356	4.977974951	78.66163774
10 to 14	0.000196398	2.964015769	99575.08963	4.976645576	71.32982396	0.000372047	2.726234391	99530.92514	4.972031177	73.70532805
15 to 19	0.000389939	2.643602731	99477.40388	4.969238679	66.39689013	0.000206924	2.69641798	99346.44959	4.96492903	68.83726908
20 to 24	0.000401437	2.693937148	99283.79692	4.959609434	61.51990207	0.000495329	2.611054541	99243.76095	4.956309556	63.905723
25 to 29	0.000777747	2.619472507	99084.8138	4.945248435	56.63735037	0.000461481	2.602936519	98998.39573	4.944447964	59.05748916
30 to 34	0.001008151	2.588802845	98700.90889	4.923103885	51.84471729	0.000725181	2.618631648	98770.34572	4.929979485	54.18765522
35 to 39	0.001179716	2.551892191	98207.72077	4.896179078	47.08816552	0.00085347	2.589379159	98413.14628	4.910532257	49.3743555
40 to 44	0.001130643	2.807681039	97634.99563	4.86933133	42.34658374	0.001073417	2.668784944	97994.46686	4.887512633	44.57327493
45 to 49	0.002455989	2.692402788	97087.74693	4.827035014	37.56763276	0.001770005	2.665728528	97470.24611	4.853336318	39.79794043
50 to 54	0.0033665	2.679847738	95906.83619	4.758126538	32.99441787	0.002372322	2.657600814	96613.05348	4.803905577	35.1255196
55 to 59	0.005289293	2.65519539	94312.73709	4.658018865	28.50346287	0.003582711	2.652894054	95475.13895	4.733964408	30.51107283
60 to 64	0.007586941	2.695344754	91858.541	4.514324977	24.19095698	0.005147065	2.644471984	93782.39236	4.633086367	26.01203261
65 to 69	0.013017806	2.673148427	88459.14438	4.293503997	20.0105432	0.007543759	2.673329165	91403.9964	4.491478671	21.61710148
70 to 74	0.019719894	2.618270293	82921.51883	3.961711407	16.16304584	0.0120345	2.800306036	88030.97598	4.287873318	17.33971225
75 to 79	0.028874876	2.7183726	75183.00577	3.53026284	12.55078321	0.026910775	2.735959278	82907.57403	3.908179713	13.23639272
80 to 84	0.061958082	2.623902103	65250.01746	2.854039888	9.044352235	0.052453138	2.68362285	72475.47536	3.234698269	9.746242232
85 to 89	0.115584727	2.480396537	48112.71513	1.882471858	6.307885525	0.101359654	2.538890646	55725.06265	2.239439936	6.847890462
90 to 94	0.208858667	2.213755648	27353.43937	0.879252963	4.301585532	0.184359022	2.298399724	33530.0722	1.128745414	4.728662644
95 plus	0.307724967	3.267634632	9772.129113	0.329034692	3.267634632	0.282343938	3.558338876	13224.56286	0.479640607	3.558338876

Table 15: Belize 2017 life table, by age and sex. mx=mortality rate, ax=mean person-years lived in an age interval among those who die in that age interval, lx=number of persons left alive at age x, nLx=person-years lived between age x and x+n, ex=life expectancy at age x.

Age Group	Male					Female				
	mx	ax	lx	nLx	ex	mx	ax	lx	nLx	ex
Early Neonatal	0.385223187	0.009577234	100000	0.019107416	70.83697832	0.422934476	0.009576078	100000	0.019100519	77.08581992
Late Neonatal	0.066300271	0.028748834	99263.96866	0.057002004	71.34289736	0.047228865	0.028754095	99192.2447	0.05699206	77.69417204
Post Neonatal	0.007234175	0.461129932	98886.10508	0.90996198	71.55777735	0.004184593	0.461346569	98923.13704	0.911583496	77.84792507
1 to 4	0.000809125	1.998921167	98228.08638	3.922774501	71.11082581	0.000656845	1.999124206	98541.7885	3.936499584	77.22417546
5 to 9	0.000342031	2.499287436	97910.81318	4.891357887	67.33485786	0.00025829	2.499461897	98283.30602	4.910993993	73.42207568
10 to 14	0.000404463	3.064259162	97743.55017	4.883352996	62.44583092	0.000248361	2.724874488	98156.4811	4.905052232	68.51372783
15 to 19	0.001442689	2.769577482	97546.06149	4.861659661	57.56606486	0.000527199	2.690231609	98034.66881	4.895772026	63.59547668
20 to 24	0.002294199	2.670568409	96844.70039	4.816495076	52.96288014	0.000732516	2.69279645	97776.57162	4.880580387	58.75623705
25 to 29	0.003382058	2.612228561	95739.71693	4.748638407	48.54327394	0.00121266	2.615565722	97419.07189	4.856910217	53.9619589
30 to 34	0.004229401	2.537790677	94133.72738	4.658178851	44.3268172	0.001418146	2.557830837	96830.11114	4.824796132	49.27424256
35 to 39	0.004306238	2.527075469	92163.64023	4.559628164	40.22001285	0.00162663	2.605690546	96145.90376	4.788645345	44.60666084
40 to 44	0.004994343	2.57072127	90200.21376	4.455950532	36.04040337	0.002275698	2.668993945	95366.97851	4.743188088	39.94966225
45 to 49	0.006274954	2.586224508	87974.85578	4.333115677	31.88694266	0.003538053	2.680461393	94287.5928	4.676005965	35.37638584
50 to 54	0.008007873	2.658447694	85256.00859	4.18434573	27.82126117	0.005493901	2.663454858	92633.24089	4.572961947	30.96023142
55 to 59	0.013266549	2.627377152	81905.49809	3.970314445	23.85050835	0.008202271	2.627654861	90120.99613	4.42004605	26.74894441
60 to 64	0.017893038	2.570702257	76638.78039	3.672330632	20.3088991	0.011236913	2.630792464	86495.75417	4.212644394	22.75980769
65 to 69	0.022573172	2.569660903	70068.67781	3.321256582	16.9721033	0.016778435	2.633955391	81762.40885	3.932041306	18.92498678
70 to 74	0.031201853	2.584904972	62572.7551	2.90944082	13.69746455	0.025329289	2.60946984	75165.81399	3.543750712	15.3545783
75 to 79	0.046525051	2.604926946	53496.68796	2.406723562	10.58289907	0.037237959	2.602477015	66191.31588	3.038369092	12.0825501
80 to 84	0.084046449	2.526319631	42302.48824	1.751167201	7.694351952	0.060891503	2.551621621	54879.9377	2.388083451	9.036604463
85 to 89	0.144662253	2.393407168	27589.22574	1.001824321	5.451136903	0.113429071	2.494664011	40343.31843	1.57091329	6.373708315
90 to 94	0.239962975	2.166585461	13101.06382	0.389975089	3.83377798	0.198947896	2.274521325	22530.89486	0.730555193	4.441367001
95 plus	0.33334203	2.999966997	3745.335837	0.112376788	2.999966997	0.296056555	3.377855775	8001.24955	0.270334437	3.377855775

Table 15: Belize 2100 life table, by age and sex. mx=mortality rate, ax=mean person-years lived in an age interval among those who die in that age interval, lx=number of persons left alive at age x, nLx=person-years lived between age x and x+n, ex=life expectancy at age x.

Age Group	Male					Female				
	mx	ax	lx	nLx	ex	mx	ax	lx	nLx	ex
Early Neonatal	0.083680349	0.009586476	100000	0.019162702	80.08827124	0.11732458	0.009585445	100000	0.019156525	83.61521437
Late Neonatal	0.023807542	0.028760556	99839.65877	0.057402681	80.19748726	0.020193037	0.028761553	99775.28435	0.057371632	83.78387518
Post Neonatal	0.001624602	0.461528427	99703.03691	0.919855956	80.24961406	0.0009234	0.461578239	99659.46292	0.919751473	83.82361346
1 to 4	0.000197184	1.999737088	99553.63766	3.98057677	79.4456891	0.000173329	1.999768894	99574.54949	3.981602851	82.97114804
5 to 9	0.000102539	2.499786377	99475.22653	4.972490837	75.5058444	0.000116869	2.499756523	99505.61763	4.973831744	79.026529
10 to 14	0.000175071	3.261066093	99424.43273	4.969538802	70.54202349	0.00012944	2.957048017	99447.67443	4.970997353	74.07045423
15 to 19	0.000588848	2.777844445	99337.90445	4.960360036	65.59947565	0.000325462	2.669979749	99383.63936	4.965387462	69.11577819
20 to 24	0.000933635	2.760969987	99046.82864	4.941783444	60.78030663	0.00035848	2.733616861	99222.44712	4.957104393	64.22299796
25 to 29	0.001533318	2.65419718	98587.69088	4.911769892	56.044319	0.000737863	2.612180857	99045.22033	4.943586489	59.33230492
30 to 34	0.002115187	2.509031876	97838.15327	4.866799883	51.44629919	0.000767348	2.540659889	98681.82794	4.924760602	54.5387575
35 to 39	0.002031713	2.481723547	96817.72843	4.817223166	46.95124245	0.000806153	2.595210813	98306.06075	4.905772843	49.73505306
40 to 44	0.002425638	2.669213826	95862.63148	4.765785472	42.3775214	0.001076885	2.75690491	97912.64293	4.883706605	44.92183087
45 to 49	0.002920818	2.668997499	94735.7738	4.704745934	37.84112104	0.001970113	2.717340638	97389.21417	4.847542946	40.1462236
50 to 54	0.004417061	2.71093149	93381.07995	4.622771602	33.34557018	0.002958138	2.694708276	96438.7462	4.78923783	35.51167955
55 to 59	0.007726925	2.630110676	91363.81157	4.487044969	29.01244453	0.004678277	2.623662311	95027.79892	4.699400004	30.99489578
60 to 64	0.0095504	2.572410866	87956.15857	4.299882748	25.01865752	0.006023323	2.622863884	92838.85079	4.577019134	26.65889951
65 to 69	0.011848589	2.595293981	83932.29706	4.082808811	21.07675359	0.008649022	2.673381531	90110.68824	4.417088292	22.37556201
70 to 74	0.016591773	2.631881227	79213.7885	3.816393332	17.16623842	0.013472041	2.71542198	86339.47347	4.189202775	18.22987029
75 to 79	0.026087839	2.730649517	73141.26906	3.462000226	13.35549949	0.023876942	2.68700659	80796.51556	3.831005878	14.2901199
80 to 84	0.053583817	2.606458151	64674.02784	2.890194258	9.745692113	0.040989047	2.581029823	71815.09618	3.273485756	10.74231583
85 to 89	0.102964624	2.521036015	50362.20855	2.043772478	6.787404726	0.08377147	2.60381387	58697.21462	2.455474913	7.565622124
90 to 94	0.1939322	2.224452983	31218.33882	1.044289681	4.573639593	0.162823703	2.330170853	38705.86851	1.360794766	5.168743935
95 plus	0.295124815	3.42359609	12531.51513	0.451617558	3.42359609	0.262072499	3.835316384	17163.28266	0.671932104	3.835316384

Table 15: Bermuda 2017 life table, by age and sex. mx=mortality rate, ax=mean person-years lived in an age interval among those who die in that age interval, lx=number of persons left alive at age x, nLx=person-years lived between age x and x+n, ex=life expectancy at age x.

Age Group	Male					Female				
	mx	ax	lx	nLx	ex	mx	ax	lx	nLx	ex
Early Neonatal	0.139391034	0.009584769	100000	0.019152471	76.99874314	0.079175799	0.009586614	100000	0.019163529	85.54070517
Late Neonatal	0.008435768	0.028764796	99733.03563	0.057366728	77.18560126	0.011567976	0.028763932	99848.27288	0.057427839	85.65147836
Post Neonatal	0.001266957	0.461553833	99684.64359	0.919837949	77.16551325	0.000960509	0.461575603	99781.84229	0.920865079	85.65094104
1 to 4	0.00040933	1.999454227	99568.11091	3.979465964	76.3319817	0.000347587	1.999536551	99693.39577	3.984965011	84.80322433
5 to 9	0.000143613	2.499700807	99405.2326	4.968477649	72.45375002	9.68608E-05	2.499798207	99554.88772	4.976539243	80.91842451
10 to 14	0.000150337	3.043893882	99333.88196	4.965234349	67.50399218	8.15085E-05	2.662166304	99506.68591	4.974386572	75.95641083
15 to 19	0.000537407	2.773435019	99259.23964	4.957029727	62.55245572	0.000160587	2.684815808	99466.14094	4.971458846	70.98628018
20 to 24	0.000857859	2.58204943	98992.86069	4.939399678	57.71327288	0.000224337	2.587941709	99386.30672	4.966627881	66.04112186
25 to 29	0.000882692	2.533208712	98569.15361	4.917749262	52.95018288	0.000255787	2.594284073	99274.88746	4.960691682	61.11230552
30 to 34	0.001005825	2.588111787	98135.10078	4.894878667	48.17311847	0.000340727	2.628421004	99148.00041	4.953397555	56.18716133
35 to 39	0.001317118	2.647030935	97642.80077	4.867056451	43.40290968	0.000467219	2.670236627	98979.2284	4.943580656	51.27844283
40 to 44	0.001955608	2.684657809	97001.80651	4.828229994	38.67218342	0.000725545	2.694731032	98748.26074	4.929168691	46.39207396
45 to 49	0.003098403	2.689322726	96057.69721	4.768746083	34.02584242	0.001151792	2.689380533	98390.64368	4.90647442	41.55080208
50 to 54	0.004888902	2.681499449	94580.35053	4.676019069	29.51529681	0.001788158	2.66899754	97825.55729	4.870975973	36.77514525
55 to 59	0.007645763	2.675443441	92294.6944	4.534157107	25.17986382	0.002638964	2.667564506	96954.63024	4.818078336	32.0813257
60 to 64	0.012039538	2.661110299	88828.73369	4.319812398	21.05800232	0.003994296	2.682332156	95683.32719	4.74029153	27.47188329
65 to 69	0.018690664	2.651005701	83628.80321	4.0056073	17.20181505	0.006335976	2.707675018	93790.28789	4.622396794	22.97188934
70 to 74	0.029886563	2.63938306	76143.33413	3.556331768	13.63212283	0.01085517	2.731195524	90862.43152	4.433963729	18.62440549
75 to 79	0.049895488	2.603538511	65517.54766	2.926127805	10.41466839	0.020091502	2.735313677	86051.56379	4.115434157	14.51236756
80 to 84	0.084983122	2.53060493	50923.41149	2.104744379	7.653062767	0.039711501	2.686982405	77789.06115	3.562540554	10.76273425
85 to 89	0.145838397	2.390221534	33047.78826	1.197121866	5.424409313	0.082556172	2.60592215	63657.2974	2.658200325	7.555588988
90 to 94	0.241120195	2.164294299	15602.3422	0.463460525	3.819472951	0.161955285	2.336491068	41743.0785	1.458794924	5.15611002
95 plus	0.334267632	2.991784973	4434.664475	0.132735951	2.991784973	0.261395104	3.826582471	18150.42956	0.695281846	3.826582471

Table 15: Bermuda 2100 life table, by age and sex. mx=mortality rate, ax=mean person-years lived in an age interval among those who die in that age interval, lx=number of persons left alive at age x, nLx=person-years lived between age x and x+n, ex=life expectancy at age x.

Age Group	Male					Female				
	mx	ax	lx	nLx	ex	mx	ax	lx	nLx	ex
Early Neonatal	0.047358895	0.00958759	100000	0.019169376	82.40010346	0.03107267	0.009588089	100000	0.019172369	89.02758301
Late Neonatal	0.00236425	0.028766471	99909.23061	0.057478114	82.45575809	0.005627957	0.028765571	99940.4316	0.057490667	89.06145253
Post Neonatal	0.000331431	0.461620291	99895.64218	0.922183057	82.40943313	0.000261551	0.461625256	99908.07833	0.922327607	89.03274667
1 to 4	0.00014088	1.99981216	99865.08153	3.993478039	81.51122317	9.44327E-05	1.99987409	99883.95637	3.994603823	88.13084048
5 to 9	4.72704E-05	2.499901522	99808.82657	4.989851672	77.55600365	4.98295E-05	2.499896188	99846.23756	4.991690049	84.16337474
10 to 14	5.76032E-05	3.421920963	99785.24274	4.988757514	72.57370773	4.06668E-05	2.641801638	99821.36567	4.990588039	79.18372164
15 to 19	0.000307036	2.77595243	99756.51134	4.984389309	67.59363478	7.59485E-05	2.726116516	99801.07094	4.989190588	74.19928555
20 to 24	0.000536106	2.578192297	99603.49045	4.973710816	62.69307432	0.000121869	2.584935151	99763.1797	4.986691411	69.2264324
25 to 29	0.000549126	2.529970916	99336.90102	4.960124753	57.85416732	0.000125801	2.540198314	99702.41054	4.983583707	64.26705374
30 to 34	0.000634235	2.540121361	99064.63061	4.945561638	53.00578341	0.000151283	2.594880269	99639.72474	4.980163665	59.30587045
35 to 39	0.000732748	2.556557957	98751.18319	4.928908718	48.16539031	0.000184431	2.674062795	99564.39954	4.976071604	54.3487424
40 to 44	0.001042306	2.674150184	98391.63289	4.907324632	43.32971638	0.000292097	2.756423164	99472.64618	4.970344758	49.39631289
45 to 49	0.001321483	2.691835213	97883.38169	4.879162087	38.53880537	0.000515157	2.729557871	99327.4997	4.960550499	44.46435221
50 to 54	0.002405734	2.686935255	97240.43198	4.835103793	33.77398551	0.000840066	2.691290396	99072.0131	4.944005371	39.57179333
55 to 59	0.004008653	2.660516411	96080.20698	4.75945026	29.14639206	0.001286673	2.677099025	98656.78088	4.918126378	34.72676692
60 to 64	0.006373915	2.656471913	94179.63858	4.639871288	24.67645241	0.001935056	2.7035413	98024.24396	4.879501293	29.93313651
65 to 69	0.01033041	2.668562008	91240.19318	4.455182705	20.37963134	0.003187145	2.75736701	97080.64833	4.819507933	25.19703474
70 to 74	0.018078502	2.6548681	86675.10471	4.158744987	16.30612862	0.005915707	2.830625856	95546.19804	4.716581023	20.55635387
75 to 79	0.030974304	2.641137835	79247.40109	3.69553223	12.57983844	0.012908619	2.84497955	92760.95105	4.512234832	16.08699613
80 to 84	0.058692007	2.577744833	67974.97708	2.984073915	9.226496063	0.029794278	2.777111781	86953.39866	4.07805513	11.96961928
85 to 89	0.11108465	2.493521957	50898.90327	2.00718272	6.432253673	0.066088293	2.675234231	74867.62871	3.248811395	8.438415232
90 to 94	0.204148957	2.22151532	29454.02379	0.952628394	4.369091356	0.139609991	2.351934712	53633.97041	1.965085494	5.720006432
95 plus	0.303873634	3.306261603	10693.31768	0.362233446	3.306261603	0.239855488	4.184225577	26569.67396	1.123205373	4.184225577

Table 15: Cuba 2017 life table, by age and sex. mx=mortality rate, ax=mean person-years lived in an age interval among those who die in that age interval, lx=number of persons left alive at age x, nLx=person-years lived between age x and x+n, ex=life expectancy at age x.

Age Group	Male					Female				
	mx	ax	lx	nLx	ex	mx	ax	lx	nLx	ex
Early Neonatal	0.089071787	0.009586311	100000	0.019161711	76.00433183	0.066138087	0.009587014	100000	0.019165925	80.46520272
Late Neonatal	0.014726817	0.028763061	99829.32449	0.057411724	76.11507403	0.012307055	0.028763728	99873.24071	0.057440978	80.54813878
Post Neonatal	0.001837806	0.461513281	99744.77693	0.920150362	76.12203201	0.001574036	0.461532019	99802.54848	0.920795383	80.54763861
1 to 4	0.00034017	1.99954644	99575.67622	3.980318577	75.32723206	0.000268138	1.999642483	99657.61422	3.984167621	79.74082435
5 to 9	0.00018123	2.499622438	99440.28362	4.969762254	71.42707399	0.000138516	2.499711426	99550.78603	4.975816062	75.82425097
10 to 14	0.000230671	2.768758194	99350.2202	4.964955762	66.48956035	0.000161675	2.690364045	99481.86442	4.972236948	70.87505189
15 to 19	0.00047987	2.71452857	99235.69584	4.956349711	61.56307276	0.000287042	2.623623262	99401.47667	4.966686466	65.93017126
20 to 24	0.000728009	2.626539059	98997.85924	4.941354701	56.70432426	0.000333165	2.59641627	99258.91321	4.958974966	61.02102897
25 to 29	0.000927039	2.594598986	98638.13449	4.920933849	51.90135113	0.000442852	2.621495922	99093.70055	4.949471945	56.11835086
30 to 34	0.001157783	2.618027671	98181.96629	4.895598955	47.13021744	0.00059376	2.645558519	98874.51876	4.936825468	51.23682229
35 to 39	0.001599444	2.647965533	97615.20316	4.862470935	42.38839035	0.00086268	2.667317534	98581.4041	4.919171646	46.3811425
40 to 44	0.002325089	2.679151991	96837.57014	4.815896117	37.7071276	0.001295392	2.698735083	98157.07172	4.893266563	41.56986924
45 to 49	0.003668393	2.699697773	95718.03796	4.745863913	33.11627334	0.002119024	2.705392738	97523.27684	4.852570979	36.82216048
50 to 54	0.006016221	2.687201447	93977.56621	4.634415509	28.67880193	0.003440261	2.700654955	96495.17704	4.786899783	32.18518527
55 to 59	0.009515325	2.671172738	91190.6059	4.460733127	24.47207809	0.005581784	2.709561425	94848.7587	4.682589934	27.69632444
60 to 64	0.01493668	2.633259562	86948.75516	4.199112714	20.53437922	0.009474727	2.662988834	92235.99208	4.51194485	23.40320395
65 to 69	0.021506469	2.624809992	80682.22643	3.83828049	16.92345569	0.013993859	2.646794139	87963.41499	4.258068276	19.40936739
70 to 74	0.033451854	2.605841635	72438.05194	3.353784098	13.54980864	0.021721139	2.65931919	82010.00516	3.902355143	15.62488169
75 to 79	0.051257575	2.567572434	61240.22815	2.723358737	10.55087633	0.036780617	2.629859391	73545.69713	3.38297188	12.11591559
80 to 84	0.079750188	2.511515626	47319.04784	1.975302003	7.902012031	0.06092615	2.572240847	61130.24606	2.663675799	9.042593344
85 to 89	0.13917769	2.408729877	31620.3422	1.163091697	5.584934197	0.113532202	2.494584435	44950.75411	1.751188794	6.374764163
90 to 94	0.2344537	2.177023396	15487.66865	0.466531936	3.90556688	0.199018397	2.274150305	25136.67651	0.815786647	4.442252543
95 plus	0.328898195	3.041013897	4578.830105	0.139492944	3.041013897	0.296105637	3.378463004	8951.480773	0.30313067	3.378463004

Table 15: Cuba 2100 life table, by age and sex. mx=mortality rate, ax=mean person-years lived in an age interval among those who die in that age interval, lx=number of persons left alive at age x, nLx=person-years lived between age x and x+n, ex=life expectancy at age x.										
Age Group	Male					Female				
	mx	ax	lx	nLx	ex	mx	ax	lx	nLx	ex
Early Neonatal	0.020557882	0.009588411	100000	0.019174302	82.0729271	0.016133379	0.009588547	100000	0.019175116	85.70971459
Late Neonatal	0.003710656	0.0287661	99960.58482	0.057505431	82.08611641	0.004313642	0.028765933	99969.06574	0.057509313	85.71699733
Post Neonatal	0.000420611	0.461613956	99939.24827	0.922547631	82.0460861	0.000358535	0.461618366	99944.26029	0.922620331	85.68067642
1 to 4	7.94234E-05	1.999894102	99900.44951	3.995383391	81.15449168	6.42729E-05	1.999914303	99911.18482	3.995933798	84.78553971
5 to 9	4.75485E-05	2.499900941	99868.72232	4.992842634	77.1796217	5.53947E-05	2.499884594	99885.50664	4.993583995	80.80676267
10 to 14	8.5148E-05	2.901521151	99844.98461	4.991331281	72.19729147	7.39279E-05	2.694986025	99857.85562	4.992027194	75.828352
15 to 19	0.000186528	2.770208043	99802.49418	4.988024731	67.22670781	0.000110005	2.579965923	99820.96421	4.989722238	70.85532509
20 to 24	0.000302402	2.643447571	99709.48741	4.981928578	62.28622942	0.000118008	2.632987394	99766.09817	4.986900522	65.89270831
25 to 29	0.000398549	2.643330949	99558.90513	4.973258834	57.37563141	0.000173833	2.680756247	99707.28092	4.983332854	60.92989089
30 to 34	0.000559499	2.643954385	99360.89134	4.96149187	52.4834809	0.000246551	2.662155126	99620.7136	4.978140276	55.98025323
35 to 39	0.000769463	2.667517183	99083.62647	4.945326142	47.62165305	0.000339406	2.677214018	99498.06067	4.970976244	51.04564569
40 to 44	0.001192826	2.709707672	98703.7288	4.921730391	42.79289418	0.000526772	2.764199219	99329.45203	4.960609902	46.12726861
45 to 49	0.001946554	2.750026349	98118.1792	4.884471588	38.02919322	0.000988036	2.785117227	99068.35458	4.94250108	41.24076062
50 to 54	0.003472173	2.731200796	97170.58993	4.820579659	33.36915429	0.001781736	2.746302142	98580.67207	4.909206205	36.42961278
55 to 59	0.005832273	2.684097443	95503.90783	4.71166509	28.89817507	0.00299413	2.739022041	97707.37609	4.852373596	31.72886141
60 to 64	0.008765411	2.644263292	92772.05519	4.545193443	24.66311482	0.005139545	2.699338072	96257.29312	4.756400255	27.16314765
65 to 69	0.012514773	2.64798732	88817.68587	4.314747067	20.63510179	0.007820184	2.688981792	93820.70162	4.607489973	22.79447079
70 to 74	0.019227828	2.645708681	83469.87349	3.99448269	16.77905574	0.012313108	2.722117934	90234.43311	4.388678358	18.58824428
75 to 79	0.029961553	2.64150724	75887.62456	3.548009567	13.18352942	0.022512481	2.705571864	84864.27155	4.036277759	14.58612831
80 to 84	0.050240316	2.597432044	65478.09761	2.930832389	9.855463376	0.040434326	2.643379771	75896.84691	3.472014402	10.97241886
85 to 89	0.099106924	2.53098254	51239.92232	2.077489333	6.849984405	0.082199152	2.614189175	62246.78264	2.621592914	7.739595755
90 to 94	0.190469343	2.234894312	31637.88886	1.052837667	4.60348881	0.159773423	2.325876179	41769.62442	1.48811067	5.286821033
95 plus	0.292424999	3.440597803	12468.53066	0.442073388	3.440597803	0.258925646	3.911030834	19277.16114	0.784537599	3.911030834

Table 15: Dominica 2017 life table, by age and sex. mx=mortality rate, ax=mean person-years lived in an age interval among those who die in that age interval, lx=number of persons left alive at age x, nLx=person-years lived between age x and x+n, ex=life expectancy at age x.

Age Group	Male					Female				
	mx	ax	lx	nLx	ex	mx	ax	lx	nLx	ex
Early Neonatal	0.961193903	0.009559581	100000	0.019002441	70.21423667	0.702638498	0.009567505	100000	0.019049463	74.87996135
Late Neonatal	0.084124288	0.028743918	98174.13363	0.056347381	71.49973577	0.074374626	0.028746607	98661.78156	0.05664309	75.87580631
Post Neonatal	0.011097788	0.460855469	97700.4902	0.897455524	71.78872774	0.009880151	0.460941967	98240.69817	0.902922238	76.14338636
1 to 4	0.001197381	1.998403493	96705.58365	3.85898662	71.5997659	0.000994972	1.99867337	97349.19366	3.886235587	75.91349399
5 to 9	0.000390081	2.49918733	96244.12866	4.807519434	67.93388423	0.000314865	2.499344032	96962.8491	4.844329434	72.20824354
10 to 14	0.000755843	2.737139072	96056.70999	4.794636189	63.06163442	0.000556959	2.645642788	96810.36842	4.834180491	67.31807682
15 to 19	0.001255584	2.690919835	95694.44264	4.77089209	58.29006369	0.000707117	2.693747056	96541.16157	4.819200088	62.49840116
20 to 24	0.001925808	2.560880649	95095.48483	4.732545415	53.64019177	0.00122284	2.614566073	96200.40512	4.796031261	57.71017063
25 to 29	0.001845701	2.554981501	94184.14395	4.68805245	49.13431855	0.001392573	2.536864793	95613.95614	4.764356743	53.04796376
30 to 34	0.002457257	2.552406623	93318.93225	4.638054148	44.56607668	0.001488209	2.503888734	94950.52952	4.729957563	48.40073857
35 to 39	0.002514458	2.667932822	92179.32983	4.582100273	40.08534828	0.001440873	2.659032703	94246.67166	4.696493652	43.743358
40 to 44	0.004595039	2.618547337	91027.27219	4.502103337	35.55874834	0.002624949	2.642325812	93570.02891	4.649727352	39.04030547
45 to 49	0.005304365	2.590581144	88958.76285	4.391816898	31.32441281	0.003296544	2.581032191	92349.62509	4.580955058	34.5210509
50 to 54	0.007236342	2.639866162	86629.52383	4.258754811	27.09669507	0.004017086	2.614145384	90839.70141	4.498873378	30.05169067
55 to 59	0.010795468	2.652160879	83548.34382	4.074176093	22.99830235	0.005686984	2.761873462	89032.78766	4.395701631	25.60827718
60 to 64	0.016524293	2.630189855	79151.20714	3.808469181	19.1282258	0.011751199	2.682870385	86533.51113	4.212019274	21.26781489
65 to 69	0.024161643	2.655514159	72860.11389	3.447785425	15.5523865	0.017468364	2.648526403	81585.40023	3.91838731	17.3944183
70 to 74	0.042754965	2.598107712	64533.6226	2.926358361	12.2161774	0.028025825	2.664108379	74743.80877	3.507712112	13.74359653
75 to 79	0.063197027	2.531937409	52030.36312	2.250784599	9.52764268	0.050346976	2.612389267	64920.29693	2.898019623	10.41966172
80 to 84	0.096614596	2.480549626	37819.68722	1.521159611	7.157510769	0.08526109	2.505442983	50346.15994	2.076380209	7.680062729
85 to 89	0.159314756	2.354233811	23140.42408	0.814239849	5.127260078	0.145344007	2.398627881	32670.71147	1.18596956	5.482272838
90 to 94	0.254270242	2.137894737	10183.12073	0.294820017	3.66049235	0.233699503	2.199035451	15463.47544	0.467627764	3.919780058
95 plus	0.344747517	2.900825131	2693.365579	0.078176518	2.900825131	0.327511217	3.053805442	4552.382223	0.13918847	3.053805442

Table 15: Dominica 2100 life table, by age and sex. mx=mortality rate, ax=mean person-years lived in an age interval among those who die in that age interval, lx=number of persons left alive at age x, nLx=person-years lived between age x and x+n, ex=life expectancy at age x.

Age Group	Male					Female				
	mx	ax	lx	nLx	ex	mx	ax	lx	nLx	ex
Early Neonatal	0.406098264	0.009576594	100000	0.019103647	75.83582927	0.333456633	0.009578821	100000	0.019116923	80.05089081
Late Neonatal	0.037495393	0.02875678	99225.02157	0.057026883	76.40766897	0.046851641	0.028754199	99363.04264	0.057090838	80.54394564
Post Neonatal	0.005231063	0.46127223	99011.38159	0.911958382	76.5146577	0.005402661	0.46126004	99095.73779	0.912662528	80.70351122
1 to 4	0.00042386	1.999434854	98534.99997	3.938066468	75.95907458	0.00039332	1.999475574	98603.19999	3.941030863	80.18048556
5 to 9	0.000181237	2.499622424	98368.3807	4.91619255	72.08440724	0.000185097	2.499614382	98448.39411	4.920145093	76.303334
10 to 14	0.00041521	2.844456929	98279.33938	4.909402036	67.14725596	0.000369199	2.658057719	98357.42961	4.913611814	71.371523
15 to 19	0.000697768	2.724268945	98075.74935	4.895954885	62.28035327	0.000452901	2.688235223	98176.1271	4.903701608	66.49836655
20 to 24	0.001108976	2.57109122	97734.45915	4.873614196	57.48737164	0.000808323	2.637926185	97954.17221	4.888310288	61.64287605
25 to 29	0.001094033	2.599634759	97194.46278	4.84700394	52.7914534	0.000914224	2.537176965	97559.68966	4.867007137	56.8813458
30 to 34	0.001633181	2.563048605	96665.31643	4.814104638	48.0648167	0.000950713	2.478118711	97115.1388	4.84416301	52.13001425
35 to 39	0.001569603	2.691197266	95880.67125	4.776726898	43.43602597	0.000828257	2.630878959	96655.1719	4.823283187	47.366084
40 to 44	0.003084181	2.632139331	95132.78804	4.722179287	38.75511696	0.001462015	2.667638772	96256.10468	4.796435339	42.55132626
45 to 49	0.003543889	2.59113248	93679.99142	4.644394459	34.31320997	0.001997519	2.590412265	95555.52707	4.754871182	37.84344618
50 to 54	0.00471826	2.655856052	92038.47034	4.551684947	29.87731588	0.0023141	2.618879051	94607.07834	4.704410677	33.19645869
55 to 59	0.007314766	2.67003051	89897.52078	4.419700775	25.5238068	0.003333689	2.775898357	93519.59815	4.641475533	28.55173517
60 to 64	0.011172328	2.657172301	86677.8092	4.223713113	21.37053127	0.006789826	2.709492735	91974.14224	4.528346519	23.98449707
65 to 69	0.017128785	2.69401639	81979.92335	3.943872627	17.43973839	0.010699746	2.688356643	88903.29091	4.337693938	19.71809405
70 to 74	0.031169728	2.649644852	75265.48325	3.508319234	13.7528201	0.017191285	2.758531237	84280.70363	4.05782666	15.65147377
75 to 79	0.04981771	2.588061781	64444.39237	2.879506175	10.61751543	0.036989512	2.686717538	77324.69662	3.562309735	11.80944919
80 to 84	0.080515399	2.542168228	50244.62103	2.101364355	7.894869816	0.067328153	2.548618156	64210.11541	2.758385003	8.670684538
85 to 89	0.140158065	2.406707577	33522.10116	1.234173053	5.575501632	0.12200057	2.468582178	45763.17989	1.752793303	6.130829715
90 to 94	0.235258941	2.174716195	16467.86936	0.49731311	3.900835242	0.208315981	2.254184226	24614.85886	0.786587583	4.2993088
95 plus	0.329490781	3.038293062	4910.113102	0.150453608	3.038293062	0.304554388	3.289576903	8421.028199	0.279632046	3.289576903

Table 15: Dominican Republic 2017 life table, by age and sex. mx=mortality rate, ax=mean person-years lived in an age interval among those who die in that age interval, lx=number of persons left alive at age x, nLx=person-years lived between age x and x+n, ex=life expectancy at age x.

Age Group	Male					Female				
	mx	ax	lx	nLx	ex	mx	ax	lx	nLx	ex
Early Neonatal	0.8581312	0.00956274	100000	0.019021162	69.9802727	0.630345157	0.009569721	100000	0.01906264	76.98927726
Late Neonatal	0.069009226	0.028748087	98368.16984	0.056483245	71.12139681	0.052235044	0.028752714	98798.59016	0.056757725	77.90584832
Post Neonatal	0.006605528	0.46117459	97978.61613	0.901873487	71.34660043	0.006243094	0.461200337	98502.22373	0.906843945	78.08263571
1 to 4	0.001133154	1.998489129	97383.32659	3.886525496	70.8568844	0.000643852	1.999141531	97936.3397	3.912415978	77.60797268
5 to 9	0.000497875	2.49896276	96943.28915	4.841139128	67.16970598	0.000345503	2.499280202	97684.56951	4.880013177	73.80293004
10 to 14	0.000480386	2.741524544	96702.37662	4.82987986	62.33090379	0.000315841	2.618246026	97516.00632	4.872136208	68.92620041
15 to 19	0.001059137	2.791810654	96470.42346	4.812268203	57.47413874	0.000526746	2.675480208	97362.1481	4.862155997	64.03094238
20 to 24	0.001981614	2.6463412	95960.77921	4.775765245	52.76412981	0.000764055	2.629648876	97106.05797	4.846527618	59.19259571
25 to 29	0.002485121	2.595111329	95014.52072	4.722508613	48.26252228	0.001009673	2.631234924	96735.7939	4.825252998	54.40883401
30 to 34	0.003184242	2.584051699	93841.15794	4.656248823	43.83279003	0.00141412	2.646639788	96248.66933	4.796473923	49.67055239
35 to 39	0.003874622	2.588897956	92358.90924	4.57522116	39.4939334	0.002028241	2.623556877	95570.52507	4.7556074	45.00379042
40 to 44	0.005004162	2.605488667	90586.91747	4.475743127	35.21477081	0.002656785	2.622956231	94606.25022	4.700630752	40.4351265
45 to 49	0.00668444	2.624351208	88348.4327	4.348419553	31.03963478	0.003670283	2.635979982	93357.85755	4.627752327	35.94007242
50 to 54	0.00951819	2.600744471	85443.94206	4.176910334	27.00373423	0.005202786	2.616987186	91660.11516	4.526907913	31.55608321
55 to 59	0.012199783	2.636752432	81472.56874	3.959621197	23.19107189	0.006870222	2.654642488	89306.27802	4.394553923	27.31781259
60 to 64	0.019502927	2.606885471	76649.24721	3.661866823	19.48236784	0.010927006	2.635920563	86289.45548	4.205930243	23.17900655
65 to 69	0.025953521	2.600085358	69521.49075	3.272759287	16.21068974	0.015255802	2.645312653	81698.53746	3.943457851	19.33209997
70 to 74	0.04038498	2.558057223	61050.94991	2.779439282	13.09842747	0.024596284	2.612629406	75691.11381	3.575039138	15.65547089
75 to 79	0.052707021	2.528988998	49868.02337	2.207389336	10.46414075	0.034859236	2.607213317	66916.24228	3.088835859	12.36547519
80 to 84	0.080498698	2.518067974	38294.30647	1.59751942	7.86909437	0.058138704	2.590600785	56178.30527	2.46479552	9.23207358
85 to 89	0.14013349	2.40613152	25512.45657	0.937118443	5.563098066	0.109709324	2.50727476	41896.79211	1.646320634	6.500289133
90 to 94	0.235395367	2.175156955	12455.61564	0.374798128	3.893887063	0.194658891	2.28276622	23900.30854	0.782556406	4.516680565
95 plus	0.329651957	3.03433455	3672.04414	0.111753348	3.03433455	0.292083872	3.424945309	8716.259139	0.299249083	3.424945309

Table 15: Dominican Republic 2100 life table, by age and sex. mx=mortality rate, ax=mean person-years lived in an age interval among those who die in that age interval, lx=number of persons left alive at age x, nLx=person-years lived between age x and x+n, ex=life expectancy at age x.

Age Group	Male					Female				
	mx	ax	lx	nLx	ex	mx	ax	lx	nLx	ex
Early Neonatal	0.209239108	0.009582628	100000	0.019139676	78.59230009	0.175083453	0.009583675	100000	0.019145932	84.33824605
Late Neonatal	0.014396377	0.028763152	99599.85031	0.057280304	78.88826288	0.021172805	0.028761283	99664.96252	0.057306587	84.60218554
Post Neonatal	0.001185667	0.461559608	99517.41387	0.918329495	78.89594209	0.001271561	0.461553506	99543.68968	0.918535449	84.64758836
1 to 4	0.000190578	1.999745896	99408.59237	3.974829175	78.05842431	0.000101507	1.999864657	99426.92908	3.976270158	83.82292651
5 to 9	0.000154623	2.499677869	99332.88057	4.964725012	74.11629033	0.000129626	2.499729945	99386.58383	4.96771949	79.85604029
10 to 14	0.000201252	2.867610666	99256.13279	4.960658315	69.17162949	0.00015126	2.67937139	99322.20427	4.964357716	74.90614291
15 to 19	0.000491537	2.783858299	99156.31528	4.952413373	64.23828321	0.000250224	2.631577419	99247.13092	4.959411804	69.96071757
20 to 24	0.000865694	2.641795219	98912.95296	4.935563627	59.38875744	0.000303634	2.617172574	99123.06994	4.952574072	65.04483784
25 to 29	0.001074505	2.596319746	98485.9354	4.911612955	54.63361477	0.00042482	2.635139812	98972.74072	4.943671094	60.13946795
30 to 34	0.001365652	2.576920291	97958.6926	4.881800085	49.91236005	0.0005791	2.639080722	98762.85313	4.931371754	55.26100565
35 to 39	0.001594619	2.597099818	97292.99247	4.846104145	45.23452079	0.000781708	2.637145747	98477.52522	4.914792215	50.41243793
40 to 44	0.002122784	2.653340523	96521.92947	4.802198522	40.572758	0.00108578	2.682906942	98093.73605	4.892345992	45.59771192
45 to 49	0.003174808	2.696256891	95505.30287	4.74062636	35.97271329	0.00170261	2.677429388	97563.28038	4.85896546	40.82899172
50 to 54	0.005160396	2.662408061	94005.73734	4.644378513	31.49762269	0.002552744	2.654789432	96737.61224	4.808061012	36.15121353
55 to 59	0.00733837	2.663860806	91620.94053	4.503949482	27.23982445	0.003541305	2.678702873	95514.44731	4.736677666	31.57515452
60 to 64	0.01111871	2.640397033	88339.82311	4.304671089	23.14370482	0.005475301	2.642556169	93844.20797	4.632612836	27.08426864
65 to 69	0.016018157	2.638478216	83588.43855	4.028270402	19.29804836	0.007534932	2.687581187	91319.62693	4.48798045	22.75114996
70 to 74	0.024498365	2.612525304	77204.33833	3.64965977	15.66397641	0.012730219	2.712628013	87961.24494	4.274458303	18.50751941
75 to 79	0.035721908	2.610754842	68396.48329	3.15638425	12.33036879	0.022448385	2.711096489	82576.51042	3.929840494	14.52049032
80 to 84	0.06043166	2.583049966	57385.55288	2.518449396	9.184355056	0.042120041	2.655858436	73931.20751	3.375104688	10.88012278
85 to 89	0.113031636	2.488853419	42897.69188	1.694573819	6.409493314	0.084492076	2.607027038	60315.6512	2.53501641	7.680051928
90 to 94	0.205781385	2.215830292	24955.99351	0.810907827	4.359104679	0.162240718	2.319333912	40320.29867	1.436754555	5.254331938
95 plus	0.305110755	3.300604017	9205.169082	0.315774499	3.300604017	0.261166014	3.891045598	18655.47428	0.76527191	3.891045598

Table 15: Grenada 2017 life table, by age and sex. mx=mortality rate, ax=mean person-years lived in an age interval among those who die in that age interval, lx=number of persons left alive at age x, nLx=person-years lived between age x and x+n, ex=life expectancy at age x.

Age Group	Male					Female				
	mx	ax	lx	nLx	ex	mx	ax	lx	nLx	ex
Early Neonatal	0.390143797	0.009577083	100000	0.019106519	73.50224688	0.320123263	0.009579229	100000	0.019119336	75.50159646
Late Neonatal	0.019794497	0.028761663	99254.65645	0.057072921	74.03482944	0.024169843	0.028760456	99388.00472	0.057142405	75.94706488
Post Neonatal	0.003119203	0.461422253	99141.71157	0.914046716	74.0616085	0.002286596	0.4614814	99249.91334	0.9153958	75.99511805
1 to 4	0.000469501	1.999373998	98856.68292	3.950558926	73.35053761	0.000411566	1.999451245	99040.64863	3.95836866	75.23139123
5 to 9	0.000321184	2.499330867	98671.32724	4.929607774	69.48463818	0.000256672	2.499465267	98877.83424	4.940721306	71.35196976
10 to 14	0.00046178	2.595569415	98513.02629	4.920188081	64.59229576	0.000280781	2.707921125	98751.04537	4.934375904	66.44036666
15 to 19	0.000534048	2.544890358	98285.84752	4.907857735	59.73560123	0.000537179	2.668299233	98612.51381	4.924457672	61.52989582
20 to 24	0.000579503	2.929578035	98023.7534	4.895314167	54.88849419	0.000717141	2.618279807	98348.00109	4.909015832	56.68818563
25 to 29	0.001740286	2.636101367	97740.07277	4.866981849	50.03927203	0.000950156	2.621712022	97995.96587	4.888751513	51.8823828
30 to 34	0.001734759	2.639849367	96893.09237	4.824900686	45.45356541	0.001282789	2.653518229	97531.45719	4.861938411	47.11693599
35 to 39	0.002954225	2.625351389	96056.10577	4.769348306	40.82654934	0.001915989	2.645520195	96907.77228	4.823627389	42.40301894
40 to 44	0.003590968	2.607635723	94647.17355	4.692051636	36.39508605	0.002660282	2.616186885	95983.57992	4.768937838	37.78573131
45 to 49	0.004959522	2.728962955	92962.35201	4.596350731	32.00728915	0.003473624	2.675284719	94714.97457	4.697812974	33.25666763
50 to 54	0.009443181	2.685066175	90682.94137	4.437157771	27.7430444	0.005749112	2.653845741	93083.27401	4.592226405	28.79258536
55 to 59	0.014351495	2.616532346	86493.28972	4.18164497	23.95654793	0.008067134	2.658074207	90443.42399	4.438328826	24.55534425
60 to 64	0.019639458	2.584487328	80492.92181	3.842400309	20.54706757	0.012695207	2.685175889	86863.42506	4.219199632	20.45760381
65 to 69	0.026449575	2.554840019	72948.22294	3.425907179	17.40467004	0.021501644	2.651012751	81507.92719	3.879501441	16.62504147
70 to 74	0.033674985	2.531477974	63889.44398	2.949391363	14.51018137	0.03367765	2.618258069	73168.33209	3.386870295	13.21730132
75 to 79	0.043339716	2.530113505	53961.30235	2.437300167	11.71435592	0.054304603	2.57712817	61767.25389	2.729519839	10.17306637
80 to 84	0.060649634	2.549508753	43403.62519	1.889526094	8.949011962	0.087687266	2.513741073	46956.94891	1.928151536	7.568607609
85 to 89	0.114732339	2.479774384	31950.53374	1.239371098	6.244039699	0.14847551	2.390113597	30076.7814	1.084472991	5.407661243
90 to 94	0.20910249	2.220875357	17738.71814	0.561046092	4.261584534	0.236977534	2.191529997	14006.36961	0.420828106	3.87648738
95 plus	0.308213035	3.244601259	6011.939213	0.195115793	3.244601259	0.330419918	3.027039507	4052.059653	0.122832346	3.027039507

Table 15: Grenada 2100 life table, by age and sex. mx=mortality rate, ax=mean person-years lived in an age interval among those who die in that age interval, lx=number of persons left alive at age x, nLx=person-years lived between age x and x+n, ex=life expectancy at age x.

Age Group	Male					Female				
	mx	ax	lx	nLx	ex	mx	ax	lx	nLx	ex
Early Neonatal	0.093588616	0.009586173	100000	0.019160885	80.84183603	0.089773766	0.00958629	100000	0.019161585	81.50908264
Late Neonatal	0.004453519	0.028765895	99820.72372	0.057423744	80.96766885	0.007718063	0.028764994	99828.02069	0.057422551	81.63017264
Post Neonatal	0.000807554	0.461586468	99795.15108	0.921052947	80.93085151	0.000520502	0.46160686	99783.70678	0.921069343	81.6088598
1 to 4	0.000105627	1.999859164	99720.78027	3.98798893	80.06755142	8.9408E-05	1.999880789	99735.76987	3.988717758	80.72452914
5 to 9	9.68557E-05	2.499798217	99678.67046	4.982727131	76.10047454	9.75136E-05	2.499796847	99700.12172	4.983791221	76.75262086
10 to 14	0.00020737	2.65025886	99630.42008	4.979082028	71.13607394	0.000123229	2.787064088	99651.53147	4.981208324	71.78877146
15 to 19	0.000233854	2.378863028	99527.17991	4.97343797	66.2070895	0.00025789	2.66180248	99590.15696	4.976504042	66.83125111
20 to 24	0.000192567	3.377115122	99410.93973	4.968855658	61.28105398	0.000320901	2.628865025	99461.83212	4.969305894	61.91401491
25 to 29	0.000915314	2.668942073	99315.29818	4.955191506	56.3367209	0.000452786	2.662797683	99302.3866	4.959863213	57.00912158
30 to 34	0.000939714	2.659220977	98861.82689	4.932241036	51.582319	0.000669127	2.646800114	99077.85722	4.946089992	52.13204016
35 to 39	0.001646355	2.626550374	98398.50926	4.900817045	46.8118547	0.000912675	2.640942705	98746.99514	4.92674368	47.29744046
40 to 44	0.002000479	2.595830617	97592.09537	4.856260722	42.17504588	0.001294405	2.664307615	98297.49801	4.900046476	42.50098865
45 to 49	0.002593536	2.781055134	96621.9912	4.803390016	37.56853011	0.001932941	2.696814034	97663.58708	4.861465435	37.75863111
50 to 54	0.00553092	2.747032485	95378.32805	4.710146721	33.01841896	0.003078407	2.671089234	96724.80247	4.801789232	33.09764193
55 to 59	0.009327639	2.63228444	92780.38016	4.538972676	28.85907469	0.004509394	2.666179103	95248.15705	4.71278655	28.56756085
60 to 64	0.012160216	2.58175384	88562.08495	4.302168252	25.10030553	0.006825696	2.677586177	93126.05519	4.583785416	24.15528163
65 to 69	0.015668568	2.561301015	83356.44119	4.015554414	21.49670676	0.010979688	2.699491118	90002.41285	4.389295993	19.89621634
70 to 74	0.019609849	2.555451066	77109.9639	3.681929817	18.0219564	0.018464586	2.728859875	85204.92785	4.08932209	15.85717536
75 to 79	0.024587017	2.573792283	70022.49662	3.30695559	14.5777801	0.036054642	2.685512969	77707.29706	3.588751208	12.11332043
80 to 84	0.0355677	2.650365633	62024.90567	2.869045278	11.12331029	0.064890578	2.592271575	64981.90776	2.81931262	8.950145967
85 to 89	0.077239593	2.601397016	52154.77294	2.214397846	7.712952619	0.118104697	2.485316372	47154.15474	1.835209881	6.321297389
90 to 94	0.164155019	2.247988614	35791.20249	1.248381324	5.099081587	0.203220537	2.261226285	26385.49893	0.862220417	4.415620042
95 plus	0.270164328	3.724884661	16120.09264	0.616654492	3.724884661	0.29962039	3.362920441	9632.211285	0.335219714	3.362920441

Table 15: Guyana 2017 life table, by age and sex. mx=mortality rate, ax=mean person-years lived in an age interval among those who die in that age interval, lx=number of persons left alive at age x, nLx=person-years lived between age x and x+n, ex=life expectancy at age x.

Age Group	Male					Female				
	mx	ax	lx	nLx	ex	mx	ax	lx	nLx	ex
Early Neonatal	0.785838421	0.009564955	100000	0.01903431	66.55264372	0.544434907	0.009572354	100000	0.019078317	72.23856027
Late Neonatal	0.04384104	0.02875503	98504.52809	0.056602454	67.54333014	0.018087193	0.028762134	98961.44234	0.056907113	72.9771913
Post Neonatal	0.006438133	0.461186482	98256.49131	0.904500591	67.65624992	0.004961294	0.461291394	98858.54095	0.91066205	72.99558978
1 to 4	0.000739236	1.999014353	97674.49602	3.901212882	67.13348912	0.000740272	1.999012971	98406.88527	3.930455574	72.40525993
5 to 9	0.000470702	2.49901937	97386.29352	4.863591022	63.32635501	0.000378927	2.499210569	98116.04	4.90115842	68.61402655
10 to 14	0.000496139	2.818303803	97157.43757	4.852620227	58.46966511	0.000412531	2.678543605	97930.35565	4.89183427	63.73940516
15 to 19	0.001234563	2.7949157	96916.72838	4.83268246	53.60783751	0.000735962	2.736456749	97728.57166	4.878303601	58.86541287
20 to 24	0.002266299	2.697450826	96320.155	4.791008321	48.9222128	0.001256485	2.646512407	97369.56557	4.854126102	54.07215476
25 to 29	0.003441562	2.599629646	95234.46561	4.722714187	44.44854859	0.001636066	2.613872921	96759.69978	4.81917442	49.39590243
30 to 34	0.004019778	2.561469589	93609.34513	4.635045319	40.17422274	0.002181156	2.601253663	95971.34859	4.773598392	44.77968745
35 to 39	0.004799664	2.591427514	91746.57469	4.534924162	35.93693862	0.002747699	2.600999822	94930.34142	4.715442304	40.24157669
40 to 44	0.006392735	2.619503225	89570.7388	4.411438149	31.74582863	0.003595783	2.648962332	93635.0422	4.642514785	35.76143723
45 to 49	0.008933448	2.626616975	86752.13254	4.247608919	27.69082264	0.005478189	2.657771168	91966.33261	4.540085427	31.36135022
50 to 54	0.012727792	2.63468779	82960.44124	4.026903987	23.83490431	0.008087124	2.655088803	89480.43417	4.390805233	27.15766571
55 to 59	0.019085306	2.612052138	77840.43931	3.722577696	20.22789712	0.012282373	2.642720422	85931.92094	4.175795264	23.1681894
60 to 64	0.026929483	2.574780213	70745.40076	3.320749206	16.99342235	0.018242721	2.607157793	80807.77199	3.871588354	19.46829051
65 to 69	0.036320434	2.562315514	61818.18563	2.840017161	14.07525316	0.025197827	2.599331779	73753.84707	3.477687785	16.07956706
70 to 74	0.052795685	2.525640686	51526.56469	2.27943619	11.37574555	0.037748723	2.585185113	65006.71712	2.979434085	12.89265342
75 to 79	0.070272156	2.489144882	39528.1162	1.681019114	9.065672268	0.056571164	2.554131452	53789.42571	2.363614321	10.04290292
80 to 84	0.104469878	2.429403346	27763.29457	1.095412078	6.860293383	0.088997011	2.492077069	40467.64883	1.655570509	7.512394611
85 to 89	0.16779747	2.332544642	16372.60045	0.566332073	4.95643128	0.149873658	2.386469951	25800.37756	0.92824031	5.376387728
90 to 94	0.262355382	2.121102655	6909.746563	0.197140212	3.569098027	0.238420937	2.188191129	11952.11223	0.358419691	3.858365745
95 plus	0.351121439	2.848468223	1754.156351	0.050072994	2.848468223	0.331690123	3.0158542	3440.427014	0.104063937	3.0158542

Table 15: Guyana 2100 life table, by age and sex. mx=mortality rate, ax=mean person-years lived in an age interval among those who die in that age interval, lx=number of persons left alive at age x, nLx=person-years lived between age x and x+n, ex=life expectancy at age x.

Age Group	Male					Female				
	mx	ax	lx	nLx	ex	mx	ax	lx	nLx	ex
Early Neonatal	0.174213563	0.009583701	100000	0.019146087	76.99084964	0.123692208	0.00958525	100000	0.019155356	82.06680892
Late Neonatal	0.011292847	0.028764008	99666.55062	0.057323779	77.22842487	0.004511222	0.028765879	99763.1072	0.057390505	82.24130821
Post Neonatal	0.001331984	0.461549214	99601.83418	0.919046509	77.22086712	0.001347253	0.461548129	99737.22228	0.920289178	82.20498128
1 to 4	0.00016574	1.999779014	99479.48797	3.977861845	76.39134862	0.000152712	1.999796384	99613.28389	3.983315187	81.38251828
5 to 9	0.000122281	2.499745249	99413.61391	4.969162015	72.44026168	0.000157615	2.499671636	99552.48303	4.97566445	77.43062058
10 to 14	0.000159963	3.156390976	99352.87369	4.96607894	67.48296626	0.000199564	2.694550115	99474.10967	4.971395765	72.48926843
15 to 19	0.000556003	2.78999099	99273.4841	4.957577729	62.53443093	0.000320957	2.691409968	99374.99397	4.965056369	67.55803648
20 to 24	0.000980952	2.688002935	98997.96433	4.938715301	57.69770798	0.00047978	2.614208292	99215.79143	4.955143303	62.66124458
25 to 29	0.00150234	2.565813607	98514.25983	4.907994044	52.96256239	0.000603703	2.692214351	98978.42221	4.94189982	57.80363682
30 to 34	0.001684838	2.557466968	97779.97362	4.86907335	48.33305929	0.000889824	2.660615018	98681.01426	4.923660817	52.96708063
35 to 39	0.001923573	2.635885417	96968.58185	4.826405458	43.70670812	0.001131084	2.62366681	98244.35749	4.899009764	48.18747356
40 to 44	0.0026443	2.658544233	96052.05142	4.773100706	39.0921642	0.001484983	2.73886818	97692.48707	4.867948896	43.44073363
45 to 49	0.00371763	2.682936817	94806.15437	4.700040072	34.56391392	0.002459077	2.70876851	96973.48758	4.821244351	38.73749516
50 to 54	0.00580913	2.710454664	93083.89962	4.593343862	30.14463968	0.00360727	2.686619861	95794.55753	4.7499315	34.17411163
55 to 59	0.009314091	2.653313611	90460.58714	4.427163465	25.92808264	0.005461485	2.63760033	94091.53577	4.644701727	29.7358735
60 to 64	0.012709964	2.629064047	86413.01187	4.196259917	22.00804206	0.007086002	2.647005061	91575.18262	4.504110864	25.47051437
65 to 69	0.018925094	2.659510668	81187.8449	3.89114585	18.24394887	0.010961091	2.66612226	88411.83011	4.311291991	21.27449461
70 to 74	0.029573128	2.604817083	74077.20338	3.465908487	14.73447887	0.01647405	2.698106047	83762.80869	4.037076386	17.29026045
75 to 79	0.042293252	2.589820254	64178.50527	2.923311466	11.60392884	0.029232443	2.678712806	77255.74185	3.623389684	13.4978691
80 to 84	0.068828065	2.50252903	52372.34047	2.252768152	8.655525094	0.051850117	2.583469931	67014.13667	2.999301651	10.12476731
85 to 89	0.123882091	2.456355946	37700.10297	1.456729499	6.077571037	0.098326815	2.559160864	52488.51275	2.15556017	7.156749541
90 to 94	0.217563648	2.200021862	20827.66158	0.662398885	4.175826941	0.178737761	2.294502931	33334.04715	1.162137124	4.932255806
95 plus	0.314839546	3.195636734	7228.394742	0.240294226	3.195636734	0.276514671	3.688493958	14578.57652	0.578134062	3.688493958

Table 15: Haiti 2017 life table, by age and sex. mx=mortality rate, ax=mean person-years lived in an age interval among those who die in that age interval, lx=number of persons left alive at age x, nLx=person-years lived between age x and x+n, ex=life expectancy at age x.

Age Group	Male					Female				
	mx	ax	lx	nLx	ex	mx	ax	lx	nLx	ex
Early Neonatal	1.039150444	0.009557191	100000	0.018988268	63.79374423	0.833910872	0.009563482	100000	0.019025555	66.0073474
Late Neonatal	0.107568213	0.028737451	98027.17012	0.056225095	65.0580341	0.139235521	0.028728715	98413.66067	0.056395444	67.05182256
Post Neonatal	0.026590692	0.459754898	97422.60395	0.888543205	65.40415997	0.023930589	0.459943862	97628.7109	0.891510857	67.53330503
1 to 4	0.004235151	1.99435316	95061.2093	3.770447773	66.09395554	0.002785429	1.996286102	95496.01283	3.798651556	68.10730097
5 to 9	0.00085672	2.498215167	93465.74436	4.663294937	63.1891356	0.000807593	2.498317515	94438.54924	4.712409072	64.84788978
10 to 14	0.00073316	2.647682996	93066.34066	4.645308454	58.44941671	0.000610548	2.569947604	94058.07115	4.695940522	60.09986236
15 to 19	0.001388389	2.704310588	92725.81353	4.621567396	53.65406168	0.001022323	2.68301069	93771.41216	4.677496406	55.27553286
20 to 24	0.002123841	2.612029045	92084.20196	4.58097941	49.00810534	0.001524932	2.644903118	93293.28346	4.647974567	50.5443438
25 to 29	0.002570293	2.586507914	91111.51061	4.527502273	44.5018913	0.002107095	2.62286574	92584.69691	4.606169462	45.90986053
30 to 34	0.003264351	2.599110721	89948.33308	4.462464807	40.04253511	0.002812599	2.620077851	91614.54028	4.5502775	41.36695098
35 to 39	0.00423706	2.612528288	88492.67499	4.380369939	35.65697743	0.003808145	2.629547164	90335.45836	4.476395232	36.914066
40 to 44	0.005762616	2.632841636	86638.487	4.27370428	31.3622976	0.005348693	2.632025257	88632.08563	4.376241171	32.57084751
45 to 49	0.008299597	2.643119083	84179.20193	4.128342126	27.19880212	0.007532119	2.639857671	86293.81989	4.239437466	28.37879459
50 to 54	0.012269108	2.634873129	80759.22657	3.924341899	23.23542589	0.01107953	2.619666929	83105.55113	4.048728382	24.36197094
55 to 59	0.017929841	2.62906002	75956.86298	3.643412799	19.53432229	0.015216712	2.623923935	78629.75391	3.794718901	20.59462064
60 to 64	0.027119618	2.6051193	69445.85078	3.261301466	16.11661123	0.022898915	2.621801045	72875.52248	3.456375154	17.00855617
65 to 69	0.039719005	2.575519259	60637.90893	2.766835328	13.07821253	0.034656853	2.597508058	64997.96974	3.001581591	13.74827366
70 to 74	0.059122946	2.532251159	49707.30928	2.170799771	10.39013353	0.052789757	2.557297433	54665.57092	2.423616161	10.85499956
75 to 79	0.085393343	2.477123531	36961.02833	1.52280308	8.109079506	0.079559232	2.505088617	41991.34258	1.755658356	8.366359092
80 to 84	0.126225258	2.40260586	24066.59009	0.908434393	6.145801857	0.123036847	2.425909859	28206.14839	1.075417273	6.253878954
85 to 89	0.191623376	2.274895606	12706.25187	0.418727847	4.524780526	0.189285749	2.291524978	15185.80067	0.504887874	4.585680518
90 to 94	0.284521748	2.074116555	4750.26173	0.130066737	3.337343611	0.278280373	2.09609229	5777.231041	0.16090411	3.39916136
95 plus	0.368378875	2.715355864	1073.661707	0.029282952	2.715355864	0.366235318	2.732894064	1359.771168	0.037534789	2.732894064

Table 15: Haiti 2100 life table, by age and sex. mx=mortality rate, ax=mean person-years lived in an age interval among those who die in that age interval, lx=number of persons left alive at age x, nLx=person-years lived between age x and x+n, ex=life expectancy at age x.

Age Group	Male					Female				
	mx	ax	lx	nLx	ex	mx	ax	lx	nLx	ex
Early Neonatal	0.226785286	0.00958209	100000	0.019136443	74.92422317	0.205279614	0.009582749	100000	0.019140386	79.83501536
Late Neonatal	0.015591418	0.028762822	99566.10816	0.057258933	75.22950093	0.030622256	0.028758676	99607.16754	0.057257801	80.1288827
Post Neonatal	0.003803299	0.461373656	99476.86335	0.916848384	75.23817124	0.003647131	0.46138475	99431.91445	0.916500526	80.21087901
1 to 4	0.000750149	1.998999853	99128.54439	3.959288314	74.56890018	0.000555002	1.999260043	99098.14535	3.959619281	79.54773548
5 to 9	0.000439924	2.499083567	98837.3589	4.936739845	70.74487419	0.00042981	2.499104623	98884.19104	4.939193616	75.68511973
10 to 14	0.000581915	2.799189235	98633.77716	4.925796496	65.85193066	0.000435568	2.631447893	98684.85177	4.929410022	70.81089647
15 to 19	0.001036564	2.72920898	98378.28086	4.908544824	60.97955834	0.000546047	2.652237118	98487.54876	4.918427555	65.93160036
20 to 24	0.001351797	2.623046752	97937.07711	4.882992394	56.20956912	0.000685895	2.618967889	98240.20536	4.904552444	61.08112387
25 to 29	0.001583524	2.594301919	97364.36076	4.85213738	51.50729632	0.00084493	2.6073756	97930.72551	4.887316889	56.2599549
30 to 34	0.001831588	2.588140806	96704.06231	4.816303731	46.83759332	0.001013703	2.619733037	97549.37958	4.866388309	51.46654336
35 to 39	0.00223126	2.602301407	95926.4805	4.774136977	42.20088194	0.001291524	2.64631822	97089.0543	4.840546506	46.69767722
40 to 44	0.002717467	2.64137513	95006.9049	4.722765309	37.59948974	0.001816969	2.675812705	96502.52653	4.805848254	41.96632153
45 to 49	0.003799593	2.684328798	93844.19882	4.654253529	33.046016	0.002678851	2.686160941	95678.84846	4.755646134	37.30709997
50 to 54	0.005982363	2.695074102	92210.51969	4.551646587	28.59935002	0.004106213	2.653821329	94461.65878	4.67962165	32.757309
55 to 59	0.00944069	2.68344063	89662.7421	4.391511516	24.35421693	0.005769267	2.643705016	92613.92311	4.570923674	28.36278167
60 to 64	0.014670886	2.651707423	85717.84284	4.147077845	20.37235397	0.008189982	2.658660086	90085.32415	4.422141658	24.09335887
65 to 69	0.022219566	2.634957599	79811.55674	3.796584702	16.69754451	0.012476476	2.667434862	86585.19234	4.209807913	19.96895809
70 to 74	0.034232484	2.607523456	71622.08691	3.316308993	13.32546414	0.019709665	2.686687541	81474.79541	3.900224805	16.06362979
75 to 79	0.052938198	2.57860787	60558.86778	2.692668851	10.30564849	0.034975556	2.649416516	73986.71827	3.424280152	12.42804116
80 to 84	0.086816432	2.509212322	46712.53747	1.930547102	7.632437178	0.058754206	2.586798921	62306.5789	2.738458879	9.275607044
85 to 89	0.147365691	2.387711589	30425.13306	1.107625846	5.418893916	0.110008617	2.509075566	46683.59492	1.844288407	6.537250747
90 to 94	0.24227232	2.160775585	14556.62283	0.435607799	3.816802423	0.194510907	2.280688751	26993.71383	0.891467655	4.541283563
95 plus	0.335068187	2.990168707	4233.982668	0.128441367	2.990168707	0.291776144	3.440853689	10100.41082	0.353952844	3.440853689

Table 15: Jamaica 2017 life table, by age and sex. mx=mortality rate, ax=mean person-years lived in an age interval among those who die in that age interval, lx=number of persons left alive at age x, nLx=person-years lived between age x and x+n, ex=life expectancy at age x.

Age Group	Male					Female				
	mx	ax	lx	nLx	ex	mx	ax	lx	nLx	ex
Early Neonatal	0.630959383	0.009569702	100000	0.019062533	71.61505844	0.495731965	0.009573847	100000	0.019087214	77.16109732
Late Neonatal	0.042648832	0.028755359	98797.509	0.056772755	72.46713848	0.045241573	0.028754643	99053.92091	0.056915844	77.87863818
Post Neonatal	0.003798155	0.461374021	98555.50314	0.908358007	72.58752272	0.003149577	0.461420095	98796.50852	0.910851412	78.02396867
1 to 4	0.00051877	1.999308306	98210.69098	3.924357353	71.9175472	0.00050872	1.999321707	98509.73179	3.936384669	77.32652113
5 to 9	0.00021636	2.499549251	98007.24957	4.897713758	68.06277198	0.000219412	2.499542891	98309.57145	4.912783782	73.4799494
10 to 14	0.000265009	3.033015169	97901.31999	4.892516654	63.13373395	0.000244015	2.617425799	98201.79964	4.907238235	68.55784218
15 to 19	0.000897364	2.837729086	97771.69038	4.879119186	58.21337341	0.000358611	2.653846216	98082.06838	4.899982502	63.63827502
20 to 24	0.001732933	2.650253147	97333.89119	4.846958619	53.46201914	0.000511749	2.617236811	97906.35981	4.889357381	58.74753672
25 to 29	0.002173254	2.581311597	96494.0311	4.799476653	48.90346705	0.000650153	2.669322719	97656.16578	4.875421344	53.89106392
30 to 34	0.002628802	2.574810043	95451.16207	4.742334082	44.40868315	0.00104604	2.676058847	97339.22165	4.855161975	49.05747155
35 to 39	0.003191982	2.583465269	94204.8264	4.674204562	39.96115199	0.001548569	2.658384998	96831.4316	4.82408201	44.3001379
40 to 44	0.004021341	2.586524016	92713.45965	4.591142653	35.56119481	0.002250277	2.667599954	96084.5787	4.779148662	39.62294734
45 to 49	0.005033306	2.632702675	90868.36688	4.489962143	31.22936456	0.003415158	2.665247549	95009.54756	4.712911043	35.04002111
50 to 54	0.007545532	2.658891496	88610.44495	4.353693061	26.95653822	0.005090244	2.66026992	93400.85216	4.615108677	30.59628276
55 to 59	0.01146108	2.644521558	85329.26544	4.154459657	22.88908374	0.007632389	2.672367227	91053.3169	4.473262908	26.31487565
60 to 64	0.016986742	2.648601833	80575.19869	3.87428848	19.08170155	0.012138383	2.638765446	87642.48515	4.26017906	22.23298683
65 to 69	0.027181126	2.633929854	74006.95342	3.47727381	15.53861257	0.017318715	2.627759778	82478.37849	3.961493778	18.45742081
70 to 74	0.043316122	2.57221668	64580.24174	2.922752621	12.42147408	0.026378943	2.620418709	75631.96148	3.558902236	14.88798082
75 to 79	0.061396132	2.519056294	51966.90973	2.256399006	9.813922485	0.040714319	2.604988002	66274.92109	3.020592893	11.6184475
80 to 84	0.089600385	2.459093733	38184.75776	1.556999445	7.45431137	0.067071377	2.562841403	54038.02151	2.324435096	8.661374914
85 to 89	0.150708386	2.377199932	24319.91255	0.87307538	5.316780664	0.121879065	2.468064207	38551.70366	1.475670188	6.120229442
90 to 94	0.245875094	2.154752904	11237.98026	0.331302272	3.761894401	0.208330538	2.254794413	20695.33335	0.659991305	4.292364232
95 plus	0.338058142	2.958843271	3128.245006	0.09283575	2.958843271	0.304624068	3.285081534	7033.203021	0.232135104	3.285081534

Table 15: Jamaica 2100 life table, by age and sex. mx=mortality rate, ax=mean person-years lived in an age interval among those who die in that age interval, lx=number of persons left alive at age x, nLx=person-years lived between age x and x+n, ex=life expectancy at age x.

Age Group	Male					Female				
	mx	ax	lx	nLx	ex	mx	ax	lx	nLx	ex
Early Neonatal	0.182406767	0.00958345	100000	0.019144585	78.50923245	0.150922256	0.009584415	100000	0.019150359	82.85865092
Late Neonatal	0.012464992	0.028763685	99650.91206	0.057312853	78.7645618	0.019008438	0.02876188	99711.0432	0.057336647	83.07920757
Post Neonatal	0.000921856	0.461578348	99579.4912	0.919014281	78.76352139	0.000867807	0.461582188	99602.08599	0.919245637	83.11253176
1 to 4	0.000139861	1.999813519	99494.83631	3.978681402	77.90649872	0.000138855	1.99981486	99522.35413	3.979789593	82.25522506
5 to 9	5.77034E-05	2.499879785	99439.24101	4.971245674	73.94867025	8.2977E-05	2.499827131	99467.13214	4.97232592	78.29962671
10 to 14	9.3447E-05	2.941763263	99410.58844	4.969571779	68.96896126	0.000130243	2.683784442	99425.90885	4.969793317	73.33091364
15 to 19	0.000439526	2.634449107	99364.1744	4.963067922	63.99958442	0.000195684	2.623508603	99361.20705	4.965767525	68.37683963
20 to 24	0.000678037	2.740182704	99146.91435	4.949435255	59.12648146	0.000262881	2.54316445	99264.10576	4.960014789	63.44102929
25 to 29	0.001053002	2.802090353	98816.52427	4.928825541	54.30404855	0.000349951	2.898963202	99133.88503	4.952851424	58.52067401
30 to 34	0.001638078	2.659977012	98312.89833	4.896989158	49.55351375	0.000671461	2.772884603	98965.42183	4.940651956	53.61504465
35 to 39	0.001590151	2.649464984	97551.73466	4.859763773	44.91048151	0.000901306	2.650086237	98634.68602	4.921324175	48.78437091
40 to 44	0.002380325	2.654891077	96810.11442	4.814008668	40.22572278	0.001322577	2.685440538	98191.69656	4.894558844	43.99150745
45 to 49	0.002668653	2.748529981	95709.9463	4.756581377	35.6476045	0.002032891	2.656677839	97545.829	4.854093508	39.26336346
50 to 54	0.00463234	2.699920306	94477.33789	4.674809194	31.06671741	0.002776689	2.674971958	96561.54129	4.797006527	34.6345335
55 to 59	0.007005193	2.708397859	92384.38213	4.546738895	26.68406142	0.004267181	2.665536284	95234.12686	4.71468281	30.07684448
60 to 64	0.010472079	2.688511185	89321.04327	4.362651687	22.48304842	0.00619455	2.642085059	93228.47095	4.594460942	25.66264136
65 to 69	0.017876129	2.707664669	84915.82144	4.083827587	18.46600829	0.008852907	2.677392864	90393.2833	4.428794553	21.37868606
70 to 74	0.03156436	2.607991116	78011.0559	3.646325342	14.81841276	0.014529875	2.736625457	86496.03212	4.187551551	17.21480519
75 to 79	0.046289077	2.595304342	67498.3977	3.074466797	11.66908779	0.027418221	2.72909924	80485.17025	3.790138499	13.28661604
80 to 84	0.075366626	2.483068643	55217.81543	2.3804856	8.728254099	0.052531991	2.617437376	70293.6896	3.132543351	9.810156808
85 to 89	0.12861999	2.452077692	40037.13451	1.577647505	6.154667422	0.100822807	2.542932543	54269.35626	2.191703836	6.908263576
90 to 94	0.219997159	2.182201098	23279.69653	0.763538734	4.225889279	0.183298684	2.297746176	33057.9562	1.121626694	4.767956077
95 plus	0.316107989	3.223900472	8893.954046	0.315098843	3.223900472	0.281226329	3.583453663	13344.18532	0.493238465	3.583453663

Table 15: Puerto Rico 2017 life table, by age and sex. mx=mortality rate, ax=mean person-years lived in an age interval among those who die in that age interval, lx=number of persons left alive at age x, nLx=person-years lived between age x and x+n, ex=life expectancy at age x.

Age Group	Male					Female				
	mx	ax	lx	nLx	ex	mx	ax	lx	nLx	ex
Early Neonatal	0.224723346	0.009582153	100000	0.019136816	74.21725167	0.163670397	0.009584025	100000	0.019148015	81.15374484
Late Neonatal	0.030875331	0.028758606	99569.96038	0.057235977	74.51856249	0.02890806	0.028759149	99686.6071	0.05730627	81.38966108
Post Neonatal	0.002685616	0.461453054	99393.25137	0.916548892	74.59346511	0.002248425	0.461484111	99520.95021	0.917911595	81.46755733
1 to 4	0.000563502	1.999248664	99147.11547	3.961418502	73.85422128	0.000630156	1.999159792	99314.57074	3.967580398	80.71260788
5 to 9	0.000464035	2.499033261	98923.89354	4.940461144	70.01636517	0.000445285	2.499072322	99064.55425	4.947717828	76.91126468
10 to 14	0.000659714	2.820729598	98694.64089	4.927647703	65.17319889	0.000462162	2.529846728	98844.24061	4.936576413	72.07712238
15 to 19	0.001488574	2.794335756	98369.55897	4.902382744	60.37925158	0.000513761	2.5875907	98616.09108	4.924701048	67.2380173
20 to 24	0.002797056	2.601299063	97639.80739	4.849454453	55.80957093	0.00068148	2.571851358	98363.07936	4.910029353	62.40429873
25 to 29	0.002907006	2.498158937	96283.40453	4.779410855	51.55899332	0.000753173	2.570256446	98028.47193	4.892470471	57.60850142
30 to 34	0.002853015	2.504986922	94894.05844	4.711168772	47.27715567	0.00094199	2.604779231	97659.98637	4.872007186	52.81613606
35 to 39	0.003062353	2.543670108	93550.00054	4.642580115	42.92028133	0.001237124	2.610515246	97201.05269	4.845728713	48.05316304
40 to 44	0.003596373	2.590536421	92128.35168	4.566847689	38.54319747	0.001614951	2.633620569	96601.58671	4.811691488	43.33508792
45 to 49	0.004774287	2.61959994	90486.06534	4.473469041	34.19556149	0.002303817	2.648382335	95824.54346	4.765410583	38.66504994
50 to 54	0.0065952	2.634688248	88350.53001	4.349682021	29.95858888	0.003317169	2.654287373	94726.72519	4.69976884	34.08234301
55 to 59	0.009548363	2.618522937	85482.24823	4.179099995	25.87518644	0.004890968	2.643614776	93167.82604	4.605319354	29.60805173
60 to 64	0.01300238	2.611097079	81492.68155	3.951911276	22.01349434	0.0069573	2.648042773	90915.57956	4.472601846	25.27584336
65 to 69	0.018403624	2.611990565	76355.55971	3.657108432	18.31865785	0.01041478	2.66921297	87804.25784	4.286187532	21.07741942
70 to 74	0.026765507	2.6048782	69627.47564	3.271730827	14.83624881	0.016804255	2.666601318	83341.17389	4.009873811	17.06292917
75 to 79	0.040118264	2.601712356	60874.7306	2.776742377	11.59496295	0.027365037	2.668302465	76605.01774	3.600608331	13.32854752
80 to 84	0.066321935	2.556197555	49742.32463	2.140475576	8.608161097	0.049193114	2.614626791	66756.80386	2.987503605	9.901061973
85 to 89	0.122336298	2.45704439	35557.90151	1.356316152	6.023668377	0.096881036	2.551821944	52070.5081	2.104731451	6.956607504
90 to 94	0.217184753	2.207984773	18978.70696	0.590884719	4.141901351	0.179656737	2.310353353	31696.16613	1.068781445	4.789691736
95 plus	0.314862396	3.176145313	6153.80589	0.195537593	3.176145313	0.278124794	3.595907117	12509.48914	0.450089265	3.595907117

Table 15: Puerto Rico 2100 life table, by age and sex. mx=mortality rate, ax=mean person-years lived in an age interval among those who die in that age interval, lx=number of persons left alive at age x, nLx=person-years lived between age x and x+n, ex=life expectancy at age x.

Age Group	Male					Female				
	mx	ax	lx	nLx	ex	mx	ax	lx	nLx	ex
Early Neonatal	0.065241487	0.009587041	100000	0.019166092	81.59930149	0.052241548	0.00958744	100000	0.01916848	86.16092814
Late Neonatal	0.009862148	0.028764403	99874.99589	0.05744603	81.68229994	0.012301961	0.02876373	99899.88494	0.057456314	86.2280834
Post Neonatal	0.000707885	0.461593549	99818.35639	0.921309533	81.67105717	0.000618073	0.461599929	99829.21832	0.921447977	86.23151716
1 to 4	4.20494E-05	1.999943933	99753.15565	3.989790831	80.80091246	6.87068E-05	1.999908391	99772.28063	3.990343073	85.35716598
5 to 9	4.19906E-05	2.499912516	99736.38732	4.986296366	76.81411657	6.68622E-05	2.499860702	99744.87537	4.986411994	81.38004004
10 to 14	6.2572E-05	3.361771622	99715.47339	4.985225995	71.82949378	5.80887E-05	2.966550462	99711.63597	4.984920193	76.40620197
15 to 19	0.000397802	2.934957404	99684.31598	4.979925424	66.85080174	0.000128294	2.903529712	99682.71611	4.982723675	71.42750426
20 to 24	0.001038964	2.599964424	99486.38742	4.961932285	61.97669675	0.000256123	2.614826063	99618.89121	4.977892427	66.47137082
25 to 29	0.00115259	2.502049812	98971.78378	4.934472084	57.28238283	0.000262594	2.613716467	99491.53228	4.97144892	61.55288102
30 to 34	0.00116588	2.50916831	98405.52181	4.906066091	52.59431883	0.00039588	2.705095798	99361.20172	4.963396664	56.62970757
35 to 39	0.00115016	2.553832408	97837.48544	4.878150573	47.88285788	0.000519303	2.663141732	99165.233	4.952165153	51.7352367
40 to 44	0.001462324	2.613300964	97280.51467	4.847100066	43.13995078	0.000740961	2.723214411	98908.55124	4.936974369	46.86126827
45 to 49	0.00216341	2.626832555	96576.41887	4.80419344	38.43209654	0.00123948	2.673925689	98543.5858	4.912989937	42.02300864
50 to 54	0.003298543	2.644334159	95544.19402	4.740482509	33.81458281	0.001830456	2.670025718	97935.99427	4.87598446	37.26475186
55 to 59	0.00547742	2.600416516	93992.04726	4.638871325	29.32371238	0.002836733	2.630381432	97046.20191	4.819868819	32.57849432
60 to 64	0.00707506	2.601066708	91475.9243	4.497872554	25.05235908	0.003691133	2.632634634	95684.60931	4.742967877	28.0005195
65 to 69	0.011004509	2.607150578	88320.81176	4.303679704	20.84851704	0.005608898	2.70243378	93941.89891	4.637373217	23.46596799
70 to 74	0.016947686	2.607705998	83636.53536	4.02104875	16.86497874	0.009716354	2.738425623	91360.0067	4.470150445	19.04639632
75 to 79	0.028023679	2.615550562	76938.59664	3.60991678	13.10301195	0.0189631	2.759690748	87056.52366	4.176205502	14.84447989
80 to 84	0.05202203	2.553292013	67042.48307	2.981450867	9.659608993	0.039401812	2.665890243	79263.86864	3.635426615	11.02588092
85 to 89	0.101882587	2.521344342	51923.11491	2.086816225	6.720443865	0.080882044	2.617998683	65301.75246	2.754744263	7.772469562
90 to 94	0.194070748	2.234543654	31383.549	1.032859663	4.528500874	0.158374501	2.329246112	43957.92636	1.566698113	5.305463721
95 plus	0.295529676	3.397562212	11961.13933	0.415228307	3.397562212	0.257671792	3.922540575	20291.46663	0.824169651	3.922540575

Table 15: Saint Lucia 2017 life table, by age and sex. mx=mortality rate, ax=mean person-years lived in an age interval among those who die in that age interval, lx=number of persons left alive at age x, nLx=person-years lived between age x and x+n, ex=life expectancy at age x.

Age Group	Male					Female				
	mx	ax	lx	nLx	ex	mx	ax	lx	nLx	ex
Early Neonatal	0.533608465	0.009572686	100000	0.019080299	72.80994137	0.363951857	0.009577886	100000	0.019111312	77.95986984
Late Neonatal	0.026765911	0.02875974	98982.06596	0.056904778	73.53925543	0.033867456	0.028757781	99304.5154	0.05707849	78.48649811
Post Neonatal	0.003120775	0.461422141	98829.82185	0.911170882	73.59498633	0.002816799	0.461443735	99111.25266	0.913893432	78.58196112
1 to 4	0.000293662	1.999608451	98545.61489	3.939511852	72.88269939	0.000376473	1.999498036	98853.90834	3.951181787	77.86208184
5 to 9	0.000241549	2.499496774	98430.00138	4.918530116	68.96600616	0.000216528	2.4995489	98705.2197	4.93259078	73.97640096
10 to 14	0.000388611	2.879911006	98311.22735	4.911513929	64.04632834	0.000223279	2.619203095	98598.43086	4.927302362	69.05382339
15 to 19	0.000951969	2.792581685	98120.3913	4.895732216	59.16529872	0.000344839	2.698961456	98488.42324	4.920517103	64.12802159
20 to 24	0.001738373	2.662503995	97654.35522	4.862957708	54.43426272	0.00055434	2.605673635	98318.74993	4.909421709	59.23400182
25 to 29	0.002338021	2.581176109	96809.01398	4.813231382	49.88621053	0.000628755	2.616428283	98046.61092	4.894994865	54.39113082
30 to 34	0.002701346	2.5255467	95683.70864	4.752420373	45.4424	0.000910929	2.602105622	97738.84721	4.876291804	49.55410063
35 to 39	0.002736113	2.583006627	94399.97531	4.688992159	41.02585783	0.001083137	2.763112076	97294.67028	4.852975376	44.76836338
40 to 44	0.00389178	2.609711152	93117.09837	4.612947211	36.55531668	0.002301764	2.682039113	96769.05026	4.812775112	39.99640922
45 to 49	0.004937536	2.634599974	91322.01302	4.513394072	32.22235348	0.003144407	2.689299288	95661.33903	4.74856687	35.42829567
50 to 54	0.007386189	2.681880289	89093.81138	4.379712368	27.9620892	0.005298959	2.62890494	94168.3369	4.649999349	30.94713728
55 to 59	0.012107739	2.616594268	85859.46426	4.172588259	23.91411626	0.006683254	2.673866702	91704.63903	4.51505092	26.70761184
60 to 64	0.015564284	2.590976851	80808.63354	3.894452791	20.24497914	0.011540763	2.637028823	88687.66036	4.316692238	22.52486693
65 to 69	0.021661916	2.615875755	74749.09594	3.553982924	16.67589234	0.01558886	2.653547193	83707.11344	4.037713668	18.70777539
70 to 74	0.033293454	2.622374576	67053.67941	3.106878491	13.289395	0.026423856	2.612720415	77415.14603	3.641189606	15.01222724
75 to 79	0.055077597	2.569159656	56715.64238	2.501165871	10.23392821	0.036903134	2.620791536	67799.15216	3.116538121	11.7706469
80 to 84	0.084117008	2.491369456	42950.41152	1.773614652	7.691302838	0.067228155	2.587726445	56307.75221	2.422869389	8.638306152
85 to 89	0.144436853	2.394050451	28045.40334	1.019075475	5.456890263	0.122343234	2.466120851	40036.31278	1.528535363	6.098814044
90 to 94	0.239733448	2.167008772	13339.60061	0.397338736	3.836868097	0.208924547	2.253864964	21356.92815	0.678822461	4.279430879
95 plus	0.333155862	3.001733325	3820.802874	0.114744821	3.001733325	0.305196639	3.276940794	7189.018877	0.235757725	3.276940794

Table 15: Saint Lucia 2100 life table, by age and sex. mx=mortality rate, ax=mean person-years lived in an age interval among those who die in that age interval, lx=number of persons left alive at age x, nLx=person-years lived between age x and x+n, ex=life expectancy at age x.

Age Group	Male					Female				
	mx	ax	lx	nLx	ex	mx	ax	lx	nLx	ex
Early Neonatal	0.142814994	0.009584664	100000	0.019151849	80.48819988	0.094371127	0.009586149	100000	0.01916074	84.24320484
Late Neonatal	0.007293562	0.028765111	99726.59004	0.057364908	80.68957662	0.013069491	0.028763518	99819.21489	0.05740865	84.3764817
Post Neonatal	0.00075661	0.461590087	99684.76027	0.920055763	80.66586375	0.000778099	0.461588561	99744.20129	0.920595229	84.38234475
1 to 4	4.81403E-05	1.999935813	99615.1625	3.984222976	79.79861866	8.52856E-05	1.999886286	99672.57917	3.986223282	83.51932524
5 to 9	5.67634E-05	2.499881754	99595.98705	4.979093559	75.81359591	9.08327E-05	2.499810765	99638.58727	4.980798629	79.54711768
10 to 14	0.000146744	3.179951818	99567.76465	4.976881798	70.83434642	0.00011812	2.645141672	99593.36498	4.978256265	74.58207432
15 to 19	0.000393738	2.773769323	99494.78602	4.970348462	65.8841334	0.000146395	2.699387798	99534.58565	4.975026148	69.62454518
20 to 24	0.000678967	2.702883709	99299.13927	4.957228955	61.00800197	0.000234562	2.670701578	99461.7859	4.970326413	64.67349903
25 to 29	0.001114939	2.640319152	98962.74456	4.935100948	56.20538648	0.000292963	2.665116949	99345.26146	4.96386415	59.74605624
30 to 34	0.001417343	2.531013451	98413.61662	4.903459633	51.50204806	0.00046199	2.600216736	99199.88176	4.954480935	54.82959382
35 to 39	0.001244226	2.580058958	97720.39059	4.871336793	46.8481083	0.000493963	2.768724867	98971.15988	4.943046358	49.94986008
40 to 44	0.001886394	2.610774932	97115.88022	4.833972754	42.12204387	0.001040898	2.706275017	98727.16465	4.924551298	45.06598295
45 to 49	0.002234344	2.688299344	96206.11985	4.78559933	37.49356807	0.001484165	2.695203437	98214.92661	4.894002194	40.28621761
50 to 54	0.004052933	2.756142875	95139.49449	4.713860515	32.88123359	0.002454158	2.667922096	97489.24296	4.846632783	35.56506669
55 to 59	0.007297687	2.596365218	93236.5022	4.581794963	28.49146483	0.003395983	2.672472717	96302.01629	4.777402565	30.96906357
60 to 64	0.008225494	2.625488076	89906.82928	4.409522821	24.44389977	0.005458109	2.669280672	94682.646	4.674312759	26.45159936
65 to 69	0.012563603	2.642229329	86328.6735	4.19284553	20.33601699	0.007357779	2.716182952	92148.75242	4.531095275	22.10359893
70 to 74	0.018326692	2.696554417	81112.35111	3.892882886	16.46764839	0.013380883	2.720566528	88828.69436	4.309933213	17.8277423
75 to 79	0.036410684	2.619362929	74068.0453	3.414971247	12.76338093	0.023255085	2.727270239	83086.46733	3.946051673	13.86988878
80 to 84	0.053030474	2.542877595	62009.35137	2.757747356	9.707761728	0.046593465	2.667046091	73956.91664	3.338576428	10.24156445
85 to 89	0.102440435	2.521466014	48086.52315	1.9421031	6.764768392	0.092517351	2.571131904	58591.39095	2.401435972	7.199124825
90 to 94	0.193869811	2.228511777	29450.65214	0.97819151	4.557963805	0.173632024	2.315004492	36885.93348	1.268760677	4.943035052
95 plus	0.295185567	3.414553033	11564.19728	0.410462229	3.414553033	0.272269984	3.693137382	15425.08398	0.581263203	3.693137382

Table 15: Saint Vincent and the Grenadines 2017 life table, by age and sex. mx=mortality rate, ax=mean person-years lived in an age interval among those who die in that age interval, lx=number of persons left alive at age x, nLx=person-years lived between age x and x+n, ex=life expectancy at age x.

Age Group	Male					Female				
	mx	ax	lx	nLx	ex	mx	ax	lx	nLx	ex
Early Neonatal	0.584079098	0.009571139	100000	0.019071083	69.96758952	0.300731697	0.009579824	100000	0.019122887	75.68054114
Late Neonatal	0.047848386	0.028753924	98886.30112	0.056815279	70.73600613	0.047722344	0.028753959	99424.96067	0.057124958	76.09891003
Post Neonatal	0.004489975	0.461324875	98614.56065	0.908612366	70.87332582	0.005183746	0.461275591	99152.40148	0.913275258	76.25048284
1 to 4	0.000527569	1.999296574	98206.80628	3.924132946	70.2424735	0.000408865	1.999454847	98679.1252	3.943940345	75.69076127
5 to 9	0.000434564	2.499094659	97999.91635	4.894677862	66.38661713	0.000201881	2.499579415	98517.93729	4.923411993	71.81137709
10 to 14	0.000548837	2.697402419	97787.27563	4.883192009	61.52557921	0.000643862	2.681614655	98418.5592	4.913593619	66.8813764
15 to 19	0.000957109	2.824891493	97519.3176	4.865836597	56.68723907	0.000765988	2.517572314	98102.21702	4.895802045	62.08838371
20 to 24	0.002058902	2.586188478	97053.63124	4.82868487	51.94564709	0.000713651	2.662808545	97727.21636	4.878224741	57.31691946
25 to 29	0.001833757	2.640773395	96059.49299	4.782285689	47.45638964	0.001333356	2.661126578	97379.0933	4.853818444	52.51225114
30 to 34	0.003360683	2.622011972	95182.55971	4.721398317	42.86919892	0.001762197	2.594003185	96731.9327	4.816178223	47.8456661
35 to 39	0.003896054	2.60853626	93595.9233	4.636599126	38.55133442	0.002160471	2.565243481	95883.26871	4.76907844	43.2460592
40 to 44	0.00558236	2.607781799	91789.59376	4.529004549	34.25848162	0.002484035	2.714524136	94852.97802	4.71587667	38.6877558
45 to 49	0.007098516	2.588849639	89261.5818	4.387986094	30.15464534	0.004827896	2.653488807	93681.6237	4.631615107	34.13737744
50 to 54	0.009149825	2.613233781	86147.17538	4.215317391	26.15094844	0.006258839	2.549257155	91445.75194	4.503222357	29.90689323
55 to 59	0.013056753	2.626073314	82290.92446	3.990871557	22.25368915	0.006650143	2.685721668	88627.65413	4.364226813	25.77650265
60 to 64	0.01900627	2.627364991	77081.36854	3.687811696	18.57999262	0.012991726	2.674378432	85725.92394	4.1606171	21.55785459
65 to 69	0.028968081	2.618904544	70074.28611	3.27771265	15.17503411	0.019343145	2.621724549	80321.89289	3.839525509	17.82796597
70 to 74	0.045546468	2.581228851	60582.97208	2.72868348	12.14218283	0.028549221	2.626736802	72897.75677	3.413713317	14.37628086
75 to 79	0.070011591	2.491399299	48160.91576	2.048495275	9.608635271	0.047119054	2.57862347	63157.27738	2.83469375	11.18823807
80 to 84	0.085513563	2.405931238	33828.18545	1.384524318	7.625393429	0.069616955	2.519375724	49811.15915	2.12411301	8.495594272
85 to 89	0.145389662	2.391423532	21997.61169	0.797605127	5.434356636	0.125137047	2.45743532	35038.9183	1.329441007	6.016791384
90 to 94	0.240681729	2.165173834	10409.06617	0.309443437	3.824793685	0.212007815	2.247294215	18420.40917	0.581825735	4.231316385
95 plus	0.333917958	2.99482883	2965.061688	0.088827802	2.99482883	0.308005238	3.247006073	6096.767756	0.198099819	3.247006073

Table 15: Saint Vincent and the Grenadines 2100 life table, by age and sex. mx=mortality rate, ax=mean person-years lived in an age interval among those who die in that age interval, lx=number of persons left alive at age x, nLx=person-years lived between age x and x+n, ex=life expectancy at age x.

Age Group	Male					Female				
	mx	ax	lx	nLx	ex	mx	ax	lx	nLx	ex
Early Neonatal	0.149567395	0.009584457	100000	0.019150607	76.49800152	0.06629835	0.009587009	100000	0.019165896	82.37247632
Late Neonatal	0.013831269	0.028763308	99713.63385	0.05734667	76.69842872	0.019038546	0.028761872	99872.9471	0.057429691	82.45794871
Post Neonatal	0.001110524	0.461564946	99634.32627	0.919440093	76.70186942	0.001489487	0.461538025	99763.62241	0.920472213	82.49066745
1 to 4	0.000126054	1.999831928	99532.24261	3.980286929	75.85660119	0.000113755	1.999848327	99626.53533	3.984155625	81.6800498
5 to 9	0.000163699	2.49965896	99482.11721	4.972073629	71.89332315	0.000105325	2.499780574	99581.25915	4.977755064	77.71589351
10 to 14	0.000261797	2.806236125	99400.85327	4.96711755	66.94947323	0.000483855	2.883573201	99528.9635	4.970798668	72.75492588
15 to 19	0.000473399	2.853343589	99271.10491	4.958470541	62.03278414	0.000441655	2.4702978	99289.46952	4.958906037	67.92256784
20 to 24	0.001012955	2.625546427	99036.91487	4.940013653	57.17117038	0.000372696	2.70845462	99070.9729	4.949257238	63.06628729
25 to 29	0.001117837	2.711653966	98537.54405	4.914407337	52.444673	0.000753132	2.769435563	98887.17794	4.935821919	58.17759366
30 to 34	0.002232392	2.667188965	97990.30763	4.873974047	47.71832873	0.001098534	2.589665836	98516.67924	4.912732941	53.38468117
35 to 39	0.00262284	2.600433248	96909.6352	4.815202853	43.21410782	0.001104202	2.539092709	97978.54018	4.885663993	48.66225547
40 to 44	0.003423847	2.63103211	95654.17008	4.744206163	38.74357142	0.00131594	2.737389631	97439.82374	4.857587196	43.91640713
45 to 49	0.004588812	2.608636085	94041.79135	4.651152578	34.35877299	0.002708312	2.687615319	96801.81422	4.809695925	39.18616388
50 to 54	0.005844004	2.639863297	91918.90528	4.533611537	30.08879984	0.003423531	2.54133715	95506.15156	4.735421624	34.67501886
55 to 59	0.008847584	2.634346803	89281.64857	4.372965471	25.89593155	0.003244623	2.727163561	93893.45717	4.660348896	30.22205621
60 to 64	0.012382063	2.646083663	85431.871	4.151484566	21.93901643	0.007202752	2.718152787	92387.16406	4.544584526	25.66756567
65 to 69	0.01948322	2.667066384	80333.42302	3.843545153	18.1530629	0.010578083	2.635402849	89146.72944	4.349150397	21.48975502
70 to 74	0.03157342	2.62735269	72954.58222	3.396912814	14.70842247	0.015040071	2.76134157	84593.32088	4.092133386	17.49467356
75 to 79	0.05001543	2.522672344	62410.52368	2.785420307	11.74153794	0.030785724	2.662715166	78567.65178	3.668969144	13.61513609
80 to 84	0.057986357	2.455632572	48894.80293	2.143019095	9.292557519	0.046967835	2.575743888	67566.20756	3.044622717	10.39129559
85 to 89	0.109419553	2.498884687	37012.91639	1.469980281	6.497399447	0.091824405	2.578500661	53829.3517	2.225444816	7.336148657
90 to 94	0.202151377	2.222665162	21783.52964	0.710114047	4.406289059	0.171663815	2.309736694	34650.21292	1.211309864	5.037283695
95 plus	0.302183898	3.327605514	8095.754379	0.278105597	3.327605514	0.270130033	3.753780517	15218.50284	0.601048539	3.753780517

Table 15: Suriname 2017 life table, by age and sex. mx=mortality rate, ax=mean person-years lived in an age interval among those who die in that age interval, lx=number of persons left alive at age x, nLx=person-years lived between age x and x+n, ex=life expectancy at age x.

Age Group	Male					Female				
	mx	ax	lx	nLx	ex	mx	ax	lx	nLx	ex
Early Neonatal	0.859368585	0.009562702	100000	0.019020919	68.7710451	0.608466999	0.009570392	100000	0.019066623	75.11402799
Late Neonatal	0.07552789	0.028746289	98365.55839	0.056471124	69.89413727	0.065549142	0.028749042	98839.91307	0.056759714	75.97624247
Post Neonatal	0.009539822	0.460966143	97939.13626	0.900290361	70.14079541	0.00836139	0.461049857	98467.89829	0.905642202	76.20563971
1 to 4	0.000829256	1.998894325	97080.52513	3.876789967	69.8338739	0.000649233	1.999134355	97710.77111	3.903361192	75.86932252
5 to 9	0.000321804	2.499329575	96759.1528	4.834068573	66.05924068	0.000353688	2.499263151	97457.39896	4.868564314	72.061403
10 to 14	0.000556207	2.792597965	96603.63209	4.824259059	61.16158834	0.000419765	2.761735016	97285.22452	4.859695925	67.18452762
15 to 19	0.001107633	2.691493369	96335.34661	4.804484351	56.32409205	0.000884865	2.59708412	97081.24421	4.843764154	62.31984951
20 to 24	0.001588276	2.671114781	95803.22183	4.772508345	51.62168417	0.000837773	2.608657464	96652.65066	4.82297125	57.58453272
25 to 29	0.002443233	2.615223141	95045.26913	4.724736852	47.01162392	0.001333364	2.629887056	96248.615	4.797271567	52.8151604
30 to 34	0.002995715	2.591519716	93891.03007	4.660929217	42.55683299	0.001686482	2.605644463	95609.00961	4.761227437	48.15064427
35 to 39	0.003859812	2.590860748	92494.97863	4.582153058	38.15938207	0.002221456	2.580136129	94806.11702	4.714963633	43.53605887
40 to 44	0.004836457	2.598257789	90726.81988	4.484272597	33.8517216	0.002588359	2.604518952	93758.86142	4.659058681	38.99311167
45 to 49	0.006399913	2.665884871	88558.8829	4.362805058	29.61584989	0.003599151	2.633183995	92553.16639	4.588577907	34.46674686
50 to 54	0.010493307	2.644482945	85768.31101	4.185045326	25.49161135	0.005032184	2.68795661	90902.07748	4.492845416	30.04448653
55 to 59	0.014850042	2.609177186	81380.27312	3.929630302	21.72219902	0.008492525	2.657724922	88641.9133	4.345685437	25.74147071
60 to 64	0.02068024	2.619263241	75550.95444	3.600503971	18.19575661	0.012245717	2.618422737	84952.91407	4.127342525	21.74312054
65 to 69	0.03193784	2.575833858	68115.1024	3.161420139	14.89545785	0.01708999	2.648292536	79901.66841	3.84084224	17.95138142
70 to 74	0.042287859	2.559051861	58036.55971	2.630925513	12.03497739	0.028303415	2.634960842	73343.2471	3.437346499	14.3190963
75 to 79	0.065581314	2.541141227	46938.98603	2.021977576	9.277164603	0.044793447	2.59969262	63626.71229	2.872983226	11.10303235
80 to 84	0.102798835	2.463566448	33722.74608	1.33854222	6.922322687	0.07395025	2.535878762	50781.48391	2.148502844	8.254992053
85 to 89	0.16629942	2.336340375	20016.72127	0.694466551	4.986060071	0.130981282	2.439843525	34931.3548	1.308863652	5.853890076
90 to 94	0.260933781	2.124069496	8510.356807	0.243436804	3.584954827	0.218366969	2.233408304	17831.5123	0.556336358	4.136107107
95 plus	0.350003131	2.857555277	2176.286983	0.062307215	2.857555277	0.313761546	3.1878693	5710.553423	0.182355192	3.1878693

Table 15: Suriname 2100 life table, by age and sex. mx=mortality rate, ax=mean person-years lived in an age interval among those who die in that age interval, lx=number of persons left alive at age x, nLx=person-years lived between age x and x+n, ex=life expectancy at age x.

Age Group	Male					Female				
	mx	ax	lx	nLx	ex	mx	ax	lx	nLx	ex
Early Neonatal	0.167797568	0.009583898	100000	0.019147261	77.03252523	0.136316921	0.009584863	100000	0.019153038	82.4152483
Late Neonatal	0.016318705	0.028762622	99678.77086	0.057322519	77.26114873	0.020704997	0.028761412	99738.94905	0.057349891	82.61131168
Post Neonatal	0.002061622	0.461497381	99585.24121	0.918583911	77.27606354	0.002103142	0.461494432	99620.22392	0.918888948	82.65207438
1 to 4	0.000195699	1.999739067	99395.91078	3.974280972	76.49872693	0.000169399	1.999774134	99427.00635	3.975733317	81.88819268
5 to 9	0.000104306	2.499782696	99318.14877	4.96461335	72.55697745	0.000144307	2.499699361	99359.66773	4.966193464	77.94228406
10 to 14	0.000252947	3.108674835	99266.39539	4.960706023	67.59327251	0.000224872	2.833744998	99288.10923	4.961893964	72.99649978
15 to 19	0.000623468	2.695229853	99141.0321	4.949945821	62.67460824	0.000451333	2.599077298	99176.62808	4.953393413	68.07516782
20 to 24	0.000858971	2.71624699	98832.69337	4.931827648	57.85937675	0.000369311	2.641910143	98953.55841	4.943373162	63.22163896
25 to 29	0.001389601	2.627405656	98409.94533	4.904288642	53.09251128	0.000669936	2.658578298	98771.21394	4.930792765	58.33293812
30 to 34	0.001668141	2.560893023	97730.03732	4.86678268	48.43986117	0.000840578	2.641744253	98441.84855	4.912234478	53.5176509
35 to 39	0.001941431	2.550706043	96920.89647	4.823380918	43.81887201	0.001098724	2.572120357	98030.495	4.888497924	48.72936013
40 to 44	0.002360145	2.622433558	95989.62068	4.772787278	39.21325983	0.001214021	2.632616569	97494.95221	4.860820172	43.98075869
45 to 49	0.003271663	2.755928005	94873.77803	4.708917657	34.63505655	0.001917105	2.670355363	96906.879	4.823581011	39.2292217
50 to 54	0.006051052	2.695284575	93349.17644	4.603295837	30.14710583	0.002508139	2.742447906	95987.17547	4.772299393	34.57618753
55 to 59	0.008674978	2.61147789	90594.64057	4.438366814	25.97159604	0.004883516	2.633957888	94794.25744	4.685700715	29.97436308
60 to 64	0.011377807	2.636510507	86777.18088	4.226574932	21.99069057	0.005788403	2.600493365	92518.14406	4.562902391	25.64207786
65 to 69	0.018146	2.644940398	82027.57546	3.93569655	18.09643252	0.008128367	2.725332169	89894.62225	4.413302305	21.3063792
70 to 74	0.026830434	2.644387814	75033.1226	3.533176764	14.52382649	0.015369199	2.728066878	86339.14326	4.17227445	17.06483727
75 to 79	0.047452972	2.608342977	65772.78904	2.966558267	11.18056903	0.027279751	2.709933866	80022.14996	3.768507693	13.18737545
80 to 84	0.075788636	2.506973345	52372.1998	2.227252069	8.373216651	0.053467791	2.609584462	69917.81067	3.108215741	9.699294426
85 to 89	0.132084743	2.435014549	36668.34654	1.401371529	5.900125291	0.102389596	2.536277289	53710.43833	2.159560206	6.829204503
90 to 94	0.225552415	2.184558647	19773.22697	0.624526463	4.080116835	0.185374652	2.295429922	32371.34551	1.091771335	4.718799304
95 plus	0.321185588	3.14074759	6746.243809	0.223121325	3.14074759	0.283231255	3.552381711	12845.82633	0.46880852	3.552381711

Table 15: Trinidad and Tobago 2017 life table, by age and sex. mx=mortality rate, ax=mean person-years lived in an age interval among those who die in that age interval, lx=number of persons left alive at age x, nLx=person-years lived between age x and x+n, ex=life expectancy at age x.

Age Group	Male					Female				
	mx	ax	lx	nLx	ex	mx	ax	lx	nLx	ex
Early Neonatal	0.466817336	0.009574733	100000	0.019092499	71.03400543	0.345039528	0.009578466	100000	0.019114773	77.26168754
Late Neonatal	0.071373108	0.028747435	99108.86974	0.056904668	71.65328211	0.050690005	0.02875314	99340.5272	0.05707158	77.7552406
Post Neonatal	0.005059192	0.461284439	98702.88162	0.909187524	71.89042077	0.004615288	0.461315973	99051.29862	0.912583287	77.92468148
1 to 4	0.000650741	1.999132345	98243.15395	3.924619307	71.30156011	0.000518537	1.999308618	98630.25494	3.941123154	77.33216691
5 to 9	0.000304815	2.499364968	97987.92563	4.89566593	67.48220682	0.000234314	2.499511847	98425.97517	4.918417596	73.48858458
10 to 14	0.000349145	3.006839599	97838.74974	4.888538595	62.58132838	0.00026329	2.678235876	98310.75128	4.912537017	68.57179795
15 to 19	0.001160965	2.835512704	97668.10191	4.87116791	57.68530809	0.000462782	2.678713476	98181.42082	4.903805814	63.65847534
20 to 24	0.00224202	2.621158942	97102.62043	4.829375763	53.00377497	0.000664978	2.642868883	97954.49375	4.890062223	58.79938068
25 to 29	0.002502751	2.549902436	96020.04367	4.771748816	48.57005684	0.000925496	2.610833015	97629.3472	4.870700182	53.98584346
30 to 34	0.002906322	2.532642257	94826.16503	4.707570484	44.14776389	0.001167045	2.618400128	97178.64439	4.845472068	49.22331369
35 to 39	0.003041651	2.558586762	93458.66087	4.638518982	39.75485565	0.001608751	2.63829653	96613.30968	4.812388479	44.49504228
40 to 44	0.003877348	2.627473267	92048.86089	4.560536007	35.32261495	0.002264087	2.657073103	95839.46304	4.766695324	39.83157545
45 to 49	0.005598528	2.650686309	90282.50636	4.455603335	30.96000711	0.003369913	2.670252244	94760.93096	4.701159542	35.25294992
50 to 54	0.008293968	2.653854309	87791.83008	4.305953916	26.76028485	0.005148242	2.66819951	93178.05652	4.603692123	30.80427896
55 to 59	0.012529318	2.645315621	84227.91209	4.090992462	22.77699363	0.007837416	2.666329529	90810.79391	4.459103271	26.53495949
60 to 64	0.018844017	2.61986809	79116.26229	3.786539299	19.07434143	0.012149992	2.631641425	87321.90589	4.244239735	22.48519137
65 to 69	0.027298648	2.60063579	72006.05498	3.379930246	15.69647554	0.017108663	2.620044486	82177.47088	3.948644902	18.72413557
70 to 74	0.040529061	2.580308051	62823.34865	2.862268882	12.60983093	0.025465898	2.624948062	75446.03132	3.558197979	15.15735112
75 to 79	0.061555535	2.529907098	51299.46669	2.229060777	9.866369905	0.040258499	2.598801995	66434.06973	3.030915336	11.85543299
80 to 84	0.088113405	2.468523138	37701.4813	1.544392921	7.525561684	0.063033484	2.547857599	54328.81266	2.355998841	8.92142106
85 to 89	0.148952942	2.38209617	24239.80249	0.87466592	5.35935334	0.116211	2.486408946	39632.07842	1.537671402	6.298736176
90 to 94	0.244115112	2.15810965	11342.01363	0.336020859	3.784721129	0.201947043	2.267763606	21959.65811	0.710304857	4.39783307
95 plus	0.336639418	2.971893932	3202.083726	0.095650327	2.971893932	0.298763785	3.350863497	7755.7806	0.26178805	3.350863497

Table 15: Trinidad and Tobago 2100 life table, by age and sex. mx=mortality rate, ax=mean person-years lived in an age interval among those who die in that age interval, lx=number of persons left alive at age x, nLx=person-years lived between age x and x+n, ex=life expectancy at age x.

Age Group	Male					Female				
	mx	ax	lx	nLx	ex	mx	ax	lx	nLx	ex
Early Neonatal	0.107163284	0.009585757	100000	0.019158394	79.6866805	0.086200281	0.009586399	100000	0.019162242	85.47447363
Late Neonatal	0.020763087	0.028761396	99794.78652	0.057381905	79.831346	0.017183768	0.028762383	99834.87598	0.057410864	85.59638321
Post Neonatal	0.001178416	0.461560123	99675.67458	0.919792928	79.86908894	0.001263503	0.461554078	99736.24157	0.92031564	85.62335845
1 to 4	0.000166574	1.999777901	99567.33051	3.981367201	79.0320366	0.00014229	1.999810279	99619.99481	3.983666418	84.7991656
5 to 9	9.32632E-05	2.499805702	99501.03835	4.973892661	75.0832757	0.00010043	2.49979077	99563.3325	4.976917634	80.84617867
10 to 14	0.000139206	3.102664856	99454.67648	4.971331856	70.11697221	0.000139388	2.907548813	99513.38326	4.974072014	75.8853954
15 to 19	0.000625796	2.850288166	99385.53666	4.962275862	65.1635465	0.000250476	2.657772791	99444.17633	4.969244227	70.93597237
20 to 24	0.001234995	2.617626911	99075.75854	4.939155089	60.35359071	0.000293669	2.724219087	99319.82216	4.962620508	66.02127499
25 to 29	0.001280074	2.564764337	98470.02779	4.908193705	55.70201917	0.000514225	2.657036952	99174.24994	4.952695164	61.11391744
30 to 34	0.001537769	2.5231873	97848.06979	4.873968059	51.03595239	0.000641316	2.616910084	98920.05656	4.938368657	56.26308654
35 to 39	0.001400374	2.535375361	97107.6093	4.838834715	46.40331851	0.000785777	2.605164578	98604.25314	4.920941971	51.43343338
40 to 44	0.001685981	2.66311798	96440.58164	4.802942679	41.70498856	0.0010385	2.667362156	98218.44999	4.898858174	46.62349489
45 to 49	0.002462838	2.677363073	95641.98621	4.75483012	37.02771075	0.001437474	2.653706257	97711.19101	4.869101096	41.84990216
50 to 54	0.003749523	2.677295416	94484.37718	4.683359786	32.4429178	0.002098504	2.658610318	97012.86009	4.826830238	37.12902536
55 to 59	0.005876719	2.675420219	92744.96883	4.574708071	27.99283172	0.002997944	2.648960197	96003.1433	4.766577373	32.48758969
60 to 64	0.009176433	2.678853723	90090.75007	4.410843958	23.72589625	0.00441011	2.666487214	94579.47327	4.680699117	27.93190304
65 to 69	0.014600316	2.642047425	86119.41776	4.164377065	19.68109517	0.006408146	2.686666478	92532.25797	4.559011887	23.48259918
70 to 74	0.02255641	2.631882377	80160.49648	3.808955168	15.93643348	0.010384878	2.758979225	89634.63432	4.379975073	19.14839756
75 to 79	0.038325615	2.577970985	71796.74987	3.295593877	12.47557155	0.020785018	2.729556737	85142.76835	4.066752904	15.00297293
80 to 84	0.058024442	2.52622673	59702.05961	2.632100102	9.466528752	0.037571161	2.6135032	76878.99878	3.537088026	11.30939416
85 to 89	0.1085344	2.504587443	45475.07747	1.823930659	6.615545825	0.077479609	2.633522059	64047.40938	2.728172873	7.993872764
90 to 94	0.200014923	2.217126618	27451.08603	0.908843829	4.477768565	0.15338253	2.329184694	44119.53791	1.594588878	5.451667184
95 plus	0.300142553	3.368625321	10711.63102	0.380842805	3.368625321	0.252784683	4.015731116	21191.04124	0.891402647	4.015731116

Table 15: Virgin Islands, U.S. 2017 life table, by age and sex. mx=mortality rate, ax=mean person-years lived in an age interval among those who die in that age interval, lx=number of persons left alive at age x, nLx=person-years lived between age x and x+n, ex=life expectancy at age x.

Age Group	Male					Female				
	mx	ax	lx	nLx	ex	mx	ax	lx	nLx	ex
Early Neonatal	0.288776029	0.00958019	100000	0.019125078	69.51099674	0.182552346	0.009583446	100000	0.019144551	78.69364643
Late Neonatal	0.030915731	0.028758595	99447.76594	0.057165678	69.87777372	0.022291212	0.028760974	99650.52961	0.057296436	78.95035269
Post Neonatal	0.002080355	0.461496051	99271.07299	0.91567813	69.94459907	0.001646691	0.461526857	99522.82436	0.918183906	78.99408189
1 to 4	0.000379269	1.999494308	99080.63239	3.960221489	69.15491698	0.000361212	1.999518384	99371.65171	3.971996454	78.19026611
5 to 9	0.000291311	2.499393102	98930.48114	4.942923479	65.25689231	0.000168691	2.49964856	99228.20645	4.959318627	74.30041842
10 to 14	0.000321733	2.905418201	98786.49346	4.93599518	60.34822007	0.000186225	2.768996236	99144.55046	4.955168876	69.36096592
15 to 19	0.000917298	2.746003851	98627.69663	4.921211118	55.44064564	0.000409879	2.673489553	99052.2768	4.947895533	64.42297783
20 to 24	0.001414914	2.598764361	98176.30359	4.892196489	50.68297396	0.000528685	2.59954516	98849.4872	4.936212063	59.5496159
25 to 29	0.001606662	2.574795846	97484.17273	4.855290827	46.02463811	0.000667341	2.618917262	98588.52566	4.921611866	54.70026743
30 to 34	0.00201903	2.627669844	96704.18088	4.812162746	41.37528682	0.000920358	2.642544026	98260.10029	4.902371244	49.87413292
35 to 39	0.00289346	2.672610112	95732.69647	4.754630752	36.76840794	0.001308787	2.658945182	97808.95463	4.875506443	45.09161279
40 to 44	0.004534191	2.691625338	94357.11473	4.669005279	32.26467887	0.001933484	2.66789126	97170.99116	4.836736482	40.36975533
45 to 49	0.007337058	2.678375805	92240.60358	4.534813777	27.94143509	0.002901714	2.667324376	96236.10212	4.779454404	35.73551297
50 to 54	0.011464124	2.649224466	88915.10764	4.329166027	23.88363012	0.004353012	2.664522302	94849.74664	4.694767629	31.21833732
55 to 59	0.017038664	2.625588696	83956.51795	4.034794087	20.13452539	0.006555327	2.665039322	92806.9286	4.570412524	26.8463138
60 to 64	0.025055247	2.603246666	77090.83607	3.636618166	16.6903868	0.010059206	2.65898393	89812.21413	4.387339752	22.65204287
65 to 69	0.036475734	2.580920072	67999.33282	3.125101524	13.57007692	0.01543847	2.655827668	85401.09752	4.121005439	18.68412262
70 to 74	0.054169593	2.547228386	56638.9578	2.501242736	10.7720119	0.024551191	2.64511522	79043.04184	3.736346981	14.97269724
75 to 79	0.080634959	2.496531072	43158.21059	1.797633931	8.343311057	0.039993015	2.622885499	69878.98692	3.191088525	11.58833285
80 to 84	0.121946051	2.417096806	28767.34291	1.096549443	6.279717032	0.068242466	2.568656611	57138.7255	2.451489077	8.586469361
85 to 89	0.187145236	2.285510195	15528.95081	0.516860319	4.602983405	0.123540284	2.462714156	40464.72778	1.54196129	6.068449418
90 to 94	0.280386707	2.082902414	5955.340153	0.164531873	3.379426427	0.210195663	2.250965821	21493.4691	0.682181063	4.261821146
95 plus	0.365173288	2.739557957	1381.403071	0.038078616	2.739557957	0.306334697	3.266039917	7209.760521	0.236157263	3.266039917

Table 15: Virgin Islands, U.S. 2100 life table, by age and sex. mx=mortality rate, ax=mean person-years lived in an age interval among those who die in that age interval, lx=number of persons left alive at age x, nLx=person-years lived between age x and x+n, ex=life expectancy at age x.

Age Group	Male					Female				
	mx	ax	lx	nLx	ex	mx	ax	lx	nLx	ex
Early Neonatal	0.08139602	0.009586546	100000	0.019163123	74.22887778	0.055017124	0.009587355	100000	0.019167969	82.98187084
Late Neonatal	0.009832166	0.028764411	99844.05264	0.057428281	74.32559964	0.008990136	0.028764643	99894.55408	0.057458719	83.05020356
Post Neonatal	0.000560486	0.46160402	99787.59539	0.921088256	74.31008079	0.000552204	0.461604608	99842.9029	0.921602282	83.03559171
1 to 4	0.000101659	1.999864455	99735.97862	3.988628339	73.42498392	0.000108456	1.999855392	99792.01768	3.990815077	82.15437813
5 to 9	9.11882E-05	2.499810025	99695.44169	4.983636006	69.45399869	5.47688E-05	2.499885899	99748.73978	4.986754207	78.18912612
10 to 14	0.000100081	2.947700439	99650.00248	4.981465425	64.48452206	7.69665E-05	2.901733952	99721.42987	4.985260838	73.20984249
15 to 19	0.000295369	2.848013694	99600.15202	4.97683544	59.51532733	0.000197927	2.707428544	99683.06298	4.981893677	68.23689341
20 to 24	0.000586242	2.651153338	99453.1627	4.965826549	54.59870682	0.000275346	2.613549401	99584.46501	4.975963713	63.30172209
25 to 29	0.000729323	2.622701225	99162.07756	4.94954778	49.75068519	0.000358355	2.643611981	99447.46923	4.968174317	58.38521936
30 to 34	0.001044601	2.659017631	98801.22775	4.928019011	44.92169752	0.000518761	2.648303073	99269.50004	4.957398032	53.48488624
35 to 39	0.001539371	2.709747394	98286.87197	4.897060757	40.14110197	0.000699285	2.664111352	99012.4632	4.942542832	48.61654042
40 to 44	0.002586893	2.750443225	97534.09359	4.848451891	35.4271769	0.001065607	2.705885903	98666.99727	4.921287509	43.77699005
45 to 49	0.004659534	2.726670525	96282.16126	4.763704964	30.8478199	0.001728869	2.690930998	98142.92655	4.887605616	38.99568164
50 to 54	0.00789524	2.684005739	94067.53339	4.619120176	26.50385151	0.002643242	2.682953387	97298.54225	4.835300704	34.30994958
55 to 59	0.012305539	2.639524064	90433.55407	4.394576957	22.45220622	0.004102689	2.659807248	96021.40151	4.755414206	29.72966496
60 to 64	0.017719244	2.629205414	85054.29138	4.08240451	18.69532745	0.00594374	2.672513772	94072.00427	4.639463331	25.28944233
65 to 69	0.026944235	2.62209182	77877.51827	3.661656185	15.16516211	0.009468447	2.684803988	91316.91405	4.467992094	20.97020979
70 to 74	0.041767769	2.598808125	68125.80658	3.100205423	11.9530484	0.015445648	2.709762591	87092.13771	4.206062654	16.8549674
75 to 79	0.066970217	2.556666151	55393.96258	2.387891551	9.103310728	0.028427847	2.700059074	80609.4637	3.783842618	12.98936193
80 to 84	0.111349562	2.46670732	39780.87774	1.563260575	6.695461192	0.054356847	2.628448739	69893.83067	3.098619817	9.562018512
85 to 89	0.175474652	2.315935478	22959.17293	0.791556838	4.846865152	0.104141599	2.528051993	53206.23252	2.122036507	6.725110821
90 to 94	0.269165343	2.105981282	9636.582666	0.275778814	3.509826175	0.187863833	2.293621575	31441.17533	1.048259338	4.653109512
95 plus	0.356288489	2.814194829	2478.375371	0.071570926	2.814194829	0.28567555	3.510709273	12065.41207	0.428954785	3.510709273

Table 15: Central Latin America 2017 life table, by age and sex. mx=mortality rate, ax=mean person-years lived in an age interval among those who die in that age interval, lx=number of persons left alive at age x, nLx=person-years lived between age x and x+n, ex=life expectancy at age x.

Age Group	Male					Female				
	mx	ax	lx	nLx	ex	mx	ax	lx	nLx	ex
Early Neonatal	0.30194969	0.009579786	100000	0.019122661	73.21425997	0.222653238	0.009582217	100000	0.019137195	79.31123359
Late Neonatal	0.038342023	0.028756547	99422.59538	0.057138995	73.62021339	0.028977846	0.02875913	99573.90556	0.057241367	79.63139929
Post Neonatal	0.006133401	0.461208129	99203.51639	0.913345351	73.72519489	0.005173398	0.461276326	99408.03372	0.915633624	79.70668956
1 to 4	0.000706157	1.999058457	98643.33556	3.940166192	73.21796384	0.000667834	1.999109555	98934.34559	3.952092868	79.16282408
5 to 9	0.000321128	2.499330984	98365.10524	4.914308965	69.41941357	0.00024746	2.499484458	98670.41546	4.930469928	75.36923024
10 to 14	0.00038889	3.122039423	98207.29561	4.90678134	64.52694892	0.000277492	2.681620685	98548.40684	4.924252455	70.459448
15 to 19	0.001489467	2.803441721	98016.47737	4.884842305	59.64648865	0.000490651	2.636611952	98411.763	4.914888916	65.55355474
20 to 24	0.002586852	2.603562643	97288.90015	4.834475095	55.07155031	0.000601462	2.57630271	98170.61403	4.901385712	60.70809827
25 to 29	0.002829373	2.519871504	96038.30583	4.768454266	50.75471651	0.000714683	2.57894739	97875.81475	4.885337788	55.8831799
30 to 34	0.002934156	2.523756496	94689.15471	4.700307311	46.44192285	0.00087776	2.612246051	97526.6694	4.866134753	51.07399643
35 to 39	0.003254488	2.550607859	93310.03905	4.628605771	42.09098661	0.001196573	2.63906855	97099.54178	4.841300288	46.28715823
40 to 44	0.003836984	2.577029818	91803.70383	4.547904998	37.73973067	0.001692931	2.6555106	96520.24934	4.806933474	41.54910321
45 to 49	0.004833229	2.615707462	90058.7328	4.451638705	33.42099846	0.002494147	2.679135571	95706.4769	4.757783259	36.87978324
50 to 54	0.006796601	2.632588186	87907.23809	4.325762234	29.1748975	0.003916113	2.677057517	94519.83159	4.683387871	32.30912296
55 to 59	0.009685861	2.633370837	84967.33924	4.153170295	25.09321286	0.006006917	2.669854436	92685.79666	4.570320519	27.89543005
60 to 64	0.014082673	2.626003023	80944.89993	3.916323948	21.20926126	0.009252047	2.655051384	89940.51253	4.401535108	23.66534331
65 to 69	0.020463287	2.613372132	75430.12978	3.595907558	17.56784776	0.013893833	2.645404149	85868.33883	4.157417057	19.66165524
70 to 74	0.029977976	2.60375141	68072.55213	3.175546138	14.18416673	0.021249655	2.643882804	80092.41919	3.813697606	15.88871458
75 to 79	0.045916029	2.581366415	58554.33702	2.635131841	11.06664729	0.034148402	2.635753133	71989.15451	3.330595649	12.37951204
80 to 84	0.072270353	2.530396211	46457.28282	1.97114221	8.276331371	0.058382195	2.593207079	60617.26836	2.657516418	9.207447211
85 to 89	0.129917931	2.434889537	32215.22211	1.208219563	5.816999384	0.110115805	2.505662935	45105.16637	1.769383048	6.482331443
90 to 94	0.225074869	2.194428042	16521.98893	0.506394909	4.030258148	0.195171446	2.282014799	25625.86599	0.837254129	4.505728183
95 plus	0.321309248	3.112306816	5126.411616	0.159569324	3.112306816	0.292572646	3.418046096	9288.476025	0.317534557	3.418046096

Table 15: Central Latin America 2100 life table, by age and sex. mx=mortality rate, ax=mean person-years lived in an age interval among those who die in that age interval, lx=number of persons left alive at age x, nLx=person-years lived between age x and x+n, ex=life expectancy at age x.

Age Group	Male					Female				
	mx	ax	lx	nLx	ex	mx	ax	lx	nLx	ex
Early Neonatal	0.057303193	0.009587285	100000	0.019167549	81.1590785	0.048568962	0.009587552	100000	0.019169154	85.0996787
Late Neonatal	0.008905484	0.028764667	99890.17559	0.05745634	81.22906607	0.008960981	0.028764651	99906.90512	0.057465872	85.15970268
Post Neonatal	0.001192566	0.461559118	99839.01355	0.921294056	81.21309722	0.001101972	0.461565553	99855.41654	0.921483946	85.14602143
1 to 4	0.00013929	1.99981428	99729.15869	3.988055493	80.37865322	0.000137317	1.999816911	99753.88537	3.989060002	84.30881248
5 to 9	8.97654E-05	2.499812989	99673.62258	4.982563207	76.42222817	8.77127E-05	2.499817265	99699.12109	4.983863391	80.35393818
10 to 14	0.000141167	3.151965928	99628.91028	4.980100661	71.45531154	0.000131079	2.736885216	99655.41878	4.981281657	75.38800158
15 to 19	0.000493709	2.808855654	99558.63685	4.972524379	66.50345705	0.000225345	2.618502558	99590.14567	4.976833633	70.43553966
20 to 24	0.000854555	2.632214503	99313.21636	4.955603651	61.66052077	0.000256397	2.579628033	99478.02125	4.970815667	65.511871
25 to 29	0.001007523	2.558116262	98890.02152	4.932359142	56.91276234	0.000322657	2.594156558	99350.60744	4.963675085	60.59242771
30 to 34	0.001127102	2.549594258	98393.53401	4.906109701	52.18651819	0.000399935	2.600689294	99190.50706	4.954765861	55.68575603
35 to 39	0.001257187	2.577985722	97841.18059	4.877219128	47.46623879	0.000510999	2.648399076	98992.42644	4.943682255	50.79162746
40 to 44	0.001611586	2.621408728	97228.72947	4.842864855	42.74815655	0.000766321	2.708069939	98739.91465	4.928336161	45.914254
45 to 49	0.002199514	2.685574403	96449.21396	4.798036685	38.07120882	0.001276648	2.723768517	98362.4446	4.903854258	41.07938915
50 to 54	0.003612854	2.684515481	95395.34788	4.730216607	33.46055453	0.002131609	2.700055576	97736.88655	4.862997157	36.32378479
55 to 59	0.005525381	2.654956893	93689.35976	4.624566004	29.01861889	0.003351182	2.667876274	96701.34615	4.797535403	31.68243906
60 to 64	0.007965498	2.654424618	91140.29048	4.473633862	24.75348127	0.004867933	2.651552232	95095.9452	4.701152809	27.17054467
65 to 69	0.012222382	2.646734368	87587.12838	4.257178878	20.64572698	0.007196603	2.677975072	92810.90746	4.564324511	22.7713999
70 to 74	0.01807099	2.652760623	82405.46941	3.95343813	16.77393643	0.011541772	2.715586078	89534.39034	4.362085242	18.50370748
75 to 79	0.029734095	2.646313183	75301.46077	3.520709877	13.10070739	0.021122097	2.727980608	84517.16078	4.033121407	14.43599534
80 to 84	0.049854693	2.578135499	64934.64086	2.901699563	9.766469846	0.040769513	2.653224906	76058.34079	3.473973025	10.73308383
85 to 89	0.09931465	2.528617728	50682.03185	2.04283972	6.782004413	0.083772851	2.603034916	62048.85475	2.590448803	7.546329469
90 to 94	0.19150936	2.239920748	30811.12469	1.014799303	4.560866269	0.163024489	2.331481665	40711.60686	1.426502038	5.154839555
95 plus	0.293461243	3.416049365	11746.69368	0.406641708	3.416049365	0.26230741	3.826314569	17872.43024	0.693251367	3.826314569

Table 15: Colombia 2017 life table, by age and sex. mx=mortality rate, ax=mean person-years lived in an age interval among those who die in that age interval, lx=number of persons left alive at age x, nLx=person-years lived between age x and x+n, ex=life expectancy at age x.

Age Group	Male					Female				
	mx	ax	lx	nLx	ex	mx	ax	lx	nLx	ex
Early Neonatal	0.328971737	0.009578958	100000	0.019117716	77.04023778	0.227502441	0.009582068	100000	0.019136307	82.20232424
Late Neonatal	0.039810822	0.028756142	99371.14556	0.057107028	77.50841248	0.02411463	0.028760471	99564.66825	0.057244066	82.54248333
Post Neonatal	0.004574994	0.461318836	99143.85131	0.91345292	77.62851009	0.004831106	0.461300642	99426.64275	0.915949948	82.59949972
1 to 4	0.000584307	1.999220925	98726.07411	3.944433347	77.03183343	0.000538421	1.999282105	98984.21572	3.955109038	82.04339951
5 to 9	0.000303366	2.499367987	98495.68516	4.921051892	73.20740147	0.000213336	2.49955555	98771.31404	4.935932978	78.21598228
10 to 14	0.000332815	3.093132937	98346.4285	4.914202644	68.31473555	0.000237265	2.663548529	98666.02384	4.930568446	73.29679318
15 to 19	0.001255478	2.822400766	98182.89689	4.89576146	63.42334478	0.000400855	2.621648894	98549.04421	4.922759696	68.38061197
20 to 24	0.00229821	2.611688338	97568.27576	4.851784081	58.80478145	0.000473361	2.567164998	98351.71649	4.911929745	63.51246654
25 to 29	0.002529068	2.505112799	96453.31504	4.792429643	54.45381307	0.000556161	2.583023962	98119.21123	4.899375162	58.65678077
30 to 34	0.002418069	2.47531801	95241.42444	4.733181696	50.1143355	0.000698598	2.600559759	97846.73845	4.884151086	53.81280353
35 to 39	0.00230228	2.49083282	94097.10898	4.67784049	45.6932465	0.000899405	2.616455767	97505.55171	4.864849291	48.99184816
40 to 44	0.002374403	2.542953951	93020.40516	4.62405269	41.19290648	0.001210471	2.640691258	97068.03949	4.839580968	44.2006562
45 to 49	0.002856037	2.609280805	91922.82399	4.564983684	36.65400568	0.001732857	2.662498233	96482.28751	4.804654392	39.45271189
50 to 54	0.003969163	2.649780802	90619.57883	4.489121032	32.14318913	0.002597192	2.673896679	95649.83456	4.753777127	34.77257855
55 to 59	0.005896986	2.675925606	88838.66349	4.38191014	27.7339182	0.003982191	2.683320282	94415.43938	4.677628977	30.19181312
60 to 64	0.009387683	2.6669364	86256.23062	4.220438342	23.4835229	0.006299742	2.671020812	92553.25288	4.560772762	25.74471801
65 to 69	0.014466513	2.655987964	82297.17719	3.980024801	19.48424723	0.009625009	2.678227781	89681.25594	4.386104723	21.48289412
70 to 74	0.022780441	2.63618774	76545.23393	3.631947338	15.74813446	0.015733588	2.680317147	85462.24931	4.122779564	17.41030618
75 to 79	0.035478385	2.609884917	68283.20933	3.147744412	12.33436008	0.026443603	2.666705473	78982.04811	3.71989587	13.61785738
80 to 84	0.056369752	2.561413871	57137.86279	2.512389436	9.232203751	0.046043953	2.61835078	69160.41975	3.116908579	10.17257878
85 to 89	0.108897238	2.497672106	43011.38574	1.691008678	6.426348922	0.092142741	2.569349554	54839.81521	2.241288877	7.146438959
90 to 94	0.202727622	2.229904402	24643.81285	0.789692206	4.361400015	0.173889164	2.31950483	34239.90247	1.168651508	4.905048087
95 plus	0.302925683	3.301724042	8667.315122	0.286562101	3.301724042	0.272694592	3.668427132	13966.75694	0.513275126	3.668427132

Table 15: Colombia 2100 life table, by age and sex. mx=mortality rate, ax=mean person-years lived in an age interval among those who die in that age interval, lx=number of persons left alive at age x, nLx=person-years lived between age x and x+n, ex=life expectancy at age x.

Age Group	Male					Female				
	mx	ax	lx	nLx	ex	mx	ax	lx	nLx	ex
Early Neonatal	0.062030874	0.00958714	100000	0.01916668	84.76517491	0.046631513	0.009587612	100000	0.01916951	88.36870873
Late Neonatal	0.010281774	0.028764287	99881.1185	0.057448857	84.84691729	0.00782384	0.028764965	99910.61869	0.057469887	88.42851765
Post Neonatal	0.001032561	0.461570484	99822.05663	0.921205609	84.8395487	0.001298728	0.461551576	99865.66126	0.921494818	88.41076422
1 to 4	0.000136448	1.999818069	99726.95122	3.987989936	83.99665829	0.000132289	1.999823615	99746.00431	3.988784965	87.5929083
5 to 9	8.41182E-05	2.499824754	99672.55266	4.982580141	80.04132102	7.77075E-05	2.499838109	99693.25027	4.983694655	83.63812261
10 to 14	0.000112555	3.020228197	99630.65785	4.980381893	75.07385315	0.000109172	2.69765957	99654.54011	4.981461879	78.6695691
15 to 19	0.000329293	2.831973252	99574.63817	4.975172875	70.11434823	0.000168208	2.602673376	99600.18642	4.977997886	73.71097531
20 to 24	0.000637066	2.657907721	99410.89565	4.963094331	65.22432784	0.000187459	2.594288309	99516.4926	4.973574307	68.77068627
25 to 29	0.000766876	2.574118911	99095.10572	4.9455303	60.42226896	0.000245336	2.634263615	99423.3017	4.968273073	63.83262672
30 to 34	0.000875117	2.51875348	98716.64399	4.925148906	55.64288761	0.000336724	2.598951492	99301.47229	4.961056897	58.907532
35 to 39	0.000838076	2.51156674	98286.77735	4.904157069	50.87425659	0.000399085	2.6076246	99134.4942	4.95199657	54.00218506
40 to 44	0.00094151	2.593472636	97877.35568	4.882830847	46.07563935	0.000542658	2.663245466	98936.95203	4.940573253	49.10446317
45 to 49	0.001247999	2.666649294	97419.80547	4.856828121	41.27858074	0.000815915	2.672275008	98668.9751	4.924097745	44.23020823
50 to 54	0.001872962	2.706916335	96816.63396	4.820056726	36.51682778	0.001222274	2.680944922	98267.39683	4.899470067	39.39949355
55 to 59	0.002985356	2.701032807	95918.26655	4.763064752	31.83042718	0.001874947	2.671381175	97668.96557	4.862207765	34.62358857
60 to 64	0.004520395	2.708742614	94504.51977	4.676897893	27.26192594	0.002777312	2.669515783	96758.10381	4.806841706	29.92344298
65 to 69	0.007614721	2.701337957	92404.69163	4.540902379	22.81286464	0.004242157	2.70672191	95424.77778	4.725209619	25.3022929
70 to 74	0.012011944	2.720496712	88980.55581	4.331242076	18.57878436	0.00707423	2.746371327	93424.47602	4.598049689	20.78393052
75 to 79	0.021475449	2.727533783	83858.54876	3.999806622	14.536531	0.013298551	2.782094262	90181.57863	4.379879053	16.42884546
80 to 84	0.038823228	2.626723981	75476.61004	3.463614364	10.84642398	0.027236748	2.715397048	84390.08194	3.974481089	12.3621314
85 to 89	0.082042367	2.586024596	62422.85789	2.62431124	7.525646989	0.061422406	2.696392446	73665.16595	3.232665627	8.744349829
90 to 94	0.16990622	2.245005676	41904.87175	1.44842454	4.991534653	0.132738901	2.35122646	54162.27539	2.014694839	5.922950189
95 plus	0.275022371	3.663192038	18411.24513	0.695318751	3.663192038	0.233137974	4.31362396	27980.08882	1.225563824	4.31362396

Table 15: Costa Rica 2017 life table, by age and sex. mx=mortality rate, ax=mean person-years lived in an age interval among those who die in that age interval, lx=number of persons left alive at age x, nLx=person-years lived between age x and x+n, ex=life expectancy at age x.

Age Group	Male					Female				
	mx	ax	lx	nLx	ex	mx	ax	lx	nLx	ex
Early Neonatal	0.239296389	0.009581707	100000	0.019134144	76.07408286	0.198954191	0.009582943	100000	0.019141542	82.19325586
Late Neonatal	0.02231449	0.028760968	99542.14029	0.057234075	76.40475911	0.017128455	0.028762398	99619.17749	0.057286912	82.48824566
Post Neonatal	0.00191385	0.461507879	99414.43387	0.917070787	76.44533924	0.001723994	0.461521366	99521.05758	0.918134775	82.5120128
1 to 4	0.000301911	1.999597452	99238.93432	3.96716163	75.6564339	0.00024689	1.999670814	99362.77879	3.972549349	81.71943314
5 to 9	0.00018835	2.499607605	99119.1715	4.953625844	71.74543845	0.000119555	2.499750927	99264.70489	4.961752139	77.79820077
10 to 14	0.000228244	3.026105442	99025.877	4.949062989	66.81068176	0.000166541	2.734014575	99205.3866	4.958398076	72.84322632
15 to 19	0.00076547	2.800665516	98912.92275	4.937334095	61.8835196	0.000306981	2.641097908	99122.81023	4.95255434	67.90162664
20 to 24	0.001340568	2.627968699	98534.99032	4.91113282	57.11008553	0.000375422	2.592884536	98970.77727	4.944071134	63.00185471
25 to 29	0.001604825	2.542540276	97876.62733	4.874607199	52.47647151	0.000475998	2.58734929	98785.16793	4.933592689	58.11533013
30 to 34	0.00169207	2.559736516	97094.35327	4.834755285	47.87868983	0.000577343	2.615751429	98550.3329	4.920743417	53.24761039
35 to 39	0.002123425	2.598144699	96276.30193	4.789389768	43.26366881	0.000800924	2.648564197	98266.24257	4.904076381	48.39394407
40 to 44	0.002741567	2.625554998	95259.35361	4.732164734	38.69767796	0.001155976	2.686686598	97873.47279	4.880622184	43.57745376
45 to 49	0.003863998	2.64503934	93962.08021	4.655742263	34.19555811	0.001853927	2.670474107	97309.30374	4.844543231	38.81443847
50 to 54	0.005605991	2.649159019	92163.26548	4.548229391	29.81116932	0.002711522	2.675202437	96411.20379	4.790364565	34.15100823
55 to 59	0.008244715	2.65350509	89613.85394	4.395665283	25.58368118	0.00422626	2.681933403	95112.37423	4.709484846	29.58067646
60 to 64	0.012496709	2.642706311	85990.36459	4.176509157	21.54968662	0.006622623	2.677708774	93122.21934	4.58559548	25.1553272
65 to 69	0.018609337	2.634492374	80772.23954	3.868371987	17.77090434	0.01041551	2.683274157	90085.78635	4.398184054	20.91268084
70 to 74	0.028642808	2.627130662	73575.60445	3.444749508	14.25125732	0.017191085	2.676146132	85505.86918	4.111109981	16.88874804
75 to 79	0.046110431	2.589213066	63713.05379	2.867120071	11.05061518	0.028722931	2.662101821	78440.95667	3.675366062	13.16846031
80 to 84	0.072504899	2.529854376	50500.55374	2.14173763	8.26484354	0.050431969	2.610492392	67890.15856	3.02968015	9.801103527
85 to 89	0.130206623	2.434078796	34983.65953	1.31139815	5.809895735	0.098697618	2.54529333	52622.91898	2.118394277	6.887806788
90 to 94	0.225365563	2.193877811	17921.22007	0.549061593	4.026448674	0.181825682	2.306631956	31734.04372	1.065422126	4.7482127
95 plus	0.321544	3.110129018	5554.606921	0.172824459	3.110129018	0.280155976	3.569879136	12378.629	0.442188144	3.569879136

Table 15: Costa Rica 2100 life table, by age and sex. mx=mortality rate, ax=mean person-years lived in an age interval among those who die in that age interval, lx=number of persons left alive at age x, nLx=person-years lived between age x and x+n, ex=life expectancy at age x.

Age Group	Male					Female				
	mx	ax	lx	nLx	ex	mx	ax	lx	nLx	ex
Early Neonatal	0.059040452	0.009587232	100000	0.01916723	81.7005434	0.052919061	0.009587419	100000	0.019168355	87.25500227
Late Neonatal	0.005335889	0.028765651	99886.85519	0.057460332	81.77379665	0.005112361	0.028765713	99898.57883	0.057467445	87.3242485
Post Neonatal	0.00045705	0.461611367	99856.20504	0.921765559	81.74130518	0.000405624	0.461615021	99869.20976	0.921907489	87.29229319
1 to 4	7.42406E-05	1.999901012	99814.08307	3.991970652	80.85233076	6.29183E-05	1.999916109	99831.82208	3.992770517	86.40145323
5 to 9	5.99787E-05	2.499875045	99784.45134	4.988474471	76.87575847	4.53983E-05	2.499905421	99806.705	4.989769215	82.42266371
10 to 14	9.04382E-05	3.171205415	99754.5302	4.986843955	71.89809154	8.22732E-05	2.88520307	99784.06587	4.988291082	77.44073572
15 to 19	0.000352462	2.834344651	99709.43118	4.981594226	66.92929233	0.000156436	2.650484865	99743.04035	4.985305696	72.47140337
20 to 24	0.000676735	2.658761262	99533.91374	4.968755505	62.04155327	0.000182433	2.617231669	99665.08834	4.981074882	67.52592857
25 to 29	0.000841895	2.557867762	99198.00289	4.949712271	57.24152845	0.000245421	2.582756925	99574.26013	4.975751237	62.58491178
30 to 34	0.000907553	2.560353354	98781.91251	4.928185857	52.47070199	0.000269673	2.615205939	99452.23457	4.96938355	57.65773027
35 to 39	0.001125005	2.575915306	98335.67176	4.903470571	47.69597682	0.000363074	2.644380639	99318.33258	4.961684171	52.73122127
40 to 44	0.001410966	2.661199663	97785.73717	4.873086143	42.94771832	0.000532332	2.73386521	99138.3549	4.950908343	47.82096738
45 to 49	0.002102519	2.673575998	97101.69712	4.831457262	38.22790716	0.000924703	2.718878794	98875.21078	4.933293006	42.9391252
50 to 54	0.003236677	2.674758973	96090.04451	4.768665951	33.59753685	0.001438467	2.705780293	98420.11867	4.904733788	38.12245702
55 to 59	0.004993598	2.68197148	94556.45276	4.673563449	29.091289	0.002243146	2.717451129	97717.23576	4.860710133	33.37363643
60 to 64	0.00748048	2.666812557	92245.61192	4.533525086	24.74342298	0.003460625	2.698345672	96632.74255	4.793510153	28.71389827
65 to 69	0.011737995	2.653665751	88890.23422	4.32636311	20.56469875	0.005581763	2.731507457	94982.23457	4.689666353	24.15928176
70 to 74	0.017953285	2.675265418	83885.30877	4.028904303	16.62192943	0.009695161	2.734849053	92383.44595	4.520443181	19.755393
75 to 79	0.031558544	2.652453328	76820.38773	3.581939752	12.89337621	0.017044021	2.754095018	88062.33636	4.242063239	15.57750633
80 to 84	0.05361731	2.559674623	65861.86482	2.924759439	9.601135154	0.033295096	2.657659649	80967.98007	3.762346296	11.69440347
85 to 89	0.103809138	2.516235922	50770.33826	2.039068706	6.684883163	0.071107528	2.65728868	68763.11918	2.962992138	8.261924189
90 to 94	0.195853591	2.229727696	30667.95689	1.01088971	4.510773792	0.145495362	2.33997709	48566.11033	1.773341661	5.617145886
95 plus	0.29693426	3.387463254	11762.63295	0.410919853	3.387463254	0.245346705	4.119992576	23923.1612	1.018420336	4.119992576

Table 15: El Salvador 2017 life table, by age and sex. mx=mortality rate, ax=mean person-years lived in an age interval among those who die in that age interval, lx=number of persons left alive at age x, nLx=person-years lived between age x and x+n, ex=life expectancy at age x.

Age Group	Male					Female				
	mx	ax	lx	nLx	ex	mx	ax	lx	nLx	ex
Early Neonatal	0.231192263	0.009581955	100000	0.019135631	69.29417516	0.197416942	0.00958299	100000	0.019141825	78.14776733
Late Neonatal	0.033482648	0.028757887	99557.6366	0.05722461	69.58275308	0.018272698	0.028762083	99622.13289	0.05728673	78.42495872
Post Neonatal	0.005521712	0.461251583	99366.07284	0.915100517	69.65930005	0.005716424	0.461237751	99517.47009	0.916412303	78.44988496
1 to 4	0.000544052	1.999274597	98860.89876	3.950137596	69.08959124	0.000337334	1.999550222	98993.68457	3.957077621	77.93925723
5 to 9	0.000269963	2.499437577	98646.06078	4.928976344	65.23573448	0.000222173	2.49953714	98860.22703	4.940267147	74.04179676
10 to 14	0.000455995	3.384470429	98513.02311	4.92202763	60.32046648	0.00030448	2.712212822	98750.47926	4.934088859	69.1213159
15 to 19	0.002222577	2.746819729	98288.60849	4.889946421	55.45032814	0.000535387	2.61384857	98600.25409	4.923724754	64.22239496
20 to 24	0.003136505	2.617519462	97201.88749	4.824050252	51.03809868	0.000601326	2.5628945	98336.65424	4.909639535	59.38724347
25 to 29	0.00409274	2.608812101	95689.14229	4.738101422	46.80166169	0.000722174	2.617452938	98041.44689	4.893653954	54.55798106
30 to 34	0.005458567	2.571782192	93750.65786	4.626252278	42.71332828	0.001015512	2.639769971	97688.08355	4.872729809	49.74541122
35 to 39	0.006261093	2.523954625	91226.83173	4.491776572	38.82137262	0.001417905	2.637847983	97193.34366	4.843449248	44.9845713
40 to 44	0.006557142	2.543556371	88417.1288	4.350870075	34.97220034	0.00197592	2.672378679	96506.7984	4.803252231	40.2849012
45 to 49	0.008129831	2.574712336	85568.0743	4.195817182	31.04967143	0.003097524	2.67909453	95558.1241	4.743815693	35.65720202
50 to 54	0.010164342	2.583488949	82163.17256	4.009878368	27.22757384	0.004748659	2.671368709	94089.57797	4.653060265	31.17045965
55 to 59	0.013321709	2.590052588	78096.83314	3.783683807	23.50905938	0.007271954	2.653007316	91881.79563	4.517074082	26.8533732
60 to 64	0.017838413	2.579949386	73070.38295	3.502778915	19.94703953	0.010690694	2.640739709	88600.80558	4.321216842	22.7472023
65 to 69	0.02350147	2.601392215	66842.40935	3.164438519	16.56511012	0.015826061	2.63563893	83988.67849	4.048305204	18.8487321
70 to 74	0.036106116	2.584996394	59435.58749	2.734541068	13.30648506	0.023953332	2.641791816	77597.41325	3.673111988	15.1814718
75 to 79	0.052124095	2.557517385	49613.08688	2.202136542	10.43325204	0.039395986	2.619598083	68831.889	3.147912299	11.77660649
80 to 84	0.082136728	2.527610324	38210.64505	1.590097885	7.792814011	0.065211548	2.566678792	56498.49642	2.440435357	8.7772555
85 to 89	0.142231255	2.400340457	25247.38756	0.923486951	5.513235736	0.119354256	2.476099331	40699.69371	1.56690314	6.19751059
90 to 94	0.237486533	2.17113468	12203.2672	0.365843153	3.867150662	0.205510608	2.260632359	22145.44328	0.710448837	4.33790403
95 plus	0.331333414	3.019044816	3560.610233	0.107873421	3.019044816	0.302043685	3.313458209	7648.136362	0.254759607	3.313458209

Table 15: El Salvador 2100 life table, by age and sex. mx=mortality rate, ax=mean person-years lived in an age interval among those who die in that age interval, lx=number of persons left alive at age x, nLx=person-years lived between age x and x+n, ex=life expectancy at age x.										
Age Group	Male					Female				
	mx	ax	lx	nLx	ex	mx	ax	lx	nLx	ex
Early Neonatal	0.035303071	0.009587959	100000	0.019171592	77.40356502	0.036009312	0.009587937	100000	0.019171462	83.82017003
Late Neonatal	0.006796159	0.028765249	99932.32401	0.057484071	77.43660893	0.004809583	0.028765797	99930.97026	0.057486578	83.85860373
Post Neonatal	0.001086488	0.461566653	99893.26076	0.921839782	77.40925543	0.001245357	0.461555368	99903.32555	0.921865071	83.82413902
1 to 4	0.000270076	1.999639902	99793.12194	3.989570108	76.56295146	0.000222289	1.999703608	99788.54042	3.989768103	82.99645299
5 to 9	7.2309E-05	2.499849356	99685.41045	4.983370245	72.64329741	7.4608E-05	2.499844567	99699.88431	4.984065372	79.06834752
10 to 14	0.00013827	3.398478424	99649.40394	4.981294599	67.66837373	0.000119511	2.749854341	99662.73531	4.981780217	74.09673539
15 to 19	0.000722604	2.730539276	99580.58358	4.970792532	62.71245864	0.000200695	2.590879341	99603.25307	4.977750292	69.13918304
20 to 24	0.000993486	2.662787136	99221.84674	4.949462647	57.92624036	0.000199473	2.590069204	99503.46026	4.972773516	64.20555822
25 to 29	0.001404272	2.66246721	98731.82937	4.920364305	53.19717144	0.000275758	2.6855186	99404.39767	4.967037855	59.26659056
30 to 34	0.002134108	2.597174101	98043.74177	4.877205854	48.5484766	0.000430517	2.648969082	99267.61214	4.958331572	54.3437142
35 to 39	0.002475707	2.553388676	97009.31259	4.821447873	44.03386512	0.000548243	2.660856493	99054.49671	4.946388076	49.45333421
40 to 44	0.002824246	2.616672387	95825.26361	4.759416234	39.53921512	0.000852561	2.770880349	98783.86276	4.929738956	44.57887548
45 to 49	0.004175906	2.638767568	94493.8048	4.678942539	35.05055599	0.00156008	2.759226187	98364.78457	4.901073883	39.75327299
50 to 54	0.005980392	2.651142462	92562.624	4.564506509	30.71552662	0.002735457	2.731401563	97603.21952	4.849977729	35.03579715
55 to 59	0.008863981	2.630450448	89875.72388	4.401971158	26.54572342	0.004437578	2.6790385	96284.72672	4.764999457	30.46944422
60 to 64	0.0117368	2.581704981	86043.44789	4.185061663	22.609269	0.006161572	2.634355265	94189.20677	4.642540969	26.07806021
65 to 69	0.01557668	2.63895125	81201.32523	3.917793859	18.79638627	0.008972846	2.690503847	91358.17678	4.475396556	21.78969584
70 to 74	0.025739437	2.620788616	75212.29071	3.547164392	15.08571764	0.013929607	2.751177687	87411.59452	4.238630515	17.64136741
75 to 79	0.039306324	2.61075038	66280.90459	3.037219315	11.76578109	0.027042982	2.743767016	81627.47339	3.850966342	13.67912009
80 to 84	0.06803826	2.561511589	54762.32528	2.361346464	8.710550565	0.050962804	2.634191774	71607.05867	3.211814797	10.20313863
85 to 89	0.123051447	2.458990218	39352.57199	1.51945405	6.103426486	0.097055248	2.563724885	56173.47812	2.308902385	7.20777528
90 to 94	0.216661506	2.201530095	21702.23351	0.689621383	4.189687557	0.177191218	2.296679569	35744.44325	1.247283961	4.963801566
95 plus	0.314088719	3.20354931	7510.483927	0.249063846	3.20354931	0.275065774	3.708363142	15669.87059	0.622426364	3.708363142

Table 15: Guatemala 2017 life table, by age and sex. mx=mortality rate, ax=mean person-years lived in an age interval among those who die in that age interval, lx=number of persons left alive at age x, nLx=person-years lived between age x and x+n, ex=life expectancy at age x.

Age Group	Male					Female				
	mx	ax	lx	nLx	ex	mx	ax	lx	nLx	ex
Early Neonatal	0.37785371	0.00957746	100000	0.019108768	69.27983463	0.265095918	0.009580916	100000	0.019129416	76.23136273
Late Neonatal	0.085734486	0.028743474	99278.05079	0.056978275	69.76402853	0.067390315	0.028748534	99492.91801	0.057131689	76.60045866
Post Neonatal	0.013096784	0.460713464	98789.69945	0.906623272	70.05110621	0.010960758	0.460865203	99107.96983	0.910438415	76.8402464
1 to 4	0.001788098	1.997615872	97602.96644	3.89020011	69.97411027	0.001631841	1.997824214	98110.38011	3.911640695	76.69358906
5 to 9	0.000518636	2.498919509	96907.88865	4.83912009	66.46200461	0.000366699	2.499236044	97472.36272	4.869153973	73.18268285
10 to 14	0.000533587	2.895013417	96657.02402	4.827429757	61.62808744	0.000403739	2.70853148	97293.85092	4.860197384	68.3123961
15 to 19	0.001538067	2.828856336	96399.50356	4.803935284	56.78495771	0.000774444	2.609272209	97097.6483	4.845911784	63.44491262
20 to 24	0.002998724	2.6580343	95660.68642	4.74967923	52.20127196	0.000815334	2.549732891	96722.38272	4.826478326	58.68076939
25 to 29	0.003896425	2.577795942	94236.50928	4.667776634	47.94924068	0.000977218	2.597083559	96328.89584	4.805162912	53.90985713
30 to 34	0.004602657	2.557138604	92418.01016	4.569535311	43.84117271	0.001281797	2.625344609	95859.37655	4.778427257	49.16094348
35 to 39	0.005377657	2.544476586	90315.28198	4.45693336	39.8014651	0.001768513	2.624335544	95246.96033	4.742426364	44.45984621
40 to 44	0.006040265	2.553577484	87919.33925	4.331986346	35.8157871	0.002370934	2.657874202	94408.40021	4.694355331	39.83097806
45 to 49	0.007328201	2.564884307	85304.053	4.190474459	31.83460546	0.00363393	2.67552186	93295.65581	4.625717998	35.27381711
50 to 54	0.008878514	2.557494875	82235.3714	4.024567644	27.92593494	0.005591093	2.656778599	91615.20882	4.521543707	30.87107331
55 to 59	0.010573603	2.597921432	78665.43768	3.835943978	24.07645764	0.008197524	2.65013635	89088.20112	4.370267727	26.67052289
60 to 64	0.015210984	2.622532283	74613.99588	3.600646812	20.24221713	0.012297685	2.627308768	85507.69392	4.154258725	22.67535007
65 to 69	0.022154859	2.61684257	69144.15568	3.284082829	16.6357978	0.017373917	2.597810741	80402.9178	3.859256299	18.9471498
70 to 74	0.033358805	2.605229837	61880.06305	2.865552022	13.28165737	0.023692417	2.587944434	73705.56318	3.486364666	15.43188689
75 to 79	0.052066592	2.585879252	52341.08988	2.325530902	10.22860592	0.033605309	2.649174624	65459.53526	3.033832514	12.04962335
80 to 84	0.087936606	2.532327231	40265.86153	1.655253415	7.523960631	0.064996965	2.608520694	55288.32215	2.393445701	8.779981474
85 to 89	0.149435464	2.380520828	25755.47229	0.926339973	5.343415617	0.119381389	2.475625839	39776.97638	1.529466313	6.190485711
90 to 94	0.244650368	2.157274685	11953.66023	0.352893154	3.776129009	0.20560616	2.260720658	21575.75662	0.69089268	4.333449539
95 plus	0.337088099	2.966991359	3339.803642	0.09924301	2.966991359	0.302155194	3.310607947	7410.802923	0.245862849	3.310607947

Table 15: Guatemala 2100 life table, by age and sex. mx=mortality rate, ax=mean person-years lived in an age interval among those who die in that age interval, lx=number of persons left alive at age x, nLx=person-years lived between age x and x+n, ex=life expectancy at age x.

Age Group	Male					Female				
	mx	ax	lx	nLx	ex	mx	ax	lx	nLx	ex
Early Neonatal	0.06696748	0.009586989	100000	0.019165773	80.36350752	0.05063235	0.009587489	100000	0.019168775	86.03048158
Late Neonatal	0.019512938	0.028761741	99871.66915	0.057428177	80.4472256	0.019945729	0.028761621	99902.95809	0.057445452	86.09455766
Post Neonatal	0.00257249	0.46146109	99759.63999	0.919975776	80.47948714	0.00233978	0.461477622	99788.4045	0.920339816	86.13543638
1 to 4	0.000319795	1.999573607	99523.05941	3.978382735	79.74506056	0.000299102	1.999601198	99573.13162	3.980548792	85.39611623
5 to 9	0.000169046	2.499647821	99396.16789	4.967726233	75.84008359	0.000132023	2.499724953	99454.40028	4.971094551	81.49218809
10 to 14	0.000231082	3.110684252	99312.97053	4.963249651	70.89733508	0.000187691	2.832010894	99389.45189	4.967357362	76.54099864
15 to 19	0.000594129	2.912796457	99200.13536	4.953669072	65.96977496	0.000313918	2.618309468	99297.40895	4.961139588	71.60674724
20 to 24	0.001168234	2.720376092	98909.27553	4.932242046	61.14985872	0.000313047	2.561394083	99143.06748	4.953387451	66.71208483
25 to 29	0.001643687	2.602697985	98338.69559	4.897769933	56.48304522	0.00038466	2.59876533	98989.61493	4.9449569	61.80993791
30 to 34	0.001949338	2.563937276	97541.77723	4.85421262	51.91682344	0.000491601	2.632639491	98801.4688	4.934317557	56.92044397
35 to 39	0.002193375	2.56301406	96606.61508	4.804973472	47.38835727	0.000633021	2.656458893	98561.60083	4.920789719	52.0497257
40 to 44	0.002655351	2.596982405	95566.97539	4.748270931	42.86922465	0.000909816	2.764225729	98253.36978	4.902599117	47.20086265
45 to 49	0.003271268	2.61644802	94325.16881	4.680210739	38.38980156	0.001602381	2.745841181	97811.79298	4.872909533	42.39681085
50 to 54	0.00452282	2.622933818	92817.58394	4.592118883	33.95995616	0.002609571	2.721542902	97037.82646	4.823128782	37.70494881
55 to 59	0.005781922	2.609976879	90782.17349	4.478235487	29.64621001	0.004105103	2.661126218	95792.31078	4.744183993	33.14848978
60 to 64	0.007798626	2.637026941	88245.97896	4.334797368	25.40839817	0.00550277	2.601597923	93871.59811	4.633742522	28.75877952
65 to 69	0.011566101	2.656380586	84976.75691	4.140143926	21.25468861	0.007248786	2.630681091	91374.55009	4.492653511	24.4505919
70 to 74	0.017490874	2.685273398	80380.71493	3.870597944	17.29990432	0.00963899	2.660210726	88205.41387	4.315724895	20.22259185
75 to 79	0.027765525	2.693215606	74083.45671	3.494609736	13.51902093	0.015389731	2.806713375	84190.47282	4.075466737	16.03568048
80 to 84	0.05039837	2.659024511	65105.71405	2.937256652	10.02688325	0.033244841	2.740206907	78221.03373	3.651398228	12.03203815
85 to 89	0.098294029	2.536151505	51593.52433	2.118655051	6.974620625	0.06976508	2.669152039	66906.98411	2.913325299	8.542548232
90 to 94	0.188276641	2.226906514	32879.32518	1.114276006	4.681511679	0.14170216	2.323726249	48535.07184	1.810687063	5.82312155
95 plus	0.29033408	3.485467507	13708.32157	0.505539309	3.485467507	0.24128242	4.25343751	25464.66858	1.164115939	4.25343751

Table 15: Honduras 2017 life table, by age and sex. mx=mortality rate, ax=mean person-years lived in an age interval among those who die in that age interval, lx=number of persons left alive at age x, nLx=person-years lived between age x and x+n, ex=life expectancy at age x.										
**Age Group**	**Male**					**Female**				
	**mx**	**ax**	**lx**	**nLx**	**ex**	**mx**	**ax**	**lx**	**nLx**	**ex**
Early Neonatal	0.294533294	0.009580014	100000	0.019124021	73.0243015	0.249093994	0.009581406	100000	0.019132348	74.99579788
Late Neonatal	0.033011743	0.028758017	99436.75612	0.057155898	73.41865773	0.029967351	0.028758857	99523.43592	0.057210728	75.33566949
Post Neonatal	0.004843161	0.461299786	99248.09184	0.91429992	73.50063188	0.003289839	0.461410131	99351.99993	0.915913134	75.40808168
1 to 4	0.00070415	1.999061134	98805.34089	3.946653922	72.90466046	0.000666487	1.99911135	99050.70245	3.956752017	74.71278288
5 to 9	0.000489778	2.498979629	98527.48736	4.920348079	69.10465174	0.000370037	2.49922909	98787.01661	4.934784617	70.90689303
10 to 14	0.000414615	2.767610599	98286.53491	4.909784732	64.2679588	0.000339103	2.66021534	98604.42466	4.926313896	66.03356299
15 to 19	0.001028708	2.847677858	98082.98859	4.893318332	59.39546404	0.000634078	2.68878017	98437.37947	4.914669198	61.14099919
20 to 24	0.002152498	2.643281945	97579.64704	4.854358693	54.68637413	0.000920288	2.628256613	98125.76699	4.895610223	56.32625885
25 to 29	0.002545317	2.564405708	96534.92921	4.797014735	50.24808119	0.001216188	2.62521899	97675.2642	4.869709234	51.57327428
30 to 34	0.003004448	2.550776633	95314.32992	4.73092386	45.85716328	0.001676573	2.640010112	97083.11892	4.835031808	46.87067066
35 to 39	0.003370893	2.544419294	93893.6487	4.656179327	41.51077666	0.002375989	2.624392438	96272.78123	4.78663008	42.24142537
40 to 44	0.003850457	2.566255715	92325.33998	4.573467596	37.17084704	0.003148842	2.61407161	95136.16979	4.721359296	37.71279975
45 to 49	0.004768343	2.61461829	90566.44615	4.47748012	32.84091511	0.004200721	2.638774455	93650.806	4.636591827	33.2673464
50 to 54	0.00674825	2.643818501	88435.12489	4.35268479	28.56721234	0.006148067	2.641583069	91705.44183	4.519823478	28.91465261
55 to 59	0.009944874	2.65483721	85504.6258	4.178021794	24.45342921	0.008795525	2.64432916	88930.97508	4.35644558	24.73151812
60 to 64	0.0152958	2.637579481	81361.61362	3.926614752	20.56154575	0.013136356	2.640834255	85107.34455	4.127750842	20.72070465
65 to 69	0.02265884	2.624355061	75375.52636	3.576953082	16.98402395	0.019681849	2.648039754	79698.83503	3.809222471	16.94439072
70 to 74	0.034890825	2.582051573	67303.11291	3.104460745	13.70704834	0.032038223	2.629298472	72228.95554	3.357696498	13.41938196
75 to 79	0.048981656	2.553013064	56523.2174	2.52521852	10.83256006	0.051925735	2.593043388	61527.14704	2.737093401	10.29336032
80 to 84	0.0749564	2.510757144	44225.61015	1.865359966	8.143011489	0.087963221	2.525703522	47436.1796	1.952449334	7.583348036
85 to 89	0.133138212	2.425822269	30326.75568	1.131008062	5.737080382	0.148723681	2.390527084	30489.45839	1.103328865	5.416290985
90 to 94	0.2283264	2.188331982	15350.46175	0.468277121	3.987358393	0.237091383	2.190946088	14332.8621	0.43302476	3.881786346
95 plus	0.323937444	3.087779944	4702.289728	0.145593432	3.087779944	0.330448136	3.030464238	4215.679229	0.129254242	3.030464238

Table 15: Honduras 2100 life table, by age and sex. mx=mortality rate, ax=mean person-years lived in an age interval among those who die in that age interval, lx=number of persons left alive at age x, nLx=person-years lived between age x and x+n, ex=life expectancy at age x.

Age Group	Male					Female				
	mx	ax	lx	nLx	ex	mx	ax	lx	nLx	ex
Early Neonatal	0.049070084	0.009587537	100000	0.019169062	81.52445646	0.052116644	0.009587444	100000	0.019168502	82.67237306
Late Neonatal	0.00659176	0.028765305	99905.95062	0.057469239	81.58175067	0.007582443	0.028765032	99900.1152	0.057464245	82.73536528
Post Neonatal	0.000740105	0.46159126	99868.07438	0.921754735	81.55496671	0.000599208	0.461601269	99856.55142	0.921708318	82.71373854
1 to 4	0.000166497	1.999778005	99799.876	3.990669318	80.68630386	0.000173619	1.999768509	99801.34098	3.990671351	81.83533263
5 to 9	0.000153235	2.49968076	99733.64008	4.984784588	76.7356589	0.000141285	2.499705656	99732.28418	4.984864688	77.88852023
10 to 14	0.000196965	2.955005583	99657.80986	4.980721645	71.7892409	0.000175189	2.76414671	99662.35794	4.981082182	72.93971113
15 to 19	0.000401041	2.845840747	99560.94977	4.973647848	66.85320872	0.000262357	2.676395887	99575.95752	4.975719043	67.9992725
20 to 24	0.000713454	2.652118953	99363.84539	4.959879897	61.97705322	0.000331635	2.623779983	99446.36935	4.968408197	63.08348878
25 to 29	0.000840565	2.596293181	99013.56036	4.940741557	57.1842632	0.000438792	2.658039086	99282.75064	4.959014909	58.18234174
30 to 34	0.001044408	2.582310346	98602.97445	4.917812164	52.40964192	0.00060559	2.67208234	99066.66764	4.946319826	53.30221082
35 to 39	0.001206082	2.578972816	98095.15998	4.890596414	47.66651881	0.000862482	2.666380314	98768.91178	4.928487258	48.45340656
40 to 44	0.001465031	2.620385062	97512.3487	4.858826377	42.93554546	0.001209312	2.700994986	98346.26531	4.903615817	43.64808853
45 to 49	0.002036624	2.681777648	96808.35989	4.817810548	38.22858751	0.001904686	2.707300948	97756.75076	4.86657377	38.89248102
50 to 54	0.00318863	2.705665251	95837.39048	4.757242748	33.58795606	0.003026808	2.673579046	96834.70526	4.80794551	34.23307791
55 to 59	0.005183626	2.692550457	94333.34288	4.661160833	29.07949245	0.004420995	2.656476784	95387.25332	4.720559566	29.70662298
60 to 64	0.008141368	2.668999558	91934.65465	4.511581766	24.76585683	0.00633685	2.65849103	93314.86614	4.597933579	25.30068372
65 to 69	0.012383206	2.659970868	88289.37764	4.290883536	20.67508625	0.009624591	2.692130613	90424.25604	4.423598114	21.01583802
70 to 74	0.019211019	2.638452461	83019.82921	3.972298063	16.81585838	0.01602848	2.706331153	86213.21703	4.15934834	16.89980505
75 to 79	0.029302197	2.631911651	75460.93687	3.530976078	13.23512296	0.028400775	2.708729823	79645.18896	3.742909698	13.05785381
80 to 84	0.047906651	2.577625014	65257.5114	2.92834902	9.896598706	0.055586027	2.631881228	69247.81837	3.071737434	9.599248841
85 to 89	0.096593825	2.536980308	51451.48089	2.085418395	6.86732755	0.105170259	2.528387659	52799.22294	2.117071397	6.762282489
90 to 94	0.188532796	2.244133977	31669.84547	1.047779129	4.607562881	0.18829154	2.288208143	31650.51419	1.066994751	4.680942621
95 plus	0.290991138	3.442773846	12216.8746	0.424883497	3.442773846	0.285839122	3.529050563	12570.78683	0.46117656	3.529050563

Table 15: Mexico 2017 life table, by age and sex. mx=mortality rate, ax=mean person-years lived in an age interval among those who die in that age interval, lx=number of persons left alive at age x, nLx=person-years lived between age x and x+n, ex=life expectancy at age x.

Age Group	Male					Female				
	mx	ax	lx	nLx	ex	mx	ax	lx	nLx	ex
Early Neonatal	0.259485541	0.009581088	100000	0.019130442	72.61538553	0.189881375	0.009583221	100000	0.019143205	78.59133763
Late Neonatal	0.027834949	0.028759445	99503.59378	0.057202827	72.95842362	0.021542681	0.028761181	99636.50667	0.057289603	78.85884045
Post Neonatal	0.006143518	0.461207411	99344.37056	0.914637865	73.01777649	0.004913106	0.461294817	99513.08966	0.916711324	78.89907184
1 to 4	0.000610467	1.999186044	98782.46433	3.946478257	72.50721387	0.000610185	1.99918642	99062.70107	3.957676269	78.33240211
5 to 9	0.000299534	2.499375972	98541.54665	4.923389636	68.67959527	0.00024088	2.499498167	98821.21068	4.938086232	74.51893923
10 to 14	0.000388076	3.061693449	98394.0756	4.916005801	63.77878549	0.000273468	2.670353342	98702.26249	4.931971037	69.60573178
15 to 19	0.001351774	2.798741123	98203.29789	4.89559754	58.89673943	0.000465622	2.646851936	98567.38929	4.922975479	64.69732144
20 to 24	0.002350068	2.605267177	97541.52427	4.849782703	54.27733449	0.000603809	2.581613537	98338.1648	4.909738839	59.84195848
25 to 29	0.002584078	2.533476179	96401.79342	4.789562525	49.88823186	0.00070573	2.568930557	98041.71062	4.893689539	55.01509767
30 to 34	0.002832775	2.55193137	95164.13452	4.725436727	45.50409811	0.000842511	2.612944503	97696.34841	4.875013208	50.20049526
35 to 39	0.003376906	2.579006445	93825.52705	4.653234138	41.11688831	0.001170981	2.654921535	97285.62366	4.850960272	45.40139932
40 to 44	0.004236768	2.589560357	92254.17804	4.566078195	36.77328714	0.001729771	2.668544268	96717.5864	4.816455136	40.65245082
45 to 49	0.005389282	2.623704955	90319.64555	4.458879909	32.5054469	0.002606239	2.700926738	95884.45191	4.765667097	35.98248383
50 to 54	0.007787741	2.63402234	87916.64589	4.316302446	28.32218144	0.004334967	2.689507808	94642.40929	4.68519418	31.41924612
55 to 59	0.011083272	2.622241909	84555.25439	4.119209093	24.3433665	0.006777299	2.67172486	92611.40286	4.558637941	27.04928771
60 to 64	0.01566637	2.616353232	79989.88399	3.855519472	20.58306946	0.010478321	2.653316975	89521.89978	4.368673463	22.89056767
65 to 69	0.022638611	2.597538502	73949.78834	3.506766231	17.05054861	0.015773658	2.633290908	84944.31337	4.094368549	18.98111717
70 to 74	0.031825735	2.593666404	66011.1394	3.065776632	13.78869566	0.023424673	2.63318798	78486.10599	3.718168422	15.32627146
75 to 79	0.049029133	2.575870404	56254.38558	2.513941817	10.73037216	0.037543773	2.625865146	69776.64842	3.203318288	11.91059555
80 to 84	0.077266873	2.523778671	43929.25327	1.843721217	8.01830757	0.06385709	2.585062506	57750.65136	2.501754536	8.84405645
85 to 89	0.136217899	2.416905055	29684.09373	1.09790975	5.655185881	0.11772464	2.480676739	41776.0131	1.611022505	6.237482661
90 to 94	0.231504945	2.182721004	14729.30256	0.445751597	3.943209794	0.203795154	2.264663366	22811.41234	0.73234821	4.360920092
95 plus	0.326527318	3.062538308	4410.309396	0.13507071	3.062538308	0.300511778	3.327677069	7887.299461	0.262474294	3.327677069

Table 15: Mexico 2100 life table, by age and sex. mx=mortality rate, ax=mean person-years lived in an age interval among those who die in that age interval, lx=number of persons left alive at age x, nLx=person-years lived between age x and x+n, ex=life expectancy at age x.

Age Group	Male					Female				
	mx	ax	lx	nLx	ex	mx	ax	lx	nLx	ex
Early Neonatal	0.048226425	0.009587563	100000	0.019169216	80.45093887	0.038213956	0.00958787	100000	0.019171057	84.15940866
Late Neonatal	0.006206665	0.028765411	99907.55829	0.0574708	80.5060347	0.006039315	0.028765457	99926.74236	0.057482112	84.20176571
Post Neonatal	0.001155589	0.461561745	99871.8901	0.921613164	80.47717361	0.001006699	0.461572321	99892.02865	0.921862341	84.17342066
1 to 4	0.000102912	1.999862784	99765.40452	3.989795025	79.63902537	0.000108314	1.999855581	99799.23659	3.991104916	83.32769822
5 to 9	7.27508E-05	2.499848436	99724.34971	4.985310779	75.67092794	7.74466E-05	2.499838653	99756.01253	4.986835171	79.36286486
10 to 14	0.000126923	3.067404148	99688.08374	4.983169891	70.69751594	0.000123741	2.731668714	99717.39707	4.984468342	74.39254441
15 to 19	0.000410783	2.81018299	99624.84054	4.97674695	65.74041073	0.000212723	2.627627924	99655.72788	4.980273026	69.43676627
20 to 24	0.00072576	2.63910563	99420.4614	4.962497537	60.86926573	0.000253964	2.578798117	99549.80755	4.974429773	64.50761315
25 to 29	0.000872256	2.577808921	99060.57415	4.942572903	56.08009496	0.000306884	2.577254497	99423.51083	4.967481493	59.58596446
30 to 34	0.001033875	2.585551477	98630.00367	4.919178158	51.31279073	0.000366839	2.603739574	99271.11995	4.959190761	54.67309319
35 to 39	0.001247949	2.618152374	98122.24322	4.89154186	46.56414897	0.000483563	2.677209145	99089.27599	4.948902113	49.76820891
40 to 44	0.001708697	2.637536586	97512.7475	4.856004308	41.83788332	0.000776053	2.723007408	98850.07122	4.933786278	44.88164397
45 to 49	0.002353478	2.701829703	96684.10518	4.808192908	37.17266562	0.001319581	2.744444419	98467.37935	4.908730718	40.04475138
50 to 54	0.004033254	2.695315663	95553.97042	4.73372353	32.57901895	0.00231006	2.710729481	97820.16797	4.865276686	35.29022447
55 to 59	0.006303575	2.655162686	93647.59188	4.614227341	28.1852753	0.003698233	2.673509527	96697.29915	4.79360856	30.66682278
60 to 64	0.00909861	2.651940142	90744.86072	4.44252937	23.99938967	0.00548074	2.656185946	94926.88409	4.686247772	26.18648983
65 to 69	0.013964764	2.634094412	86712.24009	4.197250362	19.98808656	0.008188208	2.677640066	92362.10553	4.532059528	21.83645213
70 to 74	0.020085724	2.641801241	80871.10299	3.86145132	16.23737914	0.01324955	2.720171934	88660.6353	4.303563521	17.63168142
75 to 79	0.033047629	2.631649949	73149.22111	3.394147841	12.66683628	0.024993504	2.719251209	82980.17339	3.926640221	13.64552997
80 to 84	0.054287854	2.564803636	62041.7923	2.745632604	9.457189921	0.048139022	2.636128034	73257.91762	3.293469793	10.08635127
85 to 89	0.105473092	2.509579783	47396.02524	1.886338373	6.580015742	0.094861977	2.561910466	57647.24184	2.350795433	7.092097539
90 to 94	0.198369191	2.231537281	27986.9122	0.910913398	4.449022471	0.176622407	2.31133148	35862.7367	1.225836997	4.876598752
95 plus	0.299173179	3.351995238	10322.39284	0.351461229	3.351995238	0.275117533	3.651198991	14735.42174	0.548192052	3.651198991

Table 15: Nicaragua 2017 life table, by age and sex. mx=mortality rate, ax=mean person-years lived in an age interval among those who die in that age interval, lx=number of persons left alive at age x, nLx=person-years lived between age x and x+n, ex=life expectancy at age x.

Age Group	Male					Female				
	mx	ax	lx	nLx	ex	mx	ax	lx	nLx	ex
Early Neonatal	0.360343245	0.009577997	100000	0.01911197	76.6819303	0.255329552	0.009581215	100000	0.019131205	80.65582356
Late Neonatal	0.049345241	0.028753511	99311.35584	0.05705702	77.19437851	0.041109487	0.028755783	99511.54909	0.057185568	81.03248777
Post Neonatal	0.005568084	0.461248288	99029.84808	0.911984413	77.35622285	0.004108678	0.461351962	99276.47253	0.914871202	81.16675058
1 to 4	0.000516008	1.99931199	98522.1171	3.936821248	76.82926869	0.000442219	1.999410375	98900.61677	3.952528317	80.55018286
5 to 9	0.000203221	2.499576624	98319.01601	4.913454375	72.98388764	0.000218847	2.499544069	98725.85115	4.933592997	76.68925223
10 to 14	0.00022178	2.902189246	98219.17599	4.908675165	68.05554407	0.000217703	2.685917889	98617.8885	4.928411973	71.77047335
15 to 19	0.000632808	2.800474012	98110.31829	4.898698177	63.12781297	0.00041427	2.639230942	98510.60019	4.92071815	66.84568704
20 to 24	0.00114051	2.63537194	97800.33073	4.876864101	58.31885269	0.000496493	2.559347512	98306.75492	4.909388743	61.97872244
25 to 29	0.001384922	2.645366934	97244.13634	4.846403283	53.63703385	0.00055795	2.591950113	98063.01608	4.89657221	57.12629431
30 to 34	0.00213239	2.597761549	96572.98529	4.80404385	48.99108083	0.00074641	2.652796099	97789.82614	4.880941568	52.27852375
35 to 39	0.002426435	2.589120175	95548.67442	4.749654929	44.48795177	0.001113273	2.643486965	97425.53295	4.85853221	47.46392266
40 to 44	0.00324181	2.600864682	94396.39178	4.683403848	39.99885234	0.001526981	2.658849249	96884.6993	4.826981156	42.713844
45 to 49	0.004105067	2.633174969	92878.50926	4.599254346	35.60935193	0.002306393	2.663971846	96147.73255	4.781628435	38.02051856
50 to 54	0.006075335	2.640767739	90991.19194	4.485299786	31.2925751	0.003403826	2.655150106	95045.12612	4.714635691	33.43019871
55 to 59	0.008620018	2.618109044	88267.63752	4.324642078	27.1757448	0.004973959	2.684141876	93440.81448	4.618854587	28.95796388
60 to 64	0.01176118	2.611912114	84542.37623	4.111728228	23.25694668	0.008126824	2.66120291	91144.36372	4.472258176	24.61918861
65 to 69	0.016493047	2.609697831	79710.78946	3.834539933	19.50757337	0.012002765	2.659396976	87511.9885	4.256122093	20.52963574
70 to 74	0.023506988	2.603526226	73393.96714	3.47428369	15.96143995	0.0191289	2.645814133	82407.8397	3.943031032	16.63551312
75 to 79	0.034474486	2.590425825	65240.29177	3.012313764	12.63105561	0.029677109	2.646417322	74874.79807	3.499696761	13.04223789
80 to 84	0.052100496	2.536446009	54878.40536	2.432518491	9.528405446	0.05160977	2.630932266	64507.40697	2.874684322	9.713135834
85 to 89	0.102824137	2.516547705	42238.489	1.683252641	6.624222041	0.100481434	2.539076688	49706.38049	1.993681479	6.824074025
90 to 94	0.195985159	2.238681294	24975.19935	0.810873746	4.470315832	0.183914772	2.302793316	29727.07799	0.99439221	4.710087286
95 plus	0.297309224	3.364077687	9115.313935	0.307051222	3.364077687	0.282101121	3.546002871	11483.98126	0.40798347	3.546002871

Table 15: Nicaragua 2100 life table, by age and sex. mx=mortality rate, ax=mean person-years lived in an age interval among those who die in that age interval, lx=number of persons left alive at age x, nLx=person-years lived between age x and x+n, ex=life expectancy at age x.

Age Group	Male					Female				
	mx	ax	lx	nLx	ex	mx	ax	lx	nLx	ex
Early Neonatal	0.067489484	0.009586973	100000	0.019165678	83.54094342	0.052674222	0.009587427	100000	0.0191684	86.6390924
Late Neonatal	0.010904776	0.028764115	99870.67282	0.057441819	83.62964276	0.013491056	0.028763402	99899.05414	0.057453871	86.707126
Post Neonatal	0.000902558	0.461579719	99808.0394	0.921131548	83.62445094	0.000747773	0.461590715	99821.55819	0.921322127	86.71669782
1 to 4	0.000201046	1.999731939	99724.92226	3.987396264	82.76986481	0.000196769	1.999737641	99752.68179	3.988540709	85.85250338
5 to 9	8.90155E-05	2.499814551	99644.94049	4.981149096	78.83206836	0.000103175	2.499785053	99674.40909	4.982445156	81.91646188
10 to 14	0.000121981	3.166951788	99601.07506	4.978778685	73.86310794	0.000123998	2.782877956	99623.44192	4.979752148	76.95562988
15 to 19	0.000303566	2.859913852	99541.49151	4.973784395	68.90256344	0.000205393	2.645619875	99562.44823	4.975692003	71.99989373
20 to 24	0.000548485	2.660966699	99392.71376	4.963292041	63.99844107	0.000223754	2.575393218	99461.13174	4.970365456	67.069727
25 to 29	0.000669933	2.671961137	99124.26056	4.948521345	59.16200678	0.000268215	2.618109363	99350.93605	4.964387397	62.14068716
30 to 34	0.001023708	2.603471989	98797.61506	4.927821077	54.34721977	0.000359563	2.63966524	99219.01416	4.956751241	57.21925686
35 to 39	0.001076629	2.609528133	98299.07534	4.90245367	49.60866275	0.000482962	2.647149152	99042.2706	4.946512063	52.31612043
40 to 44	0.001568301	2.62736316	97777.65133	4.870915523	44.85842083	0.0006801	2.695546062	98805.17036	4.932525637	47.43470263
45 to 49	0.00203877	2.712024992	97021.51873	4.82859492	40.18650381	0.001073083	2.693535154	98471.92832	4.911442669	42.58549344
50 to 54	0.003521343	2.691826188	96047.15105	4.763663147	35.56527581	0.00162761	2.686675684	97947.70645	4.879003517	37.79815568
55 to 59	0.005133692	2.626387259	94383.88187	4.662529185	31.14311707	0.002459435	2.694724066	97157.60065	4.830457262	33.0825382
60 to 64	0.006596781	2.628647976	92010.17034	4.530130131	26.87827807	0.003831079	2.674507333	95975.37606	4.756553446	28.45545493
65 to 69	0.009715284	2.639548336	89045.18072	4.352957243	22.68303815	0.005815453	2.707298446	94161.50497	4.646176866	23.94952926
70 to 74	0.013842108	2.649081113	84853.94466	4.110175223	18.67211001	0.009741799	2.728519341	91476.19853	4.475108616	19.56931864
75 to 79	0.021704313	2.648662892	79232.70519	3.771271896	14.80444073	0.017439204	2.748778855	87149.87375	4.193711574	15.39683896
80 to 84	0.034344531	2.555677764	71168.77488	3.287807763	11.1809196	0.034076308	2.70645793	79925.29886	3.710508904	11.53134115
85 to 89	0.075458431	2.606748674	60102.11315	2.555421132	7.756681303	0.072768633	2.648889947	67519.48973	2.894011554	8.12999612
90 to 94	0.162365905	2.252611165	41344.14188	1.441098397	5.120650552	0.148169951	2.341867325	47080.7324	1.704769187	5.527998376
95 plus	0.268703451	3.737198611	18551.69359	0.705443238	3.737198611	0.248008793	4.062967675	22640.31021	0.942432592	4.062967675

Table 15: Panama 2017 life table, by age and sex. mx=mortality rate, ax=mean person-years lived in an age interval among those who die in that age interval, lx=number of persons left alive at age x, nLx=person-years lived between age x and x+n, ex=life expectancy at age x.

Age Group	Male					Female				
	mx	ax	lx	nLx	ex	mx	ax	lx	nLx	ex
Early Neonatal	0.315943117	0.009579357	100000	0.019120101	76.57614012	0.263121387	0.009580976	100000	0.019129778	81.70296239
Late Neonatal	0.044132771	0.028754949	99395.97321	0.057114199	77.02208388	0.031348741	0.028758476	99496.68762	0.057193084	82.09692771
Post Neonatal	0.006721292	0.461166367	99143.9725	0.912550595	77.16023172	0.005778563	0.461233336	99317.42215	0.914544197	82.1875117
1 to 4	0.000990351	1.998679533	98530.84549	3.933440807	76.71430895	0.000905554	1.998792596	98789.07196	3.944416628	81.70135723
5 to 9	0.000320527	2.499332236	98141.45706	4.903143872	73.01086498	0.000231891	2.499516893	98431.97832	4.918747245	77.99056721
10 to 14	0.000344501	2.984292052	97984.34005	4.895816112	68.12396056	0.000256023	2.692386611	98317.93361	4.912994134	73.0781461
15 to 19	0.001123984	2.759787701	97815.70483	4.878501955	63.23627064	0.000469253	2.596901202	98192.15896	4.904078158	68.16829905
20 to 24	0.001760173	2.593560068	97267.39243	4.842856782	58.57711428	0.000476035	2.615538429	97962.04067	4.892548891	63.32230071
25 to 29	0.001940603	2.532518172	96414.98584	4.797776172	54.07194354	0.000736725	2.606623833	97729.14561	4.877856715	58.46693635
30 to 34	0.002100713	2.505281943	95483.95729	4.749309536	49.57436747	0.000857995	2.58377673	97369.79481	4.858418365	53.67304057
35 to 39	0.002034819	2.528688672	94486.30574	4.700678843	45.07123486	0.001090174	2.614588711	96952.96166	4.835074843	48.89262975
40 to 44	0.002429683	2.595149066	93529.85628	4.649328877	40.50616013	0.001471083	2.617347913	96425.87311	4.804453968	44.14550986
45 to 49	0.003211419	2.643442063	92400.30425	4.585317304	35.96946938	0.001941576	2.636529695	95719.13236	4.764095701	39.45202998
50 to 54	0.004767468	2.649051276	90927.92017	4.496010263	31.50895119	0.002787565	2.680497215	94794.20475	4.709263206	34.81113
55 to 59	0.006880573	2.644139721	88784.75536	4.36843743	27.20539647	0.004457964	2.658224769	93481.57295	4.625792069	30.26214942
60 to 64	0.010078905	2.639745736	85779.5513	4.189338615	23.06565655	0.006391673	2.668037685	91419.63877	4.503860816	25.88454346
65 to 69	0.014814094	2.647800736	81558.09998	3.940630003	19.12268102	0.010137118	2.668816734	88541.35446	4.324886355	21.63902457
70 to 74	0.023307103	2.638299367	75722.17602	3.588653079	15.39220501	0.015853181	2.656152266	84158.14716	4.057200902	17.62675453
75 to 79	0.036774924	2.622033534	67361.63543	3.09737263	11.97506313	0.025073943	2.662537096	77728.43185	3.671347057	13.86483768
80 to 84	0.06181772	2.559732095	55977.97112	2.432264941	8.87738336	0.0437947	2.660271995	68527.7396	3.10811516	10.36878492
85 to 89	0.116354539	2.474890755	40953.84674	1.58298522	6.196097226	0.088869674	2.581487111	54925.49457	2.260764412	7.278195562
90 to 94	0.21083879	2.218179619	22549.6136	0.710845645	4.235499058	0.169898432	2.325792578	34850.65767	1.198464158	4.98516584
95 plus	0.309644608	3.229679289	7571.731422	0.244647139	3.229679289	0.268935591	3.718801677	14504.76586	0.539716548	3.718801677

Table 15: Panama 2100 life table, by age and sex. mx=mortality rate, ax=mean person-years lived in an age interval among those who die in that age interval, lx=number of persons left alive at age x, nLx=person-years lived between age x and x+n, ex=life expectancy at age x.										
Age Group	Male					Female				
	mx	ax	lx	nLx	ex	mx	ax	lx	nLx	ex
Early Neonatal	0.067879999	0.009586961	100000	0.019165605	83.04495791	0.061353414	0.009587161	100000	0.019166804	86.72767774
Late Neonatal	0.010925362	0.02876411	99869.91685	0.057441351	83.13385206	0.009720762	0.028764442	99882.41523	0.05745053	86.81039336
Post Neonatal	0.00177054	0.461518059	99807.16796	0.920754605	83.12856219	0.001568303	0.461532426	99826.57728	0.921019627	86.80132188
1 to 4	0.000221486	1.999704685	99644.17731	3.984002598	82.34039144	0.000210347	1.999719537	99682.16605	3.985610203	86.00291468
5 to 9	9.68085E-05	2.499798316	99555.96742	4.976594238	78.41148582	8.05981E-05	2.499832087	99598.35731	4.978915352	82.07348813
10 to 14	0.000137507	3.071714352	99507.80725	4.974044768	73.4481208	0.000121777	2.899043671	99558.26138	4.976559487	77.10541893
15 to 19	0.00047198	2.819639032	99439.42415	4.966753024	68.49643656	0.000235834	2.612876867	99497.71657	4.972072433	72.15046991
20 to 24	0.000781629	2.638642448	99205.21376	4.951074006	63.65036738	0.000235075	2.61919881	99380.53013	4.966266406	67.23233239
25 to 29	0.00094003	2.534855447	98818.93344	4.929532219	58.88770107	0.000389313	2.617546659	99263.85163	4.958603699	62.30812571
30 to 34	0.000922739	2.45686915	98356.96102	4.906412336	54.15130299	0.000458999	2.599222347	99071.14558	4.948029388	57.42325691
35 to 39	0.000789491	2.527527247	97905.9271	4.885833823	49.38873163	0.000526333	2.605144695	98844.65505	4.935969592	52.54797152
40 to 44	0.001049917	2.660016017	97522.65203	4.864145642	44.57160393	0.00067874	2.667477842	98585.3313	4.921452507	47.67819386
45 to 49	0.001520732	2.758036385	97014.43721	4.83408899	39.78958086	0.001043981	2.708727645	98251.81349	4.900869795	42.82986295
50 to 54	0.00275232	2.708730695	96283.28369	4.783926239	35.0684465	0.001721365	2.731454554	97741.35941	4.867985114	38.03761362
55 to 59	0.004159085	2.687663994	94974.0925	4.703195956	30.51185301	0.002871435	2.672434458	96906.30216	4.812945266	33.33934353
60 to 64	0.006108541	2.66456692	93032.09693	4.586320146	26.09110928	0.003835851	2.656454267	95530.8368	4.734035476	28.77841407
65 to 69	0.009323031	2.669748542	90244.07025	4.416495605	21.81126641	0.005836676	2.696406413	93722.03305	4.623923629	24.27914998
70 to 74	0.014597763	2.68014662	86151.27869	4.167092624	17.71822428	0.009483999	2.715370431	91036.9242	4.455313961	19.91241412
75 to 79	0.023683198	2.697181849	80138.61652	3.801119813	13.8428333	0.016190374	2.756224031	86841.89022	4.19000758	15.73834443
80 to 84	0.044509828	2.60346664	71243.80146	3.223733513	10.23445543	0.031884326	2.70465063	80134.81286	3.736894457	11.82111879
85 to 89	0.091273416	2.554808666	57140.8697	2.346731149	7.097594185	0.069032628	2.664966805	68427.65588	2.957379656	8.347738348
90 to 94	0.181861463	2.246037507	36298.79649	1.220200122	4.74027383	0.14293968	2.343574417	48636.61098	1.780021548	5.669951066
95 plus	0.285288248	3.518939303	14687.25211	0.526299532	3.518939303	0.242941265	4.153243014	24088.40697	1.027185232	4.153243014

**Table 15: Venezuela 2017 life table, by age and sex. mx=mortality rate, ax=mean person-years lived in an age interval among those who die in that age interval, lx=number of persons left alive at age x, nLx=person-years lived between age x and x+n, ex=life expectancy at age x.**										
**Age Group**	**Male**					**Female**				
	**mx**	**ax**	**lx**	**nLx**	**ex**	**mx**	**ax**	**lx**	**nLx**	**ex**
Early Neonatal	0.397688062	0.009576852	100000	0.019105137	71.06450731	0.310475198	0.009579525	100000	0.019121101	79.39773375
Late Neonatal	0.048226878	0.02875382	99240.27401	0.057018018	71.58909926	0.039466877	0.028756236	99406.36508	0.057127825	79.85259303
Post Neonatal	0.004561516	0.461319793	98965.34706	0.911813856	71.73033738	0.003808807	0.461373264	99180.92436	0.914117216	79.97650352
1 to 4	0.000638939	1.999148081	98549.52445	3.93694907	71.10776976	0.000573837	1.999234884	98832.80401	3.948779099	79.33332001
5 to 9	0.000297537	2.499380131	98298.03735	4.911248467	67.28459548	0.000230709	2.499519357	98606.23824	4.92746961	75.5110313
10 to 14	0.000431112	3.710998718	98151.93776	4.904873873	62.38103757	0.00027796	2.723607096	98492.56808	4.921515869	70.59530121
15 to 19	0.002828201	2.784857414	97940.50835	4.86654133	57.50758715	0.000531727	2.62937668	98355.77635	4.911599271	65.6896255
20 to 24	0.004383723	2.549597645	96564.3029	4.776911978	53.28596514	0.000611909	2.568926411	98094.62213	4.897447095	60.85728115
25 to 29	0.004007799	2.436509037	94470.77013	4.675519457	49.40826309	0.000739169	2.577415586	97794.96185	4.881009009	56.0356065
30 to 34	0.003315606	2.442830875	92597.69176	4.590983613	45.35692654	0.00089274	2.601053199	97434.20989	4.861302619	51.23319051
35 to 39	0.003226756	2.507663277	91076.3623	4.517518823	41.07266892	0.001183063	2.635652398	97000.28604	4.836488581	46.45033634
40 to 44	0.003551704	2.585159408	89619.7984	4.442922108	36.69840869	0.001679354	2.662226492	96428.22822	4.802558997	41.70968751
45 to 49	0.004833418	2.646850522	88043.36221	4.352718944	32.30815348	0.002521784	2.67004748	95621.95664	4.753177288	37.03821572
50 to 54	0.007243031	2.655050152	85942.11391	4.225437452	28.03209228	0.003810779	2.667815955	94423.79869	4.679619156	32.47339524
55 to 59	0.010834365	2.643464222	82886.29812	4.041308472	23.96619416	0.005762801	2.662569827	92641.49255	4.570550329	28.04562135
60 to 64	0.016050766	2.621141176	78516.01718	3.7817152	20.15155257	0.008699702	2.656777953	90009.63451	4.410660997	23.78629292
65 to 69	0.023016128	2.613962621	72459.91838	3.434897419	16.61558336	0.013211377	2.66129595	86176.69495	4.179887638	19.7242708
70 to 74	0.034894713	2.597363031	64577.56807	2.980004952	13.32434782	0.02111961	2.6565077	80663.51179	3.843399205	15.88844356
75 to 79	0.053207378	2.562019043	54220.54536	2.401195007	10.37521165	0.034650403	2.635214333	72566.98686	3.354453625	12.3629965
80 to 84	0.082529144	2.501154393	41513.17936	1.722714669	7.77343789	0.058550425	2.58637616	60988.1692	2.673483363	9.210143771
85 to 89	0.142498479	2.39959417	27387.24983	1.000934989	5.506693851	0.110228324	2.505748429	45415.77689	1.783416929	6.486685153
90 to 94	0.237755316	2.170629777	13211.39004	0.395761614	3.863636409	0.195214242	2.281475469	25871.26716	0.846786734	4.508838813
95 plus	0.331550268	3.017035112	3846.355559	0.11641286	3.017035112	0.292584264	3.42009169	9428.573613	0.323762191	3.42009169

Table 15: Venezuela 2100 life table, by age and sex. mx=mortality rate, ax=mean person-years lived in an age interval among those who die in that age interval, lx=number of persons left alive at age x, nLx=person-years lived between age x and x+n, ex=life expectancy at age x.

Age Group	Male					Female				
	mx	ax	lx	nLx	ex	mx	ax	lx	nLx	ex
Early Neonatal	0.083909269	0.009586469	100000	0.019162674	79.60532738	0.084330737	0.009586456	100000	0.019162594	85.54973868
Late Neonatal	0.012812192	0.028763589	99839.43387	0.05742072	79.71382649	0.015753344	0.028762778	99838.59297	0.057415396	85.66831101
Post Neonatal	0.000853359	0.461583214	99765.95275	0.920764544	79.71476632	0.00072153	0.461592579	99748.30077	0.920657404	85.68820561
1 to 4	0.000149815	1.999800247	99687.53371	3.986308678	78.85337617	0.000138883	1.999814823	99681.96055	3.986172786	84.82134556
5 to 9	0.000100482	2.499790662	99627.91826	4.980147345	74.898885	0.000105383	2.499780453	99626.69709	4.980024618	80.86693243
10 to 14	0.000172282	3.381479257	99577.99205	4.977413565	69.93466186	0.000148138	2.788234912	99574.30089	4.9770496	75.90796776
15 to 19	0.001053292	2.756763412	99492.40652	4.962649752	64.99157969	0.000273625	2.619266567	99500.72938	4.971781594	70.96187228
20 to 24	0.001627592	2.590603698	98970.63812	4.929109072	60.31664457	0.000285098	2.578435617	99364.88862	4.96481337	66.0551632
25 to 29	0.001691936	2.498559054	98171.20267	4.887909634	55.7836782	0.000373382	2.615649124	99223.56793	4.956747017	61.14524284
30 to 34	0.001499288	2.477413646	97347.96702	4.849105577	51.23249931	0.000468999	2.585148525	99038.78814	4.946324441	56.25399316
35 to 39	0.001409598	2.509974531	96624.91858	4.814436661	46.59651545	0.000546045	2.612509469	98807.17233	4.933937331	51.3792623
40 to 44	0.001573512	2.597405413	95949.9946	4.779483187	41.90534894	0.000768301	2.701762073	98538.1867	4.91820729	46.51181192
45 to 49	0.00228839	2.667338822	95202.02783	4.734856373	37.21160997	0.001266597	2.696673297	98160.96033	4.893760919	41.67921518
50 to 54	0.003779397	2.657949069	94125.15551	4.665028148	32.60258839	0.001948937	2.684966675	97542.23988	4.85523062	36.92508652
55 to 59	0.005765793	2.633423603	92373.40296	4.556634166	28.16383689	0.003023374	2.674141441	96598.17475	4.796022249	32.25763987
60 to 64	0.008344593	2.626718561	89768.75076	4.401796175	23.89765152	0.00428489	2.654884686	95153.73227	4.710482286	27.70438324
65 to 69	0.012842471	2.643331217	86129.14975	4.180864743	19.78700457	0.006414187	2.711354185	93141.87188	4.589597632	23.2418043
70 to 74	0.020581387	2.635291906	80832.49581	3.856524796	15.90202909	0.010895361	2.74569501	90213.18192	4.402401116	18.90452336
75 to 79	0.035190767	2.612863739	73030.67008	3.375108799	12.31047103	0.019972455	2.755871418	85455.10364	4.089838977	14.80003041
80 to 84	0.060500029	2.512215572	61513.26736	2.686509419	9.128023494	0.039559939	2.671498652	77393.30483	3.5489883	11.04723156
85 to 89	0.11311034	2.487731759	45870.52441	1.80556886	6.379774299	0.08096185	2.618684911	63693.41946	2.687782117	7.789421368
90 to 94	0.20622336	2.217775366	26438.98772	0.854197262	4.340848371	0.158259061	2.328041243	42925.27021	1.532026503	5.317402591
95 plus	0.305558731	3.290107368	9575.951224	0.324327232	3.290107368	0.257506725	3.930280716	19900.37784	0.811314524	3.930280716

Table 15: Tropical Latin America 2017 life table, by age and sex. mx=mortality rate, ax=mean person-years lived in an age interval among those who die in that age interval, lx=number of persons left alive at age x, nLx=person-years lived between age x and x+n, ex=life expectancy at age x.

Age Group	Male					Female				
	mx	ax	lx	nLx	ex	mx	ax	lx	nLx	ex
Early Neonatal	0.354060711	0.009578189	100000	0.019113118	71.98706111	0.311643196	0.009579489	100000	0.019120885	78.94321327
Late Neonatal	0.02928102	0.028759046	99323.28202	0.057096794	72.45828076	0.016612586	0.028762541	99404.11266	0.057164085	79.39720684
Post Neonatal	0.008640099	0.461030058	99156.09774	0.91185417	72.52286734	0.006553169	0.46117831	99309.14886	0.914140874	79.41556809
1 to 4	0.000454812	1.999393584	98368.25799	3.931153413	72.17673227	0.000479851	1.999360199	98710.10233	3.944617268	78.97143615
5 to 9	0.00029057	2.499394647	98189.46696	4.905908757	68.3045181	0.000221586	2.499538363	98520.82167	4.923313261	75.11931815
10 to 14	0.000389483	3.295252467	98046.91783	4.899092872	63.40019234	0.000251876	2.681623809	98411.72894	4.917714752	70.19982026
15 to 19	0.001786988	2.740446259	97856.10776	4.873128712	58.51739195	0.000442147	2.632575664	98287.86379	4.909254431	65.28490877
20 to 24	0.00248103	2.551669059	96985.28723	4.819985936	54.0182058	0.00053499	2.575904407	98070.80266	4.897189135	60.42357741
25 to 29	0.002452343	2.506529072	95789.43502	4.760362892	49.66072051	0.000640015	2.607951844	97808.80814	4.882964858	55.57852991
30 to 34	0.002621762	2.541382082	94622.03194	4.700800693	45.24248494	0.000871417	2.648341747	97496.29155	4.864845187	50.74832215
35 to 39	0.003046619	2.582282143	93389.5953	4.635336545	40.80599717	0.001269566	2.657453056	97072.36138	4.839226131	45.95838127
40 to 44	0.003929153	2.616487505	91977.38638	4.556199732	36.39287622	0.001849705	2.664574757	96457.99004	4.802154934	41.23417656
45 to 49	0.005420103	2.635122164	90187.18849	4.452290617	32.06332727	0.002771975	2.66132618	95569.73395	4.747708533	36.59265244
50 to 54	0.007785951	2.639834449	87774.00669	4.309508455	27.87239612	0.004085979	2.660332927	94253.68241	4.668058338	32.06642776
55 to 59	0.011350411	2.627857011	84418.65495	4.110265105	23.87529858	0.006115715	2.658285269	92346.32685	4.55212433	27.67378809
60 to 64	0.016196418	2.61637495	79753.3546	3.839442251	20.11819632	0.009175697	2.65716231	89562.3843	4.383878544	23.45136068
65 to 69	0.023372914	2.610036115	73534.86736	3.482226112	16.59823605	0.014052603	2.653768331	85539.88418	4.140480505	19.42919853
70 to 74	0.034862611	2.594357926	65395.94993	3.016790887	13.3391491	0.021939809	2.652740281	79721.46204	3.790851677	15.65353609
75 to 79	0.053116207	2.559878816	54878.73655	2.429104846	10.39833375	0.03624594	2.628029234	71404.4741	3.287579577	12.16782574
80 to 84	0.081592102	2.502388169	41976.43087	1.743523102	7.807657432	0.060256655	2.577574041	59488.48066	2.595563009	9.078720475
85 to 89	0.141439897	2.402286997	27750.90037	1.01472215	5.527254041	0.112680533	2.497106839	43848.75271	1.710140791	6.397531418
90 to 94	0.236757065	2.172794247	13398.91466	0.401320981	3.874562028	0.198102329	2.276237373	24579.16239	0.798245882	4.455439235
95 plus	0.330765922	3.023288525	3897.477038	0.117832975	3.023288525	0.295279042	3.386634274	8766.000999	0.296876282	3.386634274

Table 15: Tropical Latin America 2100 life table, by age and sex. mx=mortality rate, ax=mean person-years lived in an age interval among those who die in that age interval, lx=number of persons left alive at age x, nLx=person-years lived between age x and x+n, ex=life expectancy at age x.

Age Group	Male					Female				
	mx	ax	lx	nLx	ex	mx	ax	lx	nLx	ex
Early Neonatal	0.069180691	0.009586921	100000	0.019165366	80.10719572	0.067158681	0.009586983	100000	0.019165737	85.24202469
Late Neonatal	0.005744219	0.028765539	99867.4163	0.057448472	80.19432351	0.00422349	0.028765958	99871.29036	0.057453214	85.33265756
Post Neonatal	0.001706171	0.461522632	99834.4174	0.921033301	80.16328423	0.001406058	0.461543952	99847.02691	0.921277212	85.29584774
1 to 4	9.45059E-05	1.999873992	99677.29385	3.986338363	79.36558139	9.0836E-05	1.999878885	99717.50514	3.987975791	84.48265555
5 to 9	8.36581E-05	2.499825712	99639.62693	4.980939553	75.39481962	8.13839E-05	2.49983045	99681.28692	4.983050515	80.51259775
10 to 14	0.000147887	3.386590064	99597.95835	4.978694279	70.42528566	0.000118723	2.751388263	99640.73663	4.980703249	75.54432234
15 to 19	0.000699384	2.762537718	99524.3329	4.9684259	65.47485287	0.000220984	2.601551277	99581.60898	4.976442538	70.58751483
20 to 24	0.001017679	2.561744971	99176.90557	4.946555078	60.69410133	0.000226303	2.550821467	99471.64642	4.970825651	65.66259782
25 to 29	0.000988935	2.515211399	98673.70968	4.921586165	55.99009745	0.000275042	2.613189905	99359.1651	4.964698726	60.73398051
30 to 34	0.001089856	2.545513062	98187.2277	4.896264531	51.25474069	0.000376003	2.635249175	99222.62819	4.956721415	55.81387443
35 to 39	0.00123258	2.596200717	97653.92114	4.868272491	46.52041492	0.000517561	2.656579298	99036.27774	4.945815435	50.91381211
40 to 44	0.001668608	2.649732334	97054.25063	4.833762573	41.79145411	0.000767706	2.700217157	98780.33275	4.930307186	46.0386873
45 to 49	0.002459792	2.686697857	96248.20983	4.785193858	37.11875798	0.00125717	2.684829898	98401.89935	4.905812193	41.20511206
50 to 54	0.003944017	2.681636322	95072.02963	4.71055151	32.54398478	0.001894573	2.682175398	97785.29036	4.867895407	36.4476304
55 to 59	0.006071401	2.647741565	93215.81077	4.595201031	28.13752123	0.002961124	2.669125954	96863.28061	4.809961035	31.76852634
60 to 64	0.008620712	2.645155601	90428.99047	4.431588903	23.92170061	0.004378869	2.678031668	95439.72766	4.723983522	27.20170724
65 to 69	0.012955693	2.65241195	86613.89145	4.203026407	19.85691691	0.006946925	2.700058309	93372.52015	4.595221434	22.7432558
70 to 74	0.020046316	2.661829162	81178.9809	3.877607635	16.00644149	0.011618936	2.727422251	90183.85021	4.393319512	18.45012975
75 to 79	0.033763996	2.641315825	73425.67498	3.401552257	12.41293814	0.02160212	2.714715021	85086.79888	4.054613356	14.38970463
80 to 84	0.057177456	2.570303791	61994.96995	2.724526897	9.21184738	0.040459053	2.651825619	76352.55219	3.48804787	10.72193215
85 to 89	0.109840912	2.495329697	46544.31984	1.829630923	6.414201369	0.083492335	2.603095234	62313.30275	2.599442441	7.534280819
90 to 94	0.203530195	2.227176646	26678.2087	0.856165257	4.355913243	0.162926306	2.333541295	40788.35766	1.425761798	5.145044238
95 plus	0.303538839	3.298627508	9437.320157	0.313664973	3.298627508	0.262271975	3.819848533	17767.76368	0.683529449	3.819848533

Table 15: Brazil 2017 life table, by age and sex. mx=mortality rate, ax=mean person-years lived in an age interval among those who die in that age interval, lx=number of persons left alive at age x, nLx=person-years lived between age x and x+n, ex=life expectancy at age x.

Age Group	Male					Female				
	mx	ax	lx	nLx	ex	mx	ax	lx	nLx	ex
Early Neonatal	0.359404537	0.009578025	100000	0.01911214	71.93182683	0.320834648	0.009579208	100000	0.019119202	78.93514948
Late Neonatal	0.029802286	0.028758902	99313.10361	0.057090087	72.41009073	0.016720654	0.028762511	99386.59199	0.057153831	79.40309154
Post Neonatal	0.008732512	0.461023493	99142.96313	0.911694541	72.4767704	0.006521657	0.461180548	99291.02762	0.913987352	79.42195237
1 to 4	0.000461706	1.999384392	98346.8368	3.930243171	72.13645965	0.000492626	1.999343165	98694.96213	3.943911504	78.97554771
5 to 9	0.000293106	2.499389363	98165.37767	4.90467408	68.26610987	0.000222367	2.499536736	98500.67694	4.922296977	75.12737894
10 to 14	0.000391645	3.303710838	98021.62064	4.897827007	63.36256343	0.00025185	2.680036186	98391.22242	4.916688354	70.20817387
15 to 19	0.001813937	2.740150136	97829.80094	4.871520604	58.48032463	0.000440978	2.633816509	98267.39607	4.90824837	65.29326605
20 to 24	0.002512473	2.551103132	96946.13949	4.817664925	53.9883941	0.00053685	2.576899304	98050.95337	4.896178528	60.43158351
25 to 29	0.002481581	2.50552133	95735.7151	4.757336675	49.63873427	0.000642098	2.607465007	97788.1025	4.881905344	55.58709442
30 to 34	0.002643506	2.540594468	94555.14463	4.697218512	45.2272155	0.00087288	2.648032941	97474.63662	4.86374664	50.75746958
35 to 39	0.003071569	2.581544175	93313.43298	4.631268518	40.79523996	0.001271432	2.657108412	97050.09028	4.838092676	45.96792389
40 to 44	0.003951328	2.615622287	91890.90801	4.551662166	36.38680801	0.001850512	2.664145947	96434.95983	4.800995569	41.24418925
45 to 49	0.005442962	2.634412741	90092.39944	4.447356786	32.06097715	0.00277068	2.660918801	95546.53047	4.74656479	36.60291957
50 to 54	0.007806889	2.639283499	87671.72442	4.304259528	27.87346081	0.004080205	2.660387012	94231.41052	4.667018744	32.07662084
55 to 59	0.011370083	2.627334129	84311.44497	4.104834769	23.87917897	0.006110683	2.658178704	92327.17367	4.551230114	27.68332353
60 to 64	0.016204397	2.615852156	79644.22902	3.83408714	20.12455012	0.009161812	2.657025223	89546.06678	4.383213991	23.46054575
65 to 69	0.02335684	2.609693556	73431.34814	3.477423858	16.6059231	0.014027907	2.654062883	85530.26039	4.140263382	19.43730464
70 to 74	0.034812344	2.594561719	65309.23075	3.013145764	13.34654873	0.021922099	2.652943316	79722.36366	3.791060273	15.65998337
75 to 79	0.053091366	2.559904844	54819.8449	2.42663042	10.4038674	0.036218928	2.62795437	71411.62234	3.288094088	12.17371135
80 to 84	0.081466543	2.502274834	41936.66875	1.742310808	7.813588348	0.060174701	2.577583117	59502.62674	2.596630593	9.084222361
85 to 89	0.141283925	2.402719685	27742.84514	1.014772427	5.530994932	0.112565817	2.497485888	43877.71209	1.71170961	6.401261489
90 to 94	0.236601191	2.173093233	13405.91643	0.401653161	3.876567512	0.197971903	2.276497891	24610.03447	0.799459086	4.457647378
95 plus	0.330640441	3.02443524	3902.844332	0.118039744	3.02443524	0.295158828	3.388012755	8783.23248	0.297580457	3.388012755

Table 15: Brazil 2100 life table, by age and sex. mx=mortality rate, ax=mean person-years lived in an age interval among those who die in that age interval, lx=number of persons left alive at age x, nLx=person-years lived between age x and x+n, ex=life expectancy at age x.

Age Group	Male					Female				
	mx	ax	lx	nLx	ex	mx	ax	lx	nLx	ex
Early Neonatal	0.071680226	0.009586844	100000	0.019164907	80.11056916	0.070770636	0.009586872	100000	0.019165074	85.26099281
Late Neonatal	0.0059643	0.028765478	99862.62935	0.057445355	80.20154085	0.004301856	0.028765937	99864.37271	0.057449105	85.3575548
Post Neonatal	0.001698514	0.461523176	99828.36798	0.920980747	80.17151918	0.001350431	0.461547903	99839.66092	0.9212329	85.32113191
1 to 4	8.33391E-05	1.999888881	99671.95865	3.986214024	79.37325729	8.3678E-05	1.999888429	99715.26977	3.987943486	84.50359939
5 to 9	8.50162E-05	2.499822883	99638.74464	4.980878537	75.39903815	8.21681E-05	2.499828816	99681.90674	4.98307174	80.53118169
10 to 14	0.000149499	3.384009225	99596.40011	4.978604099	70.43000157	0.000119099	2.748488165	99640.96589	4.980709522	75.56321742
15 to 19	0.000708282	2.761877492	99521.97313	4.968204535	65.48011632	0.000221284	2.602655614	99581.6508	4.976442341	70.60655741
20 to 24	0.001025922	2.560902033	99170.14018	4.946114231	60.70220544	0.000228063	2.550652791	99471.53955	4.970798714	65.68174953
25 to 29	0.000996637	2.51328905	98662.89601	4.92094516	56.00070115	0.000275574	2.610147453	99358.18502	4.964639534	60.75369905
30 to 34	0.001091739	2.544783627	98172.66658	4.895511885	51.26749892	0.000374248	2.634224431	99221.38605	4.956678344	55.83377419
35 to 39	0.001237451	2.595426469	97638.49688	4.867442361	46.53371855	0.000515394	2.656500198	99035.90824	4.945821544	50.93327969
40 to 44	0.001669283	2.648695058	97036.5241	4.83286313	41.80593559	0.000763757	2.699927268	98781.03569	4.930386043	46.05768281
45 to 49	0.002457041	2.686098568	96230.27265	4.784325631	37.13351968	0.001250194	2.683280803	98404.54412	4.90601373	41.22332394
50 to 54	0.003936798	2.681259741	95055.55822	4.70980521	32.55849303	0.001875032	2.682049954	97791.32847	4.868414173	36.46462966
55 to 59	0.006054179	2.647168498	93202.97415	4.594737819	28.15125286	0.002935229	2.668796637	96878.71756	4.811016139	31.78238381
60 to 64	0.008588999	2.645096845	90424.14735	4.431669825	23.93367102	0.004338041	2.679035803	95467.25123	4.725804666	27.21198452
65 to 69	0.01290742	2.652075332	86622.86795	4.203905491	19.86599432	0.006898137	2.70147598	93418.51885	4.598039937	22.74869443
70 to 74	0.019949249	2.663262564	81206.70848	3.879867417	16.01181328	0.011580838	2.728369769	90250.05393	4.396959534	18.45081928
75 to 79	0.033770695	2.6419495	73485.71067	3.404332106	12.41184292	0.02156145	2.715659734	85165.02534	4.058769958	14.38741436
80 to 84	0.05718003	2.570254275	62042.22073	2.726535206	9.210982429	0.040504648	2.65181892	76436.93526	3.491534761	10.71682836
85 to 89	0.109849709	2.495291554	46576.98859	1.830796961	6.413573677	0.083567651	2.602786768	62365.85411	2.601123659	7.530550854
90 to 94	0.203544458	2.227188989	26692.53312	0.856544226	4.355545702	0.163025452	2.333442474	40804.16517	1.425971803	5.14270202
95 plus	0.303551736	3.298416263	9439.594489	0.313680296	3.298416263	0.262367053	3.818366613	17762.7933	0.683006104	3.818366613

Table 15: Paraguay 2017 life table, by age and sex. mx=mortality rate, ax=mean person-years lived in an age interval among those who die in that age interval, lx=number of persons left alive at age x, nLx=person-years lived between age x and x+n, ex=life expectancy at age x.

Age Group	Male					Female				
	mx	ax	lx	nLx	ex	mx	ax	lx	nLx	ex
Early Neonatal	0.245704663	0.00958151	100000	0.01913297	73.51047521	0.12206808	0.0095853	100000	0.019155652	78.92582956
Late Neonatal	0.018714437	0.028761961	99529.92781	0.057232981	73.83835472	0.014385996	0.028763155	99766.17727	0.057375972	79.09157203
Post Neonatal	0.006744352	0.461164728	99422.8367	0.915107338	73.86031816	0.007211386	0.461131551	99683.64173	0.91730997	79.09949207
1 to 4	0.000313474	1.999582034	98805.82165	3.949756781	73.39546059	0.00021346	1.999715387	99022.23643	3.959199239	78.70149024
5 to 9	0.00023493	2.499510562	98682.04485	4.931205968	69.48502835	0.000204146	2.499574695	98937.73822	4.944363282	74.7670088
10 to 14	0.000336784	3.038168519	98566.2167	4.925059299	64.56375947	0.000252524	2.718651179	98836.81028	4.938997039	69.8408117
15 to 19	0.001111174	2.745409453	98400.36617	4.90772629	59.66736784	0.000471913	2.601311192	98712.09482	4.930025951	64.92553026
20 to 24	0.001660893	2.563694313	97855.06897	4.873036298	54.98383565	0.000485267	2.544227015	98479.44926	4.918113008	60.07249759
25 to 29	0.001639268	2.532530067	97045.82107	4.832746397	50.41992158	0.000578678	2.619777255	98240.80599	4.905285087	55.21194868
30 to 34	0.001947005	2.552418524	96253.78148	4.789872723	45.81307148	0.000822606	2.654394758	97956.97945	4.888420529	50.36396927
35 to 39	0.002168431	2.603666878	95321.49331	4.741451776	41.23516541	0.001198651	2.669834663	97554.91975	4.864162858	45.56009169
40 to 44	0.003089461	2.651223063	94293.84388	4.680750498	36.65502464	0.001816738	2.682511354	96972.0295	4.828275449	40.81710993
45 to 49	0.00452513	2.666496727	92848.74935	4.593970752	32.18269627	0.002826452	2.679054739	96095.18902	4.773455767	36.16403532
50 to 54	0.006955921	2.664429688	90771.97955	4.466126872	27.85595574	0.004337978	2.658515914	94746.7054	4.689729573	31.63922771
55 to 59	0.010582271	2.650349301	87669.67077	4.277301355	23.74485731	0.006338202	2.663630665	92713.82984	4.568107639	27.27286952
60 to 64	0.01588218	2.637937131	83151.80624	4.00758631	19.88822152	0.009807841	2.663805942	89821.57403	4.390618644	23.06310132
65 to 69	0.024046751	2.62384877	76802.57126	3.633204605	16.31176853	0.015217894	2.641161571	85521.87819	4.128231281	19.086069
70 to 74	0.036979823	2.586483822	68095.80808	3.127017318	13.06025409	0.022783419	2.643548906	79254.09994	3.761437857	15.38371662
75 to 79	0.054189085	2.55895392	56588.91455	2.500866474	10.19121429	0.037533638	2.631930504	70715.12691	3.248466497	11.92001733
80 to 84	0.086850443	2.507190198	43130.51023	1.775435293	7.580371937	0.064166347	2.577522918	58587.89925	2.537692443	8.843622704
85 to 89	0.147802011	2.385171025	27840.80649	1.006517904	5.384804065	0.117978052	2.480516056	42421.32142	1.63818872	6.240249088
90 to 94	0.242991015	2.160357771	13086.20576	0.388316938	3.798344713	0.203967046	2.263796595	23247.68559	0.748165546	4.36312482
95 plus	0.335743325	2.979689154	3710.889737	0.111052072	2.979689154	0.300628944	3.329181565	8097.557115	0.271051558	3.329181565

Table 15: Paraguay 2100 life table, by age and sex. mx=mortality rate, ax=mean person-years lived in an age interval among those who die in that age interval, lx=number of persons left alive at age x, nLx=person-years lived between age x and x+n, ex=life expectancy at age x.

Age Group	Male					Female				
	mx	ax	lx	nLx	ex	mx	ax	lx	nLx	ex
Early Neonatal	0.039862587	0.009587819	100000	0.019170754	80.10309673	0.022873064	0.00958834	100000	0.019173877	85.04148134
Late Neonatal	0.003167894	0.028766249	99923.59376	0.057485049	80.14511255	0.003210646	0.028766238	99956.15106	0.057503707	85.05951518
Post Neonatal	0.001831735	0.461513712	99905.38547	0.921634761	80.10214921	0.002117775	0.461493392	99937.69005	0.921811083	85.01768507
1 to 4	0.000235391	1.999686146	99736.63242	3.987587905	79.31350886	0.000182396	1.999756805	99742.53813	3.988246555	84.25935369
5 to 9	6.58563E-05	2.499862799	99642.77883	4.98131892	75.38636003	7.30082E-05	2.4998479	99669.79901	4.982580853	80.3193871
10 to 14	0.000124601	3.314230742	99609.98029	4.979382601	70.41028534	0.000119296	2.901857027	99633.43803	4.980330564	75.34766849
15 to 19	0.00057147	2.749712874	99547.95773	4.970961079	65.45199344	0.000214982	2.577443467	99574.08936	4.976102317	70.39058703
20 to 24	0.00087813	2.569988991	99264.01709	4.952608262	60.63020627	0.000190195	2.563402573	99467.17337	4.971043939	65.46361899
25 to 29	0.000846234	2.565297247	98830.0406	4.931263057	55.88313576	0.000260976	2.693050486	99372.67854	4.965611451	60.52355357
30 to 34	0.001057657	2.556016953	98413.98881	4.908009185	51.10759307	0.000399858	2.668706138	99243.2153	4.957503137	55.59871045
35 to 39	0.00113325	2.613390299	97896.45786	4.88158051	46.36355856	0.000548836	2.668596419	99045.14332	4.945918787	50.70400326
40 to 44	0.001650079	2.660663864	97345.48794	4.8485784	41.60994392	0.000837498	2.713044758	98773.9272	4.929235815	45.83480849
45 to 49	0.002516483	2.692074854	96548.15206	4.799471079	36.92934274	0.001396366	2.72089288	98361.54046	4.90244166	41.0137333
50 to 54	0.004083889	2.675897391	95345.56746	4.722524028	32.3563277	0.002298861	2.685801	97678.27927	4.858104276	36.27755918
55 to 59	0.006433057	2.660062826	93425.16404	4.601890392	27.95998033	0.003507646	2.700129334	96564.72859	4.78927269	31.65895891
60 to 64	0.009332962	2.636306999	90490.59921	4.427477178	23.77088708	0.005297554	2.651517671	94894.42266	4.687021936	27.1605369
65 to 69	0.014139676	2.656416127	86394.64295	4.182277948	19.75925451	0.008168905	2.694387739	92423.9153	4.536148965	22.8017178
70 to 74	0.022609685	2.628144256	80572.55417	3.826958844	15.98302551	0.012767778	2.735064922	88782.5866	4.315604664	18.60184934
75 to 79	0.034267821	2.613682888	72116.60215	3.339458298	12.52967796	0.023253114	2.7154397	83409.91941	3.964562097	14.60047589
80 to 84	0.059838714	2.560769392	60981.65633	2.679370367	9.325931496	0.041104564	2.662941553	74525.09953	3.413850558	10.99957763
85 to 89	0.111357786	2.495836266	45875.85929	1.826390266	6.513627738	0.082810893	2.61399846	61250.38579	2.587643712	7.767221738
90 to 94	0.203287716	2.21481063	27216.00098	0.894005542	4.419516477	0.15999206	2.321150896	41429.02599	1.484868604	5.309734864
95 plus	0.302883883	3.335214552	10377.94506	0.363692304	3.335214552	0.259003118	3.926153585	19467.70807	0.80690009	3.926153585

Table 15: North Africa and Middle East 2017 life table, by age and sex. mx=mortality rate, ax=mean person-years lived in an age interval among those who die in that age interval, lx=number of persons left alive at age x, nLx=person-years lived between age x and x+n, ex=life expectancy at age x.

Age Group	Male					Female				
	mx	ax	lx	nLx	ex	mx	ax	lx	nLx	ex
Early Neonatal	0.565528399	0.009571708	100000	0.019074457	71.80922891	0.424428554	0.009576032	100000	0.019100242	76.65652223
Late Neonatal	0.067032305	0.028748632	98921.29484	0.056804017	72.57297604	0.060332931	0.028750481	99189.33614	0.056968906	77.26375491
Post Neonatal	0.010374107	0.460906877	98540.5329	0.905469391	72.79575028	0.010521733	0.46089639	98845.6311	0.908211043	77.47477994
1 to 4	0.001418911	1.99810812	97601.22648	3.892991525	72.56860025	0.001358961	1.998188053	97890.06448	3.904979909	77.30326053
5 to 9	0.000593741	2.498763039	97048.87358	4.845248188	68.97026636	0.000507991	2.498941686	97359.41274	4.861793734	73.71371705
10 to 14	0.000576994	2.722705032	96761.19635	4.831711017	64.16789235	0.000432685	2.582544729	97112.4412	4.85054854	68.89482237
15 to 19	0.001217429	2.698789717	96482.41247	4.810643389	59.34543367	0.00068258	2.602706741	96902.56662	4.837213319	64.0384397
20 to 24	0.001754474	2.544019756	95896.75272	4.774265664	54.69136711	0.000773532	2.53316771	96572.38954	4.819423469	59.2484711
25 to 29	0.00161109	2.487452796	95059.12465	4.733794347	50.15084731	0.000811645	2.555381877	96199.59561	4.800455036	54.46823471
30 to 34	0.001686186	2.546799966	94296.47472	4.695401344	45.53631659	0.00099677	2.609318591	95809.97549	4.779110475	49.67932006
35 to 39	0.002021595	2.602634923	93504.75256	4.652689059	40.90029294	0.001345957	2.646291211	95333.61756	4.751628077	44.91449164
40 to 44	0.002729583	2.65010492	92564.18486	4.598712971	36.28941208	0.001963504	2.667925484	94694.0861	4.713123246	40.19992455
45 to 49	0.004079624	2.682288286	91308.96352	4.522685817	31.75180531	0.002975497	2.656032158	93768.69495	4.655962395	35.57028258
50 to 54	0.006512135	2.677103184	89463.95041	4.406542669	27.35124122	0.004291564	2.651271654	92383.37802	4.573075248	31.06379261
55 to 59	0.010127427	2.6825035	86594.50409	4.230441554	23.16876123	0.006306852	2.672773782	90420.913	4.455650905	26.68038981
60 to 64	0.016722616	2.653917235	82310.46383	3.960169321	19.23489271	0.010027781	2.674521626	87610.96907	4.280730621	22.45030927
65 to 69	0.02573211	2.622820732	75688.68679	3.566313539	15.68538406	0.015949951	2.662089283	83318.65446	4.01618482	18.46901197
70 to 74	0.039639032	2.587946986	66513.18006	3.035489129	12.48729066	0.025575378	2.644107874	76913.49456	3.627157838	14.78528656
75 to 79	0.060024655	2.550764824	54483.30647	2.375087712	9.67312407	0.041687391	2.612838929	67638.25454	3.075877158	11.45015053
80 to 84	0.095608349	2.486666133	40230.8563	1.621928732	7.196668389	0.069232584	2.546937339	54818.32175	2.343092562	8.516926087
85 to 89	0.158206268	2.35709617	24728.83397	0.871974659	5.150013886	0.124810748	2.458389083	38600.96152	1.465357822	6.025443254
90 to 94	0.253208761	2.140088914	10938.01973	0.317237898	3.672660464	0.211657429	2.248078838	20317.34089	0.642026338	4.236350883
95 plus	0.343908765	2.907793153	2907.293861	0.084552513	2.907793153	0.307689883	3.250127426	6732.176222	0.21885053	3.250127426

Table 15: North Africa and Middle East 2100 life table, by age and sex. mx=mortality rate, ax=mean person-years lived in an age interval among those who die in that age interval, lx=number of persons left alive at age x, nLx=person-years lived between age x and x+n, ex=life expectancy at age x.

Age Group	Male					Female				
	mx	ax	lx	nLx	ex	mx	ax	lx	nLx	ex
Early Neonatal	0.102160186	0.00958591	100000	0.01915931	81.65000396	0.081334773	0.009586548	100000	0.019163134	85.5388421
Late Neonatal	0.011007785	0.028764087	99804.30544	0.057403477	81.79081278	0.011430603	0.02876397	99844.15361	0.057425698	85.65310713
Post Neonatal	0.001374194	0.461546215	99741.12355	0.920313631	81.785017	0.001477832	0.461538853	99778.5183	0.920614631	85.65185646
1 to 4	0.000266731	1.999644359	99614.68012	3.982462807	80.96492603	0.000290302	1.999612931	99642.49141	3.983386998	84.84479271
5 to 9	0.00015984	2.499666999	99508.48239	4.97343692	77.04899495	0.000181868	2.499621108	99526.8853	4.97408296	80.94091761
10 to 14	0.000214452	2.927664614	99429.00627	4.969231268	72.10845334	0.000192839	2.652185891	99436.44834	4.969570598	76.01219926
15 to 19	0.000590363	2.778809591	99322.47758	4.959632933	67.18250626	0.000320915	2.606707709	99340.647	4.963216263	71.08290662
20 to 24	0.001020318	2.581059206	99029.79904	4.939310586	62.37242257	0.000351813	2.545431761	99181.46691	4.954792228	66.19271062
25 to 29	0.000984078	2.498168009	98526.78199	4.914233713	57.67693385	0.000394766	2.590981218	99007.22613	4.945655729	61.30467771
30 to 34	0.00099541	2.512416541	98043.99472	4.890091878	52.94840047	0.000521439	2.606586883	98812.09294	4.934441357	56.42052466
35 to 39	0.001032437	2.550214978	97558.11573	4.865610508	48.19944351	0.000656178	2.621015118	98554.93122	4.920065071	51.56078759
40 to 44	0.001247743	2.615379597	97056.55239	4.838441292	43.43510749	0.000902965	2.667415954	98232.23668	4.901283885	46.72125535
45 to 49	0.001732041	2.686422309	96453.64716	4.803454026	38.6896611	0.001383382	2.671368901	97789.85584	4.873785764	41.92004737
50 to 54	0.002835939	2.705420936	95622.65842	4.750234398	34.00119776	0.002048335	2.665724405	97116.00679	4.832691922	37.19147584
55 to 59	0.004601831	2.68927026	94277.4876	4.66429619	29.44523724	0.003046147	2.653233219	96126.81077	4.772223359	32.54558304
60 to 64	0.00719286	2.659800811	92135.74017	4.530674706	25.06359761	0.004357185	2.651663215	94674.64987	4.685850511	28.00252875
65 to 69	0.010632611	2.666574631	88887.13154	4.337040038	20.87729128	0.006447665	2.666048631	92635.76948	4.563189244	23.55819842
70 to 74	0.016935657	2.679048684	84295.98554	4.056177673	16.86400182	0.009917368	2.724054955	89700.39551	4.38624071	19.23915808
75 to 79	0.028996713	2.663298301	77465.5635	3.629525018	13.10876874	0.018522994	2.733566543	85364.65774	4.096839803	15.07402129
80 to 84	0.050359844	2.594971069	67047.72314	2.99521896	9.727370888	0.03556522	2.664630567	77814.38182	3.594606429	11.26667015
85 to 89	0.100090049	2.526146704	52204.73186	2.100793392	6.753692355	0.075548955	2.635507653	65144.01322	2.768821338	7.929985511
90 to 94	0.192411445	2.239133882	31615.35645	1.039442197	4.544929997	0.152453032	2.343403792	44514.19181	1.591185169	5.394571535
95 plus	0.29422012	3.406914688	11990.68956	0.413819044	3.406914688	0.252232842	3.977785299	20621.50154	0.829709296	3.977785299

Table 15: Afghanistan 2017 life table, by age and sex. mx=mortality rate, ax=mean person-years lived in an age interval among those who die in that age interval, lx=number of persons left alive at age x, nLx=person-years lived between age x and x+n, ex=life expectancy at age x.

Age Group	Male					Female				
	mx	ax	lx	nLx	ex	mx	ax	lx	nLx	ex
Early Neonatal	1.175072914	0.009553026	100000	0.018963621	62.82153357	0.879317594	0.00956209	100000	0.019017292	62.45337476
Late Neonatal	0.147766962	0.028726362	97771.94099	0.056013977	64.23281584	0.125850961	0.028732407	98327.94107	0.056367965	63.49547561
Post Neonatal	0.027515644	0.459689192	96944.49676	0.883807627	64.72314119	0.031866894	0.459380098	97618.70623	0.888176106	63.89893637
1 to 4	0.0034109	1.995452147	94513.84638	3.754898699	65.45275486	0.003192612	1.995743196	94789.5365	3.767488483	64.8693801
5 to 9	0.000895234	2.49813493	93234.03135	4.651285609	62.32432261	0.000921547	2.498080111	93587.47083	4.668612286	61.67739867
10 to 14	0.000755762	2.671582282	92817.70689	4.632737354	57.5922331	0.000711101	2.651425016	93157.35232	4.650112288	56.95038615
15 to 19	0.001533044	2.721602994	92467.62466	4.607294109	52.79977523	0.001459312	2.690422172	92826.7325	4.625755589	52.14337392
20 to 24	0.002420006	2.59546919	91761.34871	4.561538039	48.18409777	0.002076364	2.614864961	92151.73334	4.584896356	47.50423829
25 to 29	0.002699914	2.552657755	90657.77472	4.503161624	43.73740701	0.002659375	2.606352363	91200.02099	4.531188081	42.97077031
30 to 34	0.003192282	2.589059122	89442.72111	4.438013774	39.29507975	0.003526863	2.626781449	89995.82274	4.462474971	38.50833173
35 to 39	0.004179619	2.62656156	88027.51766	4.358194806	34.88334823	0.004940282	2.631567128	88423.95615	4.370115415	34.14331716
40 to 44	0.005925367	2.645940168	86208.71753	4.251229841	30.56204446	0.006873094	2.624842353	86269.11845	4.244282723	29.92767014
45 to 49	0.008717322	2.67481953	83694.82965	4.101763912	26.39875626	0.009534639	2.63970535	83358.74783	4.076393435	25.87881498
50 to 54	0.014180696	2.668183469	80128.27177	3.87848289	22.45342726	0.014303214	2.639499542	79482.59848	3.844653527	22.01067875
55 to 59	0.022538864	2.624795505	74644.92299	3.543123598	18.90664605	0.021440949	2.612047871	73999.53499	3.520264183	18.44550162
60 to 64	0.033286189	2.538194654	66684.91497	3.08250706	15.85203587	0.030844713	2.582979665	66475.29441	3.093839118	15.2382317
65 to 69	0.038184925	2.532818046	56460.29443	2.580720913	13.26789233	0.044100048	2.556523115	56963.37575	2.571981938	12.35331891
70 to 74	0.057814421	2.537598267	46639.4105	2.042083061	10.53396966	0.064101372	2.523520956	45660.40035	1.971538447	9.78184658
75 to 79	0.083184554	2.479536181	34865.21104	1.441730897	8.239983861	0.096250632	2.463460631	33082.46458	1.331346168	7.54859136
80 to 84	0.122381319	2.409076061	22899.91617	0.86979979	6.255484688	0.140284129	2.370692232	20355.41398	0.745276352	5.744841935
85 to 89	0.187601429	2.284076368	12276.41249	0.406929226	4.590117071	0.20754021	2.252866538	9985.860494	0.318984942	4.279446954
90 to 94	0.280861607	2.081957658	4657.040399	0.128075224	3.372638186	0.296225343	2.055143865	3416.559406	0.091579105	3.220025664
95 plus	0.365564463	2.735701811	1065.425836	0.029177918	2.735701811	0.381415234	2.622733511	721.9221073	0.019029986	2.622733511

Table 15: Afghanistan 2100 life table, by age and sex. mx=mortality rate, ax=mean person-years lived in an age interval among those who die in that age interval, lx=number of persons left alive at age x, nLx=person-years lived between age x and x+n, ex=life expectancy at age x.

Age Group	Male					Female				
	mx	ax	lx	nLx	ex	mx	ax	lx	nLx	ex
Early Neonatal	0.200622741	0.009582892	100000	0.019141275	77.95274108	0.141467364	0.009584705	100000	0.019152106	80.64253525
Late Neonatal	0.018526943	0.028762013	99616.6139	0.057283162	78.23208212	0.018614717	0.028761988	99729.31006	0.057347816	80.84110841
Post Neonatal	0.003122291	0.461422033	99510.60062	0.917447991	78.25749339	0.003528423	0.461393182	99622.65305	0.918308937	80.86971698
1 to 4	0.000596808	1.999204256	99224.66126	3.964257768	77.55802457	0.000701067	1.999065245	99299.14668	3.966409013	80.20730417
5 to 9	0.000307388	2.499359608	98988.36588	4.94562067	73.73625838	0.000397991	2.499170852	99021.49899	4.946158634	76.42528215
10 to 14	0.000382513	2.935714616	98836.51559	4.937878968	68.84426762	0.000399166	2.66054323	98824.92998	4.936639773	71.5716665
15 to 19	0.00105804	2.820056545	98648.19041	4.921103669	63.96741799	0.00070539	2.636000646	98628.2727	4.923205889	66.70861304
20 to 24	0.00213875	2.585229465	98128.82232	4.8815359	59.28655128	0.000817307	2.558121765	98283.04466	4.904351059	61.93249881
25 to 29	0.001883001	2.480671768	97103.29051	4.832391865	54.87629652	0.000897711	2.579030745	97883.36893	4.88357242	57.1747503
30 to 34	0.001820537	2.495947893	96203.72987	4.788588492	50.364342	0.001147477	2.601771389	97446.59554	4.858969426	52.41911657
35 to 39	0.001765273	2.532559017	95345.68613	4.746821554	45.79580337	0.001416876	2.599196834	96891.73194	4.828179965	47.70438704
40 to 44	0.002113672	2.600760928	94516.77435	4.702245956	41.17501453	0.001811916	2.641147957	96209.56386	4.790009582	43.02387833
45 to 49	0.002811092	2.69531545	93534.46786	4.646856928	36.57797911	0.002636619	2.681594908	95343.86648	4.738204227	38.38980874
50 to 54	0.0047365	2.744695341	92243.8006	4.563502326	32.04793657	0.00408159	2.670187183	94098.26295	4.660587078	33.8615389
55 to 59	0.008153729	2.66146429	90107.91925	4.421936414	27.73562109	0.006060089	2.621398718	92200.08993	4.544567058	29.50253797
60 to 64	0.01144175	2.578448146	86554.34009	4.212861142	23.75664387	0.007889146	2.610167922	89452.9236	4.390082543	25.32699123
65 to 69	0.013080402	2.63726167	81833.30101	3.970660467	19.97433324	0.010937776	2.621390582	85998.72514	4.191174896	21.23747636
70 to 74	0.020999837	2.652855075	76755.31036	3.659688376	16.12758031	0.015466062	2.68125894	81431.72319	3.931142188	17.27978031
75 to 79	0.033301267	2.626665706	69179.43429	3.209191982	12.61083898	0.027825703	2.671286325	75378.95459	3.54077257	13.44973393
80 to 84	0.054439853	2.569475105	58663.96815	2.596075785	9.413384629	0.047942609	2.598095681	65591.83578	2.943873533	10.05545246
85 to 89	0.10590291	2.507721061	44789.99866	1.779483922	6.547911494	0.094688565	2.561237866	51617.71818	2.101949795	7.071636741
90 to 94	0.199086396	2.232407667	26321.23606	0.853839111	4.429950487	0.176700015	2.313132485	31984.77205	1.089501307	4.861858585
95 plus	0.29982206	3.341028137	9601.990446	0.324327354	3.341028137	0.275266341	3.641604086	12998.33307	0.478375436	3.641604086

Table 15: Algeria 2017 life table, by age and sex. mx=mortality rate, ax=mean person-years lived in an age interval among those who die in that age interval, lx=number of persons left alive at age x, nLx=person-years lived between age x and x+n, ex=life expectancy at age x.

Age Group	Male					Female				
	mx	ax	lx	nLx	ex	mx	ax	lx	nLx	ex
Early Neonatal	0.44729186	0.009575332	100000	0.019096063	77.62155067	0.362391582	0.009577934	100000	0.019111594	79.02679151
Late Neonatal	0.031150352	0.02875853	99145.88598	0.056991757	78.27089252	0.030784461	0.028758631	99307.43585	0.05708522	79.55862466
Post Neonatal	0.004880691	0.46129712	98968.37171	0.911707272	78.35369398	0.004536851	0.461321545	99131.71581	0.913356822	79.64206383
1 to 4	0.000557218	1.999257043	98523.45187	3.936550408	77.78217314	0.00058855	1.999215267	98717.3833	3.944051878	79.0511282
5 to 9	0.00040052	2.499165583	98304.15326	4.910289549	73.95126316	0.000354742	2.499260954	98485.30538	4.919900891	75.23273042
10 to 14	0.000352844	2.645051505	98107.49445	4.901302003	69.09449391	0.000312152	2.524113416	98310.78192	4.911743084	70.36185279
15 to 19	0.000648289	2.684766183	97934.5598	4.889389541	64.21182623	0.000392105	2.573390663	98157.46374	4.903208025	65.46780632
20 to 24	0.000932517	2.56108257	97617.59042	4.869804034	59.41157726	0.000451905	2.565320952	97965.20798	4.89287717	60.59121952
25 to 29	0.000928576	2.523991052	97163.47874	4.847029961	54.67723602	0.000536006	2.620650565	97744.09847	4.880980106	55.72246084
30 to 34	0.001048905	2.569309489	96713.40277	4.823373032	49.91990371	0.000765909	2.648033612	97482.47851	4.8653598	50.86494812
35 to 39	0.001289666	2.601336475	96207.48761	4.795540095	45.16885852	0.001087086	2.650751486	97109.84192	4.843123764	46.04993348
40 to 44	0.001694907	2.643191055	95589.04097	4.760436966	40.44420928	0.001566249	2.657073893	96583.36324	4.81151194	41.28644775
45 to 49	0.002489623	2.679465201	94782.22328	4.711890668	35.76591417	0.002296576	2.655611381	95829.78204	4.765830156	36.59014934
50 to 54	0.003918114	2.680971195	93609.20398	4.638317889	31.18042545	0.003342499	2.658658743	94735.3155	4.699985606	31.98210297
55 to 59	0.006078962	2.699307446	91791.99261	4.526300459	26.74450032	0.004974413	2.682263201	93164.43015	4.605130578	27.47642718
60 to 64	0.010231861	2.669182197	89040.74551	4.348347584	22.48730524	0.008014636	2.673035494	90873.82032	4.460511756	23.10125939
65 to 69	0.015599658	2.645361237	84592.17143	4.079778923	18.5293144	0.012398599	2.671460132	87299.26336	4.242498484	18.93752884
70 to 74	0.024006891	2.655268848	78229.08715	3.703065445	14.82110824	0.02015848	2.712077201	82040.01463	3.921192901	14.98005802
75 to 79	0.041119944	2.628083588	69341.73322	3.159093301	11.38025393	0.039157291	2.680168138	74137.52139	3.398293145	11.28750557
80 to 84	0.070076515	2.57127379	56357.07996	2.408211906	8.396910432	0.075476988	2.577076299	60836.11249	2.571760169	8.169383715
85 to 89	0.127345795	2.442354248	39490.69999	1.489651123	5.885854955	0.133325299	2.432766071	41436.51635	1.543798851	5.788260177
90 to 94	0.222414207	2.199089047	20531.79582	0.632668732	4.067400726	0.220920487	2.227839162	20867.78051	0.647267861	4.097743815
95 plus	0.319139739	3.133543857	6467.062419	0.202713959	3.133543857	0.316074386	3.164038278	6577.377076	0.208211713	3.164038278

Table 15: Algeria 2100 life table, by age and sex. mx=mortality rate, ax=mean person-years lived in an age interval among those who die in that age interval, lx=number of persons left alive at age x, nLx=person-years lived between age x and x+n, ex=life expectancy at age x.

Age Group	Male					Female				
	mx	ax	lx	nLx	ex	mx	ax	lx	nLx	ex
Early Neonatal	0.125856086	0.009585184	100000	0.019154959	85.91185288	0.107142733	0.009585757	100000	0.019158395	86.19564243
Late Neonatal	0.00678808	0.028765251	99758.96889	0.057384367	86.10013583	0.007686252	0.028765003	99794.76985	0.057403478	86.35352341
Post Neonatal	0.000777396	0.461588611	99720.02249	0.920372386	86.07620442	0.00072089	0.461592625	99750.65614	0.92067913	86.33414212
1 to 4	0.000115051	1.999846599	99648.48594	3.98502262	85.214359	0.000119712	1.999840384	99684.29726	3.986417622	85.46796306
5 to 9	8.42035E-05	2.499824576	99602.64938	4.97908441	81.25262611	9.98797E-05	2.499791917	99636.58864	4.980586059	81.50788024
10 to 14	0.000113435	2.850594097	99560.73058	4.976803592	76.28575426	0.000131184	2.633238885	99586.85863	4.977791572	76.54729788
15 to 19	0.000256298	2.725572807	99504.28825	4.972311191	71.32740058	0.000177948	2.549688411	99521.57425	4.973908578	71.59575702
20 to 24	0.000386681	2.579240902	99376.87139	4.964197094	66.41528556	0.000172088	2.543360333	99433.08412	4.969553319	66.6571681
25 to 29	0.000403634	2.551451113	99184.9733	4.954347279	61.53870907	0.00021372	2.649696345	99347.58247	4.964881378	61.712325
30 to 34	0.000481385	2.571069791	98985.08756	4.943460507	56.65774704	0.000321951	2.648955223	99241.49819	4.958315575	56.77541796
35 to 39	0.000554237	2.576212372	98747.22598	4.930734553	51.78800123	0.000438049	2.650603592	99081.90321	4.948995895	51.86252874
40 to 44	0.0006818	2.634444776	98474.0592	4.91576563	46.92445487	0.000633953	2.691837265	98865.15988	4.936031806	46.97030585
45 to 49	0.000987977	2.688119246	98139.03415	4.895766278	42.0755377	0.001022546	2.679597203	98552.29219	4.915951622	42.11068438
50 to 54	0.001580332	2.695137295	97655.48532	4.865059817	37.27039255	0.00152687	2.65254437	98049.70695	4.884974807	37.31241852
55 to 59	0.002500636	2.70225803	96886.88034	4.816658076	32.54429646	0.002160486	2.661784391	97304.05028	4.8407334	32.57737607
60 to 64	0.004065566	2.701593799	95683.00742	4.739908731	27.91901829	0.003236303	2.677592446	96258.64425	4.777058139	27.90140172
65 to 69	0.006679448	2.674791312	93757.17939	4.616157855	23.43564815	0.005068823	2.697471052	94713.29011	4.681062693	23.31180475
70 to 74	0.010046049	2.721221359	90679.35736	4.432562888	19.13786086	0.008348076	2.811603511	92343.18963	4.534400735	18.83834542
75 to 79	0.01873432	2.710328757	86236.19993	4.134842394	14.98182843	0.018843763	2.801965557	88565.49991	4.252478834	14.51771083
80 to 84	0.033542911	2.682629265	78514.87926	3.643989738	11.18619851	0.0415678	2.675809899	80587.32133	3.676970784	10.67059092
85 to 89	0.07488177	2.607987675	66360.32316	2.817437146	7.737035114	0.084997463	2.598651231	65453.64622	2.725330589	7.500319482
90 to 94	0.162242948	2.258535249	45448.77846	1.577230931	5.103961375	0.164483069	2.328953847	42695.05926	1.49240994	5.127138123
95 plus	0.268683477	3.727517908	20089.38336	0.753600437	3.727517908	0.263672193	3.808936464	18630.47022	0.720548084	3.808936464

Table 15: Bahrain 2017 life table, by age and sex. mx=mortality rate, ax=mean person-years lived in an age interval among those who die in that age interval, lx=number of persons left alive at age x, nLx=person-years lived between age x and x+n, ex=life expectancy at age x.

Age Group	Male					Female				
	mx	ax	lx	nLx	ex	mx	ax	lx	nLx	ex
Early Neonatal	0.107292532	0.009585753	100000	0.019158365	79.0189861	0.082090099	0.009586525	100000	0.019162994	80.48653293
Late Neonatal	0.017755363	0.028762225	99794.44705	0.057386667	79.16253939	0.015726195	0.028762785	99842.69153	0.057417761	80.59414851
Post Neonatal	0.002691334	0.461452648	99692.55741	0.919306479	79.18588071	0.00290358	0.46143757	99752.39633	0.919768174	80.60954089
1 to 4	0.000317684	1.999576422	99445.15113	3.975279886	78.45845019	0.000352307	1.999530257	99485.34002	3.97661105	79.9014042
5 to 9	0.000234507	2.499511445	99318.87015	4.963033392	74.5556698	0.000194381	2.499595039	99345.24559	4.964849248	76.01126405
10 to 14	0.000203254	2.606382894	99202.48835	4.957712357	69.64020788	0.000140737	2.586016499	99248.74001	4.960751865	71.08274594
15 to 19	0.000338859	2.664469347	99101.72335	4.951168081	64.70835632	0.000253084	2.627520217	99178.92446	4.955970718	66.13095357
20 to 24	0.000472282	2.607238697	98933.95054	4.941113758	59.81352321	0.000296424	2.522355995	99053.49722	4.949040296	61.21133447
25 to 29	0.000584119	2.559166624	98700.59351	4.92800376	54.94871153	0.000285798	2.53561659	98906.79729	4.941859365	56.29834379
30 to 34	0.000641383	2.541557933	98412.74539	4.912891088	50.10187146	0.000346334	2.596869196	98765.56198	4.934171765	51.37518528
35 to 39	0.000716516	2.600096509	98097.65017	4.896463466	45.25455632	0.00044838	2.651830884	98594.67819	4.924549131	46.45967978
40 to 44	0.000992322	2.68724325	97746.82633	4.876151497	40.4075498	0.00067563	2.706509062	98373.87782	4.911083765	41.55793771
45 to 49	0.001621921	2.729656113	97262.98566	4.845308649	35.59505506	0.001123684	2.833344864	98042.08327	4.890198283	36.68932453
50 to 54	0.002816661	2.734015977	96477.18729	4.793268897	30.86252409	0.002497578	2.779820988	97492.60886	4.847750712	31.87999633
55 to 59	0.004891528	2.801914316	95127.27035	4.705772711	26.26134539	0.004565409	2.74918505	96281.94396	4.765136041	27.24561955
60 to 64	0.010329726	2.725810498	92825.8931	4.534791896	21.84246308	0.008254003	2.730005151	94106.76177	4.618814971	22.81136447
65 to 69	0.017418593	2.676481958	88143.03651	4.235798277	17.85728696	0.014613037	2.718359559	90295.26579	4.369142246	18.65832758
70 to 74	0.028930011	2.631644942	80768.70462	3.779670934	14.24240354	0.026610304	2.640814993	83913.22826	3.947981688	14.86967683
75 to 79	0.045381881	2.591849559	69843.56884	3.148528031	11.05793247	0.040281918	2.604953485	73415.38274	3.348125759	11.61730691
80 to 84	0.073175084	2.537672235	55574.47022	2.355141192	8.232183879	0.067190518	2.552349355	59945.46705	2.574592697	8.64238656
85 to 89	0.131096426	2.431574369	38372.34848	1.436060733	5.787851595	0.12205838	2.467096422	42677.45486	1.630768561	6.108588547
90 to 94	0.226263475	2.192190379	19581.94973	0.599122678	4.014618701	0.208595335	2.254504118	22812.62256	0.725813595	4.285234035
95 plus	0.322269651	3.103365749	6046.872757	0.187853312	3.103365749	0.304891096	3.2805686	7700.456524	0.252972248	3.2805686

Table 15: Bahrain 2100 life table, by age and sex. mx=mortality rate, ax=mean person-years lived in an age interval among those who die in that age interval, lx=number of persons left alive at age x, nLx=person-years lived between age x and x+n, ex=life expectancy at age x.

Age Group	Male					Female				
	mx	ax	lx	nLx	ex	mx	ax	lx	nLx	ex
Early Neonatal	0.018118222	0.009588486	100000	0.019174751	84.63206023	0.012595834	0.009588655	100000	0.019175766	86.38962191
Late Neonatal	0.003528225	0.02876615	99965.26211	0.057508424	84.64227733	0.002735837	0.028766369	99975.84747	0.057515824	86.39129936
Post Neonatal	0.000582623	0.461602447	99944.97289	0.922531484	84.601911	0.000598142	0.461601345	99960.11273	0.922664616	86.34735424
1 to 4	8.27472E-05	1.99988967	99891.23027	3.99498805	83.72390198	0.000107215	1.999857047	99904.9288	3.995340421	85.47147194
5 to 9	6.92248E-05	2.499855781	99858.1742	4.99204475	79.75095137	7.45318E-05	2.499844726	99862.09564	4.992174666	81.5072755
10 to 14	6.99833E-05	2.723357707	99823.61854	4.990360086	74.77769242	6.19617E-05	2.688692437	99824.89458	4.990523102	76.53669791
15 to 19	0.000120225	2.720864621	99788.69782	4.988051308	69.80292541	0.000132039	2.715132859	99793.97786	4.988121794	71.55955715
20 to 24	0.00018206	2.646068521	99728.73224	4.984293744	64.84326551	0.000130501	2.447346607	99728.1494	4.984761209	66.60500176
25 to 29	0.000241596	2.570834309	99637.99704	4.97898697	59.89984093	0.00010524	2.524539268	99663.11435	4.981849558	61.64667922
30 to 34	0.000273717	2.525998768	99517.73599	4.972515177	54.96895304	0.00013491	2.608799697	99610.70219	4.978928051	56.677628
35 to 39	0.000270767	2.590408738	99381.69683	4.965829367	50.04054751	0.000177774	2.641863753	99543.54739	4.975083712	51.71387867
40 to 44	0.000375316	2.677037077	99247.32606	4.958011868	45.10433706	0.000251081	2.79369786	99455.15738	4.969940894	46.75683863
45 to 49	0.000559552	2.737848033	99061.35514	4.94677092	40.18314952	0.000474098	2.828530775	99330.47204	4.961346581	41.81123868
50 to 54	0.000983187	2.798905031	98784.7111	4.928549143	35.2869042	0.00094407	2.794676905	99095.46029	4.944364943	36.90244514
55 to 59	0.001965448	2.854441219	98300.47648	4.894260607	30.44512845	0.001723692	2.737046525	98629.31359	4.912275516	32.06131564
60 to 64	0.004266945	2.79613461	97339.86635	4.821764132	25.71380589	0.002932395	2.766713823	97784.00681	4.857514863	27.31166166
65 to 69	0.008392542	2.771668883	95287.09677	4.67693946	21.20026954	0.005743429	2.844034164	96363.98599	4.758658679	22.66783564
70 to 74	0.015829236	2.745925828	91390.32156	4.412921468	16.97169155	0.01211839	2.767625571	93653.33953	4.559424526	18.23233618
75 to 79	0.029109368	2.703219899	84517.58665	3.965094623	13.11121224	0.022313924	2.741931714	88193.64321	4.19956101	14.17835454
80 to 84	0.0525528	2.638429127	73279.84881	3.270352857	9.699742846	0.044418869	2.635584622	78969.52121	3.580419992	10.51033903
85 to 89	0.102449618	2.520753594	56681.27326	2.282426813	6.741672899	0.088739241	2.587184447	63441.43494	2.629663289	7.401571485
90 to 94	0.194177847	2.230413528	34455.55013	1.139427219	4.543376013	0.168504718	2.318858197	41020.32178	1.432362103	5.071771837
95 plus	0.295506442	3.406162407	13346.08846	0.469257008	3.406162407	0.267319447	3.774756389	17904.6006	0.697950796	3.774756389

Table 15: Egypt 2017 life table, by age and sex. mx=mortality rate, ax=mean person-years lived in an age interval among those who die in that age interval, lx=number of persons left alive at age x, nLx=person-years lived between age x and x+n, ex=life expectancy at age x.

Age Group	Male					Female				
	mx	ax	lx	nLx	ex	mx	ax	lx	nLx	ex
Early Neonatal	0.219516622	0.009582313	100000	0.019137772	67.97206063	0.158190194	0.009584193	100000	0.019149021	74.30395164
Late Neonatal	0.075182669	0.028746384	99579.92093	0.057168845	68.23950017	0.061781548	0.028750081	99697.09155	0.057258156	74.51046966
Post Neonatal	0.009093886	0.460997822	99150.1924	0.911609591	68.47758912	0.008899401	0.461011638	99343.38039	0.913467469	74.71812937
1 to 4	0.001037964	1.998616048	98321.32436	3.924702006	68.12765622	0.000898085	1.998802554	98530.53482	3.934151705	74.40744962
5 to 9	0.000489839	2.498979502	97914.07123	4.88971458	64.40275762	0.00038123	2.49920577	98177.27097	4.904188232	70.66802276
10 to 14	0.000542625	2.680644771	97674.61045	4.87759135	59.5545526	0.000310004	2.672858784	97990.31805	4.895983582	65.79800455
15 to 19	0.000963593	2.647749703	97409.97912	4.859486068	54.70903054	0.000640093	2.648045681	97838.54989	4.884575743	60.8958918
20 to 24	0.00123592	2.577795917	96941.74529	4.832620676	49.96029519	0.00077012	2.569467458	97525.89277	4.867187616	56.08246515
25 to 29	0.001440095	2.586736256	96344.49435	4.800542074	45.25376376	0.000905898	2.594200435	97151.08	4.846992037	51.28867849
30 to 34	0.001858689	2.638586534	95653.21149	4.761763543	40.56183345	0.001189602	2.643883331	96712.04037	4.822085846	46.50940268
35 to 39	0.002719894	2.655712645	94768.23525	4.708396193	35.91562171	0.001742039	2.675981906	96138.49419	4.78754184	41.77072313
40 to 44	0.003979243	2.683301239	93487.81253	4.631703309	31.37063178	0.00269464	2.672849936	95304.66338	4.735541478	37.11231217
45 to 49	0.006428222	2.697112079	91645.23674	4.515440563	26.94668941	0.004061034	2.661724478	94028.92463	4.657231365	32.57901451
50 to 54	0.010595975	2.666986938	88743.81688	4.33020139	22.73852617	0.006035977	2.656655263	92138.24053	4.54268264	28.19218699
55 to 59	0.016220809	2.66957046	84158.41446	4.054759822	18.83092466	0.009030127	2.655671064	89397.42849	4.377253963	23.97425026
60 to 64	0.027260215	2.636235995	77587.10342	3.644755353	15.19857662	0.013754043	2.654280919	85447.10551	4.138915586	19.95876513
65 to 69	0.042572374	2.604571332	67663.20113	3.07052783	12.04018243	0.021517046	2.649140188	79758.79913	3.796130899	16.19172293
70 to 74	0.070998843	2.531514464	54612.97518	2.324248352	9.294981847	0.034929722	2.628156108	71599.8508	3.306554644	12.73354325
75 to 79	0.103227635	2.44128899	38149.83009	1.51000671	7.216364048	0.057980568	2.585513732	60072.51204	2.635699625	9.671495423
80 to 84	0.14982925	2.354922755	22614.05838	0.810753803	5.504947692	0.099289173	2.501557218	44835.68871	1.797942886	7.080628378
85 to 89	0.215866651	2.220805398	10514.35416	0.329084867	4.145515548	0.162620262	2.353531691	27070.939	0.948011151	5.094320369
90 to 94	0.306444802	2.027291888	3435.929188	0.090043429	3.131695101	0.251553498	2.157859216	11740.37172	0.343146822	3.694824858
95 plus	0.385166758	2.596579402	684.3118304	0.017802505	2.596579402	0.343227676	2.914917783	3152.752591	0.092259329	2.914917783

Table 15: Egypt 2100 life table, by age and sex. mx=mortality rate, ax=mean person-years lived in an age interval among those who die in that age interval, lx=number of persons left alive at age x, nLx=person-years lived between age x and x+n, ex=life expectancy at age x.

Age Group	Male					Female				
	mx	ax	lx	nLx	ex	mx	ax	lx	nLx	ex
Early Neonatal	0.028730055	0.009588161	100000	0.019172801	80.0026378	0.022629748	0.009588347	100000	0.019173922	85.77761509
Late Neonatal	0.010923825	0.02876411	99944.94297	0.057484509	80.02722923	0.009842742	0.028764408	99956.62505	0.057493015	85.79536799
Post Neonatal	0.000945125	0.461576695	99882.17057	0.921797658	80.01968576	0.000940377	0.461577033	99900.05389	0.921964697	85.78607499
1 to 4	0.000103062	1.999862584	99795.08419	3.990981205	79.16555626	7.72211E-05	1.999897038	99813.38391	3.991919161	84.93661194
5 to 9	6.80683E-05	2.499858191	99753.98019	4.98685068	75.19722595	7.83697E-05	2.49983673	99782.57643	4.988151808	80.96212575
10 to 14	0.000136895	2.961817927	99720.04932	4.984592977	70.22187482	8.66043E-05	2.786324056	99743.49881	4.986221882	75.99273886
15 to 19	0.000353553	2.716344094	99651.83509	4.978541709	65.26783155	0.000200038	2.668826452	99700.33231	4.982673473	71.02430084
20 to 24	0.000474327	2.591999153	99475.88797	4.968116102	60.37812394	0.000228622	2.564364815	99600.7246	4.977265906	66.09229377
25 to 29	0.000559486	2.579256949	99240.3301	4.955310463	55.51485668	0.000271487	2.623421014	99486.97386	4.971143729	61.16464561
30 to 34	0.000691699	2.621646341	98963.27099	4.940004592	50.66239912	0.000390646	2.639676838	99352.11254	4.96302195	56.24350191
35 to 39	0.000930084	2.643914212	98621.90421	4.920289644	45.82780733	0.00052394	2.728074651	99158.40852	4.951979503	51.34733511
40 to 44	0.00131064	2.692708392	98164.74349	4.893406237	41.02751327	0.000916676	2.732177266	98899.26384	4.934653842	46.4732978
45 to 49	0.002116425	2.740544002	97524.12005	4.853040865	36.27740995	0.001495457	2.65747778	98447.46682	4.905189139	41.67193189
50 to 54	0.003838011	2.708729024	96498.32798	4.78291842	31.63048348	0.002041401	2.692636883	97715.24589	4.862837408	36.96062583
55 to 59	0.006106592	2.704651215	94667.46548	4.667981028	27.18248249	0.003364905	2.645647171	96725.2146	4.798271443	32.30675134
60 to 64	0.010070854	2.663189388	91830.35328	4.486370637	22.92878124	0.00439571	2.639785393	95116.56376	4.707207045	27.80263791
65 to 69	0.014876907	2.664685676	87342.42239	4.221553295	18.9587266	0.006548307	2.669214096	93057.58036	4.58329148	23.35267427
70 to 74	0.024550378	2.651092914	81120.71226	3.837806238	15.19664492	0.010095726	2.731228937	90079.27738	4.40397654	19.02821815
75 to 79	0.039950333	2.618323223	71844.81558	3.28696445	11.80169613	0.0191772	2.756997936	85682.67642	4.109523542	14.85268562
80 to 84	0.065461991	2.553192024	59062.44508	2.558132658	8.788990414	0.03878114	2.666022578	77939.07994	3.580953074	11.04146917
85 to 89	0.120179962	2.465983556	42930.74024	1.662950975	6.150471277	0.080155122	2.619751252	64432.25528	2.721537153	7.778309666
90 to 94	0.214064766	2.208043047	23821.59056	0.756976746	4.213923651	0.157725542	2.332213537	43473.09341	1.549174784	5.306323968
95 plus	0.312079091	3.217419923	8223.866835	0.271393905	3.217419923	0.257118437	3.922762322	20034.88622	0.809990594	3.922762322

Table 15: Iran 2017 life table, by age and sex. mx=mortality rate, ax=mean person-years lived in an age interval among those who die in that age interval, lx=number of persons left alive at age x, nLx=person-years lived between age x and x+n, ex=life expectancy at age x.

Age Group	Male					Female				
	mx	ax	lx	nLx	ex	mx	ax	lx	nLx	ex
Early Neonatal	0.41229038	0.009576404	100000	0.019102462	75.3692829	0.313902711	0.00957942	100000	0.019120471	79.26680057
Late Neonatal	0.042406886	0.028755425	99212.42471	0.057011543	75.94832911	0.039318656	0.028756277	99399.80361	0.057124291	79.72619316
Post Neonatal	0.005335385	0.461264819	98970.65709	0.91153688	76.07625282	0.004651041	0.461313433	99175.19892	0.913709137	79.84915182
1 to 4	0.000615868	1.999178843	98484.31871	3.934524484	75.52637022	0.000531184	1.999291755	98750.22998	3.945815815	79.26750812
5 to 9	0.000430238	2.499103671	98242.00503	4.906820615	71.70772538	0.000330351	2.499311769	98540.63505	4.922964873	75.43185696
10 to 14	0.000445296	2.832399518	98030.89527	4.896818222	66.85676649	0.000332223	2.592476884	98378.00465	4.914969074	70.55242312
15 to 19	0.001146061	2.679482918	97812.84188	4.877670159	61.99949552	0.000479324	2.575092385	98214.71835	4.905034723	65.66540943
20 to 24	0.001443952	2.527400115	97253.83132	4.845391986	57.34046359	0.000506924	2.53803238	97979.60848	4.892873971	60.81679934
25 to 29	0.001353101	2.491988005	96554.17996	4.811381128	52.73764946	0.000574606	2.547988247	97731.57697	4.879703645	55.96470378
30 to 34	0.001411737	2.547347632	95903.15163	4.778611649	48.07873611	0.000642594	2.564698288	97451.18658	4.864946127	51.11839603
35 to 39	0.001696215	2.58694282	95228.53772	4.742017515	43.40128689	0.000778636	2.608647609	97138.56813	4.847901652	46.27465462
40 to 44	0.002150821	2.626798337	94424.18986	4.697233274	38.74896096	0.001055656	2.659879617	96761.09338	4.826132372	41.44499941
45 to 49	0.003060168	2.68226108	93413.89979	4.637800627	34.13962824	0.001602671	2.690756081	96251.61991	4.794835516	36.65029366
50 to 54	0.004951676	2.693712298	91994.65619	4.547797067	29.62493382	0.002555776	2.717407496	95483.16603	4.746468392	31.9236007
55 to 59	0.007974783	2.670174304	89742.73718	4.405287368	25.30071809	0.004358674	2.735278167	94270.07591	4.667430761	27.29943129
60 to 64	0.012220515	2.646641034	86229.62172	4.190952629	21.22271535	0.007747319	2.708953079	92235.69703	4.531355806	22.84122314
65 to 69	0.01831049	2.640102069	81108.07277	3.887425045	17.39569402	0.012894177	2.699702267	88725.11713	4.308465191	18.6377912
70 to 74	0.028587566	2.643179177	73990.02928	3.465979438	13.81521979	0.022337828	2.703320223	83169.72132	3.955555696	14.7023849
75 to 79	0.048507806	2.610127078	64081.6835	2.871231639	10.54264586	0.041897021	2.654705837	74333.91133	3.384166884	11.12866556
80 to 84	0.082935617	2.537557266	50154.06329	2.082425246	7.745484796	0.076263809	2.580147459	60155.37344	2.539176225	8.125964257
85 to 89	0.143393762	2.396883739	32883.4922	1.197272333	5.480730037	0.134397003	2.429561239	40790.87642	1.515877532	5.758722793
90 to 94	0.238705523	2.169039316	15715.50779	0.468906749	3.84962744	0.22208307	2.225284965	20418.21808	0.631670387	4.080498333
95 plus	0.332333098	3.00903075	4522.525314	0.136084845	3.00903075	0.317125412	3.153331169	6390.069128	0.201502031	3.153331169

Table 15: Iran 2100 life table, by age and sex. mx=mortality rate, ax=mean person-years lived in an age interval among those who die in that age interval, lx=number of persons left alive at age x, nLx=person-years lived between age x and x+n, ex=life expectancy at age x.

Age Group	Male					Female				
	mx	ax	lx	nLx	ex	mx	ax	lx	nLx	ex
Early Neonatal	0.105326711	0.009585813	100000	0.019158731	82.71513378	0.08605905	0.009586403	100000	0.019162269	85.6696844
Late Neonatal	0.010694165	0.028764173	99798.28582	0.057400535	82.86282039	0.011050669	0.028764075	99835.1563	0.057421155	85.79147642
Post Neonatal	0.00097258	0.461574745	99736.91707	0.920445404	82.8561734	0.000819617	0.461585611	99771.72306	0.92083163	85.78834651
1 to 4	0.00015902	1.999787973	99647.41424	3.984629578	82.00676833	0.000147341	1.999803545	99696.26725	3.986676273	84.92950169
5 to 9	0.000136532	2.499715559	99584.07596	4.977506036	78.05732684	0.000135565	2.499717574	99637.5577	4.9801914	80.9781229
10 to 14	0.000203376	3.145636484	99516.18204	4.973761555	73.10850649	0.000181666	2.694377186	99570.11323	4.97638091	76.03101142
15 to 19	0.000599455	2.728120105	99415.1617	4.963967242	68.17937348	0.000264065	2.58453081	99479.814	4.970812156	71.09737939
20 to 24	0.000830529	2.569212694	99117.84625	4.945908362	63.3752892	0.000279231	2.528786777	99348.65937	4.964019942	66.18766202
25 to 29	0.000871749	2.534961322	98707.56038	4.92479523	58.62764997	0.000311827	2.571348731	99210.20561	4.956734119	61.27621833
30 to 34	0.00096997	2.542650831	98278.89767	4.902259399	53.87203446	0.000360229	2.561427893	99055.85846	4.948437222	56.36738867
35 to 39	0.001061634	2.55971639	97804.1435	4.877575834	49.1210899	0.000406055	2.591170681	98877.79664	4.93905773	51.46410992
40 to 44	0.001272436	2.5909458	97287.12219	4.849501274	44.36846479	0.000534027	2.673505729	98677.44419	4.927743892	46.56318864
45 to 49	0.001625152	2.654572821	96670.93907	4.815208902	39.63463177	0.000842285	2.691544001	98414.56188	4.911177723	41.68022723
50 to 54	0.002505909	2.686239333	95889.48911	4.766847649	34.93565339	0.001308727	2.707662562	98001.21416	4.88540445	36.84439595
55 to 59	0.003924184	2.665269778	94696.52032	4.691859658	30.3413801	0.002158442	2.713433375	97362.33737	4.844216517	32.06791421
60 to 64	0.005777329	2.656491864	92857.46735	4.58091242	25.88854202	0.003572798	2.694950637	96317.47185	4.77655687	27.38565117
65 to 69	0.008654633	2.671378231	90214.49137	4.421730024	21.56723353	0.005645343	2.716857357	94612.37782	4.670481205	22.82912543
70 to 74	0.013840091	2.712873794	86394.05978	4.187403797	17.3998812	0.009898685	2.787060062	91978.41088	4.500458428	18.40283447
75 to 79	0.025548386	2.701834762	80614.82917	3.808053432	13.44845779	0.020947583	2.767114486	87531.80306	4.181697989	14.19117876
80 to 84	0.0481315	2.632709405	70928.07575	3.187525849	9.909378997	0.04441569	2.695472883	78800.35791	3.577759606	10.44658003
85 to 89	0.096954224	2.536190784	55768.81365	2.2589244	6.870850072	0.089246456	2.583558974	63124.64677	2.606935945	7.341947844
90 to 94	0.18872755	2.242008519	34296.51482	1.135638265	4.611179454	0.169531313	2.320233606	40440.43222	1.403253706	5.031706069
95 plus	0.291115053	3.444896092	13282.15134	0.464120207	3.444896092	0.26838857	3.749071533	17324.40641	0.664273265	3.749071533

Table 15: Iraq 2017 life table, by age and sex. mx=mortality rate, ax=mean person-years lived in an age interval among those who die in that age interval, lx=number of persons left alive at age x, nLx=person-years lived between age x and x+n, ex=life expectancy at age x.

Age Group	Male					Female				
	mx	ax	lx	nLx	ex	mx	ax	lx	nLx	ex
Early Neonatal	0.52241914	0.009573029	100000	0.019082334	74.32145307	0.414727308	0.00957633	100000	0.019102018	79.24026266
Late Neonatal	0.054522802	0.028752083	99003.17427	0.056871497	75.05036625	0.053734562	0.028752301	99207.82224	0.056990341	79.85364514
Post Neonatal	0.006985887	0.46114757	98693.14503	0.908289865	75.22850528	0.007709937	0.461096135	98901.61894	0.909904478	80.04324374
1 to 4	0.001445852	1.998072198	98058.76251	3.911032028	74.78898701	0.001468075	1.998042567	98200.1909	3.916498096	79.68843636
5 to 9	0.000945573	2.498030058	97493.38659	4.863165382	71.21118211	0.000892306	2.49814103	97625.27799	4.870391875	76.14599158
10 to 14	0.000850096	2.695942653	97033.5917	4.842194973	66.53681904	0.000700048	2.497245795	97190.72053	4.851036883	71.4753059
15 to 19	0.001759025	2.780886873	96621.98251	4.81231445	61.80878172	0.000889887	2.553199843	96851.13529	4.832035867	66.71715153
20 to 24	0.003268389	2.578399907	95775.49101	4.751170498	57.33044665	0.000934458	2.499126275	96421.14396	4.809817224	62.00326235
25 to 29	0.003060841	2.467133778	94222.6281	4.674888896	53.23273693	0.000894363	2.520157654	95971.69355	4.787966018	57.28189234
30 to 34	0.002881621	2.472717333	92791.72688	4.606042883	49.01548934	0.001030394	2.577089344	95543.48479	4.765278168	52.52727932
35 to 39	0.002768518	2.481930936	91464.4539	4.541563358	44.69079337	0.001287744	2.561633564	95052.4856	4.737748549	47.78524416
40 to 44	0.002719721	2.565513608	90207.13814	4.48069186	40.27898591	0.001427244	2.627296945	94442.40111	4.706183494	43.07729214
45 to 49	0.003720936	2.635428864	88988.55767	4.410624924	35.79528653	0.002187791	2.661751623	93770.73908	4.664675511	38.36693352
50 to 54	0.00530089	2.607980973	87347.49874	4.312696372	31.41807202	0.003180773	2.626024209	92750.25959	4.602759649	33.75964074
55 to 59	0.006747643	2.71130793	85061.63161	4.188407252	27.192005	0.004216217	2.728532647	91286.33639	4.521023263	29.25873648
60 to 64	0.012912703	2.639014323	82235.87817	3.990172179	23.03290766	0.008103838	2.690515579	89380.37692	4.386927098	24.82425169
65 to 69	0.01691015	2.60756437	77084.64745	3.704412877	19.39533404	0.012369136	2.642548585	85825.84733	4.169734765	20.74057276
70 to 74	0.025119709	2.587655227	70822.55549	3.338896689	15.87939629	0.018320712	2.653599825	80669.57211	3.867297099	16.896947
75 to 79	0.033946721	2.579735931	62439.57916	2.885101665	12.66382658	0.030237678	2.630487709	73587.38851	3.433510424	13.26737305
80 to 84	0.051923522	2.563019538	52652.69222	2.337157224	9.538625578	0.047851348	2.632858239	63212.03337	2.83926592	10.01319362
85 to 89	0.102700748	2.516883871	40528.17337	1.61494905	6.626563806	0.094940042	2.558896219	49637.90853	2.015311242	7.032116857
90 to 94	0.195870521	2.238998943	23957.23133	0.777632894	4.471474796	0.177318011	2.31422784	30523.86525	1.034174908	4.835380954
95 plus	0.297218483	3.364736128	8736.414107	0.294093921	3.364736128	0.275928901	3.624602285	12203.28381	0.442629772	3.624602285

Table 15: Iraq 2100 life table, by age and sex. mx=mortality rate, ax=mean person-years lived in an age interval among those who die in that age interval, lx=number of persons left alive at age x, nLx=person-years lived between age x and x+n, ex=life expectancy at age x.

Age Group	Male					Female				
	mx	ax	lx	nLx	ex	mx	ax	lx	nLx	ex
Early Neonatal	0.096700858	0.009586077	100000	0.019160313	86.52671998	0.097218307	0.009586061	100000	0.019160218	90.19175097
Late Neonatal	0.00889468	0.02876467	99814.76245	0.057412981	86.66783021	0.012434174	0.028763693	99813.77222	0.057406568	90.34059707
Post Neonatal	0.001024857	0.461571031	99763.69922	0.920670324	86.65451385	0.001673703	0.461524939	99742.40139	0.920198279	90.34758454
1 to 4	0.000491218	1.999345043	99669.35548	3.982862833	85.81246776	0.000573234	1.999235689	99588.43083	3.97897763	89.56279424
5 to 9	0.000332541	2.499307206	99473.89894	4.969567652	81.97418871	0.000372364	2.499224242	99360.59104	4.963414611	85.76212206
10 to 14	0.00035176	2.835759555	99308.87523	4.961702606	77.10447823	0.000338331	2.534115927	99176.07813	4.954688375	80.9163068
15 to 19	0.000941682	2.836127994	99134.66554	4.946607169	72.23336381	0.00043967	2.537379023	99008.7705	4.945097667	76.04830706
20 to 24	0.001814016	2.600512419	98670.62108	4.912128272	67.55508468	0.000416228	2.468048416	98792.02095	4.934412211	71.20915961
25 to 29	0.00166895	2.458520907	97786.12293	4.868793998	63.13690064	0.000371928	2.507027318	98587.33041	4.92480562	66.35154998
30 to 34	0.001413177	2.444233824	96983.13439	4.831872375	58.63723924	0.000417082	2.555912146	98404.93621	4.91522612	61.46957489
35 to 39	0.001250913	2.451685374	96309.30954	4.800366576	54.03049937	0.000455985	2.53733112	98200.74763	4.904535094	56.59179595
40 to 44	0.001105941	2.530017315	95718.12625	4.772944635	49.35032687	0.000483564	2.625110036	97978.02178	4.893265661	51.71428234
45 to 49	0.001325216	2.598341962	95197.09387	4.744831348	44.60735726	0.00071384	2.672309118	97742.22092	4.87898059	46.83223342
50 to 54	0.001664674	2.604025051	94574.89751	4.71005251	39.88369549	0.001034928	2.628621647	97394.89005	4.857821133	41.98880846
55 to 59	0.002112857	2.720789525	93797.59985	4.667420407	35.19197612	0.001325245	2.733847866	96893.73758	4.830130829	37.1911385
60 to 64	0.003827565	2.662110736	92818.3637	4.599977939	30.5334933	0.002454032	2.677512852	96255.79447	4.785585719	32.41796633
65 to 69	0.005199316	2.654969796	91069.59246	4.498853359	26.06579431	0.00344489	2.663580043	95086.22857	4.71627313	27.77982764
70 to 74	0.007766128	2.632840194	88751.49793	4.358066991	21.67505071	0.004991655	2.736611393	93471.41668	4.621229778	23.20990761
75 to 79	0.010609566	2.696403945	85400.95246	4.168890058	17.42032663	0.008943383	2.765350279	91182.4916	4.469620134	18.71852978
80 to 84	0.018872363	2.679703929	81033.79961	3.883569626	13.213824	0.016499115	2.821003577	87224.28574	4.209886573	14.43740669
85 to 89	0.048604822	2.699409444	73814.14799	3.325437515	9.23526947	0.041080531	2.793253963	80399.02452	3.693112512	10.38891332
90 to 94	0.12451036	2.212651364	57973.53824	2.163220777	6.026446931	0.100221104	2.315170562	65709.06459	2.612069074	7.066154606
95 plus	0.235837725	4.257654923	31590.20071	1.362309715	4.257654923	0.200867935	5.047782286	40603.26927	2.105853443	5.047782286

Table 15: Jordan 2017 life table, by age and sex. mx=mortality rate, ax=mean person-years lived in an age interval among those who die in that age interval, lx=number of persons left alive at age x, nLx=person-years lived between age x and x+n, ex=life expectancy at age x.

Age Group	Male					Female				
	mx	ax	lx	nLx	ex	mx	ax	lx	nLx	ex
Early Neonatal	0.445918004	0.009575374	100000	0.019096317	77.46250927	0.396701345	0.009576882	100000	0.019105317	80.55407878
Late Neonatal	0.057746522	0.028751194	99148.54168	0.056949729	78.10846305	0.04001579	0.028756085	99242.13364	0.057032545	81.14987323
Post Neonatal	0.005105278	0.461281165	98819.75123	0.910244247	78.31077792	0.004937939	0.461293053	99013.944	0.912103115	81.27928786
1 to 4	0.000553837	1.99926155	98355.19149	3.929854638	77.75535125	0.000742285	1.999010287	98563.63596	3.936699544	80.7252625
5 to 9	0.000287747	2.499400527	98137.62231	4.903353974	73.92339312	0.000241549	2.499496772	98271.48776	4.910608765	76.95934667
10 to 14	0.000257919	2.672919524	97996.57082	4.896886501	69.02624657	0.000185519	2.476210723	98152.88679	4.905348031	72.04932802
15 to 19	0.000499382	2.692280575	97870.30373	4.887882715	64.11188945	0.000221101	2.552040545	98061.88907	4.900442567	67.11387276
20 to 24	0.00072165	2.57564552	97626.2264	4.872786616	59.26535485	0.000241576	2.562984035	97953.54323	4.894795772	62.18524599
25 to 29	0.000765395	2.54361612	97274.61743	4.85460444	54.47018804	0.000294973	2.606699128	97835.30099	4.888314343	57.2572591
30 to 34	0.000887941	2.572327005	96903.09022	4.834734049	49.66920887	0.000393734	2.647067468	97691.11504	4.880035359	52.33786136
35 to 39	0.001082325	2.611690208	96473.84195	4.811257673	44.87867453	0.000575187	2.65981301	97498.98073	4.868396213	47.43570517
40 to 44	0.001482947	2.669382646	95953.15706	4.781137195	40.10786554	0.000838736	2.690993376	97218.97168	4.851552661	42.56454122
45 to 49	0.002318393	2.700238623	95244.21399	4.736958777	35.38624745	0.001352508	2.71970604	96812.08394	4.825721558	37.73195766
50 to 54	0.003782953	2.703239431	94146.12833	4.666768088	30.76692462	0.002288953	2.716311149	96159.46509	4.782973887	32.9693398
55 to 59	0.006194545	2.710966001	92381.08005	4.554491713	26.30226854	0.003795885	2.759759562	95064.81768	4.713167666	28.31733029
60 to 64	0.010571922	2.687028116	89560.81256	4.371210189	22.04385922	0.007245267	2.750416368	93276.12645	4.589032065	23.80688813
65 to 69	0.017069434	2.641077341	84942.45343	4.082886419	18.09460198	0.013262606	2.66450103	89952.32901	4.362559009	19.58406779
70 to 74	0.025398421	2.647126631	77980.48697	3.679505975	14.47232451	0.019614555	2.677411263	84169.71192	4.02526932	15.74522958
75 to 79	0.043637108	2.624278804	68651.93171	3.110956625	11.07753999	0.035526356	2.657286435	76282.08119	3.521443223	12.09530267
80 to 84	0.074806931	2.559551261	55116.12146	2.331833712	8.153837541	0.062438157	2.611321344	63791.7078	2.776418975	8.942795097
85 to 89	0.133281777	2.425457111	37745.02431	1.406799237	5.734280072	0.11585453	2.486958655	46496.79805	1.801757128	6.299968815
90 to 94	0.228461945	2.188040938	19078.12524	0.58170609	3.985867026	0.201651215	2.268864831	25679.80879	0.82881822	4.397999146
95 plus	0.32404396	3.086926304	5836.362678	0.180604351	3.086926304	0.298529425	3.350844189	9009.452256	0.302483496	3.350844189

Table 15: Jordan 2100 life table, by age and sex. mx=mortality rate, ax=mean person-years lived in an age interval among those who die in that age interval, lx=number of persons left alive at age x, nLx=person-years lived between age x and x+n, ex=life expectancy at age x.

Age Group	Male					Female				
	mx	ax	lx	nLx	ex	mx	ax	lx	nLx	ex
Early Neonatal	0.099736327	0.009585984	100000	0.019159756	84.69623816	0.099964047	0.009585977	100000	0.019159714	87.99605301
Late Neonatal	0.013170244	0.02876349	99808.95604	0.057402586	84.83886577	0.010123245	0.028764331	99808.52598	0.057407367	88.14537421
Post Neonatal	0.000861168	0.46158266	99733.38021	0.92046009	84.84550359	0.000872311	0.461581868	99750.42411	0.920612662	88.13908974
1 to 4	9.99903E-05	1.99986668	99654.12929	3.985368431	83.98929117	0.000138583	1.999815223	99670.13576	3.985701032	87.28634468
5 to 9	5.06123E-05	2.499894558	99614.29585	4.98008478	80.02203504	5.56395E-05	2.499884085	99614.92214	4.980053843	83.33352857
10 to 14	6.6803E-05	3.099600055	99589.09701	4.978778124	75.04161349	6.70796E-05	2.640401794	99587.23381	4.978557352	78.35590166
15 to 19	0.000200254	2.736266376	99555.84939	4.975526933	70.06564807	8.44504E-05	2.549318815	99553.86804	4.976654807	73.38118777
20 to 24	0.000284916	2.590582866	99456.23375	4.969396887	65.13305124	7.81523E-05	2.522947421	99511.86783	4.974634055	68.41099405
25 to 29	0.000321229	2.547382647	99314.68109	4.961823814	60.22214136	9.62324E-05	2.623588983	99473.0102	4.972507505	63.43660997
30 to 34	0.000357357	2.532426962	99155.33557	4.95339825	55.31474593	0.000128941	2.653433122	99425.19474	4.969739689	58.46565562
35 to 39	0.000376312	2.580069369	98978.37304	4.944418843	50.40901273	0.000174668	2.646511063	99361.1552	4.966018426	53.50135811
40 to 44	0.000505112	2.657873631	98792.36687	4.933793475	45.49885468	0.000253107	2.780712961	99274.4618	4.960898755	48.54531791
45 to 49	0.000773085	2.719368598	98543.23672	4.918489241	40.60675873	0.000478494	2.783946762	99148.98985	4.952132414	43.60242497
50 to 54	0.001324394	2.764431334	98163.20464	4.893636226	35.75231859	0.000840255	2.721710491	98912.258	4.936148997	38.69872045
55 to 59	0.002440079	2.727566086	97515.63239	4.848851159	30.9697483	0.001358581	2.780733717	98497.98324	4.909956558	33.84794926
60 to 64	0.003995883	2.752058558	96334.04299	4.773849987	26.31344801	0.002534356	2.776698376	97832.47918	4.864215146	29.05574538
65 to 69	0.007492121	2.71556735	94430.28039	4.642103667	21.78408117	0.004747596	2.719630079	96604.11208	4.778375083	24.38365529
70 to 74	0.012169724	2.752878558	90966.75268	4.427754226	17.50329169	0.007328207	2.840192047	94353.36695	4.643792134	19.89122892
75 to 79	0.024728211	2.753580114	85608.56561	4.056758139	13.41829134	0.016575813	2.859425576	90992.48819	4.392829753	15.50896112
80 to 84	0.050713793	2.686582041	75711.12549	3.39474047	9.802253028	0.0363025	2.768484066	83871.28534	3.885400142	11.5712874
85 to 89	0.100225116	2.527181722	58912.16194	2.378176189	6.800076247	0.075181339	2.64531064	70297.50152	3.014294607	8.182948888
90 to 94	0.191889742	2.234636132	35997.98783	1.191742658	4.57459728	0.149627424	2.326013774	49217.08381	1.798601259	5.580189579
95 plus	0.293642884	3.424023	13969.29318	0.490524235	3.424023	0.249034223	4.09802868	24404.28839	1.058361705	4.09802868

Table 15: Kuwait 2017 life table, by age and sex. mx=mortality rate, ax=mean person-years lived in an age interval among those who die in that age interval, lx=number of persons left alive at age x, nLx=person-years lived between age x and x+n, ex=life expectancy at age x.

Age Group	Male					Female				
	mx	ax	lx	nLx	ex	mx	ax	lx	nLx	ex
Early Neonatal	0.174504516	0.009583693	100000	0.019146027	81.17569656	0.144191519	0.009584622	100000	0.01915159	86.62547526
Late Neonatal	0.027068118	0.028759657	99665.90279	0.057297402	81.42857781	0.023544456	0.028760629	99723.85314	0.057336525	86.84613748
Post Neonatal	0.001967397	0.461504075	99510.8204	0.917937195	81.49790062	0.00199994	0.461501763	99588.86085	0.918643295	86.90628297
1 to 4	0.000306615	1.999591179	99330.23095	3.970773937	80.72193509	0.000238644	1.999681808	99405.14414	3.974308659	86.14276139
5 to 9	0.000213692	2.499554808	99208.4911	4.957775647	76.81853937	0.000146568	2.49969465	99310.30402	4.963696244	82.22311953
10 to 14	0.000232113	2.788018278	99102.55375	4.952584246	71.89798643	0.000103759	2.497397803	99237.55465	4.960589481	77.2815655
15 to 19	0.000535085	2.671344741	98987.60238	4.943220949	66.97823793	0.000145278	2.607417392	99186.0852	4.957581088	72.32037163
20 to 24	0.000674879	2.532494207	98723.10029	4.927948697	62.15049619	0.000178888	2.578940465	99114.06306	4.95355782	67.3710248
25 to 29	0.000644112	2.527812613	98390.52561	4.911705054	57.35196934	0.000213418	2.604250876	99025.4504	4.94874228	62.4289972
30 to 34	0.000765697	2.573371936	98074.15997	4.894613765	52.52878138	0.000286225	2.619897475	98919.83609	4.942624723	57.49286225
35 to 39	0.00091998	2.624029044	97699.38625	4.874315191	47.72036578	0.000379061	2.667757296	98778.36666	4.934555907	52.57143948
40 to 44	0.001324022	2.674316145	97250.96792	4.847621784	42.92824633	0.000593329	2.69622712	98591.31846	4.922836895	47.66610095
45 to 49	0.002049713	2.709097688	96609.15256	4.807882075	38.19559286	0.000942275	2.694734259	98299.2351	4.904308689	42.79969968
50 to 54	0.003437896	2.698010796	95623.72376	4.743646911	33.56117953	0.001485095	2.691116035	97837.1202	4.875139774	37.98909352
55 to 59	0.005462882	2.677432682	93993.02838	4.640774917	29.09646716	0.002331893	2.698892534	97113.12955	4.829740851	33.25219479
60 to 64	0.008452634	2.636848881	91458.10794	4.483363774	24.82851047	0.00378133	2.691299422	95986.92028	4.757812397	28.61060565
65 to 69	0.011777353	2.663776629	87669.08823	4.266099283	20.78739044	0.005980641	2.699535294	94187.91898	4.645487082	24.10557229
70 to 74	0.019597053	2.63354193	82645.91027	3.949205412	16.88872167	0.009983597	2.695257531	91409.83035	4.467702847	19.75600449
75 to 79	0.028466188	2.617548285	74909.21685	3.507677127	13.36086645	0.016614688	2.711010874	86950.02196	4.188244346	15.63091316
80 to 84	0.045739699	2.582533553	64928.96654	2.923382646	10.01235229	0.03083632	2.669163499	79992.79446	3.731507315	11.75445783
85 to 89	0.093817863	2.545119263	51565.45273	2.095872646	6.938395054	0.068025965	2.665428672	68489.49722	2.955298091	8.280382528
90 to 94	0.185710041	2.249958692	31914.50189	1.056461629	4.644878421	0.142778789	2.354787938	48393.00037	1.756500534	5.612527173
95 plus	0.288691636	3.464076507	12304.80818	0.426390243	3.464076507	0.243004555	4.115448033	23323.08196	0.960108825	4.115448033

Table 15: Kuwait 2100 life table, by age and sex. mx=mortality rate, ax=mean person-years lived in an age interval among those who die in that age interval, lx=number of persons left alive at age x, nLx=person-years lived between age x and x+n, ex=life expectancy at age x.										
Age Group	Male					Female				
	mx	ax	lx	nLx	ex	mx	ax	lx	nLx	ex
Early Neonatal	0.041198489	0.009587778	100000	0.019170512	86.17783213	0.033451309	0.009588016	100000	0.019171934	91.72609188
Late Neonatal	0.007250001	0.028765123	99921.08253	0.05747686	86.22661843	0.006760151	0.028765259	99935.90211	0.057486192	91.76569755
Post Neonatal	0.000413082	0.461614491	99879.44097	0.921998824	86.20492698	0.000439507	0.461612614	99897.05943	0.922150212	91.74381483
1 to 4	7.46412E-05	1.999900478	99841.37971	3.993059504	85.31429036	5.7584E-05	1.999923223	99856.55668	3.993802631	90.85748728
5 to 9	7.89325E-05	2.499835556	99811.59914	4.98959546	81.3391315	6.4957E-05	2.499864672	99833.5774	4.990868611	86.87789893
10 to 14	0.000112162	3.065500231	99772.22459	4.987435699	76.37024393	5.18609E-05	2.540602443	99801.17019	4.989419472	81.90526562
15 to 19	0.000314379	2.743191277	99716.31355	4.982192495	71.41136878	7.20614E-05	2.580664546	99775.30317	4.987891315	76.92581575
20 to 24	0.00040926	2.562380618	99559.74595	4.973015433	66.51918375	7.53897E-05	2.531350773	99739.3745	4.986043772	71.95257888
25 to 29	0.000420977	2.500069964	99356.37106	4.962615589	61.64969687	8.59988E-05	2.608576843	99701.7999	4.984058056	66.97868521
30 to 34	0.000428238	2.508032529	99147.63663	4.952110373	56.77356456	0.000113388	2.616169731	99658.9575	4.981592501	62.0062423
35 to 39	0.00045122	2.557032949	98935.79419	4.941362752	51.88915045	0.000140407	2.682794503	99602.49342	4.978495339	57.03975934
40 to 44	0.000574087	2.628643382	98713.14957	4.928936804	46.99906205	0.000227097	2.744882487	99532.61534	4.974069999	52.07769223
45 to 49	0.000806687	2.719783597	98430.6279	4.912455331	42.12414952	0.000394602	2.719605321	99419.6978	4.966504239	47.13337622
50 to 54	0.001417659	2.746189525	98035.1996	4.886101588	37.27967389	0.000632513	2.696962228	99223.80689	4.953962134	42.22037934
55 to 59	0.002505349	2.747705877	97344.84718	4.839651398	32.51886539	0.000982132	2.704309118	98910.68903	4.934368235	37.34447945
60 to 64	0.004172008	2.708639947	96139.34423	4.761457766	27.88325858	0.001558096	2.69563212	98426.59279	4.903714821	32.51346094
65 to 69	0.006743077	2.731678898	94168.32132	4.637783263	23.39795693	0.002445877	2.719117079	97663.62085	4.856092623	27.74443201
70 to 74	0.012249887	2.684731302	91076.91677	4.42974358	19.08435603	0.004167338	2.770647089	96478.4254	4.779550215	23.04907626
75 to 79	0.019162541	2.689457459	85752.63523	4.109414537	15.08225605	0.008021542	2.828606125	94494.23933	4.643690987	18.46953962
80 to 84	0.034638605	2.628451933	78096.48764	3.619160379	11.28335258	0.017338505	2.758954847	90794.34336	4.37117991	14.09995408
85 to 89	0.075310633	2.608355792	66104.3287	2.823662442	7.83671349	0.043135197	2.781613261	83294.10582	3.805536514	10.10308126
90 to 94	0.161201969	2.242946209	46029.06889	1.61934611	5.176293081	0.104480292	2.331202023	67170.458	2.639683711	6.851283138
95 plus	0.267568532	3.769275727	21280.36405	0.830085425	3.769275727	0.205276825	4.908074452	40223.39175	2.006758452	4.908074452

Table 15: Lebanon 2017 life table, by age and sex. mx=mortality rate, ax=mean person-years lived in an age interval among those who die in that age interval, lx=number of persons left alive at age x, nLx=person-years lived between age x and x+n, ex=life expectancy at age x.

Age Group	Male					Female				
	mx	ax	lx	nLx	ex	mx	ax	lx	nLx	ex
Early Neonatal	0.135070256	0.009584901	100000	0.019153265	75.58678437	0.124149884	0.009585236	100000	0.01915527	79.5859925
Late Neonatal	0.019447168	0.028761759	99741.30933	0.057353323	75.76348422	0.019120959	0.028761849	99762.19635	0.057365871	79.75644502
Post Neonatal	0.001888643	0.46150967	99629.78691	0.919068118	75.79067668	0.00297813	0.461432274	99652.51724	0.918815774	79.7866439
1 to 4	0.000274679	1.999633762	99456.2376	3.976065242	74.99877704	0.000210959	1.999718721	99378.92185	3.97348041	79.08172734
5 to 9	0.000194452	2.499594892	99347.04509	4.96493842	71.07898377	0.000180075	2.499624843	99295.11068	4.962521286	75.14679062
10 to 14	0.000218208	3.070185443	99250.5074	4.960436715	66.14568488	0.000177979	2.693798151	99205.75422	4.958253103	70.21222437
15 to 19	0.000793784	2.764789241	99142.27621	4.948332077	61.21453012	0.000346655	2.63977319	99117.5114	4.951822558	65.27232056
20 to 24	0.001231248	2.569252155	98749.51865	4.922744376	56.4469564	0.000410132	2.563778688	98945.85292	4.942353705	60.38093753
25 to 29	0.001215596	2.512292082	98143.4289	4.8923775	51.77961075	0.000473194	2.585913883	98743.15396	4.931524239	55.49960578
30 to 34	0.001319039	2.565127621	97548.74119	4.861822241	47.07989901	0.0006077	2.630840958	98509.79962	4.91840906	50.62494105
35 to 39	0.001646383	2.625203125	96907.49249	4.826504817	42.37441127	0.000859469	2.677394599	98210.90887	4.900762824	45.77098983
40 to 44	0.002340347	2.660557934	96112.93333	4.779481064	37.7029798	0.001349233	2.69526988	97789.70781	4.874327788	40.95656731
45 to 49	0.003516558	2.675078589	94994.48249	4.71121013	33.11554427	0.002146551	2.688018286	97132.06177	4.832622118	36.21555287
50 to 54	0.005446285	2.679279706	93338.00231	4.608654607	28.65577501	0.003344431	2.686112701	96094.75755	4.767848137	31.57736297
55 to 59	0.008564534	2.670063045	90828.50659	4.452587049	24.3735215	0.005277871	2.695703245	94500.32684	4.66825498	27.06462481
60 to 64	0.013318591	2.657805587	87015.92203	4.219210064	20.32447271	0.008668889	2.692794359	92036.92927	4.511631095	22.71657513
65 to 69	0.020802184	2.648763475	81397.78636	3.880166813	16.54357176	0.014254135	2.680878506	88127.18153	4.26540523	18.60462114
70 to 74	0.033516629	2.627692617	73328.87185	3.396506071	13.07210251	0.02361078	2.669544551	82050.18771	3.888642078	14.78396127
75 to 79	0.05503858	2.584589866	61950.56468	2.734273335	9.990097369	0.040716632	2.643018812	72874.36064	3.324802898	11.30966535
80 to 84	0.091589379	2.508705673	46911.97465	1.910143692	7.364184146	0.073340764	2.571845889	59343.74979	2.518856633	8.285896392
85 to 89	0.153637177	2.369201826	29432.93857	1.048369196	5.249005896	0.13045665	2.441299209	40879.85679	1.532749048	5.866714097
90 to 94	0.248771613	2.149074111	13342.41608	0.390461899	3.725604048	0.217814761	2.23467617	20898.88097	0.652346807	4.143538647
95 plus	0.340379949	2.938089118	3637.104819	0.106924361	2.938089118	0.313269591	3.192465118	6700.531325	0.214041595	3.192465118

Table 15: Lebanon 2100 life table, by age and sex. mx=mortality rate, ax=mean person-years lived in an age interval among those who die in that age interval, lx=number of persons left alive at age x, nLx=person-years lived between age x and x+n, ex=life expectancy at age x.

Age Group	Male					Female				
	mx	ax	lx	nLx	ex	mx	ax	lx	nLx	ex
Early Neonatal	0.025978314	0.009588245	100000	0.019173306	82.82855374	0.026289707	0.009588235	100000	0.019173249	84.6503796
Late Neonatal	0.004008231	0.028766018	99950.19674	0.057498963	82.85068011	0.004365048	0.028765919	99949.59862	0.057498029	84.67385163
Post Neonatal	0.000341764	0.461619557	99927.15278	0.922469553	82.81224689	0.000538244	0.4616056	99924.50295	0.922361439	84.63755634
1 to 4	8.87831E-05	1.99988162	99895.63098	3.995115968	81.91492778	8.61906E-05	1.999885071	99874.86443	3.994307192	83.7560634
5 to 9	6.45679E-05	2.499865483	99860.17095	4.992202811	77.94324703	7.36049E-05	2.499846656	99840.51996	4.991108442	79.78401883
10 to 14	9.71415E-05	3.451250446	99827.94396	4.990605911	72.9675317	9.06498E-05	2.795666325	99803.82132	4.989184821	74.81242324
15 to 19	0.000469625	2.801972267	99779.48007	4.983824684	68.00120045	0.000192851	2.656331133	99758.62337	4.985670824	69.84501777
20 to 24	0.0007748	2.593632783	99545.50398	4.968010158	63.15374436	0.000227334	2.572849699	99662.54699	4.980376067	64.90976245
25 to 29	0.000810566	2.524532379	99161.0005	4.948129472	58.38739709	0.000268004	2.588145868	99549.39787	4.974252121	59.98056365
30 to 34	0.00087852	2.52522491	98760.2024	4.927309483	53.61325354	0.000337611	2.599791172	99416.14766	4.966782119	55.05742676
35 to 39	0.00092075	2.572570641	98327.98695	4.905435302	48.83687757	0.000428971	2.662084337	99248.52689	4.957453453	50.14587015
40 to 44	0.001195137	2.619624304	97876.90952	4.879957795	44.04910587	0.000670678	2.733890646	99035.95401	4.944276568	45.24755632
45 to 49	0.001598957	2.655367784	97294.50685	4.846566754	39.29584206	0.001180066	2.711163397	98704.4468	4.921922614	40.38989818
50 to 54	0.002406213	2.68810812	96520.68446	4.799391007	34.58789292	0.001871578	2.707314693	98123.9311	4.8852332	35.61205737
55 to 59	0.003842865	2.681752368	95368.53568	4.726394669	29.97063313	0.0030771	2.702833491	97210.1038	4.826425715	30.92017389
60 to 64	0.005887353	2.678154248	93557.67162	4.614929343	25.49487918	0.005003863	2.683437842	95726.07078	4.731533726	26.35569889
65 to 69	0.009210113	2.689852852	90850.56956	4.448238045	21.16973477	0.007790886	2.688191322	93361.99517	4.585638959	21.95149742
70 to 74	0.015349452	2.713468336	86772.51511	4.192417466	17.03228287	0.012756987	2.729310489	89798.5649	4.363882126	17.71108915
75 to 79	0.0282114	2.686443687	80388.16772	3.775600808	13.16136298	0.024173875	2.728462017	84254.50959	3.994562886	13.69050512
80 to 84	0.050526861	2.613428901	69877.92371	3.12405873	9.731539923	0.047984074	2.653321713	74664.55245	3.359511515	10.0901816
85 to 89	0.100284126	2.525749942	54398.2854	2.190076457	6.755645158	0.094739262	2.562084271	58759.05644	2.395502874	7.091478977
90 to 94	0.192516658	2.238055794	32989.41084	1.085938945	4.546818056	0.176531066	2.31183417	36527.05909	1.247708178	4.875807014
95 plus	0.294285901	3.408018716	12564.90699	0.435208106	3.408018716	0.275047784	3.650638947	14976.67406	0.556013481	3.650638947

Table 15: Libya 2017 life table, by age and sex. mx=mortality rate, ax=mean person-years lived in an age interval among those who die in that age interval, lx=number of persons left alive at age x, nLx=person-years lived between age x and x+n, ex=life expectancy at age x.

Age Group	Male					Female				
	mx	ax	lx	nLx	ex	mx	ax	lx	nLx	ex
Early Neonatal	0.167171297	0.009583917	100000	0.019147373	71.43633838	0.157963679	0.0095842	100000	0.019149062	75.29706705
Late Neonatal	0.017154977	0.028762391	99679.91955	0.057321799	71.64648027	0.017390799	0.028762326	99697.52045	0.057331532	75.50629726
Post Neonatal	0.002999978	0.461430722	99581.59013	0.918152491	71.6596568	0.00283111	0.461442718	99597.82094	0.918373668	75.52431771
1 to 4	0.001143927	1.998474764	99306.17063	3.96317393	70.93382683	0.00113052	1.998492641	99337.83584	3.964543321	74.79749129
5 to 9	0.000520902	2.498914787	98852.88514	4.936213899	67.24990925	0.00045804	2.49904575	98889.67769	4.938826576	71.12740861
10 to 14	0.000570983	2.891557714	98595.78291	4.9238617	62.41876604	0.000430517	2.601181573	98663.47189	4.928085064	66.28475833
15 to 19	0.001603636	2.751669693	98314.65694	4.898073726	57.58890574	0.000670619	2.644208343	98451.31539	4.914802674	61.42192705
20 to 24	0.002538112	2.569343797	97529.21681	4.846562697	53.03004657	0.000900762	2.578032042	98121.72619	4.895408355	56.61917683
25 to 29	0.002496831	2.521475309	96299.18745	4.785350062	48.67389753	0.001017015	2.599187129	97680.78494	4.87214541	51.86283487
30 to 34	0.002867508	2.539860965	95104.50404	4.721924048	44.2530065	0.001399075	2.60380676	97185.31898	4.843034609	47.1136207
35 to 39	0.003129099	2.569830726	93750.74305	4.652176293	39.85456805	0.001736466	2.644679797	96507.82778	4.80574086	42.42557334
40 to 44	0.004035625	2.610207405	92295.48165	4.57071701	35.44153821	0.002644977	2.647319018	95673.51051	4.754097485	37.77178298
45 to 49	0.005451507	2.6126318	90451.79648	4.464526391	31.10957738	0.003679799	2.630855259	94416.46606	4.680036653	33.23850856
50 to 54	0.007308008	2.628430439	88019.70772	4.326076123	26.89555252	0.005104128	2.645835706	92695.07478	4.579751894	28.80582784
55 to 59	0.010577182	2.691535901	84861.40513	4.142048063	22.79714743	0.007556837	2.747888992	90358.92696	4.442391305	24.48090332
60 to 64	0.018634325	2.637958348	80486.13175	3.854904703	18.88846947	0.01505665	2.670872966	87004.64742	4.202994756	20.31721099
65 to 69	0.026497074	2.608606602	73315.2014	3.447792028	15.47625478	0.022306727	2.634553903	80683.56554	3.832310647	16.69739528
70 to 74	0.040890642	2.58344415	64201.6684	2.922269789	12.30209173	0.035686145	2.591259933	72150.35871	3.322693169	13.35839497
75 to 79	0.061243841	2.550613002	52293.00187	2.274969912	9.516754018	0.051277401	2.56379237	60327.25719	2.682679085	10.46759622
80 to 84	0.099862958	2.484769876	38426.01321	1.537386008	7.03685436	0.083099378	2.522778485	46632.72026	1.935552428	7.791819045
85 to 89	0.163112406	2.344580817	23159.97002	0.809491908	5.051751742	0.142685391	2.406333533	30643.38722	1.120349515	5.552021918
90 to 94	0.257878357	2.130365599	10028.35072	0.288774792	3.620096297	0.230851958	2.205407985	14757.61064	0.449639868	3.960385785
95 plus	0.347587725	2.877679365	2613.367493	0.075423632	2.877679365	0.324952607	3.078976963	4435.171494	0.137129109	3.078976963

Table 15: Libya 2100 life table, by age and sex. mx=mortality rate, ax=mean person-years lived in an age interval among those who die in that age interval, lx=number of persons left alive at age x, nLx=person-years lived between age x and x+n, ex=life expectancy at age x.

Age Group	Male					Female				
	mx	ax	lx	nLx	ex	mx	ax	lx	nLx	ex
Early Neonatal	0.029194451	0.009588146	100000	0.019172715	77.50135951	0.034371864	0.009587988	100000	0.019171765	81.59423517
Late Neonatal	0.003311508	0.02876621	99944.03758	0.057496574	77.52385983	0.004671575	0.028765835	99934.13342	0.057488633	81.6266073
Post Neonatal	0.000553704	0.461604501	99925.00653	0.922359639	77.48017105	0.000762678	0.461589656	99907.3109	0.922107896	81.58988391
1 to 4	0.000599786	1.99920029	99873.9749	3.990198288	76.59358014	0.000702707	1.99906307	99837.16097	3.98793822	80.72033703
5 to 9	0.000360559	2.499248839	99636.49417	4.977406543	72.74614916	0.000386413	2.499194977	99560.68755	4.973330603	76.92439376
10 to 14	0.000465547	3.1245144	99460.10782	4.968471343	67.85236199	0.000430328	2.695458013	99372.93943	4.963703894	72.05794324
15 to 19	0.001553375	2.827034463	99233.71017	4.94528332	62.9823769	0.000633521	2.651337355	99165.62316	4.950999992	67.19746265
20 to 24	0.002772323	2.618708184	98493.96973	4.894240085	58.38394446	0.00073028	2.576710818	98862.70115	4.934564564	62.39179551
25 to 29	0.00273721	2.582153622	97238.49575	4.832140428	54.05319282	0.000755356	2.627889333	98515.14413	4.917079072	57.6017178
30 to 34	0.002916631	2.54002921	96048.45979	4.770950917	49.68983525	0.001050112	2.637995483	98155.38414	4.89569631	52.8022012
35 to 39	0.002592525	2.536454328	94796.45547	4.711959882	45.33771907	0.0011702	2.665931913	97656.27788	4.869447862	48.0575207
40 to 44	0.002913311	2.566116423	93680.99223	4.652973964	40.87221075	0.001575943	2.655832213	97100.80738	4.837357005	43.31677181
45 to 49	0.003343245	2.58605532	92410.59264	4.585220561	36.4174125	0.002254732	2.630223271	96350.97971	4.792253077	38.63362447
50 to 54	0.004295735	2.627893431	90950.75881	4.503354101	31.97274615	0.002984895	2.633153478	95285.68025	4.731294229	34.03381643
55 to 59	0.006251623	2.709166041	89088.1291	4.393074181	27.59299342	0.00420601	2.75217607	93893.97961	4.65114177	29.49629397
60 to 64	0.01080137	2.634355743	86428.39576	4.216200958	23.35873678	0.00820758	2.667519379	91962.99523	4.512586179	25.05134638
65 to 69	0.014571473	2.627724658	81983.12709	3.965622135	19.47929251	0.011218166	2.649533262	88304.84175	4.303760931	20.9630716
70 to 74	0.022958295	2.624484265	76356.01571	3.626507036	15.7126118	0.019047987	2.667369925	83544.07684	4.005911938	16.99196863
75 to 79	0.035265504	2.637549895	68311.23549	3.163793346	12.24585528	0.029396269	2.699577365	76262.47663	3.581247506	13.31210561
80 to 84	0.062119424	2.554660087	57713.15456	2.523919019	9.028071756	0.055343558	2.616316166	66336.41808	2.954633142	9.900066166
85 to 89	0.115480466	2.480634188	42919.64407	1.686468297	6.30808636	0.10307276	2.544099562	51237.97331	2.08849502	6.99721693
90 to 94	0.208785665	2.214184203	24636.75508	0.794574876	4.301425337	0.184206089	2.285723406	31996.02915	1.107204961	4.834270969
95 plus	0.307673166	3.26753323	8879.02405	0.299971299	3.26753323	0.2815178	3.626964061	13718.70105	0.5368206	3.626964061

Table 15: Morocco 2017 life table, by age and sex. mx=mortality rate, ax=mean person-years lived in an age interval among those who die in that age interval, lx=number of persons left alive at age x, nLx=person-years lived between age x and x+n, ex=life expectancy at age x.

Age Group	Male					Female				
	mx	ax	lx	nLx	ex	mx	ax	lx	nLx	ex
Early Neonatal	0.607127182	0.009570433	100000	0.019066874	73.07035903	0.399182071	0.009576806	100000	0.019104863	74.54373727
Late Neonatal	0.066053173	0.028748903	98842.55243	0.056760427	73.9064943	0.067234586	0.028748577	99237.41993	0.056985231	75.0971306
Post Neonatal	0.007744294	0.461093694	98467.74737	0.905899337	74.13019016	0.005522412	0.461251533	98854.34711	0.910387636	75.33046466
1 to 4	0.000679107	1.999094524	97766.4942	3.905355812	73.73548311	0.00081559	1.998912547	98351.71766	3.927660442	74.78981826
5 to 9	0.00055427	2.498845272	97501.41899	4.86832405	69.93061158	0.000357261	2.499255707	98031.48147	4.897199395	71.02764892
10 to 14	0.000487654	2.579353707	97231.66856	4.855853779	65.11774219	0.000295808	2.679224586	97856.54705	4.889470551	66.15000995
15 to 19	0.000744743	2.695362991	96994.91724	4.84144018	60.27027915	0.000613102	2.646172699	97711.92834	4.878559955	61.24387955
20 to 24	0.001196598	2.595252435	96634.38262	4.817855765	55.48459061	0.000732809	2.566891231	97412.83421	4.861979302	56.42347563
25 to 29	0.001302296	2.562249942	96057.94333	4.787699547	50.80121893	0.000859402	2.595654448	97056.58767	4.842826113	51.6206649
30 to 34	0.001604077	2.578488215	95434.55306	4.753271677	46.11559504	0.001137171	2.645313265	96640.49122	4.819121879	46.83109487
35 to 39	0.001935733	2.600672107	94672.28768	4.711743153	41.46526749	0.001665629	2.677656963	96092.65711	4.786120326	42.08224037
40 to 44	0.002586741	2.622175039	93760.58113	4.659388873	36.84207344	0.002584989	2.675745464	95295.83864	4.736343822	37.41072801
45 to 49	0.00353033	2.669114263	92556.02804	4.590059825	32.28597863	0.003919452	2.664391985	94072.19691	4.6609629	32.86130844
50 to 54	0.005614179	2.709629686	90936.97214	4.489180092	27.81144774	0.005847877	2.657875194	92246.77036	4.550071478	28.45726609
55 to 59	0.009599054	2.706645366	88419.70505	4.325886502	23.52360423	0.008751067	2.658338173	89588.54403	4.389578165	24.22096787
60 to 64	0.016335368	2.658846386	84274.33136	4.058826909	19.54431721	0.013403477	2.655841498	85752.58067	4.157219633	20.18333317
65 to 69	0.025114145	2.618605304	77659.98015	3.664563828	15.97885098	0.02094999	2.649562659	80190.33176	3.821780729	16.39679797
70 to 74	0.037835656	2.580657359	68489.42818	3.138630284	12.76504989	0.033898227	2.63090378	72203.89382	3.342753683	12.91495071
75 to 79	0.055254427	2.570354763	56677.3394	2.500645741	9.888144771	0.056422861	2.589814785	60917.60208	2.683073717	9.818762033
80 to 84	0.095144549	2.517964303	42965.46211	1.7410793	7.231094443	0.096708413	2.508366507	45867.34725	1.851303419	7.193672265
85 to 89	0.157854729	2.358441072	26557.03565	0.939948765	5.164627029	0.159476954	2.361843356	28125.06311	0.993013134	5.166192073
90 to 94	0.25278431	2.140700421	11859.39485	0.345430811	3.680483625	0.248292992	2.165373089	12448.16746	0.366906746	3.736494381
95 plus	0.34354112	2.912241578	3192.569236	0.093447496	2.912241578	0.340350248	2.940653086	3421.523586	0.101314149	2.940653086

Table 15: Morocco 2100 life table, by age and sex. mx=mortality rate, ax=mean person-years lived in an age interval among those who die in that age interval, lx=number of persons left alive at age x, nLx=person-years lived between age x and x+n, ex=life expectancy at age x.

Age Group	Male					Female				
	mx	ax	lx	nLx	ex	mx	ax	lx	nLx	ex
Early Neonatal	0.116850002	0.00958546	100000	0.019156612	83.488824	0.078719002	0.009586628	100000	0.019163614	85.85807843
Late Neonatal	0.01045075	0.02876424	99776.19048	0.057388227	83.65671108	0.013036343	0.028763527	99849.16297	0.057425927	85.96844792
Post Neonatal	0.001145835	0.461562437	99716.22348	0.920180821	83.6494002	0.000748687	0.46159065	99774.30972	0.920885622	85.97532894
1 to 4	0.000115601	1.999845866	99610.8001	3.983511379	82.81405041	0.000138103	1.999815863	99705.37414	3.987114097	85.11107922
5 to 9	9.53889E-05	2.499801273	99564.77739	4.977052712	78.85116883	9.55682E-05	2.4998009	99650.34115	4.981327674	81.15678099
10 to 14	0.000132159	2.810103249	99517.33835	4.974393846	73.88737406	0.000108823	2.763971643	99602.7724	4.978914614	76.19421478
15 to 19	0.000258347	2.754046772	99451.68273	4.969681377	68.93411068	0.000220368	2.60053636	99548.64681	4.974796617	71.23403907
20 to 24	0.000426074	2.638703888	99323.45081	4.961171674	64.01933958	0.000208673	2.532139896	99439.09391	4.969398884	66.30953201
25 to 29	0.000528485	2.586084623	99112.33687	4.949298746	59.1497961	0.000254326	2.628208382	99335.48077	4.963775147	61.3759667
30 to 34	0.000634521	2.554409526	98851.15367	4.934895156	54.29891808	0.000358645	2.649117889	99209.35977	4.956278543	56.45051014
35 to 39	0.000681634	2.571218547	98538.52828	4.918787034	49.46280973	0.000498493	2.680357239	99031.76252	4.945858503	51.54680473
40 to 44	0.00086236	2.623634428	98203.85351	4.900147987	44.62229013	0.000778587	2.705626629	98785.40852	4.930449954	46.66840262
45 to 49	0.001181569	2.686551895	97782.03345	4.87577617	39.80267257	0.001256577	2.672547349	98401.78254	4.905732437	41.83934645
50 to 54	0.001918086	2.739295368	97206.9684	4.839319892	35.02047464	0.001818964	2.664281514	97785.74338	4.868603581	37.08526134
55 to 59	0.003351744	2.737214683	96280.60897	4.777839417	30.32735429	0.002716636	2.654032581	96900.85889	4.814360105	32.39843575
60 to 64	0.005863558	2.739172525	94682.92412	4.672274233	25.7862615	0.00388027	2.669507245	95594.32805	4.736917999	27.80327944
65 to 69	0.010367948	2.680385513	91956.04156	4.490286169	21.45627468	0.006045015	2.695764462	93758.83649	4.6236124	23.29302523
70 to 74	0.015803813	2.665188411	87334.31328	4.213159359	17.43239117	0.009986928	2.748779665	90969.64048	4.448698853	18.92141993
75 to 79	0.026730044	2.67766153	80754.32768	3.807068128	13.61520852	0.019478011	2.746525012	86541.92045	4.145871024	14.74346436
80 to 84	0.046863899	2.623859546	70879.79887	3.201387861	10.11753501	0.038591557	2.665454715	78516.00597	3.604452756	10.96206817
85 to 89	0.094273588	2.546206746	56516.63051	2.317454878	7.023435792	0.080321457	2.616788388	64765.31141	2.72521957	7.710709244
90 to 94	0.184883644	2.239790105	35808.3348	1.204579935	4.701292478	0.158553137	2.336235245	43267.18571	1.530021049	5.257915074
95 plus	0.287737894	3.496665198	14546.04079	0.524012385	3.496665198	0.258039141	3.891481042	19477.51085	0.769199459	3.891481042

Table 15: Palestine 2017 life table, by age and sex. mx=mortality rate, ax=mean person-years lived in an age interval among those who die in that age interval, lx=number of persons left alive at age x, nLx=person-years lived between age x and x+n, ex=life expectancy at age x.

Age Group	Male					Female				
	mx	ax	lx	nLx	ex	mx	ax	lx	nLx	ex
Early Neonatal	0.24587531	0.009581505	100000	0.019132939	76.10530007	0.220572033	0.009582281	100000	0.019137577	77.4666092
Late Neonatal	0.041215184	0.028755754	99529.59462	0.057195762	76.44579558	0.032636793	0.02875812	99577.8879	0.057237635	77.77576499
Post Neonatal	0.005932216	0.461222421	99293.86285	0.914262291	76.56968611	0.005120459	0.461280087	99391.09288	0.915500077	77.8643541
1 to 4	0.000646292	1.999138277	98751.59032	3.944963218	76.06444795	0.000574126	1.999234498	98922.35294	3.952354595	77.30787461
5 to 9	0.000289112	2.499397682	98496.68199	4.921277052	72.25620077	0.000237777	2.499504631	98695.46448	4.931841125	73.4810295
10 to 14	0.000263077	2.779787918	98354.43493	4.914845516	67.35713628	0.00020993	2.500965426	98578.20382	4.92632585	68.5654742
15 to 19	0.000638107	2.706895302	98225.16317	4.904082231	62.4421664	0.000239292	2.552697136	98474.78958	4.920857897	63.63485097
20 to 24	0.000900462	2.565339872	97912.24559	4.884903644	57.63305579	0.000271093	2.58639262	98357.04186	4.91463646	58.707968
25 to 29	0.000927214	2.532628204	97472.40811	4.862497071	52.88154387	0.000352504	2.663865709	98223.81446	4.907149738	53.78408148
30 to 34	0.001055142	2.574347787	97021.58582	4.838696022	48.1155078	0.000549883	2.631861592	98050.842	4.896166566	48.87425129
35 to 39	0.001315773	2.622168577	96511.0815	4.810504338	43.35642307	0.00070355	2.707312752	97781.61787	4.881207689	44.00154829
40 to 44	0.00184675	2.679657584	95878.17634	4.773455464	38.62530774	0.001257763	2.65925333	97438.21218	4.857609743	39.14704954
45 to 49	0.002953177	2.699004499	94996.68414	4.717777363	33.9587766	0.00168043	2.735949656	96827.25797	4.823014094	34.37720704
50 to 54	0.00477979	2.704669929	93603.48188	4.629388644	29.42383963	0.003207736	2.738778715	96016.81733	4.766270973	29.64418797
55 to 59	0.007951226	2.701327758	91390.84785	4.487529774	25.07038158	0.005489884	2.813819052	94488.00167	4.668374147	25.07933362
60 to 64	0.013291847	2.66903466	87823.05313	4.259212916	20.97846037	0.012050049	2.701603805	91925.32196	4.472414289	20.6997495
65 to 69	0.02084173	2.642359419	82162.93925	3.915803673	17.23892127	0.018762764	2.668471437	86536.79942	4.145533277	16.81991746
70 to 74	0.032745031	2.617472173	74004.8536	3.432619634	13.84707169	0.031880035	2.650214869	78760.71737	3.663702661	13.21648849
75 to 79	0.052116904	2.5471861	62772.49076	2.783255861	10.85562705	0.054649009	2.59205129	67086.42346	2.96451061	10.05468732
80 to 84	0.070956121	2.473089187	48285.11307	2.047744752	8.348790642	0.090776669	2.504278496	50898.75426	2.075309618	7.428279607
85 to 89	0.127827723	2.441008902	33781.18298	1.27334564	5.874207006	0.152257319	2.379901447	32081.50034	1.147096652	5.318474554
90 to 94	0.222896753	2.198158876	17534.08492	0.540007904	4.061165729	0.240927145	2.182469618	14638.49752	0.436213826	3.824751372
95 plus	0.319528671	3.129979813	5515.244231	0.17279836	3.129979813	0.333919829	2.995062209	4141.246224	0.124144101	2.995062209

Table 15: Palestine 2100 life table, by age and sex. mx=mortality rate, ax=mean person-years lived in an age interval among those who die in that age interval, lx=number of persons left alive at age x, nLx=person-years lived between age x and x+n, ex=life expectancy at age x.

Age Group	Male					Female				
	mx	ax	lx	nLx	ex	mx	ax	lx	nLx	ex
Early Neonatal	0.086033054	0.009586404	100000	0.019162273	82.52266805	0.088649321	0.009586324	100000	0.019161792	83.01828129
Late Neonatal	0.007797027	0.028764972	99835.20308	0.057426551	82.63919457	0.008999549	0.028764641	99830.17695	0.057421676	83.1394737
Post Neonatal	0.001482233	0.46153854	99790.43129	0.92072266	82.61857838	0.001256024	0.46155461	99778.51165	0.920708871	83.12479592
1 to 4	0.000374108	1.99950119	99653.97699	3.983180834	81.80736878	0.000442884	1.999409488	99662.90149	3.982992882	82.29693423
5 to 9	0.000230793	2.499519182	99505.14692	4.972393687	77.9235086	0.000240312	2.499499349	99486.87153	4.971365177	78.43744198
10 to 14	0.00027126	2.979890058	99390.64679	4.966781027	73.00832769	0.000262139	2.563891908	99367.78611	4.965237601	73.52779698
15 to 19	0.000823093	2.797083052	99256.36132	4.953921748	68.10077128	0.00032802	2.523270463	99238.16837	4.957921349	68.61991412
20 to 24	0.001498019	2.574999547	98851.06545	4.924872221	63.36252853	0.000317553	2.513876525	99076.48837	4.949897479	63.72735035
25 to 29	0.001356511	2.478037058	98123.46228	4.889574871	58.80660197	0.000302774	2.63751146	98920.50387	4.94241583	58.82373518
30 to 34	0.001170055	2.494066282	97473.83303	4.859538184	54.18161171	0.000420525	2.594158078	98771.94056	4.933587156	53.90830837
35 to 39	0.00111685	2.540581951	96917.83564	4.83272218	49.48039925	0.000446836	2.658493845	98565.69069	4.923092231	49.01574708
40 to 44	0.001206857	2.606177541	96389.90227	4.805678137	44.74042429	0.000694588	2.640983834	98346.86743	4.909304889	44.11894759
45 to 49	0.001554326	2.649542266	95820.11324	4.77364706	39.99359197	0.000899144	2.750877083	98007.04421	4.890439398	39.26275366
50 to 54	0.002208277	2.691286446	95086.85995	4.730321946	35.28337011	0.001733261	2.728279274	97568.81387	4.85932053	34.42633782
55 to 59	0.003543405	2.703852836	94050.16032	4.664689032	30.64334304	0.002804722	2.814367857	96728.37673	4.807025236	29.70103681
60 to 64	0.005743267	2.712772094	92406.61641	4.560600642	26.14018177	0.006144582	2.697108872	95382.57911	4.702787647	25.07820214
65 to 69	0.009720369	2.695961259	89800.50239	4.391867993	21.81805816	0.009046927	2.718355691	92501.49865	4.531593153	20.76631288
70 to 74	0.015705387	2.678692799	85554.07593	4.127926292	17.764562	0.01634412	2.740738525	88426.56334	4.264681564	16.58849263
75 to 79	0.026401969	2.633638747	79106.26509	3.724692456	13.98838758	0.031568288	2.692922322	81517.51356	3.802308948	12.74717354
80 to 84	0.039969535	2.567216618	69379.31098	3.165479995	10.57632227	0.057186714	2.600905277	69724.3502	3.074970463	9.435316372
85 to 89	0.084647694	2.57573044	56906.03407	2.367863195	7.329721542	0.107583652	2.518750595	52609.37028	2.092567882	6.649440728
90 to 94	0.174284732	2.253876247	37222.58721	1.265524545	4.869840506	0.191397221	2.284534285	30935.15719	1.031968848	4.610760806
95 plus	0.278936708	3.593180942	15526.64655	0.564080525	3.593180942	0.288804778	3.484725213	11922.18242	0.427510689	3.484725213

Table 15: Oman 2017 life table, by age and sex. mx=mortality rate, ax=mean person-years lived in an age interval among those who die in that age interval, lx=number of persons left alive at age x, nLx=person-years lived between age x and x+n, ex=life expectancy at age x.

Age Group	Male					Female				
	mx	ax	lx	nLx	ex	mx	ax	lx	nLx	ex
Early Neonatal	0.276264066	0.009580574	100000	0.019127367	75.39643945	0.206713362	0.009582705	100000	0.019140118	79.39061979
Late Neonatal	0.024987068	0.028760231	99471.58275	0.057189108	75.77774726	0.025504284	0.028760088	99604.35094	0.057264589	79.68675436
Post Neonatal	0.0033453	0.461406191	99328.68584	0.915674697	75.82919273	0.003369579	0.461404466	99458.30371	0.916859332	79.74619579
1 to 4	0.00057974	1.999227013	99022.3709	3.956305779	75.13906161	0.000503735	1.999328353	99149.36826	3.961981714	79.06995449
5 to 9	0.000366205	2.499237072	98793.00857	4.935130933	71.30886026	0.000176906	2.499631445	98949.78563	4.945301843	75.22539369
10 to 14	0.000428175	2.702877455	98612.28395	4.925772767	66.43496501	0.00017073	2.700050372	98862.30101	4.941177203	70.28973389
15 to 19	0.000789522	2.688938589	98401.37639	4.911111459	61.5714404	0.000343761	2.638723725	98777.94113	4.934890394	65.34740995
20 to 24	0.001154508	2.528622173	98013.64764	4.886739916	56.80385975	0.000399565	2.560662032	98608.30458	4.925611764	60.45509625
25 to 29	0.000956352	2.472354334	97449.52801	4.860727385	52.11734619	0.000458975	2.585784241	98411.50405	4.915128675	55.57072763
30 to 34	0.001037998	2.543768101	96984.74748	4.836909326	47.35473934	0.000590414	2.638300115	98185.92407	4.902461501	50.69233911
35 to 39	0.001186995	2.610076154	96482.78873	4.810499213	42.58732457	0.000855853	2.682810717	97896.4969	4.885139089	45.83426127
40 to 44	0.001684893	2.687403511	95911.9664	4.776993832	37.82459068	0.001352094	2.704263464	97478.4482	4.858844599	41.01906298
45 to 49	0.002741735	2.720083278	95107.44701	4.725847054	33.12089234	0.002207309	2.698235459	96821.59923	4.816622894	36.27855586
50 to 54	0.004681898	2.738977942	93812.54606	4.641525189	28.53909146	0.003522612	2.690430152	95758.71204	4.749325214	31.65055918
55 to 59	0.008418502	2.726543361	91641.35761	4.496101711	24.14821777	0.005593044	2.690058971	94086.60907	4.644379278	27.16401804
60 to 64	0.014782348	2.70324806	87861.2098	4.249035644	20.06679334	0.009023923	2.679461725	91491.47226	4.480847624	22.85664066
65 to 69	0.025771329	2.621491486	81592.00909	3.844590282	16.39713935	0.014333839	2.672827306	87454.29161	4.231778593	18.78649356
70 to 74	0.036616475	2.578579411	71713.3787	3.294836505	13.29105432	0.023437805	2.665013518	81401.46177	3.859314547	14.98388978
75 to 79	0.054996697	2.540739242	59705.49612	2.631717445	10.44512256	0.039855513	2.636495978	72379.59677	3.308103798	11.52011478
80 to 84	0.078754507	2.477925616	45331.84122	1.893752214	7.958393696	0.069692405	2.570856257	59228.48654	2.533554568	8.494938888
85 to 89	0.137600797	2.413347226	30547.63319	1.129160086	5.627916535	0.125552331	2.45625916	41613.7982	1.578071862	6.006431825
90 to 94	0.232813142	2.179863636	15141.74181	0.458372994	3.928760338	0.212446812	2.246273548	21851.47823	0.689976509	4.225336216
95 plus	0.327557459	3.054273651	4541.015085	0.139318549	3.054273651	0.308397906	3.243308611	7227.456884	0.234844074	3.243308611

Table 15: Oman 2100 life table, by age and sex. mx=mortality rate, ax=mean person-years lived in an age interval among those who die in that age interval, lx=number of persons left alive at age x, nLx=person-years lived between age x and x+n, ex=life expectancy at age x.

Age Group	Male					Female				
	mx	ax	lx	nLx	ex	mx	ax	lx	nLx	ex
Early Neonatal	0.060340885	0.009587192	100000	0.019166994	83.78861537	0.048207751	0.009587564	100000	0.019169222	86.33312269
Late Neonatal	0.005179607	0.028765695	99884.40692	0.057459181	83.86637676	0.006243653	0.028765401	99907.62863	0.057470781	86.39371291
Post Neonatal	0.000611475	0.461600398	99854.65463	0.921685591	83.83379266	0.000617174	0.461599993	99871.75775	0.921841039	86.36718936
1 to 4	0.000118425	1.999842101	99798.31788	3.990987539	82.9575884	8.7027E-05	1.999883964	99814.88887	3.991901003	85.49277289
5 to 9	9.22069E-05	2.499807902	99751.06553	4.986403816	78.99599714	4.71117E-05	2.499901851	99780.16469	4.988420774	81.52182905
10 to 14	0.000170336	2.892884874	99705.09137	4.983394154	74.03124287	6.46445E-05	2.89405672	99756.66745	4.987137301	76.54043488
15 to 19	0.000406464	2.909762226	99620.23515	4.976359719	69.09177765	0.000152898	2.63088765	99724.43258	4.984406745	71.56424855
20 to 24	0.000587838	2.579140923	99418.28835	4.963790989	64.2265414	0.000151616	2.55725909	99648.23103	4.980567265	66.61696718
25 to 29	0.000531374	2.510973291	99126.68766	4.949786883	59.40781311	0.000195052	2.606118306	99572.72166	4.976310304	61.66554152
30 to 34	0.00061261	2.545830627	98863.72077	4.935766979	54.55899757	0.0002491	2.620668814	99475.66525	4.970834612	56.7231106
35 to 39	0.000678756	2.57154833	98561.42195	4.919960934	49.7183193	0.000337397	2.670837351	99351.85282	4.963687625	51.79043342
40 to 44	0.000862406	2.628797038	98227.56485	4.901345393	44.87825701	0.000522649	2.736272296	99184.39938	4.953353863	46.87316481
45 to 49	0.001234463	2.669420959	97805.04383	4.876228553	40.06011853	0.000927908	2.713805988	98925.5532	4.935800002	41.98833188
50 to 54	0.001938326	2.704142454	97203.36186	4.838647294	35.29011732	0.00147639	2.689645532	98467.67653	4.906643377	37.17027833
55 to 59	0.003291771	2.73887625	96266.19894	4.777771671	30.60430581	0.002288355	2.683841029	97743.58607	4.861412276	32.42466277
60 to 64	0.006072632	2.72051677	94695.91878	4.670223695	26.06075328	0.00354123	2.67435804	96632.03973	4.79212566	27.7650635
65 to 69	0.010473087	2.643526364	91869.68532	4.483419494	21.76725074	0.005333985	2.717607275	94937.56661	4.689758142	23.21043363
70 to 74	0.014875357	2.648722999	87204.93419	4.214548275	17.77580369	0.009382964	2.765472488	92440.97955	4.527328811	18.76140771
75 to 79	0.024405293	2.674967145	81026.42158	3.837619578	13.90781032	0.018674483	2.799209438	88205.15226	4.23651777	14.52460026
80 to 84	0.045074557	2.594001811	71864.83723	3.255069981	10.32114172	0.041881043	2.70430621	80344.31782	3.667984373	10.66349017
85 to 89	0.091296485	2.556425698	57793.7499	2.387419937	7.169957382	0.085451841	2.597452506	65173.60196	2.712856288	7.493921413
90 to 94	0.180913956	2.238445072	37283.2004	1.266815023	4.788111195	0.164914298	2.327286189	42497.05617	1.486292475	5.124566192
95 plus	0.284312453	3.546527631	15617.57964	0.574743701	3.546527631	0.264049491	3.807477784	18585.11671	0.721302984	3.807477784

Table 15: Qatar 2017 life table, by age and sex. mx=mortality rate, ax=mean person-years lived in an age interval among those who die in that age interval, lx=number of persons left alive at age x, nLx=person-years lived between age x and x+n, ex=life expectancy at age x.

Age Group	Male					Female				
	mx	ax	lx	nLx	ex	mx	ax	lx	nLx	ex
Early Neonatal	0.133024649	0.009584964	100000	0.01915364	79.15497562	0.118121581	0.009585421	100000	0.019156376	80.99851397
Late Neonatal	0.018878634	0.028761916	99745.2193	0.057356508	79.33789597	0.014018551	0.028763256	99773.72775	0.057380921	81.16298127
Post Neonatal	0.002325421	0.461478642	99636.94676	0.9189489	79.36652584	0.002044447	0.461498602	99693.29297	0.919587756	81.17090337
1 to 4	0.000296728	1.999604363	99423.28022	3.974572335	78.61279874	0.000243813	1.999674916	99505.30287	3.97827205	80.40009439
5 to 9	0.000235471	2.499509435	99305.3607	4.962346498	74.70376347	0.000186188	2.499612109	99408.31586	4.968103038	76.47658254
10 to 14	0.000293637	2.827098845	99188.52228	4.956265309	69.78881369	0.000140791	2.52669421	99315.82006	4.96406326	71.54548081
15 to 19	0.000699811	2.614350936	99042.99654	4.943898218	64.88713822	0.000205316	2.562003726	99245.93238	4.95981461	66.59404938
20 to 24	0.00068268	2.491195982	98697.03534	4.926414386	60.10507942	0.000202841	2.479123232	99144.10115	4.954671972	61.65973371
25 to 29	0.000675225	2.467962636	98360.7475	4.909643907	55.30173912	0.000185724	2.57210771	99043.60295	4.949948328	56.71969686
30 to 34	0.000582584	2.496579807	98029.27507	4.894327378	50.48010389	0.000267719	2.652396272	98951.67453	4.944476856	51.76991414
35 to 39	0.00067055	2.611399907	97744.18257	4.879396513	45.61981705	0.000382932	2.684170327	98819.30845	4.936587915	46.83558691
40 to 44	0.000947772	2.66054852	97417.06032	4.860080231	40.76394016	0.000608109	2.719298903	98630.28507	4.924683839	41.92000919
45 to 49	0.001412207	2.775088153	96956.55643	4.832647466	35.94449554	0.001026955	2.889131032	98330.84221	4.905906178	37.03910477
50 to 54	0.002846111	2.763703873	96274.32421	4.783281219	31.17893784	0.002547907	2.807528066	97827.10351	4.864186409	32.21451273
55 to 59	0.005122969	2.771051138	94913.60417	4.692125272	26.58520189	0.00488266	2.750665317	96588.04398	4.776954356	27.59078554
60 to 64	0.009910844	2.739767018	92511.52747	4.524315471	22.20186429	0.008753833	2.707692852	94256.59897	4.620181389	23.20356094
65 to 69	0.017897553	2.656255378	88032.25674	4.224677663	18.1896588	0.014593654	2.662301722	90215.16394	4.362130797	19.11928431
70 to 74	0.026849209	2.616277574	80484.16452	3.782745123	14.64343701	0.022638524	2.63278232	83857.80708	3.980074165	15.36373845
75 to 79	0.041317066	2.610962835	70356.67072	3.202968062	11.37281516	0.03501334	2.657156165	74869.24191	3.460613628	11.88916624
80 to 84	0.070369756	2.576482002	57180.94233	2.444558055	8.393150703	0.067105049	2.588261774	62800.87398	2.704826169	8.662248895
85 to 89	0.127660659	2.441691423	40080.13554	1.513093268	5.883414848	0.122066929	2.467565481	44759.15788	1.712517148	6.116098645
90 to 94	0.222663445	2.198237489	20883.92409	0.644421911	4.066327023	0.208523259	2.25431632	24004.17939	0.765298366	4.290019347
95 plus	0.319321422	3.132935212	6607.804631	0.207738285	3.132935212	0.304794257	3.28364056	8152.74901	0.269078445	3.28364056

Table 15: Qatar 2100 life table, by age and sex. mx=mortality rate, ax=mean person-years lived in an age interval among those who die in that age interval, lx=number of persons left alive at age x, nLx=person-years lived between age x and x+n, ex=life expectancy at age x.

Age Group	Male					Female				
	mx	ax	lx	nLx	ex	mx	ax	lx	nLx	ex
Early Neonatal	0.024231241	0.009588298	100000	0.019173627	85.59311797	0.021232526	0.00958839	100000	0.019174178	86.76475802
Late Neonatal	0.00337995	0.028766191	99953.54084	0.057501926	85.61371787	0.002692812	0.02876638	99959.2891	0.057506369	86.78091537
Post Neonatal	0.00039966	0.461615444	99934.10587	0.922509068	85.5728184	0.000364696	0.461617928	99943.80391	0.922613482	86.73682374
1 to 4	5.82823E-05	1.99992229	99897.23774	3.995423777	84.68093438	5.32092E-05	1.999929054	99910.15716	3.995981037	85.84259049
5 to 9	6.41816E-05	2.499866289	99873.95215	4.992896518	80.70020886	6.02681E-05	2.499874442	99888.89555	4.993692343	81.86043826
10 to 14	9.80791E-05	3.072968379	99841.91194	4.991151456	75.7251858	5.51359E-05	2.592145048	99858.79976	4.992276694	76.88435134
15 to 19	0.000337225	2.689753707	99792.96959	4.985690035	70.76073422	8.41859E-05	2.534658137	99831.27513	4.990530808	71.90482496
20 to 24	0.00033183	2.500537396	99625.0217	4.977117342	65.87517077	7.18384E-05	2.471515732	99789.26485	4.988555394	66.93401803
25 to 29	0.000326926	2.455748104	99460.05609	4.968871385	60.9800534	7.26675E-05	2.607901055	99753.43218	4.986801381	61.95715451
30 to 34	0.00025645	2.482801505	99297.92915	4.961669065	56.07546386	0.000106223	2.64548066	99717.19711	4.984611027	56.97870314
35 to 39	0.000277661	2.592981629	99170.87405	4.955242386	51.14407916	0.000144799	2.669801822	99664.25303	4.981533265	52.00753584
40 to 44	0.000387496	2.665028611	99033.4834	4.94718929	46.211314	0.000225779	2.766578127	99592.12651	4.977093806	47.0431692
45 to 49	0.000570459	2.81404125	98842.15646	4.935923042	41.29528351	0.000431534	2.924009787	99479.76966	4.969505952	42.09291689
50 to 54	0.001215738	2.805759365	98561.1156	4.914858155	36.40433223	0.001077871	2.806098627	99265.37002	4.951559572	37.17694158
55 to 59	0.002275345	2.787483478	97964.632	4.873751009	31.60611941	0.00202967	2.748764455	98731.83239	4.914165887	32.3614073
60 to 64	0.004469187	2.813038958	96857.65262	4.795851583	26.93088478	0.003571987	2.744204798	97735.2673	4.847668422	27.66065128
65 to 69	0.009023453	2.708589984	94722.71853	4.640723106	22.46195543	0.006290005	2.71301562	96007.30025	4.732268494	23.10414816
70 to 74	0.014547714	2.677134927	90564.24465	4.381736024	18.35153077	0.010261965	2.736399062	93040.70335	4.546519214	18.74863415
75 to 79	0.022841099	2.681880043	84310.00971	4.006912498	14.49295609	0.019051685	2.802878112	88399.90215	4.242425737	14.5824875
80 to 84	0.040090782	2.674871815	75387.94119	3.459268589	10.87971396	0.043636342	2.700606518	80391.34871	3.658898994	10.75273278
85 to 89	0.083179709	2.583927309	62165.9469	2.618751068	7.559846207	0.086808559	2.598836982	64881.2041	2.710008381	7.582517673
90 to 94	0.170231166	2.235090866	42002.422	1.461904524	5.019771475	0.165091889	2.3162382	42775.11408	1.514427542	5.192334728
95 plus	0.27512099	3.67953522	18891.66938	0.729002496	3.67953522	0.263835781	3.851869812	19450.93398	0.786940035	3.851869812

Table 15: Saudi Arabia 2017 life table, by age and sex. mx=mortality rate, ax=mean person-years lived in an age interval among those who die in that age interval, lx=number of persons left alive at age x, nLx=person-years lived between age x and x+n, ex=life expectancy at age x.

Age Group	Male					Female				
	mx	ax	lx	nLx	ex	mx	ax	lx	nLx	ex
Early Neonatal	0.186990753	0.00958331	100000	0.019143737	74.96730821	0.159588359	0.00958415	100000	0.019148765	79.32220254
Late Neonatal	0.026777321	0.028759737	99642.05614	0.057284176	75.21722447	0.024857285	0.028760266	99694.42741	0.057317448	79.54598523
Post Neonatal	0.002607652	0.461458592	99488.69176	0.9174622	75.27554399	0.002831386	0.461442699	99551.97403	0.917950996	79.60219175
1 to 4	0.000412672	1.999449771	99249.51069	3.966706504	74.53249952	0.000405378	1.999459496	99292.12799	3.968467486	78.88596853
5 to 9	0.000193968	2.4995959	99085.8623	4.951891719	70.65226498	0.000169196	2.499647508	99131.29198	4.954468843	75.01070537
10 to 14	0.000225021	3.078943695	98989.82208	4.947353255	65.71839127	0.0001832	2.767779175	99047.47363	4.950349593	70.07204987
15 to 19	0.000822046	2.774786718	98878.50916	4.934898426	60.78888416	0.00040554	2.656278464	98956.79105	4.94314194	65.13370922
20 to 24	0.001316062	2.572708595	98472.88153	4.907972605	56.0277043	0.000489052	2.568729094	98756.34409	4.931957366	60.26045373
25 to 29	0.001300051	2.508467578	97827.01264	4.875563831	51.38029021	0.000573371	2.597419554	98515.15214	4.918986319	55.40157857
30 to 34	0.001390287	2.558424888	97193.26481	4.84322463	46.69861214	0.000765587	2.639720959	98233.12438	4.902798598	50.5530008
35 to 39	0.001709429	2.618389609	96520.08638	4.806441264	42.00614567	0.001094535	2.667423928	97857.81765	4.880426374	45.73650989
40 to 44	0.002395188	2.662873145	95698.73035	4.75831119	37.34387426	0.001652829	2.680444526	97323.7671	4.847599128	40.97256273
45 to 49	0.003652897	2.67713218	94559.54588	4.688218903	32.7613175	0.002549433	2.679094975	96522.81803	4.797752947	36.29001044
50 to 54	0.005662407	2.676618853	92848.13357	4.582156685	28.31546079	0.003911562	2.675539187	95300.20755	4.722086915	31.72091232
55 to 59	0.008821956	2.670046442	90256.0291	4.42197032	24.05156887	0.006019261	2.67603624	93454.1508	4.608271335	27.29436397
60 to 64	0.013768479	2.656281603	86359.35976	4.18310756	20.01639873	0.009430874	2.669195107	90682.12139	4.436636566	23.04664858
65 to 69	0.021400747	2.644362472	80606.16782	3.837081475	16.25551344	0.014742933	2.660695325	86501.08998	4.180957905	19.03137039
70 to 74	0.034169365	2.625354863	72404.2006	3.348821942	12.79755841	0.023458245	2.648155625	80342.04524	3.807229586	15.28624079
75 to 79	0.056170491	2.595442986	60976.32159	2.686441809	9.704569447	0.038216837	2.62376827	71418.84197	3.273942807	11.86523218
80 to 84	0.099691215	2.523525923	45906.08828	1.841356253	7.039271787	0.064232076	2.57055194	58920.24233	2.548867425	8.825585451
85 to 89	0.163409311	2.343679636	27575.05918	0.961920069	5.043445052	0.118123383	2.479586785	42573.50319	1.641058094	6.228174727
90 to 94	0.258188709	2.129796173	11880.96478	0.341418692	3.615659929	0.204209911	2.263666582	23225.57137	0.745519851	4.355588973
95 plus	0.347842676	2.875149449	3077.453745	0.088560967	2.875149449	0.300880181	3.324390816	8028.70243	0.267266257	3.324390816

Table 15: Saudi Arabia 2100 life table, by age and sex. mx=mortality rate, ax=mean person-years lived in an age interval among those who die in that age interval, lx=number of persons left alive at age x, nLx=person-years lived between age x and x+n, ex=life expectancy at age x.										
**Age Group**	**Male**					**Female**				
	**mx**	**ax**	**lx**	**nLx**	**ex**	**mx**	**ax**	**lx**	**nLx**	**ex**
Early Neonatal	0.027325963	0.009588204	100000	0.019173058	80.59958612	0.029031958	0.009588151	100000	0.019172745	84.67622428
Late Neonatal	0.003887385	0.028766051	99947.61171	0.057497676	80.62271791	0.004824022	0.028765793	99944.34246	0.057494246	84.70418887
Post Neonatal	0.00036479	0.461617922	99925.26251	0.922442283	80.58325068	0.000470824	0.461610389	99916.6082	0.922317245	84.67015583
1 to 4	6.36817E-05	1.999915091	99891.61413	3.995155727	79.68695072	6.651E-05	1.99991132	99873.18452	3.994396049	83.7834705
5 to 9	4.16959E-05	2.499913134	99866.17354	4.992788216	75.70672052	4.7066E-05	2.499901946	99846.61945	4.991743628	79.80522078
10 to 14	7.0534E-05	3.674155593	99845.35595	4.991784349	70.72194305	7.3613E-05	3.029421925	99823.12673	4.99042254	74.82340428
15 to 19	0.000436131	2.80384071	99810.14782	4.985709002	65.74556691	0.000224771	2.660342932	99786.39272	4.986691101	69.84982659
20 to 24	0.000703718	2.579397539	99592.7324	4.971172925	60.88244242	0.000240908	2.573673988	99674.31411	4.980801274	64.92535801
25 to 29	0.00071437	2.512092306	99243.06485	4.953362465	56.08691986	0.000307472	2.623332771	99554.33346	4.974079778	60.00047907
30 to 34	0.00075603	2.557059138	98889.54294	4.935341009	51.27735777	0.000421625	2.640595	99401.41437	4.965122075	55.08866399
35 to 39	0.000899509	2.625984281	98516.90542	4.91533934	46.46071541	0.000583978	2.667098517	99192.11424	4.952860378	50.19919115
40 to 44	0.001298147	2.669007315	98075.43364	4.888967298	41.65697392	0.000895141	2.716632746	98902.94262	4.93506081	45.33777663
45 to 49	0.00196565	2.681886114	97441.8502	4.850024872	36.90886978	0.001523477	2.693510532	98461.34343	4.905830266	40.52819113
50 to 54	0.003098152	2.705710263	96490.50608	4.790518539	32.24344915	0.002330009	2.667880572	97714.41564	4.859318928	35.81600596
55 to 59	0.005180364	2.673124491	95010.86442	4.694154425	27.69855689	0.003442478	2.654199772	96583.39763	4.790469849	31.20226809
60 to 64	0.007763325	2.660895914	92590.67593	4.547415328	23.34408857	0.0049335	2.651115801	94937.14361	4.692566114	26.69513879
65 to 69	0.011942044	2.685261986	89087.20986	4.335396659	19.14384747	0.007283319	2.691835213	92627.09288	4.554827751	22.29149942
70 to 74	0.019980736	2.717368535	83971.23668	4.017879167	15.13140451	0.012153855	2.736882723	89319.94218	4.346850124	18.01438753
75 to 79	0.039077448	2.686308231	76077.74762	3.495718463	11.3984026	0.02340153	2.724579039	84056.99147	3.991411185	13.96554108
80 to 84	0.077796313	2.600611339	62796.72078	2.666812537	8.21525249	0.045014682	2.639361006	74784.98507	3.383799111	10.35308117
85 to 89	0.135674663	2.423651459	43155.77204	1.629264926	5.778534937	0.090180369	2.57907261	59748.38387	2.460526042	7.279350679
90 to 94	0.22959155	2.179703952	22590.665	0.704025029	4.012844483	0.170928563	2.320111095	38008.89335	1.312721709	4.990877878
95 plus	0.324563121	3.102258551	7408.799639	0.239642829	3.102258551	0.26976528	3.723067656	16055.99391	0.607741329	3.723067656

Table 15: Sudan 2017 life table, by age and sex. mx=mortality rate, ax=mean person-years lived in an age interval among those who die in that age interval, lx=number of persons left alive at age x, nLx=person-years lived between age x and x+n, ex=life expectancy at age x.

Age Group	Male					Female				
	mx	ax	lx	nLx	ex	mx	ax	lx	nLx	ex
Early Neonatal	1.160992397	0.009553457	100000	0.018966169	68.60484533	0.773887946	0.009565322	100000	0.019036473	71.7915703
Late Neonatal	0.104090128	0.02873841	97798.30676	0.056099411	70.1293966	0.098550111	0.028739938	98526.90525	0.056526337	72.84536621
Post Neonatal	0.019149919	0.460283466	97214.52032	0.889686632	70.49280566	0.018088887	0.460358838	97969.94124	0.897036259	73.20179083
1 to 4	0.003247186	1.995670432	95512.23688	3.795807213	70.8180423	0.002814649	1.996247142	96348.31245	3.832334788	73.50282762
5 to 9	0.000783127	2.498368486	94280.83457	4.70482919	67.71783093	0.000563884	2.498825243	95270.48459	4.756817588	70.31244424
10 to 14	0.000598402	2.569478156	93912.57458	4.688810748	62.97386908	0.000448929	2.560864672	95002.34571	4.744924022	65.50373675
15 to 19	0.000988432	2.67890016	93632.06869	4.67089026	58.15482543	0.000699602	2.637567318	94789.37879	4.731652879	60.64505057
20 to 24	0.001461858	2.586220359	93170.36874	4.642138867	53.42920202	0.000918963	2.613566976	94458.40319	4.712587468	55.84793069
25 to 29	0.001612069	2.559175169	92491.78744	4.606466804	48.80143018	0.001209556	2.62983771	94025.43816	4.687836494	51.09250113
30 to 34	0.001948661	2.584112831	91749.30555	4.565978366	44.1748385	0.001689998	2.634085795	93458.57411	4.654326707	46.38576619
35 to 39	0.002443322	2.609294356	90859.77431	4.516623782	39.58106883	0.002331561	2.623942472	92672.23907	4.608101548	41.75596224
40 to 44	0.003307527	2.644111873	89756.63495	4.453162882	35.03403864	0.003144179	2.628287147	91598.31362	4.546043519	37.21324246
45 to 49	0.004876939	2.664985526	88284.59949	4.364586538	30.57194116	0.004389216	2.643730864	90169.86793	4.462394746	32.7588448
50 to 54	0.007478357	2.671477735	86158.09005	4.234287237	26.25745907	0.006410758	2.639362742	88213.23203	4.345005951	28.42392429
55 to 59	0.0117292	2.674036399	82996.79089	4.0398724	22.15121575	0.009134169	2.653211865	85432.50758	4.182160306	24.25948076
60 to 64	0.018990014	2.643443058	78270.97912	3.746476234	18.32171085	0.014135305	2.651053378	81621.89069	3.950331029	20.26445707
65 to 69	0.028905275	2.615107999	71184.16828	3.330813357	14.87733693	0.021725076	2.642042829	76056.99845	3.61830911	16.54967576
70 to 74	0.044670738	2.580337112	61611.53308	2.782158219	11.77975985	0.034664287	2.615635354	68234.15723	3.152673599	13.14204868
75 to 79	0.068323091	2.533457097	49282.72711	2.111945841	9.084726305	0.054896089	2.573327263	57374.76038	2.533812777	10.13615947
80 to 84	0.107257758	2.459975478	35005.26881	1.379421214	6.772176749	0.088586319	2.506385018	43575.60309	1.787728212	7.540000931
85 to 89	0.171254641	2.324222414	20398.41034	0.702418344	4.894770899	0.14942491	2.388035858	27890.43475	1.006013565	5.391550539
90 to 94	0.265559063	2.114250063	8517.226581	0.242283425	3.53601925	0.237907056	2.189292846	13003.31375	0.391134236	3.867218696
95 plus	0.35361141	2.829468808	2145.769215	0.0611222	2.829468808	0.331210963	3.02136797	3775.579536	0.114782685	3.02136797

Table 15: Sudan 2100 life table, by age and sex. mx=mortality rate, ax=mean person-years lived in an age interval among those who die in that age interval, lx=number of persons left alive at age x, nLx=person-years lived between age x and x+n, ex=life expectancy at age x.

Age Group	Male					Female				
	mx	ax	lx	nLx	ex	mx	ax	lx	nLx	ex
Early Neonatal	0.226704879	0.009582093	100000	0.019136456	82.93899412	0.138405527	0.009584799	100000	0.019152654	86.37862154
Late Neonatal	0.017318356	0.028762346	99566.24141	0.057256163	83.28070093	0.019498573	0.028761745	99734.95141	0.057349584	86.58855813
Post Neonatal	0.002733923	0.461449622	99467.10323	0.917209695	83.3060707	0.002803322	0.461444692	99623.14818	0.918619247	86.6280305
1 to 4	0.000316492	1.999578011	99216.42182	3.966146361	82.5919916	0.000301366	1.999598179	99365.71231	3.972234488	85.92768903
5 to 9	0.000142877	2.49970234	99090.92468	4.952777232	78.6939363	0.000135478	2.499717754	99246.039	4.960622161	82.02877089
10 to 14	0.000170535	2.80602067	99020.17367	4.949159522	73.74830908	0.000153322	2.627190093	99178.85578	4.957137981	77.08259773
15 to 19	0.000396295	2.741858549	98935.7868	4.942374086	68.80874939	0.0002275	2.626575538	99102.87674	4.952466902	72.1396413
20 to 24	0.000638767	2.605112283	98739.99098	4.929453122	63.93955599	0.000288277	2.675229394	98990.2529	4.946196812	67.2186661
25 to 29	0.000710904	2.562160221	98425.37652	4.912745478	59.13539159	0.000468995	2.688359197	98847.71646	4.937032602	62.31167347
30 to 34	0.000838726	2.555570457	98076.49231	4.893788131	54.33655705	0.000712415	2.634405931	98616.25527	4.922508875	57.45148114
35 to 39	0.000927833	2.566900487	97666.47434	4.872322527	49.55388799	0.00092231	2.6035014	98265.73058	4.902443905	52.64684249
40 to 44	0.001132571	2.60356884	97214.88718	4.847583104	44.77205755	0.001166918	2.610459417	97813.83968	4.877084031	47.87778775
45 to 49	0.001488078	2.645043481	96666.39479	4.816442618	40.0110961	0.00153909	2.614898495	97245.08378	4.84446936	43.14223525
50 to 54	0.002181996	2.689252306	95950.30163	4.773448069	35.2894341	0.002029455	2.607289977	96499.93965	4.801685885	38.45462779
55 to 59	0.003511039	2.688363601	94909.70984	4.707288786	30.645868	0.002616764	2.620145837	95526.17217	4.746751444	33.81922081
60 to 64	0.005478042	2.674293496	93258.68105	4.604345762	26.13892399	0.003594885	2.636863687	94285.14481	4.674599996	29.22878405
65 to 69	0.008450033	2.689682331	90740.16271	4.450249907	21.78697599	0.005144897	2.667187983	92606.4878	4.575472497	24.70908411
70 to 74	0.014019791	2.705351582	86989.29336	4.214295336	17.60573503	0.008017784	2.730287656	90257.05357	4.432333486	20.28026121
75 to 79	0.024917946	2.690572168	81104.29239	3.835726676	13.68154453	0.014887331	2.754650147	86714.09447	4.195826423	15.99347448
80 to 84	0.045047785	2.611823757	71614.69052	3.236517795	10.13237969	0.029382513	2.683295923	80498.36361	3.770094317	12.01077701
85 to 89	0.092474827	2.550221427	57208.67995	2.338952253	7.021567639	0.06530015	2.678422398	69508.21861	3.022111865	8.477391482
90 to 94	0.183689462	2.24802609	35931.40078	1.199608732	4.694579365	0.138574719	2.352904387	50009.20284	1.836004399	5.744625583
95 plus	0.286905827	3.492663034	14228.58263	0.502166308	3.492663034	0.238864315	4.199808429	24905.20831	1.056422631	4.199808429

Table 15: Syria 2017 life table, by age and sex. mx=mortality rate, ax=mean person-years lived in an age interval among those who die in that age interval, lx=number of persons left alive at age x, nLx=person-years lived between age x and x+n, ex=life expectancy at age x.

Age Group	Male					Female				
	mx	ax	lx	nLx	ex	mx	ax	lx	nLx	ex
Early Neonatal	0.238254403	0.009581739	100000	0.019134335	65.02572559	0.203592761	0.009582801	100000	0.019140691	74.68431465
Late Neonatal	0.025616932	0.028760057	99544.13412	0.057229787	65.3042491	0.026033315	0.028759942	99610.31794	0.057267149	74.95724877
Post Neonatal	0.003901141	0.461366705	99397.54133	0.916074522	65.34298053	0.004487355	0.461325061	99461.23918	0.916413662	75.01202107
1 to 4	0.002655307	1.996459598	99040.18604	3.940643979	64.6537889	0.003233685	1.995688433	99050.01926	3.936487578	74.39821643
5 to 9	0.001471893	2.496933559	97993.86739	4.881708436	61.32281574	0.001377151	2.497130937	97777.10494	4.872062184	71.34078731
10 to 14	0.001627422	2.905889555	97275.35175	4.847248302	56.75733399	0.001299303	2.584803708	97106.15246	4.840119313	66.81645496
15 to 19	0.004723296	2.80885437	96486.51411	4.774907328	52.19751457	0.001930868	2.569404677	96477.27696	4.801331061	62.23511213
20 to 24	0.008961174	2.560030155	94231.20404	4.610746895	48.37918864	0.00197311	2.474640476	95550.20728	4.753824091	57.81387603
25 to 29	0.007803173	2.416021846	90099.46682	4.415936075	45.47993815	0.00172684	2.483026541	94612.23327	4.710142423	53.36236496
30 to 34	0.006436881	2.410636134	86653.69725	4.261657881	42.19188875	0.001869915	2.531170407	93798.88648	4.668397248	48.80338302
35 to 39	0.005486434	2.443544758	83910.61264	4.13750795	38.4918435	0.002045504	2.554145368	92925.98661	4.623176553	44.2378262
40 to 44	0.005288169	2.510808581	81640.77891	4.029023895	34.49335097	0.002451366	2.60740868	91980.43766	4.572211992	39.66604854
45 to 49	0.006089477	2.599490885	79510.57695	3.918288985	30.34908019	0.003381291	2.640849708	90859.88334	4.507053437	35.12274444
50 to 54	0.008674637	2.639227383	77125.54387	3.778954923	26.20549484	0.004871898	2.645367866	89336.43009	4.416189909	30.67625613
55 to 59	0.012780554	2.652832045	73850.07892	3.585087595	22.2481676	0.007060794	2.656078598	87185.98696	4.28837791	26.36708145
60 to 64	0.020086297	2.616660428	69274.15633	3.305751541	18.53950299	0.010769181	2.656942019	84160.38184	4.104540176	22.21891975
65 to 69	0.028309731	2.589534119	62647.46888	2.932826242	15.2206319	0.016542583	2.650834749	79744.96765	3.838254767	18.30173598
70 to 74	0.041332357	2.576623423	54370.3093	2.472016532	12.14158432	0.02602454	2.644816854	73404.27219	3.458510611	14.6541125
75 to 79	0.063022885	2.553176955	44200.36387	1.916436908	9.343402281	0.043074854	2.608912213	64417.44805	2.920434599	11.33091639
80 to 84	0.104137815	2.499274329	32203.34715	1.27977183	6.880941445	0.070506629	2.530137429	51852.30216	2.208372519	8.445681236
85 to 89	0.16821764	2.331712207	18988.21799	0.657138918	4.95170326	0.126389419	2.453638891	36293.8731	1.373230647	5.981506403
90 to 94	0.26271293	2.120265484	8025.465865	0.229191173	3.566527721	0.213374407	2.244321402	18956.4548	0.597138767	4.210683067
95 plus	0.351386607	2.846972715	2043.659485	0.058441557	2.846972715	0.309243985	3.234186308	6229.090831	0.20163736	3.234186308

Table 15: Syria 2100 life table, by age and sex. mx=mortality rate, ax=mean person-years lived in an age interval among those who die in that age interval, lx=number of persons left alive at age x, nLx=person-years lived between age x and x+n, ex=life expectancy at age x.

Age Group	Male					Female				
	mx	ax	lx	nLx	ex	mx	ax	lx	nLx	ex
Early Neonatal	0.061523655	0.009587155	100000	0.019166775	76.08379591	0.072591177	0.009586816	100000	0.019164742	83.42766991
Late Neonatal	0.007143916	0.028765153	99882.1231	0.057454623	76.15192129	0.010942693	0.028764105	99860.9308	0.057436161	83.52240054
Post Neonatal	0.001025214	0.461571006	99841.09477	0.92138458	76.12455534	0.001696603	0.461523312	99798.11654	0.920703112	83.51634419
1 to 4	0.001548177	1.99793577	99746.68516	3.977568413	75.26959265	0.002127449	1.997163415	99642.09102	3.968837822	82.71981551
5 to 9	0.000893654	2.498138225	99132.88949	4.945648895	71.69309629	0.000984609	2.497948736	98801.99628	4.928056783	79.38879498
10 to 14	0.001040203	2.99973447	98693.68387	4.924430163	66.98046157	0.000951763	2.729706329	98321.03139	4.904932657	74.75755316
15 to 19	0.003368595	2.806067524	98185.56033	4.874158673	62.2910309	0.001419542	2.593611385	97859.48334	4.876330077	70.09267329
20 to 24	0.006642822	2.579233155	96570.10208	4.75407411	58.22423267	0.001392575	2.474755671	97177.01987	4.841984173	65.5621043
25 to 29	0.00567211	2.437038251	93521.49064	4.611694337	54.96968098	0.001122918	2.477182574	96515.07332	4.812228662	60.99327052
30 to 34	0.004493727	2.430162025	91041.72111	4.502595107	51.39478878	0.001124989	2.532031892	95985.32523	4.786069042	56.31647118
35 to 39	0.0035148	2.471328697	89148.12933	4.419974279	47.45855251	0.001113101	2.564495176	95458.40365	4.760048366	51.6146875
40 to 44	0.003101939	2.52714607	87704.17643	4.353349031	43.2285924	0.001221018	2.638571653	94940.26214	4.733358203	46.88434502
45 to 49	0.002943739	2.607484797	86451.44263	4.293481581	38.84701929	0.001640917	2.66085932	94374.11438	4.70076699	42.15139987
50 to 54	0.003731225	2.64922965	85267.21398	4.227653893	34.37368726	0.002284146	2.660128013	93616.55391	4.656147872	37.47268832
55 to 59	0.005205469	2.653292342	83765.25056	4.139364001	29.9605143	0.003236065	2.651278855	92570.27061	4.593848166	32.86808205
60 to 64	0.00742469	2.627560887	81694.32067	4.016156558	25.66952435	0.004507543	2.651982073	91103.62565	4.507994576	28.35617069
65 to 69	0.010072903	2.636213673	78810.46875	3.851190283	21.52393266	0.006686342	2.683770128	89096.97802	4.387398891	23.93605247
70 to 74	0.01488441	2.663784076	75040.02062	3.62917268	17.48365066	0.010544404	2.707249556	86200.1805	4.209020761	19.65150959
75 to 79	0.024410229	2.705214102	69787.59604	3.308908344	13.60664066	0.018364539	2.697005478	81807.96736	3.925735612	15.56055634
80 to 84	0.047563411	2.655328847	61938.25684	2.796334391	9.998719078	0.032043227	2.602926058	74678.97893	3.472584101	11.78652706
85 to 89	0.095918814	2.54011522	49105.70273	2.002539894	6.931457131	0.069183543	2.664146091	63777.0277	2.756153657	8.330246686
90 to 94	0.187220043	2.241063754	30696.08482	1.024945819	4.646947759	0.143230366	2.344402999	45314.7737	1.65737453	5.657210153
95 plus	0.289786226	3.465450928	12189.1441	0.432549483	3.465450928	0.243235507	4.145026832	22393.88244	0.951494955	4.145026832

Table 15: Tunisia 2017 life table, by age and sex. mx=mortality rate, ax=mean person-years lived in an age interval among those who die in that age interval, lx=number of persons left alive at age x, nLx=person-years lived between age x and x+n, ex=life expectancy at age x.

Age Group	Male					Female				
	mx	ax	lx	nLx	ex	mx	ax	lx	nLx	ex
Early Neonatal	0.17339191	0.009583727	100000	0.019146231	75.85887095	0.213316146	0.009582503	100000	0.019138908	80.44464495
Late Neonatal	0.045492097	0.028754574	99668.02918	0.057268277	76.09228114	0.04022325	0.028756028	99591.74729	0.057233115	80.75517189
Post Neonatal	0.003749742	0.46137746	99407.52397	0.916230643	76.23405948	0.001870297	0.461510973	99361.55323	0.916601409	80.88466402
1 to 4	0.00052253	1.999303294	99064.00356	3.95842254	75.57352712	0.000279011	1.999627985	99190.13716	3.965392515	80.10037099
5 to 9	0.000241355	2.499497178	98857.19651	4.939878883	71.72745304	0.000194609	2.499594564	99079.5094	4.951566146	76.18758372
10 to 14	0.000257945	2.738020648	98737.98296	4.934022087	66.8110374	0.000184681	2.571866098	98983.15214	4.946940864	71.25932339
15 to 19	0.00053889	2.707344064	98610.72161	4.924454317	61.89363879	0.000260284	2.607852533	98891.79415	4.941514309	66.32270611
20 to 24	0.000799486	2.603634448	98345.36123	4.907864486	57.05290148	0.000321364	2.580516698	98763.17778	4.934323022	61.40550522
25 to 29	0.000940931	2.58102819	97953.02182	4.886529379	52.27038978	0.000386091	2.622127096	98604.61433	4.925709039	56.49987905
30 to 34	0.001173989	2.567579639	97493.30484	4.860789291	47.50406774	0.000549674	2.640783965	98414.45275	4.914352222	51.6037097
35 to 39	0.001336757	2.601776081	96922.78345	4.830661519	42.76783426	0.000762688	2.657921941	98144.35576	4.898470006	46.73808006
40 to 44	0.001852462	2.665373186	96277.26617	4.793146865	38.03624776	0.001135535	2.656596533	97770.82952	4.87556933	41.90590202
45 to 49	0.002853604	2.689800745	95389.81129	4.738276439	33.36408301	0.001629217	2.670421872	97217.35294	4.842494145	37.12855829
50 to 54	0.004555854	2.712667082	94038.72811	4.653488558	28.80297935	0.002499596	2.710843006	96428.73773	4.794020825	32.40931226
55 to 59	0.0077828	2.695234768	91921.05546	4.515172199	24.40149422	0.004244664	2.715338729	95231.15952	4.71586327	27.78130149
60 to 64	0.01258742	2.673465526	88412.86727	4.295132476	20.25925763	0.007122796	2.707498049	93231.31512	4.586768619	23.31669073
65 to 69	0.020214403	2.650928356	83019.61187	3.963424694	16.3975973	0.012007919	2.6998119	89969.06317	4.377800967	19.06073164
70 to 74	0.032155768	2.633584673	75037.58473	3.487901225	12.85581419	0.020550641	2.694386986	84724.82129	4.04524623	15.0691574
75 to 79	0.05363223	2.628518595	63886.83539	2.836520473	9.638015743	0.036921724	2.694277137	76444.71875	3.52393459	11.40492303
80 to 84	0.106391973	2.533685173	48805.26164	1.937788861	6.808798434	0.075898786	2.606806757	63516.76693	2.691483868	8.175722081
85 to 89	0.171193768	2.324535903	28429.84932	0.979194439	4.898349753	0.133896754	2.432155124	43285.88953	1.615632903	5.788263457
90 to 94	0.265474405	2.114375768	11878.84719	0.33808268	3.537898199	0.221378099	2.226324303	21906.22788	0.681844482	4.098260055
95 plus	0.353534085	2.830528805	2997.685894	0.085474385	2.830528805	0.316417522	3.164529776	6978.872167	0.222744163	3.164529776

Table 15: Tunisia 2100 life table, by age and sex. mx=mortality rate, ax=mean person-years lived in an age interval among those who die in that age interval, lx=number of persons left alive at age x, nLx=person-years lived between age x and x+n, ex=life expectancy at age x.										
Age Group	Male					Female				
	mx	ax	lx	nLx	ex	mx	ax	lx	nLx	ex
Early Neonatal	0.02903113	0.009588151	100000	0.019172745	83.25903559	0.047711272	0.009587579	100000	0.019169311	87.71476465
Late Neonatal	0.008911162	0.028764665	99944.34132	0.057487486	83.28618763	0.009994313	0.028764366	99908.54964	0.05746511	87.77574707
Post Neonatal	0.000646918	0.46159788	99893.11662	0.922025483	83.2713341	0.000234358	0.461627187	99851.12606	0.921813419	87.76857779
1 to 4	9.79154E-05	1.999869446	99833.47982	3.992557423	82.39747892	5.80136E-05	1.999922645	99829.52661	3.992717888	86.86414106
5 to 9	4.29811E-05	2.499910456	99794.39498	4.989183654	78.42894697	4.66864E-05	2.499902736	99806.36941	4.989736215	82.88379239
10 to 14	6.90787E-05	2.971044172	99772.95209	4.987942098	73.44525523	6.18086E-05	2.697546829	99783.08042	4.988440918	77.90251109
15 to 19	0.000193116	2.806469422	99738.49818	4.984792356	68.46959035	0.000102176	2.628795953	99752.25214	4.986402167	72.92572947
20 to 24	0.00033315	2.657065517	99642.25125	4.978205522	63.53286466	0.000122599	2.59914132	99701.31309	4.983598137	67.96160099
25 to 29	0.000423581	2.606212267	99476.45975	4.968778266	58.63415192	0.000159798	2.656426712	99640.22729	4.980139696	63.00158182
30 to 34	0.000542476	2.565767939	99266.09409	4.956750473	53.75277967	0.000236048	2.650745671	99560.66729	4.975265595	58.04967085
35 to 39	0.000584949	2.585208608	98997.38112	4.942903711	48.89157945	0.00032193	2.658456934	99443.2624	4.96840976	53.11483091
40 to 44	0.000796753	2.668124815	98708.49102	4.926240386	44.02680882	0.000473032	2.691653736	99283.37327	4.958736685	48.19557879
45 to 49	0.001194553	2.708639553	98316.4625	4.902357752	39.19117525	0.000740869	2.691087942	99048.91161	4.943996572	43.30233685
50 to 54	0.001948405	2.730215589	97731.62951	4.865087022	34.40829693	0.001163453	2.721444201	98682.83215	4.921047641	38.45141365
55 to 59	0.00339858	2.709383725	96784.98388	4.801817282	29.71589688	0.001934941	2.699408213	98110.94103	4.88380098	33.65672131
60 to 64	0.005353694	2.70758896	95157.74127	4.700226115	25.17233642	0.003029484	2.700518051	97167.27216	4.824772853	28.95354542
65 to 69	0.008924641	2.698757619	92651.50191	4.539844472	20.77291736	0.004908891	2.696448161	95709.46909	4.732131075	24.3457791
70 to 74	0.014907365	2.737404123	88626.6422	4.28735337	16.58334594	0.00795368	2.757133528	93395.72582	4.588496095	19.87227985
75 to 79	0.027430126	2.771451461	82332.78583	3.881692059	12.62996448	0.015748635	2.816231428	89777.34554	4.340847305	15.54366442
80 to 84	0.061499034	2.632794003	71891.44497	3.147774098	9.053557603	0.035426373	2.730068703	83062.33309	3.852359144	11.54456368
85 to 89	0.115157835	2.481252523	53076.16995	2.076450919	6.311467092	0.074287455	2.646236089	69913.38993	2.998822125	8.154223573
90 to 94	0.208514221	2.214792431	30159.88851	0.968846537	4.303324766	0.149173025	2.332388093	48921.96996	1.782077682	5.554327826
95 plus	0.307473695	3.268638026	10752.51486	0.361546361	3.268638026	0.248762338	4.080800491	23994.74654	1.024518272	4.080800491

Table 15: Turkey 2017 life table, by age and sex. mx=mortality rate, ax=mean person-years lived in an age interval among those who die in that age interval, lx=number of persons left alive at age x, nLx=person-years lived between age x and x+n, ex=life expectancy at age x.

Age Group	Male					Female				
	mx	ax	lx	nLx	ex	mx	ax	lx	nLx	ex
Early Neonatal	0.342503725	0.009578543	100000	0.019115236	74.85363242	0.340914107	0.009578592	100000	0.019115528	82.85091058
Late Neonatal	0.027144199	0.028759636	99345.33881	0.05711299	75.32763607	0.025254389	0.028760157	99348.37509	0.057117833	83.37509087
Post Neonatal	0.005361256	0.461262981	99190.33269	0.913549349	75.38778613	0.003616607	0.461386918	99204.1273	0.914412018	83.43873966
1 to 4	0.000740177	1.999013097	98700.59854	3.942185853	74.83627291	0.000653848	1.999128202	98873.44288	3.949770765	82.79299432
5 to 9	0.000285283	2.499405661	98408.84242	4.916934897	71.05224002	0.000211104	2.499560199	98615.21116	4.928159437	79.00457849
10 to 14	0.000318982	2.799876452	98268.58696	4.909983209	66.15009668	0.000161529	2.527336012	98511.1847	4.92359269	74.08537573
15 to 19	0.000746201	2.686465293	98111.97792	4.897145114	61.25120791	0.00023258	2.611188186	98431.6584	4.918850283	69.14318312
20 to 24	0.00099234	2.547000372	97746.56055	4.875460187	56.47005076	0.000286349	2.549089942	98317.25813	4.912415506	64.220574
25 to 29	0.000978084	2.49339732	97262.75854	4.851244604	51.73815118	0.000300937	2.577395015	98176.59432	4.905253729	59.30890069
30 to 34	0.000970967	2.52905704	96788.28016	4.827831942	46.97944823	0.000399359	2.646157311	98028.98058	4.896846262	54.39428917
35 to 39	0.00112445	2.624374231	96319.53254	4.803147524	42.19565854	0.000583318	2.657921796	97833.42577	4.884997715	49.49767341
40 to 44	0.001659155	2.70027941	95779.47434	4.770772388	37.41864182	0.000845469	2.716201675	97548.48245	4.868024494	44.63440583
45 to 49	0.002756998	2.742106231	94987.99783	4.720021027	32.70771671	0.00147066	2.70484736	97136.9231	4.840507931	39.81189291
50 to 54	0.004973608	2.724950977	93686.85931	4.631939383	28.12354771	0.002317228	2.717258401	96425.0892	4.795887589	35.08564055
55 to 59	0.008451487	2.69865671	91383.57548	4.482024766	23.763181	0.003960783	2.709481822	95313.87047	4.722851222	30.46274573
60 to 64	0.013940548	2.67221724	87596.75033	4.242228584	19.67310382	0.006499498	2.696459127	93443.50996	4.603269875	26.01785504
65 to 69	0.02235513	2.653330935	81685.48863	3.880814615	15.90258792	0.010630487	2.68324112	90452.26987	4.413942957	21.78854938
70 to 74	0.036621676	2.62222923	73015.81097	3.358622107	12.47498933	0.017282652	2.636435874	85761.72757	4.119889732	17.83276959
75 to 79	0.059928307	2.5794853	60729.12929	2.652321952	9.468080456	0.02521894	2.620590312	78645.83723	3.709871051	14.2070321
80 to 84	0.103201888	2.474275061	44860.12337	1.780048955	6.906167037	0.03961603	2.591468497	69299.00111	3.163476443	10.76955414
85 to 89	0.166910999	2.334748978	26529.93654	0.918782389	4.973313156	0.082170639	2.607426151	56783.06622	2.373268438	7.572811829
90 to 94	0.261521666	2.122859813	11229.22952	0.320681352	3.578139458	0.161467255	2.337125723	37311.051	1.305193539	5.166751297
95 plus	0.350468516	2.853653546	2858.12008	0.081663993	2.853653546	0.260931151	3.833295229	16266.43339	0.624195549	3.833295229

Table 15: Turkey 2100 life table, by age and sex. mx=mortality rate, ax=mean person-years lived in an age interval among those who die in that age interval, lx=number of persons left alive at age x, nLx=person-years lived between age x and x+n, ex=life expectancy at age x.

Age Group	Male					Female				
	mx	ax	lx	nLx	ex	mx	ax	lx	nLx	ex
Early Neonatal	0.057431297	0.009587281	100000	0.019167525	82.14104483	0.074947334	0.009586744	100000	0.019164308	88.42229882
Late Neonatal	0.004105544	0.028765991	99889.93139	0.057464133	82.212333	0.004462888	0.028765892	99856.395	0.05744425	88.53031611
Post Neonatal	0.000805307	0.461586628	99866.34056	0.921710947	82.17418875	0.000476699	0.461609972	99830.76076	0.921522315	88.49550185
1 to 4	0.000153798	1.999794936	99792.12711	3.990457781	81.31169187	0.000128426	1.999828766	99786.83756	3.990448599	87.61097383
5 to 9	8.14047E-05	2.499830407	99730.76987	4.985523907	77.36047567	7.31237E-05	2.499847659	99735.59773	4.985868474	83.65494592
10 to 14	0.00014194	2.99954665	99690.1898	4.983045029	72.39091173	7.69046E-05	2.6224471	99699.14394	4.984041589	78.68460592
15 to 19	0.000376026	2.666803542	99619.47147	4.97658452	67.44018175	0.000114139	2.572266307	99660.81982	4.981657746	73.71384239
20 to 24	0.000422969	2.516998296	99432.39342	4.966418102	62.56183587	0.000113666	2.534969444	99603.9685	4.978803265	68.75443459
25 to 29	0.00042382	2.484743217	99222.45616	4.955844266	57.68848061	0.000133	2.612561735	99547.38601	4.975785973	63.79204714
30 to 34	0.000393787	2.464993387	99012.59451	4.945704144	52.8050094	0.00018217	2.63849376	99481.2216	4.971920023	58.83269005
35 to 39	0.000364451	2.624877163	98818.05438	4.936591625	47.90356527	0.000252049	2.666252764	99390.66354	4.966602943	53.88378715
40 to 44	0.000571139	2.688025409	98638.39602	4.925410692	42.98530887	0.000374937	2.747839846	99265.50967	4.959084906	48.94814287
45 to 49	0.000875203	2.799677543	98357.31062	4.908366689	38.0996138	0.000696526	2.74402754	99079.61743	4.9461958	44.03453801
50 to 54	0.001791722	2.771496557	97928.19414	4.876942458	33.25245047	0.001182042	2.713569882	98735.23402	4.923488598	39.17786898
55 to 59	0.003251923	2.743720123	97055.31116	4.817367081	28.52370918	0.001953852	2.733848046	98153.57514	4.885932632	34.39285552
60 to 64	0.005631084	2.759147184	95491.9443	4.7151472	23.94001694	0.0032841	2.717219004	97200.37453	4.823881905	29.70137214
65 to 69	0.010657468	2.731692264	92844.27679	4.5329045	19.53496219	0.005502549	2.706430575	95618.44833	4.721345465	25.1457307
70 to 74	0.018864979	2.739623135	88035.86846	4.223069262	15.4384906	0.008972849	2.697999954	93027.80779	4.557326613	20.7677465
75 to 79	0.03863384	2.691335339	80137.56348	3.685269163	11.66499355	0.014767873	2.700712794	88955.13178	4.301833341	16.59200365
80 to 84	0.074643163	2.545853406	66288.19823	2.827430147	8.496435734	0.025288113	2.655779295	82642.99207	3.902487682	12.65122426
85 to 89	0.130513389	2.440863599	46499.83175	1.785185237	5.976547283	0.057889812	2.712167323	72867.79855	3.222712739	8.97108774
90 to 94	0.223487287	2.185220392	25379.32964	0.807565142	4.123572633	0.127590467	2.350944953	54538.5919	2.048979878	6.073275867
95 plus	0.319387268	3.165625439	8865.117741	0.297875279	3.165625439	0.228115775	4.409449287	28944.71702	1.295769776	4.409449287

Table 15: United Arab Emirates 2017 life table, by age and sex. mx=mortality rate, ax=mean person-years lived in an age interval among those who die in that age interval, lx=number of persons left alive at age x, nLx=person-years lived between age x and x+n, ex=life expectancy at age x.

Age Group	Male					Female				
	mx	ax	lx	nLx	ex	mx	ax	lx	nLx	ex
Early Neonatal	0.291051768	0.00958012	100000	0.01912466	71.33001782	0.238805889	0.009581722	100000	0.019134235	76.3109894
Late Neonatal	0.038908764	0.02875639	99443.41794	0.057150041	71.70993214	0.030071181	0.028758828	99543.09251	0.057221861	76.64201111
Post Neonatal	0.003250167	0.461412949	99221.09676	0.914723338	71.81301271	0.003064205	0.46142616	99371.04589	0.916184254	76.71713487
1 to 4	0.000694951	1.999073398	98923.87401	3.951461958	71.1041382	0.000572532	1.999236624	99090.36763	3.959080718	76.00989145
5 to 9	0.000261159	2.499455919	98649.35418	4.929249256	67.29650015	0.000200589	2.499582106	98863.75681	4.940710094	72.17958848
10 to 14	0.000432977	3.084939827	98520.64424	4.921954464	62.38116728	0.000202342	2.675625742	98764.6636	4.93591315	67.24950073
15 to 19	0.001487388	2.597598132	98307.55671	4.897881106	57.50957897	0.000373133	2.593759566	98664.79496	4.928816128	62.31479297
20 to 24	0.001143076	2.394364879	97579.11534	4.864470087	52.9185591	0.00037275	2.528563816	98480.8922	4.919515218	57.42610968
25 to 29	0.000923295	2.470607822	97023.16154	4.839857938	48.20735488	0.000428095	2.57091923	98297.52803	4.90977466	52.52827024
30 to 34	0.001023784	2.608279742	96576.40083	4.817030827	43.41838918	0.000523749	2.597908314	98087.36349	4.898210359	47.63497058
35 to 39	0.001471243	2.686328474	96083.38514	4.78788037	38.62717584	0.000680873	2.698635417	97830.86096	4.883887592	42.75258215
40 to 44	0.002370776	2.712581696	95379.25911	4.743255463	33.89156354	0.001175169	2.800065009	97498.41769	4.862340156	37.88852003
45 to 49	0.003966301	2.721823912	94255.39655	4.670596074	29.26185068	0.002382586	2.79899206	96927.2145	4.821074376	33.09441475
50 to 54	0.006801397	2.748354855	92404.51633	4.550599438	24.79117676	0.004668706	2.768214227	95779.08267	4.739585843	28.45618401
55 to 59	0.012737801	2.793321494	89313.49281	4.343740417	20.55077792	0.008696332	2.776884171	93567.74518	4.589713099	24.06109922
60 to 64	0.027607741	2.649081717	83790.99216	3.934816675	16.71659878	0.017441307	2.677345486	89580.34282	4.304869511	20.00522336
65 to 69	0.040011117	2.558888392	72958.38795	3.324596757	13.79950124	0.026933442	2.594877909	82083.77356	3.855098547	16.58299611
70 to 74	0.055841833	2.498563927	59716.44445	2.622034497	11.28966612	0.037305341	2.527310129	71728.40231	3.28476748	13.59784936
75 to 79	0.068709738	2.477867714	45175.3858	1.927954271	9.124812824	0.044769962	2.563879588	59530.6409	2.685690983	10.86452595
80 to 84	0.104718271	2.467527913	32058.84662	1.270124579	6.86086515	0.080101949	2.563674803	47595.3978	1.994401807	7.950145823
85 to 89	0.168469808	2.331121524	18910.52707	0.654648325	4.947458654	0.139105786	2.416822491	31778.42901	1.172471736	5.647670248
90 to 94	0.262943697	2.119765195	7998.980774	0.228519516	3.56424761	0.226999702	2.2139567	15643.99323	0.48119466	4.01615128
95 plus	0.3515648	2.845661341	2039.206749	0.058347758	2.845661341	0.321483851	3.113569029	4825.618336	0.151332931	3.113569029

Table 15: United Arab Emirates 2100 life table, by age and sex. mx=mortality rate, ax=mean person-years lived in an age interval among those who die in that age interval, lx=number of persons left alive at age x, nLx=person-years lived between age x and x+n, ex=life expectancy at age x.

Age Group	Male					Female				
	mx	ax	lx	nLx	ex	mx	ax	lx	nLx	ex
Early Neonatal	0.096744017	0.009586076	100000	0.019160308	77.12948703	0.085595996	0.009586418	100000	0.019162355	82.38806712
Late Neonatal	0.011873763	0.028763848	99814.73535	0.057408049	77.25353512	0.011305767	0.028764005	99836.05543	0.057421247	82.50411023
Post Neonatal	0.000871755	0.461581908	99746.58956	0.92057753	77.24875692	0.000889532	0.461580645	99771.14865	0.920796663	82.50021428
1 to 4	0.000173659	1.999768455	99666.36418	3.985270422	76.38737445	0.000155675	1.999792433	99689.27272	3.986329803	81.64428798
5 to 9	6.63312E-05	2.49986181	99597.16821	4.979032717	72.43903324	7.04434E-05	2.499853243	99627.22644	4.980484197	77.69386815
10 to 14	0.000161162	3.24098671	99564.14259	4.976856483	67.46205598	9.50408E-05	2.803968995	99592.14372	4.978570948	72.72033948
15 to 19	0.001033167	2.738675556	99483.94513	4.961948062	62.5132289	0.00021208	2.604291573	99544.82844	4.974703776	67.75355769
20 to 24	0.000747752	2.513517874	98974.51331	4.939448957	57.8144029	0.000191029	2.534491841	99439.35671	4.969610828	62.82251589
25 to 29	0.000707712	2.526964108	98606.8311	4.921691743	53.01919292	0.000227629	2.615577199	99344.45084	4.96451438	57.88007071
30 to 34	0.00073501	2.571368908	98260.09444	4.904243184	48.19631423	0.000304089	2.611071106	99231.47238	4.957964603	52.94287616
35 to 39	0.000947953	2.66854749	97900.50647	4.884279225	43.36328738	0.000382767	2.693194682	99080.74254	4.949654445	48.01913035
40 to 44	0.001637477	2.68153327	97439.23886	4.853496106	38.55416567	0.000648212	2.818850869	98891.3337	4.937575509	43.10546024
45 to 49	0.002430886	2.713026195	96649.37004	4.805541021	33.84159861	0.001370866	2.800423575	98571.38516	4.913641703	38.23529176
50 to 54	0.003932921	2.786249022	95490.70242	4.733028607	29.21097731	0.0025323	2.775722239	97898.35813	4.867383259	33.47622857
55 to 59	0.007847687	2.805417598	93641.4356	4.603001938	24.72091425	0.004666339	2.76184018	96667.3118	4.783660676	28.86314732
60 to 64	0.017338345	2.677477114	90051.39002	4.329852151	20.57696897	0.008991548	2.695245082	94438.35964	4.625985448	24.47342567
65 to 69	0.026549343	2.577998195	82650.23883	3.888196816	17.13348061	0.012959059	2.642171066	90318.24606	4.382701688	20.44856304
70 to 74	0.035890082	2.538012071	72585.21657	3.347465793	14.10838279	0.019247183	2.621110199	84695.54712	4.051971996	16.6231636
75 to 79	0.049341123	2.56128904	61178.17146	2.756704709	11.22215353	0.027350259	2.71260666	77044.14152	3.628348358	13.00608046
80 to 84	0.0778953	2.53713733	48909.34687	2.089085599	8.423362782	0.057047617	2.648997418	67351.14796	2.977934755	9.497890702
85 to 89	0.133517562	2.434495868	34453.93369	1.329790324	5.939140981	0.107290756	2.521402121	50836.00417	2.026400096	6.688970869
90 to 94	0.226094616	2.178585113	19060.90076	0.611029083	4.104214304	0.190749352	2.284085001	30065.51141	1.007363681	4.636296106
95 plus	0.321352976	3.154387863	6812.623588	0.232378203	3.154387863	0.288103952	3.501050314	11742.83312	0.425886706	3.501050314

Table 15: Yemen 2017 life table, by age and sex. mx=mortality rate, ax=mean person-years lived in an age interval among those who die in that age interval, lx=number of persons left alive at age x, nLx=person-years lived between age x and x+n, ex=life expectancy at age x.

Age Group	Male					Female				
	mx	ax	lx	nLx	ex	mx	ax	lx	nLx	ex
Early Neonatal	0.932448106	0.009560462	100000	0.019007635	66.18943673	0.673985978	0.009568384	100000	0.019054676	70.53443295
Late Neonatal	0.085534776	0.028743529	98227.82982	0.056375841	67.3625347	0.080545892	0.028744905	98715.84737	0.056664046	71.43170381
Post Neonatal	0.016079998	0.460501544	97745.73214	0.89581075	67.63672992	0.01811008	0.460357332	98259.52756	0.899677706	71.70549535
1 to 4	0.002065533	1.99724596	96306.02753	3.836378617	67.71394356	0.002407554	1.996789933	96630.87317	3.84669089	71.97866498
5 to 9	0.000894465	2.498136533	95514.00387	4.765038907	64.2581451	0.000845848	2.498237818	95705.16987	4.775157207	68.65464845
10 to 14	0.000820418	2.767330867	95087.87081	4.745701467	59.53479897	0.000702678	2.564207465	95301.40424	4.75692923	63.93466196
15 to 19	0.001962979	2.762653225	94698.59887	4.714230465	54.76804616	0.001069745	2.609629021	94967.19837	4.736252383	59.1505368
20 to 24	0.00334652	2.566011259	93773.39799	4.65080058	50.28085337	0.001277727	2.550187395	94460.63374	4.7083031	54.45357564
25 to 29	0.00310324	2.475353169	92217.46499	4.575049008	46.08525833	0.001397441	2.576782874	93859.26928	4.677138541	49.78571795
30 to 34	0.00308657	2.515143938	90798.3416	4.505389804	41.76608044	0.001820167	2.620239143	93206.053	4.640221749	45.1159354
35 to 39	0.003439476	2.565432175	89408.56377	4.43335689	37.37532084	0.002490594	2.629797628	92362.05607	4.591038774	40.50339865
40 to 44	0.00431964	2.620087795	87885.1988	4.349628901	32.97735846	0.003456446	2.640713762	91219.70936	4.524153823	35.97637669
45 to 49	0.006173931	2.659624755	86009.26478	4.239342485	28.63745935	0.004974851	2.656105241	89658.28618	4.431338367	31.55481863
50 to 54	0.009534876	2.664503353	83397.59041	4.079286532	24.44830931	0.007484623	2.641773328	87458.05093	4.297238696	27.27922238
55 to 59	0.014794958	2.654955469	79519.52588	3.843088384	20.50740825	0.010683815	2.644968461	84250.25659	4.109435793	23.21412349
60 to 64	0.023110313	2.642774071	73855.7117	3.50277345	16.87352944	0.016269457	2.654386434	79875.13727	3.847526637	19.33779664
65 to 69	0.037135535	2.578791414	65798.07403	3.019891147	13.61500832	0.026031009	2.610266188	73642.88841	3.467654606	15.74745047
70 to 74	0.050736331	2.552884932	54648.43245	2.432673078	10.86896443	0.037061712	2.601829239	64671.29131	2.971224859	12.57021758
75 to 79	0.081347242	2.512407097	42401.12195	1.765929162	8.279768738	0.061590678	2.56359247	53734.98816	2.338720392	9.603466534
80 to 84	0.123985392	2.415823011	28163.05585	1.069230262	6.214430157	0.097338078	2.482030447	39450.81235	1.587602509	7.164149065
85 to 89	0.189387515	2.280148922	15039.88104	0.498107346	4.563161925	0.159965035	2.360437203	24158.32695	0.852129644	5.153575035
90 to 94	0.282464573	2.078498671	5694.840867	0.156615375	3.358013307	0.248813501	2.16419202	10665.80335	0.313976908	3.729169654
95 plus	0.366787209	2.727249137	1303.491474	0.035730648	2.727249137	0.340817424	2.936116869	2920.941206	0.086302408	2.936116869

Table 15: Yemen 2100 life table, by age and sex. mx=mortality rate, ax=mean person-years lived in an age interval among those who die in that age interval, lx=number of persons left alive at age x, nLx=person-years lived between age x and x+n, ex=life expectancy at age x.

Age Group	Male					Female				
	mx	ax	lx	nLx	ex	mx	ax	lx	nLx	ex
Early Neonatal	0.217453225	0.009582376	100000	0.019138158	79.4649947	0.155070957	0.009584288	100000	0.019149599	83.36752287
Late Neonatal	0.016715986	0.028762512	99583.99025	0.057267374	79.7741116	0.01661364	0.02876254	99703.13781	0.057336056	83.59340361
Post Neonatal	0.001948794	0.461505397	99488.32711	0.9177386	79.79253046	0.002694229	0.461452442	99607.9327	0.918525904	83.6149551
1 to 4	0.000306336	1.999591552	99309.72189	3.969957939	79.01015239	0.000396729	1.999471028	99360.71748	3.971279518	82.89632179
5 to 9	0.000186638	2.499611171	99188.2104	4.957099064	75.1034396	0.000220892	2.499539807	99203.3215	4.957431008	79.02380807
10 to 14	0.000230479	2.905984059	99095.77167	4.952407949	70.17054599	0.000234206	2.636512426	99093.94632	4.951942327	74.10778715
15 to 19	0.00064472	2.781306252	98981.74852	4.942056303	65.24735541	0.000358283	2.607711203	98978.12054	4.944659658	69.1910482
20 to 24	0.001133164	2.579186765	98663.73626	4.919701029	60.44688942	0.000406499	2.543015238	98801.23079	4.935124176	64.30974782
25 to 29	0.001043265	2.496100804	98108.50481	4.892614869	55.77259146	0.000426992	2.59524914	98600.98512	4.924983308	59.43448972
30 to 34	0.001028475	2.517949347	97600.89128	4.867613687	51.04872754	0.000586145	2.62776551	98391.08201	4.912722276	54.55484424
35 to 39	0.001064015	2.558739321	97103.27555	4.842596214	46.29678555	0.000777202	2.621886901	98103.65399	4.896149671	49.70579008
40 to 44	0.001276653	2.632361602	96591.09411	4.81505714	41.52761461	0.001046334	2.674151186	97723.89057	4.874378719	44.88690821
45 to 49	0.001872225	2.700258638	95979.73141	4.778503039	36.77281661	0.001685718	2.693609207	97215.15533	4.841980627	40.10485602
50 to 54	0.003104935	2.7234991	95090.02903	4.721279616	32.08629353	0.002627636	2.654449357	96402.15736	4.790675235	35.41457414
55 to 59	0.005265237	2.70873577	93634.33276	4.62613158	27.53405041	0.003645253	2.647024491	95150.47364	4.717280495	30.83763214
60 to 64	0.008411119	2.711703282	91222.75625	4.47582705	23.17876839	0.005262892	2.676395362	93444.93486	4.616355053	26.34270487
65 to 69	0.014530318	2.663411171	87508.96958	4.233767902	19.03375905	0.008436006	2.671368612	91043.51546	4.465701802	21.95557141
70 to 74	0.021130827	2.684811833	81481.11896	3.88731724	15.23364399	0.012586703	2.739777734	87348.95515	4.248650063	17.75797213
75 to 79	0.039306337	2.656413022	73439.80177	3.369414563	11.60531986	0.024881589	2.727705802	82120.31821	3.891462562	13.70536801
80 to 84	0.069866098	2.562667468	60550.72481	2.601602475	8.518780461	0.048378713	2.642871084	72705.00781	3.275862647	10.1226876
85 to 89	0.126159204	2.448249868	43076.73612	1.646165438	5.971921492	0.094867417	2.563611417	57478.25328	2.353122622	7.11976282
90 to 94	0.220505421	2.19898635	23138.00717	0.724734162	4.115684971	0.176342098	2.310599796	36086.83956	1.239327803	4.894434329
95 plus	0.317384989	3.161140257	7658.138612	0.247003306	3.161140257	0.274752719	3.662633524	15019.38731	0.563421738	3.662633524

Table 15: South Asia 2017 life table, by age and sex. mx=mortality rate, ax=mean person-years lived in an age interval among those who die in that age interval, lx=number of persons left alive at age x, nLx=person-years lived between age x and x+n, ex=life expectancy at age x.										
Age Group	Male					Female				
	mx	ax	lx	nLx	ex	mx	ax	lx	nLx	ex
Early Neonatal	1.116301222	0.009554827	100000	0.018974254	67.97200419	0.978290686	0.009559057	100000	0.018999296	70.27010779
Late Neonatal	0.083187092	0.028744176	97881.92797	0.056181082	69.42343586	0.09864512	0.028739912	98141.33468	0.056304951	71.58155177
Post Neonatal	0.011841934	0.460802606	97414.58691	0.894518004	69.6988214	0.01483034	0.460590316	97585.92581	0.894858494	71.9312638
1 to 4	0.001490136	1.998013154	96355.34375	3.842750407	69.53668343	0.001823403	1.997568798	96258.86102	3.836347473	71.99331352
5 to 9	0.000657069	2.498631106	95782.74665	4.781279154	65.94044788	0.000838209	2.498253733	95559.36496	4.767970085	68.50569599
10 to 14	0.000578435	2.552189708	95468.5916	4.766680648	61.14921888	0.000562157	2.526972672	95159.71769	4.751380599	63.78291654
15 to 19	0.00080676	2.700877919	95192.87424	4.750831787	56.31893585	0.000917346	2.653227514	94892.61773	4.734438793	58.9553319
20 to 24	0.0013657	2.616633659	94809.59846	4.725099797	51.53567368	0.001245824	2.572776416	94458.30783	4.708677017	54.21418653
25 to 29	0.00158716	2.617384513	94164.2958	4.690477214	46.87088792	0.001366562	2.544904975	93871.69406	4.67789027	49.53687493
30 to 34	0.002291574	2.643929177	93419.84842	4.645908911	42.2235133	0.001559739	2.582137923	93232.4387	4.64410817	44.85905473
35 to 39	0.003229557	2.62862807	92355.21397	4.582665036	37.67974333	0.002008966	2.652720494	92508.0884	4.603695476	40.19006407
40 to 44	0.004399385	2.644325865	90875.23784	4.497156495	33.25053702	0.00308479	2.625902593	91583.23687	4.545870076	35.56909968
45 to 49	0.006511552	2.648522057	88896.80789	4.377810424	28.93163712	0.003965203	2.755472558	90180.96118	4.46927202	31.08130793
50 to 54	0.009528277	2.681808676	86046.2631	4.209339144	24.80227874	0.008244118	2.657821179	88408.84985	4.336706575	26.64904086
55 to 59	0.016070768	2.633443826	82035.68161	3.951506849	20.88363269	0.010832721	2.641538111	84833.73685	4.136021894	22.66001894
60 to 64	0.022499855	2.598630031	75685.68413	3.590312833	17.41474228	0.017141923	2.638219779	80353.50989	3.861356469	18.77610653
65 to 69	0.032449686	2.592584403	67608.20387	3.13549069	14.18485109	0.025509766	2.645034087	73734.84855	3.477831434	15.22461748
70 to 74	0.049144594	2.556181685	57434.70248	2.563858654	11.23821618	0.043627287	2.574851442	64863.81795	2.932921812	11.94500018
75 to 79	0.071206751	2.526346362	44836.56733	1.906143528	8.677807329	0.059194167	2.564297087	52070.06631	2.275488568	9.247308995
80 to 84	0.119359399	2.458775595	31266.15002	1.199549562	6.348034245	0.112512824	2.50216067	38603.17697	1.50679428	6.578924938
85 to 89	0.185079799	2.289974912	16951.72964	0.56451371	4.632923281	0.17854823	2.314731296	21653.8322	0.731885351	4.770596061
90 to 94	0.278547523	2.086907599	6506.070115	0.179600022	3.39568964	0.267690987	2.120399631	8589.410344	0.24255084	3.507086129
95 plus	0.363776251	2.748967264	1504.238734	0.041355987	2.748967264	0.357243943	2.799254411	2097.98787	0.058737848	2.799254411

Table 15: South Asia 2100 life table, by age and sex. mx=mortality rate, ax=mean person-years lived in an age interval among those who die in that age interval, lx=number of persons left alive at age x, nLx=person-years lived between age x and x+n, ex=life expectancy at age x.

Age Group	Male					Female				
	mx	ax	lx	nLx	ex	mx	ax	lx	nLx	ex
Early Neonatal	0.212441957	0.00958253	100000	0.019139071	78.47762536	0.199355237	0.009582931	100000	0.019141471	80.6171811
Late Neonatal	0.010885046	0.028764121	99593.47238	0.057282417	78.77841709	0.01912524	0.028761848	99618.45581	0.05728321	80.90618591
Post Neonatal	0.001216284	0.461557433	99531.12652	0.918442894	78.77013936	0.001838889	0.461513204	99508.91493	0.917974142	80.93749705
1 to 4	0.000135361	1.999819518	99419.43355	3.975701083	77.93470305	0.000153962	1.999794717	99340.13422	3.972382282	80.1507159
5 to 9	0.000113933	2.499762639	99365.6259	4.966866838	73.9757872	0.000156276	2.499674424	99278.9868	4.962011214	76.19876942
10 to 14	0.00016228	2.771527045	99309.0538	4.963649856	69.01639122	0.000157992	2.702200578	99201.47359	4.958256387	71.25607242
15 to 19	0.000317824	2.854583944	99228.52942	4.957994164	64.06999657	0.000292389	2.663848866	99123.1788	4.952771797	66.30996562
20 to 24	0.000653442	2.640798688	99071.02529	4.945923114	59.16691981	0.00038342	2.600017745	98978.42831	4.944363004	61.40267295
25 to 29	0.000756936	2.628721768	98747.95884	4.928535445	54.35129386	0.000467202	2.589007576	98788.95647	4.933887891	56.51504934
30 to 34	0.001106681	2.62656455	98375.16973	4.905890735	49.54652256	0.000579977	2.607963288	98558.58127	4.921090137	51.64054999
35 to 39	0.001452133	2.619713414	97832.63252	4.874785965	44.80587843	0.000755152	2.678870447	98273.37454	4.905091788	46.78205663
40 to 44	0.001941774	2.653722781	97125.91775	4.834264522	40.11101511	0.00125175	2.679485871	97903.20353	4.881013256	41.94763594
45 to 49	0.002890223	2.671282051	96188.72061	4.777338588	35.47403696	0.001868467	2.79444293	97292.87554	4.84486947	37.19152483
50 to 54	0.004415671	2.741567015	94810.6956	4.693904861	30.94783277	0.004134527	2.683889257	96389.29069	4.773643495	32.51011004
55 to 59	0.008398637	2.670240538	92743.9709	4.548302062	26.57201127	0.005284866	2.673817314	94427.61193	4.663958046	28.12064351
60 to 64	0.011519797	2.622569293	88947.61055	4.329546075	22.58630338	0.008473411	2.645551726	91977.98085	4.509485992	23.79396131
65 to 69	0.016666924	2.634718214	83994.8736	4.041442724	18.7540977	0.011911329	2.688608423	88183.55353	4.291729717	19.69444009
70 to 74	0.024895263	2.610036096	77326.20693	3.651177115	15.14211117	0.020967507	2.642537255	83119.05885	3.962426797	15.72463356
75 to 79	0.036426854	2.636410538	68333.9287	3.148937419	11.79103026	0.030445335	2.711601069	74925.7156	3.505366928	12.14754879
80 to 84	0.066912536	2.589665402	57021.87442	2.460294599	8.615593576	0.067834693	2.630639233	64433.6519	2.784055027	8.687070446
85 to 89	0.122997946	2.455898435	40809.27252	1.560695803	6.025437785	0.122880606	2.46744319	45976.59304	1.76573379	6.129728823
90 to 94	0.217618712	2.205763485	21936.27251	0.686035088	4.143787267	0.209029817	2.251615819	24906.7339	0.799882891	4.299830701
95 plus	0.315147756	3.177249161	7216.087517	0.231474919	3.177249161	0.305103693	3.290191363	8651.03819	0.290762369	3.290191363

Table 15: Bangladesh 2017 life table, by age and sex. mx=mortality rate, ax=mean person-years lived in an age interval among those who die in that age interval, lx=number of persons left alive at age x, nLx=person-years lived between age x and x+n, ex=life expectancy at age x.										
Age Group	Male					Female				
	mx	ax	lx	nLx	ex	mx	ax	lx	nLx	ex
Early Neonatal	1.120833323	0.009554688	100000	0.018973445	71.49761043	0.892194449	0.009561696	100000	0.01901495	74.23755576
Late Neonatal	0.082159813	0.02874446	97873.62502	0.056178003	73.03119217	0.069428327	0.028747972	98303.64804	0.056445474	75.49910059
Post Neonatal	0.007205925	0.461131939	97412.18013	0.896410428	73.31948443	0.009690591	0.460955433	97911.83435	0.899976235	75.74360172
1 to 4	0.001520124	1.997973169	96766.50624	3.858920775	72.88237184	0.001499066	1.998001246	97039.86335	3.869983276	75.49683777
5 to 9	0.000883392	2.498159601	96180.13286	4.79840413	69.31465457	0.000863087	2.498201903	96459.88457	4.812604166	71.93887187
10 to 14	0.000800568	2.471895889	95756.34561	4.778148394	64.61042313	0.000548816	2.432554725	96044.58183	4.795473746	67.23917564
15 to 19	0.000782326	2.51380298	95373.87933	4.759439446	59.85954748	0.000691669	2.567751624	95781.42032	4.781029427	62.41715692
20 to 24	0.000861019	2.547284335	95001.5621	4.74006814	55.08409041	0.000779529	2.551118407	95450.7451	4.763445141	57.62432342
25 to 29	0.000985086	2.589492867	94593.45886	4.718469362	50.31053491	0.000889859	2.541024792	95079.44383	4.743593696	52.83918585
30 to 34	0.001295262	2.606316259	94128.68586	4.691889634	45.54594113	0.00096271	2.598052871	94657.36782	4.721951695	48.06322293
35 to 39	0.001664651	2.653651763	93521.02573	4.657862124	40.82465462	0.001355771	2.677285709	94202.83226	4.695357483	43.28234048
40 to 44	0.002559872	2.723736382	92745.76774	4.610429726	36.14332717	0.002140201	2.639810465	93566.34338	4.654806956	38.55816568
45 to 49	0.004508446	2.738154062	91565.80967	4.532087613	31.57339021	0.002835373	2.786792192	92570.32358	4.599656648	33.94409175
50 to 54	0.008000463	2.667130969	89523.21202	4.394182213	27.23034489	0.006203816	2.65529782	91266.49923	4.497919985	29.38858697
55 to 59	0.011611228	2.665381796	86009.45986	4.187042485	23.23244222	0.007787238	2.663569819	88477.16212	4.34484553	25.23023886
60 to 64	0.018983473	2.626153058	81151.46259	3.882769517	19.46222997	0.013193327	2.661328194	85095.6811	4.127527626	21.12583502
65 to 69	0.026916037	2.57765991	73788.38726	3.463903054	16.14088114	0.019989618	2.62526369	79654.60849	3.802446039	17.38566909
70 to 74	0.036660497	2.555963277	64479.22009	2.959367878	13.09842537	0.029788221	2.60470108	72063.34227	3.36361725	13.93918334
75 to 79	0.051311442	2.572460591	53653.96676	2.386335607	10.22632416	0.045109868	2.613332819	62063.58	2.802307267	10.7648382
80 to 84	0.088879921	2.532504031	41446.46043	1.700713375	7.483973735	0.081962227	2.55774068	49459.10092	2.061794486	7.843757716
85 to 89	0.150572703	2.37748928	26385.24191	0.946824405	5.318440554	0.141564553	2.409327466	32624.02154	1.194902859	5.577992941
90 to 94	0.245758842	2.155044635	12179.04368	0.358846496	3.762768298	0.229686124	2.208096687	15777.70061	0.481444172	3.975436101
95 plus	0.337971076	2.959346872	3384.439943	0.100344179	2.959346872	0.323921703	3.088273754	4760.122767	0.147411648	3.088273754

Table 15: Bangladesh 2100 life table, by age and sex. mx=mortality rate, ax=mean person-years lived in an age interval among those who die in that age interval, lx=number of persons left alive at age x, nLx=person-years lived between age x and x+n, ex=life expectancy at age x.

Age Group	Male					Female				
	mx	ax	lx	nLx	ex	mx	ax	lx	nLx	ex
Early Neonatal	0.193920377	0.009583097	100000	0.01914247	82.59926141	0.172205901	0.009583763	100000	0.019146453	84.54859271
Late Neonatal	0.00873496	0.028764714	99628.87159	0.057306323	82.88694732	0.010000645	0.028764365	99670.35131	0.057328095	84.80793558
Post Neonatal	0.000739434	0.461591307	99578.8285	0.919085364	82.87083231	0.001184347	0.461559702	99613.03418	0.919212415	84.79889553
1 to 4	0.000198958	1.999734723	99510.89135	3.978855643	82.00286642	0.000183414	1.999755449	99504.22151	3.978712879	83.96686388
5 to 9	0.000187862	2.499608622	99431.94945	4.969277067	78.06298047	0.000189039	2.49960617	99431.48372	4.969238244	80.02441037
10 to 14	0.000328898	2.705593971	99339.20982	4.963144566	73.13001261	0.000230072	2.597688788	99338.11235	4.964131199	75.09513559
15 to 19	0.000412762	2.627873786	99177.49427	4.954000306	68.24067635	0.000245399	2.564183669	99224.86632	4.958271551	70.1760402
20 to 24	0.000498237	2.552421798	98975.8119	4.942844609	63.37041474	0.000273203	2.567994067	99104.26363	4.951935773	65.25708288
25 to 29	0.000526243	2.61569466	98733.62356	4.930507371	58.51669551	0.000318305	2.568630833	98970.23274	4.944715974	60.34087571
30 to 34	0.000674399	2.621191767	98479.75453	4.916113894	53.65832368	0.000372481	2.636914488	98814.41548	4.936366228	55.43091473
35 to 39	0.000784187	2.636089503	98154.56543	4.89872488	48.82598091	0.00050959	2.678680069	98632.49558	4.925811271	50.52700131
40 to 44	0.001092675	2.751165608	97777.86689	4.876989452	44.00292375	0.00077147	2.727661286	98383.8216	4.910490996	45.64635089
45 to 49	0.001986694	2.764090803	97253.92326	4.841266672	39.2245748	0.001192603	2.885210156	98008.29761	4.888062224	40.80854917
50 to 54	0.003465917	2.718133877	96303.76961	4.7775298	34.58255252	0.00289568	2.672677834	97429.37624	4.838955057	36.03174325
55 to 59	0.005498077	2.667144442	94664.13782	4.673646863	30.1322673	0.003580354	2.640898663	96036.0708	4.761773238	31.50993146
60 to 64	0.00815619	2.629614079	92114.56928	4.519445682	25.88995384	0.005380905	2.656900302	94344.08648	4.658979708	27.02173375
65 to 69	0.011545065	2.637014133	88468.92868	4.307079668	21.8364381	0.00795665	2.681944491	91861.51404	4.510464927	22.66794152
70 to 74	0.016088493	2.599558066	83587.40833	4.026347121	17.94710917	0.012464267	2.692610882	88326.47582	4.294330844	18.45598627
75 to 79	0.022104153	2.694524085	77219.57133	3.678436414	14.20397932	0.020586902	2.748741199	83076.61671	3.972860166	14.44014463
80 to 84	0.042099542	2.703068723	69375.18862	3.1710727	10.50302506	0.042550243	2.704553949	75099.88385	3.430044241	10.67484181
85 to 89	0.087502401	2.567622361	56494.9924	2.347240243	7.276436057	0.08610967	2.596800523	60995.15188	2.545673057	7.509802869
90 to 94	0.176922687	2.245133024	36890.42767	1.257689955	4.845622223	0.16527943	2.323306684	40052.03186	1.40858006	5.138726502
95 plus	0.281039885	3.579434988	15570.11024	0.573514352	3.579434988	0.264287074	3.81693589	17817.8198	0.703508111	3.81693589

Table 15: Bhutan 2017 life table, by age and sex. mx=mortality rate, ax=mean person-years lived in an age interval among those who die in that age interval, lx=number of persons left alive at age x, nLx=person-years lived between age x and x+n, ex=life expectancy at age x.

Age Group	Male					Female				
	mx	ax	lx	nLx	ex	mx	ax	lx	nLx	ex
Early Neonatal	0.682633789	0.009568119	100000	0.01905311	72.85649427	0.556757748	0.009571977	100000	0.019076067	76.61703658
Late Neonatal	0.053670707	0.028752318	98699.64768	0.056698553	73.7966584	0.054815468	0.028752002	98938.06158	0.056833626	77.41994386
Post Neonatal	0.005442297	0.461257224	98395.48465	0.906196029	73.96718169	0.006578104	0.461176538	98626.61551	0.907848712	77.60682792
1 to 4	0.000839436	1.998880752	97902.59677	3.909540994	73.41408875	0.000893238	1.998809016	98029.65802	3.914192807	77.15347987
5 to 9	0.000520809	2.498914982	97574.64156	4.872387259	69.65425922	0.000380841	2.499206581	97680.1947	4.879363338	73.42249521
10 to 14	0.000436402	2.63758689	97320.95943	4.861037973	64.82931732	0.000323469	2.57807683	97494.398	4.870905365	68.55761466
15 to 19	0.000812983	2.678976615	97108.86839	4.84630076	59.96508937	0.000504706	2.617138273	97336.856	4.860998459	63.66432914
20 to 24	0.001143379	2.566467387	96714.89292	4.822324636	55.19796529	0.000611007	2.58015451	97091.53202	4.847409792	58.81834878
25 to 29	0.001185421	2.534706112	96163.57297	4.794169032	50.49915448	0.000743217	2.6039536	96795.38596	4.831167782	53.99012767
30 to 34	0.001354631	2.579023764	95595.36319	4.764149808	45.78386017	0.00098865	2.635266145	96436.37778	4.810576793	49.18113818
35 to 39	0.001722223	2.626561881	94950.14166	4.728191689	41.07698346	0.001399253	2.64995552	95960.86139	4.782322251	44.41132981
40 to 44	0.002442264	2.66727291	94136.08241	4.680158288	36.40869426	0.002019795	2.658642192	95291.86273	4.742175798	39.7038199
45 to 49	0.003765324	2.694131905	92993.53916	4.609685263	31.82191467	0.002987665	2.670395955	94334.3554	4.684132634	35.07886146
50 to 54	0.006144144	2.691817374	91258.96036	4.499207218	27.37341942	0.004575074	2.66135339	92935.59098	4.597617067	30.5652321
55 to 59	0.009903398	2.687825885	88497.48531	4.325958706	23.14029593	0.00676723	2.672766592	90833.77576	4.471334534	26.20900552
60 to 64	0.01631832	2.663052422	84220.7897	4.056702859	19.17440312	0.010762213	2.670317376	87811.21987	4.283316771	22.0169377
65 to 69	0.025872213	2.627881815	77618.43574	3.657327449	15.57395232	0.016966996	2.66027062	83208.77582	4.001906848	18.084338
70 to 74	0.039961288	2.593851975	68195.07333	3.112319789	12.35844736	0.027403853	2.637675961	76434.11821	3.590069103	14.44868032
75 to 79	0.062192075	2.552406454	55837.65292	2.426021953	9.518821808	0.044099544	2.605597445	66631.11541	3.014803173	11.18438275
80 to 84	0.099233515	2.485239941	40893.50692	1.640549442	7.074083021	0.073164087	2.546804997	53406.09192	2.266422475	8.310629732
85 to 89	0.16226019	2.347152945	24819.46734	0.871122694	5.075207525	0.1299305	2.443434983	36944.0539	1.389515681	5.889467064
90 to 94	0.256992765	2.132017852	10860.95363	0.31413109	3.632607018	0.217158409	2.235824077	19032.39857	0.596444096	4.15713313
95 plus	0.346861648	2.8848121	2867.180066	0.083259675	2.8848121	0.312640133	3.201000199	6171.672492	0.198603412	3.201000199

Table 15: Bhutan 2100 life table, by age and sex. mx=mortality rate, ax=mean person-years lived in an age interval among those who die in that age interval, lx=number of persons left alive at age x, nLx=person-years lived between age x and x+n, ex=life expectancy at age x.

Age Group	Male					Female				
	mx	ax	lx	nLx	ex	mx	ax	lx	nLx	ex
Early Neonatal	0.113697665	0.009585556	100000	0.019157195	80.3345394	0.101223135	0.009585939	100000	0.019159484	85.68284243
Late Neonatal	0.006062254	0.028765451	99782.29001	0.05739898	80.49027266	0.008867399	0.028764677	99806.13331	0.057408066	85.82963452
Post Neonatal	0.000681414	0.461595429	99747.50155	0.9206668	80.46073426	0.000911238	0.461579103	99755.24315	0.920640664	85.81578946
1 to 4	0.000134318	1.999820909	99684.78231	3.986320779	79.58769296	0.000133817	1.999821578	99671.38634	3.985789275	84.96418034
5 to 9	9.78997E-05	2.499796042	99631.26606	4.98034449	75.62919825	7.78585E-05	2.499837795	99618.08696	4.979935229	81.00843073
10 to 14	0.000128741	2.820513921	99582.51794	4.977725424	70.66495402	9.52377E-05	2.668463694	99579.32507	4.977859862	76.03895848
15 to 19	0.000292473	2.71482637	99518.44462	4.972591026	65.70860973	0.000153701	2.600780088	99531.93001	4.9747634	71.07386582
20 to 24	0.000423008	2.589873373	99373.0438	4.963599388	60.80067743	0.000170526	2.598329845	99455.49523	4.970748619	66.12641491
25 to 29	0.000482724	2.554705654	99163.1391	4.952361986	55.9237201	0.000243001	2.63751629	99370.77122	4.965698792	61.18049326
30 to 34	0.000598549	2.587783819	98924.22687	4.939088854	51.05219827	0.000340251	2.650511718	99250.19665	4.958513728	56.25143463
35 to 39	0.000739361	2.633798776	98628.9607	4.922807014	46.1968153	0.000454762	2.65557788	99081.66689	4.948786272	51.34242889
40 to 44	0.001037086	2.686892031	98265.64942	4.901465289	41.35739867	0.000656232	2.712802223	98856.86538	4.935416354	46.45294584
45 to 49	0.001602214	2.736162724	97758.33805	4.870193464	36.55743894	0.00110509	2.7088297	98533.31217	4.914210846	41.59627206
50 to 54	0.0027976	2.757016797	96979.53761	4.818717629	31.82806198	0.001755438	2.674440134	97990.79147	4.879602517	36.8110212
55 to 59	0.005093419	2.742996783	95633.87146	4.727243819	27.23538593	0.002570391	2.688863231	97135.27606	4.828006042	32.11089447
60 to 64	0.008861375	2.716777309	93231.51789	4.569338748	22.86402274	0.00404596	2.686636631	95896.35766	4.75039596	27.49021239
65 to 69	0.015372263	2.677491987	89193.49102	4.306240393	18.77027453	0.006369827	2.715329664	93977.54644	4.631394959	22.99460251
70 to 74	0.024240817	2.647568282	82608.64552	3.908813243	15.04793441	0.010919512	2.737362522	91035.41634	4.442065476	18.64823835
75 to 79	0.039605398	2.629343159	73200.86856	3.348770337	11.63452221	0.020193715	2.746421654	86202.57802	4.122761912	14.53747915
80 to 84	0.067577668	2.571317911	60099.83537	2.586789878	8.598538624	0.040248463	2.681355089	77924.95173	3.565580423	10.78752361
85 to 89	0.12359626	2.45457684	42890.74404	1.639483236	6.019448821	0.082995674	2.606107424	63696.75289	2.66301872	7.582401196
90 to 94	0.218109603	2.20418303	23039.70933	0.720996735	4.141000844	0.162026941	2.332641721	41929.13626	1.471597408	5.177317704
95 plus	0.3155124	3.175664143	7599.615197	0.244424699	3.175664143	0.261356689	3.840512987	18490.47179	0.719488421	3.840512987

Table 15: India 2017 life table, by age and sex. mx=mortality rate, ax=mean person-years lived in an age interval among those who die in that age interval, lx=number of persons left alive at age x, nLx=person-years lived between age x and x+n, ex=life expectancy at age x.

Age Group	Male					Female				
	mx	ax	lx	nLx	ex	mx	ax	lx	nLx	ex
Early Neonatal	1.01213335	0.00955802	100000	0.018993151	67.97361602	0.903029706	0.009561363	100000	0.019012971	70.34572507
Late Neonatal	0.074360531	0.028746611	98077.65553	0.056307706	69.28653038	0.093716293	0.028741272	98283.08686	0.056394264	71.55523695
Post Neonatal	0.012091387	0.460784885	97658.956	0.896658832	69.52593131	0.015371416	0.460551879	97754.59065	0.896181803	71.88440401
1 to 4	0.001322136	1.998237153	96574.79814	3.852795284	69.37799062	0.001736879	1.997684163	96377.07334	3.841722558	71.98200805
5 to 9	0.000574781	2.49880254	96065.41636	4.796375476	65.73527044	0.000776656	2.498381966	95709.83002	4.776212023	68.46991151
10 to 14	0.000496512	2.56063725	95789.73477	4.783692896	60.91726772	0.000513822	2.524941997	95338.89106	4.760890037	63.72659704
15 to 19	0.000722889	2.744180315	95552.22078	4.769832905	56.06232364	0.000843458	2.65790526	95094.26793	4.74533927	58.8840315
20 to 24	0.001352261	2.632632589	95207.41571	4.745180025	51.25541382	0.001160724	2.578061661	94694.01928	4.721428138	54.1216759
25 to 29	0.001599172	2.638581999	94565.74509	4.710498972	46.58532631	0.001290587	2.555437961	94145.99321	4.692495244	49.42169808
30 to 34	0.00245029	2.646586864	93812.45769	4.663729444	41.93819148	0.001521845	2.588548235	93540.38839	4.65991834	44.72510735
35 to 39	0.003388169	2.62515185	92669.71402	4.596500829	37.42268794	0.001963173	2.663426309	92831.22454	4.62036728	40.04698358
40 to 44	0.004611822	2.64031502	91112.35396	4.506575806	33.01744196	0.003113841	2.633600806	91924.17158	4.56258879	35.41583608
45 to 49	0.006748878	2.6361307	89034.0283	4.381797863	28.72649555	0.004054088	2.759991217	90503.46687	4.48444953	30.93040833
50 to 54	0.009523808	2.689815849	86076.86859	4.211192089	24.62277392	0.008485741	2.657844348	88685.45592	4.347860973	26.50785185
55 to 59	0.016752919	2.62525686	82066.33161	3.946322505	20.69456883	0.011142043	2.630410535	84996.05054	4.140488206	22.54303732
60 to 64	0.022301217	2.594380555	75455.37149	3.58068148	17.27763356	0.017023142	2.63675768	80382.84982	3.86371298	18.68576284
65 to 69	0.032702768	2.597588613	67470.46924	3.127804494	14.01530728	0.025660154	2.650539385	73805.91012	3.480480356	15.11583885
70 to 74	0.05009908	2.559607971	57242.48744	2.550349333	11.0553984	0.044510041	2.573609942	64875.59802	2.927634056	11.83166416
75 to 79	0.073870657	2.52429434	44466.90808	1.879645389	8.49636539	0.059825226	2.563087644	51846.18531	2.262514237	9.158308976
80 to 84	0.123879807	2.45035192	30583.99283	1.162192261	6.207502801	0.115171264	2.499487386	38312.92631	1.487388225	6.488107657
85 to 89	0.189965982	2.278449911	16189.43758	0.533636167	4.548686093	0.181634835	2.307660767	21186.01914	0.71146334	4.71306749
90 to 94	0.283045493	2.07729905	6053.986073	0.165670257	3.350368047	0.270771082	2.113263511	8266.211228	0.232006338	3.473651626
95 plus	0.367258188	2.722899656	1365.429167	0.037182858	2.722899656	0.359887918	2.778684507	1985.395191	0.055176117	2.778684507

Table 15: India 2100 life table, by age and sex. mx=mortality rate, ax=mean person-years lived in an age interval among those who die in that age interval, lx=number of persons left alive at age x, nLx=person-years lived between age x and x+n, ex=life expectancy at age x.

Age Group	Male					Female				
	mx	ax	lx	nLx	ex	mx	ax	lx	nLx	ex
Early Neonatal	0.192052901	0.009583155	100000	0.01914281	78.03705265	0.184814918	0.009583377	100000	0.019144138	80.66226635
Late Neonatal	0.009261745	0.028764568	99632.39708	0.05730748	78.30524154	0.0174195	0.028762318	99646.2288	0.057301991	80.92852861
Post Neonatal	0.001125124	0.461563909	99579.32558	0.91892631	78.28932091	0.00184257	0.461512943	99546.42623	0.918318633	80.95182105
1 to 4	0.000102157	1.999863791	99475.94704	3.978225096	77.44673821	0.000132919	1.999822775	99377.24627	3.97403354	80.16523187
5 to 9	8.15106E-05	2.499830186	99435.31071	4.970752818	73.47752299	0.000124924	2.499739742	99324.43594	4.964671906	76.20668403
10 to 14	0.000120145	2.83934487	99394.80538	4.968439683	68.50629107	0.00012522	2.716455689	99262.44859	4.961684614	71.25228962
15 to 19	0.000267254	3.002130299	99335.13522	4.963978389	63.545483	0.000236047	2.672923412	99200.35548	4.957287815	66.29480288
20 to 24	0.000641256	2.658019039	99202.55181	4.952684209	58.62580028	0.0003136	2.61310048	99083.40242	4.95044949	61.3693051
25 to 29	0.000748392	2.659895992	98885.12114	4.935569068	53.80457983	0.000390483	2.610036571	98928.25302	4.941797572	56.46083622
30 to 34	0.001172872	2.628885166	98516.14668	4.912175049	48.99499796	0.000515644	2.626989549	98735.41007	4.930721426	51.56530834
35 to 39	0.001505328	2.619655882	97940.5832	4.879548314	44.2663176	0.000687264	2.710533656	98481.38316	4.91634731	46.69048236
40 to 44	0.002027014	2.65583142	97207.82993	4.837385097	39.57778028	0.001225325	2.696853037	98143.77437	4.893397475	41.840407
45 to 49	0.003000769	2.662259428	96229.51119	4.778026628	34.95066505	0.001864588	2.79951602	97544.9293	4.857582972	37.07783571
50 to 54	0.00445672	2.761212695	94799.49476	4.693357514	30.43411229	0.004228092	2.692442243	96640.97641	4.785175703	32.39382191
55 to 59	0.009027829	2.664048686	92715.18064	4.540239925	26.05075351	0.005386868	2.660944449	94633.17313	4.672730997	28.01179521
60 to 64	0.011838765	2.620863099	88646.71344	4.311813222	22.11583658	0.008270262	2.650084697	92134.67728	4.519538519	23.69335787
65 to 69	0.017505952	2.637220638	83586.43391	4.014656443	18.28477903	0.011976975	2.697477526	88430.20026	4.303772578	19.56395438
70 to 74	0.026303068	2.614019249	76642.3624	3.608552379	14.69892385	0.021471389	2.640996979	83331.74556	3.968575597	15.58811936
75 to 79	0.039407509	2.636153636	67285.27827	3.081844655	11.37911052	0.030585542	2.718461954	74959.78101	3.507271109	12.02405709
80 to 84	0.073318579	2.577195754	55358.03228	2.357631146	8.27429164	0.070777746	2.630041802	64443.35872	2.77083209	8.54415852
85 to 89	0.13116491	2.432445938	38424.27653	1.445799158	5.810195576	0.126672415	2.456873978	45418.93876	1.734520852	6.035621148
90 to 94	0.226028534	2.191050612	19884.37436	0.612980321	4.027504101	0.213039482	2.242569829	24296.88869	0.777103855	4.24508967
95 plus	0.321989518	3.110739059	6285.487421	0.198016932	3.110739059	0.308678666	3.256294943	8356.759802	0.28000617	3.256294943

Table 15: Nepal 2017 life table, by age and sex. mx=mortality rate, ax=mean person-years lived in an age interval among those who die in that age interval, lx=number of persons left alive at age x, nLx=person-years lived between age x and x+n, ex=life expectancy at age x.

Age Group	Male					Female				
	mx	ax	lx	nLx	ex	mx	ax	lx	nLx	ex
Early Neonatal	0.857698847	0.009562753	100000	0.019021223	68.89582254	0.655182275	0.00956896	100000	0.019058104	73.46765178
Late Neonatal	0.069528149	0.028747944	98368.70811	0.056482671	70.0188507	0.063375134	0.028749641	98751.44542	0.056712462	74.37719791
Post Neonatal	0.007150229	0.461135896	97976.08037	0.901622624	70.24182188	0.009333163	0.460980824	98392.09483	0.904539719	74.59124671
1 to 4	0.001005977	1.998658697	97331.64731	3.885446351	69.78058847	0.00101065	1.998652467	97548.03668	3.894047342	74.30929945
5 to 9	0.0005273	2.498901459	96940.93582	4.840663888	66.05388009	0.000450112	2.499062267	97154.59833	4.852268147	70.60220561
10 to 14	0.000472819	2.713853592	96685.7324	4.829066974	61.22169388	0.000366981	2.591591109	96936.20981	4.842530692	65.75556314
15 to 19	0.001017026	2.693843843	96457.43541	4.811588426	56.36015315	0.000613627	2.639973296	96758.51523	4.830930918	60.87155751
20 to 24	0.001431351	2.55538551	95968.09829	4.781676819	51.6335521	0.00078364	2.585811287	96462.09482	4.813999603	56.05042377
25 to 29	0.001415634	2.530120225	95283.76134	4.747594862	46.98567526	0.000944159	2.611428408	96084.88033	4.793436554	51.26013808
30 to 34	0.001660926	2.602384772	94611.86682	4.711837768	42.30109337	0.001300709	2.649149108	95632.35248	4.767045255	46.49006546
35 to 39	0.002266406	2.658044797	93829.53398	4.666720296	37.63172568	0.001897781	2.65954629	95012.38597	4.729622171	41.77572072
40 to 44	0.003447937	2.685798115	92772.32024	4.601917926	33.0296601	0.002803096	2.660342233	94114.99708	4.675101642	37.14803452
45 to 49	0.005494755	2.698455914	91186.49574	4.50241765	28.55635397	0.004150479	2.665433405	92804.9851	4.595733987	32.63376393
50 to 54	0.009065944	2.688845767	88714.46928	4.344761604	24.27543265	0.006300315	2.650175293	90898.59396	4.478669045	28.26094471
55 to 59	0.014725893	2.668455285	84779.75306	4.098431815	20.2755986	0.009134559	2.655721603	88079.21693	4.311713068	24.07922174
60 to 64	0.023614649	2.632986619	78752.82495	3.729509088	16.62126004	0.014130419	2.652995034	84145.41481	4.072388837	20.07921957
65 to 69	0.036170388	2.597356547	69961.77347	3.218954317	13.37781049	0.021889415	2.643868885	78399.34573	3.728056074	16.35513882
70 to 74	0.055415333	2.553890876	58345.66419	2.569918883	10.52456866	0.035083345	2.618620684	70256.02847	3.24251792	12.94368519
75 to 79	0.084033086	2.495951679	44145.79255	1.824701805	8.091464007	0.056480269	2.575007522	58907.53435	2.591570426	9.933405336
80 to 84	0.128973775	2.412734661	28866.10029	1.083396371	6.060522094	0.092499056	2.502235344	44318.36934	1.801532217	7.358922026
85 to 89	0.194847255	2.267298882	14952.07643	0.48863964	4.469307726	0.154324888	2.374675618	27726.91496	0.988027019	5.274263145
90 to 94	0.287488272	2.067777746	5471.01544	0.148703965	3.307495245	0.243041863	2.17755404	12548.72399	0.372859744	3.799144177
95 plus	0.370674791	2.698187078	1210.681234	0.032747573	2.698187078	0.335769751	2.979273821	3523.323961	0.105290835	2.979273821

Table 15: Nepal 2100 life table, by age and sex. mx=mortality rate, ax=mean person-years lived in an age interval among those who die in that age interval, lx=number of persons left alive at age x, nLx=person-years lived between age x and x+n, ex=life expectancy at age x.

Age Group	Male					Female				
	mx	ax	lx	nLx	ex	mx	ax	lx	nLx	ex
Early Neonatal	0.131396993	0.009585014	100000	0.019153941	78.63607455	0.105833071	0.009585797	100000	0.019158634	83.52484607
Late Neonatal	0.00707333	0.028765172	99748.35971	0.057377793	78.81470686	0.00885611	0.02876468	99797.25866	0.057402977	83.67494767
Post Neonatal	0.000843881	0.461583888	99707.77968	0.920231153	78.78915443	0.001343379	0.461548404	99746.42853	0.920375687	83.65993966
1 to 4	0.000135477	1.999819364	99630.13828	3.984126363	77.9267695	0.000134686	1.999820419	99622.81604	3.983839865	82.83967586
5 to 9	8.66984E-05	2.499819378	99576.18977	4.97773084	73.96765355	8.41011E-05	2.499824789	99569.18702	4.977413124	78.88305401
10 to 14	0.000122991	3.015203487	99533.04749	4.975422191	68.99853297	9.94081E-05	2.720089567	99527.34143	4.975234038	73.9150907
15 to 19	0.000376092	2.757470726	99471.87173	4.969402642	64.03903437	0.000183633	2.628193668	99477.90192	4.971727955	68.9503964
20 to 24	0.000589058	2.565505721	99285.02089	4.957157923	59.15396577	0.000210228	2.586010702	99386.63828	4.966818186	64.01108126
25 to 29	0.000576485	2.49528742	98993.14904	4.942615587	54.31998613	0.000276743	2.64150704	99282.26298	4.960897145	59.07542024
30 to 34	0.000665673	2.570886784	98708.51353	4.927513536	49.46831269	0.000418163	2.67891631	99145.08515	4.952418229	54.15312668
35 to 39	0.000847551	2.685494522	98381.17923	4.909322649	44.62316689	0.000603722	2.690107619	98938.25786	4.93998465	49.26023279
40 to 44	0.00129545	2.735705744	97966.48015	4.88387151	39.7995177	0.000925782	2.72642876	98640.44639	4.921624454	44.40015295
45 to 49	0.002156009	2.764162493	97336.27042	4.843362525	35.03770185	0.001570039	2.725517515	98185.43814	4.891778681	39.5921432
50 to 54	0.003901411	2.773857465	96296.21316	4.773311449	30.38325049	0.00261099	2.674248575	97418.50319	4.841526924	34.88075961
55 to 59	0.007304003	2.72819232	94441.73308	4.645076819	25.92017356	0.003774866	2.688514496	96157.02394	4.766141635	30.30089261
60 to 64	0.012278673	2.67102991	91066.33	4.427327633	21.77186089	0.005957398	2.655257851	94363.49118	4.653338109	25.82320401
65 to 69	0.018930622	2.637225589	85669.20245	4.101380063	17.96306234	0.008578155	2.678457513	91600.20873	4.490765427	21.51734147
70 to 74	0.027943166	2.618819994	77996.68753	3.659459408	14.46203249	0.013983339	2.716400134	87768.78243	4.25326843	17.33548262
75 to 79	0.043842886	2.608576712	67920.7249	3.079500332	11.21236246	0.025589102	2.735077753	81865.98894	3.870521802	13.38300748
80 to 84	0.073607863	2.5440416	54711.807	2.326405017	8.296423635	0.05173717	2.655582267	72082.97273	3.219368672	9.823848851
85 to 89	0.131010852	2.433684603	38057.44952	1.436735269	5.833740462	0.100072421	2.54405404	55717.2544	2.248258525	6.908641923
90 to 94	0.225621041	2.190328219	19869.96757	0.615866201	4.041122066	0.182696583	2.300320848	33852.13134	1.145637027	4.766499628
95 plus	0.321588908	3.118542038	6391.758135	0.203723707	3.118542038	0.280755216	3.582242082	13555.26234	0.497345897	3.582242082

Table 15: Pakistan 2017 life table, by age and sex. mx=mortality rate, ax=mean person-years lived in an age interval among those who die in that age interval, lx=number of persons left alive at age x, nLx=person-years lived between age x and x+n, ex=life expectancy at age x.

Age Group	Male					Female				
	mx	ax	lx	nLx	ex	mx	ax	lx	nLx	ex
Early Neonatal	1.583400366	0.009540511	100000	0.018889857	65.91886166	1.371849918	0.009546995	100000	0.018928018	66.93943722
Late Neonatal	0.122863541	0.028733232	97009.54627	0.055616985	67.93074732	0.138076068	0.028729035	97403.67025	0.055818517	68.7039167
Post Neonatal	0.013573871	0.460679573	96326.50297	0.883823997	68.35476214	0.015788515	0.46052225	96633.157	0.885732747	69.1940183
1 to 4	0.002350534	1.996865959	95127.64601	3.787286573	68.28714335	0.002515975	1.996645372	95235.38043	3.790320317	69.2794017
5 to 9	0.000964585	2.497990449	94238.09438	4.700565504	64.91327553	0.00118595	2.497529273	94282.24862	4.700166441	65.95989144
10 to 14	0.000897682	2.569757017	93784.89222	4.679043299	60.21501782	0.000856074	2.581060218	93724.96127	4.67656947	61.33726968
15 to 19	0.001279759	2.636003034	93364.97862	4.654177009	55.47403988	0.001538147	2.671716289	93324.66703	4.649587828	56.58902117
20 to 24	0.001757993	2.578299165	92769.44315	4.618810984	50.81229838	0.002153022	2.569385715	92609.57384	4.606380558	52.00456999
25 to 29	0.001967527	2.521049361	91957.62639	4.575570315	46.23685654	0.002298783	2.513663399	91618.01273	4.554877519	47.53842136
30 to 34	0.001991823	2.644031858	91057.65299	4.531629729	41.66768023	0.002357811	2.548734517	90571.29053	4.502557951	43.05749721
35 to 39	0.003412879	2.638041442	90155.43773	4.471752413	37.05698026	0.002920628	2.593134548	89510.15931	4.444292741	38.53645185
40 to 44	0.004369762	2.632560847	88630.28302	4.386184564	32.64715037	0.003763913	2.566888356	88213.01894	4.37066337	34.06328564
45 to 49	0.006431006	2.696449419	86715.55862	4.272564405	28.3073233	0.004263295	2.716092752	86569.51095	4.286777133	29.6591681
50 to 54	0.011009502	2.638258181	83971.91317	4.092386424	24.14084626	0.008512753	2.657417981	84744.31164	4.154492298	25.23733899
55 to 59	0.014919903	2.689768261	79475.93581	3.841707649	20.35298021	0.011358239	2.730820005	81213.6509	3.958843357	21.21563776
60 to 64	0.028094372	2.603100548	73760.99465	3.456161491	16.71772198	0.023396108	2.63101789	76727.69882	3.635480802	17.29298958
65 to 69	0.035732854	2.561532781	64088.51127	2.9488595	13.84465375	0.031000872	2.60721728	68248.42327	3.177789311	14.11036678
70 to 74	0.05429227	2.525691018	53610.1254	2.365206799	11.05045884	0.052430315	2.555822794	58445.15107	2.59234687	11.03765921
75 to 79	0.069753651	2.507695931	40868.7219	1.743593017	8.715827672	0.07063885	2.521878487	44949.89829	1.915673486	8.58744278
80 to 84	0.1205618	2.466328852	28837.24003	1.107753178	6.324170817	0.125525077	2.473723573	31560.72091	1.202048735	6.17518566
85 to 89	0.18640106	2.287316409	15642.73162	0.521693294	4.616333816	0.19299114	2.283447172	16669.6252	0.549595383	4.521009986
90 to 94	0.279697126	2.084391723	6029.042506	0.166823122	3.386570923	0.281943343	2.087692376	6204.790144	0.171443347	3.361419705
95 plus	0.364636942	2.743657351	1404.321472	0.038761555	2.743657351	0.369350507	2.709678987	1428.398613	0.039051023	2.709678987

Table 15: Pakistan 2100 life table, by age and sex. mx=mortality rate, ax=mean person-years lived in an age interval among those who die in that age interval, lx=number of persons left alive at age x, nLx=person-years lived between age x and x+n, ex=life expectancy at age x.

Age Group	Male					Female				
	mx	ax	lx	nLx	ex	mx	ax	lx	nLx	ex
Early Neonatal	0.300645345	0.009579826	100000	0.019122921	79.22577074	0.267600832	0.009580839	100000	0.019128967	79.01042301
Late Neonatal	0.017831851	0.028762204	99425.41553	0.057174339	79.66296621	0.028474839	0.028759269	99488.30239	0.057193	79.39647833
Post Neonatal	0.001693844	0.461523508	99323.49566	0.916325343	79.68692816	0.002036839	0.461499142	99325.51065	0.91619896	79.46868805
1 to 4	0.000251063	1.99966525	99168.39496	3.964745581	78.88707429	0.000233103	1.999689196	99139.02709	3.963713866	78.69353964
5 to 9	0.000228392	2.499524183	99068.90368	4.950620162	74.96407877	0.00027786	2.499421125	99046.68333	4.948898866	74.76485718
10 to 14	0.000298329	2.698077739	98955.92894	4.944390571	70.04650409	0.000278954	2.747463407	98909.30952	4.942276579	69.86483435
15 to 19	0.000501667	2.695876519	98808.4946	4.934698838	65.14680847	0.00052329	2.668690428	98771.7158	4.932548513	64.95788774
20 to 24	0.000747365	2.604524257	98561.12798	4.919232488	60.30306251	0.000679363	2.58634943	98513.88801	4.917631342	60.12054067
25 to 29	0.000859462	2.540442787	98193.72254	4.899365196	55.51852941	0.000799611	2.555060684	98180.2538	4.899434964	55.31547709
30 to 34	0.000949313	2.62531126	97772.96813	4.877644073	50.74581089	0.000883633	2.577164581	97789.08342	4.878982725	50.52548856
35 to 39	0.001420305	2.630264039	97310.55694	4.849206071	45.9734398	0.001091643	2.609085575	97358.71304	4.855309914	45.73626021
40 to 44	0.001831102	2.64478628	96622.94839	4.810418326	41.2800827	0.001499032	2.623655235	96829.39227	4.824388341	40.97009203
45 to 49	0.002715626	2.703244551	95743.6869	4.757568209	36.63295274	0.002083751	2.781553422	96107.52697	4.783150616	36.25474858
50 to 54	0.004573012	2.669766614	94454.9053	4.673168984	32.09283206	0.004195467	2.677565903	95114.83358	4.709779861	31.59898056
55 to 59	0.006734714	2.711522558	92326.00984	4.54608225	27.76699971	0.005515873	2.74515959	93148.99914	4.600084868	27.20278656
60 to 64	0.01133337	2.633708468	89287.09759	4.348366849	23.61696301	0.010671769	2.63711368	90627.96505	4.420713624	22.87732801
65 to 69	0.015011008	2.628281286	84396.01107	4.075486492	19.82625568	0.013593687	2.668371509	85944.96432	4.166129217	18.96376729
70 to 74	0.022550726	2.594527181	78338.31542	3.716747776	16.15716182	0.0235	2.64501644	80356.54297	3.810249675	15.08619821
75 to 79	0.030037331	2.632430759	70025.63057	3.270702401	12.76772207	0.036443714	2.693510077	71549.60559	3.306617859	11.60237128
80 to 84	0.05459321	2.622891528	60296.35416	2.672628576	9.407377321	0.074628301	2.617064332	59915.12283	2.556863394	8.337931755
85 to 89	0.106317482	2.50648773	45910.3335	1.821275852	6.535899808	0.131607195	2.442378312	41508.35429	1.570596535	5.901030933
90 to 94	0.199527301	2.23171706	26888.39305	0.871127467	4.423412308	0.218384817	2.231125455	21732.04682	0.688957021	4.166028495
95 plus	0.300184601	3.337286882	9775.122548	0.329671973	3.337286882	0.313493762	3.207160528	7301.024757	0.241574305	3.207160528

Table 15: Southeast Asia, East Asia, and Oceania 2017 life table, by age and sex. mx=mortality rate, ax=mean person-years lived in an age interval among those who die in that age interval, lx=number of persons left alive at age x, nLx=person-years lived between age x and x+n, ex=life expectancy at age x.										
**Age Group**	**Male**					**Female**				
	**mx**	**ax**	**lx**	**nLx**	**ex**	**mx**	**ax**	**lx**	**nLx**	**ex**
Early Neonatal	0.314160443	0.009579412	100000	0.019120424	72.96437755	0.250943037	0.00958135	100000	0.019132008	78.56239724
Late Neonatal	0.027924095	0.02875942	99399.31316	0.057142731	73.38607568	0.022565165	0.028760899	99519.89626	0.057220871	78.9221722
Post Neonatal	0.00631266	0.461195395	99239.74797	0.913603363	73.44649098	0.00494645	0.461292448	99390.77682	0.915570502	78.96712904
1 to 4	0.000872062	1.998837251	98663.02504	3.939645843	72.94982848	0.000763088	1.998982549	98937.89655	3.951482173	78.4031966
5 to 9	0.000497933	2.49896264	98319.46701	4.909858893	69.19775475	0.000355347	2.499259694	98636.36558	4.927439623	74.63676584
10 to 14	0.000449489	2.61147547	98074.99018	4.898490435	64.36401914	0.00029561	2.510082626	98461.27119	4.919442663	69.76504845
15 to 19	0.000741772	2.64463456	97854.80895	4.884207138	59.50296498	0.000370863	2.57038079	98315.84782	4.911367039	64.86452645
20 to 24	0.000970007	2.606627428	97492.51291	4.863334942	54.71425064	0.000422277	2.587306515	98133.70346	4.901691195	59.98014583
25 to 29	0.001248232	2.589218564	97020.76754	4.836484402	49.96760438	0.000549865	2.611133437	97926.7165	4.889912667	55.10145226
30 to 34	0.001520386	2.587388541	96417.06458	4.803234628	45.26424335	0.000718865	2.623308864	97657.83791	4.874563642	50.24596549
35 to 39	0.001910771	2.629576882	95686.79185	4.76276766	40.58993146	0.000981205	2.641707373	97307.42368	4.854138893	45.41745129
40 to 44	0.002752965	2.686168871	94776.74477	4.708842817	35.95440549	0.001397395	2.68456912	96831.13574	4.825942159	40.62784345
45 to 49	0.004471352	2.682646975	93480.43482	4.626088486	31.41570881	0.002244867	2.685967918	96156.76563	4.782992316	35.89393291
50 to 54	0.006915209	2.633491423	91411.99058	4.497008074	27.06581663	0.003462531	2.64453736	95083.05788	4.715692731	31.26890036
55 to 59	0.009397631	2.685779469	88302.3123	4.321141744	22.92616042	0.004773796	2.71300178	93450.25801	4.622051736	26.76899743
60 to 64	0.016594386	2.68237728	84241.62692	4.056094393	18.90173915	0.008692134	2.727293096	91243.82986	4.473815486	22.35066966
65 to 69	0.027358571	2.613565555	77511.18191	3.638052967	15.30999641	0.015290308	2.675563715	87355.25117	4.217861541	18.22411725
70 to 74	0.039407502	2.588772857	67558.89397	3.084858964	12.18024853	0.02435768	2.666810891	80906.36186	3.827798194	14.46335206
75 to 79	0.063014944	2.564033192	55403.93541	2.40161747	9.284491993	0.042794735	2.644448486	71583.60003	3.251460391	10.99958498
80 to 84	0.105746325	2.476356103	40273.29737	1.589580173	6.809558838	0.078394303	2.555952844	57671.12018	2.41998861	8.015144696
85 to 89	0.169904344	2.32708848	23468.74673	0.807042741	4.912965388	0.136991633	2.422048362	38704.01299	1.430240558	5.690720139
90 to 94	0.264375305	2.116929913	9760.668123	0.276975675	3.545859096	0.224861564	2.219070418	19116.05918	0.588131932	4.040903698
95 plus	0.352718519	2.835162402	2439.810471	0.069183588	2.835162402	0.319621483	3.128785942	5894.478663	0.184460308	3.128785942

Table 15: Southeast Asia, East Asia, and Oceania 2100 life table, by age and sex. mx=mortality rate, ax=mean person-years lived in an age interval among those who die in that age interval, lx=number of persons left alive at age x, nLx=person-years lived between age x and x+n, ex=life expectancy at age x.

Age Group	Male					Female				
	mx	ax	lx	nLx	ex	mx	ax	lx	nLx	ex
Early Neonatal	0.072040799	0.009586833	100000	0.019164841	80.08974465	0.062365753	0.00958713	100000	0.019166618	84.03940814
Late Neonatal	0.006082494	0.028765445	99861.94764	0.057444768	80.18117422	0.005925043	0.028765489	99880.47391	0.057455685	84.12069891
Post Neonatal	0.001160485	0.461561397	99827.01066	0.921196943	80.15166112	0.00087972	0.461581342	99846.43425	0.921495571	84.09181356
1 to 4	0.000183996	1.999754672	99720.12317	3.987337677	79.31373515	0.000166336	1.999778218	99765.37825	3.989288066	83.23644318
5 to 9	0.000121767	2.499746319	99646.7706	4.980822384	75.37059538	0.000113406	2.499763737	99699.0334	4.983538873	79.29046567
10 to 14	0.000158119	2.894459715	99586.13157	4.977631586	70.41491422	0.000129719	2.632750782	99642.52745	4.980590577	74.33397938
15 to 19	0.0004043	2.707546187	99507.44178	4.970768233	65.46824831	0.000189848	2.601397827	99577.9328	4.976630206	69.3804548
20 to 24	0.000564666	2.611925053	99306.52166	4.958649807	60.59509354	0.000221773	2.606531591	99483.4728	4.971533645	64.44383022
25 to 29	0.0007185	2.58672978	99026.64143	4.942763766	55.75875607	0.000302154	2.645930916	99373.24654	4.965124171	59.51238009
30 to 34	0.000865185	2.583441488	98671.77101	4.923261359	50.94960047	0.000427171	2.636372888	99223.26888	4.956150935	54.59817483
35 to 39	0.001029857	2.617081439	98246.23253	4.90028105	46.15874956	0.00057403	2.649676466	99011.62548	4.943911191	49.70901492
40 to 44	0.001442861	2.679895328	97741.95245	4.87080128	41.38302527	0.000841338	2.70109164	98727.93146	4.926865297	44.84392121
45 to 49	0.002299471	2.719631727	97039.72988	4.826672581	36.66246892	0.001389991	2.701679996	98313.59946	4.900021804	40.02107754
50 to 54	0.003910092	2.676434182	95931.02314	4.753362893	32.05341558	0.002197774	2.658853548	97632.93403	4.856663161	35.28018523
55 to 59	0.005720793	2.695730423	94074.99781	4.642560334	27.63065799	0.003102823	2.698066183	96566.53777	4.794044618	30.63871311
60 to 64	0.009620911	2.693267228	91424.35396	4.472143733	23.35075128	0.005190289	2.701643152	95081.2166	4.698122107	26.07327458
65 to 69	0.015813286	2.633729037	87131.58931	4.199931489	19.36376845	0.008507824	2.680217526	92646.45616	4.542734581	21.68429755
70 to 74	0.022075971	2.629475542	80523.27298	3.826830584	15.72778103	0.01316716	2.723405473	88794.59692	4.310854522	17.50386642
75 to 79	0.035666267	2.626374203	72121.74131	3.32715862	12.24802834	0.025203479	2.723396917	83139.8747	3.932498977	13.50441624
80 to 84	0.059965797	2.565834103	60374.91601	2.641473001	9.108295101	0.049530199	2.644220012	73291.50411	3.285376716	9.946688794
85 to 89	0.11303855	2.486963176	44897.13467	1.760721005	6.354097257	0.097087649	2.553154566	57213.81865	2.319212606	6.990841033
90 to 94	0.206506856	2.219745106	25631.7982	0.82346657	4.325123989	0.179457834	2.307790293	35096.06738	1.190744208	4.813838364
95 plus	0.305880897	3.281069736	9120.622745	0.30530015	3.281069736	0.27781758	3.611588119	14121.81181	0.517384002	3.611588119

Table 15: East Asia 2017 life table, by age and sex. mx=mortality rate, ax=mean person-years lived in an age interval among those who die in that age interval, lx=number of persons left alive at age x, nLx=person-years lived between age x and x+n, ex=life expectancy at age x.

Age Group	Male					Female				
	mx	ax	lx	nLx	ex	mx	ax	lx	nLx	ex
Early Neonatal	0.157596769	0.009584211	100000	0.019149129	74.59358523	0.136196538	0.009584867	100000	0.019153058	79.93460827
Late Neonatal	0.015302036	0.028762902	99698.21625	0.057335375	74.80016817	0.014399551	0.028763151	99739.14222	0.0573604	80.12446539
Post Neonatal	0.004488711	0.461324965	99610.48167	0.917788163	74.80849025	0.003478524	0.461396727	99656.546	0.918640629	80.13331513
1 to 4	0.000565778	1.999245629	99198.51493	3.963454062	74.19396125	0.000484175	1.999354433	99336.99582	3.969634617	79.46631877
5 to 9	0.000451301	2.49905979	98974.27281	4.943134465	70.35752986	0.000296862	2.499381537	99144.79713	4.953562646	75.61649454
10 to 14	0.000413953	2.509950653	98751.18973	4.932475329	65.51082543	0.000249817	2.427385448	98997.74509	4.946708154	70.72510359
15 to 19	0.000473121	2.567145992	98547.00913	4.92168561	60.64135314	0.00021049	2.486459845	98874.16834	4.941094283	65.81046149
20 to 24	0.000568953	2.686453193	98314.1543	4.909245547	55.77888852	0.000236742	2.666313883	98770.16353	4.93578125	60.8771372
25 to 29	0.000987173	2.637915564	98034.84225	4.890338915	50.93014146	0.000400335	2.645203307	98653.3131	4.928019996	55.94608033
30 to 34	0.001231494	2.574284206	97552.08366	4.863077094	46.16910424	0.000517341	2.585062713	98456.02778	4.916658831	51.05287444
35 to 39	0.001441305	2.6410095	96953.20334	4.831234102	41.43836331	0.000614224	2.649518631	98201.66988	4.903004951	46.17840171
40 to 44	0.002234981	2.727467229	96256.88359	4.788523432	36.71899154	0.000963239	2.752233235	97900.51753	4.884450408	41.31228605
45 to 49	0.003954044	2.698581694	95186.67721	4.716415996	32.101118	0.001794775	2.721239417	97430.03213	4.851659153	36.49846902
50 to 54	0.006193473	2.611939002	93321.8395	4.598086841	27.68858202	0.00290969	2.629410948	96559.2798	4.794890746	31.80302583
55 to 59	0.007635275	2.716325103	90474.15554	4.446185707	23.47776405	0.003681202	2.761130313	95164.1439	4.719312818	27.23066292
60 to 64	0.015143638	2.710940465	87079.57464	4.208117074	19.28699568	0.007781187	2.764066182	93426.91808	4.591465937	22.68560071
65 to 69	0.025906494	2.619596779	80707.51334	3.801005723	15.59556108	0.014412697	2.681797197	89854.37338	4.347473489	18.47754891
70 to 74	0.036952226	2.598900429	70861.79285	3.254398486	12.39832975	0.022775347	2.677439982	83588.99518	3.96950056	14.6613427
75 to 79	0.06063882	2.578993483	58838.65861	2.565424572	9.400734292	0.041066935	2.659710958	74549.59847	3.400709323	11.11427157
80 to 84	0.104593899	2.485435629	43287.0824	1.713797158	6.85184533	0.077484505	2.563678688	60587.07545	2.548425095	8.062618565
85 to 89	0.168688975	2.330154756	25369.04584	0.874692863	4.936779243	0.135863436	2.425323962	40847.29071	1.513245162	5.720414926
90 to 94	0.263224627	2.119339419	10620.05339	0.302054831	3.558603767	0.223652123	2.221771566	20295.91161	0.625992687	4.058197569
95 plus	0.351814324	2.842466964	2671.881479	0.075964751	2.842466964	0.318534401	3.139508054	6300.611094	0.197864076	3.139508054

Table 15: East Asia 2100 life table, by age and sex. mx=mortality rate, ax=mean person-years lived in an age interval among those who die in that age interval, lx=number of persons left alive at age x, nLx=person-years lived between age x and x+n, ex=life expectancy at age x.

Age Group	Male					Female				
	mx	ax	lx	nLx	ex	mx	ax	lx	nLx	ex
Early Neonatal	0.024467139	0.009588291	100000	0.019173584	82.51073626	0.021677056	0.009588377	100000	0.019174097	85.9193639
Late Neonatal	0.00186841	0.028766608	99953.09217	0.057504168	82.53024224	0.00208654	0.028766548	99958.44037	0.057506884	85.93582126
Post Neonatal	0.000590482	0.461601889	99942.34933	0.922503958	82.4815722	0.000419207	0.461614056	99946.44267	0.92261466	85.88857713
1 to 4	8.27808E-05	1.999889626	99887.89366	3.994854423	81.60297061	6.72038E-05	1.999910395	99907.77576	3.995774043	84.99829862
5 to 9	6.7039E-05	2.499860335	99854.82971	4.991904922	77.62930139	6.46047E-05	2.499865407	99880.92798	4.993240123	81.02057328
10 to 14	0.000113701	2.87670966	99821.36986	4.989800653	72.65442156	8.73619E-05	2.571640818	99848.67945	4.991359682	76.04585655
15 to 19	0.000211771	2.629526882	99764.65799	4.985736536	67.694023	7.93566E-05	2.4816164	99805.09727	4.989259572	71.07784472
20 to 24	0.000251703	2.679592377	99659.10453	4.980094572	62.76278091	8.19015E-05	2.660984263	99765.51546	4.987318918	66.1050216
25 to 29	0.00047523	2.674970212	99533.8333	4.97115269	57.83807431	0.000141764	2.724076792	99724.6866	4.984596237	61.1309452
30 to 34	0.00059978	2.602034724	99298.01343	4.957657372	52.96840325	0.000205075	2.651919087	99654.06758	4.980265642	56.17212022
35 to 39	0.000647098	2.610127559	99001.3348	4.942397631	48.11885438	0.000251576	2.64883169	99551.99178	4.974653165	51.22674089
40 to 44	0.000912834	2.743651295	98681.91037	4.923979071	43.26576609	0.000381138	2.79831547	99426.87339	4.967184434	46.28768771
45 to 49	0.001742242	2.769805516	98233.03246	4.89256366	38.44996488	0.000808165	2.78955356	99237.62024	4.952977518	41.37023332
50 to 54	0.00310241	2.646761785	97382.44031	4.833806951	33.75896448	0.001455124	2.655370899	98837.65397	4.925049166	36.52502319
55 to 59	0.003886942	2.72611614	95887.56538	4.752329404	29.23859304	0.001865509	2.784567216	98121.86215	4.885757431	31.76992821
60 to 64	0.007350909	2.740282246	94048.64202	4.625878129	24.74989606	0.003895154	2.772867198	97212.08758	4.818911253	27.03898174
65 to 69	0.013049819	2.643251032	90668.296	4.399140981	20.55980792	0.007260269	2.687078964	95338.32351	4.688099584	22.51229704
70 to 74	0.017006347	2.660683446	85007.7749	4.089250961	16.73196544	0.010476147	2.748813397	91951.52935	4.491880643	18.23564616
75 to 79	0.029698465	2.679460588	78149.32549	3.660401242	12.95721358	0.021500846	2.765727947	87265.66941	4.164374941	14.06225376
80 to 84	0.055064272	2.600951236	67526.92513	2.996835759	9.553330482	0.045388749	2.659634484	78379.45966	3.547264451	10.33391193
85 to 89	0.105587599	2.51164697	51771.26414	2.076228389	6.651557978	0.090740561	2.577333972	62527.04955	2.573407233	7.264451259
90 to 94	0.19751092	2.225721492	31189.57105	1.028201553	4.493543927	0.171532047	2.318573402	39730.77576	1.372114615	4.982568787
95 plus	0.298236136	3.377622391	11979.0266	0.419339649	3.377622391	0.270313841	3.717939711	16789.84376	0.636579608	3.717939711

Table 15: China 2017 life table, by age and sex. mx=mortality rate, ax=mean person-years lived in an age interval among those who die in that age interval, lx=number of persons left alive at age x, nLx=person-years lived between age x and x+n, ex=life expectancy at age x.

Age Group	Male					Female				
	mx	ax	lx	nLx	ex	mx	ax	lx	nLx	ex
Early Neonatal	0.153631935	0.009584332	100000	0.019149857	74.66828598	0.132802817	0.009584971	100000	0.019153681	79.98474876
Late Neonatal	0.014949115	0.028763	99705.79737	0.057340317	74.86940153	0.01406677	0.028763243	99745.63396	0.057364683	80.1695185
Post Neonatal	0.004418587	0.461329947	99620.0789	0.917906283	74.87626371	0.003410111	0.461401587	99664.94055	0.918747011	80.17686994
1 to 4	0.000558225	1.9992557	99214.49584	3.964152438	74.25717868	0.000477853	1.999362862	99351.63872	3.970269947	79.5049626
5 to 9	0.000455697	2.499050632	98993.20847	4.944025867	70.41870263	0.000298472	2.499378183	99161.91912	4.954398172	75.65324971
10 to 14	0.000416964	2.497856709	98767.91179	4.933248775	65.57363276	0.000251142	2.41867358	99014.04457	4.947494939	70.76250328
15 to 19	0.000453423	2.555978436	98562.2136	4.922655708	60.70526645	0.000201133	2.473536528	98889.79257	4.941978415	65.84837174
20 to 24	0.000541167	2.700580751	98339.00978	4.910839455	55.83723731	0.000226178	2.677641612	98790.39317	4.936926458	60.91213268
25 to 29	0.000979076	2.642861988	98073.25224	4.892371941	50.98121231	0.000394796	2.648408755	98678.73107	4.929360164	55.97802397
30 to 34	0.001221333	2.570970007	97594.25455	4.865279301	46.21843604	0.00050893	2.580390831	98484.12249	4.918149925	51.08339514
35 to 39	0.001409737	2.639574587	97000.04677	4.833917369	41.48578621	0.000593724	2.64836405	98233.82434	4.904842995	46.20696721
40 to 44	0.002192784	2.730366098	96318.60035	4.792081212	36.76058751	0.000936554	2.757096501	97942.61386	4.886865368	41.33646289
45 to 49	0.00390475	2.700006717	95267.82079	4.720993403	32.13588117	0.001763201	2.724164113	97484.93668	4.854766009	36.51756401
50 to 54	0.006126548	2.609443657	93424.44655	4.603798291	27.71659799	0.002873008	2.627475208	96628.9558	4.798738647	31.81687837
55 to 59	0.007464223	2.721889203	90604.04172	4.454460835	23.49804178	0.003597055	2.768258833	95250.30435	4.724588594	27.23930422
60 to 64	0.015079665	2.714959102	87279.34702	4.218615655	19.28935064	0.00775773	2.768168311	93550.8948	4.597939736	22.68377058
65 to 69	0.025950831	2.619112927	80918.40605	3.810515704	15.5920874	0.014451107	2.680397498	89984.10799	4.353291008	18.47305826
70 to 74	0.036822014	2.599089082	71031.23409	3.263141164	12.39770295	0.022676381	2.677482693	83693.65863	3.975344149	14.65985706
75 to 79	0.06063442	2.580238297	59018.43673	2.573462121	9.39212468	0.041000489	2.661551608	74680.38728	3.407398186	11.10584984
80 to 84	0.104928041	2.485907214	43419.55587	1.71797473	6.839667985	0.07775961	2.564197324	60713.29712	2.552392548	8.04841566
85 to 89	0.169080737	2.329168029	25400.85151	0.875064653	4.929128335	0.13622644	2.424274716	40873.10908	1.513007378	5.710926194
90 to 94	0.263595248	2.118562742	10611.65025	0.301600332	3.554508835	0.224040594	2.220902054	20270.71112	0.624732766	4.052673315
95 plus	0.352105441	2.840119803	2664.405477	0.075690574	2.840119803	0.318883264	3.136083751	6279.736927	0.196997983	3.136083751

Table 15: China 2100 life table, by age and sex. mx=mortality rate, ax=mean person-years lived in an age interval among those who die in that age interval, lx=number of persons left alive at age x, nLx=person-years lived between age x and x+n, ex=life expectancy at age x.

Age Group	Male					Female				
	mx	ax	lx	nLx	ex	mx	ax	lx	nLx	ex
Early Neonatal	0.021524454	0.009588381	100000	0.019174125	82.63067515	0.019186055	0.009588453	100000	0.019174555	85.97673025
Late Neonatal	0.001635115	0.028766672	99958.73319	0.057507799	82.64557	0.001840799	0.028766615	99963.21585	0.057510038	85.9890899
Post Neonatal	0.000550183	0.461604751	99949.33134	0.92258557	82.59580205	0.000383737	0.461616575	99952.63083	0.922686893	85.94063242
1 to 4	7.89882E-05	1.999894682	99898.58964	3.995312496	81.71419953	6.37486E-05	1.999915002	99917.23427	3.996179949	85.04756718
5 to 9	6.68921E-05	2.499860641	99867.03724	4.992517033	77.73935494	6.40647E-05	2.499866532	99891.76467	4.99378863	81.06870405
10 to 14	0.000114025	2.861214356	99833.64685	4.990399954	72.76445409	8.74034E-05	2.55852647	99859.78305	4.991909172	76.0937838
15 to 19	0.000201605	2.618111168	99776.76775	4.986451161	67.80423718	7.38911E-05	2.458719535	99816.17758	4.98987515	71.12580192
20 to 24	0.000235361	2.692397717	99676.26858	4.98115809	62.86980004	7.56436E-05	2.676573892	99779.31888	4.988086247	66.1511232
25 to 29	0.000467343	2.684668997	99559.11112	4.972518576	57.94027978	0.000136171	2.737377387	99741.60645	4.985508555	61.17506936
30 to 34	0.000588272	2.602104209	99327.16498	4.959241891	53.06861715	0.000196016	2.656420238	99673.76613	4.981354461	56.21461944
35 to 39	0.000625441	2.606550061	99036.12234	4.944377291	48.21643052	0.000237407	2.647740102	99576.1846	4.976025917	51.26683738
40 to 44	0.000877204	2.749161475	98727.28037	4.926663206	43.35871517	0.000360333	2.80961514	99458.08346	4.968990314	46.32438637
45 to 49	0.001702753	2.776756393	98295.7086	4.896157156	38.53603617	0.000783619	2.80092344	99279.10226	4.955345358	41.40243527
50 to 54	0.003054811	2.645971025	97463.87548	4.838371916	33.83851524	0.001428789	2.656130953	98891.12494	4.928017013	36.55250851
55 to 59	0.003791534	2.731840402	95990.76327	4.75855051	29.31159754	0.00182042	2.793908834	98187.91411	4.889597986	31.79304291
60 to 64	0.007272758	2.744314796	94195.05354	4.634019483	24.81095961	0.003871165	2.77724062	97299.51516	4.823585283	27.05557856
65 to 69	0.012984287	2.642940742	90845.69712	4.40843852	20.61335128	0.007271995	2.686239024	95435.60011	4.692718381	22.52603033
70 to 74	0.016774827	2.662704794	85206.31328	4.101113818	16.78068484	0.010402799	2.749112522	92040.83992	4.496982121	18.25063736
75 to 79	0.029480601	2.682552798	78427.50734	3.675678614	12.99084973	0.021426873	2.767410817	87383.27207	4.170813322	14.07179231
80 to 84	0.054896877	2.602599282	67852.86006	3.013191293	9.575902987	0.045376838	2.659876068	78516.72656	3.553752589	10.33897582
85 to 89	0.105282676	2.512758461	52092.68379	2.091624774	6.667273201	0.090702231	2.577582143	62648.0152	2.578984997	7.268410314
90 to 94	0.197096216	2.225656545	31475.35847	1.039218061	4.502692931	0.171460134	2.318499107	39831.06967	1.376141319	4.985215988
95 plus	0.297874678	3.382872725	12145.0449	0.426403807	3.382872725	0.270239907	3.719634857	16853.04478	0.639701999	3.719634857

Table 15: North Korea 2017 life table, by age and sex. mx=mortality rate, ax=mean person-years lived in an age interval among those who die in that age interval, lx=number of persons left alive at age x, nLx=person-years lived between age x and x+n, ex=life expectancy at age x.

Age Group	Male					Female				
	mx	ax	lx	nLx	ex	mx	ax	lx	nLx	ex
Early Neonatal	0.4850403	0.009574175	100000	0.019089166	68.46090126	0.397202187	0.009576867	100000	0.019105225	74.84856219
Late Neonatal	0.045358966	0.028754611	99074.20219	0.0569273	69.08097868	0.040617589	0.028755919	99241.18841	0.057031016	75.40146318
Post Neonatal	0.011949829	0.460794941	98816.05107	0.90734364	69.20378278	0.009700434	0.460954734	99009.57854	0.910062693	75.52023151
1 to 4	0.001278169	1.998295775	97732.23597	3.899318512	69.04283311	0.001056754	1.998590996	98127.00369	3.916798993	75.27212145
5 to 9	0.000453745	2.499054699	97234.1231	4.856196505	65.38641176	0.000352181	2.49926629	97713.24218	4.881363412	71.58249563
10 to 14	0.000438291	2.887094209	97013.8208	4.846206874	60.52918639	0.00028829	2.657065595	97541.34527	4.873775404	66.70409441
15 to 19	0.001277327	2.708257554	96801.43647	4.825952406	55.65555344	0.000571059	2.642453981	97400.85041	4.863497654	61.79641055
20 to 24	0.001740109	2.53793847	96184.92172	4.788737071	50.99435112	0.000684596	2.565247418	97123.10763	4.848077623	56.96528372
25 to 29	0.001624432	2.508349294	95351.68069	4.748369079	46.41698057	0.000792805	2.601317112	96791.23533	4.830376306	52.15140017
30 to 34	0.001834051	2.595197546	94580.60071	4.708270661	41.77425972	0.001076533	2.651487934	96408.34879	4.808263755	47.34771731
35 to 39	0.002498216	2.662171041	93717.57861	4.658683262	37.13442966	0.001589466	2.673326265	95890.85915	4.776884299	42.5883851
40 to 44	0.003848235	2.690297451	92554.73318	4.586989643	32.5668516	0.002431031	2.673537494	95131.86549	4.72985172	37.90604215
45 to 49	0.006181922	2.693967215	90791.4869	4.475820194	28.14637346	0.003681643	2.667435288	93982.60817	4.659136759	33.33575094
50 to 54	0.010059431	2.679828448	88028.25359	4.301135194	23.94477106	0.005542605	2.657461689	92268.47272	4.554338486	28.90408198
55 to 59	0.016067712	2.653182414	83708.19997	4.033512085	20.04205819	0.008225341	2.660886907	89746.54174	4.402705923	24.6399966
60 to 64	0.024874307	2.623971477	77237.51611	3.64664922	16.49931501	0.012694837	2.663343983	86130.1067	4.182613159	20.56099137
65 to 69	0.038001893	2.582243188	68180.25457	3.122523547	13.34362149	0.020153156	2.652762274	80829.11396	3.859274921	16.73294748
70 to 74	0.055458355	2.54445809	56331.19321	2.479394459	10.60897899	0.032436909	2.63891935	73069.01039	3.39426572	13.22665362
75 to 79	0.084177521	2.48824384	42600.00273	1.758688317	8.210727351	0.055181397	2.584910079	62093.548	2.740988805	10.09752951
80 to 84	0.122375035	2.409114261	27815.95241	1.056355747	6.255261451	0.089283489	2.509615997	47036.59982	1.92627807	7.505383478
85 to 89	0.187600869	2.284064725	14905.8316	0.493980564	4.589949467	0.150359023	2.385396618	29948.28489	1.077134687	5.368038844
90 to 94	0.280863018	2.081956611	5651.311163	0.155384401	3.372553302	0.238897135	2.18704183	13865.04569	0.415830053	3.853568235
95 plus	0.365566393	2.735654793	1292.035675	0.035373413	2.735654793	0.332096165	3.012918236	3993.534682	0.120907504	3.012918236

Table 15: North Korea 2100 life table, by age and sex. mx=mortality rate, ax=mean person-years lived in an age interval among those who die in that age interval, lx=number of persons left alive at age x, nLx=person-years lived between age x and x+n, ex=life expectancy at age x.

Age Group	Male					Female				
	mx	ax	lx	nLx	ex	mx	ax	lx	nLx	ex
Early Neonatal	0.132903064	0.009584968	100000	0.019153667	78.32812904	0.104974832	0.009585824	100000	0.019158793	83.74810531
Late Neonatal	0.011711196	0.028763893	99745.52525	0.057368517	78.50803067	0.011532994	0.028763942	99798.92394	0.057399521	83.89665812
Post Neonatal	0.00262567	0.461457312	99678.37545	0.919204498	78.50328384	0.001981096	0.461503102	99732.75278	0.919979119	83.89451902
1 to 4	0.000274148	1.99963447	99437.26677	3.975313185	77.76799492	0.000223814	1.99970158	99550.62839	3.980244808	83.12265209
5 to 9	0.000101429	2.499788689	99328.41823	4.965162755	73.85057039	0.000103903	2.499783536	99461.62952	4.971790799	79.19475623
10 to 14	0.000142598	3.22462	99278.09663	4.962624981	68.88659885	0.000116422	2.796224377	99410.00735	4.969220878	74.23439027
15 to 19	0.000575091	2.753606234	99207.36618	4.953970669	63.93330733	0.000264585	2.64423437	99352.18935	4.964517258	69.27579599
20 to 24	0.00084529	2.558598327	98922.60752	4.93596584	59.1092811	0.000300684	2.556136625	99220.90593	4.957409074	64.36365022
25 to 29	0.000833916	2.523248836	98505.64019	4.915164861	54.34843574	0.000350659	2.621071991	99071.94736	4.94947341	59.45612755
30 to 34	0.000969199	2.577135312	98096.25074	4.89333774	49.56432735	0.000507216	2.642209476	98898.54788	4.939020343	54.55518176
35 to 39	0.001199105	2.649431411	97622.84122	4.867414574	44.79133074	0.000693338	2.666147283	98648.29564	4.924441478	49.68603337
40 to 44	0.001811429	2.707006804	97040.43994	4.831898604	40.04255369	0.001052686	2.709956055	98307.25371	4.903542138	44.84805703
45 to 49	0.002937811	2.72169289	96167.40866	4.776412919	35.37860139	0.00175621	2.687409016	97791.68398	4.869825991	40.06872986
50 to 54	0.004985798	2.69539437	94767.86074	4.684651329	30.8565637	0.002662434	2.655067887	96937.66804	4.816856128	35.39572281
55 to 59	0.007831852	2.649452004	92439.86126	4.538701604	26.55900777	0.003805312	2.645652878	95657.84707	4.740494456	30.83037614
60 to 64	0.011201179	2.65703013	88900.33944	4.331848416	22.50243685	0.005445675	2.657562181	93859.07117	4.633998557	26.36633257
65 to 69	0.0177124	2.632731111	84077.97866	4.035847583	18.63158733	0.008231702	2.676628921	91344.93017	4.48187387	22.01415888
70 to 74	0.025654553	2.626528204	76991.03436	3.630806943	15.09537749	0.013199131	2.724844938	87674.86294	4.256650751	17.81791493
75 to 79	0.041212608	2.59729956	67789.98998	3.088750415	11.78299315	0.024947638	2.701525602	82098.78179	3.884393047	13.83643225
80 to 84	0.063926069	2.536080951	55288.00511	2.398062402	8.861322078	0.045549791	2.627603701	72520.09074	3.278093059	10.30073397
85 to 89	0.118238756	2.471379327	40422.70259	1.569844536	6.198256951	0.090990533	2.575945449	57861.81748	2.380342343	7.243197944
90 to 94	0.212106868	2.211625236	22558.52715	0.718103844	4.239694846	0.171953862	2.318838792	36717.43406	1.266357171	4.968433184
95 plus	0.310499397	3.232168259	7822.427656	0.258519161	3.232168259	0.270738003	3.70890194	15449.11023	0.583022318	3.70890194

Table 15: Taiwan (Province of China) 2017 life table, by age and sex. mx=mortality rate, ax=mean person-years lived in an age interval among those who die in that age interval, lx=number of persons left alive at age x, nLx=person-years lived between age x and x+n, ex=life expectancy at age x.

Age Group	Male					Female				
	mx	ax	lx	nLx	ex	mx	ax	lx	nLx	ex
Early Neonatal	0.108752351	0.009585708	100000	0.019158097	76.56311461	0.088722198	0.009586322	100000	0.019161776	83.0128048
Late Neonatal	0.009865213	0.028764402	99791.65389	0.057398086	76.70376519	0.009002389	0.02876464	99829.99451	0.057421564	83.13497715
Post Neonatal	0.00160328	0.461529941	99735.03111	0.920160051	76.68976306	0.001413007	0.461543458	99778.30269	0.920640116	83.12049841
1 to 4	0.000234935	1.999686754	99587.50921	3.981629207	75.87939976	0.00018423	1.999754361	99648.21988	3.984460475	82.30512033
5 to 9	0.000114695	2.499761052	99493.96609	4.973272164	71.94885589	9.88077E-05	2.499794151	99574.81302	4.977511023	78.36432057
10 to 14	0.000158836	2.986565121	99436.92593	4.970256645	66.98869492	9.58778E-05	2.715383743	99525.63197	4.975191815	73.40181005
15 to 19	0.000486357	2.708097241	99357.98098	4.962367686	62.03954445	0.000198114	2.662759333	99477.93118	4.971594581	68.43570232
20 to 24	0.000646828	2.579280191	99116.63403	4.948084035	57.18398636	0.000251073	2.606313733	99379.43695	4.965987402	63.50087923
25 to 29	0.000736348	2.655509284	98796.58017	4.931315814	52.36084162	0.000326981	2.664068768	99254.75474	4.958950121	58.57735997
30 to 34	0.001204307	2.704428869	98433.467	4.908104936	47.54416106	0.000510014	2.671523145	99092.60769	4.948753667	53.66883282
35 to 39	0.001936668	2.698856805	97842.3887	4.87041497	42.8149801	0.000750042	2.654016426	98840.21665	4.93333031	48.79902623
40 to 44	0.003100744	2.679477154	96899.17491	4.810348164	38.20536297	0.001070869	2.671876894	98470.20165	4.911265684	43.97237484
45 to 49	0.004723061	2.645743415	95407.67428	4.717927041	33.76057627	0.001647551	2.682328148	97944.28149	4.878585527	39.19408158
50 to 54	0.006650901	2.619276089	93179.53222	4.586363162	29.50439208	0.002545903	2.658006905	97140.53581	4.828239491	34.49610115
55 to 59	0.009009181	2.608146505	90129.53018	4.411429003	25.4139543	0.003650914	2.653153839	95911.37012	4.75483077	29.90401756
60 to 64	0.012216261	2.602351803	86155.81898	4.185227526	21.46558712	0.005387115	2.671878716	94175.54001	4.650456906	25.40616461
65 to 69	0.016628612	2.65923674	81044.08541	3.900424816	17.65516397	0.008456184	2.729285356	91670.52624	4.4971852	21.02723677
70 to 74	0.028849576	2.642161068	74560.01452	3.490645243	13.95912708	0.01577877	2.715001959	87868.17629	4.240550442	16.81882374
75 to 79	0.045970464	2.611849037	64493.56827	2.905833085	10.7254929	0.028088099	2.696710456	81178.6971	3.812375659	12.98073875
80 to 84	0.080871803	2.532981512	51142.77432	2.132066321	7.843937094	0.053766005	2.622803704	70474.61589	3.124583756	9.542536979
85 to 89	0.140786194	2.404134372	33912.55847	1.242056007	5.543616216	0.103626534	2.527879067	53684.57634	2.137131117	6.707019771
90 to 94	0.236095294	2.174027031	16438.71513	0.4931318	3.883348859	0.187647964	2.296252762	31553.60367	1.046909089	4.639674263
95 plus	0.33023048	3.028312035	4802.804735	0.145500756	3.028312035	0.285591082	3.501844224	11921.63022	0.417688311	3.501844224

Table 15: Taiwan (Province of China) 2100 life table, by age and sex. mx=mortality rate, ax=mean person-years lived in an age interval among those who die in that age interval, lx=number of persons left alive at age x, nLx=person-years lived between age x and x+n, ex=life expectancy at age x.										
Age Group	Male					Female				
	mx	ax	lx	nLx	ex	mx	ax	lx	nLx	ex
Early Neonatal	0.05139762	0.009587466	100000	0.01916864	80.60726677	0.041802822	0.00958776	100000	0.019170402	85.6146337
Late Neonatal	0.002645264	0.028766394	99901.57664	0.057473247	80.66745741	0.00278469	0.028766355	99919.94526	0.057483584	85.66406556
Post Neonatal	0.000365787	0.461617851	99886.37882	0.922082948	80.62221833	0.000315361	0.461621433	99903.94382	0.922266557	85.62024599
1 to 4	5.68548E-05	1.999924192	99852.6604	3.993652351	79.7259888	4.61489E-05	1.999938467	99874.86777	3.994626064	84.72173989
5 to 9	3.69382E-05	2.499923046	99829.95842	4.991037018	75.74363768	4.5865E-05	2.499904447	99856.43633	4.992249482	80.7369951
10 to 14	6.88143E-05	3.440668704	99811.52307	4.990024302	70.75713177	5.39943E-05	2.93698792	99833.54444	4.991090903	75.75491188
15 to 19	0.000332257	2.785825766	99777.18688	4.985174919	65.78025303	0.000129904	2.673772173	99806.59946	4.988817944	70.77458266
20 to 24	0.000508596	2.586248047	99611.60591	4.97446094	60.88468902	0.000158012	2.604376444	99741.80113	4.985193998	65.81880681
25 to 29	0.000528054	2.632757212	99358.82213	4.961717967	56.03256322	0.000200222	2.688796659	99663.05032	4.980830047	60.86876696
30 to 34	0.000822215	2.671961362	99097.12619	4.945364422	51.17301444	0.000322479	2.658736985	99563.35024	4.974393854	55.9270068
35 to 39	0.001176428	2.679065645	98691.09789	4.921116628	46.37160032	0.000428599	2.64902082	99402.98356	4.965134389	51.01283863
40 to 44	0.001827927	2.68427593	98113.1683	4.884921005	41.62756758	0.000618479	2.70159549	99190.23908	4.952458412	46.11623051
45 to 49	0.002725895	2.669710711	97222.35282	4.830463613	36.98217635	0.001017031	2.705599671	98884.03721	4.932694031	41.25020719
50 to 54	0.004101984	2.654557853	95908.59441	4.749777665	32.4493372	0.001633875	2.66696402	98382.57037	4.900475654	36.44579253
55 to 59	0.005860686	2.620389203	93966.61158	4.633827257	28.06102478	0.002371396	2.656456659	97582.52456	4.852094666	31.72106645
60 to 64	0.00771419	2.636977317	91262.04583	4.481743469	23.81137207	0.003368499	2.688836358	96433.88293	4.784443353	27.0649527
65 to 69	0.011476123	2.71289754	87826.86843	4.279403134	19.63492496	0.005505744	2.778051473	94825.28711	4.683907927	22.47568592
70 to 74	0.020917522	2.668886634	82952.40859	3.955999328	15.62768016	0.011011244	2.786896147	92253.87635	4.502918704	18.02144428
75 to 79	0.032780244	2.685343204	74755.05132	3.476366821	12.04630693	0.021991331	2.775500307	87319.43714	4.162873856	13.87627476
80 to 84	0.066569755	2.592036799	63490.43295	2.742568359	8.708060376	0.047710486	2.676545311	78226.81028	3.52554527	10.15971333
85 to 89	0.122022515	2.460244922	45582.81276	1.75265456	6.089696844	0.094152692	2.565528163	61667.88913	2.520399904	7.141174606
90 to 94	0.216120529	2.205471723	24853.82022	0.78398447	4.180370776	0.175542713	2.31160572	38580.24269	1.323638403	4.908159589
95 plus	0.313793188	3.198208191	8401.474672	0.274283704	3.198208191	0.274043294	3.671283756	16028.22476	0.601926293	3.671283756

Table 15: Oceania 2017 life table, by age and sex. mx=mortality rate, ax=mean person-years lived in an age interval among those who die in that age interval, lx=number of persons left alive at age x, nLx=person-years lived between age x and x+n, ex=life expectancy at age x.

Age Group	Male					Female				
	mx	ax	lx	nLx	ex	mx	ax	lx	nLx	ex
Early Neonatal	0.695516832	0.009567724	100000	0.019050753	58.70016825	0.535625156	0.009572624	100000	0.019079922	63.78717841
Late Neonatal	0.101983685	0.028738991	98675.13129	0.056605804	59.46860214	0.085818658	0.02874345	98978.10953	0.056805979	64.42606415
Post Neonatal	0.016916958	0.460442088	98097.99801	0.898692875	59.76069765	0.011959088	0.460794284	98490.70204	0.904352007	64.6870942
1 to 4	0.002978728	1.996028372	96578.3667	3.840225616	59.77063022	0.003028363	1.995962193	97409.52777	3.872887751	64.47656777
5 to 9	0.00114287	2.497619022	95435.21887	4.758157043	56.46308033	0.000822886	2.498285655	96237.26231	4.801979968	61.23780412
10 to 14	0.000957457	2.740229961	94891.58348	4.734338739	51.77207473	0.000699885	2.714192977	95842.20821	4.78445929	56.47982876
15 to 19	0.002270253	2.760712357	94438.4009	4.698053887	47.00715713	0.001555629	2.713152553	95507.40682	4.758450367	51.66807608
20 to 24	0.003879969	2.628203354	93371.9831	4.626053941	42.51147858	0.002323644	2.64945405	94767.27377	4.712651639	47.04980312
25 to 29	0.004814752	2.589394481	91577.54249	4.526374149	38.29035701	0.003307008	2.631429669	93672.46359	4.64726163	42.56742679
30 to 34	0.006202718	2.600285532	89399.54009	4.404473104	34.15720838	0.004555782	2.623839937	92136.392	4.557525438	38.23082026
35 to 39	0.008211056	2.618557947	86670.49895	4.250521527	30.14788889	0.006241082	2.610185288	90062.26672	4.43700777	34.04819879
40 to 44	0.011647236	2.611768025	83185.87523	4.046946981	26.29837947	0.008232126	2.601906683	87297.88945	4.280514444	30.04148067
45 to 49	0.015899438	2.602451535	78482.99145	3.780412829	22.71549323	0.010935282	2.600996426	83781.90852	4.082205479	26.19187456
50 to 54	0.022228863	2.582560221	72490.21703	3.440197357	19.37752671	0.014796446	2.593963113	79328.17383	3.83034786	22.51625346
55 to 59	0.029836975	2.567773416	64867.67696	3.024633276	16.35239058	0.019926609	2.593929219	73674.55132	3.515565581	19.04587027
60 to 64	0.041707339	2.548003793	55874.89906	2.535408861	13.57440988	0.028220612	2.590099522	66686.5455	3.122443425	15.77154597
65 to 69	0.057816308	2.5088093	45339.2987	1.98252984	11.14260757	0.041067709	2.571772514	57894.60566	2.632799435	12.77558297
70 to 74	0.078154577	2.461676175	33920.64073	1.416101499	9.057885489	0.061288445	2.530249799	47107.56994	2.046437968	10.11547582
75 to 79	0.104849766	2.410477592	22889.99052	0.900636113	7.246658132	0.089633199	2.471107474	34598.00837	1.411043532	7.863465781
80 to 84	0.144433192	2.341340726	13470.18825	0.487078986	5.637385745	0.132082189	2.39259309	21987.68748	0.818332149	5.964673734
85 to 89	0.209950189	2.233448162	6456.348814	0.20442324	4.231368061	0.199048288	2.269863471	11205.5805	0.363306769	4.411421899
90 to 94	0.30116028	2.03854263	2175.35632	0.057552798	3.178585591	0.28795793	2.073780261	3988.982084	0.108340837	3.297659462
95 plus	0.381152215	2.623764362	445.3317228	0.011699027	2.623764362	0.374492837	2.670468547	874.423597	0.023379165	2.670468547

Table 15: Oceania 2100 life table, by age and sex. mx=mortality rate, ax=mean person-years lived in an age interval among those who die in that age interval, lx=number of persons left alive at age x, nLx=person-years lived between age x and x+n, ex=life expectancy at age x.

Age Group	Male					Female				
	mx	ax	lx	nLx	ex	mx	ax	lx	nLx	ex
Early Neonatal	0.130132881	0.009585053	100000	0.019154172	72.71115816	0.093348143	0.00958618	100000	0.019160926	77.55242125
Late Neonatal	0.02252375	0.02876091	99750.7651	0.057353687	72.87340864	0.021399819	0.02876122	99821.14456	0.057396008	77.67195031
Post Neonatal	0.002822279	0.461443346	99621.60985	0.918596849	72.91017642	0.002034745	0.461499291	99698.3389	0.919638512	77.7098959
1 to 4	0.000401737	1.99946435	99362.3969	3.971305419	72.1756286	0.000460784	1.999385622	99511.25492	3.976785914	76.93144857
5 to 9	0.00028883	2.49939827	99202.92591	4.956568492	68.28793984	0.000243845	2.49949199	99328.11032	4.963381106	73.06907579
10 to 14	0.000331917	3.001224211	99059.85588	4.949650937	63.38252523	0.000264424	2.797313452	99207.16454	4.957442096	68.15482373
15 to 19	0.001030734	2.773903297	98895.75957	4.933462551	58.48228208	0.000591948	2.694402004	99076.20848	4.947050625	63.24098913
20 to 24	0.001686378	2.628906499	98387.62501	4.899799138	53.76933554	0.00080859	2.636039073	98783.53281	4.929754709	58.42020616
25 to 29	0.002093732	2.575077451	97562.11935	4.853486186	49.2012754	0.00112295	2.630631904	98385.17227	4.90620776	53.64576961
30 to 34	0.002474749	2.578826877	96547.33108	4.798632413	44.69060094	0.001519182	2.628097101	97834.69734	4.874153256	48.93207802
35 to 39	0.003065306	2.605097685	95361.8615	4.733390319	40.21350579	0.002045764	2.626725428	97095.04747	4.831276543	44.28381213
40 to 44	0.004104768	2.625297527	93913.82614	4.650418307	35.79278893	0.002761418	2.631663929	96107.94718	4.774166347	39.71034397
45 to 49	0.005679242	2.63970986	92009.25506	4.539691162	31.47834416	0.003824556	2.636809157	94791.49245	4.69714745	35.22367828
50 to 54	0.008219802	2.64057309	89437.40676	4.386916242	27.30595376	0.005402336	2.631992206	92997.82119	4.591220234	30.84996804
55 to 59	0.011897042	2.617473981	85841.01973	4.173994547	23.33741657	0.007525157	2.603025897	90522.35393	4.446053487	26.6187777
60 to 64	0.016317435	2.639445074	80890.49302	3.894888139	19.60335361	0.009630749	2.622821884	87185.07174	4.261951191	22.53506659
65 to 69	0.026193696	2.612164206	74554.87617	3.509071	16.04177727	0.014334234	2.658969647	83093.03877	4.020239064	18.51217332
70 to 74	0.037591711	2.575440016	65402.47277	2.998428699	12.91733092	0.023057251	2.678287956	77356.51087	3.672379117	14.68379305
75 to 79	0.055543406	2.559333551	54200.96331	2.388841025	10.05398278	0.041351369	2.660337729	68944.32278	3.145750974	11.14367762
80 to 84	0.090074483	2.477762938	41044.0172	1.676303081	7.461603678	0.078030898	2.554957163	56075.82261	2.360348697	8.089860707
85 to 89	0.151248906	2.376488083	26138.58183	0.940045638	5.318175217	0.136104613	2.426749798	37944.91136	1.412771856	5.74399914
90 to 94	0.24624144	2.153434005	12140.33827	0.359103411	3.762784696	0.223605108	2.220984699	19100.72321	0.59394341	4.072831817
95 plus	0.338294411	2.959315307	3416.096583	0.102056746	2.959315307	0.318353557	3.148902785	6076.023877	0.194201967	3.148902785

Table 15: American Samoa 2017 life table, by age and sex. mx=mortality rate, ax=mean person-years lived in an age interval among those who die in that age interval, lx=number of persons left alive at age x, nLx=person-years lived between age x and x+n, ex=life expectancy at age x.

Age Group	Male					Female				
	mx	ax	lx	nLx	ex	mx	ax	lx	nLx	ex
Early Neonatal	0.196009189	0.009583033	100000	0.019142082	70.13628257	0.127784602	0.009585125	100000	0.019154602	73.9251394
Late Neonatal	0.025531372	0.02876008	99624.81089	0.05727631	70.38116622	0.02719275	0.028759622	99755.2395	0.057348555	74.08728593
Post Neonatal	0.004779259	0.461304325	99478.59025	0.916450334	70.42703711	0.002632889	0.4614568	99599.30312	0.91847132	74.14568784
1 to 4	0.000510442	1.999319411	99040.65293	3.957586113	69.81314046	0.00063653	1.999151293	99357.49348	3.969245766	73.40169825
5 to 9	0.000253658	2.499471546	98838.72775	4.938804029	65.95173522	0.000251991	2.499475018	99104.91096	4.952125374	69.58366698
10 to 14	0.000289142	2.975258093	98713.46022	4.932783802	61.03216828	0.000250229	2.796308117	98980.13028	4.946278696	64.66822128
15 to 19	0.000914632	2.772771068	98570.84699	4.918524276	56.11614238	0.000609004	2.709517547	98856.36694	4.935933891	59.74568055
20 to 24	0.001496831	2.601220878	98121.04121	4.888500134	51.36088878	0.000866811	2.705839988	98555.77864	4.918011243	54.91958814
25 to 29	0.001658394	2.562716854	97389.4338	4.849864839	46.72764845	0.001478096	2.673159297	98129.49467	4.889658319	50.14630036
30 to 34	0.002019208	2.610340274	96585.2637	4.806070777	42.09582132	0.002119187	2.620703648	97406.78946	4.845905004	45.49828777
35 to 39	0.00277085	2.657956379	95614.90376	4.749927134	37.49674685	0.002750292	2.612543888	96379.92191	4.78756368	40.95485727
40 to 44	0.004221744	2.689994634	94298.77	4.669410015	32.98262363	0.003689731	2.615155961	95063.3511	4.711714629	36.48559866
45 to 49	0.006862589	2.68259241	92327.4711	4.544118003	28.628267	0.004938442	2.624870017	93325.19337	4.612174605	32.11609082
50 to 54	0.010820322	2.655337123	89209.42354	4.350145048	24.53309755	0.006928364	2.636542746	91048.13425	4.479083277	27.85331972
55 to 59	0.016296146	2.625275634	84504.21912	4.067909109	20.74839878	0.010030231	2.629029635	87946.0976	4.29519508	23.74246838
60 to 64	0.023609538	2.601557213	77880.26039	3.685629737	17.28624989	0.014254088	2.640289787	83639.84757	4.045962618	19.82933055
65 to 69	0.034043164	2.583253278	69192.43793	3.197266441	14.12579066	0.022126729	2.64050328	77875.51438	3.700667882	16.1016134
70 to 74	0.050260643	2.552706499	58338.58514	2.598638874	11.27008192	0.035084268	2.628585197	69691.55092	3.217074625	12.68248343
75 to 79	0.07419258	2.507040194	45335.14768	1.914838932	8.771129572	0.05934298	2.580381325	58411.41816	2.55413596	9.624417841
80 to 84	0.111743692	2.444450278	31219.4293	1.216793916	6.611934505	0.098777451	2.496985449	43267.26386	1.7348312	7.091135864
85 to 89	0.176181057	2.311919673	17748.22838	0.604190903	4.799833866	0.162026908	2.354599375	26146.05446	0.91533591	5.101806653
90 to 94	0.270206571	2.10452018	7201.908468	0.202797178	3.485147015	0.250995591	2.159179301	11327.8576	0.330754989	3.69918174
95 plus	0.357255901	2.80029398	1762.758477	0.049614587	2.80029398	0.342769364	2.917567171	3032.123678	0.088512179	2.917567171

Table 15: American Samoa 2100 life table, by age and sex. mx=mortality rate, ax=mean person-years lived in an age interval among those who die in that age interval, lx=number of persons left alive at age x, nLx=person-years lived between age x and x+n, ex=life expectancy at age x.

Age Group	Male					Female				
	mx	ax	lx	nLx	ex	mx	ax	lx	nLx	ex
Early Neonatal	0.047756483	0.009587577	100000	0.019169303	74.32119547	0.025798127	0.00958825	100000	0.019173339	76.82717793
Late Neonatal	0.007080842	0.02876517	99908.45975	0.057469873	74.37000499	0.00898946	0.028764644	99950.53854	0.057490922	76.84596838
Post Neonatal	0.001808183	0.461515385	99867.7695	0.921297638	74.34268708	0.000830885	0.461584811	99898.86076	0.922000211	76.82809336
1 to 4	0.000168274	1.999775634	99701.20782	3.986709742	73.54226262	0.000232297	1.999690271	99822.26638	3.991039585	75.96302922
5 to 9	0.00012441	2.499740812	99634.33289	4.980180112	69.58793576	0.000156506	2.499673947	99729.77821	4.984549708	72.03000763
10 to 14	0.000181008	3.271204329	99572.93572	4.976846712	64.62705079	0.000196751	2.870480926	99652.26767	4.980439776	67.08289339
15 to 19	0.000532551	2.809886687	99484.21621	4.968342198	59.67950899	0.000403472	2.730736107	99555.14252	4.973169518	62.14472112
20 to 24	0.000847462	2.666879338	99222.22072	4.951329131	54.82758149	0.000589306	2.790394328	99355.4615	4.961286839	57.26366769
25 to 29	0.001137707	2.600147114	98806.48955	4.926939905	50.04573065	0.001179599	2.704426093	99064.21576	4.939824118	52.42353754
30 to 34	0.001352749	2.613380234	98251.03429	4.896827793	45.3133892	0.001717694	2.625599331	98482.97897	4.904151355	47.71656065
35 to 39	0.001839823	2.670321525	97594.43468	4.858999527	40.60078401	0.002203905	2.59460443	97642.4933	4.856407991	43.10415099
40 to 44	0.002839339	2.68962171	96707.2665	4.803981504	35.94917709	0.002746915	2.613678941	96574.27353	4.797311904	38.55183651
45 to 49	0.004460768	2.697961798	95351.30791	4.719273902	31.42237601	0.003771257	2.618581783	95259.15407	4.720633539	34.04766925
50 to 54	0.007269604	2.692224792	93255.91049	4.586092327	27.06752625	0.00503455	2.675374447	93482.35153	4.620125649	29.64454645
55 to 59	0.011764342	2.610369589	89935.09954	4.374154616	22.96675324	0.008327948	2.613683683	91161.20057	4.46938043	25.33071813
60 to 64	0.014709795	2.642338244	84807.50574	4.098830668	19.19619955	0.010163732	2.609603388	87446.71169	4.268896892	21.29520159
65 to 69	0.024948486	2.648120053	78803.74261	3.722728852	15.45468509	0.015018378	2.670734838	83117.90144	4.015769121	17.26751182
70 to 74	0.039316551	2.59197703	69572.40847	3.179848203	12.15089945	0.025314617	2.719749922	77108.26202	3.645800104	13.40432553
75 to 79	0.059084365	2.597654531	57172.60926	2.50699815	9.219727789	0.051911348	2.66503293	67924.23521	3.031145503	9.846206396
80 to 84	0.118490531	2.485788273	42554.35058	1.648977411	6.500276985	0.102218228	2.522585073	52320.5128	2.090992915	6.994645235
85 to 89	0.183608811	2.297941817	23518.95737	0.798632907	4.719793706	0.166206502	2.345895988	31123.62079	1.084528181	5.034335539
90 to 94	0.276535805	2.090475826	9517.287489	0.269524846	3.440678582	0.255069766	2.149706665	13341.58344	0.38889183	3.659895149
95 plus	0.36193232	2.774198824	2379.48305	0.068040149	2.774198824	0.34620505	2.893474027	3561.088999	0.104168503	2.893474027

Table 15: Federated States of Micronesia 2017 life table, by age and sex. mx=mortality rate, ax=mean person-years lived in an age interval among those who die in that age interval, lx=number of persons left alive at age x, nLx=person-years lived between age x and x+n, ex=life expectancy at age x.

Age Group	Male					Female				
	mx	ax	lx	nLx	ex	mx	ax	lx	nLx	ex
Early Neonatal	0.362024684	0.009577945	100000	0.019111663	65.02483021	0.27604906	0.00958058	100000	0.019127408	69.71341596
Late Neonatal	0.049285789	0.028753528	99308.15703	0.05705528	65.45842174	0.040754532	0.028755881	99472.01258	0.057163436	70.06406969
Post Neonatal	0.00653157	0.461179844	99027.00069	0.911553374	65.58662108	0.004404976	0.461330914	99239.0702	0.914401647	70.1708827
1 to 4	0.000952384	1.998730155	98431.75794	3.929782565	65.05715746	0.000914012	1.998781317	98836.3414	3.946236895	69.53159058
5 to 9	0.000578271	2.498795269	98057.61199	4.895800611	61.29779582	0.00038008	2.499208168	98475.72636	4.919111219	65.77887653
10 to 14	0.000510253	2.812931809	97774.55062	4.883275938	56.46784933	0.000364739	2.780604521	98288.78191	4.910465235	60.89918355
15 to 19	0.001349122	2.776009568	97525.42399	4.861695761	51.60479374	0.000875409	2.732557963	98109.69465	4.895771173	56.00516494
20 to 24	0.002331363	2.637161148	96869.60756	4.816971017	46.934752	0.001353784	2.686608576	97681.15619	4.868822683	51.23850539
25 to 29	0.002955114	2.599078822	95746.89427	4.753650624	42.45248921	0.002122309	2.652595393	97022.11585	4.827077224	46.5674417
30 to 34	0.003846854	2.611986168	94343.01185	4.674245196	38.04338993	0.002971142	2.627412407	95997.95398	4.766318588	42.03433201
35 to 39	0.005177047	2.633677942	92546.87275	4.571395909	33.72873421	0.004038025	2.621011006	94582.68392	4.68416381	37.62194791
40 to 44	0.00745506	2.636122732	90183.91119	4.431217343	29.54136959	0.005478278	2.617540563	92693.25466	4.5750016	33.33318158
45 to 49	0.010632198	2.631014918	86887.14295	4.237819518	25.5605134	0.007423073	2.622345663	90190.61853	4.43142413	29.18354686
50 to 54	0.015375295	2.615372782	82392.5065	3.974243762	21.81047611	0.010437177	2.615068076	86906.89967	4.239994772	25.18559265
55 to 59	0.021683202	2.606780509	76298.16296	3.627159733	18.3440828	0.014346871	2.608451758	82490.89962	3.98800843	21.39274473
60 to 64	0.031860105	2.58296208	68454.55437	3.178628975	15.14893626	0.020171673	2.610514718	76783.28182	3.663038986	17.7886249
65 to 69	0.04527528	2.545617823	58356.14677	2.626789874	12.32672732	0.02973579	2.606453807	69413.41264	3.240726124	14.40042876
70 to 74	0.063358868	2.515723405	46499.04363	2.009605707	9.826157396	0.045707661	2.590298684	59807.41494	2.694719485	11.29621455
75 to 79	0.094166274	2.46882848	33803.78109	1.365404578	7.578659105	0.0745828	2.538316824	47538.93715	2.009804181	8.546881011
80 to 84	0.141572018	2.378790207	20969.83535	0.765277324	5.711097633	0.120994936	2.44869944	32623.81157	1.24807756	6.303349367
85 to 89	0.207741284	2.238281032	10163.77288	0.323232655	4.264721183	0.187502724	2.294782172	17602.31149	0.584989965	4.609887938
90 to 94	0.299175138	2.04278344	3464.579291	0.092003598	3.196724318	0.276574985	2.099902971	6686.855253	0.185928849	3.413506962
95 plus	0.379637872	2.634257237	716.9543572	0.018909201	2.634257237	0.364830096	2.741699168	1565.36157	0.043042558	2.741699168

Table 15: Federated States of Micronesia 2100 life table, by age and sex. mx=mortality rate, ax=mean person-years lived in an age interval among those who die in that age interval, lx=number of persons left alive at age x, nLx=person-years lived between age x and x+n, ex=life expectancy at age x.

Age Group	Male					Female				
	mx	ax	lx	nLx	ex	mx	ax	lx	nLx	ex
Early Neonatal	0.07483114	0.009586748	100000	0.019164328	73.58765485	0.051003732	0.009587478	100000	0.019168706	77.87018673
Late Neonatal	0.011699124	0.028763896	99856.6005	0.057432413	73.67385736	0.010828094	0.028764136	99902.23844	0.057460101	77.92695943
Post Neonatal	0.001455278	0.461540455	99789.41557	0.920724773	73.66580957	0.000971595	0.461574815	99840.02504	0.921397349	77.9178683
1 to 4	0.000235159	1.999686454	99655.45088	3.984344967	72.84057366	0.000216575	1.999711233	99750.51408	3.98829375	77.06383503
5 to 9	0.000188433	2.49960743	99561.823	4.975747895	68.9064534	0.000128988	2.499731275	99664.19618	4.981604256	73.12823858
10 to 14	0.000193689	3.037671004	99468.11041	4.971477126	63.96867516	0.000150334	2.901261518	99599.98328	4.978395081	68.17349864
15 to 19	0.000663429	2.791382125	99371.8683	4.96131371	59.02744586	0.000394126	2.721911708	99525.18446	4.971788624	63.22235528
20 to 24	0.001137823	2.664184214	99042.80811	4.939012455	54.21386673	0.000576321	2.705597501	99329.29046	4.959924075	58.34140028
25 to 29	0.001585094	2.580306642	98481.04732	4.905286203	49.50738233	0.000995655	2.637610097	99043.55929	4.94060694	53.50140238
30 to 34	0.001802269	2.562088367	97704.06869	4.864181827	44.87918568	0.001287425	2.611188238	98552.2055	4.912575297	48.75304954
35 to 39	0.002444335	2.637434767	96829.78547	4.813878323	40.25725456	0.001724363	2.639237003	97921.81605	4.876480553	44.04704089
40 to 44	0.003501306	2.755603917	95661.8248	4.744119255	35.71150134	0.002595022	2.800840968	97086.30061	4.825066434	39.40002031
45 to 49	0.004686734	2.641110396	94023.13247	4.64980789	31.28688524	0.003563867	2.633895787	95848.36636	4.752350921	34.87471713
50 to 54	0.006931991	2.658312354	91851.49391	4.519378461	26.96280882	0.004981589	2.634540828	94161.17552	4.653349908	30.4515557
55 to 59	0.010616716	2.640801316	88729.88674	4.328423044	22.81663783	0.007133889	2.608170375	91850.22275	4.515634947	26.14955228
60 to 64	0.015436588	2.673130727	84155.97172	4.062731614	18.91272998	0.00924586	2.61902403	88640.04493	4.337030625	22.0001717
65 to 69	0.026921525	2.639061601	77933.48582	3.665176338	15.20888738	0.013786917	2.687855399	84647.51713	4.102106067	17.90979305
70 to 74	0.040971167	2.586012624	68162.32812	3.103256832	12.01486093	0.024094754	2.715158003	79037.5195	3.747107567	13.98769369
75 to 79	0.0640139	2.558989661	55538.19402	2.40439898	9.159844634	0.048149925	2.653726363	70098.82379	3.152756613	10.42159874
80 to 84	0.110835682	2.458218829	40286.72629	1.575773401	6.666990084	0.089818728	2.541710698	55139.31081	2.264710649	7.53829112
85 to 89	0.175183547	2.315182084	23027.33563	0.787424875	4.83019611	0.150926968	2.386222025	35114.95821	1.267695253	5.3846715
90 to 94	0.269141642	2.106551115	9456.129259	0.267767421	3.501195948	0.239198569	2.185930236	16424.30614	0.496089668	3.863484714
95 plus	0.356370048	2.809407832	2354.225767	0.066832722	2.809407832	0.332204432	3.019298922	4833.782876	0.14859335	3.019298922

Table 15: Fiji 2017 life table, by age and sex. mx=mortality rate, ax=mean person-years lived in an age interval among those who die in that age interval, lx=number of persons left alive at age x, nLx=person-years lived between age x and x+n, ex=life expectancy at age x.

Age Group	Male					Female				
	mx	ax	lx	nLx	ex	mx	ax	lx	nLx	ex
Early Neonatal	0.459422251	0.00957496	100000	0.01909385	66.023692	0.420193134	0.009576162	100000	0.019101021	70.50499691
Late Neonatal	0.061777241	0.028750082	99122.91356	0.056928424	66.58832054	0.06012496	0.028750538	99197.47225	0.056973942	71.05595679
Post Neonatal	0.008301908	0.461054082	98771.34845	0.90845925	66.76766554	0.006034998	0.46121512	98855.00387	0.910178809	71.24448056
1 to 4	0.001796789	1.997604283	98017.55425	3.906656996	66.3544198	0.001407243	1.998123677	98305.91384	3.921195373	70.71663801
5 to 9	0.000604291	2.498741061	97316.16357	4.858467808	62.81859724	0.000503534	2.498950972	97754.38497	4.881573016	67.10453569
10 to 14	0.000657607	2.634974797	97022.69753	4.84360366	58.00112768	0.000467592	2.640336074	97508.63918	4.870060977	62.26739986
15 to 19	0.001036115	2.69235218	96704.25844	4.823682795	53.18337989	0.000824033	2.613944621	97280.94492	4.854504887	57.40685599
20 to 24	0.001630094	2.592274595	96204.50739	4.791420915	48.44529348	0.000925821	2.57407017	96880.93939	4.833194064	52.63275525
25 to 29	0.001780052	2.546730178	95423.51656	4.750433248	43.81998849	0.001164299	2.603968909	96433.51124	4.808264451	47.86460682
30 to 34	0.002062829	2.636215697	94578.01356	4.705959877	39.1883706	0.001522486	2.643404793	95873.7624	4.77655637	43.12833094
35 to 39	0.003188545	2.680240782	93607.42528	4.646017359	34.56669492	0.00224357	2.674725943	95146.68152	4.732649944	38.43703269
40 to 44	0.004947376	2.719138969	92126.44717	4.554944171	30.07823046	0.00346517	2.691278253	94085.20199	4.666932956	33.83946266
45 to 49	0.008743678	2.700498427	89873.99617	4.405184141	25.76241306	0.005571531	2.70601953	92468.75014	4.565116954	29.38261504
50 to 54	0.014317459	2.65238375	86025.1362	4.161521069	21.79203524	0.009382926	2.672806148	89926.87734	4.400333024	25.13488077
55 to 59	0.021539854	2.624167637	80073.88606	3.809061445	18.21198496	0.014371322	2.663314321	85801.81734	4.150863903	21.21242623
60 to 64	0.03253193	2.579835906	71883.18035	3.332364004	14.98600988	0.023271519	2.609659553	79844.42936	3.782212166	17.59376384
65 to 69	0.044911953	2.554288974	61067.23336	2.752012756	12.18241872	0.032144372	2.588127797	71059.65608	3.298010862	14.44355659
70 to 74	0.067369564	2.516441247	48746.77019	2.089286275	9.617266984	0.049257183	2.549160075	60489.45388	2.69992713	11.51394167
75 to 79	0.09680176	2.453467922	34732.74279	1.39484961	7.488302417	0.068037926	2.516642364	47248.7861	2.022793079	9.028377927
80 to 84	0.14316708	2.380923732	21309.0233	0.776437024	5.674253175	0.109651908	2.472437844	33572.44125	1.316610362	6.690139577
85 to 89	0.209418878	2.234782369	10268.37623	0.325960822	4.241393982	0.174632989	2.324327726	19245.82383	0.657599307	4.850750217
90 to 94	0.300663769	2.039622404	3483.919937	0.092408298	3.183925572	0.263720156	2.129639185	7849.497106	0.22413435	3.553536282
95 plus	0.380763827	2.62681987	718.6615392	0.018938206	2.62681987	0.353790914	2.827869357	1977.854599	0.056209966	2.827869357

Table 15: Fiji 2100 life table, by age and sex. mx=mortality rate, ax=mean person-years lived in an age interval among those who die in that age interval, lx=number of persons left alive at age x, nLx=person-years lived between age x and x+n, ex=life expectancy at age x.

Age Group	Male					Female				
	mx	ax	lx	nLx	ex	mx	ax	lx	nLx	ex
Early Neonatal	0.106395363	0.00958578	100000	0.019158532	74.32869581	0.102502699	0.009585899	100000	0.019159247	78.59171456
Late Neonatal	0.01812441	0.028762124	99796.19964	0.057387077	74.46080389	0.02354869	0.028760627	99803.65324	0.057382421	78.7265489
Post Neonatal	0.00238804	0.461474193	99692.23652	0.919432588	74.48064301	0.001753373	0.461519279	99668.614	0.919483993	78.77537199
1 to 4	0.000469649	1.999373801	99472.78542	3.975179105	73.72039664	0.000348331	1.999535559	99507.50239	3.977530503	77.97852368
5 to 9	0.000154443	2.499678244	99286.24092	4.962397074	69.85503331	0.000158606	2.499669571	99369.06106	4.966485572	74.08413819
10 to 14	0.000285635	2.821995551	99209.65693	4.957338144	64.90686954	0.000187576	2.830635842	99290.3755	4.962436121	69.14058702
15 to 19	0.000543723	2.701231026	99068.15539	4.947193574	59.99540498	0.000392975	2.594467112	99197.41198	4.955185571	64.20248618
20 to 24	0.000788983	2.61235913	98799.37994	4.930645841	55.15082902	0.000362463	2.56826102	99002.83817	4.945786751	59.32322743
25 to 29	0.00094072	2.546359602	98410.62653	4.909215656	50.35799974	0.000512299	2.684603985	98823.80209	4.935293814	54.42551521
30 to 34	0.001017267	2.600823759	97949.15757	4.885490485	45.58264442	0.000771594	2.660498375	98571.27118	4.91965034	49.55737282
35 to 39	0.001398022	2.656527718	97452.88553	4.856729487	40.80066303	0.001072746	2.726923762	98192.08371	4.897506865	44.73757341
40 to 44	0.002091437	2.681899122	96774.82785	4.815408807	36.06660042	0.001782665	2.68432053	97667.64834	4.863262205	39.96155222
45 to 49	0.003286518	2.788125413	95769.60343	4.753618455	31.41442756	0.002618462	2.723551246	96801.95031	4.81105497	35.29302755
50 to 54	0.006466447	2.751581064	94212.81261	4.642830282	26.8826881	0.0042076	2.726556567	95546.84012	4.731992604	30.71719313
55 to 59	0.01119061	2.667210005	91223.23955	4.445159111	22.66717543	0.00725997	2.657223974	93560.24147	4.599753082	26.30835065
60 to 64	0.01629235	2.649852241	86277.60867	4.155510474	18.80573848	0.009901809	2.628692721	90233.66029	4.408465849	22.17469903
65 to 69	0.026363551	2.651259485	79550.12358	3.74744474	15.16016254	0.014247591	2.684324637	85892.40336	4.15796387	18.1543752
70 to 74	0.043310195	2.592588878	69803.07165	3.166172836	11.89593954	0.02484104	2.698536689	80014.97862	3.786509576	14.28321107
75 to 79	0.066690002	2.564231973	56377.45178	2.437678239	9.094920241	0.046178286	2.666803138	70739.64794	3.199498259	10.78839888
80 to 84	0.123054134	2.452349087	40781.09338	1.581811127	6.597402186	0.086406482	2.564307985	56393.14528	2.345373485	7.855759082
85 to 89	0.186289558	2.300371978	22814.38224	0.793325911	4.789554377	0.1456329	2.406993249	36952.16876	1.366262135	5.597597152
90 to 94	0.277893402	2.086475946	9843.171139	0.287843802	3.474869664	0.232815018	2.198431897	18357.27054	0.572352521	3.989259543
95 plus	0.362477474	2.7923811	2714.195669	0.081724458	2.7923811	0.326110568	3.097920014	5922.285941	0.193324805	3.097920014

Table 15: Guam 2017 life table, by age and sex. mx=mortality rate, ax=mean person-years lived in an age interval among those who die in that age interval, lx=number of persons left alive at age x, nLx=person-years lived between age x and x+n, ex=life expectancy at age x.

Age Group	Male					Female				
	mx	ax	lx	nLx	ex	mx	ax	lx	nLx	ex
Early Neonatal	0.297347462	0.009579927	100000	0.019123505	70.38122926	0.247698773	0.009581449	100000	0.019132603	76.26959979
Late Neonatal	0.029821884	0.028758897	99431.38745	0.057158054	70.76441918	0.036990644	0.028756919	99526.10136	0.057200707	76.61348551
Post Neonatal	0.005951843	0.461221027	99260.9452	0.91395086	70.82833843	0.00363269	0.461385775	99314.52754	0.91542281	76.71909346
1 to 4	0.000462389	1.999383482	98717.04507	3.945033185	70.2927624	0.000491637	1.999344484	98981.99947	3.955389901	76.0519572
5 to 9	0.000221754	2.499538012	98534.67153	4.924003573	66.41918428	0.000260525	2.49945724	98787.56008	4.936162558	72.19770715
10 to 14	0.000240082	3.486674648	98425.49167	4.919486423	61.49009418	0.000271496	2.64243204	98658.97027	4.929793046	67.28855287
15 to 19	0.001363465	2.683503234	98307.39157	4.899893985	56.55976651	0.000447094	2.630869005	98525.13316	4.921044793	62.37635583
20 to 24	0.001452281	2.668424061	97639.32749	4.865491434	51.92823026	0.000554646	2.707742845	98305.12238	4.909015395	57.51002571
25 to 29	0.002572314	2.632276011	96932.74327	4.817298436	47.28713235	0.001005625	2.593585952	98032.851	4.889811157	52.66214702
30 to 34	0.003144993	2.564615648	95693.63999	4.748316627	42.86507798	0.001015049	2.647462444	97541.13119	4.865439553	47.91440587
35 to 39	0.003646463	2.622863278	94200.40544	4.669549349	38.50359154	0.001741749	2.649792734	97047.29081	4.832582022	43.14456522
40 to 44	0.005471592	2.624939445	92497.8666	4.565572293	34.16364887	0.002294825	2.697918364	96205.64959	4.785002431	38.49856667
45 to 49	0.007234439	2.652994085	90000.20875	4.424896321	30.0383953	0.003995606	2.683318236	95107.72357	4.711774204	33.91157093
50 to 54	0.011477286	2.61904103	86799.87337	4.224586515	26.04754275	0.005988878	2.641331484	93225.39345	4.596352308	29.54175503
55 to 59	0.015100398	2.593288919	81952.96681	3.954014546	22.43252388	0.008450753	2.656847567	90473.25395	4.435844892	25.35965452
60 to 64	0.020795673	2.568323069	75985.18644	3.616487669	18.9901864	0.013256224	2.631789291	86725.58459	4.204330687	21.34028229
65 to 69	0.026187628	2.606717716	68469.13682	3.221696993	15.79260679	0.018729456	2.618112726	81153.95954	3.884491387	17.62421265
70 to 74	0.043649471	2.561205125	60039.15457	2.713415262	12.64409681	0.028003656	2.635627887	73882.08974	3.464873566	14.10060012
75 to 79	0.056364719	2.517207973	48208.04507	2.114875009	10.11950132	0.047147371	2.604649577	64187.18997	2.884056447	10.83177241
80 to 84	0.08583749	2.501993791	36305.13291	1.495203912	7.614179224	0.077990852	2.518573549	50607.36483	2.120741836	8.039724931
85 to 89	0.14662329	2.388087463	23492.1548	0.849738486	5.406426821	0.136138565	2.424675669	34100.02825	1.263171101	5.715287511
90 to 94	0.241894223	2.162767804	11052.2963	0.327885052	3.809847337	0.223925384	2.221098796	16941.21843	0.522600908	4.055273177
95 plus	0.334887349	2.986279847	3130.269321	0.093551774	2.986279847	0.318770222	3.137717857	5262.274063	0.165365475	3.137717857

Table 15: Guam 2100 life table, by age and sex. mx=mortality rate, ax=mean person-years lived in an age interval among those who die in that age interval, lx=number of persons left alive at age x, nLx=person-years lived between age x and x+n, ex=life expectancy at age x.

Age Group	Male					Female				
	mx	ax	lx	nLx	ex	mx	ax	lx	nLx	ex
Early Neonatal	0.11220479	0.009585602	100000	0.019157466	76.39442495	0.0874158	0.009586362	100000	0.019162017	80.93730689
Late Neonatal	0.011653255	0.028763909	99785.10255	0.057391375	76.53952967	0.020897568	0.028761359	99832.52285	0.057403389	81.05352492
Post Neonatal	0.002274986	0.461482224	99718.25893	0.919720589	76.53329605	0.001316628	0.461550305	99712.62287	0.920075237	81.09324648
1 to 4	0.000127908	1.999829456	99509.13559	3.979347736	75.76987188	0.000139722	1.999813704	99591.53845	3.982549013	80.26786782
5 to 9	5.66107E-05	2.499882061	99458.2559	4.972209147	71.80760419	0.00011353	2.499763478	99535.91803	4.975384099	76.3115644
10 to 14	7.48316E-05	3.513419508	99430.11158	4.971002792	66.82716661	0.000124699	2.73761959	99479.45169	4.972563068	71.3534131
15 to 19	0.000778013	2.600088671	99392.92323	4.960376243	61.85082442	0.000249131	2.570138912	99417.4666	4.967875949	66.39614386
20 to 24	0.000776985	2.678831571	99007.02715	4.941373851	57.08100049	0.000217598	3.01609623	99293.7471	4.962452337	61.47551455
25 to 29	0.001896652	2.613648698	98623.27803	4.90885532	52.29111626	0.0006982	2.656407116	99185.87109	4.951136115	56.53889218
30 to 34	0.00245838	2.536030711	97693.17655	4.855244134	47.76122381	0.000684161	2.647315641	98840.44791	4.934043565	51.72655641
35 to 39	0.002633023	2.572765103	96501.59634	4.794399225	43.31676651	0.001149016	2.633768329	98503.06469	4.911799696	46.89427394
40 to 44	0.003895083	2.556985986	95242.10386	4.717221828	38.85287637	0.001430452	2.752552387	97938.88158	4.881251468	42.14876829
45 to 49	0.004384048	2.601113361	93408.28011	4.621846422	34.56258162	0.002914628	2.660866395	97240.99927	4.829091246	37.430662
50 to 54	0.00782344	2.573285513	91385.10028	4.484258081	30.26809264	0.003694659	2.62614076	95834.68176	4.750075204	32.93896377
55 to 59	0.009807324	2.528734911	87886.82193	4.290654897	26.36540537	0.005283923	2.624546338	94081.17366	4.645832937	28.50200714
60 to 64	0.011638988	2.535633696	83691.88559	4.068614122	22.55546618	0.0071938	2.642922113	91629.02319	4.505242634	24.19186343
65 to 69	0.015500584	2.608892402	78988.79448	3.80896904	18.73870677	0.010816601	2.648961477	88398.0674	4.310627664	19.97307596
70 to 74	0.029909354	2.529164369	73129.05944	3.407097149	15.02421351	0.016223623	2.770738284	83754.53951	4.042073827	15.92774849
75 to 79	0.035277897	2.529383548	63044.19199	2.903691973	12.01502941	0.036072832	2.689007962	77253.58787	3.56770781	12.02667595
80 to 84	0.065041476	2.501985908	52996.33643	2.28920898	8.814245396	0.065079542	2.554215125	64512.15175	2.789803185	8.866426801
85 to 89	0.119314572	2.468543537	38576.2666	1.49565271	6.176189535	0.118607605	2.481293963	46700.32687	1.809482552	6.265802305
90 to 94	0.213134691	2.209337598	21453.06041	0.682433383	4.228106512	0.204200015	2.261208689	25830.71918	0.837442831	4.380547083
95 plus	0.311313747	3.225542389	7430.067499	0.245698483	3.225542389	0.300668628	3.340575912	9206.563146	0.314231047	3.340575912

Table 15: Kiribati 2017 life table, by age and sex. mx=mortality rate, ax=mean person-years lived in an age interval among those who die in that age interval, lx=number of persons left alive at age x, nLx=person-years lived between age x and x+n, ex=life expectancy at age x.

Age Group	Male					Female				
	mx	ax	lx	nLx	ex	mx	ax	lx	nLx	ex
Early Neonatal	0.687634569	0.009567965	100000	0.019052191	58.89024285	0.554351976	0.00957205	100000	0.019076504	66.57987367
Late Neonatal	0.077144201	0.028745843	98690.07145	0.056654802	59.65211173	0.069422875	0.028747973	98942.58768	0.056812361	67.2717933
Post Neonatal	0.015440019	0.460547006	98253.14116	0.900726434	59.85964766	0.012843127	0.460731483	98548.26597	0.904512302	67.48325747
1 to 4	0.00257266	1.996569793	96863.07003	3.854668131	59.78884683	0.002932314	1.996090257	97386.99937	3.872734994	67.3591983
5 to 9	0.001060376	2.497790884	95872.06545	4.780922795	56.38651741	0.000707707	2.49852561	96252.00449	4.804097133	64.13029667
10 to 14	0.000888803	2.75370391	95365.29699	4.75876581	51.67281769	0.00054365	2.654078353	95912.08229	4.789504542	59.34853098
15 to 19	0.002162048	2.771124847	94942.46113	4.72437407	46.89052434	0.001124433	2.687334898	95651.72827	4.770189416	54.50256907
20 to 24	0.003778914	2.63127904	93921.20144	4.654422482	42.36931416	0.001575522	2.606319313	95115.35285	4.737903142	49.7937012
25 to 29	0.004691465	2.590900578	92162.7629	4.556666797	38.12490284	0.001953499	2.602528485	94369.05271	4.69646825	45.16549717
30 to 34	0.006069048	2.601003744	90026.28422	4.436766202	33.96531166	0.002581649	2.635550801	93452.03606	4.644266211	40.58158152
35 to 39	0.008022543	2.62001669	87336.34879	4.285103047	29.92837072	0.003704205	2.651814186	92254.04125	4.572943457	36.07252365
40 to 44	0.011432006	2.614758182	83903.68929	4.084034053	26.04288652	0.005411974	2.650306583	90562.17121	4.4713059	31.69494863
45 to 49	0.01572492	2.606455387	79244.74816	3.818865654	22.4177759	0.007924242	2.64397688	88145.86647	4.326623944	27.48932059
50 to 54	0.022197549	2.588911783	73256.99259	3.477305638	19.03582793	0.011571701	2.631403769	84723.61676	4.123357606	23.49115136
55 to 59	0.030404573	2.570736351	65563.42671	3.053447977	15.96650942	0.016708471	2.625696191	79961.61789	3.845836317	19.73216101
60 to 64	0.042595748	2.547151669	56313.99676	2.55030617	13.16975153	0.024886539	2.61469224	73550.53332	3.471943664	16.22223874
65 to 69	0.059211744	2.51262203	45494.48351	1.983856426	10.70182358	0.037529463	2.592878164	64932.91699	2.978412828	13.02778005
70 to 74	0.082893242	2.467256608	33799.85507	1.397824919	8.544700536	0.057866756	2.552911293	53790.27723	2.357248346	10.19007616
75 to 79	0.116264953	2.403969062	22261.32037	0.855716061	6.706862955	0.089307482	2.491194037	40212.83414	1.644763933	7.772518769
80 to 84	0.16416734	2.312938497	12344.83638	0.428979177	5.174653072	0.138663797	2.39817657	25628.32454	0.944114771	5.791237935
85 to 89	0.229582566	2.192337786	5336.473312	0.162565685	3.955868902	0.206318954	2.255463405	12658.09783	0.405613878	4.300029054
90 to 94	0.318612149	2.001536553	1620.902083	0.041533992	3.027510783	0.295024421	2.05789033	4368.086206	0.117472143	3.232056971
95 plus	0.394365269	2.535998495	302.2578994	0.007684339	2.535998495	0.380398595	2.630131036	931.8834659	0.024672034	2.630131036

Table 15: Kiribati 2100 life table, by age and sex. mx=mortality rate, ax=mean person-years lived in an age interval among those who die in that age interval, lx=number of persons left alive at age x, nLx=person-years lived between age x and x+n, ex=life expectancy at age x.

Age Group	Male					Female				
	mx	ax	lx	nLx	ex	mx	ax	lx	nLx	ex
Early Neonatal	0.133784393	0.009584941	100000	0.019153502	71.36930943	0.099932843	0.009585978	100000	0.019159717	77.73415272
Late Neonatal	0.015558717	0.028762831	99743.7789	0.057361158	71.53277295	0.014615686	0.028763092	99808.54229	0.057399958	77.86350323
Post Neonatal	0.00297402	0.461432566	99654.54542	0.918836384	71.53907499	0.002228976	0.461485493	99724.65879	0.919798865	77.87121207
1 to 4	0.000362794	1.999516275	99381.36442	3.972372995	70.81039526	0.000404541	1.999460612	99519.68236	3.977569618	77.10666302
5 to 9	0.000229192	2.499522517	99237.32371	4.959025504	66.90983051	0.000154215	2.499678719	99358.84752	4.966028149	73.22761345
10 to 14	0.000265303	3.130769943	99123.71938	4.953716084	61.98339863	0.000154804	2.835726982	99282.28874	4.962453576	68.28199601
15 to 19	0.001022006	2.81777466	98992.34491	4.938599473	57.06131069	0.000405666	2.690882855	99205.48937	4.955630937	63.33251117
20 to 24	0.001831387	2.633243259	98487.79186	4.903130905	52.33851441	0.000524487	2.598807014	99004.55164	4.943998712	58.45511228
25 to 29	0.00220709	2.564550976	97590.31182	4.853452529	47.79456584	0.000651614	2.598680884	98745.39444	4.929550624	53.60112848
30 to 34	0.002566491	2.575520111	96520.16826	4.796167224	43.29467855	0.000826463	2.640151497	98424.42769	4.91162754	48.7667832
35 to 39	0.003165506	2.606515613	95291.23536	4.728801639	38.81847067	0.001193873	2.684298185	98018.83651	4.88741742	43.95682753
40 to 44	0.004291939	2.615789619	93797.66777	4.642410168	34.39277137	0.001878047	2.688543833	97435.82076	4.850731016	39.20252151
45 to 49	0.005641758	2.637402197	91810.78579	4.530200184	30.07791202	0.002913791	2.673979612	96525.75536	4.793790081	34.54517973
50 to 54	0.008272759	2.664614226	89261.7805	4.378593865	25.85944559	0.004361862	2.670897741	95130.76545	4.708716496	30.01023305
55 to 59	0.012884961	2.621037808	85649.60565	4.155379611	21.83543397	0.006677659	2.642406175	93079.71076	4.581914213	25.61004677
60 to 64	0.01731179	2.658804109	80312.17899	3.859764887	18.10949163	0.009325899	2.656220856	90026.51752	4.405311759	21.38497727
65 to 69	0.030438043	2.635453845	73659.20634	3.437291319	14.50013241	0.014779218	2.710067273	85931.37995	4.156345456	17.27077824
70 to 74	0.046860339	2.577158007	63291.25777	2.8455777	11.43902124	0.027100769	2.697887143	79823.31224	3.758495349	13.37688105
75 to 79	0.071305922	2.53428474	50144.02116	2.136830311	8.767900019	0.050273459	2.669460559	69738.66197	3.125907386	9.907983565
80 to 84	0.117068392	2.440413453	35122.12379	1.356322517	6.45354793	0.10517892	2.529138457	54296.51734	2.168686658	6.961530986
85 to 89	0.181962755	2.298522068	19490.07708	0.657551651	4.703449322	0.16924386	2.342019126	32198.47332	1.127011607	5.014216953
90 to 94	0.275493555	2.093184652	7738.461179	0.216570427	3.433199126	0.257777017	2.143412545	13981.5765	0.41156934	3.647867237
95 plus	0.361333286	2.770381027	1863.574244	0.052199395	2.770381027	0.348310614	2.886305065	3847.684201	0.115038244	2.886305065

Table 15: Marshall Islands 2017 life table, by age and sex. mx=mortality rate, ax=mean person-years lived in an age interval among those who die in that age interval, lx=number of persons left alive at age x, nLx=person-years lived between age x and x+n, ex=life expectancy at age x.

Age Group	Male					Female				
	mx	ax	lx	nLx	ex	mx	ax	lx	nLx	ex
Early Neonatal	0.472203799	0.009574568	100000	0.019091512	62.71211531	0.341012662	0.009578589	100000	0.019115511	67.01231837
Late Neonatal	0.062749151	0.028749814	99098.59343	0.056912859	63.26300894	0.049238663	0.028753541	99348.19815	0.057078368	67.43256427
Post Neonatal	0.007969374	0.461077705	98741.56666	0.908324128	63.43407813	0.00526289	0.461269969	99067.21619	0.912457407	67.56618555
1 to 4	0.001147257	1.998470324	98017.98114	3.911737856	62.97571765	0.001340902	1.998212131	98587.1619	3.932929954	66.9696914
5 to 9	0.000538324	2.498878492	97569.26774	4.871905375	59.25619469	0.000467255	2.499026552	98059.81904	4.897268956	63.31904742
10 to 14	0.000619598	2.918232061	97307.06558	4.859086661	54.40914801	0.000453544	2.788662625	97831.02871	4.886651976	58.46125126
15 to 19	0.001793464	2.82541909	97006.04979	4.831465471	49.56883463	0.00110177	2.743169813	97609.42236	4.868369668	53.5875098
20 to 24	0.003474853	2.595219179	96139.6348	4.76715645	44.98937424	0.001756156	2.691318869	97073.09664	4.83406645	48.86799387
25 to 29	0.003470901	2.485859908	94483.40814	4.683322949	40.73101368	0.002761513	2.652617	96224.27784	4.780241656	44.27445443
30 to 34	0.003366163	2.586413449	92858.41239	4.605526858	36.39882489	0.003870235	2.628015776	94904.54987	4.702084531	39.85161751
35 to 39	0.005031259	2.687215519	91309.01648	4.512969117	31.9710663	0.005308656	2.613790169	93085.66874	4.596099661	35.57695349
40 to 44	0.008212271	2.657195146	89040.27533	4.368058509	27.71536613	0.007060148	2.603752646	90647.98928	4.457062286	31.46110656
45 to 49	0.011976127	2.615237067	85457.20477	4.154387578	23.76371145	0.009336334	2.609950841	87505.16712	4.279877935	27.49557667
50 to 54	0.016344194	2.652283129	80490.14049	3.876061388	20.06645133	0.012953537	2.601419494	83515.46708	4.05014733	23.68285488
55 to 59	0.027539792	2.635006227	74169.17539	3.48220552	16.54889494	0.017418108	2.592711333	78278.84675	3.756750997	20.09202037
60 to 64	0.042840295	2.564337336	64604.4357	2.92583192	13.6083358	0.024023054	2.596824241	71750.19415	3.392147062	16.68389058
65 to 69	0.059372782	2.485636408	52108.15414	2.268153951	11.25861752	0.035229132	2.586797479	63621.58593	2.93255538	13.48415222
70 to 74	0.070917936	2.453974429	38695.17087	1.640203238	9.30557101	0.052720935	2.567023778	53323.70447	2.364201055	10.59052784
75 to 79	0.10133994	2.445490125	27124.89296	1.078660055	7.239251868	0.085005918	2.507691012	40911.99866	1.689586503	8.029654907
80 to 84	0.151657438	2.36285282	16255.12163	0.58168165	5.461431554	0.130849473	2.409760924	26626.04851	0.995835456	6.004189313
85 to 89	0.21795519	2.216413707	7488.192283	0.233570758	4.11601542	0.197956116	2.272326961	13669.38752	0.444716342	4.431294659
90 to 94	0.30830292	2.023347092	2424.880191	0.063372055	3.115532512	0.286873305	2.076300211	4911.189115	0.133862503	3.309207535
95 plus	0.386574465	2.587194119	479.1010428	0.012429446	2.587194119	0.373564485	2.677567609	1087.57155	0.02921139	2.677567609

Table 15: Marshall Islands 2100 life table, by age and sex. mx=mortality rate, ax=mean person-years lived in an age interval among those who die in that age interval, lx=number of persons left alive at age x, nLx=person-years lived between age x and x+n, ex=life expectancy at age x.

Age Group	Male					Female				
	mx	ax	lx	nLx	ex	mx	ax	lx	nLx	ex
Early Neonatal	0.115927273	0.009585488	100000	0.019156781	71.68164121	0.073793621	0.009586779	100000	0.019164519	75.69156313
Late Neonatal	0.018119023	0.028762125	99777.95902	0.057376595	71.82099779	0.015629564	0.028762812	99858.59268	0.057427072	75.77899872
Post Neonatal	0.001943963	0.46150574	99674.03835	0.919453098	71.83797159	0.001228693	0.461556551	99768.8635	0.920631509	75.7892656
1 to 4	0.000262618	1.999649842	99495.38974	3.977727681	71.04200986	0.000338334	1.999548887	99655.79216	3.983537677	74.9510011
5 to 9	0.000152622	2.499682037	99391.01739	4.967656438	67.1139043	0.00016368	2.499659	99521.13288	4.97402199	71.04900403
10 to 14	0.000218779	3.311131859	99315.25148	4.963810835	62.16283515	0.000190319	2.900625318	99439.75869	4.969992525	66.10486388
15 to 19	0.000907255	2.828223145	99206.74897	4.950524233	57.22681705	0.000520435	2.755304032	99345.21739	4.961454239	61.16478477
20 to 24	0.00162326	2.604588334	98757.89604	4.918847339	52.47242949	0.000817986	2.733168434	99087.14402	4.945195192	56.31629798
25 to 29	0.001820668	2.525161409	97960.28421	4.876082813	47.87645347	0.001448116	2.684160346	98682.9052	4.917601999	51.53493801
30 to 34	0.001835086	2.5845608	97075.70994	4.832296838	43.2856419	0.002050417	2.647945623	97971.73066	4.874978215	46.88702018
35 to 39	0.002481449	2.667617063	96191.53916	4.781850652	38.65703794	0.002809418	2.617144755	96974.16253	4.816424914	42.33919089
40 to 44	0.003778838	2.645539107	95009.2369	4.708596013	34.10049678	0.003599196	2.612249772	95624.16302	4.740454617	37.89628665
45 to 49	0.005179027	2.629227164	93236.08638	4.605618378	29.6922555	0.004767827	2.626392721	93922.54263	4.643627577	33.53186154
50 to 54	0.007562049	2.747686106	90859.85115	4.466915318	25.39162671	0.006656972	2.626244389	91714.79477	4.514547977	29.27193554
55 to 59	0.01423978	2.69673795	87523.12421	4.237774435	21.23759039	0.009196559	2.588260249	88719.98604	4.340004112	25.16723476
60 to 64	0.021716901	2.656351184	81581.13641	3.884261629	17.57428702	0.011191592	2.615960585	84746.57884	4.127808087	21.22237499
65 to 69	0.037073399	2.578968665	73284.56349	3.369915151	14.23804635	0.017245255	2.659873853	80148.57479	3.85307503	17.28309592
70 to 74	0.047035934	2.536871672	61174.63665	2.751789266	11.53699204	0.027750801	2.688669872	73569.12934	3.4592731	13.5822774
75 to 79	0.072196854	2.539881317	48727.24464	2.085228042	8.848769463	0.053658554	2.626677773	64107.56011	2.850421044	10.17700638
80 to 84	0.120923696	2.441771407	34477.24967	1.33982158	6.511977456	0.092848033	2.50657575	49197.30023	2.011082952	7.470453778
85 to 89	0.184791595	2.29824324	19415.22949	0.668919452	4.740200653	0.153836544	2.381208209	31191.95392	1.128077167	5.352034812
90 to 94	0.277226799	2.088653653	8161.339977	0.23506944	3.45041814	0.241894424	2.179288259	14671.92323	0.445657831	3.844841875
95 plus	0.362286187	2.779288844	2146.678662	0.0629492	2.779288844	0.334401321	3.008057102	4396.283398	0.137103782	3.008057102

Table 15: Northern Mariana Islands 2017 life table, by age and sex. mx=mortality rate, ax=mean person-years lived in an age interval among those who die in that age interval, lx=number of persons left alive at age x, nLx=person-years lived between age x and x+n, ex=life expectancy at age x.

Age Group	Male					Female				
	mx	ax	lx	nLx	ex	mx	ax	lx	nLx	ex
Early Neonatal	0.113505972	0.009585562	100000	0.019157224	73.75072283	0.102291914	0.009585906	100000	0.019159284	79.3431168
Late Neonatal	0.023476232	0.028760647	99782.56379	0.057370394	73.89210296	0.017449688	0.02876231	99804.02405	0.057392679	79.4796814
Post Neonatal	0.001509605	0.461536596	99647.88504	0.919395841	73.9343929	0.000843703	0.4615839	99703.87741	0.920195166	79.50195144
1 to 4	0.000558394	1.999255474	99509.11114	3.975922782	73.11350179	0.000487475	1.999350033	99626.24302	3.981167366	78.64025875
5 to 9	0.000185154	2.499614262	99287.11295	4.962058594	69.27250779	0.00013999	2.499708355	99432.18977	4.969870011	74.789827
10 to 14	0.000231149	3.147327056	99195.24518	4.957637992	64.33431476	0.00015672	2.820536036	99362.61884	4.966434639	69.84041934
15 to 19	0.000904753	2.773468859	99080.6557	4.944070472	59.40503068	0.000381736	2.676070056	99284.78693	4.959839277	64.89294091
20 to 24	0.001424744	2.583629124	98633.36243	4.914752079	54.66169134	0.00048038	2.587606743	99095.45865	4.949041169	60.01175325
25 to 29	0.001496388	2.531049286	97933.16421	4.878642594	50.03385232	0.000589448	2.610784913	98857.72089	4.935939254	55.14974876
30 to 34	0.001678606	2.57385282	97203.19284	4.840454376	45.39040903	0.000802068	2.645848896	98566.78624	4.919052204	50.30466239
35 to 39	0.002127275	2.630301881	96390.83409	4.795371751	40.75105332	0.001158589	2.668593618	98172.29202	4.895387559	45.49593432
40 to 44	0.003059184	2.667679056	95371.08515	4.734777398	36.15843368	0.001749619	2.677906085	97605.23855	4.860510927	40.7444724
45 to 49	0.004695794	2.673036507	93923.31684	4.645425527	31.67441543	0.002680853	2.676101476	96755.08305	4.807800597	36.07864116
50 to 54	0.007217584	2.661612599	91743.24089	4.511066847	27.36329165	0.004091339	2.672154784	95466.64372	4.728307535	31.52913901
55 to 59	0.010891043	2.649955524	88489.91238	4.314156047	23.27127589	0.006253459	2.67235293	93532.92285	4.609571717	27.12539381
60 to 64	0.016434274	2.635595891	83795.5894	4.033201225	19.42612432	0.009737263	2.66370166	90651.66533	4.431807073	22.90222876
65 to 69	0.024732809	2.621757672	77173.37972	3.644541895	15.86566243	0.015034422	2.65756301	86338.57844	4.170147008	18.91282375
70 to 74	0.038109161	2.598539494	68170.09794	3.1231692	12.61285682	0.023884141	2.645155001	80072.85116	3.790603224	15.18439486
75 to 79	0.059612063	2.557647688	56289.51077	2.457624335	9.724976798	0.038672933	2.623434404	71026.35557	3.252697337	11.78109259
80 to 84	0.09476246	2.489725043	41680.31774	1.684830887	7.238325185	0.065465173	2.573300284	58461.56137	2.522988415	8.749171092
85 to 89	0.157168655	2.360024191	25777.99198	0.912127303	5.175470928	0.119827297	2.474172675	41975.34706	1.612000421	6.17628581
90 to 94	0.252165945	2.142101113	11501.08552	0.334734095	3.686271977	0.206110017	2.259703829	22702.00343	0.725996098	4.325052717
95 plus	0.343066562	2.915569197	3088.048376	0.090233971	2.915569197	0.302618365	3.305368933	7769.456875	0.257207844	3.305368933

Table 15: Northern Mariana Islands 2100 life table, by age and sex. mx=mortality rate, ax=mean person-years lived in an age interval among those who die in that age interval, lx=number of persons left alive at age x, nLx=person-years lived between age x and x+n, ex=life expectancy at age x.

Age Group	Male					Female				
	mx	ax	lx	nLx	ex	mx	ax	lx	nLx	ex
Early Neonatal	0.037665581	0.009587887	100000	0.019171159	78.04320726	0.030579299	0.009588104	100000	0.019172461	83.59140456
Late Neonatal	0.007756192	0.028764984	99927.8164	0.057479895	78.08034472	0.006044096	0.028765456	99941.38459	0.05749053	83.62117523
Post Neonatal	0.000480585	0.461609696	99883.25365	0.922005264	78.05768464	0.00031911	0.461621167	99906.65187	0.922289965	83.59267969
1 to 4	0.000198198	1.999735737	99838.96164	3.991976679	77.16882502	0.00014617	1.999805107	99877.23079	3.993921942	82.69386583
5 to 9	6.13352E-05	2.499872218	99759.89114	4.987229816	73.22843676	5.44327E-05	2.499886599	99818.87476	4.990264674	78.74103463
10 to 14	8.92472E-05	3.567517059	99729.30317	4.985807337	68.25011311	7.29598E-05	2.91999237	99791.71348	4.988823669	73.76177842
15 to 19	0.000500294	2.783941726	99684.80846	4.978714335	63.27900246	0.000196879	2.673250706	99755.31788	4.985478856	68.78761914
20 to 24	0.000768345	2.601887169	99435.73709	4.962643299	58.43039343	0.000233689	2.568279333	99657.17199	4.980028898	63.85270869
25 to 29	0.000881445	2.539109362	99054.47176	4.942010655	53.64508707	0.000274083	2.594467871	99540.80397	4.973765828	58.92430937
30 to 34	0.000946526	2.567163393	98618.94468	4.919618734	48.8705009	0.000363226	2.657934605	99404.49869	4.965983124	54.00137361
35 to 39	0.001193741	2.6398603	98153.4536	4.89387839	44.08974971	0.000533418	2.700233625	99224.16534	4.955116937	49.09449196
40 to 44	0.001754331	2.675526254	97569.51107	4.858651547	39.3373378	0.000865692	2.738980849	98959.92882	4.93829798	44.2180642
45 to 49	0.002691098	2.688092753	96717.56147	4.805969515	34.65954712	0.001501827	2.70044602	98532.5958	4.909639956	39.39750172
50 to 54	0.004253399	2.698391943	95425.01325	4.725003707	30.0915606	0.002295325	2.702104593	97795.62455	4.864115805	34.67333841
55 to 59	0.006954722	2.652071853	93417.08635	4.595842184	25.67885336	0.003776071	2.678044506	96679.75708	4.791949517	30.04147651
60 to 64	0.009771346	2.693555596	90225.73222	4.412053977	21.49124133	0.005629388	2.668232226	94871.77899	4.682196013	25.56166979
65 to 69	0.017468286	2.685718928	85924.67447	4.129668943	17.42753151	0.008727781	2.692501149	92238.57596	4.520898536	21.21300413
70 to 74	0.028633261	2.641075714	78747.10764	3.689579836	13.76676011	0.014532553	2.722593176	88300.77103	4.273886422	17.03605711
75 to 79	0.046280941	2.640379406	68260.66782	3.079811012	10.47196371	0.027299071	2.716038171	82108.43457	3.865285351	13.11058876
80 to 84	0.08954014	2.529538333	54171.0886	2.223479888	7.513570173	0.053463228	2.629184034	71615.61412	3.181167262	9.629499214
85 to 89	0.150873509	2.378289578	34540.84207	1.245235423	5.339923777	0.102844142	2.532561009	54774.89343	2.191091967	6.77173158
90 to 94	0.245715966	2.154011054	16147.30384	0.479327593	3.774441045	0.186341481	2.296348285	32589.54534	1.089923318	4.681112483
95 plus	0.337818253	2.965928184	4593.327992	0.138048071	2.965928184	0.284258836	3.528256175	12610.92081	0.450615551	3.528256175

Table 15: Papua New Guinea 2017 life table, by age and sex. mx=mortality rate, ax=mean person-years lived in an age interval among those who die in that age interval, lx=number of persons left alive at age x, nLx=person-years lived between age x and x+n, ex=life expectancy at age x.

Age Group	Male					Female				
	mx	ax	lx	nLx	ex	mx	ax	lx	nLx	ex
Early Neonatal	0.747529113	0.009566129	100000	0.01904128	56.83130367	0.571930814	0.009571512	100000	0.019073295	61.73762194
Late Neonatal	0.111203392	0.028736448	98576.82117	0.056534446	57.63189487	0.093002718	0.028741469	98909.25535	0.056754754	62.39856525
Post Neonatal	0.018816395	0.460307157	97948.36682	0.89653915	57.94385242	0.013289284	0.460699789	98381.5599	0.90279709	62.67538055
1 to 4	0.003331439	1.995558096	96262.42159	3.824976623	58.02754806	0.00343079	1.995425629	97182.32614	3.860758279	62.51965051
5 to 9	0.001284098	2.497324799	94989.29002	4.734256192	54.77914681	0.000922017	2.498079131	95858.67028	4.781906178	59.35560816
10 to 14	0.001060233	2.735919944	94381.61246	4.70778436	50.1154839	0.000781458	2.714285068	95417.91691	4.762393864	54.61808983
15 to 19	0.002513512	2.762675122	93882.64877	4.667907223	45.36696915	0.001742585	2.7153348	95045.84382	4.733458807	49.82091357
20 to 24	0.004332663	2.630314575	92709.61303	4.588408626	40.90445962	0.002623933	2.649886876	94221.16438	4.682223994	45.23225251
25 to 29	0.005422464	2.593329666	90722.32386	4.477730853	36.7388429	0.003740385	2.63258728	92992.96254	4.608894214	40.7926198
30 to 34	0.007090824	2.601788388	88296.39033	4.341089244	32.67246898	0.005194948	2.62455358	91270.28897	4.50795385	36.5091235
35 to 39	0.009421579	2.615563423	85222.862	4.167700507	28.7522497	0.007141101	2.608620107	88931.88266	4.37205845	32.39588574
40 to 44	0.013328363	2.607144882	81305.11923	3.939972129	25.00726512	0.009406841	2.59914488	85817.42051	4.196315513	28.47320368
45 to 49	0.018158848	2.595036291	76071.12245	3.645004301	21.54469733	0.012483637	2.599251547	81882.59622	3.975335958	24.71446991
50 to 54	0.02517145	2.574682308	69481.65812	3.275082734	18.34037864	0.017009166	2.589746837	76936.88868	3.695862546	21.13559178
55 to 59	0.033749867	2.554864268	61278.96378	2.831582512	15.45260615	0.022846885	2.584629153	70674.62323	3.349602649	17.77988331
60 to 64	0.046242933	2.535836214	51777.25003	2.32550001	12.82526581	0.0320334	2.583647312	63053.03658	2.92696354	14.61928093
65 to 69	0.064477166	2.49306423	41089.51571	1.770225909	10.51189961	0.04739418	2.559764412	53713.41745	2.408306698	11.71590594
70 to 74	0.084983503	2.441794524	29748.98588	1.223187561	8.584477563	0.070188936	2.517657835	42346.7799	1.804483473	9.179557155
75 to 79	0.114104664	2.391151303	19414.83444	0.74896904	6.872259764	0.107105534	2.448274844	29742.31373	1.169521615	7.012717882
80 to 84	0.154979218	2.311900654	10910.11922	0.385907367	5.383073204	0.157363844	2.341086762	17292.73279	0.610769771	5.317878897
85 to 89	0.22016473	2.211761388	4970.27232	0.154391268	4.084758505	0.225170173	2.218992138	7738.449592	0.238450537	4.019626293
90 to 94	0.310268822	2.019169694	1591.783867	0.04145974	3.098413382	0.313301142	2.017162746	2396.344021	0.06208906	3.066339256
95 plus	0.388064024	2.577255188	311.4393904	0.008052591	2.577255188	0.395610462	2.52817939	459.3026735	0.011647804	2.52817939

Table 15: Papua New Guinea 2100 life table, by age and sex. mx=mortality rate, ax=mean person-years lived in an age interval among those who die in that age interval, lx=number of persons left alive at age x, nLx=person-years lived between age x and x+n, ex=life expectancy at age x.

Age Group	Male					Female				
	mx	ax	lx	nLx	ex	mx	ax	lx	nLx	ex
Early Neonatal	0.143054773	0.009584656	100000	0.019151801	72.37946206	0.101447785	0.009585932	100000	0.019159439	77.28549475
Late Neonatal	0.02521528	0.028760168	99726.05664	0.057335045	72.55877409	0.023570147	0.028760621	99805.64312	0.057383515	77.41648209
Post Neonatal	0.003110796	0.46142285	99581.52308	0.918105029	72.6063019	0.002251417	0.461483899	99670.42029	0.919289121	77.46371991
1 to 4	0.000428101	1.999429199	99295.97465	3.968442039	71.89012272	0.000511478	1.999318029	99463.50523	3.974475625	76.70006646
5 to 9	0.000322261	2.499328624	99126.18969	4.952321556	68.00912859	0.000269656	2.499438218	99260.36516	4.959676818	72.85218594
10 to 14	0.000360798	2.987972869	98966.72668	4.944680572	63.11408864	0.000288906	2.790251002	99126.74647	4.953142269	67.94663126
15 to 19	0.001101463	2.77448758	98788.60174	4.927347541	58.22190133	0.000637685	2.693800418	98983.83708	4.941914393	63.04040485
20 to 24	0.001811295	2.628087771	98246.40514	4.891321899	53.5270038	0.000871252	2.632805256	98668.93187	4.923293567	58.2327783
25 to 29	0.002237275	2.573849535	97361.54325	4.841826789	48.98851036	0.001194657	2.627503312	98240.34147	4.898139662	53.47495666
30 to 34	0.002641271	2.577361973	96280.21587	4.783430634	44.50880216	0.00160964	2.626941245	97655.80432	4.864189737	48.77842275
35 to 39	0.003254202	2.601794229	95019.60457	4.71425269	40.06435066	0.00215915	2.624309773	96873.92562	4.818956333	44.14972434
40 to 44	0.00432114	2.623146097	93489.41805	4.627027332	35.67685846	0.002889924	2.628263033	95835.0739	4.759127252	39.59835232
45 to 49	0.005960731	2.63506888	91495.78681	4.511307689	31.39601973	0.003967886	2.634597457	94462.14961	4.679225694	35.13384946
50 to 54	0.008509823	2.634565477	88814.93906	4.353288112	27.26236455	0.005589897	2.628130019	92608.8988	4.569935539	30.78173224
55 to 59	0.012161293	2.614160958	85122.32775	4.136423096	23.3287229	0.007695847	2.599270108	90060.22198	4.421483777	26.57525805
60 to 64	0.016622293	2.63709937	80110.3879	3.854593076	19.62235904	0.009786096	2.621722811	86667.36178	4.235111876	22.51054498
65 to 69	0.026564632	2.606850876	73727.27827	3.466908396	16.0897005	0.01457882	2.656206316	82537.47728	3.991072421	18.50200022
70 to 74	0.037490654	2.571212153	64562.65601	2.960359928	12.99952404	0.023255153	2.674112911	76748.98526	3.641750141	14.69272543
75 to 79	0.054999988	2.556642129	53542.72761	2.362518172	10.14574948	0.041336219	2.658018134	68341.88924	3.118385604	11.16596378
80 to 84	0.088024394	2.47699825	40669.94309	1.668305658	7.554009202	0.077743614	2.551799072	55603.82356	2.342212168	8.114058219
85 to 89	0.14874937	2.383292032	26193.34046	0.947508968	5.376172648	0.135641222	2.42844741	37720.19137	1.407005786	5.76126742
90 to 94	0.243765929	2.158215762	12338.54494	0.366939532	3.793917367	0.223056562	2.222042242	19074.39643	0.594503611	4.083068574
95 plus	0.336310201	2.977127862	3525.508526	0.106066499	2.977127862	0.317837519	3.15530628	6108.028806	0.196060398	3.15530628

Table 15: Samoa 2017 life table, by age and sex. mx=mortality rate, ax=mean person-years lived in an age interval among those who die in that age interval, lx=number of persons left alive at age x, nLx=person-years lived between age x and x+n, ex=life expectancy at age x.

Age Group	Male					Female				
	mx	ax	lx	nLx	ex	mx	ax	lx	nLx	ex
Early Neonatal	0.221020909	0.009582267	100000	0.019137495	71.41343796	0.1819062	0.009583466	100000	0.019144669	74.68387317
Late Neonatal	0.030844136	0.028758615	99577.03557	0.057240097	71.69748373	0.027445367	0.028759553	99651.75378	0.057288645	74.92556507
Post Neonatal	0.004327064	0.461336448	99400.49776	0.915921924	71.76721698	0.003021356	0.461429204	99494.53093	0.917340735	74.98635059
1 to 4	0.000957569	1.998723241	99004.22268	3.952595647	71.12932395	0.000721932	1.999037424	99217.39075	3.962971469	74.27118213
5 to 9	0.000316698	2.499340213	98625.805	4.927388664	67.39458816	0.000275095	2.499426885	98931.32199	4.94316606	70.48012646
10 to 14	0.000341342	2.95017676	98469.78326	4.920047519	62.49741884	0.000261169	2.741806145	98795.35197	4.936855725	65.57362509
15 to 19	0.001058819	2.706035586	98301.85841	4.903191867	57.59909381	0.000579333	2.683231075	98666.42467	4.926709445	60.65563737
20 to 24	0.00140613	2.53576339	97782.72744	4.87226495	52.89015748	0.000774087	2.626131011	98381.02632	4.910035654	55.82363412
25 to 29	0.001324612	2.500020925	97097.73647	4.838870305	48.24485745	0.001060882	2.632961623	98000.97708	4.887787297	51.02958987
30 to 34	0.001427935	2.573717991	96456.99656	4.806201693	43.54829434	0.001474836	2.642817186	97482.50582	4.857237839	46.28632566
35 to 39	0.00184894	2.654812531	95771.0556	4.767880111	38.84144107	0.00209119	2.644539697	96766.34881	4.814592212	41.60847141
40 to 44	0.002832639	2.704050396	94890.12902	4.713856463	34.17709785	0.002955595	2.651640833	95759.97029	4.754995955	37.01706819
45 to 49	0.0047129	2.711213514	93555.95022	4.627902289	29.62566899	0.004328479	2.651829754	94355.3806	4.670319527	32.52769361
50 to 54	0.007882067	2.69690616	91376.87212	4.487437945	25.26728879	0.006317295	2.648251875	92335.15436	4.549218714	28.1802748
55 to 59	0.012924264	2.675992175	87843.46248	4.264198511	21.17510146	0.00928458	2.64686545	89463.61106	4.377623764	23.99850415
60 to 64	0.020836697	2.643237603	82338.18573	3.924367526	17.41228417	0.013859171	2.648588327	85403.58697	4.135558843	20.01205824
65 to 69	0.032305561	2.612209507	74169.26929	3.443117313	14.03947642	0.021493961	2.644639644	79679.0132	3.792280019	16.25802971
70 to 74	0.050417988	2.569750703	63057.04017	2.808986839	11.05416049	0.034455543	2.627258997	71541.94634	3.307359968	12.80459603
75 to 79	0.077351594	2.510705143	48907.52698	2.050815438	8.509824096	0.057230567	2.588684874	60174.47773	2.645232112	9.725437843
80 to 84	0.118181933	2.429093773	33056.4208	1.267887339	6.387416592	0.098728709	2.513826916	45101.71504	1.813399547	7.11093572
85 to 89	0.183283748	2.294330035	18082.87095	0.6046263	4.665520718	0.162021159	2.355368214	27337.14584	0.959752759	5.111234431
90 to 94	0.276876357	2.090458221	7012.103898	0.194272065	3.413180296	0.250900494	2.159317585	11932.76222	0.349863942	3.704660723
95 plus	0.362474865	2.759010972	1638.206388	0.045229654	2.759010972	0.342634321	2.921021154	3233.651647	0.095138262	2.921021154

Table 15: Samoa 2100 life table, by age and sex. mx=mortality rate, ax=mean person-years lived in an age interval among those who die in that age interval, lx=number of persons left alive at age x, nLx=person-years lived between age x and x+n, ex=life expectancy at age x.

Age Group	Male					Female				
	mx	ax	lx	nLx	ex	mx	ax	lx	nLx	ex
Early Neonatal	0.047548206	0.009587584	100000	0.019169341	77.85922729	0.035002073	0.009587968	100000	0.019171647	80.56612926
Late Neonatal	0.007846817	0.028764959	99908.86026	0.057468838	77.91078824	0.007646484	0.028765014	99932.89881	0.057482996	80.60077512
Post Neonatal	0.001164843	0.461561087	99863.77107	0.921534355	77.88830544	0.000771755	0.461589011	99888.94979	0.921933906	80.57851471
1 to 4	0.000238107	1.999682524	99756.45315	3.988360163	77.04785808	0.000192415	1.999743447	99817.81153	3.991177793	79.71199779
5 to 9	0.000116285	2.49975774	99661.58748	4.981635572	73.11813479	0.000110058	2.499770712	99741.10599	4.985687508	75.77086638
10 to 14	0.000156209	3.432256993	99603.86056	4.978743576	68.15802942	0.00012591	2.94987306	99686.41565	4.982934439	70.81039551
15 to 19	0.00059072	2.713796186	99526.56711	4.969558971	63.20732533	0.000278769	2.709702304	99623.98565	4.977991926	65.85241388
20 to 24	0.00069248	2.525487727	99234.01276	4.953246507	58.38429179	0.000370642	2.644427827	99485.58031	4.969942298	60.93974291
25 to 29	0.000678169	2.499697129	98892.45974	4.936320202	53.57616517	0.000529555	2.665002927	99301.81743	4.958948413	56.0470984
30 to 34	0.000715034	2.543768794	98559.68829	4.91939369	48.74775934	0.000767877	2.66007627	99039.8821	4.943068008	51.18741834
35 to 39	0.000825087	2.671495759	98210.36325	4.901070543	43.91154016	0.00106065	2.663206741	98661.27533	4.920821131	46.37259715
40 to 44	0.001303317	2.6957522	97809.07525	4.875807704	39.08019568	0.001537928	2.694288186	98140.58521	4.889676221	41.60327775
45 to 49	0.001988583	2.737850574	97177.29653	4.837073217	34.31633697	0.002476093	2.662324372	97390.16178	4.841508974	36.90200571
50 to 54	0.003494389	2.757107693	96220.53932	4.773648134	29.6297408	0.003502086	2.650130604	96193.84946	4.770458768	32.32564373
55 to 59	0.006395346	2.69373792	94559.10269	4.659309294	25.10122825	0.005073168	2.639833139	94527.25355	4.670501414	27.84614428
60 to 64	0.009784377	2.725748881	91589.33685	4.480002573	20.82670658	0.00710939	2.656899749	92163.96893	4.532966237	23.48966815
65 to 69	0.018491869	2.70515805	87218.8106	4.183891314	16.73240785	0.011137007	2.707283665	88951.36395	4.337182514	19.23690073
70 to 74	0.032654427	2.638032777	79509.13175	3.69236483	13.08936768	0.019715964	2.714320313	84148.28013	4.027227351	15.17272329
75 to 79	0.05240634	2.611852418	67537.85209	3.004450381	9.936110862	0.036581286	2.714449796	76283.79731	3.523239012	11.44086829
80 to 84	0.098309784	2.498070808	51960.23523	2.090951641	7.134966165	0.078609245	2.597511401	63596.51419	2.687365064	8.167813199
85 to 89	0.161150668	2.350762102	31692.23914	1.117080478	5.110451576	0.13646701	2.429812636	43141.04369	1.617711136	5.793854045
90 to 94	0.255783096	2.134078676	14024.09592	0.407843438	3.651462367	0.223390425	2.219489626	22133.97437	0.697403135	4.104026971
95 plus	0.345851511	2.895553202	3764.631214	0.110302527	2.895553202	0.317891387	3.168901496	7330.444031	0.241729367	3.168901496

Table 15: Solomon Islands 2017 life table, by age and sex. mx=mortality rate, ax=mean person-years lived in an age interval among those who die in that age interval, lx=number of persons left alive at age x, nLx=person-years lived between age x and x+n, ex=life expectancy at age x.

Age Group	Male					Female				
	mx	ax	lx	nLx	ex	mx	ax	lx	nLx	ex
Early Neonatal	0.513890771	0.00957329	100000	0.019083893	64.31271009	0.390162297	0.009577083	100000	0.019106513	67.63632672
Late Neonatal	0.068390897	0.028748258	99019.37338	0.056858134	64.93014682	0.056476908	0.028751544	99254.57426	0.057012705	68.12482507
Post Neonatal	0.009041239	0.461001562	98630.58812	0.90685472	65.12841763	0.006344745	0.461193116	98932.62423	0.910762943	68.28882992
1 to 4	0.001564997	1.997913339	97810.90967	3.900220462	64.74710656	0.001550072	1.997933239	98354.8756	3.922026621	67.76390643
5 to 9	0.000709806	2.498521237	97200.75912	4.851424986	61.14111334	0.000510478	2.498936505	97747.09153	4.881123676	64.17282393
10 to 14	0.000616819	2.768277995	96856.44595	4.836164428	56.34924603	0.000463005	2.75910992	97497.96206	4.869846194	59.33034228
15 to 19	0.001512548	2.747564545	96558.18538	4.811523997	51.51456538	0.001091732	2.720432881	97272.51396	4.8515556	54.46134024
20 to 24	0.002446633	2.617694224	95830.52264	4.763783826	46.88440961	0.001633223	2.652461645	96742.9236	4.818688065	49.74426344
25 to 29	0.002969203	2.586878736	94665.23978	4.699631152	42.42796211	0.002331833	2.634688239	95956.03846	4.77151305	45.12969321
30 to 34	0.003810899	2.610407752	93270.4895	4.621471702	38.02163544	0.003223478	2.630041508	94843.72158	4.706250157	40.62652286
35 to 39	0.005146597	2.635247606	91511.00532	4.520574358	33.70006435	0.004456388	2.624791883	93327.69306	4.617537915	36.24194197
40 to 44	0.00742937	2.631935311	89187.77575	4.382374296	29.50709661	0.006088296	2.621200575	91272.27637	4.498516184	31.99728222
45 to 49	0.010402413	2.634077838	85937.97246	4.193845624	25.52162083	0.008362324	2.623403338	88537.68168	4.340730295	27.90335452
50 to 54	0.015359399	2.622044929	81584.05931	3.935741748	21.74201617	0.011804626	2.613791692	84913.99331	4.129573507	23.98150722
55 to 59	0.021973106	2.607741275	75552.73809	3.589380988	18.26804033	0.016291211	2.608573909	80048.95674	3.852626849	20.28015143
60 to 64	0.032219253	2.581599208	67684.35483	3.1402062	15.08925106	0.023260482	2.604613202	73785.38553	3.494898776	16.78094754
65 to 69	0.045754139	2.546863498	57595.27449	2.589996238	12.2824454	0.034108311	2.59599266	65671.56871	3.035203309	13.53353411
70 to 74	0.064781413	2.512896408	45783.4669	1.972290068	9.798732066	0.052565629	2.571032857	55340.03559	2.454340895	10.57722023
75 to 79	0.095098984	2.459494109	33041.08889	1.331567092	7.613651981	0.084012014	2.512020418	42467.88219	1.757184022	8.006948684
80 to 84	0.138149333	2.370288891	20424.71818	0.750308245	5.806212017	0.133387331	2.416866064	27747.73271	1.032724093	5.928559059
85 to 89	0.203888935	2.246953014	10119.82068	0.324826853	4.325837859	0.200961212	2.265962624	14014.62755	0.45274436	4.380947369
90 to 94	0.295683836	2.050254999	3534.783936	0.094640218	3.229810717	0.289825578	2.069549133	4941.337166	0.133768429	3.27977139
95 plus	0.376960306	2.653351557	749.1718094	0.019940858	2.653351557	0.376061878	2.659470621	1073.330406	0.028592564	2.659470621

Table 15: Solomon Islands 2100 life table, by age and sex. mx=mortality rate, ax=mean person-years lived in an age interval among those who die in that age interval, lx=number of persons left alive at age x, nLx=person-years lived between age x and x+n, ex=life expectancy at age x.

Age Group	Male					Female				
	mx	ax	lx	nLx	ex	mx	ax	lx	nLx	ex
Early Neonatal	0.097372627	0.009586057	100000	0.019160187	74.71143686	0.068903745	0.009586929	100000	0.019165417	79.06909888
Late Neonatal	0.014558057	0.028763107	99813.44044	0.05740287	74.83166299	0.01371707	0.028763339	99867.94823	0.057435607	79.15423678
Post Neonatal	0.001756739	0.46151904	99729.88236	0.92004746	74.83668026	0.001277003	0.461553119	99789.17297	0.92079829	79.15900322
1 to 4	0.000311948	1.999584069	99568.27958	3.980248654	74.03374509	0.00031407	1.99958124	99671.61023	3.984362535	78.3282111
5 to 9	0.000202786	2.499577529	99444.19189	4.969692886	70.1228189	0.000174142	2.499637205	99546.55688	4.975164599	74.42345053
10 to 14	0.000237194	3.137281503	99343.55415	4.964870492	65.19063494	0.000197867	2.896663125	99460.05094	4.970877141	69.48560543
15 to 19	0.000813876	2.799588933	99226.11696	4.952404562	60.26343987	0.00049222	2.722252174	99361.91057	4.962515127	64.55111415
20 to 24	0.00137038	2.61786402	98823.6966	4.925127048	55.49651358	0.000703057	2.653423099	99117.91498	4.947736257	59.70298317
25 to 29	0.001601144	2.563163007	98149.83232	4.888456538	50.85855401	0.001010783	2.63690519	98770.42033	4.926749469	54.90327007
30 to 34	0.001881839	2.584647058	97368.77609	4.846429833	46.24502069	0.001360923	2.619315968	98273.04488	4.89776766	50.16708252
35 to 39	0.002368871	2.61380173	96459.07451	4.795865722	41.65578788	0.001773265	2.619016587	97607.3878	4.859848323	45.49059374
40 to 44	0.003191109	2.624224964	95325.90566	4.730476326	37.11893522	0.00238045	2.62495841	96746.72777	4.810147541	40.87121012
45 to 49	0.004332129	2.64389994	93820.41963	4.643700922	32.67115559	0.003233548	2.626920495	95603.2623	4.743795844	36.32771989
50 to 54	0.006346288	2.663822358	91814.51803	4.523768599	28.32602704	0.004447572	2.632808083	94071.66326	4.654631735	31.87524357
55 to 59	0.009730809	2.641977875	88951.84137	4.348036673	24.15068598	0.006260247	2.61669706	92005.29954	4.532708169	27.53055267
60 to 64	0.013936514	2.660907634	84733.32483	4.103273711	20.22026426	0.008283882	2.635322352	89174.02142	4.373215392	23.32025844
65 to 69	0.022965397	2.633182545	79035.03969	3.74875499	16.4843209	0.012480823	2.667810823	85559.58802	4.15725117	19.19194498
70 to 74	0.034255954	2.604089598	70465.51594	3.257330281	13.16597613	0.020192395	2.697288516	80388.23513	3.841464399	15.25159729
75 to 79	0.053869298	2.57991505	59371.50327	2.628536405	10.13950975	0.037394295	2.680488671	72667.11812	3.345081688	11.58098506
80 to 84	0.089465281	2.487657867	45327.67521	1.854386262	7.486818932	0.071853253	2.58707421	60257.09261	2.571870079	8.411750708
85 to 89	0.150624683	2.378131279	28926.33	1.041303664	5.331583217	0.128288453	2.449385105	41987.2943	1.587885829	5.950573433
90 to 94	0.245636366	2.154656604	13465.87981	0.398625004	3.769962831	0.215211923	2.239451363	21924.29159	0.691944172	4.193541952
95 plus	0.337813757	2.963424586	3797.206118	0.113537772	2.963424586	0.310813693	3.22380034	7255.46112	0.236682433	3.22380034

Table 15: Tonga 2017 life table, by age and sex. mx=mortality rate, ax=mean person-years lived in an age interval among those who die in that age interval, lx=number of persons left alive at age x, nLx=person-years lived between age x and x+n, ex=life expectancy at age x.

Age Group	Male					Female				
	mx	ax	lx	nLx	ex	mx	ax	lx	nLx	ex
Early Neonatal	0.31108706	0.009579506	100000	0.019120989	68.64940576	0.226954039	0.009582085	100000	0.019136407	75.02197691
Late Neonatal	0.04211505	0.028755506	99405.20372	0.05712281	69.04076427	0.033947162	0.028757759	99565.70879	0.05722848	75.32986891
Post Neonatal	0.005447151	0.461256879	99164.66268	0.913277039	69.15058602	0.003661146	0.461383754	99371.45321	0.915935648	75.41949807
1 to 4	0.001440279	1.998079629	98667.28662	3.935347701	68.5735121	0.000827452	1.998896731	99036.1644	3.954899236	74.74994041
5 to 9	0.00040643	2.49915327	98100.65191	4.900053174	64.95807808	0.000293503	2.499388535	98708.98098	4.931830035	70.99106937
10 to 14	0.000435638	2.931918688	97901.54324	4.890668015	60.08503837	0.000258	2.724971361	98564.25639	4.92532117	66.09158658
15 to 19	0.001312259	2.726376981	97688.52142	4.869897382	55.20962903	0.000572657	2.665304121	98437.19677	4.915289201	61.1733195
20 to 24	0.001877999	2.558504388	97049.54146	4.830331384	50.55538574	0.000715515	2.590497932	98155.74856	4.899348832	56.34093182
25 to 29	0.001866842	2.519337412	96142.53091	4.784980358	46.00824183	0.000896223	2.611119375	97805.21654	4.879825412	51.53322594
30 to 34	0.002100159	2.589440468	95249.35525	4.738496937	41.41563968	0.001214019	2.645243512	97367.9101	4.854519933	46.75233211
35 to 39	0.002834777	2.656320974	94254.40684	4.681634006	36.82462171	0.001759521	2.665640415	96778.69971	4.819126159	42.02005187
40 to 44	0.004334076	2.683384092	92927.84668	4.60022412	32.31111712	0.00262696	2.66920031	95931.14302	4.767364306	37.3668111
45 to 49	0.006872928	2.680946118	90935.61788	4.475491879	27.95861323	0.003957185	2.662298038	94679.48921	4.690595389	32.82453151
50 to 54	0.01085564	2.662830607	87863.055	4.284553574	23.84092787	0.00587548	2.65513842	92824.65232	4.578191101	28.42608858
55 to 59	0.016704149	2.640908365	83218.79862	4.003395197	20.02157842	0.008720194	2.655089192	90136.90991	4.416602352	24.19332218
60 to 64	0.025359412	2.611868269	76542.76372	3.608941468	16.53713556	0.013265554	2.653801661	86289.09124	4.184355994	20.15240848
65 to 69	0.037484812	2.580374281	67407.561	3.090559944	13.4249064	0.020649546	2.650037292	80744.23378	3.85065323	16.3524619
70 to 74	0.055391782	2.542429307	55843.51829	2.458173971	10.6719855	0.033472481	2.633079434	72805.43811	3.373683299	12.84434294
75 to 79	0.081995064	2.489894288	42252.49393	1.752806123	8.289133462	0.055987671	2.60151231	61543.09293	2.714338952	9.710317312
80 to 84	0.12198378	2.413625393	27917.09173	1.062175155	6.271380236	0.100960993	2.517229285	46418.68649	1.859003473	7.026067349
85 to 89	0.187207563	2.285134375	15014.15636	0.498503258	4.598587265	0.164746607	2.348718689	27814.99719	0.971354627	5.055574061
90 to 94	0.280481348	2.082761642	5720.146137	0.157568573	3.377134661	0.253665443	2.152927113	11983.68885	0.349648389	3.67234726
95 plus	0.365262141	2.738268687	1314.889954	0.036082708	2.738268687	0.345036513	2.901104734	3204.962677	0.093725285	2.901104734

Table 15: Tonga 2100 life table, by age and sex. mx=mortality rate, ax=mean person-years lived in an age interval among those who die in that age interval, lx=number of persons left alive at age x, nLx=person-years lived between age x and x+n, ex=life expectancy at age x.

Age Group	Male					Female				
	mx	ax	lx	nLx	ex	mx	ax	lx	nLx	ex
Early Neonatal	0.088354625	0.009586333	100000	0.019161844	74.5105023	0.055708929	0.009587334	100000	0.019167841	80.78340838
Late Neonatal	0.014356362	0.028763163	99830.7069	0.057413133	74.6175941	0.013288854	0.028763458	99893.22352	0.05745085	80.85048636
Post Neonatal	0.001913828	0.46150788	99748.2903	0.920150608	74.62172257	0.001305612	0.461551087	99816.88491	0.921041831	80.85471964
1 to 4	0.000398438	1.99946875	99572.22994	3.979717514	73.82943806	0.000252742	1.999663011	99696.65859	3.98585157	80.02824976
5 to 9	0.000123847	2.499741986	99413.69371	4.969146109	69.94391091	9.78079E-05	2.499796234	99595.94011	4.978579698	76.10711772
10 to 14	0.000174713	3.351726375	99352.15753	4.966149558	64.98566442	0.000101391	2.894978185	99547.25225	4.976293906	71.14309142
15 to 19	0.000812827	2.76329313	99265.40053	4.954234947	60.03953899	0.00028385	2.681129033	99496.80261	4.971563815	66.17767345
20 to 24	0.001179988	2.584339997	98862.77294	4.929097371	55.27237436	0.000344692	2.60324319	99355.69829	4.963684141	61.26781032
25 to 29	0.001307846	2.520589806	98281.3228	4.898209733	50.58321569	0.000455451	2.625598991	99184.62847	4.953873145	56.36891256
30 to 34	0.00134332	2.567343179	97641.10586	4.866152792	45.89702615	0.000619451	2.637576054	98959.05772	4.940710751	51.49122759
35 to 39	0.001747582	2.63937253	96988.12804	4.829495258	41.1875352	0.000854861	2.666506413	98653.11266	4.922816891	46.64238116
40 to 44	0.002534148	2.677020252	96145.37782	4.779126989	36.52346805	0.001290211	2.690529657	98232.42601	4.897011907	41.83019888
45 to 49	0.003917134	2.704700363	94936.74642	4.704533922	31.95175302	0.002033667	2.657978818	97600.83709	4.856901403	37.08292907
50 to 54	0.006476931	2.70533052	93098.53227	4.586804537	27.5264703	0.002858868	2.677140043	96613.59205	4.798817286	32.43370143
55 to 59	0.01068204	2.642464601	90136.96276	4.396390044	23.33941066	0.004560025	2.659955017	95242.47934	4.711870637	27.86087041
60 to 64	0.014791968	2.661806197	85458.51132	4.130598362	19.47050714	0.006574668	2.66638461	93095.79365	4.584601333	23.44007303
65 to 69	0.025145071	2.644556547	79375.40952	3.748022823	15.75642203	0.01043335	2.712793478	90086.0508	4.399397177	19.13087976
70 to 74	0.039167798	2.592149149	70016.79428	3.201382534	12.50801558	0.018450441	2.75453519	85512.85873	4.105975541	15.00488624
75 to 79	0.05921078	2.563148172	57589.18322	2.5198132	9.648965947	0.038092492	2.726415873	77979.38542	3.589920604	11.18266305
80 to 84	0.099011737	2.474831866	42838.68152	1.717799108	7.101330662	0.081077248	2.595656298	64444.86343	2.702319668	7.956851125
85 to 89	0.161752698	2.349049063	26035.11147	0.91567606	5.094891728	0.140250136	2.415740507	42840.48626	1.58192337	5.648528224
90 to 94	0.256399584	2.132979614	11456.57761	0.332347046	3.643070163	0.227912874	2.211081718	21156.81043	0.653515146	4.017457482
95 plus	0.346352045	2.890751597	3052.333586	0.089078809	2.890751597	0.322158329	3.114692793	6617.980551	0.21011987	3.114692793

Table 15: Vanuatu 2017 life table, by age and sex. mx=mortality rate, ax=mean person-years lived in an age interval among those who die in that age interval, lx=number of persons left alive at age x, nLx=person-years lived between age x and x+n, ex=life expectancy at age x.

Age Group	Male					Female				
	mx	ax	lx	nLx	ex	mx	ax	lx	nLx	ex
Early Neonatal	0.576531261	0.009571371	100000	0.019072458	62.25083193	0.436800492	0.009575653	100000	0.019097983	67.87891438
Late Neonatal	0.077431628	0.028745764	98900.56764	0.056775176	62.92068028	0.063981819	0.028749474	99165.86619	0.056949469	68.42898676
Post Neonatal	0.010772227	0.460878596	98461.09413	0.904575387	63.14281957	0.007539255	0.46110826	98801.56337	0.909055906	68.623007
1 to 4	0.001635391	1.99781948	97487.15584	3.886767795	62.8436444	0.00161776	1.997842988	98116.40063	3.911989764	68.17456711
5 to 9	0.000832432	2.498265766	96851.94127	4.832538367	59.24151986	0.000544859	2.498864876	97483.78096	4.867557917	64.60309916
10 to 14	0.000703989	2.755054654	96449.87535	4.81489047	54.47753752	0.000475086	2.74758806	97218.65723	4.855739074	59.77218045
15 to 19	0.001709342	2.760806943	96111.10737	4.787256302	49.65940057	0.001115347	2.703861433	96988.06072	4.837025452	54.90748688
20 to 24	0.0028934	2.631818455	95293.40836	4.732292992	45.06062	0.001584052	2.616804285	96448.79683	4.804332124	50.19874828
25 to 29	0.003631158	2.596329617	93925.80432	4.655721113	40.67602629	0.002047828	2.614793436	95688.2741	4.761205455	45.57582891
30 to 34	0.004719724	2.60605219	92238.02158	4.56046666	36.37014186	0.00278204	2.633403463	94714.44174	4.704794356	41.01578492
35 to 39	0.006263916	2.624930173	90090.19402	4.438622631	32.17229722	0.003914071	2.634848249	93407.89065	4.627625049	36.55052903
40 to 44	0.008917761	2.619510402	87317.00146	4.275357287	28.10802797	0.005461696	2.637814068	91600.75609	4.521811166	32.21748186
45 to 49	0.012224669	2.621662814	83516.65681	4.058276655	24.26525245	0.007820386	2.632830048	89137.94253	4.376086682	28.03292767
50 to 54	0.017759141	2.609252875	78575.86544	3.769446582	20.62441085	0.011098917	2.624255027	85725.83171	4.176496676	24.04271366
55 to 59	0.024914583	2.59498676	71912.99311	3.39336424	17.29345981	0.015762483	2.620931608	81106.70186	3.909207751	20.26217575
60 to 64	0.036067691	2.56996466	63504.14475	2.92070631	14.24221889	0.023045234	2.612523118	74966.58946	3.5534922	16.70787815
65 to 69	0.050878238	2.53609232	53032.09158	2.358079071	11.55305822	0.034232194	2.600468139	66807.23515	3.087664396	13.4314014
70 to 74	0.072467351	2.49837562	41119.05031	1.742422955	9.177087575	0.053297283	2.572569887	56276.76203	2.492688517	10.46152628
75 to 79	0.106032847	2.43634026	28582.7458	1.125370899	7.123564396	0.085735897	2.511254963	43046.46373	1.775520091	7.892171133
80 to 84	0.151481059	2.340507958	16728.2396	0.597669191	5.466622008	0.137220413	2.413711437	27904.21157	1.03142461	5.82441195
85 to 89	0.217231943	2.217969944	7745.728209	0.242100777	4.126644912	0.205121364	2.25759752	13832.37322	0.443575434	4.315902118
90 to 94	0.30765553	2.024725419	2522.196784	0.06603495	3.121331387	0.29387837	2.060400845	4782.026757	0.128604125	3.241530463
95 plus	0.386081994	2.590554052	501.0007999	0.01302366	2.590554052	0.379458115	2.635958249	1019.742943	0.026970187	2.635958249

Table 15: Vanuatu 2100 life table, by age and sex. mx=mortality rate, ax=mean person-years lived in an age interval among those who die in that age interval, lx=number of persons left alive at age x, nLx=person-years lived between age x and x+n, ex=life expectancy at age x.

Age Group	Male					Female				
	mx	ax	lx	nLx	ex	mx	ax	lx	nLx	ex
Early Neonatal	0.127481826	0.009585134	100000	0.019154659	72.73805886	0.089108505	0.00958631	100000	0.019161705	78.14628917
Late Neonatal	0.01916875	0.028761836	99755.83439	0.057362136	72.8965338	0.01825882	0.028762087	99829.26225	0.057405861	78.26049893
Post Neonatal	0.0023813	0.461474672	99645.89899	0.919007883	72.91924417	0.001678124	0.461524624	99724.46633	0.92003095	78.28500696
1 to 4	0.000347998	1.999536002	99427.11297	3.974319455	72.15491705	0.000364765	1.999513646	99570.11702	3.979902339	77.48200068
5 to 9	0.000266411	2.499444978	99288.90566	4.961143693	68.25172841	0.000212735	2.499556803	99425.05121	4.968612789	73.591473
10 to 14	0.000290468	3.00449463	99156.88554	4.954889416	63.33857385	0.000219511	2.836123283	99319.49065	4.963567887	68.66663014
15 to 19	0.000885365	2.772614517	99013.29539	4.940887153	58.42538059	0.000515582	2.69665726	99210.7572	4.954639494	63.73851802
20 to 24	0.001410019	2.632836835	98576.52667	4.91243103	53.67109129	0.000688221	2.612073103	98955.58481	4.939669043	58.89561525
25 to 29	0.001779929	2.58027076	97884.96776	4.873287349	49.03085446	0.000889987	2.621855399	98615.99835	4.920379355	54.08904969
30 to 34	0.002115929	2.595535035	97019.238	4.826419792	44.44443276	0.001197785	2.638338619	98178.68329	4.895054578	49.31775324
35 to 39	0.00276114	2.621660787	96000.22053	4.768733589	39.88766174	0.001650391	2.634850702	97593.18614	4.860670857	44.59724432
40 to 44	0.003782471	2.626331058	94686.49293	4.692265652	35.40361478	0.002254756	2.656413127	96792.1067	4.814161233	39.9438026
45 to 49	0.00518293	2.65198121	92915.91441	4.590023135	31.0267619	0.003366259	2.646219196	95708.11215	4.747808973	35.36530309
50 to 54	0.00782174	2.657414283	90543.56202	4.44585593	26.76858918	0.00470805	2.653827475	94112.26706	4.654239668	30.91894236
55 to 59	0.01172826	2.623052822	87075.5957	4.23594908	22.72731646	0.007068461	2.621945588	91924.5159	4.5203323	26.59026412
60 to 64	0.016100985	2.648914584	82122.28949	3.956810301	18.93844739	0.009311177	2.620951332	88735.02576	4.340802435	22.45038642
65 to 69	0.026722717	2.628331704	75773.38723	3.563817877	15.30081967	0.013503421	2.667666328	84702.2707	4.10609314	18.39257432
70 to 74	0.040342502	2.592926008	66299.25744	3.023312266	12.10990272	0.022479471	2.695406096	79175.16918	3.764429595	14.48843451
75 to 79	0.063579933	2.563065411	54189.51032	2.3487324	9.238054535	0.041775496	2.67782388	70746.98571	3.226145501	10.89083885
80 to 84	0.108007094	2.455745969	39390.70574	1.548906724	6.755217139	0.083294721	2.563172213	57359.5373	2.387856383	7.807233587
85 to 89	0.172002027	2.322692581	22851.99939	0.785929438	4.885323082	0.143121878	2.406095555	37659.78939	1.37825608	5.554417618
90 to 94	0.266208417	2.112791355	9515.227907	0.27056082	3.530869586	0.231180272	2.20435359	18189.98789	0.555615712	3.962061151
95 plus	0.354098071	2.826477859	2395.490896	0.068259085	2.826477859	0.325180441	3.080146636	5510.403534	0.171453292	3.080146636

Table 15: Southeast Asia 2017 life table, by age and sex. mx=mortality rate, ax=mean person-years lived in an age interval among those who die in that age interval, lx=number of persons left alive at age x, nLx=person-years lived between age x and x+n, ex=life expectancy at age x.

Age Group	Male					Female				
	mx	ax	lx	nLx	ex	mx	ax	lx	nLx	ex
Early Neonatal	0.582365945	0.009571192	100000	0.019071384	69.37363396	0.429462085	0.009575878	100000	0.019099321	75.6876196
Late Neonatal	0.048138797	0.028753844	98889.35635	0.056816531	70.13348111	0.033807574	0.028757797	99179.76065	0.057006869	76.2943101
Post Neonatal	0.009308886	0.460982549	98615.85323	0.906606481	70.27037701	0.007171027	0.461134418	98987.03655	0.910916266	76.38526241
1 to 4	0.001271552	1.998304598	97771.9279	3.900948573	69.94965112	0.001077438	1.998563416	98333.82739	3.924889562	75.96632299
5 to 9	0.000545728	2.498863067	97275.92195	4.85716649	66.2961428	0.00041604	2.49913325	97910.95485	4.890459467	72.28579405
10 to 14	0.000487966	2.739350724	97010.85827	4.845198112	61.47045835	0.000344865	2.589957295	97707.4944	4.881317744	67.43111145
15 to 19	0.001111952	2.711822812	96774.43192	4.826441647	56.6139367	0.000567048	2.62955987	97539.15584	4.870411388	62.54301284
20 to 24	0.001631873	2.584754214	96237.75707	4.792997029	51.91449735	0.00070066	2.582254891	97262.98096	4.85492481	57.71311571
25 to 29	0.001799039	2.554093487	95455.60809	4.751871161	47.31865811	0.00084879	2.603363406	96922.81797	4.836302719	52.90658049
30 to 34	0.002133798	2.59110442	94600.74149	4.705849191	42.72313674	0.001129911	2.643237001	96512.32135	4.812800088	48.12050862
35 to 39	0.002783434	2.629441503	93596.63007	4.649156194	38.15362709	0.001640985	2.655351194	95968.52509	4.780035227	43.37816952
40 to 44	0.003954462	2.657195894	92302.61064	4.572768074	33.65158057	0.002384895	2.657078876	95184.14332	4.732762883	38.71370414
45 to 49	0.005957278	2.668812914	90494.4148	4.462747937	29.27078711	0.003506094	2.65700535	94055.45886	4.664456808	34.14632186
50 to 54	0.009216443	2.662843001	87836.02515	4.29920288	25.07576899	0.005171712	2.648997067	92420.11933	4.565498415	29.7034194
55 to 59	0.014180341	2.650268412	83874.09892	4.058491663	21.13432532	0.007511875	2.661050849	90059.10541	4.425210455	25.41256446
60 to 64	0.021845566	2.626864365	78119.8156	3.713503847	17.49570379	0.011679789	2.662861927	86735.22372	4.221536526	21.28430172
65 to 69	0.032971408	2.597097774	70008.8655	3.243523138	14.21823561	0.018301955	2.657322533	81805.14117	3.922119513	17.40638077
70 to 74	0.049492687	2.558341377	59316.90878	2.646162706	11.31295091	0.029685582	2.637042867	74628.10562	3.486870979	13.82465384
75 to 79	0.073517929	2.509718219	46224.1439	1.953667277	8.79285167	0.048593856	2.600070634	64279.61822	2.878407548	10.62568916
80 to 84	0.11099428	2.436561205	31866.72039	1.240544681	6.624317201	0.081539135	2.530524276	50297.19873	2.093510189	7.856957977
85 to 89	0.175352963	2.313538825	18104.03423	0.615434357	4.80917575	0.140844166	2.411073484	33234.89153	1.217899025	5.592248388
90 to 94	0.269500013	2.10613137	7317.282165	0.20559234	3.490270781	0.228961576	2.209831248	16090.24845	0.491003409	3.983634107
95 plus	0.356732025	2.803282063	1778.619795	0.049872267	2.803282063	0.323292989	3.09331002	4853.322873	0.15018039	3.09331002

Table 15: Southeast Asia 2100 life table, by age and sex. mx=mortality rate, ax=mean person-years lived in an age interval among those who die in that age interval, lx=number of persons left alive at age x, nLx=person-years lived between age x and x+n, ex=life expectancy at age x.

Age Group	Male					Female				
	mx	ax	lx	nLx	ex	mx	ax	lx	nLx	ex
Early Neonatal	0.10599651	0.009585792	100000	0.019158604	77.79247556	0.090517831	0.009586267	100000	0.019161446	82.42746475
Late Neonatal	0.008174993	0.028764868	99796.9467	0.057403921	77.93151152	0.007576956	0.028765033	99826.56442	0.057421945	82.55142701
Post Neonatal	0.001495403	0.461537605	99750.02153	0.920344187	77.91060854	0.001138915	0.461562929	99783.05799	0.920800475	82.5298582
1 to 4	0.000251378	1.99966483	99612.40323	3.982493718	77.09428131	0.00022008	1.999706559	99678.19207	3.985373409	81.69288825
5 to 9	0.000154877	2.499677339	99512.30103	4.973689251	73.16976127	0.00014121	2.499705813	99590.49328	4.977767425	77.7630173
10 to 14	0.000184094	2.940457352	99435.27993	4.969868244	68.22444376	0.00015352	2.657256714	99520.21293	4.974216309	72.81613782
15 to 19	0.000533819	2.734363674	99343.80179	4.961187295	63.28451614	0.000252018	2.625259688	99443.86178	4.96921506	67.86997871
20 to 24	0.000784317	2.585873053	99079.00439	4.944589461	58.44621524	0.000301208	2.594414356	99318.64954	4.962336256	62.95219792
25 to 29	0.000861629	2.555318083	98691.29887	4.92419603	53.66549245	0.000388369	2.638147636	99169.20529	4.953911897	58.04310612
30 to 34	0.001020317	2.586808254	98267.1993	4.901282964	48.88583034	0.000555536	2.645149194	98976.84818	4.942371554	53.15064741
35 to 39	0.001283106	2.629774865	97767.37755	4.873546586	44.12225069	0.000772599	2.660702786	98702.33491	4.926213164	48.29088073
40 to 44	0.001831085	2.660940307	97142.3775	4.836405051	39.38879433	0.001156542	2.690456677	98321.82886	4.902989155	43.46704498
45 to 49	0.002727773	2.707192478	96257.25511	4.78295541	34.72597208	0.001837388	2.685347525	97754.9623	4.867049918	38.70292044
50 to 54	0.004635344	2.709218902	94953.42207	4.697799198	30.16486897	0.002823574	2.67035785	96861.15566	4.811421527	34.03413505
55 to 59	0.007628496	2.685398047	92777.82898	4.558390395	25.80750939	0.004229973	2.667773798	95503.74403	4.728519066	29.47812959
60 to 64	0.011998856	2.663792495	89305.39357	4.343635337	21.70528454	0.006359435	2.659892637	93506.40062	4.606877009	25.04855612
65 to 69	0.018773421	2.639885601	84101.08148	4.026919349	17.88051961	0.00960525	2.685474492	90581.57315	4.430701101	20.76765198
70 to 74	0.028488628	2.622271956	76561.38476	3.586013027	14.37814677	0.015951747	2.716544493	86338.95067	4.165729549	16.65200009
75 to 79	0.045263366	2.590532281	66385.38008	2.994673837	11.17528843	0.029808957	2.697469775	79723.8808	3.73160855	12.80186938
80 to 84	0.071681978	2.525819097	52920.60856	2.251412866	8.356206558	0.056378646	2.629752959	68685.05263	3.033758174	9.419961121
85 to 89	0.128763524	2.439266043	36973.02586	1.39591226	5.87182142	0.107009941	2.518394247	51805.07992	2.054727701	6.627680383
90 to 94	0.223576616	2.195444013	19285.56155	0.596277578	4.060743657	0.191169782	2.28737179	30228.00777	1.002262877	4.594955816
95 plus	0.320000922	3.129751618	6146.025693	0.194320988	3.129751618	0.28873362	3.474335625	11431.24667	0.403031187	3.474335625

Table 15: Cambodia 2017 life table, by age and sex. mx=mortality rate, ax=mean person-years lived in an age interval among those who die in that age interval, lx=number of persons left alive at age x, nLx=person-years lived between age x and x+n, ex=life expectancy at age x.										
Age Group	Male					Female				
	mx	ax	lx	nLx	ex	mx	ax	lx	nLx	ex
Early Neonatal	0.725304392	0.009566811	100000	0.019045326	66.82720091	0.634802373	0.009569585	100000	0.019061821	72.76411925
Late Neonatal	0.067151678	0.0287486	98618.79637	0.056630144	67.7435359	0.044590231	0.028754823	98790.05674	0.056765283	73.63587069
Post Neonatal	0.013495016	0.460685175	98238.61953	0.901399833	67.94804882	0.008943083	0.461008535	98536.9901	0.906035123	73.76738838
1 to 4	0.001300142	1.998266478	97022.66328	3.870837813	67.87077428	0.001336045	1.998218607	97726.94218	3.898654546	73.45186357
5 to 9	0.000611099	2.498726878	96519.67017	4.818620108	64.2142408	0.000456908	2.499048109	97206.25521	4.854765745	69.83476174
10 to 14	0.00056832	2.7321506	96225.28555	4.805071783	59.40299357	0.000375554	2.601003494	96984.45952	4.844858365	64.98867547
15 to 19	0.001250716	2.701972239	95952.25788	4.783866544	54.56415098	0.000641409	2.646052846	96802.52656	4.832831194	60.1058895
20 to 24	0.001800708	2.569509373	95353.93844	4.746927499	49.88903809	0.0008308	2.593316436	96492.54841	4.815001995	55.29027903
25 to 29	0.001881213	2.549154693	94499.32534	4.703295814	45.31632045	0.001022154	2.618861473	96092.54183	4.792962232	50.50934442
30 to 34	0.002289335	2.614044548	93614.97181	4.655334702	40.7197377	0.001425689	2.655936824	95602.6888	4.764215134	45.75431559
35 to 39	0.003194723	2.658678544	92549.93389	4.593160306	36.1576838	0.00211346	2.66812791	94923.57833	4.722911034	41.0621751
40 to 44	0.004839709	2.66984066	91083.79197	4.503439694	31.69628536	0.003187869	2.658559817	93925.66621	4.661501493	36.46935312
45 to 49	0.007408549	2.668125249	88906.5175	4.369902695	27.40650636	0.004657561	2.654593761	92440.19358	4.572087144	32.01152057
50 to 54	0.011466088	2.652435849	85673.03595	4.171491454	23.3397055	0.00689788	2.642034349	90311.88237	4.443382353	27.70189135
55 to 59	0.017285217	2.638451625	80897.10951	3.886410092	19.56107852	0.009900978	2.646302458	87249.49206	4.263234192	23.57972196
60 to 64	0.026441659	2.611320905	74189.47579	3.489366295	16.09194181	0.015015535	2.647690158	83034.14475	4.010284009	19.64041746
65 to 69	0.039208648	2.580359895	64975.72892	2.967595805	13.0051906	0.023247911	2.637919207	77022.98383	3.651125907	15.96488364
70 to 74	0.058804889	2.538149264	53354.73028	2.330629219	10.27811605	0.03691226	2.614058781	68556.85534	3.151179032	12.60964477
75 to 79	0.086938531	2.482328252	39660.50752	1.627049301	7.95257713	0.059686314	2.5695184	56963.3288	2.488673875	9.644964822
80 to 84	0.131478645	2.403700742	25520.33712	0.951295805	5.984281201	0.097971643	2.491959949	42173.47479	1.694522823	7.13115963
85 to 89	0.197444273	2.261215264	13012.58563	0.422291537	4.425051218	0.160916531	2.357718592	25658.13121	0.901701755	5.129360732
90 to 94	0.289873733	2.062683363	4675.435412	0.12627271	3.283661464	0.249823746	2.161877871	11223.9105	0.329087385	3.715137332
95 plus	0.372518952	2.684471819	1015.599757	0.027266435	2.684471819	0.341722737	2.927433669	3039.570174	0.08927681	2.927433669

Table 15: Cambodia 2100 life table, by age and sex. mx=mortality rate, ax=mean person-years lived in an age interval among those who die in that age interval, lx=number of persons left alive at age x, nLx=person-years lived between age x and x+n, ex=life expectancy at age x.										
Age Group	Male					Female				
	mx	ax	lx	nLx	ex	mx	ax	lx	nLx	ex
Early Neonatal	0.113249698	0.00958557	100000	0.019157272	80.07856951	0.110142946	0.009585665	100000	0.019157844	84.12644184
Late Neonatal	0.008818053	0.028764691	99783.0706	0.05739488	80.2325978	0.006990774	0.028765195	99789.02824	0.057401328	84.28359972
Post Neonatal	0.0013532	0.461547707	99732.47104	0.920242841	80.21536471	0.000945319	0.461576682	99748.92747	0.920568325	84.25937411
1 to 4	0.000128391	1.99982882	99608.00017	3.983312969	79.39053421	0.00017412	1.99976786	99662.04343	3.985131872	83.40744522
5 to 9	0.000104377	2.499782552	99557.89224	4.976635612	75.4206829	0.000108964	2.499772998	99595.0069	4.978458887	79.45491603
10 to 14	0.000138779	3.056584782	99507.66586	4.974069152	70.45166329	0.000119453	2.708188075	99543.50965	4.975865696	74.49178317
15 to 19	0.000462219	2.756112311	99441.37173	4.967229445	65.49075407	0.000204562	2.640651316	99487.4239	4.972092444	69.53044706
20 to 24	0.000752622	2.575142387	99226.40353	4.953425503	60.61174269	0.000239767	2.593276589	99391.59515	4.966852243	64.59363198
25 to 29	0.000729451	2.542590108	98903.27904	4.937628912	55.7884686	0.000280811	2.672405342	99279.36481	4.960834506	59.66368255
30 to 34	0.0007958	2.603223688	98600.2605	4.921737931	50.95365978	0.0004349	2.684066368	99145.97903	4.952435855	54.74082226
35 to 39	0.000990033	2.662598641	98257.73241	4.902520298	46.12845762	0.000627251	2.694465944	98937.31163	4.939852044	49.85142189
40 to 44	0.001431025	2.692471837	97816.42181	4.875494999	41.3320362	0.000987732	2.72696765	98634.20363	4.920782128	44.99715715
45 to 49	0.002198567	2.728087564	97154.00143	4.834178734	36.60191897	0.001674757	2.705412533	98154.73838	4.889096221	40.20424214
50 to 54	0.00378615	2.722382075	96120.16997	4.765450972	31.97099327	0.002637349	2.669474762	97343.51306	4.837640287	35.51715983
55 to 59	0.006307752	2.683794387	94342.01434	4.649749138	27.52530697	0.003875202	2.655526419	96076.87913	4.760800655	30.95002681
60 to 64	0.009684854	2.662985942	91437.92834	4.471319635	23.31668366	0.005646925	2.656740077	94243.02251	4.650925351	26.49972258
65 to 69	0.014834644	2.656892746	87139.49845	4.211399409	19.33691209	0.008487995	2.664671757	91630.89391	4.492940821	22.17733692
70 to 74	0.023419211	2.636483436	80931.10904	3.835541339	15.61566361	0.01315958	2.704715563	87839.86575	4.263931569	18.01676341
75 to 79	0.036521109	2.609281298	72008.25263	3.313530585	12.2210517	0.023830249	2.697454551	82265.2592	3.900801374	14.05012034
80 to 84	0.058499464	2.565452664	60012.36044	2.631195346	9.139586921	0.043344426	2.648634187	73048.57719	3.318385571	10.47786761
85 to 89	0.111505555	2.490484444	44842.1801	1.758714058	6.3694945	0.087791981	2.587347783	58855.34805	2.435567294	7.363963158
90 to 94	0.205253761	2.224139636	25573.68517	0.819434461	4.331829936	0.168138858	2.325261381	37842.34343	1.312494023	5.041473868
95 plus	0.30494518	3.284857903	9011.875249	0.299206017	3.284857903	0.267170719	3.754748053	16154.12786	0.614425501	3.754748053

Table 15: Indonesia 2017 life table, by age and sex. mx=mortality rate, ax=mean person-years lived in an age interval among those who die in that age interval, lx=number of persons left alive at age x, nLx=person-years lived between age x and x+n, ex=life expectancy at age x.

Age Group	Male					Female				
	mx	ax	lx	nLx	ex	mx	ax	lx	nLx	ex
Early Neonatal	0.717182674	0.00956706	100000	0.019046796	69.13255806	0.468348487	0.009574686	100000	0.019092211	73.90954979
Late Neonatal	0.060505876	0.028750433	98634.00974	0.056649676	70.07065896	0.030847694	0.028758614	99105.824	0.05696922	74.557135
Post Neonatal	0.009957693	0.460936459	98291.25318	0.903352134	70.25737453	0.007940938	0.461079725	98930.08916	0.910069073	74.6319923
1 to 4	0.001211518	1.998384643	97391.75018	3.886246233	69.97872531	0.001056506	1.998591326	98207.42096	3.920008159	74.25449467
5 to 9	0.000502849	2.498952398	96920.94218	4.83996024	66.30896284	0.00039372	2.499179751	97793.27901	4.88485423	70.56049807
10 to 14	0.000450908	2.726934895	96677.56945	4.828929214	61.46960665	0.000320767	2.64205427	97600.95335	4.876359505	65.69459777
15 to 19	0.000998726	2.691485256	96459.83222	4.811897799	56.6022037	0.000614584	2.648251083	97444.53669	4.865195192	60.79579971
20 to 24	0.001379155	2.548941019	95979.2571	4.782795243	51.87208501	0.000761748	2.577827752	97145.52923	4.848331049	55.97473482
25 to 29	0.001337718	2.526401445	95319.64823	4.750264374	47.21334067	0.000905141	2.600891797	96776.21131	4.828325747	51.17845991
30 to 34	0.001569221	2.603674724	94684.22013	4.716476009	42.51320705	0.001209409	2.646870402	96339.18669	4.803289876	46.3987697
35 to 39	0.002147612	2.662158511	93944.13201	4.673742116	37.82757916	0.001775335	2.673292662	95758.28623	4.768218973	41.66412265
40 to 44	0.003299026	2.69092652	92940.4453	4.611893065	33.20726835	0.002725133	2.671851499	94911.79609	4.715672592	37.01177739
45 to 49	0.005311122	2.701788645	91419.06893	4.515838096	28.71495716	0.004111915	2.662596146	93626.7765	4.636776511	32.48293888
50 to 54	0.008813751	2.691970642	89020.88196	4.362316609	24.41545173	0.006133532	2.654434643	91720.31346	4.520980789	28.10255584
55 to 59	0.014419419	2.670838156	85176.63027	4.120474669	20.39544717	0.009099528	2.654912572	88947.64793	4.354474684	23.89554558
60 to 64	0.023208411	2.634919498	79236.61763	3.755749983	16.72356469	0.013896644	2.654552018	84985.94297	4.115195205	19.88533445
65 to 69	0.035668276	2.598262344	70523.49983	3.248076584	13.46358493	0.021759625	2.649136047	79268.51977	3.77060786	16.1277353
70 to 74	0.054522105	2.556001617	58944.90489	2.600945594	10.59754958	0.035362847	2.627122944	71066.66944	3.278382492	12.68285685
75 to 79	0.083066824	2.498863964	44775.88333	1.854045466	8.142620283	0.058671956	2.581239111	59479.74414	2.604668159	9.641406285
80 to 84	0.127596861	2.415879254	29392.86184	1.105670731	6.098171502	0.099106218	2.498427626	44211.26053	1.77189823	7.079957375
85 to 89	0.193431191	2.270469398	15306.95848	0.501193526	4.491474171	0.162427276	2.353676451	26675.67491	0.933313583	5.09431034
90 to 94	0.286208643	2.070527357	5627.605449	0.153158911	3.319497426	0.251395098	2.158247143	11540.6081	0.336819946	3.694833867
95 plus	0.369694846	2.705115166	1249.670164	0.033834988	2.705115166	0.343112719	2.914893722	3085.700859	0.090046504	2.914893722

Table 15: Indonesia 2100 life table, by age and sex. mx=mortality rate, ax=mean person-years lived in an age interval among those who die in that age interval, lx=number of persons left alive at age x, nLx=person-years lived between age x and x+n, ex=life expectancy at age x.

Age Group	Male					Female				
	mx	ax	lx	nLx	ex	mx	ax	lx	nLx	ex
Early Neonatal	0.131801019	0.009585001	100000	0.019153867	77.27343034	0.083524279	0.009586481	100000	0.019162731	81.82452706
Late Neonatal	0.009002849	0.02876464	99747.58724	0.057374163	77.4496266	0.005079221	0.028765722	99839.9622	0.057433778	81.93635604
Post Neonatal	0.001352927	0.461547726	99695.93759	0.919905676	77.43218569	0.001077631	0.461567283	99810.79248	0.921082477	81.9027416
1 to 4	0.000237651	1.999683132	99571.4919	3.980967489	76.60504737	0.000179305	1.999760926	99711.54242	3.98703193	81.06045851
5 to 9	0.000150572	2.499686309	99476.89973	4.971973661	72.67589799	0.000118761	2.499752581	99640.06416	4.980524752	77.11711051
10 to 14	0.000166505	2.967419981	99402.05816	4.968404533	67.72864738	0.000121853	2.717924546	99580.93362	4.977655134	72.16136288
15 to 19	0.000507899	2.717529908	99319.37378	4.960208686	62.78248144	0.000238806	2.627578041	99520.30544	4.973194677	67.20363506
20 to 24	0.000687637	2.539520179	99067.53983	4.945012203	57.93507193	0.000264746	2.586641577	99401.57948	4.966903167	62.2807108
25 to 29	0.000640366	2.505541894	98727.64965	4.928512925	53.12572133	0.000346486	2.650798459	99270.12557	4.959466386	57.35970671
30 to 34	0.000707825	2.586852905	98412.23201	4.912219234	48.2878957	0.000512832	2.640917924	99098.34012	4.94892724	52.45447487
35 to 39	0.000935107	2.651157119	98064.71804	4.892490634	43.4497726	0.000692213	2.68788661	98844.60902	4.93433469	47.58222806
40 to 44	0.001391834	2.695628686	97607.46887	4.86476844	38.64079456	0.001143216	2.708467171	98503.13798	4.912286304	42.73766828
45 to 49	0.002257604	2.745092744	96930.69361	4.821989007	33.89160565	0.001847648	2.688096304	97941.71982	4.876257015	37.96676119
50 to 54	0.004114676	2.746643221	95842.54285	4.748096989	29.24492811	0.002845613	2.678778971	97041.11635	4.820215	33.29348356
55 to 59	0.007338398	2.721431542	93889.88177	4.61726821	24.79531995	0.00436764	2.668788513	95670.36517	4.735287496	28.73101132
60 to 64	0.012548721	2.686304986	90504.27197	4.397579992	20.61981814	0.006542893	2.663787274	93604.33978	4.609814129	24.30466556
65 to 69	0.020410112	2.652003603	84992.71483	4.055523319	16.78051227	0.010007681	2.687986052	90592.05005	4.427269812	20.0219284
70 to 74	0.032255028	2.633347045	76732.55077	3.565273258	13.29824017	0.016757568	2.738850956	86171.92058	4.151599046	15.90800846
75 to 79	0.054060849	2.587713562	65272.98784	2.888939842	10.16662541	0.033524691	2.706250009	79237.76874	3.680076057	12.05698246
80 to 84	0.088445172	2.51326896	49748.12319	2.042347557	7.5303224	0.065892993	2.622851258	66966.36924	2.898258186	8.766298322
85 to 89	0.149640729	2.380736526	31850.76849	1.148307525	5.353012599	0.120429837	2.473756759	48045.36599	1.847831355	6.181741956
90 to 94	0.24467906	2.156573369	14880.47347	0.44104526	3.781444901	0.206532054	2.25774445	26095.74607	0.837635507	4.329460994
95 plus	0.337052151	2.969996804	4210.382388	0.126067874	2.969996804	0.302911003	3.3083989	9037.241414	0.302280189	3.3083989

Table 15: Laos 2017 life table, by age and sex. mx=mortality rate, ax=mean person-years lived in an age interval among those who die in that age interval, lx=number of persons left alive at age x, nLx=person-years lived between age x and x+n, ex=life expectancy at age x.

Age Group	Male					Female				
	mx	ax	lx	nLx	ex	mx	ax	lx	nLx	ex
Early Neonatal	1.285843357	0.009549631	100000	0.018943583	64.99123296	0.958260984	0.009559671	100000	0.019002947	70.29084103
Late Neonatal	0.101702791	0.028739069	97564.69278	0.05596928	66.59307965	0.071249378	0.028747469	98179.24396	0.0563711	71.57448184
Post Neonatal	0.029415535	0.459554232	96995.751	0.883506234	66.92596913	0.024849024	0.459878618	97777.71381	0.892496377	71.81071615
1 to 4	0.002445641	1.996739151	94398.84983	3.757563261	67.83211052	0.002180795	1.997092277	95560.94697	3.805824363	72.54296184
5 to 9	0.000774268	2.498386943	93480.83201	4.66500998	64.47934408	0.000651785	2.498642115	94731.49077	4.728866499	69.16102233
10 to 14	0.000632195	2.626080059	93119.80166	4.649015171	59.71973038	0.000490001	2.552478297	94423.33764	4.715513017	64.37842897
15 to 19	0.001164778	2.704510564	92825.96311	4.628926414	54.90037911	0.000779879	2.64648764	94192.31562	4.700989439	59.53000311
20 to 24	0.001795999	2.594469322	92286.82769	4.594493805	50.2047845	0.001045488	2.595397965	93825.75351	4.679526237	54.75209293
25 to 29	0.002006076	2.559339735	91461.79999	4.550814291	45.63344607	0.001269614	2.60033277	93336.63055	4.652661268	50.02516331
30 to 34	0.002409902	2.598589067	90549.17017	4.501422082	41.06689674	0.001676049	2.636522911	92746.08327	4.619013208	45.32677626
35 to 39	0.003214177	2.634630599	89464.92458	4.43952015	36.53239504	0.002403098	2.632353767	91972.11732	4.572602999	40.68543534
40 to 44	0.004616389	2.660389612	88038.83216	4.354941154	32.08050917	0.003271287	2.640582792	90873.63703	4.508899612	36.14454089
45 to 49	0.007029326	2.672352558	86029.9953	4.232315566	27.76579114	0.004742544	2.659964941	89399.28381	4.420933187	31.69546668
50 to 54	0.011041172	2.657859279	83058.0786	4.048340239	23.66167157	0.007188618	2.646409851	87304.03975	4.292639479	27.39031707
55 to 59	0.016809566	2.64322965	78594.65693	3.780217731	19.85192402	0.010363698	2.654907662	84221.63952	4.111292449	23.29335418
60 to 64	0.025942831	2.609406754	72252.25173	3.402090859	16.36026834	0.016216061	2.645796059	79967.5967	3.851636865	19.3888161
65 to 69	0.037786898	2.587537734	63447.22316	2.908100716	13.26699216	0.024773592	2.628546527	73735.65567	3.482766758	15.8016941
70 to 74	0.058597435	2.537002186	52494.90443	2.2949783	10.49529369	0.038788594	2.598410805	65134.94578	2.980201206	12.5400642
75 to 79	0.082710532	2.478945547	39107.06334	1.619622189	8.224525207	0.060216745	2.55604985	53622.17809	2.338650576	9.676302668
80 to 84	0.124233806	2.407025754	25788.77398	0.976935439	6.203709125	0.096305982	2.485359346	39610.37563	1.596481176	7.201265381
85 to 89	0.189546057	2.279673365	13732.32979	0.454086328	4.559013652	0.158793317	2.363214948	24335.97582	0.859500136	5.176425127
90 to 94	0.282626033	2.078172833	5178.193866	0.142168363	3.355820073	0.247635737	2.166938042	10776.98982	0.317531521	3.742415097
95 plus	0.366918862	2.726001437	1179.29598	0.032251829	2.726001437	0.33979979	2.944268261	2957.786631	0.087440605	2.944268261

Table 15: Laos 2100 life table, by age and sex. mx=mortality rate, ax=mean person-years lived in an age interval among those who die in that age interval, lx=number of persons left alive at age x, nLx=person-years lived between age x and x+n, ex=life expectancy at age x.

Age Group	Male					Female				
	mx	ax	lx	nLx	ex	mx	ax	lx	nLx	ex
Early Neonatal	0.188374885	0.009583267	100000	0.019143484	80.42924494	0.13958015	0.009584763	100000	0.019152437	84.52508159
Late Neonatal	0.013285037	0.028763459	99639.42515	0.057304891	80.70092093	0.012584422	0.028763652	99732.68693	0.057359684	84.73224779
Post Neonatal	0.003325854	0.461407573	99563.30343	0.91784599	80.70503233	0.00285261	0.461441191	99660.50949	0.91894269	84.73600767
1 to 4	0.000250492	1.99966601	99258.10847	3.96833693	80.02817533	0.000236698	1.999684403	99398.41864	3.974056166	84.03476111
5 to 9	0.000107177	2.499776714	99158.76625	4.956610503	76.10584399	0.000115756	2.499758841	99304.41591	4.963784654	80.1121972
10 to 14	0.000137911	2.933734992	99105.6591	4.953861919	71.14519231	0.000130484	2.670332868	99246.97631	4.960837051	75.15705204
15 to 19	0.000385711	2.786183212	99037.35545	4.947643093	66.19215895	0.000218622	2.629383017	99182.26453	4.956545149	70.20429912
20 to 24	0.000669862	2.597061706	98846.55712	4.934405346	61.31429689	0.000266816	2.585157299	99073.93062	4.950513492	65.27815499
25 to 29	0.000718769	2.509573101	98516.15768	4.917119312	56.51064697	0.000334005	2.629289308	98941.88914	4.943187992	60.36179048
30 to 34	0.000809181	2.576380263	98163.18902	4.898559337	51.70370669	0.000480151	2.657359883	98776.91606	4.933269192	55.45801269
35 to 39	0.001006074	2.647410065	97767.88178	4.876791326	46.90113666	0.000666603	2.649557583	98540.30071	4.91928726	50.58455821
40 to 44	0.00143346	2.686556794	97279.1131	4.847804467	42.1224259	0.000936205	2.693631152	98212.71657	4.90002884	45.7441242
45 to 49	0.002171567	2.725986397	96587.31912	4.805572773	37.40390401	0.001511971	2.700230303	97754.42369	4.87077105	40.94551353
50 to 54	0.00367181	2.702756606	95549.04833	4.737438946	32.78008372	0.002389057	2.666970883	97018.67954	4.824043205	36.23476234
55 to 59	0.00565495	2.686808416	93817.95618	4.630333443	28.33494591	0.003458318	2.665708533	95867.52165	4.754971209	31.6364696
60 to 64	0.008914818	2.657767279	91209.4404	4.467414464	24.06860407	0.005211925	2.6601668	94225.45369	4.654603503	27.13970073
65 to 69	0.013184662	2.66112649	87242.06159	4.231887177	20.04136309	0.007786856	2.674428815	91803.81968	4.508619667	22.78219045
70 to 74	0.020989272	2.63566135	81688.10946	3.892043116	16.22283812	0.012330344	2.697053947	88303.35097	4.293580593	18.57594439
75 to 79	0.031680225	2.636499192	73561.96279	3.423447723	12.7162186	0.021387912	2.697854422	83030.32486	3.957530613	14.57971409
80 to 84	0.05464797	2.575793762	62807.81086	2.777228389	9.437595703	0.038773488	2.653904823	74615.28159	3.422229354	10.91575269
85 to 89	0.105942078	2.508175958	47845.75823	1.901300124	6.566769867	0.080706704	2.614620126	61474.83882	2.582929421	7.675953754
90 to 94	0.198862521	2.230686372	28154.46372	0.915219805	4.441892237	0.159221929	2.336879217	40915.04534	1.442949518	5.234482743
95 plus	0.29957842	3.347920844	10350.03601	0.351932384	3.347920844	0.258712918	3.876481505	18269.18736	0.715888851	3.876481505

Table 15: Malaysia 2017 life table, by age and sex. mx=mortality rate, ax=mean person-years lived in an age interval among those who die in that age interval, lx=number of persons left alive at age x, nLx=person-years lived between age x and x+n, ex=life expectancy at age x.

Age Group	Male					Female				
	mx	ax	lx	nLx	ex	mx	ax	lx	nLx	ex
Early Neonatal	0.166456722	0.009583939	100000	0.019147504	72.50862544	0.133730325	0.009584942	100000	0.019153511	77.20895882
Late Neonatal	0.018067368	0.028762139	99681.28518	0.057321081	72.72124993	0.015417721	0.02876287	99743.86403	0.057361437	77.38801707
Post Neonatal	0.002135945	0.461492102	99577.72729	0.91848293	72.7393185	0.001927812	0.461506887	99655.42947	0.919287919	77.39913265
1 to 4	0.000358252	1.999522331	99381.54984	3.972415223	71.95870901	0.000315653	1.99957913	99478.20966	3.976617468	76.61290622
5 to 9	0.000265576	2.499446717	99239.2456	4.958669444	68.05903601	0.000207742	2.499567203	99352.69027	4.965055486	72.70717177
10 to 14	0.000348518	2.956262815	99107.56143	4.95185058	63.14615131	0.00021346	2.610465521	99249.54704	4.959947639	67.78013415
15 to 19	0.001032225	2.694332443	98934.98587	4.935004716	58.25112534	0.000321736	2.611414689	99143.67323	4.953377347	62.84971238
20 to 24	0.00132085	2.556468797	98425.59092	4.905447257	53.53850947	0.00038678	2.581673912	98984.30644	4.944590616	57.9466578
25 to 29	0.001405807	2.565977484	97777.6731	4.872212647	48.87615568	0.000475151	2.621766625	98793.06225	4.934077768	53.05378197
30 to 34	0.001789048	2.600492197	97092.76324	4.83388896	44.20265938	0.000667333	2.669080251	98558.62413	4.920278391	48.1736761
35 to 39	0.00230383	2.622976223	96228.01082	4.785198887	39.57629703	0.001022764	2.687549112	98230.28786	4.899925957	43.32568168
40 to 44	0.003211517	2.649279979	95125.68863	4.720652028	35.00421759	0.001600867	2.716060203	97729.16595	4.868657163	38.53390549
45 to 49	0.004728442	2.68137926	93609.86385	4.629745116	30.52774364	0.00271694	2.717591235	96949.8158	4.817617581	33.82162198
50 to 54	0.007618143	2.680093881	91421.19817	4.491697686	26.19386383	0.004534492	2.685508377	95641.03649	4.732391336	29.24694912
55 to 59	0.012022995	2.669114542	88000.46206	4.280122271	22.10718338	0.006998138	2.681562819	93495.47499	4.600152376	24.8560484
60 to 64	0.019162392	2.649275766	82856.82985	3.964366135	18.31307338	0.011254503	2.679473878	90276.99192	4.399000965	20.64602682
65 to 69	0.030158998	2.613372166	75264.9065	3.510762249	14.89230574	0.018282763	2.692394511	85327.8928	4.093770443	16.68738746
70 to 74	0.045922068	2.564151525	64686.36591	2.909300567	11.89988836	0.032825998	2.654703524	77847.563	3.614359622	13.03131132
75 to 79	0.066777127	2.516305499	51343.83201	2.20256799	9.326485297	0.056050784	2.590442294	65994.12091	2.90757507	9.894370946
80 to 84	0.099222976	2.448905104	36663.39443	1.463580756	7.056103273	0.094135344	2.494191578	49721.37925	2.012385912	7.28541655
85 to 89	0.161997964	2.347311627	22174.94246	0.77605808	5.072254665	0.156289056	2.369403047	30817.90549	1.092758701	5.228348472
90 to 94	0.256838938	2.132586548	9630.53507	0.277533609	3.631072436	0.245089129	2.172850527	13779.11212	0.407374664	3.772514909
95 plus	0.346776875	2.883976956	2514.481459	0.072599722	2.883976956	0.337581816	2.962818487	3815.943099	0.113241443	2.962818487

Table 15: Malaysia 2100 life table, by age and sex. mx=mortality rate, ax=mean person-years lived in an age interval among those who die in that age interval, lx=number of persons left alive at age x, nLx=person-years lived between age x and x+n, ex=life expectancy at age x.

Age Group	Male					Female				
	mx	ax	lx	nLx	ex	mx	ax	lx	nLx	ex
Early Neonatal	0.046731557	0.009587609	100000	0.019169493	79.26019747	0.042267677	0.009587746	100000	0.019170313	82.19143689
Late Neonatal	0.005660197	0.028765562	99910.45347	0.057473369	79.31221593	0.005803725	0.028765522	99918.99856	0.057478047	82.23886704
Post Neonatal	0.000540792	0.461605419	99877.92878	0.921930452	79.28049943	0.000558677	0.461604148	99885.6443	0.921994069	82.20878203
1 to 4	0.00020057	1.999732574	99828.08203	3.991522097	78.39652804	0.000188023	1.999749302	99834.14824	3.991864807	81.32760225
5 to 9	8.74925E-05	2.499817724	99748.03476	4.986311372	74.45779007	8.08871E-05	2.499831485	99759.10264	4.986946949	77.38724353
10 to 14	0.000162765	3.511066941	99704.42499	4.983955138	69.48915917	0.000105405	2.7591311	99718.77893	4.984739816	72.4174973
15 to 19	0.000845539	2.726016741	99623.32483	4.971582068	64.54260288	0.000190888	2.610710184	99666.25159	4.981042579	67.45421316
20 to 24	0.001081096	2.543793656	99203.21985	4.947056304	59.80108473	0.000208562	2.582031615	99571.18235	4.976046357	62.51609587
25 to 29	0.001096225	2.546705744	98669.28875	4.920254742	55.10735795	0.000269764	2.6442509	99467.42425	4.970202788	57.57860802
30 to 34	0.001332648	2.593980219	98131.60119	4.890844277	50.39185044	0.000385828	2.662670093	99333.38303	4.962189716	52.65265183
35 to 39	0.001619617	2.606575693	97482.26321	4.855266014	45.70705884	0.000567465	2.697213914	99141.97418	4.950617103	47.749001
40 to 44	0.002110699	2.605513637	96698.85686	4.81072062	41.05336693	0.000912761	2.75525573	98861.1559	4.932937364	42.87652674
45 to 49	0.002781224	2.667059348	95686.89036	4.753655317	36.45705677	0.001681561	2.7484743	98411.09835	4.901989497	38.0592175
50 to 54	0.004493431	2.687594459	94372.09437	4.67020404	31.92315971	0.002940435	2.685267387	97587.39669	4.84636832	33.35557964
55 to 59	0.006981214	2.665615372	92287.19001	4.540612807	27.57817816	0.004336282	2.664621977	96164.32116	4.759933786	28.80652684
60 to 64	0.010439982	2.638460302	89141.12971	4.35070601	23.45192317	0.006412767	2.642091888	94104.44043	4.635415662	24.3758905
65 to 69	0.015643231	2.655958296	84634.11646	4.08334301	19.55267513	0.009350841	2.734409352	91138.28659	4.462203921	20.0773551
70 to 74	0.023796114	2.618556791	78358.616	3.710156376	15.90063796	0.017490468	2.752995148	86991.61227	4.185551794	15.89642331
75 to 79	0.035822425	2.598235712	69644.42116	3.211174967	12.55748199	0.034630627	2.708408159	79726.36263	3.695894036	12.08354264
80 to 84	0.056055424	2.519432212	58390.72764	2.574840471	9.477483211	0.067888427	2.575588828	67130.52608	2.894250868	8.830834054
85 to 89	0.106711246	2.5080538	44510.1337	1.779184055	6.613297258	0.121652443	2.476459386	48118.80362	1.864864456	6.247753011
90 to 94	0.198778993	2.224063559	26618.37837	0.875253967	4.472604491	0.206832239	2.252666431	26688.79228	0.870709885	4.373327522
95 plus	0.299299534	3.365634858	10157.89929	0.3549805	3.365634858	0.302770843	3.336873144	9718.312533	0.339024756	3.336873144

Table 15: Maldives 2017 life table, by age and sex. mx=mortality rate, ax=mean person-years lived in an age interval among those who die in that age interval, lx=number of persons left alive at age x, nLx=person-years lived between age x and x+n, ex=life expectancy at age x.

Age Group	Male					Female				
	mx	ax	lx	nLx	ex	mx	ax	lx	nLx	ex
Early Neonatal	0.232452362	0.009581916	100000	0.019135399	79.4434921	0.178882694	0.009583558	100000	0.019145224	82.89396236
Late Neonatal	0.014228655	0.028763198	99555.20435	0.057254899	79.77918889	0.016413701	0.028762596	99657.53465	0.057310147	83.15959884
Post Neonatal	0.001898441	0.461508973	99473.74215	0.917624403	79.78696491	0.001194768	0.461558961	99563.46924	0.918750414	83.18060702
1 to 4	0.000423383	1.999435489	99299.54785	3.968620743	79.0028347	0.000364838	1.999513549	99453.70364	3.975246968	82.34861554
5 to 9	0.000218707	2.499544361	99131.5372	4.953867938	75.13334937	0.000198761	2.499585915	99308.68038	4.962967619	78.4659575
10 to 14	0.000204059	2.567605017	99023.20017	4.948703284	70.21281962	0.000140043	2.444392797	99210.04081	4.958727229	73.54149025
15 to 19	0.00028496	2.66185246	98922.22191	4.942817922	65.2818686	0.00016152	2.583450435	99140.59882	4.955095985	68.59128409
20 to 24	0.000426515	2.56977218	98781.37327	4.933954479	60.37113874	0.00020513	2.606065428	99060.56489	4.950597256	63.64460092
25 to 29	0.000429192	2.547449386	98570.93452	4.923364333	55.49451211	0.000266472	2.635670463	98959.0145	4.944835419	58.70722028
30 to 34	0.000526383	2.63290965	98359.62961	4.911861497	50.6082329	0.000379533	2.673238355	98827.25041	4.93700296	53.78195512
35 to 39	0.00076857	2.640189725	98101.08076	4.896174228	45.73464324	0.000584046	2.605328308	98639.87801	4.925105782	48.87900486
40 to 44	0.00105005	2.69202892	97724.78192	4.874426356	40.90053515	0.000677838	2.673083678	98352.23426	4.909867368	44.01427902
45 to 49	0.001751785	2.745241899	97212.95569	4.841525012	36.10163859	0.001153683	2.801351112	98019.4336	4.888571613	39.15458097
50 to 54	0.003156467	2.755640977	96364.85738	4.784350518	31.3951024	0.002369582	2.713580669	97455.46766	4.846516648	34.36485918
55 to 59	0.005768559	2.756262137	94854.78204	4.682141003	26.85087385	0.003645074	2.788403575	96307.10808	4.776849383	29.74209224
60 to 64	0.010746331	2.691802396	92154.10835	4.496192291	22.55674442	0.007659904	2.677912665	94566.0599	4.645681215	25.23809589
65 to 69	0.017028993	2.648231458	87323.03618	4.198061851	18.6554157	0.010771349	2.648073798	91008.01226	4.437996926	21.11969405
70 to 74	0.02629329	2.613979417	80175.79737	3.772215636	15.08202023	0.016770584	2.655298057	86228.84705	4.148380354	17.14301622
75 to 79	0.039169748	2.593465158	70261.03951	3.210571353	11.8412148	0.02670951	2.669746312	79274.5869	3.731606762	13.41347364
80 to 84	0.062155947	2.547967228	57692.3378	2.503357764	8.85612718	0.04852451	2.61726546	69314.05935	3.106801528	9.957139241
85 to 89	0.116748788	2.473703373	42143.85848	1.627542043	6.184427284	0.095889966	2.555444379	54252.30839	2.197962039	6.995371124
90 to 94	0.211261014	2.217526011	23156.77118	0.729373983	4.229147891	0.178460367	2.312321072	33199.00608	1.122259388	4.813163611
95 plus	0.309992784	3.22604609	7757.270059	0.250355165	3.22604609	0.277000897	3.610651216	13191.86667	0.476684136	3.610651216

Table 15: Maldives 2100 life table, by age and sex. mx=mortality rate, ax=mean person-years lived in an age interval among those who die in that age interval, lx=number of persons left alive at age x, nLx=person-years lived between age x and x+n, ex=life expectancy at age x.

Age Group	Male					Female				
	mx	ax	lx	nLx	ex	mx	ax	lx	nLx	ex
Early Neonatal	0.049289377	0.00958753	100000	0.019169022	87.40918184	0.031170487	0.009588086	100000	0.019172351	90.37530264
Late Neonatal	0.002707616	0.028766376	99905.5299	0.057475417	87.47250481	0.004996648	0.028765745	99940.24502	0.057491603	90.41003039
Post Neonatal	0.000395017	0.461615774	99889.96916	0.922103613	87.42854328	0.000164571	0.461632145	99911.521	0.92240068	90.37842351
1 to 4	8.37939E-05	1.999888275	99853.54601	3.993472587	86.53694849	5.73641E-05	1.999923515	99896.34183	3.995395303	89.46878727
5 to 9	6.12219E-05	2.499872455	99820.08589	4.990240681	82.56525609	4.59738E-05	2.499904221	99873.42466	4.993097399	85.48884394
10 to 14	7.41658E-05	2.684726445	99789.54436	4.988600251	77.58973666	4.84304E-05	2.536426888	99850.47251	4.991929046	80.50790228
15 to 19	0.000108247	2.705477577	99752.55907	4.986377485	72.61750742	5.52957E-05	2.521523198	99826.30338	4.990629924	75.52674141
20 to 24	0.000169753	2.597101704	99698.60797	4.982884908	67.65517428	5.26578E-05	2.573872178	99798.7164	4.989300275	70.54690447
25 to 29	0.000173159	2.527552337	99614.08656	4.978582087	62.70993802	7.70787E-05	2.724399749	99772.45328	4.987722814	65.56478427
30 to 34	0.000200603	2.571329928	99527.94517	4.973985506	57.76159814	0.000111483	2.674513959	99734.03337	4.985392057	60.58879448
35 to 39	0.000251069	2.621736117	99428.29134	4.968428681	52.81621425	0.000152633	2.631073302	99678.47962	4.982112253	55.62087371
40 to 44	0.000325365	2.707146771	99303.75926	4.961459934	47.87830556	0.000197274	2.704875581	99602.47276	4.977872021	50.6609783
45 to 49	0.000545704	2.779065082	99142.61466	4.951045623	42.95077965	0.00034955	2.860607181	99504.31267	4.97146795	45.70797605
50 to 54	0.000968735	2.804566631	98872.99186	4.933160495	38.0589701	0.00077561	2.729322688	99330.65548	4.957730957	40.78226378
55 to 59	0.001977995	2.849404869	98395.99857	4.89845892	33.22829146	0.001133737	2.807618516	98946.54723	4.934895509	35.92894946
60 to 64	0.003784458	2.777649837	97430.61487	4.83091236	28.52657716	0.002299261	2.726553467	98387.77476	4.893730268	31.11609487
65 to 69	0.007175015	2.704389426	95607.53161	4.702772251	24.01274616	0.003586235	2.729560117	97264.5715	4.823691883	26.44018357
70 to 74	0.01083208	2.664425215	92254.19923	4.499394497	19.7797171	0.006069842	2.755213822	95539.62693	4.71294519	21.86383545
75 to 79	0.016779402	2.697498766	87422.94948	4.209932701	15.71717829	0.011568018	2.786122222	92691.90166	4.518795093	17.43879775
80 to 84	0.029710017	2.686463258	80471.19109	3.76979855	11.83093659	0.022730108	2.773931546	87527.82678	4.16848622	13.28452511
85 to 89	0.067336412	2.635127866	69577.66711	3.017440816	8.223195792	0.052688417	2.738888487	78281.12417	3.510547669	9.476831451
90 to 94	0.150654679	2.238242984	50156.85746	1.794818395	5.407721846	0.118498642	2.333549008	60587.79357	2.33194192	6.433300738
95 plus	0.25850067	3.902177723	24339.51169	0.978667057	3.902177723	0.218969995	4.641389412	34416.43255	1.658554253	4.641389412

Table 15: Mauritius 2017 life table, by age and sex. mx=mortality rate, ax=mean person-years lived in an age interval among those who die in that age interval, lx=number of persons left alive at age x, nLx=person-years lived between age x and x+n, ex=life expectancy at age x.

Age Group	Male					Female				
	mx	ax	lx	nLx	ex	mx	ax	lx	nLx	ex
Early Neonatal	0.353871084	0.009578195	100000	0.019113154	71.56098162	0.304091502	0.009579721	100000	0.019122269	78.13767559
Late Neonatal	0.054777461	0.028752013	99323.65725	0.057055169	72.02900676	0.038762142	0.028756431	99418.51496	0.057135961	78.57545361
Post Neonatal	0.003476743	0.461396854	99011.1423	0.912692115	72.19873605	0.003473029	0.461397117	99197.05	0.914407332	78.69328496
1 to 4	0.000426536	1.999431285	98693.84887	3.944388513	71.50609389	0.000374993	1.999500009	98879.48601	3.952214742	78.02126016
5 to 9	0.000168185	2.499649615	98525.62507	4.924210691	67.62478413	0.000105023	2.499781202	98731.28832	4.935268555	74.13537816
10 to 14	0.00019904	3.275724162	98442.81422	4.920451383	62.67957278	0.0002838	2.592420325	98679.45844	4.930604088	69.17300525
15 to 19	0.00091117	2.741742343	98344.88226	4.907147123	57.73872275	0.000231358	2.670279464	98539.53028	4.924322586	64.26753713
20 to 24	0.001263863	2.61884964	97897.76526	4.880201591	52.98982268	0.000474329	2.665655856	98425.60297	4.915837275	59.33881487
25 to 29	0.001648982	2.593149619	97280.98464	4.844821203	48.3090724	0.000610709	2.602128172	98192.43263	4.902442669	54.47334616
30 to 34	0.002028581	2.640534942	96482.10264	4.801126463	43.68747409	0.000777747	2.631063548	97893.0407	4.885651079	49.63192507
35 to 39	0.003071294	2.645139183	95508.19206	4.741122129	39.10588056	0.001107498	2.707017173	97513.06919	4.863303498	44.81499207
40 to 44	0.004271468	2.644595668	94052.14408	4.655769978	34.67009893	0.00189554	2.716740918	96974.47786	4.827829209	40.04873718
45 to 49	0.0062529	2.647256359	92063.64557	4.536453547	30.36151464	0.003125649	2.649522461	96059.39208	4.767942454	35.40419132
50 to 54	0.009145112	2.632852295	89227.4842	4.36685961	26.24204422	0.004238369	2.652698254	94569.21937	4.68188649	30.92007012
55 to 59	0.01301945	2.630329515	85234.84052	4.13422816	22.34753141	0.00645481	2.702232074	92585.09295	4.561607896	26.52555763
60 to 64	0.01927767	2.620157632	79853.96285	3.817619555	18.67574303	0.011083023	2.66418708	89641.15277	4.368979695	22.3075747
65 to 69	0.028361974	2.602922126	72497.4428	3.394234135	15.30462056	0.016562954	2.659548133	84800.18469	4.081834469	18.4284516
70 to 74	0.042514931	2.572793723	62876.04636	2.849932879	12.24813705	0.027343993	2.630264077	78042.04031	3.664767964	14.79344956
75 to 79	0.063111329	2.53190488	50769.01148	2.196653418	9.555862695	0.042192885	2.590017118	68027.22702	3.087671986	11.58369261
80 to 84	0.095752593	2.48072854	36920.52749	1.487644122	7.192033047	0.066019883	2.568594671	55011.66802	2.370530947	8.711900207
85 to 89	0.158300313	2.356885164	22694.31948	0.800310343	5.148620047	0.120576748	2.471692208	39381.04192	1.509524562	6.151929613
90 to 94	0.253292451	2.13989695	10040.80602	0.291264114	3.671915296	0.206961847	2.25799154	21204.28998	0.676707047	4.310651208
95 plus	0.343972549	2.907364126	2670.230375	0.07768223	2.907364126	0.303403777	3.296380224	7216.000251	0.238082813	3.296380224

Table 15: Mauritius 2100 life table, by age and sex. mx=mortality rate, ax=mean person-years lived in an age interval among those who die in that age interval, lx=number of persons left alive at age x, nLx=person-years lived between age x and x+n, ex=life expectancy at age x.

Age Group	Male					Female				
	mx	ax	lx	nLx	ex	mx	ax	lx	nLx	ex
Early Neonatal	0.063419948	0.009587097	100000	0.019166427	79.74664641	0.063485399	0.009587095	100000	0.019166414	84.70405629
Late Neonatal	0.017577425	0.028762275	99878.49336	0.057435303	79.82450001	0.014313203	0.028763175	99878.36021	0.057440616	84.7878017
Post Neonatal	0.000798418	0.461587117	99777.59177	0.920894797	79.84758687	0.000964807	0.461575297	99796.1793	0.920995672	84.79998629
1 to 4	0.000101744	1.999864342	99704.08373	3.987351972	78.9827978	9.26661E-05	1.999876445	99707.35409	3.987555182	83.951783
5 to 9	4.99231E-05	2.499895993	99663.51814	4.98255415	75.01410608	3.4448E-05	2.499928231	99670.40783	4.983091325	79.98216555
10 to 14	7.63174E-05	3.153949111	99638.64997	4.98121611	70.03217312	0.000130244	2.673828839	99653.24604	4.981105539	74.99550405
15 to 19	0.00055869	2.719398028	99600.65366	4.973479288	65.05766952	0.000101901	2.648117932	99588.40894	4.97821124	70.0425631
20 to 24	0.000832363	2.613595822	99323.11372	4.956209707	60.22978025	0.000188497	2.736821609	99537.71191	4.974725681	65.07688986
25 to 29	0.001125564	2.548080354	98911.49511	4.931919852	55.46762256	0.000280593	2.718824381	99443.9837	4.968947895	60.13554441
30 to 34	0.001120734	2.567892823	98358.24454	4.904499712	50.76362326	0.000420073	2.683300943	99304.67698	4.960303852	55.21556732
35 to 39	0.001565056	2.586460788	97810.36428	4.872080238	46.03261181	0.000556324	2.701464895	99096.50983	4.948472731	50.32492889
40 to 44	0.002046525	2.590399198	97050.42798	4.82867021	41.37078279	0.000946643	2.786695598	98821.40787	4.930534693	45.45652201
45 to 49	0.002854067	2.600840159	96065.67429	4.770656218	36.7658571	0.001685961	2.642534444	98355.38038	4.898231544	40.65696299
50 to 54	0.004297827	2.598151829	94708.73486	4.6871494	32.25234735	0.002051025	2.640595604	97530.91849	4.853102115	35.97599076
55 to 59	0.006170345	2.608442327	92705.00277	4.567864243	27.88912332	0.003159253	2.722122047	96537.51633	4.7920739	31.31657864
60 to 64	0.009464148	2.594613163	89910.69713	4.395890256	23.67207435	0.005164185	2.666999458	95029.83435	4.695159203	26.76686801
65 to 69	0.014213972	2.607341614	85783.74925	4.148900899	19.67657824	0.007815791	2.734769423	92613.15497	4.54990247	22.38960633
70 to 74	0.023582653	2.567753929	79960.15818	3.783644724	15.91589997	0.014294242	2.734352295	89083.82856	4.315064463	18.16236646
75 to 79	0.035098875	2.545625701	71189.15165	3.281367568	12.55115707	0.02632908	2.666254759	82985.5176	3.911743182	14.28124754
80 to 84	0.056613097	2.520649683	59906.86312	2.636897112	9.438420998	0.042001811	2.670106288	72891.16718	3.32742033	10.88429243
85 to 89	0.107406827	2.505873505	45501.51724	1.814585221	6.588132783	0.084331018	2.607535124	59404.95971	2.494855153	7.680173294
90 to 94	0.199586018	2.223423954	27062.04673	0.887586185	4.458331581	0.162100074	2.320360389	39638.29702	1.410590746	5.252997331
95 plus	0.299976656	3.35745029	10250.49691	0.35662682	3.35745029	0.261043195	3.890076082	18265.80811	0.745362344	3.890076082

Table 15: Myanmar 2017 life table, by age and sex. mx=mortality rate, ax=mean person-years lived in an age interval among those who die in that age interval, lx=number of persons left alive at age x, nLx=person-years lived between age x and x+n, ex=life expectancy at age x.

Age Group	Male					Female				
	mx	ax	lx	nLx	ex	mx	ax	lx	nLx	ex
Early Neonatal	1.008497649	0.009558131	100000	0.018993832	65.00787923	0.776928486	0.009565228	100000	0.019035922	72.24044811
Late Neonatal	0.065992686	0.028748919	98084.81494	0.056325404	66.25711396	0.071286573	0.028747459	98521.19114	0.056567369	73.30508918
Post Neonatal	0.01860431	0.460322223	97713.2592	0.894474399	66.45137525	0.015789613	0.460522172	98118.0365	0.899342506	73.54861171
1 to 4	0.002058178	1.997255766	96050.13807	3.82624595	66.67101663	0.001562846	1.997916207	96698.52459	3.855881722	73.69842831
5 to 9	0.000777591	2.49838002	95263.25743	4.753916505	63.20559376	0.000543394	2.49886793	96096.19668	4.798289536	70.14804305
10 to 14	0.000681666	2.729432501	94893.64472	4.737349828	58.44178143	0.000449868	2.590069881	95835.50345	4.786586637	65.33194338
15 to 19	0.00153831	2.723299376	94570.74669	4.712036762	53.63178074	0.000741474	2.637773807	95620.19951	4.772652604	60.47316043
20 to 24	0.002359362	2.583415122	93845.91342	4.665699275	49.02442828	0.000946771	2.597539603	95266.35603	4.752509782	55.68778874
25 to 29	0.002530981	2.540217022	92745.29088	4.608585696	44.57474267	0.001194618	2.617843964	94816.46859	4.727374393	50.93941479
30 to 34	0.002920448	2.584417548	91579.25727	4.546905247	40.10920386	0.001640745	2.635945608	94251.82528	4.694387574	46.22855354
35 to 39	0.00381434	2.625169182	90252.07865	4.472123592	35.66024077	0.00229744	2.626967337	93481.75563	4.648754914	41.58709411
40 to 44	0.005382935	2.644417212	88547.55773	4.371989881	31.29533068	0.003103085	2.631899244	92414.04781	4.587011839	37.03641081
45 to 49	0.007876505	2.655999455	86196.69141	4.231782455	27.07587474	0.004376713	2.650272586	90991.2606	4.50327658	32.57312338
50 to 54	0.012004918	2.646197461	82868.0174	4.029676878	23.05619455	0.00648782	2.642419793	89021.57103	4.384074446	28.23367209
55 to 59	0.01792853	2.634023446	78038.07862	3.743342482	19.31941882	0.009275469	2.652798445	86180.00491	4.217284467	24.07565904
60 to 64	0.0272954	2.608368422	71337.6556	3.348616412	15.88729091	0.014337702	2.65059108	82273.49283	3.979824662	20.09105287
65 to 69	0.040430492	2.576839668	62212.13627	2.833429018	12.83655006	0.022065695	2.642223677	76577.35564	3.639917099	16.38666523
70 to 74	0.060494932	2.532384545	50773.33809	2.209358477	10.14981137	0.035354859	2.613666625	68565.18756	3.162239524	12.99174115
75 to 79	0.088484846	2.476776457	37427.58251	1.530266286	7.86822838	0.055863689	2.572193264	57419.36138	2.529284022	10.00683875
80 to 84	0.133816946	2.397996629	23907.76224	0.887096398	5.92026521	0.091036137	2.505782347	43346.8089	1.76809095	7.424464493
85 to 89	0.199841024	2.255850779	12058.32641	0.389659362	4.387430233	0.152532326	2.379479572	27338.69551	0.978317052	5.316085999
90 to 94	0.292041589	2.058041855	4285.374766	0.115341563	3.263279039	0.241174113	2.181842607	12501.51967	0.373022892	3.823404591
95 plus	0.374179591	2.672704055	921.8516736	0.024663533	2.672704055	0.334117124	2.994265498	3550.635724	0.10672162	2.994265498

Table 15: Myanmar 2100 life table, by age and sex. mx=mortality rate, ax=mean person-years lived in an age interval among those who die in that age interval, lx=number of persons left alive at age x, nLx=person-years lived between age x and x+n, ex=life expectancy at age x.

Age Group	Male					Female				
	mx	ax	lx	nLx	ex	mx	ax	lx	nLx	ex
Early Neonatal	0.170432474	0.009583817	100000	0.019146776	77.39440487	0.138220226	0.009584805	100000	0.019152688	82.90473417
Late Neonatal	0.01041695	0.02876425	99673.71748	0.057329342	77.62845412	0.013745261	0.028763332	99735.29881	0.057359271	83.10543884
Post Neonatal	0.002303803	0.461480177	99614.00182	0.918746452	77.61737597	0.002128824	0.461492608	99656.46608	0.919212314	83.11353501
1 to 4	0.000241585	1.999677887	99402.37491	3.974177114	76.85794057	0.000208229	1.999722361	99460.8152	3.976778356	82.35247421
5 to 9	0.000160236	2.499666175	99306.53759	4.96334604	72.92814296	0.000157132	2.499672641	99378.14529	4.966963259	78.41802609
10 to 14	0.000212534	2.956752458	99227.34604	4.959145407	67.98276396	0.000172073	2.685881994	99300.43077	4.963016115	73.47634742
15 to 19	0.000543044	2.792347446	99122.83855	4.950164072	63.04936799	0.000271208	2.634811105	99215.53953	4.957586561	68.53610187
20 to 24	0.00089056	2.60551068	98856.06674	4.932309411	58.20994531	0.00032301	2.599228812	99081.73349	4.950248628	63.62445278
25 to 29	0.000957702	2.542411272	98419.66814	4.909492449	53.45500886	0.000409707	2.645117355	98922.56905	4.941361775	58.72220112
30 to 34	0.001069624	2.579223996	97953.19732	4.885069177	48.69673627	0.000589326	2.648303422	98721.05233	4.929211939	53.83617608
35 to 39	0.001335469	2.642207257	97434.80883	4.856515538	43.94188144	0.000797182	2.634659982	98431.69257	4.91234357	48.98618192
40 to 44	0.001950796	2.683430897	96791.20651	4.817861887	39.21653705	0.00110256	2.690763462	98041.38425	4.889609024	44.17007079
45 to 49	0.003024739	2.721979374	95857.54372	4.760148742	34.57252445	0.001771419	2.692874787	97504.03283	4.855350391	39.39791965
50 to 54	0.005191648	2.715504064	94425.23847	4.666036134	30.0558463	0.002716385	2.659675117	96646.39763	4.801843525	34.72270198
55 to 59	0.008601207	2.671400953	92013.56583	4.510532516	25.77278499	0.003896473	2.656735238	95346.02385	4.724205099	30.15836958
60 to 64	0.012923554	2.649114023	88149.56765	4.277826046	21.78657435	0.00569397	2.665196764	93512.55438	4.614493833	25.69592718
65 to 69	0.019696063	2.614940645	82639.88914	3.947069926	18.06298287	0.008757343	2.695995	90896.67194	4.45511084	21.35614007
70 to 74	0.027772086	2.602081932	74892.87031	3.511630351	14.66233228	0.014598438	2.735728034	87016.87827	4.212102355	17.1855216
75 to 79	0.042224138	2.596033229	65182.74076	2.960293338	11.46198584	0.028067961	2.700623094	80910.3951	3.801684262	13.27132209
80 to 84	0.068411385	2.551824312	52760.48642	2.262415393	8.544241879	0.051069193	2.638007509	70348.75119	3.14320387	9.85296861
85 to 89	0.124622572	2.451431647	37428.28058	1.425501564	5.987736576	0.099148251	2.546637043	54543.5778	2.203357514	6.929606808
90 to 94	0.21922562	2.202558434	19932.32318	0.62149301	4.123672101	0.181741073	2.302834098	33210.46711	1.124047702	4.778123482
95 plus	0.31643706	3.165751376	6506.411119	0.208154188	3.165751376	0.279903705	3.589372104	13290.60486	0.486491275	3.589372104

Table 15: Philippines 2017 life table, by age and sex. mx=mortality rate, ax=mean person-years lived in an age interval among those who die in that age interval, lx=number of persons left alive at age x, nLx=person-years lived between age x and x+n, ex=life expectancy at age x.

Age Group	Male					Female				
	mx	ax	lx	nLx	ex	mx	ax	lx	nLx	ex
Early Neonatal	0.519393317	0.009573122	100000	0.019082887	66.61469617	0.471426421	0.009574592	100000	0.019091651	73.23527889
Late Neonatal	0.04594744	0.028754449	99008.92605	0.056888825	67.26213677	0.037874686	0.028756676	99100.01157	0.056954374	73.88105539
Post Neonatal	0.010606491	0.460890369	98747.58356	0.907275314	67.38255117	0.006727063	0.461165957	98884.3217	0.910157621	73.98461649
1 to 4	0.00182157	1.997571243	97785.50083	3.897209174	67.1175973	0.001673812	1.997768253	98272.14692	3.917758456	73.51936587
5 to 9	0.00082298	2.49828546	97075.8297	4.843821035	63.59378505	0.000689835	2.498562844	97616.51357	4.872418861	69.99983452
10 to 14	0.000639725	2.568602011	96677.28669	4.826359286	58.84570569	0.000536512	2.485035754	97280.43469	4.857469253	65.23307483
15 to 19	0.001041543	2.71453443	96368.57709	4.806989586	54.02587244	0.000656564	2.605497532	97019.84268	4.843380601	60.40152098
20 to 24	0.001729291	2.646640768	95867.9423	4.773969437	49.29334773	0.000876654	2.615455116	96701.85072	4.825007757	55.59129663
25 to 29	0.002287826	2.600365669	95042.46403	4.726180221	44.69772239	0.001151656	2.610661107	96278.89094	4.800735999	50.82363432
30 to 34	0.0028854	2.601246074	93961.37522	4.66578612	40.18117726	0.001502857	2.636390606	95726.07578	4.769367515	46.10155584
35 to 39	0.003785015	2.622662651	92615.46204	4.589496283	35.7262185	0.002165298	2.630780491	95009.4369	4.726234097	41.42878374
40 to 44	0.005283204	2.645783489	90879.08258	4.488167969	31.35727571	0.00291386	2.637430339	93986.36688	4.667198562	36.85016074
45 to 49	0.007818339	2.649606934	88509.4891	4.345690495	27.1241197	0.004184387	2.64878075	92627.00676	4.586250256	32.35111646
50 to 54	0.011610476	2.650137354	85115.34455	4.142881784	23.09781673	0.006102071	2.643737007	90709.11058	4.471216952	27.97775437
55 to 59	0.017799065	2.635451473	80312.31732	3.853717575	19.31819282	0.008817824	2.663875814	87983.03581	4.310450544	23.76107322
60 to 64	0.026820671	2.617583641	73466.8587	3.453234338	15.87033864	0.014020061	2.656050369	84186.75915	4.075603961	19.71051623
65 to 69	0.041388266	2.579907206	64229.50761	2.920024442	12.77491408	0.02163772	2.650076119	78481.96257	3.734632051	15.94814324
70 to 74	0.061359576	2.526616747	52187.96249	2.267075997	10.12796473	0.035529713	2.628973246	70420.44515	3.248315094	12.46858616
75 to 79	0.088583952	2.473414001	38348.52031	1.568792939	7.876980064	0.059004502	2.597707965	58921.15685	2.581954286	9.388529256
80 to 84	0.133687133	2.393994063	24548.29023	0.912290673	5.929903999	0.108171047	2.523189114	43767.61849	1.728462159	6.744198073
85 to 89	0.199554302	2.256712046	12449.96408	0.403456318	4.394651961	0.173581318	2.326847095	25195.57676	0.862610942	4.871621843
90 to 94	0.291754738	2.0586597	4458.039162	0.120333834	3.267065929	0.262659285	2.132102306	10328.04164	0.295475229	3.565644927
95 plus	0.373946757	2.674851975	967.2218043	0.025971633	2.674851975	0.352872451	2.835326095	2616.209654	0.074532665	2.835326095

Table 15: Philippines 2100 life table, by age and sex. mx=mortality rate, ax=mean person-years lived in an age interval among those who die in that age interval, lx=number of persons left alive at age x, nLx=person-years lived between age x and x+n, ex=life expectancy at age x.

Age Group	Male					Female				
	mx	ax	lx	nLx	ex	mx	ax	lx	nLx	ex
Early Neonatal	0.100981572	0.009585946	100000	0.019159525	76.76273366	0.120677683	0.009585342	100000	0.019155908	81.02646773
Late Neonatal	0.009185078	0.02876459	99806.55132	0.057407778	76.89203209	0.010403622	0.028764253	99768.85638	0.057384085	81.19471693
Post Neonatal	0.002104583	0.46149433	99753.82684	0.920120723	76.87504616	0.001370566	0.461546473	99709.16193	0.920020246	81.18571157
1 to 4	0.000345027	1.999539964	99560.2323	3.979663173	76.10005318	0.000336003	1.999551996	99583.08838	3.980648603	80.36450469
5 to 9	0.000217013	2.49954789	99422.96631	4.968453035	72.20222167	0.000219867	2.499541943	99449.38231	4.969737406	76.46973269
10 to 14	0.000233388	2.727050175	99315.17801	4.9631108	67.27776599	0.000233158	2.591927916	99340.13674	4.964215469	71.55102336
15 to 19	0.000456974	2.72880462	99199.37316	4.954815933	62.35307015	0.000318834	2.628963206	99224.41873	4.95745727	66.6313454
20 to 24	0.000726553	2.637318684	98973.01487	4.940164079	57.48911676	0.000414827	2.611701831	99066.41381	4.948417494	61.73323218
25 to 29	0.000932139	2.606258427	98614.21833	4.919738004	52.68812641	0.000541244	2.637448357	98861.19663	4.936733404	56.85562782
30 to 34	0.00120999	2.613417413	98155.90061	4.893635241	47.92088852	0.000759303	2.653982687	98594.11982	4.920917149	52.00181806
35 to 39	0.001566992	2.647793055	97564.28936	4.860288414	43.19418587	0.001082418	2.648255718	98220.66606	4.89854854	47.18843393
40 to 44	0.002325721	2.655693983	96803.33582	4.813920252	38.51106815	0.00152033	2.66378634	97690.78824	4.867213808	42.42842688
45 to 49	0.003343984	2.689268655	95684.7881	4.747574958	33.92797417	0.002253578	2.661543221	96951.48655	4.822161677	37.72972889
50 to 54	0.005483104	2.693682106	94099.60082	4.646295204	29.45094224	0.003294267	2.655966233	95866.27815	4.756609932	33.12310881
55 to 59	0.008768094	2.669193437	91557.74039	4.486258261	25.18949611	0.004802394	2.674500575	94302.54804	4.663030833	28.62357641
60 to 64	0.013195521	2.649959933	87638.94964	4.250643307	21.19186512	0.007416525	2.66268854	92070.48652	4.525372164	24.24687301
65 to 69	0.020399795	2.635897055	82053.40448	3.914876647	17.4442275	0.011238784	2.689653635	88728.37141	4.324550959	20.04988166
70 to 74	0.030674876	2.618176639	74134.07587	3.456920144	14.01569388	0.018995344	2.693900732	83903.9662	4.020984387	16.03766928
75 to 79	0.049315658	2.586011824	63657.50442	2.85065739	10.87245676	0.033654642	2.68531244	76365.44229	3.547146743	12.33129004
80 to 84	0.079991764	2.508703947	49915.47039	2.099084734	8.134813865	0.06425661	2.633109643	64701.43333	2.822544381	9.051932779
85 to 89	0.137632748	2.419006005	33999.04955	1.280358062	5.745167734	0.117049353	2.49070768	47334.98005	1.855230041	6.388479277
90 to 94	0.231388999	2.175704651	17706.59025	0.551500593	3.995084135	0.201680542	2.262527026	26945.49605	0.88903175	4.457550079
95 plus	0.32595524	3.092046893	5805.107414	0.188087404	3.092046893	0.298082734	3.389488648	10113.78174	0.359355475	3.389488648

Table 15: Sri Lanka 2017 life table, by age and sex. mx=mortality rate, ax=mean person-years lived in an age interval among those who die in that age interval, lx=number of persons left alive at age x, nLx=person-years lived between age x and x+n, ex=life expectancy at age x.

Age Group	Male					Female				
	mx	ax	lx	nLx	ex	mx	ax	lx	nLx	ex
Early Neonatal	0.220022761	0.009582297	100000	0.019137679	73.86776863	0.179036674	0.009583554	100000	0.019145196	81.06871908
Late Neonatal	0.025226438	0.028760165	99578.96224	0.057250457	74.16073149	0.021575738	0.028761172	99657.24863	0.057301479	81.32824442
Post Neonatal	0.001906452	0.461508404	99434.56785	0.917259782	74.21082225	0.001741041	0.461520155	99533.63189	0.918243641	81.3716648
1 to 4	0.00034778	1.999536293	99259.74078	3.967630039	73.4174147	0.000286167	1.999618444	99373.78844	3.972677817	80.57851199
5 to 9	0.000220906	2.499539779	99121.79419	4.953354096	69.51680534	0.000149245	2.499689072	99260.12463	4.96115508	76.66849206
10 to 14	0.000265676	2.959859662	99012.38999	4.947938582	64.59086213	0.000135479	2.681061611	99186.08784	4.957749906	71.72387318
15 to 19	0.00081025	2.805501937	98880.94914	4.93527441	59.6727223	0.000270251	2.659907248	99118.92418	4.952812116	66.77058705
20 to 24	0.001464696	2.598593075	98481.09538	4.906798503	54.90315568	0.000341694	2.58202016	98985.07695	4.945166718	61.85707239
25 to 29	0.001520552	2.512850359	97762.47199	4.869712421	50.28679703	0.000404435	2.599713536	98816.1109	4.936013293	56.95825625
30 to 34	0.001583912	2.559364234	97022.14505	4.832435575	45.65050943	0.000536328	2.637115101	98616.49295	4.924585099	52.06809757
35 to 39	0.002005252	2.641770465	96256.9639	4.790207867	40.99218453	0.000761643	2.673000156	98352.39235	4.908922391	47.20062616
40 to 44	0.00300095	2.666568334	95296.8684	4.731730911	36.37745614	0.001178241	2.694694798	97978.55043	4.885660801	42.37016586
45 to 49	0.004512117	2.667584435	93877.89849	4.645053026	31.8855168	0.001881862	2.685806009	97403.01552	4.849042736	37.60399724
50 to 54	0.006873379	2.648094635	91784.1685	4.516288425	27.55011232	0.002903119	2.676768595	96490.7805	4.792246006	32.93315782
55 to 59	0.009913756	2.651324568	88684.54983	4.333487093	23.41825432	0.004461215	2.684703078	95100.28469	4.706464888	28.37407829
60 to 64	0.015242328	2.649726584	84396.99565	4.074212087	19.47118304	0.007132751	2.690338822	93002.74902	4.574890203	23.95120169
65 to 69	0.023606243	2.655171024	78202.11825	3.705588103	15.80202748	0.011624333	2.693432495	89745.51153	4.370353753	19.71983596
70 to 74	0.040125559	2.598199576	69482.12903	3.169839787	12.45115862	0.019638196	2.692627789	84679.80483	4.05100879	15.73538167
75 to 79	0.059425136	2.553864135	56815.92929	2.482005288	9.649497716	0.034851267	2.666280343	76756.56229	3.550317185	12.0800199
80 to 84	0.097622465	2.494663644	42149.54204	1.695600314	7.125757893	0.063578075	2.597732692	64443.04149	2.797342117	8.879564436
85 to 89	0.160583629	2.351158122	25706.45987	0.903639978	5.104508132	0.117293202	2.482650369	46762.79573	1.808103291	6.260700703
90 to 94	0.255446574	2.135356713	11287.80101	0.326689881	3.648315593	0.203207308	2.265365584	25701.53427	0.828188735	4.375195626
95 plus	0.345662544	2.893836265	2983.828109	0.086634328	2.893836265	0.299936566	3.336700999	8983.310146	0.301327839	3.336700999

Table 15: Sri Lanka 2100 life table, by age and sex. mx=mortality rate, ax=mean person-years lived in an age interval among those who die in that age interval, lx=number of persons left alive at age x, nLx=person-years lived between age x and x+n, ex=life expectancy at age x.

Age Group	Male					Female				
	mx	ax	lx	nLx	ex	mx	ax	lx	nLx	ex
Early Neonatal	0.037237051	0.0095879	100000	0.019171248	81.2721615	0.031730262	0.009588069	100000	0.019172252	87.17579044
Late Neonatal	0.005788205	0.028765527	99928.79691	0.05748372	81.31052458	0.005080462	0.028765724	99939.2318	0.05749089	87.20938204
Post Neonatal	0.000365323	0.461617877	99895.57976	0.922168132	81.27990482	0.000414364	0.4616144	99910.05989	0.922280958	87.1770348
1 to 4	0.000138245	1.999815673	99861.91943	3.993374068	80.38374083	0.000143789	1.999808281	99871.88161	3.993728032	86.28675629
5 to 9	9.52417E-05	2.499801583	99806.80769	4.989154811	76.42635007	9.08749E-05	2.499810678	99814.54464	4.989596152	82.33465355
10 to 14	0.000132358	3.289588316	99759.40187	4.986699049	71.46086366	0.000100467	2.849921771	99769.3163	4.987296235	77.37049894
15 to 19	0.00052099	2.847882994	99693.60801	4.979030097	66.50528198	0.000168787	2.675241332	99719.42525	4.983982659	72.40755759
20 to 24	0.001055818	2.596320154	99434.79785	4.959158401	61.66859315	0.000207354	2.596084776	99635.53991	4.979262462	67.46603831
25 to 29	0.00103759	2.484917228	98912.97768	4.932825266	56.97556318	0.00022495	2.599081338	99532.61492	4.973920549	62.53292966
30 to 34	0.000967755	2.492054812	98403.89468	4.90834889	52.25368885	0.0002858	2.637868646	99421.03995	4.967663082	57.60002206
35 to 39	0.001013107	2.593837537	97932.12942	4.884738248	47.49004938	0.000372175	2.670640291	99279.55123	4.959654753	52.67810507
40 to 44	0.001469497	2.641572161	97441.34884	4.855294767	42.71278347	0.000559755	2.756660102	99095.51909	4.948494775	47.77024367
45 to 49	0.002119829	2.679933497	96734.19168	4.813129357	38.00037971	0.000991599	2.706783957	98819.23838	4.929711836	42.89476908
50 to 54	0.003466923	2.656786234	95724.94965	4.747901079	33.36585573	0.001500262	2.671750129	98331.50997	4.89948186	38.09140945
55 to 59	0.004967381	2.684478848	94100.44706	4.651623412	28.88693719	0.002244694	2.684984866	97598.40864	4.854653994	33.3531859
60 to 64	0.007924631	2.661946136	91828.39153	4.508688925	24.5270344	0.003448445	2.675663578	96513.03081	4.787412638	28.69189492
65 to 69	0.012125859	2.684765333	88318.05264	4.296457109	20.38395565	0.005274578	2.710589167	94871.1731	4.687004984	24.13230658
70 to 74	0.020572646	2.635562929	83225.75374	3.97225333	16.46042377	0.008779392	2.774736765	92420.78813	4.533090721	19.68917911
75 to 79	0.031466371	2.623518218	75272.82002	3.509873081	12.90885985	0.017904372	2.766393457	88491.10105	4.256521928	15.42030556
80 to 84	0.053881181	2.565432607	64658.95312	2.87281467	9.601243234	0.03522691	2.702249209	81046.01892	3.758161094	11.55682214
85 to 89	0.104012792	2.515875245	49895.69846	2.006128236	6.686535988	0.073971712	2.64719812	68322.34476	2.932913608	8.166201803
90 to 94	0.195971632	2.228819389	30221.84726	0.997711183	4.512242461	0.148806173	2.332747718	47891.87061	1.746063049	5.56226799
95 plus	0.297008016	3.388317277	11646.41748	0.408134424	3.388317277	0.248424358	4.085844686	23544.96992	1.008231928	4.085844686

Table 15: Seychelles 2017 life table, by age and sex. mx=mortality rate, ax=mean person-years lived in an age interval among those who die in that age interval, lx=number of persons left alive at age x, nLx=person-years lived between age x and x+n, ex=life expectancy at age x.

Age Group	Male					Female				
	mx	ax	lx	nLx	ex	mx	ax	lx	nLx	ex
Early Neonatal	0.304569014	0.009579706	100000	0.019122182	70.11743568	0.251654586	0.009581328	100000	0.019131879	77.60484102
Late Neonatal	0.02740949	0.028759562	99417.61604	0.057154101	70.50890467	0.023804837	0.028760557	99518.55685	0.05721806	77.96104726
Post Neonatal	0.004442348	0.461328259	99260.96834	0.91458754	70.56259754	0.003913011	0.461365862	99382.35124	0.915929505	78.01031303
1 to 4	0.000509849	1.999320201	98854.71412	3.950159728	69.92740468	0.000381888	1.999490816	99023.96211	3.95793497	77.36769096
5 to 9	0.000448237	2.499066174	98653.34145	4.9271441	66.06607316	0.000218295	2.499545219	98872.82515	4.94094443	73.48290743
10 to 14	0.000391351	2.770596391	98432.50536	4.917334136	61.2086957	0.000191242	2.683808495	98764.97175	4.936062014	68.56042598
15 to 19	0.000959443	2.778022654	98240.07408	4.90155441	56.32316142	0.000387597	2.666363348	98670.57598	4.929070573	63.6234428
20 to 24	0.001684207	2.592157604	97769.80597	4.868746175	51.58067326	0.000502256	2.657052447	98479.52861	4.918189097	58.74167396
25 to 29	0.001725454	2.593356764	96949.81842	4.827444947	46.99494427	0.000769588	2.683593052	98232.51309	4.902885567	53.8826711
30 to 34	0.002497869	2.579157417	96116.87891	4.776958762	42.37966331	0.001187844	2.625007046	97855.19969	4.878996343	49.08003388
35 to 39	0.002727597	2.679076387	94923.68482	4.716328557	37.8798728	0.001496381	2.598594002	97275.66543	4.846368841	44.35671825
40 to 44	0.004967994	2.65429825	93637.30403	4.627936642	33.36336297	0.001920006	2.676832241	96550.48885	4.806087196	39.67025045
45 to 49	0.006649212	2.620269508	91338.26852	4.495780396	29.136168	0.003177369	2.682038903	95627.76237	4.746432105	35.02708752
50 to 54	0.009308863	2.668912114	88349.17009	4.323645491	25.03309438	0.004814661	2.632385774	94119.74661	4.652951355	30.54516673
55 to 59	0.015370494	2.623040595	84324.80779	4.067649532	21.10022231	0.006478043	2.655206719	91879.71761	4.525256558	26.22547449
60 to 64	0.02074195	2.623809522	78073.60842	3.720351105	17.57945053	0.010185326	2.648320255	88948.6218	4.343412679	22.00194912
65 to 69	0.033294672	2.574735713	70358.46506	3.25514705	14.21927842	0.014886763	2.675123355	84525.5177	4.084932517	18.01441902
70 to 74	0.043040859	2.587965842	59523.61695	2.696363469	11.33887973	0.025761031	2.6873116	78446.00997	3.701838425	14.20290672
75 to 79	0.078334305	2.540965684	47922.87358	2.009302529	8.457468747	0.047323402	2.618613791	68913.62978	3.096883782	10.79552619
80 to 84	0.119319463	2.432397205	32191.44686	1.232297501	6.349670408	0.078661724	2.535357806	54267.35509	2.273061052	8.002586826
85 to 89	0.18461138	2.291107268	17497.33499	0.583342614	4.64137218	0.137162964	2.421608409	36402.5781	1.344956761	5.687013573
90 to 94	0.278112278	2.087833553	6734.82925	0.186101622	3.400223856	0.225037011	2.218655535	17972.33966	0.552895283	4.038767457
95 plus	0.363437557	2.75157134	1561.616948	0.042983091	2.75157134	0.319775438	3.127470058	5540.87231	0.173402025	3.127470058

Table 15: Seychelles 2100 life table, by age and sex. mx=mortality rate, ax=mean person-years lived in an age interval among those who die in that age interval, lx=number of persons left alive at age x, nLx=person-years lived between age x and x+n, ex=life expectancy at age x.

Age Group	Male					Female				
	mx	ax	lx	nLx	ex	mx	ax	lx	nLx	ex
Early Neonatal	0.067838416	0.009586962	100000	0.019165613	77.62604171	0.061556024	0.009587154	100000	0.019166767	82.1366289
Late Neonatal	0.007322	0.028765104	99869.99967	0.057447352	77.70761293	0.006794605	0.028765249	99882.02427	0.05745514	82.2143085
Post Neonatal	0.001564961	0.461532663	99827.94138	0.921033592	77.68273662	0.001394823	0.46154475	99842.99003	0.921244762	82.18886667
1 to 4	0.000145134	1.999806488	99683.82791	3.986196287	76.87088788	9.9374E-05	1.999867501	99714.51334	3.987788224	81.37080364
5 to 9	0.000146397	2.499695007	99625.99503	4.979478558	72.9142887	6.42104E-05	2.499866228	99674.90148	4.982945326	77.40233759
10 to 14	0.000140248	3.11000941	99553.17597	4.976202381	67.96576546	7.04441E-05	2.916114381	99642.91392	4.981390138	72.42634876
15 to 19	0.000452308	2.826351996	99483.43593	4.969171299	63.01125154	0.000182518	2.742438129	99607.83233	4.978318905	67.45081105
20 to 24	0.00079188	2.591593819	99258.83327	4.953476808	58.14624276	0.000262452	2.672878365	99517.00073	4.972805596	62.50974009
25 to 29	0.000800311	2.526666061	98866.8982	4.933620926	53.36481851	0.000393028	2.82579446	99386.542	4.965026894	57.58807207
30 to 34	0.000949511	2.56625479	98472.58841	4.912221952	48.56679553	0.000831632	2.597452724	99191.51419	4.949548258	52.69548741
35 to 39	0.001041403	2.732799856	98007.32991	4.888764555	43.7827921	0.000630341	2.509876	98780.84714	4.931294936	47.90270048
40 to 44	0.00211677	2.641692813	97499.03199	4.850782354	38.99543272	0.000850075	2.794734998	98470.15769	4.914221389	43.04558769
45 to 49	0.002646621	2.672377466	96473.85205	4.794139729	34.37930118	0.001770465	2.763477991	98052.76546	4.883303197	38.21629044
50 to 54	0.004449548	2.734427781	95209.03209	4.712899441	29.79661201	0.003134616	2.618085887	97188.68133	4.823469136	33.53053891
55 to 59	0.008084838	2.635137706	93118.8386	4.568847655	25.39993933	0.003651932	2.64037802	95678.30506	4.743053235	29.01662386
60 to 64	0.010675457	2.68281132	89439.55604	4.364412432	21.3311838	0.00572626	2.66371841	93949.49663	4.635542794	24.49999808
65 to 69	0.019567266	2.614678304	84813.74546	4.052720246	17.34071948	0.008478417	2.724670617	91301.84083	4.478696401	20.1299396
70 to 74	0.02539417	2.66573762	76952.88683	3.634322792	13.83807605	0.015672746	2.824460067	87518.8603	4.231706576	15.8795593
75 to 79	0.052162178	2.619358639	67833.86751	3.023005028	10.33296311	0.037073435	2.689303903	80926.10479	3.728767428	11.93830774
80 to 84	0.089443076	2.511925076	52380.30114	2.156581985	7.602587049	0.065430417	2.577924116	67215.82179	2.906092538	8.820192288
85 to 89	0.149896408	2.383180752	33803.02601	1.232396771	5.403522834	0.119372421	2.477980926	48452.07376	1.870777358	6.228772541
90 to 94	0.244256414	2.154969264	16268.66749	0.490044103	3.809108775	0.205196045	2.259715402	26572.02399	0.857578556	4.358119798
95 plus	0.336482267	2.985640522	4839.112809	0.149109851	2.985640522	0.301635002	3.326449485	9350.021515	0.316435348	3.326449485

Table 15: Thailand 2017 life table, by age and sex. mx=mortality rate, ax=mean person-years lived in an age interval among those who die in that age interval, lx=number of persons left alive at age x, nLx=person-years lived between age x and x+n, ex=life expectancy at age x.

Age Group	Male					Female				
	mx	ax	lx	nLx	ex	mx	ax	lx	nLx	ex
Early Neonatal	0.204370776	0.009582777	100000	0.019140548	73.86065175	0.164413289	0.009584002	100000	0.019147879	81.31883244
Late Neonatal	0.018458806	0.028762031	99608.83235	0.057278772	74.13148137	0.015899036	0.028762738	99685.18926	0.0573269	81.55640515
Post Neonatal	0.002647853	0.461455737	99503.10844	0.917577898	74.15268537	0.002326427	0.46147857	99594.04909	0.918552741	81.57347368
1 to 4	0.000644119	1.999141175	99260.15744	3.965296179	73.40977082	0.000484613	1.99935385	99380.36154	3.971364264	80.824586
5 to 9	0.000463622	2.49903412	99004.76195	4.944504987	69.59399754	0.000265867	2.49944611	99187.91427	4.956100846	76.97752417
10 to 14	0.000438672	2.918476995	98775.52615	4.934272147	64.74970998	0.000290065	2.628647506	99056.1488	4.949403608	72.07659571
15 to 19	0.001352931	2.716050277	98559.07489	4.912774069	59.88545661	0.000446713	2.612174613	98912.58516	4.940359894	67.1773648
20 to 24	0.001859309	2.633662075	97894.42104	4.873279673	55.27331846	0.000532742	2.593125214	98691.89546	4.928275728	62.32167386
25 to 29	0.002584237	2.61343984	96988.36779	4.81969427	50.76470791	0.000688108	2.634326397	98429.35033	4.913468966	57.48091295
30 to 34	0.003335265	2.573371601	95742.93709	4.748717838	46.39064058	0.000981616	2.652584142	98091.26085	4.893287771	52.66985498
35 to 39	0.003867071	2.555123337	94159.29267	4.663878129	42.1271112	0.001418081	2.621444509	97610.94641	4.864141078	47.91585068
40 to 44	0.004513278	2.562987082	92356.05705	4.567578037	37.89922079	0.001828382	2.60728533	96921.21379	4.824954124	43.2379945
45 to 49	0.005447846	2.582490277	90295.10726	4.456089439	33.70519154	0.002396014	2.621298554	96039.10412	4.774745169	38.61094942
50 to 54	0.006997524	2.597031818	87868.38534	4.320799881	29.56410588	0.003288832	2.615507199	94895.20704	4.707846831	34.04448715
55 to 59	0.009240025	2.617673528	84846.41616	4.150999135	25.52394672	0.004334004	2.667167201	93347.14992	4.620651057	29.56532435
60 to 64	0.01320936	2.628060963	81013.36924	3.927697527	21.60712185	0.007020352	2.687404568	91345.0575	4.494310855	25.15442367
65 to 69	0.01937849	2.621419311	75829.23996	3.624560187	17.90412131	0.011268054	2.665184413	88190.9821	4.296567956	20.9573285
70 to 74	0.028840771	2.606784708	68812.69039	3.218774748	14.46212902	0.017470486	2.657352013	83352.1544	4.003868896	17.01848353
75 to 79	0.043761904	2.584803435	59542.8355	2.69304554	11.30811915	0.028279368	2.652153423	76363.09822	3.580690953	13.33213167
80 to 84	0.069010958	2.520250368	47780.71751	2.040655218	8.457295514	0.048490558	2.593199342	66250.23186	2.966846492	9.962168444
85 to 89	0.125616507	2.447457343	33731.6395	1.277868266	5.934210826	0.095739497	2.556068778	51889.47923	2.103355437	7.002795877
90 to 94	0.220598965	2.202121438	17716.93149	0.548183683	4.093574412	0.178262372	2.312538278	31793.33898	1.075486626	4.817788741
95 plus	0.317652227	3.148511519	5646.106344	0.17798558	3.148511519	0.276810623	3.613575663	12658.31876	0.458078371	3.613575663

Table 15: Thailand 2100 life table, by age and sex. mx=mortality rate, ax=mean person-years lived in an age interval among those who die in that age interval, lx=number of persons left alive at age x, nLx=person-years lived between age x and x+n, ex=life expectancy at age x.

Age Group	Male					Female				
	mx	ax	lx	nLx	ex	mx	ax	lx	nLx	ex
Early Neonatal	0.031670403	0.00958807	100000	0.019172259	81.32886587	0.027669748	0.009588193	100000	0.019172995	86.2839034
Late Neonatal	0.002436582	0.028766451	99939.28468	0.057495285	81.35905407	0.003218647	0.028766235	99946.95183	0.057498402	86.31048073
Post Neonatal	0.000407328	0.4616149	99925.27667	0.922424301	81.3129205	0.000404696	0.461615087	99928.44633	0.922454683	86.26891223
1 to 4	0.00016272	1.99978304	99887.70551	3.994208369	80.42000656	0.000114927	1.999846764	99891.11707	3.994726519	85.37768853
5 to 9	9.59013E-05	2.499800206	99822.72211	4.989939714	76.4710155	6.20775E-05	2.499870672	99845.21368	4.99148607	81.41600096
10 to 14	0.000182649	3.29565919	99774.87106	4.987217414	71.50633301	0.000117455	2.827483625	99814.23139	4.989419059	76.44046155
15 to 19	0.000821456	2.705102452	99683.78633	4.974837027	66.56837843	0.000228183	2.613847782	99755.63504	4.985071901	71.48367662
20 to 24	0.00101643	2.577533386	99275.4654	4.951724814	61.82853882	0.000246463	2.564950582	99641.90177	4.979134847	66.562171
25 to 29	0.001323779	2.59427933	98773.62857	4.923093839	57.12592564	0.000331924	2.691439567	99519.23469	4.972107176	61.64085847
30 to 34	0.001623555	2.627781632	98126.52471	4.887271771	52.4817212	0.000505218	2.707554625	99354.41273	4.961857089	56.73818681
35 to 39	0.001917898	2.568511242	97341.48455	4.844431855	47.88216944	0.000717072	2.602190823	99104.07012	4.946672895	51.87407173
40 to 44	0.002043118	2.576900574	96420.0416	4.79729797	43.31412327	0.000830236	2.610450921	98749.63954	4.927705958	47.05018074
45 to 49	0.002612524	2.631314177	95446.0246	4.74300727	38.728619	0.001159065	2.661896686	98340.82317	4.903747328	42.23406462
50 to 54	0.003643666	2.648761645	94214.35062	4.670825494	34.19896526	0.001729058	2.64797112	97773.08534	4.868878241	37.46241845
55 to 59	0.005287259	2.655129584	92521.3466	4.56949621	29.77468991	0.002418823	2.699824826	96932.45382	4.819682159	32.76247937
60 to 64	0.007568067	2.659834945	90121.80211	4.427980986	25.49574349	0.00393447	2.715291235	95769.63008	4.745854454	28.12537925
65 to 69	0.011702875	2.642846577	86788.40175	4.223315573	21.36937242	0.00663556	2.698708855	93906.56901	4.624691091	23.6257097
70 to 74	0.016640916	2.638299817	81880.43042	3.94020391	17.48965518	0.010561535	2.710473287	90847.99645	4.435273728	19.32593276
75 to 79	0.025800592	2.657963303	75381.54075	3.556156602	13.76463431	0.018450224	2.733712416	86187.99739	4.13711394	15.21683583
80 to 84	0.044508298	2.572909924	66319.34671	2.998778407	10.28061044	0.035556647	2.658619469	78629.96158	3.63403688	11.40759173
85 to 89	0.090875196	2.556663785	53275.93745	2.193560514	7.137805578	0.074888827	2.641232747	65970.7788	2.816563583	8.04849231
90 to 94	0.181055914	2.243776064	34067.12646	1.150192563	4.765407966	0.150717984	2.337649377	45616.96504	1.646599397	5.47871907
95 plus	0.284548149	3.533407463	13978.72416	0.506233727	3.533407463	0.250395578	4.03202714	21773.99792	0.904155006	4.03202714

Table 15: Timor-Leste 2017 life table, by age and sex. mx=mortality rate, ax=mean person-years lived in an age interval among those who die in that age interval, lx=number of persons left alive at age x, nLx=person-years lived between age x and x+n, ex=life expectancy at age x.

Age Group	Male					Female				
	mx	ax	lx	nLx	ex	mx	ax	lx	nLx	ex
Early Neonatal	0.80743189	0.009564293	100000	0.01903037	69.13115258	0.543458463	0.009572384	100000	0.01907849	73.30664595
Late Neonatal	0.032594038	0.028758132	98463.58966	0.056597212	70.19028951	0.02318635	0.028760727	98963.21703	0.056899785	74.05523569
Post Neonatal	0.012645641	0.460745512	98279.15932	0.902124234	70.26442151	0.011961506	0.460794112	98831.30289	0.907477794	74.09650276
1 to 4	0.001821585	1.997571222	97138.72416	3.87143375	70.16080711	0.001757726	1.997656367	97746.02513	3.896132028	73.99086766
5 to 9	0.000590552	2.498769684	96433.83133	4.814581026	66.65928578	0.000461031	2.49903952	97061.38728	4.847480579	70.49882763
10 to 14	0.000501002	2.665334197	96149.55002	4.801860316	61.84907171	0.000373259	2.589376502	96837.92206	4.83754374	65.65568099
15 to 19	0.000991925	2.689204729	95909.00363	4.784484877	56.99753494	0.00062355	2.641167816	96657.37551	4.825771817	60.77348291
20 to 24	0.001413763	2.568572074	95434.41427	4.755376076	52.26745042	0.000800055	2.587321399	96356.48611	4.8085444	55.9549574
25 to 29	0.001475399	2.538094662	94762.14185	4.720962785	47.61971849	0.000966533	2.612309666	95971.80682	4.787544348	51.16872302
30 to 34	0.00170866	2.592621513	94065.70944	4.684023612	42.95316952	0.001333702	2.6494411	95509.12086	4.760535834	46.40371209
35 to 39	0.002270869	2.640536066	93265.51559	4.638433861	38.29908159	0.001946472	2.657077456	94874.28661	4.722188524	41.69611896
40 to 44	0.003307623	2.673846483	92212.44964	4.575434339	33.705687	0.00285175	2.659810581	93955.28987	4.666631951	37.07736711
45 to 49	0.005173268	2.69122136	90699.58506	4.481479729	29.22219946	0.004233006	2.663256579	92624.88192	4.5858967	32.57070934
50 to 54	0.008400951	2.684598111	88382.45274	4.334867681	24.91618645	0.006377556	2.648015404	90684.61009	4.467261317	28.20937355
55 to 59	0.01349781	2.686767466	84743.77174	4.109027467	20.86851931	0.00921566	2.669339209	87837.69049	4.299607764	24.03645565
60 to 64	0.022903015	2.634608495	79204.84619	3.757045977	17.13720424	0.014955738	2.645933759	83879.59347	4.051509796	20.04333429
65 to 69	0.034001793	2.582237517	70616.3961	3.26322884	13.89851492	0.022191598	2.621259257	77828.35483	3.696619502	16.39484231
70 to 74	0.048769819	2.570646266	59550.30885	2.663167874	11.00037482	0.033545934	2.623725077	69640.93518	3.225448618	13.01369929
75 to 79	0.079637159	2.521403202	46612.47489	1.948094091	8.342068904	0.056479728	2.583417686	58843.81571	2.589699189	9.920836135
80 to 84	0.123385512	2.426336552	31179.21287	1.18529652	6.231506456	0.092868603	2.501415401	44258.07006	1.797411918	7.341784831
85 to 89	0.18892299	2.281200983	16656.46829	0.551651164	4.570564681	0.154784155	2.373422162	27627.96925	0.983321769	5.263264756
90 to 94	0.282043565	2.07940607	6306.67175	0.173416914	3.362012747	0.243523132	2.176452092	12467.07607	0.369975494	3.792763073
95 plus	0.366464147	2.729555471	1442.780702	0.039533864	2.729555471	0.336197118	2.975328392	3488.462322	0.104064593	2.975328392

Table 15: Timor-Leste 2100 life table, by age and sex. mx=mortality rate, ax=mean person-years lived in an age interval among those who die in that age interval, lx=number of persons left alive at age x, nLx=person-years lived between age x and x+n, ex=life expectancy at age x.										
Age Group	Male					Female				
	mx	ax	lx	nLx	ex	mx	ax	lx	nLx	ex
Early Neonatal	0.157196248	0.009584223	100000	0.019149206	80.16468944	0.095931048	0.009586101	100000	0.019160452	83.47174913
Late Neonatal	0.003808166	0.028766073	99699.02453	0.0573548	80.38713048	0.00309979	0.028766268	99816.20976	0.057423384	83.60600499
Post Neonatal	0.001100173	0.461565681	99677.18563	0.919840002	80.34714553	0.001236784	0.461555977	99798.41288	0.920900661	83.56332438
1 to 4	0.000319604	1.99957386	99576.01471	3.980499153	79.5044875	0.000322261	1.999570321	99684.54943	3.984817339	82.73453337
5 to 9	0.000122144	2.499745533	99449.01872	4.970935103	75.60180693	0.000117174	2.499755888	99556.4025	4.976365439	78.83719939
10 to 14	0.000155148	2.947467531	99388.39695	4.967829915	70.64573259	0.000133807	2.704762404	99498.2274	4.973380995	73.88153263
15 to 19	0.000449697	2.769309774	99311.45205	4.96059735	65.69750924	0.000243803	2.651305558	99431.83416	4.968745873	68.92879446
20 to 24	0.000734292	2.572786344	99088.98418	4.945706536	60.83707925	0.000304072	2.584321647	99311.04746	4.96191862	64.00893406
25 to 29	0.000730969	2.498492227	98728.14455	4.927550714	56.04760113	0.000367963	2.622820156	99160.61147	4.953727154	59.1016143
30 to 34	0.000795557	2.567507522	98371.66495	4.909151058	51.24021471	0.000535627	2.694841006	98979.0196	4.94276336	54.20452402
35 to 39	0.000959074	2.63416509	97985.39991	4.888164975	46.43097055	0.000767396	2.686301575	98715.38909	4.926952986	49.3412694
40 to 44	0.001305539	2.687582635	97521.03554	4.861337753	41.63806341	0.00113032	2.722418574	98338.99094	4.904186717	44.51871146
45 to 49	0.001983586	2.745727657	96892.47965	4.822940714	36.8885382	0.001810385	2.715587894	97787.24983	4.869127455	39.75330057
50 to 54	0.003407597	2.744258662	95944.64277	4.760590443	32.22221406	0.002884115	2.666748576	96909.61057	4.81313221	35.08606623
55 to 59	0.005833132	2.713452415	94335.72935	4.654858397	27.71881042	0.004162503	2.675421164	95526.86046	4.730573476	30.55188044
60 to 64	0.009512921	2.657766377	91643.84599	4.48302276	23.44513505	0.006434484	2.641190636	93566.99737	4.608704546	26.13187096
65 to 69	0.013871854	2.631651348	87422.03029	4.233366021	19.43940642	0.008988565	2.642530644	90616.59326	4.437225301	21.89001622
70 to 74	0.020166023	2.674009512	81623.97213	3.900841568	15.62582566	0.013361491	2.710725528	86657.15891	4.205197022	17.76266463
75 to 79	0.036605592	2.655809758	73885.34727	3.407787233	11.97373093	0.025444896	2.697078304	81086.17946	3.832359747	13.78794771
80 to 84	0.064697228	2.564040848	61691.2246	2.676066351	8.815669177	0.046267269	2.637924353	71469.69258	3.228136893	10.26981239
85 to 89	0.11937568	2.46798364	44940.11331	1.741441438	6.164455374	0.091920916	2.573328263	56875.53665	2.337847783	7.223413105
90 to 94	0.213327766	2.209836796	24949.38268	0.792465156	4.221175418	0.172902446	2.316079849	36041.14633	1.24348595	4.957935378
95 plus	0.311505365	3.221567302	8597.066563	0.283192791	3.221567302	0.271583453	3.702515369	15195.34	0.575615763	3.702515369

Table 15: Vietnam 2017 life table, by age and sex. mx=mortality rate, ax=mean person-years lived in an age interval among those who die in that age interval, lx=number of persons left alive at age x, nLx=person-years lived between age x and x+n, ex=life expectancy at age x.

Age Group	Male					Female				
	mx	ax	lx	nLx	ex	mx	ax	lx	nLx	ex
Early Neonatal	0.332174989	0.00957886	100000	0.019117127	70.03064957	0.197957982	0.009582974	100000	0.019141725	79.22958384
Late Neonatal	0.025521921	0.028760083	99365.01349	0.057126965	70.45878352	0.02270201	0.028760861	99621.08542	0.057278828	79.51175381
Post Neonatal	0.003716575	0.461379816	99219.23349	0.914509252	70.50470919	0.003285602	0.461410432	99491.06013	0.917196938	79.55811505
1 to 4	0.000803618	1.99892851	98879.40769	3.948827574	69.822117	0.000454422	1.999394104	99189.73706	3.963986217	78.8751706
5 to 9	0.000315848	2.499341983	98562.14379	4.924218535	66.0404332	0.000190447	2.499603236	99009.62912	4.948125327	75.01505831
10 to 14	0.00036581	3.004171238	98406.63889	4.916742501	61.14082414	0.000185848	2.696476819	98915.39898	4.943654692	70.08411687
15 to 19	0.001205989	2.735908357	98226.80202	4.897964735	56.24724111	0.000367372	2.633048845	98823.52776	4.936883861	65.14673644
20 to 24	0.001742637	2.563981857	97636.18775	4.861175461	51.57104273	0.000421766	2.558172656	98642.16638	4.92703226	60.26150066
25 to 29	0.001765779	2.519134711	96789.15548	4.818354971	46.99990825	0.000484963	2.587209389	98434.3718	4.915966559	55.38315833
30 to 34	0.001940297	2.574951732	95938.43878	4.77446378	42.39419561	0.000627242	2.638100698	98195.98396	4.902537569	50.51120329
35 to 39	0.002512317	2.643319239	95012.15174	4.72265874	37.78203302	0.000906109	2.68094881	97888.51007	4.884164665	45.66142181
40 to 44	0.003755196	2.685710078	93825.90707	4.650892074	33.22556915	0.001425602	2.702587489	97446.01726	4.856399968	40.85632129
45 to 49	0.006043285	2.690413342	92080.13044	4.540659294	28.80345138	0.002320545	2.696566727	96753.83196	4.811983355	36.12883338
50 to 54	0.009724989	2.67184349	89338.14052	4.368080168	24.60352161	0.003687917	2.689603709	95637.54503	4.741501975	31.5183079
55 to 59	0.015164679	2.647363017	85095.15416	4.108348237	20.69582995	0.005852352	2.685951957	93889.873	4.631815267	27.05376108
60 to 64	0.023087446	2.620549227	78874.21964	3.738622805	17.11880814	0.009337373	2.67408753	91181.65421	4.462271876	22.77603258
65 to 69	0.034559875	2.592375912	70256.56527	3.24338738	13.89735783	0.014686899	2.667129842	87020.98032	4.20711735	18.73564763
70 to 74	0.051858219	2.554402063	59066.1034	2.621400677	11.04008883	0.023760504	2.658938324	80853.87525	3.830077267	14.96044479
75 to 79	0.077439964	2.500887792	45494.53093	1.906489018	8.573045457	0.039907401	2.634200517	71775.14014	3.279830156	11.51689313
80 to 84	0.115268038	2.426834179	30759.35618	1.187024775	6.484853198	0.069712761	2.570535559	58719.1313	2.511639287	8.494007655
85 to 89	0.179975838	2.302390735	17119.7003	0.576882057	4.726079637	0.125575266	2.456198671	41253.17873	1.564341736	6.005879682
90 to 94	0.273792914	2.097006047	6769.946995	0.188839938	3.445656883	0.212470921	2.24622325	21660.26781	0.683908523	4.225007981
95 plus	0.360071251	2.777654514	1612.487397	0.044863513	2.777654514	0.308419182	3.243104861	7163.227638	0.232728432	3.243104861

Table 15: Vietnam 2100 life table, by age and sex. mx=mortality rate, ax=mean person-years lived in an age interval among those who die in that age interval, lx=number of persons left alive at age x, nLx=person-years lived between age x and x+n, ex=life expectancy at age x.

Age Group	Male					Female				
	mx	ax	lx	nLx	ex	mx	ax	lx	nLx	ex
Early Neonatal	0.056508615	0.009587309	100000	0.019167694	78.58076091	0.034665483	0.009587979	100000	0.019171709	84.43287393
Late Neonatal	0.004326321	0.02876593	99891.69175	0.05746478	78.64667021	0.005254649	0.028765674	99933.54315	0.057487321	84.46978443
Post Neonatal	0.000471249	0.461610359	99866.83203	0.921857592	78.60867418	0.000545642	0.461605074	99903.33728	0.922162907	84.43775906
1 to 4	0.000110823	1.999852236	99823.39167	3.992050928	77.71934745	6.38694E-05	1.999914841	99853.02441	3.993611096	83.55674607
5 to 9	6.12005E-05	2.499872499	99779.16013	4.988195081	73.75276076	4.37582E-05	2.499908837	99827.5348	4.990831218	79.57747117
10 to 14	0.0001094	3.532728003	99748.64581	4.986611661	68.77444897	6.01013E-05	2.88714967	99805.71601	4.989645831	74.59427814
15 to 19	0.000584806	2.797249754	99694.11539	4.978266791	63.81002725	0.000148834	2.661581682	99775.75418	4.987049592	69.6157808
20 to 24	0.000919702	2.60028764	99403.13832	4.959200025	58.98810444	0.000172246	2.582040275	99701.57882	4.98300252	64.66553388
25 to 29	0.001010928	2.546603512	98947.59267	4.935142515	54.24703024	0.000214521	2.627254751	99615.80871	4.978256107	59.71894655
30 to 34	0.001143018	2.551212551	98449.37562	4.908746398	49.50830678	0.000302067	2.653473456	99509.07403	4.971925934	54.78010183
35 to 39	0.001304127	2.608481077	97889.04205	4.879245963	44.77670536	0.000432802	2.705915177	99358.96539	4.963018473	49.85870157
40 to 44	0.001834148	2.666811127	97253.56629	4.841969659	40.0516589	0.000728311	2.774394383	99144.25528	4.949190508	44.96059098
45 to 49	0.002803116	2.719349759	96366.47079	4.78776904	35.39466462	0.001397846	2.750478633	98783.92764	4.923715449	40.11409539
50 to 54	0.004919479	2.698142002	95026.00363	4.698186561	30.85347116	0.002431186	2.718079675	98095.96889	4.877745971	35.37530515
55 to 59	0.007766708	2.665727234	92719.46183	4.553552971	26.54938811	0.00401015	2.679998111	96910.9841	4.800828484	30.77316337
60 to 64	0.01166212	2.654161591	89195.03087	4.341397565	22.48728568	0.005967317	2.646445097	94988.39535	4.6837594	26.33965658
65 to 69	0.017889288	2.632528175	84156.9752	4.037828967	18.66697049	0.00863017	2.665308396	92198.2916	4.518940459	22.05184971
70 to 74	0.026457202	2.615464659	76985.78568	3.622841047	15.15255781	0.013529412	2.694545394	88311.43445	4.282448984	17.90037589
75 to 79	0.040402601	2.586355006	67502.95642	3.078731658	11.90958899	0.02359586	2.70942403	82546.62308	3.91680268	13.95565128
80 to 84	0.061272203	2.545289801	55232.9698	2.407047986	8.982632293	0.0447302	2.674708562	73380.94393	3.326770887	10.35478915
85 to 89	0.115017902	2.48023419	40790.05929	1.590242601	6.272299375	0.089981297	2.579132996	58681.65007	2.416387632	7.273634431
90 to 94	0.208930879	2.218104892	22948.90223	0.731743333	4.279038159	0.170831481	2.321192995	37310.54264	1.28738731	4.986331375
95 plus	0.307952318	3.254653169	7981.288513	0.263500088	3.254653169	0.269712448	3.720049229	15710.96925	0.59273346	3.720049229

Table 15: Sub-Saharan Africa 2017 life table, by age and sex. mx=mortality rate, ax=mean person-years lived in an age interval among those who die in that age interval, lx=number of persons left alive at age x, nLx=person-years lived between age x and x+n, ex=life expectancy at age x.

Age Group	Male					Female				
	mx	ax	lx	nLx	ex	mx	ax	lx	nLx	ex
Early Neonatal	1.29203283	0.009549441	100000	0.018942431	61.74108647	0.932430687	0.009560462	100000	0.019007628	66.34338469
Late Neonatal	0.113144722	0.028735912	97552.61794	0.055943884	63.27043943	0.087086558	0.028743101	98227.69048	0.05637322	67.52096299
Post Neonatal	0.027363927	0.459699968	96919.66737	0.883638354	63.62588544	0.024796291	0.459882364	97736.76906	0.892140472	67.80241586
1 to 4	0.00769	1.989746829	94501.80003	3.722530662	64.31866805	0.007122661	1.990503247	95524.65937	3.767071644	68.43856762
5 to 9	0.001195581	2.497509207	91639.42459	4.568303853	62.26557257	0.000950558	2.498019671	92841.67678	4.631070214	66.35886713
10 to 14	0.00093159	2.583709437	91093.27384	4.544434535	57.62392405	0.000714913	2.541721647	92401.48056	4.611969109	61.6630939
15 to 19	0.001585761	2.678960039	90669.93251	4.516872364	52.88090219	0.001102732	2.66327544	92071.77197	4.591757403	56.87478907
20 to 24	0.002322254	2.623119142	89953.68096	4.472995235	48.28059623	0.001593427	2.666539398	91565.43407	4.561312879	52.17452092
25 to 29	0.003013652	2.617230216	88914.96651	4.414053238	43.8139126	0.002408173	2.651830306	90838.64453	4.516393507	47.57055542
30 to 34	0.004121871	2.615626476	87584.77184	4.33661927	39.43951846	0.003412909	2.61951431	89751.06776	4.451388468	43.11477557
35 to 39	0.005483686	2.608571737	85797.36345	4.234342258	35.20659795	0.004486368	2.593581805	88231.94236	4.364479834	38.81191114
40 to 44	0.007306342	2.604347204	83475.54264	4.101983013	31.11321609	0.005634877	2.582135757	86274.02233	4.255723207	34.6337483
45 to 49	0.009721302	2.606157196	80478.72343	3.93243169	27.17470727	0.007039971	2.591846902	83876.15292	4.123900633	30.54989277
50 to 54	0.013313475	2.602412776	76656.24932	3.714266023	23.39967478	0.009288836	2.592730959	80973.19662	3.960120776	26.55199563
55 to 59	0.018199041	2.609993005	71711.89171	3.436158128	19.83344193	0.012087535	2.619460957	77295.21797	3.756683433	22.69181404
60 to 64	0.026818707	2.596078212	65459.41907	3.074777196	16.47845286	0.018006323	2.627255239	72755.26408	3.488745377	18.94410282
65 to 69	0.038438119	2.568265008	57214.88935	2.616260764	13.47881569	0.026816849	2.612723623	66475.02226	3.123829699	15.48546426
70 to 74	0.055677798	2.530195984	47161.06354	2.073080035	10.80479562	0.040904799	2.583236168	58100.7236	2.643791932	12.34081754
75 to 79	0.078935085	2.485479501	35622.80176	1.486292222	8.485274912	0.061862913	2.544401076	47291.17653	2.052901359	9.571356478
80 to 84	0.116811587	2.42246396	23896.94259	0.918501472	6.430210242	0.097325912	2.48116549	34599.55201	1.389603166	7.149767343
85 to 89	0.181668392	2.298161978	13175.34664	0.441986586	4.693648802	0.160075194	2.359541166	21086.09533	0.741270822	5.143689252
90 to 94	0.275386419	2.093640287	5151.28434	0.143101152	3.428303053	0.248997708	2.163814828	9229.884442	0.270572517	3.723450317
95 plus	0.361320154	2.767705797	1212.559847	0.033572104	2.767705797	0.34102105	2.932533062	2497.460834	0.073277393	2.932533062

Table 15: Sub-Saharan Africa 2100 life table, by age and sex. mx=mortality rate, ax=mean person-years lived in an age interval among those who die in that age interval, lx=number of persons left alive at age x, nLx=person-years lived between age x and x+n, ex=life expectancy at age x.

Age Group	Male					Female				
	mx	ax	lx	nLx	ex	mx	ax	lx	nLx	ex
Early Neonatal	0.252839186	0.009581292	100000	0.019131665	76.61436443	0.203902881	0.009582791	100000	0.019140635	81.52209373
Late Neonatal	0.020294672	0.028761525	99516.34593	0.057222568	76.96730788	0.02079281	0.028761388	99609.74584	0.057275453	81.82214284
Post Neonatal	0.003772303	0.461375858	99400.22383	0.916153721	76.99961144	0.003677176	0.461382615	99490.66272	0.917027513	81.8624757
1 to 4	0.000809379	1.998920828	99054.64431	3.955779625	76.34322184	0.000764443	1.998980743	99153.47347	3.960081931	81.2158836
5 to 9	0.000236232	2.49950785	98734.51385	4.933811615	72.58412673	0.000202692	2.499577725	98850.78112	4.940035694	77.45833246
10 to 14	0.000233574	2.742297343	98617.9742	4.928300985	67.66689002	0.000185941	2.594322464	98750.6643	4.935325034	72.53429395
15 to 19	0.000509931	2.732810978	98502.87638	4.919461188	62.74269103	0.000286506	2.657387678	98658.91261	4.929638166	67.59931203
20 to 24	0.000810323	2.624158296	98252.07095	4.903161318	57.8957071	0.000403835	2.670740226	98517.70253	4.921259749	62.69235478
25 to 29	0.000991524	2.598749556	97854.97104	4.881126831	53.11974484	0.000622131	2.641119108	98319.01273	4.908749938	57.81358743
30 to 34	0.001285228	2.586963526	97371.23681	4.853510091	48.37047855	0.000830516	2.603692936	98013.70638	4.890940407	52.98532966
35 to 39	0.001537826	2.602059261	96747.75206	4.819625504	43.66519585	0.001027752	2.604471799	97607.67584	4.868391459	48.19479834
40 to 44	0.00206592	2.655507537	96007.04034	4.777220428	38.98140744	0.001345847	2.652836693	97107.56088	4.84008264	43.42929496
45 to 49	0.003123705	2.689020398	95020.82234	4.717015104	34.35731248	0.002024795	2.679272514	96456.47055	4.800270334	38.70391734
50 to 54	0.005024259	2.692379558	93548.65715	4.623861975	29.85380238	0.003130771	2.663361939	95485.06563	4.739598527	34.06910195
55 to 59	0.008044859	2.679650841	91228.9431	4.477951084	25.54164682	0.004579582	2.656477538	94002.64907	4.650229732	29.56221389
60 to 64	0.012675683	2.656102566	87634.2329	4.25555985	21.4763059	0.006742137	2.656107298	91876.52748	4.522478316	25.18233038
65 to 69	0.019349949	2.633357373	82256.11045	3.933252314	17.70317072	0.010151117	2.665187874	88833.9583	4.339053014	20.94997311
70 to 74	0.0291964	2.620399415	74676.79245	3.492357619	14.2305728	0.015907683	2.69887252	84444.07453	4.073597656	16.89674591
75 to 79	0.046519509	2.586367503	64532.39394	2.903044435	11.05409588	0.028850103	2.685353209	77992.45251	3.656807763	13.06713494
80 to 84	0.072860856	2.52076096	51131.65575	2.168911819	8.27589965	0.052628037	2.619512327	67514.01816	3.003122773	9.675402973
85 to 89	0.130364297	2.434283455	35494.52483	1.334178895	5.820674739	0.101685718	2.536068747	51872.3182	2.079616077	6.803579357
90 to 94	0.225338756	2.192963	18314.01759	0.563404552	4.032776448	0.185085874	2.299209058	31011.62758	1.038755391	4.699532078
95 plus	0.321467142	3.113751827	5749.982723	0.180321246	3.113751827	0.283121136	3.539677853	12042.384	0.430615526	3.539677853

Table 15: Central Sub-Saharan Africa 2017 life table, by age and sex. mx=mortality rate, ax=mean person-years lived in an age interval among those who die in that age interval, lx=number of persons left alive at age x, nLx=person-years lived between age x and x+n, ex=life expectancy at age x.

Age Group	Male					Female				
	mx	ax	lx	nLx	ex	mx	ax	lx	nLx	ex
Early Neonatal	1.321274033	0.009548545	100000	0.018937179	59.9944091	0.964969478	0.009559465	100000	0.019001737	64.11808218
Late Neonatal	0.101132094	0.028739226	97498.4866	0.055932227	61.51344339	0.077290924	0.028745803	98166.74148	0.056354162	65.29562893
Post Neonatal	0.03049566	0.459477503	96933.14765	0.882490646	61.81453187	0.029118964	0.459575298	97731.39075	0.890320761	65.5288526
1 to 4	0.008418876	1.988775048	94242.19785	3.706950428	62.64310188	0.007563927	1.989914921	95139.06302	3.748588305	66.37850825
5 to 9	0.001158193	2.4975871	91122.93504	4.542982541	60.72019261	0.001009309	2.497897274	92304.71798	4.603612174	64.35623391
10 to 14	0.000957685	2.638931381	90596.87577	4.519625833	56.05813268	0.00073431	2.546627695	91840.16052	4.583751376	59.66908479
15 to 19	0.001815129	2.694229631	90164.09399	4.489418129	51.31440259	0.001183277	2.657755272	91503.6247	4.562538408	54.8791673
20 to 24	0.00268821	2.590269671	89349.27194	4.438713686	46.75744285	0.001645826	2.642064018	90963.82962	4.530613603	50.18902797
25 to 29	0.003044742	2.571407706	88156.1796	4.375460439	42.35461054	0.002337927	2.646174107	90218.31001	4.486231898	45.58184036
30 to 34	0.003835461	2.604765486	86824.20693	4.301700708	37.96436626	0.003349041	2.637285006	89169.66076	4.423483156	41.08659275
35 to 39	0.005137734	2.627572179	85174.74244	4.207471525	33.6485073	0.004666045	2.613693072	87688.47912	4.336152843	36.73573527
40 to 44	0.00728974	2.635680672	83013.82252	4.080398998	29.45526432	0.006126638	2.598950283	85665.59362	4.221206075	32.54097155
45 to 49	0.010498409	2.635931401	80040.80549	3.9051818	25.4503855	0.007989832	2.609656964	83079.92023	4.076184175	28.47149751
50 to 54	0.015423578	2.619324168	75943.90269	3.66282055	21.67994129	0.011076644	2.60636134	79824.28505	3.888196716	24.52440247
55 to 59	0.021951341	2.608464052	70300.11976	3.339871575	18.20896654	0.0149658	2.622629633	75520.67094	3.646431733	20.77102677
60 to 64	0.032407789	2.583278637	62977.6225	2.920504859	15.0221456	0.022601989	2.618907851	70070.08399	3.324858514	17.18065958
65 to 69	0.046224981	2.551927348	53528.39087	2.40484899	12.21801821	0.033671978	2.597995725	62568.29712	2.894850934	13.92497892
70 to 74	0.067342753	2.506791684	42435.72189	1.817434822	9.746521916	0.051520481	2.559358872	52846.19652	2.348030581	11.00865395
75 to 79	0.094638956	2.447188907	30228.32805	1.218060538	7.674749269	0.077101838	2.507649592	40789.88514	1.711919129	8.508991452
80 to 84	0.134958171	2.373037262	18735.5439	0.692267888	5.889526414	0.118948862	2.432690134	27646.08572	1.06029196	6.369796843
85 to 89	0.200569779	2.254236429	9425.555364	0.304289954	4.376192158	0.18488725	2.30061807	15100.24251	0.504592635	4.656947922
90 to 94	0.292698775	2.056635978	3341.475074	0.089869122	3.257180231	0.273978775	2.105890129	5818.252754	0.162626079	3.440911174
95 plus	0.374682047	2.669179863	717.2904718	0.019176665	2.669179863	0.362612519	2.758555592	1382.002482	0.03824523	2.758555592

Table 15: Central Sub-Saharan Africa 2100 life table, by age and sex. mx=mortality rate, ax=mean person-years lived in an age interval among those who die in that age interval, lx=number of persons left alive at age x, nLx=person-years lived between age x and x+n, ex=life expectancy at age x.

Age Group	Male					Female				
	mx	ax	lx	nLx	ex	mx	ax	lx	nLx	ex
Early Neonatal	0.33816732	0.009578676	100000	0.019116046	74.38972773	0.223084893	0.009582204	100000	0.019137122	80.09871137
Late Neonatal	0.019792872	0.028761663	99353.85249	0.057129973	74.85211254	0.017010013	0.028762431	99573.18752	0.05726067	80.4212198
Post Neonatal	0.003648852	0.461384627	99240.84043	0.914737405	74.87937984	0.00395907	0.46136259	99475.82715	0.916772001	80.44196779
1 to 4	0.000941354	1.998744862	98907.22291	3.948856079	74.20622959	0.000937993	1.998749343	99112.99846	3.957097014	79.81044121
5 to 9	0.000181741	2.499621374	98535.83724	4.92455613	70.4771708	0.000186659	2.499611128	98742.10577	4.934804132	76.1014154
10 to 14	0.00020888	2.976574063	98446.42294	4.920235676	65.53863465	0.000177206	2.667375049	98650.07541	4.930461546	71.16980146
15 to 19	0.000653431	2.792853495	98343.7427	4.9101145	60.60367868	0.000323664	2.67255788	98562.78449	4.924428681	66.23024621
20 to 24	0.001135897	2.604782524	98023.19149	4.887867373	55.79188766	0.000442448	2.646390997	98403.53091	4.915059988	61.33275229
25 to 29	0.001223582	2.558059756	97468.76135	4.858922613	51.09320228	0.000634142	2.639664747	98186.25792	4.901977829	56.46210809
30 to 34	0.001470898	2.572047186	96875.30529	4.826544212	46.38937681	0.000867864	2.622909621	97875.676	4.88369808	51.63226919
35 to 39	0.001735393	2.621648683	96166.82049	4.788609969	41.71089318	0.001134182	2.624246186	97452.25196	4.859527495	46.84441677
40 to 44	0.002512269	2.675607669	95337.65226	4.739240493	37.04924716	0.001553584	2.680276043	96901.72703	4.827695519	42.09438968
45 to 49	0.003895221	2.704170643	94150.05528	4.665843555	32.48050144	0.002492987	2.699220669	96152.85328	4.780226111	37.39936334
50 to 54	0.006479054	2.694891582	92337.94118	4.549078298	28.06160429	0.003968751	2.666146192	94963.44302	4.704627749	32.8306553
55 to 59	0.010349749	2.669685652	89401.39524	4.365100047	23.89049274	0.005748444	2.651384175	93101.59154	4.593168559	28.42897516
60 to 64	0.016039263	2.638680469	84903.73494	4.09096652	20.01012016	0.00840335	2.652071545	90471.27633	4.436378819	24.17289507
65 to 69	0.023759601	2.619483562	78380.72879	3.710544137	16.45148463	0.012632421	2.649769484	86762.93078	4.213776902	20.08572375
70 to 74	0.035967934	2.596581295	69632.70373	3.20692323	13.18770269	0.019151148	2.674732569	81481.26772	3.901846791	16.20830308
75 to 79	0.055076327	2.557491137	58209.702	2.569348545	10.26730056	0.033402907	2.656296805	74090.391	3.438987067	12.55137851
80 to 84	0.084786825	2.493757672	44247.39504	1.830899944	7.708815315	0.057901906	2.602139034	62782.17299	2.763937285	9.331588383
85 to 89	0.144887803	2.394028844	29022.25597	1.060104333	5.46980429	0.108921166	2.51257142	47148.27203	1.86634736	6.573423607
90 to 94	0.239892924	2.165532312	13996.57344	0.420055398	3.844204908	0.19326044	2.28283845	27394.79821	0.907220814	4.563225241
95 plus	0.333189118	3.005887519	4103.866395	0.124933256	3.005887519	0.290630334	3.454613896	10333.05491	0.364273833	3.454613896

Table 15: Angola 2017 life table, by age and sex. mx=mortality rate, ax=mean person-years lived in an age interval among those who die in that age interval, lx=number of persons left alive at age x, nLx=person-years lived between age x and x+n, ex=life expectancy at age x.

Age Group	Male					Female				
	mx	ax	lx	nLx	ex	mx	ax	lx	nLx	ex
Early Neonatal	1.217807917	0.009551716	100000	0.01895589	61.77730821	0.830590535	0.009563584	100000	0.019026154	66.7787019
Late Neonatal	0.075142216	0.028746395	97691.97962	0.056085072	63.21631458	0.044277882	0.028754909	98419.85167	0.05655307	67.8309759
Post Neonatal	0.024769623	0.459884259	97270.72277	0.887902823	63.43235067	0.021839233	0.460092424	98169.49786	0.897312428	67.94630196
1 to 4	0.00565957	1.992453974	95072.81831	3.760227903	63.96506218	0.005556316	1.992591641	96210.47328	3.805988301	68.39712947
5 to 9	0.001025579	2.497863378	92946.70914	4.635442804	61.38380815	0.000839261	2.498251539	94096.84498	4.694986547	65.88933852
10 to 14	0.000825365	2.62385815	92471.41252	4.614522904	56.68610825	0.000604435	2.5394137	93702.89369	4.678188521	61.15568361
15 to 19	0.001530354	2.705819945	92090.59899	4.588425035	51.90936189	0.000960544	2.68459159	93420.17619	4.660647754	56.33303556
20 to 24	0.002369092	2.614494787	91388.44636	4.543747704	47.28666955	0.001469473	2.705668826	92972.57363	4.63301933	51.59119206
25 to 29	0.002885961	2.596446047	90312.16011	4.48450999	42.81787335	0.002447852	2.679964161	92291.93209	4.588538691	46.95134492
30 to 34	0.003798429	2.610542001	89018.38699	4.41089974	38.40126839	0.003645103	2.628603514	91169.11952	4.519380256	42.49593652
35 to 39	0.00505218	2.623238816	87343.87053	4.315410831	34.08621472	0.004806435	2.589265517	89522.52526	4.424869587	38.22852139
40 to 44	0.007078937	2.628932381	85165.31268	4.18803947	29.88970554	0.005923766	2.575870683	87396.92702	4.308019053	34.09448585
45 to 49	0.009997092	2.631815472	82203.79619	4.015252316	25.87005684	0.00733435	2.589069008	84846.35835	4.168673351	30.03973544
50 to 54	0.01455478	2.620989033	78195.5993	3.779142466	22.05942836	0.009684258	2.59471873	81791.03922	3.996589449	26.06192729
55 to 59	0.02077644	2.613867422	72705.71697	3.463913934	18.52578288	0.012756421	2.619998788	77926.03288	3.78171732	22.22156665
60 to 64	0.030911835	2.590195145	65524.93155	3.049679089	15.26905738	0.019002102	2.623617608	73112.64318	3.498158118	18.50799291
65 to 69	0.04453407	2.558764424	56124.14307	2.531847349	12.3937682	0.028152589	2.611935906	66487.43618	3.115858985	15.08725273
70 to 74	0.065266206	2.514711501	44886.29024	1.932117569	9.859507771	0.043411359	2.582131184	57759.10188	2.615166744	11.97103707
75 to 79	0.093167739	2.455921199	32323.97402	1.307626547	7.720831565	0.066213586	2.536650491	46480.03874	2.00034341	9.253074697
80 to 84	0.134784033	2.377838347	20192.85927	0.746965166	5.89518974	0.103757694	2.467811959	33341.35462	1.323128715	6.911398845
85 to 89	0.200467211	2.254498205	10171.80024	0.328592078	4.378215698	0.167532416	2.341556011	19754.17476	0.685709143	4.994287763
90 to 94	0.292601695	2.056842503	3612.199081	0.097213447	3.258259314	0.256532947	2.146316564	8382.595434	0.243022558	3.636794271
95 plus	0.374605687	2.669797481	776.9135018	0.020788084	2.669797481	0.347553236	2.879160396	2202.137205	0.063804415	2.879160396

Table 15: Angola 2100 life table, by age and sex. mx=mortality rate, ax=mean person-years lived in an age interval among those who die in that age interval, lx=number of persons left alive at age x, nLx=person-years lived between age x and x+n, ex=life expectancy at age x.

Age Group	Male					Female				
	mx	ax	lx	nLx	ex	mx	ax	lx	nLx	ex
Early Neonatal	0.266267789	0.00958088	100000	0.019129208	75.37114286	0.148998226	0.009584474	100000	0.019150711	81.49859399
Late Neonatal	0.009494239	0.028764504	99490.79044	0.057225649	75.73708951	0.005430641	0.028765625	99714.71567	0.05736115	81.71204092
Post Neonatal	0.003007987	0.461430153	99436.47005	0.916811288	75.72084829	0.002964414	0.461433249	99683.57115	0.919108007	81.67995507
1 to 4	0.000403832	1.999461557	99160.77001	3.963231697	75.00641309	0.000464361	1.999380852	99411.17534	3.972758653	80.97867092
5 to 9	0.00013439	2.499720021	99000.87185	4.94838201	71.12372343	0.000125184	2.499739199	99226.82839	4.959790432	77.1248297
10 to 14	0.000161397	2.946310642	98934.41831	4.945077861	66.16958255	0.000132063	2.708189143	99164.79818	4.956728662	72.17135426
15 to 19	0.000476145	2.793210753	98854.66465	4.937560145	61.22035561	0.000245252	2.716785806	99099.40597	4.952195342	67.21706642
20 to 24	0.000844638	2.629713346	98619.83343	4.921132832	56.35855435	0.000383564	2.706715269	98978.06757	4.944574611	62.29590257
25 to 29	0.000979257	2.586847483	98205.18706	4.898677067	51.5841628	0.000644016	2.661752719	98788.59371	4.932007121	57.40979778
30 to 34	0.001225158	2.590214956	97726.84247	4.87194734	46.82306471	0.000883622	2.64414129	98471.3773	4.913204456	52.58557159
35 to 39	0.001475849	2.638242238	97131.55847	4.839714278	42.09312689	0.001109477	2.621538085	98038.13729	4.888942593	47.80575476
40 to 44	0.002190312	2.688927682	96419.30185	4.796680244	37.38324005	0.001453451	2.68555719	97496.95683	4.858404395	43.05594701
45 to 49	0.003453663	2.722259129	95371.48842	4.731375846	32.76225666	0.002295168	2.690939419	96792.40157	4.814053358	38.34875282
50 to 54	0.005955114	2.712309663	93742.1979	4.624170478	28.28086714	0.003524344	2.656449455	95689.41219	4.745271115	33.75747797
55 to 59	0.009813622	2.683394677	90998.21445	4.449010194	24.04652582	0.004982259	2.647700039	94021.01018	4.646624282	29.3058416
60 to 64	0.015567491	2.648281219	86652.0428	4.180286473	20.11231956	0.007215558	2.652411582	91712.95407	4.509477268	24.97259483
65 to 69	0.023422748	2.624152526	80186.25309	3.799341771	16.51442558	0.010799645	2.66308504	88472.46757	4.315125392	20.78455775
70 to 74	0.035545706	2.59867143	71362.12525	3.290052143	13.22914729	0.016858277	2.697573497	83840.38222	4.036291447	16.77979942
75 to 79	0.054460203	2.562109433	59792.82572	2.643558766	10.2867719	0.030453159	2.668256396	77093.90479	3.601465835	13.00665555
80 to 84	0.085022566	2.497206093	45603.08117	1.886667004	7.699779927	0.052709213	2.619719929	66253.36917	2.948598371	9.695861553
85 to 89	0.145189653	2.393235537	29876.45684	1.090795768	5.463593002	0.101650635	2.536982678	50970.57855	2.046984268	6.820241245
90 to 94	0.240181535	2.164905889	14393.59283	0.431871661	3.840920224	0.184881848	2.298564475	30606.59696	1.028253418	4.710747803
95 plus	0.333417622	3.004009775	4218.485335	0.128443143	3.004009775	0.282884047	3.54690039	11993.18534	0.432223102	3.54690039

Table 15: Central African Republic 2017 life table, by age and sex. mx=mortality rate, ax=mean person-years lived in an age interval among those who die in that age interval, lx=number of persons left alive at age x, nLx=person-years lived between age x and x+n, ex=life expectancy at age x.

Age Group	Male					Female				
	mx	ax	lx	nLx	ex	mx	ax	lx	nLx	ex
Early Neonatal	2.337853169	0.009517389	100000	0.018754662	46.87532423	1.937064021	0.009529672	100000	0.01882633	52.63494636
Late Neonatal	0.287535967	0.028687807	95617.88253	0.054561028	48.99690532	0.184494723	0.028716231	96354.83026	0.055144113	54.60142186
Post Neonatal	0.069449401	0.456710627	94051.05878	0.841158195	49.75378874	0.064494769	0.457062523	95338.44691	0.854590757	55.12486039
1 to 4	0.016315003	1.978248287	88221.83665	3.416507199	52.08833741	0.015291915	1.979612117	89836.32175	3.486015188	57.55024277
5 to 9	0.002366685	2.495069417	82664.80452	4.108905585	51.46546159	0.001870571	2.496102982	84519.4106	4.206288609	57.05422335
10 to 14	0.002010558	2.697198886	81693.34429	4.065857079	47.04727081	0.00141013	2.576338036	83733.36342	4.17242034	52.56626444
15 to 19	0.004360801	2.704195259	80876.44234	4.003752032	42.49462073	0.002428728	2.646025615	83145.30234	4.133644011	47.91938324
20 to 24	0.006493041	2.581536646	79131.02396	3.895415234	38.37014395	0.003180503	2.632530167	82141.48092	4.076405953	43.47084187
25 to 29	0.007284985	2.551626565	76602.7422	3.76307795	34.54803384	0.004586956	2.644184066	80845.69363	3.999111525	39.12313725
30 to 34	0.008901344	2.587076842	73863.20523	3.61559314	30.73060937	0.006599351	2.642360133	79013.10941	3.890156857	34.96667812
35 to 39	0.011899879	2.6064153	70648.87649	3.434780609	27.00683641	0.009534803	2.609873393	76449.42631	3.737400658	31.04807645
40 to 44	0.016563012	2.590698758	66569.61294	3.201074619	23.49794701	0.012513687	2.567382224	72891.97921	3.53711729	27.43231349
45 to 49	0.021979751	2.572215872	61282.46516	2.909439074	20.29760203	0.015166695	2.555625893	68472.84655	3.301543599	24.02992246
50 to 54	0.029348009	2.548557535	54912.32179	2.562195163	17.35111695	0.019076635	2.554801054	63477.00949	3.03293802	20.7110457
55 to 59	0.037498236	2.534498824	47431.86181	2.172011873	14.68653975	0.023979698	2.57368753	57715.22199	2.72799035	17.51565085
60 to 64	0.050699088	2.520062616	39338.24327	1.748561138	12.19255076	0.034182743	2.580571041	51215.9128	2.366607592	14.40700418
65 to 69	0.069269073	2.483035614	30533.34546	1.301590192	9.99259149	0.050282828	2.551356988	43192.37497	1.925192489	11.60367956
70 to 74	0.093654682	2.430193683	21589.44544	0.871544444	8.122243715	0.073943311	2.49852135	33620.87496	1.421980485	9.188857567
75 to 79	0.124361722	2.36270228	13494.74625	0.509363585	6.562826177	0.105536663	2.429534213	23263.94147	0.919049452	7.192516474
80 to 84	0.160748119	2.278459656	7219.81326	0.251873676	5.25144141	0.148782452	2.340657276	13759.60579	0.496403097	5.538727897
85 to 89	0.225166098	2.20135759	3205.307219	0.09859561	4.015310937	0.215845733	2.237160685	6541.502282	0.20667367	4.159610979
90 to 94	0.314708628	2.009771818	999.6611778	0.025821026	3.060277074	0.304253295	2.037372722	2172.902475	0.057729426	3.148989003
95 plus	0.391421993	2.555084248	190.8439954	0.00489112	2.555084248	0.388070503	2.579020528	448.091749	0.011716543	2.579020528

Table 15: Central African Republic 2100 life table, by age and sex. mx=mortality rate, ax=mean person-years lived in an age interval among those who die in that age interval, lx=number of persons left alive at age x, nLx=person-years lived between age x and x+n, ex=life expectancy at age x.

Age Group	Male					Female				
	mx	ax	lx	nLx	ex	mx	ax	lx	nLx	ex
Early Neonatal	0.642073736	0.009569362	100000	0.019060581	66.56712305	0.59592341	0.009570776	100000	0.019068986	72.23853778
Late Neonatal	0.06614048	0.028748879	98777.62024	0.056723174	67.35952209	0.05452298	0.028752083	98864.84115	0.056792165	73.03449638
Post Neonatal	0.011482071	0.460828171	98403.22035	0.903757919	67.55522339	0.012256374	0.460773166	98555.73137	0.904835068	73.20291202
1 to 4	0.002937916	1.996082792	97368.35165	3.872037442	67.33822988	0.003081205	1.995891741	97449.44366	3.874142123	73.09559183
5 to 9	0.000614564	2.498719659	96236.08236	4.804442589	64.09959043	0.000564632	2.498823684	96260.41706	4.806259125	69.96424706
10 to 14	0.00063481	2.951172099	95941.78743	4.79085511	59.28703093	0.000490495	2.641687471	95990.0938	4.793966333	65.15258047
15 to 19	0.00198005	2.784129962	95638.62471	4.761088232	54.46413513	0.000885131	2.700190622	95756.01074	4.778068262	60.30399192
20 to 24	0.003369646	2.610193144	94698.82273	4.697197947	49.97329024	0.001296308	2.693615757	95335.10209	4.752605431	55.55601673
25 to 29	0.003785955	2.55621166	93122.67805	4.613606663	45.77085132	0.002113007	2.658309949	94721.60703	4.71291261	50.89445709
30 to 34	0.004467234	2.556446208	91385.87085	4.520257322	41.58909458	0.002999973	2.609423926	93730.60072	4.653278772	46.39808061
35 to 39	0.005157888	2.569066734	89381.11471	4.414120766	37.46095271	0.003652773	2.583660892	92345.54586	4.577152152	42.04792288
40 to 44	0.006389769	2.588189874	87124.61962	4.290653316	33.36074061	0.004380763	2.582131296	90694.48813	4.487721273	37.76008463
45 to 49	0.008169291	2.61442208	84410.70785	4.140604279	29.34497597	0.005417633	2.610642956	88756.80825	4.381689865	33.52195273
50 to 54	0.011536749	2.616388228	81064.5508	3.94581219	25.44134536	0.007380905	2.605449148	86415.09604	4.246651099	29.35040724
55 to 59	0.015924219	2.605543079	76563.68873	3.689211447	21.77352661	0.009493529	2.610548897	83329.13246	4.075353382	25.33048397
60 to 64	0.022260623	2.597087924	70766.55464	3.361319424	18.33522862	0.013219963	2.630709484	79525.63482	3.857655367	21.40428105
65 to 69	0.031696294	2.575863719	63407.1183	2.948409591	15.15659963	0.019716487	2.625614948	74527.25521	3.563638814	17.64947525
70 to 74	0.044333235	2.55500456	54260.73583	2.454149139	12.28082592	0.0295863	2.626255286	67683.86171	3.169833622	14.15460786
75 to 79	0.065166705	2.516391905	43678.94538	1.889380933	9.657211562	0.048436402	2.603151826	58672.2544	2.643069961	10.92086958
80 to 84	0.093596742	2.45428742	31817.15084	1.29769418	7.369660426	0.080123267	2.532484656	46582.11552	1.968181091	8.111030254
85 to 89	0.154697065	2.368909933	20285.66695	0.731999131	5.268445063	0.13763586	2.427174629	31949.36603	1.204931313	5.771078855
90 to 94	0.249301455	2.146481142	9508.19326	0.282726746	3.736087332	0.224577135	2.217015199	16610.93345	0.525990888	4.090421661
95 plus	0.340620423	2.943905723	2719.576278	0.082014927	2.943905723	0.318907493	3.160460378	5571.46625	0.18474008	3.160460378

Table 15: Congo 2017 life table, by age and sex. mx=mortality rate, ax=mean person-years lived in an age interval among those who die in that age interval, lx=number of persons left alive at age x, nLx=person-years lived between age x and x+n, ex=life expectancy at age x.										
**Age Group**	**Male**					**Female**				
	**mx**	**ax**	**lx**	**nLx**	**ex**	**mx**	**ax**	**lx**	**nLx**	**ex**
Early Neonatal	0.972720377	0.009559228	100000	0.019000347	62.87811534	0.680578336	0.009568182	100000	0.019053482	63.00744419
Late Neonatal	0.060154392	0.02875053	98152.43041	0.056373724	64.04160667	0.037970969	0.028756649	98703.49595	0.056726346	63.8154612
Post Neonatal	0.018585804	0.460323538	97813.57371	0.895398748	64.20587731	0.015030312	0.460576111	98488.16948	0.903049801	63.89731032
1 to 4	0.004727499	1.993696707	96150.10086	3.80990069	64.3853099	0.004719372	1.993707543	97131.17299	3.848822647	63.85993367
5 to 9	0.001083783	2.49774212	94350.70746	4.704779239	61.57619198	0.001025316	2.497863927	95315.74489	4.753594692	61.03857675
10 to 14	0.000931211	2.648931471	93840.92632	4.681801073	56.89673238	0.00081696	2.63473122	94828.46106	4.732288811	56.33906444
15 to 19	0.001769397	2.670351834	93405.02214	4.651084894	52.14954411	0.001575828	2.678874433	94441.92135	4.704899823	51.55848551
20 to 24	0.002414843	2.601944318	92582.1074	4.602459556	47.58830872	0.002206869	2.672810452	93700.59724	4.66109161	46.94364047
25 to 29	0.003010171	2.594160767	91471.00857	4.540686042	43.13330019	0.003461129	2.660308481	92672.3654	4.596409425	42.43284595
30 to 34	0.003877794	2.609325394	90104.89834	4.463889676	38.74647815	0.004993314	2.627648049	91082.62027	4.500831084	38.12474718
35 to 39	0.005206688	2.621843345	88375.3824	4.364774157	34.45223083	0.006747091	2.605635167	88837.69227	4.371330672	34.0195148
40 to 44	0.007215716	2.621373234	86105.59628	4.232718856	30.28979566	0.008856364	2.590003738	85892.77718	4.205003307	30.09438049
45 to 49	0.009959824	2.620836726	83056.22921	4.056837945	26.30402856	0.01136427	2.581774333	82174.76026	3.999033052	26.33658939
50 to 54	0.014099809	2.612010718	79023.29829	3.822722012	22.51124564	0.014685183	2.575661248	77638.70708	3.7487654	22.72141327
55 to 59	0.019691712	2.61298019	73646.04763	3.517381425	18.96336016	0.018963742	2.589190182	72146.43314	3.450072781	19.25268914
60 to 64	0.029287379	2.593692844	66738.70178	3.117849782	15.65600931	0.026950137	2.596381324	65624.67067	3.082327489	15.9067409
65 to 69	0.042163165	2.564024301	57635.35987	2.614132916	12.72127163	0.039680215	2.580326194	57350.70767	2.617453995	12.82612364
70 to 74	0.061864591	2.524185451	46648.85827	2.023568252	10.11724283	0.060201594	2.542032157	47016.29037	2.049744973	10.07923339
75 to 79	0.089632652	2.467392452	34172.33458	1.393482661	7.895169608	0.091411789	2.479437469	34768.38633	1.415923315	7.740986023
80 to 84	0.130610309	2.390558232	21727.38616	0.81110614	6.012790422	0.137542846	2.389274115	21972.50218	0.811664199	5.827746675
85 to 89	0.196221807	2.264133648	11175.83434	0.364138011	4.446557394	0.204826245	2.258738287	10972.069	0.353383179	4.326231439
90 to 94	0.288744049	2.06509752	4057.717956	0.109999016	3.295213661	0.293549144	2.061293037	3839.117996	0.103799181	3.247324302
95 plus	0.371642408	2.691110604	891.0906905	0.024030175	2.691110604	0.379142442	2.639516977	831.7556981	0.022174924	2.639516977

Table 15: Congo 2100 life table, by age and sex. mx=mortality rate, ax=mean person-years lived in an age interval among those who die in that age interval, lx=number of persons left alive at age x, nLx=person-years lived between age x and x+n, ex=life expectancy at age x.										
**Age Group**	**Male**					**Female**				
	**mx**	**ax**	**lx**	**nLx**	**ex**	**mx**	**ax**	**lx**	**nLx**	**ex**
Early Neonatal	0.258906574	0.009581106	100000	0.019130564	75.65380175	0.175579825	0.00958366	100000	0.019145838	77.15732555
Late Neonatal	0.011843398	0.028763856	99504.93872	0.057229924	76.00821126	0.010131354	0.028764329	99663.97448	0.057324216	77.39563028
Post Neonatal	0.00514677	0.461278218	99437.18607	0.915913891	76.00209669	0.004331199	0.461336155	99605.9272	0.917813339	77.3826889
1 to 4	0.000585591	1.999219213	98965.9249	3.954011468	75.43749607	0.000727649	1.999029802	99208.52676	3.962579559	76.76630292
5 to 9	0.000283979	2.499408378	98734.78565	4.933240709	71.6068726	0.000296232	2.49938285	98920.65246	4.942377926	72.98126522
10 to 14	0.000291129	2.82461624	98594.88702	4.926624263	66.70362462	0.000278315	2.753512089	98774.51414	4.935621107	68.08437664
15 to 19	0.000739101	2.739626931	98451.71283	4.914402956	61.79523417	0.000616332	2.688565701	98637.47572	4.92484921	63.17400507
20 to 24	0.001155486	2.605497212	98089.75603	4.891015316	57.00908576	0.000823684	2.649119889	98334.65193	4.907226535	58.35796887
25 to 29	0.001297078	2.56222654	97529.05405	4.861184603	52.31715463	0.001196652	2.620025721	97931.56135	4.88273163	53.58466404
30 to 34	0.001524998	2.577157481	96904.40708	4.827466349	47.63504016	0.001555308	2.631291422	97349.12221	4.849455883	48.88557625
35 to 39	0.001803169	2.599546847	96175.16994	4.788145291	42.97414901	0.001999019	2.63886075	96598.28257	4.807125014	44.2407011
40 to 44	0.002365633	2.652249466	95319.00001	4.739676985	38.3339518	0.002735723	2.65869756	95642.17904	4.751537771	39.65091472
45 to 49	0.003444838	2.678717263	94206.45599	4.673100912	33.74996483	0.003914244	2.657723237	94348.74533	4.674585249	35.15074881
50 to 54	0.005388847	2.693060436	92607.09556	4.573820266	29.27813735	0.005724267	2.643420669	92527.83339	4.565052758	30.77769258
55 to 59	0.008739542	2.686368736	90162.00623	4.419477721	24.98559176	0.008165565	2.625234678	89931.76235	4.411614761	26.57077845
60 to 64	0.013905641	2.658732362	86346.65308	4.183135326	20.94973564	0.011153064	2.625736721	86368.76255	4.208632617	22.53174794
65 to 69	0.021382261	2.642701144	80635.69084	3.842771711	17.21821232	0.016319037	2.639513004	81757.13702	3.939572346	18.61381521
70 to 74	0.032979832	2.609167602	72665.67781	3.377210203	13.79082508	0.025238663	2.686011565	75506.83802	3.575718915	14.8851792
75 to 79	0.051176043	2.593471974	61989.19109	2.779597602	10.6948705	0.047759363	2.646442506	66966.18092	3.03656079	11.36935053
80 to 84	0.088554751	2.511647783	48750.62885	2.036462574	7.881904317	0.08669212	2.533361123	53871.22135	2.288685346	8.425593803
85 to 89	0.146864493	2.397563454	32631.64076	1.225999395	5.591109376	0.141193047	2.440600794	37510.69656	1.464438186	6.019714273
90 to 94	0.239862101	2.157186874	16941.97409	0.52939168	3.912652544	0.225225827	2.204980135	21314.41633	0.71439899	4.248606607
95 plus	0.332505412	3.044517085	5629.221272	0.18479204	3.044517085	0.317904458	3.261698536	8455.56976	0.320277564	3.261698536

Table 15: Democratic Republic of the Congo 2017 life table, by age and sex. mx=mortality rate, ax=mean person-years lived in an age interval among those who die in that age interval, lx=number of persons left alive at age x, nLx=person-years lived between age x and x+n, ex=life expectancy at age x.

Age Group	Male					Female				
	mx	ax	lx	nLx	ex	mx	ax	lx	nLx	ex
Early Neonatal	1.338831617	0.009548007	100000	0.018934046	60.00971622	0.988819366	0.009558734	100000	0.01899743	63.98610392
Late Neonatal	0.104740338	0.028738231	97466.30527	0.055908062	61.54873902	0.086847861	0.028743166	98122.23771	0.056313209	65.19023938
Post Neonatal	0.031793998	0.459385276	96881.37905	0.88149349	61.86272979	0.031261425	0.459423107	97633.62988	0.888555338	65.45886748
1 to 4	0.009428502	1.987428972	94079.09106	3.693142806	62.76829112	0.008251632	1.98899803	94856.17944	3.732352251	66.43859491
5 to 9	0.001154704	2.497594369	90600.12842	4.516959827	61.1038551	0.001038457	2.497836548	91778.51266	4.57703733	64.6009476
10 to 14	0.000948953	2.631153362	90078.76926	4.493839953	56.44279017	0.000741525	2.534012253	91303.39338	4.556839323	59.92402481
15 to 19	0.001769983	2.690639614	89652.43757	4.464379287	51.69840545	0.001168922	2.648595782	90965.60282	4.535817625	55.13713119
20 to 24	0.002606345	2.584450223	88862.38291	4.415328482	47.13341702	0.001590662	2.617825232	90435.5626	4.50471708	50.4447167
25 to 29	0.002887573	2.563432281	87711.83917	4.354962945	42.71660894	0.002097572	2.627816322	89719.29696	4.463763731	45.82637713
30 to 34	0.003579579	2.600911971	86454.77814	4.285951777	38.29930797	0.002928962	2.639355584	88783.37312	4.408692915	41.28144489
35 to 39	0.004765666	2.627971235	84921.42834	4.198647314	33.94260895	0.00415072	2.624142468	87492.55749	4.331930721	36.85092755
40 to 44	0.006777718	2.642677762	82921.99221	4.080964768	29.69613023	0.005594268	2.612661698	85695.20195	4.228333789	32.56776583
45 to 49	0.009949719	2.646354539	80158.97823	3.916358869	25.62640879	0.007545289	2.627902663	83330.75334	4.093344926	28.41501299
50 to 54	0.014937134	2.627789856	76268.09892	3.68312972	21.7962254	0.010923668	2.618685446	80244.64246	3.910643866	24.40314202
55 to 59	0.021571183	2.614178802	70777.75352	3.366053703	18.28058267	0.015048147	2.629427825	75979.37906	3.668375005	20.62150222
60 to 64	0.032138189	2.585720081	63534.83779	2.948682716	15.06488405	0.023012048	2.620765468	70472.47086	3.341258582	17.02362642
65 to 69	0.045894716	2.554465437	54089.81089	2.43264117	12.243811	0.034387222	2.597604487	62809.40403	2.901873213	13.77805872
70 to 74	0.067355918	2.508487909	42974.97596	1.841417616	9.753227257	0.052722409	2.557582698	52880.87531	2.34409185	10.87733081
75 to 79	0.094672876	2.447219026	30639.62956	1.235415951	7.679286436	0.078932652	2.504214843	40602.01821	1.698249027	8.399604445
80 to 84	0.134940555	2.372993369	19019.60539	0.703540801	5.893233695	0.121722638	2.42793809	27304.12347	1.042197205	6.286008343
85 to 89	0.200512289	2.254479841	9595.422008	0.31023521	4.378527324	0.187925332	2.294102621	14739.43378	0.490206403	4.605163401
90 to 94	0.292632189	2.056779119	3415.273692	0.091996505	3.258380513	0.276970773	2.099034006	5610.78496	0.15619178	3.410665541
95 plus	0.374624139	2.66985295	736.5464599	0.019731198	2.66985295	0.365149373	2.73995865	1318.139264	0.036321318	2.73995865

Table 15: Democratic Republic of the Congo 2100 life table, by age and sex. mx=mortality rate, ax=mean person-years lived in an age interval among those who die in that age interval, lx=number of persons left alive at age x, nLx=person-years lived between age x and x+n, ex=life expectancy at age x.

Age Group	Male					Female				
	mx	ax	lx	nLx	ex	mx	ax	lx	nLx	ex
Early Neonatal	0.361830357	0.009577951	100000	0.019111724	74.05268779	0.244911822	0.009581535	100000	0.019133125	79.77690616
Late Neonatal	0.022922096	0.0287608	99308.92273	0.057099011	74.54461762	0.020491671	0.028761471	99531.5847	0.057231023	80.12999226
Post Neonatal	0.003794433	0.461374285	99178.14888	0.914098663	74.58454907	0.004222744	0.461343859	99414.37967	0.916094679	80.16612237
1 to 4	0.001111589	1.998517882	98831.5744	3.944498266	73.91956343	0.00108467	1.998553774	99027.76386	3.952538989	79.55211079
5 to 9	0.000192513	2.499598931	98393.70275	4.917321722	70.23735841	0.000202327	2.499578486	98599.53106	4.927487087	75.88651517
10 to 14	0.00022008	2.995948977	98299.18337	4.912779209	65.30200999	0.00018799	2.660146792	98499.9715	4.922828541	70.96019174
15 to 19	0.000702054	2.795780283	98191.22084	4.901983338	60.37004738	0.000339932	2.661936668	98407.55736	4.916470982	66.02392309
20 to 24	0.001221958	2.600980988	97847.53154	4.878090256	55.57088095	0.000449114	2.62542109	98240.64417	4.906803543	61.13093187
25 to 29	0.00129362	2.552156636	97252.59528	4.847295399	50.89288815	0.000609688	2.631512122	98020.57707	4.893967829	56.26139275
30 to 34	0.001542619	2.568815538	96627.10514	4.813338969	46.20387026	0.000834448	2.62764061	97722.61812	4.876470379	51.42385744
35 to 39	0.001810747	2.619948204	95886.78248	4.773824786	41.53848586	0.001107096	2.632903305	97316.33546	4.853114083	46.6262401
40 to 44	0.002618954	2.675725883	95025.23844	4.722579034	36.88853271	0.00154799	2.68905235	96780.03763	4.821767385	41.86797169
45 to 49	0.004059272	2.702298671	93793.26952	4.646451035	32.33386369	0.002518242	2.708541737	96035.47216	4.774232955	37.1684475
50 to 54	0.006709248	2.692009067	91915.88465	4.525948965	27.93349874	0.004078764	2.672832114	94836.94683	4.697331103	32.5987174
55 to 59	0.010624377	2.666955069	88897.26892	4.337903459	23.78330705	0.005968824	2.655737131	92929.59073	4.58256403	28.20515623
60 to 64	0.016359917	2.636937512	84322.22356	4.060362828	19.92128071	0.008797379	2.654431459	90210.58677	4.419912951	23.96636407
65 to 69	0.02411162	2.619079979	77743.85703	3.678372354	16.3767825	0.01328037	2.647027301	86354.62646	4.188136918	19.9070062
70 to 74	0.036557102	2.596767713	68987.62115	3.17469753	13.1202602	0.019965894	2.668059412	80859.9649	3.865620958	16.0678507
75 to 79	0.056181992	2.555088843	57568.26283	2.537437633	10.21100082	0.034399967	2.653740988	73273.3805	3.395683445	12.44454438
80 to 84	0.086015697	2.490821571	43627.82028	1.804269145	7.674980919	0.05988199	2.599508135	61880.80384	2.717611276	9.244368839
85 to 89	0.146169682	2.391090982	28591.3956	1.04515699	5.450402442	0.111389712	2.50594265	46216.37795	1.826477361	6.518129444
90 to 94	0.241036769	2.16283616	13820.69866	0.41551166	3.833937676	0.195807055	2.27655995	26772.44525	0.886870418	4.531833835
95 plus	0.334063904	2.999987537	4075.945264	0.124555817	2.999987537	0.292883436	3.435298243	10122.28864	0.358557095	3.435298243

Table 15: Equatorial Guinea 2017 life table, by age and sex. mx=mortality rate, ax=mean person-years lived in an age interval among those who die in that age interval, lx=number of persons left alive at age x, nLx=person-years lived between age x and x+n, ex=life expectancy at age x.

Age Group	Male					Female				
	mx	ax	lx	nLx	ex	mx	ax	lx	nLx	ex
Early Neonatal	0.858431654	0.00956273	100000	0.019021114	65.0473376	0.665383928	0.009568647	100000	0.019056253	67.10209083
Late Neonatal	0.06156362	0.028750141	98367.70433	0.056495075	66.10143481	0.045119851	0.028754677	98732.27796	0.056731236	67.93939232
Post Neonatal	0.018366214	0.460339137	98020.14925	0.897385443	66.27709795	0.016947923	0.460439889	98476.41724	0.902148287	68.05738237
1 to 4	0.003632691	1.995156431	96373.80735	3.82712137	66.47380131	0.003328576	1.995561913	96948.52869	3.85226457	68.19438002
5 to 9	0.001194664	2.497511118	94985.73072	4.735134094	63.41352109	0.001016107	2.497883111	95667.61384	4.771258511	65.07734459
10 to 14	0.000925177	2.538976157	94420.19863	4.710287078	58.77831379	0.000763023	2.517261253	95183.14191	4.750157968	60.39533674
15 to 19	0.001379165	2.635071436	93984.63007	4.68397542	54.03886826	0.001082643	2.664850193	94820.91837	4.729115393	55.61619965
20 to 24	0.001847508	2.640333532	93339.24473	4.646744784	49.39371079	0.001650107	2.782886252	94309.3452	4.698311589	50.90240728
25 to 29	0.002676912	2.653997019	92481.97228	4.595291657	44.82593051	0.003388101	2.727102947	93534.89601	4.640988787	46.29872205
30 to 34	0.003920645	2.632617077	91253.6947	4.520768433	40.39186855	0.005416562	2.632537854	91964.65444	4.540026265	42.03973613
35 to 39	0.005307924	2.604533948	89484.43061	4.418144154	36.13673541	0.007027771	2.566812977	89510.19278	4.400394641	38.11783209
40 to 44	0.006848721	2.595955504	87144.80869	4.286826966	32.03521458	0.008082229	2.53807597	86425.51069	4.237160075	34.38396104
45 to 49	0.008937465	2.595358812	84217.22232	4.122558541	28.05657254	0.009129462	2.536148985	83010.06302	4.05951371	30.69003207
50 to 54	0.011821273	2.602196104	80545.02859	3.916734176	24.21477565	0.010598406	2.541250697	79315.42946	3.865586883	26.99495885
55 to 59	0.016311083	2.620820891	75936.35327	3.655717996	20.52379627	0.012467537	2.579349523	75239.31082	3.652567876	23.31172258
60 to 64	0.02439511	2.608745261	70008.33745	3.308641453	17.03851727	0.017251123	2.609973602	70719.85682	3.397350164	19.62879416
65 to 69	0.035584919	2.581642097	61991.31271	2.855738786	13.90625666	0.025012033	2.610389459	64921.16967	3.06539831	16.14193381
70 to 74	0.052372502	2.545168338	51911.20134	2.302402112	11.11176159	0.037728026	2.591239508	57365.95496	2.633411576	12.92181092
75 to 79	0.076092604	2.497274752	39969.72789	1.681688889	8.68591552	0.057471916	2.55519738	47621.57462	2.093914068	10.04478289
80 to 84	0.112542969	2.428070162	27307.75337	1.061555606	6.580108789	0.089633492	2.496827453	35879.15861	1.473416733	7.526411628
85 to 89	0.176885061	2.310042863	15485.38834	0.526352804	4.784560677	0.150351358	2.386878828	23064.82429	0.835466399	5.387062836
90 to 94	0.270889769	2.103118454	6258.802138	0.17594695	3.476999172	0.238704203	2.187178416	10870.94474	0.328991932	3.864828549
95 plus	0.357799482	2.79563501	1524.351467	0.042804703	2.79563501	0.331826913	3.020046225	3213.604418	0.098859608	3.020046225

Table 15: Equatorial Guinea 2100 life table, by age and sex. mx=mortality rate, ax=mean person-years lived in an age interval among those who die in that age interval, lx=number of persons left alive at age x, nLx=person-years lived between age x and x+n, ex=life expectancy at age x.

Age Group	Male					Female				
	mx	ax	lx	nLx	ex	mx	ax	lx	nLx	ex
Early Neonatal	0.18334546	0.009583422	100000	0.019144414	78.66080098	0.15970288	0.009584146	100000	0.01914875	81.73195246
Late Neonatal	0.007836597	0.028764962	99649.14948	0.057319466	78.91835557	0.008028692	0.028764909	99694.29578	0.057345118	81.96320587
Post Neonatal	0.003421303	0.461400792	99604.23513	0.918182925	78.89635387	0.003677757	0.461382574	99648.26336	0.91848006	81.94350614
1 to 4	0.000263009	1.999649322	99290.17468	3.969519692	78.2208198	0.000256964	1.999657381	99310.53576	3.970381692	81.29662881
5 to 9	0.000181594	2.499621678	99185.83491	4.957041421	74.30099159	0.000167549	2.49965094	99208.57252	4.958352157	77.37794553
10 to 14	0.000168819	2.75865111	99095.83789	4.952898604	69.3659911	0.000149748	2.733775119	99125.52836	4.95455927	72.44044935
15 to 19	0.000372756	2.729523194	99012.24685	4.946420929	64.42210289	0.000300679	2.700502067	99051.36106	4.949170155	67.49261674
20 to 24	0.000574867	2.653774655	98827.93691	4.934744714	59.53636047	0.000463261	2.714787352	98902.60105	4.940017784	62.58962767
25 to 29	0.000799262	2.598188253	98544.50485	4.917846445	54.69865439	0.000893402	2.650280719	98673.99668	4.923439042	57.72697471
30 to 34	0.001006391	2.583389321	98152.03329	4.895675959	49.90525802	0.001157349	2.670305176	98235.69809	4.898194917	52.97012471
35 to 39	0.001161937	2.570572404	97660.76546	4.869313915	45.14173798	0.001395789	2.558531377	97672.77062	4.867018294	48.25875968
40 to 44	0.001378742	2.678576457	97097.41855	4.839240759	40.38702316	0.001392364	2.681209621	96999.3581	4.833990982	43.57541555
45 to 49	0.002148812	2.732925049	96433.67966	4.798114769	35.64369456	0.002132756	2.671575214	96331.60686	4.792664429	38.85773983
50 to 54	0.003528672	2.772659272	95407.94708	4.732902479	30.99184438	0.002981141	2.65307705	95313.90667	4.732600539	34.24043273
55 to 59	0.006426634	2.745204167	93748.64009	4.62024674	26.4823154	0.004250361	2.660933099	93911.54988	4.649321076	29.70667843
60 to 64	0.01071921	2.698759952	90808.76349	4.431683772	22.23802558	0.006094156	2.671881387	91950.10984	4.53352388	25.27726002
65 to 69	0.017246373	2.67069979	86119.86433	4.141733623	18.28652207	0.009557193	2.696219283	89211.35052	4.364976348	20.96357469
70 to 74	0.027311459	2.63883365	79115.31092	3.720916906	14.65752276	0.015593235	2.735849106	85091.29785	4.110798411	16.84134816
75 to 79	0.042980268	2.614369637	69216.37575	3.148179775	11.37064954	0.030005251	2.701847536	78782.98159	3.688629346	12.96186853
80 to 84	0.07200881	2.550064187	56151.94742	2.400626689	8.420494442	0.054892893	2.633269138	67947.28398	3.015163775	9.599325544
85 to 89	0.128669837	2.44126627	39546.59843	1.505457804	5.914294703	0.104460863	2.529286894	51831.63035	2.075232529	6.757340089
90 to 94	0.222973964	2.193774647	21069.43545	0.658902176	4.085260511	0.187778143	2.291109306	30939.54782	1.039022864	4.675565709
95 plus	0.319365864	3.143779213	6955.534146	0.224687009	3.143779213	0.285455648	3.525303501	12138.11854	0.439921045	3.525303501

Table 15: Gabon 2017 life table, by age and sex. mx=mortality rate, ax=mean person-years lived in an age interval among those who die in that age interval, lx=number of persons left alive at age x, nLx=person-years lived between age x and x+n, ex=life expectancy at age x.

Age Group	Male					Female				
	mx	ax	lx	nLx	ex	mx	ax	lx	nLx	ex
Early Neonatal	1.024595047	0.009557638	100000	0.01899091	64.47958679	0.796175802	0.009564638	100000	0.01903242	71.68353325
Late Neonatal	0.089225791	0.02874251	98054.54192	0.056270441	65.73884599	0.063615756	0.028749575	98484.86357	0.056558984	72.76675626
Post Neonatal	0.014100124	0.460642189	97552.6659	0.894857105	66.01933871	0.011096686	0.460855547	98125.16021	0.901353776	72.97587859
1 to 4	0.003375249	1.995499683	96291.56217	3.825796281	65.95483278	0.002060109	1.997253192	97125.31136	3.869056903	72.79928264
5 to 9	0.000781447	2.498371986	95001.19928	4.740795643	62.82409542	0.000518701	2.498919373	96328.64395	4.810192605	69.38515137
10 to 14	0.000779103	2.69274521	94630.86958	4.723054536	58.06012953	0.000506414	2.5837736	96079.16879	4.7980861	64.55868598
15 to 19	0.001512581	2.682325634	94262.9718	4.696690406	53.27610397	0.000723801	2.617712226	95836.21425	4.783565656	59.71576329
20 to 24	0.002134128	2.579682309	93552.56139	4.653601294	48.65957644	0.000923294	2.624846827	95490.00734	4.76405883	54.92256469
25 to 29	0.002375066	2.566495202	92559.66678	4.601404112	44.15297493	0.001291862	2.669384566	95050.19693	4.738249752	50.16416854
30 to 34	0.002958128	2.619152396	91467.37337	4.541398144	39.64866414	0.001998262	2.680054019	94438.20307	4.700115138	45.47143189
35 to 39	0.004160907	2.649235772	90124.98676	4.462627193	35.19933337	0.003054411	2.650104003	93499.31174	4.641657157	40.90046362
40 to 44	0.006132173	2.647077877	88269.90552	4.350776993	30.88252222	0.004293999	2.615454463	92082.22949	4.557476667	36.48799544
45 to 49	0.008903075	2.644591453	85605.17355	4.192447565	26.7606074	0.005632663	2.605053696	90126.39775	4.446382412	32.2211901
50 to 54	0.013225682	2.629604974	81878.08425	3.969647511	22.85790807	0.007477446	2.60038467	87624.0423	4.304070946	28.06462373
55 to 59	0.019049999	2.619781079	76637.6147	3.665948003	19.24151883	0.009836328	2.626367458	84409.93345	4.124365818	24.03152874
60 to 64	0.02834394	2.596632617	69668.10765	3.26162699	15.9059796	0.014596522	2.639934196	80361.23289	3.884551505	20.10703161
65 to 69	0.040953739	2.566376252	60441.26477	2.748578474	12.94066963	0.022142905	2.636187729	74705.52739	3.550072783	16.42683483
70 to 74	0.059819204	2.525972041	49203.27151	2.1432789	10.31360417	0.034962857	2.614382224	66873.22769	3.087255681	13.04031259
75 to 79	0.086014359	2.474745818	36393.19649	1.495087104	8.056316392	0.05574494	2.573423757	56127.64718	2.47378645	10.03698244
80 to 84	0.127401245	2.403620335	23538.94722	0.88444939	6.104026941	0.090492874	2.503765512	42423.11919	1.732937244	7.453764385
85 to 89	0.193002764	2.271464968	12272.78663	0.402045048	4.498658187	0.151788955	2.381667821	26869.23936	0.963928022	5.335978373
90 to 94	0.285816438	2.071367104	4517.82256	0.123007184	3.323368967	0.240374348	2.183633835	12363.38706	0.369933692	3.834978661
95 plus	0.369392192	2.707344075	1004.435984	0.027207566	2.707344075	0.333396272	3.001441633	3539.090479	0.106844709	3.001441633

Table 15: Gabon 2100 life table, by age and sex. mx=mortality rate, ax=mean person-years lived in an age interval among those who die in that age interval, lx=number of persons left alive at age x, nLx=person-years lived between age x and x+n, ex=life expectancy at age x.

Age Group	Male					Female				
	mx	ax	lx	nLx	ex	mx	ax	lx	nLx	ex
Early Neonatal	0.287089961	0.009580242	100000	0.019125394	74.64352186	0.26631007	0.009580879	100000	0.019129202	81.42554609
Late Neonatal	0.02353374	0.028760632	99451.09524	0.057179729	75.03586996	0.025663392	0.028760044	99490.74593	0.057199027	81.82243121
Post Neonatal	0.002530851	0.461464048	99316.57038	0.915907374	75.0797799	0.002376175	0.461475036	99344.01338	0.91622592	81.88551081
1 to 4	0.000558915	1.99925478	99084.82946	3.958968873	74.33082484	0.000282277	1.99962363	99126.37845	3.962818399	81.14065692
5 to 9	0.000197654	2.499588221	98863.71014	4.94074454	70.4924117	0.000147647	2.499692401	99014.56411	4.948901937	77.22987438
10 to 14	0.000259658	2.85672482	98766.08852	4.935537856	65.55952677	0.000190421	2.655055381	98941.52312	4.944859505	72.28492494
15 to 19	0.00062363	2.736823192	98637.97487	4.924955988	60.64091298	0.000280495	2.593227228	98847.4033	4.93903818	67.35107635
20 to 24	0.000979126	2.595361255	98330.94302	4.905008914	55.82109376	0.000317689	2.608013143	98708.91685	4.931699238	62.44170373
25 to 29	0.001083994	2.560415712	97850.96985	4.879662847	51.08116718	0.000445996	2.645297615	98552.31233	4.922438468	57.53656049
30 to 34	0.001311601	2.584277225	97322.61382	4.850754312	46.34352588	0.000619995	2.635331538	98332.94206	4.909414812	52.65860574
35 to 39	0.001600857	2.635504614	96687.47825	4.816151079	41.63000644	0.00081079	2.656199717	98028.92544	4.892129608	47.81301836
40 to 44	0.002354045	2.689333253	95918.15744	4.769920478	36.94164297	0.001203536	2.711680437	97632.85548	4.868214857	42.99521289
45 to 49	0.00370327	2.705638233	94798.13197	4.699959007	32.34496876	0.002011246	2.706982909	97047.99771	4.830074936	38.23567131
50 to 54	0.006062708	2.708960322	93062.45454	4.5893918	27.89641641	0.003149751	2.679369969	96079.03469	4.769039451	33.58960452
55 to 59	0.010069212	2.672834938	90289.14361	4.411272023	23.66862214	0.004659532	2.651107921	94582.59628	4.677954082	29.07274217
60 to 64	0.015359286	2.644319325	85864.58014	4.143725521	19.74966367	0.006529356	2.66450523	92414.2685	4.551610465	24.68560267
65 to 69	0.023269559	2.632497675	79525.38303	3.769342183	16.1129909	0.010221245	2.68908611	89462.24829	4.37039963	20.40425047
70 to 74	0.036425428	2.611332745	70792.12735	3.25732369	12.77724241	0.016634204	2.73029621	85039.09877	4.098550081	16.31681931
75 to 79	0.058327091	2.566903654	58978.8067	2.584009134	9.815384094	0.032150333	2.701727886	78311.4211	3.649796522	12.47414794
80 to 84	0.093859119	2.494207099	43981.38856	1.782793291	7.29032627	0.061284719	2.622520678	66790.47106	2.924861425	9.151477651
85 to 89	0.156035457	2.363431471	27366.02651	0.972238933	5.207221005	0.113467979	2.499122926	49383.16856	1.94042553	6.451246379
90 to 94	0.250978848	2.144208095	12336.10082	0.360705818	3.70330847	0.198162145	2.271831966	28235.38742	0.930349119	4.492256971
95 plus	0.342091978	2.925294943	3358.592583	0.098843077	2.925294943	0.295051341	3.410599952	10529.30743	0.370415698	3.410599952

Table 15: Eastern Sub-Saharan Africa 2017 life table, by age and sex. mx=mortality rate, ax=mean person-years lived in an age interval among those who die in that age interval, lx=number of persons left alive at age x, nLx=person-years lived between age x and x+n, ex=life expectancy at age x.

Age Group	Male					Female				
	mx	ax	lx	nLx	ex	mx	ax	lx	nLx	ex
Early Neonatal	1.148017043	0.009553855	100000	0.018968505	62.66530801	0.816012509	0.00956403	100000	0.019028798	67.64011644
Late Neonatal	0.103834952	0.028738481	97822.41078	0.056113611	64.04080244	0.079580136	0.028745171	98447.23666	0.056511408	68.68759983
Post Neonatal	0.023546788	0.459971124	97239.77075	0.888114194	64.36680189	0.021309587	0.460130048	97997.52464	0.895956444	68.94513269
1 to 4	0.004997251	1.993337044	95148.63367	3.76816115	64.84802532	0.004588511	1.99388202	96088.32693	3.808476965	69.38257636
5 to 9	0.001144357	2.497615925	93265.70254	4.649969806	62.11703494	0.000938147	2.498045527	94340.87063	4.70599778	66.63082835
10 to 14	0.000976757	2.63191892	92733.59747	4.625979299	57.45913361	0.000765268	2.567373827	93899.38594	4.686244912	61.93235036
15 to 19	0.001779885	2.667350727	92281.76236	4.595010619	52.7275785	0.001194718	2.639342855	93540.7677	4.663885478	57.15993824
20 to 24	0.002438901	2.598265043	91463.91179	4.546564435	48.17517554	0.001579532	2.634757309	92983.56912	4.631874979	52.48662336
25 to 29	0.002989728	2.594719889	90355.06955	4.485499396	43.73444885	0.002246743	2.628528072	92251.96328	4.588152543	47.88193749
30 to 34	0.003898392	2.604618449	89014.06611	4.409527047	39.35416649	0.003019562	2.611797292	91221.1636	4.528400388	43.39325735
35 to 39	0.005110335	2.60989902	87295.14181	4.31209031	35.07775363	0.003960975	2.600887315	89853.85963	4.450402429	39.01379261
40 to 44	0.006882545	2.615187069	85091.65097	4.185880989	30.91847157	0.005102987	2.597714417	88091.1522	4.351218792	34.74235191
45 to 49	0.009442734	2.619070527	82210.89142	4.020167621	26.91016443	0.006634673	2.609048377	85870.82417	4.226498915	30.57339184
50 to 54	0.013290453	2.612620252	78415.03444	3.800185058	23.08591092	0.00907399	2.601155614	83066.8132	4.064866707	26.51720897
55 to 59	0.018578396	2.616142522	73364.9269	3.512694034	19.49506752	0.011904388	2.627316337	79378.65451	3.859920311	22.62823952
60 to 64	0.027729181	2.597022103	66839.7782	3.133245602	16.14276047	0.018118622	2.632709423	74784.23667	3.585449268	18.85683866
65 to 69	0.039886634	2.567221255	58152.99561	2.650509472	13.16617401	0.027239783	2.612506379	68289.02297	3.205997085	15.39982637
70 to 74	0.058167389	2.528032073	47583.2436	2.080141357	10.52064171	0.041420437	2.582624067	59558.07235	2.706944627	12.27433986
75 to 79	0.083189988	2.477612344	35486.78485	1.466679173	8.24543035	0.062959188	2.540520661	48349.40177	2.093440524	9.521303943
80 to 84	0.121946566	2.407914015	23289.52263	0.884880201	6.266869107	0.097637972	2.475429482	35174.7198	1.411091212	7.136528887
85 to 89	0.187124641	2.285131841	12502.75065	0.414596038	4.597403652	0.160393131	2.358711332	21404.05632	0.751857837	5.136563298
90 to 94	0.280432764	2.082882622	4747.307198	0.130578412	3.376587924	0.249326059	2.163055167	9350.831172	0.273901923	3.719321414
95 plus	0.365236903	2.737983396	1086.433153	0.029751684	2.737983396	0.34131009	2.929984516	2524.701009	0.073997111	2.929984516

Table 15: Eastern Sub-Saharan Africa 2100 life table, by age and sex. mx=mortality rate, ax=mean person-years lived in an age interval among those who die in that age interval, lx=number of persons left alive at age x, nLx=person-years lived between age x and x+n, ex=life expectancy at age x.

Age Group	Male					Female				
	mx	ax	lx	nLx	ex	mx	ax	lx	nLx	ex
Early Neonatal	0.235928302	0.00958181	100000	0.019134765	76.1262724	0.180973796	0.009583494	100000	0.019144841	81.88896928
Late Neonatal	0.020018758	0.028761601	99548.62155	0.057241581	76.45204673	0.020917804	0.028761353	99653.5528	0.057300437	82.15432254
Post Neonatal	0.003895189	0.461367128	99434.04224	0.916413532	76.48252166	0.003771224	0.461375934	99533.70495	0.917384493	82.19561873
1 to 4	0.000652783	1.999129623	99077.12241	3.957916601	75.83285692	0.00061997	1.999173374	99187.77225	3.962596689	81.557178
5 to 9	0.000270348	2.499436775	98818.82878	4.937604738	72.02538188	0.000247155	2.499485093	98942.17435	4.944054992	77.75431438
10 to 14	0.000293173	2.762577676	98685.3943	4.93103948	67.11910408	0.000233246	2.575841895	98820.05654	4.938210728	72.84714147
15 to 19	0.000644463	2.695627223	98540.91561	4.919760296	62.21313007	0.000330518	2.604283665	98704.98191	4.931348479	67.92895192
20 to 24	0.000916509	2.59531382	98224.24992	4.900434414	57.40422164	0.000399703	2.629985833	98542.17582	4.922451323	63.03677178
25 to 29	0.001053947	2.583895223	97776.81544	4.87643368	52.654247	0.000580152	2.629927886	98345.72399	4.910538655	58.15737486
30 to 34	0.001324893	2.574162573	97264.41542	4.847659401	47.91785987	0.000763643	2.60501974	98061.08066	4.894100546	53.31844424
35 to 39	0.001526524	2.604128375	96623.58494	4.813612945	43.21842323	0.000959662	2.616106608	97687.73009	4.873238183	48.51216678
40 to 44	0.002108318	2.672251709	95890.67684	4.7711457	38.52861852	0.001297725	2.656847321	97220.55397	4.846283239	43.73257285
45 to 49	0.003303835	2.688065933	94886.7742	4.708407767	33.90730877	0.001934409	2.679923712	96592.28439	4.80803805	38.99943519
50 to 54	0.005209764	2.697838125	93333.34273	4.611406676	29.42547013	0.002994657	2.662588399	95663.21384	4.749922217	34.35156064
55 to 59	0.008557039	2.686901492	90935.11508	4.458597303	25.12813736	0.004339589	2.659352787	94242.65913	4.664715791	29.82807909
60 to 64	0.013704439	2.647403941	87127.97298	4.220637384	21.10605587	0.006428116	2.669264299	92221.39981	4.543090777	25.42238925
65 to 69	0.020325335	2.628371258	81360.09677	3.881504445	17.41135703	0.010057027	2.66577492	89305.97242	4.362987433	21.16283851
70 to 74	0.030884397	2.617644184	73503.84721	3.424389671	13.98983658	0.015467333	2.711684992	84929.60686	4.101618694	17.11375043
75 to 79	0.049173476	2.572527406	62981.16511	2.815343254	10.88892838	0.029244775	2.668060242	78607.16277	3.680293918	13.26940394
80 to 84	0.074055953	2.504226161	49238.02132	2.081162125	8.212806062	0.049119885	2.615992505	67895.05973	3.040943077	9.940317443
85 to 89	0.131774285	2.430245606	33980.81454	1.27326158	5.78387158	0.096594072	2.553811159	53058.20526	2.14957442	6.986904547
90 to 94	0.226785933	2.190396928	17402.80892	0.533831453	4.012919849	0.179107863	2.309950158	32485.11257	1.099653729	4.809576307
95 plus	0.32264332	3.102395659	5419.888196	0.169333614	3.102395659	0.277555099	3.608630124	12972.43082	0.471478567	3.608630124

Table 15: Burundi 2017 life table, by age and sex. mx=mortality rate, ax=mean person-years lived in an age interval among those who die in that age interval, lx=number of persons left alive at age x, nLx=person-years lived between age x and x+n, ex=life expectancy at age x.										
**Age Group**	**Male**					**Female**				
	**mx**	**ax**	**lx**	**nLx**	**ex**	**mx**	**ax**	**lx**	**nLx**	**ex**
Early Neonatal	1.071049385	0.009556214	100000	0.018982479	60.11711194	0.829149305	0.009563628	100000	0.019026415	63.96660932
Late Neonatal	0.145812449	0.028726901	97967.20883	0.056129013	61.34391533	0.10884809	0.028737098	98422.55444	0.056449765	64.97179655
Post Neonatal	0.029816816	0.459525726	97149.09377	0.884738779	61.80245517	0.0262346	0.459780192	97808.22959	0.892205517	65.32198248
1 to 4	0.00760888	1.98985499	94512.82693	3.723617686	62.59024901	0.006835433	1.990886206	95468.37917	3.767029922	65.98819338
5 to 9	0.001188913	2.497523099	91682.60838	4.570536506	60.46259572	0.001042263	2.497828621	92894.94719	4.632669076	63.7617062
10 to 14	0.001006275	2.674263756	91139.39394	4.546331534	55.80765353	0.000771804	2.56929846	92412.23622	4.611961144	59.08164344
15 to 19	0.002051305	2.679262014	90682.00556	4.512621255	51.07526076	0.001310104	2.645183908	92056.34679	4.588663739	54.30000845
20 to 24	0.002814334	2.563825119	89756.4486	4.457273225	46.57338625	0.001702064	2.590957722	91455.28296	4.554096947	49.63917077
25 to 29	0.002987014	2.549544891	88502.3076	4.392976914	42.19555181	0.002084135	2.598674754	90680.35594	4.511449602	45.0407883
30 to 34	0.003628647	2.606249917	87190.67699	4.322016325	37.79047643	0.002740705	2.639279536	89740.4246	4.458187021	40.48464159
35 to 39	0.004991503	2.646976962	85623.43249	4.231514823	33.43287883	0.004006626	2.655100558	88519.05503	4.384781917	36.00579702
40 to 44	0.007451221	2.646781173	83513.27939	4.103790342	29.20886068	0.005919289	2.637575631	86763.10939	4.278381601	31.67917626
45 to 49	0.010871562	2.636267168	80459.46213	3.92233783	25.21447843	0.008321256	2.637449742	84232.39289	4.130507683	27.54870315
50 to 54	0.015914802	2.615602517	76202.84286	3.671132474	21.47301948	0.012235219	2.613842685	80799.30613	3.925553676	23.60332754
55 to 59	0.022437485	2.606398925	70374.52721	3.339845811	18.03248031	0.016576804	2.619653643	76005.29006	3.656351364	19.92282874
60 to 64	0.033178919	2.583120211	62902.76122	2.912437739	14.86386022	0.025049357	2.617473341	69961.59326	3.30171413	16.41371997
65 to 69	0.047572891	2.544746382	53275.91733	2.38638128	12.08397383	0.037929768	2.584211477	61721.52236	2.828272604	13.25297919
70 to 74	0.067323993	2.50501925	41978.17939	1.798514726	9.656382129	0.056191002	2.545305797	51052.43074	2.245169235	10.48279406
75 to 79	0.097163413	2.449874738	29937.48153	1.201216484	7.543069888	0.084913381	2.492818242	38528.9469	1.591178016	8.069531946
80 to 84	0.139034425	2.36820781	18340.60625	0.672740274	5.781629971	0.129439209	2.407420805	25155.15438	0.944800252	6.054216711
85 to 89	0.204781986	2.244927017	9053.049575	0.290121799	4.311484415	0.19623432	2.276343213	13075.43831	0.427985671	4.464076909
90 to 94	0.296494986	2.048517701	3148.933654	0.084190727	3.22204989	0.285151334	2.08032191	4774.869084	0.130990475	3.328214475
95 plus	0.377583284	2.648875621	664.6665135	0.017663909	2.648875621	0.372081182	2.689256203	1077.178868	0.029183547	2.689256203

Table 15: Burundi 2100 life table, by age and sex. mx=mortality rate, ax=mean person-years lived in an age interval among those who die in that age interval, lx=number of persons left alive at age x, nLx=person-years lived between age x and x+n, ex=life expectancy at age x.

Age Group	Male					Female				
	mx	ax	lx	nLx	ex	mx	ax	lx	nLx	ex
Early Neonatal	0.235940809	0.00958181	100000	0.019134795	75.44871206	0.208385539	0.009582654	100000	0.019139836	79.28870029
Late Neonatal	0.030548802	0.028758696	99549.10185	0.057224648	75.76146317	0.030942067	0.028758588	99601.53665	0.057254117	79.57722282
Post Neonatal	0.004385058	0.461332329	99374.76001	0.915667836	75.83309299	0.00407641	0.461354254	99424.75961	0.91625691	79.65716601
1 to 4	0.001186521	1.998417976	98975.09662	3.949686477	75.2054142	0.001168677	1.998441767	99052.62183	3.95290441	79.02220097
5 to 9	0.000336826	2.49929828	98509.8725	4.92137405	71.53838742	0.00035559	2.499259187	98593.21479	4.925311684	75.37103204
10 to 14	0.000343355	2.883487986	98345.19487	4.913756756	66.64873659	0.000310702	2.606567613	98419.37084	4.917320191	70.49715449
15 to 19	0.001016958	2.716732145	98177.81302	4.897915997	61.75245754	0.000495296	2.599716387	98268.06303	4.907591549	65.5998866
20 to 24	0.00170006	2.572230759	97685.98739	4.864724701	57.03622797	0.000507743	2.592251815	98027.94337	4.895377753	60.75171859
25 to 29	0.001537138	2.566592689	96884.53215	4.82634681	52.47133996	0.000608346	2.617420196	97782.69969	4.882065065	55.89573462
30 to 34	0.001697517	2.567406254	96171.08465	4.78921913	47.83927731	0.00078761	2.599952508	97488.93359	4.865302437	51.05406985
35 to 39	0.001745958	2.592752709	95390.56228	4.750004594	43.21131011	0.000950217	2.637259616	97110.0446	4.844707656	46.24003027
40 to 44	0.002178031	2.666595234	94591.69563	4.706126345	38.55688226	0.001395414	2.693391821	96655.11963	4.817376996	41.44043543
45 to 49	0.003273457	2.6798963	93596.06325	4.645139478	33.93740076	0.002237302	2.714372064	95991.2845	4.775331516	36.69983087
50 to 54	0.004915072	2.691664911	92109.65746	4.554655578	29.43560202	0.003640341	2.671554228	94938.36667	4.707503976	32.06287843
55 to 59	0.007904746	2.697026277	89915.72408	4.416892526	25.07848641	0.005307304	2.682883264	93252.15622	4.606928837	27.57591757
60 to 64	0.012994548	2.666188802	86499.21958	4.200451672	20.94823474	0.008529954	2.692598278	90860.56416	4.457409968	23.20721741
65 to 69	0.020012351	2.643388789	81176.19746	3.881059978	17.13031804	0.013981029	2.65626561	87166.99999	4.224973424	19.04646247
70 to 74	0.031353254	2.64334403	73647.89469	3.438156572	13.59733351	0.021270612	2.707708491	81496.18255	3.895177702	15.15231263
75 to 79	0.054267348	2.591508308	63305.18901	2.817740478	10.38397613	0.042164154	2.679451044	73706.07476	3.381735918	11.43727137
80 to 84	0.087512245	2.506740677	48898.73611	2.036089487	7.715139949	0.081108969	2.576877793	60695.8127	2.590565645	8.308470331
85 to 89	0.147154393	2.391314156	32487.70231	1.200784348	5.476289089	0.136848864	2.438008407	42380.87209	1.624786968	5.903967264
90 to 94	0.24145559	2.160266428	16148.04942	0.491931893	3.847971928	0.222777207	2.219225401	22939.4982	0.741941721	4.169579927
95 plus	0.334201422	3.007773759	4953.814668	0.154627915	3.007773759	0.316728707	3.2102166	8169.233843	0.281736012	3.2102166

Table 15: Comoros 2017 life table, by age and sex. mx=mortality rate, ax=mean person-years lived in an age interval among those who die in that age interval, lx=number of persons left alive at age x, nLx=person-years lived between age x and x+n, ex=life expectancy at age x.

Age Group	Male					Female				
	mx	ax	lx	nLx	ex	mx	ax	lx	nLx	ex
Early Neonatal	1.063832048	0.009556435	100000	0.018983811	67.74281885	0.817429482	0.009563987	100000	0.019028559	70.66950193
Late Neonatal	0.065339108	0.0287491	97981.12005	0.056266948	69.11850196	0.054054272	0.028752212	98444.84521	0.056551553	71.76589658
Post Neonatal	0.013235399	0.460703617	97613.74675	0.895776402	69.32105582	0.012768352	0.460736795	98139.28784	0.900790789	71.93168782
1 to 4	0.001805735	1.997592356	96429.32738	3.843293576	69.24422122	0.002167182	1.997110428	96989.81177	3.862838395	71.85563234
5 to 9	0.00067273	2.49859848	95736.21055	4.778772984	65.73174373	0.000619559	2.498709253	96153.3429	4.800230892	68.46376807
10 to 14	0.000561766	2.634633678	95414.89023	4.764414214	60.94481927	0.000464943	2.556342097	95856.04702	4.787364627	63.66832667
15 to 19	0.001039856	2.694633723	95147.32801	4.745992235	56.10878529	0.000748977	2.65061292	95633.52817	4.773279443	58.81056302
20 to 24	0.001548912	2.585291827	94653.80387	4.715057779	51.38690834	0.00101235	2.601336449	95276.11361	4.752269989	54.02126273
25 to 29	0.001696035	2.550094453	93923.55143	4.67675014	46.76572178	0.001252426	2.611203134	94795.16353	4.725625127	49.28202386
30 to 34	0.001988158	2.59567322	93130.55702	4.634385531	42.1416	0.001699887	2.644693782	94203.48017	4.691397196	44.57482321
35 to 39	0.002657327	2.638170201	92209.53869	4.581743021	37.5359595	0.002469244	2.650258378	93406.19348	4.643386619	39.93218662
40 to 44	0.003842596	2.670059731	90992.63793	4.509288482	33.00173612	0.003562804	2.65001472	92259.9399	4.574721814	35.39443004
45 to 49	0.005981554	2.686007864	89261.08879	4.4021726	28.58858259	0.005197437	2.661383509	90630.6768	4.477153534	30.98095192
50 to 54	0.009634077	2.671836532	86630.43746	4.236598454	24.37317781	0.007925116	2.642638778	88305.35302	4.334381924	26.72391369
55 to 59	0.01502387	2.660104923	82554.35513	3.987735898	20.44215863	0.011313256	2.64752387	84874.75152	4.13389613	22.69370783
60 to 64	0.023924922	2.627697741	76573.95688	3.623465271	16.82854662	0.01748229	2.643922588	80207.35878	3.852108763	18.85669282
65 to 69	0.036157618	2.589342319	67924.3876	3.124655924	13.6352326	0.02700964	2.6185026	73491.93863	3.453318098	15.33514988
70 to 74	0.05402464	2.546083333	56660.79319	2.502654111	10.83084483	0.041137078	2.589111632	64203.90868	2.921839578	12.1733972
75 to 79	0.079291008	2.4961975	43198.54975	1.803772369	8.416560313	0.064022667	2.548349239	52245.96929	2.259921195	9.369488577
80 to 84	0.120040619	2.419347156	28973.09752	1.107775711	6.333560984	0.102281841	2.475008109	37873.47735	1.507774292	6.966510922
85 to 89	0.185133872	2.290088163	15759.88409	0.525913011	4.635284586	0.165871503	2.345576336	22586.72421	0.786446998	5.027815225
90 to 94	0.278561514	2.086840979	6081.96799	0.168287078	3.396862001	0.254851552	2.150205527	9658.014737	0.280809912	3.656258276
95 plus	0.363771524	2.749608049	1416.148422	0.039063301	2.749608049	0.346094668	2.891154204	2557.280507	0.074366501	2.891154204

Table 15: Comoros 2100 life table, by age and sex. mx=mortality rate, ax=mean person-years lived in an age interval among those who die in that age interval, lx=number of persons left alive at age x, nLx=person-years lived between age x and x+n, ex=life expectancy at age x.

Age Group	Male					Female				
	mx	ax	lx	nLx	ex	mx	ax	lx	nLx	ex
Early Neonatal	0.20180001	0.009582856	100000	0.01914103	77.69878111	0.163925449	0.009584017	100000	0.019147977	81.06585939
Late Neonatal	0.00883869	0.028764685	99613.90101	0.057297544	77.97830798	0.012210441	0.028763755	99686.2559	0.057333607	81.29920382
Post Neonatal	0.001501114	0.461537199	99563.28091	0.918619245	77.96010908	0.001725696	0.461521245	99616.29392	0.919013102	81.29820256
1 to 4	0.000239681	1.999680426	99425.49613	3.975117176	77.14305122	0.000298577	1.999601897	99457.80953	3.975941331	80.50251246
5 to 9	0.000157825	2.499671198	99330.40271	4.964568486	73.21299595	0.000159858	2.499666963	99339.30952	4.964988039	76.5943608
10 to 14	0.000203287	2.799272124	99252.37758	4.960371386	68.26684858	0.000174194	2.671745849	99260.24874	4.960979102	71.65224857
15 to 19	0.00040662	2.681558069	99152.32292	4.952947048	63.33091846	0.000271377	2.625892648	99174.34671	4.955516717	66.71110787
20 to 24	0.000525684	2.59010115	98952.43828	4.941393514	58.4512861	0.000317131	2.596653795	99040.46271	4.948257953	61.79697098
25 to 29	0.000615585	2.604953377	98694.93479	4.927522732	53.59512785	0.000409077	2.655338656	98884.24949	4.939475305	56.88973491
30 to 34	0.000808568	2.60926052	98394.64016	4.910295576	48.74926865	0.000603423	2.652328844	98683.09436	4.927181381	51.99950943
35 to 39	0.001017742	2.639276809	98001.12501	4.888395414	43.9332579	0.000835428	2.6645248	98386.8609	4.909793338	47.14717999
40 to 44	0.001477338	2.710546846	97508.13598	4.859048439	39.14072964	0.001268363	2.693267961	97978.10598	4.884652543	42.33159744
45 to 49	0.002483375	2.727286515	96795.95431	4.812713173	34.40657191	0.002022644	2.709403033	97360.67298	4.845604442	37.58063155
50 to 54	0.004159068	2.738882024	95608.79785	4.736014366	29.79605426	0.003297265	2.680819975	96384.04484	4.782682011	32.93024812
55 to 59	0.00732913	2.721404399	93651.31916	4.605951166	25.35591039	0.004927923	2.67342208	94813.09363	4.687090058	28.42638297
60 to 64	0.012446554	2.677645741	90298.671	4.388921384	21.18757047	0.00761866	2.687094157	92514.76163	4.546105822	24.05958998
65 to 69	0.019547051	2.651603474	84884.51436	4.059759104	17.35681515	0.012493067	2.663627342	89074.84547	4.328384668	19.87466449
70 to 74	0.030905164	2.633068186	77047.82613	3.59363627	13.84210865	0.018980371	2.698749601	83721.00396	4.012757966	15.9627828
75 to 79	0.050529883	2.585145281	66137.38562	2.954938725	10.68429459	0.035741479	2.65783168	76206.64051	3.520819794	12.25941652
80 to 84	0.080337412	2.508840079	51577.82899	2.162894844	7.976930127	0.062006619	2.596075182	63860.70924	2.79104144	9.10644658
85 to 89	0.139063295	2.411529374	34879.37598	1.298296265	5.638522633	0.114317315	2.496491074	47161.04152	1.851781776	6.424804757
90 to 94	0.233708702	2.175580346	17617.03667	0.539307788	3.935629371	0.199117382	2.270078448	26918.06275	0.886090529	4.476316062
95 plus	0.328099582	3.05815147	5473.603432	0.171630439	3.05815147	0.295920953	3.400630188	10009.01543	0.351220492	3.400630188

Table 15: Djibouti 2017 life table, by age and sex. mx=mortality rate, ax=mean person-years lived in an age interval among those who die in that age interval, lx=number of persons left alive at age x, nLx=person-years lived between age x and x+n, ex=life expectancy at age x.

Age Group	Male					Female				
	mx	ax	lx	nLx	ex	mx	ax	lx	nLx	ex
Early Neonatal	0.624099474	0.009569913	100000	0.019063786	67.10727083	0.482017898	0.009574267	100000	0.019089721	69.81539123
Late Neonatal	0.054167784	0.028752181	98810.52891	0.056761446	67.89159682	0.045710062	0.028754514	99079.97892	0.056930051	70.44121906
Post Neonatal	0.012339578	0.460767255	98503.23647	0.904311037	68.04481662	0.011606983	0.460819296	98819.83898	0.90752231	70.56821627
1 to 4	0.002515372	1.996646177	97388.29819	3.876022166	67.89220819	0.002350431	1.996866098	97767.05015	3.892369712	70.39679705
5 to 9	0.000773615	2.498388302	96414.4999	4.811418634	64.55570082	0.000679378	2.498584629	96852.90659	4.834434195	67.04018233
10 to 14	0.000679454	2.64926176	96042.4872	4.794469943	59.79582985	0.000555402	2.595760186	96524.64895	4.819796905	62.25924352
15 to 19	0.00126844	2.658222595	95716.90834	4.771685667	54.99000259	0.000938462	2.631731698	96257.04175	4.802189235	57.42494296
20 to 24	0.001663356	2.587137897	95112.06962	4.736621139	50.32229646	0.001163141	2.643201033	95806.55824	4.777255506	52.68215111
25 to 29	0.001998595	2.598267936	94325.10634	4.693760039	45.71956937	0.001769764	2.694668473	95251.31828	4.743228117	47.97294183
30 to 34	0.002658334	2.644438129	93388.23252	4.64038876	41.1509944	0.002852955	2.687648977	94412.71006	4.68969035	43.37379317
35 to 39	0.003919921	2.64092713	92156.51673	4.565668915	36.66430118	0.004395429	2.631088494	93076.31413	4.605893526	38.95637729
40 to 44	0.005469807	2.624956225	90370.029	4.460683686	32.33505955	0.00578707	2.586827982	91054.62828	4.490137851	34.76055267
45 to 49	0.007534125	2.630614352	87935.78262	4.319899739	28.15492364	0.007157253	2.583148322	88460.64877	4.348019095	30.70023316
50 to 54	0.010861817	2.634759609	84690.86483	4.128876739	24.12974553	0.009237631	2.589594012	85356.11928	4.175211523	26.71794638
55 to 59	0.015912221	2.645322588	80224.51305	3.86701367	20.32311325	0.01208726	2.61940116	81514.19086	3.962299011	22.84921197
60 to 64	0.025026226	2.615506844	74103.29502	3.497647201	16.78109751	0.017933932	2.621709548	76751.85186	3.681679188	19.09869172
65 to 69	0.036532335	2.58178308	65404.07854	3.006476661	13.6656338	0.026162539	2.61990186	70198.91061	3.306082454	15.63238152
70 to 74	0.054315118	2.542092676	54500.68671	2.406688127	10.88802249	0.041172568	2.584777775	61639.71073	2.806317183	12.43928907
75 to 79	0.078471964	2.492809068	41549.85583	1.738939263	8.503025081	0.061019583	2.54262443	50230.64753	2.188447055	9.686395223
80 to 84	0.117345409	2.421283242	28042.67346	1.079216426	6.421606873	0.095745449	2.483745722	37083.49907	1.499749974	7.241580002
85 to 89	0.182150413	2.297280705	15511.34798	0.521507999	4.688962471	0.157934626	2.366116275	22989.60872	0.816524269	5.203950901
90 to 94	0.275790026	2.092721472	6100.521212	0.169916611	3.425690856	0.246668874	2.169078005	10325.94176	0.306247441	3.758413036
95 plus	0.361615552	2.766169653	1447.500126	0.040233172	2.766169653	0.338903758	2.95421232	2887.475428	0.086276555	2.95421232

Table 15: Djibouti 2100 life table, by age and sex. mx=mortality rate, ax=mean person-years lived in an age interval among those who die in that age interval, lx=number of persons left alive at age x, nLx=person-years lived between age x and x+n, ex=life expectancy at age x.										
Age Group	Male					Female				
	mx	ax	lx	nLx	ex	mx	ax	lx	nLx	ex
Early Neonatal	0.118813053	0.009585399	100000	0.01915626	77.59847665	0.103122925	0.00958588	100000	0.019159138	81.15433409
Late Neonatal	0.008817534	0.028764691	99772.5658	0.057388839	77.7557452	0.010776279	0.028764151	99802.54033	0.057402848	81.29506371
Post Neonatal	0.001827076	0.461514043	99721.9771	0.919944868	77.73753209	0.001759669	0.461518832	99740.70259	0.920146251	81.28774415
1 to 4	0.000286772	1.999617638	99553.98545	3.979878321	76.94431407	0.000252768	1.999662976	99578.87298	3.981143558	80.49523926
5 to 9	0.000146378	2.499695045	99439.96531	4.970179934	73.02990853	0.000141574	2.499705054	99478.33232	4.972157495	76.57419322
10 to 14	0.000189997	2.833128453	99367.2421	4.966307959	68.08136088	0.00015761	2.685940685	99407.97727	4.968578174	71.62639778
15 to 19	0.000441112	2.672711075	99272.91878	4.958554071	63.14322042	0.000273562	2.647730493	99329.70251	4.963297035	66.68055833
20 to 24	0.000553567	2.606676907	99054.30568	4.946161676	58.27627222	0.000356649	2.690475509	99193.98118	4.955641862	61.76789802
25 to 29	0.000722406	2.61763264	98780.72169	4.930576126	53.42989084	0.00062373	2.696486926	99017.33403	4.943772204	56.87293461
30 to 34	0.000986117	2.612510085	98424.83259	4.909668723	48.61286047	0.00095149	2.655570919	98709.30145	4.924396695	52.04133594
35 to 39	0.001236248	2.61579506	97941.35953	4.882693796	43.83890659	0.001243072	2.63139456	98241.6221	4.897575533	47.27581849
40 to 44	0.001684798	2.688550358	97338.78878	4.848042248	39.09265364	0.0016352	2.663585155	97634.25867	4.863078258	42.55287918
45 to 49	0.002737951	2.724787342	96524.0732	4.796204645	34.39695861	0.002464045	2.682394185	96840.89272	4.814492314	37.87777094
50 to 54	0.004517452	2.731471945	95215.56711	4.712364859	29.82759966	0.003741561	2.652603484	95657.78205	4.741247729	33.30900319
55 to 59	0.007670255	2.741597444	93098.30548	4.575408003	25.4373281	0.005172809	2.655682557	93892.20271	4.638234768	28.87899156
60 to 64	0.013208311	2.668416656	89621.1381	4.348005487	21.30999973	0.007376138	2.660957666	91509.99917	4.498424855	24.55564361
65 to 69	0.019687146	2.649269348	83941.35818	4.013006734	17.56090989	0.011528199	2.683884218	88218.15378	4.296617057	20.36029197
70 to 74	0.030078737	2.624392345	76171.51222	3.557184341	14.08234582	0.017939232	2.706778082	83329.85522	4.003297003	16.38747189
75 to 79	0.048120657	2.583688404	65602.94453	2.943453435	10.93110554	0.033565055	2.660297338	76237.93042	3.538408047	12.65268886
80 to 84	0.075048709	2.511717041	51678.09608	2.184604558	8.19112361	0.056705424	2.595642103	64580.49797	2.851022445	9.452083441
85 to 89	0.132855241	2.427814823	35624.367	1.336174113	5.770627297	0.106985528	2.520229351	48873.85123	1.946365215	6.660328735
90 to 94	0.227706321	2.187798144	18301.18584	0.562820892	4.006293907	0.190806754	2.28617614	28811.62424	0.961648903	4.616656953
95 plus	0.323336109	3.098607087	5748.762837	0.180696756	3.098607087	0.288291169	3.488301806	11113.39552	0.3982267	3.488301806

Table 15: Eritrea 2017 life table, by age and sex. mx=mortality rate, ax=mean person-years lived in an age interval among those who die in that age interval, lx=number of persons left alive at age x, nLx=person-years lived between age x and x+n, ex=life expectancy at age x.

Age Group	Male					Female				
	mx	ax	lx	nLx	ex	mx	ax	lx	nLx	ex
Early Neonatal	1.108857458	0.009555055	100000	0.018975654	58.11004086	0.723371737	0.00956687	100000	0.019045685	64.74654743
Late Neonatal	0.103901755	0.028738462	97896.68249	0.056156243	59.33725568	0.068330211	0.028748274	98622.55804	0.056630404	65.63044402
Post Neonatal	0.021342286	0.460127726	97313.71485	0.889701446	59.63483223	0.019991165	0.460223705	98235.77201	0.898684979	65.83102505
1 to 4	0.005737585	1.99234996	95417.36639	3.773307997	59.88804128	0.004840864	1.993545557	96440.5302	3.820553077	66.12450427
5 to 9	0.001117127	2.497672654	93256.54024	4.649833651	57.2318305	0.000757711	2.498421435	94593.22384	4.720716008	63.37797569
10 to 14	0.000958454	2.740506931	92737.29383	4.626847799	52.53771096	0.000607751	2.667627431	94235.64368	4.70512314	58.60867812
15 to 19	0.002244724	2.726416944	92294.02733	4.591285195	47.77653925	0.001264253	2.667388645	93949.7351	4.683685599	53.77854279
20 to 24	0.003464684	2.610457861	91263.69145	4.525745511	43.28403078	0.001650301	2.602667253	93357.59298	4.64949034	49.10123521
25 to 29	0.004214408	2.589051362	89696.15678	4.439737839	38.99225935	0.002107102	2.61934351	92590.53367	4.606429469	44.48455996
30 to 34	0.00548244	2.616394211	87826.15422	4.334711238	34.76398092	0.002904523	2.659789985	91620.54839	4.550108033	39.92568274
35 to 39	0.007615746	2.640057539	85452.18877	4.197268459	30.65299995	0.004417393	2.664376575	90300.3285	4.46893023	35.4681588
40 to 44	0.011274743	2.622751138	82260.90768	4.005889034	26.735667	0.006574992	2.631260288	88329.01015	4.348808739	31.19768817
45 to 49	0.015591503	2.592357087	77755.23907	3.747480373	23.12894492	0.009005222	2.60161419	85474.34358	4.183527147	27.14911695
50 to 54	0.020694477	2.574675037	71930.9826	3.425237254	19.78923721	0.011794646	2.598329791	81715.07515	3.973459429	23.27556166
55 to 59	0.027523995	2.570915964	64869.39486	3.041003545	16.66257542	0.016100147	2.616512309	77040.4025	3.710080331	19.52804175
60 to 64	0.038351328	2.594437528	56535.78588	2.589075566	13.74116485	0.023809428	2.693640423	71086.33998	3.369937068	15.94237413
65 to 69	0.063954505	2.511778426	46654.66556	2.014139065	11.10580626	0.047458511	2.564224508	63094.01851	2.829262923	12.61902318
70 to 74	0.075228017	2.427728874	33850.20215	1.419669042	9.367174736	0.055808789	2.500641506	49734.4652	2.184359557	10.32057438
75 to 79	0.099781693	2.415293812	23240.74281	0.925495026	7.549700151	0.087493562	2.501505933	37637.74532	1.547477846	7.840670727
80 to 84	0.134553951	2.348617169	14086.47876	0.520950347	5.910373972	0.138257335	2.404922306	24252.13067	0.896050507	5.808699406
85 to 89	0.199574522	2.256828801	7166.065784	0.232641325	4.396403762	0.205937048	2.256532344	12044.55663	0.387093657	4.30912337
90 to 94	0.291751566	2.058661267	2578.41943	0.069736358	3.26792737	0.294630089	2.05886672	4190.255329	0.113073469	3.237246027
95 plus	0.373934353	2.675322024	562.7861143	0.015152891	2.675322024	0.380046275	2.633317943	902.9090208	0.024019792	2.633317943

Table 15: Eritrea 2100 life table, by age and sex. mx=mortality rate, ax=mean person-years lived in an age interval among those who die in that age interval, lx=number of persons left alive at age x, nLx=person-years lived between age x and x+n, ex=life expectancy at age x.

Age Group	Male					Female				
	mx	ax	lx	nLx	ex	mx	ax	lx	nLx	ex
Early Neonatal	0.20060165	0.009582893	100000	0.019141284	71.85733431	0.153787719	0.009584328	100000	0.019149854	77.21184756
Late Neonatal	0.01664099	0.028762533	99616.72831	0.057286342	72.10955982	0.017383696	0.028762328	99705.89641	0.057336391	77.41572977
Post Neonatal	0.003196971	0.461416728	99521.53883	0.917518566	72.11944869	0.003399545	0.461402338	99606.3546	0.918214403	77.43404888
1 to 4	0.000710311	1.999052921	99229.04293	3.963563432	71.40259787	0.000652741	1.999129683	99294.98389	3.96665662	76.74718688
5 to 9	0.000305201	2.499364166	98949.5291	4.943730143	67.5871054	0.000239438	2.499501172	99038.30139	4.948989698	72.93233613
10 to 14	0.000347289	2.900496161	98799.79384	4.936444619	62.67996687	0.000241438	2.711377939	98921.40559	4.943345384	68.01327692
15 to 19	0.001004953	2.722242753	98630.04648	4.920557355	57.7771117	0.000448085	2.653499	98804.06272	4.935056992	63.08928351
20 to 24	0.001581124	2.613395754	98144.97785	4.88954754	53.03293973	0.00052756	2.658155769	98586.75912	4.9232727	58.22070324
25 to 29	0.001809417	2.631904006	97407.99206	4.850362447	48.39641092	0.000759984	2.676765971	98331.5456	4.907947109	53.36396317
30 to 34	0.002403601	2.630765595	96575.74315	4.802254904	43.78553268	0.001116582	2.655246257	97962.62714	4.88538477	48.5538929
35 to 39	0.002989418	2.635857197	95469.60523	4.740736118	39.26066096	0.001532315	2.636481957	97422.10042	4.853640007	43.8071131
40 to 44	0.004035174	2.626925933	94099.18266	4.661542036	34.79138875	0.002130746	2.662800182	96684.06686	4.810602076	39.11929578
45 to 49	0.005732938	2.664125561	92265.1628	4.553560437	30.42913251	0.003429053	2.683523916	95668.47288	4.745947522	34.49854503
50 to 54	0.008500127	2.680087936	89726.70844	4.401121495	26.19777373	0.005002758	2.679119176	94064.4607	4.649499064	30.02268791
55 to 59	0.013186866	2.679803026	86086.13346	4.179551385	22.17988431	0.007175957	2.690848093	91787.23145	4.515282367	25.68184139
60 to 64	0.02059949	2.700838361	80763.46415	3.860722798	18.45534504	0.01066768	2.795452172	88645.32608	4.332082488	21.48097553
65 to 69	0.036432884	2.583175548	73133.39545	3.372017173	15.10485712	0.021979504	2.665365462	84213.61682	4.012216512	17.44433235
70 to 74	0.042290503	2.523773265	61426.09833	2.787449215	12.54639137	0.0247055	2.643437443	75926.82158	3.592080978	14.08131966
75 to 79	0.062893891	2.517222683	49941.71199	2.167882399	9.891077433	0.048830976	2.639738791	67251.03854	3.024419061	10.56905396
80 to 84	0.087017044	2.448997299	36672.5155	1.510141232	7.611544409	0.086086714	2.546287961	52938.36183	2.201940836	7.740065032
85 to 89	0.147167632	2.387925028	23975.30768	0.874438329	5.418834217	0.146021058	2.40068462	34793.02115	1.278627915	5.518210927
90 to 94	0.242127297	2.161124741	11521.91962	0.345377865	3.816872494	0.233889683	2.197452521	16994.99486	0.52317359	3.941690368
95 plus	0.334972194	2.990244893	3367.488037	0.102387192	2.990244893	0.327418737	3.067873104	5272.444867	0.166981222	3.067873104

Table 15: Ethiopia 2017 life table, by age and sex. mx=mortality rate, ax=mean person-years lived in an age interval among those who die in that age interval, lx=number of persons left alive at age x, nLx=person-years lived between age x and x+n, ex=life expectancy at age x.

Age Group	Male					Female				
	mx	ax	lx	nLx	ex	mx	ax	lx	nLx	ex
Early Neonatal	1.181803682	0.009552819	100000	0.018962388	66.75894926	0.804426619	0.009564386	100000	0.019030909	70.75467828
Late Neonatal	0.118439417	0.028734452	97759.11657	0.056053788	68.26965621	0.067522101	0.028748497	98469.13716	0.056543579	71.83524693
Post Neonatal	0.019062154	0.460289699	97095.28573	0.888627079	68.67867825	0.017201982	0.46042184	98087.3622	0.898475179	72.05718748
1 to 4	0.003966035	1.994711975	95401.61979	3.785960653	68.96658535	0.003611695	1.995184423	96541.9272	3.8339195	72.28003838
5 to 9	0.000889106	2.498147697	93900.39529	4.684600623	66.03751649	0.000688422	2.498565788	95157.38967	4.749690652	69.30278178
10 to 14	0.000713971	2.608571232	93483.93871	4.666229804	61.32064992	0.000568133	2.597636364	94830.42425	4.735058732	64.53307613
15 to 19	0.001270247	2.659439292	93150.8026	4.643734418	56.53063101	0.000960461	2.6256014	94561.41647	4.71731347	59.70923654
20 to 24	0.001702811	2.565189592	92560.93571	4.608938668	51.87385202	0.00115579	2.568836562	94108.34823	4.692233263	54.98398015
25 to 29	0.001829089	2.547134466	91776.13881	4.568312662	47.29536725	0.001355927	2.592484164	93566.0515	4.663081581	50.28767513
30 to 34	0.002153246	2.593437926	90940.60535	4.523592108	42.70637126	0.001778881	2.638039981	92933.81405	4.627249923	45.61204698
35 to 39	0.002848392	2.628910023	89966.66057	4.468160053	38.14048729	0.002572487	2.636371289	92110.74483	4.577704717	40.99589971
40 to 44	0.004010084	2.658788606	88694.10618	4.393463329	33.64980836	0.003534206	2.632973823	90933.25092	4.508946603	36.49241343
45 to 49	0.006103618	2.663395911	86932.5368	4.285519597	29.27750145	0.004970965	2.639392565	89339.8666	4.415190837	32.09600633
50 to 54	0.009242614	2.655737777	84317.30034	4.126477062	25.10255736	0.007147244	2.627056216	87145.4134	4.284618026	27.8373938
55 to 59	0.01402655	2.655308637	80504.37019	3.897092227	21.16510511	0.009886368	2.648286556	84083.78756	4.108690368	23.75485345
60 to 64	0.022123293	2.625173857	75040.19045	3.564805536	17.51231124	0.015418638	2.645500508	80023.09335	3.861046166	19.82536917
65 to 69	0.032780423	2.594375492	67157.71911	3.112599126	14.25914472	0.023422627	2.631159398	74072.66155	3.509052629	16.20491656
70 to 74	0.049184776	2.558894774	56961.79684	2.543044366	11.34696023	0.036768082	2.603059638	65858.8666	3.026453942	12.89741879
75 to 79	0.073026027	2.511368598	44465.93657	1.881748085	8.817331895	0.057122977	2.559452831	54741.5107	2.402537348	9.988202332
80 to 84	0.110722045	2.439538629	30741.51937	1.197955668	6.634781896	0.089818932	2.49596544	41034.35109	1.675496737	7.470952778
85 to 89	0.175074981	2.314257195	17496.04939	0.595295283	4.814865309	0.150977625	2.383323768	26009.40962	0.93267646	5.348217072
90 to 94	0.269234127	2.106684331	7087.470529	0.199302773	3.493310418	0.239595027	2.185533911	11951.59793	0.357149978	3.841997747
95 plus	0.356521982	2.805022254	1726.946719	0.048473958	2.805022254	0.332741617	3.00571769	3407.017142	0.102517677	3.00571769

Table 15: Ethiopia 2100 life table, by age and sex. mx=mortality rate, ax=mean person-years lived in an age interval among those who die in that age interval, lx=number of persons left alive at age x, nLx=person-years lived between age x and x+n, ex=life expectancy at age x.

Age Group	Male					Female				
	mx	ax	lx	nLx	ex	mx	ax	lx	nLx	ex
Early Neonatal	0.223626087	0.009582187	100000	0.019137023	80.07844836	0.162275536	0.009584067	100000	0.019148274	84.95908525
Late Neonatal	0.024147936	0.028760462	99572.1583	0.057248318	80.40275183	0.01555027	0.028762834	99689.32055	0.057329853	85.20423251
Post Neonatal	0.001971661	0.461503772	99433.93518	0.917226272	80.45684532	0.002086639	0.461495604	99600.18369	0.918711078	85.22279758
1 to 4	0.000285523	1.999619303	99253.11829	3.967859339	79.67911941	0.000264013	1.999647982	99408.51172	3.974242449	84.46269352
5 to 9	0.000165488	2.499655234	99139.87421	4.954943994	75.76749975	0.000132918	2.499723088	99303.63446	4.963533046	80.54951698
10 to 14	0.000183202	2.704916581	99057.89819	4.950822702	70.82797407	0.000140951	2.612255244	99237.69642	4.960213533	75.60127827
15 to 19	0.000355008	2.672874589	98967.22612	4.944301685	65.89022603	0.000206399	2.572118258	99167.82133	4.955907328	70.65264007
20 to 24	0.000501021	2.57773313	98791.84493	4.933612563	61.00199306	0.000210908	2.555485255	99065.60015	4.950724563	65.72280547
25 to 29	0.000539369	2.565487954	98545.21665	4.92078445	56.1477027	0.000258257	2.620632613	98961.26546	4.945023322	60.7893462
30 to 34	0.000641695	2.572484617	98280.54421	4.906377708	51.29184712	0.000358013	2.631774099	98833.64132	4.937489947	55.8643776
35 to 39	0.000740538	2.599064956	97966.51037	4.889630346	46.44796771	0.000477577	2.63416087	98656.99349	4.927282701	50.95954827
40 to 44	0.000979429	2.685623291	97605.19332	4.869226945	41.61021	0.000665243	2.683710384	98421.81262	4.913518259	46.07480412
45 to 49	0.001612821	2.749183505	97129.03765	4.838891418	36.8008112	0.001064549	2.728812527	98095.14182	4.892918051	41.21898463
50 to 54	0.002930242	2.767210778	96349.55878	4.786163613	32.07553496	0.001833159	2.702836366	97574.64999	4.85826667	36.42375413
55 to 59	0.005466345	2.753851764	94948.67977	4.68980528	27.50657279	0.002866395	2.71213632	96684.81644	4.802675999	31.73329713
60 to 64	0.009875426	2.685609116	92389.52903	4.516348802	23.19020595	0.004753221	2.695644191	95309.92024	4.713913273	27.15105197
65 to 69	0.01524312	2.653223251	87940.62097	4.245457474	19.22428328	0.007594577	2.681072784	93072.44711	4.573092274	22.73711198
70 to 74	0.023498922	2.644135096	81495.84534	3.861825419	15.53225005	0.0118669	2.732650707	89607.58803	4.363104108	18.51074338
75 to 79	0.038197962	2.603174164	72464.94528	3.321295062	12.13507922	0.022812424	2.690382724	84446.24487	4.011573315	14.47242082
80 to 84	0.058750552	2.542117319	59873.45603	2.620181534	9.133962282	0.038786512	2.653524733	75337.40896	3.454286576	10.8947257
85 to 89	0.111657113	2.490231725	44681.74216	1.752042973	6.370465796	0.080820042	2.613700984	62024.72079	2.60350368	7.658882464
90 to 94	0.205345413	2.223565752	25475.21086	0.816514375	4.332642019	0.159489956	2.337525995	41179.21173	1.449674733	5.222677374
95 plus	0.305002765	3.285330941	8987.96981	0.298751068	3.285330941	0.258994748	3.868888986	18288.02019	0.712952222	3.868888986

Table 15: Kenya 2017 life table, by age and sex. mx=mortality rate, ax=mean person-years lived in an age interval among those who die in that age interval, lx=number of persons left alive at age x, nLx=person-years lived between age x and x+n, ex=life expectancy at age x.

Age Group	Male					Female				
	mx	ax	lx	nLx	ex	mx	ax	lx	nLx	ex
Early Neonatal	0.922802933	0.009560757	100000	0.019009377	63.31319349	0.670053625	0.009568504	100000	0.019055386	68.86061159
Late Neonatal	0.073658025	0.028746805	98245.81855	0.056405389	64.42428969	0.077087584	0.028745859	98723.19117	0.056673873	69.73190177
Post Neonatal	0.017381863	0.460409062	97830.35239	0.89604632	64.64023085	0.014769761	0.460594619	98286.30884	0.901305984	69.98419839
1 to 4	0.002645945	1.99647208	96272.87338	3.830608361	64.75520436	0.002573177	1.996569104	96955.10866	3.85831422	70.01546354
5 to 9	0.000868195	2.49819126	95259.33345	4.752643717	61.42294617	0.000668224	2.498607867	95962.30598	4.790108715	66.71917473
10 to 14	0.000797912	2.709905145	94846.71399	4.733685049	56.67927886	0.000586017	2.652091888	95642.22094	4.775539574	61.9340936
15 to 19	0.001684844	2.693652645	94469.0111	4.705167386	51.8950492	0.001101781	2.693075553	95362.36846	4.756030756	57.10806386
20 to 24	0.002393431	2.625556403	93676.26934	4.657346837	47.3113794	0.001621151	2.698756612	94838.36066	4.724294804	52.40868869
25 to 29	0.003189453	2.626495085	92561.58653	4.59330907	42.84943202	0.002685496	2.671074137	94072.49494	4.674389652	47.8133219
30 to 34	0.004447434	2.62189435	91096.63354	4.507163048	38.49616399	0.003906234	2.617327185	92817.24824	4.598066423	43.42373409
35 to 39	0.006004489	2.61489897	89092.23813	4.391720298	34.3031675	0.005037664	2.587069974	91021.26776	4.496409554	39.22883401
40 to 44	0.008155755	2.600606355	86455.48213	4.239812771	30.26952533	0.006272169	2.56710771	88756.32996	4.371119992	35.16380959
45 to 49	0.01064171	2.59041235	82997.9868	4.046158999	26.42206112	0.00745175	2.562634799	86014.93619	4.224034641	31.20259525
50 to 54	0.013992646	2.590821952	78692.72801	3.806339467	22.72579656	0.009098466	2.563455892	82867.59587	4.053528059	27.2901936
55 to 59	0.018880933	2.606168273	73367.49106	3.509767004	19.1872083	0.011074141	2.602332058	79179.95899	3.85661289	23.44160539
60 to 64	0.027819081	2.598404434	66741.91686	3.128143051	15.83323504	0.016034583	2.629152108	74909.80878	3.608344494	19.62932303
65 to 69	0.040644554	2.573329191	58041.41333	2.641584274	12.81722464	0.02403181	2.625419499	69125.19795	3.269722452	16.05173377
70 to 74	0.060668612	2.531419095	47307.13707	2.057319262	10.14178893	0.037406438	2.600194396	61269.77163	2.811222562	12.77297205
75 to 79	0.088692112	2.475373032	34828.78666	1.422924186	7.868719575	0.058107524	2.558416781	50758.01881	2.222719435	9.879741038
80 to 84	0.133306681	2.396908584	22212.34943	0.82458737	5.932694208	0.091917963	2.493819928	37849.24826	1.538345683	7.377220765
85 to 89	0.199288856	2.257030583	11224.02306	0.362902035	4.395397014	0.153575234	2.376371386	23719.37149	0.845558704	5.287773156
90 to 94	0.291549294	2.059095615	3994.365515	0.107541444	3.26762703	0.242299742	2.179313252	10743.74756	0.319202309	3.806948313
95 plus	0.373805854	2.675224072	859.8997736	0.023008775	2.675224072	0.335134216	2.984062161	3014.833976	0.090011425	2.984062161

Table 15: Kenya 2100 life table, by age and sex. mx=mortality rate, ax=mean person-years lived in an age interval among those who die in that age interval, lx=number of persons left alive at age x, nLx=person-years lived between age x and x+n, ex=life expectancy at age x.

Age Group	Male					Female				
	mx	ax	lx	nLx	ex	mx	ax	lx	nLx	ex
Early Neonatal	0.25889997	0.009581106	100000	0.019130556	73.71372072	0.201456817	0.009582866	100000	0.019141086	81.18989653
Late Neonatal	0.017403581	0.028762323	99504.82027	0.057220702	74.06014677	0.027636997	0.0287595	99614.45559	0.057266897	81.48425372
Post Neonatal	0.003451109	0.461398675	99405.2588	0.91633611	74.07654918	0.003547982	0.461391793	99456.23271	0.916764998	81.55611249
1 to 4	0.000351749	1.999531002	99089.08778	3.960777874	73.3876124	0.000369485	1.999507353	99131.02823	3.962313814	80.89831769
5 to 9	0.000226345	2.499528447	98949.84208	4.944694199	69.48764842	0.000155825	2.499675365	98984.70252	4.94730831	77.01449051
10 to 14	0.000246477	2.828673238	98837.94737	4.939254522	64.56336107	0.000155984	2.698606759	98907.64037	4.943604111	72.07238303
15 to 19	0.000616838	2.672972507	98716.23203	4.928743865	59.6393648	0.000301356	2.617277253	98830.54817	4.937987751	67.12638627
20 to 24	0.00076626	2.588426379	98412.31953	4.911554663	54.81475881	0.000331435	2.566989133	98681.77525	4.930130733	62.22346097
25 to 29	0.000958323	2.608402469	98036.1677	4.890617566	50.01436472	0.000424835	2.623867079	98518.4435	4.920947947	57.32210282
30 to 34	0.001285013	2.585237644	97567.86392	4.863298061	45.24092375	0.000576028	2.626189799	98309.54384	4.908745053	52.43795498
35 to 39	0.001478493	2.639467284	96943.65972	4.830334444	40.51442066	0.000751681	2.641763519	98027.04579	4.89267903	47.58099407
40 to 44	0.002289715	2.718160144	96230.57045	4.78651903	35.7936826	0.00108672	2.687842325	97659.59641	4.870738161	42.74942927
45 to 49	0.00390478	2.705437468	95136.59487	4.714616628	31.17206046	0.001730925	2.699132691	97130.87028	4.837269048	37.96649179
50 to 54	0.00625488	2.711646905	93300.35486	4.599218915	26.72918143	0.002744565	2.700274081	96294.70409	4.784523943	33.27128586
55 to 59	0.010584284	2.697664703	90433.79482	4.414324288	22.48690428	0.004404622	2.672616988	94983.72716	4.700987342	28.69144447
60 to 64	0.01744291	2.652018751	85782.27152	4.121016409	18.55680216	0.006511345	2.68414152	92917.24315	4.576961983	24.26812541
65 to 69	0.026557394	2.630145864	78632.8492	3.700067285	14.99939163	0.010682151	2.689489749	89943.9857	4.389116658	19.97855807
70 to 74	0.042166266	2.61129967	68874.8984	3.130937401	11.75141662	0.017578699	2.73142556	85270.49624	4.100593762	15.92263355
75 to 79	0.070875836	2.547588312	55786.15031	2.380283549	8.898302959	0.035052477	2.677892737	78095.02647	3.612540128	12.13025199
80 to 84	0.112035256	2.454388044	39110.83132	1.526601809	6.619002216	0.063150923	2.603158094	65522.80401	2.849724089	8.940400706
85 to 89	0.176513638	2.311664186	22249.75907	0.758721708	4.801771923	0.116521258	2.486284147	47742.03869	1.853048086	6.303371946
90 to 94	0.270427141	2.103905285	9071.731134	0.256134286	3.486024104	0.202144926	2.266659965	26489.70103	0.858264907	4.401358045
95 plus	0.357390817	2.800730716	2239.222073	0.063316851	2.800730716	0.298891098	3.353234589	9407.69512	0.319238691	3.353234589

Table 15: Madagascar 2017 life table, by age and sex. mx=mortality rate, ax=mean person-years lived in an age interval among those who die in that age interval, lx=number of persons left alive at age x, nLx=person-years lived between age x and x+n, ex=life expectancy at age x.										
**Age Group**	**Male**					**Female**				
	**mx**	**ax**	**lx**	**nLx**	**ex**	**mx**	**ax**	**lx**	**nLx**	**ex**
Early Neonatal	1.234971217	0.00955119	100000	0.018952791	62.36223841	0.917564298	0.009560918	100000	0.019010348	64.74131154
Late Neonatal	0.113572483	0.028735794	97659.96069	0.056004843	63.83511623	0.096905828	0.028740392	98255.99568	0.05637361	65.86951574
Post Neonatal	0.030720871	0.459461507	97024.24563	0.883237369	64.1953693	0.028586559	0.459613119	97709.92884	0.890349773	66.17962122
1 to 4	0.006042174	1.99194385	94313.21877	3.727357377	65.10416634	0.006223922	1.991701528	95166.55269	3.759715698	67.0123043
5 to 9	0.000966461	2.49798654	92063.84256	4.592094528	62.64750626	0.000914208	2.4980954	92829.07649	4.630868605	64.65060137
10 to 14	0.000773835	2.622945776	91620.29883	4.572609284	57.93843495	0.000625579	2.525739839	92405.99177	4.613163588	59.93511127
15 to 19	0.001436657	2.704764495	91266.59595	4.548340491	53.15248165	0.000999752	2.669687024	92117.53958	4.595176636	55.11479979
20 to 24	0.002215256	2.59626082	90613.33337	4.506676625	48.51522769	0.001452536	2.624828031	91658.30859	4.56716728	50.3772641
25 to 29	0.00250246	2.569006411	89615.34676	4.453686747	44.02505571	0.001892789	2.61915862	90995.24899	4.52936281	45.72476801
30 to 34	0.003112166	2.605464939	88501.46573	4.392365881	39.54539134	0.002575814	2.641808139	90138.41464	4.479724885	41.13380588
35 to 39	0.004187962	2.632578333	87135.59034	4.314058541	35.12283026	0.003724767	2.645342915	88985.16964	4.410609412	36.63155255
40 to 44	0.005993322	2.647520427	85330.75908	4.207306734	30.80801809	0.005344413	2.634617511	87343.40197	4.312714782	32.26866457
45 to 49	0.008861633	2.652447163	82812.97685	4.056422681	26.66116861	0.007521103	2.644907469	85040.49833	4.178121748	28.06779936
50 to 54	0.013413788	2.637249796	79225.7443	3.839892409	22.74480174	0.011272069	2.626805891	81902.43884	3.988635582	24.03751348
55 to 59	0.019706341	2.622315839	74089.72889	3.539189901	19.13498422	0.015749164	2.628437584	77416.34223	3.731833903	20.2729703
60 to 64	0.029453521	2.592148192	67139.81823	3.135598249	15.84170348	0.023932212	2.619223703	71558.57627	3.385810873	16.71261112
65 to 69	0.0420623	2.561896775	57947.21582	2.629298413	12.94311437	0.03598198	2.594006427	63491.22824	2.923068988	13.49998623
70 to 74	0.061441233	2.516892071	46954.55442	2.03895787	10.37754927	0.054933359	2.551830574	53041.89739	2.340067923	10.64835736
75 to 79	0.085156636	2.46188531	34519.95674	1.421543592	8.220886254	0.082120243	2.497334707	40298.94287	1.674827096	8.216269265
80 to 84	0.120794076	2.39861108	22521.65176	0.858931333	6.310948182	0.126355789	2.418436133	26707.09204	1.010647962	6.14893465
85 to 89	0.185616959	2.288973086	12245.43192	0.408605387	4.627295631	0.192943271	2.28347406	14119.55035	0.465638805	4.521014289
90 to 94	0.2790025	2.085894372	4725.056591	0.130747333	3.392554495	0.281902497	2.087769715	5258.883451	0.145321013	3.361453227
95 plus	0.364111299	2.747127191	1100.422475	0.030360028	2.747127191	0.369321322	2.709698695	1210.853534	0.033100222	2.709698695

Table 15: Madagascar 2100 life table, by age and sex. mx=mortality rate, ax=mean person-years lived in an age interval among those who die in that age interval, lx=number of persons left alive at age x, nLx=person-years lived between age x and x+n, ex=life expectancy at age x.

Age Group	Male					Female				
	mx	ax	lx	nLx	ex	mx	ax	lx	nLx	ex
Early Neonatal	0.253983217	0.009581257	100000	0.019131458	76.03275987	0.216761114	0.009582397	100000	0.01913828	78.49780928
Late Neonatal	0.016405934	0.028762598	99514.20304	0.057227741	76.38304954	0.019159226	0.028761838	99585.23893	0.057264058	78.80413513
Post Neonatal	0.004228467	0.461343452	99420.34182	0.916147045	76.39718271	0.004427387	0.461329321	99475.55751	0.916571802	78.83306251
1 to 4	0.000828011	1.998895986	99033.15379	3.954779591	75.76890917	0.000930236	1.998759686	99069.97771	3.955442841	78.22854361
5 to 9	0.000122228	2.499745359	98706.04868	4.933800537	72.01088612	0.000179003	2.499627077	98702.44142	4.932916102	74.50882638
10 to 14	0.000148096	3.139340462	98645.99192	4.930767221	67.05222247	0.00016056	2.62071239	98614.21659	4.928828568	69.57287981
15 to 19	0.000403092	2.845480304	98573.25476	4.924282918	62.09870109	0.000271663	2.747519767	98535.14181	4.923763393	64.62633515
20 to 24	0.000710121	2.673564505	98375.21994	4.910660182	57.21681551	0.000500794	2.785399176	98401.50516	4.914732744	59.70957589
25 to 29	0.001002599	2.64267502	98026.99826	4.889908123	52.40849602	0.001063925	2.716043024	98155.76534	4.895992658	54.8490352
30 to 34	0.001496539	2.640284652	97538.0969	4.859784457	47.65373216	0.001664233	2.64712641	97637.65742	4.862948389	50.11808806
35 to 39	0.001995527	2.642320835	96814.61334	4.818136746	42.98461202	0.0021972	2.633406589	96836.51503	4.81695631	45.50323559
40 to 44	0.002823505	2.668401242	95860.29726	4.7617678	38.3810924	0.002956217	2.628407376	95793.24985	4.756611209	40.96522904
45 to 49	0.004198563	2.658681605	94528.04635	4.680608001	33.87935167	0.003859159	2.63186028	94410.25728	4.67807741	36.52440976
50 to 54	0.006000925	2.669811734	92581.61466	4.565650819	29.52779415	0.005117924	2.617383762	92635.69745	4.576406374	32.1727654
55 to 59	0.00938182	2.663586984	89867.85141	4.397871688	25.32492576	0.00659633	2.618630496	90327.00469	4.44707346	27.9266569
60 to 64	0.014222545	2.640881204	85790.95657	4.152381574	21.37766065	0.009037811	2.646501153	87429.81122	4.281332646	23.76268764
65 to 69	0.021197279	2.624749591	79990.97956	3.811975127	17.70689063	0.013739901	2.646484857	83607.64835	4.050860603	19.72265773
70 to 74	0.030804936	2.60958149	72137.13268	3.366537411	14.32577068	0.020445266	2.671510828	78120.29979	3.730948365	15.91616778
75 to 79	0.049555214	2.570270832	62099.62153	2.788615346	11.20715814	0.03598105	2.646115368	70620.42508	3.260634186	12.31488067
80 to 84	0.071532959	2.504161801	49121.15896	2.107838637	8.520209353	0.060340522	2.589399178	59166.55951	2.592581015	9.188973074
85 to 89	0.127139259	2.44721456	35161.37033	1.354350828	5.992204588	0.112118891	2.502735653	44006.09035	1.733329623	6.480282271
90 to 94	0.220921225	2.194580635	19281.10636	0.611288739	4.129135495	0.196783815	2.275420406	25287.59866	0.834017657	4.508434836
95 plus	0.31756358	3.168886677	6630.636606	0.219247878	3.168886677	0.293832954	3.42051305	9441.545073	0.331381584	3.42051305

Table 15: Malawi 2017 life table, by age and sex. mx=mortality rate, ax=mean person-years lived in an age interval among those who die in that age interval, lx=number of persons left alive at age x, nLx=person-years lived between age x and x+n, ex=life expectancy at age x.

Age Group	Male					Female				
	mx	ax	lx	nLx	ex	mx	ax	lx	nLx	ex
Early Neonatal	1.193067418	0.009552474	100000	0.018960367	59.78054128	0.894468268	0.009561626	100000	0.019014536	67.20625979
Late Neonatal	0.111997471	0.028736229	97738.29681	0.056052276	61.14342046	0.074779612	0.028746495	98299.35963	0.056434331	68.34917933
Post Neonatal	0.022254743	0.460062907	97110.77486	0.887467927	61.48067951	0.019952328	0.460226464	97877.42942	0.895419626	68.58611331
1 to 4	0.005739682	1.99234716	95136.90764	3.762158994	61.82353167	0.004974631	1.993367204	96091.40868	3.805683337	68.92914153
5 to 9	0.001481583	2.496913372	92979.40076	4.631794655	59.21278489	0.001171241	2.497559916	94199.08529	4.696191467	66.27431617
10 to 14	0.001386921	2.706882775	92293.23737	4.600023285	54.6341432	0.001088678	2.630702781	93649.11282	4.670400304	61.64866795
15 to 19	0.002889037	2.625022944	91655.30389	4.551531833	49.99534335	0.001864672	2.584226512	93140.69844	4.636169621	56.97072298
20 to 24	0.003177748	2.548980957	90340.45881	4.482122657	45.68433173	0.001885437	2.573645658	92276.31535	4.592819759	52.47974425
25 to 29	0.003749836	2.593963108	88916.39769	4.406108998	41.37437745	0.002587041	2.629389292	91410.64842	4.542697095	47.95190941
30 to 34	0.005061936	2.60970652	87264.95525	4.311095826	37.1074863	0.003572263	2.619228255	90236.3464	4.473727322	43.54154026
35 to 39	0.006645868	2.612401732	85084.81015	4.187820136	32.9912239	0.004674357	2.595976006	88640.0116	4.382761133	39.27881267
40 to 44	0.009098519	2.608901493	82304.55587	4.027670327	29.01732036	0.005921442	2.583905214	86592.89133	4.268607068	35.14555091
45 to 49	0.012287316	2.603108506	78643.13771	3.819756174	25.24586988	0.007439354	2.579440508	84066.04809	4.128993901	31.12232382
50 to 54	0.016868866	2.582136459	73953.44963	3.55290837	21.67968065	0.009389736	2.558787183	80994.80822	3.959037795	27.20120095
55 to 59	0.021799213	2.588580017	67968.11615	3.228923653	18.36048473	0.010900542	2.603438611	77281.16833	3.765833746	23.38228194
60 to 64	0.031708065	2.581398368	60941.19983	2.83040791	15.17949537	0.016326642	2.633250938	73183.5057	3.523346867	19.54316493
65 to 69	0.04499918	2.553661027	51984.30471	2.341939877	12.35197532	0.024403026	2.627319326	67445.75614	3.188287038	15.9793321
70 to 74	0.065541578	2.512242274	41467.646	1.783216102	9.840119861	0.038423083	2.599290649	59692.73933	2.733616601	12.7123688
75 to 79	0.093236104	2.454941262	29803.01871	1.204821606	7.712214537	0.05942751	2.54992876	49239.15737	2.150624417	9.861434431
80 to 84	0.134972395	2.379645354	18590.60759	0.687042083	5.887918539	0.091350942	2.482794757	36532.93253	1.487180951	7.412032642
85 to 89	0.200728085	2.2538384	9335.124932	0.301106943	4.373167691	0.152669693	2.379174231	23044.70917	0.82476864	5.313624778
90 to 94	0.292847406	2.056316722	3301.778431	0.08872763	3.255566868	0.2413103	2.181528999	10541.55766	0.314593087	3.821970088
95 plus	0.374798501	2.668256069	707.0354001	0.018883651	2.668256069	0.334233371	2.993383261	2995.334889	0.09005115	2.993383261

Table 15: Malawi 2100 life table, by age and sex. mx=mortality rate, ax=mean person-years lived in an age interval among those who die in that age interval, lx=number of persons left alive at age x, nLx=person-years lived between age x and x+n, ex=life expectancy at age x.

Age Group	Male					Female				
	mx	ax	lx	nLx	ex	mx	ax	lx	nLx	ex
Early Neonatal	0.219701048	0.009582307	100000	0.019137747	74.46932939	0.180597162	0.009583506	100000	0.019144916	83.58072298
Late Neonatal	0.018425181	0.028762041	99579.7089	0.057262088	74.76308703	0.015769291	0.028762773	99654.36353	0.057309393	83.84973579
Post Neonatal	0.005632217	0.461243732	99474.23806	0.916050118	74.78452094	0.005423114	0.461258587	99564.01733	0.916965268	83.86797108
1 to 4	0.000495766	1.999338978	98958.45816	3.954420326	74.24781068	0.000437666	1.999416445	99066.87904	3.959211803	83.362111
5 to 9	0.00031104	2.499352	98762.63987	4.934295886	70.39023716	0.0002485	2.499482291	98893.77319	4.941619739	79.50342113
10 to 14	0.000264303	2.947227257	98609.23809	4.927751871	65.49562438	0.000186117	2.685573158	98771.04458	4.93640602	74.59872858
15 to 19	0.000844077	2.674682744	98479.08287	4.914257844	60.57811003	0.00039432	2.564186331	98679.24137	4.929264393	69.66531322
20 to 24	0.000919307	2.578808521	98064.63466	4.892353702	55.82212555	0.000340284	2.573614288	98485.05463	4.920221049	64.79705861
25 to 29	0.001203414	2.588468256	97615.32475	4.866719248	51.06614642	0.00054126	2.578962545	98317.89484	4.909475755	59.90230946
30 to 34	0.001500733	2.560689608	97030.61233	4.83381139	46.3568906	0.000543618	2.561245643	98052.99804	4.896056439	55.05654336
35 to 39	0.001587183	2.602830407	96307.3082	4.797118663	41.68451153	0.000570353	2.592185958	97788.19061	4.882677129	50.19829654
40 to 44	0.002266909	2.703276262	95548.1749	4.752678229	36.99305792	0.000753224	2.706386217	97510.46672	4.867097101	45.33297234
45 to 49	0.003819725	2.720169526	94473.70969	4.682935614	32.37924247	0.001292436	2.736658638	97144.52526	4.842995116	40.49183022
50 to 54	0.006381395	2.707281983	92691.64132	4.567841722	27.94438991	0.002147325	2.70219398	96520.0969	4.802223704	35.73322545
55 to 59	0.010429757	2.686532634	89792.29335	4.384226106	23.75260606	0.003278343	2.691897636	95491.9655	4.738677034	31.08469002
60 to 64	0.016676348	2.64289436	85253.41452	4.102472209	19.86916359	0.005092405	2.687953964	93944.90808	4.642765788	26.54699937
65 to 69	0.024517257	2.614656891	78474.03003	3.70872367	16.35561519	0.008091824	2.696236203	91594.2831	4.496299517	22.15099125
70 to 74	0.036497867	2.594697158	69477.82531	3.196073521	13.13831558	0.01318886	2.75374842	87989.94186	4.273863062	17.93803266
75 to 79	0.056307518	2.549801234	57933.34333	2.549251761	10.24543548	0.026352115	2.681263167	82426.24815	3.887214393	13.95170311
80 to 84	0.083484162	2.488042141	43755.87214	1.813294964	7.752509289	0.043717614	2.626101548	72361.91702	3.285974232	10.50990926
85 to 89	0.143372254	2.397775106	28841.13036	1.055112669	5.49763172	0.087944651	2.588517647	58393.29683	2.423149366	7.39619021
90 to 94	0.238481948	2.168625699	13954.74283	0.418997657	3.859012917	0.167923763	2.322717029	37815.64125	1.318575815	5.064681122
95 plus	0.3320878	3.014374169	4094.059576	0.124542381	3.014374169	0.266867206	3.769816037	16406.75383	0.633535951	3.769816037

Table 15: Mozambique 2017 life table, by age and sex. mx=mortality rate, ax=mean person-years lived in an age interval among those who die in that age interval, lx=number of persons left alive at age x, nLx=person-years lived between age x and x+n, ex=life expectancy at age x.

Age Group	Male					Female				
	mx	ax	lx	nLx	ex	mx	ax	lx	nLx	ex
Early Neonatal	1.233645548	0.00955123	100000	0.01895302	54.93348215	0.905584195	0.009561285	100000	0.019012517	62.21768246
Late Neonatal	0.087462722	0.028742997	97662.27478	0.056048167	56.22783205	0.070672205	0.028747628	98278.43518	0.056428983	63.28759291
Post Neonatal	0.033842211	0.459239782	97172.24451	0.883314947	56.4535763	0.031915093	0.459376674	97879.72996	0.890531216	63.48765912
1 to 4	0.005524043	1.992634671	94184.73237	3.726105477	57.30647935	0.004839599	1.993547243	95038.76056	3.765011611	64.44859459
5 to 9	0.001893877	2.496054429	92128.19209	4.584676203	54.54203244	0.001528665	2.496815285	93217.74473	4.643124661	61.66931419
10 to 14	0.001572311	2.648383984	91260.23065	4.546202286	50.03696692	0.001157413	2.548839083	92508.14939	4.612320831	57.12323179
15 to 19	0.003057933	2.677821373	90545.66309	4.49537331	45.41087013	0.001818614	2.691502887	91974.4275	4.579533341	52.43996541
20 to 24	0.004282573	2.663678485	89171.32926	4.414436708	41.06882144	0.002907528	2.738864958	91141.75595	4.527351126	47.89370037
25 to 29	0.006686079	2.640407823	87281.48108	4.296356434	36.89903428	0.005294905	2.642955153	89826.23179	4.435984149	43.55375633
30 to 34	0.009285964	2.593831636	84411.25148	4.128364875	33.06162375	0.006760374	2.559640633	87480.87242	4.303055684	39.64927662
35 to 39	0.011638739	2.564295677	80583.10568	3.918221521	29.50768703	0.007486901	2.523215806	84578.20736	4.152028867	35.92296336
40 to 44	0.014222015	2.553618388	76030.02219	3.673849589	26.12002539	0.008112598	2.530857979	81475.07683	3.99381825	32.19490788
45 to 49	0.017346108	2.55786626	70813.08084	3.396968468	22.85447867	0.009362307	2.56872476	78237.96585	3.824896703	28.41979263
50 to 54	0.022299266	2.552858894	64929.43076	3.078775929	19.69083181	0.012230417	2.570047382	74658.58773	3.625299055	24.65450234
55 to 59	0.028076516	2.560259696	58077.76393	2.718195019	16.71068338	0.014975613	2.59981684	70229.70249	3.389921016	21.04183792
60 to 64	0.03937505	2.553689582	50468.00421	2.302334932	13.84444764	0.022177939	2.617451362	65166.26048	3.095320563	17.47000216
65 to 69	0.054627503	2.52076792	41431.9458	1.825381404	11.30916615	0.033118713	2.595823803	58326.843	2.702301824	14.20788118
70 to 74	0.076224659	2.477478585	31500.81891	1.322156328	9.085200682	0.049808978	2.558477506	49426.77265	2.205134233	11.29761969
75 to 79	0.105523338	2.420578113	21474.8633	0.845319606	7.180664066	0.07400089	2.51011577	38525.86767	1.62918425	8.775575713
80 to 84	0.148386792	2.343197033	12616.88542	0.453537999	5.542972565	0.11227853	2.438879397	26593.0495	1.035731859	6.604015022
85 to 89	0.214057338	2.224761945	5938.503716	0.18678762	4.172786433	0.177162018	2.318675317	15110.71032	0.514472735	4.805177591
90 to 94	0.304819547	2.030766763	1966.440193	0.051755865	3.146525657	0.266230645	2.123841287	6108.663854	0.173925747	3.527009396
95 plus	0.383927217	2.605158441	396.6189105	0.010366439	2.605158441	0.355935947	2.811554492	1527.608992	0.043293237	2.811554492

Table 15: Mozambique 2100 life table, by age and sex. mx=mortality rate, ax=mean person-years lived in an age interval among those who die in that age interval, lx=number of persons left alive at age x, nLx=person-years lived between age x and x+n, ex=life expectancy at age x.

Age Group	Male					Female				
	mx	ax	lx	nLx	ex	mx	ax	lx	nLx	ex
Early Neonatal	0.216536427	0.009582404	100000	0.019138322	70.71921836	0.163989385	0.009584015	100000	0.01914796	79.02172754
Late Neonatal	0.012129099	0.028763777	99585.6674	0.05727588	70.99432657	0.013047484	0.028763524	99686.04598	0.057332098	79.25102407
Post Neonatal	0.01050217	0.46089778	99516.21159	0.914381453	70.98643033	0.010400835	0.460904979	99611.25833	0.915297474	79.25288442
1 to 4	0.000405702	1.999459064	98556.1799	3.939051725	70.7500906	0.000337029	1.999550628	98659.52	3.943723123	79.08892225
5 to 9	0.000211099	2.49956021	98396.46243	4.917229655	66.8611244	0.000135815	2.499717052	98526.67231	4.924662675	75.192524
10 to 14	0.000253117	3.147391888	98292.74612	4.912258344	61.92896441	0.000128639	2.965046602	98459.84615	4.921687621	70.24170554
15 to 19	0.000931103	2.774489194	98168.52679	4.898259686	57.00331783	0.000406755	2.83141141	98396.58649	4.915541338	65.2848292
20 to 24	0.00147352	2.683412743	97712.84993	4.869100134	52.25443875	0.000820553	2.717775995	98196.89364	4.900751574	60.41093015
25 to 29	0.002305094	2.637898014	96996.58319	4.823702764	47.61726577	0.001343222	2.592715609	97795.55351	4.874111999	55.64621249
30 to 34	0.003132277	2.582760033	95887.59638	4.758294281	43.13276066	0.001449014	2.618544328	97144.47084	4.839984768	51.00069508
35 to 39	0.003392449	2.597233774	94405.09952	4.682162452	38.7658018	0.001497776	2.582434208	96449.66354	4.804941332	46.35134892
40 to 44	0.004674464	2.649505425	92827.07523	4.590995279	34.37763243	0.001777303	2.660201101	95735.50487	4.76676402	41.67901423
45 to 49	0.006784345	2.65239219	90693.40856	4.46382561	30.11828383	0.00264009	2.686556693	94890.87923	4.715656384	37.02529575
50 to 54	0.010063145	2.643729743	87680.8455	4.282971623	26.05289734	0.004127054	2.669604341	93646.60038	4.637716871	32.47748198
55 to 59	0.014694578	2.621749073	83399.94387	4.030127184	22.24375057	0.006101693	2.645180433	91736.6658	4.521889201	28.09179978
60 to 64	0.020764906	2.591979117	77529.8968	3.693607277	18.72054223	0.008585619	2.65210341	88987.48325	4.36182081	23.87074053
65 to 69	0.028294614	2.576142012	69942.6718	3.275513298	15.46387449	0.013285854	2.646925448	85260.52065	4.134597658	19.78786795
70 to 74	0.039779716	2.589781479	60806.96942	2.778856505	12.39851388	0.019747159	2.696328432	79813.39618	3.818659332	15.94601558
75 to 79	0.065258682	2.543765672	49965.08128	2.160937814	9.535069204	0.037482758	2.636252953	72366.55989	3.328338047	12.29891999
80 to 84	0.096861639	2.460281323	36235.41823	1.464253373	7.214420325	0.059201678	2.579975377	60122.42897	2.638386989	9.262103653
85 to 89	0.158794736	2.357513705	22537.05758	0.802745937	5.169869701	0.110512644	2.507905419	44941.19663	1.775367035	6.53166062
90 to 94	0.253397518	2.138631003	10233.9124	0.300616709	3.68323489	0.194965152	2.278918328	26000.224	0.860106394	4.539098389
95 plus	0.34391842	2.91369405	2827.110741	0.083913055	2.91369405	0.292165183	3.439671542	9786.973107	0.345242839	3.439671542

Table 15: Rwanda 2017 life table, by age and sex. mx=mortality rate, ax=mean person-years lived in an age interval among those who die in that age interval, lx=number of persons left alive at age x, nLx=person-years lived between age x and x+n, ex=life expectancy at age x.

Age Group	Male					Female				
	mx	ax	lx	nLx	ex	mx	ax	lx	nLx	ex
Early Neonatal	0.811899513	0.009564157	100000	0.019029556	65.80128476	0.633410587	0.009569627	100000	0.019062073	70.85431416
Late Neonatal	0.087005377	0.028743123	98455.13673	0.056503909	66.81416768	0.071862989	0.0287473	98792.66177	0.056722287	71.70084718
Post Neonatal	0.017559516	0.460396443	97963.62686	0.897196735	67.09170466	0.01532031	0.46055551	98385.09645	0.90198441	71.94024022
1 to 4	0.003851778	1.994864318	96389.12297	3.826036024	67.256673	0.003522218	1.995303726	97003.65244	3.85295116	72.03479411
5 to 9	0.000748971	2.498439644	94916.51739	4.736952784	64.26990236	0.000614376	2.49872005	95647.12258	4.775019821	69.02856079
10 to 14	0.000715949	2.669109167	94561.81678	4.720212869	59.50149652	0.000564381	2.609028616	95353.82135	4.761265373	64.23309347
15 to 19	0.001336802	2.674069267	94223.93778	4.696597176	54.70516526	0.000912836	2.631193845	95085.15235	4.744002469	59.40719118
20 to 24	0.00185458	2.57596877	93596.11459	4.658865712	50.05374555	0.001148924	2.594753496	94652.15672	4.719569055	54.66679747
25 to 29	0.002045362	2.555083745	92732.2196	4.613548295	45.49533353	0.001450716	2.610739641	94110.01149	4.689251923	49.9665573
30 to 34	0.002442795	2.600887141	91788.86485	4.562714176	40.93600536	0.001945139	2.63364166	93429.88805	4.650089271	45.31098827
35 to 39	0.003296488	2.642514558	90674.80167	4.498799855	36.40632056	0.002735306	2.63229742	92525.61407	4.596522471	40.72762154
40 to 44	0.004831102	2.655985749	89192.64063	4.409731191	31.9664846	0.003767975	2.625124554	91268.66616	4.522977484	36.25148023
45 to 49	0.007187492	2.664242246	87063.95451	4.281381176	27.68174184	0.00516174	2.63507905	89564.96887	4.424268883	31.88968246
50 to 54	0.011119822	2.648479423	83989.96363	4.092603186	23.59578817	0.007430778	2.620167018	87282.44902	4.28834478	27.65293388
55 to 59	0.016504107	2.642494111	79445.33079	3.823708119	19.79251628	0.010029091	2.640700237	84098.70238	4.107846123	23.59850667
60 to 64	0.025688062	2.617547724	73145.34366	3.446715349	16.26849775	0.015450024	2.645136163	79984.50503	3.859055163	19.67447258
65 to 69	0.038393139	2.584564558	64308.90699	2.943150932	13.14386216	0.023588049	2.635021987	74033.10796	3.506511601	16.04161603
70 to 74	0.05790885	2.540930295	53036.56008	2.322218769	10.3889289	0.037462068	2.605140098	65783.78829	3.019145588	12.72177446
75 to 79	0.08550237	2.484660131	39633.2808	1.632080229	8.046770183	0.058582125	2.560957947	54509.57788	2.385973071	9.815111214
80 to 84	0.129027667	2.404654841	25734.01519	0.965110457	6.059338727	0.093767948	2.493869615	40590.76971	1.645204903	7.307049141
85 to 89	0.194755962	2.267524264	13339.77794	0.436077778	4.470961913	0.155781107	2.370972668	25253.65623	0.897515639	5.242787231
90 to 94	0.287403759	2.067964381	4884.672287	0.132793815	3.308375736	0.244534924	2.174097553	11356.34355	0.336624261	3.780897534
95 plus	0.370608978	2.698691137	1081.496986	0.02925667	2.698691137	0.337077377	2.968018315	3168.254106	0.094406349	2.968018315

Table 15: Rwanda 2100 life table, by age and sex. mx=mortality rate, ax=mean person-years lived in an age interval among those who die in that age interval, lx=number of persons left alive at age x, nLx=person-years lived between age x and x+n, ex=life expectancy at age x.

Age Group	Male					Female				
	mx	ax	lx	nLx	ex	mx	ax	lx	nLx	ex
Early Neonatal	0.140779523	0.009584726	100000	0.019152307	79.61200004	0.1255152	0.009585194	100000	0.019155102	85.16725751
Late Neonatal	0.014404419	0.02876315	99731.79018	0.057356218	79.8055746	0.016516926	0.028762567	99760.87892	0.057369498	85.34723288
Post Neonatal	0.002313948	0.461479462	99649.43939	0.919077687	79.8122292	0.002231051	0.461485356	99666.50087	0.919276483	85.36860865
1 to 4	0.000588883	1.999215588	99439.34164	3.973320567	79.03714036	0.000586889	1.999218457	99465.6795	3.974517002	84.59117039
5 to 9	0.000243232	2.499493385	99231.42365	4.959046351	75.1486848	0.000366506	2.499238082	99265.52951	4.960160449	80.70603153
10 to 14	0.000379521	2.724043146	99131.36293	4.953321903	70.2042278	0.000309027	2.588003972	99144.75226	4.954555521	75.77756847
15 to 19	0.000984968	2.627383174	98992.92236	4.94351497	65.27445938	0.000385872	2.506297783	99037.19184	4.948765876	70.84916313
20 to 24	0.001574444	2.553502938	98745.77988	4.930910444	60.39340595	0.000424718	2.537329759	98913.09369	4.942856864	65.93171387
25 to 29	0.001161502	2.553674061	98498.25143	4.919271586	55.53857888	0.000353209	2.617749618	98801.339	4.937105937	61.0099401
30 to 34	0.001010819	2.58898716	98269.67255	4.907159003	50.67846434	0.000457592	2.640029392	98679.1386	4.930213269	56.087817
35 to 39	0.00125074	2.637600299	98009.00148	4.892879863	45.81991721	0.000572553	2.640885709	98522.26531	4.921197299	51.18109433
40 to 44	0.001368464	2.729642232	97689.06042	4.87380096	40.9816322	0.000742286	2.724290521	98314.54257	4.909075228	46.29245519
45 to 49	0.001910105	2.77128917	97222.83673	4.843109226	36.18138595	0.001148251	2.729167688	98024.66021	4.889946067	41.43054218
50 to 54	0.003299682	2.774359458	96421.32367	4.7889649	31.46680253	0.00186832	2.715213188	97531.26132	4.858252756	36.63309996
55 to 59	0.005774332	2.759994199	94992.65224	4.692268532	26.90571516	0.002698252	2.714326207	96738.32511	4.808803183	31.92374635
60 to 64	0.009990186	2.701418765	92455.68749	4.522547971	22.57325084	0.004321564	2.706674964	95518.64069	4.731433452	27.30410999
65 to 69	0.015728371	2.680978092	88118.20111	4.25487089	18.55448876	0.006879492	2.709181647	93583.76626	4.608195894	22.82103576
70 to 74	0.025399543	2.666583721	81644.87408	3.859344991	14.81964562	0.011097574	2.777462664	90504.90802	4.417731888	18.50801606
75 to 79	0.042770944	2.613245249	72126.2597	3.278965889	11.43530375	0.022869275	2.712834283	85700.09595	4.075657455	14.38971208
80 to 84	0.069197987	2.536635382	58420.94079	2.506109071	8.52064751	0.040498266	2.663876777	76569.97896	3.504034199	10.7853139
85 to 89	0.125394398	2.449653793	41565.10532	1.586363156	5.976327338	0.083228436	2.605779538	62690.48739	2.624590684	7.584863905
90 to 94	0.219911942	2.20084241	22249.60419	0.695474828	4.117758206	0.162188883	2.331505614	41404.15226	1.456088774	5.179945261
95 plus	0.316962513	3.162351403	7317.913866	0.235168731	3.162351403	0.261477473	3.842308698	18365.57323	0.718274628	3.842308698

Table 15: Somalia 2017 life table, by age and sex. mx=mortality rate, ax=mean person-years lived in an age interval among those who die in that age interval, lx=number of persons left alive at age x, nLx=person-years lived between age x and x+n, ex=life expectancy at age x.

Age Group	Male					Female				
	mx	ax	lx	nLx	ex	mx	ax	lx	nLx	ex
Early Neonatal	1.009740167	0.009558093	100000	0.018993611	57.70326551	0.750560088	0.009566037	100000	0.019040725	61.7297549
Late Neonatal	0.154605638	0.028724476	98082.5533	0.056180944	58.80624405	0.135890228	0.028729638	98571.04061	0.056491043	62.6019519
Post Neonatal	0.035174782	0.459145124	97214.42215	0.883162591	59.27128576	0.031075618	0.459436307	97803.60183	0.890183405	63.03374198
1 to 4	0.00869461	1.988407434	94110.94235	3.699823812	60.28049325	0.008165931	1.989112294	95038.86058	3.740191147	63.92551171
5 to 9	0.001486236	2.496903677	90899.19848	4.528143076	58.33550107	0.001387203	2.497109997	91987.51226	4.583471443	61.97589274
10 to 14	0.001203567	2.661018125	90227.36785	4.498719003	53.75063563	0.000959802	2.513153075	91352.0828	4.55673788	57.38922401
15 to 19	0.002441451	2.728790468	89686.78339	4.459645239	49.05813021	0.001467123	2.647132069	90915.05309	4.530131539	52.65269705
20 to 24	0.003956102	2.581257649	88599.6464	4.388063165	44.62579192	0.002021184	2.605974601	90251.07191	4.490850303	48.01999878
25 to 29	0.004109912	2.530240042	86866.50469	4.299772252	40.46345267	0.002540451	2.602869654	89344.40346	4.440222227	43.48000336
30 to 34	0.004745505	2.570997978	85102.86182	4.206776369	36.24831651	0.003352771	2.626373016	88217.98128	4.376134922	39.000734
35 to 39	0.005984922	2.612443098	83111.43749	4.097210161	32.05381863	0.004709889	2.639407571	86753.26061	4.290073454	34.61324011
40 to 44	0.008413291	2.634433199	80666.72913	3.954930533	27.94436643	0.006774687	2.63019398	84736.97856	4.170091311	30.37177234
45 to 49	0.012245154	2.636677932	77352.42688	3.75932332	24.02672522	0.009463204	2.636717144	81919.86999	4.006695576	26.32256127
50 to 54	0.018194176	2.613395771	72770.93476	3.487922625	20.37156703	0.014053684	2.614266232	78141.94132	3.780881718	22.46412507
55 to 59	0.025692606	2.595606807	66461.77865	3.130889017	17.05685546	0.019249426	2.613416433	72852.69183	3.483492895	18.90171341
60 to 64	0.037415201	2.566348225	58469.98759	2.681207672	14.03561548	0.028906007	2.602134044	66187.13608	3.096185906	15.53994336
65 to 69	0.052312545	2.53535755	48515.29817	2.15100625	11.39543359	0.04273393	2.571061943	57299.23189	2.59776829	12.54718711
70 to 74	0.076231585	2.488986658	37367.25366	1.570954615	9.052171455	0.063764248	2.524826921	46300.85692	2.002781327	9.922775206
75 to 79	0.106470959	2.422259719	25520.18602	1.004053024	7.12330468	0.092917184	2.464368174	33685.23916	1.367428127	7.710635531
80 to 84	0.151103518	2.341724198	14963.20217	0.535987911	5.47955399	0.136938144	2.382255401	21184.98027	0.783752123	5.845160436
85 to 89	0.21681138	2.219001396	6971.34129	0.218506417	4.134259652	0.203993558	2.260446379	10646.49474	0.343740447	4.339549071
90 to 94	0.307266007	2.02557416	2287.370186	0.060048645	3.125397315	0.292737138	2.063146811	3749.518238	0.101586414	3.255125162
95 plus	0.385778822	2.592883609	458.0395553	0.011945257	2.592883609	0.378459547	2.644308829	816.9410031	0.021822676	2.644308829

Table 15: Somalia 2100 life table, by age and sex. mx=mortality rate, ax=mean person-years lived in an age interval among those who die in that age interval, lx=number of persons left alive at age x, nLx=person-years lived between age x and x+n, ex=life expectancy at age x.

Age Group	Male					Female				
	mx	ax	lx	nLx	ex	mx	ax	lx	nLx	ex
Early Neonatal	0.209570341	0.009582618	100000	0.019139611	70.39091977	0.145404235	0.009584584	100000	0.019151375	75.60929285
Late Neonatal	0.031789289	0.028758354	99599.16332	0.057251319	70.64634235	0.026994278	0.028759677	99721.65179	0.05732961	75.79430081
Post Neonatal	0.004500612	0.46132412	99417.40052	0.916007993	70.71445175	0.004140032	0.461349735	99567.04527	0.917538336	75.85072205
1 to 4	0.001102359	1.99853019	99006.09361	3.95155622	70.07456781	0.000999513	1.998667317	99187.89376	3.959615269	75.20752652
5 to 9	0.000437621	2.499088289	98572.16008	4.923237671	66.36486306	0.000448211	2.499066228	98793.21868	4.934143654	71.49139472
10 to 14	0.000436193	2.820491468	98357.44029	4.913183304	61.5011465	0.000362034	2.533191463	98572.62339	4.924236505	66.64243721
15 to 19	0.001082321	2.746227564	98143.75862	4.895262243	56.62648896	0.00050007	2.59306427	98394.79749	4.91383801	61.75599895
20 to 24	0.001711314	2.583677227	97615.43967	4.860727413	51.91281641	0.00058489	2.592497933	98149.73016	4.900609476	56.90064336
25 to 29	0.001786667	2.544452545	96786.26108	4.818269692	47.32838141	0.000766444	2.631453025	97863.99106	4.884380002	52.05546623
30 to 34	0.002135755	2.56477935	95929.19294	4.771809614	42.72060362	0.001090728	2.634602231	97491.02956	4.862071252	47.23941433
35 to 39	0.002527807	2.619986508	94916.01016	4.717703316	38.13876079	0.001493316	2.670600967	96963.13679	4.831450177	42.47513867
40 to 44	0.003756327	2.675028498	93732.76463	4.64653455	33.57363479	0.002348175	2.69143036	96245.97028	4.786551063	37.76176402
45 to 49	0.005906707	2.68787007	92007.28778	4.539233066	29.13110966	0.003719162	2.718501086	95131.2774	4.716905879	33.15550672
50 to 54	0.009471101	2.674193524	89368.08718	4.373870061	24.88054472	0.006390261	2.661681594	93396.57272	4.602074952	28.69412441
55 to 59	0.014718966	2.663277718	85315.23839	4.1276742	20.89369594	0.009142895	2.651675488	90501.25912	4.431988331	24.48506264
60 to 64	0.023700215	2.625952446	79418.31541	3.76790454	17.1996858	0.014082371	2.666129727	86544.55957	4.19393634	20.43050463
65 to 69	0.035524566	2.598516862	70884.49623	3.282563915	13.90249428	0.023067041	2.637802648	80844.67137	3.843909761	16.61770182
70 to 74	0.054528385	2.567635974	60027.13377	2.678947803	10.9205815	0.036050874	2.630689879	72469.30001	3.361327798	13.15478025
75 to 79	0.086853645	2.506470147	46802.19525	1.971310278	8.321190578	0.063005205	2.585873527	61460.38285	2.717603448	9.980775941
80 to 84	0.13490045	2.407768449	32001.83542	1.245367233	6.200036296	0.108526234	2.496149373	46803.86277	1.93297764	7.336342799
85 to 89	0.197907101	2.271588319	18196.33139	0.629920371	4.558241922	0.168121043	2.365773136	30516.23791	1.141765277	5.287866728
90 to 94	0.28896008	2.064854978	7735.280261	0.223421934	3.349470488	0.254556644	2.150666128	15632.98575	0.496634036	3.805269672
95 plus	0.371169196	2.720331464	2047.12929	0.060023425	2.720331464	0.344044847	2.984567927	5317.036703	0.179254654	2.984567927

Table 15: South Sudan 2017 life table, by age and sex. mx=mortality rate, ax=mean person-years lived in an age interval among those who die in that age interval, lx=number of persons left alive at age x, nLx=person-years lived between age x and x+n, ex=life expectancy at age x.

Age Group	Male					Female				
	mx	ax	lx	nLx	ex	mx	ax	lx	nLx	ex
Early Neonatal	1.699854798	0.009536942	100000	0.018868896	56.26182719	1.309581795	0.009548903	100000	0.018939278	61.11313461
Late Neonatal	0.174890714	0.02871888	96793.30067	0.055410192	58.10147312	0.151475244	0.028725339	97520.11615	0.055863763	62.64512465
Post Neonatal	0.049135851	0.458153431	95824.72719	0.864984313	58.62980745	0.044350401	0.458493348	96674.20754	0.874562768	63.13478661
1 to 4	0.011057898	1.985256633	91578.26112	3.583395353	60.40067365	0.01080018	1.98560022	92797.87333	3.632947692	64.82765992
5 to 9	0.00158178	2.496704629	87620.95894	4.363784978	59.0386114	0.001405864	2.497071118	88877.96276	4.428325237	63.59968316
10 to 14	0.001276032	2.65982506	86931.35404	4.333632421	54.48724442	0.001027153	2.524855855	88255.77914	4.401600399	59.03022362
15 to 19	0.002590081	2.732955893	86378.80068	4.29374753	49.81861226	0.001542584	2.645411898	87803.88059	4.374320071	54.32100478
20 to 24	0.004258813	2.587807935	85267.49029	4.220055573	45.4311107	0.002137585	2.621556493	87129.42213	4.334461868	49.72026939
25 to 29	0.004531876	2.53484034	83471.61406	4.127526731	41.35106653	0.002854337	2.610582633	86203.52228	4.281035638	45.22482148
30 to 34	0.005256572	2.564726685	81602.88082	4.028660196	37.23788183	0.003777374	2.612841488	84982.70034	4.211229712	40.83459079
35 to 39	0.006488131	2.58713064	79488.1124	3.913284774	33.15773103	0.005076726	2.595598658	83394.36584	4.119551724	36.55926513
40 to 44	0.008462483	2.59856311	76954.40366	3.771318031	29.16117353	0.006376266	2.582860651	81308.07635	4.003888012	32.42711414
45 to 49	0.011282868	2.609632101	73773.13661	3.59215748	25.30274006	0.00801826	2.604497668	78764.22037	3.864263914	28.38665639
50 to 54	0.015833184	2.612029223	69737.25656	3.360441183	21.61223327	0.011093302	2.613601252	75679.24558	3.686813549	24.43337503
55 to 59	0.022568413	2.614657554	64445.11714	3.058584669	18.16876936	0.015374079	2.641619883	71610.30237	3.455908454	20.66790828
60 to 64	0.034270416	2.566296343	57585.95387	2.659237328	15.01966725	0.024535663	2.602447643	66329.39965	3.133499371	17.0985525
65 to 69	0.04481693	2.543417036	48546.02567	2.188740242	12.34123711	0.03346128	2.581043981	58700.82264	2.717282123	13.97981818
70 to 74	0.067033924	2.513450462	38836.69931	1.667286962	9.800973524	0.050658552	2.565947703	49702.88952	2.21539804	11.04716531
75 to 79	0.094213442	2.448007199	27794.15461	1.123509721	7.718632828	0.078098196	2.510859801	38623.43916	1.620785977	8.494444541
80 to 84	0.133940309	2.37509484	17357.33331	0.644783063	5.92663449	0.11905984	2.429124058	26150.39076	1.00520154	6.378426191
85 to 89	0.199413119	2.257157842	8847.977633	0.287416521	4.398468016	0.184820334	2.301299833	14379.63043	0.482435002	4.664019458
90 to 94	0.29161095	2.058970887	3188.225535	0.086253136	3.269058352	0.273863485	2.106235284	5599.394262	0.157245637	3.444857713
95 plus	0.373828917	2.6759794	696.3199613	0.018747894	2.6759794	0.362476072	2.761000603	1348.208051	0.037574835	2.761000603

Table 15: South Sudan 2100 life table, by age and sex. mx=mortality rate, ax=mean person-years lived in an age interval among those who die in that age interval, lx=number of persons left alive at age x, nLx=person-years lived between age x and x+n, ex=life expectancy at age x.

Age Group	Male					Female				
	mx	ax	lx	nLx	ex	mx	ax	lx	nLx	ex
Early Neonatal	0.243153823	0.009581588	100000	0.019133446	74.9641263	0.19581606	0.009583039	100000	0.019142122	80.20029533
Late Neonatal	0.024742135	0.028760298	99534.92042	0.057225934	75.29388837	0.027182418	0.028759625	99625.2554	0.057273852	80.48195471
Post Neonatal	0.004582862	0.461318277	99393.36658	0.915749253	75.3433417	0.00395344	0.46136299	99469.61	0.916717304	80.55007773
1 to 4	0.001033838	1.99862155	98974.08141	3.950796707	74.73527609	0.000911894	1.998784142	99107.41079	3.957080509	79.91771818
5 to 9	0.000350656	2.499269466	98566.08281	4.923989411	71.03500355	0.000318228	2.499337025	98746.8712	4.933420523	76.20087067
10 to 14	0.000396599	2.850246156	98393.54895	4.91548721	66.15459064	0.000305778	2.605368688	98589.99661	4.925889593	71.31774776
15 to 19	0.001018769	2.757485133	98198.77946	4.898755833	61.2794905	0.000467776	2.632016392	98439.51659	4.916526851	66.4224263
20 to 24	0.001665136	2.598001555	97700.32574	4.865545523	56.57644228	0.00059544	2.643230896	98209.79559	4.903635388	61.57100641
25 to 29	0.001771853	2.551624854	96892.01875	4.823693551	52.02463358	0.000900692	2.636776271	97918.17329	4.88560075	56.74570296
30 to 34	0.002098175	2.556952503	96039.84805	4.777568493	47.46122914	0.001266318	2.623283143	97479.09262	4.859393496	51.98738962
35 to 39	0.002368804	2.584936779	95041.03832	4.725179017	42.92933471	0.001675841	2.621547954	96866.30103	4.824146809	47.29695355
40 to 44	0.003180669	2.623624714	93926.62729	4.66130474	38.40349559	0.002218784	2.628358967	96063.06578	4.778034422	42.66790212
45 to 49	0.004352054	2.639399545	92453.87688	4.575926694	33.96217342	0.00290083	2.658834684	95012.20734	4.718626322	38.10716794
50 to 54	0.006170016	2.661051031	90479.24074	4.460138864	29.63294532	0.004303105	2.641074548	93655.72667	4.635993684	33.61572157
55 to 59	0.009421589	2.666061508	87759.08852	4.294760556	25.4518237	0.005843958	2.64958714	91679.44166	4.522165394	29.27517502
60 to 64	0.014605137	2.615537846	83777.74877	4.050941276	21.51526823	0.008482342	2.625738053	89068.01248	4.366630169	25.04742728
65 to 69	0.019193225	2.644028345	78014.57698	3.736296669	17.89770939	0.011583603	2.648966082	85421.78784	4.159092797	20.99128609
70 to 74	0.030854552	2.619375571	71089.33389	3.317333008	14.39226759	0.016975827	2.693982737	80705.48146	3.885221725	17.06051696
75 to 79	0.046176221	2.564446364	61150.6915	2.756330083	11.32921161	0.030870137	2.64210321	74221.94606	3.463222996	13.31659868
80 to 84	0.067526677	2.509450933	48785.9094	2.097398962	8.588906931	0.048045288	2.593288336	63728.34209	2.863156483	10.08128841
85 to 89	0.123234522	2.455406512	35048.03781	1.343732137	6.023894055	0.094625192	2.562423459	50301.22659	2.054342648	7.093886225
90 to 94	0.217802085	2.205091726	18953.96601	0.594376731	4.143173684	0.176415393	2.312213304	31393.37412	1.074106936	4.87701723
95 plus	0.315280333	3.17690334	6284.77551	0.202462205	3.17690334	0.274943495	3.651368182	12925.46988	0.480874556	3.651368182

Table 15: Tanzania 2017 life table, by age and sex. mx=mortality rate, ax=mean person-years lived in an age interval among those who die in that age interval, lx=number of persons left alive at age x, nLx=person-years lived between age x and x+n, ex=life expectancy at age x.

Age Group	Male					Female				
	mx	ax	lx	nLx	ex	mx	ax	lx	nLx	ex
Early Neonatal	1.199045511	0.009552291	100000	0.01895929	64.80262731	0.822768519	0.009563823	100000	0.019027578	69.12631982
Late Neonatal	0.081020167	0.028744774	97727.18601	0.056095815	66.28937417	0.071672598	0.028747352	98434.62717	0.056517039	70.20569874
Post Neonatal	0.021405366	0.460123244	97272.91244	0.889297738	66.54125073	0.020862941	0.460161776	98029.64638	0.896436967	70.43800013
1 to 4	0.004756953	1.993657436	95370.65924	3.778795247	66.93646271	0.004783369	1.993622215	96160.10288	3.809859294	70.87523889
5 to 9	0.001041936	2.497829301	93574.8874	4.666584928	64.18399478	0.000866775	2.498194218	94338.73048	4.706731419	68.20569883
10 to 14	0.000964704	2.600985644	93088.93588	4.643684802	59.50633332	0.00079563	2.558008683	93930.8236	4.687423309	63.49089156
15 to 19	0.001504054	2.634183862	92641.10385	4.615635665	54.78153174	0.001084799	2.611537186	93557.92326	4.665814797	58.73366959
20 to 24	0.001959762	2.596426785	91946.92426	4.575797771	50.17501006	0.001396423	2.616179144	93051.79054	4.637160503	54.0386022
25 to 29	0.002453947	2.60820887	91050.31056	4.525968973	45.64315189	0.001890971	2.623011887	92404.3531	4.599550061	49.39849267
30 to 34	0.003313822	2.620507621	89940.01899	4.461821048	41.17403778	0.002552045	2.624755429	91534.90254	4.549154847	44.84239865
35 to 39	0.004474952	2.607732926	88462.25266	4.376278118	36.81787313	0.003462203	2.601829813	90374.50339	4.481518051	40.38410799
40 to 44	0.005823454	2.610767911	86505.11776	4.26592877	32.59154464	0.004351311	2.598881845	88823.59738	4.395274921	36.04324011
45 to 49	0.007944863	2.616659558	84022.29978	4.12308779	28.47659538	0.005737688	2.613195771	86911.74308	4.286902897	31.77804197
50 to 54	0.01098086	2.616439524	80748.36585	3.934506289	24.52363346	0.007843697	2.603383574	84452.96049	4.144771632	27.62594397
55 to 59	0.015423576	2.629957649	76431.39618	3.686926236	20.75894087	0.010262145	2.633114949	81204.1586	3.964016418	23.62569675
60 to 64	0.023453225	2.613343826	70751.58785	3.350304155	17.21251286	0.015730663	2.640351405	77140.42197	3.719156971	19.73032347
65 to 69	0.034269278	2.589215584	62905.83326	2.905706108	14.03197034	0.023714859	2.629796524	71298.70632	3.375542477	16.1293559
70 to 74	0.051518387	2.55029591	52969.08379	2.352369932	11.17829936	0.037289394	2.601076671	63309.16339	2.906154577	12.83255046
75 to 79	0.074796587	2.501210248	40883.40185	1.723223785	8.731326111	0.057587045	2.558234292	52501.49551	2.302360784	9.940089554
80 to 84	0.112063022	2.432750246	28038.91284	1.089699378	6.591688607	0.090853256	2.495302392	39285.66263	1.601268771	7.427816354
85 to 89	0.176462966	2.310932393	15873.59031	0.538924706	4.79024409	0.15223402	2.380082247	24790.71617	0.887099538	5.320513048
90 to 94	0.270519337	2.103945982	6396.145465	0.179560187	3.480084904	0.240888279	2.182539678	11333.89934	0.338054838	3.825943946
95 plus	0.357520155	2.797422498	1551.295211	0.043470582	2.797422498	0.33387746	2.995811661	3214.887159	0.096522128	2.995811661

Table 15: Tanzania 2100 life table, by age and sex. mx=mortality rate, ax=mean person-years lived in an age interval among those who die in that age interval, lx=number of persons left alive at age x, nLx=person-years lived between age x and x+n, ex=life expectancy at age x.

Age Group	Male					Female				
	mx	ax	lx	nLx	ex	mx	ax	lx	nLx	ex
Early Neonatal	0.261972563	0.009581012	100000	0.019129997	78.38628984	0.18009704	0.009583521	100000	0.019145004	82.88420418
Late Neonatal	0.014835978	0.028763031	99499.01519	0.05722159	78.7609705	0.018392721	0.02876205	99655.26478	0.057305586	83.15112762
Post Neonatal	0.00303352	0.461428339	99414.14533	0.916594728	78.77056798	0.002972442	0.461432678	99549.8894	0.91787216	83.18145539
1 to 4	0.000597396	1.999203471	99136.19284	3.960716322	78.0664446	0.000613032	1.999182624	99277.15679	3.966223849	82.48481466
5 to 9	0.000273083	2.499431078	98899.72761	4.941613138	74.24793331	0.000222097	2.499537297	99034.14505	4.948960041	78.68175541
10 to 14	0.000325311	2.658013853	98764.83052	4.934470207	69.34581191	0.000257837	2.555447298	98924.2786	4.94309542	73.76618202
15 to 19	0.000508404	2.590316622	98604.36509	4.924183655	64.45424956	0.000287001	2.520503133	98796.89543	4.936359738	68.85780156
20 to 24	0.000543596	2.588880705	98354.11588	4.911273316	59.61154826	0.000311734	2.59236427	98655.31588	4.929088899	63.95265369
25 to 29	0.000745754	2.626801428	98087.26744	4.895724969	54.76650133	0.000445673	2.609603631	98501.79385	4.919843323	59.04796162
30 to 34	0.001023956	2.590436215	97722.42635	4.874064916	49.96074876	0.000535976	2.608863295	98282.85944	4.907779305	54.17328031
35 to 39	0.001156254	2.594327804	97223.91803	4.847692255	45.20321239	0.000642882	2.615666792	98020.38165	4.893477411	49.31097964
40 to 44	0.001528133	2.694360461	96664.04959	4.81620573	40.4495798	0.000845338	2.713252829	97706.27374	4.87585449	44.46057047
45 to 49	0.002568916	2.697340232	95928.87009	4.768206566	35.7382256	0.001476022	2.728792582	97294.50356	4.848447548	39.63649691
50 to 54	0.003979703	2.713682666	94705.2875	4.692488944	31.16383497	0.002456203	2.696158948	96579.56797	4.801798915	34.90843448
55 to 59	0.006716105	2.693298731	92840.8476	4.571203055	26.73347001	0.003818744	2.664127307	95401.83324	4.72788287	30.30394586
60 to 64	0.010609369	2.658281278	89776.19293	4.380162821	22.55208681	0.005505345	2.672566984	93600.23286	4.620921977	25.83339071
65 to 69	0.016054074	2.647230975	85138.77303	4.102408743	18.63189011	0.008728876	2.690948914	91063.51382	4.463450385	21.47384373
70 to 74	0.024987755	2.64842205	78575.5557	3.711739513	14.96197319	0.014351506	2.740487472	87184.32465	4.222896392	17.30365181
75 to 79	0.041722525	2.606506694	69353.88389	3.155239969	11.59370993	0.028306801	2.68587327	81165.48225	3.810742018	13.37602288
80 to 84	0.065979019	2.534043069	56308.9753	2.426183041	8.674732147	0.0490478	2.627839428	70493.70758	3.162218867	9.988702829
85 to 89	0.121395945	2.460761982	40526.42858	1.55523378	6.073849897	0.096291568	2.556100121	55228.97454	2.244100529	7.022996778
90 to 94	0.215890322	2.208361742	21963.85471	0.689280938	4.170186065	0.178486544	2.309291123	34064.38319	1.158737094	4.833447226
95 plus	0.313719217	3.192351387	7297.194061	0.235251641	3.192351387	0.276902909	3.623920056	13803.1805	0.508002359	3.623920056

Table 15: Uganda 2017 life table, by age and sex. mx=mortality rate, ax=mean person-years lived in an age interval among those who die in that age interval, lx=number of persons left alive at age x, nLx=person-years lived between age x and x+n, ex=life expectancy at age x.

Age Group	Male					Female				
	mx	ax	lx	nLx	ex	mx	ax	lx	nLx	ex
Early Neonatal	1.160881357	0.00955346	100000	0.018966188	62.55833945	0.836485265	0.009563403	100000	0.019025079	69.42886573
Late Neonatal	0.081813604	0.028744555	97798.49479	0.056135437	63.94675225	0.070317287	0.028747726	98408.68588	0.056504337	70.53190061
Post Neonatal	0.021004495	0.460151721	97339.33857	0.890066305	64.19071546	0.018086585	0.460359001	98011.42175	0.897415045	70.76009731
1 to 4	0.004919992	1.993440054	95470.43434	3.781502333	64.51523929	0.003994812	1.994673607	96388.67292	3.824915155	71.02038634
5 to 9	0.001395249	2.497093234	93610.82719	4.664255081	61.75787941	0.001148075	2.497608179	94861.17823	4.729473016	68.13215366
10 to 14	0.001146315	2.570350146	92960.14087	4.63508684	57.17252193	0.000909937	2.503405103	94318.26832	4.705215522	63.50974591
15 to 19	0.001790491	2.645070631	92428.8956	4.602044476	52.48623981	0.001164081	2.59596992	93890.18715	4.681416548	58.78778478
20 to 24	0.002429755	2.604790281	91604.96351	4.553750663	47.93403301	0.001467222	2.616091494	93345.29243	4.651000599	54.11549166
25 to 29	0.003074486	2.592186981	90498.67913	4.49170082	43.48731713	0.002007585	2.614717528	92663.02317	4.611073913	49.49426696
30 to 34	0.00390041	2.589962954	89118.14062	4.41441661	39.12009624	0.002611527	2.602733413	91737.62819	4.558336388	44.96677735
35 to 39	0.004900442	2.603799857	87397.29949	4.319171696	34.83877139	0.00334779	2.593493562	90547.69719	4.49121553	40.52298712
40 to 44	0.006605021	2.622908952	85282.06933	4.198233777	30.63750256	0.004231302	2.599492254	89044.74054	4.407489975	36.16223416
45 to 49	0.009292097	2.637095839	82511.23981	4.037004322	26.5767372	0.005567801	2.619706856	87180.60771	4.302054544	31.87843172
50 to 54	0.01374781	2.619188744	78763.50612	3.813495473	22.71378873	0.007801268	2.604257811	84786.64213	4.161616931	27.70264507
55 to 59	0.019185005	2.61407294	73527.55332	3.515704727	19.14247295	0.010086516	2.631968407	81543.32626	3.982182367	23.69866074
60 to 64	0.028562633	2.597054644	66794.70584	3.125672942	15.80687867	0.015477795	2.642251338	77532.85453	3.740414773	19.78624771
65 to 69	0.041444716	2.569310693	57887.594	2.63018147	12.83884799	0.023427307	2.632527111	71756.17219	3.399761744	16.16441101
70 to 74	0.061529302	2.525067332	47019.33238	2.04131726	10.21387944	0.037018977	2.603792862	63814.93008	2.931682764	12.84733723
75 to 79	0.088033402	2.46629213	34508.23157	1.412032691	8.006670272	0.057466513	2.560752264	53005.83765	2.32585279	9.937772569
80 to 84	0.127190697	2.390418978	22138.45241	0.832316878	6.113663541	0.091252932	2.497061784	39706.08146	1.618039696	7.41486056
85 to 89	0.192500037	2.272742689	11611.86748	0.381481288	4.508537966	0.152709693	2.379006778	25030.67889	0.895481612	5.311936826
90 to 94	0.285340776	2.0723752	4306.44058	0.117571941	3.32864276	0.241359289	2.181425065	11438.35502	0.34119295	3.820987322
95 plus	0.369018235	2.710363858	965.1224266	0.026230229	2.710363858	0.334280784	2.992769941	3245.754953	0.097504453	2.992769941

Table 15: Uganda 2100 life table, by age and sex. mx=mortality rate, ax=mean person-years lived in an age interval among those who die in that age interval, lx=number of persons left alive at age x, nLx=person-years lived between age x and x+n, ex=life expectancy at age x.

Age Group	Male					Female				
	mx	ax	lx	nLx	ex	mx	ax	lx	nLx	ex
Early Neonatal	0.24544132	0.009581518	100000	0.019133025	74.51664162	0.202489679	0.009582835	100000	0.019140897	83.33957103
Late Neonatal	0.014362165	0.028763161	99530.53072	0.057240493	74.84794472	0.019326703	0.028761792	99612.48049	0.057279445	83.64383022
Post Neonatal	0.003496076	0.46139548	99448.33982	0.916714203	74.85211234	0.003459518	0.461398077	99501.80374	0.917222526	83.67915634
1 to 4	0.000414552	1.999447264	99127.91495	3.961832133	74.16897041	0.000350193	1.999533076	99184.55972	3.964606228	83.02153708
5 to 9	0.000293466	2.499388613	98963.74092	4.944559407	70.28840823	0.00026385	2.499450313	99045.78865	4.949025402	79.13467311
10 to 14	0.000289561	2.671495754	98818.67539	4.937586926	65.38782333	0.000226659	2.529645418	98915.26049	4.942973539	74.23560324
15 to 19	0.00051492	2.660398894	98675.76454	4.927853387	60.47857907	0.000273914	2.535297598	98803.29679	4.936851093	69.31667328
20 to 24	0.000685558	2.587937532	98422.25374	4.912988827	55.62687364	0.000290421	2.559432125	98668.1574	4.929914849	64.40788502
25 to 29	0.000797885	2.588277897	98085.93673	4.894891832	50.80767316	0.000355873	2.602019078	98525.11937	4.92203385	59.49740809
30 to 34	0.001029066	2.60175356	97696.07462	4.872755599	45.99885504	0.000439778	2.625848172	98350.17353	4.912358137	54.59834317
35 to 39	0.001261788	2.651071703	97195.8143	4.845458418	41.22057255	0.000596412	2.64192441	98134.4025	4.899836042	49.71215237
40 to 44	0.001960135	2.741143822	96585.93177	4.807973567	36.46169771	0.000850605	2.705580743	97842.46344	4.882566199	44.85186659
45 to 49	0.003487244	2.753045413	95646.58373	4.745128859	31.78849788	0.001404278	2.715799149	97427.7835	4.855759772	40.03014146
50 to 54	0.006187471	2.741043045	93999.57493	4.635245886	27.29024	0.002252052	2.698302619	96747.03231	4.812352548	35.29089664
55 to 59	0.010773078	2.69753036	91152.27855	4.44776588	23.04493435	0.003509222	2.689201648	95665.45161	4.744668211	30.65693297
60 to 64	0.017153407	2.646018283	86409.4861	4.154367728	19.14720714	0.005344975	2.683323222	94005.28442	4.642894023	26.14806981
65 to 69	0.025628309	2.636191938	79375.74283	3.745321205	15.59606139	0.008524654	2.687313479	91531.79455	4.488309006	21.77716924
70 to 74	0.040927106	2.604330021	69959.18811	3.191352844	12.33903297	0.0136171	2.742891735	87727.44369	4.256128551	17.59887907
75 to 79	0.06417275	2.545254363	57190.43776	2.47954518	9.523192187	0.026986179	2.689936172	81976.48991	3.86027498	13.63389177
80 to 84	0.098206208	2.459781868	41720.02855	1.679538787	7.145758728	0.046677404	2.634110658	71677.63754	3.232545104	10.20044888
85 to 89	0.160486664	2.352638655	25695.18746	0.910334781	5.12719383	0.092707937	2.569505369	56844.22275	2.329027152	7.171841649
90 to 94	0.255109035	2.135314888	11515.10162	0.336546601	3.66047131	0.174070212	2.315726287	35739.86464	1.22724182	4.924722507
95 plus	0.345305798	2.900705234	3135.952213	0.092506047	2.900705234	0.272735905	3.681386335	14860.96223	0.556561659	3.681386335

Table 15: Zambia 2017 life table, by age and sex. mx=mortality rate, ax=mean person-years lived in an age interval among those who die in that age interval, lx=number of persons left alive at age x, nLx=person-years lived between age x and x+n, ex=life expectancy at age x.

Age Group	Male					Female				
	mx	ax	lx	nLx	ex	mx	ax	lx	nLx	ex
Early Neonatal	1.04239429	0.009557092	100000	0.018987683	60.67697052	0.667360863	0.009568587	100000	0.019055885	66.60161266
Late Neonatal	0.097656018	0.028740185	98021.1414	0.056237661	61.88136378	0.080742675	0.02874485	98728.4133	0.056670945	67.43960243
Post Neonatal	0.02333768	0.459985979	97472.20337	0.890328682	62.17201613	0.019505067	0.460258236	98270.94611	0.899205405	67.69577596
1 to 4	0.005787631	1.992283231	95395.91308	3.772052183	62.59203898	0.004408623	1.994121867	96517.76196	3.82688932	67.99381567
5 to 9	0.001263832	2.497367018	93215.2576	4.646069844	60.01104161	0.000989295	2.49793897	94831.72648	4.729881851	65.16787369
10 to 14	0.001262696	2.644821234	92628.15837	4.617669201	55.37533289	0.000974116	2.58147992	94363.93495	4.707102877	60.47857142
15 to 19	0.002158957	2.648932874	92045.15292	4.579019677	50.70914167	0.001376045	2.629230461	93905.50722	4.680025489	55.76131009
20 to 24	0.002857058	2.606849249	91056.57259	4.52192276	46.23043252	0.001863555	2.651875572	93261.64046	4.642783627	51.12807821
25 to 29	0.003721356	2.615185453	89764.82846	4.448804372	41.85737021	0.002789255	2.644519064	92396.74993	4.589694241	46.58173634
30 to 34	0.005102146	2.608482107	88110.13604	4.352408618	37.59350466	0.003875768	2.613687761	91117.42699	4.514085394	42.19858482
35 to 39	0.006599881	2.599273193	85891.86747	4.227649803	33.49681063	0.004980966	2.592826039	89369.42387	4.415539962	37.97321622
40 to 44	0.008673291	2.598924723	83105.08074	4.070545634	29.53279401	0.006333449	2.582191531	87171.32207	4.292854692	33.86510022
45 to 49	0.011496216	2.604797198	79578.28547	3.87238002	25.72577026	0.007905506	2.592050092	84453.08837	4.143810354	29.87001731
50 to 54	0.01597019	2.585774138	75129.98875	3.617163028	22.09277397	0.01056647	2.571276815	81177.65995	3.957390098	25.96734821
55 to 59	0.020630532	2.593207796	69360.07474	3.304169834	18.71412905	0.012590318	2.602252882	76999.43607	3.737313085	22.2331086
60 to 64	0.03023861	2.587264154	62554.28657	2.915393502	15.46786169	0.018744521	2.627962331	72301.9131	3.461538294	18.50503915
65 to 69	0.043184682	2.560370405	53755.18798	2.432055336	12.57718678	0.028172178	2.61349366	65829.59834	3.08479257	15.06315709
70 to 74	0.063418508	2.518926732	43274.21354	1.870139506	10.00539559	0.043294132	2.583346581	57171.83168	2.588946644	11.94711481
75 to 79	0.090639949	2.462664221	31441.37217	1.278758735	7.826672416	0.066601609	2.537303369	46016.24332	1.978307898	9.219603544
80 to 84	0.132711656	2.387939508	19880.46319	0.738742472	5.952063706	0.104346512	2.464029202	32920.98747	1.303859334	6.886046466
85 to 89	0.198449277	2.259026188	10105.45323	0.327623448	4.41004269	0.168216491	2.339755835	19424.37772	0.672821064	4.978870725
90 to 94	0.290774835	2.060752674	3622.015747	0.097764681	3.275497038	0.257239302	2.144665273	8198.571394	0.237179151	3.627884003
95 plus	0.373205524	2.679747866	785.5765421	0.021083845	2.679747866	0.348176082	2.873664615	2141.152478	0.061864482	2.873664615

Table 15: Zambia 2100 life table, by age and sex. mx=mortality rate, ax=mean person-years lived in an age interval among those who die in that age interval, lx=number of persons left alive at age x, nLx=person-years lived between age x and x+n, ex=life expectancy at age x.

Age Group	Male					Female				
	mx	ax	lx	nLx	ex	mx	ax	lx	nLx	ex
Early Neonatal	0.234978672	0.009581839	100000	0.019134944	74.46046536	0.163350466	0.009584034	100000	0.019148076	81.60185779
Late Neonatal	0.017213793	0.028762375	99550.51065	0.057247287	74.77660391	0.019901001	0.028761634	99687.25983	0.057321495	81.83801805
Post Neonatal	0.006613214	0.461174044	99451.98305	0.915430759	74.79296709	0.006067173	0.461212834	99573.19938	0.916777268	81.87397376
1 to 4	0.000603761	1.999194986	98846.72692	3.949101332	74.32450094	0.000405249	1.999459668	99017.0826	3.957476306	81.40738355
5 to 9	0.000421877	2.499121091	98608.44876	4.925227458	70.49873697	0.00031841	2.499336645	98856.78137	4.938907649	77.53556389
10 to 14	0.000445599	2.632652892	98400.72507	4.914847495	65.64214197	0.00032125	2.523472598	98699.56804	4.931054099	72.65482947
15 to 19	0.000703213	2.606870605	98181.78509	4.900860181	60.78252106	0.000353559	2.509125977	98541.20078	4.922806617	67.76730916
20 to 24	0.000823634	2.598172894	97837.38584	4.882227847	55.98663038	0.000416038	2.591863042	98367.24596	4.913481346	62.88211373
25 to 29	0.00110851	2.592111374	97435.6102	4.858867312	51.20579399	0.000575331	2.571410492	98163.06357	4.901295568	58.00695987
30 to 34	0.001364687	2.567675704	96897.79662	4.828802595	46.4744426	0.00059123	2.589373072	97881.77705	4.88698464	53.16574635
35 to 39	0.001468669	2.601155435	96240.59284	4.795138551	41.77305968	0.000671888	2.619237902	97593.80938	4.871912284	48.31463388
40 to 44	0.002063003	2.712633645	95538.20739	4.754429601	37.05918231	0.000984779	2.723564597	97266.93296	4.852425605	43.4669072
45 to 49	0.003528402	2.727379921	94560.10178	4.690277476	32.41152291	0.001681042	2.7492985	96789.97718	4.821123414	38.66474145
50 to 54	0.005820461	2.723727365	92911.21773	4.584705624	27.93376218	0.002856541	2.698965489	95981.73204	4.767569177	33.96273817
55 to 59	0.009817987	2.704294472	90255.75769	4.41346761	23.66966163	0.004182702	2.690117485	94626.04842	4.685684809	29.40464559
60 to 64	0.016138892	2.653852453	85951.8792	4.141528349	19.71288279	0.006248928	2.693128849	92677.96377	4.568296665	24.96064159
65 to 69	0.024042787	2.624928931	79323.60906	3.75355596	16.13200714	0.010363588	2.693832661	89841.71794	4.387708929	20.65226566
70 to 74	0.036765465	2.60740277	70391.98234	3.238148698	12.84312025	0.016473498	2.742434609	85347.41439	4.115925438	16.58358612
75 to 79	0.058596316	2.562711103	58643.94835	2.571022878	9.895581077	0.0331091	2.679757718	78680.47043	3.658714585	12.73738053
80 to 84	0.092251399	2.49026884	43832.11501	1.787072791	7.386990412	0.056914489	2.609389414	66876.57447	2.95648011	9.499149902
85 to 89	0.153808976	2.370119794	27705.85982	0.993892754	5.269876158	0.106965173	2.522178415	50711.16842	2.026277534	6.696395431
90 to 94	0.248642176	2.148296474	12796.57592	0.3780978	3.736988094	0.190412964	2.284699264	30154.70503	1.012685379	4.640905423
95 plus	0.340173198	2.944526772	3592.962059	0.107380053	2.944526772	0.287811721	3.503917308	11852.01951	0.431800798	3.503917308

Table 15: Southern Sub-Saharan Africa 2017 life table, by age and sex. mx=mortality rate, ax=mean person-years lived in an age interval among those who die in that age interval, lx=number of persons left alive at age x, nLx=person-years lived between age x and x+n, ex=life expectancy at age x.

Age Group	Male					Female				
	mx	ax	lx	nLx	ex	mx	ax	lx	nLx	ex
Early Neonatal	0.676493874	0.009568307	100000	0.019054213	61.78131932	0.521839665	0.009573047	100000	0.019082436	68.72822546
Late Neonatal	0.071183395	0.028747487	98711.01602	0.056676506	62.56869943	0.054301234	0.028752144	99004.21513	0.056872445	69.40019072
Post Neonatal	0.015951552	0.460510668	98307.5892	0.90101091	62.76780121	0.014389909	0.460621603	98695.39782	0.905215966	69.55971543
1 to 4	0.002240231	1.997013029	96870.41611	3.857508755	62.76890744	0.002004122	1.997327841	97392.85027	3.880141556	69.56057694
5 to 9	0.000955945	2.498008449	96006.31488	4.788862407	59.3159113	0.000744898	2.498448129	96615.26834	4.821778744	66.10436951
10 to 14	0.000905167	2.701555655	95548.5466	4.767506964	54.58812753	0.000662219	2.652579191	96256.10481	4.805333543	61.3416957
15 to 19	0.001845967	2.743729113	95117.02461	4.736124989	49.82352852	0.001236032	2.758046456	95937.89501	4.783639471	56.53635075
20 to 24	0.003107822	2.69507166	94242.76786	4.678625125	45.26021597	0.002215402	2.742207613	95346.62899	4.743605264	51.86975469
25 to 29	0.004874447	2.668114432	92788.76093	4.587298971	40.92712821	0.003880584	2.674499644	94295.76112	4.672623912	47.41711123
30 to 34	0.007323904	2.618756526	90552.78958	4.450035872	36.8716581	0.005632619	2.595386259	92482.64967	4.562345046	43.29402319
35 to 39	0.009597021	2.578157126	87293.88262	4.265563448	33.15020278	0.006758239	2.550989783	89913.23368	4.422479446	39.45680585
40 to 44	0.011859822	2.551835457	83200.75933	4.042677687	29.65405615	0.007738909	2.530764149	86925.10015	4.264776874	35.72533188
45 to 49	0.013941437	2.551204279	78407.10075	3.790955063	26.31097788	0.008482502	2.535233552	83625.55592	4.095668545	32.03504165
50 to 54	0.017371357	2.549401946	73123.00851	3.506886807	23.02795477	0.009933544	2.541547634	80152.39562	3.912102864	28.31335976
55 to 59	0.021107785	2.559604892	67032.23069	3.187447878	19.88882157	0.011444806	2.572065729	76267.36827	3.710292095	24.62628083
60 to 64	0.028426496	2.56298032	60305.34137	2.819942437	16.82197142	0.015417004	2.60220459	72021.99998	3.472750251	20.92634974
65 to 69	0.038303184	2.537141453	52290.44663	2.389175795	14.00768687	0.021691777	2.597929034	66669.18587	3.168406116	17.39763302
70 to 74	0.050325537	2.512945979	43140.64375	1.917126657	11.44071261	0.03085582	2.580058003	59797.92713	2.782205338	14.09831784
75 to 79	0.068094317	2.511010467	33494.45217	1.432061639	9.012179674	0.04391313	2.594891112	51215.53329	2.316216812	11.02869327
80 to 84	0.109323435	2.465886614	23745.00509	0.929730178	6.681957383	0.076409552	2.55788784	41047.23728	1.729683706	8.118301656
85 to 89	0.173865618	2.317197045	13582.71375	0.463142274	4.836908148	0.134415235	2.429520882	27834.02038	1.034396408	5.7584218
90 to 94	0.268107875	2.10907711	5531.525695	0.155819917	3.505134187	0.222100772	2.225239804	13933.4161	0.431071287	4.08032876
95 plus	0.355644493	2.811810776	1354.355472	0.038084823	2.811810776	0.317140506	3.15322804	4361.169893	0.137537331	3.15322804

Table 15: Southern Sub-Saharan Africa 2100 life table, by age and sex. mx=mortality rate, ax=mean person-years lived in an age interval among those who die in that age interval, lx=number of persons left alive at age x, nLx=person-years lived between age x and x+n, ex=life expectancy at age x.

Age Group	Male					Female				
	mx	ax	lx	nLx	ex	mx	ax	lx	nLx	ex
Early Neonatal	0.236293555	0.009581799	100000	0.019134704	73.18125339	0.203575391	0.009582802	100000	0.019140699	80.13348154
Late Neonatal	0.024986753	0.028760231	99548.01804	0.057233068	73.49276417	0.021845387	0.028761097	99610.43454	0.057274125	80.42582687
Post Neonatal	0.005113717	0.461280566	99405.06615	0.915632705	73.54034279	0.005124583	0.461279794	99485.36123	0.916368293	80.46882796
1 to 4	0.000428992	1.999428011	98937.15089	3.954096228	72.96085982	0.000412247	1.999450337	99016.24874	3.957391327	79.92152784
5 to 9	0.000268293	2.499441056	98767.71614	4.935078846	69.08185323	0.000222697	2.499536047	98853.37254	4.939921487	76.0488504
10 to 14	0.00028949	2.780344534	98635.47217	4.928586176	64.1706043	0.000199791	2.631769483	98743.51104	4.934845985	71.13001771
15 to 19	0.000636139	2.827556184	98492.96286	4.917810991	59.25900465	0.000352628	2.826838432	98645.06809	4.928459043	66.19778144
20 to 24	0.001246908	2.656421359	98180.57508	4.894725567	54.43680482	0.000730561	2.648063052	98471.71	4.915179093	61.30775038
25 to 29	0.001565807	2.576993214	97571.24532	4.860284045	49.75706095	0.000895192	2.545714537	98113.62258	4.895054037	56.51912853
30 to 34	0.001967013	2.573484448	96812.43017	4.817793325	45.12290179	0.001026349	2.552891282	97677.4936	4.871628285	51.75648585
35 to 39	0.002330453	2.63400375	95870.8647	4.767353633	40.5347217	0.001043517	2.61118204	97182.41291	4.84703986	47.00273886
40 to 44	0.003380095	2.656927007	94770.79215	4.701416067	35.96733197	0.001492545	2.716769777	96680.84129	4.817502671	42.22811037
45 to 49	0.004698017	2.723503939	93199.36495	4.610788589	31.52043593	0.002347282	2.73339026	95968.5245	4.772902031	37.51516804
50 to 54	0.008212822	2.695293278	91061.46535	4.469093891	27.18917529	0.003823886	2.705191145	94858.8399	4.701773968	32.91409958
55 to 59	0.012587467	2.655653303	87441.47899	4.247926458	23.19250385	0.005976335	2.657558795	93079.72005	4.590145065	28.47993177
60 to 64	0.018682769	2.618275389	82173.84374	3.935828091	19.49710359	0.008242669	2.645980975	90375.83451	4.433975531	24.23501954
65 to 69	0.026592336	2.590727055	74933.03017	3.524997805	16.11454146	0.012314025	2.674716763	86786.42121	4.220657016	20.10305373
70 to 74	0.037708275	2.56428452	65750.39801	3.018346476	12.9972522	0.019888133	2.669945426	81715.00275	3.910293429	16.15853454
75 to 79	0.053479411	2.58070205	54711.77611	2.434048842	10.10170345	0.033210058	2.691377233	74218.26283	3.458820385	12.48912449
80 to 84	0.095674524	2.525175564	42268.9248	1.72733464	7.342786359	0.06494887	2.632070043	63386.5236	2.776731815	9.144168518
85 to 89	0.157428136	2.363330828	26655.26975	0.960968905	5.23813434	0.117039334	2.495292244	46884.19892	1.857468001	6.461750981
90 to 94	0.25161076	2.140636988	12489.653	0.372601295	3.720098182	0.200930049	2.260769632	27400.42136	0.917601587	4.504652516
95 plus	0.342335508	2.934645984	3616.930916	0.110173038	2.934645984	0.297106711	3.419637067	10731.36432	0.39330781	3.419637067

Table 15: Botswana 2017 life table, by age and sex. mx=mortality rate, ax=mean person-years lived in an age interval among those who die in that age interval, lx=number of persons left alive at age x, nLx=person-years lived between age x and x+n, ex=life expectancy at age x.

Age Group	Male					Female				
	mx	ax	lx	nLx	ex	mx	ax	lx	nLx	ex
Early Neonatal	0.496212147	0.009573832	100000	0.019087127	67.12081373	0.404734924	0.009576636	100000	0.019103848	71.06669689
Late Neonatal	0.03877594	0.028756427	99053.01065	0.056925879	67.74320144	0.023589401	0.028760616	99226.86502	0.057050712	71.6009389
Post Neonatal	0.005851269	0.461228171	98832.27131	0.91004652	67.83691832	0.00507248	0.461283495	99092.30947	0.912768459	71.64057085
1 to 4	0.001595987	1.997872018	98299.95984	3.919479051	67.27860439	0.001263796	1.998314939	98629.41642	3.935224548	71.05131556
5 to 9	0.000493571	2.498971727	97674.67041	4.877713223	63.69674387	0.000389208	2.49918915	98132.23714	4.90184112	67.40119407
10 to 14	0.000451717	2.760571165	97433.95793	4.866768716	58.84797501	0.000338019	2.765988012	97941.46994	4.893374134	62.52753346
15 to 19	0.001056609	2.760461675	97214.15384	4.849236322	53.97488987	0.000819167	2.789461608	97776.07921	4.879975217	57.62858565
20 to 24	0.001789967	2.685404915	96701.79899	4.815170687	49.24619227	0.001493858	2.698138982	97376.31757	4.852150614	52.85349271
25 to 29	0.002716272	2.675972568	95839.99431	4.761995474	44.66431492	0.00228386	2.672076946	96651.5838	4.807038021	48.22906876
30 to 34	0.004220364	2.669588721	94547.22922	4.681299665	40.23691169	0.003446759	2.65547737	95554.34365	4.739374107	43.75138808
35 to 39	0.006279882	2.650689388	92574.27942	4.561489133	36.03581559	0.004906089	2.626747411	93922.48897	4.642095789	39.46502502
40 to 44	0.009153045	2.596717255	89715.77873	4.389381461	32.09753251	0.006650752	2.578529872	91647.66863	4.509793675	35.37896661
45 to 49	0.011218411	2.554140543	85708.74266	4.171284647	28.47465253	0.007784741	2.552420004	88651.72626	4.349802481	31.48680964
50 to 54	0.013354329	2.534789245	81041.39761	3.923253991	24.96429194	0.009262567	2.540581931	85268.68085	4.168549041	27.63335911
55 to 59	0.01509908	2.560933031	75819.41413	3.656751187	21.50851494	0.010374241	2.580997546	81412.43309	3.971093767	23.82135013
60 to 64	0.020320634	2.600183881	70319.89445	3.352988687	17.99126745	0.014586232	2.627491195	77299.21521	3.735844433	19.9513707
65 to 69	0.0295787	2.602618262	63529.04405	2.966743854	14.63714651	0.021791615	2.63590996	71857.73884	3.417106421	16.26304831
70 to 74	0.045065401	2.582763074	54782.21642	2.470958731	11.55954389	0.034628942	2.624949036	64422.64193	2.976770271	12.83559294
75 to 79	0.071004075	2.535737426	43689.44163	1.860503876	8.841223032	0.057324549	2.583170066	54134.65268	2.378093894	9.776380269
80 to 84	0.113072121	2.459762165	30542.31993	1.187983721	6.562278577	0.096446526	2.501513711	40537.36089	1.634404689	7.191412431
85 to 89	0.177946168	2.30745486	17188.82358	0.582232182	4.764929473	0.15918671	2.362038209	24828.46521	0.875318179	5.165774729
90 to 94	0.271882121	2.101035667	6887.079822	0.193009201	3.466453631	0.24805947	2.165970822	10945.6924	0.321865344	3.736244883
95 plus	0.358573888	2.789578798	1662.512166	0.046510401	2.789578798	0.34018301	2.940446231	2987.495324	0.088058123	2.940446231

Table 15: Botswana 2100 life table, by age and sex. mx=mortality rate, ax=mean person-years lived in an age interval among those who die in that age interval, lx=number of persons left alive at age x, nLx=person-years lived between age x and x+n, ex=life expectancy at age x.

Age Group	Male					Female				
	mx	ax	lx	nLx	ex	mx	ax	lx	nLx	ex
Early Neonatal	0.137052353	0.00958484	100000	0.019152908	78.74743066	0.12369018	0.00958525	100000	0.019155359	80.11504158
Late Neonatal	0.009782785	0.028764425	99737.62318	0.057367148	78.9351834	0.008550245	0.028764765	99763.14672	0.057383863	80.28534405
Post Neonatal	0.001525022	0.461535501	99681.51675	0.919700129	78.9219905	0.00166633	0.461525462	99714.09538	0.919940813	80.26714618
1 to 4	0.000211274	1.999718301	99541.42214	3.979976789	78.10881027	0.000185828	1.999752229	99560.99462	3.980961621	79.4648433
5 to 9	0.000106688	2.499777734	99457.43336	4.971546218	74.17299479	0.000110687	2.499769402	99487.09834	4.972979878	75.52189777
10 to 14	0.000119475	2.831892114	99404.42042	4.968915904	69.21120183	0.00011831	2.883657093	99432.10282	4.970357647	70.56199623
15 to 19	0.000279027	2.804340509	99345.09191	4.964213694	64.25086799	0.000325051	2.7569386	99373.35448	4.965032288	65.60168328
20 to 24	0.000528371	2.670029204	99206.5932	4.954298713	59.33639912	0.0005011	2.633202123	99212.17607	4.954779369	60.70252148
25 to 29	0.000778466	2.602145346	98944.91267	4.938173443	54.48550039	0.000685357	2.602335034	98964.29622	4.940140128	55.8463358
30 to 34	0.001047377	2.631773907	98561.10224	4.915820112	49.68613486	0.000864458	2.627727748	98626.68831	4.921081181	51.02674993
35 to 39	0.001368511	2.632516888	98048.34808	4.886642449	44.93067626	0.001017731	2.638671136	98203.36151	4.898376101	46.23290431
40 to 44	0.001894443	2.666917468	97384.28786	4.847640212	40.2160244	0.001476794	2.740935306	97707.38469	4.868977907	41.44995097
45 to 49	0.002575093	2.694777667	96474.78958	4.795071615	35.56599844	0.002522237	2.720532768	96992.48385	4.821765942	36.72782672
50 to 54	0.003893882	2.707698153	95252.35683	4.720295357	30.983917	0.003889233	2.690151543	95785.53414	4.746498244	32.14478927
55 to 59	0.005971132	2.688270745	93434.24039	4.608145851	26.53245237	0.005663417	2.648802909	93959.19696	4.636483506	27.70039493
60 to 64	0.009085321	2.684180479	90709.20287	4.442348575	22.24813745	0.007690383	2.675973802	91369.86318	4.48898925	23.3892625
65 to 69	0.014683884	2.697267128	86702.39705	4.193960588	18.15051478	0.01223086	2.716114201	87980.16494	4.281306649	19.16445254
70 to 74	0.025507145	2.670101796	80589.2425	3.805137576	14.31966466	0.021439625	2.727604403	82876.32833	3.956757415	15.1510387
75 to 79	0.043195649	2.649252746	70981.02925	3.225638193	10.89230102	0.040285335	2.704347714	74737.35755	3.435241184	11.4737851
80 to 84	0.081638142	2.575320789	57245.09942	2.396570588	7.871662138	0.080154995	2.592989269	61754.19203	2.625301572	8.306925624
85 to 89	0.141569846	2.403539707	38044.09765	1.400098006	5.555512461	0.136789445	2.43710957	42598.04806	1.627306657	5.90167456
90 to 94	0.236501534	2.171704304	18683.54344	0.564804645	3.890299274	0.222612328	2.217843536	22898.03031	0.740872802	4.1706909
95 plus	0.330434942	3.032235209	5592.591817	0.171911946	3.032235209	0.316646546	3.211339199	8189.493653	0.285352201	3.211339199

Table 15: Lesotho 2017 life table, by age and sex. mx=mortality rate, ax=mean person-years lived in an age interval among those who die in that age interval, lx=number of persons left alive at age x, nLx=person-years lived between age x and x+n, ex=life expectancy at age x.

Age Group	Male					Female				
	mx	ax	lx	nLx	ex	mx	ax	lx	nLx	ex
Early Neonatal	1.116815563	0.009554811	100000	0.01897418	51.17687911	0.8669141	0.00956247	100000	0.019019549	59.97713439
Late Neonatal	0.080681372	0.028744867	97881.25631	0.056184781	52.26481096	0.06777785	0.028748427	98351.35554	0.056475552	60.96310106
Post Neonatal	0.026156625	0.459785733	97428.09957	0.888771175	52.45022403	0.019535371	0.460256084	97968.67188	0.896425909	61.14363398
1 to 4	0.003478007	1.995362673	95104.78144	3.77787269	52.79647191	0.002980004	1.996026672	96218.07077	3.825886145	61.32405483
5 to 9	0.002036527	2.495757244	93791.93811	4.665806167	49.50796441	0.001382774	2.497119224	95078.52349	4.73753163	58.03540787
10 to 14	0.001858095	2.698605012	92841.92878	4.622327094	44.98861778	0.001271473	2.620640631	94423.50079	4.706931585	53.42025899
15 to 19	0.003872458	2.719214571	91983.23522	4.55892267	40.38305776	0.002140386	2.757322707	93825.09	4.668906705	48.74399052
20 to 24	0.006128401	2.687757959	90218.18884	4.447948916	36.11865348	0.004049304	2.755824722	92825.99618	4.599531531	44.23696421
25 to 29	0.009928991	2.646518046	87493.70239	4.274925963	32.15628462	0.007349546	2.64565428	90965.40115	4.470961662	40.08302024
30 to 34	0.014184495	2.587384754	83255.87093	4.025253736	28.65445799	0.009559093	2.564196573	87687.94052	4.284699621	36.48210859
35 to 39	0.017661421	2.549077702	77561.80208	3.71756225	25.56767293	0.010847879	2.522477	83605.84787	4.071087028	33.14120967
40 to 44	0.021431044	2.537992915	71014.25328	3.373159867	22.69039824	0.011852838	2.521889168	79198.02961	3.847027573	29.84396695
45 to 49	0.026288913	2.524162262	63802.9687	2.995618983	19.96697901	0.013623762	2.529702512	74640.69296	3.610632514	26.50485335
50 to 54	0.031418587	2.50661779	55945.34758	2.594551731	17.41368584	0.015777249	2.517868681	69724.31032	3.355099223	23.18400601
55 to 59	0.037132037	2.514763042	47816.74642	2.189569258	14.94746214	0.017402788	2.561348571	64443.40639	3.091634246	19.86801017
60 to 64	0.049293805	2.518641963	39719.4224	1.77039573	12.48610166	0.024698012	2.602492432	59091.99741	2.790531063	16.42748194
65 to 69	0.067143635	2.483112274	31031.60312	1.328238265	10.28437773	0.036527309	2.590308652	52251.50076	2.403217136	13.23168963
70 to 74	0.088806223	2.433079663	22156.63502	0.902968447	8.421247719	0.055853778	2.557036799	43567.15976	1.920276401	10.35183468
75 to 79	0.117402735	2.376040092	14173.30637	0.542348004	6.808146773	0.086572841	2.504461865	33006.36386	1.362501578	7.85695484
80 to 84	0.153761231	2.299935963	7832.78054	0.277273726	5.411629036	0.140295573	2.413464073	21471.6336	0.793997827	5.771599916
85 to 89	0.218627212	2.214979833	3595.452643	0.111998328	4.106308491	0.208143252	2.252946351	10646.04967	0.342556476	4.28332135
90 to 94	0.308902483	2.022068422	1160.135058	0.030285839	3.110230672	0.296721658	2.054418591	3717.362826	0.100590284	3.221604763
95 plus	0.387029696	2.584120336	228.4895458	0.005921306	2.584120336	0.381737313	2.623679298	808.0475749	0.021613155	2.623679298

Table 15: Lesotho 2100 life table, by age and sex. mx=mortality rate, ax=mean person-years lived in an age interval among those who die in that age interval, lx=number of persons left alive at age x, nLx=person-years lived between age x and x+n, ex=life expectancy at age x.

Age Group	Male					Female				
	mx	ax	lx	nLx	ex	mx	ax	lx	nLx	ex
Early Neonatal	0.253691122	0.009581265	100000	0.019131526	64.79542968	0.234414111	0.009581856	100000	0.019135054	74.8619711
Late Neonatal	0.021895196	0.028761084	99514.99791	0.057219176	65.08966414	0.022786855	0.028760838	99551.69886	0.057238814	75.17696241
Post Neonatal	0.008568122	0.461035171	99389.79154	0.914035261	65.11349437	0.007358011	0.461121135	99421.35783	0.914835296	75.2169395
1 to 4	0.000478369	1.999362174	98607.08713	3.940520316	64.70172517	0.000419163	1.999441116	98748.52247	3.946636248	74.80022306
5 to 9	0.000794961	2.498343832	98419.01179	4.911190131	60.82019265	0.000614602	2.49871958	98583.3484	4.921606314	70.920299
10 to 14	0.000718399	2.718611372	98028.86123	4.893412673	56.05187126	0.000519692	2.589462179	98281.06772	4.907909665	66.12952849
15 to 19	0.001544297	2.718879822	97677.58375	4.866800947	51.24319861	0.000842648	2.667614953	98026.22054	4.891998607	61.29357431
20 to 24	0.002420859	2.691790978	96926.68997	4.819537284	46.61499721	0.001491239	2.681688778	97614.75978	4.864100941	56.53641484
25 to 29	0.003944299	2.646795523	95762.24241	4.744248298	42.14085187	0.002293257	2.585306199	96892.34509	4.818154098	51.93259462
30 to 34	0.005334403	2.591060563	93900.64076	4.635561269	37.91273797	0.002463639	2.599064971	95797.69121	4.761009881	47.49245153
35 to 39	0.006072656	2.575866842	91451.60831	4.506913963	33.84945221	0.002377316	2.531582562	94641.74517	4.70471245	43.04083935
40 to 44	0.007869121	2.61855638	88747.58627	4.356646957	29.79170431	0.002796317	2.645791982	93535.58491	4.646311764	38.51297771
45 to 49	0.010962048	2.645681795	85370.18649	4.162400791	25.85138359	0.004339486	2.687794039	92245.62379	4.566275713	33.9989166
50 to 54	0.016310265	2.629368193	80888.898	3.896331384	22.12062779	0.006258424	2.651171181	90284.3213	4.449201389	29.6568124
55 to 59	0.023282922	2.590044276	74668.13248	3.539102003	18.7349612	0.008743555	2.623919938	87541.35888	4.28909809	25.47619167
60 to 64	0.031001471	2.572808788	66632.14542	3.10347115	15.685371	0.011453983	2.624000717	83871.55212	4.084811272	21.44581596
65 to 69	0.044446802	2.554111298	57245.75316	2.588843767	12.84562857	0.016659993	2.671730518	79316.43874	3.821967914	17.4995527
70 to 74	0.062499829	2.506206997	46078.3971	2.001353043	10.37169099	0.027567262	2.700211237	73193.11166	3.450898317	13.71288433
75 to 79	0.085697346	2.458455635	33936.79978	1.401588093	8.2356229	0.051851985	2.675944717	64208.0043	2.88745542	10.22716891
80 to 84	0.122250567	2.394736712	22302.34574	0.854368474	6.319364541	0.104265061	2.544941165	50466.87676	2.060022968	7.289682803
85 to 89	0.186703557	2.288266914	12268.21019	0.413418627	4.633926963	0.165850621	2.362696406	31601.74053	1.155535855	5.239389415
90 to 94	0.279729716	2.084050553	4861.647471	0.136230838	3.395384009	0.252948949	2.152920561	15342.0188	0.478017921	3.779726554
95 plus	0.364555317	2.748491417	1176.803304	0.033103704	2.748491417	0.343249005	2.968737971	4974.137866	0.165037112	2.968737971

Table 15: Namibia 2017 life table, by age and sex. mx=mortality rate, ax=mean person-years lived in an age interval among those who die in that age interval, lx=number of persons left alive at age x, nLx=person-years lived between age x and x+n, ex=life expectancy at age x.

Age Group	Male					Female				
	mx	ax	lx	nLx	ex	mx	ax	lx	nLx	ex
Early Neonatal	0.906651207	0.009561252	100000	0.019012328	62.41263434	0.506604396	0.009573514	100000	0.019085223	70.95152288
Late Neonatal	0.049199335	0.028753552	98276.49269	0.056462713	63.48726442	0.045090866	0.028754685	99033.19071	0.056904165	71.62473901
Post Neonatal	0.014917302	0.460584139	97998.78583	0.89861072	63.60952388	0.013850448	0.460659926	98776.63874	0.906187161	71.75314138
1 to 4	0.002388517	1.996815316	96658.88487	3.847954714	63.56168235	0.0021868	1.997084271	97521.80089	3.883865854	71.74726053
5 to 9	0.000885849	2.498154483	95740.33372	4.776434406	60.152589	0.00054986	2.498854458	96672.74153	4.82699938	68.36004119
10 to 14	0.001055167	2.697458219	95317.35786	4.754306448	55.4083945	0.0006764	2.64966703	96407.35618	4.812709684	63.54119382
15 to 19	0.001890071	2.670931375	94815.81083	4.720018843	50.68715597	0.001034046	2.671659739	96081.85226	4.792571698	58.74737394
20 to 24	0.002648663	2.619764669	93923.7643	4.6667874	46.14270125	0.001558217	2.702780653	95586.31972	4.762297898	54.03735527
25 to 29	0.003500739	2.64690107	92688.07433	4.596585448	41.72197333	0.00260961	2.718447975	94844.46454	4.714159843	49.43782091
30 to 34	0.005293974	2.64473238	91080.13938	4.497923435	37.41051684	0.004368521	2.6504504	93615.35233	4.633170351	45.04963972
35 to 39	0.007412854	2.611195477	88701.99911	4.358006987	33.34163678	0.005839085	2.575406079	91594.57518	4.515899271	40.98350808
40 to 44	0.009788453	2.582573062	85476.47701	4.175156153	29.50043037	0.006789297	2.532137554	88962.82434	4.374951352	37.11754946
45 to 49	0.012220202	2.578452611	81396.69647	3.953050766	25.84865164	0.007269196	2.523917149	85999.19131	4.224113908	33.3071744
50 to 54	0.01602335	2.571789998	76574.30279	3.685587869	22.31272063	0.00821188	2.522267035	82934.97939	4.064254032	29.44131984
55 to 59	0.020317341	2.584512626	70682.14574	3.369143232	18.95866139	0.008773553	2.574801368	79606.71902	3.89769961	25.56391673
60 to 64	0.029005358	2.58712271	63854.95698	2.984356285	15.71149059	0.012291745	2.629345255	76200.95265	3.702574166	21.58917258
65 to 69	0.041676117	2.564643895	55219.28137	2.507027917	12.76743004	0.018136782	2.639245237	71668.93946	3.436989027	17.78511903
70 to 74	0.061101129	2.526249412	44791.93629	1.945890696	10.14666189	0.028434754	2.629551135	65466.27864	3.067866695	14.21700355
75 to 79	0.088927012	2.471528827	32917.47268	1.343960723	7.89843877	0.045524676	2.605577307	56802.14951	2.563257161	10.98264373
80 to 84	0.131341171	2.393619811	20975.50102	0.781388178	5.98943938	0.076809186	2.551585878	45244.61293	1.908002202	8.127122269
85 to 89	0.197095986	2.262053436	10715.57472	0.348061686	4.431253209	0.13466322	2.429920686	30778.19608	1.147616497	5.767743095
90 to 94	0.289551191	2.063373867	3859.264524	0.104319874	3.286988669	0.222203933	2.224523303	15538.32963	0.483115362	4.08624879
95 plus	0.372268332	2.686382674	840.4427437	0.022587517	2.686382674	0.317160359	3.157072971	4935.272518	0.15723234	3.157072971

Table 15: Namibia 2100 life table, by age and sex. mx=mortality rate, ax=mean person-years lived in an age interval among those who die in that age interval, lx=number of persons left alive at age x, nLx=person-years lived between age x and x+n, ex=life expectancy at age x.

Age Group	Male					Female				
	mx	ax	lx	nLx	ex	mx	ax	lx	nLx	ex
Early Neonatal	0.263607758	0.009580962	100000	0.0191297	74.86531514	0.146049319	0.009584565	100000	0.019151252	83.66294336
Late Neonatal	0.013733245	0.028763335	99495.94114	0.057221643	75.22405111	0.01688415	0.028762466	99720.34527	0.057345498	83.877249
Post Neonatal	0.006574036	0.461176827	99417.40165	0.915130449	75.22555774	0.006311165	0.461195501	99623.54901	0.917138584	83.9007046
1 to 4	0.000313792	1.999581611	98816.32508	3.950177288	74.755412	0.000304795	1.999593607	99045.09844	3.959392913	83.46177153
5 to 9	0.00029762	2.499379958	98692.57203	4.930962392	70.84611237	0.000199716	2.499583926	98924.57926	4.943762658	79.56013999
10 to 14	0.000441551	2.649030353	98545.96442	4.922165512	65.94765316	0.000309166	2.576198213	98825.9463	4.937589554	74.63662327
15 to 19	0.000588914	2.636166205	98328.80213	4.909625608	61.08735018	0.000302585	2.586955834	98673.42827	4.930179938	69.74749175
20 to 24	0.000844081	2.642496612	98039.94768	4.892319476	56.25860786	0.000536854	2.706642348	98524.4926	4.920231669	64.84775741
25 to 29	0.001220888	2.629085704	97627.49103	4.867426468	51.48363041	0.000894442	2.633041596	98260.78006	4.902831568	60.01299265
30 to 34	0.001730607	2.610326444	97034.55249	4.831699559	46.77977233	0.001255168	2.631927105	97823.80252	4.876401624	55.26690622
35 to 39	0.002040937	2.609223925	96201.91374	4.78677065	42.15926822	0.001323482	2.50293095	97216.16326	4.845127971	50.59390833
40 to 44	0.002748311	2.649403738	95230.78181	4.73089014	37.55901213	0.001482207	2.624671177	96581.33203	4.811888313	45.90696231
45 to 49	0.00377231	2.693067434	93940.57771	4.656509041	33.0336014	0.001798192	2.654186934	95876.5486	4.773526814	41.22260473
50 to 54	0.00609674	2.686146495	92198.49263	4.545870171	28.60001736	0.002478772	2.646512253	95026.90859	4.723615451	36.56342209
55 to 59	0.009093298	2.668068198	89452.79299	4.380181302	24.38783788	0.003143634	2.646847406	93868.09165	4.658904532	31.97788273
60 to 64	0.013884062	2.6638247	85508.45324	4.142010513	20.38344248	0.004337424	2.671741047	92421.24201	4.574971956	27.43216779
65 to 69	0.022063705	2.652705623	79810.68323	3.795813428	16.6411658	0.006542111	2.719086167	90458.75132	4.456660855	22.96206521
70 to 74	0.035692626	2.60299034	71530.93476	3.298524166	13.25310496	0.011176949	2.75842099	87581.86726	4.272840548	18.61613325
75 to 79	0.053699087	2.569573105	59942.13518	2.658996566	10.30447712	0.021059958	2.772193343	82898.74665	3.962015597	14.4960878
80 to 84	0.088062685	2.501118117	46047.62648	1.901943118	7.645579981	0.043397137	2.721240336	74797.39422	3.414261923	10.74650234
85 to 89	0.14828134	2.387083626	30017.61511	1.098345643	5.431801116	0.086688268	2.598385424	60644.67085	2.537789779	7.574659833
90 to 94	0.242768191	2.158280632	14563.29153	0.439505848	3.824147416	0.165082121	2.316590011	40140.97403	1.423262759	5.187551619
95 plus	0.335326605	2.994269986	4350.240774	0.134096458	2.994269986	0.263876945	3.848782432	18324.89075	0.744051603	3.848782432

Table 15: South Africa 2017 life table, by age and sex. mx=mortality rate, ax=mean person-years lived in an age interval among those who die in that age interval, lx=number of persons left alive at age x, nLx=person-years lived between age x and x+n, ex=life expectancy at age x.

Age Group	Male					Female				
	mx	ax	lx	nLx	ex	mx	ax	lx	nLx	ex
Early Neonatal	0.506276671	0.009573524	100000	0.01908528	63.07865618	0.388810798	0.009577124	100000	0.019106758	69.95779283
Late Neonatal	0.059047586	0.028750835	99033.77157	0.056881662	63.67476075	0.046953475	0.028754171	99257.11533	0.057029769	70.46211535
Post Neonatal	0.013561301	0.460680466	98697.91132	0.905584691	63.83379767	0.01290917	0.460726792	98989.34416	0.908531494	70.59509909
1 to 4	0.001743193	1.997675744	97469.88821	3.88523539	63.70894382	0.001500715	1.997999048	97816.55196	3.90094259	70.51269042
5 to 9	0.000771363	2.498392994	96792.67037	4.830313128	60.14073103	0.000617491	2.498713561	97231.16447	4.854061235	66.92520282
10 to 14	0.00068271	2.732784821	96420.09445	4.813553905	55.36347378	0.000520244	2.716141003	96931.43925	4.840820199	62.12442452
15 to 19	0.001544615	2.796092429	96091.47688	4.788273317	50.54346372	0.001163354	2.806764382	96679.60216	4.821678991	57.27917511
20 to 24	0.002912656	2.730459599	95351.88258	4.736286956	45.91373207	0.002255258	2.765590003	96118.67843	4.781840268	52.59693363
25 to 29	0.004877634	2.683913197	93972.38611	4.646136237	41.54754677	0.004114688	2.679472771	95040.29282	4.707075898	48.16212851
30 to 34	0.0075059	2.619884978	91706.27706	4.504843577	37.50762953	0.005987422	2.587776196	93103.69529	4.588920022	44.10775821
35 to 39	0.009764639	2.569862322	88325.34475	4.313918898	33.84275715	0.006966381	2.53599582	90356.73605	4.441621971	40.36961185
40 to 44	0.011727317	2.53963313	84113.78037	4.087770011	30.40836594	0.007664746	2.516027856	87263.70428	4.281697146	36.71041793
45 to 49	0.01333765	2.543220525	79321.2785	3.840258838	27.09218038	0.008121196	2.523848599	83983.48288	4.116427433	33.04609202
50 to 54	0.01641062	2.549674116	74200.81545	3.566653498	23.78647009	0.009285508	2.537906054	80642.06675	3.942012894	29.3110565
55 to 59	0.019994321	2.559571561	68349.1988	3.25849077	20.60490914	0.010674403	2.565944307	76983.27308	3.751713009	25.58386719
60 to 64	0.026571012	2.56052969	61835.22776	2.90357958	17.50610048	0.01395258	2.592535784	72979.74558	3.530423874	21.84686367
65 to 69	0.035162268	2.536019022	54121.16046	2.490324537	14.6365338	0.019029335	2.594515408	68054.93155	3.253826969	18.24042462
70 to 74	0.045254776	2.519688016	45365.64962	2.039396911	11.97210468	0.026609979	2.588397787	61864.06989	2.906697198	14.80630984
75 to 79	0.061342649	2.536828599	36137.5258	1.569725449	9.386104373	0.03817605	2.625636315	54130.32813	2.481600024	11.55205446
80 to 84	0.103980079	2.492111646	26509.60261	1.051349975	6.873921126	0.07046588	2.587276793	44657.58768	1.908446957	8.445644269
85 to 89	0.168067858	2.331706372	15578.80396	0.537790789	4.948711171	0.126736851	2.452458941	31210.82419	1.179696272	5.969797661
90 to 94	0.262639315	2.120571136	6541.117413	0.186230403	3.564992152	0.213773601	2.243529747	16261.20726	0.511615177	4.203752584
95 plus	0.351355463	2.846129934	1650.317841	0.046972372	2.846129934	0.309613904	3.229857198	5325.175538	0.172006678	3.229857198

Table 15: South Africa 2100 life table, by age and sex. mx=mortality rate, ax=mean person-years lived in an age interval among those who die in that age interval, lx=number of persons left alive at age x, nLx=person-years lived between age x and x+n, ex=life expectancy at age x.

Age Group	Male					Female				
	mx	ax	lx	nLx	ex	mx	ax	lx	nLx	ex
Early Neonatal	0.155082369	0.009584288	100000	0.019149596	75.07258429	0.114533782	0.009585531	100000	0.019157037	82.33797666
Late Neonatal	0.016498067	0.028762572	99703.10426	0.05733622	75.27536985	0.014695129	0.02876307	99780.62014	0.057383771	82.4981016
Post Neonatal	0.003825686	0.461372065	99608.53453	0.918051511	75.28879644	0.003761153	0.46137665	99696.31151	0.918887801	82.50964334
1 to 4	0.000245021	1.999673305	99257.4471	3.968354363	74.62860943	0.000208533	1.999721956	99350.81835	3.972377007	81.86913106
5 to 9	7.61072E-05	2.499841444	99160.28964	4.957072893	70.69910605	6.7222E-05	2.499859955	99268.04601	4.962570011	77.93471527
10 to 14	7.23917E-05	3.109091164	99122.63064	4.955427636	65.72448297	5.5638E-05	2.961664402	99234.75926	4.961179053	72.95910988
15 to 19	0.000292854	3.329723654	99086.80905	4.951548599	60.74674225	0.000207452	3.087236775	99207.21526	4.958282668	67.97783808
20 to 24	0.000961885	2.673537074	98942.01042	4.936016519	55.82897603	0.000590976	2.629075238	99104.6671	4.948377782	63.04270282
25 to 29	0.001108901	2.541777068	98467.92586	4.910236214	51.08097034	0.000671606	2.444760514	98813.66548	4.932661064	58.2153623
30 to 34	0.001448076	2.547297561	97924.81919	4.879179782	46.3461141	0.000807101	2.557213125	98485.8213	4.914679499	53.39449558
35 to 39	0.001692674	2.694870061	97224.2137	4.841934722	41.6534657	0.00075515	2.670839281	98099.73966	4.89605589	48.586624
40 to 44	0.002450686	2.686351622	96417.62007	4.793455454	36.97121396	0.000998822	2.791876119	97736.74011	4.875581324	43.75165281
45 to 49	0.003383405	2.773177034	95259.63945	4.727228105	32.37903208	0.001569161	2.765312446	97258.617	4.845731478	38.94779227
50 to 54	0.006715062	2.735549663	93682.63791	4.614430608	27.86790789	0.002892283	2.7107099	96506.76282	4.793738112	34.22317697
55 to 59	0.011400274	2.675789253	90632.5396	4.415957019	23.69337073	0.00489465	2.645925791	95134.47901	4.703142102	29.66459635
60 to 64	0.017554578	2.621628876	85698.14412	4.116594068	19.87567909	0.006982759	2.621104476	92865.12138	4.569003715	25.29754203
65 to 69	0.025212149	2.584830043	78654.75559	3.713732891	16.38970319	0.010311272	2.64568482	89744.90237	4.383984136	21.0433269
70 to 74	0.035718066	2.566257971	69614.89766	3.215891982	13.16341498	0.017089243	2.655139797	85367.7707	4.111999995	16.93374591
75 to 79	0.051894477	2.602536834	58769.00005	2.634053013	10.11749842	0.029896142	2.695904195	78721.15213	3.70202677	13.07113057
80 to 84	0.100823647	2.523425065	46126.84409	1.878416072	7.221481854	0.063231595	2.630513093	68690.39505	3.039199479	9.533916571
85 to 89	0.162766587	2.352003132	28823.5127	1.039709068	5.165539772	0.112919137	2.517241605	52142.50323	2.113757978	6.753158781
90 to 94	0.256313732	2.129997314	13548.58884	0.406030964	3.680930122	0.194673745	2.263400379	32213.00659	1.113519285	4.690642372
95 plus	0.345891173	2.911946524	3985.749531	0.122776994	2.911946524	0.29080618	3.537743274	13814.13751	0.544680704	3.537743274

Table 15: Swaziland 2017 life table, by age and sex. mx=mortality rate, ax=mean person-years lived in an age interval among those who die in that age interval, lx=number of persons left alive at age x, nLx=person-years lived between age x and x+n, ex=life expectancy at age x.

Age Group	Male					Female				
	mx	ax	lx	nLx	ex	mx	ax	lx	nLx	ex
Early Neonatal	0.666249414	0.009568621	100000	0.019056085	55.25317512	0.490376483	0.009574011	100000	0.019088187	65.30638015
Late Neonatal	0.049132351	0.02875357	98730.47158	0.056723633	55.94405104	0.064338282	0.028749376	99064.00928	0.056890385	65.90398149
Post Neonatal	0.023789982	0.459953849	98451.8148	0.899085316	56.04473849	0.019285176	0.460273856	98698.03197	0.903203402	66.09068595
1 to 4	0.00232201	1.996903993	96313.43842	3.834713995	56.35554718	0.001788595	1.997615209	96956.58915	3.864427667	66.3463112
5 to 9	0.001641121	2.496581001	95423.71959	4.751667795	52.86249777	0.001156901	2.497589791	96265.62505	4.799388536	62.8083349
10 to 14	0.001625554	2.656523379	94644.07377	4.714207478	48.27712231	0.00120462	2.554250219	95710.46672	4.771429189	58.1576493
15 to 19	0.002861469	2.707312674	93878.01299	4.663327299	43.64924094	0.001444391	2.685807167	95135.9052	4.740983265	53.4933385
20 to 24	0.004590686	2.693452308	92543.81916	4.578745187	39.23851717	0.002559672	2.739148794	94451.19928	4.695393094	48.86062778
25 to 29	0.007419205	2.655708008	90442.64087	4.444889218	35.08464566	0.00446932	2.664705729	93249.92435	4.614315102	44.45364598
30 to 34	0.010747243	2.598834464	87148.27777	4.2478736	31.30721943	0.006226584	2.594685193	91190.01672	4.492176084	40.39701378
35 to 39	0.013527779	2.573379746	82591.29842	3.998522016	27.88919057	0.007523378	2.566579588	88396.78556	4.34045102	36.59144904
40 to 44	0.017435813	2.553549658	77192.52135	3.702029959	24.65737645	0.009232972	2.536950203	85133.9847	4.162148052	32.89286321
45 to 49	0.021019816	2.544316043	70750.8116	3.364327931	21.66623226	0.010002393	2.537154516	81293.50717	3.967111259	29.3211189
50 to 54	0.026653597	2.529298725	63697.64956	2.988719477	18.77939043	0.012227137	2.527422135	77329.5437	3.753282675	25.68524537
55 to 59	0.031639303	2.53246846	55760.06705	2.586961361	16.09178768	0.012991356	2.56727134	72752.27254	3.526722949	22.13389517
60 to 64	0.042881105	2.537281027	47614.15531	2.154410503	13.41440252	0.018642887	2.620423119	68194.42012	3.265824678	18.43353833
65 to 69	0.058703705	2.504671939	38425.78761	1.677172962	11.02213093	0.02777286	2.615056161	62150.44525	2.916231042	14.96451898
70 to 74	0.079408971	2.461939747	28642.14943	1.193190695	8.943817293	0.042974997	2.592604087	54135.13178	2.456062299	11.78981704
75 to 79	0.108449581	2.406341626	19226.00427	0.751364935	7.134730591	0.068263556	2.550128957	43727.1809	1.878174777	8.98456782
80 to 84	0.146333457	2.328021431	11128.67706	0.401131889	5.595326858	0.112789928	2.470213939	31149.71618	1.218537277	6.607357485
85 to 89	0.211581071	2.23013669	5313.539049	0.168121882	4.209804302	0.178016704	2.317621769	17740.63715	0.605677882	4.799509113
90 to 94	0.302596172	2.03549516	1787.557104	0.047299656	3.166695348	0.266994429	2.122147435	7225.588252	0.206595889	3.523504764
95 plus	0.382232804	2.616837179	366.2720692	0.009632252	2.616837179	0.356527506	2.809445108	1829.91357	0.052265443	2.809445108

Table 15: Swaziland 2100 life table, by age and sex. mx=mortality rate, ax=mean person-years lived in an age interval among those who die in that age interval, lx=number of persons left alive at age x, nLx=person-years lived between age x and x+n, ex=life expectancy at age x.

Age Group	Male					Female				
	mx	ax	lx	nLx	ex	mx	ax	lx	nLx	ex
Early Neonatal	0.202757034	0.009582827	100000	0.019140858	68.0246497	0.140441529	0.009584737	100000	0.019152285	78.68255611
Late Neonatal	0.012906501	0.028763563	99612.13584	0.057289829	68.26840407	0.031739798	0.028758368	99731.12603	0.057327231	78.87315223
Post Neonatal	0.007303981	0.461124973	99538.23465	0.915933971	68.2611134	0.00675686	0.46116384	99549.32808	0.916267324	78.9579498
1 to 4	0.000278578	1.999628562	98869.67114	3.952589282	67.79434828	0.000241483	1.999678023	98930.66888	3.955320066	78.52260289
5 to 9	0.000605164	2.498739242	98759.82571	4.930532792	63.86640156	0.000466092	2.499028975	98835.35601	4.936018356	74.59535668
10 to 14	0.000558796	2.661565635	98461.64783	4.916617774	59.05164394	0.000418511	2.504010522	98605.47962	4.925124585	69.76233361
15 to 19	0.000999443	2.70587717	98187.16972	4.898153455	54.20863678	0.000466171	2.55057945	98399.60402	4.914534252	64.90200576
20 to 24	0.001570309	2.695929619	97698.297	4.867332559	49.46357695	0.000684266	2.662941259	98171.07222	4.900728044	60.04478463
25 to 29	0.002500427	2.677720443	96935.83404	4.818732997	44.82578573	0.000992001	2.583735469	97836.99992	4.880190349	55.23817323
30 to 34	0.003579842	2.633426935	95736.86921	4.746384379	40.34470804	0.00106261	2.615505094	97355.8487	4.85522071	50.49539609
35 to 39	0.004370902	2.608980996	94050.86604	4.654237322	36.01183455	0.00122147	2.6196868	96844.47393	4.828210641	45.7448185
40 to 44	0.005981345	2.635371082	92036.60047	4.538341809	31.73141971	0.001706214	2.711429326	96259.10676	4.794200058	41.00048483
45 to 49	0.008327382	2.675807396	89367.40087	4.384414403	27.58365333	0.002830317	2.706218896	95447.13591	4.741487106	36.31776756
50 to 54	0.012822494	2.668676467	85796.76239	4.166949749	23.60653581	0.004172004	2.691864438	94120.04484	4.661161829	31.77824078
55 to 59	0.01926096	2.616702951	80575.98216	3.855254524	19.95705013	0.00636729	2.649103694	92200.24216	4.542553734	27.36693913
60 to 64	0.026164917	2.593902789	73333.50021	3.453487479	16.6744171	0.008527976	2.662588376	89357.16141	4.381611877	23.13335583
65 to 69	0.038032274	2.582951484	64495.89072	2.959118521	13.61120214	0.013377787	2.694934027	85694.07143	4.158707434	18.98873829
70 to 74	0.056317159	2.537056325	53527.05897	2.357516584	10.89375003	0.021977593	2.719323367	80287.03617	3.828128811	15.06604851
75 to 79	0.080714833	2.476059111	40579.56613	1.693611007	8.58929387	0.04152182	2.692795197	72212.94805	3.308714622	11.43034998
80 to 84	0.113448769	2.405993909	27279.09946	1.064002806	6.616361184	0.079285484	2.595523427	59284.45866	2.522355802	8.338039644
85 to 89	0.177044224	2.31183001	15693.00206	0.539868274	4.813854724	0.135797904	2.439386915	40963.44353	1.565869548	5.921812346
90 to 94	0.270676384	2.102880774	6549.939477	0.186997535	3.492174594	0.221587909	2.219897718	22053.36752	0.714052105	4.182765626
95 plus	0.357485646	2.804093731	1672.6749	0.048141793	2.804093731	0.315767076	3.21886114	7903.126289	0.275619341	3.21886114

Table 15: Zimbabwe 2017 life table, by age and sex. mx=mortality rate, ax=mean person-years lived in an age interval among those who die in that age interval, lx=number of persons left alive at age x, nLx=person-years lived between age x and x+n, ex=life expectancy at age x.

Age Group	Male					Female				
	mx	ax	lx	nLx	ex	mx	ax	lx	nLx	ex
Early Neonatal	1.02216433	0.009557712	100000	0.018991344	58.49478973	0.814745719	0.009564069	100000	0.019029037	64.72823229
Late Neonatal	0.106639543	0.028737707	98059.00282	0.056244834	59.63254348	0.073999932	0.02874671	98449.75089	0.056521937	65.72787887
Post Neonatal	0.021312654	0.46012983	97459.37213	0.891037993	59.94161379	0.018115466	0.460356949	98031.5695	0.897587978	65.9505894
1 to 4	0.003403081	1.995462573	95561.08138	3.796558289	60.1998749	0.003222471	1.995703385	96406.00821	3.831502547	66.13174657
5 to 9	0.001328085	2.497233159	94269.79002	4.697878899	56.99738394	0.001039438	2.497834505	95171.7908	4.746247226	62.96380815
10 to 14	0.001369247	2.673935369	93646.06289	4.667424745	52.3603198	0.000957369	2.576160554	94678.53176	4.722954905	58.27857603
15 to 19	0.002485127	2.680143285	93007.16692	4.623704689	47.70161616	0.001387848	2.654235737	94226.46254	4.696048502	53.54564264
20 to 24	0.003582956	2.608026472	91858.32001	4.553904508	43.26428734	0.002020087	2.643382285	93574.78071	4.656576177	48.89947914
25 to 29	0.004480408	2.599218278	90227.01661	4.463372165	38.99855968	0.0028243	2.645559037	92634.34971	4.601117668	44.36839841
30 to 34	0.005956728	2.62070304	88228.04519	4.349767553	34.82223608	0.004063653	2.6440802	91335.66797	4.523446744	39.96102156
35 to 39	0.008305691	2.62403768	85638.84405	4.199127029	30.79479235	0.005751422	2.623153358	89498.96843	4.414621773	35.72640415
40 to 44	0.011658673	2.607533722	82154.11795	3.996335743	26.98820031	0.007805535	2.597791054	86961.48617	4.268098424	31.69122708
45 to 49	0.015775055	2.595727691	77499.48706	3.733545235	23.45021521	0.010062677	2.590875539	83631.79057	4.082705898	27.84714457
50 to 54	0.02168059	2.562959328	71616.86436	3.401429002	20.15968998	0.013341083	2.563451816	79526.38567	3.851272894	24.14686809
55 to 59	0.026884443	2.560225981	64256.73749	3.015530885	17.17315631	0.015798789	2.588294641	74395.97394	3.583560752	20.63172981
60 to 64	0.037736217	2.557035003	56171.4161	2.572140872	14.27636197	0.023134886	2.612723306	68747.89516	3.257998977	17.11071198
65 to 69	0.052273845	2.523968042	46494.3076	2.059177104	11.71750739	0.034438895	2.592188786	61234.75177	2.828234669	13.88688976
70 to 74	0.072409734	2.481248901	35770.0522	1.513731874	9.47853306	0.051808386	2.555585058	51538.92725	2.288910602	11.01103043
75 to 79	0.098901316	2.426249863	24860.25144	0.992078766	7.55845583	0.077547777	2.50535678	39757.29114	1.668101131	8.521226271
80 to 84	0.135078876	2.355866662	15108.34017	0.557631564	5.888552228	0.118280518	2.428910627	26938.99832	1.03563158	6.399362616
85 to 89	0.200333982	2.25484563	7623.830961	0.246469624	4.380874334	0.183973853	2.30300366	14824.29299	0.497354007	4.677232652
90 to 94	0.29247584	2.057118355	2712.738866	0.073054902	3.259667958	0.273039664	2.108103366	5772.034745	0.162045458	3.452616161
95 plus	0.374506561	2.670601379	584.5611329	0.015652116	2.670601379	0.36178559	2.765768217	1388.372321	0.038663403	2.765768217

Table 15: Zimbabwe 2100 life table, by age and sex. mx=mortality rate, ax=mean person-years lived in an age interval among those who die in that age interval, lx=number of persons left alive at age x, nLx=person-years lived between age x and x+n, ex=life expectancy at age x.

Age Group	Male					Female				
	mx	ax	lx	nLx	ex	mx	ax	lx	nLx	ex
Early Neonatal	0.351567765	0.009578266	100000	0.019113603	69.85988803	0.33620839	0.009578736	100000	0.019116407	76.16386251
Late Neonatal	0.039108953	0.028756335	99328.47118	0.057083689	70.30592854	0.032707477	0.028758101	99357.61228	0.057110942	76.62899412
Post Neonatal	0.006822864	0.461159151	99105.40465	0.912157264	70.40421854	0.007108563	0.461138857	99170.97068	0.912644341	76.7132172
1 to 4	0.000718964	1.999041381	98484.4154	3.933733546	69.91182455	0.000730275	1.9990263	98524.72127	3.935262366	76.27230892
5 to 9	0.000523605	2.498909156	98202.42921	4.903715058	66.10295645	0.000434302	2.499095205	98238.58444	4.906614822	72.48257297
10 to 14	0.000586498	2.731993124	97946.30313	4.890792629	61.26681762	0.000390257	2.550525319	98026.09769	4.89666245	67.63161772
15 to 19	0.001153066	2.706344792	97660.11269	4.870177762	56.43615321	0.000557326	2.692247168	97835.68755	4.885589224	62.75559611
20 to 24	0.001753753	2.632728701	97100.51434	4.83509129	51.7395691	0.000957251	2.625705735	97565.45789	4.867380507	57.91535122
25 to 29	0.002359013	2.569146636	96256.52251	4.785847098	47.15968034	0.001239122	2.548007649	97103.08961	4.840690504	53.16986873
30 to 34	0.002845323	2.585167839	95137.54696	4.724797173	42.66742637	0.001352276	2.584880381	96509.83072	4.809725591	48.47096395
35 to 39	0.003484341	2.631996544	93815.78186	4.653304638	38.21094104	0.001494047	2.646659198	95870.6144	4.777028233	43.76530465
40 to 44	0.005245888	2.663267061	92234.10908	4.557302918	33.78990817	0.002403487	2.727582818	95171.14981	4.732962913	39.05054619
45 to 49	0.007528384	2.709767628	89924.80919	4.421989778	29.54692649	0.00392585	2.71798514	94057.65063	4.661586938	34.45562757
50 to 54	0.012041351	2.658787774	86744.75504	4.222583422	25.48995412	0.005932404	2.6814537	92281.53241	4.552812811	30.03146503
55 to 59	0.016317646	2.62777356	81915.13259	3.949737205	21.80846754	0.008836666	2.651049922	89670.88018	4.395249866	25.78965575
60 to 64	0.023261881	2.603680972	75813.68743	3.599302951	18.33999467	0.011952966	2.648625969	85962.88938	4.185970421	21.73950204
65 to 69	0.032750271	2.581237425	67856.04703	3.156881707	15.18718784	0.018727057	2.675700391	81222.44571	3.902207004	17.80377449
70 to 74	0.04712495	2.541709697	58125.99907	2.624002251	12.32493792	0.030487089	2.644605837	74462.4234	3.495412624	14.13397307
75 to 79	0.065002259	2.507738213	46649.6927	2.034999394	9.802202839	0.050550383	2.635788779	64861.70203	2.940002295	10.81813599
80 to 84	0.093638247	2.458664058	34706.86538	1.432056406	7.464035156	0.090878282	2.535753315	52155.6543	2.198081714	7.888453089
85 to 89	0.15386656	2.373691468	22709.54706	0.833791306	5.330960897	0.148779124	2.407254892	35491.75867	1.348748025	5.64195641
90 to 94	0.247973464	2.147389495	11114.95182	0.336918193	3.76986107	0.234994869	2.190730311	18862.98484	0.608726479	4.018082724
95 plus	0.339378551	2.963043506	3364.926075	0.104508753	2.963043506	0.327445881	3.116805583	6713.498157	0.234276031	3.116805583

Table 15: Western Sub-Saharan Africa 2017 life table, by age and sex. mx=mortality rate, ax=mean person-years lived in an age interval among those who die in that age interval, lx=number of persons left alive at age x, nLx=person-years lived between age x and x+n, ex=life expectancy at age x.

Age Group	Male					Female				
	mx	ax	lx	nLx	ex	mx	ax	lx	nLx	ex
Early Neonatal	1.475061924	0.009543831	100000	0.018909368	61.83678951	1.069919264	0.009556248	100000	0.018982668	65.50945414
Late Neonatal	0.128992233	0.028731541	97210.89696	0.055722554	63.59069574	0.099894413	0.028739568	97969.07464	0.056204115	66.84768678
Post Neonatal	0.031108895	0.459433942	96492.20858	0.878229822	64.00642244	0.02784228	0.459665988	97407.67432	0.887893047	67.17516508
1 to 4	0.010537058	1.985951009	93760.60483	3.6725054	64.93414023	0.009856544	1.986858284	94935.85984	3.723562958	67.98871248
5 to 9	0.001281401	2.497330416	89891.9148	4.480230767	63.64332963	0.000970339	2.497978461	91266.46122	4.552272452	66.64252487
10 to 14	0.000885309	2.499156506	89317.92936	4.456032884	59.03633703	0.000670667	2.498453308	90824.79427	4.533636264	61.9544478
15 to 19	0.001292262	2.669048423	88923.48796	4.432825205	54.28709192	0.00097524	2.671044304	90520.76545	4.515784806	57.15404532
20 to 24	0.001956031	2.634133186	88350.7261	4.397191712	49.6215592	0.001485434	2.676705113	90080.40826	4.488534013	52.42015238
25 to 29	0.002592668	2.624889863	87490.75486	4.347772039	45.08313919	0.002264161	2.659158608	89413.75688	4.447119823	47.79066462
30 to 34	0.003589677	2.625885637	86363.7429	4.281702661	40.6368756	0.003271086	2.629000947	88407.05502	4.38633274	43.30414037
35 to 39	0.004899655	2.616786896	84827.16589	4.192413575	36.32508789	0.004394044	2.595385567	86972.6312	4.303172724	38.97455889
40 to 44	0.006589284	2.6059194	82773.78202	4.074433396	32.16094273	0.00547144	2.577693145	85082.44875	4.198492911	34.78218814
45 to 49	0.008710613	2.601694421	80090.06392	3.922592858	28.15083407	0.006740946	2.585891606	82786.07682	4.073052095	30.67476585
50 to 54	0.011670738	2.602667398	76674.80141	3.729454258	24.28817722	0.00875462	2.595283129	80041.604	3.919617107	26.6368699
55 to 59	0.015962458	2.620105028	72324.89521	3.483986324	20.59172863	0.011579074	2.626367977	76612.39616	3.728245153	22.71184541
60 to 64	0.02392698	2.611815152	66767.94851	3.158099633	17.08689241	0.017423815	2.63391035	72299.71384	3.472019242	18.90879669
65 to 69	0.035237118	2.585965015	59218.73127	2.729070523	13.93185648	0.026300389	2.62258531	66258.15266	3.118237237	15.39180417
70 to 74	0.052449771	2.544115709	49614.11792	2.198055636	11.12845883	0.041152877	2.592583867	58070.63213	2.642311339	12.19175329
75 to 79	0.075249256	2.496171578	38105.23989	1.603829561	8.722645331	0.06388235	2.546278849	47220.61482	2.041985537	9.398112938
80 to 84	0.11172706	2.434288745	26066.21496	1.013686442	6.602675248	0.101088667	2.475410965	34218.61509	1.364281433	7.005490657
85 to 89	0.176114187	2.311775864	14776.60586	0.50197126	4.796557426	0.164509872	2.348637448	20486.29904	0.714260526	5.05242657
90 to 94	0.270194909	2.104633666	5962.684147	0.167472383	3.483474779	0.25350064	2.153351662	8787.28036	0.255679916	3.670549769
95 plus	0.357267601	2.799369598	1448.129796	0.040601248	2.799369598	0.344939126	2.899937385	2330.509408	0.067776027	2.899937385

Table 15: Western Sub-Saharan Africa 2100 life table, by age and sex. mx=mortality rate, ax=mean person-years lived in an age interval among those who die in that age interval, lx=number of persons left alive at age x, nLx=person-years lived between age x and x+n, ex=life expectancy at age x.

Age Group	Male					Female				
	mx	ax	lx	nLx	ex	mx	ax	lx	nLx	ex
Early Neonatal	0.245137231	0.009581528	100000	0.019133077	77.80176445	0.214111649	0.009582479	100000	0.019138765	81.73940149
Late Neonatal	0.020114429	0.028761575	99531.05906	0.057231326	78.1486143	0.021419709	0.028761215	99590.27244	0.057263225	82.05603444
Post Neonatal	0.00359884	0.46138818	99415.95621	0.916372119	78.18142874	0.003418869	0.461400965	99467.63428	0.916924597	82.09953517
1 to 4	0.000915566	1.998779245	99086.20366	3.956200862	77.5165802	0.000851916	1.998864113	99154.18321	3.959418622	81.43408789
5 to 9	0.000223812	2.499533725	98724.06897	4.933443032	73.79331908	0.000175593	2.499634181	98816.94671	4.938679444	77.70489275
10 to 14	0.000195983	2.668069677	98613.67332	4.928430021	68.87307337	0.000156042	2.59127397	98730.24402	4.934657312	72.7708843
15 to 19	0.000381113	2.738646999	98517.0987	4.921615004	63.93794123	0.000243857	2.679215732	98653.25755	4.929877884	67.82560415
20 to 24	0.000635483	2.654235634	98329.55717	4.909162707	59.05449043	0.000370877	2.705073646	98533.06375	4.922479134	62.90497601
25 to 29	0.000857246	2.625725092	98017.65128	4.890940653	54.23359562	0.000625209	2.656653817	98350.54858	4.910343809	58.01650662
30 to 34	0.001170848	2.604574131	97598.51204	4.866273834	49.45475916	0.000851848	2.612505881	98043.71816	4.892201098	53.18949216
35 to 39	0.001448627	2.596691639	97029.05254	4.834621697	44.72918945	0.001049091	2.598724053	97627.37551	4.869078811	48.40501561
40 to 44	0.001854147	2.642689472	96329.248	4.795497982	40.03445813	0.001323409	2.648212358	97117.14204	4.840756769	43.64524006
45 to 49	0.00273656	2.683960573	95440.98035	4.742032905	35.38100358	0.001962555	2.674441964	96477.18053	4.801941072	38.9162679
50 to 54	0.004374125	2.688427117	94144.88471	4.660188578	30.82850513	0.002994907	2.662208116	95535.56852	4.743602664	34.27146079
55 to 59	0.006892305	2.680543511	92111.20336	4.533239987	26.44571197	0.004393848	2.658591549	94116.8812	4.657972774	29.74470857
60 to 64	0.010842298	2.674724326	88997.64735	4.340812633	22.27272246	0.00650222	2.649748657	92075.38705	4.534663102	25.34121686
65 to 69	0.017267215	2.646082018	84314.98103	4.051816275	18.356564	0.009523587	2.670740829	89136.98712	4.360414169	21.08344123
70 to 74	0.026030441	2.634638954	77362.90153	3.645188014	14.76582967	0.015252373	2.699340418	85005.69406	4.106925201	16.97273699
75 to 79	0.042456745	2.605023335	67943.01389	3.086303424	11.44654079	0.027286006	2.71063145	78780.6673	3.709325137	13.09483758
80 to 84	0.068159724	2.538775146	54971.67287	2.357997655	8.537096907	0.053870973	2.625799563	68761.6142	3.053209425	9.602877683
85 to 89	0.124377645	2.451728335	39102.38302	1.48988888	5.983970492	0.103387592	2.530784588	52567.8994	2.102425076	6.75444061
90 to 94	0.219104774	2.203536422	20833.65537	0.649111028	4.12115592	0.186955548	2.295096465	31265.17979	1.045597185	4.670912612
95 plus	0.316374917	3.164300304	6779.562699	0.216262759	3.164300304	0.284823573	3.521893611	12099.19511	0.432506218	3.521893611

Table 15: Benin 2017 life table, by age and sex. mx=mortality rate, ax=mean person-years lived in an age interval among those who die in that age interval, lx=number of persons left alive at age x, nLx=person-years lived between age x and x+n, ex=life expectancy at age x.

Age Group	Male					Female				
	mx	ax	lx	nLx	ex	mx	ax	lx	nLx	ex
Early Neonatal	1.456339562	0.009544405	100000	0.018912766	62.86448531	1.06450178	0.009556414	100000	0.018983657	66.82319483
Late Neonatal	0.078689002	0.028745417	97246.09984	0.055823394	64.62421709	0.084772883	0.028743739	97979.35654	0.056234462	68.1812677
Post Neonatal	0.028402185	0.459626218	96806.98669	0.882197695	64.85962388	0.02312739	0.460000917	97502.73718	0.890689824	68.45679516
1 to 4	0.008261278	1.988985173	94303.70982	3.710572894	65.64465039	0.007690447	1.989746255	95443.39089	3.759647579	69.00056766
5 to 9	0.001094107	2.497720611	91242.03261	4.549657893	63.78259036	0.00089297	2.498139645	92554.43513	4.617408778	67.09263457
10 to 14	0.000819935	2.563290026	90744.73966	4.528195566	59.11888487	0.000640589	2.531147305	92142.22456	4.599839123	62.38127132
15 to 19	0.001355055	2.680134302	90373.66912	4.504532813	54.35107483	0.000997143	2.657648943	91847.62577	4.581685747	57.57302269
20 to 24	0.002019721	2.600253834	89763.41013	4.466525308	49.70156773	0.001410389	2.624501313	91390.84031	4.554289102	52.84699157
25 to 29	0.002361913	2.577213291	88861.54009	4.417804667	45.17836722	0.001863213	2.61074774	90748.67572	4.517330692	48.20152412
30 to 34	0.002953949	2.600723824	87818.53583	4.360043106	40.68322951	0.002437724	2.621655903	89907.31363	4.469461605	43.62714757
35 to 39	0.003887265	2.617608435	86531.3367	4.286902934	36.24847891	0.00335165	2.608010129	88818.28896	4.405617942	39.12870097
40 to 44	0.005329812	2.639217688	84866.112	4.190641956	31.90673255	0.004295123	2.608985181	87342.58772	4.322776264	34.74404974
45 to 49	0.007803306	2.647646052	82634.8891	4.057387463	27.6942069	0.005811851	2.632138037	85487.47125	4.21642439	30.43900663
50 to 54	0.011599942	2.640033244	79473.53873	3.868022311	23.68675559	0.008393838	2.63194917	83039.87069	4.071214524	26.25566693
55 to 59	0.017151601	2.632361419	74997.46787	3.603963628	19.93809134	0.011915326	2.64568439	79629.15051	3.873078011	22.26370594
60 to 64	0.025908191	2.6050335	68837.48724	3.241601854	16.48265651	0.018478058	2.636629039	75026.83295	3.594919832	18.4634202
65 to 69	0.037504584	2.582149279	60478.33606	2.77386232	13.39900116	0.027944985	2.619038984	68410.46892	3.20815266	14.99086971
70 to 74	0.056755066	2.540164082	50139.34505	2.201899082	10.63224262	0.043663282	2.587505039	59494.25455	2.693018898	11.844376
75 to 79	0.081903925	2.484818183	37738.45346	1.5671389	8.301795598	0.067736132	2.538645501	47821.29526	2.051922265	9.10900667
80 to 84	0.121796454	2.412947646	25021.48503	0.953561635	6.279653702	0.107169396	2.462648405	34042.60763	1.34124697	6.782359544
85 to 89	0.186949173	2.285816076	13513.18316	0.44955254	4.604024331	0.171537068	2.331786391	19813.7957	0.681939	4.912717185
90 to 94	0.280232855	2.083282192	5174.420274	0.142797487	3.380029997	0.260592055	2.136896328	8230.522711	0.236675592	3.589488066
95 plus	0.365065138	2.73992384	1195.788804	0.032887094	2.73992384	0.351079779	2.850013961	2114.501783	0.060637913	2.850013961

Table 15: Benin 2100 life table, by age and sex. mx=mortality rate, ax=mean person-years lived in an age interval among those who die in that age interval, lx=number of persons left alive at age x, nLx=person-years lived between age x and x+n, ex=life expectancy at age x.

Age Group	Male					Female				
	mx	ax	lx	nLx	ex	mx	ax	lx	nLx	ex
Early Neonatal	0.23328764	0.009581891	100000	0.01913525	77.49036149	0.188067989	0.009583277	100000	0.019143543	82.02136553
Late Neonatal	0.012009273	0.028763811	99553.68742	0.057257683	77.81782034	0.024917669	0.02876025	99640.05066	0.05728609	82.29802123
Post Neonatal	0.002864892	0.461440318	99484.93376	0.917318768	77.8139923	0.002743487	0.461448943	99497.35411	0.917484537	82.35831071
1 to 4	0.000748951	1.999001399	99222.23018	3.962953291	77.09479009	0.000698751	1.999068332	99245.69945	3.964288782	81.64231252
5 to 9	0.000155527	2.499675986	98925.59669	4.944357977	73.31938994	0.000131508	2.499726024	98968.89121	4.94681865	77.86445321
10 to 14	0.000158822	2.792689763	98848.73278	4.940701846	68.37437061	0.000135231	2.688229854	98903.86219	4.943646549	72.91394104
15 to 19	0.000376066	2.74922255	98770.28747	4.934338211	63.42639023	0.000252452	2.644873918	98837.03166	4.9389176	67.96137051
20 to 24	0.000609673	2.624201715	98584.78055	4.922105646	58.54025721	0.000312913	2.634875518	98712.38555	4.931973137	63.04374697
25 to 29	0.000736528	2.608672279	98284.81006	4.905604365	53.71035437	0.000458842	2.648653475	98558.11367	4.922597916	58.13817168
30 to 34	0.001000099	2.610790825	97923.68033	4.884507271	48.89813792	0.000643437	2.633091909	98332.35234	4.909120591	53.26534462
35 to 39	0.001266074	2.628264864	97435.49944	4.857211881	44.12914106	0.000849727	2.623311876	98016.67286	4.890952891	48.42817225
40 to 44	0.001809963	2.677029196	96821.03708	4.820807643	39.39122147	0.001145236	2.687029881	97601.33034	4.867167856	43.6226171
45 to 49	0.002847673	2.707406339	95949.59175	4.766392749	34.72244725	0.001881613	2.706695546	97044.30137	4.831369462	38.85668507
50 to 54	0.004726249	2.709857941	94595.00683	4.679105572	30.17737277	0.003044616	2.672529497	96135.96833	4.772982744	34.19661769
55 to 59	0.007750261	2.668940969	92390.22488	4.537709292	25.82835391	0.004471166	2.649562898	94684.83419	4.685030971	29.67681736
60 to 64	0.01154696	2.667097073	88887.74594	4.328354707	21.73530407	0.006408313	2.646978183	92594.41418	4.561137506	25.28336397
65 to 69	0.018679312	2.652446318	83918.1536	4.020804811	17.85736293	0.009504086	2.678740872	89679.39433	4.387494569	21.01341667
70 to 74	0.02885938	2.624099381	76476.20961	3.580675244	14.33267387	0.015646629	2.697358959	85528.67376	4.128517871	16.89697215
75 to 79	0.045533912	2.590838613	66252.66645	2.989084983	11.13930684	0.027507463	2.70932067	79113.07785	3.723029707	13.0414464
80 to 84	0.07224475	2.518149522	52833.11821	2.245760173	8.32017775	0.054572877	2.613896298	68974.79864	3.056724149	9.555134284
85 to 89	0.129465965	2.437117463	36878.19886	1.391144226	5.850093098	0.104331401	2.527664751	52559.46152	2.098193384	6.723823259
90 to 94	0.224341129	2.194338319	19194.03302	0.592753925	4.048865579	0.188028256	2.292940973	31130.67599	1.03938842	4.652815238
95 plus	0.320634399	3.122954692	6094.707298	0.192275972	3.122954692	0.285812229	3.510597258	11997.41123	0.428098603	3.510597258

Table 15: Burkina Faso 2017 life table, by age and sex. mx=mortality rate, ax=mean person-years lived in an age interval among those who die in that age interval, lx=number of persons left alive at age x, nLx=person-years lived between age x and x+n, ex=life expectancy at age x.

Age Group	Male					Female				
	mx	ax	lx	nLx	ex	mx	ax	lx	nLx	ex
Early Neonatal	1.151053074	0.009553762	100000	0.018967978	59.1163676	0.835036487	0.009563447	100000	0.019025343	64.55363912
Late Neonatal	0.145802641	0.028726904	97817.07161	0.056043018	60.41387853	0.11298612	0.028735956	98411.41982	0.056436714	65.57550629
Post Neonatal	0.039765493	0.458819027	97000.29429	0.879361169	60.86415334	0.032952576	0.459302976	97774.09598	0.889145809	65.94461257
1 to 4	0.013626734	1.981831963	93506.16995	3.640266721	62.19675259	0.011765935	1.984312683	94845.15159	3.705974938	67.04315173
5 to 9	0.001339526	2.497209324	88552.76834	4.41286495	61.56765932	0.001041904	2.497829368	90488.07901	4.512644457	66.17728546
10 to 14	0.000979126	2.569606269	87962.49358	4.387691078	56.96460626	0.000730731	2.513330661	90018.10897	4.492744604	61.5096372
15 to 19	0.00168193	2.680668468	87533.25757	4.359665099	52.23144484	0.001098297	2.650408852	89689.90832	4.472957323	56.72541965
20 to 24	0.002471726	2.593342613	86800.27937	4.314356316	47.64952111	0.001539653	2.610066609	89198.73559	4.443591076	52.02289705
25 to 29	0.002842838	2.573739348	85734.23894	4.257357806	43.20917572	0.001937222	2.594925192	88514.74698	4.405218339	47.40422385
30 to 34	0.003565933	2.60612958	84524.44881	4.190469637	38.79029073	0.002461157	2.621677832	87661.64857	4.357584529	42.83973178
35 to 39	0.004804407	2.623428502	83030.9144	4.104711031	34.44060046	0.003444943	2.613087139	86589.60372	4.294186198	38.33704224
40 to 44	0.006679134	2.637709645	81060.05711	3.990093424	30.21343633	0.004454257	2.618419362	85110.95876	4.210900422	33.95682896
45 to 49	0.009749677	2.638167487	78396.88612	3.831693153	26.14880061	0.00620588	2.640498658	83236.28875	4.101793844	29.661519
50 to 54	0.014250479	2.622557762	74664.50401	3.611030201	22.32225778	0.009101518	2.632724046	80692.41106	3.949599505	25.51196161
55 to 59	0.020395621	2.611512061	69525.30124	3.315036995	18.77627381	0.012926881	2.642416199	77101.09352	3.741182324	21.57578567
60 to 64	0.02992098	2.590064574	62776.08539	2.928139036	15.51265855	0.020000726	2.635679362	72271.50833	3.450702114	17.8391638
65 to 69	0.043129848	2.561855107	54035.76463	2.44542009	12.6025837	0.030614238	2.610636581	65383.89734	3.046903295	14.43914226
70 to 74	0.063191698	2.520636283	43522.01171	1.882329627	10.03047611	0.047071137	2.578485888	56082.16524	2.518127649	11.40097904
75 to 79	0.091238741	2.461375183	31673.364	1.287012153	7.846197142	0.073624372	2.523053555	44273.72392	1.873555156	8.757240411
80 to 84	0.130786923	2.383502085	19985.89846	0.745510852	6.007552991	0.112985626	2.442567491	30541.39043	1.186161917	6.568547124
85 to 89	0.196278876	2.263993301	10282.56662	0.335041591	4.445475076	0.178152056	2.315902991	17207.84639	0.583074958	4.780966537
90 to 94	0.288797809	2.064986164	3733.510762	0.101204396	3.294631905	0.267269509	2.121399831	6870.555202	0.194564163	3.51304858
95 plus	0.371684491	2.690776429	819.7081743	0.022101276	2.690776429	0.356863433	2.80293629	1691.819937	0.047564444	2.80293629

Table 15: Burkina Faso 2100 life table, by age and sex. mx=mortality rate, ax=mean person-years lived in an age interval among those who die in that age interval, lx=number of persons left alive at age x, nLx=person-years lived between age x and x+n, ex=life expectancy at age x.

Age Group	Male					Female				
	mx	ax	lx	nLx	ex	mx	ax	lx	nLx	ex
Early Neonatal	0.171366849	0.009583789	100000	0.019146605	75.8101949	0.14502444	0.009584596	100000	0.019151439	81.6008288
Late Neonatal	0.018207127	0.028762101	99671.93705	0.057315477	76.04012417	0.019695437	0.02876169	99722.28502	0.057341978	81.80849461
Post Neonatal	0.004193654	0.461345925	99567.60006	0.917518417	76.06210683	0.003641359	0.46138516	99609.38372	0.918137257	81.84342184
1 to 4	0.001236453	1.998351396	99182.95415	3.95752858	75.43144361	0.000958285	1.998722287	99275.15176	3.963408416	81.19366103
5 to 9	0.00016379	2.499658772	98693.91872	4.93267705	71.79480902	0.000117661	2.499754872	98895.53408	4.943323196	77.49704089
10 to 14	0.000176702	2.855599095	98613.175	4.928782339	66.85149922	0.000122395	2.703527346	98837.39978	4.940478642	72.54109285
15 to 19	0.000457001	2.73377928	98526.10895	4.921202083	61.9080363	0.000234279	2.629456668	98776.95668	4.936105926	67.58378061
20 to 24	0.000684406	2.611114289	98301.24977	4.907039511	57.04324973	0.000267003	2.600983761	98661.35176	4.929909648	62.6598157
25 to 29	0.000824516	2.622660373	97965.4886	4.888692726	52.22937368	0.000363459	2.646902975	98529.76559	4.922276601	57.73993974
30 to 34	0.001176505	2.637170926	97562.52467	4.864606374	47.43371715	0.000521275	2.656466415	98350.938	4.911542062	52.83992017
35 to 39	0.001612751	2.64780148	96990.41405	4.83120346	42.69713547	0.000750789	2.659777982	98095.02663	4.896147796	47.97058096
40 to 44	0.002353119	2.663673975	96211.65507	4.784311099	38.02003603	0.00109919	2.708792409	97727.58467	4.874106606	43.14051413
45 to 49	0.003541643	2.702285206	95086.7131	4.715990249	33.43627154	0.001866874	2.714692833	97192.06667	4.838954396	38.36260373
50 to 54	0.005941072	2.699740222	93419.04482	4.608001612	28.98116104	0.003051815	2.683554937	96289.27002	4.7806656	33.69557074
55 to 59	0.00955862	2.658392787	90687.87832	4.435355832	24.76774875	0.004634488	2.657944269	94831.68248	4.690691166	29.17029157
60 to 64	0.014170904	2.651140628	86462.51397	4.184372908	20.84239032	0.006764802	2.66154544	92660.82801	4.561034529	24.78891213
65 to 69	0.022069588	2.613898377	80565.25565	3.827857056	17.16728518	0.010459469	2.676467579	89581.60685	4.373070102	20.54524018
70 to 74	0.031264653	2.612867257	72174.13094	3.360341705	13.85497617	0.016932206	2.698362576	85023.39669	4.092398632	16.49788239
75 to 79	0.051352268	2.570467518	61778.12499	2.750323184	10.74628472	0.030572966	2.686820608	78130.56923	3.65030066	12.70870718
80 to 84	0.07574262	2.495168679	47850.05317	2.016558357	8.136046188	0.057190665	2.593423953	67068.60692	2.952577682	9.35621318
85 to 89	0.133752658	2.424826044	32837.05689	1.227356487	5.737953953	0.108034691	2.514760845	50421.57656	1.995456073	6.589505695
90 to 94	0.228743832	2.18653751	16723.11985	0.512130388	3.988366411	0.192386434	2.285275864	29269.24137	0.9680233	4.57192845
95 plus	0.324213706	3.088355717	5186.912023	0.161883404	3.088355717	0.289870163	3.459889947	10991.37928	0.385727092	3.459889947

Table 15: Cameroon 2017 life table, by age and sex. mx=mortality rate, ax=mean person-years lived in an age interval among those who die in that age interval, lx=number of persons left alive at age x, nLx=person-years lived between age x and x+n, ex=life expectancy at age x.

Age Group	Male					Female				
	mx	ax	lx	nLx	ex	mx	ax	lx	nLx	ex
Early Neonatal	0.905052074	0.009561301	100000	0.019012617	61.73357724	0.750360951	0.009566043	100000	0.019040758	65.73268855
Late Neonatal	0.095916895	0.028740665	98279.48497	0.056388674	62.79455746	0.071626781	0.028747365	98571.37619	0.056595626	66.66587904
Post Neonatal	0.024018691	0.459937603	97738.79063	0.892480963	63.084241	0.022164073	0.460069349	98166.0824	0.897146122	66.88348012
1 to 4	0.006454303	1.991394362	95596.14485	3.774941399	63.56400094	0.005662261	1.992450385	96178.18805	3.803906446	67.33251217
5 to 9	0.001096722	2.497715163	93161.52587	4.645332555	61.17404249	0.000969253	2.497980725	94025.33037	4.689894554	64.82902632
10 to 14	0.000935627	2.624261943	92652.24463	4.62234016	56.49647109	0.000761717	2.561840254	93570.82091	4.66986843	60.1314522
15 to 19	0.001670743	2.672821863	92219.86148	4.593142248	51.74883162	0.001204406	2.660222742	93215.15012	4.647669939	55.35096567
20 to 24	0.002356808	2.618366428	91452.57645	4.547111323	47.15973309	0.001712591	2.68811045	92655.44656	4.61452113	50.66882475
25 to 29	0.003067837	2.627502777	90381.16946	4.486421479	42.68637116	0.002806651	2.682269561	91865.36524	4.563582925	46.08066191
30 to 34	0.004349964	2.636310238	89005.40016	4.404985814	38.3042725	0.004245739	2.639570846	90585.26053	4.48429391	41.69295476
35 to 39	0.006104456	2.620906591	87090.51857	4.292223067	34.0872555	0.005774789	2.584174829	88682.88442	4.373163021	37.52993273
40 to 44	0.008277493	2.602779068	84472.58353	4.141526082	30.06097333	0.006879623	2.554010518	86159.44237	4.236737113	33.55215888
45 to 49	0.010916464	2.595527807	81047.85486	3.948887928	26.21856703	0.008049029	2.565439133	83246.73741	4.082438765	29.63374646
50 to 54	0.014611514	2.59595798	76742.6613	3.707186156	22.53995191	0.010164679	2.57980161	79963.63868	3.902368877	25.74065985
55 to 59	0.020002015	2.602807504	71337.9212	3.404148337	19.04661476	0.013060011	2.616032141	76004.83965	3.685821145	21.94160172
60 to 64	0.029087248	2.590200607	64550.36267	3.016939152	15.77227866	0.019538243	2.624614968	71206.41987	3.403073374	18.23897094
65 to 69	0.041884525	2.564585962	55813.82707	2.533746449	12.83412409	0.02911251	2.609867836	64588.58523	3.020591402	14.83452026
70 to 74	0.061395651	2.522414745	45265.75222	1.966460462	10.23011711	0.044723964	2.580384249	55855.00701	2.522174879	11.74493905
75 to 79	0.087503225	2.468310511	33285.87276	1.364878119	8.015236267	0.068688071	2.534776915	44676.40646	1.91333298	9.044234778
80 to 84	0.127920112	2.3956411	21456.27593	0.806956232	6.09627916	0.108657151	2.461424485	31675.8479	1.245035641	6.733027589
85 to 89	0.193336024	2.270949548	11240.68383	0.369237539	4.496423936	0.173232046	2.327949907	18324.46912	0.629085589	4.881593108
90 to 94	0.286086378	2.070774705	4167.904317	0.113811245	3.322038464	0.262280123	2.133000505	7564.302009	0.217056221	3.57137528
95 plus	0.369584528	2.706537924	934.7608343	0.025419237	2.706537924	0.352525539	2.838869295	1932.393866	0.055289696	2.838869295

Table 15: Cameroon 2100 life table, by age and sex. mx=mortality rate, ax=mean person-years lived in an age interval among those who die in that age interval, lx=number of persons left alive at age x, nLx=person-years lived between age x and x+n, ex=life expectancy at age x.

Age Group	Male					Female				
	mx	ax	lx	nLx	ex	mx	ax	lx	nLx	ex
Early Neonatal	0.14779078	0.009584511	100000	0.019150933	74.42057873	0.158358409	0.009584187	100000	0.019148993	79.94259444
Late Neonatal	0.016059395	0.028762693	99717.02421	0.057344946	74.6119992	0.018399901	0.028762048	99696.81749	0.057329468	80.16587211
Post Neonatal	0.003656637	0.461384074	99624.94686	0.918274395	74.62325685	0.003664969	0.461383482	99591.35561	0.917961289	80.19305631
1 to 4	0.000801322	1.998931571	99289.308	3.965218438	73.94971798	0.000704527	1.999060631	99255.07332	3.964617899	79.53883475
5 to 9	0.000265222	2.499447454	98971.80425	4.945312225	70.17973389	0.000237964	2.499504241	98975.9689	4.945857375	75.75669716
10 to 14	0.00027122	2.806966918	98840.71566	4.939086007	65.26923668	0.000221364	2.631995877	98858.35115	4.940321629	70.843609
15 to 19	0.000653461	2.74406712	98706.82606	4.928072421	60.35376389	0.000371509	2.650092607	98749.05011	4.933166259	65.91893465
20 to 24	0.001034486	2.622876986	98384.95471	4.90717433	55.54139668	0.000508457	2.664033742	98565.86566	4.922499317	61.03612963
25 to 29	0.001259757	2.596274994	97877.67232	4.879138167	50.81438457	0.000823756	2.625481745	98315.74934	4.906232879	56.18404687
30 to 34	0.001649614	2.601246429	97263.64696	4.843996604	46.11745998	0.001038486	2.623695007	97912.27278	4.883390533	51.40402586
35 to 39	0.002042268	2.604697859	96465.87567	4.799849346	41.4758768	0.001247121	2.58973609	97406.64004	4.855709075	46.65711027
40 to 44	0.002709777	2.652622502	95487.7518	4.744205805	36.87197523	0.001526188	2.662577966	96802.66167	4.822867781	41.93138681
45 to 49	0.004029727	2.698901452	94205.82396	4.667027403	32.33421999	0.00236758	2.697292543	96068.29344	4.77730889	37.2300472
50 to 54	0.006635432	2.693988397	92331.82277	4.54706123	27.93132339	0.00373229	2.666917534	94939.24134	4.705961036	32.63819273
55 to 59	0.010470638	2.659192698	89328.50229	4.359864956	23.7743474	0.005395565	2.656006849	93187.02342	4.601226459	28.19811202
60 to 64	0.015587297	2.638994847	84789.01159	4.089918773	19.89909291	0.008017731	2.652523326	90711.85138	4.452096256	23.89061034
65 to 69	0.023678969	2.622915127	78460.679	3.715786656	16.28297146	0.011985713	2.66463017	87157.43062	4.239780911	19.75007841
70 to 74	0.035612414	2.609437471	69756.46422	3.217478616	12.98287788	0.019174828	2.681221161	82107.43996	3.931934722	15.79425216
75 to 79	0.057727027	2.567541454	58456.25531	2.568814552	9.98700117	0.03347934	2.689510875	74636.62883	3.466781705	12.0992697
80 to 84	0.090584615	2.482308854	43914.93927	1.796211613	7.458901609	0.065893426	2.594612241	63186.79729	2.734556033	8.800795321
85 to 89	0.151719523	2.375724361	28038.08051	1.010814851	5.316578547	0.120027439	2.476235313	45541.88111	1.758843182	6.21309254
90 to 94	0.246595155	2.152358037	13105.54889	0.38890648	3.762005087	0.205883787	2.258228282	24991.8159	0.806908419	4.348885366
95 plus	0.338539425	2.958841132	3724.257231	0.111869981	2.958841132	0.302242965	3.320724287	8803.645033	0.29809433	3.320724287

Table 15: Cape Verde 2017 life table, by age and sex. mx=mortality rate, ax=mean person-years lived in an age interval among those who die in that age interval, lx=number of persons left alive at age x, nLx=person-years lived between age x and x+n, ex=life expectancy at age x.

Age Group	Male					Female				
	mx	ax	lx	nLx	ex	mx	ax	lx	nLx	ex
Early Neonatal	0.738927513	0.009566393	100000	0.019042853	72.27062632	0.464394899	0.009574807	100000	0.01909294	78.69223645
Late Neonatal	0.038308135	0.028756556	98593.17039	0.0566624	73.28213352	0.035968896	0.028757201	99113.43492	0.056965219	79.3766662
Post Neonatal	0.007302505	0.461125078	98376.20713	0.905241958	73.3861709	0.006034756	0.461215137	98908.5898	0.910672246	79.48346444
1 to 4	0.001012891	1.998649479	97715.54492	3.900720193	72.95611374	0.000860555	1.998852594	98359.21493	3.927607929	79.00162919
5 to 9	0.000322934	2.499327221	97320.73901	4.862111686	69.24416364	0.000202202	2.499578747	98021.38022	4.898592752	75.26713116
10 to 14	0.00039375	2.748143157	97163.77081	4.85388504	64.35202223	0.000240794	2.638934612	97922.34659	4.893335532	70.34073655
15 to 19	0.000794175	2.704768272	96972.68177	4.839812955	59.47340863	0.00036349	2.660723484	97804.52767	4.886073261	65.42228771
20 to 24	0.001181863	2.723375933	96588.32525	4.816456185	54.69917366	0.000524078	2.676426618	97626.92959	4.875413231	60.53641835
25 to 29	0.002081247	2.621685523	96019.102	4.777310299	50.00709619	0.00081417	2.703806103	97371.43263	4.859487636	55.68816954
30 to 34	0.002434574	2.682459739	95024.90384	4.7245881	45.50259114	0.001329209	2.672594268	96975.83035	4.833831813	50.90419127
35 to 39	0.004305293	2.619719506	93874.80699	4.646134797	41.0269232	0.00192715	2.625889308	96333.42399	4.794730864	46.2256817
40 to 44	0.005066961	2.581102492	91874.8507	4.538133305	36.8624968	0.00252469	2.607573281	95409.60358	4.741837022	41.6476798
45 to 49	0.00656706	2.584610323	89575.94369	4.408885173	32.74178071	0.003291723	2.530617388	94212.71314	4.672659374	37.14349164
50 to 54	0.008175902	2.609348866	86681.53363	4.251019735	28.74814974	0.003091616	2.615753281	92674.92273	4.599843365	32.7176536
55 to 59	0.011663505	2.565767785	83207.47137	4.045574364	24.8389098	0.005159945	2.648579075	91253.16325	4.507971766	28.18652256
60 to 64	0.013047276	2.578860915	78491.64825	3.804480687	21.17657927	0.007040158	2.67593914	88927.58958	4.374816044	23.85416019
65 to 69	0.018732321	2.648049069	73531.32662	3.521544566	17.43085391	0.011783644	2.738445387	85848.41663	4.181032742	19.61352343
70 to 74	0.030963148	2.613787283	66940.47221	3.116979275	13.88622187	0.022697217	2.653439431	80923.21632	3.841644934	15.64024135
75 to 79	0.046003401	2.595847003	57300.34517	2.580085049	10.78307186	0.033952623	2.639996686	72207.92256	3.342728543	12.20731634
80 to 84	0.079350698	2.560903278	45449.24734	1.904512934	7.919462347	0.061642723	2.600021206	60866.82055	2.651456443	8.989987098
85 to 89	0.139096393	2.408882273	30363.40693	1.116508951	5.585387771	0.114715732	2.490515202	44538.74331	1.729612696	6.333600074
90 to 94	0.234391064	2.177226148	14859.6985	0.447408085	3.905773894	0.20039242	2.271546178	24719.98801	0.799418493	4.417724812
95 plus	0.328853674	3.041133551	4386.802269	0.133527726	3.041133551	0.297381747	3.36311792	8717.26988	0.293411608	3.36311792

Table 15: Cape Verde 2100 life table, by age and sex. mx=mortality rate, ax=mean person-years lived in an age interval among those who die in that age interval, lx=number of persons left alive at age x, nLx=person-years lived between age x and x+n, ex=life expectancy at age x.

Age Group	Male					Female				
	mx	ax	lx	nLx	ex	mx	ax	lx	nLx	ex
Early Neonatal	0.146097792	0.009584563	100000	0.019151247	80.30210483	0.135064137	0.009584901	100000	0.019153279	84.8267645
Late Neonatal	0.006276783	0.028765392	99720.31203	0.057362973	80.50807975	0.010619374	0.028764194	99741.52163	0.057368011	85.02722801
Post Neonatal	0.001094109	0.461566112	99684.30985	0.919908302	80.47959922	0.000987722	0.461573669	99680.61941	0.919919446	85.02161554
1 to 4	0.000164167	1.99978111	99583.68003	3.982040092	79.63717868	0.000125708	1.99983239	99589.78215	3.982590386	84.17542358
5 to 9	7.7681E-05	2.499838165	99518.33317	4.974950607	75.68815555	5.79313E-05	2.49987931	99539.74219	4.976266579	80.21671941
10 to 14	0.000126829	2.892305523	99479.69379	4.972644226	70.71659555	8.7259E-05	2.687009898	99510.9225	4.974539153	75.23922035
15 to 19	0.000305922	2.742228249	99416.63188	4.967403274	65.7596713	0.000133253	2.605785572	99467.52541	4.971792385	70.27086228
20 to 24	0.000486117	2.750410883	99264.67213	4.957803425	60.85587304	0.000157005	2.689348054	99401.28737	4.968264344	65.31591137
25 to 29	0.000885203	2.655585989	99023.68692	4.940926363	55.99679437	0.000278308	2.730650385	99323.29884	4.96302531	60.36502861
30 to 34	0.001148895	2.721441545	98586.39176	4.916448084	51.23270798	0.00046049	2.706759029	99185.21038	4.954000942	55.44519121
35 to 39	0.002124251	2.643539892	98021.69642	4.876683406	46.51150467	0.000708481	2.655891287	98957.18376	4.93964412	50.56655959
40 to 44	0.002649849	2.621639516	96986.37909	4.818984773	41.97792412	0.000981415	2.674992467	98607.37268	4.919125946	45.73626529
45 to 49	0.003770001	2.608406901	95710.55505	4.742771494	37.50030844	0.001521988	2.572278541	98124.84551	4.888181533	40.94748486
50 to 54	0.004679696	2.646016641	93925.75649	4.645148181	33.15969714	0.001520143	2.685763568	97381.19885	4.851979703	36.23964324
55 to 59	0.007296463	2.574604572	91755.35391	4.508195292	28.87859921	0.002899424	2.659222273	96644.06795	4.799646504	31.49460712
60 to 64	0.007788035	2.604860638	88472.75357	4.343040575	24.85023179	0.003813193	2.696542705	95253.36554	4.7212542	26.91406014
65 to 69	0.012158146	2.656190138	85105.90083	4.13790914	20.72280198	0.006727027	2.774206594	93455.1147	4.603893233	22.37717559
70 to 74	0.01837873	2.646346463	80107.6775	3.840490425	16.84283515	0.013362095	2.690769353	90364.15929	4.383386389	18.04327878
75 to 79	0.029172305	2.643725528	73107.37783	3.422987868	13.19473723	0.020981169	2.73990715	84528.44467	4.035962828	14.09617271
80 to 84	0.049335828	2.657741948	63254.41447	2.841155597	9.831608564	0.044790397	2.66193934	76123.27017	3.448674866	10.34377199
85 to 89	0.098636871	2.531071466	49561.44576	2.00392662	6.817199551	0.090069539	2.578681034	60845.97645	2.504395402	7.266772873
90 to 94	0.190582201	2.239718174	30359.00727	1.003800714	4.5813995	0.170972977	2.321264609	38644.10257	1.332434409	4.981752044
95 plus	0.292653149	3.427842408	11710.81183	0.408372152	3.427842408	0.269853258	3.717123369	16236.83906	0.611265537	3.717123369

Table 15: Chad 2017 life table, by age and sex. mx=mortality rate, ax=mean person-years lived in an age interval among those who die in that age interval, lx=number of persons left alive at age x, nLx=person-years lived between age x and x+n, ex=life expectancy at age x.

Age Group	Male					Female				
	mx	ax	lx	nLx	ex	mx	ax	lx	nLx	ex
Early Neonatal	1.359996284	0.009547358	100000	0.018930163	58.81421385	1.113664113	0.009554908	100000	0.018974743	61.73776676
Late Neonatal	0.16460771	0.028721716	97425.8606	0.055788728	60.34703382	0.104322102	0.028738346	97887.04936	0.056149938	63.04995324
Post Neonatal	0.042151725	0.458649525	96507.84913	0.873937279	60.86284315	0.040031613	0.458800122	97301.4206	0.881977896	63.37149485
1 to 4	0.013611146	1.981852725	92826.10911	3.613859468	62.33485035	0.013342658	1.982210653	93772.07948	3.652609075	64.81564128
5 to 9	0.001479933	2.496916809	87911.79456	4.37938176	61.71072911	0.001243065	2.497410284	88901.98443	4.431319806	64.25942464
10 to 14	0.001033538	2.537956734	87264.27862	4.352145704	57.15064824	0.000849277	2.513226004	88351.38094	4.408262301	59.64406943
15 to 19	0.001687488	2.669234173	86814.71815	4.323738385	52.43347793	0.001310267	2.662388485	87977.12146	4.385430805	54.88693458
20 to 24	0.002441672	2.604151719	86085.22537	4.279231019	47.85451108	0.00189427	2.637435321	87402.6422	4.350669352	50.22973777
25 to 29	0.002963692	2.596949162	85040.56234	4.221967271	43.40933526	0.002601833	2.6231196	86578.76015	4.302340503	45.6817683
30 to 34	0.003920417	2.614282339	83789.63301	4.150672489	39.01762334	0.003504594	2.61856465	85459.82001	4.237633832	41.24455846
35 to 39	0.005282347	2.611052101	82163.02408	4.056984766	34.7371468	0.004723669	2.59012065	83975.45836	4.151544291	36.92621639
40 to 44	0.00704182	2.615205891	80021.18523	3.935028971	30.59555943	0.005772039	2.585093355	82015.61915	4.044451427	32.7451956
45 to 49	0.009740081	2.615857318	77252.40014	3.775059858	26.59627335	0.00745197	2.608643956	79682.92774	3.91447132	28.6259174
50 to 54	0.013527267	2.614243132	73579.67975	3.564155188	22.79062895	0.010310462	2.616371174	76768.96641	3.746522426	24.61059825
55 to 59	0.019228605	2.613225201	68767.12151	3.287816417	19.19977099	0.014398977	2.633828826	72912.77094	3.525795029	20.77015729
60 to 64	0.028198073	2.591749817	62461.5263	2.925061005	15.87217562	0.022103091	2.618380678	67848.89355	3.223355117	17.12035184
65 to 69	0.040244413	2.578208564	54240.8617	2.47209881	12.88477007	0.032330806	2.62072421	60751.05804	2.821564716	13.81206163
70 to 74	0.062347871	2.528892625	44334.63981	1.922122701	10.19067638	0.054329497	2.566714252	51676.14366	2.28391065	10.77775555
75 to 79	0.087621862	2.463430849	32412.56468	1.327492281	8.017306303	0.079753023	2.497526127	39353.665	1.642550547	8.355967715
80 to 84	0.127226301	2.39587992	20853.90028	0.784486884	6.111859336	0.122492474	2.42614664	26359.34268	1.004368553	6.260430874
85 to 89	0.192636777	2.27239709	10933.1101	0.359066626	4.505848769	0.188785189	2.292153525	14168.69381	0.470300559	4.589378688
90 to 94	0.285470384	2.072103973	4051.155076	0.110558116	3.327203861	0.277826502	2.097051386	5366.044715	0.149093364	3.401488608
95 plus	0.369120136	2.709539159	906.7915161	0.024629965	2.709539159	0.365882482	2.734314469	1253.929203	0.034470505	2.734314469

Table 15: Chad 2100 life table, by age and sex. mx=mortality rate, ax=mean person-years lived in an age interval among those who die in that age interval, lx=number of persons left alive at age x, nLx=person-years lived between age x and x+n, ex=life expectancy at age x.

Age Group	Male					Female				
	mx	ax	lx	nLx	ex	mx	ax	lx	nLx	ex
Early Neonatal	0.195721324	0.009583042	100000	0.019142137	75.74901304	0.187774461	0.009583286	100000	0.019143596	80.12397161
Late Neonatal	0.023029266	0.028760771	99625.39848	0.057280771	76.01414774	0.015642437	0.028762808	99640.60049	0.057301686	80.39219872
Post Neonatal	0.003819149	0.46137253	99493.50367	0.916993992	76.05730027	0.0037425	0.461377975	99550.98852	0.917556264	80.40669841
1 to 4	0.000990455	1.998679393	99143.40698	3.957895129	75.40010498	0.001029878	1.998626829	99207.70552	3.960150125	79.75901856
5 to 9	0.000260606	2.499457071	98751.63244	4.934368397	71.69012766	0.000230838	2.499519088	98800.10405	4.937157743	76.07825192
10 to 14	0.000248543	2.784050421	98623.13394	4.928439101	66.77987929	0.000213152	2.604452069	98686.2297	4.931786948	71.16274403
15 to 19	0.000596038	2.753407756	98500.72736	4.918450377	61.85909453	0.000329993	2.670961079	98581.20277	4.92527366	66.23541926
20 to 24	0.00097332	2.635371033	98207.79578	4.89910214	57.03423906	0.000481598	2.683295313	98418.78311	4.915458714	61.33989145
25 to 29	0.001213764	2.617230879	97731.48618	4.87247851	52.29747326	0.000756585	2.653031302	98182.22978	4.900421686	56.48058742
30 to 34	0.001650228	2.608589013	97140.91105	4.837957117	47.59748616	0.001048357	2.615016424	97811.78192	4.878369757	51.68350514
35 to 39	0.002079012	2.601589152	96343.68357	4.793322334	42.96740166	0.001311282	2.593491897	97300.95956	4.849755727	46.93994841
40 to 44	0.002712579	2.629036481	95348.82785	4.737038292	38.38537819	0.001645824	2.646437868	96665.92618	4.8146425	42.22955826
45 to 49	0.00383698	2.660875075	94067.01729	4.661646224	33.8677419	0.002465052	2.685204219	95874.98376	4.76657977	37.55316452
50 to 54	0.005875173	2.67949624	92283.923	4.552364563	29.46305258	0.003891628	2.666360641	94702.46994	4.692625215	32.97949816
55 to 59	0.009265377	2.66545695	89623.20832	4.386729863	25.24666422	0.005731556	2.656986799	92882.68254	4.58273593	28.56433695
60 to 64	0.013950836	2.638383752	85590.69995	4.144174322	21.29694874	0.008374657	2.631456601	90271.89553	4.426443348	24.30235042
65 to 69	0.020669566	2.627083165	79866.55053	3.809080378	17.62046169	0.011816198	2.671443598	86593.79862	4.214887832	20.20826853
70 to 74	0.031262348	2.60702749	72117.37318	3.359677124	14.21978249	0.019694039	2.668810277	81678.5344	3.907352095	16.24762433
75 to 79	0.047994543	2.568510509	61841.72659	2.778306127	11.1467501	0.032263007	2.665505584	74122.27463	3.452449051	12.61673955
80 to 84	0.070967226	2.507328412	48956.7268	2.092011605	8.429593504	0.059304518	2.596700376	63287.71301	2.783685247	9.31367395
85 to 89	0.127402826	2.44391615	34675.5798	1.320952336	5.925016511	0.110382046	2.510200653	47475.7605	1.883376107	6.569117765
90 to 94	0.221955449	2.197094661	18484.74618	0.5770739	4.090162928	0.194468907	2.277982449	27758.37182	0.924493406	4.563639217
95 plus	0.318619265	3.146584246	6060.264606	0.194528303	3.146584246	0.291592849	3.455414654	10661.37725	0.382325201	3.455414654

Table 15: Cote d'Ivoire 2017 life table, by age and sex. mx=mortality rate, ax=mean person-years lived in an age interval among those who die in that age interval, lx=number of persons left alive at age x, nLx=person-years lived between age x and x+n, ex=life expectancy at age x.

Age Group	Male					Female				
	mx	ax	lx	nLx	ex	mx	ax	lx	nLx	ex
Early Neonatal	1.665300331	0.009538001	100000	0.018875123	60.61296723	0.951567085	0.009559876	100000	0.019004168	65.63636349
Late Neonatal	0.106577275	0.028737724	96857.57554	0.05555587	62.55945048	0.067536256	0.028748493	98191.9182	0.056384403	66.82535177
Post Neonatal	0.028404873	0.459626026	96265.82657	0.877259274	62.88643766	0.024434483	0.459908066	97811.25863	0.89296936	67.02782228
1 to 4	0.006880803	1.990825715	93774.86025	3.699881818	63.62089287	0.006343459	1.991542147	95629.66255	3.777083296	67.62258153
5 to 9	0.001122016	2.497662469	91231.00321	4.548787964	61.34054388	0.000885565	2.498155073	93234.69912	4.6514314	65.30890707
10 to 14	0.000924919	2.592931463	90720.99599	4.52597606	56.67162535	0.000709101	2.565627242	92822.86247	4.633145431	60.58733373
15 to 19	0.001547339	2.699552951	90302.54206	4.49911929	51.92220328	0.001116205	2.698933913	92494.37271	4.612883084	55.79332223
20 to 24	0.002441592	2.631253804	89606.48262	4.454567081	47.30397743	0.001802626	2.674350763	91979.55151	4.579793127	51.09016286
25 to 29	0.003146459	2.615826757	88519.06556	4.393015334	42.85171824	0.002672618	2.644734043	91154.27208	4.529214339	46.52763105
30 to 34	0.004309443	2.623176965	87137.3854	4.31270611	38.48873815	0.003730391	2.616185517	89944.63285	4.457587018	42.11688211
35 to 39	0.005882715	2.613750578	85280.01959	4.205002589	34.26904466	0.004854204	2.582238708	88283.33002	4.362994414	37.85944863
40 to 44	0.007878876	2.61085762	82808.26626	4.063966905	30.2130682	0.005864707	2.577498761	86167.47635	4.248080739	33.72479198
45 to 49	0.010748664	2.606002989	79609.08792	3.880694106	26.32043591	0.007409246	2.596909291	83678.53133	4.110824486	29.64909009
50 to 54	0.014636598	2.597442601	75441.47011	3.644113946	22.62738333	0.009947244	2.596275061	80636.01498	3.937796869	25.66674925
55 to 59	0.019935803	2.599233234	70115.81704	3.345991222	19.1448987	0.013046747	2.623162816	76725.87228	3.721167151	21.83861071
60 to 64	0.028666348	2.58593111	63460.95103	2.9682874	15.87686716	0.019751101	2.625831588	71883.50579	3.433693485	18.12975299
65 to 69	0.040677849	2.568450503	54980.72826	2.502607996	12.9253916	0.029406114	2.612132264	65125.66749	3.043568694	14.73576061
70 to 74	0.060768151	2.52815774	44848.06151	1.951044398	10.26750551	0.045778565	2.578968069	56220.50744	2.532133386	11.65576946
75 to 79	0.087011255	2.470536637	33061.47789	1.35668943	8.034925605	0.069908526	2.527593234	44703.79517	1.907917306	8.999245179
80 to 84	0.127435221	2.398383629	21341.9731	0.803028696	6.107846205	0.108444609	2.45460358	31466.24124	1.235499034	6.73455974
85 to 89	0.192886022	2.271896687	11185.87505	0.367394964	4.502670214	0.172947443	2.328361379	18189.65913	0.623965243	4.884151303
90 to 94	0.28568822	2.071635743	4145.871068	0.113167454	3.325450673	0.262020354	2.133585974	7492.820616	0.214757559	3.572914215
95 plus	0.369283467	2.708517268	928.6556531	0.025234198	2.708517268	0.352319581	2.839802271	1907.612083	0.054469496	2.839802271

Table 15: Cote d'Ivoire 2100 life table, by age and sex. mx=mortality rate, ax=mean person-years lived in an age interval among those who die in that age interval, lx=number of persons left alive at age x, nLx=person-years lived between age x and x+n, ex=life expectancy at age x.

Age Group	Male					Female				
	mx	ax	lx	nLx	ex	mx	ax	lx	nLx	ex
Early Neonatal	0.317110496	0.009579322	100000	0.019119903	77.08508346	0.178526594	0.009583569	100000	0.019145295	81.8382325
Late Neonatal	0.013909161	0.028763286	99393.99327	0.057162712	77.53604645	0.010731086	0.028764163	99658.31174	0.057319964	82.09929612
Post Neonatal	0.004133677	0.461350186	99314.49511	0.915211276	77.54055111	0.004009625	0.461358999	99596.80931	0.91786537	82.09239765
1 to 4	0.000725811	1.999032253	98936.27893	3.951714408	76.91164106	0.00076293	1.998982761	99228.87336	3.963107602	81.47116586
5 to 9	0.000179143	2.499626785	98649.58974	4.930272076	73.12911915	0.000159179	2.499668378	98926.67631	4.944367167	77.71353713
10 to 14	0.000187839	2.827100884	98561.30755	4.926041512	68.1922908	0.000163907	2.688572851	98848.02199	4.940516482	72.77330726
15 to 19	0.000461101	2.761344733	98468.81568	4.918354519	63.2536267	0.000294382	2.69173467	98767.0913	4.935025568	67.83067461
20 to 24	0.00075874	2.635186293	98242.08641	4.903307843	58.39280191	0.000458412	2.663026842	98621.88523	4.925865006	62.92637693
25 to 29	0.00095632	2.603022344	97870.25049	4.882354174	53.60360986	0.000695928	2.627316003	98396.4001	4.911728976	58.06390889
30 to 34	0.001265011	2.608259181	97403.79955	4.855467509	48.84668656	0.000889427	2.633110457	98055.41523	4.892342715	53.25623981
35 to 39	0.00157647	2.603206371	96790.56058	4.821327642	44.13845519	0.001110886	2.615215954	97621.80551	4.868150324	48.48112098
40 to 44	0.00205488	2.648739945	96032.02679	4.778482476	39.46492095	0.001427488	2.649116691	97082.96647	4.837867796	43.73537481
45 to 49	0.00300582	2.681024376	95052.56809	4.719761883	34.84142725	0.002060604	2.68028257	96394.46497	4.796775886	39.02773338
50 to 54	0.004734538	2.698617346	93637.58453	4.631429241	30.32306946	0.003193505	2.66796089	95408.26108	4.735114088	34.40129363
55 to 59	0.007604893	2.675874156	91454.25333	4.493389715	25.97686698	0.004625518	2.644074358	93900.04693	4.644414706	29.90744776
60 to 64	0.011491078	2.662501295	88055.28849	4.288123015	21.87088176	0.006488433	2.639966568	91758.77681	4.518990479	25.54006917
65 to 69	0.018015106	2.646312246	83159.39615	3.990049685	17.99462908	0.009494477	2.667258422	88837.38847	4.346050054	21.28747373
70 to 74	0.027660804	2.636178374	76037.59972	3.570990076	14.42666148	0.015169545	2.692119031	84734.67157	4.094318847	17.18245969
75 to 79	0.04544291	2.598916986	66281.80895	2.992817504	11.16058231	0.02639938	2.706397983	78573.97803	3.706410771	13.31259939
80 to 84	0.071994255	2.522710569	52917.67993	2.251729639	8.334798145	0.051259274	2.622457996	68902.00996	3.075621631	9.799289084
85 to 89	0.129149775	2.438043407	37003.35388	1.397228463	5.858778739	0.099593748	2.543953108	53376.00238	2.152344563	6.890776288
90 to 94	0.22401119	2.194898591	19303.54574	0.596668303	4.053562791	0.182504229	2.303036399	32346.75984	1.090938453	4.752867095
95 plus	0.320364604	3.12564083	6144.802233	0.194079885	3.12564083	0.280681961	3.573250873	12803.5743	0.463950752	3.573250873

Table 15: The Gambia 2017 life table, by age and sex. mx=mortality rate, ax=mean person-years lived in an age interval among those who die in that age interval, lx=number of persons left alive at age x, nLx=person-years lived between age x and x+n, ex=life expectancy at age x.

Age Group	Male					Female				
	mx	ax	lx	nLx	ex	mx	ax	lx	nLx	ex
Early Neonatal	1.125454488	0.009554546	100000	0.018972634	63.8016987	0.878833812	0.009562105	100000	0.019017389	67.86535487
Late Neonatal	0.057318422	0.028751312	97865.35681	0.056213404	65.17316191	0.046767365	0.028754223	98328.99009	0.056496829	68.99853272
Post Neonatal	0.013635121	0.460675222	97543.33237	0.894963179	65.33060916	0.011544639	0.460823725	98064.87675	0.900614903	69.12668414
1 to 4	0.00387437	1.994834195	96323.75691	3.82327581	65.22856152	0.003917282	1.99477698	97025.69618	3.850802041	68.93883767
5 to 9	0.000857702	2.498213121	94843.93363	4.732044491	62.21591347	0.000696073	2.498549849	95518.41951	4.767622316	65.99577615
10 to 14	0.000739004	2.65516159	94438.13911	4.713740402	57.47221459	0.000533348	2.569282583	95186.66667	4.753172616	61.21697963
15 to 19	0.001419287	2.701022217	94089.83361	4.689196502	52.67488072	0.000878338	2.671211323	94933.21674	4.736976424	56.37350349
20 to 24	0.002136664	2.617046029	93424.31888	4.64755705	48.03017586	0.001267335	2.67741228	94517.20942	4.711996144	51.60953642
25 to 29	0.002663819	2.609599496	92431.52247	4.592345705	43.51694981	0.001980254	2.673490903	93920.18996	4.674473416	46.92000962
30 to 34	0.003620247	2.624384907	91208.72017	4.521561642	39.06439277	0.002955305	2.637380909	92994.82614	4.617495162	42.3595801
35 to 39	0.004958739	2.626526189	89572.89708	4.426579637	34.72890121	0.004003461	2.613500723	91630.76015	4.538197196	37.95013663
40 to 44	0.006895238	2.620236232	87379.91365	4.298528323	30.53363391	0.005308483	2.603574531	89814.75402	4.434368452	33.66346513
45 to 49	0.009442577	2.6206159	84419.59343	4.128339897	26.51157631	0.006955599	2.615716437	87462.20379	4.301840782	29.49674048
50 to 54	0.013357676	2.619578317	80527.09971	3.902464004	22.66580769	0.009743138	2.615491693	84472.83436	4.127884579	25.44497884
55 to 59	0.019085732	2.618768942	75323.94651	3.602748572	19.05072284	0.013404193	2.626724634	80457.16658	3.899087234	21.58068002
60 to 64	0.028451848	2.599395321	68461.28555	3.204597685	15.6990024	0.020037118	2.628616251	75243.21042	3.592014335	17.89025457
65 to 69	0.041595671	2.570490263	59361.60252	2.696105181	12.70920222	0.030460145	2.608703423	68070.19421	3.173396773	14.49536392
70 to 74	0.061785117	2.527715549	48166.70926	2.089669315	10.06818923	0.046572792	2.576791473	58450.21263	2.627817032	11.4507124
75 to 79	0.090056893	2.47032344	35276.28067	1.436998932	7.826100253	0.072144568	2.529474996	46291.62742	1.966977323	8.785550311
80 to 84	0.13367258	2.390366334	22355.99531	0.829137947	5.924632768	0.114329166	2.450036543	32221.55128	1.250429294	6.5307143
85 to 89	0.199535523	2.256551763	11293.39116	0.365185815	4.392441078	0.17974389	2.312592171	18071.99553	0.611486534	4.755036786
90 to 94	0.291763063	2.058638196	4020.555317	0.108277999	3.265984154	0.268826171	2.117834643	7190.89677	0.20348645	3.497884131
95 plus	0.373965193	2.674262881	866.3705584	0.023194578	2.674262881	0.358175893	2.793620173	1767.815921	0.049701639	2.793620173

Table 15: The Gambia 2100 life table, by age and sex. mx=mortality rate, ax=mean person-years lived in an age interval among those who die in that age interval, lx=number of persons left alive at age x, nLx=person-years lived between age x and x+n, ex=life expectancy at age x.

Age Group	Male					Female				
	mx	ax	lx	nLx	ex	mx	ax	lx	nLx	ex
Early Neonatal	0.210957886	0.009582575	100000	0.019139346	76.23669156	0.212985084	0.009582513	100000	0.019138975	80.411079
Late Neonatal	0.008504209	0.028764777	99596.33933	0.057287989	76.52613482	0.010134271	0.028764328	99592.47813	0.057283083	80.72044893
Post Neonatal	0.002368977	0.461475547	99547.62744	0.91810673	76.5059984	0.002448268	0.461469915	99534.43641	0.917951535	80.70989673
1 to 4	0.000488861	1.999348186	99330.18028	3.969326959	75.74906739	0.000525056	1.999299925	99309.75727	3.968224387	79.96788405
5 to 9	0.000182298	2.499620213	99136.23874	4.954554179	71.89314976	0.000163784	2.499658783	99101.54744	4.953049749	76.13128447
10 to 14	0.000192904	2.873234747	99045.94264	4.950257724	66.95635053	0.000152473	2.649454449	99020.45402	4.949246716	71.19147753
15 to 19	0.00052031	2.766496065	98950.46644	4.941770307	62.0181535	0.000270943	2.661771773	98945.01211	4.944122178	66.2436697
20 to 24	0.000851273	2.619996187	98693.37163	4.924693497	57.17238627	0.000366524	2.683664029	98811.08855	4.936383504	61.32971414
25 to 29	0.001018466	2.586931324	98274.20501	4.901689084	52.40466455	0.000614511	2.683468293	98630.20921	4.924501508	56.43704005
30 to 34	0.001309326	2.606345962	97775.15128	4.873474889	47.65833285	0.000909609	2.651028658	98327.77698	4.905838945	51.60205544
35 to 39	0.0016867	2.64605096	97137.49982	4.837658736	42.95330897	0.001205625	2.656995146	97882.008	4.880271046	46.82473627
40 to 44	0.002501102	2.664443558	96322.31264	4.78812313	38.29344236	0.001777479	2.655469682	97294.30203	4.844494767	42.09099008
45 to 49	0.003677047	2.698253696	95126.21743	4.716390587	33.74010908	0.002513727	2.688521253	96434.15129	4.793839136	37.44178485
50 to 54	0.006130437	2.676612547	93394.28859	4.604170649	29.31415508	0.004094534	2.658423516	95230.39724	4.716331199	32.87948947
55 to 59	0.009226182	2.643455905	90576.78409	4.432586157	25.14090641	0.005778242	2.62076848	93302.04375	4.601877974	28.50125819
60 to 64	0.013359335	2.634270174	86495.8161	4.192551416	21.20092436	0.007706229	2.630123619	90648.60594	4.451328233	24.25590202
65 to 69	0.019879349	2.623015418	80908.15861	3.863309151	17.48166035	0.011310679	2.661860145	87226.62117	4.249292134	20.09999027
70 to 74	0.029550504	2.621417603	73250.20737	3.422794615	14.03407012	0.018046458	2.681903088	82438.35696	3.95716365	16.10819845
75 to 79	0.047915287	2.588647116	63174.54932	2.833071126	10.85358146	0.031387156	2.695475071	75335.88555	3.51447132	12.36894162
80 to 84	0.076149289	2.5117828	49672.26308	2.090788379	8.101336176	0.062197643	2.598738413	64399.9797	2.806298505	9.008386855
85 to 89	0.134514806	2.422346327	33884.2135	1.261534261	5.71163099	0.115136044	2.490984131	47181.65754	1.837759059	6.35076467
90 to 94	0.229606747	2.18539517	17090.87872	0.521146083	3.973974797	0.200544927	2.269699705	26396.43305	0.858568353	4.429609541
95 plus	0.324939129	3.08012743	5234.18135	0.162266563	3.08012743	0.297408877	3.370904807	9475.042741	0.323643047	3.370904807

Table 15: Ghana 2017 life table, by age and sex. mx=mortality rate, ax=mean person-years lived in an age interval among those who die in that age interval, lx=number of persons left alive at age x, nLx=person-years lived between age x and x+n, ex=life expectancy at age x.

Age Group	Male					Female				
	mx	ax	lx	nLx	ex	mx	ax	lx	nLx	ex
Early Neonatal	1.215220966	0.009551795	100000	0.018956349	63.03539608	0.969178594	0.009559336	100000	0.01900096	68.77688002
Late Neonatal	0.070581341	0.028747653	97696.68388	0.056095109	64.50163232	0.046719832	0.028754236	98158.62005	0.056399002	70.0474707
Post Neonatal	0.015000398	0.460578236	97300.87091	0.89217768	64.70636421	0.012256883	0.460773129	97895.17421	0.898760379	70.17836496
1 to 4	0.004416952	1.994110762	95963.42303	3.804848852	64.67849237	0.002987453	1.996016739	96793.96262	3.848725806	70.0481359
5 to 9	0.000980209	2.497957899	94284.02753	4.70266854	61.79576456	0.000755308	2.498426442	95644.63855	4.773215076	66.86615279
10 to 14	0.000847251	2.638046686	93823.09356	4.681784625	57.0867905	0.000606836	2.563628645	95284.19212	4.757176598	62.10965123
15 to 19	0.001553442	2.690485188	93426.44635	4.6546254	52.3177481	0.000945835	2.646884341	94995.56056	4.739233805	57.29063899
20 to 24	0.002297615	2.59631379	92703.37152	4.609714157	47.70437794	0.00128516	2.646221157	94547.37927	4.71311976	52.54971189
25 to 29	0.002665444	2.584841326	91644.34651	4.552916923	43.22493155	0.001872607	2.665694733	93941.7993	4.676651376	47.8713445
30 to 34	0.003466445	2.619255971	90431.09274	4.484555782	38.76949891	0.002815974	2.646513609	93066.27588	4.622667975	43.2963935
35 to 39	0.004782585	2.635690182	88877.21563	4.394193929	34.40086009	0.003920468	2.614915929	91764.93769	4.545745098	38.87261055
40 to 44	0.00683847	2.63385985	86776.88672	4.269796314	30.16900113	0.005130224	2.598995358	89983.34915	4.444441464	34.59003712
45 to 49	0.009694627	2.633003259	83859.1519	4.098971852	26.12617805	0.006645528	2.606560539	87703.80594	4.316567373	30.41996933
50 to 54	0.01410674	2.621527695	79889.0556	3.864904971	22.29282746	0.009044573	2.60474464	84836.17571	4.151936501	26.35781114
55 to 59	0.020115938	2.615603123	74443.3218	3.551998107	18.73135369	0.012080088	2.625467077	81084.07341	3.941288489	22.45402251
60 to 64	0.029978063	2.593363851	67307.36814	3.139212657	15.4400197	0.018078035	2.631214531	76329.91224	3.660066075	18.68615834
65 to 69	0.043363931	2.563392897	57910.82202	2.619231999	12.52537215	0.027272739	2.618449485	69727.16309	3.274313085	15.20404352
70 to 74	0.064022993	2.518992799	46571.07508	2.009875164	9.952805382	0.042303777	2.587686084	60826.19002	2.760644286	12.04453981
75 to 79	0.091638608	2.460146609	33725.93458	1.368440475	7.787034223	0.065278867	2.542936054	49193.39729	2.121195095	9.282584614
80 to 84	0.133300656	2.382902299	21210.22479	0.786731821	5.934939009	0.103680368	2.472255119	35418.21447	1.405285198	6.910355245
85 to 89	0.19899336	2.257772294	10747.56896	0.347970662	4.401044669	0.167536774	2.341377322	20951.11739	0.726520203	4.992258565
90 to 94	0.291271679	2.059689601	3838.634761	0.103487569	3.270641916	0.256554775	2.146258742	8867.470414	0.256776742	3.635646644
95 plus	0.373588328	2.676950907	829.6717728	0.022237145	2.676950907	0.347583915	2.878443288	2321.734029	0.067149674	2.878443288

Table 15: Ghana 2100 life table, by age and sex. mx=mortality rate, ax=mean person-years lived in an age interval among those who die in that age interval, lx=number of persons left alive at age x, nLx=person-years lived between age x and x+n, ex=life expectancy at age x.

Age Group	Male					Female				
	mx	ax	lx	nLx	ex	mx	ax	lx	nLx	ex
Early Neonatal	0.239469135	0.009581701	100000	0.019134116	73.60655026	0.220474611	0.009582284	100000	0.019137601	81.26992583
Late Neonatal	0.012554257	0.02876366	99541.87696	0.057249996	73.92559452	0.009833712	0.028764411	99578.17154	0.057275351	81.59451851
Post Neonatal	0.002429145	0.461471273	99470.02191	0.917365662	73.9213913	0.002066923	0.461497005	99521.86468	0.917997102	81.58308577
1 to 4	0.000498279	1.999335628	99247.26232	3.965939161	73.16258887	0.000283851	1.999621533	99332.17344	3.971033289	80.81437956
5 to 9	0.000146664	2.499694449	99049.77	4.950673616	69.30415079	0.000123275	2.499743176	99219.51589	4.959447683	76.90364034
10 to 14	0.000189716	2.877735182	98977.18381	4.946864831	64.35307723	0.000135843	2.686108761	99158.39804	4.956359079	71.94942264
15 to 19	0.000488662	2.766234539	98883.35241	4.938776115	59.41135787	0.000241679	2.639891828	99091.08777	4.951737755	66.99640817
20 to 24	0.000815257	2.614702411	98642.08207	4.922530009	54.54963668	0.000305612	2.666695336	98971.44191	4.945060485	62.07408702
25 to 29	0.000938171	2.605423986	98240.92915	4.901049237	49.76105856	0.000500792	2.661939499	98820.36559	4.935243366	57.16475332
30 to 34	0.001308038	2.627366889	97781.38924	4.873940604	44.98178235	0.000697767	2.637474285	98573.37709	4.920502441	52.30104313
35 to 39	0.001741048	2.657065511	97144.42348	4.837498266	40.25817557	0.000904558	2.641727317	98230.39719	4.901035875	47.47424101
40 to 44	0.002648215	2.694292135	96303.14623	4.785943337	35.5849653	0.001283358	2.703506642	97787.52817	4.874974374	42.67694068
45 to 49	0.004264257	2.725738041	95037.84951	4.706285678	31.01997639	0.002134247	2.703320914	97162.43249	4.834417219	37.93329399
50 to 54	0.007460466	2.698309318	93035.71931	4.573364473	26.62513204	0.003400436	2.663595225	96131.39212	4.768699307	33.30953395
55 to 59	0.011876124	2.659863075	89636.123	4.360946325	22.52743257	0.004904747	2.664914218	94511.92666	4.672108327	28.83199319
60 to 64	0.017973236	2.642198499	84481.15327	4.053052319	18.73548417	0.00749361	2.667309507	92225.04419	4.532181524	24.47792688
65 to 69	0.027889816	2.605837714	77234.63616	3.621397215	15.24111803	0.011573837	2.666102735	88837.23191	4.325340048	20.3053642
70 to 74	0.04034744	2.594487417	67204.02647	3.065305015	12.12540809	0.01823945	2.673008051	83850.53128	4.022542323	16.34971103
75 to 79	0.066369162	2.55080497	54950.79768	2.367391211	9.253747707	0.030945312	2.675516218	76552.65439	3.572425582	12.6493937
80 to 84	0.104120021	2.450260051	39431.67041	1.563526223	6.904269134	0.057648755	2.601048031	65583.86698	2.884982368	9.314629871
85 to 89	0.167401728	2.334490923	23420.27765	0.814774544	4.979109191	0.108775738	2.511954621	49159.96731	1.941325358	6.559035337
90 to 94	0.261808023	2.121829848	10034.60426	0.288362445	3.581112262	0.193296457	2.283831034	28390.87944	0.936514801	4.55344226
95 plus	0.350624507	2.855265366	2603.90383	0.075179863	2.855265366	0.290730783	3.448266315	10583.50262	0.369584576	3.448266315

Table 15: Guinea 2017 life table, by age and sex. mx=mortality rate, ax=mean person-years lived in an age interval among those who die in that age interval, lx=number of persons left alive at age x, nLx=person-years lived between age x and x+n, ex=life expectancy at age x.

Age Group	Male					Female				
	mx	ax	lx	nLx	ex	mx	ax	lx	nLx	ex
Early Neonatal	1.395797344	0.009546261	100000	0.018923701	59.55150669	1.01034871	0.009558074	100000	0.018993484	62.35237144
Late Neonatal	0.143890917	0.028727431	97359.10922	0.055783696	61.14600468	0.130773034	0.02873105	98081.15859	0.05621854	63.5520207
Post Neonatal	0.032534934	0.459332644	96556.76647	0.878247642	61.59612634	0.030136984	0.459502982	97346.12419	0.886399311	63.97391722
1 to 4	0.009978539	1.986695647	93701.25825	3.674305642	62.53614177	0.009633532	1.987155616	94675.70415	3.715029155	64.84186278
5 to 9	0.001266857	2.497360716	90038.65153	4.487715352	61.00145695	0.001186478	2.497528171	91098.88352	4.541463939	63.31063978
10 to 14	0.000975394	2.583388241	89470.56548	4.463012383	56.37312431	0.000797702	2.501473099	90560.2083	4.519005464	58.67229218
15 to 19	0.001673497	2.685839602	89035.43778	4.434603368	51.63594988	0.001202965	2.674645821	90199.82602	4.497414668	53.89670986
20 to 24	0.002502749	2.604087046	88293.44688	4.38836122	47.04688886	0.001824652	2.649925976	89658.94004	4.463814063	49.20565671
25 to 29	0.0029814	2.589953403	87195.31867	4.328671307	42.60584881	0.00255513	2.627440604	88844.70983	4.415476808	44.63223518
30 to 34	0.00389032	2.608017624	85905.12895	4.255666932	38.20618124	0.003458837	2.618288555	87716.8718	4.350013541	40.1721732
35 to 39	0.005161855	2.616716465	84250.14494	4.161341224	33.90472054	0.004643132	2.607196598	86212.75333	4.26329329	35.82696999
40 to 44	0.007087024	2.628616993	82103.11443	4.037348057	29.72195579	0.006084308	2.606905731	84233.91717	4.151288317	31.60661916
45 to 49	0.010126347	2.634785361	79243.45563	3.869571466	25.69784009	0.008193177	2.6283737	81709.06564	4.007636674	27.50072562
50 to 54	0.014845441	2.622532344	75328.19834	3.638150105	21.89407887	0.011947323	2.617814929	78427.63093	3.812972815	23.5388313
55 to 59	0.02131484	2.611788292	69934.08373	3.327595912	18.37742789	0.016474745	2.626428962	73877.5349	3.555071519	19.82422311
60 to 64	0.031515385	2.5857578	62854.16075	2.92101866	15.15072227	0.025242345	2.616519424	68031.13914	3.208932318	16.29971275
65 to 69	0.04499782	2.558999269	53672.94232	2.418915455	12.29864888	0.037800656	2.590019876	59950.80262	2.748043849	13.14245201
70 to 74	0.066911333	2.513501751	42829.29944	1.837267428	9.766343872	0.057935657	2.544995265	49600.19521	2.172389181	10.34536169
75 to 79	0.094916476	2.448675675	30595.56366	1.233021711	7.673840381	0.085985517	2.486870603	37070.78522	1.52572356	7.987098912
80 to 84	0.134868648	2.371432549	18961.9124	0.701280473	5.895051881	0.131530621	2.406725828	24025.13596	0.897303907	5.985791708
85 to 89	0.20040608	2.254713006	9567.467983	0.309327008	4.380112479	0.198645405	2.270947234	12299.47117	0.399733438	4.420587938
90 to 94	0.29253718	2.056985772	3405.098131	0.091714929	3.25923981	0.287544188	2.074786509	4407.047462	0.120020767	3.302910806
95 plus	0.374551762	2.670349411	734.1402477	0.019663018	2.670349411	0.374126228	2.673696398	973.7196241	0.026132828	2.673696398

Table 15: Guinea 2100 life table, by age and sex. mx=mortality rate, ax=mean person-years lived in an age interval among those who die in that age interval, lx=number of persons left alive at age x, nLx=person-years lived between age x and x+n, ex=life expectancy at age x.

Age Group	Male					Female				
	mx	ax	lx	nLx	ex	mx	ax	lx	nLx	ex
Early Neonatal	0.181505908	0.009583478	100000	0.019144746	74.39797594	0.152064645	0.00958438	100000	0.019150147	79.80144949
Late Neonatal	0.019897573	0.028761635	99652.57818	0.057301558	74.6378572	0.026946239	0.02875969	99708.83493	0.057322286	80.01499522
Post Neonatal	0.003011366	0.461429913	99538.57639	0.917751165	74.6656979	0.003020138	0.46142929	99554.39342	0.917893257	80.08143456
1 to 4	0.000756183	1.998991756	99262.25614	3.964493331	73.94875292	0.00072628	1.999031627	99277.21791	3.965327736	79.38021227
5 to 9	0.000150721	2.499685998	98962.5714	4.946265198	70.16635378	0.000153453	2.499680306	98989.31709	4.947568063	75.60492821
10 to 14	0.000179416	2.90838614	98888.04634	4.942541667	65.21729472	0.000152129	2.668322776	98913.4155	4.943915112	70.66096828
15 to 19	0.000497106	2.784842568	98799.39556	4.934526684	60.27316023	0.0002744	2.698454937	98838.22339	4.938794067	65.71264093
20 to 24	0.000851576	2.630646471	98554.17299	4.917775625	55.41604316	0.000415584	2.676076825	98702.73458	4.930384078	60.79904039
25 to 29	0.001023972	2.617472602	98135.53646	4.894843771	50.6407661	0.000635562	2.642388794	98497.88604	4.917526067	55.9198012
30 to 34	0.001445597	2.626992879	97634.52741	4.865029583	45.8865551	0.00085147	2.625363223	98185.48746	4.899326317	51.08912161
35 to 39	0.001910604	2.646921308	96931.64722	4.824902601	41.19910752	0.001107963	2.649980183	97768.60883	4.875719331	46.29558433
40 to 44	0.002834049	2.677485725	96010.53388	4.769146999	36.56706063	0.001640504	2.691417441	97228.73349	4.843087877	41.53747224
45 to 49	0.004401338	2.7130715	94660.57853	4.685884788	32.04750288	0.002633014	2.707395939	96434.68402	4.792792402	36.8564546
50 to 54	0.007540893	2.692892489	92602.25474	4.550974385	27.6953729	0.004323863	2.668719305	95173.63782	4.711214395	32.30744259
55 to 59	0.01185393	2.641118355	89181.60023	4.338070738	23.64915471	0.006313217	2.63857156	93139.09761	4.588558892	27.95236598
60 to 64	0.016841824	2.626256447	84061.83139	4.042150615	19.92352569	0.008836793	2.636089628	90247.9668	4.420260241	23.76075813
65 to 69	0.025277323	2.605702119	77288.40204	3.645171926	16.43412869	0.013034608	2.653936042	86349.93125	4.189698493	19.70985531
70 to 74	0.036514309	2.587808766	68139.13941	3.133598072	13.28671059	0.020426649	2.65426492	80908.66115	3.861190489	15.85207706
75 to 79	0.056197999	2.540415219	56813.68301	2.500074333	10.4193753	0.033316648	2.663904125	73058.45767	3.39087131	12.26572389
80 to 84	0.079020265	2.469512213	42971.21466	1.795924498	7.971056393	0.062283107	2.584022477	61851.17873	2.69246781	9.002819432
85 to 89	0.137660908	2.413781702	29028.8506	1.075973576	5.639116949	0.115177581	2.490849737	45298.38346	1.764043839	6.349721327
90 to 94	0.232715327	2.179309214	14492.84876	0.440456217	3.935126236	0.20058873	2.269564728	25331.40202	0.823810635	4.429063022
95 plus	0.327429504	3.057906089	4399.884581	0.13597387	3.057906089	0.297449087	3.370571737	9090.188848	0.310526651	3.370571737

Table 15: Guinea-Bissau 2017 life table, by age and sex. mx=mortality rate, ax=mean person-years lived in an age interval among those who die in that age interval, lx=number of persons left alive at age x, nLx=person-years lived between age x and x+n, ex=life expectancy at age x.

Age Group	Male					Female				
	mx	ax	lx	nLx	ex	mx	ax	lx	nLx	ex
Early Neonatal	1.57343649	0.009540816	100000	0.018891663	57.32067103	1.166186945	0.009553298	100000	0.01896523	62.47953816
Late Neonatal	0.131405311	0.028730875	97028.23751	0.055614085	59.05473322	0.07567787	0.028746248	97788.59195	0.056139655	63.8723402
Post Neonatal	0.020649468	0.460176941	96297.85003	0.880690152	59.44463764	0.019828714	0.460235245	97363.86551	0.890772777	64.09321815
1 to 4	0.007402706	1.990129876	94480.65643	3.723879258	59.65567754	0.006395269	1.991473071	95598.29689	3.77546516	64.34519036
5 to 9	0.001538778	2.496794217	91727.09179	4.568763289	57.38781306	0.001197933	2.497504308	93185.35632	4.645345031	61.96062907
10 to 14	0.001289014	2.616391716	91024.34704	4.537278392	52.81130203	0.000912366	2.533802448	92629.00442	4.621050027	57.31762997
15 to 19	0.002286091	2.678827819	90439.67597	4.498124288	48.13548187	0.001354857	2.650422946	92207.47636	4.595755091	52.56791489
20 to 24	0.003313642	2.618769054	89411.57918	4.435593301	43.65696203	0.001921276	2.69182483	91584.88094	4.559035926	47.90646257
25 to 29	0.004289806	2.619857524	87942.31181	4.352695594	39.3409578	0.003179878	2.678429116	90709.19677	4.502218943	43.34188776
30 to 34	0.00599572	2.617566439	86076.12259	4.243226602	35.13514833	0.004740542	2.619222498	89278.28918	4.414094048	38.99234514
35 to 39	0.008041597	2.607554792	83534.18268	4.097950146	31.12276491	0.006076823	2.585035214	87187.34354	4.296364661	34.86338922
40 to 44	0.010843097	2.597455366	80242.88798	3.910422637	27.29035113	0.007596854	2.578184989	84578.88549	4.152620503	30.85710018
45 to 49	0.014355035	2.594706023	76009.72581	3.673883796	23.66345481	0.009529266	2.5984853	81427.65077	3.980406925	26.94896479
50 to 54	0.019648818	2.583961364	70746.93099	3.377434213	20.22887924	0.01314306	2.598384898	77639.78652	3.763404558	23.13380566
55 to 59	0.026342085	2.581473369	64129.42839	3.01501156	17.04891048	0.017575374	2.609189755	72702.47497	3.488894527	19.52494429
60 to 64	0.037809293	2.562506934	56214.52364	2.574376802	14.0871454	0.026012282	2.609764473	66587.56052	3.135106169	16.0750178
65 to 69	0.052778789	2.527691916	46518.89666	2.058572316	11.49305564	0.039038869	2.584852853	58461.3021	2.672340594	12.944373
70 to 74	0.074322763	2.483731479	35704.17047	1.505105632	9.216495555	0.059129929	2.542476519	48081.77824	2.100974439	10.18061328
75 to 79	0.10307147	2.426388944	24570.63714	0.972081319	7.278748521	0.088932639	2.485085669	35748.99894	1.463613472	7.821798345
80 to 84	0.145644607	2.351423185	14602.89879	0.527639731	5.607722105	0.136746521	2.399107492	22871.07028	0.846594026	5.847488493
85 to 89	0.211420261	2.230299201	6952.763616	0.219584192	4.209943466	0.204172928	2.259967546	11444.65513	0.368796788	4.335538076
90 to 94	0.302474258	2.035744841	2327.044871	0.061438429	3.1668889	0.292919992	2.062696	4009.681022	0.108425419	3.252833071
95 plus	0.382150869	2.616984619	473.5425234	0.012413628	2.616984619	0.378621357	2.642902479	868.8367121	0.023154192	2.642902479

Table 15: Guinea-Bissau 2100 life table, by age and sex. mx=mortality rate, ax=mean person-years lived in an age interval among those who die in that age interval, lx=number of persons left alive at age x, nLx=person-years lived between age x and x+n, ex=life expectancy at age x.

Age Group	Male					Female				
	mx	ax	lx	nLx	ex	mx	ax	lx	nLx	ex
Early Neonatal	0.244709273	0.009581541	100000	0.019133157	74.5900108	0.194525699	0.009583079	100000	0.019142356	79.47931798
Late Neonatal	0.018017977	0.028762153	99531.88756	0.057235252	74.92126804	0.014392108	0.028763153	99627.67716	0.057296312	79.75677055
Post Neonatal	0.002484795	0.46146732	99428.7697	0.916961526	74.94133131	0.003020481	0.461429266	99545.22908	0.917808657	79.76514971
1 to 4	0.000600239	1.999199682	99200.97338	3.963281062	74.18875	0.000550511	1.999265986	99268.06008	3.966355668	79.06275305
5 to 9	0.000209255	2.499564052	98963.18546	4.945572487	70.36180577	0.000165012	2.499656225	99049.81443	4.950449331	75.23205087
10 to 14	0.000229549	2.84219417	98859.73408	4.940521089	65.43263786	0.000169463	2.698590859	98968.17158	4.946471207	70.29187215
15 to 19	0.000568942	2.787851877	98746.35956	4.9310942	60.50437161	0.000318018	2.716734735	98884.39375	4.940642137	65.34900232
20 to 24	0.00100102	2.67592283	98465.90899	4.91187483	55.66845092	0.000509445	2.729115096	98727.35539	4.930707386	60.44835207
25 to 29	0.001424775	2.638688407	97974.541	4.882345605	50.9333955	0.000921916	2.684095097	98476.34144	4.913291462	55.59493602
30 to 34	0.002002506	2.612902672	97279.57192	4.840832521	46.27716084	0.001283325	2.629325623	98024.11995	4.886216685	50.83834125
35 to 39	0.002511323	2.620805582	96311.66882	4.78698968	41.71425687	0.001575087	2.638442733	97398.74091	4.851796428	46.14727763
40 to 44	0.003483808	2.626787486	95111.97851	4.716617645	37.2053167	0.002212445	2.652966804	96636.84078	4.806795167	41.48950093
45 to 49	0.004682485	2.662778603	93472.79704	4.623088148	32.80874123	0.003100324	2.656480678	95576.15663	4.744313388	36.91898905
50 to 54	0.007332719	2.651200058	91313.94358	4.488527836	28.51792735	0.004553084	2.637039692	94108.54514	4.65538488	32.45085814
55 to 59	0.010481721	2.635956087	88033.33827	4.295532071	24.47741697	0.00624812	2.620784649	91993.85608	4.532421161	28.13285907
60 to 64	0.015337963	2.63504137	83548.50575	4.031805104	20.64545605	0.008508925	2.632388871	89170.31695	4.370744309	23.93671093
65 to 69	0.023156703	2.60358796	77394.63468	3.667496232	17.07314822	0.01252739	2.653188941	85463.54714	4.151634874	19.85514465
70 to 74	0.032436808	2.593208884	68961.0374	3.200406055	13.84053708	0.019574928	2.662765331	80289.10862	3.839981447	15.95727905
75 to 79	0.05014868	2.564619803	58677.03972	2.617845052	10.81450421	0.032718003	2.676138242	72829.46859	3.386759639	12.31240432
80 to 84	0.074954296	2.506213136	45705.46165	1.929767425	8.167320297	0.062327166	2.593852131	61886.86206	2.696862583	9.013833599
85 to 89	0.132912488	2.427009226	31445.85552	1.176372712	5.754892621	0.115199237	2.491233092	45378.56312	1.769411549	6.356636671
90 to 94	0.22794451	2.188273237	16043.55315	0.491447945	3.997337428	0.200533256	2.269287018	25457.27751	0.829526219	4.43360029
95 plus	0.323582549	3.093486164	4977.490519	0.155263163	3.093486164	0.297370082	3.373495119	9188.591329	0.315305134	3.373495119

Table 15: Liberia 2017 life table, by age and sex. mx=mortality rate, ax=mean person-years lived in an age interval among those who die in that age interval, lx=number of persons left alive at age x, nLx=person-years lived between age x and x+n, ex=life expectancy at age x.

Age Group	Male					Female				
	mx	ax	lx	nLx	ex	mx	ax	lx	nLx	ex
Early Neonatal	1.036979966	0.009557258	100000	0.018988657	64.33790934	0.811292583	0.009564175	100000	0.019029665	65.61255279
Late Neonatal	0.101643682	0.028739085	98031.18449	0.056236957	65.60952826	0.078842009	0.028745375	98456.26211	0.056517804	66.62166795
Post Neonatal	0.025150746	0.459857185	97459.75382	0.889471161	65.9363056	0.024275078	0.459919389	98010.74204	0.894857945	66.86645101
1 to 4	0.00645273	1.991396475	95223.76862	3.760279724	66.55030574	0.00601329	1.991982371	95839.07824	3.787859474	67.44806293
5 to 9	0.001009054	2.497897805	92801.28716	4.628388871	64.23673769	0.000946433	2.498028265	93563.50561	4.667127117	65.04038749
10 to 14	0.00078832	2.580290698	92334.6628	4.607946872	59.54916991	0.000690889	2.547281219	93121.92871	4.64822164	60.3368268
15 to 19	0.001322216	2.666465957	91971.59256	4.584442071	54.77411479	0.001111646	2.658369853	92800.87199	4.628000227	55.53674629
20 to 24	0.001868902	2.601140388	91365.53406	4.547892154	50.11925194	0.001550252	2.628242483	92286.51638	4.597428727	50.83139659
25 to 29	0.002263392	2.593131864	90515.7892	4.501279252	45.56449877	0.002094176	2.62771348	91574.01113	4.556077699	46.20624815
30 to 34	0.002940073	2.622894822	89497.376	4.443826016	41.05267097	0.002888084	2.643968305	90620.19685	4.500388353	41.66452392
35 to 39	0.004095923	2.630661169	88191.57249	4.367215636	36.62080172	0.004178728	2.626757287	89320.8919	4.422200079	37.23145448
40 to 44	0.005685539	2.634687606	86404.05347	4.262915996	32.32292673	0.005609183	2.609673656	87473.73297	4.315858788	32.96119543
45 to 49	0.00814333	2.630149508	83982.25613	4.119697435	28.17730139	0.007491305	2.622155448	85053.88856	4.178336646	28.82213849
50 to 54	0.011538099	2.62228348	80630.77419	3.924055421	24.23658487	0.010689079	2.61167226	81925.96965	3.994468593	24.81875638
55 to 59	0.016425839	2.623048604	76110.57166	3.662821408	20.51683817	0.014437043	2.624242559	77662.83386	3.754635886	21.03294574
60 to 64	0.024365644	2.594527153	70108.63804	3.311888432	17.0457854	0.021816905	2.614077187	72255.63264	3.434589143	17.40636701
65 to 69	0.033655896	2.60907442	62064.67616	2.872965	13.91736964	0.031607867	2.621720501	64788.8697	3.013990913	14.10785148
70 to 74	0.057054286	2.542325794	52436.06568	2.300849376	10.99513087	0.053291075	2.557718187	55312.90781	2.449034624	11.07505418
75 to 79	0.074434801	2.473748269	39377.80616	1.658964453	8.805359579	0.073368966	2.501433337	42351.83667	1.791916689	8.687920708
80 to 84	0.109396357	2.443215196	27107.95728	1.060684798	6.685114947	0.11658842	2.445691478	29316.18896	1.132250995	6.453766486
85 to 89	0.173575983	2.318096562	15575.92152	0.532415772	4.845049679	0.182308554	2.306705213	16251.41434	0.546961551	4.707042731
90 to 94	0.267805921	2.109662949	6383.284612	0.180264084	3.509446041	0.271387842	2.111909856	6378.977752	0.179596693	3.469980597
95 plus	0.355395985	2.814263572	1574.404423	0.044419794	2.814263572	0.360375687	2.776448919	1546.364846	0.043203536	2.776448919

Table 15: Liberia 2100 life table, by age and sex. mx=mortality rate, ax=mean person-years lived in an age interval among those who die in that age interval, lx=number of persons left alive at age x, nLx=person-years lived between age x and x+n, ex=life expectancy at age x.

Age Group	Male					Female				
	mx	ax	lx	nLx	ex	mx	ax	lx	nLx	ex
Early Neonatal	0.159926522	0.009584139	100000	0.019148744	77.79269188	0.143591379	0.00958464	100000	0.019151737	80.38320074
Late Neonatal	0.013614739	0.028763368	99694.41496	0.05733602	78.00594696	0.015347749	0.02876289	99725.57459	0.057351095	80.579464
Post Neonatal	0.002448518	0.461469897	99616.53728	0.918712537	78.00822241	0.002689118	0.461452805	99637.79135	0.918806561	80.59143181
1 to 4	0.000586411	1.999218119	99392.58301	3.971064854	77.25592374	0.000644414	1.999140782	99391.72246	3.970571245	79.86254189
5 to 9	0.00019607	2.499591521	99160.81008	4.95561695	73.42828931	0.000200049	2.499583231	99137.01998	4.954379509	76.0593714
10 to 14	0.000199488	2.823473598	99063.89089	4.951059445	68.49668335	0.000188678	2.658494613	99038.1852	4.94971112	71.13218838
15 to 19	0.000516613	2.754026755	98965.35966	4.942579293	63.56116273	0.000326779	2.666548485	98945.06952	4.943478441	66.19620593
20 to 24	0.000861673	2.630598068	98710.91336	4.925500819	58.71567835	0.000435879	2.671476583	98783.95369	4.934178943	61.29935507
25 to 29	0.001005383	2.619339592	98289.59761	4.902724738	53.95357672	0.000663934	2.676094064	98569.46478	4.920885346	56.42637303
30 to 34	0.001329428	2.613995921	97800.88986	4.87459407	49.20909044	0.000990133	2.6342993	98243.47783	4.900671267	51.60393322
35 to 39	0.001647399	2.612208715	97157.54955	4.838874635	44.51675247	0.001263726	2.62050344	97759.33407	4.873284303	46.8454928
40 to 44	0.00216586	2.640619851	96364.96107	4.793854933	39.86029081	0.001678551	2.649398427	97144.88185	4.838206081	42.12407151
45 to 49	0.003144436	2.679196388	95331.92326	4.73224829	35.26017939	0.002506632	2.687292199	96334.86385	4.789096627	37.45315888
50 to 54	0.005017078	2.679545744	93851.57895	4.638830263	30.76742773	0.004048838	2.648867294	95138.44101	4.71227602	32.88479554
55 to 59	0.007644639	2.647417548	91541.38836	4.496796389	26.46573469	0.005537664	2.624012695	93241.70007	4.601892105	28.49047134
60 to 64	0.0109047	2.637402783	88138.12574	4.297707128	22.37250665	0.007557687	2.626866913	90714.48051	4.456672593	24.20101519
65 to 69	0.016270501	2.698714179	83519.83781	4.027675531	18.44721859	0.010851579	2.704566274	87383.69605	4.264277645	20.01004235
70 to 74	0.029358066	2.62986248	77112.71646	3.610638052	14.74501681	0.019576714	2.680066025	82831.75044	3.964782286	15.94794204
75 to 79	0.042825305	2.579556197	66800.82476	3.035724251	11.61384948	0.031822819	2.696481659	75233.05405	3.510651336	12.27647567
80 to 84	0.065997718	2.545841301	54249.93308	2.348126051	8.727849215	0.065621148	2.598306067	64363.26483	2.796105802	8.891600991
85 to 89	0.121058784	2.462834323	39399.49487	1.522931416	6.109858032	0.119209909	2.481417216	46807.75351	1.822168289	6.278690291
90 to 94	0.21519652	2.207568365	21737.16685	0.688189601	4.190900122	0.204552874	2.259221666	26192.63395	0.854921283	4.389509502
95 plus	0.31305872	3.204219709	7415.459998	0.242757869	3.204219709	0.300862815	3.34648666	9520.527544	0.329681106	3.34648666

Table 15: Mali 2017 life table, by age and sex. mx=mortality rate, ax=mean person-years lived in an age interval among those who die in that age interval, lx=number of persons left alive at age x, nLx=person-years lived between age x and x+n, ex=life expectancy at age x.

Age Group	Male					Female				
	mx	ax	lx	nLx	ex	mx	ax	lx	nLx	ex
Early Neonatal	1.970544609	0.009528646	100000	0.018820283	60.82428978	1.380079877	0.009546742	100000	0.01892653	62.84950283
Late Neonatal	0.136225001	0.028729546	96292.32835	0.055184645	63.14355941	0.124200511	0.028732863	97388.28685	0.055831949	64.51420474
Post Neonatal	0.037454049	0.458983217	95541.00395	0.867051866	63.58199705	0.033240967	0.45928249	96695.03464	0.879217416	64.91877215
1 to 4	0.01509424	1.979875617	92296.28716	3.582748644	64.87748003	0.014177233	1.98109806	93773.65307	3.646641777	66.00328029
5 to 9	0.001589847	2.496687822	86896.09177	4.327601425	64.79043495	0.001165431	2.49757202	88607.60956	4.417502468	65.73792939
10 to 14	0.000972598	2.436032136	86208.87942	4.299729956	60.2876731	0.000774971	2.486925308	88092.99013	4.396092328	61.10730549
15 to 19	0.001308457	2.643968399	85790.9631	4.276372545	55.56953919	0.001130125	2.654378365	87752.38067	4.376023621	56.33465685
20 to 24	0.001903287	2.607225543	85231.4822	4.24225524	50.9164355	0.001630842	2.639571962	87257.89333	4.346167912	51.63841275
25 to 29	0.002323391	2.591266837	84424.08802	4.197715789	46.37754578	0.002253102	2.625619763	86549.23387	4.304439338	47.03889154
30 to 34	0.002981036	2.605581399	83448.93459	4.142881074	41.88828944	0.003043882	2.624482194	85579.67512	4.248271993	42.54124874
35 to 39	0.003930602	2.609312783	82214.23293	4.072456933	37.47718923	0.004171058	2.599084968	84287.04153	4.172584414	38.15253945
40 to 44	0.005210261	2.619052893	80614.17428	3.981346479	33.16798267	0.005199904	2.590764669	82547.52596	4.076334346	33.90050518
45 to 49	0.007216723	2.6241224	78541.0312	3.860907937	28.97276467	0.006724651	2.608581107	80429.18366	3.9578622	29.72371689
50 to 54	0.010098876	2.626341039	75757.12866	3.699283482	24.93897236	0.009223903	2.616196332	77769.69737	3.804913981	25.64922236
55 to 59	0.014503664	2.637721106	72026.09397	3.482187505	21.09262364	0.012820271	2.634842813	74264.07138	3.604088925	21.73436071
60 to 64	0.022213399	2.617835218	66984.79143	3.181257983	17.4795715	0.019530948	2.631628656	69651.09909	3.328909675	17.99713857
65 to 69	0.032524281	2.59895462	59934.37912	2.780235196	14.22662656	0.029572754	2.618030105	63164.83455	2.951008948	14.57303261
70 to 74	0.050080944	2.559374135	50919.29135	2.269678469	11.28556995	0.046768854	2.581273572	54466.42181	2.447653169	11.48176396
75 to 79	0.073387478	2.509100208	39600.03056	1.675402066	8.783700596	0.072186794	2.524582143	43070.93854	1.828682645	8.839637118
80 to 84	0.111966988	2.44300833	27371.14071	1.065438418	6.597228312	0.111664723	2.44728868	29947.32879	1.16704715	6.616889622
85 to 89	0.176478362	2.310962661	15512.94363	0.527057922	4.790988275	0.176654905	2.319489855	17003.38365	0.578300952	4.810678363
90 to 94	0.270521964	2.103916424	6262.581634	0.175955319	3.4804686	0.265759337	2.124907047	6853.976082	0.194820159	3.530284791
95 plus	0.357517482	2.797635396	1522.677103	0.042720602	2.797635396	0.355555956	2.813550408	1705.373332	0.04818093	2.813550408

Table 15: Mali 2100 life table, by age and sex. mx=mortality rate, ax=mean person-years lived in an age interval among those who die in that age interval, lx=number of persons left alive at age x, nLx=person-years lived between age x and x+n, ex=life expectancy at age x.

Age Group	Male					Female				
	mx	ax	lx	nLx	ex	mx	ax	lx	nLx	ex
Early Neonatal	0.247477375	0.009581456	100000	0.019132651	79.26657465	0.176203272	0.00958364	100000	0.019145719	83.50561821
Late Neonatal	0.014895276	0.028763014	99526.6308	0.05723737	79.62381607	0.019223265	0.028761821	99662.70267	0.057308494	83.76842552
Post Neonatal	0.003703267	0.461380762	99441.38285	0.916562533	79.63443284	0.003554487	0.461391331	99552.56239	0.917650256	83.80334992
1 to 4	0.001082233	1.998557023	99102.042	3.955519945	78.98199296	0.001014188	1.99864775	99226.46506	3.961023173	83.15344365
5 to 9	0.000209391	2.499563768	98674.30704	4.931135031	75.31514659	0.000139533	2.499709306	98824.99433	4.939527441	79.48239868
10 to 14	0.000182679	2.635994837	98571.11337	4.926434666	70.39127224	0.0001356	2.615409514	98756.11241	4.93620827	74.53601807
15 to 19	0.000334633	2.728747421	98481.16187	4.920330514	65.45303938	0.000212735	2.672489089	98689.22057	4.932016838	69.58470266
20 to 24	0.000561179	2.652938854	98316.63148	4.909356137	60.55776481	0.000308642	2.707330645	98584.36491	4.925730738	64.65580243
25 to 29	0.000731374	2.636443392	98041.55352	4.89359845	55.71982695	0.000517486	2.676136839	98432.41629	4.915714265	59.75135303
30 to 34	0.001011343	2.616941185	97684.29724	4.872458698	50.91359704	0.000750863	2.624931804	98178.18075	4.900142629	54.89889346
35 to 39	0.001280915	2.616294125	97192.17231	4.844815318	46.15766444	0.000939431	2.604656475	97810.54963	4.879529617	50.09510639
40 to 44	0.001725674	2.643997549	96572.37567	4.809065662	41.43640517	0.001204	2.639614968	97352.61692	4.853793814	45.31807579
45 to 49	0.00248482	2.688440085	95743.47384	4.759861093	36.77108574	0.001707596	2.662442166	96768.76814	4.819206275	40.57502452
50 to 54	0.004047603	2.696519891	94562.35356	4.68446409	32.19458404	0.002562502	2.663664966	95946.42521	4.76877491	35.89875753
55 to 59	0.006447655	2.677748781	92670.05595	4.565207207	27.79369021	0.003769601	2.64034481	94725.88482	4.694549274	31.32501179
60 to 64	0.00990751	2.654443813	89735.08684	4.385115569	23.6113571	0.005199228	2.643950796	92959.30524	4.591862603	26.8676752
65 to 69	0.014878615	2.635619674	85405.04332	4.125710485	19.66928603	0.007716332	2.6723107	90577.39395	4.44917132	22.50061572
70 to 74	0.022060097	2.635430766	79296.16283	3.769419672	15.97694156	0.012263938	2.702026255	87157.15524	4.238903271	18.27384542
75 to 79	0.03511921	2.608500707	71039.56027	3.279070121	12.52469456	0.021557572	2.726469646	81987.60046	3.909001269	14.25027968
80 to 84	0.054044237	2.558453049	59637.91455	2.638057311	9.423435679	0.042523818	2.640886868	73630.8603	3.349357552	10.55361109
85 to 89	0.105418778	2.508929092	45557.35177	1.809412343	6.555027514	0.086513604	2.592177425	59562.22561	2.471505746	7.419062973
90 to 94	0.198662062	2.233688727	26745.52907	0.866814227	4.43337879	0.1665433	2.327355931	38535.19539	1.340651391	5.075601031
95 plus	0.299495159	3.342974671	9726.834107	0.327795556	3.342974671	0.265662208	3.776266435	16589.84045	0.634843342	3.776266435

Table 15: Mauritania 2017 life table, by age and sex. mx=mortality rate, ax=mean person-years lived in an age interval among those who die in that age interval, lx=number of persons left alive at age x, nLx=person-years lived between age x and x+n, ex=life expectancy at age x.

Age Group	Male					Female				
	mx	ax	lx	nLx	ex	mx	ax	lx	nLx	ex
Early Neonatal	1.020346822	0.009557768	100000	0.018991675	70.3635822	0.724374488	0.009566839	100000	0.019045491	71.34777514
Late Neonatal	0.092732824	0.028741543	98062.43798	0.056269287	71.73392526	0.082454328	0.028744378	98620.48736	0.056606201	72.32624985
Post Neonatal	0.013199743	0.460706151	97540.79308	0.89512021	72.05984162	0.011608542	0.460819186	98153.82382	0.901403928	72.61243555
1 to 4	0.002326891	1.996897483	96360.20126	3.836539249	72.0140208	0.002099507	1.997200661	97107.66647	3.868051239	72.46652591
5 to 9	0.000718584	2.498502951	95468.16781	4.764847213	68.66871939	0.000558742	2.498835954	96296.10006	4.808086914	69.06068531
10 to 14	0.000540669	2.550511032	95125.92661	4.750008135	63.90690883	0.000434431	2.568880668	96027.50208	4.796310254	64.24677455
15 to 19	0.000855481	2.667323379	94869.1702	4.734014243	59.07290199	0.000705603	2.646365753	95819.17053	4.783016628	59.38088213
20 to 24	0.001238837	2.580085443	94464.18602	4.709092031	54.31427752	0.000935615	2.597508762	95481.72181	4.763381743	54.58134259
25 to 29	0.001343644	2.549044159	93880.86755	4.678638027	49.63514417	0.001153692	2.613262371	95036.11666	4.738761278	49.82488864
30 to 34	0.001574089	2.584087528	93252.35106	4.644960748	44.95197761	0.001580261	2.647606202	94489.49303	4.706982658	45.09761516
35 to 39	0.002009059	2.618594228	92521.40292	4.604054323	40.2861003	0.002306142	2.653788083	93745.79069	4.662081367	40.43378363
40 to 44	0.002767925	2.661002887	91596.77508	4.550397231	35.66547437	0.003356632	2.652494012	92670.89593	4.597341292	35.87096799
45 to 49	0.004249363	2.684217068	90337.88787	4.472912217	31.12418104	0.004903798	2.661762084	91128.37693	4.504797914	31.43131922
50 to 54	0.006758781	2.685865719	88438.54467	4.35389592	26.73309304	0.007455766	2.646942672	88920.93638	4.369470059	27.14309488
55 to 59	0.010854358	2.683408939	85498.98356	4.170231308	22.55717112	0.010781955	2.646581183	85667.17816	4.177519091	23.07051959
60 to 64	0.017803132	2.653065803	80979.93767	3.886933652	18.6628651	0.016416872	2.644605534	81171.48401	3.907818441	19.19869642
65 to 69	0.027631727	2.619897095	74076.40346	3.475958044	15.15151837	0.025389137	2.628699898	74772.50814	3.527006992	15.61289113
70 to 74	0.042531758	2.584163521	64505.29804	2.926047951	12.00872175	0.039644184	2.597534664	65851.86832	3.007369626	12.37073934
75 to 79	0.065120954	2.544571624	52122.32064	2.248988893	9.249185312	0.061965034	2.554305983	53982.05403	2.345658747	9.521793582
80 to 84	0.104881295	2.473229009	37579.62269	1.488085906	6.852843011	0.099432139	2.485487422	39531.06207	1.583646279	7.076080604
85 to 89	0.168751398	2.330345999	22105.29673	0.764163606	4.940980955	0.162583068	2.353667133	23902.44257	0.837698104	5.095635707
90 to 94	0.263221127	2.119203591	9317.392163	0.265833446	3.560797102	0.251510412	2.157953148	10386.28385	0.303817655	3.6955899
95 plus	0.351787162	2.843690734	2366.363508	0.067598882	2.843690734	0.343187139	2.915394095	2795.505808	0.08190324	2.915394095

Table 15: Mauritania 2100 life table, by age and sex. mx=mortality rate, ax=mean person-years lived in an age interval among those who die in that age interval, lx=number of persons left alive at age x, nLx=person-years lived between age x and x+n, ex=life expectancy at age x.

Age Group	Male					Female				
	mx	ax	lx	nLx	ex	mx	ax	lx	nLx	ex
Early Neonatal	0.161104679	0.009584103	100000	0.019148488	80.41335324	0.14110004	0.009584716	100000	0.019152159	82.62685078
Late Neonatal	0.016535982	0.028762562	99691.55126	0.057329509	80.64272398	0.024156191	0.02876046	99729.79469	0.057338935	82.83136569
Post Neonatal	0.001683289	0.461524258	99596.75846	0.918850565	80.66185625	0.001826841	0.46151406	99591.30271	0.918739291	82.88887589
1 to 4	0.000236687	1.999684417	99442.14339	3.975804315	79.86314907	0.000223582	1.999701891	99423.49293	3.975162733	82.10459007
5 to 9	0.000162671	2.499661102	99348.09046	4.965385257	75.93678414	0.00013284	2.499723251	99334.66055	4.965084233	78.17614016
10 to 14	0.000160838	2.721444471	99267.33129	4.961545347	70.99643874	0.000143887	2.675762302	99268.71643	4.961773966	73.22634338
15 to 19	0.00033115	2.702994505	99187.53869	4.955602324	66.05132494	0.000251935	2.604163952	99197.33181	4.956873879	68.27707217
20 to 24	0.000479356	2.577041519	99023.45275	4.945425624	61.15617787	0.000269321	2.566481789	99072.46305	4.950379777	63.35978302
25 to 29	0.000506196	2.55326481	98786.44209	4.933218616	56.29656305	0.000339414	2.628263334	98939.155	4.942979159	58.44162729
30 to 34	0.00061639	2.580327084	98536.79459	4.919502254	51.4324614	0.000479572	2.648554308	98771.4101	4.933003096	53.53627911
35 to 39	0.000744736	2.610921135	98233.68855	4.902961794	46.58280323	0.000678491	2.66285481	98534.8868	4.918946603	48.65822274
40 to 44	0.001015556	2.676527626	97868.75665	4.881915238	41.74621057	0.001016217	2.699049415	98201.21852	4.898609693	43.81412853
45 to 49	0.001607857	2.733162157	97373.33197	4.850979022	36.94413249	0.001662442	2.734340138	97703.58965	4.866845496	39.02264628
50 to 54	0.00282882	2.746727129	96594.14954	4.799056713	32.21866547	0.002913746	2.694318142	96894.96689	4.812434547	34.3237377
55 to 59	0.004966915	2.728050923	95238.57236	4.708724294	27.63558795	0.004468781	2.658628	95494.17856	4.725238209	29.78459794
60 to 64	0.008412606	2.700795313	92904.83035	4.557260737	23.25780652	0.006414936	2.647381218	93386.71841	4.600087849	25.39269646
65 to 69	0.013913506	2.675160224	89082.42433	4.31501607	19.13280174	0.009536613	2.673822357	90442.3533	4.42424957	21.12626597
70 to 74	0.022238323	2.667027011	83110.58464	3.951998241	15.30603576	0.015424326	2.689032683	86241.58608	4.164579385	17.01594172
75 to 79	0.038189091	2.64379749	74396.57283	3.416527012	11.77517613	0.026702342	2.719748369	79864.70001	3.766305912	13.14747609
80 to 84	0.066466425	2.564531484	61551.46705	2.656682746	8.675491572	0.054531324	2.636784403	69941.2609	3.10561181	9.614983705
85 to 89	0.121996692	2.459530463	44280.96813	1.701224474	6.071222285	0.104037599	2.530413594	53409.06787	2.139123506	6.766403185
90 to 94	0.216338032	2.206498702	24077.91965	0.757533609	4.169506127	0.187345689	2.292227835	31901.7753	1.071317577	4.680620839
95 plus	0.314038024	3.191983285	8067.347308	0.261660994	3.191983285	0.285073919	3.528402756	12511.12208	0.453013393	3.528402756

Table 15: Niger 2017 life table, by age and sex. mx=mortality rate, ax=mean person-years lived in an age interval among those who die in that age interval, lx=number of persons left alive at age x, nLx=person-years lived between age x and x+n, ex=life expectancy at age x.

Age Group	Male					Female				
	mx	ax	lx	nLx	ex	mx	ax	lx	nLx	ex
Early Neonatal	1.036192167	0.009557282	100000	0.0189888	61.78128386	0.68754279	0.009567968	100000	0.019052202	64.23494697
Late Neonatal	0.11637399	0.028735022	98032.6648	0.056214013	63.0000017	0.09234694	0.028741649	98690.15437	0.056630082	65.06754688
Post Neonatal	0.028032738	0.45965246	97378.68999	0.887555487	63.36493167	0.027345309	0.459701291	98167.26536	0.895022762	65.3562379
1 to 4	0.014700307	1.980400766	94891.97085	3.686341594	64.0891378	0.014768675	1.980309607	95720.44961	3.717976238	66.09129413
5 to 9	0.001338307	2.497211863	89478.79132	4.459022039	63.84844233	0.00109232	2.497724334	90232.79492	4.499346809	65.99172406
10 to 14	0.000901814	2.505570855	88882.76019	4.434169286	59.26042399	0.00073827	2.50400251	89741.52664	4.478826952	61.3391169
15 to 19	0.001374535	2.667422943	88483.17795	4.410028865	54.51680201	0.001117982	2.65212791	89410.95615	4.458849535	56.55644053
20 to 24	0.002034782	2.598562658	87877.14739	4.37249534	49.87380211	0.001572612	2.614037827	88912.54433	4.429014429	51.85812398
25 to 29	0.002374755	2.579035953	86987.61418	4.324525379	45.3563117	0.002006268	2.602858136	88216.20281	4.389703985	47.24599664
30 to 34	0.002997941	2.610201408	85960.95955	4.267486815	40.86629588	0.002604507	2.627417353	87335.83084	4.339982997	42.69506436
35 to 39	0.004063955	2.620580408	84682.12944	4.193578951	36.44292157	0.00367758	2.610088042	86205.98015	4.272766206	38.219149
40 to 44	0.005534513	2.634011867	82978.8715	4.095360533	32.13580811	0.004686422	2.608975149	84635.50151	4.184915669	33.87844924
45 to 49	0.007987263	2.632749905	80714.15615	3.960901172	27.9618124	0.006381566	2.627801124	82675.65201	4.072199687	29.61784175
50 to 54	0.011346167	2.631486109	77554.1743	3.776387718	23.99169188	0.009080015	2.633673622	80079.4945	3.919868725	25.49053196
55 to 59	0.016645292	2.632501172	73276.86175	3.525215329	20.23623265	0.013156198	2.645527997	76525.55332	3.711535407	21.54911013
60 to 64	0.025167279	2.605563304	67423.20762	3.180046174	16.76286665	0.020394019	2.628920946	71652.98143	3.417853305	17.83167799
65 to 69	0.036263504	2.584947282	59443.36961	2.733603845	13.66325982	0.030470993	2.614020982	64704.46186	3.016779861	14.46182434
70 to 74	0.055163894	2.542214252	49566.47847	2.183588133	10.87311472	0.048226742	2.574148785	55551.62304	2.488126641	11.41376091
75 to 79	0.0787985	2.485507258	37574.97201	1.569462247	8.538470712	0.072832717	2.516406638	43621.789	1.848981804	8.836130423
80 to 84	0.114861676	2.42034203	25273.18287	0.976002678	6.497424592	0.110955909	2.440729937	30249.10708	1.180126398	6.642744055
85 to 89	0.179423322	2.303694673	14117.65664	0.476457096	4.735651562	0.175720735	2.321715278	17258.7601	0.588355652	4.829082541
90 to 94	0.273283555	2.098089024	5604.334153	0.156526833	3.45081017	0.264818143	2.127090183	6998.253633	0.199376665	3.540966169
95 plus	0.359676744	2.780619015	1339.681449	0.037325533	2.780619015	0.354741979	2.820127888	1752.349626	0.049654959	2.820127888

Table 15: Niger 2100 life table, by age and sex. mx=mortality rate, ax=mean person-years lived in an age interval among those who die in that age interval, lx=number of persons left alive at age x, nLx=person-years lived between age x and x+n, ex=life expectancy at age x.

Age Group	Male					Female				
	mx	ax	lx	nLx	ex	mx	ax	lx	nLx	ex
Early Neonatal	0.137212569	0.009584836	100000	0.019152874	78.24385295	0.091529078	0.009586236	100000	0.019161261	83.08844971
Late Neonatal	0.01201532	0.028763809	99737.23894	0.057363241	78.43038786	0.012502071	0.028763675	99824.63238	0.057412701	83.21487751
Post Neonatal	0.001836779	0.461513354	99668.32255	0.919445667	78.42694484	0.001802763	0.46151577	99752.86143	0.920239946	83.21705926
1 to 4	0.000931833	1.998757557	99499.49122	3.972574276	77.63558682	0.000801355	1.998931527	99587.00409	3.977104447	82.43111368
5 to 9	0.000184652	2.499615308	99129.4842	4.954187688	73.91732619	0.000151538	2.499684295	99268.40787	4.961540977	78.68864684
10 to 14	0.000174453	2.774458452	99038.03843	4.949973642	68.98312227	0.00014472	2.621277605	99193.24117	4.957952913	73.74623849
15 to 19	0.00040842	2.746855298	98951.70727	4.943038263	64.04080062	0.000233377	2.629711655	99121.50801	4.953334894	68.79759948
20 to 24	0.000661207	2.617584891	98749.86168	4.929726345	59.16577478	0.000289518	2.630366499	99005.9334	4.946902556	63.87468127
25 to 29	0.000782551	2.597719513	98423.9918	4.91196762	54.35240913	0.000414483	2.646052212	98862.74804	4.938318987	58.96310808
30 to 34	0.001034299	2.609422563	98039.74257	4.889899675	49.55441961	0.000580454	2.640820755	98658.12381	4.926156397	54.07945716
35 to 39	0.001334836	2.614571842	97534.22737	4.861255038	44.79652325	0.000804533	2.630529429	98372.28685	4.909256746	49.22832786
40 to 44	0.001801323	2.670003795	96885.77322	4.824068987	40.07703415	0.001088173	2.667691858	97977.52016	4.886495	44.41485092
45 to 49	0.002852959	2.697926289	96017.84699	4.769623206	35.41201399	0.001709502	2.706820745	97446.12918	4.853299319	39.64076818
50 to 54	0.004620042	2.7050587	94659.72153	4.683377411	30.87645612	0.002821187	2.688251528	96617.55456	4.799594483	34.95383779
55 to 59	0.007562855	2.673398906	92503.4788	4.545431207	26.52550373	0.004305603	2.664765581	95266.69514	4.715997321	30.40552246
60 to 64	0.011444106	2.65756173	89082.60002	4.338494819	22.43244632	0.0063574	2.651503868	93243.7653	4.593907133	25.99991493
65 to 69	0.017711686	2.639828347	84152.32376	4.040248685	18.58133193	0.009346395	2.673200239	90339.71692	4.421426828	21.74073326
70 to 74	0.026693588	2.622414604	77073.01703	3.626416159	15.03767959	0.015036504	2.681297702	86243.14956	4.168271334	17.63629299
75 to 79	0.041642428	2.584955682	67527.46133	3.072927055	11.79095423	0.025189769	2.689016907	80048.62133	3.785397115	13.7843333
80 to 84	0.062285201	2.528358036	54974.13838	2.38951881	8.904393177	0.046282381	2.613051425	70683.06448	3.189883018	10.25000925
85 to 89	0.116382626	2.475837611	40442.79288	1.572096163	6.223731146	0.091956925	2.572692322	56278.15056	2.312807207	7.21142988
90 to 94	0.21049879	2.216383595	22591.59106	0.717866061	4.252099735	0.173056975	2.316578325	35636.10212	1.228379039	4.949754024
95 plus	0.309271316	3.239228182	7776.332878	0.255200062	3.239228182	0.271758036	3.697245678	14979.11062	0.565610086	3.697245678

Table 15: Nigeria 2017 life table, by age and sex. mx=mortality rate, ax=mean person-years lived in an age interval among those who die in that age interval, lx=number of persons left alive at age x, nLx=person-years lived between age x and x+n, ex=life expectancy at age x.

Age Group	Male					Female				
	mx	ax	lx	nLx	ex	mx	ax	lx	nLx	ex
Early Neonatal	1.657493146	0.00953824	100000	0.018876512	62.74943086	1.210420462	0.009551942	100000	0.018957217	66.01139104
Late Neonatal	0.145229257	0.028727062	96871.82777	0.055502375	64.75256122	0.111897038	0.028736257	97705.65607	0.056033702	67.54025397
Post Neonatal	0.033290549	0.459278969	96066.1358	0.873481938	65.23713428	0.029750116	0.459530464	97078.84681	0.884123682	67.91822588
1 to 4	0.011626734	1.984498263	93160.12848	3.641157602	66.33278812	0.010971198	1.985372218	94449.70466	3.696343682	68.87181669
5 to 9	0.001370875	2.497144013	88930.82118	4.431347571	65.39289269	0.000958631	2.498002853	90397.3599	4.509058734	67.87083651
10 to 14	0.000865196	2.417658055	88323.77719	4.406354015	60.82542826	0.000628649	2.4586304	89965.33549	4.491103856	63.18476105
15 to 19	0.00104602	2.65179614	87942.76149	4.386380542	56.07831829	0.000850682	2.684933365	89683.10759	4.47535477	58.37541276
20 to 24	0.001652448	2.674779946	87484.25429	4.35748518	51.35753654	0.001404184	2.713672945	89302.54942	4.45084864	53.61164054
25 to 29	0.002466896	2.658514549	86764.76795	4.313347666	46.75990974	0.002319881	2.680374671	88677.91616	4.410164214	48.96843615
30 to 34	0.003603848	2.635084591	85701.64136	4.248889269	42.30535055	0.003457143	2.631408737	87655.59776	4.347179143	44.50617265
35 to 39	0.004921631	2.613086896	84172.18696	4.159782667	38.02449372	0.004581832	2.591337212	86154.30885	4.260729744	40.23373143
40 to 44	0.006493621	2.588405225	82128.15137	4.043174204	33.90394035	0.005645473	2.563840801	84204.88116	4.153185111	36.10227819
45 to 49	0.00808211	2.57367045	79507.07678	3.899055071	29.93395687	0.00660578	2.559442665	81863.59539	4.028366195	32.05796834
50 to 54	0.010054153	2.574968968	76362.16099	3.727498685	26.05738391	0.00802637	2.575108219	79207.03288	3.884960708	28.04268344
55 to 59	0.01280123	2.616157993	72625.50537	3.524156126	22.26174561	0.010228005	2.613964803	76097.51282	3.714606026	24.07789601
60 to 64	0.019208712	2.630787113	68133.21714	3.259082291	18.55352649	0.014916727	2.626781032	72314.35876	3.492798055	20.19480598
65 to 69	0.029143872	2.605806036	61906.2912	2.894684356	15.15266898	0.021854803	2.627361929	67136.4055	3.192630449	16.54350931
70 to 74	0.043586623	2.569266337	53528.60351	2.422195796	12.11660992	0.034034859	2.62036644	60219.3563	2.787824767	13.13756217
75 to 79	0.064307922	2.531575104	43071.92317	1.861692311	9.441514722	0.055554339	2.579743379	50847.92929	2.245402667	10.07722876
80 to 84	0.099112908	2.474418889	31246.57346	1.253190208	7.075997544	0.090475371	2.506670149	38582.41892	1.580158075	7.477024699
85 to 89	0.162014744	2.347706857	18999.5434	0.66728775	5.078969235	0.15160382	2.383059211	24581.89043	0.885787276	5.351856481
90 to 94	0.256772548	2.132511116	8326.620976	0.240916719	3.634625542	0.24006773	2.184146792	11438.69482	0.344233055	3.844330308
95 plus	0.346693255	2.885974951	2199.98146	0.063892001	2.885974951	0.333064641	3.007325641	3329.123604	0.101541276	3.007325641

Table 15: Nigeria 2100 life table, by age and sex. mx=mortality rate, ax=mean person-years lived in an age interval among those who die in that age interval, lx=number of persons left alive at age x, nLx=person-years lived between age x and x+n, ex=life expectancy at age x.

Age Group	Male					Female				
	mx	ax	lx	nLx	ex	mx	ax	lx	nLx	ex
Early Neonatal	0.302968088	0.009579755	100000	0.019122488	78.96147915	0.279527103	0.009580474	100000	0.019126779	81.87715607
Late Neonatal	0.024168554	0.028760456	99420.86238	0.057161306	79.40065578	0.027420156	0.028759559	99465.51361	0.05718163	82.29660839
Post Neonatal	0.004182982	0.461346684	99282.76363	0.91489802	79.45324021	0.003936179	0.461364216	99308.7726	0.915241907	82.36861055
1 to 4	0.000939211	1.998747719	98900.14895	3.948588255	78.83505265	0.000887832	1.998816224	98948.60319	3.950928499	81.742798
5 to 9	0.000249194	2.499480845	98529.51568	4.923409517	75.12333207	0.000185086	2.499614405	98598.05043	4.927623488	78.02537421
10 to 14	0.00019573	2.551351851	98406.89166	4.917987632	70.21366314	0.000151428	2.545529581	98506.90397	4.923518041	73.0950722
15 to 19	0.000297113	2.723134354	98310.67671	4.91221594	65.27978942	0.000220101	2.698717379	98432.39785	4.919148489	68.14831857
20 to 24	0.000518497	2.696387057	98164.80968	4.902400779	60.37244022	0.000379916	2.7370286	98324.21378	4.91203563	63.22000221
25 to 29	0.000801316	2.643348735	97910.78026	4.886354498	55.52149901	0.000698257	2.660676486	98137.77253	4.89892053	58.33431851
30 to 34	0.001111171	2.603356954	97519.61831	4.863011599	50.73280321	0.000944176	2.630685231	97796.32163	4.878749214	53.52788817
35 to 39	0.001324993	2.576306096	96980.21256	4.833471446	45.99984501	0.001115498	2.599664527	97337.15571	4.853761667	48.76764236
40 to 44	0.001558251	2.635805217	96341.45337	4.799303491	41.28698265	0.001341928	2.660184764	96797.81043	4.824539517	44.02434962
45 to 49	0.002230988	2.674116581	95595.74444	4.755125118	36.58717584	0.001911252	2.661782388	96152.63795	4.786186188	39.30071294
50 to 54	0.00343728	2.683219457	94537.68319	4.689589639	31.96325985	0.002768629	2.655751795	95239.70493	4.731303128	34.64897825
55 to 59	0.005288439	2.709566425	92933.04905	4.591194825	27.46380089	0.004021533	2.669612937	93933.27667	4.653122919	30.08939924
60 to 64	0.008951222	2.715796681	90520.74798	4.435626317	23.11832389	0.006115678	2.653999313	92070.62143	4.53866971	25.6381848
65 to 69	0.014985945	2.671017725	86588.16207	4.184160485	19.04027727	0.008837071	2.675823472	89311.96934	4.376074385	21.33978718
70 to 74	0.023000214	2.653826484	80382.14957	3.814946749	15.30210649	0.014099682	2.718014842	85478.94279	4.141683363	17.16887817
75 to 79	0.038297035	2.629356791	71696.27077	3.289610713	11.83372184	0.026327545	2.731056584	79695.53141	3.763031417	13.20930896
80 to 84	0.064338912	2.56562782	59256.86284	2.566922503	8.770580986	0.053745895	2.643730438	69948.18095	3.11222149	9.660017986
85 to 89	0.119413016	2.466599098	42995.44488	1.657484291	6.129873303	0.103002305	2.533539385	53634.23779	2.152488194	6.795632184
90 to 94	0.213799765	2.21172	23550.56692	0.742192557	4.200650805	0.186215171	2.294651392	32177.89468	1.082249074	4.69777327
95 plus	0.312005377	3.209782108	7917.370709	0.256700984	3.209782108	0.284049898	3.53906556	12666.43953	0.45935081	3.53906556

Table 15: Sao Tome and Principe 2017 life table, by age and sex. mx=mortality rate, ax=mean person-years lived in an age interval among those who die in that age interval, lx=number of persons left alive at age x, nLx=person-years lived between age x and x+n, ex=life expectancy at age x.

Age Group	Male					Female				
	mx	ax	lx	nLx	ex	mx	ax	lx	nLx	ex
Early Neonatal	0.671180222	0.00956847	100000	0.019055188	68.0098288	0.397566656	0.009576856	100000	0.019105157	71.69203113
Late Neonatal	0.086035456	0.02874339	98721.16711	0.056658161	68.8712357	0.049039644	0.028753596	99240.47564	0.057016796	72.22141003
Post Neonatal	0.012383736	0.460764118	98233.81436	0.90181668	69.15515847	0.011401072	0.460833924	98960.90428	0.908902374	72.36781025
1 to 4	0.001814578	1.997580565	97117.35542	3.870635731	69.02164651	0.001281053	1.99829193	97924.83055	3.906977051	72.20543258
5 to 9	0.000733975	2.498470885	96415.29067	4.811931984	65.50984398	0.000589631	2.498771602	97424.4538	4.864050276	68.56613551
10 to 14	0.000633304	2.588133775	96062.20581	4.79578551	60.74149905	0.000351165	2.658379854	97137.6987	4.852895502	63.76122803
15 to 19	0.001007181	2.682380052	95758.54729	4.776780975	55.92587439	0.000861151	2.703285416	96967.29353	4.83879435	58.86854785
20 to 24	0.001530849	2.586934535	95277.4546	4.746341571	51.19437678	0.001198592	2.584964465	96550.60412	4.813598202	54.11068696
25 to 29	0.001665909	2.572617188	94550.95062	4.708511887	46.56736896	0.001364118	2.574168305	95973.68275	4.782859756	49.42013168
30 to 34	0.002143663	2.627503538	93766.76647	4.664622826	41.93485785	0.001706298	2.609861475	95321.29887	4.746712575	44.74031986
35 to 39	0.00303272	2.618576163	92767.16166	4.605117467	37.35789871	0.002300758	2.645427587	94511.47401	4.70011852	40.1008074
40 to 44	0.00397065	2.652580554	91371.15861	4.526396519	32.88793073	0.003378108	2.63760715	93430.32861	4.634548493	35.53333905
45 to 49	0.006146932	2.692981432	89575.01313	4.416161909	28.49321404	0.004662648	2.683446592	91865.24572	4.544185336	31.09267013
50 to 54	0.010148695	2.657544367	86862.74204	4.242369225	24.29758502	0.007762181	2.650159078	89747.71566	4.407054681	26.76130629
55 to 59	0.014946249	2.654542018	82561.9422	3.98843138	20.42361799	0.010896083	2.654580713	86329.20797	4.209005449	22.71450331
60 to 64	0.024047903	2.622824686	76608.65404	3.623575696	16.80355323	0.017366262	2.634990077	81748.5145	3.926391841	18.83624454
65 to 69	0.035466635	2.579066359	67907.94692	3.127370707	13.62022477	0.025492651	2.623926042	74940.06083	3.53347119	15.30607972
70 to 74	0.051134808	2.56332066	56837.53611	2.527703421	10.77172399	0.040528753	2.609028441	65954.1877	3.007300967	12.0319389
75 to 79	0.082329532	2.512451653	43944.68614	1.824590287	8.183115269	0.066459703	2.554132332	53810.54512	2.31594392	9.158171942
80 to 84	0.12678485	2.41502712	28963.30003	1.091500738	6.1225024	0.106957152	2.467781183	38497.00175	1.517057386	6.789971563
85 to 89	0.19252382	2.272577505	15162.94657	0.497597506	4.506711391	0.171364812	2.332198893	22390.83982	0.770408509	4.916171785
90 to 94	0.28537755	2.072303146	5607.021161	0.152897586	3.327711706	0.26041753	2.137294134	9294.344874	0.267206895	3.59150111
95 plus	0.369053413	2.70984479	1252.12348	0.033975843	2.70984479	0.350928706	2.851255889	2386.461529	0.068425908	2.851255889

Table 15: Sao Tome and Principe 2100 life table, by age and sex. mx=mortality rate, ax=mean person-years lived in an age interval among those who die in that age interval, lx=number of persons left alive at age x, nLx=person-years lived between age x and x+n, ex=life expectancy at age x.

Age Group	Male					Female				
	mx	ax	lx	nLx	ex	mx	ax	lx	nLx	ex
Early Neonatal	0.168467142	0.009583878	100000	0.01914714	76.34640549	0.087227447	0.009586368	100000	0.019162051	79.93823541
Late Neonatal	0.021581596	0.02876117	99677.52284	0.057313136	76.57384201	0.013608565	0.028763369	99832.8759	0.057415616	80.05269524
Post Neonatal	0.001735909	0.46152052	99553.89636	0.918433061	76.61122991	0.002169776	0.461489698	99754.75657	0.920101836	80.05772902
1 to 4	0.000246221	1.999671705	99394.5684	3.973827057	75.80956	0.000159339	1.999787547	99555.22677	3.980941501	79.29324621
5 to 9	0.000153066	2.499681113	99296.80411	4.962941694	71.88203463	0.000136632	2.499715351	99491.85771	4.972895079	75.34225644
10 to 14	0.00018878	2.856441334	99220.87425	4.959014583	66.93499611	0.000121642	2.869145346	99423.9541	4.969902711	70.39186894
15 to 19	0.000454043	2.747142784	99127.30682	4.951300215	61.99534461	0.000345443	2.669913309	99363.53117	4.964181611	65.43284124
20 to 24	0.000728305	2.569909465	98902.55002	4.936402528	57.12958352	0.000402847	2.562535672	99192.14927	4.954738062	60.5409647
25 to 29	0.000711042	2.556042263	98543.16156	4.918630083	52.32798278	0.000460013	2.581847756	98992.71811	4.944145735	55.65742912
30 to 34	0.000938366	2.628021085	98193.73029	4.898774328	47.50428091	0.000590982	2.635924596	98765.47475	4.93136531	50.77892365
35 to 39	0.001276875	2.632539229	97734.60431	4.872029756	42.71404952	0.000823504	2.70801172	98474.40484	4.914428239	45.92042953
40 to 44	0.00177414	2.715167635	97113.51098	4.835989493	37.96865569	0.00139253	2.749155897	98070.36766	4.888146128	41.09670233
45 to 49	0.003027915	2.763884782	96257.48573	4.780478792	33.27973013	0.002439754	2.76370664	97391.01283	4.842918009	36.36195258
50 to 54	0.005648991	2.712048908	94813.39639	4.680229657	28.74212993	0.00426927	2.702667234	96213.40031	4.763946751	31.76971874
55 to 59	0.009023971	2.689787106	92178.13633	4.514836547	24.48224382	0.006663567	2.640693604	94186.35865	4.636443972	27.392046
60 to 64	0.014497268	2.650289006	88122.20431	4.261389212	20.48233776	0.008876374	2.618376406	91111.41302	4.461703595	23.2250545
65 to 69	0.021727891	2.618426062	81969.48957	3.897672069	16.81468817	0.012804245	2.673488384	87164.52489	4.232444841	19.1519854
70 to 74	0.031974453	2.626191221	73547.06232	3.419901546	13.43523726	0.021054367	2.686169974	81783.10848	3.900241712	15.23213883
75 to 79	0.054355368	2.581132744	62710.37711	2.775191454	10.29634581	0.037281676	2.678928254	73634.2971	3.391158954	11.61390401
80 to 84	0.0854205	2.496694327	47824.48074	1.977926983	7.699932233	0.072793641	2.576968883	61145.72586	2.607360442	8.433889331
85 to 89	0.14553489	2.392775808	31314.19179	1.144243464	5.464515694	0.128967352	2.450094804	42600.89223	1.616969046	5.972641539
90 to 94	0.240414969	2.164044535	15123.31028	0.454551704	3.841530183	0.215528138	2.237229163	22468.25416	0.714536295	4.208113652
95 plus	0.333568255	3.004336019	4457.119368	0.136180985	3.004336019	0.310923073	3.233317635	7612.712648	0.253087914	3.233317635

Table 15: Senegal 2017 life table, by age and sex. mx=mortality rate, ax=mean person-years lived in an age interval among those who die in that age interval, lx=number of persons left alive at age x, nLx=person-years lived between age x and x+n, ex=life expectancy at age x.

Age Group	Male					Female				
	mx	ax	lx	nLx	ex	mx	ax	lx	nLx	ex
Early Neonatal	0.938553194	0.009560275	100000	0.019006534	66.29257285	0.626770477	0.009569831	100000	0.019063286	70.19027929
Late Neonatal	0.099185161	0.028739763	98216.44928	0.05634723	67.47636438	0.074664796	0.028746527	98805.27152	0.056724966	71.01936044
Post Neonatal	0.015922461	0.460512735	97657.79752	0.895070741	67.80462447	0.01305002	0.460716786	98381.82196	0.902898401	71.26732818
1 to 4	0.003603696	1.995195089	96233.48507	3.821750732	67.87836036	0.003559429	1.995254111	97203.90911	3.860620296	71.20211763
5 to 9	0.000726708	2.498486026	94857.41459	4.734267189	64.83488894	0.00062222	2.498703709	95830.40601	4.784076111	68.1944371
10 to 14	0.000620664	2.636614362	94513.48217	4.7187535	60.06177511	0.000485351	2.566715738	95532.79317	4.771006047	63.39906637
15 to 19	0.001140446	2.687920237	94220.66609	4.698647522	55.24019996	0.000781662	2.647482945	95301.27224	4.756318858	58.54687869
20 to 24	0.001667021	2.590998235	93684.83285	4.665508811	50.54044288	0.001045233	2.618004502	94929.54161	4.734692027	53.7657785
25 to 29	0.00189436	2.56787169	92907.19335	4.624059912	45.94124595	0.001386822	2.636658257	94434.73508	4.706314974	49.03366824
30 to 34	0.002323173	2.607929347	92031.44505	4.576150543	41.35345931	0.001978608	2.647891179	93782.15172	4.667387928	44.35623626
35 to 39	0.003160291	2.636010866	90968.70854	4.514724716	36.80555814	0.0028352	2.637201323	92858.79929	4.612056254	39.7705486
40 to 44	0.004507026	2.653343874	89542.54257	4.430297382	32.34903373	0.003942868	2.62990519	91551.42279	4.535210597	35.30004994
45 to 49	0.006722336	2.667576032	87546.95579	4.309819409	28.02469696	0.005479244	2.642003785	89763.73323	4.430969075	30.94908474
50 to 54	0.010470584	2.658778077	84652.02666	4.131416081	23.89035613	0.008040236	2.634065116	87337.14251	4.285407785	26.73349489
55 to 59	0.015985362	2.648060812	80330.91286	3.871177971	20.0306	0.011372766	2.638459268	83894.80607	4.085161899	22.71973969
60 to 64	0.024887405	2.622271307	74151.53914	3.500757926	16.47752796	0.017130931	2.644596352	79255.86157	3.80938047	18.89246649
65 to 69	0.037654511	2.585993629	65454.42301	3.00059848	13.31745114	0.026830597	2.620122831	72743.82503	3.419474984	15.3447742
70 to 74	0.056194926	2.54391928	54182.79076	2.381529697	10.54999297	0.040717044	2.594531672	63598.49409	2.897183822	12.17356661
75 to 79	0.083673544	2.489425616	40844.96957	1.688964582	8.167678705	0.064921153	2.547717173	51846.29457	2.237799389	9.346707784
80 to 84	0.125596982	2.406176346	26772.20669	1.010976005	6.160949395	0.101782068	2.472975001	37390.19329	1.489058397	6.981872418
85 to 89	0.191041013	2.276127481	14137.50099	0.465765577	4.532959592	0.16528152	2.346865125	22332.1413	0.777922159	5.038041054
90 to 94	0.284005339	2.075230899	5280.909896	0.144517505	3.341809301	0.254270197	2.151558727	9558.636465	0.27795779	3.662202121
95 plus	0.367987924	2.717945437	1191.492983	0.032464806	2.717945437	0.345600059	2.894804248	2531.41163	0.073593776	2.894804248

Table 15: Senegal 2100 life table, by age and sex. mx=mortality rate, ax=mean person-years lived in an age interval among those who die in that age interval, lx=number of persons left alive at age x, nLx=person-years lived between age x and x+n, ex=life expectancy at age x.

Age Group	Male					Female				
	mx	ax	lx	nLx	ex	mx	ax	lx	nLx	ex
Early Neonatal	0.141623683	0.0095847	100000	0.019152066	77.41216844	0.103522039	0.009585868	100000	0.019159059	82.78342015
Late Neonatal	0.01452941	0.028763115	99728.83204	0.057354257	77.60321637	0.016013575	0.028762706	99801.69339	0.057393711	82.92857459
Post Neonatal	0.001893214	0.461509345	99645.50434	0.919211146	77.61050285	0.00173843	0.46152034	99709.79497	0.919869923	82.94739397
1 to 4	0.000267051	1.999643931	99471.50563	3.976736724	76.82195775	0.000276337	1.999631551	99549.909	3.979797252	82.15633673
5 to 9	0.00010306	2.499785291	99365.35324	4.966988216	72.90171355	0.000100615	2.499790386	99439.97744	4.970748751	78.24470087
10 to 14	0.000131399	2.891114641	99314.18017	4.964325451	67.93793456	0.000113007	2.68041608	99389.97704	4.968193696	73.28275016
15 to 19	0.000342229	2.75354624	99248.96292	4.958629169	62.98064961	0.000195659	2.656519636	99333.84431	4.964414339	68.32259918
20 to 24	0.000542133	2.615743665	99079.2906	4.947567394	58.08372281	0.000258498	2.683267402	99236.72625	4.958867923	63.38678945
25 to 29	0.00064308	2.60483538	98811.11432	4.932958819	53.2340835	0.000424915	2.688058897	99108.55753	4.950562283	58.46518516
30 to 34	0.000870341	2.620364236	98493.95169	4.914516085	48.39679335	0.000641129	2.639204655	98898.2419	4.937428088	53.58364319
35 to 39	0.001148503	2.640529727	98066.33443	4.890072591	43.59598241	0.000845177	2.645273834	98581.77051	4.919297712	48.74695369
40 to 44	0.001664264	2.680502911	97504.88455	4.856503693	38.83109707	0.001236018	2.667733921	98166.12129	4.894218023	43.9416372
45 to 49	0.002623867	2.739223001	96697.05155	4.806359356	34.13173505	0.001874121	2.691243512	97561.44965	4.857094341	39.19601849
50 to 54	0.004777125	2.729269937	95437.06239	4.720649871	29.54381516	0.003024673	2.674429688	96652.00059	4.798921859	34.53649355
55 to 59	0.008100652	2.696699004	93186.10599	4.573940614	25.18735374	0.004534302	2.652685079	95203.44842	4.710034148	30.0160222
60 to 64	0.012940927	2.670249789	89492.19925	4.343988198	21.11114692	0.006358573	2.657080281	93076.89542	4.585842724	25.63506656
65 to 69	0.020699271	2.628433122	83889.41111	3.999057769	17.33511843	0.00982931	2.668232815	90176.07827	4.408245707	21.36564239
70 to 74	0.030139678	2.623390344	75660.49366	3.532079472	13.9265982	0.015045845	2.691085944	85880.7018	4.15093329	17.29288414
75 to 79	0.050279444	2.583735081	65108.83366	2.907251095	10.74990412	0.026219561	2.70192747	79703.30617	3.761007068	13.41867598
80 to 84	0.077566081	2.498192666	50713.69729	2.131351646	8.070304265	0.050118012	2.628114117	69968.04617	3.132567464	9.908333031
85 to 89	0.135862763	2.419466998	34551.88331	1.288342522	5.69731293	0.097836956	2.550735234	54563.48595	2.210894271	6.967520671
90 to 94	0.230729304	2.182184884	17507.24093	0.535699043	3.966817314	0.180298886	2.306046032	33443.93138	1.134586202	4.80011539
95 plus	0.325772778	3.076022068	5423.502366	0.169433244	3.076022068	0.27858927	3.60303488	13457.39136	0.49334232	3.60303488

Table 15: Sierra Leone 2017 life table, by age and sex. mx=mortality rate, ax=mean person-years lived in an age interval among those who die in that age interval, lx=number of persons left alive at age x, nLx=person-years lived between age x and x+n, ex=life expectancy at age x.

Age Group	Male					Female				
	mx	ax	lx	nLx	ex	mx	ax	lx	nLx	ex
Early Neonatal	1.565190295	0.009541069	100000	0.018893158	59.46721784	1.238342089	0.009551086	100000	0.018952168	61.26068678
Late Neonatal	0.120270213	0.028733947	97043.71913	0.055640765	61.25604458	0.122934098	0.028733212	97653.44722	0.055986022	62.71182066
Post Neonatal	0.048622149	0.458189921	96374.96359	0.870159224	61.62291535	0.046274276	0.458356691	96965.45113	0.876425172	63.09876248
1 to 4	0.011270378	1.98497336	92148.64948	3.604220415	63.50457804	0.009664363	1.987114512	92912.51616	3.645647914	64.90800484
5 to 9	0.001433471	2.497013604	88093.22307	4.388935686	62.34022079	0.001241527	2.497413488	89392.75263	4.455798794	63.38760915
10 to 14	0.001006145	2.510967525	87464.95958	4.362331929	57.77082582	0.000843149	2.494541979	88839.77357	4.43262765	58.76638979
15 to 19	0.001502273	2.66441163	87026.40177	4.336116565	53.04941088	0.001231086	2.67091646	88466.17934	4.410668332	54.00397828
20 to 24	0.002225486	2.614789797	86375.14858	4.295955517	48.42864823	0.001873858	2.654309707	87923.36313	4.376938737	49.32070977
25 to 29	0.002775273	2.609037045	85419.24386	4.2428141	43.94027589	0.002661179	2.635678746	87103.47893	4.327951391	44.75952912
30 to 34	0.003764841	2.616416167	84242.05176	4.174648953	39.51678618	0.00368362	2.620174708	85952.17373	4.260268312	40.3232089
35 to 39	0.005028983	2.605488612	82670.92657	4.084388499	35.21684485	0.00492467	2.592574042	84383.45356	4.169764902	36.02333415
40 to 44	0.006583629	2.613167925	80617.97697	3.968588404	31.04581985	0.006125818	2.589955396	82330.88621	4.056702766	31.855619
45 to 49	0.009107866	2.620506383	78007.21459	3.817721023	26.99528638	0.008007066	2.618066539	79847.09019	3.917715321	27.7633561
50 to 54	0.012799045	2.620297967	74534.12966	3.616734421	23.12847983	0.011446666	2.618735307	76712.91198	3.734016482	23.78718405
55 to 59	0.018363248	2.620001184	69913.40715	3.349610608	19.4810746	0.01596219	2.630182726	72445.78785	3.490542001	20.02963368
60 to 64	0.027261882	2.596993676	63778.24191	2.993407451	16.10097042	0.024520119	2.615349302	66888.0963	3.160245296	16.47173075
65 to 69	0.039106525	2.579060469	55644.21616	2.542470798	13.0747663	0.036217119	2.599033975	59167.42175	2.722780257	13.27740618
70 to 74	0.059839743	2.534389866	45742.97128	1.994382594	10.34942196	0.057399085	2.552850819	49358.20455	2.165700327	10.40076699
75 to 79	0.085598164	2.473928491	33869.2117	1.393955045	8.097163846	0.085307863	2.48872757	37010.52212	1.526118584	8.027315881
80 to 84	0.126196918	2.402991471	22010.51737	0.830177298	6.142278002	0.130591603	2.409935755	24089.86798	0.90214875	6.01487194
85 to 89	0.191642113	2.274700003	11595.08498	0.381575032	4.522443112	0.197636026	2.273143127	12409.94667	0.40445958	4.438126611
90 to 94	0.284560505	2.074049259	4318.601409	0.118057767	3.336153147	0.286547514	2.0770771	4480.106746	0.122353862	3.313141703
95 plus	0.368418409	2.71469316	971.3609886	0.026432589	2.71469316	0.373278925	2.679987386	997.8021911	0.026874159	2.679987386

Table 15: Sierra Leone 2100 life table, by age and sex. mx=mortality rate, ax=mean person-years lived in an age interval among those who die in that age interval, lx=number of persons left alive at age x, nLx=person-years lived between age x and x+n, ex=life expectancy at age x.

Age Group	Male					Female				
	mx	ax	lx	nLx	ex	mx	ax	lx	nLx	ex
Early Neonatal	0.244313579	0.009581553	100000	0.019133241	77.00883911	0.212207066	0.009582537	100000	0.019139122	78.50600121
Late Neonatal	0.018204102	0.028762102	99532.82555	0.057235493	77.34975858	0.026204632	0.028759895	99594.0425	0.05725752	78.80569001
Post Neonatal	0.004893961	0.461296177	99428.67349	0.915943422	77.37307115	0.005417193	0.461259007	99444.05179	0.915863961	78.86672888
1 to 4	0.00124743	1.998336761	98980.83868	3.949388676	76.79591607	0.001065318	1.998579577	98948.33321	3.949525355	78.33399638
5 to 9	0.000254163	2.499470493	98489.0896	4.921330924	73.16774907	0.000239495	2.499501051	98528.2975	4.923470008	74.65776048
10 to 14	0.000233057	2.748339886	98364.17739	4.915637573	68.2571238	0.000220524	2.633701177	98410.52977	4.917958995	69.74386185
15 to 19	0.000538383	2.747221455	98249.75975	4.906553252	63.3330766	0.000377198	2.692706044	98302.2292	4.910835639	64.81758344
20 to 24	0.000882515	2.617220546	97986.05807	4.889021579	58.49506484	0.000563355	2.659870748	98117.25154	4.899407031	59.93434799
25 to 29	0.001011683	2.595266785	97555.89322	4.865954154	53.74010299	0.000807947	2.641279087	97841.61326	4.882783709	55.09522314
30 to 34	0.001315422	2.598477628	97065.24912	4.837988949	48.99777804	0.001114564	2.631311536	97447.63839	4.859523073	50.30655463
35 to 39	0.001613798	2.601652103	96430.79836	4.802983882	44.30227377	0.001475533	2.623844915	96906.83951	4.828411624	45.5717727
40 to 44	0.002109012	2.635652803	95658.0284	4.759209563	39.63807427	0.001988153	2.645037816	96195.52422	4.787369563	40.88800188
45 to 49	0.003010023	2.684665685	94657.23534	4.700175764	35.02764449	0.00287345	2.673677834	95245.51066	4.73068959	36.26742523
50 to 54	0.004863939	2.696874735	93247.03994	4.610785627	30.51446354	0.004457198	2.661501637	93889.22362	4.646111029	31.74969843
55 to 59	0.007788795	2.668425833	91013.18357	4.469633294	26.19417512	0.006483146	2.641021691	91825.01841	4.522219329	27.39920936
60 to 64	0.011594588	2.651029908	87548.56941	4.261808848	22.12227259	0.009131688	2.634819362	88906.34187	4.351792651	23.20763777
65 to 69	0.017686838	2.636106078	82631.7678	3.966668865	18.27741989	0.013374321	2.662831197	84953.18176	4.119697924	19.15825134
70 to 74	0.026574271	2.630076741	75662.95314	3.560698533	14.71520901	0.021649498	2.670275994	79488.31615	3.785219501	15.28594942
75 to 79	0.042986544	2.600621154	66281.74768	3.007491863	11.42467909	0.036927363	2.666089245	71378.12752	3.289117945	11.71465956
80 to 84	0.068224194	2.530044278	53514.04728	2.29468617	8.536687541	0.07018287	2.574625971	59405.22568	2.545997147	8.53825345
85 to 89	0.124382075	2.451787707	38086.28723	1.451811382	5.985543312	0.125763996	2.458209017	41918.6617	1.598909953	6.039670719
90 to 94	0.219087874	2.20344972	20314.50591	0.633278725	4.122072868	0.212270429	2.245103704	22343.82434	0.712271715	4.246486123
95 plus	0.316355059	3.164825407	6621.453249	0.211419331	3.164825407	0.308072519	3.256911915	7604.076719	0.252428822	3.256911915

Table 15: Togo 2017 life table, by age and sex. mx=mortality rate, ax=mean person-years lived in an age interval among those who die in that age interval, lx=number of persons left alive at age x, nLx=person-years lived between age x and x+n, ex=life expectancy at age x.

Age Group	Male					Female				
	mx	ax	lx	nLx	ex	mx	ax	lx	nLx	ex
Early Neonatal	1.214942767	0.009551804	100000	0.018956412	61.84300884	0.875723034	0.0095622	100000	0.019017947	67.56709984
Late Neonatal	0.070840491	0.028747582	97697.40896	0.056095129	63.28000488	0.051050661	0.028753041	98334.73993	0.056493166	68.69120872
Post Neonatal	0.019094486	0.460287402	97300.21948	0.890493178	63.48059946	0.018345204	0.460340629	98046.41009	0.897629506	68.83551098
1 to 4	0.006256832	1.991657649	95601.00997	3.776628436	63.67757897	0.005580609	1.992559251	96400.29476	3.813315386	69.07960687
5 to 9	0.001048002	2.497816664	93240.41493	4.649831443	61.24062794	0.000875809	2.498175399	94273.47575	4.70337021	66.59357457
10 to 14	0.000941127	2.668694127	92753.27164	4.627511516	56.54880467	0.00073293	2.596233648	93861.63464	4.684828062	61.87459621
15 to 19	0.001826207	2.665291587	92317.84272	4.59630006	51.80261462	0.001222514	2.622139725	93518.31579	4.662367603	57.09215408
20 to 24	0.002425751	2.580752467	91478.5442	4.547249539	47.25260066	0.001466665	2.612152248	92948.41029	4.63121094	52.42595445
25 to 29	0.002825888	2.595990516	90375.72935	4.488309476	42.79634133	0.002043764	2.646601544	92269.3549	4.591393169	47.79217867
30 to 34	0.003824073	2.633596845	89107.93447	4.415455586	38.36699614	0.002954014	2.641132727	91331.38032	4.53496067	43.2553361
35 to 39	0.005438036	2.63661308	87420.5371	4.315598199	34.05537679	0.004116923	2.600939696	89992.42649	4.455633336	38.85896494
40 to 44	0.007715981	2.621212054	85075.71989	4.177197482	29.91977498	0.005114468	2.586041753	88159.01198	4.354239879	34.61185046
45 to 49	0.010576393	2.613373895	81856.27107	3.992194302	25.9909858	0.006530888	2.607184596	85933.3187	4.230620247	30.43868674
50 to 54	0.014728518	2.609489009	77640.55409	3.75025748	22.25750402	0.008982018	2.60836824	83172.73025	4.071309482	26.35903575
55 to 59	0.020706765	2.605597551	72129.74246	3.436548258	18.75604856	0.012068152	2.629247585	79521.90607	3.865737931	22.44509616
60 to 64	0.030079877	2.592067748	65033.99373	3.032803415	15.51680805	0.018232577	2.642634189	74868.40214	3.589646281	18.67287344
65 to 69	0.044125192	2.55624297	55944.85275	2.526072016	12.61706425	0.02858966	2.602941669	68347.06966	3.199190261	15.19901897
70 to 74	0.062484512	2.512779092	44849.62235	1.942279558	10.10988706	0.040997228	2.579231798	59249.01239	2.696578217	12.13219628
75 to 79	0.088973694	2.465714693	32779.61484	1.339021434	7.916352421	0.063858485	2.553796391	48269.33939	2.089694831	9.309427953
80 to 84	0.130639856	2.39318664	20941.04267	0.782527438	6.013795177	0.104762784	2.473572827	35033.95917	1.388173049	6.873267957
85 to 89	0.19627911	2.264065975	10788.63683	0.351820259	4.446435424	0.168810535	2.338436378	20640.34987	0.714548535	4.968223981
90 to 94	0.288787787	2.06500143	3925.959716	0.106520694	3.295115128	0.257828825	2.143311309	8700.223133	0.251588761	3.621676283
95 plus	0.371672165	2.691043567	864.4167451	0.023337534	2.691043567	0.348679198	2.869845112	2269.738739	0.065552368	2.869845112

Table 15: Togo 2100 life table, by age and sex. mx=mortality rate, ax=mean person-years lived in an age interval among those who die in that age interval, lx=number of persons left alive at age x, nLx=person-years lived between age x and x+n, ex=life expectancy at age x.

Age Group	Male					Female				
	mx	ax	lx	nLx	ex	mx	ax	lx	nLx	ex
Early Neonatal	0.20841248	0.009582653	100000	0.019139811	74.8399908	0.168136553	0.009583888	100000	0.019147198	81.40493609
Late Neonatal	0.010644128	0.028764187	99601.17486	0.057287244	75.11986069	0.01312041	0.028763504	99678.11342	0.057327413	81.64813052
Post Neonatal	0.002377133	0.461474968	99540.20456	0.918034771	75.10823377	0.002752356	0.461448313	99602.90508	0.918454008	81.65213861
1 to 4	0.000671858	1.99910419	99322.01114	3.967549246	74.34854212	0.000643604	1.999141861	99350.15303	3.968897269	80.93494259
5 to 9	0.000160591	2.499665435	99055.58573	4.950792232	70.5423965	0.000150141	2.499687206	99094.83283	4.952883059	77.13761352
10 to 14	0.000180583	2.972105311	98976.11486	4.94697895	65.59688124	0.00015342	2.75433934	99020.49937	4.949309867	72.19348901
15 to 19	0.000555454	2.756275302	98886.8149	4.938173189	60.65331298	0.000327531	2.636110405	98944.60115	4.943408364	67.24660575
20 to 24	0.000852709	2.61724058	98612.60519	4.920629384	55.81374032	0.000372722	2.632985586	98782.73779	4.93479932	62.35227991
25 to 29	0.001034935	2.608309416	98193.21567	4.897559568	51.03993216	0.000581396	2.642085927	98598.89188	4.923198955	57.4634069
30 to 34	0.00141593	2.628444535	97686.75188	4.867972776	46.28980495	0.000770821	2.623686654	98312.91092	4.906587369	52.62255784
35 to 39	0.001891638	2.643848192	96998.38051	4.828408688	41.59805288	0.00096418	2.613588863	97935.20036	4.885492997	47.81518783
40 to 44	0.002740031	2.671703785	96086.57298	4.773886714	36.96518675	0.001270033	2.677606352	97464.61745	4.8589046	43.0329998
45 to 49	0.004193065	2.719178089	94781.47325	4.694170234	32.43337945	0.002062	2.709300018	96848.08216	4.819605745	38.28844578
50 to 54	0.007229045	2.69933086	92820.5977	4.565091534	28.0553664	0.003335698	2.676568791	95855.68675	4.755941423	33.65377983
55 to 59	0.011294174	2.640717402	89538.63714	4.361102984	23.97815637	0.0049446	2.656254081	94273.62081	4.659653845	29.16860448
60 to 64	0.015843245	2.630605388	84644.64312	4.080175222	20.20657493	0.007023652	2.657217508	91981.03316	4.524954052	24.82418554
65 to 69	0.024246632	2.604223664	78232.6131	3.699061815	16.63892027	0.010831714	2.65568173	88820.9693	4.331611609	20.6041944
70 to 74	0.033422674	2.59800346	69381.5872	3.214488162	13.42764788	0.015899443	2.692199706	84174.46647	4.060851707	16.58727555
75 to 79	0.053732926	2.572721903	58790.28937	2.605659547	10.38027239	0.028641283	2.720805111	77786.05944	3.653430854	12.72360438
80 to 84	0.083417098	2.495004365	45047.46452	1.87042528	7.777204162	0.058923766	2.611047356	67457.04796	2.963535265	9.253374668
85 to 89	0.143170278	2.398855903	29792.63089	1.093235397	5.512890534	0.110456031	2.50720862	50339.11974	1.984484101	6.517677739
90 to 94	0.238151434	2.16870056	14528.53775	0.437976673	3.867430267	0.195067373	2.279643789	28969.81545	0.955035476	4.529685783
95 plus	0.331780597	3.019173724	4315.120318	0.13219138	3.019173724	0.292313676	3.433590569	10791.82376	0.377421591	3.433590569
